# ADAR1-mediated 3′ UTR editing and expression control of antiapoptosis genes fine-tunes cellular apoptosis response

**DOI:** 10.1038/cddis.2017.12

**Published:** 2017-05-25

**Authors:** Chang-Ching Yang, Yi-Tung Chen, Yi-Feng Chang, Hsuan Liu, Yu-Ping Kuo, Chieh-Tien Shih, Wei-Chao Liao, Hui-Wen Chen, Wen-Sy Tsai, Bertrand Chin-Ming Tan

**Affiliations:** 1 Graduate Institute of Biomedical Sciences, College of Medicine, Chang Gung University, Kueishan, Taoyuan, Taiwan; 2 Department of Biomedical Sciences, College of Medicine, Chang Gung University, Kueishan, Taoyuan, Taiwan; 3 Molecular Medicine Research Center, Chang Gung University, Kueishan, Taoyuan, Taiwan; 4 Department of Biochemistry, College of Medicine, Chang Gung University, Kueishan, Taoyuan, Taiwan; 5 Division of Colon and Rectal Surgery, Department of Surgery, Chang Gung Memorial Hospital, Linkou, Taiwan; 6 Graduate Institute of Clinical Medical Science, College of Medicine, Chang Gung University, Kueishan, Taoyuan, Taiwan; 7 Department of Neurosurgery, Lin-Kou Medical Center, Chang Gung Memorial Hospital, Linkou, Taiwan

## Abstract

Adenosine-to-inosine RNA editing constitutes a crucial component of the cellular transcriptome and critically underpins organism survival and development. While recent high-throughput approaches have provided comprehensive documentation of the RNA editome, its functional output remains mostly unresolved, particularly for events in the non-coding regions. Gene ontology analysis of the known RNA editing targets unveiled a preponderance of genes related to apoptosis regulation, among which proto-oncogenes *XIAP* and *MDM2* encode two the most abundantly edited transcripts. To further decode this potential functional connection, here we showed that the main RNA editor ADAR1 directly targets this 3′ UTR editing of *XIAP* and *MDM2*, and further exerts a negative regulation on the expression of their protein products. This post-transcriptional silencing role was mediated via the inverted *Alu* elements in the 3′ UTR but independent of alteration in transcript stability or miRNA targeting. Rather, we discovered that ADAR1 competes transcript occupancy with the RNA shuttling factor STAU1 to facilitate nuclear retention of the *XIAP* and *MDM2* mRNAs. As a consequence, ADAR1 may acquire functionality in part by conferring spatial distribution and translation efficiency of the target transcripts. Finally, abrogation of ADAR1 expression or catalytic activity elicited a XIAP-dependent suppression of apoptotic response, whereas ectopic expression reversed this protective effect on cell death. Together, our results extended the known functions of ADAR1 and RNA editing to the critical fine-tuning of the intracellular apoptotic signaling and also provided mechanistic explanation for ADAR1’s roles in development and tumorigenesis.

Among mechanisms that demarcate the transcriptome, adenosine-to-inosine (A-to-I) RNA editing is a co-transcriptional process that remains largely unresolved in terms of functional consequences. Despite the seemingly genetic message-disrupting nature of this base conversion, editing exhibits overabundance in the repetitive *Alu* elements and in the brain transcriptome,^1, 2, 3^ and is thus regarded as a key determinant in primate evolution and development of higher brain functions.^4^ ADAR (adenosine deaminases acting on RNA) family proteins constitute the key enzymatic activity for A-to-I editing, and they catalyze hydrolytic deamination of adenosine to inosine in structured or double-stranded RNA.^2^ Activity and substrate selectivity of the editing-competent ADAR1 and ADAR2 is strictly dependent on the dsRNA-binding domain (dsRBD)-mediated recognition of the RNA substrates,^5^ such as the *Alu* retrotransposon element.^6, 7, 8^ ADAR1 is absolutely essential for life in mammals, as inactivation of this gene in mice leads to embryonic lethality attributable to apoptosis.^9, 10^ Further studies on the functional implications of ADAR1 in intestinal stem cell and virus infection also evidence that ADAR1 is intimately linked to cell survival.^11, 12^ In line with this biological significance, the dysregulation of ADAR expression level and editing frequency are observed in most cancers and presumably underlie the tumorigenic potential. In this respect, both oncogenic and tumor-suppressive roles of ADAR in cancer development are reported, which are associated with tissue types and disease states.^13, 14, 15^ Moreover, in contrast to the generally pro-growth functions, loss of ADAR1 or ADAR2 leads to pro-proliferative consequences in metastatic melanomas and astrocytoma,^16, 17^ indicative of a tumor-suppressive role. Based on these contrasting findings, the exact role of ADAR1 in carcinogenesis remains an as yet unresolved issue.

Deep sequencing technologies have in the recent years unveiled millions of A-to-I(G) editing sites in mammals,^18, 19, 20, 21, 22, 23^ providing a comprehensive physical profiling of the RNA editome. However, as opposed to the established biological relevance of several recoding events,^21^ A-to-I(G) edits in the non-coding regions of gene transcripts, such as the 3′ UTR, remains mostly uncharacterized. To provide a basis for further functional interrogation of this regulatory mechanism, we performed pathway analysis on the publicly available RNA editome data sets and subsequently revealed an enrichment of edited targets implicated in apoptosis and cell cycle progression. Moreover, two cellular apoptosis inhibitors, *XIAP* and *MDM2*, are the most prominently 3′ UTR-edited genes annotated in the database, and therefore noteworthy targets for further investigation.

Abnormal apoptosis signaling is a principal mechanism underlying cancer development and may be triggered by overexpression of antiapoptotic proteins, among which the inhibitor of apoptosis protein (IAP) family represents a potent class of effectors. The death resistance function of IAPs mainly lies in binding and neutralizing caspases via the common baculovirus-IAP-repeat (BIR)-domains, and depends on their E3 ubiquitin ligase activity. X-linked inhibitor of apoptosis protein (XIAP) is one of the best-defined members and acts by directly inactivating caspases. Consequently, the initiation of apoptosis signals emitted from either death receptors or intrinsic cell death pathways is interfered by XIAP.^24, 25, 26^ In line with its central role in the resistance to various apoptotic stimuli, XIAP is frequently overexpressed in different cancer types. Intriguingly, there exist several functional parallelisms between XIAP and MDM2. MDM2 also encodes an oncogenic E3 ubiquitin ligase. It exerts pro-tumorigenic function by negatively regulating the protein stability of tumor repressors, such as p53, via proteasomal degradation.^27, 28^ Similarly, aberrant overexpression or amplification of this gene locus has been found in a variety of different cancers, strengthening its link to cancer pathobiology.

## Results

### The 3′ UTR of proto-oncogenes *XIAP* and *MDM2* is targeted by ADAR1-mediated RNA editing

**Figure 1:**
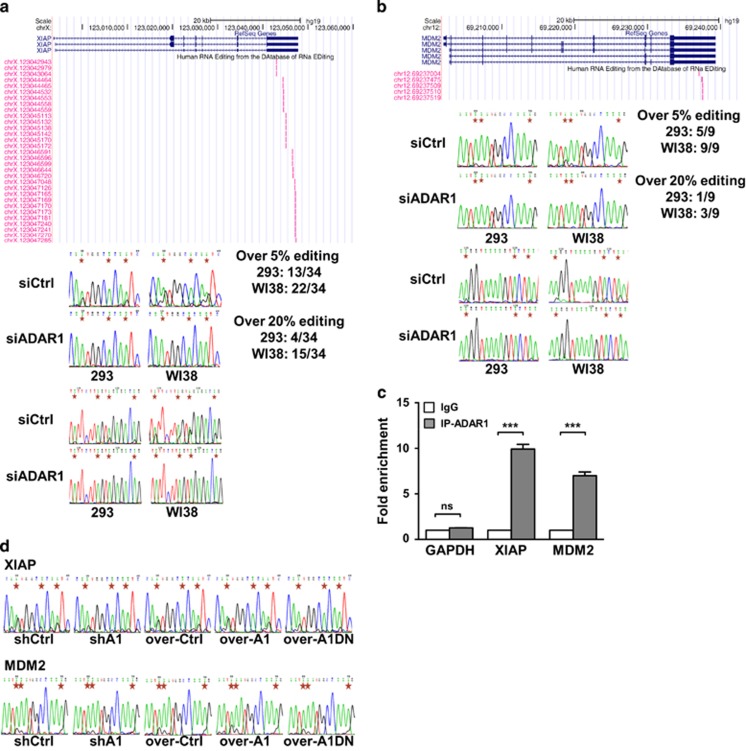
The 3′ UTRs of *XIAP* and *MDM2* transcripts are targeted by ADAR1-mediated RNA editing. (**a** and **b**) Top panel depicts the UCSC Genome Browser-based representation of the distribution of the annotated editing sites in the *XIAP* (**a**) and *MDM2* (**b**) 3′ UTR. The editing status was independently monitored by Sanger sequencing, which was performed on cDNAs derived from 293 or WI38 cells with either control (siCtrl) or ADAR1-targeting (siADAR1) siRNAs, as indicated. The Sanger sequencing chromatograms corresponding to selected editing regions of the *XIAP* and *MDM2* transcripts are shown in the lower panel, with positions of A-to-G conversion being marked by red stars. The estimated editing ratios in the indicated samples are noted in the boxes. (**c**) RNA-immunoprecipitation (RIP) assay was performed to examine the binding of *XIAP* and *MDM2* transcripts by ADAR1. The RNA precipitated by the control IgG or ADAR1 (IP-ADAR1) antibodies was subjected to real-time RT-PCR with primers specific to 3′ UTR of *XIAP* and *MDM2*. Fold of binding enrichment was normalized to the value of IgG, and shown with mean±S.D. GAPDH levels in the immunoprecipitates served as control. (**d**) Sanger sequencing chromatograms for selected editing regions of XIAP (top) and MDM2 (bottom) transcripts derived from different transfectants of 293 cells: pSUPER control vector (shCtrl) and shADAR1 (shA1) for the knockdown experiments; and pcDNA control vector (over-Ctrl), ADAR1 (over-A1) and dominant-negative ADAR1 (over-A1DN) for the ectopic expression experiments. Red stars mark the positions of RNA editing sites (for statistical analyses shown in this figure: NS, not significant or *P*>0.05; *P<0.05; **P<0.01; ***P<0.001)

Loss of *Adar1* is known to trigger widespread apoptosis and consequently lethality during embryogenesis,^9, 12^ implying a link of this RNA editor to the regulation of cell death. However, it presently remains unresolved as to the extent of its involvement as well as the underlying mode of action. We conducted pathway analysis on the results from our previous transcriptome-wide RNA editome profiling^23^ and the publicly available database (DARNED), and discovered that the candidate target set was enriched in genes implicated in apoptosis (Supplementary Figure S1). Among this group of putative targets, *XIAP* and *MDM2* transcripts were found highly edited (based on the numbers of A-to-G conversions supported by RNA-seq), with editing events congregated in their 3′ UTR (Figures 1a and b, top). We then set out to confirm the possibility that *XIAP* and *MDM2* are *bona fide* editing targets, by performing Sanger sequencing of corresponding cDNAs derived from WI38 fibroblasts and HEK293 cells. Owing to the notion that *XIAP* and *MDM2* typically undergo amplification and/or overexpression in most forms of cancer, these non-transformed, non-tumorigenic cell lines were chosen for their physiological expression of target genes. We were able to locate the majority of the predicted editing events in these non-tumorigenic cell lines and further demonstrated, via siRNA-mediated *ADAR1* knockdown assay (Supplementary Figure S2), that these edits are mediated by this editing enzyme (Figures 1a and b, bottom). Further sequencing analysis of genomes affirmed the authenticity of RNA editing event (Supplementary Figure S3).

We next sought to examine whether ADAR1 exerts these sequence changes through direct RNA binding and catalysis. To this end, we first performed native RIP assay to investigate the interaction of ADAR1 with the transcripts of *XIAP* and *MDM2* (Supplementary Figure S4a). Subsequent real-time PCR analysis showed that ADAR1 indeed specifically and efficiently associates with the 3′ UTR of these transcripts (Figure 1c and Supplementary Figure S4b). In addition, we also characterized the effect of ectopic expression – construct for a dominant-negative, enzymatically dead variant of ADAR1^32^ was generated and delivered to cells (Supplementary Figure S5). Target RNA editing was then found to be diminished in the presence of enzymatically defective variant, to a similar extent as in the context of ADAR1 knockdown (Figure 1d), thus attributing ADAR1-mediated catalysis to these editing events. Overall, these results substantiated this enzyme–substrate relationship.

### ADAR1-dependent RNA editing is linked to expression regulation of *XIAP* and *MDM2*

**Figure 2:**
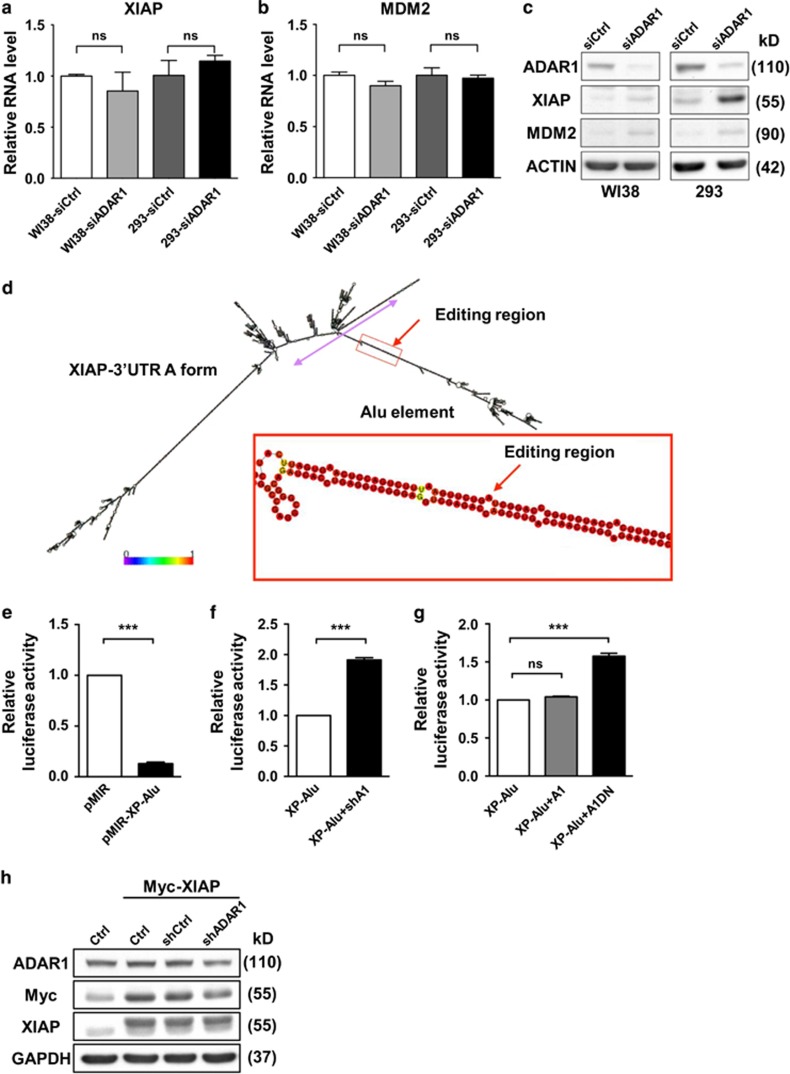
ADAR1 targets the 3′ UTR of *XIAP* and *MDM2* for post-transcriptional expression regulation. (**a** and **b**) Following transfection with control (siCtrl) or ADAR1-targeting (siADAR1) siRNAs, total RNAs were isolated from WI38 or 293 cells and subjected to real-time RT-PCR to quantify the expression levels of *XIAP* (**a**) and *MDM2* (**b**) mRNA. Relative RNA levels (to *GAPDH*) were normalized to the control group, and shown as mean±S.D. (**c**) WI38 and 293 cells were transfected with siRNAs targeting ADAR1 (siADAR1) or control siRNAs (siCtrl) prior to western blot analysis for XIAP, MDM2 and ADAR1 expression. ACTIN serves as the internal control. (**d**) Putative secondary structure of the *XIAP* 3′ UTR, as predicted by the RNAfold Webserver. Hyperediting region is denoted by a red arrow, with a magnified view of the boxed area shown below. Colors of the nucleotides correspond to base-pair probabilities, based on the color scale bar. Sequences corresponding to this particular stem loop, with the embedded IR*Alus*, were subcloned into the 3′ UTR reporter plasmid. (**e**) 3′ UTR reporter assay was conducted on cells transfected with control empty vector (pMIR vector) or the construct containing IR*Alus* derived from XIAP 3′ UTR (pMIR-XP-Alu). Luciferase activity was detected after 48 h and normalized to the co-expressed β-gal levels, with controls being represented as 1. (**f** and **g**) 3′ UTR reporter assay was done as in (**e**), except with the additional co-transfection of expression plasmids for ADAR1-targeting shRNAs (shA1), the wild-type form of ADAR1 (A1), or the dominant-negative mutant (A1DN). (**h**) The construct encoding a 3′ UTR-free Myc-tagged XIAP and control vector (Ctrl) were ectopically co-expressed with pSUPER vector (shCtrl) or ADAR1-targeting (shADAR1) shRNAs, as indicated. Protein expression of XIAP, Myc and ADAR1 in different transfectants of HeLa cells was subsequently detected by immunoblotting, with GAPDH as the loading control (for statistical analyses shown in this figure: NS, not significant or *P*>0.05; *P<0.05; **P<0.01; ***P<0.001)

To decipher whether transcript binding and editing by ADAR1 is functionally correlated with the expression of *XIAP* and *MDM2*, we next assessed their RNA and protein levels in ADAR1 knockdown cells. Interestingly, we found that the abundance of *XIAP* and *MDM2* mRNA transcripts was not significantly changed upon ADAR1 downregulation (Figures 2a and b). On the contrary, knockdown of ADAR1 led to increase in the corresponding protein levels (Figure 2c and Supplementary Figure S6a). Conversely, protein abundance underwent reduction in cells with overexpressed ADAR1 (Supplementary Figure S6a). To exclude the possibility that these expression alterations might be due to altered cellular stress response caused by ADAR1 downregulation, we monitored protein levels in the presence of stressors, such as interferon-*β* and poly(I:C). We subsequently did not detect any changes in the protein expression under these treatments (Supplementary Figure S6b). These observations thus implied that ADAR1 might directly regulate the expression of XIAP and MDM2 at particular post-transcriptional levels.

### ADAR1 targets the inverted *Alu* elements in 3′ UTR and alleviates its suppressive role

We next sought to further strengthen the functional relevance of 3′ UTR editing in target gene regulation. Sequence analysis together with structural prediction (RNAfold) of the target 3′ UTRs revealed the expected enrichment of editing in one inverted *Alu* pairs (IR*Alus*) (Figure 2d and Supplementary Figure S7). To elucidate the effect of this unique secondary structure on gene expression, we generated XIAP 3′ UTR reporter (pMIR-XP-Alu) by grafting the *Alu* element to pMIR luciferase reporter. Intriguingly, this *Alu*-encoded structural element considerably diminished the reporter protein expression (Figure 2e), indicative of its silencing function. We next characterized the effects of ADAR1 mis-expression (Supplementary Figure S8) on the *XIAP* 3′ UTR reporter activity. Simultaneous abrogation of ADAR1 by siRNAs significantly relieved the suppressive effect of the *Alu* element (Figure 2f). Ectopic expression of the dominant-negative ADAR1 mutant displayed a similar magnitude of de-repression as the knockdown experiment (Figure 2g), whereas overexpression of the wild-type ADAR1 did not affect the reporter activity. Analogous effects of ADAR1 mis-expression on the reporter were also observed for the *MDM2* 3′ UTR construct (pMIR-M2-Alu; Supplementary Figure S9). To corroborate further the notion that ADAR1 exerts its effect through the target 3′ UTR, we assessed the expression of an ectopic 3′ UTR-less XIAP-expressing construct. The expression levels of the ectopic proteins, as indicated by anti-Myc or anti-XIAP immunoblotting, remained invariable regardless of the levels of ADAR1 (Figure 2h). Considered together, our results pinpointed 3′ UTR as the site of ADAR1’s action, through which ADAR1 inhibits target expression.

### ADAR1 alters nucleocytoplasmic RNA transport of target transcripts

**Figure 3:**
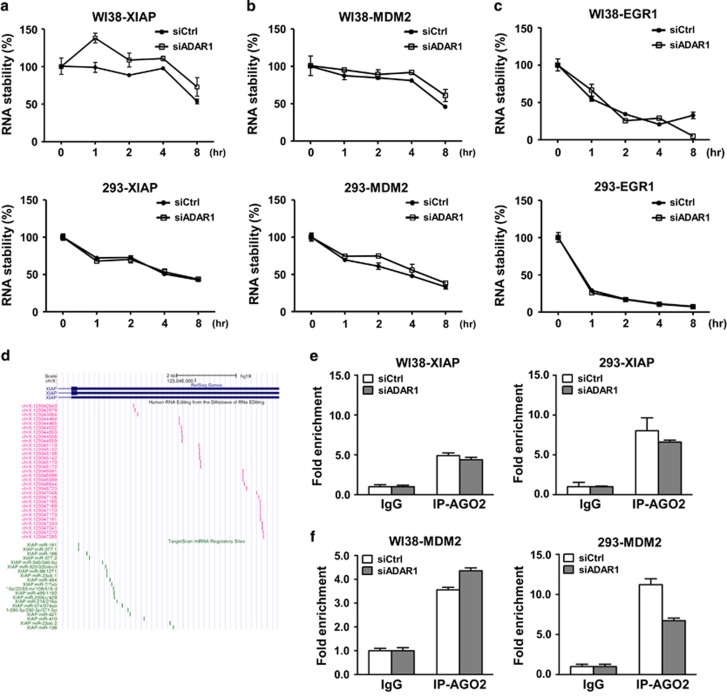
The post-transcriptional regulatory role of ADAR1 is independent of RNA stability and microRNAs targeting. (**a** and **b**) RNA stability of the *XIAP* (**a**) and *MDM2* (**b**) transcripts was measured in WI38 (top) and 293 (bottom) cells harboring control (siCtrl) or ADAR1-targeting (siADAR1) siRNAs. Dynamic changes in the abundance of *XIAP* and *MDM2* mRNAs post-transcription block (by actinomycin D treatment) were measured by real-time PCR and plotted relative to the initial time point. (**c**) The dynamic turnover of *EGR1* transcripts was monitored as above and serves as the positive experimental control. (**d**) An UCSC Genome Browser-based scheme depicting the relative locations of annotated RNA editing sites (red ticks) and miRNA binding sites (green ticks) in the *XIAP* 3′ UTR. (**e** and **f**) RIP assay was performed, using control antibody (IgG) or antibody against AGO2 (IP-AGO2), to assess AGO2’s occupancy of the *XIAP* (**e**) and *MDM2* (**f**) transcripts in ADAR1-depleted WI38 (left) and 293 (right) cells. The graphs represent the quantitative determination of bound RNA corresponding to target 3′ UTR in the immunoprecipitates, as determined by quantitative RT-PCR. Data presented are normalized to the values of IgG and shown as mean±S.D.

On the basis of 3′ UTR editing and possible post-transcriptional regulation, we hypothesized that ADAR1 may exert its regulatory effect via altering transcript stability and localization.^33^ To this end, we first interrogated the RNA stability of target transcripts and subsequently demonstrated that the stability of both *XIAP* and *MDM2* transcripts remained invariable in control *versus* ADAR1 knockdown WI38 or 293 cells (Figures 3a and b), thus excluding an involvement of ADAR1 in this functional aspect. As a control for this assay, the extents of *EGR1* turnover were indistinguishable between control and ADAR1 knockdown cells (Figure 3c). Next, given that a possible mode of gene regulation imparted through 3′ UTR is the microRNA (miRNA)-mediated gene silencing, we tested whether 3′ UTR editing might alter miRNA complementary. Analysis of the known miRNA target sites in the *XIAP* and *MDM2* 3′ UTR revealed that editing sites were not distributed in the vicinity of these target regions (Figure 3d), indicating that their occurrence may not alter miRNA:3′ UTR complementarity. We then analyzed the association of RISC complex with these transcripts, which represents the overall output of miRNA targeting. AGO2-specific RNA-IP assay showed a clear enrichment of this miRNA-targeting mediator on the target RNAs (Figures 3e and f). However, no quantitative difference in abundance of *XIAP* and *MDM2* transcripts in the AGO2 immunoprecipitates was detected between control and ADAR1-depleted cells (Figures 3e and f), suggesting an inconsequential effect of ADAR1/editing on miRNA targeting.

**Figure 4:**
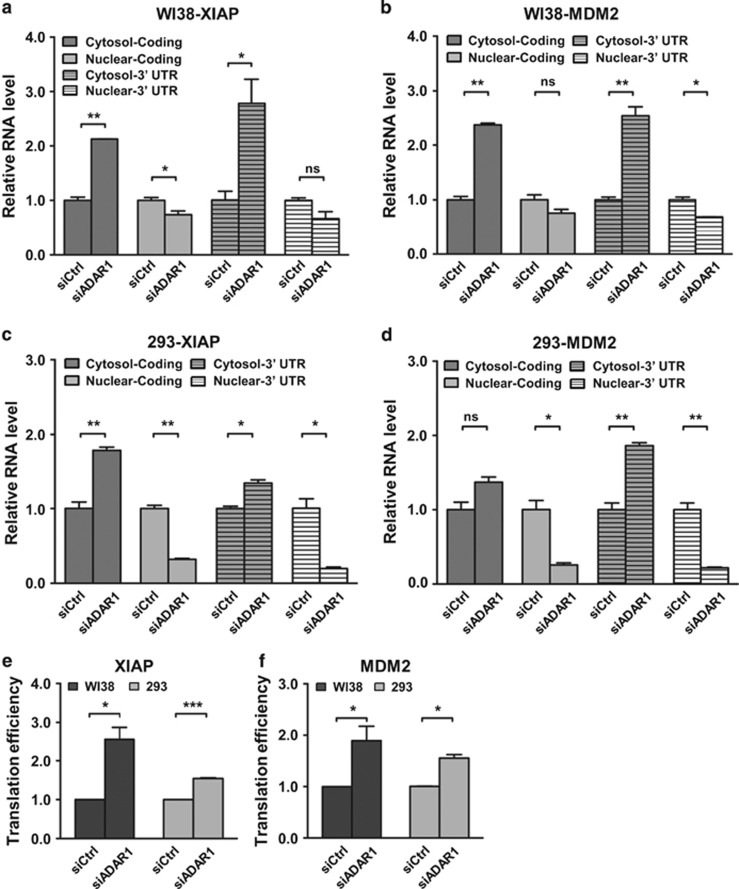
ADAR1 alters the nucleocytoplasmic transport of the *XIAP* and *MDM2* transcripts. (**a**–**d**) Control (siCtrl) and ADAR1 knockdown (siADAR1) WI38 (**a** and **b**) and 293 (**c** and **d**) cells were separated into nuclear and cytosolic fractions. Bar graphs show relative distribution of the indicated target transcripts (*XIAP* in **a** and **c**, *MDM2* in **b** and **d**) between the nuclear and cytosolic compartments, as assessed by real-time RT-PCR. Relative expression levels were normalized to the control group. Means±S.D. were calculated from three independent experiments (NS, not significant or *P*>0.05; *P<0.05; **P<0.01; ***P<0.001). (**e** and **f**) mRNA translation rate was measured by analyzing target RNA association with ribosome nascent-chains complex (RNC) (see Materials and methods). Relative RNA expression (*XIAP* in **e** and *MDM2* in **f**) was determined by real-time RT-PCR (*GAPDH* as the internal control), and normalized to the levels of control group. Data are presented as means±S.D.

Additionally, we also tested the possibility of spatial regulation of target RNAs’ localization in the cells. To this end, subcellular fractionation of RNA was performed, in which expression of marker genes detected by immunoblotting (Supplementary Figure S10) and qRT-PCR (Supplementary Figure S11) was used to confirm the efficiency of separation. Our subsequent real-time RT-PCR analysis results showed that, in the absence of ADAR1, levels of target transcripts in the nucleus relative to cytoplasmic compartment decreased notably (Figures 4a–d). Similar deviation in localization could also be seen for both the *XIAP* and *MDM2* transcripts in all the cell lines tested. Reduced nuclear distribution of target transcripts thus implies that ADAR1 may underlie targets’ subcellular localizations. Collectively, our data are consistent with the scenario that altered gene expression of *XIAP* and *MDM2* may arise post-transcriptionally, from ADAR1-dependent transcript re-distribution in the cells.

To provide further insight into the functional consequence, we next performed RNC-mRNA profiling^30^ and evaluated any differences in the levels of associated transcripts, which may be readout for expression output at the protein translation step. To this end, a significant elevation in the association of *XIAP* and *MDM2* mRNAs with the RNC fractions was observed in ADAR1 knockdown cells (Figures 4e and f, respectively), an indication that there was an increase in target protein translation rate in the absence of ADAR1. Therefore, ADAR1 might act as a negative regulator of the XIAP and MDM2 expression likely through modulating protein translation output.

This presumed role of ADAR1 protein in the spatial control of target transcripts implies that the consequent A-to-I(G) editing might also take part in influencing their subcellular distribution. To explore this possibility, we fractionated cells into different compartments – total, cytosol and RNC, and profiled these subcellular RNA editomes by using RNA-seq (Supplementary Tables S3 and S4). At comparable sequencing depths and mapping rates across all samples, we analyzed three distinct attributes of RNA editing. First, no discernable difference in the cytosolic *versus* RNC distributions of editing sites was detected, in terms of the size of RNA editome (Supplementary Figure S12a); however, RNA editing is more abundance in the total transcriptome. Second, to assess the possible impact of A-to-I(G) changes on the subcellular localization of edited transcripts, we determined their expression levels in all three subcellular transcriptomes. For this purpose, we also categorized genes with detected A-to-G changes into three groups: those with editing events found in both Cytosol and RNC counterparts, in Cytosol only, or in RNC only. On the basis of relative expression levels between fractions, we analyzed the distributions of inter-compartment expression ratios and subsequently found that they remained comparable for all three groups of the editing targets (Supplementary Figure S12b). Finally, when we further examined the editing sites associated with the *XIAP* and *MDM2* transcripts, there seemed no significant differences in the editing rate across the three fractions (Supplementary Figure S12c). This lack of compartment-biased distribution of RNA editing thus indicates that A-to-I(G) nucleotide changes *per se* may not underlie the subcellular transportation or incorporation into the polysomes of the target transcripts.

### Recruitment of the RNA shuttle protein STAU1 to 3′ UTR of editing targets is counteracted by ADAR1

**Figure 5:**
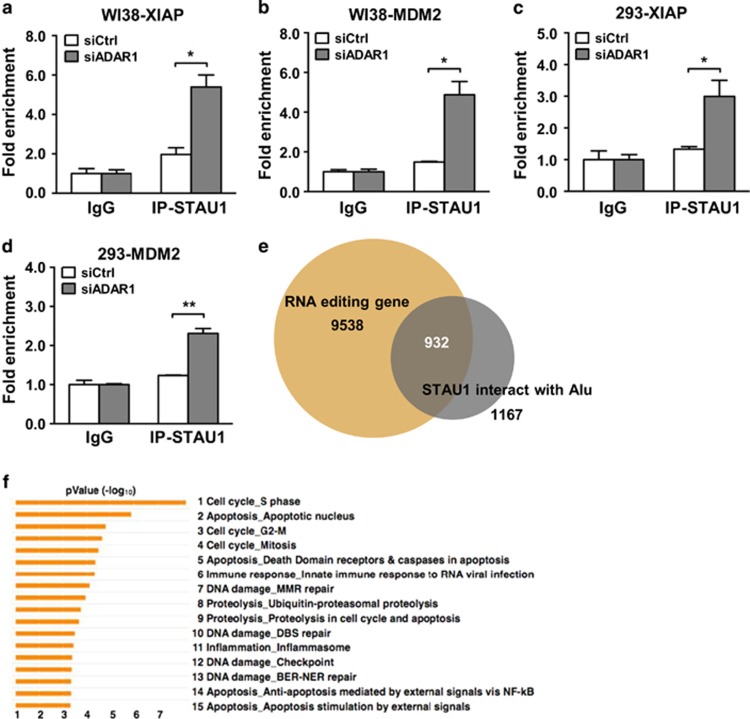
Competitive occupancy of target 3′ UTR by ADAR1 and the RNA transport regulator STAU1. (**a**–**d**) Extent of STAU1 association with target transcripts was assessed by RIP assay. Lysates prepared from control (siCtrl) and ADAR1-depleted (siADAR1) cells (WI38 in **a** and **b**, 293 in **c** and **d**) were immunoprecipitated by the control IgG or STAU1 (IP-STAU1) antibodies. Bound RNA corresponding to the indicated targets (*XIAP* in **a** and **c**, *MDM2* in **b** and **d**) was quantitatively determined by real-time RT-PCR. Data presented were normalized to the values of IgG in the control group and shown as means±S.D. (NS, not significant or *P*>0.05; *P<0.05; **P<0.01; ***P<0.001.) (**e**) The Venn diagram shows the degree of overlap between annotated RNA editing genes in the DARNED database and the known STAU1-interacting transcripts reported previously.^34^ (**f**) The 932 targets shared by the above two gene sets are enriched in distinct cell cycle and apoptosis processes, as revealed by bioinformatics analysis using MetaCore (*P*-value<0.05 and false discovery rate<0.05)

Having established that ADAR1’s regulatory role is through targeting 3′ UTR and controlling the localization of *XIAP* and *MDM2* transcripts, we next aimed to characterize how this regulation cross-talks with other post-transcriptional mechanisms. In this regard, previous studies have implicated STAU1, a regulator of RNA export, in the binding and shuttling of RNAs with the repetitive *Alu* elements (IR*Alus*) in 3′ UTR,^34^ which is a functional attribute shared by ADAR1. Interestingly, STAU1 is a negative regulator of PKR-mediated translation shutdown,^34^ and this functional antagonism is also mutually observed for ADAR1 in the context of virus infection. However, their roles in this respect might be different, owing to the seemingly distinct subcellular distributions of these proteins. In spite of these findings, it remains unresolved as to whether STAU1 interacts with 3′ UTR of these target RNAs. By using native RNA-IP, we detected marginal enrichment of STAU1 on the 3′ UTR of target transcripts (Figures 5a–d and Supplementary Figure S13). However, this occupancy of STAU1 on target transcripts elevated significantly in the absence of ADAR1, a change that is not attributable to STAU1 expression alteration in the knockdown cells (Supplementary Figure S14).

As another means to demonstrate the existence of the protein-3′ UTR complexes, we performed RNA pull-down assays. The *in vitro* transcribed transcripts corresponding to the *XIAP* 3′ UTR was used as the bait in the pull-down reaction with cell extracts. Western blot probing for the presence of proteins in the precipitated materials showed that ADAR1 were efficiently retained on the 3′ UTR (Supplementary Figure S15). We also detected a pull-down of STAU1, albeit to a lesser extent. Next, to further characterize the reciprocal transcript association between ADAR1 and STAU1 as observed above, we examined the binding of STAU1 in the absence of ADAR1. To this end, cell lysates were subjected to immunodepletion by the control or the ADAR1 antibody prior to the pull-down assay. The subsequent immunoblotting revealed the amount of recovered STAU1 notably enhanced upon ADAR1 depletion, implying that ADAR1 may impinge on the extent of STAU1 association with *XIAP* 3′ UTR sequence. Taken together, these results are suggestive of a competitive occupancy of these transcripts between ADAR1 and STAU1, balance of which may underlie the modulation of target RNA export and subsequent translation. Notably, we did not detect a co-precipitation of ADAR1 with STAU1 in a co-immunoprecipitation assay (Supplementary Figure S16), suggesting that this regulation does not involve a physical interaction between these two proteins.

The potentially antagonistic relationship between ADAR1 and STAU1 would imply a shared spectrum of target gene transcripts. To test this hypothesis, we examined publicly available large-scale data sets to determine whether there is intersection between STAU1-targeted transcripts and known editing targets. We subsequently found that the RNA interactome of STAU1 is considerably enriched in genes that undergo editing (Figure 5e). Moreover, gene ontology analyses of these mutual targets as well as the RNA interactome of STAU1 both revealed a preponderance of genes implicated in apoptosis (Figure 5f, Supplementary Table S5 and Supplementary Figure S17). Collectively, these observations provide further evidence for a regulatory antagonism between ADAR1 and STAU1 as well as its implication in the cellular response to apoptosis.

### ADAR1 contributes to cell apoptosis

**Figure 6:**
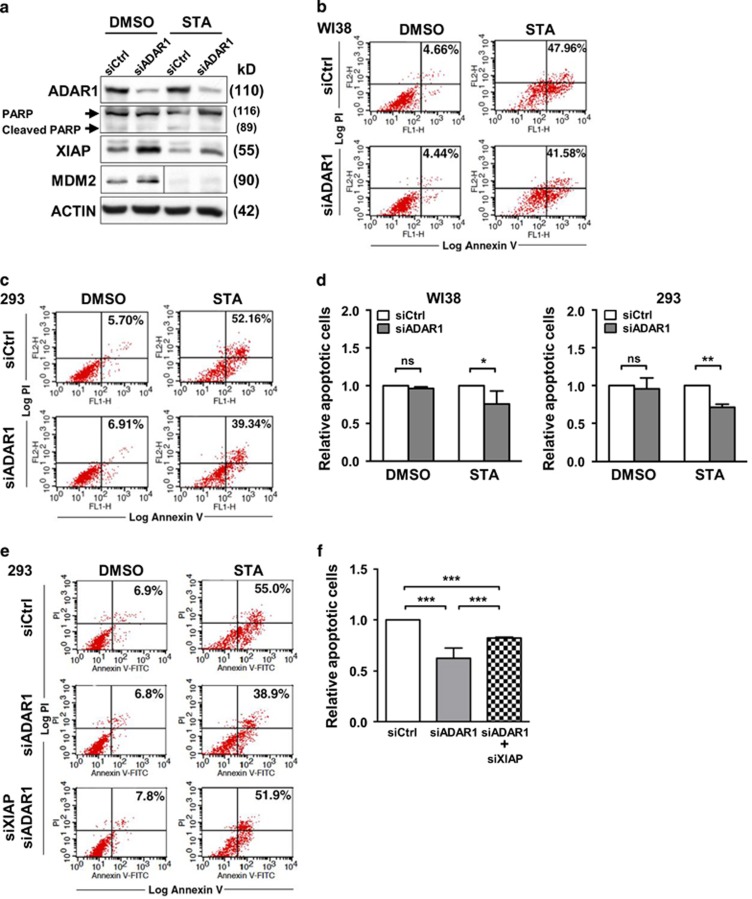
ADAR1 confers cell death protection under apoptosis signaling. (**a–d**) Role of ADAR1 in the cellular apoptotic response. For loss-of-function experiments, 293 or WI38 cells were transfected with siRNAs targeting ADAR1 (siADAR1) or GFP (siCtrl), and subsequently treated with 500 nM staurosporine (STA) for 4 h (**a**) and 12 h (**b**–**d**). Cells were harvested for either immunoblot analysis of the indicated proteins (293 cells; **a**) or flow cytometry-based apoptosis detection, with differential labeling of PI and Annexin V as the readout (see Materials and Methods) (WI38 in **b**, 293 in **c**). For the anti-MDM2 blots shown in (**a**), the control and treatment groups are of two different exposures. In (**b**) and (**c**), numbers shown in the upper right quadrant indicate the percentage of apoptotic cells. (**d**) Quantitative representation of extent of apoptosis for experiments shown in (**b**) and (**c**). Values were normalized to the control group and represented as means±S.D. (**e**) Cells harboring control (siCtrl) siRNAs or siRNAs targeting ADAR1 (siADAR1), or both ADAR1 and XIAP (siADAR1, siXIAP) were treated without or with STA and subjected to apoptosis detection by flow cytometry, which was performed as described above. (**f**) Quantitative representation of the results shown in (**e**), with columns and error bars representing means±S.D. of at least three independent experiments. Relative levels of apoptosis are shown as normalized to the Ctrl group (for statistical analyses shown in this figure: NS, not significant or *P*>0.05; *P<0.05; **P<0.01; ***P<0.001)

Upon establishing the role of ADAR1 in maintaining the proper expression of the apoptosis-related target genes, we next aimed to interrogate the cellular consequence of this regulation. Toward this end, we subjected control and ADAR1 knockdown WI38 and 293 cell lines to staurosporine, an apoptosis-inducing cytotoxic agent (Supplementary Figure S18), and monitored the extent of cell death. On the basis of PARP cleavage, a measurement of caspase 3 activity, we observed an induction of apoptosis upon drug treatment (Figure 6a). Ablation of ADAR1, which triggered upregulation of XIAP/MDM2 protein expression, reduced the levels of PARP cleavage. Using propidium iodide (PI)/Annexin V staining combined with flow cytometry analysis as a means to distinguish dying cells from viable cells, we also observed a discernable and reproducible alleviation of cell death in cells with ADAR1 knockdown (Figures 6b–d). To further strengthen the link of the ADAR1–XIAP regulatory axis to the process of apoptosis, we performed double knockdown experiments to assess the effect of XIAP downregulation on the ADAR1-mediated apoptotic response. While lowering ADAR1 expression reduced the levels of PI/Annexin V staining in the cells, concurrent knockdown of XIAP expression reversed this reduction and led to an apoptotic state comparable to that of the control cells (Figures 6e and f).

**Figure 7:**
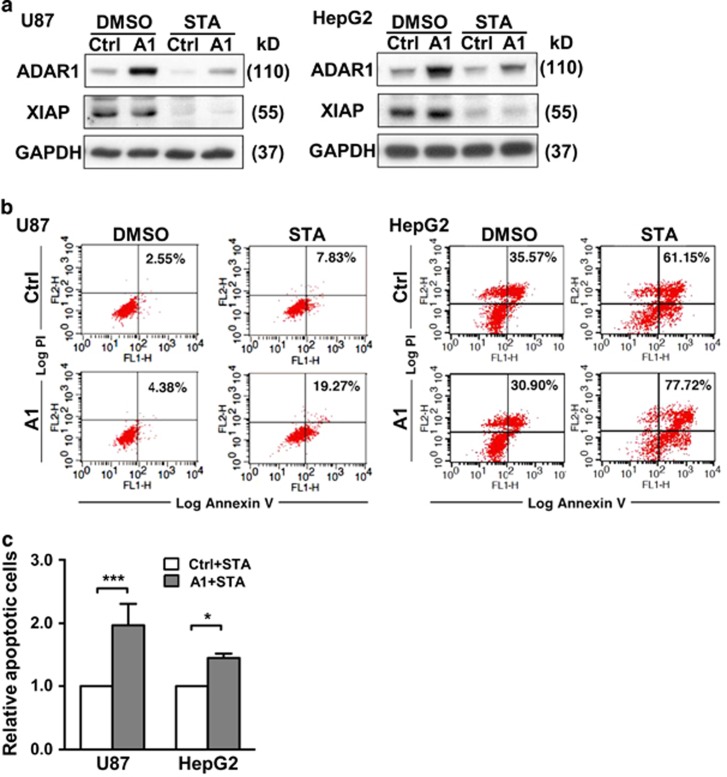
ADAR1 overexpression triggers enhanced apoptosis. (**a**) Western blot analysis was done on U87 (left) and HepG2 (right) cancer cell lines ectopically overexpressing ADAR1 and treated with STA. The indicated protein expression was examined, with GAPDH being the internal control. (**b**) The STA-treated cultures from (**a**) were analyzed for extent of apoptosis by flow cytometry. (**c**) Bar graph represents quantitatively the percentages of cell death in the STA-treated cultures of control or ADAR1-overexpressing cells, showing means±S.D. of at least three independent experiments. Apoptotic levels were normalized to the Ctrl group (for statistical analyses shown in this figure: NS, not significant or *P*>0.05; *P<0.05; **P<0.01; ***P<0.001)

Conversely, we also set out to examine the consequence of ADAR1 overexpression. Owing to the abundant expression of ADAR1 in cancer cell,^13^ we first screened available cells lines for low expressors (data not shown) and then selected U87 (glioblastoma) and HepG2 (hepatoma) cells for further interrogation. Upon introducing ectopic ADAR1 into these cell lines, we assessed the extent of staurosporine-induced cell death. While ADAR1 was effectively overexpressed, only U87 exhibited consistent downregulation of XIAP expression (Figure 7a). Correspondingly, we were able to see high levels of PI/Annexin V staining, and thus an augmented state of apoptosis, in ADAR1-overexpressing *versus* control U87 cells (Figures 7b, left and c). While the effect of ectopic ADAR1 on XIAP reduction was not evident in the HepG2 cells, a marginal but robust elevation in cell death was observed (Figures 7b, right and c). Viewed together, our results demonstrated that ADAR1 is directly involved in apoptosis regulation and that this functional aspect is mediated by modulating the expression of the apoptosis inhibitors XIAP and MDM2.

## Discussion

Up until about a decade ago, RNA editing had been a largely enigmatic component of the transcriptomes. Deep sequencing technologies have in the recent years advanced the scale, resolution, and efficiency with which RNA editomes are profiled and delineated.^18, 19, 20, 21, 22, 23^ Despite the consequent revelation of millions of A-to-I editing sites in mammals, only dozens of editing sites with recoding potential are hypothesized to modify hereditary information and functionally alter gene products.^21^ As opposed to the established biological relevance of several recoding events, much less is known about A-to-I(G) edits in the non-coding regions of gene transcripts, such as the 3′ UTR. To the short list of functionally characterized 3′ UTR editing events, we have added new important candidates with physiological implications. Our findings also illuminated a new functional aspect of RNA editing, which is to underpin the survival fitness of the cells by maintaining homeostasis in the survival *versus* death signaling.

Recent NGS-based editome profiling studies all pointed to a significant enrichment of editing events in the 3′ UTR of target transcripts, a strong indication that editing may be a functional determinant of 3′ UTR-associated transcript control, such as export, stability, miRNA targeting, etc. At the mechanistic level, while our results consequently disproved a role of ADAR1 in controlling either the stability or the miRNA targeting of the mRNA transcripts (Figure 3), they were indicative of a spatial regulation. In the context of transcript localization, RNA editing was previously shown to target aberrant and/or structured transcripts for retention in the nuclear compartment via a p54^nrb^-dependent mechanism.^33, 35^ However, results from reporter RNAs^36^ and a deep sequencing study on nuclear *versus* cytosolic editomes^37^ argue against the role of editing in nuclear retention of transcripts. These contrasting findings may be explained by the diverse distributions of editing sites relative to the sequence and/or structural motifs underlying transcript nucleocytoplasmic export, leading to differential effects on the export efficiency of the target transcripts. Alternatively, depending on the sites of editing and/or ADAR1 binding, they may affect to different extents the occupancy of RNA transport factors and consequently the transcript distribution. This potentially context-dependent regulation of transcript shuttling could be further resolved by using integrative analyses of editome sequences, export signals, as well as edited transcript localization.

Given that the dysregulation of cell death responses is directly linked to malignant transformation, our current findings of ADAR1’s apoptotic roles thus shed new light on the already extensive implications of ADAR1 in tumorigenesis. However, while a pro-growth role for this RNA editor have been speculated – by virtue of the generally upregulated abundance of ADAR1 in most cancer types,^13^ our present results are in line with a seemingly incongruent scenario, in which ADAR1 acts as a suppressor of apoptosis inhibitory factors (thus, a pro-apoptotic role). Incidentally, similarly growth-suppressive or tumor-suppressive functions have been ascribed to ADAR1 by recent studies, particularly in the contexts of melanoma^17, 38^ and glioblastoma.^39^ Intriguingly, there exists an important parallelism between these two disease types – reported tumor-associated reduction in ADAR expression attenuates editing of particular microRNAs, thus raising their tumorigenic potential. In this capacity, miRNA-455-5p and miRNA-376* are the edited miRNA targets associated respectively with melanoma and glioblastoma. In addition, overexpression of ADAR1 and ADAR2 in tumor cell lines derived from glioblastoma and astrocytoma was found to decrease tumor cell proliferation,^14, 16, 17^ a finding that is reconcilable with the pro-apoptotic effect of ectopic ADAR1 (Figure 7). Interestingly, Ma *et al.*^40^ found that, while expression of ADAR1 is upregulated in B-cell lymphoblastic leukemia, it is actually reversely correlated with clinical outcome of the disease. Taken together, these findings indicate that the tumorigenic role of ADAR1 may be disease-specific and context-dependent. They are further in support of the pro-apoptotic function evidenced by our current studies.

## Materials and methods

### Cell culture

HeLa and HepG2 cells were cultured in Dulbecco’s modified Eagle’s medium (DMEM). For HEK293 cells, DMEM was used with 1 × NEAA and 1 mM sodium pyruvate. U87 cells were cultured in DMEM/F12 with the addition of 2 mM l-glutamine. All media were supplemented with 10% heat-inactivated fetal bovine serum and 100 U/ml penicillin and streptomycin solution. All media and reagents were purchased from Thermo Fisher Scientific (Waltham, MA, USA). Cells were maintained at subconfluent densities in a 5% CO_2_ humidified incubator at 37 °C.

### Manipulation of endogenous ADAR1 abundance by RNAi and ectopic expression constructs

For transient knockdown of *ADAR1*, cells were transfected using Lipofectamine RNAiMAX (Invitrogen, Thermo Fisher Scientific) with a control siRNA (GFP-targeting) or a pool of two *ADAR1*-specific siRNAs. Twenty-five nucleotide-long siRNA duplexes (Stealth; Invitrogen) were designed targeting different regions of the mRNA. For shRNAs-mediated RNAi, corresponding sequences were cloned in pSUPER vectors for expressing the short-hairpin form. For transient overexpression experiments, the control pcDNA3.1 vector and plasmids encoding the wild-type ADAR1, a dominant-negative enzymatically-deficient variant (A1DN), or an Myc-tagged XIAP cDNA were used. Ectopic constructs were delivered by using the Lipofectamine 2000 reagent (Invitrogen).

### Reagents and antibodies

All chemicals were purchased from Sigma (St. Louis, MO, USA), except where otherwise indicated. Mouse monoclonal antibodies raised against ADAR1, ADAR2, MDM2, cleaved form of PARP, GAPDH and ACTIN were obtained from Santa Cruz Biotechnology (Santa Cruz, CA, USA). STAU1 antibodies were from GeneTex (Irvine, CA, USA). Anti-XIAP rabbit polyclonal and anti-AGO2 antibodies were purchased from Abcam (Cambridge, MA, USA). Secondary antibodies used in the western blot assays were from Vector Laboratories (Burlingame, CA, USA).

### RNA isolation, reverse transcription (RT)-PCR and real-time PCR

Total RNAs were isolated from cells and purified by the TRIzol reagent (Invitrogen), then subsequently reverse transcribed into complementary DNA (cDNA) by MMLV (Invitrogen) using random hexamers. To confirm candidate editing sites, selected fragments were amplified by specific primers using end-point PCR, gel purified, and subsequently sequenced by the Sanger method. For the quantitative determination of the target genes, cDNA samples were analyzed by real-time PCR using the Bio-Rad iQ5 Gradient Real Time SYBR-Green PCR system. Levels of cDNA were normalized to the GAPDH values of the respective samples. All results represent mean±S.D. of at least three experiments. Sequences of primers used for the Sanger sequencing, RT-PCR or real-time PCR assays are listed in Supplementary Table S1. Our real-time PCR experiments were carried out in compliance with the MIQE (Minimum Information for Publication of Quantitative Real-Time PCR Experiments) guidelines,^29^ as shown in the MIQE checklist (Supplementary Table S2).

### Nuclear and cytoplasmic fractionation

Cells were washed once with PBS and lysed with the nuclear fractionation buffer containing 10 mM Tris-HCl, pH 7.4, 10 mM NaCl, 0.2% NP-40, 3 mM MgCl_2_, protease inhibitor (Roche, Basel, Switzerland), 100 U/ml RNaseOUT, 10 mM sodium pyrophosphate, 2 mM sodium orthovanadate and 1 mM sodium fluoride for 10 min at 4 °C. Cells were centrifuged at 800 × *g* for 5 min at 4 °C. The supernatant was removed and used as the cytoplasmic fraction. The pellet was washed once with nuclear fractionation buffer and centrifuged again at 800 × *g* for 10 min at 4 °C. The pellet was used as the nuclear fraction. Cytoplasmic and nuclear fractions were controlled using GAPDH and ADAR2 as respective markers.

### 3′ UTR reporter construct and luciferase reporter assay

To generate 3′ UTR luciferase reporter, PCR fragment corresponding to the invertedly oriented tandem *Alu* elements in the human *XIAP* and *MDM2* 3′ UTRs was subcloned into pMIR-REPORT luciferase reporter vector (Thermo Fisher Scientific). For reporter assay, HeLa cells were seeded in six-well plates before being co-transfected with the reporter construct and the indicated expression vectors of ADAR1 or ADAR1-targeting siRNAs, by using respectively Lipofectamine 2000 or RNAiMAX. After 2-day incubation, cells were harvested in Reporter Lysis 5 × Buffer (Promega) according to the manufacturer’s instructions. Firefly luciferase activity was measured by a Dual-Luciferase Reporter Assay System (Promega), which was normalized to absorption units of the co-expressed *β*-gal to yield relative light units.

### Isolation of cytosolic ribosome nascent chain (RNC)

Characterization of the extent of transcript association with translating polysomes was performed based on RNC extraction outlined by a previous report.^30^ Briefly, upon pre-treatment with 100 mg/ml cycloheximide for 15 min, cells were lysed in 1% Triton X-100 in ribosome buffer (RB buffer) (20 mM HEPES-KOH (pH 7.4), 15 mM MgCl_2_, 200 mM KCl, 100 mg/ml cycloheximide, 2 mM dithiothreitol and 100 U/ml RNaseOUT). Cell debris was removed by centrifuging at 16 200 × *g* for 10 min at 4 °C. The cytosol RNCs were isolated by sedimentation through a 30% sucrose solution (30% sucrose, 20 mM HEPES/KOH, pH 7.4, 15 mM MgCl_2_, 200 mM KCl, 2 mM dithiothreitol (DTT), 100 *μ*g/ml cycloheximide) in a Ti-70 rotor (Beckman Coulter, Fullerton, CA, USA) at 185 000 × *g* for 5 h at 4 °C. The samples were then subjected to RNA purification by TRIzol reagent and reverse transcription. Abundance of target gene transcripts in total RNA and RNC fractions was quantitatively determined by real-time PCR analysis as described above and normalized to that of GAPDH expression in the respective samples. The translation rate was denoted as the normalized representation of the RNC fraction in the total target mRNA pool (RNC/total).

### RNA immunoprecipitation (RIP)

Native RIP was conducted largely as described previously.^31^ In brief, cells were washed and then harvested by scraping in ice-cold Polysomal Lysis Buffer (100 mM KCl, 5 mM MgCl_2_, 10 mM HEPES (pH 7.0), 0.5% NP-40, 1 mM DTT, 50 U/ml RNaseOUT (Invitrogen), protease inhibitor cocktail (Roche)). Cell suspension was pass through 27G needle eight times to promote lysis. After centrifugation (12 000 × *g*, 15 min, 4 °C), total lysates were collected and subsequently pre-cleared with magnetic protein-G beads (Invitrogen) at 4 °C for 1 h. Immunoprecipitation was performed by adding the normal IgG, ADAR1, AGO2 and STAU1 antibodies to cleared lysate at 4 °C overnight. Magnetic protein-G beads were then added to each IP sample and rotated for 1 h at 4 °C. The beads were pelleted and washed with Polysomal Lysis Buffer. After several washes, 10 U DNase I (Fermentas, Thermo Fisher Scientific) and 10 × reaction buffer was added and incubated at 37 °C for 15 min to remove all contaminating DNA. Then, 1 ml Trizol reagent was added to the beads and the RNA was extracted according to the manufacturer’s protocol. Finally, the immunoprecipitated RNAs were reverse transcribed to cDNA and analyzed by real-time PCR, primers of which are listed in Supplementary Table S1.

### Apoptosis assay

To induce apoptosis, cells were subjected overnight to staurosporine treatment at the indicated concentrations. Cells were collected, washed and suspended in 100 *μ*l Annexin V binding buffer at 5 × 10^5^ cells/ml, and incubated at room temperature for 15 min with 5 *μ*l of Alexa Fluor 488-labeled Annexin V and 1 *μ*l of 100 *μ*g/ml propidium iodide (Strong Biotech Corp, Taipei, Taiwan). The cells were then mixed with 400 *μ*l of medium on ice. The fractions of apoptotic or dead cells in indicated culture were determined using a flow cytometer (Beckman Coulter, Inc.).

### Statistical analysis

Data are presented as means with error bars indicating the standard deviation (S.D.). Student’s *t*-test was used to determine the statistical significance of quantitative comparisons. Degrees of statistical significance (NS, not significant; *P<0.05; **P<0.01; ***P<0.001) are indicated in the respective figure legends.
