# Supplementary material for: ADAR1-mediated 3′ UTR editing and expression control of antiapoptosis genes fine-tunes cellular apoptosis response
Source: Cell Death Dis. 2017 May 25;8(5):e2833–. doi: 10.1038/cddis.2017.12 (PMC5520689; doi:10.1038/cddis.2017.12)
Supplement: Supplementary Information [file cddis201712x1.pdf]

Supplementary Information

Materials and Methods

RNA pull-down assay

Preparation of the transcript bait and the immuno-depleted cell extracts was done similarly as a previous study[1]. Briefly, for *in vitro* synthesis of biotinylated transcripts corresponding to the *XIAP* 3' UTR, PCR-generated templates with chimeric T7 RNA polymerase promoter sequence were used with in conjunction with the AmpliScribe T7-Flash Biotin-RNA Transcription Kit (EPICENTRE; WI, USA). To remove endogenous ADAR1 from the 293 cell extracts, two rounds of immunodepletion were performed by each incubating extract supernatants with anti-ADAR1 primary antibody and subsequently with addition of protein G-agarose beads. Control depletion was performed using pre-immune rabbit IgG. For the pull-down assay, the immunodepleted extracts were first precleared with streptavidin Sepharose (Invitrogen), in the presence of SUPERase-In (Ambion) and yeast tRNA (Sigma). After centrifugation, 2 μg of *in vitro* transcribed biotinylated RNA was added to the supernatant, from which the protein-biotinylated RNA complexes were subsequently recovered by addition of streptavidin Sepharose and analyzed by SDS-PAGE and western blot. All steps in the immunodepletion and pull-down procedures were carried out at 4℃.

RNA sequencing

Cells were first subjected to nuclear, cytosolic, and RNC fractionation as described above, from which RNA was isolated using TRIzol reagent. cDNA libraries were prepared based on the TruSeq® Stranded Total RNA Sample Preparation Guide (Illumina, Part # 15031048). Equal concentrations of each library were sequenced using a NextSeq 500 (Illumina) platform to create pair-end 75-bp reads.

Gene expression analysis

All RNA reads were mapped to hg19 with STAR 2.5.2b[2]. The statistics obtained for the RNA-Seq reads are shown in Supplementary Table RNASEQ. The expression levels of genes in the three samples and the corresponding fold changes were estimated by DESeq2 1.14.1[3] with GENCODE V19 annotation[4].

RNA editing calling and analysis

To identify RNA editing events in RNA-Seq data, we adapted a previously described RNA-Seq read mapping approach[5]. We used the BWA mem program of BWA 0.7.15[6] to map RNA-Seq reads to a database that combined the hg19 reference genome and all annotated splicing junctions. Particularly, exonic sequences containing known splicing junctions derived from annotations of GENCODE[4], RefSeq, Ensembl archive 75, and UCSC hg19 KnownGene were merged into the index database to prevent simultaneous hits to exonic sequences that encompass splicing junction regions and the reference genome. For the 76-bp RNA-Seq reads studied in this work, an upstream 75 bp and another downstream 75 bp (1 bp shorter than the length of RNA-Seq reads) of exonic sequences surrounding a known splicing junction were extracted. We included more exon sequences to extend the regions to the desired length if the neighboring exons of a known splicing junction were shorter than 75 bp. For each mapped bam file, we used MarkDuplicates of Picard 1.136 to remove duplicate reads that mapped to the same location. Only uniquely mapped reads with a qualified mapping quality will be used for further analysis. In this study, we did not apply BLAT search in this search. In addition to the filtering criteria used in the previous study[5], we further excluded editing sites within 2 bp of low-quality base or indel regions, and removed sites within a homopolymer sequence.

MTT assay

Mitochondrial dehydrogenase activity, which reduces 3-(4, 5-di-methyithiazol-2-yl)-2, 5-diphenyl-tetra-zolium bromide (MTT), serves as a measure of redox activity and thus an indicator of cell viability. Cells were seeded in 96-well culture plate and treated with STA for 12 hrs. MTT reagent (Sigma) was added to culture medium for 4-hour incubation. As living cells metabolize MTT reagent into purple formazan, cell viability was determined by dissolving the precipitates by DMSO and subsequently quantifying by spectrophotometry at 570 nm.

Supplementary Tables

Supplementary Table S1. Primers used in this study

Primers	Sequence (5' to 3')	Application	Amplicon length
XIAP-1-Forwrad<br>XIAP-1-Reverse	AGCATTAAGGTTGGTGCAAAA<br>CAAGGTCTTGCACTGTCACC	Sanger sequencing	307 bp
XIAP-2-Forward<br>XIAP-2-Reverse	CTCACTGTGTTGCCCAGGAT<br>ACCGCGAAACCAGTCACTAC	Sanger sequencing	270 bp
XIAP-3-Forward<br>XIAP-3-Reverse	GATGGAGTCTTGCTTGTCACC<br>GCAGACAGTGCAAAGGTGAA	Sanger sequencing	384 bp
MDM2-1-Forward<br>MDM2-1-Reverse	TTGGAAAACAATGTATGGGTAGAA<br>ACACCCATCAGCTCAAATATCAT	Sanger sequencing	261 bp
MDM2-2-Forward<br>MDM2-2-Reverse	GGTAATTCTGCACAGCAACTTT<br>TTCTGCTTGGTTGTAGCTGAA	Sanger sequencing	166 bp
XIAP-3' UTR-Forward<br>XIAP-3' UTR-Reverse	TGCAAGTGGCAAAACACTATG<br>CTCCCCGAAGAGAAACCACAT	RNA-IP	125 bp
MDM2-3' UTR-Forward<br>MDM2-3' UTR-Forward	AGTGAGAAAATGCCTCAATTCACA<br>GGAGTTGGTGTAAAGGATGAGC	RNA-IP	127 bp
qPCR-XIAP-Coding-Forward<br>qPCR-XIAP-Coding-Reverse	TACCGTGCGGTGCTTTAGTT<br>TTTGTAGACTGCGTGGCACT	Real-time PCR	144 bp
qPCR-XIAP-3' UTR-Forward<br>qPCR-XIAP-3' UTR-Reverse	CATGTGAAATGTTTTGAGACAGAGT<br>GCCAGATTATACAGCAGAAAGCA	Real-time PCR	118 bp
qPCR-MDM2-Coding-Forward<br>qPCR-MDM2-Coding-Reverse	ATGAAAGCCTGGCTCTGTGT<br>AAGATCCGGATTCGATGGCG	Real-time PCR	92 bp
qPCR-MDM2-3' UTR-Forward<br>qPCR-MDM2-3' UTR-Reverse	AGTGTGTAGGTCTGTAGGCT<br>TCCACCCATAAAGCGCAACT	Real-time PCR	85 bp

Supplementary Table S2. MIQE checklist for quantitative real-time PCR

ITEM TO CHECK	IMPORTANCE	CHECKLIST
**EXPERIMENTAL DESIGN**		
Definition of experimental and control groups	**E**	Control group were defined as treated with control plasmid or siRNA, and DMSO treatment served as control group to STA challenge
Number within each group	**E**	Three independent experiments repeat
Assay carried out by core lab or investigator's lab?	D	Investigator's lab
Acknowledgement of authors' contributions	D	
**SAMPLE**		
Description	**E**	Analyzed samples were complementary DNA from treated cells
Volume/mass of sample processed	D	
Microdissection or macrodissection	**E**	No
Processing procedure	**E**	Cells were treated with siRNA, plasmids, or STA challenge, and Trizol reagent was used to isolate RNA. Complementary DNA was obtained from reverse transcription of purified RNA
If frozen - how and how quickly?	**E**	Not frozen
If fixed - with what, how quickly?	**E**	Not fixed
Sample storage conditions and duration (especially for FFPE samples)	**E**	-20℃
**NUCLEIC ACID EXTRACTION**		
Procedure and/or instrumentation	**E**	RNAs were isolated by the TRIzol reagent (Invitrogen)
Name of kit and details of any modifications	**E**	RNA isolation procedures were referred to manuals and protocols form Trizol reagent (Invitrogen)
Source of additional reagents used	D	
Details of DNase or RNAse treatment	**E**	3 μg RNA was treated with RNase-free DNase (Promega) in 20 μl reaction at 37℃ for 30 minutes. The enzyme inactivation was done by heating at 65℃ for 10 minutes.
Contamination assessment (DNA or RNA)	**E**	RT-minus PCR reaction was done to assess genomic DNA contamination
Nucleic acid quantification	**E**	RNA concentration was quantified by NanoDrop 2000
Instrument and method	**E**	NanoDrop 2000 spectrophotometer (Thermo Fisher Scientific)
Purity (A260/A280)	D	A260/A280 ratio was about 1.80-2.00
Yield	D	
RNA integrity method/instrument	**E**	Agilent 2100 Bioanalyzer
RIN/RQI or Cq of 3' and 5' transcripts	**E**	RIN value must be greater than 9
Electrophoresis traces	D	
Inhibition testing (Cq dilutions, spike or other)	**E**	Not applicable
**REVERSE TRANSCRIPTION**		
Complete reaction conditions	**E**	RNA and DEPC-treated water were added to 10 μl volume, and 1μl 10 mM dNTP and 1μl 250 ng random hexamer were added to mixture. The mixture was heated at 65℃ for 5 minute and chilled down on ice. The sample was mixed with 4 μl 5X first-strand buffer, 1 μl 0.1M DTT, and 1 μl RNase OUT (Invitrogen), and the mixture was incubated at 37℃ for 2 minute. 1 μl reverse transcriptase was added into mixture and reverse transcription was carried out as in the later section
Amount of RNA and reaction volume	**E**	Amount of RNA was 3 μg RNA and reaction volume was 20 μl
Priming oligonucleotide (if using GSP) and concentration	**E**	250 ng random hexamer was added in per reaction
Reverse transcriptase and concentration	**E**	200U reverse transcriptase was added to per reaction
Temperature and time	**E**	Reverse transcription was carried out by PCR machine, and reaction procedures as following: 10 min at 25℃, 37 min at 50 ℃, and 15 min at 70℃.
Manufacturer of reagents and catalogue numbers	D	
Cqs with and without RT	D*	
Storage conditions of cDNA	D	-20℃
**qPCR TARGET INFORMATION**		
If multiplex, efficiency and LOD of each assay.	**E**	Not applicable
Sequence accession number	**E**	XIAP (NM_001167.3) and MDM2 (NM_002392.5)
Location of amplicon	D	Coding sequence region (EX) and 3' untranslated region (3' UTR) of target gene
Amplicon length	**E**	As described in supplementary table 1

In silico specificity screen (BLAST, etc)	**E**	Nucleotide BLAST (NCBI) was used to screen primer specificity
Pseudogenes, retropseudogenes or other homologs?	D	
Sequence alignment	D	
Secondary structure analysis of amplicon	D	
Location of each primer by exon or intron (if applicable)	**E**	Primer sequences were exonic regions and 3' UTR.
What splice variants are targeted?	**E**	qPCR primer was used to detect the common regions of the splice variants
**qPCR OLIGONUCLEOTIDES**		
Primer sequences	**E**	As described in supplementary table 1
RTPrimerDB Identification Number	D	
Probe sequences	D**	
Location and identity of any modifications	**E**	No modifications
Manufacturer of oligonucleotides	D	
Purification method	D	RPC
**qPCR PROTOCOL**		
Complete reaction conditions	**E**	Complementary DNA (cDNA), qPCR primer, 2x iQ™ SYBR Green Supermix, and distilled water were mixed to 10 μl reaction volume. The analysis programs were described in the following part
Reaction volume and amount of cDNA/DNA	**E**	25 ng cDNA at 10 μl reaction
Primer, (probe), Mg++ and dNTP concentrations	**E**	250 nM primers and 2x iQ™ SYBR Green Supermix (Bio-Rad) containing 6 mM $MgCl_2$ and dNTPs (0.4 mM each of dATP, dTTP, dCTP, and dGTP) was used to qPCR reaction
Polymerase identity and concentration	**E**	Final iTaq™ DNA polymerase concentration was 0.25U/μl
Buffer/kit identity and manufacturer	**E**	Bio-Rad iQ5 Gradient Real Time SYBR-Green PCR system
Exact chemical constitution of the buffer	D	
Additives (SYBR Green I, DMSO, etc.)	**E**	2x iQ™ SYBR Green Supermix (Bio-Rad)
Manufacturer of plates/tubes and catalog number	D	
Complete thermocycling parameters	**E**	Initial heating denature at 95℃ for 3 minute, then 40 cycle for target detection: 95℃ for 10 second and 60℃ for 30 second; in the final, melting curve analysis was performed by gradient heating from 60℃to 95℃ for 10 minutes.
Reaction setup (manual/robotic)	D	
Manufacturer of qPCR instrument	**E**	Bio-Rad
**qPCR VALIDATION**		
Evidence of optimisation (from gradients)	D	
Specificity (gel, sequence, melt, or digest)	**E**	PCR amplicons generated from non-template control (NTC) and cDNA reactions were confirmed by 2% agarose gel electrophoresis, and products from NTC were not detected. Melting curve analysis was performed by gradient heating from 60℃to 95℃ and fluorescence intensity was detected continuously. Melting temperature of individual qPCR primer was calculated and confirmed the specificity.
For SYBR Green I, Cq of the NTC	**E**	Cq value of NTC was higher than 35 or undetected (N/A)
Standard curves with slope and y-intercept	**E**	**GAPDH**: slope=-3.502 and y-intercept=21.642<br>**U48**: slope=-3.858 and y-intercept=23.879<br>**7SL**: slope=-3.544 and y-intercept=23.229<br>**XIAP-Coding**: -3.538 and y-intercept=28.250<br>**XIAP-3' UTR**: slope=-3.463 and y-intercept=26.846<br>**MDM2-Coding**: slope=-3.430 and y-intercept=27.439<br>**MDM2-3' UTR**: slope=-3.277 and y-intercept=29.778
PCR efficiency calculated from slope	**E**	**GAPDH**: 93.0% **U48**: 81.6% **7SL**: 91.5% **XIAP-Coding**: 91.7%<br>**XIAP-3' UTR**: 94.4% **MDM2-Coding**: 95.7% **MDM2-3' UTR**: 101.9%
Confidence interval for PCR efficiency or standard error	D	
r2 of standard curve	**E**	**GAPDH**: 0.990 **U48**: 0.984 **7SL**: 0.993<br>**XIAP-Coding**: 0.981 **XIAP-3' UTR**: 0.988<br>**MDM2-Coding**: 0.988 **MDM2-3' UTR**: 0.986
Linear dynamic range	**E**	On the basis of the r2 value of standard curve of each target gene, the linear dynamic range was considered applied to

		1:1000 dilution of cDNA
Cq variation at lower limit	**E**	Cq value of GAPDH, U48, and 7SL at lower were about 30.0 and Cq variation was less than 0.5. Meanwhile, Cq value of XIAP and MDM2 (Coding or 3' UTR) at lower were more than 32.0 and Cq variation was about 1.0.
Confidence intervals throughout range	D	
Evidence for limit of detection	**E**	Regression curves displayed the lowest detection limit was 1:1000 dilution of cDNA
If multiplex, efficiency and LOD of each assay.	**E**	Not applicable
**DATA ANALYSIS**		
qPCR analysis program (source, version)	**E**	CFX Manager^{TM} Software Version 3.1
Cq method determination	**E**	The Cq value was calculated based on the threshold method
Outlier identification and disposition	**E**	No Cq value was removed
Results of NTCs	**E**	The Cq values for NTCs were higher than 35, and the melting curve of NTCs were disorganized
Justification of number and choice of reference genes	**E**	GAPDH expression served as reference genes; U48 and 7SL expression were specifically used as reference genes for nuclear and cytosolic fraction
Description of normalisation method	**E**	CFX Manager^{TM} Software Version 3.1 (Bio-Rad)
Number and concordance of biological replicates	D	
Number and stage (RT or qPCR) of technical replicates	**E**	qPCR analysis was performed at duplicate
Repeatability (intra-assay variation)	E	The standard error of the mean at duplicate repeat was smaller than 0.2
Reproducibility (inter-assay variation, %CV)	D	
Power analysis	D	
Statistical methods for result significance	**E**	Student's t-test was used to determine the statistical significance of quantitative comparisons.
Software (source, version)	E	CFX Manager^{TM} Software Version 3.1 (Bio-Rad)
Cq or raw data submission using RDML	**D**	

Supplementary Table S3. Statistics of RNA-Seq experiments and analyses (see Excel file)

Supplementary Table S4. List of A-toI(G) RNA editing sites called by RNA-Seq (see Excel file)

Supplementary Table S5. Apoptosis-related pathways overrepresented in the STAU1-interacting editing target gene set*

#2: Apoptotic nucleus	#5: Death domain receptor and caspase in apoptosis	#14: Anti-apoptosis mediated by external signals via NF-kB	#15: Apoptosis stimulation by external signals
APAF1 (68)	APAF1 (68)	CASP8 (58)	CASP8 (58)
ATM (208)	CARD8 (128),	CASP9 (26)	CASP8AP2 (107)
ATR (129)	CASP6 (23)	IRAK1 (5)	CASP9 (26)
CASP6 (23)	CASP8 (58)	IRAK2 (68)	CASP10 (46)
CASP8 (58)	CASP8AP2 (107)	PPP2R4 (23)	MAPK8 (21)
CASP9 (26)	CASP9 (26)	PRKACA (11)	MAPK9 (31)

CASP10 (46)	CASP10 (46)	SHC1A (11)	MAPK10 (3)
CUL5 (285)	DAP3 (257)	TP53 (39)	SHC1A (11)
CYCS (82)	DFFA (81)	TRADD (2)	TNFRSF10A (12)
DFFA (81)	RAD51 (41)	TRAF6 (20)	TP53 (39)
HIST1H2BD (1)	TNFRSF10A (12)	XIAP (309)	TRADD (2)
HIST1H2BK (20)	TRADD (2)		XIAP (309)
ITGB3BP (91)	XIAP (309)		
MDM4 (363)			
RAD51 (41)			
RNF7 (15)			
STK4 (182)			
TP53 (39)			
TUBA1A (2)			
VHL (226)			

*Numbers in parentheses denote the sums of A-to-I(G) events annotated in the DARNED database.

Supplementary Figures & Legends

Supplementary Figure S1

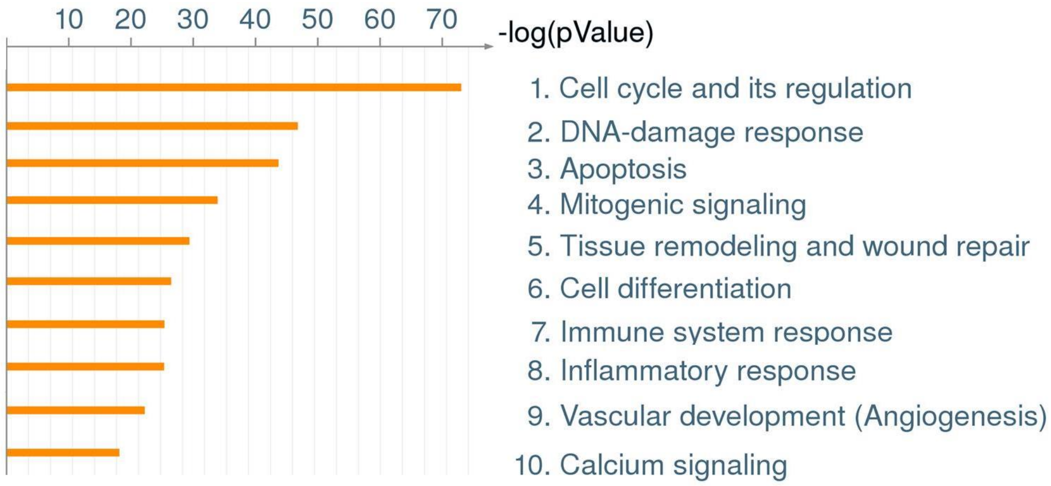

Figure S1. Pathway enrichment analysis of RNA editing genes annotated by both our previous RNA-seq data and the DARNED database. Enrichment was based on the MetaCore analysis (*P*-value < 0.05 and False Discovery Rate < 0.05).

Supplementary Figure S2

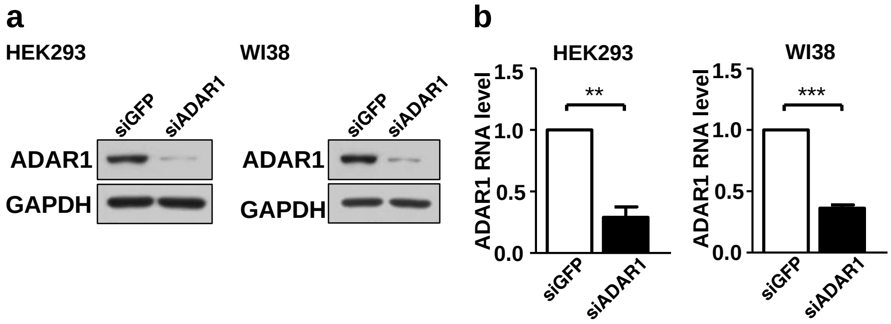

Figure S2. Confirmative profiling of ADAR1 protein and mRNA expression in transfected cells shown in Figure 1, a & b. (a) Whole cell extracts were prepared from HEK293 and WI38 cells transfected with ADAR1-targeting siRNA (siADAR1) and control siRNA (siGFP) (shown in Figure 1, a & b). Immunoblotting was performed to analyze ADAR1 protein expression, with GAPDH as the loading control. (b) RNA expression in the samples shown in (a) was measured by RT-qPCR, and the relative ADAR1 expression was normalized to GAPDH abundance.

Supplementary Figure S3

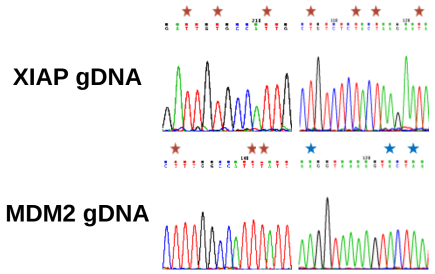

Figure S3. Sanger sequencing traces of the genomic DNA region corresponding to the editing target sites in the WI38 cells. This was done to exclude potential sequence polymorphism at RNA editing positions. Sites marked by the red stars were sequenced in the reverse orientation, while those marked by blue stars were in the forward orientation.

Supplementary Figure S4

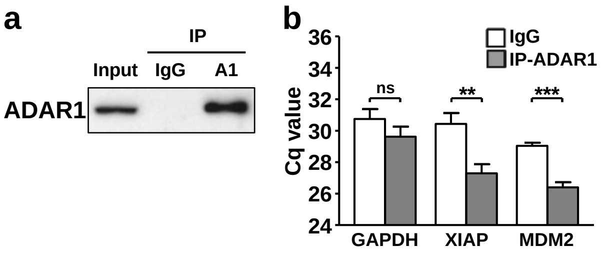

Figure S4. Independent verification of the anti-ADAR1 RIP experiments shown in Figure 1c. (a) Whole cell extract (Input) from cells was immunoprecipitated (IP) with ADAR1 antibody (A1) or control antibody (IgG) and subjected to immunoblotting analysis for ADAR1 protein expression. Input represents direct loading equivalent to one eightieth of lysate used in IP. (b) Alternative representative of the real-time RT-PCR results shown in Figure 1c. (ns, not significant; **P<0.01; ***P<0.001)

Supplementary Figure S5

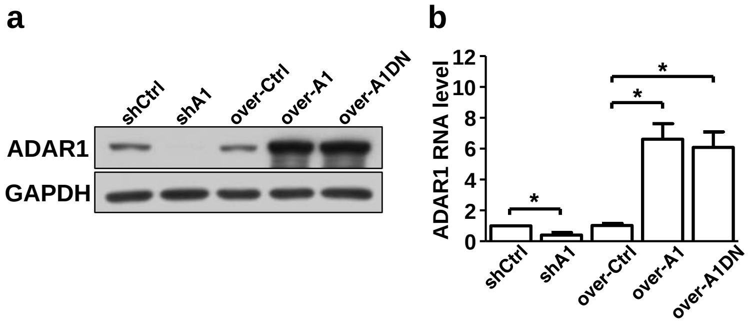

Figure S5. Confirmative profiling of ADAR1 protein and mRNA expression in transfected cells shown in Figure 1d. (a) Whole cell extracts were prepared from HEK293 cells transfected with the indicated plasmids, as shown in Figure 1d. Immunoblotting was performed to analyze ADAR1 protein expression. GAPDH expression serves as loading control. (b) RNA expression in the samples shown in (a) was measured by RT-qPCR, and the relative ADAR1 expression was normalized to GAPDH abundance. (For statistical analyses shown in this figure: ns, not significant or P>0.05; *P<0.05.)

Supplementary Figure S6

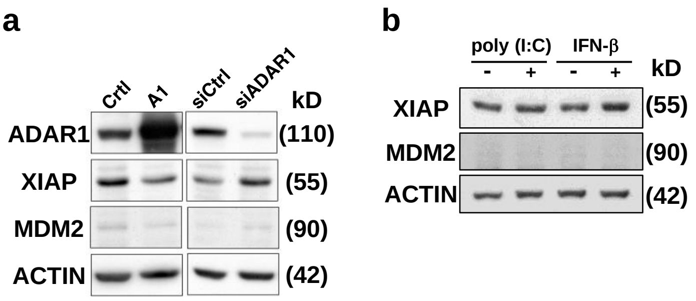

Figure S6. Effect of ADAR1 mis-expression (a) or interferon signaling (b) on the XIAP and MDM2 protein levels. (a) HeLa cells were transfected with empty (Ctrl) or ADAR1 (A1) expression vector (left panel), or siRNAs targeting control (siCtrl) or ADAR1 (siADAR1) sequences (right panel). In (b), HeLa cells were transfected with poly(I:C) or treated with IFN-β (for 6 hr). Cell lysates were harvested for immunoblotting analysis of the indicated protein expression. Expression of ACTIN is presented as the internal control.

Supplementary Figure S7

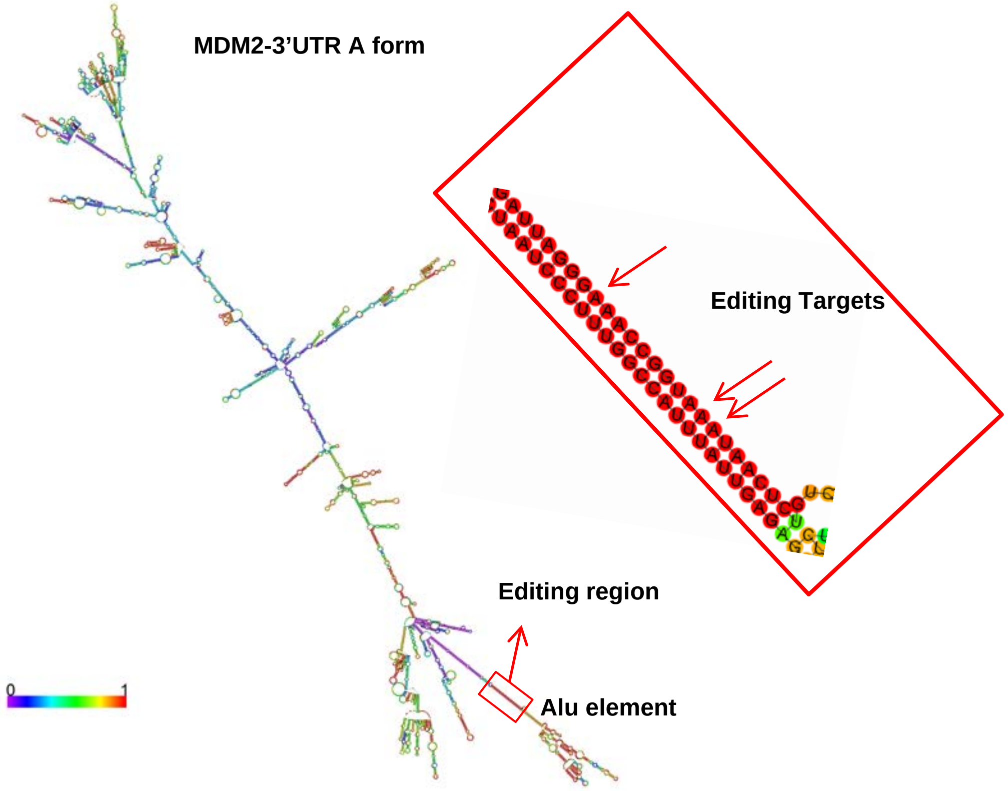

Figure S7. Putative secondary structure of the *MDM2* 3' UTR, as predicted by the RNAfold Webserver. Double-stranded region corresponding to the transcribed IR*Alus* is denoted by the red box, with a magnified view of the boxed area shown. RNA editing sites are indicated by by blue arrows. Colors of the nucleotides correspond to base-pair probabilities, based on the color scale bar. Sequences corresponding to this particular stem loop, with the embedded IR*Alus*, were sub-cloned into the 3' UTR reporter plasmid.

Supplementary Figure S8

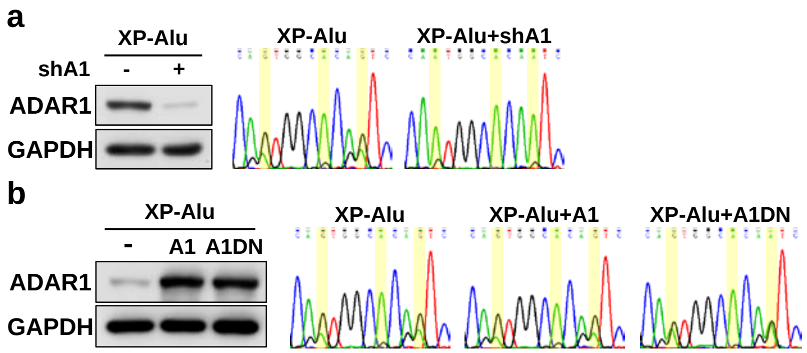

Figure S8. Protein expression and editing status in *XIAP* 3'UTR reporter experiments shown in Figure 2, f & g. Western blotting analysis for verifying ADAR1 protein expression was done for the knockdown (a) and overexpression (b) experiments. Sanger sequencing analysis of *XIAP* RNA editing was also performed on the RNA samples prepared from the same experiments and accordingly shown on the right.

Supplementary Figure S9

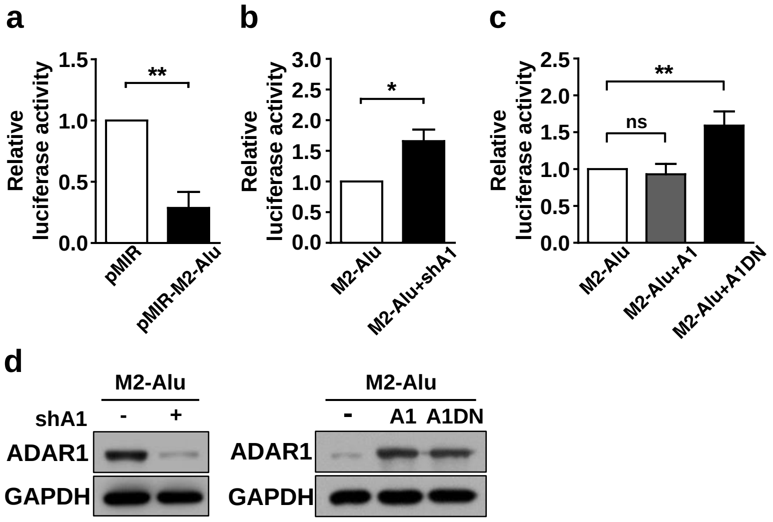

Figure S9. Role of ADAR1 on *MDM2* 3' UTR. (a) 3' UTR reporter assay was conducted on cells transfected with control empty vector (pMIR vector) or the construct containing IR*Alus* derived from MDM2 3' UTR (pMIR-M2-Alu). Luciferase activity was detected after 48 hr and normalized to the co-expressed β-gal levels, with controls being represented as 1. (b & c) 3' UTR reporter assay was done as in (a), except with the additional co-transfection of expression plasmids for ADAR1-targeting shRNAs (shA1) (b), or the wild-type form of ADAR1 (A1) and the dominant-negative mutant (A1DN) (c). (d) ADAR1 expression in the knockdown and overexpression experiments, respectively shown in (b) and (c), was monitored by western blotting analysis, with GAPDH as the loading control. (For statistical analyses shown in this figure: ns, not significant or $P>0.05$; $^*P<0.05$; $^{**}P<0.01$.)

Supplementary Figure S10

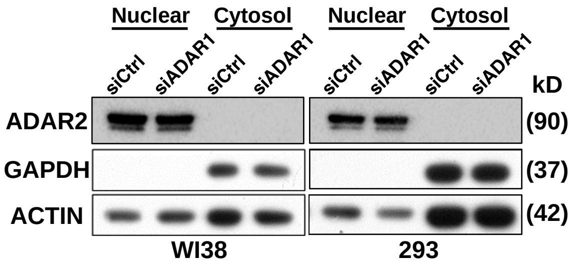

Figure S10. Controls for the subcellular fractionation experiments. Control (siCtrl) and ADAR1 knockdown (siADAR1) WI38 (left) and 293 (right) cells were separated into nuclear and cytosolic fractions. Proteins from both fractions were probed with antibodies against GAPDH and ADAR2, respectively as the cytosolic and nuclear markers.

Supplementary Figure S11

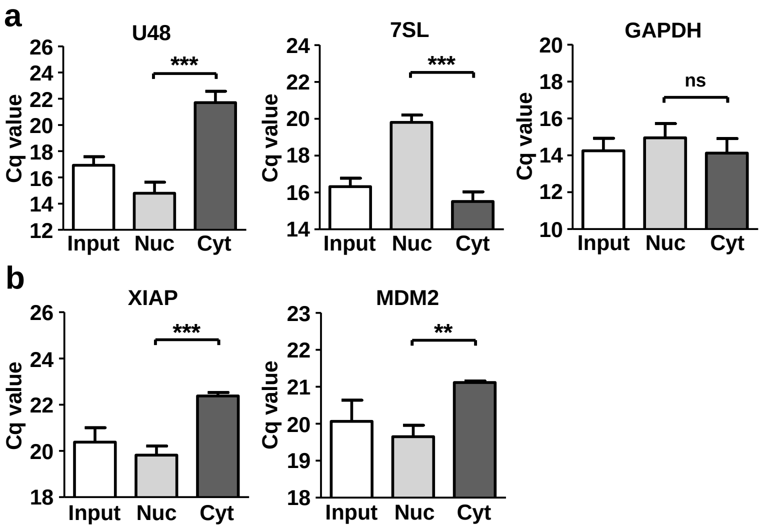

Figure S11. Target RNA expression in the nuclear and cytosolic fractions. Cells were separated into nuclear and cytosolic fractions. Bar graphs show the transcript levels (in Cq) as assessed by real-time RT-PCR, and correspond to relative distribution of the indicated target transcripts (U48, 7SL, and GAPDH in a; XIAP and MDM2 in b) between the nuclear and cytosolic compartments, as well as the total RNA (Input). Means±S.D. were calculated from three independent experiments (ns, not significant; **P<0.01; ***P<0.001).

Supplementary Figure S12

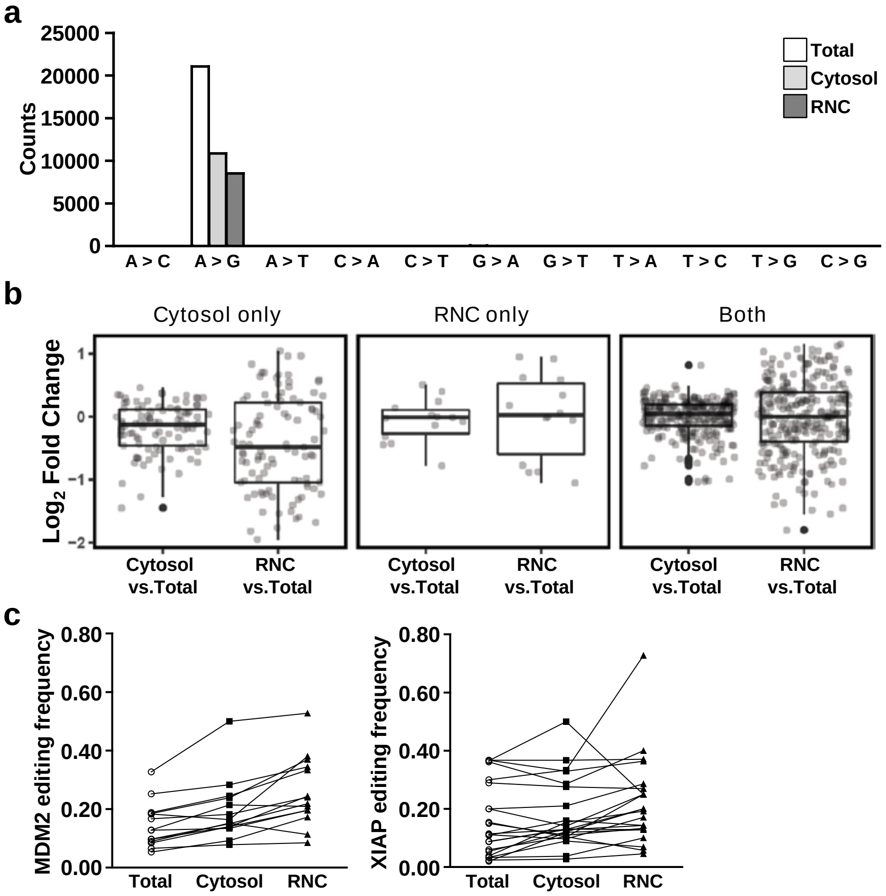

Figure S12. RNA sequencing-based profiling of subcellular RNA editomes. (a) RNA-centric variants were profiled from the RNA-seq data according to the procedure outlined in the Materials and Methods. Distributions of the different types of nucleotide substitution across the three sub-cellular transcriptomes (Cytosol, RNC: Ribosome Nascent Chain, and Total) are shown, with A-to-G variation being the most predominant event. (b) Genes with detected A-to-G changes were categorized into three groups: with editing events found in both Cytosol and RNC counterparts (both), in Cytosol only, or in RNC only. Relative expression levels between different subcellular transcriptomes (Cytosol vs. Total or RNC vs. Total) were then determined for each gene. Boxplots depict the distributions of inter-compartment expression ratios for the three groups of editing targets. (c) For the A-to-G RNA editing events detected by RNA-seq in the *XIAP* and *MDM2* genes, the editing ratio was determined based on the relative representation of the G nucleotide sequence at any given site. The distributions of editing ratios across three subcellular transcriptomes are plotted in the line curve graphs.

Supplementary Figure S13

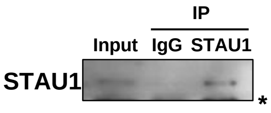

Figure S13. Independent immunoblotting verification of the anti-STAU1 RIP experiments shown in Figure 5, a to d. Whole cell extract (Input) from cells was immunoprecipitated (IP) with STAU1 antibody (STAU1) or control antibody (IgG) and subjected to immunoblotting analysis for STAU1 protein expression. Input represents direct loading equivalent to one eightieth of lysate used in IP. The asterisk denotes non-specific signals.

Supplementary Figure S14

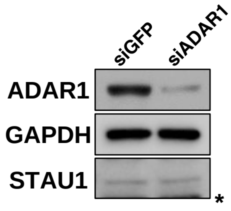

Figure S14. STAU1 expression in ADAR1 knockdown cells. For the RIP experiments shown in Figure 5, the control (siGFP) and knockdown (siADAR1) cell lysates used in the immunoprecipitation reaction were also subjected to western blotting for monitoring expression levels of ADAR1 and STAU1, as indicated. GAPDH expression serves as loading control. The asterisk denotes non-specific signals.

Supplementary Figure S15

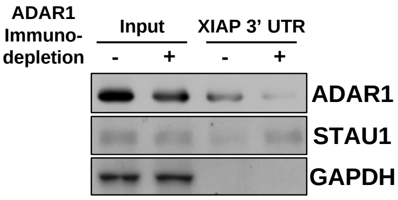

Figure S15. *XIAP* 3' UTR RNA pull-down assay. RNA pull-down assay was carried out using *in vitro* transcribed, biotinylated RNA transcripts corresponding to a fragment of *XIAP* 3' UTR that contains the embedded IR*Alus* (see Supplementary Materials and Methods). To examine the role of ADAR1 on the association of STAU1 with the 3' UTR, HEK293 cell lysates prior to pull-down reaction were subjected to immunodepletion using control IgG (−) or ADAR1-specific (+) antibodies. Precipitated proteins were visualized by immunoblotting using the indicated antibodies. Input represents direct loading equivalent to 1/50 of the lysates used in pull-down.

Supplementary Figure S16

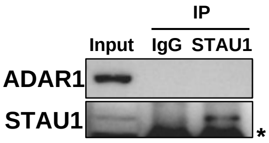

Figure S16. Co-immunoprecipitation analysis of STAU1 and ADAR1. Whole cell extracts were prepared from cells and immunoprecipitated (IP) by the anti-STAU1 antibody (STAU1) or control antibody (IgG). Immunoprecipitated complexes were resolved and analyzed by western blotting for ADAR1 and STAU1. Input corresponds to direct loading of whole cell extract that equals one eightieth of the protein used in immunoprecipitation. The asterisk denotes non-specific signals.

Supplementary Figure S17

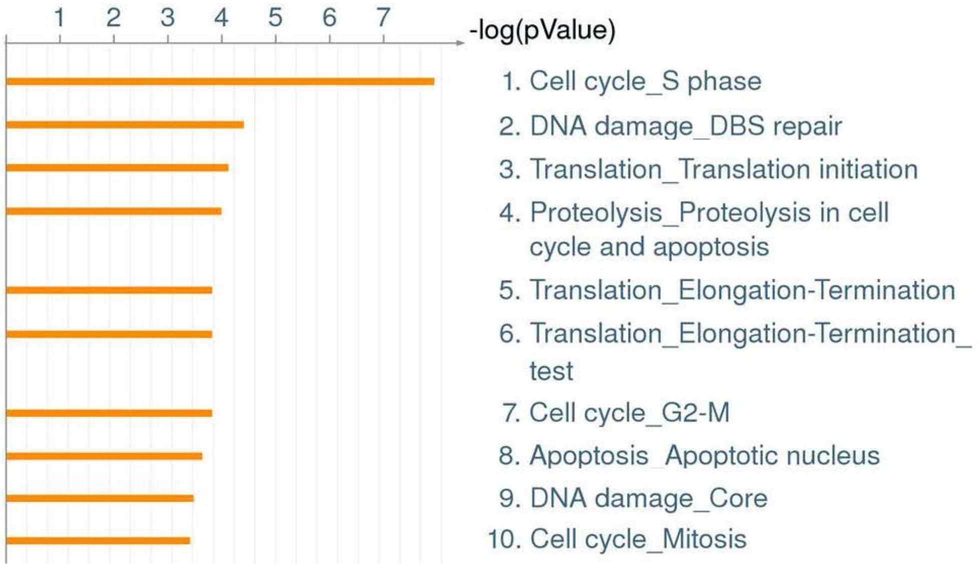

Figure S17. Pathway enrichment analysis of the STAU1-interacting RNAs. Enrichment was based on the MetaCore analysis (*P*-value < 0.05 and False Discovery Rate < 0.05).

Supplementary Figure S18

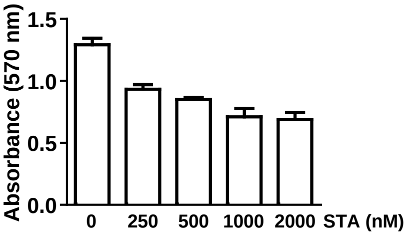

Figure S18. Cell viability at different doses of STA treatment. HEK293 cells were treated with different doses of STA and the cellular cytotoxicity was measured based on the MTT assay.

Reference

1. Hsieh CL, Lin CL, Liu H, Chang YJ, Shih CJ, Zhong CZ*, et al.* WDHD1 modulates the post-transcriptional step of the centromeric silencing pathway. *Nucleic acids research* 2011, **39**(10)**:** 4048-4062.

2. Dobin A, Davis CA, Schlesinger F, Drenkow J, Zaleski C, Jha S*, et al.* STAR: ultrafast universal RNA-seq aligner. *Bioinformatics* 2013, **29**(1)**:** 15-21.

3. Love MI, Huber W, Anders S. Moderated estimation of fold change and dispersion for RNA-seq data with DESeq2. *Genome Biol* 2014, **15**(12)**:** 550.

4. Harrow J, Frankish A, Gonzalez JM, Tapanari E, Diekhans M, Kokocinski F*, et al.* GENCODE: the reference human genome annotation for The ENCODE Project. *Genome Res* 2012, **22**(9)**:** 1760-1774.

5. Ramaswami G, Lin W, Piskol R, Tan MH, Davis C, Li JB. Accurate identification of human Alu and non-Alu RNA editing sites. *Nat Methods* 2012, **9**(6)**:** 579-581.

6. Li H. Aligning sequence reads, clone sequences and assembly contigs with BWA-MEM. *ArXiv e-prints*; 2013. p. 3997.
