# Supplementary material for: ADAR1-mediated 3′ UTR editing and expression control of antiapoptosis genes fine-tunes cellular apoptosis response
Source: Cell Death Dis. 2017 May 25;8(5):e2833–. doi: 10.1038/cddis.2017.12 (PMC5520689; doi:10.1038/cddis.2017.12)
Supplement: Supplementary Table 3 [file cddis201712x2.pdf]

Supplementary Table 3 Statistics of RNA-Seq experiments and analyses

Sample type	Read length	# of reads	Uniquely mapped reads %	% of reads mapped to multiple loci	# of mapped genes	# of called RNA editing sites	# of called sites overlap with DARNED
Total	2 * 76 bp	100,353,778	92.43%	3.50%	25,898	61,355	21,052
Cytosol	2 * 76 bp	91,069,916	90.87%	3.70%	24,199	31,531	10,862
RNC	2 * 76 bp	83,006,798	93.31%	4.05%	22,520	23,377	8,526
