# Supplementary material for: ADAR1-mediated 3′ UTR editing and expression control of antiapoptosis genes fine-tunes cellular apoptosis response
Source: Cell Death Dis. 2017 May 25;8(5):e2833–. doi: 10.1038/cddis.2017.12 (PMC5520689; doi:10.1038/cddis.2017.12)
Supplement: Supplementary Table 4 [file cddis201712x3.pdf]

Supplementary Table S4 List of A-toI(G) RNA editing sites called by RNA-Seq

id	chrom	pos	pos2	ref	alt	location	gene	annotation	ann1	ann2	ref	alt	rnastrand	genestrand	editing.freq	acgt	known	sample	Mismatch Pattern
chr1:662647:T:C:intergenic:RP5-8	chr1	662647	662647	T	C	intergenic	RP5-857K21.	dist=2717;di	.	.	A	G	-	-	0.85714286	1,0,6,0	chr1,662646,	Cytosol	A > G
chr1:1252654:A:G:intronic:CPSF3L	chr1	1252654	1252654	A	G	intronic	CPSF3L	.	.	.	A	G	+	-	0.22222222	14,0,4,0	chr1,125265	Cytosol	A > G
chr1:1252786:G:A:intronic:CPSF3L	chr1	1252786	1252786	G	A	intronic	CPSF3L	.	.	.	G	A	+	-	0.3	3,0,7,0	chr1,125278	Cytosol	G > A
chr1:1253335:T:C:ncRNA_intronic:	chr1	1253335	1253335	T	C	ncRNA_intro	RP5-890O3.9	.	.	.	A	G	-	-	0.4	3,0,2,0	chr1,125333	Cytosol	A > G
chr1:1253343:T:C:ncRNA_intronic:	chr1	1253343	1253343	T	C	ncRNA_intro	RP5-890O3.9	.	.	.	A	G	-	-	0.33333333	4,0,2,0	chr1,125334	Cytosol	A > G
chr1:1253357:T:C:ncRNA_intronic:	chr1	1253357	1253357	T	C	ncRNA_intro	RP5-890O3.9	.	.	.	A	G	-	-	0.8	1,0,4,0	chr1,125335	Cytosol	A > G
chr1:1253376:T:C:ncRNA_intronic:	chr1	1253376	1253376	T	C	ncRNA_intro	RP5-890O3.9	.	.	.	A	G	-	-	0.4	3,0,2,0	chr1,125337	Cytosol	A > G
chr1:1418494:A:G:intronic:ATAD3	chr1	1418494	1418494	A	G	intronic	ATAD3B	.	.	.	A	G	+	+	0.25	6,0,2,0	chr1,141849	Cytosol	A > G
chr1:1418532:A:G:intronic:ATAD3	chr1	1418532	1418532	A	G	intronic	ATAD3B	.	.	.	A	G	+	+	0.23076923	10,0,3,0	chr1,141853	Cytosol	A > G
chr1:1418536:A:G:intronic:ATAD3	chr1	1418536	1418536	A	G	intronic	ATAD3B	.	.	.	A	G	+	+	0.23076923	10,0,3,0	chr1,141853	Cytosol	A > G
chr1:1419690:A:G:intronic:ATAD3	chr1	1419690	1419690	A	G	intronic	ATAD3B	.	.	.	A	G	+	+	0.3	7,0,3,0	chr1,141968	Cytosol	A > G
chr1:1419700:A:G:intronic:ATAD3	chr1	1419700	1419700	A	G	intronic	ATAD3B	.	.	.	A	G	+	+	0.44444444	5,0,4,0	chr1,141969	Cytosol	A > G
chr1:1419746:A:G:intronic:ATAD3	chr1	1419746	1419746	A	G	intronic	ATAD3B	.	.	.	A	G	+	+	0.6	2,0,3,0	chr1,141974	Cytosol	A > G
chr1:1594977:T:C:UTR3:SLC35E2B	chr1	1594977	1594977	T	C	UTR3	SLC35E2B	ENST000003	.	.	A	G	-	-	0.16666667	10,0,2,0	chr1,159497	Cytosol	A > G
chr1:1595006:T:C:UTR3:SLC35E2B	chr1	1595006	1595006	T	C	UTR3	SLC35E2B	ENST000003	.	.	A	G	-	-	0.45238095	23,0,19,0	chr1,159500	Cytosol	A > G
chr1:1595074:T:C:UTR3:SLC35E2B	chr1	1595074	1595074	T	C	UTR3	SLC35E2B	ENST000003	.	.	A	G	-	-	0.07142857	65,0,5,0	chr1,159507	Cytosol	A > G
chr1:1595586:T:C:UTR3:SLC35E2B	chr1	1595586	1595586	T	C	UTR3	SLC35E2B	ENST000003	.	.	A	G	-	-	1	0,0,7,0	chr1,159558	Cytosol	A > G
chr1:1660887:T:C:ncRNA_intronic:	chr1	1660887	1660887	T	C	ncRNA_intro	RP1-283E3.4	.	.	.	A	G	-	+-	0.66666667	3,0,6,0	chr1,166088	Cytosol	A > G
chr1:1660888:T:C:ncRNA_intronic:	chr1	1660888	1660888	T	C	ncRNA_intro	RP1-283E3.4	.	.	.	A	G	-	+-	0.33333333	6,0,3,0	chr1,166088	Cytosol	A > G
chr1:1660916:T:C:ncRNA_intronic:	chr1	1660916	1660916	T	C	ncRNA_intro	RP1-283E3.4	.	.	.	A	G	-	+-	0.42857143	4,0,3,0	chr1,166091	Cytosol	A > G
chr1:1660917:T:C:ncRNA_intronic:	chr1	1660917	1660917	T	C	ncRNA_intro	RP1-283E3.4	.	.	.	A	G	-	+-	0.5	4,0,4,0	chr1,166091	Cytosol	A > G
chr1:1661047:T:C:ncRNA_intronic:	chr1	1661047	1661047	T	C	ncRNA_intro	RP1-283E3.4	.	.	.	A	G	-	+-	0.6	2,0,3,0	chr1,166104	Cytosol	A > G
chr1:1661053:T:C:ncRNA_intronic:	chr1	1661053	1661053	T	C	ncRNA_intro	RP1-283E3.4	.	.	.	A	G	-	+-	0.6	2,0,3,0	chr1,166105	Cytosol	A > G
chr1:1661099:T:C:ncRNA_intronic:	chr1	1661099	1661099	T	C	ncRNA_intro	RP1-283E3.4	.	.	.	A	G	-	+-	0.42857143	4,0,3,0	chr1,166109	Cytosol	A > G
chr1:1662652:T:C:ncRNA_exonic:	chr1	1662652	1662652	T	C	ncRNA_exon	RP1-283E3.4	.	.	.	A	G	-	+-	0.33333333	4,0,2,0	chr1,166265	Cytosol	A > G
chr1:1662694:T:C:ncRNA_exonic:	chr1	1662694	1662694	T	C	ncRNA_exon	RP1-283E3.4	.	.	.	A	G	-	+-	0.6	2,0,3,0	chr1,166269	Cytosol	A > G
chr1:1662704:T:C:ncRNA_exonic:	chr1	1662704	1662704	T	C	ncRNA_exon	RP1-283E3.4	.	.	.	A	G	-	+-	0.4	3,0,2,0	chr1,166270	Cytosol	A > G
chr1:1662708:T:C:ncRNA_exonic:	chr1	1662708	1662708	T	C	ncRNA_exon	RP1-283E3.4	.	.	.	A	G	-	+-	0.33333333	4,0,2,0	chr1,166270	Cytosol	A > G
chr1:1784018:T:C:intronic:GNB1	chr1	1784018	1784018	T	C	intronic	GNB1	.	.	.	A	G	-	-	0.125	14,0,2,0	chr1,178401	Cytosol	A > G
chr1:2300601:T:C:intronic:MORN1	chr1	2300601	2300601	T	C	intronic	MORN1	.	.	.	A	G	-	-	1	0,0,6,0	chr1,230060	Cytosol	A > G
chr1:2302209:T:C:intronic:MORN1	chr1	2302209	2302209	T	C	intronic	MORN1	.	.	.	A	G	-	-	0.66666667	2,0,4,0	chr1,230220	Cytosol	A > G
chr1:3729217:T:C:UTR3:CEP104	chr1	3729217	3729217	T	C	UTR3	CEP104	ENST000003	.	.	A	G	-	-	0.25925926	20,0,7,0	chr1,372921	Cytosol	A > G
chr1:3729857:T:C:UTR3:CEP104	chr1	3729857	3729857	T	C	UTR3	CEP104	ENST000003	.	.	A	G	-	-	0.41666667	7,0,5,0	chr1,372985	Cytosol	A > G
chr1:3729920:T:C:UTR3:CEP104	chr1	3729920	3729920	T	C	UTR3	CEP104	ENST000003	.	.	A	G	-	-	0.53846154	6,0,7,0	chr1,372991	Cytosol	A > G
chr1:3729921:T:C:UTR3:CEP104	chr1	3729921	3729921	T	C	UTR3	CEP104	ENST000003	.	.	A	G	-	-	0.23076923	10,0,3,0	chr1,372992	Cytosol	A > G
chr1:3729922:T:C:UTR3:CEP104	chr1	3729922	3729922	T	C	UTR3	CEP104	ENST000003	.	.	A	G	-	-	0.81818182	2,0,9,0	chr1,372992	Cytosol	A > G
chr1:3729930:T:C:UTR3:CEP104	chr1	3729930	3729930	T	C	UTR3	CEP104	ENST000003	.	.	A	G	-	-	0.15384615	11,0,2,0	chr1,372992	Cytosol	A > G
chr1:3729953:T:C:UTR3:CEP104	chr1	3729953	3729953	T	C	UTR3	CEP104	ENST000003	.	.	A	G	-	-	0.5	6,0,6,0	chr1,372995	Cytosol	A > G
chr1:3730158:T:C:UTR3:CEP104	chr1	3730158	3730158	T	C	UTR3	CEP104	ENST000003	.	.	A	G	-	-	0.71428571	2,0,5,0	chr1,373015	Cytosol	A > G
chr1:3730302:T:C:UTR3:CEP104	chr1	3730302	3730302	T	C	UTR3	CEP104	ENST000003	.	.	A	G	-	-	0.2	8,0,2,0	chr1,373030	Cytosol	A > G
chr1:3730305:T:C:UTR3:CEP104	chr1	3730305	3730305	T	C	UTR3	CEP104	ENST000003	.	.	A	G	-	-	0.44444444	5,0,4,0	chr1,373030	Cytosol	A > G
chr1:3730327:T:C:UTR3:CEP104	chr1	3730327	3730327	T	C	UTR3	CEP104	ENST000003	.	.	A	G	-	-	0.82352941	3,0,14,0	chr1,373032	Cytosol	A > G
chr1:3730331:T:C:UTR3:CEP104	chr1	3730331	3730331	T	C	UTR3	CEP104	ENST000003	.	.	A	G	-	-	0.58823529	7,0,10,0	chr1,373033	Cytosol	A > G
chr1:3730351:T:C:UTR3:CEP104	chr1	3730351	3730351	T	C	UTR3	CEP104	ENST000003	.	.	A	G	-	-	0.06666667	28,0,2,0	chr1,373035	Cytosol	A > G
chr1:3730400:T:C:UTR3:CEP104	chr1	3730400	3730400	T	C	UTR3	CEP104	ENST000003	.	.	A	G	-	-	0.89473684	4,0,34,0	chr1,373039	Cytosol	A > G
chr1:6279950:A:G:UTR3:RNF207	chr1	6279950	6279950	A	G	UTR3	RNF207	ENST000003	.	.	A	G	+	+	0.22222222	7,0,2,0	chr1,627994	Cytosol	A > G
chr1:6282224:T:C:UTR3:ICMT	chr1	6282224	6282224	T	C	UTR3	ICMT	ENST000003	.	.	A	G	-	-	0.8125	6,0,26,0	chr1,628222	Cytosol	A > G
chr1:6282236:T:C:UTR3:ICMT	chr1	6282236	6282236	T	C	UTR3	ICMT	ENST000003	.	.	A	G	-	-	0.52941176	16,0,18,0	chr1,628223	Cytosol	A > G
chr1:6282238:T:C:UTR3:ICMT	chr1	6282238	6282238	T	C	UTR3	ICMT	ENST000003	.	.	A	G	-	-	0.12121212	29,0,4,0	chr1,628223	Cytosol	A > G
chr1:6282301:T:C:UTR3:ICMT	chr1	6282301	6282301	T	C	UTR3	ICMT	ENST000003	.	.	A	G	-	-	0.5	11,0,11,0	chr1,628230	Cytosol	A > G
chr1:6282315:T:C:UTR3:ICMT	chr1	6282315	6282315	T	C	UTR3	ICMT	ENST000003	.	.	A	G	-	-	0.17241379	24,0,5,0	chr1,628231	Cytosol	A > G
chr1:6282318:T:C:UTR3:ICMT	chr1	6282318	6282318	T	C	UTR3	ICMT	ENST000003	.	.	A	G	-	-	0.2	24,0,6,0	chr1,628231	Cytosol	A > G
chr1:6282324:T:C:UTR3:ICMT	chr1	6282324	6282324	T	C	UTR3	ICMT	ENST000003	.	.	A	G	-	-	0.5862069	12,0,17,0	chr1,628232	Cytosol	A > G
chr1:6282383:T:C:UTR3:ICMT	chr1	6282383	6282383	T	C	UTR3	ICMT	ENST000003	.	.	A	G	-	-	0.5	24,0,24,0	chr1,628238	Cytosol	A > G
chr1:6282400:T:C:UTR3:ICMT	chr1	6282400	6282400	T	C	UTR3	ICMT	ENST000003	.	.	A	G	-	-	0.43181818	50,0,38,0	chr1,628239	Cytosol	A > G
chr1:6282401:T:C:UTR3:ICMT	chr1	6282401	6282401	T	C	UTR3	ICMT	ENST000003	.	.	A	G	-	-	0.46236559	50,0,43,0	chr1,628240	Cytosol	A > G
chr1:6282420:T:C:UTR3:ICMT	chr1	6282420	6282420	T	C	UTR3	ICMT	ENST000003	.	.	A	G	-	-	0.09615385	94,0,10,0	chr1,628241	Cytosol	A > G
chr1:6282587:T:C:UTR3:ICMT	chr1	6282587	6282587	T	C	UTR3	ICMT	ENST000003	.	.	A	G	-	-	0.07784431	308,0,26,0	chr1,628258	Cytosol	A > G
chr1:6283244:T:C:UTR3:ICMT	chr1	6283244	6283244	T	C	UTR3	ICMT	ENST000003	.	.	A	G	-	-	0.09230769	177,0,18,0	chr1,628324	Cytosol	A > G
chr1:6283260:T:C:UTR3:ICMT	chr1	6283260	6283260	T	C	UTR3	ICMT	ENST000003	.	.	A	G	-	-	0.54368932	47,0,56,0	chr1,628325	Cytosol	A > G
chr1:6283264:T:C:UTR3:ICMT	chr1	6283264	6283264	T	C	UTR3	ICMT	ENST000003	.	.	A	G	-	-	0.24509804	77,0,25,0	chr1,628326	Cytosol	A > G

chr1:6283296:T:C:UTR3:ICMT	chr1	6283296	6283296	T	C	UTR3	ICMT	ENST000003	.	.	A	G	-	-	0.18333333	49,0,11,0	chr1,628329	Cytosol	A > G
chr1:6283305:T:C:UTR3:ICMT	chr1	6283305	6283305	T	C	UTR3	ICMT	ENST000003	.	.	A	G	-	-	0.76923077	9,0,30,0	chr1,628330	Cytosol	A > G
chr1:6283306:T:C:UTR3:ICMT	chr1	6283306	6283306	T	C	UTR3	ICMT	ENST000003	.	.	A	G	-	-	0.72093023	12,0,31,0	chr1,628330	Cytosol	A > G
chr1:6283342:T:C:UTR3:ICMT	chr1	6283342	6283342	T	C	UTR3	ICMT	ENST000003	.	.	A	G	-	-	0.16666667	10,0,2,0	chr1,628334	Cytosol	A > G
chr1:6283348:T:C:UTR3:ICMT	chr1	6283348	6283348	T	C	UTR3	ICMT	ENST000003	.	.	A	G	-	-	0.66666667	2,0,4,0	chr1,628334	Cytosol	A > G
chr1:6283439:T:C:UTR3:ICMT	chr1	6283439	6283439	T	C	UTR3	ICMT	ENST000003	.	.	A	G	-	-	0.35294118	11,0,6,0	chr1,628343	Cytosol	A > G
chr1:6283440:T:C:UTR3:ICMT	chr1	6283440	6283440	T	C	UTR3	ICMT	ENST000003	.	.	A	G	-	-	0.52631579	9,0,10,0	chr1,628343	Cytosol	A > G
chr1:6313373:T:C:intronic:GPR153	chr1	6313373	6313373	T	C	intronic	GPR153	.	.	.	A	G	-	-	0.33333333	4,0,2,0	chr1,631337	Cytosol	A > G
chr1:6582149:T:C:UTR3:NOL9	chr1	6582149	6582149	T	C	UTR3	NOL9	ENST000003	.	.	A	G	-	-	0.06976744	40,0,3,0	chr1,658214	Cytosol	A > G
chr1:6582161:T:C:UTR3:NOL9	chr1	6582161	6582161	T	C	UTR3	NOL9	ENST000003	.	.	A	G	-	-	0.06666667	42,0,3,0	chr1,658216	Cytosol	A > G
chr1:6582176:T:C:UTR3:NOL9	chr1	6582176	6582176	T	C	UTR3	NOL9	ENST000003	.	.	A	G	-	-	0.225	31,0,9,0	chr1,658217	Cytosol	A > G
chr1:6582221:T:C:UTR3:NOL9	chr1	6582221	6582221	T	C	UTR3	NOL9	ENST000003	.	.	A	G	-	-	0.18461538	53,0,12,0	chr1,658222	Cytosol	A > G
chr1:6582241:T:C:UTR3:NOL9	chr1	6582241	6582241	T	C	UTR3	NOL9	ENST000003	.	.	A	G	-	-	0.03125	62,0,2,0	chr1,658224	Cytosol	A > G
chr1:6582282:T:C:UTR3:NOL9	chr1	6582282	6582282	T	C	UTR3	NOL9	ENST000003	.	.	A	G	-	-	0.03225806	90,0,3,0	chr1,658228	Cytosol	A > G
chr1:6582353:T:C:UTR3:NOL9	chr1	6582353	6582353	T	C	UTR3	NOL9	ENST000003	.	.	A	G	-	-	0.03278689	59,0,2,0	chr1,658235	Cytosol	A > G
chr1:6582425:T:C:UTR3:NOL9	chr1	6582425	6582425	T	C	UTR3	NOL9	ENST000003	.	.	A	G	-	-	0.06666667	42,0,3,0	chr1,658242	Cytosol	A > G
chr1:6582558:T:C:UTR3:NOL9	chr1	6582558	6582558	T	C	UTR3	NOL9	ENST000003	.	.	A	G	-	-	0.18867925	43,0,10,0	chr1,658255	Cytosol	A > G
chr1:6582701:T:C:UTR3:NOL9	chr1	6582701	6582701	T	C	UTR3	NOL9	ENST000003	.	.	A	G	-	-	0.26666667	11,0,4,0	chr1,658270	Cytosol	A > G
chr1:6583036:T:C:UTR3:NOL9	chr1	6583036	6583036	T	C	UTR3	NOL9	ENST000003	.	.	A	G	-	-	0.19230769	42,0,10,0	chr1,658303	Cytosol	A > G
chr1:6583383:T:C:UTR3:NOL9	chr1	6583383	6583383	T	C	UTR3	NOL9	ENST000003	.	.	A	G	-	-	0.13114754	53,0,8,0	chr1,658338	Cytosol	A > G
chr1:6583527:T:C:UTR3:NOL9	chr1	6583527	6583527	T	C	UTR3	NOL9	ENST000003	.	.	A	G	-	-	0.28571429	5,0,2,0	chr1,658352	Cytosol	A > G
chr1:6583538:T:C:UTR3:NOL9	chr1	6583538	6583538	T	C	UTR3	NOL9	ENST000003	.	.	A	G	-	-	0.11111111	16,0,2,0	chr1,658353	Cytosol	A > G
chr1:6585331:T:C:UTR3:NOL9	chr1	6585331	6585331	T	C	UTR3	NOL9	ENST000003	.	.	A	G	-	-	0.10344828	26,0,3,0	chr1,658533	Cytosol	A > G
chr1:6706971:T:C:intronic:DNAJC1	chr1	6706971	6706971	T	C	intronic	DNAJC11	.	.	.	A	G	-	-	0.4	3,0,2,0	chr1,670697	Cytosol	A > G
chr1:9160900:T:C:UTR3:GPR157	chr1	9160900	9160900	T	C	UTR3	GPR157	ENST000003	.	.	A	G	-	-	0.30555556	25,0,11,0	chr1,916089	Cytosol	A > G
chr1:9160958:T:C:UTR3:GPR157	chr1	9160958	9160958	T	C	UTR3	GPR157	ENST000003	.	.	A	G	-	-	0.10606061	59,0,7,0	chr1,916095	Cytosol	A > G
chr1:9160963:T:C:UTR3:GPR157	chr1	9160963	9160963	T	C	UTR3	GPR157	ENST000003	.	.	A	G	-	-	0.26470588	50,0,18,0	chr1,916096	Cytosol	A > G
chr1:9160975:T:C:UTR3:GPR157	chr1	9160975	9160975	T	C	UTR3	GPR157	ENST000003	.	.	A	G	-	-	0.16666667	50,0,10,0	chr1,916097	Cytosol	A > G
chr1:9160989:T:C:UTR3:GPR157	chr1	9160989	9160989	T	C	UTR3	GPR157	ENST000003	.	.	A	G	-	-	0.38571429	43,0,27,0	chr1,916098	Cytosol	A > G
chr1:9160996:T:C:UTR3:GPR157	chr1	9160996	9160996	T	C	UTR3	GPR157	ENST000003	.	.	A	G	-	-	0.14814815	69,0,12,0	chr1,916099	Cytosol	A > G
chr1:9161142:T:C:UTR3:GPR157	chr1	9161142	9161142	T	C	UTR3	GPR157	ENST000003	.	.	A	G	-	-	0.30555556	25,0,11,0	chr1,916114	Cytosol	A > G
chr1:9161231:T:C:UTR3:GPR157	chr1	9161231	9161231	T	C	UTR3	GPR157	ENST000003	.	.	A	G	-	-	0.31034483	20,0,9,0	chr1,916123	Cytosol	A > G
chr1:9161245:T:C:UTR3:GPR157	chr1	9161245	9161245	T	C	UTR3	GPR157	ENST000003	.	.	A	G	-	-	0.66666667	11,0,22,0	chr1,916124	Cytosol	A > G
chr1:9162206:T:C:UTR3:GPR157	chr1	9162206	9162206	T	C	UTR3	GPR157	ENST000003	.	.	A	G	-	-	0.20689655	23,0,6,0	chr1,916220	Cytosol	A > G
chr1:9162223:T:C:UTR3:GPR157	chr1	9162223	9162223	T	C	UTR3	GPR157	ENST000003	.	.	A	G	-	-	0.21428571	11,0,3,0	chr1,916222	Cytosol	A > G
chr1:9162224:T:C:UTR3:GPR157	chr1	9162224	9162224	T	C	UTR3	GPR157	ENST000003	.	.	A	G	-	-	0.75	3,0,9,0	chr1,916222	Cytosol	A > G
chr1:9162234:T:C:UTR3:GPR157	chr1	9162234	9162234	T	C	UTR3	GPR157	ENST000003	.	.	A	G	-	-	0.22222222	7,0,2,0	chr1,916223	Cytosol	A > G
chr1:9162252:T:C:UTR3:GPR157	chr1	9162252	9162252	T	C	UTR3	GPR157	ENST000003	.	.	A	G	-	-	0.44444444	5,0,4,0	chr1,916225	Cytosol	A > G
chr1:9162303:T:C:UTR3:GPR157	chr1	9162303	9162303	T	C	UTR3	GPR157	ENST000003	.	.	A	G	-	-	0.33333333	4,0,2,0	chr1,916230	Cytosol	A > G
chr1:9162346:T:C:UTR3:GPR157	chr1	9162346	9162346	T	C	UTR3	GPR157	ENST000003	.	.	A	G	-	-	0.05	38,0,2,0	chr1,916234	Cytosol	A > G
chr1:9162348:T:C:UTR3:GPR157	chr1	9162348	9162348	T	C	UTR3	GPR157	ENST000003	.	.	A	G	-	-	0.1875	26,0,6,0	chr1,916234	Cytosol	A > G
chr1:9162361:T:C:UTR3:GPR157	chr1	9162361	9162361	T	C	UTR3	GPR157	ENST000003	.	.	A	G	-	-	0.13043478	40,0,6,0	chr1,916236	Cytosol	A > G
chr1:9162362:T:C:UTR3:GPR157	chr1	9162362	9162362	T	C	UTR3	GPR157	ENST000003	.	.	A	G	-	-	0.125	42,0,6,0	chr1,916236	Cytosol	A > G
chr1:9162387:T:C:UTR3:GPR157	chr1	9162387	9162387	T	C	UTR3	GPR157	ENST000003	.	.	A	G	-	-	0.05263158	54,0,3,0	chr1,916238	Cytosol	A > G
chr1:9162394:T:C:UTR3:GPR157	chr1	9162394	9162394	T	C	UTR3	GPR157	ENST000003	.	.	A	G	-	-	0.27906977	31,0,12,0	chr1,916239	Cytosol	A > G
chr1:9162407:T:C:UTR3:GPR157	chr1	9162407	9162407	T	C	UTR3	GPR157	ENST000003	.	.	A	G	-	-	0.10714286	25,0,3,0	chr1,916240	Cytosol	A > G
chr1:9162423:T:C:UTR3:GPR157	chr1	9162423	9162423	T	C	UTR3	GPR157	ENST000003	.	.	A	G	-	-	0.18181818	18,0,4,0	chr1,916242	Cytosol	A > G
chr1:9162451:T:C:UTR3:GPR157	chr1	9162451	9162451	T	C	UTR3	GPR157	ENST000003	.	.	A	G	-	-	0.23809524	16,0,5,0	chr1,916245	Cytosol	A > G
chr1:9163290:T:C:UTR3:GPR157	chr1	9163290	9163290	T	C	UTR3	GPR157	ENST000003	.	.	A	G	-	-	0.23529412	26,0,8,0	chr1,916328	Cytosol	A > G
chr1:9163319:T:C:UTR3:GPR157	chr1	9163319	9163319	T	C	UTR3	GPR157	ENST000003	.	.	A	G	-	-	0.09090909	30,0,3,0	chr1,916331	Cytosol	A > G
chr1:9163361:T:C:UTR3:GPR157	chr1	9163361	9163361	T	C	UTR3	GPR157	ENST000003	.	.	A	G	-	-	0.04761905	40,0,2,0	chr1,916336	Cytosol	A > G
chr1:9163387:T:C:UTR3:GPR157	chr1	9163387	9163387	T	C	UTR3	GPR157	ENST000003	.	.	A	G	-	-	0.25714286	26,0,9,0	chr1,916338	Cytosol	A > G
chr1:9163402:T:C:UTR3:GPR157	chr1	9163402	9163402	T	C	UTR3	GPR157	ENST000003	.	.	A	G	-	-	0.21052632	30,0,8,0	chr1,916340	Cytosol	A > G
chr1:9163502:T:C:UTR3:GPR157	chr1	9163502	9163502	T	C	UTR3	GPR157	ENST000003	.	.	A	G	-	-	0.12820513	34,0,5,0	chr1,916350	Cytosol	A > G
chr1:9209151:T:C:intergenic:GPR1	chr1	9209151	9209151	T	C	intergenic	GPR157,MIR	dist=19922;d	.	.	A	G	-	-	0.6	2,0,3,0	chr1,920915	Cytosol	A > G
chr1:9326957:A:G:UTR3:H6PD	chr1	9326957	9326957	A	G	UTR3	H6PD	ENST000003	.	.	A	G	+	+	0.27272727	16,0,6,0	chr1,932695	Cytosol	A > G
chr1:9328074:A:G:UTR3:H6PD	chr1	9328074	9328074	A	G	UTR3	H6PD	ENST000003	.	.	A	G	+	+	0.25	6,0,2,0	chr1,932807	Cytosol	A > G
chr1:9328102:A:G:UTR3:H6PD	chr1	9328102	9328102	A	G	UTR3	H6PD	ENST000003	.	.	A	G	+	+	0.2	8,0,2,0	chr1,932810	Cytosol	A > G
chr1:9328120:A:G:UTR3:H6PD	chr1	9328120	9328120	A	G	UTR3	H6PD	ENST000003	.	.	A	G	+	+	0.33333333	8,0,4,0	chr1,932811	Cytosol	A > G
chr1:9328200:A:G:UTR3:H6PD	chr1	9328200	9328200	A	G	UTR3	H6PD	ENST000003	.	.	A	G	+	+	0.11764706	15,0,2,0	chr1,932819	Cytosol	A > G
chr1:9328213:A:G:UTR3:H6PD	chr1	9328213	9328213	A	G	UTR3	H6PD	ENST000003	.	.	A	G	+	+	0.11764706	15,0,2,0	chr1,932821	Cytosol	A > G
chr1:9328223:A:G:UTR3:H6PD	chr1	9328223	9328223	A	G	UTR3	H6PD	ENST000003	.	.	A	G	+	+	0.28571429	15,0,6,0	chr1,932822	Cytosol	A > G
chr1:9328242:A:G:UTR3:H6PD	chr1	9328242	9328242	A	G	UTR3	H6PD	ENST000003	.	.	A	G	+	+	0.70588235	5,0,12,0	chr1,932824	Cytosol	A > G
chr1:9328243:A:G:UTR3:H6PD	chr1	9328243	9328243	A	G	UTR3	H6PD	ENST000003	.	.	A	G	+	+	0.41176471	10,0,7,0	chr1,932824	Cytosol	A > G

chr1:9331075:A:G:UTR3:H6PD	chr1	9331075	9331075	A	G	UTR3	H6PD	ENST000003	.	.	A	G	+	+	0.16666667	10,0,2,0	chr1,933107	Cytosol	A > G
chr1:9331094:A:G:UTR3:H6PD	chr1	9331094	9331094	A	G	UTR3	H6PD	ENST000003	.	.	A	G	+	+	0.125	14,0,2,0	chr1,933109	Cytosol	A > G
chr1:9987448:T:C:intronic:LZIC	chr1	9987448	9987448	T	C	intronic	LZIC	.	.	.	A	G	-	-	0.07843137	141,0,12,0	chr1,998744	Cytosol	A > G
chr1:9987468:T:C:intronic:LZIC	chr1	9987468	9987468	T	C	intronic	LZIC	.	.	.	A	G	-	-	0.03	97,0,3,0	chr1,998746	Cytosol	A > G
chr1:9987470:T:C:intronic:LZIC	chr1	9987470	9987470	T	C	intronic	LZIC	.	.	.	A	G	-	-	0.23076923	79,0,24,1	chr1,998746	Cytosol	A > G
chr1:9987480:T:C:intronic:LZIC	chr1	9987480	9987480	T	C	intronic	LZIC	.	.	.	A	G	-	-	0.10416667	86,0,10,0	chr1,998747	Cytosol	A > G
chr1:9987484:T:C:intronic:LZIC	chr1	9987484	9987484	T	C	intronic	LZIC	.	.	.	A	G	-	-	0.34444444	59,0,31,0	chr1,998748	Cytosol	A > G
chr1:9987488:T:C:intronic:LZIC	chr1	9987488	9987488	T	C	intronic	LZIC	.	.	.	A	G	-	-	0.08536585	75,0,7,0	chr1,998748	Cytosol	A > G
chr1:9987631:T:C:intronic:LZIC	chr1	9987631	9987631	T	C	intronic	LZIC	.	.	.	A	G	-	-	0.10434783	103,0,12,0	chr1,998763	Cytosol	A > G
chr1:9987640:T:C:intronic:LZIC	chr1	9987640	9987640	T	C	intronic	LZIC	.	.	.	A	G	-	-	0.19	81,0,19,0	chr1,998763	Cytosol	A > G
chr1:9987814:T:C:intronic:LZIC	chr1	9987814	9987814	T	C	intronic	LZIC	.	.	.	A	G	-	-	0.12	22,0,3,0	chr1,998781	Cytosol	A > G
chr1:9987827:T:C:intronic:LZIC	chr1	9987827	9987827	T	C	intronic	LZIC	.	.	.	A	G	-	-	0.15	17,0,3,0	chr1,998782	Cytosol	A > G
chr1:9987847:T:C:intronic:LZIC	chr1	9987847	9987847	T	C	intronic	LZIC	.	.	.	A	G	-	-	0.42857143	8,0,6,0	chr1,998784	Cytosol	A > G
chr1:9987853:T:C:intronic:LZIC	chr1	9987853	9987853	T	C	intronic	LZIC	.	.	.	A	G	-	-	0.72727273	3,0,8,0	chr1,998785	Cytosol	A > G
chr1:9987857:T:C:intronic:LZIC	chr1	9987857	9987857	T	C	intronic	LZIC	.	.	.	A	G	-	-	0.26666667	11,0,4,0	chr1,998785	Cytosol	A > G
chr1:9987870:T:C:intronic:LZIC	chr1	9987870	9987870	T	C	intronic	LZIC	.	.	.	A	G	-	-	0.38461538	16,0,10,0	chr1,998786	Cytosol	A > G
chr1:9987880:T:C:intronic:LZIC	chr1	9987880	9987880	T	C	intronic	LZIC	.	.	.	A	G	-	-	0.38709677	19,0,12,0	chr1,998787	Cytosol	A > G
chr1:9987898:T:C:intronic:LZIC	chr1	9987898	9987898	T	C	intronic	LZIC	.	.	.	A	G	-	-	0.04166667	46,0,2,0	chr1,998789	Cytosol	A > G
chr1:9987906:T:C:intronic:LZIC	chr1	9987906	9987906	T	C	intronic	LZIC	.	.	.	A	G	-	-	0.09803922	46,0,5,0	chr1,998790	Cytosol	A > G
chr1:9987932:T:C:intronic:LZIC	chr1	9987932	9987932	T	C	intronic	LZIC	.	.	.	A	G	-	-	0.44927536	38,0,31,0	chr1,998793	Cytosol	A > G
chr1:9987951:T:C:intronic:LZIC	chr1	9987951	9987951	T	C	intronic	LZIC	.	.	.	A	G	-	-	0.32857143	47,0,23,0	chr1,998795	Cytosol	A > G
chr1:9987952:T:C:intronic:LZIC	chr1	9987952	9987952	T	C	intronic	LZIC	.	.	.	A	G	-	-	0.38356164	45,0,28,0	chr1,998795	Cytosol	A > G
chr1:9987960:T:C:intronic:LZIC	chr1	9987960	9987960	T	C	intronic	LZIC	.	.	.	A	G	-	-	0.22727273	51,0,15,0	chr1,998795	Cytosol	A > G
chr1:9987965:T:C:intronic:LZIC	chr1	9987965	9987965	T	C	intronic	LZIC	.	.	.	A	G	-	-	0.36764706	43,0,25,0	chr1,998796	Cytosol	A > G
chr1:9987968:T:C:intronic:LZIC	chr1	9987968	9987968	T	C	intronic	LZIC	.	.	.	A	G	-	-	0.04477612	64,0,3,0	chr1,998796	Cytosol	A > G
chr1:9987990:T:C:intronic:LZIC	chr1	9987990	9987990	T	C	intronic	LZIC	.	.	.	A	G	-	-	0.14130435	79,0,13,0	chr1,998798	Cytosol	A > G
chr1:9988107:T:C:intronic:LZIC	chr1	9988107	9988107	T	C	intronic	LZIC	.	.	.	A	G	-	-	0.18446602	84,0,19,0	chr1,998810	Cytosol	A > G
chr1:9988130:T:C:intronic:LZIC	chr1	9988130	9988130	T	C	intronic	LZIC	.	.	.	A	G	-	-	0.12790698	75,0,11,0	chr1,998812	Cytosol	A > G
chr1:9988147:T:C:intronic:LZIC	chr1	9988147	9988147	T	C	intronic	LZIC	.	.	.	A	G	-	-	0.39726027	44,0,29,0	chr1,998814	Cytosol	A > G
chr1:9988248:T:C:intronic:LZIC	chr1	9988248	9988248	T	C	intronic	LZIC	.	.	.	A	G	-	-	0.625	6,0,10,0	chr1,998824	Cytosol	A > G
chr1:9988249:T:C:intronic:LZIC	chr1	9988249	9988249	T	C	intronic	LZIC	.	.	.	A	G	-	-	0.4375	9,0,7,0	chr1,998824	Cytosol	A > G
chr1:9988259:T:C:intronic:LZIC	chr1	9988259	9988259	T	C	intronic	LZIC	.	.	.	A	G	-	-	0.66666667	5,0,10,0	chr1,998825	Cytosol	A > G
chr1:9988271:T:C:intronic:LZIC	chr1	9988271	9988271	T	C	intronic	LZIC	.	.	.	A	G	-	-	0.4	12,0,8,0	chr1,998827	Cytosol	A > G
chr1:9988332:T:C:intronic:LZIC	chr1	9988332	9988332	T	C	intronic	LZIC	.	.	.	A	G	-	-	0.69230769	8,0,18,0	chr1,998833	Cytosol	A > G
chr1:9988369:T:C:intronic:LZIC	chr1	9988369	9988369	T	C	intronic	LZIC	.	.	.	A	G	-	-	0.0625	45,0,3,0	chr1,998836	Cytosol	A > G
chr1:9988690:T:C:intronic:LZIC	chr1	9988690	9988690	T	C	intronic	LZIC	.	.	.	A	G	-	-	0.18023256	141,0,31,0	chr1,998868	Cytosol	A > G
chr1:9988691:T:C:intronic:LZIC	chr1	9988691	9988691	T	C	intronic	LZIC	.	.	.	A	G	-	-	0.3575419	115,0,64,0	chr1,998869	Cytosol	A > G
chr1:9988692:T:C:intronic:LZIC	chr1	9988692	9988692	T	C	intronic	LZIC	.	.	.	A	G	-	-	0.23913043	139,0,44,1	chr1,998869	Cytosol	A > G
chr1:9988718:T:C:intronic:LZIC	chr1	9988718	9988718	T	C	intronic	LZIC	.	.	.	A	G	-	-	0.0625	150,0,10,0	chr1,998871	Cytosol	A > G
chr1:9988749:T:C:intronic:LZIC	chr1	9988749	9988749	T	C	intronic	LZIC	.	.	.	A	G	-	-	0.09722222	130,0,14,0	chr1,998874	Cytosol	A > G
chr1:9988764:T:C:intronic:LZIC	chr1	9988764	9988764	T	C	intronic	LZIC	.	.	.	A	G	-	-	0.04424779	108,0,5,0	chr1,998876	Cytosol	A > G
chr1:9988766:T:C:intronic:LZIC	chr1	9988766	9988766	T	C	intronic	LZIC	.	.	.	A	G	-	-	0.07142857	104,0,8,0	chr1,998876	Cytosol	A > G
chr1:9988826:T:C:intronic:LZIC	chr1	9988826	9988826	T	C	intronic	LZIC	.	.	.	A	G	-	-	0.26666667	55,0,20,0	chr1,998882	Cytosol	A > G
chr1:9988849:T:C:intronic:LZIC	chr1	9988849	9988849	T	C	intronic	LZIC	.	.	.	A	G	-	-	0.04545455	84,0,4,0	chr1,998884	Cytosol	A > G
chr1:9988851:T:C:intronic:LZIC	chr1	9988851	9988851	T	C	intronic	LZIC	.	.	.	A	G	-	-	0.29213483	63,0,26,0	chr1,998885	Cytosol	A > G
chr1:10042910:A:G:UTR3:NMNAT	chr1	10042910	10042910	A	G	UTR3	NMNAT1	ENST000003	.	.	A	G	+	+	0.2	16,0,4,0	chr1,100429	Cytosol	A > G
chr1:10042911:A:G:UTR3:NMNAT	chr1	10042911	10042911	A	G	UTR3	NMNAT1	ENST000003	.	.	A	G	+	+	0.0952381	19,0,2,0	chr1,100429	Cytosol	A > G
chr1:10042955:A:G:UTR3:NMNAT	chr1	10042955	10042955	A	G	UTR3	NMNAT1	ENST000003	.	.	A	G	+	+	0.59459459	15,0,22,0	chr1,100429	Cytosol	A > G
chr1:10042978:A:G:UTR3:NMNAT	chr1	10042978	10042978	A	G	UTR3	NMNAT1	ENST000003	.	.	A	G	+	+	0.11764706	30,0,4,0	chr1,100429	Cytosol	A > G
chr1:10042987:A:G:UTR3:NMNAT	chr1	10042987	10042987	A	G	UTR3	NMNAT1	ENST000003	.	.	A	G	+	+	0.28125	23,0,9,0	chr1,100429	Cytosol	A > G
chr1:10042988:A:G:UTR3:NMNAT	chr1	10042988	10042988	A	G	UTR3	NMNAT1	ENST000003	.	.	A	G	+	+	0.46666667	16,0,14,0	chr1,100429	Cytosol	A > G
chr1:10043043:A:G:UTR3:NMNAT	chr1	10043043	10043043	A	G	UTR3	NMNAT1	ENST000003	.	.	A	G	+	+	0.33333333	8,0,4,0	chr1,100430	Cytosol	A > G
chr1:10043053:A:G:UTR3:NMNAT	chr1	10043053	10043053	A	G	UTR3	NMNAT1	ENST000003	.	.	A	G	+	+	0.25	9,0,3,0	chr1,100430	Cytosol	A > G
chr1:10043079:A:G:UTR3:NMNAT	chr1	10043079	10043079	A	G	UTR3	NMNAT1	ENST000003	.	.	A	G	+	+	0.54545455	5,0,6,0	chr1,100430	Cytosol	A > G
chr1:10043089:A:G:UTR3:NMNAT	chr1	10043089	10043089	A	G	UTR3	NMNAT1	ENST000003	.	.	A	G	+	+	0.875	1,0,7,0	chr1,100430	Cytosol	A > G
chr1:10043250:A:G:ncRNA_exonic	chr1	10043250	10043250	A	G	ncRNA_exon	RP11-807G9.	.	.	.	A	G	+	+	0.07317073	38,0,3,0	chr1,100432	Cytosol	A > G
chr1:10043358:A:G:ncRNA_introni	chr1	10043358	10043358	A	G	ncRNA_intro	RP11-807G9.	.	.	.	A	G	+	+	0.18867925	43,0,10,0	chr1,100433	Cytosol	A > G
chr1:10043363:A:G:ncRNA_introni	chr1	10043363	10043363	A	G	ncRNA_intro	RP11-807G9.	.	.	.	A	G	+	+	0.20689655	46,0,12,0	chr1,100433	Cytosol	A > G
chr1:10043526:A:G:ncRNA_introni	chr1	10043526	10043526	A	G	ncRNA_intro	RP11-807G9.	.	.	.	A	G	+	+	0.19354839	25,0,6,0	chr1,100435	Cytosol	A > G
chr1:10043527:A:G:ncRNA_introni	chr1	10043527	10043527	A	G	ncRNA_intro	RP11-807G9.	.	.	.	A	G	+	+	0.37931034	18,0,11,0	chr1,100435	Cytosol	A > G
chr1:10044137:A:G:ncRNA_introni	chr1	10044137	10044137	A	G	ncRNA_intro	RP11-807G9.	.	.	.	A	G	+	+	0.66666667	3,0,6,0	chr1,100441	Cytosol	A > G
chr1:10044180:A:G:ncRNA_introni	chr1	10044180	10044180	A	G	ncRNA_intro	RP11-807G9.	.	.	.	A	G	+	+	0.28571429	5,0,2,0	chr1,100441	Cytosol	A > G
chr1:10044205:A:G:ncRNA_introni	chr1	10044205	10044205	A	G	ncRNA_intro	RP11-807G9.	.	.	.	A	G	+	+	0.625	3,0,5,0	chr1,100442	Cytosol	A > G
chr1:10044206:A:G:ncRNA_introni	chr1	10044206	10044206	A	G	ncRNA_intro	RP11-807G9.	.	.	.	A	G	+	+	0.5	4,0,4,0	chr1,100442	Cytosol	A > G

chr1:10044209:A:G:ncRNA_introni	chr1	10044209	10044209	A	G	ncRNA_intro	RP11-807G9.	.	.	.	A	G	+	+	0.25	6,0,2,0	chr1,100442	Cytosol	A > G
chr1:10044310:A:G:ncRNA_introni	chr1	10044310	10044310	A	G	ncRNA_intro	RP11-807G9.	.	.	.	A	G	+	+	0.87878788	4,0,29,0	chr1,100443	Cytosol	A > G
chr1:10044366:A:G:ncRNA_exonic	chr1	10044366	10044366	A	G	ncRNA_exon	RP11-807G9.	.	.	.	A	G	+	+	0.20689655	23,0,6,0	chr1,100443	Cytosol	A > G
chr1:10044367:A:G:ncRNA_exonic	chr1	10044367	10044367	A	G	ncRNA_exon	RP11-807G9.	.	.	.	A	G	+	+	0.22727273	17,0,5,0	chr1,100443	Cytosol	A > G
chr1:10044379:A:G:ncRNA_exonic	chr1	10044379	10044379	A	G	ncRNA_exon	RP11-807G9.	.	.	.	A	G	+	+	0.19047619	17,0,4,0	chr1,100443	Cytosol	A > G
chr1:10044385:A:G:ncRNA_exonic	chr1	10044385	10044385	A	G	ncRNA_exon	RP11-807G9.	.	.	.	A	G	+	+	0.11538462	23,0,3,0	chr1,100443	Cytosol	A > G
chr1:10440678:A:G:UTR3:KIF1B	chr1	10440678	10440678	A	G	UTR3	KIF1B	ENST000003	.	.	A	G	+	+	0.125	14,0,2,0	chr1,104406	Cytosol	A > G
chr1:10517276:T:C:UTR3:DFFA	chr1	10517276	10517276	T	C	UTR3	DFFA	ENST000003	.	.	A	G	-	-	0.22941176	131,0,39,0	chr1,105172	Cytosol	A > G
chr1:10517300:T:C:UTR3:DFFA	chr1	10517300	10517300	T	C	UTR3	DFFA	ENST000003	.	.	A	G	-	-	0.10071942	125,0,14,0	chr1,105172	Cytosol	A > G
chr1:10517306:T:C:UTR3:DFFA	chr1	10517306	10517306	T	C	UTR3	DFFA	ENST000003	.	.	A	G	-	-	0.09022556	121,0,12,0	chr1,105173	Cytosol	A > G
chr1:10517309:T:C:UTR3:DFFA	chr1	10517309	10517309	T	C	UTR3	DFFA	ENST000003	.	.	A	G	-	-	0.01639344	120,0,2,0	chr1,105173	Cytosol	A > G
chr1:10517313:T:C:UTR3:DFFA	chr1	10517313	10517313	T	C	UTR3	DFFA	ENST000003	.	.	A	G	-	-	0.25984252	94,0,33,0	chr1,105173	Cytosol	A > G
chr1:10517314:T:C:UTR3:DFFA	chr1	10517314	10517314	T	C	UTR3	DFFA	ENST000003	.	.	A	G	-	-	0.14285714	114,0,19,0	chr1,105173	Cytosol	A > G
chr1:10517335:T:C:UTR3:DFFA	chr1	10517335	10517335	T	C	UTR3	DFFA	ENST000003	.	.	A	G	-	-	0.07317073	114,0,9,0	chr1,105173	Cytosol	A > G
chr1:10517352:T:C:UTR3:DFFA	chr1	10517352	10517352	T	C	UTR3	DFFA	ENST000003	.	.	A	G	-	-	0.19469027	91,0,22,0	chr1,105173	Cytosol	A > G
chr1:10517362:T:C:UTR3:DFFA	chr1	10517362	10517362	T	C	UTR3	DFFA	ENST000003	.	.	A	G	-	-	0.02272727	129,0,3,0	chr1,105173	Cytosol	A > G
chr1:10517383:T:C:UTR3:DFFA	chr1	10517383	10517383	T	C	UTR3	DFFA	ENST000003	.	.	A	G	-	-	0.02189781	134,0,3,0	chr1,105173	Cytosol	A > G
chr1:10517485:T:C:UTR3:DFFA	chr1	10517485	10517485	T	C	UTR3	DFFA	ENST000003	.	.	A	G	-	-	0.18666667	61,0,14,0	chr1,105174	Cytosol	A > G
chr1:10517486:T:C:UTR3:DFFA	chr1	10517486	10517486	T	C	UTR3	DFFA	ENST000003	.	.	A	G	-	-	0.03703704	78,0,3,0	chr1,105174	Cytosol	A > G
chr1:10517504:T:C:UTR3:DFFA	chr1	10517504	10517504	T	C	UTR3	DFFA	ENST000003	.	.	A	G	-	-	0.16326531	41,0,8,0	chr1,105175	Cytosol	A > G
chr1:10517512:T:C:UTR3:DFFA	chr1	10517512	10517512	T	C	UTR3	DFFA	ENST000003	.	.	A	G	-	-	0.4375	27,0,21,0	chr1,105175	Cytosol	A > G
chr1:10517527:T:C:UTR3:DFFA	chr1	10517527	10517527	T	C	UTR3	DFFA	ENST000003	.	.	A	G	-	-	0.74358974	10,0,29,0	chr1,105175	Cytosol	A > G
chr1:10517538:T:C:UTR3:DFFA	chr1	10517538	10517538	T	C	UTR3	DFFA	ENST000003	.	.	A	G	-	-	0.21621622	29,0,8,0	chr1,105175	Cytosol	A > G
chr1:10517550:T:C:UTR3:DFFA	chr1	10517550	10517550	T	C	UTR3	DFFA	ENST000003	.	.	A	G	-	-	0.05882353	32,0,2,0	chr1,105175	Cytosol	A > G
chr1:10517558:T:C:UTR3:DFFA	chr1	10517558	10517558	T	C	UTR3	DFFA	ENST000003	.	.	A	G	-	-	0.21875	25,0,7,0	chr1,105175	Cytosol	A > G
chr1:10517564:T:C:UTR3:DFFA	chr1	10517564	10517564	T	C	UTR3	DFFA	ENST000003	.	.	A	G	-	-	0.36538462	33,0,19,0	chr1,105175	Cytosol	A > G
chr1:10517567:T:C:UTR3:DFFA	chr1	10517567	10517567	T	C	UTR3	DFFA	ENST000003	.	.	A	G	-	-	0.27272727	48,0,18,0	chr1,105175	Cytosol	A > G
chr1:10517573:T:C:UTR3:DFFA	chr1	10517573	10517573	T	C	UTR3	DFFA	ENST000003	.	.	A	G	-	-	0.11428571	62,0,8,0	chr1,105175	Cytosol	A > G
chr1:10517613:T:C:UTR3:DFFA	chr1	10517613	10517613	T	C	UTR3	DFFA	ENST000003	.	.	A	G	-	-	0.67948718	25,0,53,0	chr1,105176	Cytosol	A > G
chr1:10517623:T:C:UTR3:DFFA	chr1	10517623	10517623	T	C	UTR3	DFFA	ENST000003	.	.	A	G	-	-	0.36708861	50,0,29,0	chr1,105176	Cytosol	A > G
chr1:10517635:T:C:UTR3:DFFA	chr1	10517635	10517635	T	C	UTR3	DFFA	ENST000003	.	.	A	G	-	-	0.18867925	43,0,10,0	chr1,105176	Cytosol	A > G
chr1:10517653:T:C:UTR3:DFFA	chr1	10517653	10517653	T	C	UTR3	DFFA	ENST000003	.	.	A	G	-	-	0.51020408	24,0,25,0	chr1,105176	Cytosol	A > G
chr1:10517659:T:C:UTR3:DFFA	chr1	10517659	10517659	T	C	UTR3	DFFA	ENST000003	.	.	A	G	-	-	0.28070175	41,0,16,0	chr1,105176	Cytosol	A > G
chr1:10517672:T:C:UTR3:DFFA	chr1	10517672	10517672	T	C	UTR3	DFFA	ENST000003	.	.	A	G	-	-	0.07017544	53,0,4,0	chr1,105176	Cytosol	A > G
chr1:10517673:T:C:UTR3:DFFA	chr1	10517673	10517673	T	C	UTR3	DFFA	ENST000003	.	.	A	G	-	-	0.03333333	58,0,2,0	chr1,105176	Cytosol	A > G
chr1:10517688:T:C:UTR3:DFFA	chr1	10517688	10517688	T	C	UTR3	DFFA	ENST000003	.	.	A	G	-	-	0.14035088	49,0,8,0	chr1,105176	Cytosol	A > G
chr1:10517689:T:C:UTR3:DFFA	chr1	10517689	10517689	T	C	UTR3	DFFA	ENST000003	.	.	A	G	-	-	0.08064516	57,0,5,0	chr1,105176	Cytosol	A > G
chr1:10517691:T:C:UTR3:DFFA	chr1	10517691	10517691	T	C	UTR3	DFFA	ENST000003	.	.	A	G	-	-	0.12903226	54,0,8,0	chr1,105176	Cytosol	A > G
chr1:10517992:T:C:UTR3:DFFA	chr1	10517992	10517992	T	C	UTR3	DFFA	ENST000003	.	.	A	G	-	-	0.03431373	197,0,7,0	chr1,105179	Cytosol	A > G
chr1:10517998:T:C:UTR3:DFFA	chr1	10517998	10517998	T	C	UTR3	DFFA	ENST000003	.	.	A	G	-	-	0.08457711	184,0,17,0	chr1,105179	Cytosol	A > G
chr1:10518020:T:C:UTR3:DFFA	chr1	10518020	10518020	T	C	UTR3	DFFA	ENST000003	.	.	A	G	-	-	0.02617801	186,0,5,0	chr1,105180	Cytosol	A > G
chr1:10518050:T:C:UTR3:DFFA	chr1	10518050	10518050	T	C	UTR3	DFFA	ENST000003	.	.	A	G	-	-	0.02061856	190,0,4,0	chr1,105180	Cytosol	A > G
chr1:10518052:T:C:UTR3:DFFA	chr1	10518052	10518052	T	C	UTR3	DFFA	ENST000003	.	.	A	G	-	-	0.10679612	184,0,22,0	chr1,105180	Cytosol	A > G
chr1:10518067:T:C:UTR3:DFFA	chr1	10518067	10518067	T	C	UTR3	DFFA	ENST000003	.	.	A	G	-	-	0.03703704	182,0,7,0	chr1,105180	Cytosol	A > G
chr1:10519874:T:C:UTR3:DFFA	chr1	10519874	10519874	T	C	UTR3	DFFA	ENST000003	.	.	A	G	-	-	0.10928962	163,0,20,0	chr1,105198	Cytosol	A > G
chr1:10519887:T:C:UTR3:DFFA	chr1	10519887	10519887	T	C	UTR3	DFFA	ENST000003	.	.	A	G	-	-	0.13761468	94,0,15,0	chr1,105198	Cytosol	A > G
chr1:10519888:T:C:UTR3:DFFA	chr1	10519888	10519888	T	C	UTR3	DFFA	ENST000003	.	.	A	G	-	-	0.28440367	78,0,31,0	chr1,105198	Cytosol	A > G
chr1:10519912:T:C:UTR3:DFFA	chr1	10519912	10519912	T	C	UTR3	DFFA	ENST000003	.	.	A	G	-	-	0.23484848	101,0,31,0	chr1,105199	Cytosol	A > G
chr1:10519932:T:C:UTR3:DFFA	chr1	10519932	10519932	T	C	UTR3	DFFA	ENST000003	.	.	A	G	-	-	0.08396947	120,0,11,0	chr1,105199	Cytosol	A > G
chr1:10519946:T:C:UTR3:DFFA	chr1	10519946	10519946	T	C	UTR3	DFFA	ENST000003	.	.	A	G	-	-	0.14935065	131,0,23,0	chr1,105199	Cytosol	A > G
chr1:10520045:T:C:UTR3:DFFA	chr1	10520045	10520045	T	C	UTR3	DFFA	ENST000003	.	.	A	G	-	-	0.14473684	65,0,11,0	chr1,105200	Cytosol	A > G
chr1:10520067:T:C:UTR3:DFFA	chr1	10520067	10520067	T	C	UTR3	DFFA	ENST000003	.	.	A	G	-	-	0.53846154	24,0,28,0	chr1,105200	Cytosol	A > G
chr1:10520072:T:C:UTR3:DFFA	chr1	10520072	10520072	T	C	UTR3	DFFA	ENST000003	.	.	A	G	-	-	0.10416667	43,0,5,0	chr1,105200	Cytosol	A > G
chr1:10520099:T:C:UTR3:DFFA	chr1	10520099	10520099	T	C	UTR3	DFFA	ENST000003	.	.	A	G	-	-	0.21212121	26,0,7,0	chr1,105200	Cytosol	A > G
chr1:10520136:T:C:UTR3:DFFA	chr1	10520136	10520136	T	C	UTR3	DFFA	ENST000003	.	.	A	G	-	-	0.23255814	33,0,10,0	chr1,105201	Cytosol	A > G
chr1:10520145:T:C:UTR3:DFFA	chr1	10520145	10520145	T	C	UTR3	DFFA	ENST000003	.	.	A	G	-	-	0.16071429	47,0,9,0	chr1,105201	Cytosol	A > G
chr1:10520153:T:C:UTR3:DFFA	chr1	10520153	10520153	T	C	UTR3	DFFA	ENST000003	.	.	A	G	-	-	0.10344828	51,0,6,1	chr1,105201	Cytosol	A > G
chr1:10520178:T:C:UTR3:DFFA	chr1	10520178	10520178	T	C	UTR3	DFFA	ENST000003	.	.	A	G	-	-	0.14754098	52,0,9,0	chr1,105201	Cytosol	A > G
chr1:10520180:T:C:UTR3:DFFA	chr1	10520180	10520180	T	C	UTR3	DFFA	ENST000003	.	.	A	G	-	-	0.58730159	26,0,37,0	chr1,105201	Cytosol	A > G
chr1:10520190:T:C:UTR3:DFFA	chr1	10520190	10520190	T	C	UTR3	DFFA	ENST000003	.	.	A	G	-	-	0.45	33,0,27,0	chr1,105201	Cytosol	A > G
chr1:10520202:T:C:UTR3:DFFA	chr1	10520202	10520202	T	C	UTR3	DFFA	ENST000003	.	.	A	G	-	-	0.18918919	30,0,7,0	chr1,105202	Cytosol	A > G
chr1:10520219:T:C:UTR3:DFFA	chr1	10520219	10520219	T	C	UTR3	DFFA	ENST000003	.	.	A	G	-	-	0.33333333	36,0,18,0	chr1,105202	Cytosol	A > G
chr1:10520226:T:C:UTR3:DFFA	chr1	10520226	10520226	T	C	UTR3	DFFA	ENST000003	.	.	A	G	-	-	0.11688312	68,0,9,0	chr1,105202	Cytosol	A > G
chr1:10520256:T:C:UTR3:DFFA	chr1	10520256	10520256	T	C	UTR3	DFFA	ENST000003	.	.	A	G	-	-	0.025	78,0,2,0	chr1,105202	Cytosol	A > G

chr1:10520258:T:C:UTR3:DFFA	chr1	10520258	10520258	T	C	UTR3	DFFA	ENST000003	.	.	A	G	-	-	0.47560976	43,0,39,0	chr1,105202	Cytosol	A > G
chr1:10520269:T:C:UTR3:DFFA	chr1	10520269	10520269	T	C	UTR3	DFFA	ENST000003	.	.	A	G	-	-	0.06329114	74,0,5,0	chr1,105202	Cytosol	A > G
chr1:10520296:T:C:UTR3:DFFA	chr1	10520296	10520296	T	C	UTR3	DFFA	ENST000003	.	.	A	G	-	-	0.62	19,0,31,0	chr1,105202	Cytosol	A > G
chr1:10520378:T:C:UTR3:DFFA	chr1	10520378	10520378	T	C	UTR3	DFFA	ENST000003	.	.	A	G	-	-	0.56578947	33,0,43,0	chr1,105203	Cytosol	A > G
chr1:10520383:T:C:UTR3:DFFA	chr1	10520383	10520383	T	C	UTR3	DFFA	ENST000003	.	.	A	G	-	-	0.26315789	56,0,20,0	chr1,105203	Cytosol	A > G
chr1:10520487:T:C:UTR3:DFFA	chr1	10520487	10520487	T	C	UTR3	DFFA	ENST000003	.	.	A	G	-	-	0.2020202	79,0,20,0	chr1,105204	Cytosol	A > G
chr1:10520532:T:C:UTR3:DFFA	chr1	10520532	10520532	T	C	UTR3	DFFA	ENST000003	.	.	A	G	-	-	0.1171875	113,0,15,0	chr1,105205	Cytosol	A > G
chr1:10520683:T:C:UTR3:DFFA	chr1	10520683	10520683	T	C	UTR3	DFFA	ENST000003	.	.	A	G	-	-	0.21917808	57,0,16,0	chr1,105206	Cytosol	A > G
chr1:10520702:T:C:UTR3:DFFA	chr1	10520702	10520702	T	C	UTR3	DFFA	ENST000003	.	.	A	G	-	-	0.07936508	58,0,5,0	chr1,105207	Cytosol	A > G
chr1:10520719:T:C:UTR3:DFFA	chr1	10520719	10520719	T	C	UTR3	DFFA	ENST000003	.	.	A	G	-	-	0.03409091	85,0,3,0	chr1,105207	Cytosol	A > G
chr1:10520725:T:C:UTR3:DFFA	chr1	10520725	10520725	T	C	UTR3	DFFA	ENST000003	.	.	A	G	-	-	0.34375	63,0,33,0	chr1,105207	Cytosol	A > G
chr1:10520735:T:C:UTR3:DFFA	chr1	10520735	10520735	T	C	UTR3	DFFA	ENST000003	.	.	A	G	-	-	0.21686747	65,0,18,0	chr1,105207	Cytosol	A > G
chr1:10520751:T:C:UTR3:DFFA	chr1	10520751	10520751	T	C	UTR3	DFFA	ENST000003	.	.	A	G	-	-	0.41584158	59,0,42,0	chr1,105207	Cytosol	A > G
chr1:10520764:T:C:UTR3:DFFA	chr1	10520764	10520764	T	C	UTR3	DFFA	ENST000003	.	.	A	G	-	-	0.14953271	91,0,16,0	chr1,105207	Cytosol	A > G
chr1:10520765:T:C:UTR3:DFFA	chr1	10520765	10520765	T	C	UTR3	DFFA	ENST000003	.	.	A	G	-	-	0.10377358	95,0,11,0	chr1,105207	Cytosol	A > G
chr1:10520767:T:C:UTR3:DFFA	chr1	10520767	10520767	T	C	UTR3	DFFA	ENST000003	.	.	A	G	-	-	0.0952381	95,0,10,0	chr1,105207	Cytosol	A > G
chr1:10520770:T:C:UTR3:DFFA	chr1	10520770	10520770	T	C	UTR3	DFFA	ENST000003	.	.	A	G	-	-	0.03571429	107,1,4,0	chr1,105207	Cytosol	A > G
chr1:10520786:T:C:UTR3:DFFA	chr1	10520786	10520786	T	C	UTR3	DFFA	ENST000003	.	.	A	G	-	-	0.05063291	75,0,4,0	chr1,105207	Cytosol	A > G
chr1:10520814:T:C:UTR3:DFFA	chr1	10520814	10520814	T	C	UTR3	DFFA	ENST000003	.	.	A	G	-	-	0.06153846	61,0,4,0	chr1,105208	Cytosol	A > G
chr1:10520819:T:C:UTR3:DFFA	chr1	10520819	10520819	T	C	UTR3	DFFA	ENST000003	.	.	A	G	-	-	0.42372881	34,0,25,0	chr1,105208	Cytosol	A > G
chr1:10520858:T:C:UTR3:DFFA	chr1	10520858	10520858	T	C	UTR3	DFFA	ENST000003	.	.	A	G	-	-	0.08064516	57,0,5,0	chr1,105208	Cytosol	A > G
chr1:10521094:T:C:UTR3:DFFA	chr1	10521094	10521094	T	C	UTR3	DFFA	ENST000003	.	.	A	G	-	-	0.07575758	61,0,5,0	chr1,105210	Cytosol	A > G
chr1:10521126:T:C:UTR3:DFFA	chr1	10521126	10521126	T	C	UTR3	DFFA	ENST000003	.	.	A	G	-	-	0.25490196	38,0,13,0	chr1,105211	Cytosol	A > G
chr1:10521130:T:C:UTR3:DFFA	chr1	10521130	10521130	T	C	UTR3	DFFA	ENST000003	.	.	A	G	-	-	0.22641509	41,0,12,0	chr1,105211	Cytosol	A > G
chr1:10521162:T:C:UTR3:DFFA	chr1	10521162	10521162	T	C	UTR3	DFFA	ENST000003	.	.	A	G	-	-	0.30357143	39,0,17,0	chr1,105211	Cytosol	A > G
chr1:10521170:T:C:UTR3:DFFA	chr1	10521170	10521170	T	C	UTR3	DFFA	ENST000003	.	.	A	G	-	-	0.27272727	55,0,21,1	chr1,105211	Cytosol	A > G
chr1:10521172:T:C:UTR3:DFFA	chr1	10521172	10521172	T	C	UTR3	DFFA	ENST000003	.	.	A	G	-	-	0.17721519	65,0,14,0	chr1,105211	Cytosol	A > G
chr1:10521200:T:C:UTR3:DFFA	chr1	10521200	10521200	T	C	UTR3	DFFA	ENST000003	.	.	A	G	-	-	0.45360825	53,0,44,0	chr1,105211	Cytosol	A > G
chr1:10521237:T:C:UTR3:DFFA	chr1	10521237	10521237	T	C	UTR3	DFFA	ENST000003	.	.	A	G	-	-	0.09375	87,0,9,0	chr1,105212	Cytosol	A > G
chr1:10521238:T:C:UTR3:DFFA	chr1	10521238	10521238	T	C	UTR3	DFFA	ENST000003	.	.	A	G	-	-	0.19791667	77,0,19,0	chr1,105212	Cytosol	A > G
chr1:10521254:T:C:UTR3:DFFA	chr1	10521254	10521254	T	C	UTR3	DFFA	ENST000003	.	.	A	G	-	-	0.13541667	83,0,13,0	chr1,105212	Cytosol	A > G
chr1:10522040:T:C:intronic:DFFA	chr1	10522040	10522040	T	C	intronic	DFFA	.	.	.	A	G	-	-	0.0952381	19,0,2,0	chr1,105220	Cytosol	A > G
chr1:10522416:T:C:intronic:DFFA	chr1	10522416	10522416	T	C	intronic	DFFA	.	.	.	A	G	-	-	0.26315789	14,0,5,0	chr1,105224	Cytosol	A > G
chr1:10522426:T:C:intronic:DFFA	chr1	10522426	10522426	T	C	intronic	DFFA	.	.	.	A	G	-	-	0.2	12,0,3,0	chr1,105224	Cytosol	A > G
chr1:10637067:A:G:intronic:PEX14	chr1	10637067	10637067	A	G	intronic	PEX14	.	.	.	A	G	+	+	1	0,0,8,0	chr1,106370	Cytosol	A > G
chr1:10637073:A:G:intronic:PEX14	chr1	10637073	10637073	A	G	intronic	PEX14	.	.	.	A	G	+	+	0.44444444	5,0,4,0	chr1,106370	Cytosol	A > G
chr1:11080926:A:G:intronic:TARDI	chr1	11080926	11080926	A	G	intronic	TARDBP	.	.	.	A	G	+	+	0.42857143	4,0,3,0	chr1,110809	Cytosol	A > G
chr1:11081325:A:G:intronic:TARDI	chr1	11081325	11081325	A	G	intronic	TARDBP	.	.	.	A	G	+	+	0.25	6,0,2,0	chr1,110813	Cytosol	A > G
chr1:11081942:A:G:intronic:TARDI	chr1	11081942	11081942	A	G	intronic	TARDBP	.	.	.	A	G	+	+	0.5	7,0,7,0	chr1,110819	Cytosol	A > G
chr1:15817289:T:C:intronic:CELA2	chr1	15817289	15817289	T	C	intronic	CELA2B	.	.	.	A	G	-	+	0.33333333	6,0,3,0	chr1,158172	Cytosol	A > G
chr1:15818008:T:C:UTR3:CASP9	chr1	15818008	15818008	T	C	UTR3	CASP9	ENST000005	.	.	A	G	-	-	0.2	12,0,3,0	chr1,158180	Cytosol	A > G
chr1:15818035:T:C:UTR3:CASP9	chr1	15818035	15818035	T	C	UTR3	CASP9	ENST000005	.	.	A	G	-	-	0.28571429	15,0,6,0	chr1,158180	Cytosol	A > G
chr1:15818247:T:C:UTR3:CASP9	chr1	15818247	15818247	T	C	UTR3	CASP9	ENST000005	.	.	A	G	-	-	0.375	5,0,3,0	chr1,158182	Cytosol	A > G
chr1:15896956:T:C:UTR3:DNAJC1	chr1	15896956	15896956	T	C	UTR3	DNAJC16	ENST000004	.	.	A	G	-	+	0.3	7,0,3,0	chr1,158969	Cytosol	A > G
chr1:15898474:T:C:intronic:DNAJC	chr1	15898474	15898474	T	C	intronic	DNAJC16	.	.	.	A	G	-	+-	1	0,0,55,0	chr1,158984	Cytosol	A > G
chr1:15898477:T:C:intronic:DNAJC	chr1	15898477	15898477	T	C	intronic	DNAJC16	.	.	.	A	G	-	+-	0.35849057	34,0,19,0	chr1,158984	Cytosol	A > G
chr1:15898516:T:C:intronic:DNAJC	chr1	15898516	15898516	T	C	intronic	DNAJC16	.	.	.	A	G	-	+-	0.7	12,0,28,0	chr1,158985	Cytosol	A > G
chr1:15898524:T:C:intronic:DNAJC	chr1	15898524	15898524	T	C	intronic	DNAJC16	.	.	.	A	G	-	+-	0.86486486	5,0,32,0	chr1,158985	Cytosol	A > G
chr1:15898583:T:C:intronic:DNAJC	chr1	15898583	15898583	T	C	intronic	DNAJC16	.	.	.	A	G	-	+-	0.56521739	10,0,13,0	chr1,158985	Cytosol	A > G
chr1:15898907:T:C:UTR3:AGMAT	chr1	15898907	15898907	T	C	UTR3	AGMAT	ENST000003	.	.	A	G	-	+-	0.84615385	4,0,22,0	chr1,158989	Cytosol	A > G
chr1:15898912:T:C:UTR3:AGMAT	chr1	15898912	15898912	T	C	UTR3	AGMAT	ENST000003	.	.	A	G	-	+-	0.06896552	27,0,2,0	chr1,158989	Cytosol	A > G
chr1:15898917:T:C:UTR3:AGMAT	chr1	15898917	15898917	T	C	UTR3	AGMAT	ENST000003	.	.	A	G	-	+-	0.42857143	16,0,12,0	chr1,158989	Cytosol	A > G
chr1:15898951:T:C:UTR3:AGMAT	chr1	15898951	15898951	T	C	UTR3	AGMAT	ENST000003	.	.	A	G	-	+-	0.41666667	14,0,10,0	chr1,158989	Cytosol	A > G
chr1:15898952:T:C:UTR3:AGMAT	chr1	15898952	15898952	T	C	UTR3	AGMAT	ENST000003	.	.	A	G	-	+-	0.76923077	6,0,20,0	chr1,158989	Cytosol	A > G
chr1:15898975:T:C:UTR3:AGMAT	chr1	15898975	15898975	T	C	UTR3	AGMAT	ENST000003	.	.	A	G	-	+-	0.47619048	11,0,10,0	chr1,158989	Cytosol	A > G
chr1:15898979:T:C:UTR3:AGMAT	chr1	15898979	15898979	T	C	UTR3	AGMAT	ENST000003	.	.	A	G	-	+-	0.16666667	20,0,4,0	chr1,158989	Cytosol	A > G
chr1:15898983:T:C:UTR3:AGMAT	chr1	15898983	15898983	T	C	UTR3	AGMAT	ENST000003	.	.	A	G	-	+-	0.31818182	15,0,7,0	chr1,158989	Cytosol	A > G
chr1:15898987:T:C:UTR3:AGMAT	chr1	15898987	15898987	T	C	UTR3	AGMAT	ENST000003	.	.	A	G	-	+-	0.95454545	1,0,21,0	chr1,158989	Cytosol	A > G
chr1:15898993:T:C:UTR3:AGMAT	chr1	15898993	15898993	T	C	UTR3	AGMAT	ENST000003	.	.	A	G	-	+-	0.81818182	4,0,18,0	chr1,158989	Cytosol	A > G
chr1:15898995:T:C:UTR3:AGMAT	chr1	15898995	15898995	T	C	UTR3	AGMAT	ENST000003	.	.	A	G	-	+-	0.86363636	3,0,19,0	chr1,158989	Cytosol	A > G
chr1:15899019:T:C:UTR3:AGMAT	chr1	15899019	15899019	T	C	UTR3	AGMAT	ENST000003	.	.	A	G	-	+-	0.46666667	8,0,7,0	chr1,158990	Cytosol	A > G
chr1:15899026:T:C:UTR3:AGMAT	chr1	15899026	15899026	T	C	UTR3	AGMAT	ENST000003	.	.	A	G	-	+-	0.5	7,0,7,0	chr1,158990	Cytosol	A > G
chr1:15899028:T:C:UTR3:AGMAT	chr1	15899028	15899028	T	C	UTR3	AGMAT	ENST000003	.	.	A	G	-	+-	0.46666667	8,0,7,0	chr1,158990	Cytosol	A > G
chr1:15899030:T:C:UTR3:AGMAT	chr1	15899030	15899030	T	C	UTR3	AGMAT	ENST000003	.	.	A	G	-	+-	0.78571429	3,0,11,0	chr1,158990	Cytosol	A > G

chr1:15899031:T:C:UTR3:AGMAT	chr1	15899031	15899031	T	C	UTR3	AGMAT	ENST000003	.	.	A	G	-	+-	0.625	6,0,10,0	chr1,158990	Cytosol	A > G
chr1:15899037:T:C:UTR3:AGMAT	chr1	15899037	15899037	T	C	UTR3	AGMAT	ENST000003	.	.	A	G	-	+-	0.15789474	16,0,3,0	chr1,158990	Cytosol	A > G
chr1:15899097:T:C:UTR3:AGMAT	chr1	15899097	15899097	T	C	UTR3	AGMAT	ENST000003	.	.	A	G	-	+-	0.72727273	3,0,8,0	chr1,158990	Cytosol	A > G
chr1:15899127:T:C:UTR3:AGMAT	chr1	15899127	15899127	T	C	UTR3	AGMAT	ENST000003	.	.	A	G	-	+-	0.11111111	16,0,2,0	chr1,158991	Cytosol	A > G
chr1:15899387:T:C:UTR3:AGMAT	chr1	15899387	15899387	T	C	UTR3	AGMAT	ENST000003	.	.	A	G	-	+-	0.23611111	55,0,17,0	chr1,158993	Cytosol	A > G
chr1:15899537:T:C:UTR3:AGMAT	chr1	15899537	15899537	T	C	UTR3	AGMAT	ENST000003	.	.	A	G	-	+-	0.3	7,0,3,0	chr1,158995	Cytosol	A > G
chr1:15899588:T:C:UTR3:AGMAT	chr1	15899588	15899588	T	C	UTR3	AGMAT	ENST000003	.	.	A	G	-	+-	0.46666667	16,0,14,0	chr1,158995	Cytosol	A > G
chr1:15899630:T:C:UTR3:AGMAT	chr1	15899630	15899630	T	C	UTR3	AGMAT	ENST000003	.	.	A	G	-	+-	0.33333333	42,0,21,0	chr1,158996	Cytosol	A > G
chr1:15899691:T:C:UTR3:AGMAT	chr1	15899691	15899691	T	C	UTR3	AGMAT	ENST000003	.	.	A	G	-	+-	0.27272727	56,0,21,0	chr1,158996	Cytosol	A > G
chr1:15899715:T:C:UTR3:AGMAT	chr1	15899715	15899715	T	C	UTR3	AGMAT	ENST000003	.	.	A	G	-	+-	0.35135135	48,0,26,0	chr1,158997	Cytosol	A > G
chr1:15899747:T:C:UTR3:AGMAT	chr1	15899747	15899747	T	C	UTR3	AGMAT	ENST000003	.	.	A	G	-	+-	0.77083333	11,0,37,0	chr1,158997	Cytosol	A > G
chr1:15993699:C:A:UTR3:DDI2	chr1	15993699	15993699	C	A	UTR3	DDI2	ENST000004	.	.	C	A	+	+	0.3125	5,11,0,0	chr1,159936	Cytosol	C > A
chr1:16555605:T:C:intronic:C1orf1	chr1	16555605	16555605	T	C	intronic	C1orf134	.	.	.	A	G	-	-	0.6	8,0,12,0	chr1,165556	Cytosol	A > G
chr1:16555606:T:C:intronic:C1orf1	chr1	16555606	16555606	T	C	intronic	C1orf134	.	.	.	A	G	-	-	0.75	5,0,15,0	chr1,165556	Cytosol	A > G
chr1:16555613:T:C:intronic:C1orf1	chr1	16555613	16555613	T	C	intronic	C1orf134	.	.	.	A	G	-	-	0.15	17,0,3,0	chr1,165556	Cytosol	A > G
chr1:16555632:T:C:intronic:C1orf1	chr1	16555632	16555632	T	C	intronic	C1orf134	.	.	.	A	G	-	-	0.15	17,0,3,0	chr1,165556	Cytosol	A > G
chr1:16555687:T:C:intronic:C1orf1	chr1	16555687	16555687	T	C	intronic	C1orf134	.	.	.	A	G	-	-	0.15384615	11,0,2,0	chr1,165556	Cytosol	A > G
chr1:16555735:T:C:intronic:C1orf1	chr1	16555735	16555735	T	C	intronic	C1orf134	.	.	.	A	G	-	-	0.11111111	16,0,2,0	chr1,165557	Cytosol	A > G
chr1:16557579:T:C:downstream:R	chr1	16557579	16557579	T	C	downstream	RSG1	.	.	.	A	G	-		0.12	22,0,3,0	chr1,165575	Cytosol	A > G
chr1:16557580:T:C:downstream:R	chr1	16557580	16557580	T	C	downstream	RSG1	.	.	.	A	G	-		0.21428571	22,0,6,0	chr1,165575	Cytosol	A > G
chr1:16557744:T:C:downstream:R	chr1	16557744	16557744	T	C	downstream	RSG1	.	.	.	A	G	-		0.27777778	13,0,5,0	chr1,165577	Cytosol	A > G
chr1:16557774:T:C:downstream:R	chr1	16557774	16557774	T	C	downstream	RSG1	.	.	.	A	G	-		0.25	6,0,2,0	chr1,165577	Cytosol	A > G
chr1:16557790:T:C:downstream:R	chr1	16557790	16557790	T	C	downstream	RSG1	.	.	.	A	G	-		0.25	6,0,2,0	chr1,165577	Cytosol	A > G
chr1:19541682:T:C:ncRNA_introni	chr1	19541682	19541682	T	C	ncRNA_intro	RP1-43E13.2	.	.	.	A	G	-	+	0.38888889	11,0,7,0	chr1,195416	Cytosol	A > G
chr1:19542250:T:C:ncRNA_introni	chr1	19542250	19542250	T	C	ncRNA_intro	RP1-43E13.2	.	.	.	A	G	-	+-	0.66666667	5,0,10,0	chr1,195422	Cytosol	A > G
chr1:19542337:T:C:ncRNA_introni	chr1	19542337	19542337	T	C	ncRNA_intro	RP1-43E13.2	.	.	.	A	G	-	+-	0.09677419	28,0,3,0	chr1,195423	Cytosol	A > G
chr1:19542344:T:C:ncRNA_introni	chr1	19542344	19542344	T	C	ncRNA_intro	RP1-43E13.2	.	.	.	A	G	-	+-	0.16	21,0,4,0	chr1,195423	Cytosol	A > G
chr1:19542401:T:C:ncRNA_introni	chr1	19542401	19542401	T	C	ncRNA_intro	RP1-43E13.2	.	.	.	A	G	-	+-	0.13043478	20,0,3,0	chr1,195424	Cytosol	A > G
chr1:19542415:T:C:ncRNA_introni	chr1	19542415	19542415	T	C	ncRNA_intro	RP1-43E13.2	.	.	.	A	G	-	+-	0.13333333	13,0,2,0	chr1,195424	Cytosol	A > G
chr1:19542433:T:C:ncRNA_introni	chr1	19542433	19542433	T	C	ncRNA_intro	RP1-43E13.2	.	.	.	A	G	-	+-	0.1	18,0,2,0	chr1,195424	Cytosol	A > G
chr1:19542455:T:C:ncRNA_introni	chr1	19542455	19542455	T	C	ncRNA_intro	RP1-43E13.2	.	.	.	A	G	-	+-	0.21428571	11,0,3,0	chr1,195424	Cytosol	A > G
chr1:19542456:T:C:ncRNA_introni	chr1	19542456	19542456	T	C	ncRNA_intro	RP1-43E13.2	.	.	.	A	G	-	+-	0.14285714	12,0,2,0	chr1,195424	Cytosol	A > G
chr1:19542463:T:C:ncRNA_introni	chr1	19542463	19542463	T	C	ncRNA_intro	RP1-43E13.2	.	.	.	A	G	-	+-	0.4	9,0,6,0	chr1,195424	Cytosol	A > G
chr1:19542609:T:C:ncRNA_introni	chr1	19542609	19542609	T	C	ncRNA_intro	RP1-43E13.2	.	.	.	A	G	-	+-	0.06521739	43,0,3,0	chr1,195426	Cytosol	A > G
chr1:19542621:T:C:ncRNA_introni	chr1	19542621	19542621	T	C	ncRNA_intro	RP1-43E13.2	.	.	.	A	G	-	+-	0.11320755	47,0,6,0	chr1,195426	Cytosol	A > G
chr1:19542631:T:C:ncRNA_introni	chr1	19542631	19542631	T	C	ncRNA_intro	RP1-43E13.2	.	.	.	A	G	-	+-	0.31914894	32,0,15,0	chr1,195426	Cytosol	A > G
chr1:19543247:T:C:ncRNA_introni	chr1	19543247	19543247	T	C	ncRNA_intro	RP1-43E13.2	.	.	.	A	G	-	+-	0.35	13,0,7,0	chr1,195432	Cytosol	A > G
chr1:19543249:T:C:ncRNA_introni	chr1	19543249	19543249	T	C	ncRNA_intro	RP1-43E13.2	.	.	.	A	G	-	+-	0.27777778	13,0,5,0	chr1,195432	Cytosol	A > G
chr1:19543282:T:C:ncRNA_introni	chr1	19543282	19543282	T	C	ncRNA_intro	RP1-43E13.2	.	.	.	A	G	-	+-	0.125	21,0,3,0	chr1,195432	Cytosol	A > G
chr1:19543349:T:C:ncRNA_introni	chr1	19543349	19543349	T	C	ncRNA_intro	RP1-43E13.2	.	.	.	A	G	-	+-	0.79166667	5,0,19,0	chr1,195433	Cytosol	A > G
chr1:19543353:T:C:ncRNA_introni	chr1	19543353	19543353	T	C	ncRNA_intro	RP1-43E13.2	.	.	.	A	G	-	+-	0.08695652	21,0,2,0	chr1,195433	Cytosol	A > G
chr1:19543356:T:C:ncRNA_introni	chr1	19543356	19543356	T	C	ncRNA_intro	RP1-43E13.2	.	.	.	A	G	-	+-	0.09090909	20,0,2,0	chr1,195433	Cytosol	A > G
chr1:19543360:T:C:ncRNA_introni	chr1	19543360	19543360	T	C	ncRNA_intro	RP1-43E13.2	.	.	.	A	G	-	+-	0.23076923	20,0,6,0	chr1,195433	Cytosol	A > G
chr1:19543373:T:C:ncRNA_introni	chr1	19543373	19543373	T	C	ncRNA_intro	RP1-43E13.2	.	.	.	A	G	-	+-	0.08	23,0,2,0	chr1,195433	Cytosol	A > G
chr1:19543456:T:C:ncRNA_introni	chr1	19543456	19543456	T	C	ncRNA_intro	RP1-43E13.2	.	.	.	A	G	-	+-	0.26470588	25,0,9,0	chr1,195434	Cytosol	A > G
chr1:19543457:T:C:ncRNA_introni	chr1	19543457	19543457	T	C	ncRNA_intro	RP1-43E13.2	.	.	.	A	G	-	+-	0.31428571	24,0,11,0	chr1,195434	Cytosol	A > G
chr1:19543459:T:C:ncRNA_introni	chr1	19543459	19543459	T	C	ncRNA_intro	RP1-43E13.2	.	.	.	A	G	-	+-	0.72222222	10,0,26,0	chr1,195434	Cytosol	A > G
chr1:19543465:T:C:ncRNA_introni	chr1	19543465	19543465	T	C	ncRNA_intro	RP1-43E13.2	.	.	.	A	G	-	+-	0.37837838	23,0,14,0	chr1,195434	Cytosol	A > G
chr1:19543785:T:C:ncRNA_introni	chr1	19543785	19543785	T	C	ncRNA_intro	RP1-43E13.2	.	.	.	A	G	-	+-	0.47368421	10,0,9,0	chr1,195437	Cytosol	A > G
chr1:19543797:T:C:ncRNA_introni	chr1	19543797	19543797	T	C	ncRNA_intro	RP1-43E13.2	.	.	.	A	G	-	+-	0.44444444	5,0,4,0	chr1,195437	Cytosol	A > G
chr1:19543850:T:C:ncRNA_introni	chr1	19543850	19543850	T	C	ncRNA_intro	RP1-43E13.2	.	.	.	A	G	-	+-	0.6	6,0,9,0	chr1,195438	Cytosol	A > G
chr1:19543879:T:C:ncRNA_introni	chr1	19543879	19543879	T	C	ncRNA_intro	RP1-43E13.2	.	.	.	A	G	-	+-	0.25	27,0,9,0	chr1,195438	Cytosol	A > G
chr1:19543973:T:C:ncRNA_introni	chr1	19543973	19543973	T	C	ncRNA_intro	RP1-43E13.2	.	.	.	A	G	-	+-	0.25	30,0,10,0	chr1,195439	Cytosol	A > G
chr1:19544009:T:C:ncRNA_introni	chr1	19544009	19544009	T	C	ncRNA_intro	RP1-43E13.2	.	.	.	A	G	-	+-	0.27272727	8,0,3,0	chr1,195440	Cytosol	A > G
chr1:19585493:A:G:UTR3:MRTO4	chr1	19585493	19585493	A	G	UTR3	MRTO4	ENST000003	.	.	A	G	+	+	0.08365019	241,0,22,0	chr1,195854	Cytosol	A > G
chr1:19585502:A:G:UTR3:MRTO4	chr1	19585502	19585502	A	G	UTR3	MRTO4	ENST000003	.	.	A	G	+	+	0.03137255	247,0,8,0	chr1,195855	Cytosol	A > G
chr1:19585519:A:G:UTR3:MRTO4	chr1	19585519	19585519	A	G	UTR3	MRTO4	ENST000003	.	.	A	G	+	+	0.11969112	228,0,31,0	chr1,195855	Cytosol	A > G
chr1:19585553:A:G:UTR3:MRTO4	chr1	19585553	19585553	A	G	UTR3	MRTO4	ENST000003	.	.	A	G	+	+	0.2890625	182,0,74,0	chr1,195855	Cytosol	A > G
chr1:19585554:A:G:UTR3:MRTO4	chr1	19585554	19585554	A	G	UTR3	MRTO4	ENST000003	.	.	A	G	+	+	0.03937008	244,0,10,0	chr1,195855	Cytosol	A > G
chr1:19586437:A:G:UTR3:MRTO4	chr1	19586437	19586437	A	G	UTR3	MRTO4	ENST000003	.	.	A	G	+	+	0.03921569	196,0,8,0	chr1,195864	Cytosol	A > G
chr1:19586455:A:G:UTR3:MRTO4	chr1	19586455	19586455	A	G	UTR3	MRTO4	ENST000003	.	.	A	G	+	+	0.14084507	122,0,20,0	chr1,195864	Cytosol	A > G
chr1:19586458:A:G:UTR3:MRTO4	chr1	19586458	19586458	A	G	UTR3	MRTO4	ENST000003	.	.	A	G	+	+	0.7027027	44,0,104,0	chr1,195864	Cytosol	A > G
chr1:19586501:A:G:UTR3:MRTO4	chr1	19586501	19586501	A	G	UTR3	MRTO4	ENST000003	.	.	A	G	+	+	0.77011494	20,0,67,0	chr1,195865	Cytosol	A > G
chr1:19586519:A:G:UTR3:MRTO4	chr1	19586519	19586519	A	G	UTR3	MRTO4	ENST000003	.	.	A	G	+	+	0.0989011	82,0,9,0	chr1,195865	Cytosol	A > G

chr1:19586520:A:G:UTR3:MRTO4	chr1	19586520	19586520	A	G	UTR3	MRTO4	ENST000003	.	.	A	G	+	+	0.12790698	75,0,11,0	chr1,195865	Cytosol	A > G
chr1:19586532:A:G:UTR3:MRTO4	chr1	19586532	19586532	A	G	UTR3	MRTO4	ENST000003	.	.	A	G	+	+	0.15789474	80,0,15,0	chr1,195865	Cytosol	A > G
chr1:19586541:A:G:UTR3:MRTO4	chr1	19586541	19586541	A	G	UTR3	MRTO4	ENST000003	.	.	A	G	+	+	0.04672897	102,0,5,0	chr1,195865	Cytosol	A > G
chr1:19620130:T:C:intergenic:AKR	chr1	19620130	19620130	T	C	intergenic	AKR7A3,AKR	dist=4386;di	.	.	A	G	-	-	0.6	2,0,3,0	chr1,196201	Cytosol	A > G
chr1:19620131:T:C:intergenic:AKR	chr1	19620131	19620131	T	C	intergenic	AKR7A3,AKR	dist=4387;di	.	.	A	G	-	-	0.6	2,0,3,0	chr1,196201	Cytosol	A > G
chr1:19620137:T:C:intergenic:AKR	chr1	19620137	19620137	T	C	intergenic	AKR7A3,AKR	dist=4393;di	.	.	A	G	-	-	0.66666667	2,0,4,0	chr1,196201	Cytosol	A > G
chr1:19620625:T:C:intergenic:AKR	chr1	19620625	19620625	T	C	intergenic	AKR7A3,AKR	dist=4881;di	.	.	A	G	-	-	0.4	3,0,2,0	chr1,196206	Cytosol	A > G
chr1:19625146:T:C:intergenic:AKR	chr1	19625146	19625146	T	C	intergenic	AKR7A3,AKR	dist=9402;di	.	.	A	G	-		0.375	5,0,3,0	chr1,196251	Cytosol	A > G
chr1:19625214:T:C:intergenic:AKR	chr1	19625214	19625214	T	C	intergenic	AKR7A3,AKR	dist=9470;di	.	.	A	G	-		0.38461538	8,0,5,0	chr1,196252	Cytosol	A > G
chr1:19655630:A:G:UTR3:PQLC2	chr1	19655630	19655630	A	G	UTR3	PQLC2	ENST000003	.	.	A	G	+	+	0.30508475	41,0,18,0	chr1,196556	Cytosol	A > G
chr1:20975993:T:C:ncRNA_exonic:	chr1	20975993	20975993	T	C	ncRNA_exon	PINK1-AS	.	.	.	A	G	-	-+	0.25	6,0,2,0	chr1,209759	Cytosol	A > G
chr1:20976001:T:C:ncRNA_exonic:	chr1	20976001	20976001	T	C	ncRNA_exon	PINK1-AS	.	.	.	A	G	-	-+	0.66666667	2,0,4,0	chr1,209760	Cytosol	A > G
chr1:20976075:T:C:ncRNA_exonic:	chr1	20976075	20976075	T	C	ncRNA_exon	PINK1-AS	.	.	.	A	G	-	-+	1	0,0,5,0	chr1,209760	Cytosol	A > G
chr1:20976078:T:C:ncRNA_exonic:	chr1	20976078	20976078	T	C	ncRNA_exon	PINK1-AS	.	.	.	A	G	-	-+	1	0,0,5,0	chr1,209760	Cytosol	A > G
chr1:20976095:T:C:ncRNA_exonic:	chr1	20976095	20976095	T	C	ncRNA_exon	PINK1-AS	.	.	.	A	G	-	-+	0.625	3,0,5,0	chr1,209760	Cytosol	A > G
chr1:20976110:T:C:ncRNA_exonic:	chr1	20976110	20976110	T	C	ncRNA_exon	PINK1-AS	.	.	.	A	G	-	-+	0.42857143	4,0,3,0	chr1,209761	Cytosol	A > G
chr1:20976118:T:C:ncRNA_exonic:	chr1	20976118	20976118	T	C	ncRNA_exon	PINK1-AS	.	.	.	A	G	-	-+	0.57142857	3,0,4,0	chr1,209761	Cytosol	A > G
chr1:20976174:T:C:ncRNA_exonic:	chr1	20976174	20976174	T	C	ncRNA_exon	PINK1-AS	.	.	.	A	G	-	-+	0.6	2,0,3,0	chr1,209761	Cytosol	A > G
chr1:20978381:T:C:ncRNA_exonic:	chr1	20978381	20978381	T	C	ncRNA_exon	PINK1-AS	.	.	.	A	G	-	-	0.01718213	286,0,5,0	chr1,209783	Cytosol	A > G
chr1:20978384:T:C:ncRNA_exonic:	chr1	20978384	20978384	T	C	ncRNA_exon	PINK1-AS	.	.	.	A	G	-	-	0.01020408	291,0,3,0	chr1,209783	Cytosol	A > G
chr1:20978451:T:C:ncRNA_exonic:	chr1	20978451	20978451	T	C	ncRNA_exon	PINK1-AS	.	.	.	A	G	-	-	0.05353319	442,0,25,0	chr1,209784	Cytosol	A > G
chr1:20978457:T:C:ncRNA_exonic:	chr1	20978457	20978457	T	C	ncRNA_exon	PINK1-AS	.	.	.	A	G	-	-	0.11816193	403,0,54,0	chr1,209784	Cytosol	A > G
chr1:20978483:T:C:ncRNA_exonic:	chr1	20978483	20978483	T	C	ncRNA_exon	PINK1-AS	.	.	.	A	G	-	-	0.08227848	290,0,26,0	chr1,209784	Cytosol	A > G
chr1:20978498:T:C:ncRNA_exonic:	chr1	20978498	20978498	T	C	ncRNA_exon	PINK1-AS	.	.	.	A	G	-	-	0.01485149	199,0,3,0	chr1,209784	Cytosol	A > G
chr1:20978513:T:C:ncRNA_exonic:	chr1	20978513	20978513	T	C	ncRNA_exon	PINK1-AS	.	.	.	A	G	-	-	0.03349282	202,0,7,0	chr1,209785	Cytosol	A > G
chr1:20978516:T:C:ncRNA_exonic:	chr1	20978516	20978516	T	C	ncRNA_exon	PINK1-AS	.	.	.	A	G	-	-	0.07786885	225,0,19,0	chr1,209785	Cytosol	A > G
chr1:20978523:T:C:ncRNA_exonic:	chr1	20978523	20978523	T	C	ncRNA_exon	PINK1-AS	.	.	.	A	G	-	-	0.06024096	234,0,15,0	chr1,209785	Cytosol	A > G
chr1:20978564:T:C:ncRNA_exonic:	chr1	20978564	20978564	T	C	ncRNA_exon	PINK1-AS	.	.	.	A	G	-	-	0.03916449	368,0,15,0	chr1,209785	Cytosol	A > G
chr1:20978590:T:C:ncRNA_exonic:	chr1	20978590	20978590	T	C	ncRNA_exon	PINK1-AS	.	.	.	A	G	-	-	0.03508772	385,0,14,0	chr1,209785	Cytosol	A > G
chr1:20978591:T:C:ncRNA_exonic:	chr1	20978591	20978591	T	C	ncRNA_exon	PINK1-AS	.	.	.	A	G	-	-	0.01449275	408,0,6,0	chr1,209785	Cytosol	A > G
chr1:21081804:T:C:intronic:HP1BP	chr1	21081804	21081804	T	C	intronic	HP1BP3	.	.	.	A	G	-	-	0.46666667	32,0,28,0	chr1,210818	Cytosol	A > G
chr1:21081817:T:C:intronic:HP1BP	chr1	21081817	21081817	T	C	intronic	HP1BP3	.	.	.	A	G	-	-	0.08695652	42,0,4,0	chr1,210818	Cytosol	A > G
chr1:21081826:T:C:intronic:HP1BP	chr1	21081826	21081826	T	C	intronic	HP1BP3	.	.	.	A	G	-	-	0.17647059	28,0,6,0	chr1,210818	Cytosol	A > G
chr1:21081840:T:C:intronic:HP1BP	chr1	21081840	21081840	T	C	intronic	HP1BP3	.	.	.	A	G	-	-	0.17241379	24,0,5,0	chr1,210818	Cytosol	A > G
chr1:21081899:T:C:intronic:HP1BP	chr1	21081899	21081899	T	C	intronic	HP1BP3	.	.	.	A	G	-	-	0.26666667	10,0,4,1	chr1,210818	Cytosol	A > G
chr1:21081936:T:C:intronic:HP1BP	chr1	21081936	21081936	T	C	intronic	HP1BP3	.	.	.	A	G	-	-	0.08	23,0,2,0	chr1,210819	Cytosol	A > G
chr1:21081956:T:C:intronic:HP1BP	chr1	21081956	21081956	T	C	intronic	HP1BP3	.	.	.	A	G	-	-	0.26666667	22,0,8,0	chr1,210819	Cytosol	A > G
chr1:21081958:T:C:intronic:HP1BP	chr1	21081958	21081958	T	C	intronic	HP1BP3	.	.	.	A	G	-	-	0.33333333	16,0,8,0	chr1,210819	Cytosol	A > G
chr1:21081959:T:C:intronic:HP1BP	chr1	21081959	21081959	T	C	intronic	HP1BP3	.	.	.	A	G	-	-	0.20833333	19,0,5,0	chr1,210819	Cytosol	A > G
chr1:21081981:T:C:intronic:HP1BP	chr1	21081981	21081981	T	C	intronic	HP1BP3	.	.	.	A	G	-	-	0.08	23,0,2,0	chr1,210819	Cytosol	A > G
chr1:21081982:T:C:intronic:HP1BP	chr1	21081982	21081982	T	C	intronic	HP1BP3	.	.	.	A	G	-	-	0.25925926	20,0,7,0	chr1,210819	Cytosol	A > G
chr1:21081985:T:C:intronic:HP1BP	chr1	21081985	21081985	T	C	intronic	HP1BP3	.	.	.	A	G	-	-	0.26923077	19,0,7,0	chr1,210819	Cytosol	A > G
chr1:21082054:T:C:intronic:HP1BP	chr1	21082054	21082054	T	C	intronic	HP1BP3	.	.	.	A	G	-	-	0.16666667	15,0,3,0	chr1,210820	Cytosol	A > G
chr1:21082062:T:C:intronic:HP1BP	chr1	21082062	21082062	T	C	intronic	HP1BP3	.	.	.	A	G	-	-	0.63636364	8,0,14,0	chr1,210820	Cytosol	A > G
chr1:21082987:T:C:intronic:HP1BP	chr1	21082987	21082987	T	C	intronic	HP1BP3	.	.	.	A	G	-	-	0.3125	11,0,5,0	chr1,210829	Cytosol	A > G
chr1:21083251:T:C:intronic:HP1BP	chr1	21083251	21083251	T	C	intronic	HP1BP3	.	.	.	A	G	-	-	0.86046512	6,0,37,0	chr1,210832	Cytosol	A > G
chr1:21083256:T:C:intronic:HP1BP	chr1	21083256	21083256	T	C	intronic	HP1BP3	.	.	.	A	G	-	-	0.10869565	41,0,5,0	chr1,210832	Cytosol	A > G
chr1:21083274:T:C:intronic:HP1BP	chr1	21083274	21083274	T	C	intronic	HP1BP3	.	.	.	A	G	-	-	0.16	21,0,4,0	chr1,210832	Cytosol	A > G
chr1:21083276:T:C:intronic:HP1BP	chr1	21083276	21083276	T	C	intronic	HP1BP3	.	.	.	A	G	-	-	0.625	9,0,15,0	chr1,210832	Cytosol	A > G
chr1:21083305:T:C:intronic:HP1BP	chr1	21083305	21083305	T	C	intronic	HP1BP3	.	.	.	A	G	-	-	0.21428571	11,0,3,0	chr1,210833	Cytosol	A > G
chr1:21083337:T:C:intronic:HP1BP	chr1	21083337	21083337	T	C	intronic	HP1BP3	.	.	.	A	G	-	-	0.27777778	13,0,5,0	chr1,210833	Cytosol	A > G
chr1:21083340:T:C:intronic:HP1BP	chr1	21083340	21083340	T	C	intronic	HP1BP3	.	.	.	A	G	-	-	0.73684211	5,0,14,0	chr1,210833	Cytosol	A > G
chr1:21083405:T:C:intronic:HP1BP	chr1	21083405	21083405	T	C	intronic	HP1BP3	.	.	.	A	G	-	-	0.54545455	10,0,12,0	chr1,210834	Cytosol	A > G
chr1:21083470:T:C:intronic:HP1BP	chr1	21083470	21083470	T	C	intronic	HP1BP3	.	.	.	A	G	-	-	0.41666667	7,0,5,0	chr1,210834	Cytosol	A > G
chr1:21083475:T:C:intronic:HP1BP	chr1	21083475	21083475	T	C	intronic	HP1BP3	.	.	.	A	G	-	-	0.36363636	7,0,4,0	chr1,210834	Cytosol	A > G
chr1:21083476:T:C:intronic:HP1BP	chr1	21083476	21083476	T	C	intronic	HP1BP3	.	.	.	A	G	-	-	0.53846154	6,0,7,0	chr1,210834	Cytosol	A > G
chr1:21083482:T:C:intronic:HP1BP	chr1	21083482	21083482	T	C	intronic	HP1BP3	.	.	.	A	G	-	-	0.16666667	10,0,2,0	chr1,210834	Cytosol	A > G
chr1:21101368:T:C:UTR5:HP1BP3	chr1	21101368	21101368	T	C	UTR5	HP1BP3	ENST000003	.	.	A	G	-	-	0.28571429	5,0,2,0	chr1,211013	Cytosol	A > G
chr1:21101369:T:C:UTR5:HP1BP3	chr1	21101369	21101369	T	C	UTR5	HP1BP3	ENST000003	.	.	A	G	-	-	0.28571429	5,0,2,0	chr1,211013	Cytosol	A > G
chr1:21101903:T:C:intronic:HP1BP	chr1	21101903	21101903	T	C	intronic	HP1BP3	.	.	.	A	G	-	-	0.83333333	2,0,10,0	chr1,211019	Cytosol	A > G
chr1:21270757:T:C:intronic:EIF4G	chr1	21270757	21270757	T	C	intronic	EIF4G3	.	.	.	A	G	-	-	0.33333333	4,0,2,0	chr1,212707	Cytosol	A > G
chr1:23707607:T:C:UTR3:TCEA3	chr1	23707607	23707607	T	C	UTR3	TCEA3	ENST000004	.	.	A	G	-	-	0.2	8,0,2,0	chr1,237076	Cytosol	A > G
chr1:23707641:T:C:UTR3:TCEA3	chr1	23707641	23707641	T	C	UTR3	TCEA3	ENST000004	.	.	A	G	-	-	0.375	15,0,9,0	chr1,237076	Cytosol	A > G
chr1:26160986:T:C:UTR3:AUNIP	chr1	26160986	26160986	T	C	UTR3	AUNIP	ENST000003	.	.	A	G	-	-	0.05172414	55,0,3,0	chr1,261609	Cytosol	A > G

chr1:26582779:A:G:intronic:CEP85	chr1	26582779	26582779	A	G	intronic	CEP85	.	.	.	A	G	+	+	0.6	2,0,3,0	chr1,265827	Cytosol	A > G
chr1:26774224:A:G:exonic:DHDDS	chr1	26774224	26774224	A	G	exonic	DHDDS	.	synonymous	DHDDS:ENST	A	G	+	+	0.5	11,0,11,0	chr1,267742	Cytosol	A > G
chr1:26774225:A:G:exonic:DHDDS	chr1	26774225	26774225	A	G	exonic	DHDDS	.	nonsynonym	DHDDS:ENST	A	G	+	+	0.27272727	16,0,6,0	chr1,267742	Cytosol	A > G
chr1:26774238:A:G:exonic:DHDDS	chr1	26774238	26774238	A	G	exonic	DHDDS	.	stoploss	DHDDS:ENST	A	G	+	+	1	0,0,24,0	chr1,267742	Cytosol	A > G
chr1:26774282:A:G:UTR3:DHDDS	chr1	26774282	26774282	A	G	UTR3	DHDDS	ENST000003	.	.	A	G	+	+	0.1	18,0,2,0	chr1,267742	Cytosol	A > G
chr1:26774861:A:G:exonic:DHDDS	chr1	26774861	26774861	A	G	exonic	DHDDS	.	nonsynonym	DHDDS:ENST	A	G	+	+	0.33333333	6,0,3,0	chr1,267748	Cytosol	A > G
chr1:26774864:A:G:exonic:DHDDS	chr1	26774864	26774864	A	G	exonic	DHDDS	.	nonsynonym	DHDDS:ENST	A	G	+	+	0.16666667	10,0,2,0	chr1,267748	Cytosol	A > G
chr1:26774866:A:G:exonic:DHDDS	chr1	26774866	26774866	A	G	exonic	DHDDS	.	nonsynonym	DHDDS:ENST	A	G	+	+	1	0,0,11,0	chr1,267748	Cytosol	A > G
chr1:26774878:A:G:exonic:DHDDS	chr1	26774878	26774878	A	G	exonic	DHDDS	.	nonsynonym	DHDDS:ENST	A	G	+	+	0.5	5,0,5,0	chr1,267748	Cytosol	A > G
chr1:26774914:A:G:UTR3:DHDDS	chr1	26774914	26774914	A	G	UTR3	DHDDS	ENST000004	.	.	A	G	+	+	0.63636364	4,0,7,0	chr1,267749	Cytosol	A > G
chr1:26774923:A:G:UTR3:DHDDS	chr1	26774923	26774923	A	G	UTR3	DHDDS	ENST000004	.	.	A	G	+	+	0.4	6,0,4,0	chr1,267749	Cytosol	A > G
chr1:26774924:A:G:UTR3:DHDDS	chr1	26774924	26774924	A	G	UTR3	DHDDS	ENST000004	.	.	A	G	+	+	0.22222222	7,0,2,0	chr1,267749	Cytosol	A > G
chr1:27202789:T:C:ncRNA_exonic:	chr1	27202789	27202789	T	C	ncRNA_exon	RP1-50O24.6	.	.	.	A	G	-	+-	0.375	5,0,3,0	chr1,272027	Cytosol	A > G
chr1:27202835:T:C:ncRNA_introni	chr1	27202835	27202835	T	C	ncRNA_intro	RP1-50O24.6	.	.	.	A	G	-	+-	0.18181818	18,0,4,0	chr1,272028	Cytosol	A > G
chr1:27202844:T:C:ncRNA_introni	chr1	27202844	27202844	T	C	ncRNA_intro	RP1-50O24.6	.	.	.	A	G	-	+-	0.12903226	27,0,4,0	chr1,272028	Cytosol	A > G
chr1:27202845:T:C:ncRNA_introni	chr1	27202845	27202845	T	C	ncRNA_intro	RP1-50O24.6	.	.	.	A	G	-	+-	0.2	24,0,6,0	chr1,272028	Cytosol	A > G
chr1:27202888:T:C:ncRNA_introni	chr1	27202888	27202888	T	C	ncRNA_intro	RP1-50O24.6	.	.	.	A	G	-	+-	0.10714286	25,0,3,0	chr1,272028	Cytosol	A > G
chr1:27203000:T:C:ncRNA_introni	chr1	27203000	27203000	T	C	ncRNA_intro	RP1-50O24.6	.	.	.	A	G	-	+-	0.33333333	4,0,2,0	chr1,272029	Cytosol	A > G
chr1:27205224:T:C:UTR3:GPN2	chr1	27205224	27205224	T	C	UTR3	GPN2	ENST000003	.	.	A	G	-	+-	0.4	6,0,4,0	chr1,272052	Cytosol	A > G
chr1:27208108:T:C:intronic:GPN2	chr1	27208108	27208108	T	C	intronic	GPN2	.	.	.	A	G	-	+-	0.625	3,0,5,0	chr1,272081	Cytosol	A > G
chr1:27208621:T:C:intronic:GPN2	chr1	27208621	27208621	T	C	intronic	GPN2	.	.	.	A	G	-	+-	0.33333333	4,0,2,0	chr1,272086	Cytosol	A > G
chr1:27208625:T:C:intronic:GPN2	chr1	27208625	27208625	T	C	intronic	GPN2	.	.	.	A	G	-	+-	0.66666667	2,0,4,0	chr1,272086	Cytosol	A > G
chr1:28661576:A:G:UTR3:MED18	chr1	28661576	28661576	A	G	UTR3	MED18	ENST000003	.	.	A	G	+	+	0.85714286	1,0,6,0	chr1,286615	Cytosol	A > G
chr1:28661656:A:G:UTR3:MED18	chr1	28661656	28661656	A	G	UTR3	MED18	ENST000003	.	.	A	G	+	+	0.37288136	37,0,22,0	chr1,286616	Cytosol	A > G
chr1:28661670:A:G:UTR3:MED18	chr1	28661670	28661670	A	G	UTR3	MED18	ENST000003	.	.	A	G	+	+	0.04651163	41,0,2,0	chr1,286616	Cytosol	A > G
chr1:28661682:A:G:UTR3:MED18	chr1	28661682	28661682	A	G	UTR3	MED18	ENST000003	.	.	A	G	+	+	0.07142857	26,0,2,0	chr1,286616	Cytosol	A > G
chr1:28661692:A:G:UTR3:MED18	chr1	28661692	28661692	A	G	UTR3	MED18	ENST000003	.	.	A	G	+	+	0.27586207	21,0,8,0	chr1,286616	Cytosol	A > G
chr1:28661719:A:G:UTR3:MED18	chr1	28661719	28661719	A	G	UTR3	MED18	ENST000003	.	.	A	G	+	+	0.06451613	29,0,2,0	chr1,286617	Cytosol	A > G
chr1:28661733:A:G:UTR3:MED18	chr1	28661733	28661733	A	G	UTR3	MED18	ENST000003	.	.	A	G	+	+	0.10526316	34,0,4,0	chr1,286617	Cytosol	A > G
chr1:28661767:A:G:UTR3:MED18	chr1	28661767	28661767	A	G	UTR3	MED18	ENST000003	.	.	A	G	+	+	0.03773585	51,0,2,0	chr1,286617	Cytosol	A > G
chr1:28662072:A:G:UTR3:MED18	chr1	28662072	28662072	A	G	UTR3	MED18	ENST000003	.	.	A	G	+	+	0.11904762	37,0,5,0	chr1,286620	Cytosol	A > G
chr1:28662105:A:G:UTR3:MED18	chr1	28662105	28662105	A	G	UTR3	MED18	ENST000003	.	.	A	G	+	+	0.15555556	38,0,7,0	chr1,286621	Cytosol	A > G
chr1:28662171:A:G:UTR3:MED18	chr1	28662171	28662171	A	G	UTR3	MED18	ENST000003	.	.	A	G	+	+	0.07142857	26,0,2,0	chr1,286621	Cytosol	A > G
chr1:28662180:A:G:UTR3:MED18	chr1	28662180	28662180	A	G	UTR3	MED18	ENST000003	.	.	A	G	+	+	0.05263158	36,0,2,0	chr1,286621	Cytosol	A > G
chr1:28662182:A:G:UTR3:MED18	chr1	28662182	28662182	A	G	UTR3	MED18	ENST000003	.	.	A	G	+	+	0.10810811	33,0,4,0	chr1,286621	Cytosol	A > G
chr1:28662186:A:G:UTR3:MED18	chr1	28662186	28662186	A	G	UTR3	MED18	ENST000003	.	.	A	G	+	+	0.1875	26,0,6,0	chr1,286621	Cytosol	A > G
chr1:28662199:A:G:UTR3:MED18	chr1	28662199	28662199	A	G	UTR3	MED18	ENST000003	.	.	A	G	+	+	0.76470588	8,0,26,0	chr1,286621	Cytosol	A > G
chr1:28662213:A:G:UTR3:MED18	chr1	28662213	28662213	A	G	UTR3	MED18	ENST000003	.	.	A	G	+	+	0.10526316	34,0,4,0	chr1,286622	Cytosol	A > G
chr1:28662253:A:G:UTR3:MED18	chr1	28662253	28662253	A	G	UTR3	MED18	ENST000003	.	.	A	G	+	+	0.29850746	47,0,20,0	chr1,286622	Cytosol	A > G
chr1:28662254:A:G:UTR3:MED18	chr1	28662254	28662254	A	G	UTR3	MED18	ENST000003	.	.	A	G	+	+	0.25396825	47,0,16,0	chr1,286622	Cytosol	A > G
chr1:28662281:A:G:UTR3:MED18	chr1	28662281	28662281	A	G	UTR3	MED18	ENST000003	.	.	A	G	+	+	0.02777778	70,0,2,0	chr1,286622	Cytosol	A > G
chr1:28662323:A:G:UTR3:MED18	chr1	28662323	28662323	A	G	UTR3	MED18	ENST000003	.	.	A	G	+	+	0.03333333	58,0,2,0	chr1,286623	Cytosol	A > G
chr1:28824304:A:G:UTR3:PHACTR	chr1	28824304	28824304	A	G	UTR3	PHACTR4	ENST000003	.	.	A	G	+	+	0.17142857	29,0,6,0	chr1,288243	Cytosol	A > G
chr1:28824360:A:G:UTR3:PHACTR	chr1	28824360	28824360	A	G	UTR3	PHACTR4	ENST000003	.	.	A	G	+	+	0.07058824	79,0,6,0	chr1,288243	Cytosol	A > G
chr1:28825331:A:G:UTR3:PHACTR	chr1	28825331	28825331	A	G	UTR3	PHACTR4	ENST000003	.	.	A	G	+	+	0.42045455	51,0,37,0	chr1,288253	Cytosol	A > G
chr1:28825337:A:G:UTR3:PHACTR	chr1	28825337	28825337	A	G	UTR3	PHACTR4	ENST000003	.	.	A	G	+	+	0.19444444	87,0,21,0	chr1,288253	Cytosol	A > G
chr1:28825370:A:G:UTR3:PHACTR	chr1	28825370	28825370	A	G	UTR3	PHACTR4	ENST000003	.	.	A	G	+	+	0.56296296	59,0,76,0	chr1,288253	Cytosol	A > G
chr1:28825381:A:G:UTR3:PHACTR	chr1	28825381	28825381	A	G	UTR3	PHACTR4	ENST000003	.	.	A	G	+	+	0.01459854	135,0,2,0	chr1,288253	Cytosol	A > G
chr1:28825390:A:G:UTR3:PHACTR	chr1	28825390	28825390	A	G	UTR3	PHACTR4	ENST000003	.	.	A	G	+	+	0.03418803	113,0,4,0	chr1,288253	Cytosol	A > G
chr1:28825396:A:G:UTR3:PHACTR	chr1	28825396	28825396	A	G	UTR3	PHACTR4	ENST000003	.	.	A	G	+	+	0.104	112,0,13,0	chr1,288253	Cytosol	A > G
chr1:28825404:A:G:UTR3:PHACTR	chr1	28825404	28825404	A	G	UTR3	PHACTR4	ENST000003	.	.	A	G	+	+	0.05633803	67,0,4,0	chr1,288254	Cytosol	A > G
chr1:28825418:A:G:UTR3:PHACTR	chr1	28825418	28825418	A	G	UTR3	PHACTR4	ENST000003	.	.	A	G	+	+	0.43835616	41,0,32,0	chr1,288254	Cytosol	A > G
chr1:28825419:A:G:UTR3:PHACTR	chr1	28825419	28825419	A	G	UTR3	PHACTR4	ENST000003	.	.	A	G	+	+	0.31578947	52,0,24,0	chr1,288254	Cytosol	A > G
chr1:28825428:A:G:UTR3:PHACTR	chr1	28825428	28825428	A	G	UTR3	PHACTR4	ENST000003	.	.	A	G	+	+	0.02631579	74,0,2,0	chr1,288254	Cytosol	A > G
chr1:28825433:A:G:UTR3:PHACTR	chr1	28825433	28825433	A	G	UTR3	PHACTR4	ENST000003	.	.	A	G	+	+	0.32857143	47,0,23,0	chr1,288254	Cytosol	A > G
chr1:28825460:A:G:UTR3:PHACTR	chr1	28825460	28825460	A	G	UTR3	PHACTR4	ENST000003	.	.	A	G	+	+	0.0754717	49,0,4,0	chr1,288254	Cytosol	A > G
chr1:28825465:A:G:UTR3:PHACTR	chr1	28825465	28825465	A	G	UTR3	PHACTR4	ENST000003	.	.	A	G	+	+	0.18867925	43,0,10,0	chr1,288254	Cytosol	A > G
chr1:28825466:A:G:UTR3:PHACTR	chr1	28825466	28825466	A	G	UTR3	PHACTR4	ENST000003	.	.	A	G	+	+	0.41509434	31,0,22,0	chr1,288254	Cytosol	A > G
chr1:28825473:A:G:UTR3:PHACTR	chr1	28825473	28825473	A	G	UTR3	PHACTR4	ENST000003	.	.	A	G	+	+	0.30232558	30,0,13,0	chr1,288254	Cytosol	A > G
chr1:28825486:A:G:UTR3:PHACTR	chr1	28825486	28825486	A	G	UTR3	PHACTR4	ENST000003	.	.	A	G	+	+	0.59459459	15,0,22,0	chr1,288254	Cytosol	A > G
chr1:28825505:A:G:UTR3:PHACTR	chr1	28825505	28825505	A	G	UTR3	PHACTR4	ENST000003	.	.	A	G	+	+	0.51219512	20,0,21,0	chr1,288255	Cytosol	A > G
chr1:28825530:A:G:UTR3:PHACTR	chr1	28825530	28825530	A	G	UTR3	PHACTR4	ENST000003	.	.	A	G	+	+	0.31818182	30,0,14,0	chr1,288255	Cytosol	A > G
chr1:28825673:A:G:UTR3:PHACTR	chr1	28825673	28825673	A	G	UTR3	PHACTR4	ENST000003	.	.	A	G	+	+	0.05357143	53,0,3,0	chr1,288256	Cytosol	A > G
chr1:28825747:A:G:UTR3:PHACTR	chr1	28825747	28825747	A	G	UTR3	PHACTR4	ENST000003	.	.	A	G	+	+	0.05555556	68,0,4,0	chr1,288257	Cytosol	A > G

chr1:28825760:A:G:UTR3:PHACTR	chr1	28825760	28825760	A	G	UTR3	PHACTR4	ENST000003	.	.	A	G	+	+	0.66176471	23,0,45,0	chr1,288257	Cytosol	A > G
chr1:28825781:A:G:UTR3:PHACTR	chr1	28825781	28825781	A	G	UTR3	PHACTR4	ENST000003	.	.	A	G	+	+	0.69565217	14,0,32,0	chr1,288257	Cytosol	A > G
chr1:28825787:A:G:UTR3:PHACTR	chr1	28825787	28825787	A	G	UTR3	PHACTR4	ENST000003	.	.	A	G	+	+	0.35135135	24,0,13,0	chr1,288257	Cytosol	A > G
chr1:28825819:A:G:UTR3:PHACTR	chr1	28825819	28825819	A	G	UTR3	PHACTR4	ENST000003	.	.	A	G	+	+	0.66666667	7,0,14,0	chr1,288258	Cytosol	A > G
chr1:28825847:A:G:UTR3:PHACTR	chr1	28825847	28825847	A	G	UTR3	PHACTR4	ENST000003	.	.	A	G	+	+	0.125	21,0,3,0	chr1,288258	Cytosol	A > G
chr1:28826015:A:G:UTR3:PHACTR	chr1	28826015	28826015	A	G	UTR3	PHACTR4	ENST000003	.	.	A	G	+	+	0.14285714	36,0,6,0	chr1,288260	Cytosol	A > G
chr1:28826031:A:G:UTR3:PHACTR	chr1	28826031	28826031	A	G	UTR3	PHACTR4	ENST000003	.	.	A	G	+	+	0.40298507	40,0,27,0	chr1,288260	Cytosol	A > G
chr1:28826053:A:G:UTR3:PHACTR	chr1	28826053	28826053	A	G	UTR3	PHACTR4	ENST000003	.	.	A	G	+	+	0.02222222	88,0,2,0	chr1,288260	Cytosol	A > G
chr1:28826068:A:G:UTR3:PHACTR	chr1	28826068	28826068	A	G	UTR3	PHACTR4	ENST000003	.	.	A	G	+	+	0.06451613	87,0,6,0	chr1,288260	Cytosol	A > G
chr1:28826085:A:G:UTR3:PHACTR	chr1	28826085	28826085	A	G	UTR3	PHACTR4	ENST000003	.	.	A	G	+	+	0.05050505	94,0,5,0	chr1,288260	Cytosol	A > G
chr1:28826095:A:G:UTR3:PHACTR	chr1	28826095	28826095	A	G	UTR3	PHACTR4	ENST000003	.	.	A	G	+	+	0.02222222	88,0,2,0	chr1,288260	Cytosol	A > G
chr1:28826114:A:G:UTR3:PHACTR	chr1	28826114	28826114	A	G	UTR3	PHACTR4	ENST000003	.	.	A	G	+	+	0.08421053	87,0,8,0	chr1,288261	Cytosol	A > G
chr1:28826118:A:G:UTR3:PHACTR	chr1	28826118	28826118	A	G	UTR3	PHACTR4	ENST000003	.	.	A	G	+	+	0.0989011	82,0,9,0	chr1,288261	Cytosol	A > G
chr1:28826128:A:G:UTR3:PHACTR	chr1	28826128	28826128	A	G	UTR3	PHACTR4	ENST000003	.	.	A	G	+	+	0.12359551	78,0,11,0	chr1,288261	Cytosol	A > G
chr1:28826190:A:G:UTR3:PHACTR	chr1	28826190	28826190	A	G	UTR3	PHACTR4	ENST000003	.	.	A	G	+	+	0.49253731	68,0,66,0	chr1,288261	Cytosol	A > G
chr1:28826223:A:G:UTR3:PHACTR	chr1	28826223	28826223	A	G	UTR3	PHACTR4	ENST000003	.	.	A	G	+	+	0.02112676	139,0,3,0	chr1,288262	Cytosol	A > G
chr1:28826250:A:G:UTR3:PHACTR	chr1	28826250	28826250	A	G	UTR3	PHACTR4	ENST000003	.	.	A	G	+	+	0.05714286	132,0,8,0	chr1,288262	Cytosol	A > G
chr1:28836973:A:G:ncRNA_exonic	chr1	28836973	28836973	A	G	ncRNA_exon	SNHG3	.	.	.	A	G	+	+	0.22727273	17,0,5,0	chr1,288369	Cytosol	A > G
chr1:28836974:A:G:ncRNA_exonic	chr1	28836974	28836974	A	G	ncRNA_exon	SNHG3	.	.	.	A	G	+	+	0.22727273	17,0,5,0	chr1,288369	Cytosol	A > G
chr1:28836975:A:G:ncRNA_exonic	chr1	28836975	28836975	A	G	ncRNA_exon	SNHG3	.	.	.	A	G	+	+	0.08695652	21,0,2,0	chr1,288369	Cytosol	A > G
chr1:28865830:A:G:downstream:R	chr1	28865830	28865830	A	G	downstream	RCC1	.	.	.	A	G	+		0.42857143	4,0,3,0	chr1,288658	Cytosol	A > G
chr1:28870206:A:G:intergenic:RCC	chr1	28870206	28870206	A	G	intergenic	RCC1,TRNAU	dist=4394;dis	.	.	A	G	+		0.83333333	1,0,5,0	chr1,288702	Cytosol	A > G
chr1:28870217:A:G:intergenic:RCC	chr1	28870217	28870217	A	G	intergenic	RCC1,TRNAU	dist=4405;dis	.	.	A	G	+		0.71428571	2,0,5,0	chr1,288702	Cytosol	A > G
chr1:29041856:A:G:UTR3:GMEB1	chr1	29041856	29041856	A	G	UTR3	GMEB1	ENST000003	.	.	A	G	+	+	0.25	12,0,4,0	chr1,290418	Cytosol	A > G
chr1:29043841:A:G:UTR3:GMEB1	chr1	29043841	29043841	A	G	UTR3	GMEB1	ENST000003	.	.	A	G	+	+	0.6	2,0,3,0	chr1,290438	Cytosol	A > G
chr1:29519595:T:C:UTR3:MECR	chr1	29519595	29519595	T	C	UTR3	MECR	ENST000003	.	.	A	G	-	-	0.15789474	16,0,3,0	chr1,295195	Cytosol	A > G
chr1:29519634:T:C:UTR3:MECR	chr1	29519634	29519634	T	C	UTR3	MECR	ENST000003	.	.	A	G	-	-	0.14285714	18,0,3,0	chr1,295196	Cytosol	A > G
chr1:29519665:T:C:UTR3:MECR	chr1	29519665	29519665	T	C	UTR3	MECR	ENST000003	.	.	A	G	-	-	0.33333333	16,0,8,0	chr1,295196	Cytosol	A > G
chr1:29519708:T:C:UTR3:MECR	chr1	29519708	29519708	T	C	UTR3	MECR	ENST000003	.	.	A	G	-	-	0.1	18,0,2,0	chr1,295197	Cytosol	A > G
chr1:29519736:T:C:UTR3:MECR	chr1	29519736	29519736	T	C	UTR3	MECR	ENST000003	.	.	A	G	-	-	0.33333333	12,0,6,0	chr1,295197	Cytosol	A > G
chr1:32696890:A:G:UTR3:EIF3I	chr1	32696890	32696890	A	G	UTR3	EIF3I	ENST000003	.	.	A	G	+	+	0.04891304	700,0,36,0	chr1,326968	Cytosol	A > G
chr1:32696891:A:G:UTR3:EIF3I	chr1	32696891	32696891	A	G	UTR3	EIF3I	ENST000003	.	.	A	G	+	+	0.01771117	721,0,13,0	chr1,326968	Cytosol	A > G
chr1:32696949:A:G:UTR3:EIF3I	chr1	32696949	32696949	A	G	UTR3	EIF3I	ENST000003	.	.	A	G	+	+	0.07643312	435,0,36,0	chr1,326969	Cytosol	A > G
chr1:32696981:A:G:UTR3:EIF3I	chr1	32696981	32696981	A	G	UTR3	EIF3I	ENST000003	.	.	A	G	+	+	0.0291616	798,0,24,1	chr1,326969	Cytosol	A > G
chr1:33068776:A:G:UTR3:ZBTB8A	chr1	33068776	33068776	A	G	UTR3	ZBTB8A	ENST000003	.	.	A	G	+	+-	0.22222222	7,0,2,0	chr1,330687	Cytosol	A > G
chr1:33071096:A:G:UTR3:ZBTB8A	chr1	33071096	33071096	A	G	UTR3	ZBTB8A	ENST000003	.	.	A	G	+	+-	0.30434783	16,0,7,0	chr1,330710	Cytosol	A > G
chr1:33071124:A:G:UTR3:ZBTB8A	chr1	33071124	33071124	A	G	UTR3	ZBTB8A	ENST000003	.	.	A	G	+	+-	0.06896552	27,0,2,0	chr1,330711	Cytosol	A > G
chr1:33071141:A:G:UTR3:ZBTB8A	chr1	33071141	33071141	A	G	UTR3	ZBTB8A	ENST000003	.	.	A	G	+	+-	0.18181818	18,0,4,0	chr1,330711	Cytosol	A > G
chr1:33071144:A:G:UTR3:ZBTB8A	chr1	33071144	33071144	A	G	UTR3	ZBTB8A	ENST000003	.	.	A	G	+	+-	0.15	17,0,3,0	chr1,330711	Cytosol	A > G
chr1:33071146:A:G:UTR3:ZBTB8A	chr1	33071146	33071146	A	G	UTR3	ZBTB8A	ENST000003	.	.	A	G	+	+-	0.15789474	16,0,3,0	chr1,330711	Cytosol	A > G
chr1:33071147:A:G:UTR3:ZBTB8A	chr1	33071147	33071147	A	G	UTR3	ZBTB8A	ENST000003	.	.	A	G	+	+-	0.1	18,0,2,0	chr1,330711	Cytosol	A > G
chr1:33071191:A:G:UTR3:ZBTB8A	chr1	33071191	33071191	A	G	UTR3	ZBTB8A	ENST000003	.	.	A	G	+	+-	0.25	9,0,3,0	chr1,330711	Cytosol	A > G
chr1:33086876:T:C:UTR3:ZBTB8OS	chr1	33086876	33086876	T	C	UTR3	ZBTB8OS	ENST000004	.	.	A	G	-	-	0.28571429	15,0,6,0	chr1,330868	Cytosol	A > G
chr1:33148096:A:G:UTR3:RBBP4	chr1	33148096	33148096	A	G	UTR3	RBBP4	ENST000003	.	.	A	G	+	+-	0.33333333	4,0,2,0	chr1,331480	Cytosol	A > G
chr1:33148186:A:G:UTR3:RBBP4	chr1	33148186	33148186	A	G	UTR3	RBBP4	ENST000003	.	.	A	G	+	+-	0.15384615	11,0,2,0	chr1,331481	Cytosol	A > G
chr1:33148196:A:G:UTR3:RBBP4	chr1	33148196	33148196	A	G	UTR3	RBBP4	ENST000003	.	.	A	G	+	+-	0.22222222	7,0,2,0	chr1,331481	Cytosol	A > G
chr1:33148282:A:G:UTR3:RBBP4	chr1	33148282	33148282	A	G	UTR3	RBBP4	ENST000003	.	.	A	G	+	+-	0.36363636	7,0,4,0	chr1,331482	Cytosol	A > G
chr1:33148283:A:G:UTR3:RBBP4	chr1	33148283	33148283	A	G	UTR3	RBBP4	ENST000003	.	.	A	G	+	+-	0.22222222	7,0,2,0	chr1,331482	Cytosol	A > G
chr1:33148284:A:G:UTR3:RBBP4	chr1	33148284	33148284	A	G	UTR3	RBBP4	ENST000003	.	.	A	G	+	+-	0.33333333	6,0,3,0	chr1,331482	Cytosol	A > G
chr1:33148287:A:G:UTR3:RBBP4	chr1	33148287	33148287	A	G	UTR3	RBBP4	ENST000003	.	.	A	G	+	+-	0.55555556	4,0,5,0	chr1,331482	Cytosol	A > G
chr1:33148623:A:G:UTR3:RBBP4	chr1	33148623	33148623	A	G	UTR3	RBBP4	ENST000003	.	.	A	G	+	+-	0.46153846	7,0,6,0	chr1,331486	Cytosol	A > G
chr1:33148691:A:G:UTR3:RBBP4	chr1	33148691	33148691	A	G	UTR3	RBBP4	ENST000003	.	.	A	G	+	+-	0.78571429	3,0,11,0	chr1,331486	Cytosol	A > G
chr1:33148706:A:G:UTR3:RBBP4	chr1	33148706	33148706	A	G	UTR3	RBBP4	ENST000003	.	.	A	G	+	+-	0.33333333	6,0,3,0	chr1,331487	Cytosol	A > G
chr1:33148736:A:G:UTR3:RBBP4	chr1	33148736	33148736	A	G	UTR3	RBBP4	ENST000003	.	.	A	G	+	+-	0.22222222	7,0,2,0	chr1,331487	Cytosol	A > G
chr1:33148789:A:G:UTR3:RBBP4	chr1	33148789	33148789	A	G	UTR3	RBBP4	ENST000003	.	.	A	G	+	+-	0.83333333	1,0,5,0	chr1,331487	Cytosol	A > G
chr1:33149256:A:G:UTR3:RBBP4	chr1	33149256	33149256	A	G	UTR3	RBBP4	ENST000003	.	.	A	G	+	+-	0.18181818	9,0,2,0	chr1,331492	Cytosol	A > G
chr1:33149376:A:G:UTR3:RBBP4	chr1	33149376	33149376	A	G	UTR3	RBBP4	ENST000003	.	.	A	G	+	+-	0.33333333	4,0,2,0	chr1,331493	Cytosol	A > G
chr1:33149443:A:G:UTR3:RBBP4	chr1	33149443	33149443	A	G	UTR3	RBBP4	ENST000003	.	.	A	G	+	+-	0.16666667	10,0,2,0	chr1,331494	Cytosol	A > G
chr1:33149486:A:G:UTR3:RBBP4	chr1	33149486	33149486	A	G	UTR3	RBBP4	ENST000003	.	.	A	G	+	+-	0.28571429	10,0,4,0	chr1,331494	Cytosol	A > G
chr1:33149502:A:G:UTR3:RBBP4	chr1	33149502	33149502	A	G	UTR3	RBBP4	ENST000003	.	.	A	G	+	+-	0.76470588	4,0,13,0	chr1,331495	Cytosol	A > G
chr1:33308260:A:G:intronic:S100P	chr1	33308260	33308260	A	G	intronic	S100PBP	.	.	.	A	G	+	+	0.57142857	3,0,4,0	chr1,333082	Cytosol	A > G
chr1:33308265:A:G:intronic:S100P	chr1	33308265	33308265	A	G	intronic	S100PBP	.	.	.	A	G	+	+	0.6	2,0,3,0	chr1,333082	Cytosol	A > G
chr1:33308296:A:G:intronic:S100P	chr1	33308296	33308296	A	G	intronic	S100PBP	.	.	.	A	G	+	+	0.83333333	1,0,5,0	chr1,333082	Cytosol	A > G
chr1:33308300:A:G:intronic:S100P	chr1	33308300	33308300	A	G	intronic	S100PBP	.	.	.	A	G	+	+	0.4	3,0,2,0	chr1,333082	Cytosol	A > G

chr1:33308321:A:G:intronic:S100P	chr1	33308321	33308321	A	G	intronic	S100PBP	.	.	.	A	G	+	+	0.66666667	2,0,4,0	chr1,333083	Cytosol	A > G
chr1:33308322:A:G:intronic:S100P	chr1	33308322	33308322	A	G	intronic	S100PBP	.	.	.	A	G	+	+	0.4	3,0,2,0	chr1,333083	Cytosol	A > G
chr1:35565653:A:G:intronic:ZMY	chr1	35565653	35565653	A	G	intronic	ZMYM1	.	.	.	A	G	+	+	0.42857143	4,0,3,0	chr1,355656	Cytosol	A > G
chr1:35566803:A:G:intronic:ZMY	chr1	35566803	35566803	A	G	intronic	ZMYM1	.	.	.	A	G	+	+	0.4	3,0,2,0	chr1,355668	Cytosol	A > G
chr1:35582937:A:G:intergenic:ZM	chr1	35582937	35582937	A	G	intergenic	ZMYM1,SFP	dist=1477;dis	.	.	A	G	+		0.92307692	1,0,12,0	chr1,355829	Cytosol	A > G
chr1:35582938:A:G:intergenic:ZM	chr1	35582938	35582938	A	G	intergenic	ZMYM1,SFP	dist=1478;dis	.	.	A	G	+		0.84615385	2,0,11,0	chr1,355829	Cytosol	A > G
chr1:35583572:A:G:intergenic:ZM	chr1	35583572	35583572	A	G	intergenic	ZMYM1,SFP	dist=2112;dis	.	.	A	G	+		0.65	7,0,13,0	chr1,355835	Cytosol	A > G
chr1:35583585:A:G:intergenic:ZM	chr1	35583585	35583585	A	G	intergenic	ZMYM1,SFP	dist=2125;dis	.	.	A	G	+		0.36842105	12,0,7,0	chr1,355835	Cytosol	A > G
chr1:35583592:A:G:intergenic:ZM	chr1	35583592	35583592	A	G	intergenic	ZMYM1,SFP	dist=2132;dis	.	.	A	G	+		0.41176471	10,0,7,0	chr1,355835	Cytosol	A > G
chr1:35583612:A:G:intergenic:ZM	chr1	35583612	35583612	A	G	intergenic	ZMYM1,SFP	dist=2152;dis	.	.	A	G	+		0.33333333	6,0,3,0	chr1,355836	Cytosol	A > G
chr1:35583699:A:G:intergenic:ZM	chr1	35583699	35583699	A	G	intergenic	ZMYM1,SFP	dist=2239;dis	.	.	A	G	+		0.4	3,0,2,0	chr1,355836	Cytosol	A > G
chr1:36065477:T:C:intergenic:TFA	chr1	36065477	36065477	T	C	intergenic	TFAP2E,PSM	dist=4548;dis	.	.	A	G	-	-	0.18571429	57,0,13,0	chr1,360654	Cytosol	A > G
chr1:36065486:T:C:intergenic:TFA	chr1	36065486	36065486	T	C	intergenic	TFAP2E,PSM	dist=4557;dis	.	.	A	G	-	-	0.18518519	44,0,10,0	chr1,360654	Cytosol	A > G
chr1:36065489:T:C:intergenic:TFA	chr1	36065489	36065489	T	C	intergenic	TFAP2E,PSM	dist=4560;dis	.	.	A	G	-	-	0.66666667	18,0,36,0	chr1,360654	Cytosol	A > G
chr1:36065490:T:C:intergenic:TFA	chr1	36065490	36065490	T	C	intergenic	TFAP2E,PSM	dist=4561;dis	.	.	A	G	-	-	0.49056604	27,0,26,0	chr1,360654	Cytosol	A > G
chr1:36065504:T:C:intergenic:TFA	chr1	36065504	36065504	T	C	intergenic	TFAP2E,PSM	dist=4575;dis	.	.	A	G	-	-	0.5106383	23,0,24,0	chr1,360655	Cytosol	A > G
chr1:36065516:T:C:intergenic:TFA	chr1	36065516	36065516	T	C	intergenic	TFAP2E,PSM	dist=4587;dis	.	.	A	G	-	-	0.30434783	32,0,14,0	chr1,360655	Cytosol	A > G
chr1:36065529:T:C:intergenic:TFA	chr1	36065529	36065529	T	C	intergenic	TFAP2E,PSM	dist=4600;dis	.	.	A	G	-	-	0.57446809	20,0,27,0	chr1,360655	Cytosol	A > G
chr1:36065562:T:C:intergenic:TFA	chr1	36065562	36065562	T	C	intergenic	TFAP2E,PSM	dist=4633;dis	.	.	A	G	-	-	0.5	18,0,18,0	chr1,360655	Cytosol	A > G
chr1:36065613:T:C:intergenic:TFA	chr1	36065613	36065613	T	C	intergenic	TFAP2E,PSM	dist=4684;dis	.	.	A	G	-	-	0.06451613	29,0,2,0	chr1,360656	Cytosol	A > G
chr1:36065615:T:C:intergenic:TFA	chr1	36065615	36065615	T	C	intergenic	TFAP2E,PSM	dist=4686;dis	.	.	A	G	-	-	0.16666667	30,0,6,0	chr1,360656	Cytosol	A > G
chr1:36065642:T:C:intergenic:TFA	chr1	36065642	36065642	T	C	intergenic	TFAP2E,PSM	dist=4713;dis	.	.	A	G	-	-	0.11111111	32,0,4,0	chr1,360656	Cytosol	A > G
chr1:36065654:T:C:intergenic:TFA	chr1	36065654	36065654	T	C	intergenic	TFAP2E,PSM	dist=4725;dis	.	.	A	G	-	-	0.16666667	25,0,5,0	chr1,360656	Cytosol	A > G
chr1:36065655:T:C:intergenic:TFA	chr1	36065655	36065655	T	C	intergenic	TFAP2E,PSM	dist=4726;dis	.	.	A	G	-	-	0.25806452	23,0,8,0	chr1,360656	Cytosol	A > G
chr1:36065700:T:C:intergenic:TFA	chr1	36065700	36065700	T	C	intergenic	TFAP2E,PSM	dist=4771;dis	.	.	A	G	-	-	0.25	18,0,6,0	chr1,360656	Cytosol	A > G
chr1:36065708:T:C:intergenic:TFA	chr1	36065708	36065708	T	C	intergenic	TFAP2E,PSM	dist=4779;dis	.	.	A	G	-	-	0.84	4,0,21,0	chr1,360657	Cytosol	A > G
chr1:36065725:T:C:intergenic:TFA	chr1	36065725	36065725	T	C	intergenic	TFAP2E,PSM	dist=4796;dis	.	.	A	G	-	-	0.41935484	18,0,13,0	chr1,360657	Cytosol	A > G
chr1:36065738:T:C:intergenic:TFA	chr1	36065738	36065738	T	C	intergenic	TFAP2E,PSM	dist=4809;dis	.	.	A	G	-	-	0.27272727	16,0,6,0	chr1,360657	Cytosol	A > G
chr1:36066765:T:C:downstream:P	chr1	36066765	36066765	T	C	downstream	PSMB2	.	.	.	A	G	-	-	0.5625	7,0,9,0	chr1,360667	Cytosol	A > G
chr1:36066783:T:C:downstream:P	chr1	36066783	36066783	T	C	downstream	PSMB2	.	.	.	A	G	-	-	0.4	6,0,4,0	chr1,360667	Cytosol	A > G
chr1:36066799:T:C:downstream:P	chr1	36066799	36066799	T	C	downstream	PSMB2	.	.	.	A	G	-	-	0.26666667	11,0,4,0	chr1,360667	Cytosol	A > G
chr1:36066805:T:C:downstream:P	chr1	36066805	36066805	T	C	downstream	PSMB2	.	.	.	A	G	-	-	0.35	13,0,7,0	chr1,360668	Cytosol	A > G
chr1:36066806:T:C:downstream:P	chr1	36066806	36066806	T	C	downstream	PSMB2	.	.	.	A	G	-	-	0.09090909	20,0,2,0	chr1,360668	Cytosol	A > G
chr1:36067677:T:C:UTR3:PSMB2	chr1	36067677	36067677	T	C	UTR3	PSMB2	ENST000003	.	.	A	G	-	-	0.13875598	360,0,58,0	chr1,360676	Cytosol	A > G
chr1:36067714:T:C:UTR3:PSMB2	chr1	36067714	36067714	T	C	UTR3	PSMB2	ENST000003	.	.	A	G	-	-	0.05379747	299,0,17,0	chr1,360677	Cytosol	A > G
chr1:36067728:T:C:UTR3:PSMB2	chr1	36067728	36067728	T	C	UTR3	PSMB2	ENST000003	.	.	A	G	-	-	0.03236246	299,0,10,0	chr1,360677	Cytosol	A > G
chr1:36067749:T:C:UTR3:PSMB2	chr1	36067749	36067749	T	C	UTR3	PSMB2	ENST000003	.	.	A	G	-	-	0.12403101	339,0,48,0	chr1,360677	Cytosol	A > G
chr1:36067752:T:C:UTR3:PSMB2	chr1	36067752	36067752	T	C	UTR3	PSMB2	ENST000003	.	.	A	G	-	-	0.17696629	293,0,63,0	chr1,360677	Cytosol	A > G
chr1:36067760:T:C:UTR3:PSMB2	chr1	36067760	36067760	T	C	UTR3	PSMB2	ENST000003	.	.	A	G	-	-	0.41775457	222,0,160,1	chr1,360677	Cytosol	A > G
chr1:36067766:T:C:UTR3:PSMB2	chr1	36067766	36067766	T	C	UTR3	PSMB2	ENST000003	.	.	A	G	-	-	0.06133333	352,0,23,0	chr1,360677	Cytosol	A > G
chr1:36067784:T:C:UTR3:PSMB2	chr1	36067784	36067784	T	C	UTR3	PSMB2	ENST000003	.	.	A	G	-	-	0.36	208,0,117,0	chr1,360677	Cytosol	A > G
chr1:36067817:T:C:UTR3:PSMB2	chr1	36067817	36067817	T	C	UTR3	PSMB2	ENST000003	.	.	A	G	-	-	0.76779026	62,0,205,0	chr1,360678	Cytosol	A > G
chr1:36067821:T:C:UTR3:PSMB2	chr1	36067821	36067821	T	C	UTR3	PSMB2	ENST000003	.	.	A	G	-	-	0.16666667	210,0,42,0	chr1,360678	Cytosol	A > G
chr1:36067833:T:C:UTR3:PSMB2	chr1	36067833	36067833	T	C	UTR3	PSMB2	ENST000003	.	.	A	G	-	-	0.07228916	231,0,18,0	chr1,360678	Cytosol	A > G
chr1:36067838:T:C:UTR3:PSMB2	chr1	36067838	36067838	T	C	UTR3	PSMB2	ENST000003	.	.	A	G	-	-	0.57818182	116,0,159,0	chr1,360678	Cytosol	A > G
chr1:36067886:T:C:UTR3:PSMB2	chr1	36067886	36067886	T	C	UTR3	PSMB2	ENST000003	.	.	A	G	-	-	0.78969957	49,0,184,0	chr1,360678	Cytosol	A > G
chr1:36067925:T:C:UTR3:PSMB2	chr1	36067925	36067925	T	C	UTR3	PSMB2	ENST000003	.	.	A	G	-	-	0.42424242	133,0,98,0	chr1,360679	Cytosol	A > G
chr1:36068166:T:C:UTR3:PSMB2	chr1	36068166	36068166	T	C	UTR3	PSMB2	ENST000003	.	.	A	G	-	-	0.095	181,0,19,0	chr1,360681	Cytosol	A > G
chr1:36068184:T:C:UTR3:PSMB2	chr1	36068184	36068184	T	C	UTR3	PSMB2	ENST000003	.	.	A	G	-	-	0.58008658	97,0,134,0	chr1,360681	Cytosol	A > G
chr1:36068195:T:C:UTR3:PSMB2	chr1	36068195	36068195	T	C	UTR3	PSMB2	ENST000003	.	.	A	G	-	-	0.05022831	208,0,11,0	chr1,360681	Cytosol	A > G
chr1:36068196:T:C:UTR3:PSMB2	chr1	36068196	36068196	T	C	UTR3	PSMB2	ENST000003	.	.	A	G	-	-	0.14634146	210,0,36,0	chr1,360681	Cytosol	A > G
chr1:36068210:T:C:UTR3:PSMB2	chr1	36068210	36068210	T	C	UTR3	PSMB2	ENST000003	.	.	A	G	-	-	0.47844828	121,0,111,0	chr1,360682	Cytosol	A > G
chr1:36068214:T:C:UTR3:PSMB2	chr1	36068214	36068214	T	C	UTR3	PSMB2	ENST000003	.	.	A	G	-	-	0.01805054	272,0,5,0	chr1,360682	Cytosol	A > G
chr1:36068227:T:C:UTR3:PSMB2	chr1	36068227	36068227	T	C	UTR3	PSMB2	ENST000003	.	.	A	G	-	-	0.06896552	270,0,20,0	chr1,360682	Cytosol	A > G
chr1:36068231:T:C:UTR3:PSMB2	chr1	36068231	36068231	T	C	UTR3	PSMB2	ENST000003	.	.	A	G	-	-	0.53054662	146,0,165,0	chr1,360682	Cytosol	A > G
chr1:36068237:T:C:UTR3:PSMB2	chr1	36068237	36068237	T	C	UTR3	PSMB2	ENST000003	.	.	A	G	-	-	0.25632911	235,0,81,0	chr1,360682	Cytosol	A > G
chr1:36068243:T:C:UTR3:PSMB2	chr1	36068243	36068243	T	C	UTR3	PSMB2	ENST000003	.	.	A	G	-	-	0.09180328	276,1,28,0	chr1,360682	Cytosol	A > G
chr1:36068275:T:C:UTR3:PSMB2	chr1	36068275	36068275	T	C	UTR3	PSMB2	ENST000003	.	.	A	G	-	-	0.20677966	234,0,61,0	chr1,360682	Cytosol	A > G
chr1:36068276:T:C:UTR3:PSMB2	chr1	36068276	36068276	T	C	UTR3	PSMB2	ENST000003	.	.	A	G	-	-	0.33469388	163,0,82,0	chr1,360682	Cytosol	A > G
chr1:36068277:T:C:UTR3:PSMB2	chr1	36068277	36068277	T	C	UTR3	PSMB2	ENST000003	.	.	A	G	-	-	0.06589147	241,0,17,0	chr1,360682	Cytosol	A > G
chr1:36068278:T:C:UTR3:PSMB2	chr1	36068278	36068278	T	C	UTR3	PSMB2	ENST000003	.	.	A	G	-	-	0.21212121	182,0,49,0	chr1,360682	Cytosol	A > G
chr1:36068281:T:C:UTR3:PSMB2	chr1	36068281	36068281	T	C	UTR3	PSMB2	ENST000003	.	.	A	G	-	-	0.22	195,0,55,0	chr1,360682	Cytosol	A > G
chr1:36068294:T:C:UTR3:PSMB2	chr1	36068294	36068294	T	C	UTR3	PSMB2	ENST000003	.	.	A	G	-	-	0.02681992	254,0,7,0	chr1,360682	Cytosol	A > G
chr1:36068295:T:C:UTR3:PSMB2	chr1	36068295	36068295	T	C	UTR3	PSMB2	ENST000003	.	.	A	G	-	-	0.61111111	105,0,165,0	chr1,360682	Cytosol	A > G

chr1:36068325:T:C:UTR3:PSMB2	chr1	36068325	36068325	T	C	UTR3	PSMB2	ENST000003	.	.	A	G	-	-	0.43807339	245,0,191,0	chr1,360683	Cytosol	A > G
chr1:36068364:T:C:UTR3:PSMB2	chr1	36068364	36068364	T	C	UTR3	PSMB2	ENST000003	.	.	A	G	-	-	0.06470588	477,0,33,0	chr1,360683	Cytosol	A > G
chr1:36068369:T:C:UTR3:PSMB2	chr1	36068369	36068369	T	C	UTR3	PSMB2	ENST000003	.	.	A	G	-	-	0.23954373	400,0,126,0	chr1,360683	Cytosol	A > G
chr1:36068370:T:C:UTR3:PSMB2	chr1	36068370	36068370	T	C	UTR3	PSMB2	ENST000003	.	.	A	G	-	-	0.45335821	293,0,243,0	chr1,360683	Cytosol	A > G
chr1:36068376:T:C:UTR3:PSMB2	chr1	36068376	36068376	T	C	UTR3	PSMB2	ENST000003	.	.	A	G	-	-	0.01380671	500,0,7,0	chr1,360683	Cytosol	A > G
chr1:36186342:T:C:UTR3:CLSPN	chr1	36186342	36186342	T	C	UTR3	CLSPN	ENST000002	.	.	A	G	-	-	0.44444444	5,0,4,0	chr1,361863	Cytosol	A > G
chr1:36201759:T:C:intronic:CLSPN	chr1	36201759	36201759	T	C	intronic	CLSPN	.	.	.	A	G	-	-	0.02631579	74,0,2,0	chr1,362017	Cytosol	A > G
chr1:36529419:A:G:UTR3:AGO3	chr1	36529419	36529419	A	G	UTR3	AGO3	ENST000003	.	.	A	G	+	+	0.44444444	5,0,4,0	chr1,365294	Cytosol	A > G
chr1:38326643:T:C:ncRNA_exonic:	chr1	38326643	38326643	T	C	ncRNA_exon	RP11-109P1	.	.	.	A	G	-	+-	0.38709677	19,0,12,0	chr1,383266	Cytosol	A > G
chr1:38326665:T:C:ncRNA_exonic:	chr1	38326665	38326665	T	C	ncRNA_exon	RP11-109P1	.	.	.	A	G	-	+-	0.1	27,0,3,0	chr1,383266	Cytosol	A > G
chr1:38326683:T:C:ncRNA_exonic:	chr1	38326683	38326683	T	C	ncRNA_exon	RP11-109P1	.	.	.	A	G	-	+-	0.19230769	21,0,5,0	chr1,383266	Cytosol	A > G
chr1:38326704:T:C:ncRNA_exonic:	chr1	38326704	38326704	T	C	ncRNA_exon	RP11-109P1	.	.	.	A	G	-	+-	0.16666667	10,0,2,0	chr1,383267	Cytosol	A > G
chr1:38326705:T:C:ncRNA_exonic:	chr1	38326705	38326705	T	C	ncRNA_exon	RP11-109P1	.	.	.	A	G	-	+-	0.13333333	13,0,2,0	chr1,383267	Cytosol	A > G
chr1:38326728:T:C:ncRNA_exonic:	chr1	38326728	38326728	T	C	ncRNA_exon	RP11-109P1	.	.	.	A	G	-	+-	0.44444444	9,1,8,0	chr1,383267	Cytosol	A > G
chr1:38326774:T:C:ncRNA_intronic	chr1	38326774	38326774	T	C	ncRNA_intro	RP11-109P1	.	.	.	A	G	-	+-	0.09090909	20,0,2,0	chr1,383267	Cytosol	A > G
chr1:38327457:T:C:UTR3:INPP5B	chr1	38327457	38327457	T	C	UTR3	INPP5B	ENST000003	.	.	A	G	-	-	0.10526316	17,0,2,0	chr1,383274	Cytosol	A > G
chr1:38327542:T:C:UTR3:INPP5B	chr1	38327542	38327542	T	C	UTR3	INPP5B	ENST000003	.	.	A	G	-	-	0.18181818	9,0,2,0	chr1,383275	Cytosol	A > G
chr1:38327578:T:C:UTR3:INPP5B	chr1	38327578	38327578	T	C	UTR3	INPP5B	ENST000003	.	.	A	G	-	-	0.625	3,0,5,0	chr1,383275	Cytosol	A > G
chr1:38327584:T:C:UTR3:INPP5B	chr1	38327584	38327584	T	C	UTR3	INPP5B	ENST000003	.	.	A	G	-	-	1	0,0,7,0	chr1,383275	Cytosol	A > G
chr1:43242821:A:G:intronic:C1orf	chr1	43242821	43242821	A	G	intronic	C1orf50	.	.	.	A	G	+	+-	0.15384615	11,0,2,0	chr1,432428	Cytosol	A > G
chr1:43242837:A:G:intronic:C1orf	chr1	43242837	43242837	A	G	intronic	C1orf50	.	.	.	A	G	+	+-	0.33333333	6,0,3,0	chr1,432428	Cytosol	A > G
chr1:43242896:A:G:intronic:C1orf	chr1	43242896	43242896	A	G	intronic	C1orf50	.	.	.	A	G	+	+-	0.33333333	4,0,2,0	chr1,432428	Cytosol	A > G
chr1:43243001:A:G:intronic:C1orf	chr1	43243001	43243001	A	G	intronic	C1orf50	.	.	.	A	G	+	+-	0.71428571	2,0,5,0	chr1,432430	Cytosol	A > G
chr1:43245805:A:G:intronic:C1orf	chr1	43245805	43245805	A	G	intronic	C1orf50	.	.	.	A	G	+	+-	0.14285714	12,0,2,0	chr1,432458	Cytosol	A > G
chr1:43245832:A:G:intronic:C1orf	chr1	43245832	43245832	A	G	intronic	C1orf50	.	.	.	A	G	+	+-	0.5625	7,0,9,0	chr1,432458	Cytosol	A > G
chr1:43872966:A:G:intronic:SZT2	chr1	43872966	43872966	A	G	intronic	SZT2	.	.	.	A	G	+	+	0.6	2,0,3,0	chr1,438729	Cytosol	A > G
chr1:43873011:A:G:intronic:SZT2	chr1	43873011	43873011	A	G	intronic	SZT2	.	.	.	A	G	+	+	0.6	2,0,3,0	chr1,438730	Cytosol	A > G
chr1:43875995:A:G:intronic:SZT2	chr1	43875995	43875995	A	G	intronic	SZT2	.	.	.	A	G	+	+	0.6	2,0,3,0	chr1,438759	Cytosol	A > G
chr1:43878066:A:G:UTR3:SZT2	chr1	43878066	43878066	A	G	UTR3	SZT2	ENST000004	.	.	A	G	+	+	0.25	6,0,2,0	chr1,438780	Cytosol	A > G
chr1:43878106:A:G:UTR3:SZT2	chr1	43878106	43878106	A	G	UTR3	SZT2	ENST000004	.	.	A	G	+	+	0.18181818	9,0,2,0	chr1,438781	Cytosol	A > G
chr1:43878130:A:G:UTR3:SZT2	chr1	43878130	43878130	A	G	UTR3	SZT2	ENST000004	.	.	A	G	+	+	0.21428571	11,0,3,0	chr1,438781	Cytosol	A > G
chr1:43878131:A:G:UTR3:SZT2	chr1	43878131	43878131	A	G	UTR3	SZT2	ENST000004	.	.	A	G	+	+	0.23076923	10,0,3,0	chr1,438781	Cytosol	A > G
chr1:43878167:A:G:UTR3:SZT2	chr1	43878167	43878167	A	G	UTR3	SZT2	ENST000004	.	.	A	G	+	+	0.22222222	7,0,2,0	chr1,438781	Cytosol	A > G
chr1:44451814:A:G:intronic:B4GA	chr1	44451814	44451814	A	G	intronic	B4GALT2	.	.	.	A	G	+	+	0.61904762	8,0,13,0	chr1,444518	Cytosol	A > G
chr1:44451857:A:G:intronic:B4GA	chr1	44451857	44451857	A	G	intronic	B4GALT2	.	.	.	A	G	+	+	0.83333333	1,0,5,0	chr1,444518	Cytosol	A > G
chr1:44451858:A:G:intronic:B4GA	chr1	44451858	44451858	A	G	intronic	B4GALT2	.	.	.	A	G	+	+	0.8	1,0,4,0	chr1,444518	Cytosol	A > G
chr1:44451862:A:G:intronic:B4GA	chr1	44451862	44451862	A	G	intronic	B4GALT2	.	.	.	A	G	+	+	1	0,0,6,0	chr1,444518	Cytosol	A > G
chr1:44451875:A:G:intronic:B4GA	chr1	44451875	44451875	A	G	intronic	B4GALT2	.	.	.	A	G	+	+	0.27272727	8,0,3,0	chr1,444518	Cytosol	A > G
chr1:44451893:A:G:intronic:B4GA	chr1	44451893	44451893	A	G	intronic	B4GALT2	.	.	.	A	G	+	+	0.46153846	7,0,6,0	chr1,444518	Cytosol	A > G
chr1:44451894:A:G:intronic:B4GA	chr1	44451894	44451894	A	G	intronic	B4GALT2	.	.	.	A	G	+	+	0.76923077	3,0,10,0	chr1,444518	Cytosol	A > G
chr1:44452987:A:G:intronic:B4GA	chr1	44452987	44452987	A	G	intronic	B4GALT2	.	.	.	A	G	+	+	0.66666667	5,0,10,0	chr1,444529	Cytosol	A > G
chr1:44453016:A:G:intronic:B4GA	chr1	44453016	44453016	A	G	intronic	B4GALT2	.	.	.	A	G	+	+	0.92857143	1,0,13,0	chr1,444530	Cytosol	A > G
chr1:44453054:A:G:intronic:B4GA	chr1	44453054	44453054	A	G	intronic	B4GALT2	.	.	.	A	G	+	+	0.88888889	1,0,8,0	chr1,444530	Cytosol	A > G
chr1:44453061:A:G:intronic:B4GA	chr1	44453061	44453061	A	G	intronic	B4GALT2	.	.	.	A	G	+	+	0.5	4,0,4,0	chr1,444530	Cytosol	A > G
chr1:44453072:A:G:intronic:B4GA	chr1	44453072	44453072	A	G	intronic	B4GALT2	.	.	.	A	G	+	+	0.875	1,0,7,0	chr1,444530	Cytosol	A > G
chr1:45243017:A:G:intronic:RPS8	chr1	45243017	45243017	A	G	intronic	RPS8	.	.	.	A	G	+	+	0.16666667	10,0,2,0	chr1,452430	Cytosol	A > G
chr1:45975125:A:G:UTR3:MMACH	chr1	45975125	45975125	A	G	UTR3	MMACHC	ENST000004	.	.	A	G	+	+	0.10714286	25,0,3,0	chr1,459751	Cytosol	A > G
chr1:45975186:A:G:UTR3:MMACH	chr1	45975186	45975186	A	G	UTR3	MMACHC	ENST000004	.	.	A	G	+	+	0.08571429	32,0,3,0	chr1,459751	Cytosol	A > G
chr1:45975250:A:G:UTR3:MMACH	chr1	45975250	45975250	A	G	UTR3	MMACHC	ENST000004	.	.	A	G	+	+	0.5	7,0,7,0	chr1,459752	Cytosol	A > G
chr1:45975345:A:G:UTR3:MMACH	chr1	45975345	45975345	A	G	UTR3	MMACHC	ENST000004	.	.	A	G	+	+	0.15	51,0,9,0	chr1,459753	Cytosol	A > G
chr1:45975346:A:G:UTR3:MMACH	chr1	45975346	45975346	A	G	UTR3	MMACHC	ENST000004	.	.	A	G	+	+	0.08474576	54,0,5,0	chr1,459753	Cytosol	A > G
chr1:45975358:A:G:UTR3:MMACH	chr1	45975358	45975358	A	G	UTR3	MMACHC	ENST000004	.	.	A	G	+	+	0.12195122	72,0,10,0	chr1,459753	Cytosol	A > G
chr1:45975498:A:G:UTR3:MMACH	chr1	45975498	45975498	A	G	UTR3	MMACHC	ENST000004	.	.	A	G	+	+	0.18269231	85,0,19,0	chr1,459754	Cytosol	A > G
chr1:45975499:A:G:UTR3:MMACH	chr1	45975499	45975499	A	G	UTR3	MMACHC	ENST000004	.	.	A	G	+	+	0.06122449	92,0,6,0	chr1,459754	Cytosol	A > G
chr1:45975628:A:G:UTR3:MMACH	chr1	45975628	45975628	A	G	UTR3	MMACHC	ENST000004	.	.	A	G	+	+	0.38823529	52,0,33,0	chr1,459756	Cytosol	A > G
chr1:45975669:A:G:UTR3:MMACH	chr1	45975669	45975669	A	G	UTR3	MMACHC	ENST000004	.	.	A	G	+	+	0.375	30,0,18,0	chr1,459756	Cytosol	A > G
chr1:45975670:A:G:UTR3:MMACH	chr1	45975670	45975670	A	G	UTR3	MMACHC	ENST000004	.	.	A	G	+	+	0.65306122	17,0,32,0	chr1,459756	Cytosol	A > G
chr1:45975673:A:G:UTR3:MMACH	chr1	45975673	45975673	A	G	UTR3	MMACHC	ENST000004	.	.	A	G	+	+	0.37209302	27,0,16,0	chr1,459756	Cytosol	A > G
chr1:45975757:A:G:UTR3:MMACH	chr1	45975757	45975757	A	G	UTR3	MMACHC	ENST000004	.	.	A	G	+	+	0.02362205	124,0,3,0	chr1,459757	Cytosol	A > G
chr1:45977838:A:G:intronic:PRDX	chr1	45977838	45977838	A	G	intronic	PRDX1	.	.	.	A	G	+	-	0.42857143	4,0,3,0	chr1,459778	Cytosol	A > G
chr1:46160600:T:C:ncRNA_exonic:	chr1	46160600	46160600	T	C	ncRNA_exon	RP11-767N6.	.	.	.	A	G	-	-	0.71428571	2,0,5,0	chr1,461605	Cytosol	A > G
chr1:46160669:T:C:ncRNA_intron	chr1	46160669	46160669	T	C	ncRNA_intro	RP11-767N6.	.	.	.	A	G	-	-	0.22222222	7,0,2,0	chr1,461606	Cytosol	A > G
chr1:46160724:T:C:ncRNA_intron	chr1	46160724	46160724	T	C	ncRNA_intro	RP11-767N6.	.	.	.	A	G	-	-	0.28571429	10,0,4,0	chr1,461607	Cytosol	A > G
chr1:46160891:T:C:ncRNA_intron	chr1	46160891	46160891	T	C	ncRNA_intro	RP11-767N6.	.	.	.	A	G	-	-	0.31578947	13,0,6,0	chr1,461608	Cytosol	A > G

chr1:46160894:T:C:ncRNA_introni	chr1	46160894	46160894	T	C	ncRNA_intro	RP11-767N6.	.	.	.	A	G	-	-	0.0952381	19,0,2,0	chr1,461608	Cytosol	A > G
chr1:46160913:T:C:ncRNA_introni	chr1	46160913	46160913	T	C	ncRNA_intro	RP11-767N6.	.	.	.	A	G	-	-	0.22222222	14,0,4,0	chr1,461609	Cytosol	A > G
chr1:46160929:T:C:ncRNA_introni	chr1	46160929	46160929	T	C	ncRNA_intro	RP11-767N6.	.	.	.	A	G	-	-	0.5	7,0,7,0	chr1,461609	Cytosol	A > G
chr1:46160961:T:C:ncRNA_introni	chr1	46160961	46160961	T	C	ncRNA_intro	RP11-767N6.	.	.	.	A	G	-	-	0.66666667	3,0,6,0	chr1,461609	Cytosol	A > G
chr1:46160975:T:C:ncRNA_introni	chr1	46160975	46160975	T	C	ncRNA_intro	RP11-767N6.	.	.	.	A	G	-	-	0.28571429	10,0,4,0	chr1,461609	Cytosol	A > G
chr1:46160981:T:C:ncRNA_introni	chr1	46160981	46160981	T	C	ncRNA_intro	RP11-767N6.	.	.	.	A	G	-	-	0.16666667	10,0,2,0	chr1,461609	Cytosol	A > G
chr1:46160991:T:C:ncRNA_introni	chr1	46160991	46160991	T	C	ncRNA_intro	RP11-767N6.	.	.	.	A	G	-	-	0.125	14,0,2,0	chr1,461609	Cytosol	A > G
chr1:46161036:T:C:ncRNA_introni	chr1	46161036	46161036	T	C	ncRNA_intro	RP11-767N6.	.	.	.	A	G	-	-	0.38888889	11,0,7,0	chr1,461610	Cytosol	A > G
chr1:46161056:T:C:ncRNA_introni	chr1	46161056	46161056	T	C	ncRNA_intro	RP11-767N6.	.	.	.	A	G	-	-	0.35	13,0,7,0	chr1,461610	Cytosol	A > G
chr1:46161099:T:C:ncRNA_introni	chr1	46161099	46161099	T	C	ncRNA_intro	RP11-767N6.	.	.	.	A	G	-	-	0.5	9,0,9,0	chr1,461610	Cytosol	A > G
chr1:46161535:T:C:ncRNA_introni	chr1	46161535	46161535	T	C	ncRNA_intro	RP11-767N6.	.	.	.	A	G	-	-	0.6	2,0,3,0	chr1,461615	Cytosol	A > G
chr1:46161554:T:C:ncRNA_introni	chr1	46161554	46161554	T	C	ncRNA_intro	RP11-767N6.	.	.	.	A	G	-	-	0.75	2,0,6,0	chr1,461615	Cytosol	A > G
chr1:46161572:T:C:ncRNA_introni	chr1	46161572	46161572	T	C	ncRNA_intro	RP11-767N6.	.	.	.	A	G	-	-	0.72727273	3,0,8,0	chr1,461615	Cytosol	A > G
chr1:46161613:T:C:ncRNA_introni	chr1	46161613	46161613	T	C	ncRNA_intro	RP11-767N6.	.	.	.	A	G	-	-	0.33333333	6,0,3,0	chr1,461616	Cytosol	A > G
chr1:46162208:T:C:ncRNA_introni	chr1	46162208	46162208	T	C	ncRNA_intro	RP11-767N6.	.	.	.	A	G	-	-	1	0,0,6,0	chr1,461622	Cytosol	A > G
chr1:46162209:T:C:ncRNA_introni	chr1	46162209	46162209	T	C	ncRNA_intro	RP11-767N6.	.	.	.	A	G	-	-	0.42857143	4,0,3,0	chr1,461622	Cytosol	A > G
chr1:46162236:T:C:ncRNA_introni	chr1	46162236	46162236	T	C	ncRNA_intro	RP11-767N6.	.	.	.	A	G	-	-	0.46666667	8,0,7,0	chr1,461622	Cytosol	A > G
chr1:46162287:T:C:ncRNA_introni	chr1	46162287	46162287	T	C	ncRNA_intro	RP11-767N6.	.	.	.	A	G	-	-	0.64285714	5,0,9,0	chr1,461622	Cytosol	A > G
chr1:46162296:T:C:ncRNA_introni	chr1	46162296	46162296	T	C	ncRNA_intro	RP11-767N6.	.	.	.	A	G	-	-	0.15384615	11,0,2,0	chr1,461622	Cytosol	A > G
chr1:46162369:T:C:ncRNA_introni	chr1	46162369	46162369	T	C	ncRNA_intro	RP11-767N6.	.	.	.	A	G	-	-	0.66666667	4,0,8,0	chr1,461623	Cytosol	A > G
chr1:46162370:T:C:ncRNA_introni	chr1	46162370	46162370	T	C	ncRNA_intro	RP11-767N6.	.	.	.	A	G	-	-	0.46153846	7,0,6,0	chr1,461623	Cytosol	A > G
chr1:46162408:T:C:ncRNA_introni	chr1	46162408	46162408	T	C	ncRNA_intro	RP11-767N6.	.	.	.	A	G	-	-	0.18181818	9,0,2,0	chr1,461624	Cytosol	A > G
chr1:46162411:T:C:ncRNA_introni	chr1	46162411	46162411	T	C	ncRNA_intro	RP11-767N6.	.	.	.	A	G	-	-	1	0,0,12,0	chr1,461624	Cytosol	A > G
chr1:46162413:T:C:ncRNA_introni	chr1	46162413	46162413	T	C	ncRNA_intro	RP11-767N6.	.	.	.	A	G	-	-	0.66666667	4,0,8,0	chr1,461624	Cytosol	A > G
chr1:46162983:T:C:intronic:IPP	chr1	46162983	46162983	T	C	intronic	IPP	.	.	.	A	G	-	-	0.21428571	11,0,3,0	chr1,461629	Cytosol	A > G
chr1:46163015:T:C:intronic:IPP	chr1	46163015	46163015	T	C	intronic	IPP	.	.	.	A	G	-	-	0.45833333	13,0,11,0	chr1,461630	Cytosol	A > G
chr1:46163019:T:C:intronic:IPP	chr1	46163019	46163019	T	C	intronic	IPP	.	.	.	A	G	-	-	0.24	19,0,6,0	chr1,461630	Cytosol	A > G
chr1:46163025:T:C:intronic:IPP	chr1	46163025	46163025	T	C	intronic	IPP	.	.	.	A	G	-	-	0.41666667	14,0,10,0	chr1,461630	Cytosol	A > G
chr1:46163034:T:C:intronic:IPP	chr1	46163034	46163034	T	C	intronic	IPP	.	.	.	A	G	-	-	0.55555556	8,0,10,0	chr1,461630	Cytosol	A > G
chr1:46163047:T:C:intronic:IPP	chr1	46163047	46163047	T	C	intronic	IPP	.	.	.	A	G	-	-	0.60869565	9,0,14,0	chr1,461630	Cytosol	A > G
chr1:46163060:T:C:intronic:IPP	chr1	46163060	46163060	T	C	intronic	IPP	.	.	.	A	G	-	-	0.7	6,0,14,0	chr1,461630	Cytosol	A > G
chr1:46163061:T:C:intronic:IPP	chr1	46163061	46163061	T	C	intronic	IPP	.	.	.	A	G	-	-	0.31818182	15,0,7,0	chr1,461630	Cytosol	A > G
chr1:46163142:T:C:intronic:IPP	chr1	46163142	46163142	T	C	intronic	IPP	.	.	.	A	G	-	-	0.46153846	7,0,6,0	chr1,461631	Cytosol	A > G
chr1:46163143:T:C:intronic:IPP	chr1	46163143	46163143	T	C	intronic	IPP	.	.	.	A	G	-	-	0.81818182	2,0,9,0	chr1,461631	Cytosol	A > G
chr1:46163166:T:C:intronic:IPP	chr1	46163166	46163166	T	C	intronic	IPP	.	.	.	A	G	-	-	0.36363636	7,0,4,0	chr1,461631	Cytosol	A > G
chr1:46163196:T:C:intronic:IPP	chr1	46163196	46163196	T	C	intronic	IPP	.	.	.	A	G	-	-	0.51851852	13,0,14,0	chr1,461631	Cytosol	A > G
chr1:46163386:T:C:intronic:IPP	chr1	46163386	46163386	T	C	intronic	IPP	.	.	.	A	G	-	-	0.28571429	10,0,4,0	chr1,461633	Cytosol	A > G
chr1:46163387:T:C:intronic:IPP	chr1	46163387	46163387	T	C	intronic	IPP	.	.	.	A	G	-	-	0.21428571	11,0,3,0	chr1,461633	Cytosol	A > G
chr1:46163446:T:C:intronic:IPP	chr1	46163446	46163446	T	C	intronic	IPP	.	.	.	A	G	-	-	0.0952381	19,0,2,0	chr1,461634	Cytosol	A > G
chr1:46163463:T:C:intronic:IPP	chr1	46163463	46163463	T	C	intronic	IPP	.	.	.	A	G	-	-	0.38888889	11,0,7,0	chr1,461634	Cytosol	A > G
chr1:46163466:T:C:intronic:IPP	chr1	46163466	46163466	T	C	intronic	IPP	.	.	.	A	G	-	-	0.13636364	19,0,3,0	chr1,461634	Cytosol	A > G
chr1:46163481:T:C:intronic:IPP	chr1	46163481	46163481	T	C	intronic	IPP	.	.	.	A	G	-	-	0.08333333	22,0,2,0	chr1,461634	Cytosol	A > G
chr1:46163508:T:C:intronic:IPP	chr1	46163508	46163508	T	C	intronic	IPP	.	.	.	A	G	-	-	0.16666667	20,0,4,0	chr1,461635	Cytosol	A > G
chr1:46163547:T:C:intronic:IPP	chr1	46163547	46163547	T	C	intronic	IPP	.	.	.	A	G	-	-	0.33333333	20,0,10,0	chr1,461635	Cytosol	A > G
chr1:46163578:T:C:intronic:IPP	chr1	46163578	46163578	T	C	intronic	IPP	.	.	.	A	G	-	-	0.08333333	22,0,2,0	chr1,461635	Cytosol	A > G
chr1:46163591:T:C:intronic:IPP	chr1	46163591	46163591	T	C	intronic	IPP	.	.	.	A	G	-	-	0.82352941	3,0,14,0	chr1,461635	Cytosol	A > G
chr1:46163712:T:C:intronic:IPP	chr1	46163712	46163712	T	C	intronic	IPP	.	.	.	A	G	-	-	0.10526316	17,0,2,0	chr1,461637	Cytosol	A > G
chr1:46163864:T:C:intronic:IPP	chr1	46163864	46163864	T	C	intronic	IPP	.	.	.	A	G	-	-	0.42857143	4,0,3,0	chr1,461638	Cytosol	A > G
chr1:46163881:T:C:intronic:IPP	chr1	46163881	46163881	T	C	intronic	IPP	.	.	.	A	G	-	-	0.5	3,0,3,0	chr1,461638	Cytosol	A > G
chr1:46163925:T:C:intronic:IPP	chr1	46163925	46163925	T	C	intronic	IPP	.	.	.	A	G	-	-	0.3	7,0,3,0	chr1,461639	Cytosol	A > G
chr1:46163963:T:C:intronic:IPP	chr1	46163963	46163963	T	C	intronic	IPP	.	.	.	A	G	-	-	0.09090909	20,0,2,0	chr1,461639	Cytosol	A > G
chr1:46164459:T:C:UTR3:IPP	chr1	46164459	46164459	T	C	UTR3	IPP	ENST000003	.	.	A	G	-	-	0.13333333	13,0,2,0	chr1,461644	Cytosol	A > G
chr1:46164483:T:C:UTR3:IPP	chr1	46164483	46164483	T	C	UTR3	IPP	ENST000003	.	.	A	G	-	-	0.4	9,0,6,0	chr1,461644	Cytosol	A > G
chr1:46164555:T:C:UTR3:IPP	chr1	46164555	46164555	T	C	UTR3	IPP	ENST000003	.	.	A	G	-	-	0.15789474	16,0,3,0	chr1,461645	Cytosol	A > G
chr1:46164671:T:C:UTR3:IPP	chr1	46164671	46164671	T	C	UTR3	IPP	ENST000003	.	.	A	G	-	-	0.07142857	26,0,2,0	chr1,461646	Cytosol	A > G
chr1:46164672:T:C:UTR3:IPP	chr1	46164672	46164672	T	C	UTR3	IPP	ENST000003	.	.	A	G	-	-	0.11111111	24,0,3,0	chr1,461646	Cytosol	A > G
chr1:46164678:T:C:UTR3:IPP	chr1	46164678	46164678	T	C	UTR3	IPP	ENST000003	.	.	A	G	-	-	0.26923077	19,0,7,0	chr1,461646	Cytosol	A > G
chr1:46165408:T:C:UTR3:IPP	chr1	46165408	46165408	T	C	UTR3	IPP	ENST000003	.	.	A	G	-	-	0.10714286	25,0,3,0	chr1,461654	Cytosol	A > G
chr1:46165437:T:C:UTR3:IPP	chr1	46165437	46165437	T	C	UTR3	IPP	ENST000003	.	.	A	G	-	-	0.09090909	20,0,2,0	chr1,461654	Cytosol	A > G
chr1:46830294:A:G:UTR3:NSUN4	chr1	46830294	46830294	A	G	UTR3	NSUN4	ENST000003	.	.	A	G	+	+	0.06818182	41,0,3,0	chr1,468302	Cytosol	A > G
chr1:52816503:T:C:UTR3:CC2D1B	chr1	52816503	52816503	T	C	UTR3	CC2D1B	ENST000003	.	.	A	G	-	-	0.16666667	15,0,3,0	chr1,528165	Cytosol	A > G
chr1:52816510:T:C:UTR3:CC2D1B	chr1	52816510	52816510	T	C	UTR3	CC2D1B	ENST000003	.	.	A	G	-	-	0.27777778	13,0,5,0	chr1,528165	Cytosol	A > G
chr1:52816555:T:C:UTR3:CC2D1B	chr1	52816555	52816555	T	C	UTR3	CC2D1B	ENST000003	.	.	A	G	-	-	0.22222222	14,0,4,0	chr1,528165	Cytosol	A > G
chr1:52816556:T:C:UTR3:CC2D1B	chr1	52816556	52816556	T	C	UTR3	CC2D1B	ENST000003	.	.	A	G	-	-	0.11111111	16,0,2,0	chr1,528165	Cytosol	A > G

chr1:52816582:T:C:UTR3:CC2D1B	chr1	52816582	52816582	T	C	UTR3	CC2D1B	ENST000003	.	.	A	G	-	-	0.28571429	10,0,4,0	chr1,528165	Cytosol	A > G
chr1:52816591:T:C:UTR3:CC2D1B	chr1	52816591	52816591	T	C	UTR3	CC2D1B	ENST000003	.	.	A	G	-	-	0.27272727	8,0,3,0	chr1,528165	Cytosol	A > G
chr1:52816622:T:C:UTR3:CC2D1B	chr1	52816622	52816622	T	C	UTR3	CC2D1B	ENST000003	.	.	A	G	-	-	0.66666667	3,0,6,0	chr1,528166	Cytosol	A > G
chr1:52816638:T:C:UTR3:CC2D1B	chr1	52816638	52816638	T	C	UTR3	CC2D1B	ENST000003	.	.	A	G	-	-	0.25	6,0,2,0	chr1,528166	Cytosol	A > G
chr1:52816642:T:C:UTR3:CC2D1B	chr1	52816642	52816642	T	C	UTR3	CC2D1B	ENST000003	.	.	A	G	-	-	0.5	3,0,3,0	chr1,528166	Cytosol	A > G
chr1:52816674:T:C:UTR3:CC2D1B	chr1	52816674	52816674	T	C	UTR3	CC2D1B	ENST000003	.	.	A	G	-	-	0.71428571	2,0,5,0	chr1,528166	Cytosol	A > G
chr1:52817390:T:C:UTR3:CC2D1B	chr1	52817390	52817390	T	C	UTR3	CC2D1B	ENST000003	.	.	A	G	-	-	0.85714286	1,0,6,0	chr1,528173	Cytosol	A > G
chr1:52817393:T:C:UTR3:CC2D1B	chr1	52817393	52817393	T	C	UTR3	CC2D1B	ENST000003	.	.	A	G	-	-	0.5	3,0,3,0	chr1,528173	Cytosol	A > G
chr1:52817399:T:C:UTR3:CC2D1B	chr1	52817399	52817399	T	C	UTR3	CC2D1B	ENST000003	.	.	A	G	-	-	0.6	2,0,3,0	chr1,528173	Cytosol	A > G
chr1:52817419:A:G:UTR3:CC2D1B	chr1	52817419	52817419	A	G	UTR3	CC2D1B	ENST000003	.	.	A	G	+	-	0.8	1,0,4,0	chr1,528174	Cytosol	A > G
chr1:52817428:T:C:UTR3:CC2D1B	chr1	52817428	52817428	T	C	UTR3	CC2D1B	ENST000003	.	.	A	G	-	-	0.71428571	2,0,5,0	chr1,528174	Cytosol	A > G
chr1:52817434:T:C:UTR3:CC2D1B	chr1	52817434	52817434	T	C	UTR3	CC2D1B	ENST000003	.	.	A	G	-	-	0.6	2,0,3,0	chr1,528174	Cytosol	A > G
chr1:52817436:A:G:UTR3:CC2D1B	chr1	52817436	52817436	A	G	UTR3	CC2D1B	ENST000003	.	.	A	G	+	-	0.33333333	4,0,2,0	chr1,528174	Cytosol	A > G
chr1:52817473:T:C:UTR3:CC2D1B	chr1	52817473	52817473	T	C	UTR3	CC2D1B	ENST000003	.	.	A	G	-	-	0.33333333	8,0,4,0	chr1,528174	Cytosol	A > G
chr1:52817480:T:C:UTR3:CC2D1B	chr1	52817480	52817480	T	C	UTR3	CC2D1B	ENST000003	.	.	A	G	-	-	0.23076923	10,0,3,0	chr1,528174	Cytosol	A > G
chr1:52817493:T:C:UTR3:CC2D1B	chr1	52817493	52817493	T	C	UTR3	CC2D1B	ENST000003	.	.	A	G	-	-	0.2	8,0,2,0	chr1,528174	Cytosol	A > G
chr1:52838676:T:C:UTR3:ORC1	chr1	52838676	52838676	T	C	UTR3	ORC1	ENST000003	.	.	A	G	-	-	0.03610108	267,0,10,0	chr1,528386	Cytosol	A > G
chr1:52838682:T:C:UTR3:ORC1	chr1	52838682	52838682	T	C	UTR3	ORC1	ENST000003	.	.	A	G	-	-	0.01805054	272,0,5,0	chr1,528386	Cytosol	A > G
chr1:52838696:T:C:UTR3:ORC1	chr1	52838696	52838696	T	C	UTR3	ORC1	ENST000003	.	.	A	G	-	-	0.04100946	304,0,13,0	chr1,528386	Cytosol	A > G
chr1:52838760:T:C:UTR3:ORC1	chr1	52838760	52838760	T	C	UTR3	ORC1	ENST000003	.	.	A	G	-	-	0.01149425	172,0,2,0	chr1,528387	Cytosol	A > G
chr1:52838798:T:C:UTR3:ORC1	chr1	52838798	52838798	T	C	UTR3	ORC1	ENST000003	.	.	A	G	-	-	0.11445783	147,0,19,0	chr1,528387	Cytosol	A > G
chr1:53288862:A:G:UTR3:ZYG11B	chr1	53288862	53288862	A	G	UTR3	ZYG11B	ENST000002	.	.	A	G	+	+	0.2	36,0,9,0	chr1,532888	Cytosol	A > G
chr1:53288870:A:G:UTR3:ZYG11B	chr1	53288870	53288870	A	G	UTR3	ZYG11B	ENST000002	.	.	A	G	+	+	0.6	20,0,30,0	chr1,532888	Cytosol	A > G
chr1:53288894:A:G:UTR3:ZYG11B	chr1	53288894	53288894	A	G	UTR3	ZYG11B	ENST000002	.	.	A	G	+	+	0.18181818	36,0,8,0	chr1,532888	Cytosol	A > G
chr1:53288897:A:G:UTR3:ZYG11B	chr1	53288897	53288897	A	G	UTR3	ZYG11B	ENST000002	.	.	A	G	+	+	0.54285714	16,0,19,0	chr1,532888	Cytosol	A > G
chr1:53288916:A:G:UTR3:ZYG11B	chr1	53288916	53288916	A	G	UTR3	ZYG11B	ENST000002	.	.	A	G	+	+	0.09756098	37,0,4,0	chr1,532889	Cytosol	A > G
chr1:53288924:A:G:UTR3:ZYG11B	chr1	53288924	53288924	A	G	UTR3	ZYG11B	ENST000002	.	.	A	G	+	+	0.71428571	12,0,30,0	chr1,532889	Cytosol	A > G
chr1:53288925:A:G:UTR3:ZYG11B	chr1	53288925	53288925	A	G	UTR3	ZYG11B	ENST000002	.	.	A	G	+	+	0.20454545	35,0,9,0	chr1,532889	Cytosol	A > G
chr1:53289547:A:G:UTR3:ZYG11B	chr1	53289547	53289547	A	G	UTR3	ZYG11B	ENST000002	.	.	A	G	+	+	0.41772152	46,0,33,0	chr1,532895	Cytosol	A > G
chr1:53289570:A:G:UTR3:ZYG11B	chr1	53289570	53289570	A	G	UTR3	ZYG11B	ENST000002	.	.	A	G	+	+	0.05882353	64,0,4,0	chr1,532895	Cytosol	A > G
chr1:53289686:A:G:UTR3:ZYG11B	chr1	53289686	53289686	A	G	UTR3	ZYG11B	ENST000002	.	.	A	G	+	+	0.4875	41,0,39,0	chr1,532896	Cytosol	A > G
chr1:53289688:A:G:UTR3:ZYG11B	chr1	53289688	53289688	A	G	UTR3	ZYG11B	ENST000002	.	.	A	G	+	+	0.18666667	61,0,14,0	chr1,532896	Cytosol	A > G
chr1:53289759:A:G:UTR3:ZYG11B	chr1	53289759	53289759	A	G	UTR3	ZYG11B	ENST000002	.	.	A	G	+	+	0.36170213	30,0,17,0	chr1,532897	Cytosol	A > G
chr1:53289852:A:G:UTR3:ZYG11B	chr1	53289852	53289852	A	G	UTR3	ZYG11B	ENST000002	.	.	A	G	+	+	0.27777778	26,0,10,0	chr1,532898	Cytosol	A > G
chr1:53289873:A:G:UTR3:ZYG11B	chr1	53289873	53289873	A	G	UTR3	ZYG11B	ENST000002	.	.	A	G	+	+	0.1369863	63,0,10,0	chr1,532898	Cytosol	A > G
chr1:53289892:A:G:UTR3:ZYG11B	chr1	53289892	53289892	A	G	UTR3	ZYG11B	ENST000002	.	.	A	G	+	+	0.30769231	54,0,24,0	chr1,532898	Cytosol	A > G
chr1:53289940:A:G:UTR3:ZYG11B	chr1	53289940	53289940	A	G	UTR3	ZYG11B	ENST000002	.	.	A	G	+	+	0.13559322	51,0,8,0	chr1,532899	Cytosol	A > G
chr1:53290190:A:G:UTR3:ZYG11B	chr1	53290190	53290190	A	G	UTR3	ZYG11B	ENST000002	.	.	A	G	+	+	0.17460317	52,0,11,0	chr1,532901	Cytosol	A > G
chr1:53290266:A:G:UTR3:ZYG11B	chr1	53290266	53290266	A	G	UTR3	ZYG11B	ENST000002	.	.	A	G	+	+	0.08571429	32,0,3,0	chr1,532902	Cytosol	A > G
chr1:53290298:A:G:UTR3:ZYG11B	chr1	53290298	53290298	A	G	UTR3	ZYG11B	ENST000002	.	.	A	G	+	+	0.225	30,0,9,1	chr1,532902	Cytosol	A > G
chr1:53291382:A:G:UTR3:ZYG11B	chr1	53291382	53291382	A	G	UTR3	ZYG11B	ENST000002	.	.	A	G	+	+	0.05194805	73,0,4,0	chr1,532913	Cytosol	A > G
chr1:53291408:A:G:UTR3:ZYG11B	chr1	53291408	53291408	A	G	UTR3	ZYG11B	ENST000002	.	.	A	G	+	+	0.12727273	96,0,14,0	chr1,532914	Cytosol	A > G
chr1:53291420:A:G:UTR3:ZYG11B	chr1	53291420	53291420	A	G	UTR3	ZYG11B	ENST000002	.	.	A	G	+	+	0.57009346	46,0,61,0	chr1,532914	Cytosol	A > G
chr1:53291425:A:G:UTR3:ZYG11B	chr1	53291425	53291425	A	G	UTR3	ZYG11B	ENST000002	.	.	A	G	+	+	0.47272727	58,0,52,0	chr1,532914	Cytosol	A > G
chr1:53291434:A:G:UTR3:ZYG11B	chr1	53291434	53291434	A	G	UTR3	ZYG11B	ENST000002	.	.	A	G	+	+	0.30973451	78,0,35,0	chr1,532914	Cytosol	A > G
chr1:53291435:A:G:UTR3:ZYG11B	chr1	53291435	53291435	A	G	UTR3	ZYG11B	ENST000002	.	.	A	G	+	+	0.28571429	75,0,30,0	chr1,532914	Cytosol	A > G
chr1:53359173:A:G:UTR3:ZYG11A	chr1	53359173	53359173	A	G	UTR3	ZYG11A	ENST000003	.	.	A	G	+	+	0.2962963	38,0,16,0	chr1,533591	Cytosol	A > G
chr1:53359181:A:G:UTR3:ZYG11A	chr1	53359181	53359181	A	G	UTR3	ZYG11A	ENST000003	.	.	A	G	+	+	0.29230769	46,0,19,0	chr1,533591	Cytosol	A > G
chr1:53359182:A:G:UTR3:ZYG11A	chr1	53359182	53359182	A	G	UTR3	ZYG11A	ENST000003	.	.	A	G	+	+	0.4516129	34,0,28,0	chr1,533591	Cytosol	A > G
chr1:53359258:A:G:UTR3:ZYG11A	chr1	53359258	53359258	A	G	UTR3	ZYG11A	ENST000003	.	.	A	G	+	+	0.93243243	5,0,69,0	chr1,533592	Cytosol	A > G
chr1:53359280:A:G:UTR3:ZYG11A	chr1	53359280	53359280	A	G	UTR3	ZYG11A	ENST000003	.	.	A	G	+	+	0.24561404	43,0,14,0	chr1,533592	Cytosol	A > G
chr1:53359285:A:G:UTR3:ZYG11A	chr1	53359285	53359285	A	G	UTR3	ZYG11A	ENST000003	.	.	A	G	+	+	0.30909091	38,0,17,0	chr1,533592	Cytosol	A > G
chr1:53359311:A:G:UTR3:ZYG11A	chr1	53359311	53359311	A	G	UTR3	ZYG11A	ENST000003	.	.	A	G	+	+	0.625	6,0,10,0	chr1,533593	Cytosol	A > G
chr1:53359362:A:G:UTR3:ZYG11A	chr1	53359362	53359362	A	G	UTR3	ZYG11A	ENST000003	.	.	A	G	+	+	0.8	2,0,8,0	chr1,533593	Cytosol	A > G
chr1:53359394:A:G:UTR3:ZYG11A	chr1	53359394	53359394	A	G	UTR3	ZYG11A	ENST000003	.	.	A	G	+	+	0.31578947	13,0,6,0	chr1,533593	Cytosol	A > G
chr1:53359629:A:G:UTR3:ZYG11A	chr1	53359629	53359629	A	G	UTR3	ZYG11A	ENST000003	.	.	A	G	+	+	0.7	6,0,14,0	chr1,533596	Cytosol	A > G
chr1:53359682:A:G:UTR3:ZYG11A	chr1	53359682	53359682	A	G	UTR3	ZYG11A	ENST000003	.	.	A	G	+	+	0.88235294	2,0,15,0	chr1,533596	Cytosol	A > G
chr1:53359745:A:G:UTR3:ZYG11A	chr1	53359745	53359745	A	G	UTR3	ZYG11A	ENST000003	.	.	A	G	+	+	0.41071429	33,0,23,0	chr1,533597	Cytosol	A > G
chr1:53366576:A:G:intronic:ECHD	chr1	53366576	53366576	A	G	intronic	ECHDC2	.	.	.	A	G	+	-	0.27272727	16,0,6,0	chr1,533665	Cytosol	A > G
chr1:53366591:A:G:intronic:ECHD	chr1	53366591	53366591	A	G	intronic	ECHDC2	.	.	.	A	G	+	-	0.32	17,0,8,0	chr1,533665	Cytosol	A > G
chr1:53366630:A:G:intronic:ECHD	chr1	53366630	53366630	A	G	intronic	ECHDC2	.	.	.	A	G	+	-	0.19047619	17,0,4,0	chr1,533666	Cytosol	A > G
chr1:53366738:A:G:intronic:ECHD	chr1	53366738	53366738	A	G	intronic	ECHDC2	.	.	.	A	G	+	-	0.25	12,0,4,0	chr1,533667	Cytosol	A > G
chr1:53367929:A:G:intronic:ECHD	chr1	53367929	53367929	A	G	intronic	ECHDC2	.	.	.	A	G	+	-	0.6	2,0,3,0	chr1,533679	Cytosol	A > G
chr1:53368152:A:G:intronic:ECHD	chr1	53368152	53368152	A	G	intronic	ECHDC2	.	.	.	A	G	+	-	0.44444444	15,0,12,0	chr1,533681	Cytosol	A > G

chr1:53679610:G:A:UTR3:CPT2	chr1	53679610	53679610	G	A	UTR3	CPT2	ENST000003	.	.	G	A	+	+	0.01652893	4,0,237,1	chr1,536796	Cytosol	G > A
chr1:54565296:A:G:UTR3:TCEANC	chr1	54565296	54565296	A	G	UTR3	TCEANC2	ENST000002	.	.	A	G	+	+	0.21052632	15,0,4,0	chr1,545652	Cytosol	A > G
chr1:54565361:A:G:UTR3:TCEANC	chr1	54565361	54565361	A	G	UTR3	TCEANC2	ENST000002	.	.	A	G	+	+	0.25	9,0,3,0	chr1,545653	Cytosol	A > G
chr1:54565362:A:G:UTR3:TCEANC	chr1	54565362	54565362	A	G	UTR3	TCEANC2	ENST000002	.	.	A	G	+	+	0.16666667	10,0,2,0	chr1,545653	Cytosol	A > G
chr1:54567347:A:G:intergenic:TCE	chr1	54567347	54567347	A	G	intergenic	TCEANC2,AL	dist=1931;dis	.	.	A	G	+	+	0.42857143	4,0,3,0	chr1,545673	Cytosol	A > G
chr1:54567369:A:G:intergenic:TCE	chr1	54567369	54567369	A	G	intergenic	TCEANC2,AL	dist=1953;dis	.	.	A	G	+	+	0.85714286	1,0,6,0	chr1,545673	Cytosol	A > G
chr1:54567390:A:G:intergenic:TCE	chr1	54567390	54567390	A	G	intergenic	TCEANC2,AL	dist=1974;dis	.	.	A	G	+	+	0.33333333	4,0,2,0	chr1,545673	Cytosol	A > G
chr1:59007205:T:C:UTR5:OMA1	chr1	59007205	59007205	T	C	UTR5	OMA1	ENST000004	.	.	A	G	-	-	0.6	2,0,3,0	chr1,590072	Cytosol	A > G
chr1:62627032:A:G:UTR3:INADL	chr1	62627032	62627032	A	G	UTR3	INADL	ENST000003	.	.	A	G	+	+	0.14285714	12,0,2,0	chr1,626270	Cytosol	A > G
chr1:62627042:A:G:UTR3:INADL	chr1	62627042	62627042	A	G	UTR3	INADL	ENST000003	.	.	A	G	+	+	0.46666667	8,0,7,0	chr1,626270	Cytosol	A > G
chr1:62627067:A:G:UTR3:INADL	chr1	62627067	62627067	A	G	UTR3	INADL	ENST000003	.	.	A	G	+	+	0.83333333	1,0,5,0	chr1,626270	Cytosol	A > G
chr1:62628530:A:G:UTR3:INADL	chr1	62628530	62628530	A	G	UTR3	INADL	ENST000003	.	.	A	G	+	+	0.11111111	16,0,2,0	chr1,626285	Cytosol	A > G
chr1:62914683:A:G:intronic:USP1	chr1	62914683	62914683	A	G	intronic	USP1	.	.	.	A	G	+	+	0.41176471	10,0,7,0	chr1,629146	Cytosol	A > G
chr1:62914720:A:G:intronic:USP1	chr1	62914720	62914720	A	G	intronic	USP1	.	.	.	A	G	+	+	0.23529412	13,0,4,0	chr1,629147	Cytosol	A > G
chr1:62914752:A:G:intronic:USP1	chr1	62914752	62914752	A	G	intronic	USP1	.	.	.	A	G	+	+	0.0625	30,0,2,0	chr1,629147	Cytosol	A > G
chr1:62915325:A:G:intronic:USP1	chr1	62915325	62915325	A	G	intronic	USP1	.	.	.	A	G	+	+	0.72727273	6,0,16,0	chr1,629153	Cytosol	A > G
chr1:62915432:A:G:intronic:USP1	chr1	62915432	62915432	A	G	intronic	USP1	.	.	.	A	G	+	+	0.15151515	28,0,5,0	chr1,629154	Cytosol	A > G
chr1:62915436:A:G:intronic:USP1	chr1	62915436	62915436	A	G	intronic	USP1	.	.	.	A	G	+	+	0.44117647	19,0,15,0	chr1,629154	Cytosol	A > G
chr1:67874689:T:C:UTR3:SERBP1	chr1	67874689	67874689	T	C	UTR3	SERBP1	ENST000003	.	.	A	G	-	-	0.72596154	57,0,151,0	chr1,678746	Cytosol	A > G
chr1:67874696:T:C:UTR3:SERBP1	chr1	67874696	67874696	T	C	UTR3	SERBP1	ENST000003	.	.	A	G	-	-	0.29126214	146,0,60,0	chr1,678746	Cytosol	A > G
chr1:67874697:T:C:UTR3:SERBP1	chr1	67874697	67874697	T	C	UTR3	SERBP1	ENST000003	.	.	A	G	-	-	0.30392157	142,0,62,0	chr1,678746	Cytosol	A > G
chr1:67874705:T:C:UTR3:SERBP1	chr1	67874705	67874705	T	C	UTR3	SERBP1	ENST000003	.	.	A	G	-	-	0.04301075	178,0,8,0	chr1,678747	Cytosol	A > G
chr1:67874731:T:C:UTR3:SERBP1	chr1	67874731	67874731	T	C	UTR3	SERBP1	ENST000003	.	.	A	G	-	-	0.46103896	83,0,71,0	chr1,678747	Cytosol	A > G
chr1:67874734:T:C:UTR3:SERBP1	chr1	67874734	67874734	T	C	UTR3	SERBP1	ENST000003	.	.	A	G	-	-	0.01910828	154,0,3,0	chr1,678747	Cytosol	A > G
chr1:67874825:T:C:UTR3:SERBP1	chr1	67874825	67874825	T	C	UTR3	SERBP1	ENST000003	.	.	A	G	-	-	0.14634146	35,0,6,0	chr1,678748	Cytosol	A > G
chr1:67874849:T:C:UTR3:SERBP1	chr1	67874849	67874849	T	C	UTR3	SERBP1	ENST000003	.	.	A	G	-	-	0.28888889	32,0,13,0	chr1,678748	Cytosol	A > G
chr1:67874859:T:C:UTR3:SERBP1	chr1	67874859	67874859	T	C	UTR3	SERBP1	ENST000003	.	.	A	G	-	-	0.3	49,0,21,0	chr1,678748	Cytosol	A > G
chr1:70905444:A:G:UTR3:CTH	chr1	70905444	70905444	A	G	UTR3	CTH	ENST000004	.	.	A	G	+	+	0.04210526	91,0,4,0	chr1,709054	Cytosol	A > G
chr1:70905509:A:G:UTR3:CTH	chr1	70905509	70905509	A	G	UTR3	CTH	ENST000004	.	.	A	G	+	+	0.13114754	53,0,8,0	chr1,709055	Cytosol	A > G
chr1:70905515:A:G:UTR3:CTH	chr1	70905515	70905515	A	G	UTR3	CTH	ENST000004	.	.	A	G	+	+	0.25423729	44,0,15,0	chr1,709055	Cytosol	A > G
chr1:70905519:A:G:UTR3:CTH	chr1	70905519	70905519	A	G	UTR3	CTH	ENST000004	.	.	A	G	+	+	0.22727273	51,0,15,0	chr1,709055	Cytosol	A > G
chr1:78255095:A:G:intronic:FAM7	chr1	78255095	78255095	A	G	intronic	FAM73A	.	.	.	A	G	+	+	0.33333333	10,0,5,0	chr1,782550	Cytosol	A > G
chr1:78256662:A:G:intronic:FAM7	chr1	78256662	78256662	A	G	intronic	FAM73A	.	.	.	A	G	+	+	0.77777778	2,0,7,0	chr1,782566	Cytosol	A > G
chr1:78256719:A:G:intronic:FAM7	chr1	78256719	78256719	A	G	intronic	FAM73A	.	.	.	A	G	+	+	0.9	1,0,9,0	chr1,782567	Cytosol	A > G
chr1:78256737:A:G:intronic:FAM7	chr1	78256737	78256737	A	G	intronic	FAM73A	.	.	.	A	G	+	+	0.85714286	1,0,6,0	chr1,782567	Cytosol	A > G
chr1:78334126:A:G:intronic:FAM7	chr1	78334126	78334126	A	G	intronic	FAM73A	.	.	.	A	G	+	+	0.25	6,0,2,0	chr1,783341	Cytosol	A > G
chr1:78342729:A:G:UTR3:FAM73A	chr1	78342729	78342729	A	G	UTR3	FAM73A	ENST000003	.	.	A	G	+	+	0.08148148	124,0,11,0	chr1,783427	Cytosol	A > G
chr1:86117191:T:C:UTR3:ZNHIT6	chr1	86117191	86117191	T	C	UTR3	ZNHIT6	ENST000004	.	.	A	G	-	-	0.36363636	7,0,4,0	chr1,861171	Cytosol	A > G
chr1:86117192:T:C:UTR3:ZNHIT6	chr1	86117192	86117192	T	C	UTR3	ZNHIT6	ENST000004	.	.	A	G	-	-	0.23076923	10,0,3,0	chr1,861171	Cytosol	A > G
chr1:92865956:A:G:UTR3:RPAP2	chr1	92865956	92865956	A	G	UTR3	RPAP2	ENST000006	.	.	A	G	+	+	0.6	2,0,3,0	chr1,928659	Cytosol	A > G
chr1:93620675:T:C:intronic:TMED	chr1	93620675	93620675	T	C	intronic	TMED5	.	.	.	A	G	-	-	0.42857143	4,0,3,0	chr1,936206	Cytosol	A > G
chr1:93621294:T:C:intronic:TMED	chr1	93621294	93621294	T	C	intronic	TMED5	.	.	.	A	G	-	-	0.375	5,0,3,0	chr1,936212	Cytosol	A > G
chr1:93729423:T:C:ncRNA_introni	chr1	93729423	93729423	T	C	ncRNA_intro	RP4-717I23.	.	.	.	A	G	-	+-	0.44444444	5,0,4,0	chr1,937294	Cytosol	A > G
chr1:100440553:A:G:UTR5:SLC35A	chr1	100440553	100440553	A	G	UTR5	SLC35A3	ENST000004	.	.	A	G	+	+	0.17647059	14,0,3,0	chr1,100440	Cytosol	A > G
chr1:100489507:A:G:UTR3:SLC35A	chr1	100489507	100489507	A	G	UTR3	SLC35A3	ENST000004	.	.	A	G	+	+	0.1754386	47,0,10,0	chr1,100489	Cytosol	A > G
chr1:100489518:A:G:UTR3:SLC35A	chr1	100489518	100489518	A	G	UTR3	SLC35A3	ENST000004	.	.	A	G	+	+	0.15714286	59,0,11,0	chr1,100489	Cytosol	A > G
chr1:100489835:A:G:UTR3:SLC35A	chr1	100489835	100489835	A	G	UTR3	SLC35A3	ENST000004	.	.	A	G	+	+	0.24242424	50,0,16,0	chr1,100489	Cytosol	A > G
chr1:100489992:A:G:UTR3:SLC35A	chr1	100489992	100489992	A	G	UTR3	SLC35A3	ENST000004	.	.	A	G	+	+	0.23529412	52,0,16,0	chr1,100489	Cytosol	A > G
chr1:100489996:A:G:UTR3:SLC35A	chr1	100489996	100489996	A	G	UTR3	SLC35A3	ENST000004	.	.	A	G	+	+	0.88235294	7,1,60,0	chr1,100489	Cytosol	A > G
chr1:100490000:A:G:UTR3:SLC35A	chr1	100490000	100490000	A	G	UTR3	SLC35A3	ENST000004	.	.	A	G	+	+	0.14516129	53,0,9,0	chr1,100489	Cytosol	A > G
chr1:100490042:A:G:UTR3:SLC35A	chr1	100490042	100490042	A	G	UTR3	SLC35A3	ENST000004	.	.	A	G	+	+	0.27777778	26,0,10,0	chr1,100490	Cytosol	A > G
chr1:100490056:A:G:UTR3:SLC35A	chr1	100490056	100490056	A	G	UTR3	SLC35A3	ENST000004	.	.	A	G	+	+	0.42424242	19,0,14,0	chr1,100490	Cytosol	A > G
chr1:100490057:A:G:UTR3:SLC35A	chr1	100490057	100490057	A	G	UTR3	SLC35A3	ENST000004	.	.	A	G	+	+	0.21875	25,0,7,0	chr1,100490	Cytosol	A > G
chr1:100490089:A:G:UTR3:SLC35A	chr1	100490089	100490089	A	G	UTR3	SLC35A3	ENST000004	.	.	A	G	+	+	0.19047619	34,0,8,0	chr1,100490	Cytosol	A > G
chr1:100490199:A:G:UTR3:SLC35A	chr1	100490199	100490199	A	G	UTR3	SLC35A3	ENST000004	.	.	A	G	+	+	0.48	13,0,12,0	chr1,100490	Cytosol	A > G
chr1:100490207:A:G:UTR3:SLC35A	chr1	100490207	100490207	A	G	UTR3	SLC35A3	ENST000004	.	.	A	G	+	+	0.64	9,0,16,0	chr1,100490	Cytosol	A > G
chr1:100490303:A:G:UTR3:SLC35A	chr1	100490303	100490303	A	G	UTR3	SLC35A3	ENST000004	.	.	A	G	+	+	0.62790698	16,0,27,0	chr1,100490	Cytosol	A > G
chr1:100611384:A:G:intronic:TRM	chr1	100611384	100611384	A	G	intronic	TRMT13	.	.	.	A	G	+	+	0.4	3,0,2,0	chr1,100611	Cytosol	A > G
chr1:100654812:T:C:UTR3:DBT	chr1	100654812	100654812	T	C	UTR3	DBT	ENST000003	.	.	A	G	-	-	0.34615385	17,0,9,0	chr1,100654	Cytosol	A > G
chr1:100654834:T:C:UTR3:DBT	chr1	100654834	100654834	T	C	UTR3	DBT	ENST000003	.	.	A	G	-	-	0.1	27,0,3,0	chr1,100654	Cytosol	A > G
chr1:100654870:T:C:UTR3:DBT	chr1	100654870	100654870	T	C	UTR3	DBT	ENST000003	.	.	A	G	-	-	0.29166667	17,0,7,0	chr1,100654	Cytosol	A > G
chr1:100654887:T:C:UTR3:DBT	chr1	100654887	100654887	T	C	UTR3	DBT	ENST000003	.	.	A	G	-	-	0.375	5,0,3,0	chr1,100654	Cytosol	A > G
chr1:100659265:T:C:UTR3:DBT	chr1	100659265	100659265	T	C	UTR3	DBT	ENST000003	.	.	A	G	-	-	0.14814815	23,0,4,0	chr1,100659	Cytosol	A > G
chr1:101541113:A:G:ncRNA_exon	chr1	101541113	101541113	A	G	ncRNA_exon	RP11-421L21	.	.	.	A	G	+	+	0.07317073	38,0,3,0	chr1,101541	Cytosol	A > G

chr1:101541134:A:G:ncRNA_exoni	chr1	101541134	101541134	A	G	ncRNA_exon	RP11-421L21	.	.	.	A	G	+	+	0.08	45,0,4,1	chr1,101541	Cytosol	A > G
chr1:101541153:A:G:ncRNA_exoni	chr1	101541153	101541153	A	G	ncRNA_exon	RP11-421L21	.	.	.	A	G	+	+	0.45652174	25,0,21,0	chr1,101541	Cytosol	A > G
chr1:101541170:A:G:ncRNA_exoni	chr1	101541170	101541170	A	G	ncRNA_exon	RP11-421L21	.	.	.	A	G	+	+	0.22413793	45,0,13,0	chr1,101541	Cytosol	A > G
chr1:101541172:A:G:ncRNA_exoni	chr1	101541172	101541172	A	G	ncRNA_exon	RP11-421L21	.	.	.	A	G	+	+	0.41071429	33,0,23,0	chr1,101541	Cytosol	A > G
chr1:109473256:A:G:UTR3:CLCC1,(	chr1	109473256	109473256	A	G	UTR3	CLCC1,GPSM	ENST000003(	.	.	A	G	+	+-	0.11111111	16,0,2,0	chr1,109473	Cytosol	A > G
chr1:109474650:T:C:UTR3:CLCC1,(	chr1	109474650	109474650	T	C	UTR3	CLCC1,GPSM	ENST000003(	.	.	A	G	-	+-	0.01904762	103,0,2,0	chr1,109474	Cytosol	A > G
chr1:109474774:T:C:UTR3:CLCC1,(	chr1	109474774	109474774	T	C	UTR3	CLCC1,GPSM	ENST000003(	.	.	A	G	-	+-	0.02631579	148,0,4,0	chr1,109474	Cytosol	A > G
chr1:109474780:T:C:UTR3:CLCC1,(	chr1	109474780	109474780	T	C	UTR3	CLCC1,GPSM	ENST000003(	.	.	A	G	-	+-	0.05035971	132,0,7,0	chr1,109474	Cytosol	A > G
chr1:109475628:A:G:UTR3:GPSM2	chr1	109475628	109475628	A	G	UTR3	GPSM2	ENST000004(	.	.	A	G	+	+-	0.17857143	23,0,5,0	chr1,109475	Cytosol	A > G
chr1:109475632:A:G:UTR3:GPSM2	chr1	109475632	109475632	A	G	UTR3	GPSM2	ENST000004(	.	.	A	G	+	+-	0.28571429	20,0,8,0	chr1,109475	Cytosol	A > G
chr1:109475641:T:C:UTR3:GPSM2	chr1	109475641	109475641	T	C	UTR3	GPSM2	ENST000004(	.	.	A	G	-	+-	0.85714286	2,0,12,0	chr1,109475	Cytosol	A > G
chr1:109475642:A:G:UTR3:GPSM2	chr1	109475642	109475642	A	G	UTR3	GPSM2	ENST000004(	.	.	A	G	+	+-	0.76923077	6,0,20,0	chr1,109475	Cytosol	A > G
chr1:109478281:A:G:intronic:AKN	chr1	109478281	109478281	A	G	intronic	AKNAD1,CLC	.	.	.	A	G	+	-	0.42857143	4,0,3,0	chr1,109478	Cytosol	A > G
chr1:109946028:T:C:intronic:PSM	chr1	109946028	109946028	T	C	intronic	PSMA5	.	.	.	A	G	-	-	0.42857143	4,0,3,0	chr1,109946	Cytosol	A > G
chr1:109951917:T:C:intronic:PSM	chr1	109951917	109951917	T	C	intronic	PSMA5	.	.	.	A	G	-	-	0.28571429	5,0,2,0	chr1,109951	Cytosol	A > G
chr1:109952319:T:C:intronic:PSM	chr1	109952319	109952319	T	C	intronic	PSMA5	.	.	.	A	G	-	-	0.16666667	10,0,2,0	chr1,109952	Cytosol	A > G
chr1:109952333:T:C:intronic:PSM	chr1	109952333	109952333	T	C	intronic	PSMA5	.	.	.	A	G	-	-	0.33333333	4,0,2,0	chr1,109952	Cytosol	A > G
chr1:110908616:A:G:intronic:SLC1	chr1	110908616	110908616	A	G	intronic	SLC16A4	.	.	.	A	G	+	-	0.28571429	5,0,2,0	chr1,110908	Cytosol	A > G
chr1:110908625:A:G:intronic:SLC1	chr1	110908625	110908625	A	G	intronic	SLC16A4	.	.	.	A	G	+	-	0.66666667	2,0,4,0	chr1,110908	Cytosol	A > G
chr1:110908631:A:G:intronic:SLC1	chr1	110908631	110908631	A	G	intronic	SLC16A4	.	.	.	A	G	+	-	0.25	6,0,2,0	chr1,110908	Cytosol	A > G
chr1:113150214:T:C:intronic:ST7L	chr1	113150214	113150214	T	C	intronic	ST7L	.	.	.	A	G	-	-	0.04347826	44,0,2,0	chr1,113150	Cytosol	A > G
chr1:113511340:A:G:ncRNA_exoni	chr1	113511340	113511340	A	G	ncRNA_exon	RP11-31F15.	.	.	.	A	G	+	+	0.11111111	16,0,2,0	chr1,113511	Cytosol	A > G
chr1:113511408:A:G:ncRNA_exoni	chr1	113511408	113511408	A	G	ncRNA_exon	RP11-31F15.	.	.	.	A	G	+	+	0.11111111	16,0,2,0	chr1,113511	Cytosol	A > G
chr1:113511831:A:G:ncRNA_intro	chr1	113511831	113511831	A	G	ncRNA_intro	RP11-31F15.	.	.	.	A	G	+	+	0.2	8,0,2,0	chr1,113511	Cytosol	A > G
chr1:113511842:A:G:ncRNA_intro	chr1	113511842	113511842	A	G	ncRNA_intro	RP11-31F15.	.	.	.	A	G	+	+	0.15384615	11,0,2,0	chr1,113511	Cytosol	A > G
chr1:113511845:A:G:ncRNA_intro	chr1	113511845	113511845	A	G	ncRNA_intro	RP11-31F15.	.	.	.	A	G	+	+	0.18181818	9,0,2,0	chr1,113511	Cytosol	A > G
chr1:113511927:A:G:ncRNA_intro	chr1	113511927	113511927	A	G	ncRNA_intro	RP11-31F15.	.	.	.	A	G	+	+	0.08695652	21,0,2,0	chr1,113511	Cytosol	A > G
chr1:113512093:A:G:ncRNA_intro	chr1	113512093	113512093	A	G	ncRNA_intro	RP11-31F15.	.	.	.	A	G	+	+	0.06666667	28,0,2,0	chr1,113512	Cytosol	A > G
chr1:113512094:A:G:ncRNA_intro	chr1	113512094	113512094	A	G	ncRNA_intro	RP11-31F15.	.	.	.	A	G	+	+	0.27586207	21,0,8,0	chr1,113512	Cytosol	A > G
chr1:113512117:A:G:ncRNA_intro	chr1	113512117	113512117	A	G	ncRNA_intro	RP11-31F15.	.	.	.	A	G	+	+	0.18181818	18,0,4,0	chr1,113512	Cytosol	A > G
chr1:113512168:A:G:ncRNA_intro	chr1	113512168	113512168	A	G	ncRNA_intro	RP11-31F15.	.	.	.	A	G	+	+	0.33333333	10,0,5,0	chr1,113512	Cytosol	A > G
chr1:113512204:A:G:ncRNA_intro	chr1	113512204	113512204	A	G	ncRNA_intro	RP11-31F15.	.	.	.	A	G	+	+	0.4	9,0,6,0	chr1,113512	Cytosol	A > G
chr1:113512241:A:G:ncRNA_intro	chr1	113512241	113512241	A	G	ncRNA_intro	RP11-31F15.	.	.	.	A	G	+	+	0.08	23,0,2,0	chr1,113512	Cytosol	A > G
chr1:113513025:A:G:ncRNA_intro	chr1	113513025	113513025	A	G	ncRNA_intro	RP11-31F15.	.	.	.	A	G	+	+	0.88888889	1,0,8,0	chr1,113513	Cytosol	A > G
chr1:113513095:A:G:ncRNA_intro	chr1	113513095	113513095	A	G	ncRNA_intro	RP11-31F15.	.	.	.	A	G	+	+	0.4	6,0,4,0	chr1,113513	Cytosol	A > G
chr1:113513102:A:G:ncRNA_intro	chr1	113513102	113513102	A	G	ncRNA_intro	RP11-31F15.	.	.	.	A	G	+	+	0.66666667	3,0,6,0	chr1,113513	Cytosol	A > G
chr1:113513105:A:G:ncRNA_intro	chr1	113513105	113513105	A	G	ncRNA_intro	RP11-31F15.	.	.	.	A	G	+	+	0.22222222	7,0,2,0	chr1,113513	Cytosol	A > G
chr1:113513119:A:G:ncRNA_intro	chr1	113513119	113513119	A	G	ncRNA_intro	RP11-31F15.	.	.	.	A	G	+	+	0.4	3,0,2,0	chr1,113513	Cytosol	A > G
chr1:113513131:A:G:ncRNA_intro	chr1	113513131	113513131	A	G	ncRNA_intro	RP11-31F15.	.	.	.	A	G	+	+	0.25	6,0,2,0	chr1,113513	Cytosol	A > G
chr1:113513132:A:G:ncRNA_intro	chr1	113513132	113513132	A	G	ncRNA_intro	RP11-31F15.	.	.	.	A	G	+	+	0.75	2,0,6,0	chr1,113513	Cytosol	A > G
chr1:113513276:A:G:ncRNA_intro	chr1	113513276	113513276	A	G	ncRNA_intro	RP11-31F15.	.	.	.	A	G	+	+	0.46153846	7,0,6,0	chr1,113513	Cytosol	A > G
chr1:113513279:A:G:ncRNA_intro	chr1	113513279	113513279	A	G	ncRNA_intro	RP11-31F15.	.	.	.	A	G	+	+	0.5	6,0,6,0	chr1,113513	Cytosol	A > G
chr1:113667521:A:G:UTR3:LRIG2	chr1	113667521	113667521	A	G	UTR3	LRIG2	ENST000003(	.	.	A	G	+	+	0.08	23,0,2,0	chr1,113667	Cytosol	A > G
chr1:114287471:T:C:intronic:PHTF	chr1	114287471	114287471	T	C	intronic	PHTF1	.	.	.	A	G	-	-	0.375	5,0,3,0	chr1,114287	Cytosol	A > G
chr1:114290697:T:C:intronic:PHTF	chr1	114290697	114290697	T	C	intronic	PHTF1	.	.	.	A	G	-	-	0.09433962	48,0,5,0	chr1,114290	Cytosol	A > G
chr1:114290789:T:C:intronic:PHTF	chr1	114290789	114290789	T	C	intronic	PHTF1	.	.	.	A	G	-	-	0.06976744	40,0,3,0	chr1,114290	Cytosol	A > G
chr1:114291294:T:C:intronic:PHTF	chr1	114291294	114291294	T	C	intronic	PHTF1	.	.	.	A	G	-	-	0.11538462	46,0,6,0	chr1,114291	Cytosol	A > G
chr1:115108995:T:C:intergenic:TR	chr1	115108995	115108995	T	C	intergenic	TRIM33,BCA	dist=55214;d	.	.	A	G	-	-	0.33333333	10,0,5,0	chr1,115108	Cytosol	A > G
chr1:115109037:T:C:intergenic:TR	chr1	115109037	115109037	T	C	intergenic	TRIM33,BCA	dist=55256;d	.	.	A	G	-	-	0.33333333	4,0,2,0	chr1,115109	Cytosol	A > G
chr1:115109156:T:C:intergenic:TR	chr1	115109156	115109156	T	C	intergenic	TRIM33,BCA	dist=55375;d	.	.	A	G	-	-	0.27777778	13,0,5,0	chr1,115109	Cytosol	A > G
chr1:115109170:T:C:intergenic:TR	chr1	115109170	115109170	T	C	intergenic	TRIM33,BCA	dist=55389;d	.	.	A	G	-	-	0.14285714	12,0,2,0	chr1,115109	Cytosol	A > G
chr1:115109576:T:C:downstream:	chr1	115109576	115109576	T	C	downstream	BCAS2	.	.	.	A	G	-	-	0.45454545	6,0,5,0	chr1,115109	Cytosol	A > G
chr1:117624935:A:G:intronic:TTF2	chr1	117624935	117624935	A	G	intronic	TTF2	.	.	.	A	G	+	+	0.10526316	17,0,2,0	chr1,117624	Cytosol	A > G
chr1:117624947:A:G:intronic:TTF2	chr1	117624947	117624947	A	G	intronic	TTF2	.	.	.	A	G	+	+	0.4375	9,0,7,0	chr1,117624	Cytosol	A > G
chr1:117625008:A:G:intronic:TTF2	chr1	117625008	117625008	A	G	intronic	TTF2	.	.	.	A	G	+	+	0.25	6,0,2,0	chr1,117625	Cytosol	A > G
chr1:117625025:A:G:intronic:TTF2	chr1	117625025	117625025	A	G	intronic	TTF2	.	.	.	A	G	+	+	0.23076923	10,0,3,0	chr1,117625	Cytosol	A > G
chr1:117625087:A:G:intronic:TTF2	chr1	117625087	117625087	A	G	intronic	TTF2	.	.	.	A	G	+	+	0.08	23,0,2,0	chr1,117625	Cytosol	A > G
chr1:117625770:A:G:intronic:TTF2	chr1	117625770	117625770	A	G	intronic	TTF2	.	.	.	A	G	+	+	0.4	3,0,2,0	chr1,117625	Cytosol	A > G
chr1:117626500:A:G:intronic:TTF2	chr1	117626500	117626500	A	G	intronic	TTF2	.	.	.	A	G	+	+	0.28571429	5,0,2,0	chr1,117626	Cytosol	A > G
chr1:119693495:A:G:ncRNA_exoni	chr1	119693495	119693495	A	G	ncRNA_exon	RP11-418J17	.	.	.	A	G	+	+	0.18604651	35,0,8,0	chr1,119693	Cytosol	A > G
chr1:119693512:A:G:ncRNA_exoni	chr1	119693512	119693512	A	G	ncRNA_exon	RP11-418J17	.	.	.	A	G	+	+	0.11904762	37,0,5,0	chr1,119693	Cytosol	A > G
chr1:119693521:A:G:ncRNA_exoni	chr1	119693521	119693521	A	G	ncRNA_exon	RP11-418J17	.	.	.	A	G	+	+	0.08571429	32,0,3,0	chr1,119693	Cytosol	A > G
chr1:144615104:T:C:ncRNA_exoni	chr1	144615104	144615104	T	C	ncRNA_exon	RP11-640M9	.	.	.	T	C	+	+	0.29411765	0,10,0,24	chr1,144615	Cytosol	T > C
chr1:144829576:A:G:UTR3:NBPF9	chr1	144829576	144829576	A	G	UTR3	NBPF9	ENST000004	.	.	A	G	+	-+	0.08219178	67,0,6,0	chr1,144829	Cytosol	A > G

chr1:144829580:A:G:UTR3:NBPF9	chr1	144829580	144829580	A	G	UTR3	NBPF9	ENST000004	.	.	A	G	+	-+	0.43661972	40,0,31,0	chr1,144829	Cytosol	A > G
chr1:144829583:A:G:UTR3:NBPF9	chr1	144829583	144829583	A	G	UTR3	NBPF9	ENST000004	.	.	A	G	+	-+	0.2739726	53,0,20,0	chr1,144829	Cytosol	A > G
chr1:144829598:A:G:UTR3:NBPF9	chr1	144829598	144829598	A	G	UTR3	NBPF9	ENST000004	.	.	A	G	+	-+	0.5	54,0,54,0	chr1,144829	Cytosol	A > G
chr1:144829809:A:G:UTR3:NBPF9	chr1	144829809	144829809	A	G	UTR3	NBPF9	ENST000004	.	.	A	G	+	-+	0.85714286	1,0,6,0	chr1,144829	Cytosol	A > G
chr1:145369498:A:G:UTR3:NBPF1	chr1	145369498	145369498	A	G	UTR3	NBPF10	ENST000003	.	.	A	G	+	+	0.46153846	7,0,6,0	chr1,145369	Cytosol	A > G
chr1:145369556:A:G:UTR3:NBPF1	chr1	145369556	145369556	A	G	UTR3	NBPF10	ENST000003	.	.	A	G	+	+	0.13043478	20,0,3,0	chr1,145369	Cytosol	A > G
chr1:145512930:A:G:ncRNA_intro	chr1	145512930	145512930	A	G	ncRNA_intro	GNRHR2	.	.	.	A	G	+	+-	0.64150943	19,0,34,0	chr1,145512	Cytosol	A > G
chr1:145512937:A:G:ncRNA_intro	chr1	145512937	145512937	A	G	ncRNA_intro	GNRHR2	.	.	.	A	G	+	+-	0.04878049	39,0,2,0	chr1,145512	Cytosol	A > G
chr1:145512944:A:G:ncRNA_intro	chr1	145512944	145512944	A	G	ncRNA_intro	GNRHR2	.	.	.	A	G	+	+-	0.775	8,0,31,1	chr1,145512	Cytosol	A > G
chr1:145512950:A:G:ncRNA_intro	chr1	145512950	145512950	A	G	ncRNA_intro	GNRHR2	.	.	.	A	G	+	+-	0.48648649	19,0,18,0	chr1,145512	Cytosol	A > G
chr1:145512951:A:G:ncRNA_intro	chr1	145512951	145512951	A	G	ncRNA_intro	GNRHR2	.	.	.	A	G	+	+-	0.69230769	12,0,27,0	chr1,145512	Cytosol	A > G
chr1:145513003:A:G:ncRNA_intro	chr1	145513003	145513003	A	G	ncRNA_intro	GNRHR2	.	.	.	A	G	+	+-	0.06	47,0,3,0	chr1,145513	Cytosol	A > G
chr1:145513017:A:G:ncRNA_intro	chr1	145513017	145513017	A	G	ncRNA_intro	GNRHR2	.	.	.	A	G	+	+-	0.51351351	18,0,19,0	chr1,145513	Cytosol	A > G
chr1:145688923:A:G:UTR3:RNF11	chr1	145688923	145688923	A	G	UTR3	RNF115	ENST000003	.	.	A	G	+	+	0.03846154	50,0,2,0	chr1,145688	Cytosol	A > G
chr1:145688924:A:G:UTR3:RNF11	chr1	145688924	145688924	A	G	UTR3	RNF115	ENST000003	.	.	A	G	+	+	0.03636364	53,0,2,0	chr1,145688	Cytosol	A > G
chr1:145688938:A:G:UTR3:RNF11	chr1	145688938	145688938	A	G	UTR3	RNF115	ENST000003	.	.	A	G	+	+	0.20689655	46,0,12,0	chr1,145688	Cytosol	A > G
chr1:145688943:A:G:UTR3:RNF11	chr1	145688943	145688943	A	G	UTR3	RNF115	ENST000003	.	.	A	G	+	+	0.11666667	53,0,7,0	chr1,145688	Cytosol	A > G
chr1:145688944:A:G:UTR3:RNF11	chr1	145688944	145688944	A	G	UTR3	RNF115	ENST000003	.	.	A	G	+	+	0.16949153	49,0,10,0	chr1,145688	Cytosol	A > G
chr1:145688961:A:G:UTR3:RNF11	chr1	145688961	145688961	A	G	UTR3	RNF115	ENST000003	.	.	A	G	+	+	0.10169492	53,0,6,0	chr1,145688	Cytosol	A > G
chr1:145688973:A:G:UTR3:RNF11	chr1	145688973	145688973	A	G	UTR3	RNF115	ENST000003	.	.	A	G	+	+	0.23333333	46,0,14,0	chr1,145688	Cytosol	A > G
chr1:145688983:A:G:UTR3:RNF11	chr1	145688983	145688983	A	G	UTR3	RNF115	ENST000003	.	.	A	G	+	+	0.2173913	36,0,10,0	chr1,145688	Cytosol	A > G
chr1:145688985:A:G:UTR3:RNF11	chr1	145688985	145688985	A	G	UTR3	RNF115	ENST000003	.	.	A	G	+	+	0.06122449	46,0,3,0	chr1,145688	Cytosol	A > G
chr1:145689025:A:G:UTR3:RNF11	chr1	145689025	145689025	A	G	UTR3	RNF115	ENST000003	.	.	A	G	+	+	0.05405405	35,0,2,0	chr1,145689	Cytosol	A > G
chr1:145689028:A:G:UTR3:RNF11	chr1	145689028	145689028	A	G	UTR3	RNF115	ENST000003	.	.	A	G	+	+	0.38709677	19,0,12,0	chr1,145689	Cytosol	A > G
chr1:145689437:A:G:UTR3:RNF11	chr1	145689437	145689437	A	G	UTR3	RNF115	ENST000003	.	.	A	G	+	+	0.18181818	9,0,2,0	chr1,145689	Cytosol	A > G
chr1:145689448:A:G:UTR3:RNF11	chr1	145689448	145689448	A	G	UTR3	RNF115	ENST000003	.	.	A	G	+	+	0.38888889	11,0,7,0	chr1,145689	Cytosol	A > G
chr1:145689491:A:G:UTR3:RNF11	chr1	145689491	145689491	A	G	UTR3	RNF115	ENST000003	.	.	A	G	+	+	0.55172414	13,0,16,0	chr1,145689	Cytosol	A > G
chr1:145689492:A:G:UTR3:RNF11	chr1	145689492	145689492	A	G	UTR3	RNF115	ENST000003	.	.	A	G	+	+	0.34285714	23,0,12,0	chr1,145689	Cytosol	A > G
chr1:145689533:A:G:UTR3:RNF11	chr1	145689533	145689533	A	G	UTR3	RNF115	ENST000003	.	.	A	G	+	+	0.48275862	30,0,28,0	chr1,145689	Cytosol	A > G
chr1:145689537:A:G:UTR3:RNF11	chr1	145689537	145689537	A	G	UTR3	RNF115	ENST000003	.	.	A	G	+	+	0.4137931	34,0,24,0	chr1,145689	Cytosol	A > G
chr1:145689566:A:G:UTR3:RNF11	chr1	145689566	145689566	A	G	UTR3	RNF115	ENST000003	.	.	A	G	+	+	0.64	18,0,32,0	chr1,145689	Cytosol	A > G
chr1:145689568:A:G:UTR3:RNF11	chr1	145689568	145689568	A	G	UTR3	RNF115	ENST000003	.	.	A	G	+	+	0.58823529	21,0,30,0	chr1,145689	Cytosol	A > G
chr1:145689590:A:G:UTR3:RNF11	chr1	145689590	145689590	A	G	UTR3	RNF115	ENST000003	.	.	A	G	+	+	0.05128205	37,0,2,0	chr1,145689	Cytosol	A > G
chr1:145689601:A:G:UTR3:RNF11	chr1	145689601	145689601	A	G	UTR3	RNF115	ENST000003	.	.	A	G	+	+	0.19444444	29,0,7,0	chr1,145689	Cytosol	A > G
chr1:145689693:A:G:UTR3:RNF11	chr1	145689693	145689693	A	G	UTR3	RNF115	ENST000003	.	.	A	G	+	+	0.02739726	71,0,2,0	chr1,145689	Cytosol	A > G
chr1:145689701:A:G:UTR3:RNF11	chr1	145689701	145689701	A	G	UTR3	RNF115	ENST000003	.	.	A	G	+	+	0.09589041	66,0,7,0	chr1,145689	Cytosol	A > G
chr1:145689717:A:G:UTR3:RNF11	chr1	145689717	145689717	A	G	UTR3	RNF115	ENST000003	.	.	A	G	+	+	0.453125	35,0,29,0	chr1,145689	Cytosol	A > G
chr1:145689790:A:G:UTR3:RNF11	chr1	145689790	145689790	A	G	UTR3	RNF115	ENST000003	.	.	A	G	+	+	0.0625	60,0,4,0	chr1,145689	Cytosol	A > G
chr1:145689812:A:G:UTR3:RNF11	chr1	145689812	145689812	A	G	UTR3	RNF115	ENST000003	.	.	A	G	+	+	0.2	24,0,6,0	chr1,145689	Cytosol	A > G
chr1:145689843:A:G:UTR3:RNF11	chr1	145689843	145689843	A	G	UTR3	RNF115	ENST000003	.	.	A	G	+	+	0.09836066	55,0,6,0	chr1,145689	Cytosol	A > G
chr1:146467146:A:G:UTR3:NBPF1	chr1	146467146	146467146	A	G	UTR3	NBPF12	ENST000004	.	.	A	G	+	+	0.11111111	24,0,3,0	chr1,146467	Cytosol	A > G
chr1:148003752:T:C:UTR3:NBPF14	chr1	148003752	148003752	T	C	UTR3	NBPF14	ENST000003	.	.	A	G	-	-	0.7755102	22,0,76,0	chr1,148003	Cytosol	A > G
chr1:148595576:A:G:UTR3:NBPF1	chr1	148595576	148595576	A	G	UTR3	NBPF15	ENST000004	.	.	A	G	+	-+	0.7	3,0,7,0	chr1,148595	Cytosol	A > G
chr1:148595664:A:G:UTR3:NBPF1	chr1	148595664	148595664	A	G	UTR3	NBPF15	ENST000004	.	.	A	G	+	-+	0.09302326	39,0,4,0	chr1,148595	Cytosol	A > G
chr1:148595665:A:G:UTR3:NBPF1	chr1	148595665	148595665	A	G	UTR3	NBPF15	ENST000004	.	.	A	G	+	-+	0.5	28,0,28,0	chr1,148595	Cytosol	A > G
chr1:150281151:A:G:UTR3:MRPS2	chr1	150281151	150281151	A	G	UTR3	MRPS21	ENST000003	.	.	A	G	+	+	0.28947368	27,0,11,0	chr1,150281	Cytosol	A > G
chr1:150281156:A:G:UTR3:MRPS2	chr1	150281156	150281156	A	G	UTR3	MRPS21	ENST000003	.	.	A	G	+	+	0.05714286	33,0,2,0	chr1,150281	Cytosol	A > G
chr1:150281191:A:G:UTR3:MRPS2	chr1	150281191	150281191	A	G	UTR3	MRPS21	ENST000003	.	.	A	G	+	+	0.25	42,0,14,0	chr1,150281	Cytosol	A > G
chr1:150281192:A:G:UTR3:MRPS2	chr1	150281192	150281192	A	G	UTR3	MRPS21	ENST000003	.	.	A	G	+	+	0.18518519	44,0,10,0	chr1,150281	Cytosol	A > G
chr1:150281203:A:G:UTR3:MRPS2	chr1	150281203	150281203	A	G	UTR3	MRPS21	ENST000003	.	.	A	G	+	+	0.24528302	40,0,13,0	chr1,150281	Cytosol	A > G
chr1:150281941:A:G:downstream:	chr1	150281941	150281941	A	G	downstream	MRPS21	.	.	.	A	G	+		0.79411765	7,0,27,0	chr1,150281	Cytosol	A > G
chr1:150281996:A:G:downstream:	chr1	150281996	150281996	A	G	downstream	MRPS21	.	.	.	A	G	+		0.33333333	22,0,11,0	chr1,150281	Cytosol	A > G
chr1:150585654:T:C:intronic:ENSA	chr1	150585654	150585654	T	C	intronic	ENSA	.	.	.	A	G	-	-	1	0,0,5,0	chr1,150585	Cytosol	A > G
chr1:150586519:T:C:intronic:ENSA	chr1	150586519	150586519	T	C	intronic	ENSA	.	.	.	A	G	-	-	0.42857143	4,0,3,0	chr1,150586	Cytosol	A > G
chr1:150591586:T:C:intronic:ENSA	chr1	150591586	150591586	T	C	intronic	ENSA	.	.	.	A	G	-	-	0.33333333	4,0,2,0	chr1,150591	Cytosol	A > G
chr1:150593315:T:C:intronic:ENSA	chr1	150593315	150593315	T	C	intronic	ENSA	.	.	.	A	G	-	-	0.42857143	4,0,3,0	chr1,150593	Cytosol	A > G
chr1:150593325:T:C:intronic:ENSA	chr1	150593325	150593325	T	C	intronic	ENSA	.	.	.	A	G	-	-	0.28571429	5,0,2,0	chr1,150593	Cytosol	A > G
chr1:150593888:T:C:UTR3:ENSA	chr1	150593888	150593888	T	C	UTR3	ENSA	ENST000003	.	.	A	G	-	-	0.44444444	5,0,4,0	chr1,150593	Cytosol	A > G
chr1:150594025:T:C:UTR3:ENSA	chr1	150594025	150594025	T	C	UTR3	ENSA	ENST000003	.	.	A	G	-	-	0.1	18,0,2,0	chr1,150594	Cytosol	A > G
chr1:150903175:A:G:intronic:SETD	chr1	150903175	150903175	A	G	intronic	SETDB1	.	.	.	A	G	+	+	0.66666667	3,0,6,0	chr1,150903	Cytosol	A > G
chr1:150903179:A:G:intronic:SETD	chr1	150903179	150903179	A	G	intronic	SETDB1	.	.	.	A	G	+	+	0.33333333	6,0,3,0	chr1,150903	Cytosol	A > G
chr1:150903474:A:G:intronic:SETD	chr1	150903474	150903474	A	G	intronic	SETDB1	.	.	.	A	G	+	+	0.83333333	1,0,5,0	chr1,150903	Cytosol	A > G
chr1:150903655:A:G:intronic:SETD	chr1	150903655	150903655	A	G	intronic	SETDB1	.	.	.	A	G	+	+	0.27272727	8,0,3,0	chr1,150903	Cytosol	A > G
chr1:150903656:A:G:intronic:SETD	chr1	150903656	150903656	A	G	intronic	SETDB1	.	.	.	A	G	+	+	0.18181818	9,0,2,0	chr1,150903	Cytosol	A > G

chr1:150965857:T:C:intronic:ANX	chr1	150965857	150965857	T	C	intronic	ANXA9	.	.	.	A	G	-	+	0.25	6,0,2,0	chr1,150965	Cytosol	A > G
chr1:150965858:T:C:intronic:ANX	chr1	150965858	150965858	T	C	intronic	ANXA9	.	.	.	A	G	-	+	0.5	5,0,5,0	chr1,150965	Cytosol	A > G
chr1:150965922:T:C:intronic:ANX	chr1	150965922	150965922	T	C	intronic	ANXA9	.	.	.	A	G	-	+	0.25	6,0,2,0	chr1,150965	Cytosol	A > G
chr1:150965964:T:C:intronic:ANX	chr1	150965964	150965964	T	C	intronic	ANXA9	.	.	.	A	G	-	+	0.15384615	11,0,2,0	chr1,150965	Cytosol	A > G
chr1:150967446:T:C:intronic:ANX	chr1	150967446	150967446	T	C	intronic	ANXA9	.	.	.	A	G	-	+	0.8	1,0,4,0	chr1,150967	Cytosol	A > G
chr1:150968658:T:C:downstream:	chr1	150968658	150968658	T	C	downstream	ANXA9,FAM	.	.	.	A	G	-		0.75	2,0,6,0	chr1,150968	Cytosol	A > G
chr1:150968661:T:C:downstream:	chr1	150968661	150968661	T	C	downstream	ANXA9,FAM	.	.	.	A	G	-		0.66666667	3,0,6,0	chr1,150968	Cytosol	A > G
chr1:150968682:T:C:downstream:	chr1	150968682	150968682	T	C	downstream	ANXA9,FAM	.	.	.	A	G	-		0.5	4,0,4,0	chr1,150968	Cytosol	A > G
chr1:150968729:T:C:downstream:	chr1	150968729	150968729	T	C	downstream	ANXA9,FAM	.	.	.	A	G	-		0.33333333	8,0,4,0	chr1,150968	Cytosol	A > G
chr1:150969199:T:C:UTR3:FAM63	chr1	150969199	150969199	T	C	UTR3	FAM63A	ENST000003	.	.	A	G	-	-	0.5	3,0,3,0	chr1,150969	Cytosol	A > G
chr1:150969257:T:C:UTR3:FAM63	chr1	150969257	150969257	T	C	UTR3	FAM63A	ENST000003	.	.	A	G	-	-	0.21428571	11,0,3,0	chr1,150969	Cytosol	A > G
chr1:150969270:T:C:UTR3:FAM63	chr1	150969270	150969270	T	C	UTR3	FAM63A	ENST000003	.	.	A	G	-	-	0.125	14,0,2,0	chr1,150969	Cytosol	A > G
chr1:150969286:T:C:UTR3:FAM63	chr1	150969286	150969286	T	C	UTR3	FAM63A	ENST000003	.	.	A	G	-	-	0.14285714	12,0,2,0	chr1,150969	Cytosol	A > G
chr1:150969311:T:C:UTR3:FAM63	chr1	150969311	150969311	T	C	UTR3	FAM63A	ENST000003	.	.	A	G	-	-	0.13333333	13,0,2,0	chr1,150969	Cytosol	A > G
chr1:150976428:T:C:intronic:FAM	chr1	150976428	150976428	T	C	intronic	FAM63A	.	.	.	A	G	-	-	0.33333333	4,0,2,0	chr1,150976	Cytosol	A > G
chr1:151045618:A:G:intronic:GAB	chr1	151045618	151045618	A	G	intronic	GABPB2	.	.	.	A	G	+	+	0.4	3,0,2,0	chr1,151045	Cytosol	A > G
chr1:151045624:A:G:intronic:GAB	chr1	151045624	151045624	A	G	intronic	GABPB2	.	.	.	A	G	+	+	1	0,0,7,0	chr1,151045	Cytosol	A > G
chr1:151091327:A:G:UTR3:GABPB	chr1	151091327	151091327	A	G	UTR3	GABPB2	ENST000003	.	.	A	G	+	+	1	0,0,6,0	chr1,151091	Cytosol	A > G
chr1:151091332:A:G:UTR3:GABPB	chr1	151091332	151091332	A	G	UTR3	GABPB2	ENST000003	.	.	A	G	+	+	0.8	1,0,4,0	chr1,151091	Cytosol	A > G
chr1:151091334:A:G:UTR3:GABPB	chr1	151091334	151091334	A	G	UTR3	GABPB2	ENST000003	.	.	A	G	+	+	0.33333333	4,0,2,0	chr1,151091	Cytosol	A > G
chr1:151091661:A:G:UTR3:GABPB	chr1	151091661	151091661	A	G	UTR3	GABPB2	ENST000003	.	.	A	G	+	+	0.13333333	13,0,2,0	chr1,151091	Cytosol	A > G
chr1:151091836:A:G:UTR3:GABPB	chr1	151091836	151091836	A	G	UTR3	GABPB2	ENST000003	.	.	A	G	+	+	0.71428571	2,0,5,0	chr1,151091	Cytosol	A > G
chr1:151092164:A:G:UTR3:GABPB	chr1	151092164	151092164	A	G	UTR3	GABPB2	ENST000003	.	.	A	G	+	+	0.27272727	8,0,3,0	chr1,151092	Cytosol	A > G
chr1:151094175:A:G:UTR3:GABPB	chr1	151094175	151094175	A	G	UTR3	GABPB2	ENST000003	.	.	A	G	+	+	0.33333333	6,0,3,0	chr1,151094	Cytosol	A > G
chr1:151094176:A:G:UTR3:GABPB	chr1	151094176	151094176	A	G	UTR3	GABPB2	ENST000003	.	.	A	G	+	+	0.44444444	5,0,4,0	chr1,151094	Cytosol	A > G
chr1:151095834:A:G:UTR3:GABPB	chr1	151095834	151095834	A	G	UTR3	GABPB2	ENST000003	.	.	A	G	+	+	0.57142857	3,0,4,0	chr1,151095	Cytosol	A > G
chr1:151096737:A:G:UTR3:GABPB	chr1	151096737	151096737	A	G	UTR3	GABPB2	ENST000003	.	.	A	G	+	+	0.3	7,0,3,0	chr1,151096	Cytosol	A > G
chr1:151096738:A:G:UTR3:GABPB	chr1	151096738	151096738	A	G	UTR3	GABPB2	ENST000003	.	.	A	G	+	+	0.375	5,0,3,0	chr1,151096	Cytosol	A > G
chr1:151096755:A:G:UTR3:GABPB	chr1	151096755	151096755	A	G	UTR3	GABPB2	ENST000003	.	.	A	G	+	+	0.35294118	11,0,6,0	chr1,151096	Cytosol	A > G
chr1:151096767:A:G:UTR3:GABPB	chr1	151096767	151096767	A	G	UTR3	GABPB2	ENST000003	.	.	A	G	+	+	0.23529412	13,0,4,0	chr1,151096	Cytosol	A > G
chr1:151096805:A:G:UTR3:GABPB	chr1	151096805	151096805	A	G	UTR3	GABPB2	ENST000003	.	.	A	G	+	+	0.64285714	5,0,9,0	chr1,151096	Cytosol	A > G
chr1:151096822:A:G:UTR3:GABPB	chr1	151096822	151096822	A	G	UTR3	GABPB2	ENST000003	.	.	A	G	+	+	0.33333333	4,0,2,0	chr1,151096	Cytosol	A > G
chr1:151096827:A:G:UTR3:GABPB	chr1	151096827	151096827	A	G	UTR3	GABPB2	ENST000003	.	.	A	G	+	+	0.71428571	2,0,5,0	chr1,151096	Cytosol	A > G
chr1:151843566:T:C:intergenic:TH	chr1	151843566	151843566	T	C	intergenic	THEM5,THEM	dist=17393;d	.	.	A	G	-	-	0.5625	14,0,18,0	chr1,151843	Cytosol	A > G
chr1:151843571:T:C:intergenic:TH	chr1	151843571	151843571	T	C	intergenic	THEM5,THEM	dist=17398;d	.	.	A	G	-	-	0.23076923	20,0,6,0	chr1,151843	Cytosol	A > G
chr1:151843629:T:C:intergenic:TH	chr1	151843629	151843629	T	C	intergenic	THEM5,THEM	dist=17456;d	.	.	A	G	-	-	0.625	6,0,10,0	chr1,151843	Cytosol	A > G
chr1:151843635:T:C:intergenic:TH	chr1	151843635	151843635	T	C	intergenic	THEM5,THEM	dist=17462;d	.	.	A	G	-	-	0.5	9,0,9,0	chr1,151843	Cytosol	A > G
chr1:151843689:T:C:intergenic:TH	chr1	151843689	151843689	T	C	intergenic	THEM5,THEM	dist=17516;d	.	.	A	G	-	-	0.11904762	37,0,5,0	chr1,151843	Cytosol	A > G
chr1:151843692:T:C:intergenic:TH	chr1	151843692	151843692	T	C	intergenic	THEM5,THEM	dist=17519;d	.	.	A	G	-	-	0.30232558	30,0,13,0	chr1,151843	Cytosol	A > G
chr1:151843718:T:C:intergenic:TH	chr1	151843718	151843718	T	C	intergenic	THEM5,THEM	dist=17545;d	.	.	A	G	-	-	0.35294118	22,0,12,0	chr1,151843	Cytosol	A > G
chr1:151843719:T:C:intergenic:TH	chr1	151843719	151843719	T	C	intergenic	THEM5,THEM	dist=17546;d	.	.	A	G	-	-	0.12195122	36,0,5,0	chr1,151843	Cytosol	A > G
chr1:151843725:T:C:intergenic:TH	chr1	151843725	151843725	T	C	intergenic	THEM5,THEM	dist=17552;d	.	.	A	G	-	-	0.43478261	26,0,20,0	chr1,151843	Cytosol	A > G
chr1:151843749:T:C:intergenic:TH	chr1	151843749	151843749	T	C	intergenic	THEM5,THEM	dist=17576;d	.	.	A	G	-	-	0.07692308	36,0,3,0	chr1,151843	Cytosol	A > G
chr1:151846936:T:C:UTR3:THEM4	chr1	151846936	151846936	T	C	UTR3	THEM4	ENST000003	.	.	A	G	-	-	0.01136364	174,0,2,0	chr1,151846	Cytosol	A > G
chr1:151846947:T:C:UTR3:THEM4	chr1	151846947	151846947	T	C	UTR3	THEM4	ENST000003	.	.	A	G	-	-	0.07586207	134,0,11,0	chr1,151846	Cytosol	A > G
chr1:151846948:T:C:UTR3:THEM4	chr1	151846948	151846948	T	C	UTR3	THEM4	ENST000003	.	.	A	G	-	-	0.0234375	125,0,3,0	chr1,151846	Cytosol	A > G
chr1:151846962:T:C:UTR3:THEM4	chr1	151846962	151846962	T	C	UTR3	THEM4	ENST000003	.	.	A	G	-	-	0.03883495	99,0,4,0	chr1,151846	Cytosol	A > G
chr1:151846966:T:C:UTR3:THEM4	chr1	151846966	151846966	T	C	UTR3	THEM4	ENST000003	.	.	A	G	-	-	0.03539823	109,0,4,0	chr1,151846	Cytosol	A > G
chr1:151846979:T:C:UTR3:THEM4	chr1	151846979	151846979	T	C	UTR3	THEM4	ENST000003	.	.	A	G	-	-	0.09230769	118,0,12,0	chr1,151846	Cytosol	A > G
chr1:151847110:T:C:UTR3:THEM4	chr1	151847110	151847110	T	C	UTR3	THEM4	ENST000003	.	.	A	G	-	-	0.17821782	83,0,18,0	chr1,151847	Cytosol	A > G
chr1:153578687:T:C:downstream:	chr1	153578687	153578687	T	C	downstream	S100A16	.	.	.	A	G	-		0.6	2,0,3,0	chr1,153578	Cytosol	A > G
chr1:153578690:T:C:downstream:	chr1	153578690	153578690	T	C	downstream	S100A16	.	.	.	A	G	-		0.4	3,0,2,0	chr1,153578	Cytosol	A > G
chr1:153937650:A:G:intronic:SLC3	chr1	153937650	153937650	A	G	intronic	SLC39A1	.	.	.	A	G	+	-	0.42857143	4,0,3,0	chr1,153937	Cytosol	A > G
chr1:153937809:A:G:intronic:SLC3	chr1	153937809	153937809	A	G	intronic	SLC39A1	.	.	.	A	G	+	-	0.6	2,0,3,0	chr1,153937	Cytosol	A > G
chr1:153938085:A:G:intronic:SLC3	chr1	153938085	153938085	A	G	intronic	SLC39A1	.	.	.	A	G	+	-	0.33333333	6,0,3,0	chr1,153938	Cytosol	A > G
chr1:153938139:A:G:intronic:SLC3	chr1	153938139	153938139	A	G	intronic	SLC39A1	.	.	.	A	G	+	-	0.625	3,0,5,0	chr1,153938	Cytosol	A > G
chr1:153938166:A:G:intronic:SLC3	chr1	153938166	153938166	A	G	intronic	SLC39A1	.	.	.	A	G	+	-	0.4	3,0,2,0	chr1,153938	Cytosol	A > G
chr1:153938315:A:G:intronic:SLC3	chr1	153938315	153938315	A	G	intronic	SLC39A1	.	.	.	A	G	+	-	0.69230769	4,0,9,0	chr1,153938	Cytosol	A > G
chr1:153938325:A:G:intronic:SLC3	chr1	153938325	153938325	A	G	intronic	SLC39A1	.	.	.	A	G	+	-	0.5	5,0,5,0	chr1,153938	Cytosol	A > G
chr1:155696517:A:G:intronic:DAP	chr1	155696517	155696517	A	G	intronic	DAP3,MSTO1	.	.	.	A	G	+	-+	0.33333333	4,0,2,0	chr1,155696	Cytosol	A > G
chr1:155697186:A:G:intronic:DAP	chr1	155697186	155697186	A	G	intronic	DAP3,MSTO1	.	.	.	A	G	+	-+	0.4	3,0,2,0	chr1,155697	Cytosol	A > G
chr1:155720655:A:G:intronic:GON	chr1	155720655	155720655	A	G	intronic	GON4L	.	.	.	A	G	+	+-	0.8	2,0,8,0	chr1,155720	Cytosol	A > G
chr1:155720712:A:G:intronic:GON	chr1	155720712	155720712	A	G	intronic	GON4L	.	.	.	A	G	+	-	0.69230769	4,0,9,0	chr1,155720	Cytosol	A > G
chr1:155720738:A:G:intronic:GON	chr1	155720738	155720738	A	G	intronic	GON4L	.	.	.	A	G	+	-	1	0,0,13,0	chr1,155720	Cytosol	A > G

chr1:155720802:A:G:intronic:GON	chr1	155720802	155720802	A	G	intronic	GON4L	.	.	.	A	G	+	-	0.47619048	11,0,10,0	chr1,155720	Cytosol	A > G
chr1:155721108:A:G:intronic:GON	chr1	155721108	155721108	A	G	intronic	GON4L	.	.	.	A	G	+	-	0.33333333	6,0,3,0	chr1,155721	Cytosol	A > G
chr1:155721135:A:G:intronic:GON	chr1	155721135	155721135	A	G	intronic	GON4L	.	.	.	A	G	+	-	0.18181818	9,0,2,0	chr1,155721	Cytosol	A > G
chr1:159996496:T:C:ncRNA_exoni	chr1	159996496	159996496	T	C	ncRNA_exon	RP11-226L15	.	.	.	A	G	-	-	0.07142857	26,0,2,0	chr1,159996	Cytosol	A > G
chr1:159996535:T:C:ncRNA_exoni	chr1	159996535	159996535	T	C	ncRNA_exon	RP11-226L15	.	.	.	A	G	-	-	0.80952381	4,0,17,0	chr1,159996	Cytosol	A > G
chr1:159996536:T:C:ncRNA_exoni	chr1	159996536	159996536	T	C	ncRNA_exon	RP11-226L15	.	.	.	A	G	-	-	0.77272727	5,0,17,0	chr1,159996	Cytosol	A > G
chr1:159996575:T:C:ncRNA_exoni	chr1	159996575	159996575	T	C	ncRNA_exon	RP11-226L15	.	.	.	A	G	-	-	1	0,0,11,0	chr1,159996	Cytosol	A > G
chr1:159996696:T:C:upstream;do	chr1	159996696	159996696	T	C	upstream;do	RP11-226L15	.	.	.	A	G	-		0.875	3,0,21,0	chr1,159996	Cytosol	A > G
chr1:160287358:C:T:intronic;intro	chr1	160287358	160287358	C	T	intronic;intro	COPA	.	.	.	C	T	+	-+	1	0,0,0,124	chr1,160287	Cytosol	C > T
chr1:160287358:C:T:intronic;intro	chr1	160287358	160287358	C	T	intronic;intro	COPA	.	.	.	G	A	-	-+	1	5,0,0,0	chr1,160287	Cytosol	G > A
chr1:160287374:T:C:intronic;intro	chr1	160287374	160287374	T	C	intronic;intro	COPA	.	.	.	A	G	-	-+	1	0,0,6,0	chr1,160287	Cytosol	A > G
chr1:160287374:T:C:intronic;intro	chr1	160287374	160287374	T	C	intronic;intro	COPA	.	.	.	T	C	+	-+	1	0,164,0,0	chr1,160287	Cytosol	T > C
chr1:160965287:T:C:UTR3:F11R	chr1	160965287	160965287	T	C	UTR3	F11R	ENST000003	.	.	A	G	-	-	0.0625	30,0,2,0	chr1,160965	Cytosol	A > G
chr1:160965314:T:C:UTR3:F11R	chr1	160965314	160965314	T	C	UTR3	F11R	ENST000003	.	.	A	G	-	-	0.19047619	17,0,4,0	chr1,160965	Cytosol	A > G
chr1:160965379:T:C:UTR3:F11R	chr1	160965379	160965379	T	C	UTR3	F11R	ENST000003	.	.	A	G	-	-	0.54285714	16,0,19,0	chr1,160965	Cytosol	A > G
chr1:160965398:T:C:UTR3:F11R	chr1	160965398	160965398	T	C	UTR3	F11R	ENST000003	.	.	A	G	-	-	0.13333333	26,0,4,0	chr1,160965	Cytosol	A > G
chr1:160965404:T:C:UTR3:F11R	chr1	160965404	160965404	T	C	UTR3	F11R	ENST000003	.	.	A	G	-	-	0.38461538	16,0,10,0	chr1,160965	Cytosol	A > G
chr1:160965420:T:C:UTR3:F11R	chr1	160965420	160965420	T	C	UTR3	F11R	ENST000003	.	.	A	G	-	-	0.20833333	19,0,5,0	chr1,160965	Cytosol	A > G
chr1:160966301:T:C:UTR3:F11R	chr1	160966301	160966301	T	C	UTR3	F11R	ENST000003	.	.	A	G	-	-	0.02475248	197,0,5,0	chr1,160966	Cytosol	A > G
chr1:160966351:T:C:UTR3:F11R	chr1	160966351	160966351	T	C	UTR3	F11R	ENST000003	.	.	A	G	-	-	0.34013605	97,0,50,0	chr1,160966	Cytosol	A > G
chr1:160966352:T:C:UTR3:F11R	chr1	160966352	160966352	T	C	UTR3	F11R	ENST000003	.	.	A	G	-	-	0.38255034	92,0,57,0	chr1,160966	Cytosol	A > G
chr1:160966353:T:C:UTR3:F11R	chr1	160966353	160966353	T	C	UTR3	F11R	ENST000003	.	.	A	G	-	-	0.25490196	114,0,39,0	chr1,160966	Cytosol	A > G
chr1:160966373:T:C:UTR3:F11R	chr1	160966373	160966373	T	C	UTR3	F11R	ENST000003	.	.	A	G	-	-	0.02752294	106,0,3,0	chr1,160966	Cytosol	A > G
chr1:160966384:T:C:UTR3:F11R	chr1	160966384	160966384	T	C	UTR3	F11R	ENST000003	.	.	A	G	-	-	0.02150538	91,0,2,0	chr1,160966	Cytosol	A > G
chr1:160966393:T:C:UTR3:F11R	chr1	160966393	160966393	T	C	UTR3	F11R	ENST000003	.	.	A	G	-	-	0.07042254	66,0,5,0	chr1,160966	Cytosol	A > G
chr1:160966408:T:C:UTR3:F11R	chr1	160966408	160966408	T	C	UTR3	F11R	ENST000003	.	.	A	G	-	-	0.03508772	55,0,2,0	chr1,160966	Cytosol	A > G
chr1:160966418:T:C:UTR3:F11R	chr1	160966418	160966418	T	C	UTR3	F11R	ENST000003	.	.	A	G	-	-	0.11320755	47,0,6,0	chr1,160966	Cytosol	A > G
chr1:160966434:T:C:UTR3:F11R	chr1	160966434	160966434	T	C	UTR3	F11R	ENST000003	.	.	A	G	-	-	0.83606557	10,0,51,0	chr1,160966	Cytosol	A > G
chr1:160967816:T:C:UTR3:F11R	chr1	160967816	160967816	T	C	UTR3	F11R	ENST000003	.	.	A	G	-	-	0.58823529	56,0,80,0	chr1,160967	Cytosol	A > G
chr1:160967817:T:C:UTR3:F11R	chr1	160967817	160967817	T	C	UTR3	F11R	ENST000003	.	.	A	G	-	-	0.21374046	103,0,28,0	chr1,160967	Cytosol	A > G
chr1:160967833:T:C:UTR3:F11R	chr1	160967833	160967833	T	C	UTR3	F11R	ENST000003	.	.	A	G	-	-	0.69026549	35,0,78,0	chr1,160967	Cytosol	A > G
chr1:160967839:T:C:UTR3:F11R	chr1	160967839	160967839	T	C	UTR3	F11R	ENST000003	.	.	A	G	-	-	0.03448276	112,0,4,0	chr1,160967	Cytosol	A > G
chr1:160967843:T:C:UTR3:F11R	chr1	160967843	160967843	T	C	UTR3	F11R	ENST000003	.	.	A	G	-	-	0.05042017	113,0,6,0	chr1,160967	Cytosol	A > G
chr1:160967848:T:C:UTR3:F11R	chr1	160967848	160967848	T	C	UTR3	F11R	ENST000003	.	.	A	G	-	-	0.13970588	117,0,19,0	chr1,160967	Cytosol	A > G
chr1:160967855:T:C:UTR3:F11R	chr1	160967855	160967855	T	C	UTR3	F11R	ENST000003	.	.	A	G	-	-	0.20769231	102,1,27,0	chr1,160967	Cytosol	A > G
chr1:160967856:T:C:UTR3:F11R	chr1	160967856	160967856	T	C	UTR3	F11R	ENST000003	.	.	A	G	-	-	0.34090909	87,0,45,0	chr1,160967	Cytosol	A > G
chr1:160967857:T:C:UTR3:F11R	chr1	160967857	160967857	T	C	UTR3	F11R	ENST000003	.	.	A	G	-	-	0.57723577	52,0,71,0	chr1,160967	Cytosol	A > G
chr1:160967860:T:C:UTR3:F11R	chr1	160967860	160967860	T	C	UTR3	F11R	ENST000003	.	.	A	G	-	-	0.07407407	125,0,10,0	chr1,160967	Cytosol	A > G
chr1:160967890:T:C:UTR3:F11R	chr1	160967890	160967890	T	C	UTR3	F11R	ENST000003	.	.	A	G	-	-	0.45517241	79,0,66,0	chr1,160967	Cytosol	A > G
chr1:160967891:T:C:UTR3:F11R	chr1	160967891	160967891	T	C	UTR3	F11R	ENST000003	.	.	A	G	-	-	0.36986301	92,0,54,0	chr1,160967	Cytosol	A > G
chr1:160967896:T:C:UTR3:F11R	chr1	160967896	160967896	T	C	UTR3	F11R	ENST000003	.	.	A	G	-	-	0.07857143	129,0,11,0	chr1,160967	Cytosol	A > G
chr1:160967899:T:C:UTR3:F11R	chr1	160967899	160967899	T	C	UTR3	F11R	ENST000003	.	.	A	G	-	-	0.36054422	94,0,53,0	chr1,160967	Cytosol	A > G
chr1:160967918:T:C:UTR3:F11R	chr1	160967918	160967918	T	C	UTR3	F11R	ENST000003	.	.	A	G	-	-	0.02013423	146,0,3,0	chr1,160967	Cytosol	A > G
chr1:160967938:T:C:UTR3:F11R	chr1	160967938	160967938	T	C	UTR3	F11R	ENST000003	.	.	A	G	-	-	0.06382979	176,0,12,0	chr1,160967	Cytosol	A > G
chr1:161336664:A:G:intronic:C1or	chr1	161336664	161336664	A	G	intronic	C1orf192	.	.	.	A	G	+	-	0.03333333	58,0,2,0	chr1,161336	Cytosol	A > G
chr1:161336681:A:G:intronic:C1or	chr1	161336681	161336681	A	G	intronic	C1orf192	.	.	.	A	G	+	-	0.04347826	44,0,2,0	chr1,161336	Cytosol	A > G
chr1:161336765:A:G:intronic:C1or	chr1	161336765	161336765	A	G	intronic	C1orf192	.	.	.	A	G	+	-	0.05357143	53,0,3,0	chr1,161336	Cytosol	A > G
chr1:161336767:A:G:intronic:C1or	chr1	161336767	161336767	A	G	intronic	C1orf192	.	.	.	A	G	+	-	0.24074074	41,0,13,0	chr1,161336	Cytosol	A > G
chr1:161336785:A:G:intronic:C1or	chr1	161336785	161336785	A	G	intronic	C1orf192	.	.	.	A	G	+	-	0.03225806	60,0,2,0	chr1,161336	Cytosol	A > G
chr1:161336817:A:G:intronic:C1or	chr1	161336817	161336817	A	G	intronic	C1orf192	.	.	.	A	G	+	-	0.265625	47,0,17,0	chr1,161336	Cytosol	A > G
chr1:161338900:A:G:ncRNA_exoni	chr1	161338900	161338900	A	G	ncRNA_exon	RP11-122G1	.	.	.	A	G	+	+	0.15	17,0,3,0	chr1,161338	Cytosol	A > G
chr1:161339929:A:G:ncRNA_intro	chr1	161339929	161339929	A	G	ncRNA_intro	RP11-122G1	.	.	.	A	G	+	+	0.5	3,0,3,0	chr1,161339	Cytosol	A > G
chr1:161340722:A:G:ncRNA_intro	chr1	161340722	161340722	A	G	ncRNA_intro	RP11-122G1	.	.	.	A	G	+	+	0.22222222	7,0,2,0	chr1,161340	Cytosol	A > G
chr1:161340735:A:G:ncRNA_intro	chr1	161340735	161340735	A	G	ncRNA_intro	RP11-122G1	.	.	.	A	G	+	+	0.4	6,0,4,0	chr1,161340	Cytosol	A > G
chr1:161341153:A:G:ncRNA_exoni	chr1	161341153	161341153	A	G	ncRNA_exon	RP11-122G1	.	.	.	A	G	+	+	0.5	5,0,5,0	chr1,161341	Cytosol	A > G
chr1:161341168:A:G:ncRNA_exoni	chr1	161341168	161341168	A	G	ncRNA_exon	RP11-122G1	.	.	.	A	G	+	+	0.18181818	9,0,2,0	chr1,161341	Cytosol	A > G
chr1:161725747:A:G:intronic:DUS	chr1	161725747	161725747	A	G	intronic	DUSP12	.	.	.	A	G	+	+	0.28571429	5,0,2,0	chr1,161725	Cytosol	A > G
chr1:168213435:A:G:UTR3:SFT2D2	chr1	168213435	168213435	A	G	UTR3	SFT2D2	ENST000002	.	.	A	G	+	+	0.21428571	11,0,3,0	chr1,168213	Cytosol	A > G
chr1:168213651:A:G:UTR3:SFT2D2	chr1	168213651	168213651	A	G	UTR3	SFT2D2	ENST000002	.	.	A	G	+	+	0.06666667	28,0,2,0	chr1,168213	Cytosol	A > G
chr1:168213679:A:G:UTR3:SFT2D2	chr1	168213679	168213679	A	G	UTR3	SFT2D2	ENST000002	.	.	A	G	+	+	0.23809524	16,0,5,0	chr1,168213	Cytosol	A > G
chr1:168218494:A:G:UTR3:SFT2D2	chr1	168218494	168218494	A	G	UTR3	SFT2D2	ENST000002	.	.	A	G	+	+	0.18461538	53,0,12,0	chr1,168218	Cytosol	A > G
chr1:168218522:A:G:UTR3:SFT2D2	chr1	168218522	168218522	A	G	UTR3	SFT2D2	ENST000002	.	.	A	G	+	+	0.26	37,0,13,0	chr1,168218	Cytosol	A > G
chr1:168218543:A:G:UTR3:SFT2D2	chr1	168218543	168218543	A	G	UTR3	SFT2D2	ENST000002	.	.	A	G	+	+	0.35294118	22,0,12,0	chr1,168218	Cytosol	A > G
chr1:168218573:A:G:UTR3:SFT2D2	chr1	168218573	168218573	A	G	UTR3	SFT2D2	ENST000002	.	.	A	G	+	+	0.22222222	35,0,10,0	chr1,168218	Cytosol	A > G

chr1:168218574:A:G:UTR3:SFT2D2	chr1	168218574	168218574	A	G	UTR3	SFT2D2	ENST000002	.	.	A	G	+	+	0.0952381	38,0,4,0	chr1,168218	Cytosol	A > G
chr1:168218600:A:G:UTR3:SFT2D2	chr1	168218600	168218600	A	G	UTR3	SFT2D2	ENST000002	.	.	A	G	+	+	0.12820513	34,0,5,0	chr1,168218	Cytosol	A > G
chr1:168218639:A:G:UTR3:SFT2D2	chr1	168218639	168218639	A	G	UTR3	SFT2D2	ENST000002	.	.	A	G	+	+	0.27777778	13,0,5,0	chr1,168218	Cytosol	A > G
chr1:168218660:A:G:UTR3:SFT2D2	chr1	168218660	168218660	A	G	UTR3	SFT2D2	ENST000002	.	.	A	G	+	+	0.33333333	6,0,3,0	chr1,168218	Cytosol	A > G
chr1:168218736:A:G:UTR3:SFT2D2	chr1	168218736	168218736	A	G	UTR3	SFT2D2	ENST000002	.	.	A	G	+	+	0.55	9,0,11,0	chr1,168218	Cytosol	A > G
chr1:168220286:A:G:UTR3:SFT2D2	chr1	168220286	168220286	A	G	UTR3	SFT2D2	ENST000002	.	.	A	G	+	+	0.26530612	36,0,13,0	chr1,168220	Cytosol	A > G
chr1:168220332:A:G:UTR3:SFT2D2	chr1	168220332	168220332	A	G	UTR3	SFT2D2	ENST000002	.	.	A	G	+	+	0.53571429	13,0,15,0	chr1,168220	Cytosol	A > G
chr1:168220340:A:G:UTR3:SFT2D2	chr1	168220340	168220340	A	G	UTR3	SFT2D2	ENST000002	.	.	A	G	+	+	0.56666667	13,0,17,0	chr1,168220	Cytosol	A > G
chr1:168220341:A:G:UTR3:SFT2D2	chr1	168220341	168220341	A	G	UTR3	SFT2D2	ENST000002	.	.	A	G	+	+	0.19354839	25,0,6,0	chr1,168220	Cytosol	A > G
chr1:168220363:A:G:UTR3:SFT2D2	chr1	168220363	168220363	A	G	UTR3	SFT2D2	ENST000002	.	.	A	G	+	+	0.34210526	25,0,13,0	chr1,168220	Cytosol	A > G
chr1:168220371:A:G:UTR3:SFT2D2	chr1	168220371	168220371	A	G	UTR3	SFT2D2	ENST000002	.	.	A	G	+	+	0.28571429	30,0,12,0	chr1,168220	Cytosol	A > G
chr1:168220407:A:G:UTR3:SFT2D2	chr1	168220407	168220407	A	G	UTR3	SFT2D2	ENST000002	.	.	A	G	+	+	0.66666667	19,0,38,0	chr1,168220	Cytosol	A > G
chr1:168220412:A:G:UTR3:SFT2D2	chr1	168220412	168220412	A	G	UTR3	SFT2D2	ENST000002	.	.	A	G	+	+	0.08	69,0,6,0	chr1,168220	Cytosol	A > G
chr1:168220427:A:G:UTR3:SFT2D2	chr1	168220427	168220427	A	G	UTR3	SFT2D2	ENST000002	.	.	A	G	+	+	0.32941176	57,0,28,0	chr1,168220	Cytosol	A > G
chr1:168220474:A:G:UTR3:SFT2D2	chr1	168220474	168220474	A	G	UTR3	SFT2D2	ENST000002	.	.	A	G	+	+	0.0952381	76,0,8,0	chr1,168220	Cytosol	A > G
chr1:168220475:A:G:UTR3:SFT2D2	chr1	168220475	168220475	A	G	UTR3	SFT2D2	ENST000002	.	.	A	G	+	+	0.25301205	62,0,21,0	chr1,168220	Cytosol	A > G
chr1:168220479:A:G:UTR3:SFT2D2	chr1	168220479	168220479	A	G	UTR3	SFT2D2	ENST000002	.	.	A	G	+	+	0.04545455	84,0,4,0	chr1,168220	Cytosol	A > G
chr1:168220489:A:G:UTR3:SFT2D2	chr1	168220489	168220489	A	G	UTR3	SFT2D2	ENST000002	.	.	A	G	+	+	0.36	48,0,27,0	chr1,168220	Cytosol	A > G
chr1:168220498:A:G:UTR3:SFT2D2	chr1	168220498	168220498	A	G	UTR3	SFT2D2	ENST000002	.	.	A	G	+	+	0.03076923	63,0,2,0	chr1,168220	Cytosol	A > G
chr1:168220529:A:G:UTR3:SFT2D2	chr1	168220529	168220529	A	G	UTR3	SFT2D2	ENST000002	.	.	A	G	+	+	0.10769231	58,0,7,0	chr1,168220	Cytosol	A > G
chr1:168220556:A:G:UTR3:SFT2D2	chr1	168220556	168220556	A	G	UTR3	SFT2D2	ENST000002	.	.	A	G	+	+	0.02105263	93,0,2,0	chr1,168220	Cytosol	A > G
chr1:168220562:T:A:UTR3:SFT2D2	chr1	168220562	168220562	T	A	UTR3	SFT2D2	ENST000002	.	.	T	A	+	+	0.0212766	2,0,0,92	chr1,168220	Cytosol	T > A
chr1:173766199:T:C:intergenic:RN	chr1	173766199	173766199	T	C	intergenic	RN7SKP160,	dist=5174;dis	.	.	A	G	-		0.15	34,0,6,0	chr1,173766	Cytosol	A > G
chr1:173766201:T:C:intergenic:RN	chr1	173766201	173766201	T	C	intergenic	RN7SKP160,	dist=5176;dis	.	.	A	G	-		0.08333333	33,0,3,0	chr1,173766	Cytosol	A > G
chr1:173766243:T:C:intergenic:RN	chr1	173766243	173766243	T	C	intergenic	RN7SKP160,	dist=5218;dis	.	.	A	G	-		0.22222222	35,0,10,0	chr1,173766	Cytosol	A > G
chr1:173766255:T:C:intergenic:RN	chr1	173766255	173766255	T	C	intergenic	RN7SKP160,	dist=5230;dis	.	.	A	G	-		0.2173913	36,0,10,0	chr1,173766	Cytosol	A > G
chr1:173766258:T:C:intergenic:RN	chr1	173766258	173766258	T	C	intergenic	RN7SKP160,	dist=5233;dis	.	.	A	G	-		0.10869565	41,0,5,0	chr1,173766	Cytosol	A > G
chr1:173766281:T:C:intergenic:RN	chr1	173766281	173766281	T	C	intergenic	RN7SKP160,	dist=5256;dis	.	.	A	G	-		0.04545455	42,0,2,0	chr1,173766	Cytosol	A > G
chr1:173766284:T:C:intergenic:RN	chr1	173766284	173766284	T	C	intergenic	RN7SKP160,	dist=5259;dis	.	.	A	G	-		0.10416667	43,0,5,0	chr1,173766	Cytosol	A > G
chr1:173766320:T:C:intergenic:RN	chr1	173766320	173766320	T	C	intergenic	RN7SKP160,	dist=5295;dis	.	.	A	G	-		0.19444444	29,0,7,0	chr1,173766	Cytosol	A > G
chr1:173766336:T:C:intergenic:RN	chr1	173766336	173766336	T	C	intergenic	RN7SKP160,	dist=5311;dis	.	.	A	G	-		0.21052632	30,0,8,0	chr1,173766	Cytosol	A > G
chr1:173769138:T:C:UTR3:CENPL	chr1	173769138	173769138	T	C	UTR3	CENPL	ENST000003	.	.	A	G	-	-	0.05035971	132,0,7,0	chr1,173769	Cytosol	A > G
chr1:173769162:T:C:UTR3:CENPL	chr1	173769162	173769162	T	C	UTR3	CENPL	ENST000003	.	.	A	G	-	-	0.03448276	112,0,4,0	chr1,173769	Cytosol	A > G
chr1:173833215:T:C:ncRNA_exoni	chr1	173833215	173833215	T	C	ncRNA_exon	GAS5;GAS5	ENST000004	.	.	A	G	-	-	0.02739726	71,0,2,0	chr1,173833	Cytosol	A > G
chr1:179042482:A:G:UTR3:FAM20	chr1	179042482	179042482	A	G	UTR3	FAM20B	ENST000002	.	.	A	G	+	+	0.4516129	34,0,28,0	chr1,179042	Cytosol	A > G
chr1:179042544:A:G:UTR3:FAM20	chr1	179042544	179042544	A	G	UTR3	FAM20B	ENST000002	.	.	A	G	+	+	0.62068966	22,0,36,0	chr1,179042	Cytosol	A > G
chr1:179042555:A:G:UTR3:FAM20	chr1	179042555	179042555	A	G	UTR3	FAM20B	ENST000002	.	.	A	G	+	+	0.12	44,0,6,0	chr1,179042	Cytosol	A > G
chr1:179042580:A:G:UTR3:FAM20	chr1	179042580	179042580	A	G	UTR3	FAM20B	ENST000002	.	.	A	G	+	+	0.88461538	3,0,23,0	chr1,179042	Cytosol	A > G
chr1:179042590:A:G:UTR3:FAM20	chr1	179042590	179042590	A	G	UTR3	FAM20B	ENST000002	.	.	A	G	+	+	0.43333333	17,0,13,0	chr1,179042	Cytosol	A > G
chr1:179042616:A:G:UTR3:FAM20	chr1	179042616	179042616	A	G	UTR3	FAM20B	ENST000002	.	.	A	G	+	+	0.87096774	4,0,27,0	chr1,179042	Cytosol	A > G
chr1:179042617:A:G:UTR3:FAM20	chr1	179042617	179042617	A	G	UTR3	FAM20B	ENST000002	.	.	A	G	+	+	0.29411765	24,0,10,0	chr1,179042	Cytosol	A > G
chr1:179042667:A:G:UTR3:FAM20	chr1	179042667	179042667	A	G	UTR3	FAM20B	ENST000002	.	.	A	G	+	+	0.03333333	58,0,2,0	chr1,179042	Cytosol	A > G
chr1:179042697:A:G:UTR3:FAM20	chr1	179042697	179042697	A	G	UTR3	FAM20B	ENST000002	.	.	A	G	+	+	0.55932203	26,0,33,0	chr1,179042	Cytosol	A > G
chr1:179042701:A:G:UTR3:FAM20	chr1	179042701	179042701	A	G	UTR3	FAM20B	ENST000002	.	.	A	G	+	+	0.69491525	18,0,41,0	chr1,179042	Cytosol	A > G
chr1:179044092:A:G:UTR3:FAM20	chr1	179044092	179044092	A	G	UTR3	FAM20B	ENST000002	.	.	A	G	+	+	0.21428571	77,0,21,0	chr1,179044	Cytosol	A > G
chr1:179044096:A:G:UTR3:FAM20	chr1	179044096	179044096	A	G	UTR3	FAM20B	ENST000002	.	.	A	G	+	+	0.03061224	95,0,3,0	chr1,179044	Cytosol	A > G
chr1:179044100:A:G:UTR3:FAM20	chr1	179044100	179044100	A	G	UTR3	FAM20B	ENST000002	.	.	A	G	+	+	0.58333333	40,0,56,0	chr1,179044	Cytosol	A > G
chr1:179044105:A:G:UTR3:FAM20	chr1	179044105	179044105	A	G	UTR3	FAM20B	ENST000002	.	.	A	G	+	+	0.16129032	78,0,15,0	chr1,179044	Cytosol	A > G
chr1:179044110:A:G:UTR3:FAM20	chr1	179044110	179044110	A	G	UTR3	FAM20B	ENST000002	.	.	A	G	+	+	0.29032258	66,0,27,0	chr1,179044	Cytosol	A > G
chr1:179044175:A:G:UTR3:FAM20	chr1	179044175	179044175	A	G	UTR3	FAM20B	ENST000002	.	.	A	G	+	+	0.22916667	37,0,11,0	chr1,179044	Cytosol	A > G
chr1:179044176:A:G:UTR3:FAM20	chr1	179044176	179044176	A	G	UTR3	FAM20B	ENST000002	.	.	A	G	+	+	0.14285714	42,0,7,0	chr1,179044	Cytosol	A > G
chr1:179044180:A:G:UTR3:FAM20	chr1	179044180	179044180	A	G	UTR3	FAM20B	ENST000002	.	.	A	G	+	+	0.9	4,0,36,0	chr1,179044	Cytosol	A > G
chr1:179044181:A:G:UTR3:FAM20	chr1	179044181	179044181	A	G	UTR3	FAM20B	ENST000002	.	.	A	G	+	+	0.19512195	33,0,8,0	chr1,179044	Cytosol	A > G
chr1:179044196:A:G:UTR3:FAM20	chr1	179044196	179044196	A	G	UTR3	FAM20B	ENST000002	.	.	A	G	+	+	0.40540541	22,0,15,0	chr1,179044	Cytosol	A > G
chr1:179044206:A:G:UTR3:FAM20	chr1	179044206	179044206	A	G	UTR3	FAM20B	ENST000002	.	.	A	G	+	+	0.78947368	8,0,30,0	chr1,179044	Cytosol	A > G
chr1:179044212:A:G:UTR3:FAM20	chr1	179044212	179044212	A	G	UTR3	FAM20B	ENST000002	.	.	A	G	+	+	0.27777778	26,0,10,0	chr1,179044	Cytosol	A > G
chr1:179044216:A:G:UTR3:FAM20	chr1	179044216	179044216	A	G	UTR3	FAM20B	ENST000002	.	.	A	G	+	+	0.65	14,0,26,0	chr1,179044	Cytosol	A > G
chr1:179044239:A:G:UTR3:FAM20	chr1	179044239	179044239	A	G	UTR3	FAM20B	ENST000002	.	.	A	G	+	+	0.13333333	26,0,4,0	chr1,179044	Cytosol	A > G
chr1:179044242:A:G:UTR3:FAM20	chr1	179044242	179044242	A	G	UTR3	FAM20B	ENST000002	.	.	A	G	+	+	0.71875	9,0,23,0	chr1,179044	Cytosol	A > G
chr1:179044252:A:G:UTR3:FAM20	chr1	179044252	179044252	A	G	UTR3	FAM20B	ENST000002	.	.	A	G	+	+	0.5	19,0,19,0	chr1,179044	Cytosol	A > G
chr1:179044279:A:G:UTR3:FAM20	chr1	179044279	179044279	A	G	UTR3	FAM20B	ENST000002	.	.	A	G	+	+	0.10416667	43,0,5,0	chr1,179044	Cytosol	A > G
chr1:179325957:A:G:UTR3:SOAT1	chr1	179325957	179325957	A	G	UTR3	SOAT1	ENST000003	.	.	A	G	+	+	0.0625	45,0,3,0	chr1,179325	Cytosol	A > G
chr1:179326003:A:G:UTR3:SOAT1	chr1	179326003	179326003	A	G	UTR3	SOAT1	ENST000003	.	.	A	G	+	+	0.17142857	29,0,6,0	chr1,179326	Cytosol	A > G
chr1:179326538:A:G:UTR3:SOAT1	chr1	179326538	179326538	A	G	UTR3	SOAT1	ENST000003	.	.	A	G	+	+	0.26315789	14,0,5,0	chr1,179326	Cytosol	A > G

chr1:179326588:A:G:UTR3:SOAT1	chr1	179326588	179326588	A	G	UTR3	SOAT1	ENST000003	.	.	A	G	+	+	0.05882353	32,0,2,0	chr1,179326	Cytosol	A > G
chr1:179326774:A:G:UTR3:SOAT1	chr1	179326774	179326774	A	G	UTR3	SOAT1	ENST000003	.	.	A	G	+	+	0.02985075	65,0,2,0	chr1,179326	Cytosol	A > G
chr1:179326847:A:G:UTR3:SOAT1	chr1	179326847	179326847	A	G	UTR3	SOAT1	ENST000003	.	.	A	G	+	+	0.25641026	29,0,10,0	chr1,179326	Cytosol	A > G
chr1:179326882:A:G:UTR3:SOAT1	chr1	179326882	179326882	A	G	UTR3	SOAT1	ENST000003	.	.	A	G	+	+	0.16666667	15,0,3,0	chr1,179326	Cytosol	A > G
chr1:179326908:A:G:UTR3:SOAT1	chr1	179326908	179326908	A	G	UTR3	SOAT1	ENST000003	.	.	A	G	+	+	0.65909091	15,0,29,0	chr1,179326	Cytosol	A > G
chr1:179326922:A:G:UTR3:SOAT1	chr1	179326922	179326922	A	G	UTR3	SOAT1	ENST000003	.	.	A	G	+	+	0.29787234	33,0,14,0	chr1,179326	Cytosol	A > G
chr1:179327052:A:G:UTR3:SOAT1	chr1	179327052	179327052	A	G	UTR3	SOAT1	ENST000003	.	.	A	G	+	+	0.02272727	86,0,2,0	chr1,179327	Cytosol	A > G
chr1:179829096:T:C:intronic:TOR1	chr1	179829096	179829096	T	C	intronic	TOR1AIP2	.	.	.	A	G	-	-	1	0,0,7,0	chr1,179829	Cytosol	A > G
chr1:179829495:T:C:intronic:TOR1	chr1	179829495	179829495	T	C	intronic	TOR1AIP2	.	.	.	A	G	-	-	1	0,0,12,0	chr1,179829	Cytosol	A > G
chr1:179829508:T:C:intronic:TOR1	chr1	179829508	179829508	T	C	intronic	TOR1AIP2	.	.	.	A	G	-	-	0.75	2,0,6,0	chr1,179829	Cytosol	A > G
chr1:179829509:T:C:intronic:TOR1	chr1	179829509	179829509	T	C	intronic	TOR1AIP2	.	.	.	A	G	-	-	0.25	6,0,2,0	chr1,179829	Cytosol	A > G
chr1:179829584:T:C:intronic:TOR1	chr1	179829584	179829584	T	C	intronic	TOR1AIP2	.	.	.	A	G	-	-	0.4	3,0,2,0	chr1,179829	Cytosol	A > G
chr1:184761123:T:C:UTR3:FAM12	chr1	184761123	184761123	T	C	UTR3	FAM129A	ENST000003	.	.	A	G	-	-	0.66666667	2,0,4,0	chr1,184761	Cytosol	A > G
chr1:184761124:T:C:UTR3:FAM12	chr1	184761124	184761124	T	C	UTR3	FAM129A	ENST000003	.	.	A	G	-	-	0.42857143	4,0,3,0	chr1,184761	Cytosol	A > G
chr1:184761125:T:C:UTR3:FAM12	chr1	184761125	184761125	T	C	UTR3	FAM129A	ENST000003	.	.	A	G	-	-	0.4	3,0,2,0	chr1,184761	Cytosol	A > G
chr1:184761162:T:C:UTR3:FAM12	chr1	184761162	184761162	T	C	UTR3	FAM129A	ENST000003	.	.	A	G	-	-	0.15384615	11,0,2,0	chr1,184761	Cytosol	A > G
chr1:184761274:T:C:UTR3:FAM12	chr1	184761274	184761274	T	C	UTR3	FAM129A	ENST000003	.	.	A	G	-	-	0.5	5,0,5,0	chr1,184761	Cytosol	A > G
chr1:184761307:T:C:UTR3:FAM12	chr1	184761307	184761307	T	C	UTR3	FAM129A	ENST000003	.	.	A	G	-	-	0.22222222	7,0,2,0	chr1,184761	Cytosol	A > G
chr1:184761312:T:C:UTR3:FAM12	chr1	184761312	184761312	T	C	UTR3	FAM129A	ENST000003	.	.	A	G	-	-	0.625	3,0,5,0	chr1,184761	Cytosol	A > G
chr1:184761350:T:C:UTR3:FAM12	chr1	184761350	184761350	T	C	UTR3	FAM129A	ENST000003	.	.	A	G	-	-	0.3	7,0,3,0	chr1,184761	Cytosol	A > G
chr1:184762498:T:C:UTR3:FAM12	chr1	184762498	184762498	T	C	UTR3	FAM129A	ENST000003	.	.	A	G	-	-	0.4	3,0,2,0	chr1,184762	Cytosol	A > G
chr1:184762676:T:C:UTR3:FAM12	chr1	184762676	184762676	T	C	UTR3	FAM129A	ENST000003	.	.	A	G	-	-	0.375	5,0,3,0	chr1,184762	Cytosol	A > G
chr1:201484784:A:G:ncRNA_intro	chr1	201484784	201484784	A	G	ncRNA_intro	RP11-134G8.	.	.	.	A	G	+	+	0.83333333	1,0,5,0	chr1,201484	Cytosol	A > G
chr1:201484908:A:G:ncRNA_intro	chr1	201484908	201484908	A	G	ncRNA_intro	RP11-134G8.	.	.	.	A	G	+	+	0.61538462	5,0,8,0	chr1,201484	Cytosol	A > G
chr1:201484973:A:G:ncRNA_intro	chr1	201484973	201484973	A	G	ncRNA_intro	RP11-134G8.	.	.	.	A	G	+	+	0.44444444	5,0,4,0	chr1,201484	Cytosol	A > G
chr1:201484979:A:G:ncRNA_intro	chr1	201484979	201484979	A	G	ncRNA_intro	RP11-134G8.	.	.	.	A	G	+	+	0.625	3,0,5,0	chr1,201484	Cytosol	A > G
chr1:201484980:A:G:ncRNA_intro	chr1	201484980	201484980	A	G	ncRNA_intro	RP11-134G8.	.	.	.	A	G	+	+	0.375	5,0,3,0	chr1,201484	Cytosol	A > G
chr1:201485039:A:G:ncRNA_intro	chr1	201485039	201485039	A	G	ncRNA_intro	RP11-134G8.	.	.	.	A	G	+	+	1	0,0,6,0	chr1,201485	Cytosol	A > G
chr1:201485049:A:G:ncRNA_intro	chr1	201485049	201485049	A	G	ncRNA_intro	RP11-134G8.	.	.	.	A	G	+	+	1	0,0,7,0	chr1,201485	Cytosol	A > G
chr1:201485056:A:G:ncRNA_intro	chr1	201485056	201485056	A	G	ncRNA_intro	RP11-134G8.	.	.	.	A	G	+	+	0.42857143	4,0,3,0	chr1,201485	Cytosol	A > G
chr1:201485075:A:G:ncRNA_intro	chr1	201485075	201485075	A	G	ncRNA_intro	RP11-134G8.	.	.	.	A	G	+	+	0.90909091	1,0,10,0	chr1,201485	Cytosol	A > G
chr1:201485111:A:G:ncRNA_intro	chr1	201485111	201485111	A	G	ncRNA_intro	RP11-134G8.	.	.	.	A	G	+	+	0.26666667	11,0,4,0	chr1,201485	Cytosol	A > G
chr1:201485114:A:G:ncRNA_intro	chr1	201485114	201485114	A	G	ncRNA_intro	RP11-134G8.	.	.	.	A	G	+	+	0.25	12,0,4,0	chr1,201485	Cytosol	A > G
chr1:201486312:A:G:ncRNA_intro	chr1	201486312	201486312	A	G	ncRNA_intro	RP11-134G8.	.	.	.	A	G	+	+	0.6	4,0,6,0	chr1,201486	Cytosol	A > G
chr1:201486379:A:G:ncRNA_intro	chr1	201486379	201486379	A	G	ncRNA_intro	RP11-134G8.	.	.	.	A	G	+	+	0.85714286	1,0,6,0	chr1,201486	Cytosol	A > G
chr1:201486380:A:G:ncRNA_intro	chr1	201486380	201486380	A	G	ncRNA_intro	RP11-134G8.	.	.	.	A	G	+	+	0.71428571	2,0,5,0	chr1,201486	Cytosol	A > G
chr1:201486586:A:G:ncRNA_intro	chr1	201486586	201486586	A	G	ncRNA_intro	RP11-134G8.	.	.	.	A	G	+	+	0.4	3,0,2,0	chr1,201486	Cytosol	A > G
chr1:201486720:A:G:ncRNA_intro	chr1	201486720	201486720	A	G	ncRNA_intro	RP11-134G8.	.	.	.	A	G	+	+	0.25	6,0,2,0	chr1,201486	Cytosol	A > G
chr1:201486900:A:G:ncRNA_intro	chr1	201486900	201486900	A	G	ncRNA_intro	RP11-134G8.	.	.	.	A	G	+	+	0.71428571	2,0,5,0	chr1,201486	Cytosol	A > G
chr1:201486905:A:G:ncRNA_intro	chr1	201486905	201486905	A	G	ncRNA_intro	RP11-134G8.	.	.	.	A	G	+	+	0.625	3,0,5,0	chr1,201486	Cytosol	A > G
chr1:201486908:A:G:ncRNA_intro	chr1	201486908	201486908	A	G	ncRNA_intro	RP11-134G8.	.	.	.	A	G	+	+	0.75	2,0,6,0	chr1,201486	Cytosol	A > G
chr1:201487041:A:G:ncRNA_intro	chr1	201487041	201487041	A	G	ncRNA_intro	RP11-134G8.	.	.	.	A	G	+	+	0.3	7,0,3,0	chr1,201487	Cytosol	A > G
chr1:201487042:A:G:ncRNA_intro	chr1	201487042	201487042	A	G	ncRNA_intro	RP11-134G8.	.	.	.	A	G	+	+	0.44444444	5,0,4,0	chr1,201487	Cytosol	A > G
chr1:201487087:A:G:ncRNA_intro	chr1	201487087	201487087	A	G	ncRNA_intro	RP11-134G8.	.	.	.	A	G	+	+	0.25	9,0,3,0	chr1,201487	Cytosol	A > G
chr1:201487457:A:G:ncRNA_intro	chr1	201487457	201487457	A	G	ncRNA_intro	RP11-134G8.	.	.	.	A	G	+	+	0.33333333	6,0,3,0	chr1,201487	Cytosol	A > G
chr1:201487970:A:G:ncRNA_exon	chr1	201487970	201487970	A	G	ncRNA_exon	RPS10P7	.	.	.	A	G	+	+	0.8	1,0,4,0	chr1,201487	Cytosol	A > G
chr1:201487999:A:G:ncRNA_exon	chr1	201487999	201487999	A	G	ncRNA_exon	RPS10P7	.	.	.	A	G	+	+	0.6	2,0,3,0	chr1,201487	Cytosol	A > G
chr1:201488170:A:G:ncRNA_exon	chr1	201488170	201488170	A	G	ncRNA_exon	RPS10P7	.	.	.	A	G	+	+	0.4	3,0,2,0	chr1,201488	Cytosol	A > G
chr1:201846835:A:G:UTR3:IPO9	chr1	201846835	201846835	A	G	UTR3	IPO9	ENST000003	.	.	A	G	+	+	0.04166667	46,0,2,0	chr1,201846	Cytosol	A > G
chr1:201964732:T:C:intronic:RNPE	chr1	201964732	201964732	T	C	intronic	RNPEP	.	.	.	A	G	-	+	0.4	3,0,2,0	chr1,201964	Cytosol	A > G
chr1:201964733:T:C:intronic:RNPE	chr1	201964733	201964733	T	C	intronic	RNPEP	.	.	.	A	G	-	+	0.6	2,0,3,0	chr1,201964	Cytosol	A > G
chr1:201967511:T:C:intronic:RNPE	chr1	201967511	201967511	T	C	intronic	RNPEP	.	.	.	A	G	-	+	0.10526316	17,0,2,0	chr1,201967	Cytosol	A > G
chr1:201967530:T:C:intronic:RNPE	chr1	201967530	201967530	T	C	intronic	RNPEP	.	.	.	A	G	-	+	0.13333333	13,0,2,0	chr1,201967	Cytosol	A > G
chr1:201967538:T:C:intronic:RNPE	chr1	201967538	201967538	T	C	intronic	RNPEP	.	.	.	A	G	-	+	0.25	12,0,4,0	chr1,201967	Cytosol	A > G
chr1:201967542:T:C:intronic:RNPE	chr1	201967542	201967542	T	C	intronic	RNPEP	.	.	.	A	G	-	+	0.13333333	13,0,2,0	chr1,201967	Cytosol	A > G
chr1:201967620:T:C:intronic:RNPE	chr1	201967620	201967620	T	C	intronic	RNPEP	.	.	.	A	G	-	+	0.22222222	7,0,2,0	chr1,201967	Cytosol	A > G
chr1:201968197:T:C:intronic:RNPE	chr1	201968197	201968197	T	C	intronic	RNPEP	.	.	.	A	G	-	+	0.25	6,0,2,0	chr1,201968	Cytosol	A > G
chr1:202554257:A:G:UTR3:PPP1R1	chr1	202554257	202554257	A	G	UTR3	PPP1R12B	ENST000006	.	.	A	G	+	+	0.19230769	21,0,5,0	chr1,202554	Cytosol	A > G
chr1:204371749:T:C:ncRNA_intror	chr1	204371749	204371749	T	C	ncRNA_intro	RP11-739N2	.	.	.	A	G	-	+	0.64705882	6,0,11,0	chr1,204371	Cytosol	A > G
chr1:204371769:T:C:ncRNA_intror	chr1	204371769	204371769	T	C	ncRNA_intro	RP11-739N2	.	.	.	A	G	-	+	0.36842105	12,0,7,0	chr1,204371	Cytosol	A > G
chr1:204372194:T:C:ncRNA_intror	chr1	204372194	204372194	T	C	ncRNA_intro	RP11-739N2	.	.	.	A	G	-	+	0.18181818	9,0,2,0	chr1,204372	Cytosol	A > G
chr1:204521159:A:G:UTR3:MDM4	chr1	204521159	204521159	A	G	UTR3	MDM4	ENST000003	.	.	A	G	+	+	0.25	6,0,2,0	chr1,204521	Cytosol	A > G
chr1:204521223:A:G:UTR3:MDM4	chr1	204521223	204521223	A	G	UTR3	MDM4	ENST000003	.	.	A	G	+	+	0.625	3,0,5,0	chr1,204521	Cytosol	A > G
chr1:204521254:A:G:UTR3:MDM4	chr1	204521254	204521254	A	G	UTR3	MDM4	ENST000003	.	.	A	G	+	+	0.8	1,0,4,0	chr1,204521	Cytosol	A > G

chr1:204521322:A:G:UTR3:MDM4	chr1	204521322	204521322	A	G	UTR3	MDM4	ENST000003	.	.	A	G	+	+	0.8	1,0,4,0	chr1,204521	Cytosol	A > G
chr1:204521391:A:G:UTR3:MDM4	chr1	204521391	204521391	A	G	UTR3	MDM4	ENST000003	.	.	A	G	+	+	0.25	9,0,3,0	chr1,204521	Cytosol	A > G
chr1:204521634:A:G:UTR3:MDM4	chr1	204521634	204521634	A	G	UTR3	MDM4	ENST000003	.	.	A	G	+	+	0.4	3,0,2,0	chr1,204521	Cytosol	A > G
chr1:204524817:A:G:UTR3:MDM4	chr1	204524817	204524817	A	G	UTR3	MDM4	ENST000003	.	.	A	G	+	+	0.25	6,0,2,0	chr1,204524	Cytosol	A > G
chr1:204524840:A:G:UTR3:MDM4	chr1	204524840	204524840	A	G	UTR3	MDM4	ENST000003	.	.	A	G	+	+	0.5	3,0,3,0	chr1,204524	Cytosol	A > G
chr1:204524950:A:G:UTR3:MDM4	chr1	204524950	204524950	A	G	UTR3	MDM4	ENST000003	.	.	A	G	+	+	0.6	2,0,3,0	chr1,204524	Cytosol	A > G
chr1:204525548:A:G:UTR3:MDM4	chr1	204525548	204525548	A	G	UTR3	MDM4	ENST000003	.	.	A	G	+	+	0.18181818	9,0,2,0	chr1,204525	Cytosol	A > G
chr1:204525593:A:G:UTR3:MDM4	chr1	204525593	204525593	A	G	UTR3	MDM4	ENST000003	.	.	A	G	+	+	0.5	5,0,5,0	chr1,204525	Cytosol	A > G
chr1:204525600:A:G:UTR3:MDM4	chr1	204525600	204525600	A	G	UTR3	MDM4	ENST000003	.	.	A	G	+	+	0.33333333	6,0,3,0	chr1,204525	Cytosol	A > G
chr1:204525647:A:G:UTR3:MDM4	chr1	204525647	204525647	A	G	UTR3	MDM4	ENST000003	.	.	A	G	+	+	0.18181818	9,0,2,0	chr1,204525	Cytosol	A > G
chr1:204525649:A:G:UTR3:MDM4	chr1	204525649	204525649	A	G	UTR3	MDM4	ENST000003	.	.	A	G	+	+	0.6	4,0,6,0	chr1,204525	Cytosol	A > G
chr1:204525744:A:G:UTR3:MDM4	chr1	204525744	204525744	A	G	UTR3	MDM4	ENST000003	.	.	A	G	+	+	0.46153846	7,0,6,0	chr1,204525	Cytosol	A > G
chr1:204526631:A:G:UTR3:MDM4	chr1	204526631	204526631	A	G	UTR3	MDM4	ENST000003	.	.	A	G	+	+	0.18181818	9,0,2,0	chr1,204526	Cytosol	A > G
chr1:204526649:A:G:UTR3:MDM4	chr1	204526649	204526649	A	G	UTR3	MDM4	ENST000003	.	.	A	G	+	+	0.5	4,0,4,0	chr1,204526	Cytosol	A > G
chr1:204526660:A:G:UTR3:MDM4	chr1	204526660	204526660	A	G	UTR3	MDM4	ENST000003	.	.	A	G	+	+	0.71428571	2,0,5,0	chr1,204526	Cytosol	A > G
chr1:204526704:A:G:UTR3:MDM4	chr1	204526704	204526704	A	G	UTR3	MDM4	ENST000003	.	.	A	G	+	+	0.42857143	4,0,3,0	chr1,204526	Cytosol	A > G
chr1:204526710:A:G:UTR3:MDM4	chr1	204526710	204526710	A	G	UTR3	MDM4	ENST000003	.	.	A	G	+	+	0.875	1,0,7,0	chr1,204526	Cytosol	A > G
chr1:204526795:A:G:UTR3:MDM4	chr1	204526795	204526795	A	G	UTR3	MDM4	ENST000003	.	.	A	G	+	+	0.30769231	9,0,4,0	chr1,204526	Cytosol	A > G
chr1:205738694:T:C:UTR3:RAB7L1	chr1	205738694	205738694	T	C	UTR3	RAB7L1	ENST000003	.	.	A	G	-	-	0.01666667	177,0,3,0	chr1,205738	Cytosol	A > G
chr1:205738721:T:C:UTR3:RAB7L1	chr1	205738721	205738721	T	C	UTR3	RAB7L1	ENST000003	.	.	A	G	-	-	0.0130719	151,0,2,0	chr1,205738	Cytosol	A > G
chr1:207270651:A:G:intronic:C4BF	chr1	207270651	207270651	A	G	intronic	C4BPB	.	.	.	A	G	+	+	0.8	1,0,4,0	chr1,207270	Cytosol	A > G
chr1:207942336:A:G:intronic:CD4	chr1	207942336	207942336	A	G	intronic	CD46	.	.	.	A	G	+	+	0.42857143	4,0,3,0	chr1,207942	Cytosol	A > G
chr1:207943187:A:G:intronic:CD4	chr1	207943187	207943187	A	G	intronic	CD46	.	.	.	A	G	+	+	0.33333333	4,0,2,0	chr1,207943	Cytosol	A > G
chr1:207943228:A:G:intronic:CD4	chr1	207943228	207943228	A	G	intronic	CD46	.	.	.	A	G	+	+	0.375	5,0,3,0	chr1,207943	Cytosol	A > G
chr1:207943276:A:G:intronic:CD4	chr1	207943276	207943276	A	G	intronic	CD46	.	.	.	A	G	+	+	0.5	3,0,3,0	chr1,207943	Cytosol	A > G
chr1:207943325:A:G:intronic:CD4	chr1	207943325	207943325	A	G	intronic	CD46	.	.	.	A	G	+	+	0.6	2,0,3,0	chr1,207943	Cytosol	A > G
chr1:212114902:T:C:UTR3:INTS7	chr1	212114902	212114902	T	C	UTR3	INTS7	ENST000003	.	.	A	G	-	-	0.06976744	80,0,6,0	chr1,212114	Cytosol	A > G
chr1:212114963:T:C:UTR3:INTS7	chr1	212114963	212114963	T	C	UTR3	INTS7	ENST000003	.	.	A	G	-	-	0.01818182	108,0,2,0	chr1,212114	Cytosol	A > G
chr1:212989565:A:G:UTR3:TATDN	chr1	212989565	212989565	A	G	UTR3	TATDN3	ENST000003	.	.	A	G	+	+	0.07017544	53,0,4,0	chr1,212989	Cytosol	A > G
chr1:214523899:T:C:ncRNA_intror	chr1	214523899	214523899	T	C	ncRNA_intro	RP11-176D1	.	.	.	A	G	-	-	0.08988764	81,0,8,0	chr1,214523	Cytosol	A > G
chr1:214524105:T:C:ncRNA_intror	chr1	214524105	214524105	T	C	ncRNA_intro	RP11-176D1	.	.	.	A	G	-	-	0.91304348	2,0,21,0	chr1,214524	Cytosol	A > G
chr1:214524106:T:C:ncRNA_intror	chr1	214524106	214524106	T	C	ncRNA_intro	RP11-176D1	.	.	.	A	G	-	-	0.57692308	11,0,15,0	chr1,214524	Cytosol	A > G
chr1:214524121:T:C:ncRNA_intror	chr1	214524121	214524121	T	C	ncRNA_intro	RP11-176D1	.	.	.	A	G	-	-	0.22222222	14,0,4,0	chr1,214524	Cytosol	A > G
chr1:214524129:T:C:ncRNA_intror	chr1	214524129	214524129	T	C	ncRNA_intro	RP11-176D1	.	.	.	A	G	-	-	0.3	14,0,6,0	chr1,214524	Cytosol	A > G
chr1:214524485:T:C:ncRNA_intror	chr1	214524485	214524485	T	C	ncRNA_intro	RP11-176D1	.	.	.	A	G	-	-	0.1025641	105,0,12,0	chr1,214524	Cytosol	A > G
chr1:214524522:T:C:ncRNA_intror	chr1	214524522	214524522	T	C	ncRNA_intro	RP11-176D1	.	.	.	A	G	-	-	0.19607843	41,0,10,0	chr1,214524	Cytosol	A > G
chr1:214524525:T:C:ncRNA_intror	chr1	214524525	214524525	T	C	ncRNA_intro	RP11-176D1	.	.	.	A	G	-	-	0.08	46,0,4,0	chr1,214524	Cytosol	A > G
chr1:214524556:T:C:ncRNA_intror	chr1	214524556	214524556	T	C	ncRNA_intro	RP11-176D1	.	.	.	A	G	-	-	0.66666667	15,0,30,0	chr1,214524	Cytosol	A > G
chr1:214524566:T:C:ncRNA_intror	chr1	214524566	214524566	T	C	ncRNA_intro	RP11-176D1	.	.	.	A	G	-	-	0.39215686	31,0,20,0	chr1,214524	Cytosol	A > G
chr1:214529137:T:C:ncRNA_intror	chr1	214529137	214529137	T	C	ncRNA_intro	RP11-176D1	.	.	.	A	G	-	-	0.21348315	70,0,19,0	chr1,214529	Cytosol	A > G
chr1:214529170:T:C:ncRNA_intror	chr1	214529170	214529170	T	C	ncRNA_intro	RP11-176D1	.	.	.	A	G	-	-	0.11965812	103,0,14,0	chr1,214529	Cytosol	A > G
chr1:214529234:T:C:ncRNA_intror	chr1	214529234	214529234	T	C	ncRNA_intro	RP11-176D1	.	.	.	A	G	-	-	0.39837398	74,0,49,0	chr1,214529	Cytosol	A > G
chr1:214529247:T:C:ncRNA_intror	chr1	214529247	214529247	T	C	ncRNA_intro	RP11-176D1	.	.	.	A	G	-	-	0.11538462	92,0,12,0	chr1,214529	Cytosol	A > G
chr1:214529252:T:C:ncRNA_intror	chr1	214529252	214529252	T	C	ncRNA_intro	RP11-176D1	.	.	.	A	G	-	-	0.1	90,0,10,0	chr1,214529	Cytosol	A > G
chr1:214529253:T:C:ncRNA_intror	chr1	214529253	214529253	T	C	ncRNA_intro	RP11-176D1	.	.	.	A	G	-	-	0.10679612	92,0,11,0	chr1,214529	Cytosol	A > G
chr1:214529725:T:C:ncRNA_intror	chr1	214529725	214529725	T	C	ncRNA_intro	RP11-176D1	.	.	.	A	G	-	-	0.60493827	32,0,49,0	chr1,214529	Cytosol	A > G
chr1:214529740:T:C:ncRNA_intror	chr1	214529740	214529740	T	C	ncRNA_intro	RP11-176D1	.	.	.	A	G	-	-	0.43589744	44,0,34,0	chr1,214529	Cytosol	A > G
chr1:214529742:T:C:ncRNA_intror	chr1	214529742	214529742	T	C	ncRNA_intro	RP11-176D1	.	.	.	A	G	-	-	0.10810811	66,0,8,0	chr1,214529	Cytosol	A > G
chr1:214529774:T:C:ncRNA_intror	chr1	214529774	214529774	T	C	ncRNA_intro	RP11-176D1	.	.	.	A	G	-	-	0.12195122	72,0,10,0	chr1,214529	Cytosol	A > G
chr1:217777142:T:C:intronic:GPAT	chr1	217777142	217777142	T	C	intronic	GPATCH2	.	.	.	A	G	-	-	0.5	4,0,4,0	chr1,217777	Cytosol	A > G
chr1:217777144:T:C:intronic:GPAT	chr1	217777144	217777144	T	C	intronic	GPATCH2	.	.	.	A	G	-	-	0.66666667	3,0,6,0	chr1,217777	Cytosol	A > G
chr1:217777147:T:C:intronic:GPAT	chr1	217777147	217777147	T	C	intronic	GPATCH2	.	.	.	A	G	-	-	0.22222222	7,0,2,0	chr1,217777	Cytosol	A > G
chr1:217777183:T:C:intronic:GPAT	chr1	217777183	217777183	T	C	intronic	GPATCH2	.	.	.	A	G	-	-	0.375	5,0,3,0	chr1,217777	Cytosol	A > G
chr1:217777237:T:C:intronic:GPAT	chr1	217777237	217777237	T	C	intronic	GPATCH2	.	.	.	A	G	-	-	0.25	6,0,2,0	chr1,217777	Cytosol	A > G
chr1:217777244:T:C:intronic:GPAT	chr1	217777244	217777244	T	C	intronic	GPATCH2	.	.	.	A	G	-	-	0.22222222	7,0,2,0	chr1,217777	Cytosol	A > G
chr1:217777463:T:C:intronic:GPAT	chr1	217777463	217777463	T	C	intronic	GPATCH2	.	.	.	A	G	-	-	0.1875	13,0,3,0	chr1,217777	Cytosol	A > G
chr1:217777527:T:C:intronic:GPAT	chr1	217777527	217777527	T	C	intronic	GPATCH2	.	.	.	A	G	-	-	0.7	3,0,7,0	chr1,217777	Cytosol	A > G
chr1:217777529:T:C:intronic:GPAT	chr1	217777529	217777529	T	C	intronic	GPATCH2	.	.	.	A	G	-	-	0.7	3,0,7,0	chr1,217777	Cytosol	A > G
chr1:217777611:T:C:intronic:GPAT	chr1	217777611	217777611	T	C	intronic	GPATCH2	.	.	.	A	G	-	-	0.5	4,0,4,0	chr1,217777	Cytosol	A > G
chr1:217777646:T:C:intronic:GPAT	chr1	217777646	217777646	T	C	intronic	GPATCH2	.	.	.	A	G	-	-	0.5	3,0,3,0	chr1,217777	Cytosol	A > G
chr1:220231254:T:C:exonic:BPNT1	chr1	220231254	220231254	T	C	exonic	BPNT1	.	stoploss	BPNT1:ENST(	A	G	-	+-	0.13872832	149,0,24,0	chr1,220231	Cytosol	A > G
chr1:220231261:T:C:splicing:BPNT	chr1	220231261	220231261	T	C	splicing	BPNT1	ENST000003	.	.	A	G	-	+-	0.06190476	197,0,13,0	chr1,220231	Cytosol	A > G
chr1:220231263:T:C:UTR3:BPNT1	chr1	220231263	220231263	T	C	UTR3	BPNT1	ENST000003	.	.	A	G	-	+-	0.7047619	62,0,148,0	chr1,220231	Cytosol	A > G
chr1:220231275:T:C:UTR3:BPNT1	chr1	220231275	220231275	T	C	UTR3	BPNT1	ENST000003	.	.	A	G	-	+-	0.75117371	53,0,160,0	chr1,220231	Cytosol	A > G

chr1:220231276:T:C:UTR3:BPNT1	chr1	220231276	220231276	T	C	UTR3	BPNT1	ENST000003	.	.	A	G	-	+-	0.34951456	134,0,72,0	chr1,220231	Cytosol	A > G
chr1:220231277:T:C:UTR3:BPNT1	chr1	220231277	220231277	T	C	UTR3	BPNT1	ENST000003	.	.	A	G	-	+-	0.04109589	210,0,9,0	chr1,220231	Cytosol	A > G
chr1:220231330:T:C:UTR3:BPNT1	chr1	220231330	220231330	T	C	UTR3	BPNT1	ENST000003	.	.	A	G	-	+-	0.81990521	38,0,173,0	chr1,220231	Cytosol	A > G
chr1:220231338:T:C:UTR3:BPNT1	chr1	220231338	220231338	T	C	UTR3	BPNT1	ENST000003	.	.	A	G	-	+-	0.15675676	156,0,29,0	chr1,220231	Cytosol	A > G
chr1:220231347:T:C:UTR3:BPNT1	chr1	220231347	220231347	T	C	UTR3	BPNT1	ENST000003	.	.	A	G	-	+-	0.25490196	114,0,39,0	chr1,220231	Cytosol	A > G
chr1:220231352:T:C:UTR3:BPNT1	chr1	220231352	220231352	T	C	UTR3	BPNT1	ENST000003	.	.	A	G	-	+-	0.06293706	134,0,9,0	chr1,220231	Cytosol	A > G
chr1:220231370:T:C:UTR3:BPNT1	chr1	220231370	220231370	T	C	UTR3	BPNT1	ENST000003	.	.	A	G	-	+-	0.11450382	116,0,15,0	chr1,220231	Cytosol	A > G
chr1:220231371:T:C:UTR3:BPNT1	chr1	220231371	220231371	T	C	UTR3	BPNT1	ENST000003	.	.	A	G	-	+-	0.03875969	124,0,5,0	chr1,220231	Cytosol	A > G
chr1:220231406:T:C:UTR3:BPNT1	chr1	220231406	220231406	T	C	UTR3	BPNT1	ENST000003	.	.	A	G	-	+-	0.05050505	94,0,5,0	chr1,220231	Cytosol	A > G
chr1:220231418:T:C:UTR3:BPNT1	chr1	220231418	220231418	T	C	UTR3	BPNT1	ENST000003	.	.	A	G	-	+-	0.07518797	123,0,10,0	chr1,220231	Cytosol	A > G
chr1:220231455:T:C:UTR3:BPNT1	chr1	220231455	220231455	T	C	UTR3	BPNT1	ENST000003	.	.	A	G	-	+-	0.276	181,0,69,0	chr1,220231	Cytosol	A > G
chr1:220231456:T:C:UTR3:BPNT1	chr1	220231456	220231456	T	C	UTR3	BPNT1	ENST000003	.	.	A	G	-	+-	0.05241935	235,0,13,0	chr1,220231	Cytosol	A > G
chr1:220231487:T:C:UTR3:BPNT1	chr1	220231487	220231487	T	C	UTR3	BPNT1	ENST000003	.	.	A	G	-	+-	0.79467681	54,0,209,0	chr1,220231	Cytosol	A > G
chr1:220231946:T:C:UTR3:BPNT1	chr1	220231946	220231946	T	C	UTR3	BPNT1	ENST000003	.	.	A	G	-	+-	0.13675214	101,0,16,0	chr1,220231	Cytosol	A > G
chr1:220231959:T:C:UTR3:BPNT1	chr1	220231959	220231959	T	C	UTR3	BPNT1	ENST000003	.	.	A	G	-	+-	0.92631579	7,0,88,0	chr1,220231	Cytosol	A > G
chr1:220231963:T:C:UTR3:BPNT1	chr1	220231963	220231963	T	C	UTR3	BPNT1	ENST000003	.	.	A	G	-	+-	0.29761905	59,0,25,0	chr1,220231	Cytosol	A > G
chr1:220231982:T:C:UTR3:BPNT1	chr1	220231982	220231982	T	C	UTR3	BPNT1	ENST000003	.	.	A	G	-	+-	0.33018868	71,0,35,0	chr1,220231	Cytosol	A > G
chr1:220231988:T:C:UTR3:BPNT1	chr1	220231988	220231988	T	C	UTR3	BPNT1	ENST000003	.	.	A	G	-	+-	0.15929204	95,0,18,0	chr1,220231	Cytosol	A > G
chr1:220231997:T:C:UTR3:BPNT1	chr1	220231997	220231997	T	C	UTR3	BPNT1	ENST000003	.	.	A	G	-	+-	0.01626016	121,0,2,0	chr1,220231	Cytosol	A > G
chr1:220232011:T:C:UTR3:BPNT1	chr1	220232011	220232011	T	C	UTR3	BPNT1	ENST000003	.	.	A	G	-	+-	0.16129032	130,0,25,0	chr1,220232	Cytosol	A > G
chr1:220232066:T:C:UTR3:BPNT1	chr1	220232066	220232066	T	C	UTR3	BPNT1	ENST000003	.	.	A	G	-	+-	0.03684211	183,0,7,0	chr1,220232	Cytosol	A > G
chr1:220232098:T:C:UTR3:BPNT1	chr1	220232098	220232098	T	C	UTR3	BPNT1	ENST000003	.	.	A	G	-	+-	0.04968944	153,0,8,0	chr1,220232	Cytosol	A > G
chr1:220232138:T:C:UTR3:BPNT1	chr1	220232138	220232138	T	C	UTR3	BPNT1	ENST000003	.	.	A	G	-	+-	0.14545455	94,0,16,0	chr1,220232	Cytosol	A > G
chr1:220232143:T:C:UTR3:BPNT1	chr1	220232143	220232143	T	C	UTR3	BPNT1	ENST000003	.	.	A	G	-	+-	0.51181102	62,0,65,0	chr1,220232	Cytosol	A > G
chr1:220232144:T:C:UTR3:BPNT1	chr1	220232144	220232144	T	C	UTR3	BPNT1	ENST000003	.	.	A	G	-	+-	0.63565891	47,0,82,0	chr1,220232	Cytosol	A > G
chr1:225679890:T:C:UTR3:ENAH	chr1	225679890	225679890	T	C	UTR3	ENAH	ENST000003	.	.	A	G	-	-	0.44444444	5,0,4,0	chr1,225679	Cytosol	A > G
chr1:225974581:A:G:exonic:SRP9	chr1	225974581	225974581	A	G	exonic	SRP9	.	synonymous	SRP9:ENST0C	A	G	+	+	0.19101124	144,0,34,0	chr1,225974	Cytosol	A > G
chr1:225974614:A:G:exonic:SRP9	chr1	225974614	225974614	A	G	exonic	SRP9	.	nonsynonym	SRP9:ENST0C	A	G	+	+	0.4952381	53,0,52,0	chr1,225974	Cytosol	A > G
chr1:225974674:A:G:UTR3:SRP9	chr1	225974674	225974674	A	G	UTR3	SRP9	ENST000003	.	.	A	G	+	+	0.10273973	131,0,15,0	chr1,225974	Cytosol	A > G
chr1:225974735:A:G:intronic:SRP9	chr1	225974735	225974735	A	G	intronic	SRP9	.	.	.	A	G	+	+	0.33333333	6,0,3,0	chr1,225974	Cytosol	A > G
chr1:225974746:A:G:intronic:SRP9	chr1	225974746	225974746	A	G	intronic	SRP9	.	.	.	A	G	+	+	0.90909091	1,0,10,0	chr1,225974	Cytosol	A > G
chr1:225974748:A:G:intronic:SRP9	chr1	225974748	225974748	A	G	intronic	SRP9	.	.	.	A	G	+	+	0.91666667	1,0,11,0	chr1,225974	Cytosol	A > G
chr1:225975110:A:G:intronic:SRP9	chr1	225975110	225975110	A	G	intronic	SRP9	.	.	.	A	G	+	+	0.8	1,0,4,0	chr1,225975	Cytosol	A > G
chr1:225975913:A:G:intronic:SRP9	chr1	225975913	225975913	A	G	intronic	SRP9	.	.	.	A	G	+	+	0.08695652	84,0,8,0	chr1,225975	Cytosol	A > G
chr1:225975921:A:G:intronic:SRP9	chr1	225975921	225975921	A	G	intronic	SRP9	.	.	.	A	G	+	+	0.09677419	56,0,6,0	chr1,225975	Cytosol	A > G
chr1:225975936:A:G:intronic:SRP9	chr1	225975936	225975936	A	G	intronic	SRP9	.	.	.	A	G	+	+	0.03174603	61,0,2,0	chr1,225975	Cytosol	A > G
chr1:225975941:A:G:intronic:SRP9	chr1	225975941	225975941	A	G	intronic	SRP9	.	.	.	A	G	+	+	0.31034483	40,0,18,0	chr1,225975	Cytosol	A > G
chr1:225975945:A:G:intronic:SRP9	chr1	225975945	225975945	A	G	intronic	SRP9	.	.	.	A	G	+	+	0.10909091	49,0,6,0	chr1,225975	Cytosol	A > G
chr1:225975946:A:G:intronic:SRP9	chr1	225975946	225975946	A	G	intronic	SRP9	.	.	.	A	G	+	+	0.44230769	29,0,23,0	chr1,225975	Cytosol	A > G
chr1:225975947:A:G:intronic:SRP9	chr1	225975947	225975947	A	G	intronic	SRP9	.	.	.	A	G	+	+	0.17647059	42,0,9,0	chr1,225975	Cytosol	A > G
chr1:225975950:A:G:intronic:SRP9	chr1	225975950	225975950	A	G	intronic	SRP9	.	.	.	A	G	+	+	0.54545455	25,0,30,0	chr1,225975	Cytosol	A > G
chr1:225975954:A:G:intronic:SRP9	chr1	225975954	225975954	A	G	intronic	SRP9	.	.	.	A	G	+	+	1	0,0,56,0	chr1,225975	Cytosol	A > G
chr1:225975976:A:G:intronic:SRP9	chr1	225975976	225975976	A	G	intronic	SRP9	.	.	.	A	G	+	+	0.6	22,0,33,0	chr1,225975	Cytosol	A > G
chr1:225975990:A:G:intronic:SRP9	chr1	225975990	225975990	A	G	intronic	SRP9	.	.	.	A	G	+	+	0.53571429	26,0,30,0	chr1,225975	Cytosol	A > G
chr1:225976051:A:G:intronic:SRP9	chr1	225976051	225976051	A	G	intronic	SRP9	.	.	.	A	G	+	+	0.98275862	1,0,57,0	chr1,225976	Cytosol	A > G
chr1:225976060:A:G:intronic:SRP9	chr1	225976060	225976060	A	G	intronic	SRP9	.	.	.	A	G	+	+	0.41509434	31,0,22,0	chr1,225976	Cytosol	A > G
chr1:225976193:A:G:intronic:SRP9	chr1	225976193	225976193	A	G	intronic	SRP9	.	.	.	A	G	+	+	0.78181818	12,0,43,0	chr1,225976	Cytosol	A > G
chr1:225976198:A:G:intronic:SRP9	chr1	225976198	225976198	A	G	intronic	SRP9	.	.	.	A	G	+	+	0.5	28,0,28,0	chr1,225976	Cytosol	A > G
chr1:225976243:A:G:intronic:SRP9	chr1	225976243	225976243	A	G	intronic	SRP9	.	.	.	A	G	+	+	0.97560976	1,0,40,0	chr1,225976	Cytosol	A > G
chr1:225976244:A:G:intronic:SRP9	chr1	225976244	225976244	A	G	intronic	SRP9	.	.	.	A	G	+	+	0.44186047	24,0,19,0	chr1,225976	Cytosol	A > G
chr1:225976265:A:G:intronic:SRP9	chr1	225976265	225976265	A	G	intronic	SRP9	.	.	.	A	G	+	+	0.1509434	45,0,8,0	chr1,225976	Cytosol	A > G
chr1:225976283:A:G:intronic:SRP9	chr1	225976283	225976283	A	G	intronic	SRP9	.	.	.	A	G	+	+	0.41860465	25,0,18,0	chr1,225976	Cytosol	A > G
chr1:225976320:A:G:intronic:SRP9	chr1	225976320	225976320	A	G	intronic	SRP9	.	.	.	A	G	+	+	0.15789474	32,0,6,0	chr1,225976	Cytosol	A > G
chr1:226435664:T:C:intronic:LIN9	chr1	226435664	226435664	T	C	intronic	LIN9	.	.	.	A	G	-	-	0.5	3,0,3,0	chr1,226435	Cytosol	A > G
chr1:228365255:A:G:UTR3:IBA57	chr1	228365255	228365255	A	G	UTR3	IBA57	ENST000003	.	.	A	G	+	+	0.33333333	4,0,2,0	chr1,228365	Cytosol	A > G
chr1:228368425:A:G:UTR3:IBA57	chr1	228368425	228368425	A	G	UTR3	IBA57	ENST000003	.	.	A	G	+	+	0.66666667	2,0,4,0	chr1,228368	Cytosol	A > G
chr1:228368487:A:G:UTR3:IBA57	chr1	228368487	228368487	A	G	UTR3	IBA57	ENST000003	.	.	A	G	+	+	0.6	2,0,3,0	chr1,228368	Cytosol	A > G
chr1:228823322:G:A:intergenic:SN	chr1	228823322	228823322	G	A	intergenic	SNORA51,RH	dist=35015;d	.	.	G	A	+	+	1	387,0,0,0	chr1,228823	Cytosol	G > A
chr1:231466398:T:C:intergenic:RN	chr1	231466398	231466398	T	C	intergenic	RNA5SP80,E)	dist=49128;d	.	.	A	G	-		0.83333333	1,0,5,0	chr1,231466	Cytosol	A > G
chr1:231466770:T:C:intergenic:RN	chr1	231466770	231466770	T	C	intergenic	RNA5SP80,E)	dist=49500;d	.	.	A	G	-		0.25	6,0,2,0	chr1,231466	Cytosol	A > G
chr1:235990199:T:C:intronic:LYST	chr1	235990199	235990199	T	C	intronic	LYST	.	.	.	A	G	-	-	0.33333333	4,0,2,0	chr1,235990	Cytosol	A > G
chr1:241748815:T:C:intronic:KMO	chr1	241748815	241748815	T	C	intronic	KMO	.	.	.	A	G	-	+	0.4	3,0,2,0	chr1,241748	Cytosol	A > G
chr1:243223724:C:T:intergenic:RP	chr1	243223724	243223724	C	T	intergenic	RP11-261C1(	dist=8170;dis	.	.	G	A	-	-	0.45454545	5,0,6,0	chr1,243223	Cytosol	G > A
chr1:243431296:C:T:intronic:SDCC	chr1	243431296	243431296	C	T	intronic	SDCCAG8	.	.	.	G	A	-	+-	1	5,0,0,0	chr1,243431	Cytosol	G > A

chr1:246824603:A:G:intronic:CNS	chr1	246824603	246824603	A	G	intronic	CNST	.	.	.	A	G	+	+	0.5	3,0,3,0	chr1,246824	Cytosol	A > G
chr1:247263719:T:C:exonic:ZNF66	chr1	247263719	247263719	T	C	exonic	ZNF669	.	nonsynonym	ZNF669:ENS	A	G	-	-	0.06896552	27,0,2,0	chr1,247263	Cytosol	A > G
chr10:5970316:A:G:intronic:FBXO	chr10	5970316	5970316	A	G	intronic	FBXO18	.	.	.	A	G	+	+	0.6	2,0,3,0	chr10,59703	Cytosol	A > G
chr10:12163009:A:G:UTR3:DHTKD	chr10	12163009	12163009	A	G	UTR3	DHTKD1	ENST00000	.	.	A	G	+	+	0.04166667	46,0,2,0	chr10,12163	Cytosol	A > G
chr10:12163023:A:G:UTR3:DHTKD	chr10	12163023	12163023	A	G	UTR3	DHTKD1	ENST00000	.	.	A	G	+	+	0.06666667	42,0,3,0	chr10,12163	Cytosol	A > G
chr10:12163032:A:G:UTR3:DHTKD	chr10	12163032	12163032	A	G	UTR3	DHTKD1	ENST00000	.	.	A	G	+	+	0.15909091	37,0,7,0	chr10,12163	Cytosol	A > G
chr10:12163033:A:G:UTR3:DHTKD	chr10	12163033	12163033	A	G	UTR3	DHTKD1	ENST00000	.	.	A	G	+	+	0.19565217	37,0,9,0	chr10,12163	Cytosol	A > G
chr10:12163043:A:G:UTR3:DHTKD	chr10	12163043	12163043	A	G	UTR3	DHTKD1	ENST00000	.	.	A	G	+	+	0.08163265	45,0,4,0	chr10,12163	Cytosol	A > G
chr10:12163126:A:G:UTR3:DHTKD	chr10	12163126	12163126	A	G	UTR3	DHTKD1	ENST00000	.	.	A	G	+	+	0.12820513	102,0,15,0	chr10,12163	Cytosol	A > G
chr10:12163168:A:G:UTR3:DHTKD	chr10	12163168	12163168	A	G	UTR3	DHTKD1	ENST00000	.	.	A	G	+	+	0.01923077	102,0,2,0	chr10,12163	Cytosol	A > G
chr10:12163174:A:G:UTR3:DHTKD	chr10	12163174	12163174	A	G	UTR3	DHTKD1	ENST00000	.	.	A	G	+	+	0.02020202	97,0,2,0	chr10,12163	Cytosol	A > G
chr10:12163206:A:G:UTR3:DHTKD	chr10	12163206	12163206	A	G	UTR3	DHTKD1	ENST00000	.	.	A	G	+	+	0.03092784	94,0,3,0	chr10,12163	Cytosol	A > G
chr10:12164435:A:G:UTR3:DHTKD	chr10	12164435	12164435	A	G	UTR3	DHTKD1	ENST00000	.	.	A	G	+	+	0.11428571	62,0,8,0	chr10,12164	Cytosol	A > G
chr10:12165327:A:G:downstream:	chr10	12165327	12165327	A	G	downstream	DHTKD1	.	.	.	A	G	+		0.66666667	3,0,6,0	chr10,12165	Cytosol	A > G
chr10:13179033:A:G:UTR3:OPTN	chr10	13179033	13179033	A	G	UTR3	OPTN	ENST00000	.	.	A	G	+	+	0.05882353	32,0,2,0	chr10,13179	Cytosol	A > G
chr10:14947979:T:C:intronic:DCLR	chr10	14947979	14947979	T	C	intronic	DCLRE1C	.	.	.	A	G	-	-	0.5	5,0,5,0	chr10,14947	Cytosol	A > G
chr10:14949134:T:C:UTR3:DCLRE1	chr10	14949134	14949134	T	C	UTR3	DCLRE1C	ENST00000	.	.	A	G	-	-	0.21428571	11,0,3,0	chr10,14949	Cytosol	A > G
chr10:14949135:T:C:UTR3:DCLRE1	chr10	14949135	14949135	T	C	UTR3	DCLRE1C	ENST00000	.	.	A	G	-	-	0.1875	13,0,3,0	chr10,14949	Cytosol	A > G
chr10:16479385:A:G:UTR5:PTER	chr10	16479385	16479385	A	G	UTR5	PTER	ENST00000	.	.	A	G	+	+	0.32258065	21,0,10,0	chr10,16479	Cytosol	A > G
chr10:16479432:A:G:UTR5:PTER	chr10	16479432	16479432	A	G	UTR5	PTER	ENST00000	.	.	A	G	+	+	0.12	22,0,3,0	chr10,16479	Cytosol	A > G
chr10:16479437:A:G:UTR5:PTER	chr10	16479437	16479437	A	G	UTR5	PTER	ENST00000	.	.	A	G	+	+	0.08	23,0,2,0	chr10,16479	Cytosol	A > G
chr10:28810188:T:C:intergenic:RP	chr10	28810188	28810188	T	C	intergenic	RP11-351M1	dist=25446;d	.	.	A	G	-	-	0.33333333	4,0,2,0	chr10,28810	Cytosol	A > G
chr10:28812034:T:C:ncRNA_exoni	chr10	28812034	28812034	T	C	ncRNA_exon	WAC-AS1	.	.	.	A	G	-	-	0.11627907	38,0,5,0	chr10,28812	Cytosol	A > G
chr10:28812596:T:C:ncRNA_exoni	chr10	28812596	28812596	T	C	ncRNA_exon	WAC-AS1	.	.	.	A	G	-	-	0.11764706	30,0,4,0	chr10,28812	Cytosol	A > G
chr10:28812603:T:C:ncRNA_exoni	chr10	28812603	28812603	T	C	ncRNA_exon	WAC-AS1	.	.	.	A	G	-	-	0.07692308	24,0,2,0	chr10,28812	Cytosol	A > G
chr10:28812618:T:C:ncRNA_exoni	chr10	28812618	28812618	T	C	ncRNA_exon	WAC-AS1	.	.	.	A	G	-	-	0.35	13,0,7,0	chr10,28812	Cytosol	A > G
chr10:28812626:T:C:ncRNA_exoni	chr10	28812626	28812626	T	C	ncRNA_exon	WAC-AS1	.	.	.	A	G	-	-	0.11111111	16,0,2,0	chr10,28812	Cytosol	A > G
chr10:28812627:T:C:ncRNA_exoni	chr10	28812627	28812627	T	C	ncRNA_exon	WAC-AS1	.	.	.	A	G	-	-	0.19047619	17,0,4,0	chr10,28812	Cytosol	A > G
chr10:28812639:T:C:ncRNA_exoni	chr10	28812639	28812639	T	C	ncRNA_exon	WAC-AS1	.	.	.	A	G	-	-	0.06060606	31,0,2,0	chr10,28812	Cytosol	A > G
chr10:28814615:T:C:ncRNA_intror	chr10	28814615	28814615	T	C	ncRNA_intro	WAC-AS1	.	.	.	A	G	-	-	0.76923077	3,0,10,0	chr10,28814	Cytosol	A > G
chr10:28814618:T:C:ncRNA_intror	chr10	28814618	28814618	T	C	ncRNA_intro	WAC-AS1	.	.	.	A	G	-	-	0.25	9,0,3,0	chr10,28814	Cytosol	A > G
chr10:28814655:T:C:ncRNA_intror	chr10	28814655	28814655	T	C	ncRNA_intro	WAC-AS1	.	.	.	A	G	-	-	0.5	6,0,6,0	chr10,28814	Cytosol	A > G
chr10:28814658:T:C:ncRNA_intror	chr10	28814658	28814658	T	C	ncRNA_intro	WAC-AS1	.	.	.	A	G	-	-	0.27272727	8,0,3,0	chr10,28814	Cytosol	A > G
chr10:28814692:T:C:ncRNA_intror	chr10	28814692	28814692	T	C	ncRNA_intro	WAC-AS1	.	.	.	A	G	-	-	0.73333333	4,0,11,0	chr10,28814	Cytosol	A > G
chr10:28827400:A:G:intronic:WAC	chr10	28827400	28827400	A	G	intronic	WAC	.	.	.	A	G	+	+	0.6	2,0,3,0	chr10,28827	Cytosol	A > G
chr10:28827426:A:G:intronic:WAC	chr10	28827426	28827426	A	G	intronic	WAC	.	.	.	A	G	+	+	0.25	6,0,2,0	chr10,28827	Cytosol	A > G
chr10:28827637:A:G:intronic:WAC	chr10	28827637	28827637	A	G	intronic	WAC	.	.	.	A	G	+	+	0.57142857	3,0,4,0	chr10,28827	Cytosol	A > G
chr10:28828689:A:G:intronic:WAC	chr10	28828689	28828689	A	G	intronic	WAC	.	.	.	A	G	+	+	0.8	1,0,4,0	chr10,28828	Cytosol	A > G
chr10:29748124:A:G:ncRNA_intro	chr10	29748124	29748124	A	G	ncRNA_intro	PTCHD3P1	.	.	.	A	G	+	+-	0.30769231	9,0,4,0	chr10,29748	Cytosol	A > G
chr10:29748923:A:G:ncRNA_intro	chr10	29748923	29748923	A	G	ncRNA_intro	PTCHD3P1	.	.	.	A	G	+	+-	0.18181818	18,0,4,0	chr10,29748	Cytosol	A > G
chr10:29748954:A:G:ncRNA_intro	chr10	29748954	29748954	A	G	ncRNA_intro	PTCHD3P1	.	.	.	A	G	+	+-	0.1875	13,0,3,0	chr10,29748	Cytosol	A > G
chr10:38354052:A:G:downstream:	chr10	38354052	38354052	A	G	downstream	ZNF33A	.	.	.	A	G	+		0.8	1,0,4,0	chr10,38354	Cytosol	A > G
chr10:38354079:A:G:downstream:	chr10	38354079	38354079	A	G	downstream	ZNF33A	.	.	.	A	G	+		0.4	3,0,2,0	chr10,38354	Cytosol	A > G
chr10:38354274:A:G:downstream:	chr10	38354274	38354274	A	G	downstream	ZNF33A	.	.	.	A	G	+		0.15384615	11,0,2,0	chr10,38354	Cytosol	A > G
chr10:43011705:T:C:ncRNA_exoni	chr10	43011705	43011705	T	C	ncRNA_exon	ZNF37BP	.	.	.	A	G	-	-	0.42857143	4,0,3,0	chr10,43011	Cytosol	A > G
chr10:43013345:T:C:ncRNA_exoni	chr10	43013345	43013345	T	C	ncRNA_exon	ZNF37BP	.	.	.	A	G	-	-	0.26666667	11,0,4,0	chr10,43013	Cytosol	A > G
chr10:43013372:T:C:ncRNA_exoni	chr10	43013372	43013372	T	C	ncRNA_exon	ZNF37BP	.	.	.	A	G	-	-	0.23529412	13,0,4,0	chr10,43013	Cytosol	A > G
chr10:43013377:T:C:ncRNA_exoni	chr10	43013377	43013377	T	C	ncRNA_exon	ZNF37BP	.	.	.	A	G	-	-	0.42857143	8,0,6,0	chr10,43013	Cytosol	A > G
chr10:43013387:T:C:ncRNA_exoni	chr10	43013387	43013387	T	C	ncRNA_exon	ZNF37BP	.	.	.	A	G	-	-	1	0,0,18,0	chr10,43013	Cytosol	A > G
chr10:43013424:T:C:ncRNA_exoni	chr10	43013424	43013424	T	C	ncRNA_exon	ZNF37BP	.	.	.	A	G	-	-	0.1875	13,0,3,0	chr10,43013	Cytosol	A > G
chr10:43013425:T:C:ncRNA_exoni	chr10	43013425	43013425	T	C	ncRNA_exon	ZNF37BP	.	.	.	A	G	-	-	0.1875	13,0,3,0	chr10,43013	Cytosol	A > G
chr10:43013442:T:C:ncRNA_exoni	chr10	43013442	43013442	T	C	ncRNA_exon	ZNF37BP	.	.	.	A	G	-	-	0.36363636	7,0,4,0	chr10,43013	Cytosol	A > G
chr10:43013497:T:C:ncRNA_exoni	chr10	43013497	43013497	T	C	ncRNA_exon	ZNF37BP	.	.	.	A	G	-	-	0.25	6,0,2,0	chr10,43013	Cytosol	A > G
chr10:43014222:T:C:ncRNA_exoni	chr10	43014222	43014222	T	C	ncRNA_exon	ZNF37BP	.	.	.	A	G	-	-	0.4	3,0,2,0	chr10,43014	Cytosol	A > G
chr10:64982454:A:G:intronic:JMJD	chr10	64982454	64982454	A	G	intronic	JMJD1C	.	.	.	A	G	+	-+	1	0,0,53,0	chr10,64982	Cytosol	A > G
chr10:64982524:A:G:intronic:JMJD	chr10	64982524	64982524	A	G	intronic	JMJD1C	.	.	.	A	G	+	-+	1	0,0,52,0	chr10,64982	Cytosol	A > G
chr10:70176055:T:C:intronic:DNA2	chr10	70176055	70176055	T	C	intronic	DNA2	.	.	.	A	G	-	-	0.33333333	4,0,2,0	chr10,70176	Cytosol	A > G
chr10:70176147:T:C:intronic:DNA2	chr10	70176147	70176147	T	C	intronic	DNA2	.	.	.	A	G	-	-	0.28571429	5,0,2,0	chr10,70176	Cytosol	A > G
chr10:70176320:T:C:intronic:DNA2	chr10	70176320	70176320	T	C	intronic	DNA2	.	.	.	A	G	-	-	0.33333333	4,0,2,0	chr10,70176	Cytosol	A > G
chr10:70239509:T:C:UTR3:SLC25A	chr10	70239509	70239509	T	C	UTR3	SLC25A16	ENST00000	.	.	A	G	-	-	1	0,0,5,0	chr10,70239	Cytosol	A > G
chr10:70239721:T:C:UTR3:SLC25A	chr10	70239721	70239721	T	C	UTR3	SLC25A16	ENST00000	.	.	A	G	-	-	0.42857143	4,0,3,0	chr10,70239	Cytosol	A > G
chr10:70239741:T:C:UTR3:SLC25A	chr10	70239741	70239741	T	C	UTR3	SLC25A16	ENST00000	.	.	A	G	-	-	0.33333333	4,0,2,0	chr10,70239	Cytosol	A > G
chr10:71923487:T:C:intronic:SAR1	chr10	71923487	71923487	T	C	intronic	SAR1A	.	.	.	T	C	+	-+	0.96	0,24,0,1	chr10,71923	Cytosol	T > C
chr10:73979859:A:G:intronic:ANA	chr10	73979859	73979859	A	G	intronic	ANAPC16	.	.	.	A	G	+	+	0.10280374	96,0,11,0	chr10,73979	Cytosol	A > G

chr10:73979932:A:G:intronic:ANA	chr10	73979932	73979932	A	G	intronic	ANAPC16	.	.	.	A	G	+	+	0.05072464	131,0,7,0	chr10,73979	Cytosol	A > G
chr10:73979944:A:G:intronic:ANA	chr10	73979944	73979944	A	G	intronic	ANAPC16	.	.	.	A	G	+	+	0.06862745	95,0,7,0	chr10,73979	Cytosol	A > G
chr10:73979967:A:G:intronic:ANA	chr10	73979967	73979967	A	G	intronic	ANAPC16	.	.	.	A	G	+	+	0.0620155	120,0,8,1	chr10,73979	Cytosol	A > G
chr10:73979976:A:G:intronic:ANA	chr10	73979976	73979976	A	G	intronic	ANAPC16	.	.	.	A	G	+	+	0.03007519	129,0,4,0	chr10,73979	Cytosol	A > G
chr10:73979990:A:G:intronic:ANA	chr10	73979990	73979990	A	G	intronic	ANAPC16	.	.	.	A	G	+	+	0.11333333	133,0,17,0	chr10,73979	Cytosol	A > G
chr10:73979993:A:G:intronic:ANA	chr10	73979993	73979993	A	G	intronic	ANAPC16	.	.	.	A	G	+	+	0.09677419	140,0,15,0	chr10,73979	Cytosol	A > G
chr10:73980019:A:G:intronic:ANA	chr10	73980019	73980019	A	G	intronic	ANAPC16	.	.	.	A	G	+	+	0.24056604	161,0,51,0	chr10,73980	Cytosol	A > G
chr10:73993783:A:G:UTR3:ANAPC	chr10	73993783	73993783	A	G	UTR3	ANAPC16	ENST000002	.	.	A	G	+	+	0.91304348	2,0,21,0	chr10,73993	Cytosol	A > G
chr10:73993821:A:G:UTR3:ANAPC	chr10	73993821	73993821	A	G	UTR3	ANAPC16	ENST000002	.	.	A	G	+	+	0.06818182	41,0,3,0	chr10,73993	Cytosol	A > G
chr10:73993829:A:G:UTR3:ANAPC	chr10	73993829	73993829	A	G	UTR3	ANAPC16	ENST000002	.	.	A	G	+	+	0.12195122	36,0,5,0	chr10,73993	Cytosol	A > G
chr10:73993839:A:G:UTR3:ANAPC	chr10	73993839	73993839	A	G	UTR3	ANAPC16	ENST000002	.	.	A	G	+	+	0.225	31,0,9,0	chr10,73993	Cytosol	A > G
chr10:73993845:A:G:UTR3:ANAPC	chr10	73993845	73993845	A	G	UTR3	ANAPC16	ENST000002	.	.	A	G	+	+	0.21621622	29,0,8,0	chr10,73993	Cytosol	A > G
chr10:73993851:A:G:UTR3:ANAPC	chr10	73993851	73993851	A	G	UTR3	ANAPC16	ENST000002	.	.	A	G	+	+	0.17241379	24,0,5,0	chr10,73993	Cytosol	A > G
chr10:73993858:A:G:UTR3:ANAPC	chr10	73993858	73993858	A	G	UTR3	ANAPC16	ENST000002	.	.	A	G	+	+	0.66666667	7,0,14,0	chr10,73993	Cytosol	A > G
chr10:73993864:A:G:UTR3:ANAPC	chr10	73993864	73993864	A	G	UTR3	ANAPC16	ENST000002	.	.	A	G	+	+	0.35714286	9,0,5,0	chr10,73993	Cytosol	A > G
chr10:73993865:A:G:UTR3:ANAPC	chr10	73993865	73993865	A	G	UTR3	ANAPC16	ENST000002	.	.	A	G	+	+	0.4	9,0,6,0	chr10,73993	Cytosol	A > G
chr10:73993890:A:G:UTR3:ANAPC	chr10	73993890	73993890	A	G	UTR3	ANAPC16	ENST000002	.	.	A	G	+	+	0.15384615	11,0,2,0	chr10,73993	Cytosol	A > G
chr10:73993946:A:G:UTR3:ANAPC	chr10	73993946	73993946	A	G	UTR3	ANAPC16	ENST000002	.	.	A	G	+	+	0.33333333	8,0,4,0	chr10,73993	Cytosol	A > G
chr10:73993980:A:G:UTR3:ANAPC	chr10	73993980	73993980	A	G	UTR3	ANAPC16	ENST000002	.	.	A	G	+	+	1	0,0,10,0	chr10,73993	Cytosol	A > G
chr10:73993988:A:G:UTR3:ANAPC	chr10	73993988	73993988	A	G	UTR3	ANAPC16	ENST000002	.	.	A	G	+	+	0.18181818	9,0,2,0	chr10,73993	Cytosol	A > G
chr10:73994000:A:G:UTR3:ANAPC	chr10	73994000	73994000	A	G	UTR3	ANAPC16	ENST000002	.	.	A	G	+	+	0.2	8,0,2,0	chr10,73993	Cytosol	A > G
chr10:73994040:A:G:UTR3:ANAPC	chr10	73994040	73994040	A	G	UTR3	ANAPC16	ENST000002	.	.	A	G	+	+	0.4375	9,0,7,0	chr10,73994	Cytosol	A > G
chr10:73994635:A:G:UTR3:ANAPC	chr10	73994635	73994635	A	G	UTR3	ANAPC16	ENST000002	.	.	A	G	+	+	0.13461538	45,0,7,0	chr10,73994	Cytosol	A > G
chr10:73994637:A:G:UTR3:ANAPC	chr10	73994637	73994637	A	G	UTR3	ANAPC16	ENST000002	.	.	A	G	+	+	0.25	39,0,13,0	chr10,73994	Cytosol	A > G
chr10:73994692:A:G:UTR3:ANAPC	chr10	73994692	73994692	A	G	UTR3	ANAPC16	ENST000002	.	.	A	G	+	+	0.3030303	23,0,10,0	chr10,73994	Cytosol	A > G
chr10:73994699:A:G:UTR3:ANAPC	chr10	73994699	73994699	A	G	UTR3	ANAPC16	ENST000002	.	.	A	G	+	+	0.2	32,0,8,0	chr10,73994	Cytosol	A > G
chr10:73994707:A:G:UTR3:ANAPC	chr10	73994707	73994707	A	G	UTR3	ANAPC16	ENST000002	.	.	A	G	+	+	0.35714286	27,0,15,0	chr10,73994	Cytosol	A > G
chr10:73994713:A:G:UTR3:ANAPC	chr10	73994713	73994713	A	G	UTR3	ANAPC16	ENST000002	.	.	A	G	+	+	0.34883721	28,0,15,0	chr10,73994	Cytosol	A > G
chr10:73994750:A:G:UTR3:ANAPC	chr10	73994750	73994750	A	G	UTR3	ANAPC16	ENST000002	.	.	A	G	+	+	0.03773585	51,0,2,0	chr10,73994	Cytosol	A > G
chr10:73994755:A:G:UTR3:ANAPC	chr10	73994755	73994755	A	G	UTR3	ANAPC16	ENST000002	.	.	A	G	+	+	0.03846154	50,0,2,0	chr10,73994	Cytosol	A > G
chr10:73994756:A:G:UTR3:ANAPC	chr10	73994756	73994756	A	G	UTR3	ANAPC16	ENST000002	.	.	A	G	+	+	0.08	46,0,4,0	chr10,73994	Cytosol	A > G
chr10:73994762:A:G:UTR3:ANAPC	chr10	73994762	73994762	A	G	UTR3	ANAPC16	ENST000002	.	.	A	G	+	+	0.24390244	31,0,10,0	chr10,73994	Cytosol	A > G
chr10:73994767:A:G:UTR3:ANAPC	chr10	73994767	73994767	A	G	UTR3	ANAPC16	ENST000002	.	.	A	G	+	+	0.09756098	37,0,4,0	chr10,73994	Cytosol	A > G
chr10:73994788:A:G:UTR3:ANAPC	chr10	73994788	73994788	A	G	UTR3	ANAPC16	ENST000002	.	.	A	G	+	+	0.18421053	31,0,7,0	chr10,73994	Cytosol	A > G
chr10:73994789:A:G:UTR3:ANAPC	chr10	73994789	73994789	A	G	UTR3	ANAPC16	ENST000002	.	.	A	G	+	+	0.26315789	28,0,10,0	chr10,73994	Cytosol	A > G
chr10:73994802:A:G:UTR3:ANAPC	chr10	73994802	73994802	A	G	UTR3	ANAPC16	ENST000002	.	.	A	G	+	+	0.20833333	19,0,5,0	chr10,73994	Cytosol	A > G
chr10:73994821:A:G:UTR3:ANAPC	chr10	73994821	73994821	A	G	UTR3	ANAPC16	ENST000002	.	.	A	G	+	+	0.0952381	19,0,2,0	chr10,73994	Cytosol	A > G
chr10:73994828:A:G:UTR3:ANAPC	chr10	73994828	73994828	A	G	UTR3	ANAPC16	ENST000002	.	.	A	G	+	+	0.41176471	10,0,7,0	chr10,73994	Cytosol	A > G
chr10:73994829:A:G:UTR3:ANAPC	chr10	73994829	73994829	A	G	UTR3	ANAPC16	ENST000002	.	.	A	G	+	+	0.22222222	14,0,4,0	chr10,73994	Cytosol	A > G
chr10:73994834:A:G:UTR3:ANAPC	chr10	73994834	73994834	A	G	UTR3	ANAPC16	ENST000002	.	.	A	G	+	+	0.47058824	9,0,8,0	chr10,73994	Cytosol	A > G
chr10:73994951:A:G:UTR3:ANAPC	chr10	73994951	73994951	A	G	UTR3	ANAPC16	ENST000002	.	.	A	G	+	+	0.18181818	9,0,2,0	chr10,73994	Cytosol	A > G
chr10:73994952:A:G:UTR3:ANAPC	chr10	73994952	73994952	A	G	UTR3	ANAPC16	ENST000002	.	.	A	G	+	+	0.38461538	8,0,5,0	chr10,73994	Cytosol	A > G
chr10:73995011:A:G:UTR3:ANAPC	chr10	73995011	73995011	A	G	UTR3	ANAPC16	ENST000002	.	.	A	G	+	+	0.17647059	14,0,3,0	chr10,73995	Cytosol	A > G
chr10:73995063:A:G:UTR3:ANAPC	chr10	73995063	73995063	A	G	UTR3	ANAPC16	ENST000002	.	.	A	G	+	+	0.875	1,0,7,0	chr10,73995	Cytosol	A > G
chr10:73995067:A:G:UTR3:ANAPC	chr10	73995067	73995067	A	G	UTR3	ANAPC16	ENST000002	.	.	A	G	+	+	0.2	8,0,2,0	chr10,73995	Cytosol	A > G
chr10:73995089:A:G:UTR3:ANAPC	chr10	73995089	73995089	A	G	UTR3	ANAPC16	ENST000002	.	.	A	G	+	+	0.42857143	8,0,6,0	chr10,73995	Cytosol	A > G
chr10:73995103:A:G:UTR3:ANAPC	chr10	73995103	73995103	A	G	UTR3	ANAPC16	ENST000002	.	.	A	G	+	+	0.1875	13,0,3,0	chr10,73995	Cytosol	A > G
chr10:73995138:A:G:UTR3:ANAPC	chr10	73995138	73995138	A	G	UTR3	ANAPC16	ENST000002	.	.	A	G	+	+	0.34482759	19,0,10,0	chr10,73995	Cytosol	A > G
chr10:73995193:A:G:UTR3:ANAPC	chr10	73995193	73995193	A	G	UTR3	ANAPC16	ENST000002	.	.	A	G	+	+	0.05405405	35,0,2,0	chr10,73995	Cytosol	A > G
chr10:75008817:T:C:ncRNA_exoni	chr10	75008817	75008817	T	C	ncRNA_exon	DNAJC9-AS1	.	.	.	A	G	-	+-	0.23244552	317,0,96,0	chr10,75008	Cytosol	A > G
chr10:75008845:T:C:ncRNA_exoni	chr10	75008845	75008845	T	C	ncRNA_exon	DNAJC9-AS1	.	.	.	A	G	-	+-	0.03729604	413,0,16,0	chr10,75008	Cytosol	A > G
chr10:75008873:T:C:ncRNA_exoni	chr10	75008873	75008873	T	C	ncRNA_exon	DNAJC9-AS1	.	.	.	A	G	-	+-	0.01612903	366,0,6,0	chr10,75008	Cytosol	A > G
chr10:75008955:T:C:ncRNA_exoni	chr10	75008955	75008955	T	C	ncRNA_exon	DNAJC9-AS1	.	.	.	A	G	-	+-	0.64049587	87,0,155,0	chr10,75008	Cytosol	A > G
chr10:75009585:T:C:ncRNA_intron	chr10	75009585	75009585	T	C	ncRNA_intro	DNAJC9-AS1	.	.	.	A	G	-	+-	0.17682927	135,0,29,0	chr10,75009	Cytosol	A > G
chr10:75009591:T:C:ncRNA_intron	chr10	75009591	75009591	T	C	ncRNA_intro	DNAJC9-AS1	.	.	.	A	G	-	+-	0.17575758	136,0,29,0	chr10,75009	Cytosol	A > G
chr10:75009720:T:C:ncRNA_intron	chr10	75009720	75009720	T	C	ncRNA_intro	DNAJC9-AS1	.	.	.	A	G	-	+-	0.18902439	133,0,31,0	chr10,75009	Cytosol	A > G
chr10:75009721:T:C:ncRNA_intron	chr10	75009721	75009721	T	C	ncRNA_intro	DNAJC9-AS1	.	.	.	A	G	-	+-	0.06214689	166,0,11,0	chr10,75009	Cytosol	A > G
chr10:75009844:T:C:ncRNA_intron	chr10	75009844	75009844	T	C	ncRNA_intro	DNAJC9-AS1	.	.	.	A	G	-	+-	0.10030395	296,0,33,0	chr10,75009	Cytosol	A > G
chr10:75009921:T:C:ncRNA_intron	chr10	75009921	75009921	T	C	ncRNA_intro	DNAJC9-AS1	.	.	.	A	G	-	+-	0.13043478	60,0,9,0	chr10,75009	Cytosol	A > G
chr10:75010087:T:C:ncRNA_intron	chr10	75010087	75010087	T	C	ncRNA_intro	DNAJC9-AS1	.	.	.	A	G	-	+-	0.07801418	130,0,11,0	chr10,75010	Cytosol	A > G
chr10:75536194:A:G:downstream:	chr10	75536194	75536194	A	G	downstream	FUT11	.	.	.	A	G	+	+	0.81818182	6,0,27,0	chr10,75536	Cytosol	A > G
chr10:75536285:A:G:downstream:	chr10	75536285	75536285	A	G	downstream	FUT11	.	.	.	A	G	+	+	0.27272727	16,0,6,0	chr10,75536	Cytosol	A > G
chr10:75536292:A:G:downstream:	chr10	75536292	75536292	A	G	downstream	FUT11	.	.	.	A	G	+	+	0.85714286	3,0,18,0	chr10,75536	Cytosol	A > G
chr10:75536458:A:G:downstream:	chr10	75536458	75536458	A	G	downstream	FUT11	.	.	.	A	G	+	+	0.28571429	5,0,2,0	chr10,75536	Cytosol	A > G

chr10:75536538:A:G:downstream:	chr10	75536538	75536538	A	G	downstream	FUT11	.	.	.	A	G	+	+	0.4	9,0,6,0	chr10,75536	Cytosol	A > G
chr10:75536653:A:G:downstream:	chr10	75536653	75536653	A	G	downstream	FUT11	.	.	.	A	G	+	+	0.25	6,0,2,0	chr10,75536	Cytosol	A > G
chr10:75537390:A:G:intergenic:FU	chr10	75537390	75537390	A	G	intergenic	FUT11,CHCH	dist=1413;dis	.	.	A	G	+	+	0.67647059	11,0,23,0	chr10,75537	Cytosol	A > G
chr10:75537412:A:G:intergenic:FU	chr10	75537412	75537412	A	G	intergenic	FUT11,CHCH	dist=1435;dis	.	.	A	G	+	+	0.23404255	36,0,11,0	chr10,75537	Cytosol	A > G
chr10:75537666:A:G:intergenic:FU	chr10	75537666	75537666	A	G	intergenic	FUT11,CHCH	dist=1689;dis	.	.	A	G	+	+	0.125	21,0,3,0	chr10,75537	Cytosol	A > G
chr10:75538625:A:G:intergenic:FU	chr10	75538625	75538625	A	G	intergenic	FUT11,CHCH	dist=2648;dis	.	.	A	G	+	+	0.25	12,0,4,0	chr10,75538	Cytosol	A > G
chr10:75539270:A:G:intergenic:FU	chr10	75539270	75539270	A	G	intergenic	FUT11,CHCH	dist=3293;dis	.	.	A	G	+	+	0.1875	13,0,3,0	chr10,75539	Cytosol	A > G
chr10:75539271:A:G:intergenic:FU	chr10	75539271	75539271	A	G	intergenic	FUT11,CHCH	dist=3294;dis	.	.	A	G	+	+	0.13333333	13,0,2,0	chr10,75539	Cytosol	A > G
chr10:82192135:A:G:UTR3:FAM21	chr10	82192135	82192135	A	G	UTR3	FAM213A	ENST000003	.	.	A	G	+	+	0.0483871	236,0,12,0	chr10,82192	Cytosol	A > G
chr10:82192142:A:G:UTR3:FAM21	chr10	82192142	82192142	A	G	UTR3	FAM213A	ENST000003	.	.	A	G	+	+	0.01384083	285,0,4,0	chr10,82192	Cytosol	A > G
chr10:82192276:A:G:UTR3:FAM21	chr10	82192276	82192276	A	G	UTR3	FAM213A	ENST000003	.	.	A	G	+	+	0.05654762	317,0,19,0	chr10,82192	Cytosol	A > G
chr10:82192303:A:G:UTR3:FAM21	chr10	82192303	82192303	A	G	UTR3	FAM213A	ENST000003	.	.	A	G	+	+	0.01807229	163,0,3,0	chr10,82192	Cytosol	A > G
chr10:82192318:A:G:UTR3:FAM21	chr10	82192318	82192318	A	G	UTR3	FAM213A	ENST000003	.	.	A	G	+	+	0.09036145	151,0,15,0	chr10,82192	Cytosol	A > G
chr10:82192352:A:G:UTR3:FAM21	chr10	82192352	82192352	A	G	UTR3	FAM213A	ENST000003	.	.	A	G	+	+	0.05777778	212,0,13,0	chr10,82192	Cytosol	A > G
chr10:82193810:A:G:upstream:RP	chr10	82193810	82193810	A	G	upstream	RP11-137H2.	.	.	.	A	G	+		0.36363636	7,0,4,0	chr10,82193	Cytosol	A > G
chr10:82194712:A:G:upstream:RP	chr10	82194712	82194712	A	G	upstream	RP11-137H2.	.	.	.	A	G	+		0.28571429	15,0,6,0	chr10,82194	Cytosol	A > G
chr10:82194849:A:G:ncRNA_exoni	chr10	82194849	82194849	A	G	ncRNA_exon	RP11-137H2.	.	.	.	A	G	+	+	0.11111111	16,0,2,0	chr10,82194	Cytosol	A > G
chr10:82194891:A:G:ncRNA_exoni	chr10	82194891	82194891	A	G	ncRNA_exon	RP11-137H2.	.	.	.	A	G	+	+	0.26666667	11,0,4,0	chr10,82194	Cytosol	A > G
chr10:82195359:A:G:ncRNA_exoni	chr10	82195359	82195359	A	G	ncRNA_exon	RP11-137H2.	.	.	.	A	G	+	+	0.13157895	33,0,5,0	chr10,82195	Cytosol	A > G
chr10:82195468:A:G:ncRNA_exoni	chr10	82195468	82195468	A	G	ncRNA_exon	RP11-137H2.	.	.	.	A	G	+	+	0.33333333	12,0,6,0	chr10,82195	Cytosol	A > G
chr10:82195480:A:G:ncRNA_exoni	chr10	82195480	82195480	A	G	ncRNA_exon	RP11-137H2.	.	.	.	A	G	+	+	0.26315789	14,0,5,0	chr10,82195	Cytosol	A > G
chr10:82195897:A:G:ncRNA_exoni	chr10	82195897	82195897	A	G	ncRNA_exon	RP11-137H2.	.	.	.	A	G	+	+	0.13333333	13,0,2,0	chr10,82195	Cytosol	A > G
chr10:82195918:A:G:ncRNA_exoni	chr10	82195918	82195918	A	G	ncRNA_exon	RP11-137H2.	.	.	.	A	G	+	+	0.92857143	1,0,13,0	chr10,82195	Cytosol	A > G
chr10:82195928:A:G:ncRNA_exoni	chr10	82195928	82195928	A	G	ncRNA_exon	RP11-137H2.	.	.	.	A	G	+	+	0.46666667	8,0,7,0	chr10,82195	Cytosol	A > G
chr10:82195929:A:G:ncRNA_exoni	chr10	82195929	82195929	A	G	ncRNA_exon	RP11-137H2.	.	.	.	A	G	+	+	0.17647059	14,0,3,0	chr10,82195	Cytosol	A > G
chr10:82195933:A:G:ncRNA_exoni	chr10	82195933	82195933	A	G	ncRNA_exon	RP11-137H2.	.	.	.	A	G	+	+	0.1875	13,0,3,0	chr10,82195	Cytosol	A > G
chr10:82195983:A:G:ncRNA_exoni	chr10	82195983	82195983	A	G	ncRNA_exon	RP11-137H2.	.	.	.	A	G	+	+	0.42105263	11,0,8,0	chr10,82195	Cytosol	A > G
chr10:82195988:A:G:ncRNA_exoni	chr10	82195988	82195988	A	G	ncRNA_exon	RP11-137H2.	.	.	.	A	G	+	+	0.35	13,0,7,0	chr10,82195	Cytosol	A > G
chr10:82196082:A:G:ncRNA_exoni	chr10	82196082	82196082	A	G	ncRNA_exon	RP11-137H2.	.	.	.	A	G	+	+	0.17391304	19,0,4,0	chr10,82196	Cytosol	A > G
chr10:82196134:A:G:ncRNA_exoni	chr10	82196134	82196134	A	G	ncRNA_exon	RP11-137H2.	.	.	.	A	G	+	+	0.07407407	25,0,2,0	chr10,82196	Cytosol	A > G
chr10:88686256:A:G:UTR3:BMPR1	chr10	88686256	88686256	A	G	UTR3	BMPR1A	ENST000003	.	.	A	G	+	+	0.15789474	16,0,3,0	chr10,88686	Cytosol	A > G
chr10:88968175:T:C:ncRNA_intror	chr10	88968175	88968175	T	C	ncRNA_intro	NUTM2A-AS	.	.	.	A	G	-	-	0.25	6,0,2,0	chr10,88968	Cytosol	A > G
chr10:92664448:A:G:intronic:RPP3	chr10	92664448	92664448	A	G	intronic	RPP30	.	.	.	A	G	+	+	0.4	3,0,2,0	chr10,92664	Cytosol	A > G
chr10:92664475:A:G:intronic:RPP3	chr10	92664475	92664475	A	G	intronic	RPP30	.	.	.	A	G	+	+	0.4	3,0,2,0	chr10,92664	Cytosol	A > G
chr10:96089296:T:C:UTR3:PLCE1	chr10	96089296	96089296	T	C	UTR3	PLCE1	ENST000003	.	.	A	G	-	+-	0.5	3,0,3,0	chr10,96089	Cytosol	A > G
chr10:96090347:T:C:UTR3:PLCE1	chr10	96090347	96090347	T	C	UTR3	PLCE1	ENST000003	.	.	A	G	-	+-	0.8	1,0,4,0	chr10,96090	Cytosol	A > G
chr10:96367532:A:G:ncRNA_intro	chr10	96367532	96367532	A	G	ncRNA_intro	RP11-119K6.	.	.	.	A	G	+	+	0.30769231	9,0,4,0	chr10,96367	Cytosol	A > G
chr10:96367614:A:G:ncRNA_intro	chr10	96367614	96367614	A	G	ncRNA_intro	RP11-119K6.	.	.	.	A	G	+	+	0.2	12,0,3,0	chr10,96367	Cytosol	A > G
chr10:96367624:A:G:ncRNA_intro	chr10	96367624	96367624	A	G	ncRNA_intro	RP11-119K6.	.	.	.	A	G	+	+	0.46666667	8,0,7,0	chr10,96367	Cytosol	A > G
chr10:96367665:A:G:splicing:HELL	chr10	96367665	96367665	A	G	splicing	HELLS	ENST000003	.	.	A	G	+	+	0.13333333	13,0,2,0	chr10,96367	Cytosol	A > G
chr10:96367668:A:G:exonic:HELLS	chr10	96367668	96367668	A	G	exonic	HELLS	.	synonymous	HELLS:ENST0	A	G	+	+	0.66666667	3,0,6,0	chr10,96367	Cytosol	A > G
chr10:96367674:A:G:exonic:HELLS	chr10	96367674	96367674	A	G	exonic	HELLS	.	synonymous	HELLS:ENST0	A	G	+	+	0.4	3,0,2,0	chr10,96367	Cytosol	A > G
chr10:96368963:A:G:ncRNA_intro	chr10	96368963	96368963	A	G	ncRNA_intro	RP11-119K6.	.	.	.	A	G	+	+	0.5	3,0,3,0	chr10,96368	Cytosol	A > G
chr10:96370092:A:G:ncRNA_intro	chr10	96370092	96370092	A	G	ncRNA_intro	RP11-119K6.	.	.	.	A	G	+	+	0.46153846	7,0,6,0	chr10,96370	Cytosol	A > G
chr10:96370093:A:G:ncRNA_intro	chr10	96370093	96370093	A	G	ncRNA_intro	RP11-119K6.	.	.	.	A	G	+	+	0.46153846	7,0,6,0	chr10,96370	Cytosol	A > G
chr10:96370530:A:G:ncRNA_intro	chr10	96370530	96370530	A	G	ncRNA_intro	RP11-119K6.	.	.	.	A	G	+	+	0.1	18,0,2,0	chr10,96370	Cytosol	A > G
chr10:96370556:A:G:ncRNA_splici	chr10	96370556	96370556	A	G	ncRNA_splici	RP11-119K6.	ENST000004	.	.	A	G	+	+	0.26666667	11,0,4,0	chr10,96370	Cytosol	A > G
chr10:96372419:A:G:UTR3:HELLS	chr10	96372419	96372419	A	G	UTR3	HELLS	ENST000003	.	.	A	G	+	+	0.08	23,0,2,0	chr10,96372	Cytosol	A > G
chr10:96372427:A:G:UTR3:HELLS	chr10	96372427	96372427	A	G	UTR3	HELLS	ENST000003	.	.	A	G	+	+	0.27586207	21,0,8,0	chr10,96372	Cytosol	A > G
chr10:96372443:A:G:UTR3:HELLS	chr10	96372443	96372443	A	G	UTR3	HELLS	ENST000003	.	.	A	G	+	+	0.13636364	19,0,3,0	chr10,96372	Cytosol	A > G
chr10:96372444:A:G:UTR3:HELLS	chr10	96372444	96372444	A	G	UTR3	HELLS	ENST000003	.	.	A	G	+	+	0.36363636	14,0,8,0	chr10,96372	Cytosol	A > G
chr10:96372483:A:G:UTR3:HELLS	chr10	96372483	96372483	A	G	UTR3	HELLS	ENST000003	.	.	A	G	+	+	0.29411765	24,0,10,0	chr10,96372	Cytosol	A > G
chr10:96372484:A:G:UTR3:HELLS	chr10	96372484	96372484	A	G	UTR3	HELLS	ENST000003	.	.	A	G	+	+	0.09090909	30,0,3,0	chr10,96372	Cytosol	A > G
chr10:96372487:A:G:UTR3:HELLS	chr10	96372487	96372487	A	G	UTR3	HELLS	ENST000003	.	.	A	G	+	+	0.06451613	29,0,2,0	chr10,96372	Cytosol	A > G
chr10:96372518:A:G:UTR3:HELLS	chr10	96372518	96372518	A	G	UTR3	HELLS	ENST000003	.	.	A	G	+	+	0.05882353	32,0,2,0	chr10,96372	Cytosol	A > G
chr10:96372562:A:G:UTR3:HELLS	chr10	96372562	96372562	A	G	UTR3	HELLS	ENST000003	.	.	A	G	+	+	0.28	18,0,7,0	chr10,96372	Cytosol	A > G
chr10:96372563:A:G:UTR3:HELLS	chr10	96372563	96372563	A	G	UTR3	HELLS	ENST000003	.	.	A	G	+	+	0.38461538	16,0,10,0	chr10,96372	Cytosol	A > G
chr10:96372568:A:G:UTR3:HELLS	chr10	96372568	96372568	A	G	UTR3	HELLS	ENST000003	.	.	A	G	+	+	0.30434783	16,0,7,0	chr10,96372	Cytosol	A > G
chr10:96372572:A:G:UTR3:HELLS	chr10	96372572	96372572	A	G	UTR3	HELLS	ENST000003	.	.	A	G	+	+	0.61538462	10,0,16,0	chr10,96372	Cytosol	A > G
chr10:96372611:A:G:UTR3:HELLS	chr10	96372611	96372611	A	G	UTR3	HELLS	ENST000003	.	.	A	G	+	+	0.1	27,0,3,0	chr10,96372	Cytosol	A > G
chr10:96372622:A:G:UTR3:HELLS	chr10	96372622	96372622	A	G	UTR3	HELLS	ENST000003	.	.	A	G	+	+	0.06666667	28,0,2,0	chr10,96372	Cytosol	A > G
chr10:96372630:A:G:UTR3:HELLS	chr10	96372630	96372630	A	G	UTR3	HELLS	ENST000003	.	.	A	G	+	+	0.20588235	27,0,7,0	chr10,96372	Cytosol	A > G
chr10:101992259:T:C:ncRNA_intro	chr10	101992259	101992259	T	C	ncRNA_intro	RP11-316M2	.	.	.	A	G	-	+-	0.10526316	34,0,4,0	chr10,10199	Cytosol	A > G
chr10:101992281:T:C:ncRNA_intro	chr10	101992281	101992281	T	C	ncRNA_intro	RP11-316M2	.	.	.	A	G	-	+-	0.41818182	32,0,23,0	chr10,10199	Cytosol	A > G

chr10:101992298:T:C:ncRNA_intro	chr10	101992298	101992298	T	C	ncRNA_intro	RP11-316M2	.	.	.	A	G	-	+-	0.07843137	94,0,8,0	chr10,10199	Cytosol	A > G
chr10:101992302:T:C:ncRNA_intro	chr10	101992302	101992302	T	C	ncRNA_intro	RP11-316M2	.	.	.	A	G	-	+-	0.08333333	99,0,9,0	chr10,10199	Cytosol	A > G
chr10:101992307:T:C:ncRNA_intro	chr10	101992307	101992307	T	C	ncRNA_intro	RP11-316M2	.	.	.	A	G	-	+-	0.76041667	23,0,73,0	chr10,10199	Cytosol	A > G
chr10:101992308:T:C:ncRNA_intro	chr10	101992308	101992308	T	C	ncRNA_intro	RP11-316M2	.	.	.	A	G	-	+-	1	0,0,96,0	chr10,10199	Cytosol	A > G
chr10:101992314:T:C:ncRNA_intro	chr10	101992314	101992314	T	C	ncRNA_intro	RP11-316M2	.	.	.	A	G	-	+-	0.49438202	45,0,44,0	chr10,10199	Cytosol	A > G
chr10:101992425:T:C:ncRNA_intro	chr10	101992425	101992425	T	C	ncRNA_intro	RP11-316M2	.	.	.	A	G	-	+-	0.19117647	55,0,13,0	chr10,10199	Cytosol	A > G
chr10:101992684:T:C:ncRNA_intro	chr10	101992684	101992684	T	C	ncRNA_intro	RP11-316M2	.	.	.	A	G	-	+-	0.02803738	208,0,6,0	chr10,10199	Cytosol	A > G
chr10:101992783:T:C:ncRNA_intro	chr10	101992783	101992783	T	C	ncRNA_intro	RP11-316M2	.	.	.	A	G	-	+-	0.23214286	129,0,39,0	chr10,10199	Cytosol	A > G
chr10:101992793:T:C:ncRNA_intro	chr10	101992793	101992793	T	C	ncRNA_intro	RP11-316M2	.	.	.	A	G	-	+-	0.3021978	127,0,55,0	chr10,10199	Cytosol	A > G
chr10:101992810:T:C:ncRNA_intro	chr10	101992810	101992810	T	C	ncRNA_intro	RP11-316M2	.	.	.	A	G	-	+-	0.56	66,0,84,0	chr10,10199	Cytosol	A > G
chr10:101992822:T:C:ncRNA_intro	chr10	101992822	101992822	T	C	ncRNA_intro	RP11-316M2	.	.	.	A	G	-	+-	0.0201005	195,0,4,0	chr10,10199	Cytosol	A > G
chr10:101992878:T:C:ncRNA_intro	chr10	101992878	101992878	T	C	ncRNA_intro	RP11-316M2	.	.	.	A	G	-	+-	0.13888889	93,0,15,0	chr10,10199	Cytosol	A > G
chr10:101992898:T:C:ncRNA_intro	chr10	101992898	101992898	T	C	ncRNA_intro	RP11-316M2	.	.	.	A	G	-	+-	0.20833333	38,0,10,0	chr10,10199	Cytosol	A > G
chr10:101992899:T:C:ncRNA_intro	chr10	101992899	101992899	T	C	ncRNA_intro	RP11-316M2	.	.	.	A	G	-	+-	0.15384615	33,0,6,0	chr10,10199	Cytosol	A > G
chr10:102764245:A:G:intronic:LZT	chr10	102764245	102764245	A	G	intronic	LZTS2	.	.	.	A	G	+	+	0.33333333	4,0,2,0	chr10,10276	Cytosol	A > G
chr10:104235928:A:G:UTR3:TMEN	chr10	104235928	104235928	A	G	UTR3	TMEM180	ENST000003	.	.	A	G	+	+	0.03174603	61,0,2,0	chr10,10423	Cytosol	A > G
chr10:104236039:A:G:UTR3:TMEN	chr10	104236039	104236039	A	G	UTR3	TMEM180	ENST000002	.	.	A	G	+	+	0.51351351	18,0,19,0	chr10,10423	Cytosol	A > G
chr10:104236045:A:G:UTR3:TMEN	chr10	104236045	104236045	A	G	UTR3	TMEM180	ENST000002	.	.	A	G	+	+	0.05128205	37,0,2,0	chr10,10423	Cytosol	A > G
chr10:104272769:A:G:intronic:SUF	chr10	104272769	104272769	A	G	intronic	SUFU	.	.	.	A	G	+	+	0.25	6,0,2,0	chr10,10427	Cytosol	A > G
chr10:104937357:T:C:intronic:NT5	chr10	104937357	104937357	T	C	intronic	NT5C2	.	.	.	A	G	-	-	0.11764706	15,0,2,0	chr10,10493	Cytosol	A > G
chr10:104937807:T:C:intronic:NT5	chr10	104937807	104937807	T	C	intronic	NT5C2	.	.	.	A	G	-	-	0.26315789	14,0,5,0	chr10,10493	Cytosol	A > G
chr10:104938079:T:C:intronic:NT5	chr10	104938079	104938079	T	C	intronic	NT5C2	.	.	.	A	G	-	-	0.4	9,0,6,0	chr10,10493	Cytosol	A > G
chr10:104938194:T:C:intronic:NT5	chr10	104938194	104938194	T	C	intronic	NT5C2	.	.	.	A	G	-	-	0.3	7,0,3,0	chr10,10493	Cytosol	A > G
chr10:104938422:T:C:intronic:NT5	chr10	104938422	104938422	T	C	intronic	NT5C2	.	.	.	A	G	-	-	0.8	2,0,8,0	chr10,10493	Cytosol	A > G
chr10:104938465:T:C:intronic:NT5	chr10	104938465	104938465	T	C	intronic	NT5C2	.	.	.	A	G	-	-	0.66666667	3,0,6,0	chr10,10493	Cytosol	A > G
chr10:104938475:T:C:intronic:NT5	chr10	104938475	104938475	T	C	intronic	NT5C2	.	.	.	A	G	-	-	0.3	7,0,3,0	chr10,10493	Cytosol	A > G
chr10:104938870:T:C:intronic:NT5	chr10	104938870	104938870	T	C	intronic	NT5C2	.	.	.	A	G	-	-	0.5	3,0,3,0	chr10,10493	Cytosol	A > G
chr10:104938913:T:C:intronic:NT5	chr10	104938913	104938913	T	C	intronic	NT5C2	.	.	.	A	G	-	-	0.22222222	7,0,2,0	chr10,10493	Cytosol	A > G
chr10:104938976:T:C:intronic:NT5	chr10	104938976	104938976	T	C	intronic	NT5C2	.	.	.	A	G	-	-	0.375	5,0,3,0	chr10,10493	Cytosol	A > G
chr10:104939349:T:C:intronic:NT5	chr10	104939349	104939349	T	C	intronic	NT5C2	.	.	.	A	G	-	-	0.15384615	11,0,2,0	chr10,10493	Cytosol	A > G
chr10:104940232:T:C:intronic:NT5	chr10	104940232	104940232	T	C	intronic	NT5C2	.	.	.	A	G	-	-	0.13333333	13,0,2,0	chr10,10494	Cytosol	A > G
chr10:112667144:T:C:intronic:BBII	chr10	112667144	112667144	T	C	intronic	BBIP1	.	.	.	A	G	-	-	0.8	1,0,4,0	chr10,11266	Cytosol	A > G
chr10:112667156:T:C:intronic:BBII	chr10	112667156	112667156	T	C	intronic	BBIP1	.	.	.	A	G	-	-	0.66666667	2,0,4,0	chr10,11266	Cytosol	A > G
chr10:112667161:T:C:intronic:BBII	chr10	112667161	112667161	T	C	intronic	BBIP1	.	.	.	A	G	-	-	0.42857143	4,0,3,0	chr10,11266	Cytosol	A > G
chr10:112667187:T:C:intronic:BBII	chr10	112667187	112667187	T	C	intronic	BBIP1	.	.	.	A	G	-	-	0.42857143	4,0,3,0	chr10,11266	Cytosol	A > G
chr10:114209254:A:G:intronic:VTI	chr10	114209254	114209254	A	G	intronic	VTI1A	.	.	.	A	G	+	+	0.6	6,0,9,0	chr10,11420	Cytosol	A > G
chr10:114209347:A:G:intronic:VTI	chr10	114209347	114209347	A	G	intronic	VTI1A	.	.	.	A	G	+	+	0.5	11,0,11,0	chr10,11420	Cytosol	A > G
chr10:114209352:A:G:intronic:VTI	chr10	114209352	114209352	A	G	intronic	VTI1A	.	.	.	A	G	+	+	0.22727273	17,0,5,0	chr10,11420	Cytosol	A > G
chr10:114209353:A:G:intronic:VTI	chr10	114209353	114209353	A	G	intronic	VTI1A	.	.	.	A	G	+	+	0.20833333	19,0,5,0	chr10,11420	Cytosol	A > G
chr10:114209390:A:G:intronic:VTI	chr10	114209390	114209390	A	G	intronic	VTI1A	.	.	.	A	G	+	+	0.5	11,0,11,0	chr10,11420	Cytosol	A > G
chr10:114209404:A:G:intronic:VTI	chr10	114209404	114209404	A	G	intronic	VTI1A	.	.	.	A	G	+	+	0.125	14,0,2,0	chr10,11420	Cytosol	A > G
chr10:114209432:A:G:intronic:VTI	chr10	114209432	114209432	A	G	intronic	VTI1A	.	.	.	A	G	+	+	0.25	6,0,2,0	chr10,11420	Cytosol	A > G
chr10:114209768:A:G:intronic:VTI	chr10	114209768	114209768	A	G	intronic	VTI1A	.	.	.	A	G	+	+	0.25	27,0,9,0	chr10,11420	Cytosol	A > G
chr10:114209859:A:G:intronic:VTI	chr10	114209859	114209859	A	G	intronic	VTI1A	.	.	.	A	G	+	+	0.15	17,0,3,0	chr10,11420	Cytosol	A > G
chr10:118590064:T:C:intergenic:H	chr10	118590064	118590064	T	C	intergenic	HSPA12A,RP	dist=87979;d	.	.	A	G	-	-	0.2	8,0,2,0	chr10,11859	Cytosol	A > G
chr10:118590081:T:C:intergenic:H	chr10	118590081	118590081	T	C	intergenic	HSPA12A,RP	dist=87996;d	.	.	A	G	-	-	0.2	8,0,2,0	chr10,11859	Cytosol	A > G
chr10:118590152:T:C:intergenic:H	chr10	118590152	118590152	T	C	intergenic	HSPA12A,RP	dist=88067;d	.	.	A	G	-	-	0.76923077	3,0,10,0	chr10,11859	Cytosol	A > G
chr10:118590160:T:C:intergenic:H	chr10	118590160	118590160	T	C	intergenic	HSPA12A,RP	dist=88075;d	.	.	A	G	-	-	0.81818182	2,0,9,0	chr10,11859	Cytosol	A > G
chr10:118591807:T:C:downstrean	chr10	118591807	118591807	T	C	downstream	RP11-539I5.1	.	.	.	A	G	-	-	0.26666667	11,0,4,0	chr10,11859	Cytosol	A > G
chr10:118591817:T:C:downstrean	chr10	118591817	118591817	T	C	downstream	RP11-539I5.1	.	.	.	A	G	-	-	0.64285714	5,0,9,0	chr10,11859	Cytosol	A > G
chr10:118591827:T:C:downstrean	chr10	118591827	118591827	T	C	downstream	RP11-539I5.1	.	.	.	A	G	-	-	0.8	2,0,8,0	chr10,11859	Cytosol	A > G
chr10:121331822:T:C:intergenic:R	chr10	121331822	121331822	T	C	intergenic	RGS10,TIAL1	dist=29602;d	.	.	A	G	-		0.85	3,0,17,0	chr10,12133	Cytosol	A > G
chr10:121331865:T:C:intergenic:R	chr10	121331865	121331865	T	C	intergenic	RGS10,TIAL1	dist=29645;d	.	.	A	G	-		0.33333333	16,0,8,0	chr10,12133	Cytosol	A > G
chr10:121331866:T:C:intergenic:R	chr10	121331866	121331866	T	C	intergenic	RGS10,TIAL1	dist=29646;d	.	.	A	G	-		0.65217391	8,0,15,0	chr10,12133	Cytosol	A > G
chr10:121331895:T:C:intergenic:R	chr10	121331895	121331895	T	C	intergenic	RGS10,TIAL1	dist=29675;d	.	.	A	G	-		0.28571429	20,0,8,0	chr10,12133	Cytosol	A > G
chr10:121331945:T:C:intergenic:R	chr10	121331945	121331945	T	C	intergenic	RGS10,TIAL1	dist=29725;d	.	.	A	G	-		0.18181818	18,0,4,0	chr10,12133	Cytosol	A > G
chr10:121331963:T:C:intergenic:R	chr10	121331963	121331963	T	C	intergenic	RGS10,TIAL1	dist=29743;d	.	.	A	G	-		0.29411765	12,0,5,0	chr10,12133	Cytosol	A > G
chr10:121331969:T:C:intergenic:R	chr10	121331969	121331969	T	C	intergenic	RGS10,TIAL1	dist=29749;d	.	.	A	G	-		0.4375	9,0,7,0	chr10,12133	Cytosol	A > G
chr10:121332087:T:C:intergenic:R	chr10	121332087	121332087	T	C	intergenic	RGS10,TIAL1	dist=29867;d	.	.	A	G	-		0.27272727	8,0,3,0	chr10,12133	Cytosol	A > G
chr10:121332119:T:C:intergenic:R	chr10	121332119	121332119	T	C	intergenic	RGS10,TIAL1	dist=29899;d	.	.	A	G	-		0.90909091	1,0,10,0	chr10,12133	Cytosol	A > G
chr10:121332403:T:C:intergenic:R	chr10	121332403	121332403	T	C	intergenic	RGS10,TIAL1	dist=30183;d	.	.	A	G	-		1	0,0,10,0	chr10,12133	Cytosol	A > G
chr10:121332406:T:C:intergenic:R	chr10	121332406	121332406	T	C	intergenic	RGS10,TIAL1	dist=30186;d	.	.	A	G	-		0.66666667	4,0,8,0	chr10,12133	Cytosol	A > G
chr10:121332485:T:C:intergenic:R	chr10	121332485	121332485	T	C	intergenic	RGS10,TIAL1	dist=30265;d	.	.	A	G	-		0.4	3,0,2,0	chr10,12133	Cytosol	A > G
chr10:121332757:T:C:intergenic:R	chr10	121332757	121332757	T	C	intergenic	RGS10,TIAL1	dist=30537;d	.	.	A	G	-		0.4	9,0,6,0	chr10,12133	Cytosol	A > G

chr10:121332786:T:C:intergenic:R	chr10	121332786	121332786	T	C	intergenic	RGS10,TIAL1	dist=30566;d	.	.	A	G	-		0.20689655	23,0,6,0	chr10,12133	Cytosol	A > G
chr10:121332787:T:C:intergenic:R	chr10	121332787	121332787	T	C	intergenic	RGS10,TIAL1	dist=30567;d	.	.	A	G	-		0.08333333	22,0,2,0	chr10,12133	Cytosol	A > G
chr10:121332793:T:C:intergenic:R	chr10	121332793	121332793	T	C	intergenic	RGS10,TIAL1	dist=30573;d	.	.	A	G	-		0.3	21,0,9,0	chr10,12133	Cytosol	A > G
chr10:121332831:T:C:intergenic:R	chr10	121332831	121332831	T	C	intergenic	RGS10,TIAL1	dist=30611;d	.	.	A	G	-		0.44117647	19,0,15,0	chr10,12133	Cytosol	A > G
chr10:126451032:T:C:exonic:METT	chr10	126451032	126451032	T	C	exonic	METTL10	.	nonsynonym	METTL10:EN	A	G	-	-	0.02531646	77,0,2,0	chr10,12645	Cytosol	A > G
chr10:127480558:T:C:intronic:URO	chr10	127480558	127480558	T	C	intronic	UROS	.	.	.	A	G	-	-	0.10810811	33,0,4,0	chr10,12748	Cytosol	A > G
chr10:134144104:T:C:intronic:STK	chr10	134144104	134144104	T	C	intronic	STK32C	.	.	.	A	G	-	-	0.42857143	4,0,3,0	chr10,13414	Cytosol	A > G
chr11:456429:A:G:intronic:PTDSS2	chr11	456429	456429	A	G	intronic	PTDSS2	.	.	.	A	G	+	+	0.33333333	4,0,2,0	chr11,45642	Cytosol	A > G
chr11:767880:T:C:UTR3:PDDC1	chr11	767880	767880	T	C	UTR3	PDDC1	ENST000003	.	.	A	G	-	-	0.44	14,0,11,0	chr11,76787	Cytosol	A > G
chr11:767881:T:C:UTR3:PDDC1	chr11	767881	767881	T	C	UTR3	PDDC1	ENST000003	.	.	A	G	-	-	0.33333333	16,0,8,0	chr11,76788	Cytosol	A > G
chr11:767886:T:C:UTR3:PDDC1	chr11	767886	767886	T	C	UTR3	PDDC1	ENST000003	.	.	A	G	-	-	0.39130435	14,0,9,0	chr11,76788	Cytosol	A > G
chr11:767927:T:C:UTR3:PDDC1	chr11	767927	767927	T	C	UTR3	PDDC1	ENST000003	.	.	A	G	-	-	0.28571429	5,0,2,0	chr11,76792	Cytosol	A > G
chr11:767943:T:C:UTR3:PDDC1	chr11	767943	767943	T	C	UTR3	PDDC1	ENST000003	.	.	A	G	-	-	0.4	3,0,2,0	chr11,76794	Cytosol	A > G
chr11:768312:T:C:UTR3:PDDC1	chr11	768312	768312	T	C	UTR3	PDDC1	ENST000003	.	.	A	G	-	-	0.5	4,0,4,0	chr11,76831	Cytosol	A > G
chr11:768321:T:C:UTR3:PDDC1	chr11	768321	768321	T	C	UTR3	PDDC1	ENST000003	.	.	A	G	-	-	0.8	1,0,4,0	chr11,76832	Cytosol	A > G
chr11:768347:T:C:UTR3:PDDC1	chr11	768347	768347	T	C	UTR3	PDDC1	ENST000003	.	.	A	G	-	-	0.45454545	6,0,5,0	chr11,76834	Cytosol	A > G
chr11:768383:T:C:UTR3:PDDC1	chr11	768383	768383	T	C	UTR3	PDDC1	ENST000003	.	.	A	G	-	-	0.35294118	11,0,6,0	chr11,76838	Cytosol	A > G
chr11:768386:T:C:UTR3:PDDC1	chr11	768386	768386	T	C	UTR3	PDDC1	ENST000003	.	.	A	G	-	-	0.76470588	4,0,13,0	chr11,76838	Cytosol	A > G
chr11:768393:T:C:UTR3:PDDC1	chr11	768393	768393	T	C	UTR3	PDDC1	ENST000003	.	.	A	G	-	-	0.3125	11,0,5,0	chr11,76839	Cytosol	A > G
chr11:768710:T:C:UTR3:PDDC1	chr11	768710	768710	T	C	UTR3	PDDC1	ENST000003	.	.	A	G	-	-	0.4	3,0,2,0	chr11,76870	Cytosol	A > G
chr11:768856:T:C:UTR3:PDDC1	chr11	768856	768856	T	C	UTR3	PDDC1	ENST000003	.	.	A	G	-	-	0.4	3,0,2,0	chr11,76885	Cytosol	A > G
chr11:768977:T:C:UTR3:PDDC1	chr11	768977	768977	T	C	UTR3	PDDC1	ENST000003	.	.	A	G	-	-	0.3	7,0,3,0	chr11,76897	Cytosol	A > G
chr11:768979:T:C:UTR3:PDDC1	chr11	768979	768979	T	C	UTR3	PDDC1	ENST000003	.	.	A	G	-	-	0.36363636	7,0,4,0	chr11,76897	Cytosol	A > G
chr11:769018:T:C:UTR3:PDDC1	chr11	769018	769018	T	C	UTR3	PDDC1	ENST000003	.	.	A	G	-	-	0.61111111	7,0,11,0	chr11,76901	Cytosol	A > G
chr11:769019:T:C:UTR3:PDDC1	chr11	769019	769019	T	C	UTR3	PDDC1	ENST000003	.	.	A	G	-	-	0.4375	9,0,7,0	chr11,76901	Cytosol	A > G
chr11:769054:T:C:UTR3:PDDC1	chr11	769054	769054	T	C	UTR3	PDDC1	ENST000003	.	.	A	G	-	-	0.84615385	2,0,11,0	chr11,76905	Cytosol	A > G
chr11:769055:T:C:UTR3:PDDC1	chr11	769055	769055	T	C	UTR3	PDDC1	ENST000003	.	.	A	G	-	-	0.4	9,0,6,0	chr11,76905	Cytosol	A > G
chr11:769057:T:C:UTR3:PDDC1	chr11	769057	769057	T	C	UTR3	PDDC1	ENST000003	.	.	A	G	-	-	0.33333333	10,0,5,0	chr11,76905	Cytosol	A > G
chr11:769388:T:C:UTR3:PDDC1	chr11	769388	769388	T	C	UTR3	PDDC1	ENST000003	.	.	A	G	-	-	0.22222222	14,0,4,0	chr11,76938	Cytosol	A > G
chr11:769393:T:C:UTR3:PDDC1	chr11	769393	769393	T	C	UTR3	PDDC1	ENST000003	.	.	A	G	-	-	0.1875	13,0,3,0	chr11,76939	Cytosol	A > G
chr11:769397:T:C:UTR3:PDDC1	chr11	769397	769397	T	C	UTR3	PDDC1	ENST000003	.	.	A	G	-	-	0.16666667	15,0,3,0	chr11,76939	Cytosol	A > G
chr11:769403:T:C:UTR3:PDDC1	chr11	769403	769403	T	C	UTR3	PDDC1	ENST000003	.	.	A	G	-	-	0.85714286	2,0,12,0	chr11,76940	Cytosol	A > G
chr11:769426:T:C:UTR3:PDDC1	chr11	769426	769426	T	C	UTR3	PDDC1	ENST000003	.	.	A	G	-	-	0.07142857	26,0,2,0	chr11,76942	Cytosol	A > G
chr11:769459:T:C:UTR3:PDDC1	chr11	769459	769459	T	C	UTR3	PDDC1	ENST000003	.	.	A	G	-	-	0.18367347	40,0,9,0	chr11,76945	Cytosol	A > G
chr11:769469:T:C:UTR3:PDDC1	chr11	769469	769469	T	C	UTR3	PDDC1	ENST000003	.	.	A	G	-	-	0.10638298	42,0,5,0	chr11,76946	Cytosol	A > G
chr11:769488:T:C:UTR3:PDDC1	chr11	769488	769488	T	C	UTR3	PDDC1	ENST000003	.	.	A	G	-	-	0.04444444	43,0,2,0	chr11,76948	Cytosol	A > G
chr11:769489:T:C:UTR3:PDDC1	chr11	769489	769489	T	C	UTR3	PDDC1	ENST000003	.	.	A	G	-	-	0.125	42,0,6,0	chr11,76948	Cytosol	A > G
chr11:769491:T:C:UTR3:PDDC1	chr11	769491	769491	T	C	UTR3	PDDC1	ENST000003	.	.	A	G	-	-	0.06	47,0,3,0	chr11,76949	Cytosol	A > G
chr11:769536:T:C:UTR3:PDDC1	chr11	769536	769536	T	C	UTR3	PDDC1	ENST000003	.	.	A	G	-	-	0.24137931	22,0,7,0	chr11,76953	Cytosol	A > G
chr11:769540:T:C:UTR3:PDDC1	chr11	769540	769540	T	C	UTR3	PDDC1	ENST000003	.	.	A	G	-	-	0.10714286	25,0,3,0	chr11,76953	Cytosol	A > G
chr11:769560:T:C:UTR3:PDDC1	chr11	769560	769560	T	C	UTR3	PDDC1	ENST000003	.	.	A	G	-	-	0.21428571	11,0,3,0	chr11,76955	Cytosol	A > G
chr11:769581:T:C:UTR3:PDDC1	chr11	769581	769581	T	C	UTR3	PDDC1	ENST000003	.	.	A	G	-	-	0.30769231	9,0,4,0	chr11,76958	Cytosol	A > G
chr11:769721:T:C:UTR3:PDDC1	chr11	769721	769721	T	C	UTR3	PDDC1	ENST000003	.	.	A	G	-	-	0.4	3,0,2,0	chr11,76972	Cytosol	A > G
chr11:769738:T:C:UTR3:PDDC1	chr11	769738	769738	T	C	UTR3	PDDC1	ENST000003	.	.	A	G	-	-	0.28571429	5,0,2,0	chr11,76973	Cytosol	A > G
chr11:769748:T:C:UTR3:PDDC1	chr11	769748	769748	T	C	UTR3	PDDC1	ENST000003	.	.	A	G	-	-	0.44444444	5,0,4,0	chr11,76974	Cytosol	A > G
chr11:769883:T:C:UTR3:PDDC1	chr11	769883	769883	T	C	UTR3	PDDC1	ENST000003	.	.	A	G	-	-	0.52941176	8,0,9,0	chr11,76988	Cytosol	A > G
chr11:769895:T:C:UTR3:PDDC1	chr11	769895	769895	T	C	UTR3	PDDC1	ENST000003	.	.	A	G	-	-	0.3	14,0,6,0	chr11,76989	Cytosol	A > G
chr11:769905:T:C:UTR3:PDDC1	chr11	769905	769905	T	C	UTR3	PDDC1	ENST000003	.	.	A	G	-	-	0.0952381	19,0,2,0	chr11,76990	Cytosol	A > G
chr11:868410:T:C:UTR3:CHID1	chr11	868410	868410	T	C	UTR3	CHID1	ENST000004	.	.	A	G	-	-	0.4	3,0,2,0	chr11,86840	Cytosol	A > G
chr11:3719660:T:C:intronic:NUP9	chr11	3719660	3719660	T	C	intronic	NUP98	.	.	.	A	G	-	-	0.48	13,0,12,0	chr11,37196	Cytosol	A > G
chr11:3719661:T:C:intronic:NUP9	chr11	3719661	3719661	T	C	intronic	NUP98	.	.	.	A	G	-	-	0.25	18,0,6,0	chr11,37196	Cytosol	A > G
chr11:3719692:T:C:intronic:NUP9	chr11	3719692	3719692	T	C	intronic	NUP98	.	.	.	A	G	-	-	0.51515152	16,0,17,0	chr11,37196	Cytosol	A > G
chr11:3719701:T:C:intronic:NUP9	chr11	3719701	3719701	T	C	intronic	NUP98	.	.	.	A	G	-	-	0.52941176	16,0,18,0	chr11,37197	Cytosol	A > G
chr11:3719713:T:C:intronic:NUP9	chr11	3719713	3719713	T	C	intronic	NUP98	.	.	.	A	G	-	-	0.63333333	11,0,19,0	chr11,37197	Cytosol	A > G
chr11:3720052:T:C:intronic:NUP9	chr11	3720052	3720052	T	C	intronic	NUP98	.	.	.	A	G	-	-	0.14285714	24,0,4,0	chr11,37200	Cytosol	A > G
chr11:3720138:T:C:intronic:NUP9	chr11	3720138	3720138	T	C	intronic	NUP98	.	.	.	A	G	-	-	0.80952381	4,0,17,0	chr11,37201	Cytosol	A > G
chr11:8707822:A:G:UTR3:RPL27A	chr11	8707822	8707822	A	G	UTR3	RPL27A	ENST000003	.	.	A	G	+	+	0.23913043	35,0,11,0	chr11,87078	Cytosol	A > G
chr11:8707840:A:G:UTR3:RPL27A	chr11	8707840	8707840	A	G	UTR3	RPL27A	ENST000003	.	.	A	G	+	+	0.52564103	37,0,41,0	chr11,87078	Cytosol	A > G
chr11:8707907:A:G:UTR3:RPL27A	chr11	8707907	8707907	A	G	UTR3	RPL27A	ENST000003	.	.	A	G	+	+	0.11494253	154,0,20,0	chr11,87079	Cytosol	A > G
chr11:8707908:A:G:UTR3:RPL27A	chr11	8707908	8707908	A	G	UTR3	RPL27A	ENST000003	.	.	A	G	+	+	0.1402439	141,0,23,0	chr11,87079	Cytosol	A > G
chr11:8707917:A:G:UTR3:RPL27A	chr11	8707917	8707917	A	G	UTR3	RPL27A	ENST000003	.	.	A	G	+	+	0.11445783	147,0,19,0	chr11,87079	Cytosol	A > G
chr11:8707944:A:G:UTR3:RPL27A	chr11	8707944	8707944	A	G	UTR3	RPL27A	ENST000003	.	.	A	G	+	+	0.04545455	126,0,6,0	chr11,87079	Cytosol	A > G
chr11:8707952:A:G:UTR3:RPL27A	chr11	8707952	8707952	A	G	UTR3	RPL27A	ENST000003	.	.	A	G	+	+	0.05454545	104,0,6,0	chr11,87079	Cytosol	A > G
chr11:8707958:A:G:UTR3:RPL27A	chr11	8707958	8707958	A	G	UTR3	RPL27A	ENST000003	.	.	A	G	+	+	0.03603604	107,0,4,0	chr11,87079	Cytosol	A > G

chr11:8707961:A:G:UTR3:RPL27A	chr11	8707961	8707961	A	G	UTR3	RPL27A	ENST000003	.	.	A	G	+	+	0.14893617	80,0,14,0	chr11,87079	Cytosol	A > G
chr11:8708757:A:G:UTR3:RPL27A	chr11	8708757	8708757	A	G	UTR3	RPL27A	ENST000003	.	.	A	G	+	+	0.0295858	164,0,5,0	chr11,87087	Cytosol	A > G
chr11:8708771:A:G:UTR3:RPL27A	chr11	8708771	8708771	A	G	UTR3	RPL27A	ENST000003	.	.	A	G	+	+	0.04678363	163,0,8,0	chr11,87087	Cytosol	A > G
chr11:8708782:A:G:UTR3:RPL27A	chr11	8708782	8708782	A	G	UTR3	RPL27A	ENST000003	.	.	A	G	+	+	0.63372093	63,0,109,0	chr11,87087	Cytosol	A > G
chr11:8708785:A:G:UTR3:RPL27A	chr11	8708785	8708785	A	G	UTR3	RPL27A	ENST000003	.	.	A	G	+	+	0.22147651	116,0,33,0	chr11,87087	Cytosol	A > G
chr11:8708795:A:G:UTR3:RPL27A	chr11	8708795	8708795	A	G	UTR3	RPL27A	ENST000003	.	.	A	G	+	+	0.04587156	104,0,5,0	chr11,87087	Cytosol	A > G
chr11:8708796:A:G:UTR3:RPL27A	chr11	8708796	8708796	A	G	UTR3	RPL27A	ENST000003	.	.	A	G	+	+	0.13265306	85,0,13,0	chr11,87087	Cytosol	A > G
chr11:8708799:A:G:UTR3:RPL27A	chr11	8708799	8708799	A	G	UTR3	RPL27A	ENST000003	.	.	A	G	+	+	0.25555556	67,0,23,0	chr11,87087	Cytosol	A > G
chr11:8708825:A:G:UTR3:RPL27A	chr11	8708825	8708825	A	G	UTR3	RPL27A	ENST000003	.	.	A	G	+	+	0.84722222	11,0,61,0	chr11,87088	Cytosol	A > G
chr11:8708884:A:G:UTR3:RPL27A	chr11	8708884	8708884	A	G	UTR3	RPL27A	ENST000003	.	.	A	G	+	+	0.06818182	41,0,3,0	chr11,87088	Cytosol	A > G
chr11:8708889:A:G:UTR3:RPL27A	chr11	8708889	8708889	A	G	UTR3	RPL27A	ENST000003	.	.	A	G	+	+	0.39534884	26,0,17,0	chr11,87088	Cytosol	A > G
chr11:8708891:A:G:UTR3:RPL27A	chr11	8708891	8708891	A	G	UTR3	RPL27A	ENST000003	.	.	A	G	+	+	0.45238095	23,0,19,0	chr11,87088	Cytosol	A > G
chr11:9771767:A:G:UTR3:SWAP70	chr11	9771767	9771767	A	G	UTR3	SWAP70	ENST000004	.	.	A	G	+	+	0.05952381	79,0,5,0	chr11,97717	Cytosol	A > G
chr11:9771768:A:G:UTR3:SWAP70	chr11	9771768	9771768	A	G	UTR3	SWAP70	ENST000004	.	.	A	G	+	+	0.12345679	71,0,10,0	chr11,97717	Cytosol	A > G
chr11:9831568:A:G:ncRNA_intron	chr11	9831568	9831568	A	G	ncRNA_intro	SBF2-AS1	.	.	.	A	G	+	+-	0.83333333	1,0,5,0	chr11,98315	Cytosol	A > G
chr11:14297917:T:C:intergenic:RP	chr11	14297917	14297917	T	C	intergenic	RP11-21L19.	dist=2680;dis	.	.	A	G	-		0.22222222	7,0,2,0	chr11,14297	Cytosol	A > G
chr11:14297932:T:C:intergenic:RP	chr11	14297932	14297932	T	C	intergenic	RP11-21L19.	dist=2695;dis	.	.	A	G	-		0.55555556	4,0,5,0	chr11,14297	Cytosol	A > G
chr11:16777886:A:G:downstream:	chr11	16777886	16777886	A	G	downstream	C11orf58	.	.	.	A	G	+	+	0.96	3,0,72,0	chr11,16777	Cytosol	A > G
chr11:16777910:A:G:downstream:	chr11	16777910	16777910	A	G	downstream	C11orf58	.	.	.	A	G	+	+	0.69135802	25,0,56,0	chr11,16777	Cytosol	A > G
chr11:16777916:A:G:downstream:	chr11	16777916	16777916	A	G	downstream	C11orf58	.	.	.	A	G	+	+	0.37662338	48,0,29,0	chr11,16777	Cytosol	A > G
chr11:16777917:A:G:downstream:	chr11	16777917	16777917	A	G	downstream	C11orf58	.	.	.	A	G	+	+	0.49315068	37,0,36,0	chr11,16777	Cytosol	A > G
chr11:16777942:A:G:downstream:	chr11	16777942	16777942	A	G	downstream	C11orf58	.	.	.	A	G	+	+	0.58227848	33,0,46,0	chr11,16777	Cytosol	A > G
chr11:16777943:A:G:downstream:	chr11	16777943	16777943	A	G	downstream	C11orf58	.	.	.	A	G	+	+	0.18292683	67,0,15,0	chr11,16777	Cytosol	A > G
chr11:16777946:A:G:downstream:	chr11	16777946	16777946	A	G	downstream	C11orf58	.	.	.	A	G	+	+	0.08536585	75,0,7,0	chr11,16777	Cytosol	A > G
chr11:16777956:A:G:downstream:	chr11	16777956	16777956	A	G	downstream	C11orf58	.	.	.	A	G	+	+	0.35365854	53,0,29,0	chr11,16777	Cytosol	A > G
chr11:16777982:A:G:downstream:	chr11	16777982	16777982	A	G	downstream	C11orf58	.	.	.	A	G	+	+	0.07142857	78,0,6,0	chr11,16777	Cytosol	A > G
chr11:16777983:A:G:downstream:	chr11	16777983	16777983	A	G	downstream	C11orf58	.	.	.	A	G	+	+	0.34146341	53,0,28,1	chr11,16777	Cytosol	A > G
chr11:16777998:A:G:downstream:	chr11	16777998	16777998	A	G	downstream	C11orf58	.	.	.	A	G	+	+	0.42028986	40,0,29,0	chr11,16777	Cytosol	A > G
chr11:16778023:A:G:downstream:	chr11	16778023	16778023	A	G	downstream	C11orf58	.	.	.	A	G	+	+	0.73809524	11,0,31,0	chr11,16778	Cytosol	A > G
chr11:16778077:A:G:downstream:	chr11	16778077	16778077	A	G	downstream	C11orf58	.	.	.	A	G	+	+	0.37037037	17,0,10,0	chr11,16778	Cytosol	A > G
chr11:16778078:A:G:downstream:	chr11	16778078	16778078	A	G	downstream	C11orf58	.	.	.	A	G	+	+	0.58333333	10,0,14,0	chr11,16778	Cytosol	A > G
chr11:16778194:A:G:downstream:	chr11	16778194	16778194	A	G	downstream	C11orf58	.	.	.	A	G	+	+	0.5	26,0,26,0	chr11,16778	Cytosol	A > G
chr11:16778269:A:G:downstream:	chr11	16778269	16778269	A	G	downstream	C11orf58	.	.	.	A	G	+	+	0.62711864	22,0,37,0	chr11,16778	Cytosol	A > G
chr11:16778270:A:G:downstream:	chr11	16778270	16778270	A	G	downstream	C11orf58	.	.	.	A	G	+	+	0.84210526	9,0,48,0	chr11,16778	Cytosol	A > G
chr11:16778308:A:G:downstream:	chr11	16778308	16778308	A	G	downstream	C11orf58	.	.	.	A	G	+	+	0.45652174	25,0,21,0	chr11,16778	Cytosol	A > G
chr11:16778451:A:G:downstream:	chr11	16778451	16778451	A	G	downstream	C11orf58	.	.	.	A	G	+	+	0.22916667	74,0,22,0	chr11,16778	Cytosol	A > G
chr11:17398771:A:G:UTR3:NCR3L	chr11	17398771	17398771	A	G	UTR3	NCR3LG1	ENST000003	.	.	A	G	+	+	0.0625	60,0,4,0	chr11,17398	Cytosol	A > G
chr11:17398814:A:G:UTR3:NCR3L	chr11	17398814	17398814	A	G	UTR3	NCR3LG1	ENST000003	.	.	A	G	+	+	0.04444444	43,0,2,0	chr11,17398	Cytosol	A > G
chr11:31452585:A:G:UTR3:DNAJC	chr11	31452585	31452585	A	G	UTR3	DNAJC24	ENST000004	.	.	A	G	+	+	0.11111111	24,0,3,0	chr11,31452	Cytosol	A > G
chr11:31452598:A:G:UTR3:DNAJC	chr11	31452598	31452598	A	G	UTR3	DNAJC24	ENST000004	.	.	A	G	+	+	0.48387097	16,0,15,0	chr11,31452	Cytosol	A > G
chr11:31452636:A:G:UTR3:DNAJC	chr11	31452636	31452636	A	G	UTR3	DNAJC24	ENST000004	.	.	A	G	+	+	0.8372093	7,0,36,0	chr11,31452	Cytosol	A > G
chr11:31452667:A:G:UTR3:DNAJC	chr11	31452667	31452667	A	G	UTR3	DNAJC24	ENST000004	.	.	A	G	+	+	0.125	42,0,6,0	chr11,31452	Cytosol	A > G
chr11:31452684:A:G:UTR3:DNAJC	chr11	31452684	31452684	A	G	UTR3	DNAJC24	ENST000004	.	.	A	G	+	+	0.29545455	31,0,13,0	chr11,31452	Cytosol	A > G
chr11:31452694:A:G:UTR3:DNAJC	chr11	31452694	31452694	A	G	UTR3	DNAJC24	ENST000004	.	.	A	G	+	+	0.14	43,0,7,0	chr11,31452	Cytosol	A > G
chr11:31452696:A:G:UTR3:DNAJC	chr11	31452696	31452696	A	G	UTR3	DNAJC24	ENST000004	.	.	A	G	+	+	0.04081633	47,0,2,0	chr11,31452	Cytosol	A > G
chr11:31452728:A:G:UTR3:DNAJC	chr11	31452728	31452728	A	G	UTR3	DNAJC24	ENST000004	.	.	A	G	+	+	0.06896552	27,0,2,0	chr11,31452	Cytosol	A > G
chr11:31452787:A:G:UTR3:DNAJC	chr11	31452787	31452787	A	G	UTR3	DNAJC24	ENST000004	.	.	A	G	+	+	0.91666667	2,0,22,0	chr11,31452	Cytosol	A > G
chr11:31452788:A:G:UTR3:DNAJC	chr11	31452788	31452788	A	G	UTR3	DNAJC24	ENST000004	.	.	A	G	+	+	0.82608696	4,0,19,0	chr11,31452	Cytosol	A > G
chr11:31452835:A:G:UTR3:DNAJC	chr11	31452835	31452835	A	G	UTR3	DNAJC24	ENST000004	.	.	A	G	+	+	0.70833333	7,0,17,0	chr11,31452	Cytosol	A > G
chr11:31452841:A:G:UTR3:DNAJC	chr11	31452841	31452841	A	G	UTR3	DNAJC24	ENST000004	.	.	A	G	+	+	0.2173913	18,0,5,0	chr11,31452	Cytosol	A > G
chr11:31452960:A:G:UTR3:DNAJC	chr11	31452960	31452960	A	G	UTR3	DNAJC24	ENST000004	.	.	A	G	+	+	0.29411765	12,0,5,0	chr11,31452	Cytosol	A > G
chr11:31453157:A:G:UTR3:DNAJC	chr11	31453157	31453157	A	G	UTR3	DNAJC24	ENST000004	.	.	A	G	+	+	1	0,0,24,0	chr11,31453	Cytosol	A > G
chr11:31453158:A:G:UTR3:DNAJC	chr11	31453158	31453158	A	G	UTR3	DNAJC24	ENST000004	.	.	A	G	+	+	0.38095238	13,0,8,0	chr11,31453	Cytosol	A > G
chr11:31453172:A:G:UTR3:DNAJC	chr11	31453172	31453172	A	G	UTR3	DNAJC24	ENST000004	.	.	A	G	+	+	0.30769231	18,0,8,0	chr11,31453	Cytosol	A > G
chr11:31453173:A:G:UTR3:DNAJC	chr11	31453173	31453173	A	G	UTR3	DNAJC24	ENST000004	.	.	A	G	+	+	0.88	3,0,22,0	chr11,31453	Cytosol	A > G
chr11:31453219:A:G:UTR3:DNAJC	chr11	31453219	31453219	A	G	UTR3	DNAJC24	ENST000004	.	.	A	G	+	+	0.8	5,0,20,0	chr11,31453	Cytosol	A > G
chr11:31453268:A:G:UTR3:DNAJC	chr11	31453268	31453268	A	G	UTR3	DNAJC24	ENST000004	.	.	A	G	+	+	0.58333333	10,0,14,0	chr11,31453	Cytosol	A > G
chr11:31453309:A:G:UTR3:DNAJC	chr11	31453309	31453309	A	G	UTR3	DNAJC24	ENST000004	.	.	A	G	+	+	0.23333333	23,0,7,0	chr11,31453	Cytosol	A > G
chr11:32625462:A:G:UTR3:EIF3M	chr11	32625462	32625462	A	G	UTR3	EIF3M	ENST000005	.	.	A	G	+	+-	0.6	2,0,3,0	chr11,32625	Cytosol	A > G
chr11:32625466:A:G:UTR3:EIF3M	chr11	32625466	32625466	A	G	UTR3	EIF3M	ENST000005	.	.	A	G	+	+-	0.83333333	1,0,5,0	chr11,32625	Cytosol	A > G
chr11:32625528:A:G:UTR3:EIF3M	chr11	32625528	32625528	A	G	UTR3	EIF3M	ENST000005	.	.	A	G	+	+-	0.28571429	5,0,2,0	chr11,32625	Cytosol	A > G
chr11:32625687:A:G:UTR3:EIF3M	chr11	32625687	32625687	A	G	UTR3	EIF3M	ENST000005	.	.	A	G	+	+-	0.33333333	6,0,3,0	chr11,32625	Cytosol	A > G
chr11:32625809:A:G:UTR3:EIF3M	chr11	32625809	32625809	A	G	UTR3	EIF3M	ENST000005	.	.	A	G	+	+-	0.77777778	2,0,7,0	chr11,32625	Cytosol	A > G
chr11:32625925:A:G:UTR3:EIF3M	chr11	32625925	32625925	A	G	UTR3	EIF3M	ENST000005	.	.	A	G	+	+-	0.57142857	6,0,8,0	chr11,32625	Cytosol	A > G

chr11:32875318:A:G:UTR3:PRRG4	chr11	32875318	32875318	A	G	UTR3	PRRG4	ENST000002	.	.	A	G	+	+	0.2	8,0,2,0	chr11,32875	Cytosol	A > G
chr11:33096218:A:G:intronic:TCP1	chr11	33096218	33096218	A	G	intronic	TCP11L1	.	.	.	A	G	+	+	0.08695652	21,0,2,0	chr11,33096	Cytosol	A > G
chr11:33096274:A:G:intronic:TCP1	chr11	33096274	33096274	A	G	intronic	TCP11L1	.	.	.	A	G	+	+	0.07407407	25,0,2,0	chr11,33096	Cytosol	A > G
chr11:33096285:A:G:intronic:TCP1	chr11	33096285	33096285	A	G	intronic	TCP11L1	.	.	.	A	G	+	+	0.68	8,0,17,0	chr11,33096	Cytosol	A > G
chr11:33096294:A:G:intronic:TCP1	chr11	33096294	33096294	A	G	intronic	TCP11L1	.	.	.	A	G	+	+	0.52173913	11,0,12,0	chr11,33096	Cytosol	A > G
chr11:33096348:A:G:intronic:TCP1	chr11	33096348	33096348	A	G	intronic	TCP11L1	.	.	.	A	G	+	+	0.13333333	13,0,2,0	chr11,33096	Cytosol	A > G
chr11:33096395:A:G:intronic:TCP1	chr11	33096395	33096395	A	G	intronic	TCP11L1	.	.	.	A	G	+	+	0.41666667	7,0,5,0	chr11,33096	Cytosol	A > G
chr11:33096405:A:G:intronic:TCP1	chr11	33096405	33096405	A	G	intronic	TCP11L1	.	.	.	A	G	+	+	0.23076923	10,0,3,0	chr11,33096	Cytosol	A > G
chr11:33096857:A:G:intronic:TCP1	chr11	33096857	33096857	A	G	intronic	TCP11L1	.	.	.	A	G	+	+	0.57142857	3,0,4,0	chr11,33096	Cytosol	A > G
chr11:33097030:A:G:intronic:TCP1	chr11	33097030	33097030	A	G	intronic	TCP11L1	.	.	.	A	G	+	+	0.42857143	12,0,9,0	chr11,33097	Cytosol	A > G
chr11:33097032:A:G:intronic:TCP1	chr11	33097032	33097032	A	G	intronic	TCP11L1	.	.	.	A	G	+	+	0.23809524	16,0,5,0	chr11,33097	Cytosol	A > G
chr11:43878407:A:G:ncRNA_intro	chr11	43878407	43878407	A	G	ncRNA_intro	RP11-613D1	.	.	.	A	G	+	-	0.4	3,0,2,0	chr11,43878	Cytosol	A > G
chr11:43878609:A:G:ncRNA_intro	chr11	43878609	43878609	A	G	ncRNA_intro	RP11-613D1	.	.	.	A	G	+	-	0.6	2,0,3,0	chr11,43878	Cytosol	A > G
chr11:61566461:T:C:intronic:FADS	chr11	61566461	61566461	T	C	intronic	FADS2	.	.	.	A	G	-	+	0.14285714	12,0,2,0	chr11,61566	Cytosol	A > G
chr11:61566470:T:C:intronic:FADS	chr11	61566470	61566470	T	C	intronic	FADS2	.	.	.	A	G	-	+	0.5	6,0,6,0	chr11,61566	Cytosol	A > G
chr11:61566500:T:C:intronic:FADS	chr11	61566500	61566500	T	C	intronic	FADS2	.	.	.	A	G	-	+	0.33333333	6,0,3,0	chr11,61566	Cytosol	A > G
chr11:61566503:T:C:intronic:FADS	chr11	61566503	61566503	T	C	intronic	FADS2	.	.	.	A	G	-	+	0.22222222	7,0,2,0	chr11,61566	Cytosol	A > G
chr11:61566727:T:C:intronic:FADS	chr11	61566727	61566727	T	C	intronic	FADS2	.	.	.	A	G	-	+	0.75	5,0,15,0	chr11,61566	Cytosol	A > G
chr11:61567668:G:T:UTR3:FADS1	chr11	61567668	61567668	G	T	UTR3	FADS1	ENST000003	.	.	C	A	-	+-	0.03151862	11,338,0,0	chr11,61567	Cytosol	C > A
chr11:61567697:T:C:UTR3:FADS1	chr11	61567697	61567697	T	C	UTR3	FADS1	ENST000003	.	.	A	G	-	+-	0.02090592	562,0,12,0	chr11,61567	Cytosol	A > G
chr11:61582062:T:C:intronic:FADS	chr11	61582062	61582062	T	C	intronic	FADS1,FADS	.	.	.	A	G	-	+-	1	0,0,5,0	chr11,61582	Cytosol	A > G
chr11:62409131:T:C:intronic:GAN	chr11	62409131	62409131	T	C	intronic	GANAB	.	.	.	A	G	-	-	0.4	3,0,2,0	chr11,62409	Cytosol	A > G
chr11:62506299:A:G:intronic:TTC9	chr11	62506299	62506299	A	G	intronic	TTC9C	.	.	.	A	G	+	+	0.10526316	17,0,2,0	chr11,62506	Cytosol	A > G
chr11:62506416:A:G:intronic:TTC9	chr11	62506416	62506416	A	G	intronic	TTC9C	.	.	.	A	G	+	+	0.33333333	32,0,16,0	chr11,62506	Cytosol	A > G
chr11:62506638:A:G:intronic:TTC9	chr11	62506638	62506638	A	G	intronic	TTC9C	.	.	.	A	G	+	+	0.21428571	22,0,6,0	chr11,62506	Cytosol	A > G
chr11:62506677:A:G:intronic:TTC9	chr11	62506677	62506677	A	G	intronic	TTC9C	.	.	.	A	G	+	+	0.25	12,0,4,0	chr11,62506	Cytosol	A > G
chr11:62506990:A:G:intronic:TTC9	chr11	62506990	62506990	A	G	intronic	TTC9C	.	.	.	A	G	+	+	0.3877551	30,0,19,0	chr11,62506	Cytosol	A > G
chr11:62507126:A:G:intronic:TTC9	chr11	62507126	62507126	A	G	intronic	TTC9C	.	.	.	A	G	+	+	0.10714286	25,0,3,0	chr11,62507	Cytosol	A > G
chr11:62507234:A:G:intronic:TTC9	chr11	62507234	62507234	A	G	intronic	TTC9C	.	.	.	A	G	+	+	0.21875	25,0,7,0	chr11,62507	Cytosol	A > G
chr11:62507236:A:G:intronic:TTC9	chr11	62507236	62507236	A	G	intronic	TTC9C	.	.	.	A	G	+	+	0.1	27,0,3,0	chr11,62507	Cytosol	A > G
chr11:62507302:A:G:intronic:TTC9	chr11	62507302	62507302	A	G	intronic	TTC9C	.	.	.	A	G	+	+	0.05405405	35,0,2,0	chr11,62507	Cytosol	A > G
chr11:62507311:A:G:intronic:TTC9	chr11	62507311	62507311	A	G	intronic	TTC9C	.	.	.	A	G	+	+	0.19444444	29,0,7,0	chr11,62507	Cytosol	A > G
chr11:62591156:T:C:intronic:STX5	chr11	62591156	62591156	T	C	intronic	STX5	.	.	.	A	G	-	-	0.57142857	3,0,4,0	chr11,62591	Cytosol	A > G
chr11:62591191:T:C:intronic:STX5	chr11	62591191	62591191	T	C	intronic	STX5	.	.	.	A	G	-	-	0.375	5,0,3,0	chr11,62591	Cytosol	A > G
chr11:62591201:T:C:intronic:STX5	chr11	62591201	62591201	T	C	intronic	STX5	.	.	.	A	G	-	-	0.66666667	2,0,4,0	chr11,62591	Cytosol	A > G
chr11:65065202:A:G:UTR3:POLA2	chr11	65065202	65065202	A	G	UTR3	POLA2	ENST000002	.	.	A	G	+	+	0.4	3,0,2,0	chr11,65065	Cytosol	A > G
chr11:65065251:A:G:UTR3:POLA2	chr11	65065251	65065251	A	G	UTR3	POLA2	ENST000002	.	.	A	G	+	+	0.14285714	12,0,2,0	chr11,65065	Cytosol	A > G
chr11:65065261:A:G:UTR3:POLA2	chr11	65065261	65065261	A	G	UTR3	POLA2	ENST000002	.	.	A	G	+	+	0.30769231	9,0,4,0	chr11,65065	Cytosol	A > G
chr11:65073024:A:G:UTR3:POLA2	chr11	65073024	65073024	A	G	UTR3	POLA2	ENST000005	.	.	A	G	+	+	0.33333333	4,0,2,0	chr11,65073	Cytosol	A > G
chr11:65090876:A:G:downstream:	chr11	65090876	65090876	A	G	downstream	CDC42EP2	.	.	.	A	G	+		0.12	22,0,3,0	chr11,65090	Cytosol	A > G
chr11:65172973:A:G:intronic:FRM	chr11	65172973	65172973	A	G	intronic	FRMD8	.	.	.	A	G	+	+	0.28571429	5,0,2,0	chr11,65172	Cytosol	A > G
chr11:65428666:T:C:intronic:RELA	chr11	65428666	65428666	T	C	intronic	RELA	.	.	.	A	G	-	-	0.6	2,0,3,0	chr11,65428	Cytosol	A > G
chr11:65543458:T:C:UTR3:AP5B1	chr11	65543458	65543458	T	C	UTR3	AP5B1	ENST000005	.	.	A	G	-	-	0.22222222	14,0,4,0	chr11,65543	Cytosol	A > G
chr11:65543634:T:C:UTR3:AP5B1	chr11	65543634	65543634	T	C	UTR3	AP5B1	ENST000005	.	.	A	G	-	-	0.18181818	18,0,4,0	chr11,65543	Cytosol	A > G
chr11:65544342:T:C:UTR3:AP5B1	chr11	65544342	65544342	T	C	UTR3	AP5B1	ENST000005	.	.	A	G	-	-	0.06521739	43,0,3,0	chr11,65544	Cytosol	A > G
chr11:65544381:T:C:UTR3:AP5B1	chr11	65544381	65544381	T	C	UTR3	AP5B1	ENST000005	.	.	A	G	-	-	0.19444444	29,0,7,0	chr11,65544	Cytosol	A > G
chr11:65544382:T:C:UTR3:AP5B1	chr11	65544382	65544382	T	C	UTR3	AP5B1	ENST000005	.	.	A	G	-	-	0.10526316	34,0,4,0	chr11,65544	Cytosol	A > G
chr11:65544426:T:C:UTR3:AP5B1	chr11	65544426	65544426	T	C	UTR3	AP5B1	ENST000005	.	.	A	G	-	-	0.07317073	38,0,3,0	chr11,65544	Cytosol	A > G
chr11:65544503:T:C:UTR3:AP5B1	chr11	65544503	65544503	T	C	UTR3	AP5B1	ENST000005	.	.	A	G	-	-	0.13333333	26,0,4,0	chr11,65544	Cytosol	A > G
chr11:65544523:T:C:UTR3:AP5B1	chr11	65544523	65544523	T	C	UTR3	AP5B1	ENST000005	.	.	A	G	-	-	0.11627907	38,0,5,0	chr11,65544	Cytosol	A > G
chr11:65544567:T:C:UTR3:AP5B1	chr11	65544567	65544567	T	C	UTR3	AP5B1	ENST000005	.	.	A	G	-	-	0.40909091	26,0,18,0	chr11,65544	Cytosol	A > G
chr11:66403610:A:G:intronic:RBM	chr11	66403610	66403610	A	G	intronic	RBM14-RBM	.	.	.	A	G	+	+	0.28571429	5,0,2,0	chr11,66403	Cytosol	A > G
chr11:66403620:A:G:intronic:RBM	chr11	66403620	66403620	A	G	intronic	RBM14-RBM	.	.	.	A	G	+	+	0.71428571	2,0,5,0	chr11,66403	Cytosol	A > G
chr11:66450312:T:C:intergenic:RB	chr11	66450312	66450312	T	C	intergenic	RBM4B,SPTB	dist=4920;dis	.	.	A	G	-		0.4	3,0,2,0	chr11,66450	Cytosol	A > G
chr11:66572957:A:G:intronic:C11c	chr11	66572957	66572957	A	G	intronic	C11orf80	.	.	.	A	G	+	+	0.8	1,0,4,0	chr11,66572	Cytosol	A > G
chr11:66573002:A:G:intronic:C11c	chr11	66573002	66573002	A	G	intronic	C11orf80	.	.	.	A	G	+	+	1	0,0,6,0	chr11,66573	Cytosol	A > G
chr11:66608686:T:C:intronic:C11o	chr11	66608686	66608686	T	C	intronic	C11orf80	.	.	.	A	G	-	+	1	0,0,6,0	chr11,66608	Cytosol	A > G
chr11:66608860:T:C:intronic:C11o	chr11	66608860	66608860	T	C	intronic	C11orf80	.	.	.	A	G	-	+	0.8	1,0,4,0	chr11,66608	Cytosol	A > G
chr11:66609427:T:C:intronic:C11o	chr11	66609427	66609427	T	C	intronic	C11orf80	.	.	.	A	G	-	+	0.33333333	6,0,3,0	chr11,66609	Cytosol	A > G
chr11:66609473:T:C:intronic:C11o	chr11	66609473	66609473	T	C	intronic	C11orf80	.	.	.	A	G	-	+	0.58333333	5,0,7,0	chr11,66609	Cytosol	A > G
chr11:66609546:T:C:intronic:C11o	chr11	66609546	66609546	T	C	intronic	C11orf80	.	.	.	A	G	-	+	0.15384615	11,0,2,0	chr11,66609	Cytosol	A > G
chr11:66609591:T:C:intronic:C11o	chr11	66609591	66609591	T	C	intronic	C11orf80	.	.	.	A	G	-	+	0.82352941	3,0,14,0	chr11,66609	Cytosol	A > G
chr11:66609592:T:C:intronic:C11o	chr11	66609592	66609592	T	C	intronic	C11orf80	.	.	.	A	G	-	+	0.6875	5,0,11,0	chr11,66609	Cytosol	A > G
chr11:67791326:A:G:intronic:ALDH	chr11	67791326	67791326	A	G	intronic	ALDH3B1	.	.	.	A	G	+	+	1	0,0,5,0	chr11,67791	Cytosol	A > G

chr11:67791386:A:G:intronic:ALDH	chr11	67791386	67791386	A	G	intronic	ALDH3B1	.	.	.	A	G	+	+	0.22222222	7,0,2,0	chr11,67791	Cytosol	A > G
chr11:68522907:T:C:UTR3:CPT1A	chr11	68522907	68522907	T	C	UTR3	CPT1A	ENST000002	.	.	A	G	-	-	0.14423077	89,0,15,0	chr11,68522	Cytosol	A > G
chr11:68522950:T:C:UTR3:CPT1A	chr11	68522950	68522950	T	C	UTR3	CPT1A	ENST000002	.	.	A	G	-	-	0.15909091	37,0,7,0	chr11,68522	Cytosol	A > G
chr11:68523128:T:C:UTR3:CPT1A	chr11	68523128	68523128	T	C	UTR3	CPT1A	ENST000002	.	.	A	G	-	-	0.18666667	61,0,14,0	chr11,68523	Cytosol	A > G
chr11:68523337:T:C:UTR3:CPT1A	chr11	68523337	68523337	T	C	UTR3	CPT1A	ENST000002	.	.	A	G	-	-	0.35772358	79,0,44,0	chr11,68523	Cytosol	A > G
chr11:68523338:T:C:UTR3:CPT1A	chr11	68523338	68523338	T	C	UTR3	CPT1A	ENST000002	.	.	A	G	-	-	0.13178295	112,0,17,0	chr11,68523	Cytosol	A > G
chr11:68523347:T:C:UTR3:CPT1A	chr11	68523347	68523347	T	C	UTR3	CPT1A	ENST000002	.	.	A	G	-	-	0.21167883	108,0,29,0	chr11,68523	Cytosol	A > G
chr11:68523433:T:C:UTR3:CPT1A	chr11	68523433	68523433	T	C	UTR3	CPT1A	ENST000002	.	.	A	G	-	-	0.14285714	72,0,12,0	chr11,68523	Cytosol	A > G
chr11:68523434:T:C:UTR3:CPT1A	chr11	68523434	68523434	T	C	UTR3	CPT1A	ENST000002	.	.	A	G	-	-	0.32098765	55,0,26,0	chr11,68523	Cytosol	A > G
chr11:68523468:T:C:UTR3:CPT1A	chr11	68523468	68523468	T	C	UTR3	CPT1A	ENST000002	.	.	A	G	-	-	0.67532468	25,0,52,0	chr11,68523	Cytosol	A > G
chr11:68523471:T:C:UTR3:CPT1A	chr11	68523471	68523471	T	C	UTR3	CPT1A	ENST000002	.	.	A	G	-	-	0.10227273	79,0,9,0	chr11,68523	Cytosol	A > G
chr11:68523472:T:C:UTR3:CPT1A	chr11	68523472	68523472	T	C	UTR3	CPT1A	ENST000002	.	.	A	G	-	-	0.49425287	44,0,43,0	chr11,68523	Cytosol	A > G
chr11:68523497:T:C:UTR3:CPT1A	chr11	68523497	68523497	T	C	UTR3	CPT1A	ENST000002	.	.	A	G	-	-	0.25490196	38,0,13,0	chr11,68523	Cytosol	A > G
chr11:68523534:T:C:UTR3:CPT1A	chr11	68523534	68523534	T	C	UTR3	CPT1A	ENST000002	.	.	A	G	-	-	0.19230769	21,0,5,0	chr11,68523	Cytosol	A > G
chr11:68523567:T:C:UTR3:CPT1A	chr11	68523567	68523567	T	C	UTR3	CPT1A	ENST000002	.	.	A	G	-	-	0.8125	3,0,13,0	chr11,68523	Cytosol	A > G
chr11:68858609:A:G:intergenic:TP	chr11	68858609	68858609	A	G	intergenic	TPCN2,RP11-	dist=2617;dis	.	.	A	G	+		0.8	1,0,4,0	chr11,68858	Cytosol	A > G
chr11:68858619:A:G:intergenic:TP	chr11	68858619	68858619	A	G	intergenic	TPCN2,RP11-	dist=2627;dis	.	.	A	G	+		1	0,0,5,0	chr11,68858	Cytosol	A > G
chr11:72000820:T:C:intergenic:PH	chr11	72000820	72000820	T	C	intergenic	PHOX2A,CLP	dist=45600;d	.	.	A	G	-		0.05128205	37,0,2,0	chr11,72000	Cytosol	A > G
chr11:72002625:T:C:downstream:	chr11	72002625	72002625	T	C	downstream	CLPB	.	.	.	A	G	-		0.83333333	2,0,10,0	chr11,72002	Cytosol	A > G
chr11:72002843:T:C:downstream:	chr11	72002843	72002843	T	C	downstream	CLPB	.	.	.	A	G	-		0.71428571	2,0,5,0	chr11,72002	Cytosol	A > G
chr11:73009859:A:G:downstream:	chr11	73009859	73009859	A	G	downstream	P2RY6	.	.	.	A	G	+		0.38461538	8,0,5,0	chr11,73009	Cytosol	A > G
chr11:73009862:A:G:downstream:	chr11	73009862	73009862	A	G	downstream	P2RY6	.	.	.	A	G	+		0.46153846	7,0,6,0	chr11,73009	Cytosol	A > G
chr11:73009909:A:G:downstream:	chr11	73009909	73009909	A	G	downstream	P2RY6	.	.	.	A	G	+		0.15384615	22,0,4,0	chr11,73009	Cytosol	A > G
chr11:73585109:T:C:intronic:COA	chr11	73585109	73585109	T	C	intronic	COA4	.	.	.	A	G	-	-	0.8	1,0,4,0	chr11,73585	Cytosol	A > G
chr11:73841395:T:C:intronic:C2CD	chr11	73841395	73841395	T	C	intronic	C2CD3	.	.	.	A	G	-	-	0.4	3,0,2,0	chr11,73841	Cytosol	A > G
chr11:74677990:A:G:intronic:SPCS	chr11	74677990	74677990	A	G	intronic	SPCS2	.	.	.	A	G	+	+	0.8	1,0,4,0	chr11,74677	Cytosol	A > G
chr11:76094702:A:G:UTR3:RP11-1	chr11	76094702	76094702	A	G	UTR3	RP11-111M2	ENST000005	.	.	A	G	+	+	0.4	3,0,2,0	chr11,76094	Cytosol	A > G
chr11:76094755:A:G:UTR3:RP11-1	chr11	76094755	76094755	A	G	UTR3	RP11-111M2	ENST000005	.	.	A	G	+	+	0.6	2,0,3,0	chr11,76094	Cytosol	A > G
chr11:76095628:A:G:intronic:RP11	chr11	76095628	76095628	A	G	intronic	RP11-111M2	.	.	.	A	G	+	+	0.6	2,0,3,0	chr11,76095	Cytosol	A > G
chr11:76095629:A:G:intronic:RP11	chr11	76095629	76095629	A	G	intronic	RP11-111M2	.	.	.	A	G	+	+	0.66666667	2,0,4,0	chr11,76095	Cytosol	A > G
chr11:76736491:A:G:UTR3:ACER3	chr11	76736491	76736491	A	G	UTR3	ACER3	ENST000005	.	.	A	G	+	+	0.75	2,0,6,0	chr11,76736	Cytosol	A > G
chr11:76736521:A:G:UTR3:ACER3	chr11	76736521	76736521	A	G	UTR3	ACER3	ENST000005	.	.	A	G	+	+	0.85714286	1,0,6,0	chr11,76736	Cytosol	A > G
chr11:76736582:A:G:UTR3:ACER3	chr11	76736582	76736582	A	G	UTR3	ACER3	ENST000005	.	.	A	G	+	+	0.80952381	4,0,17,0	chr11,76736	Cytosol	A > G
chr11:76736712:A:G:UTR3:ACER3	chr11	76736712	76736712	A	G	UTR3	ACER3	ENST000005	.	.	A	G	+	+	0.44927536	38,0,31,0	chr11,76736	Cytosol	A > G
chr11:76736724:A:G:UTR3:ACER3	chr11	76736724	76736724	A	G	UTR3	ACER3	ENST000005	.	.	A	G	+	+	0.63829787	34,0,60,0	chr11,76736	Cytosol	A > G
chr11:77779628:T:C:UTR3:NDUFC	chr11	77779628	77779628	T	C	UTR3	NDUFC2	ENST000002	.	.	A	G	-	-	0.03846154	75,0,3,0	chr11,77779	Cytosol	A > G
chr11:77779638:T:C:UTR3:NDUFC	chr11	77779638	77779638	T	C	UTR3	NDUFC2	ENST000002	.	.	A	G	-	-	0.11538462	69,0,9,0	chr11,77779	Cytosol	A > G
chr11:77779693:T:C:UTR3:NDUFC	chr11	77779693	77779693	T	C	UTR3	NDUFC2	ENST000002	.	.	A	G	-	-	0.05555556	34,0,2,0	chr11,77779	Cytosol	A > G
chr11:77779707:T:C:UTR3:NDUFC	chr11	77779707	77779707	T	C	UTR3	NDUFC2	ENST000002	.	.	A	G	-	-	0.06521739	43,0,3,0	chr11,77779	Cytosol	A > G
chr11:77779709:T:C:UTR3:NDUFC	chr11	77779709	77779709	T	C	UTR3	NDUFC2	ENST000002	.	.	A	G	-	-	0.54347826	21,0,25,0	chr11,77779	Cytosol	A > G
chr11:77779719:T:C:UTR3:NDUFC	chr11	77779719	77779719	T	C	UTR3	NDUFC2	ENST000002	.	.	A	G	-	-	0.09677419	28,0,3,0	chr11,77779	Cytosol	A > G
chr11:77780072:T:C:UTR3:NDUFC	chr11	77780072	77780072	T	C	UTR3	NDUFC2	ENST000002	.	.	A	G	-	-	0.09649123	103,0,11,0	chr11,77780	Cytosol	A > G
chr11:77780111:T:C:UTR3:NDUFC	chr11	77780111	77780111	T	C	UTR3	NDUFC2	ENST000002	.	.	A	G	-	-	0.03703704	78,0,3,0	chr11,77780	Cytosol	A > G
chr11:77780209:T:C:UTR3:NDUFC	chr11	77780209	77780209	T	C	UTR3	NDUFC2	ENST000002	.	.	A	G	-	-	0.02222222	88,0,2,0	chr11,77780	Cytosol	A > G
chr11:77846289:T:C:UTR5:ALG8	chr11	77846289	77846289	T	C	UTR5	ALG8	ENST000005	.	.	A	G	-	-	0.28571429	5,0,2,0	chr11,77846	Cytosol	A > G
chr11:77846306:T:C:UTR5:ALG8	chr11	77846306	77846306	T	C	UTR5	ALG8	ENST000005	.	.	A	G	-	-	0.85714286	1,0,6,0	chr11,77846	Cytosol	A > G
chr11:77846314:T:C:UTR5:ALG8	chr11	77846314	77846314	T	C	UTR5	ALG8	ENST000005	.	.	A	G	-	-	0.33333333	4,0,2,0	chr11,77846	Cytosol	A > G
chr11:77846315:T:C:UTR5:ALG8	chr11	77846315	77846315	T	C	UTR5	ALG8	ENST000005	.	.	A	G	-	-	0.5	3,0,3,0	chr11,77846	Cytosol	A > G
chr11:77853006:A:G:ncRNA_exoni	chr11	77853006	77853006	A	G	ncRNA_exon	KCTD21-AS1	.	.	.	A	G	+	+	0.17647059	14,0,3,0	chr11,77853	Cytosol	A > G
chr11:77853042:A:G:ncRNA_exoni	chr11	77853042	77853042	A	G	ncRNA_exon	KCTD21-AS1	.	.	.	A	G	+	+	0.28571429	10,0,4,0	chr11,77853	Cytosol	A > G
chr11:77853045:A:G:ncRNA_exoni	chr11	77853045	77853045	A	G	ncRNA_exon	KCTD21-AS1	.	.	.	A	G	+	+	0.46153846	7,0,6,0	chr11,77853	Cytosol	A > G
chr11:77853058:A:G:ncRNA_exoni	chr11	77853058	77853058	A	G	ncRNA_exon	KCTD21-AS1	.	.	.	A	G	+	+	0.33333333	8,0,4,0	chr11,77853	Cytosol	A > G
chr11:77853798:A:G:ncRNA_exoni	chr11	77853798	77853798	A	G	ncRNA_exon	KCTD21-AS1	.	.	.	A	G	+	+	0.08695652	21,0,2,0	chr11,77853	Cytosol	A > G
chr11:77853845:A:G:ncRNA_exoni	chr11	77853845	77853845	A	G	ncRNA_exon	KCTD21-AS1	.	.	.	A	G	+	+	0.20833333	19,0,5,0	chr11,77853	Cytosol	A > G
chr11:77853909:A:G:ncRNA_exoni	chr11	77853909	77853909	A	G	ncRNA_exon	KCTD21-AS1	.	.	.	A	G	+	+	0.15384615	11,0,2,0	chr11,77853	Cytosol	A > G
chr11:77853915:A:G:ncRNA_exoni	chr11	77853915	77853915	A	G	ncRNA_exon	KCTD21-AS1	.	.	.	A	G	+	+	0.25	9,0,3,0	chr11,77853	Cytosol	A > G
chr11:82797626:A:G:ncRNA_intro	chr11	82797626	82797626	A	G	ncRNA_intro	RAB30-AS1,R	.	.	.	A	G	+	+-	0.8	1,0,4,0	chr11,82797	Cytosol	A > G
chr11:85996271:A:G:intergenic:EE	chr11	85996271	85996271	A	G	intergenic	EED,C11orf7	dist=6416;dis	.	.	A	G	+		0.22222222	7,0,2,0	chr11,85996	Cytosol	A > G
chr11:85996280:A:G:intergenic:EE	chr11	85996280	85996280	A	G	intergenic	EED,C11orf7	dist=6425;dis	.	.	A	G	+		0.625	3,0,5,0	chr11,85996	Cytosol	A > G
chr11:85996893:A:G:intergenic:EE	chr11	85996893	85996893	A	G	intergenic	EED,C11orf7	dist=7038;dis	.	.	A	G	+		0.4	3,0,2,0	chr11,85996	Cytosol	A > G
chr11:88055700:T:C:UTR3:CTSC	chr11	88055700	88055700	T	C	UTR3	CTSC	ENST000005	.	.	A	G	-	-	0.6	2,0,3,0	chr11,88055	Cytosol	A > G
chr11:93466874:T:C:ncRNA_exoni	chr11	93466874	93466874	T	C	ncRNA_exon	MIR1304	.	.	.	A	G	-	-	0.5	8,0,8,0	chr11,93466	Cytosol	A > G
chr11:93467980:T:C:intronic:TAF1	chr11	93467980	93467980	T	C	intronic	TAF1D	.	.	.	A	G	-	-	0.07317073	76,0,6,0	chr11,93467	Cytosol	A > G
chr11:93468037:T:C:intronic:TAF1	chr11	93468037	93468037	T	C	intronic	TAF1D	.	.	.	A	G	-	-	0.02439024	80,0,2,0	chr11,93468	Cytosol	A > G

chr11:93468094:T:C:intronic:TAF1	chr11	93468094	93468094	T	C	intronic	TAF1D	.	.	.	A	G	-	-	0.02564103	114,0,3,0	chr11,93468	Cytosol	A > G
chr11:93468108:T:C:intronic:TAF1	chr11	93468108	93468108	T	C	intronic	TAF1D	.	.	.	A	G	-	-	0.01626016	121,0,2,0	chr11,93468	Cytosol	A > G
chr11:93468149:T:C:intronic:TAF1	chr11	93468149	93468149	T	C	intronic	TAF1D	.	.	.	A	G	-	-	0.02666667	73,0,2,0	chr11,93468	Cytosol	A > G
chr11:93468161:T:C:intronic:TAF1	chr11	93468161	93468161	T	C	intronic	TAF1D	.	.	.	A	G	-	-	0.05	76,0,4,0	chr11,93468	Cytosol	A > G
chr11:93468706:T:C:intronic:TAF1	chr11	93468706	93468706	T	C	intronic	TAF1D	.	.	.	A	G	-	-	0.14285714	12,0,2,0	chr11,93468	Cytosol	A > G
chr11:93468771:T:C:UTR3:TAF1D	chr11	93468771	93468771	T	C	UTR3	TAF1D	ENST000004	.	.	A	G	-	-	0.30769231	9,0,4,0	chr11,93468	Cytosol	A > G
chr11:93468796:T:C:UTR3:TAF1D	chr11	93468796	93468796	T	C	UTR3	TAF1D	ENST000004	.	.	A	G	-	-	0.8	3,0,12,0	chr11,93468	Cytosol	A > G
chr11:93494978:A:G:UTR3:C11orf	chr11	93494978	93494978	A	G	UTR3	C11orf54	ENST000005	.	.	A	G	+	-+	0.34782609	15,0,8,0	chr11,93494	Cytosol	A > G
chr11:93494980:A:G:UTR3:C11orf	chr11	93494980	93494980	A	G	UTR3	C11orf54	ENST000005	.	.	A	G	+	-+	0.16666667	20,0,4,0	chr11,93494	Cytosol	A > G
chr11:93494981:A:G:UTR3:C11orf	chr11	93494981	93494981	A	G	UTR3	C11orf54	ENST000005	.	.	A	G	+	-+	0.125	21,0,3,0	chr11,93494	Cytosol	A > G
chr11:93497866:A:G:UTR3:C11orf	chr11	93497866	93497866	A	G	UTR3	C11orf54	ENST000005	.	.	A	G	+	-+	0.4	3,0,2,0	chr11,93497	Cytosol	A > G
chr11:94149915:T:C:ncRNA_exoni	chr11	94149915	94149915	T	C	ncRNA_exon	AP000765.1	.	.	.	A	G	-	+	1	0,0,5,0	chr11,94149	Cytosol	A > G
chr11:94149973:T:C:downstream:	chr11	94149973	94149973	T	C	downstream	AP000765.1	.	.	.	A	G	-		0.85714286	1,0,6,0	chr11,94149	Cytosol	A > G
chr11:108236469:A:G:UTR3:ATM	chr11	108236469	108236469	A	G	UTR3	ATM	ENST000002	.	.	A	G	+	+-	0.42105263	11,0,8,0	chr11,10823	Cytosol	A > G
chr11:108236477:A:G:UTR3:ATM	chr11	108236477	108236477	A	G	UTR3	ATM	ENST000002	.	.	A	G	+	+-	0.33333333	12,0,6,0	chr11,10823	Cytosol	A > G
chr11:108236485:A:G:UTR3:ATM	chr11	108236485	108236485	A	G	UTR3	ATM	ENST000002	.	.	A	G	+	+-	0.44444444	10,0,8,0	chr11,10823	Cytosol	A > G
chr11:108236486:A:G:UTR3:ATM	chr11	108236486	108236486	A	G	UTR3	ATM	ENST000002	.	.	A	G	+	+-	0.29411765	12,0,5,0	chr11,10823	Cytosol	A > G
chr11:108236513:A:G:UTR3:ATM	chr11	108236513	108236513	A	G	UTR3	ATM	ENST000002	.	.	A	G	+	+-	0.15384615	11,0,2,0	chr11,10823	Cytosol	A > G
chr11:108236521:A:G:UTR3:ATM	chr11	108236521	108236521	A	G	UTR3	ATM	ENST000002	.	.	A	G	+	+-	0.27272727	8,0,3,0	chr11,10823	Cytosol	A > G
chr11:108236523:A:G:UTR3:ATM	chr11	108236523	108236523	A	G	UTR3	ATM	ENST000002	.	.	A	G	+	+-	0.625	3,0,5,0	chr11,10823	Cytosol	A > G
chr11:108236524:A:G:UTR3:ATM	chr11	108236524	108236524	A	G	UTR3	ATM	ENST000002	.	.	A	G	+	+-	0.3	7,0,3,0	chr11,10823	Cytosol	A > G
chr11:108236594:A:G:UTR3:ATM	chr11	108236594	108236594	A	G	UTR3	ATM	ENST000002	.	.	A	G	+	+-	0.25	9,0,3,0	chr11,10823	Cytosol	A > G
chr11:108237738:A:G:UTR3:ATM	chr11	108237738	108237738	A	G	UTR3	ATM	ENST000002	.	.	A	G	+	+-	0.22222222	14,0,4,0	chr11,10823	Cytosol	A > G
chr11:108237815:A:G:UTR3:ATM	chr11	108237815	108237815	A	G	UTR3	ATM	ENST000002	.	.	A	G	+	+-	0.28571429	15,0,6,0	chr11,10823	Cytosol	A > G
chr11:108237832:A:G:UTR3:ATM	chr11	108237832	108237832	A	G	UTR3	ATM	ENST000002	.	.	A	G	+	+-	0.28571429	15,0,6,0	chr11,10823	Cytosol	A > G
chr11:108237921:A:G:UTR3:ATM	chr11	108237921	108237921	A	G	UTR3	ATM	ENST000002	.	.	A	G	+	+-	0.25	6,0,2,0	chr11,10823	Cytosol	A > G
chr11:108237953:A:G:UTR3:ATM	chr11	108237953	108237953	A	G	UTR3	ATM	ENST000002	.	.	A	G	+	+-	0.4	6,0,4,0	chr11,10823	Cytosol	A > G
chr11:108237957:A:G:UTR3:ATM	chr11	108237957	108237957	A	G	UTR3	ATM	ENST000002	.	.	A	G	+	+-	0.77777778	2,0,7,0	chr11,10823	Cytosol	A > G
chr11:108237975:A:G:UTR3:ATM	chr11	108237975	108237975	A	G	UTR3	ATM	ENST000002	.	.	A	G	+	+-	0.44444444	5,0,4,0	chr11,10823	Cytosol	A > G
chr11:108237976:A:G:UTR3:ATM	chr11	108237976	108237976	A	G	UTR3	ATM	ENST000002	.	.	A	G	+	+-	0.7	3,0,7,0	chr11,10823	Cytosol	A > G
chr11:108237988:A:G:UTR3:ATM	chr11	108237988	108237988	A	G	UTR3	ATM	ENST000002	.	.	A	G	+	+-	0.16666667	10,0,2,0	chr11,10823	Cytosol	A > G
chr11:111586703:T:C:intronic:SIK2	chr11	111586703	111586703	T	C	intronic	SIK2	.	.	.	A	G	-	+	0.8	1,0,4,0	chr11,11158	Cytosol	A > G
chr11:111599113:A:G:UTR3:SIK2	chr11	111599113	111599113	A	G	UTR3	SIK2	ENST000003	.	.	A	G	+	+-	0.55555556	4,0,5,0	chr11,11159	Cytosol	A > G
chr11:111599185:A:G:UTR3:SIK2	chr11	111599185	111599185	A	G	UTR3	SIK2	ENST000003	.	.	A	G	+	+-	0.2	20,0,5,0	chr11,11159	Cytosol	A > G
chr11:111599256:A:G:UTR3:SIK2	chr11	111599256	111599256	A	G	UTR3	SIK2	ENST000003	.	.	A	G	+	+-	0.75862069	7,0,22,0	chr11,11159	Cytosol	A > G
chr11:111599397:A:G:UTR3:SIK2	chr11	111599397	111599397	A	G	UTR3	SIK2	ENST000003	.	.	A	G	+	+-	0.52777778	17,0,19,0	chr11,11159	Cytosol	A > G
chr11:111599398:A:G:UTR3:SIK2	chr11	111599398	111599398	A	G	UTR3	SIK2	ENST000003	.	.	A	G	+	+-	0.46875	17,0,15,0	chr11,11159	Cytosol	A > G
chr11:111599537:A:G:UTR3:SIK2	chr11	111599537	111599537	A	G	UTR3	SIK2	ENST000003	.	.	A	G	+	+-	0.7	3,0,7,0	chr11,11159	Cytosol	A > G
chr11:113239576:A:G:ncRNA_exo	chr11	113239576	113239576	A	G	ncRNA_exon	RP11-159N1	.	.	.	A	G	+	+	0.45454545	6,0,5,0	chr11,11323	Cytosol	A > G
chr11:118880600:A:G:intronic:CCI	chr11	118880600	118880600	A	G	intronic	CCDC84	.	.	.	A	G	+	+	0.66666667	2,0,4,0	chr11,11888	Cytosol	A > G
chr11:119532688:T:C:UTR3:PVRL1	chr11	119532688	119532688	T	C	UTR3	PVRL1	ENST000002	.	.	A	G	-	-	0.06976744	40,0,3,0	chr11,11953	Cytosol	A > G
chr11:122943085:T:C:UTR3:CLMP	chr11	122943085	122943085	T	C	UTR3	CLMP	ENST000004	.	.	A	G	-	-	0.3271028	72,0,35,0	chr11,12294	Cytosol	A > G
chr11:125526125:A:G:UTR3:CHEK	chr11	125526125	125526125	A	G	UTR3	CHEK1	ENST000005	.	.	A	G	+	+	0.05063291	75,0,4,0	chr11,12552	Cytosol	A > G
chr11:125526139:A:G:UTR3:CHEK	chr11	125526139	125526139	A	G	UTR3	CHEK1	ENST000005	.	.	A	G	+	+	0.44791667	53,0,43,0	chr11,12552	Cytosol	A > G
chr11:125545856:A:G:UTR3:CHEK	chr11	125545856	125545856	A	G	UTR3	CHEK1	ENST000004	.	.	A	G	+	+-	0.34482759	19,0,10,0	chr11,12554	Cytosol	A > G
chr11:125545903:A:G:UTR3:CHEK	chr11	125545903	125545903	A	G	UTR3	CHEK1	ENST000004	.	.	A	G	+	+-	0.13333333	26,0,4,0	chr11,12554	Cytosol	A > G
chr11:126077553:T:C:intronic:RPU	chr11	126077553	126077553	T	C	intronic	RPUSD4	.	.	.	A	G	-	-	0.33333333	4,0,2,0	chr11,12607	Cytosol	A > G
chr11:126164873:A:G:intronic:RP1	chr11	126164873	126164873	A	G	intronic	RP11-712L6.	.	.	.	A	G	+	+-	0.23076923	10,0,3,0	chr11,12616	Cytosol	A > G
chr11:126164949:A:G:intronic:RP1	chr11	126164949	126164949	A	G	intronic	RP11-712L6.	.	.	.	A	G	+	+-	0.11538462	23,0,3,0	chr11,12616	Cytosol	A > G
chr11:126165030:A:G:intronic:RP1	chr11	126165030	126165030	A	G	intronic	RP11-712L6.	.	.	.	A	G	+	+-	0.15	17,0,3,0	chr11,12616	Cytosol	A > G
chr11:126165100:A:G:intronic:RP1	chr11	126165100	126165100	A	G	intronic	RP11-712L6.	.	.	.	A	G	+	+-	0.21428571	11,0,3,0	chr11,12616	Cytosol	A > G
chr11:126165137:A:G:intronic:RP1	chr11	126165137	126165137	A	G	intronic	RP11-712L6.	.	.	.	A	G	+	+-	0.57142857	3,0,4,0	chr11,12616	Cytosol	A > G
chr11:126165241:A:G:intronic:RP1	chr11	126165241	126165241	A	G	intronic	RP11-712L6.	.	.	.	A	G	+	+-	0.57142857	3,0,4,0	chr11,12616	Cytosol	A > G
chr11:126165855:A:G:intronic:RP1	chr11	126165855	126165855	A	G	intronic	RP11-712L6.	.	.	.	A	G	+	+-	0.3	14,0,6,0	chr11,12616	Cytosol	A > G
chr11:126166031:A:G:intronic:RP1	chr11	126166031	126166031	A	G	intronic	RP11-712L6.	.	.	.	A	G	+	+-	0.18181818	9,0,2,0	chr11,12616	Cytosol	A > G
chr11:126166583:A:G:intronic:RP1	chr11	126166583	126166583	A	G	intronic	RP11-712L6.	.	.	.	A	G	+	+-	0.42105263	11,0,8,0	chr11,12616	Cytosol	A > G
chr11:126166588:A:G:intronic:RP1	chr11	126166588	126166588	A	G	intronic	RP11-712L6.	.	.	.	A	G	+	+-	0.5625	7,0,9,0	chr11,12616	Cytosol	A > G
chr11:126166614:A:G:intronic:RP1	chr11	126166614	126166614	A	G	intronic	RP11-712L6.	.	.	.	A	G	+	+-	0.375	10,0,6,0	chr11,12616	Cytosol	A > G
chr11:126166638:A:G:intronic:RP1	chr11	126166638	126166638	A	G	intronic	RP11-712L6.	.	.	.	A	G	+	+-	0.35294118	11,0,6,0	chr11,12616	Cytosol	A > G
chr11:126166651:A:G:intronic:RP1	chr11	126166651	126166651	A	G	intronic	RP11-712L6.	.	.	.	A	G	+	+-	0.15789474	16,0,3,0	chr11,12616	Cytosol	A > G
chr11:126166821:A:G:intronic:RP1	chr11	126166821	126166821	A	G	intronic	RP11-712L6.	.	.	.	A	G	+	+-	0.625	3,0,5,0	chr11,12616	Cytosol	A > G
chr11:126167768:A:G:intronic:RP1	chr11	126167768	126167768	A	G	intronic	RP11-712L6.	.	.	.	A	G	+	+-	0.41666667	7,0,5,0	chr11,12616	Cytosol	A > G
chr11:126167778:A:G:intronic:RP1	chr11	126167778	126167778	A	G	intronic	RP11-712L6.	.	.	.	A	G	+	+-	0.44444444	5,0,4,0	chr11,12616	Cytosol	A > G
chr11:126167841:A:G:intronic:RP1	chr11	126167841	126167841	A	G	intronic	RP11-712L6.	.	.	.	A	G	+	+-	0.4	3,0,2,0	chr11,12616	Cytosol	A > G

chr11:126216485:A:G:downstrean	chr11	126216485	126216485	A	G	downstream	DCPS	.	.	.	A	G	+	-	0.57575758	14,0,19,0	chr11,12621	Cytosol	A > G
chr11:126216489:A:G:downstrean	chr11	126216489	126216489	A	G	downstream	DCPS	.	.	.	A	G	+	-	0.3	21,0,9,0	chr11,12621	Cytosol	A > G
chr11:126216595:A:G:downstrean	chr11	126216595	126216595	A	G	downstream	DCPS	.	.	.	A	G	+	-	0.71428571	8,0,20,0	chr11,12621	Cytosol	A > G
chr11:126216638:A:G:downstrean	chr11	126216638	126216638	A	G	downstream	DCPS	.	.	.	A	G	+	-	0.4137931	17,0,12,0	chr11,12621	Cytosol	A > G
chr11:126216838:A:G:intergenic:D	chr11	126216838	126216838	A	G	intergenic	DCPS,ST3GAI	dist=1194;dis	.	.	A	G	+	-	0.37142857	22,0,13,0	chr11,12621	Cytosol	A > G
chr11:126216862:A:G:intergenic:D	chr11	126216862	126216862	A	G	intergenic	DCPS,ST3GAI	dist=1218;dis	.	.	A	G	+	-	0.80769231	5,0,21,0	chr11,12621	Cytosol	A > G
chr11:126216865:A:G:intergenic:D	chr11	126216865	126216865	A	G	intergenic	DCPS,ST3GAI	dist=1221;dis	.	.	A	G	+	-	0.76923077	6,0,20,0	chr11,12621	Cytosol	A > G
chr11:126219028:A:G:intergenic:D	chr11	126219028	126219028	A	G	intergenic	DCPS,ST3GAI	dist=3384;dis	.	.	A	G	+	-	0.44444444	35,0,28,0	chr11,12621	Cytosol	A > G
chr11:126219036:A:G:intergenic:D	chr11	126219036	126219036	A	G	intergenic	DCPS,ST3GAI	dist=3392;dis	.	.	A	G	+	-	0.54545455	25,0,30,0	chr11,12621	Cytosol	A > G
chr11:126219092:A:G:intergenic:D	chr11	126219092	126219092	A	G	intergenic	DCPS,ST3GAI	dist=3448;dis	.	.	A	G	+	-	0.42307692	15,0,11,0	chr11,12621	Cytosol	A > G
chr11:126219093:A:G:intergenic:D	chr11	126219093	126219093	A	G	intergenic	DCPS,ST3GAI	dist=3449;dis	.	.	A	G	+	-	0.42307692	15,0,11,0	chr11,12621	Cytosol	A > G
chr11:126219094:A:G:intergenic:D	chr11	126219094	126219094	A	G	intergenic	DCPS,ST3GAI	dist=3450;dis	.	.	A	G	+	-	0.52	12,0,13,0	chr11,12621	Cytosol	A > G
chr11:126219126:A:G:intergenic:D	chr11	126219126	126219126	A	G	intergenic	DCPS,ST3GAI	dist=3482;dis	.	.	A	G	+	-	0.30434783	16,0,7,0	chr11,12621	Cytosol	A > G
chr12:2997823:A:G:UTR3:RHNO1	chr12	2997823	2997823	A	G	UTR3	RHNO1	ENST000004	.	.	A	G	+	+	0.03125	124,0,4,0	chr12,29978	Cytosol	A > G
chr12:2997833:A:G:UTR3:RHNO1	chr12	2997833	2997833	A	G	UTR3	RHNO1	ENST000004	.	.	A	G	+	+	0.0137931	143,0,2,0	chr12,29978	Cytosol	A > G
chr12:2997839:A:G:UTR3:RHNO1	chr12	2997839	2997839	A	G	UTR3	RHNO1	ENST000004	.	.	A	G	+	+	0.01282051	154,0,2,0	chr12,29978	Cytosol	A > G
chr12:2997879:A:G:UTR3:RHNO1	chr12	2997879	2997879	A	G	UTR3	RHNO1	ENST000004	.	.	A	G	+	+	0.01834862	107,0,2,0	chr12,29978	Cytosol	A > G
chr12:7247809:T:C:UTR3:C1RL	chr12	7247809	7247809	T	C	UTR3	C1RL	ENST000002	.	.	A	G	-	-	0.0952381	19,0,2,0	chr12,72478	Cytosol	A > G
chr12:7247915:T:C:UTR3:C1RL	chr12	7247915	7247915	T	C	UTR3	C1RL	ENST000002	.	.	A	G	-	-	0.07692308	24,0,2,0	chr12,72479	Cytosol	A > G
chr12:7247969:T:C:UTR3:C1RL	chr12	7247969	7247969	T	C	UTR3	C1RL	ENST000002	.	.	A	G	-	-	0.10714286	25,0,3,0	chr12,72479	Cytosol	A > G
chr12:7250082:T:C:intronic:C1RL	chr12	7250082	7250082	T	C	intronic	C1RL	.	.	.	A	G	-	-	0.4	3,0,2,0	chr12,72500	Cytosol	A > G
chr12:7250117:T:C:intronic:C1RL	chr12	7250117	7250117	T	C	intronic	C1RL	.	.	.	A	G	-	-	0.33333333	4,0,2,0	chr12,72501	Cytosol	A > G
chr12:7250173:T:C:intronic:C1RL	chr12	7250173	7250173	T	C	intronic	C1RL	.	.	.	A	G	-	-	0.33333333	4,0,2,0	chr12,72501	Cytosol	A > G
chr12:8116652:T:C:intergenic:SLC	chr12	8116652	8116652	T	C	intergenic	SLC2A3,Y_RN	dist=27781;d	.	.	A	G	-		0.2	8,0,2,0	chr12,81166	Cytosol	A > G
chr12:8116674:T:C:intergenic:SLC	chr12	8116674	8116674	T	C	intergenic	SLC2A3,Y_RN	dist=27803;d	.	.	A	G	-		0.1875	13,0,3,0	chr12,81166	Cytosol	A > G
chr12:8116743:T:C:intergenic:SLC	chr12	8116743	8116743	T	C	intergenic	SLC2A3,Y_RN	dist=27872;d	.	.	A	G	-		0.33333333	4,0,2,0	chr12,81167	Cytosol	A > G
chr12:8123048:T:C:intergenic:SLC	chr12	8123048	8123048	T	C	intergenic	SLC2A3,Y_RN	dist=34177;d	.	.	A	G	-		0.4	6,0,4,0	chr12,81230	Cytosol	A > G
chr12:8123062:T:C:intergenic:SLC	chr12	8123062	8123062	T	C	intergenic	SLC2A3,Y_RN	dist=34191;d	.	.	A	G	-		0.16666667	10,0,2,0	chr12,81230	Cytosol	A > G
chr12:8123063:T:C:intergenic:SLC	chr12	8123063	8123063	T	C	intergenic	SLC2A3,Y_RN	dist=34192;d	.	.	A	G	-		0.16666667	10,0,2,0	chr12,81230	Cytosol	A > G
chr12:8123064:T:C:intergenic:SLC	chr12	8123064	8123064	T	C	intergenic	SLC2A3,Y_RN	dist=34193;d	.	.	A	G	-		0.18181818	9,0,2,0	chr12,81230	Cytosol	A > G
chr12:9812174:A:G:ncRNA_introni	chr12	9812174	9812174	A	G	ncRNA_intro	RP11-705C1	.	.	.	A	G	+	+	0.83333333	1,0,5,0	chr12,98121	Cytosol	A > G
chr12:9812177:A:G:ncRNA_introni	chr12	9812177	9812177	A	G	ncRNA_intro	RP11-705C1	.	.	.	A	G	+	+	0.5	3,0,3,0	chr12,98121	Cytosol	A > G
chr12:12605350:A:G:intronic:LOH	chr12	12605350	12605350	A	G	intronic	LOH12CR1	.	.	.	T	C	-	+-	1	0,5,0,0	chr12,12605	Cytosol	T > C
chr12:27183110:A:G:exonic:MED2	chr12	27183110	27183110	A	G	exonic	MED21	.	synonymous	MED21:ENST	A	G	+	+	0.0952381	19,0,2,0	chr12,27183	Cytosol	A > G
chr12:27183115:A:G:exonic:MED2	chr12	27183115	27183115	A	G	exonic	MED21	.	nonsynonym	MED21:ENST	A	G	+	+	0.11111111	16,0,2,0	chr12,27183	Cytosol	A > G
chr12:27183116:A:G:exonic:MED2	chr12	27183116	27183116	A	G	exonic	MED21	.	synonymous	MED21:ENST	A	G	+	+	0.1	18,0,2,0	chr12,27183	Cytosol	A > G
chr12:27187492:A:G:intergenic:M	chr12	27187492	27187492	A	G	intergenic	MED21,C12o	dist=3803;dis	.	.	A	G	+	+	0.26666667	11,0,4,0	chr12,27187	Cytosol	A > G
chr12:27187635:A:G:intergenic:M	chr12	27187635	27187635	A	G	intergenic	MED21,C12o	dist=3946;dis	.	.	A	G	+	+	0.72727273	3,0,8,0	chr12,27187	Cytosol	A > G
chr12:27187636:A:G:intergenic:M	chr12	27187636	27187636	A	G	intergenic	MED21,C12o	dist=3947;dis	.	.	A	G	+	+	0.66666667	3,0,6,0	chr12,27187	Cytosol	A > G
chr12:27188014:A:G:intergenic:M	chr12	27188014	27188014	A	G	intergenic	MED21,C12o	dist=4325;dis	.	.	A	G	+	+	0.57894737	8,0,11,0	chr12,27188	Cytosol	A > G
chr12:27574201:A:G:ncRNA_intro	chr12	27574201	27574201	A	G	ncRNA_intro	RP11-165P7.	.	.	.	A	G	+	+-	0.27027027	27,0,10,0	chr12,27574	Cytosol	A > G
chr12:27574223:A:G:ncRNA_intro	chr12	27574223	27574223	A	G	ncRNA_intro	RP11-165P7.	.	.	.	A	G	+	+-	0.35135135	24,0,13,0	chr12,27574	Cytosol	A > G
chr12:27574254:A:G:ncRNA_intro	chr12	27574254	27574254	A	G	ncRNA_intro	RP11-165P7.	.	.	.	A	G	+	+-	0.63636364	8,0,14,0	chr12,27574	Cytosol	A > G
chr12:27574269:A:G:ncRNA_intro	chr12	27574269	27574269	A	G	ncRNA_intro	RP11-165P7.	.	.	.	A	G	+	+-	0.16666667	30,0,6,0	chr12,27574	Cytosol	A > G
chr12:27574277:A:G:ncRNA_intro	chr12	27574277	27574277	A	G	ncRNA_intro	RP11-165P7.	.	.	.	A	G	+	+-	0.4047619	25,0,17,0	chr12,27574	Cytosol	A > G
chr12:27574278:A:G:ncRNA_intro	chr12	27574278	27574278	A	G	ncRNA_intro	RP11-165P7.	.	.	.	A	G	+	+-	0.17073171	34,0,7,0	chr12,27574	Cytosol	A > G
chr12:27574292:A:G:ncRNA_intro	chr12	27574292	27574292	A	G	ncRNA_intro	RP11-165P7.	.	.	.	A	G	+	+-	0.11764706	45,0,6,0	chr12,27574	Cytosol	A > G
chr12:27574302:A:G:ncRNA_intro	chr12	27574302	27574302	A	G	ncRNA_intro	RP11-165P7.	.	.	.	A	G	+	+-	0.0625	45,0,3,0	chr12,27574	Cytosol	A > G
chr12:27574303:A:G:ncRNA_intro	chr12	27574303	27574303	A	G	ncRNA_intro	RP11-165P7.	.	.	.	A	G	+	+-	0.16666667	45,0,9,0	chr12,27574	Cytosol	A > G
chr12:27574326:A:G:ncRNA_intro	chr12	27574326	27574326	A	G	ncRNA_intro	RP11-165P7.	.	.	.	A	G	+	+-	0.03614458	80,0,3,0	chr12,27574	Cytosol	A > G
chr12:27574425:A:G:ncRNA_intro	chr12	27574425	27574425	A	G	ncRNA_intro	RP11-165P7.	.	.	.	A	G	+	+-	0.03636364	53,0,2,0	chr12,27574	Cytosol	A > G
chr12:27574427:A:G:ncRNA_intro	chr12	27574427	27574427	A	G	ncRNA_intro	RP11-165P7.	.	.	.	A	G	+	+-	0.2962963	38,0,16,0	chr12,27574	Cytosol	A > G
chr12:27574434:A:G:ncRNA_intro	chr12	27574434	27574434	A	G	ncRNA_intro	RP11-165P7.	.	.	.	A	G	+	+-	0.19230769	42,0,10,0	chr12,27574	Cytosol	A > G
chr12:27574440:A:G:ncRNA_intro	chr12	27574440	27574440	A	G	ncRNA_intro	RP11-165P7.	.	.	.	A	G	+	+-	0.06	47,0,3,0	chr12,27574	Cytosol	A > G
chr12:27574499:A:G:ncRNA_intro	chr12	27574499	27574499	A	G	ncRNA_intro	RP11-165P7.	.	.	.	A	G	+	+-	0.57142857	3,0,4,0	chr12,27574	Cytosol	A > G
chr12:27574511:A:G:ncRNA_intro	chr12	27574511	27574511	A	G	ncRNA_intro	RP11-165P7.	.	.	.	A	G	+	+-	0.3	7,0,3,0	chr12,27574	Cytosol	A > G
chr12:27574536:A:G:ncRNA_intro	chr12	27574536	27574536	A	G	ncRNA_intro	RP11-165P7.	.	.	.	A	G	+	+-	0.44444444	5,0,4,0	chr12,27574	Cytosol	A > G
chr12:27576299:A:G:ncRNA_intro	chr12	27576299	27576299	A	G	ncRNA_intro	RP11-165P7.	.	.	.	A	G	+	+-	1	0,0,5,0	chr12,27576	Cytosol	A > G
chr12:27576337:A:G:ncRNA_intro	chr12	27576337	27576337	A	G	ncRNA_intro	RP11-165P7.	.	.	.	A	G	+	+-	0.57894737	8,0,11,0	chr12,27576	Cytosol	A > G
chr12:27576377:A:G:ncRNA_intro	chr12	27576377	27576377	A	G	ncRNA_intro	RP11-165P7.	.	.	.	A	G	+	+-	0.39285714	17,0,11,0	chr12,27576	Cytosol	A > G
chr12:27576399:A:G:ncRNA_intro	chr12	27576399	27576399	A	G	ncRNA_intro	RP11-165P7.	.	.	.	A	G	+	+-	0.06521739	43,0,3,0	chr12,27576	Cytosol	A > G
chr12:27576438:A:G:ncRNA_intro	chr12	27576438	27576438	A	G	ncRNA_intro	RP11-165P7.	.	.	.	A	G	+	+-	0.06666667	42,0,3,0	chr12,27576	Cytosol	A > G
chr12:27576444:A:G:ncRNA_intro	chr12	27576444	27576444	A	G	ncRNA_intro	RP11-165P7.	.	.	.	A	G	+	+-	0.15909091	37,0,7,0	chr12,27576	Cytosol	A > G
chr12:27576447:A:G:ncRNA_intro	chr12	27576447	27576447	A	G	ncRNA_intro	RP11-165P7.	.	.	.	A	G	+	+-	0.37777778	28,0,17,0	chr12,27576	Cytosol	A > G

chr12:27576461:A:G:ncRNA_intro	chr12	27576461	27576461	A	G	ncRNA_intro	RP11-165P7.	.	.	.	A	G	+	+-	0.08333333	33,0,3,0	chr12,27576	Cytosol	A > G
chr12:27576469:A:G:ncRNA_intro	chr12	27576469	27576469	A	G	ncRNA_intro	RP11-165P7.	.	.	.	A	G	+	+-	0.64102564	14,0,25,0	chr12,27576	Cytosol	A > G
chr12:27576523:A:G:ncRNA_intro	chr12	27576523	27576523	A	G	ncRNA_intro	RP11-165P7.	.	.	.	A	G	+	+-	0.39130435	28,0,18,0	chr12,27576	Cytosol	A > G
chr12:27576524:A:G:ncRNA_intro	chr12	27576524	27576524	A	G	ncRNA_intro	RP11-165P7.	.	.	.	A	G	+	+-	0.42857143	24,0,18,0	chr12,27576	Cytosol	A > G
chr12:27576536:A:G:ncRNA_intro	chr12	27576536	27576536	A	G	ncRNA_intro	RP11-165P7.	.	.	.	A	G	+	+-	0.25	44,1,15,0	chr12,27576	Cytosol	A > G
chr12:27576542:A:G:ncRNA_intro	chr12	27576542	27576542	A	G	ncRNA_intro	RP11-165P7.	.	.	.	A	G	+	+-	0.20967742	49,0,13,0	chr12,27576	Cytosol	A > G
chr12:27847199:A:G:UTR3:PPFIBP	chr12	27847199	27847199	A	G	UTR3	PPFIBP1	ENST000005	.	.	A	G	+	+	0.05714286	33,0,2,0	chr12,27847	Cytosol	A > G
chr12:31435408:T:C:UTR3:FAM60	chr12	31435408	31435408	T	C	UTR3	FAM60A	ENST000005	.	.	A	G	-	-	0.02173913	90,0,2,0	chr12,31435	Cytosol	A > G
chr12:34180221:A:G:ncRNA_exon	chr12	34180221	34180221	A	G	ncRNA_exon	AC046130.1	.	.	.	A	G	+	+-	0.6	2,0,3,0	chr12,34180	Cytosol	A > G
chr12:42753695:A:G:intronic:PPHL	chr12	42753695	42753695	A	G	intronic	PPHLN1	.	.	.	A	G	+	+	0.28571429	5,0,2,0	chr12,42753	Cytosol	A > G
chr12:42753700:A:G:intronic:PPHL	chr12	42753700	42753700	A	G	intronic	PPHLN1	.	.	.	A	G	+	+	0.875	1,0,7,0	chr12,42753	Cytosol	A > G
chr12:42753799:A:G:intronic:PPHL	chr12	42753799	42753799	A	G	intronic	PPHLN1	.	.	.	A	G	+	+	0.875	1,0,7,0	chr12,42753	Cytosol	A > G
chr12:42753813:A:G:intronic:PPHL	chr12	42753813	42753813	A	G	intronic	PPHLN1	.	.	.	A	G	+	+	0.42857143	4,0,3,0	chr12,42753	Cytosol	A > G
chr12:42754462:A:G:intronic:PPHL	chr12	42754462	42754462	A	G	intronic	PPHLN1	.	.	.	A	G	+	+	0.8	1,0,4,0	chr12,42754	Cytosol	A > G
chr12:42754480:A:G:intronic:PPHL	chr12	42754480	42754480	A	G	intronic	PPHLN1	.	.	.	A	G	+	+	0.71428571	2,0,5,0	chr12,42754	Cytosol	A > G
chr12:42754525:A:G:intronic:PPHL	chr12	42754525	42754525	A	G	intronic	PPHLN1	.	.	.	A	G	+	+	0.66666667	2,0,4,0	chr12,42754	Cytosol	A > G
chr12:42754556:A:G:intronic:PPHL	chr12	42754556	42754556	A	G	intronic	PPHLN1	.	.	.	A	G	+	+	0.4	3,0,2,0	chr12,42754	Cytosol	A > G
chr12:42754557:A:G:intronic:PPHL	chr12	42754557	42754557	A	G	intronic	PPHLN1	.	.	.	A	G	+	+	0.8	1,0,4,0	chr12,42754	Cytosol	A > G
chr12:44181631:A:G:UTR3:IRAK4	chr12	44181631	44181631	A	G	UTR3	IRAK4	ENST000004	.	.	A	G	+	+	0.06451613	29,0,2,0	chr12,44181	Cytosol	A > G
chr12:44181651:A:G:UTR3:IRAK4	chr12	44181651	44181651	A	G	UTR3	IRAK4	ENST000004	.	.	A	G	+	+	0.55172414	13,0,16,0	chr12,44181	Cytosol	A > G
chr12:44181673:A:G:UTR3:IRAK4	chr12	44181673	44181673	A	G	UTR3	IRAK4	ENST000004	.	.	A	G	+	+	0.6	8,0,12,0	chr12,44181	Cytosol	A > G
chr12:44181674:A:G:UTR3:IRAK4	chr12	44181674	44181674	A	G	UTR3	IRAK4	ENST000004	.	.	A	G	+	+	0.52380952	10,0,11,0	chr12,44181	Cytosol	A > G
chr12:44181683:A:G:UTR3:IRAK4	chr12	44181683	44181683	A	G	UTR3	IRAK4	ENST000004	.	.	A	G	+	+	0.27777778	13,0,5,0	chr12,44181	Cytosol	A > G
chr12:44181684:A:G:UTR3:IRAK4	chr12	44181684	44181684	A	G	UTR3	IRAK4	ENST000004	.	.	A	G	+	+	0.5625	7,0,9,0	chr12,44181	Cytosol	A > G
chr12:44181739:A:G:UTR3:IRAK4	chr12	44181739	44181739	A	G	UTR3	IRAK4	ENST000004	.	.	A	G	+	+	0.23529412	13,0,4,0	chr12,44181	Cytosol	A > G
chr12:44181749:A:G:UTR3:IRAK4	chr12	44181749	44181749	A	G	UTR3	IRAK4	ENST000004	.	.	A	G	+	+	0.11111111	16,0,2,0	chr12,44181	Cytosol	A > G
chr12:44182482:A:G:UTR3:IRAK4	chr12	44182482	44182482	A	G	UTR3	IRAK4	ENST000004	.	.	A	G	+	+	0.6	2,0,3,0	chr12,44182	Cytosol	A > G
chr12:44182551:A:G:UTR3:IRAK4	chr12	44182551	44182551	A	G	UTR3	IRAK4	ENST000004	.	.	A	G	+	+	1	0,0,16,0	chr12,44182	Cytosol	A > G
chr12:46314774:C:T:downstream:	chr12	46314774	46314774	C	T	downstream	SCAF11	.	.	.	G	A	-	-	0.02564103	3,0,114,0	chr12,46314	Cytosol	G > A
chr12:49326169:T:C:intronic:AC07	chr12	49326169	49326169	T	C	intronic	AC073610.5,	.	.	.	A	G	-	-	0.22222222	7,0,2,0	chr12,49326	Cytosol	A > G
chr12:49326171:T:C:intronic:AC07	chr12	49326171	49326171	T	C	intronic	AC073610.5,	.	.	.	A	G	-	-	0.77777778	2,0,7,0	chr12,49326	Cytosol	A > G
chr12:49328758:T:C:intronic:AC07	chr12	49328758	49328758	T	C	intronic	AC073610.5,	.	.	.	A	G	-	-	0.28571429	5,0,2,0	chr12,49328	Cytosol	A > G
chr12:49745431:A:G:UTR3:DNAJC	chr12	49745431	49745431	A	G	UTR3	DNAJC22	ENST000005	.	.	A	G	+	+	0.07692308	36,0,3,0	chr12,49745	Cytosol	A > G
chr12:49745500:A:G:UTR3:DNAJC	chr12	49745500	49745500	A	G	UTR3	DNAJC22	ENST000005	.	.	A	G	+	+	0.05714286	33,0,2,0	chr12,49745	Cytosol	A > G
chr12:49745536:A:G:UTR3:DNAJC	chr12	49745536	49745536	A	G	UTR3	DNAJC22	ENST000005	.	.	A	G	+	+	0.16129032	26,0,5,0	chr12,49745	Cytosol	A > G
chr12:49745562:A:G:UTR3:DNAJC	chr12	49745562	49745562	A	G	UTR3	DNAJC22	ENST000005	.	.	A	G	+	+	0.13636364	19,0,3,0	chr12,49745	Cytosol	A > G
chr12:49747267:A:G:intergenic:DN	chr12	49747267	49747267	A	G	intergenic	DNAJC22,SP	dist=1309;dis	.	.	A	G	+	+	0.32	34,0,16,0	chr12,49747	Cytosol	A > G
chr12:49747285:A:G:intergenic:DN	chr12	49747285	49747285	A	G	intergenic	DNAJC22,SP	dist=1327;dis	.	.	A	G	+	+	0.09090909	40,0,4,0	chr12,49747	Cytosol	A > G
chr12:49747289:A:G:intergenic:DN	chr12	49747289	49747289	A	G	intergenic	DNAJC22,SP	dist=1331;dis	.	.	A	G	+	+	0.375	30,0,18,0	chr12,49747	Cytosol	A > G
chr12:49747566:A:G:intergenic:DN	chr12	49747566	49747566	A	G	intergenic	DNAJC22,SP	dist=1608;dis	.	.	A	G	+	+	0.66666667	4,0,8,0	chr12,49747	Cytosol	A > G
chr12:49747589:A:G:intergenic:DN	chr12	49747589	49747589	A	G	intergenic	DNAJC22,SP	dist=1631;dis	.	.	A	G	+	+	0.54545455	5,0,6,0	chr12,49747	Cytosol	A > G
chr12:49747615:A:G:intergenic:DN	chr12	49747615	49747615	A	G	intergenic	DNAJC22,SP	dist=1657;dis	.	.	A	G	+	+	0.22222222	7,0,2,0	chr12,49747	Cytosol	A > G
chr12:49747625:A:G:intergenic:DN	chr12	49747625	49747625	A	G	intergenic	DNAJC22,SP	dist=1667;dis	.	.	A	G	+	+	0.38461538	8,0,5,0	chr12,49747	Cytosol	A > G
chr12:49747641:A:G:intergenic:DN	chr12	49747641	49747641	A	G	intergenic	DNAJC22,SP	dist=1683;dis	.	.	A	G	+	+	0.15384615	11,0,2,0	chr12,49747	Cytosol	A > G
chr12:49747652:A:G:intergenic:DN	chr12	49747652	49747652	A	G	intergenic	DNAJC22,SP	dist=1694;dis	.	.	A	G	+	+	0.28571429	10,0,4,0	chr12,49747	Cytosol	A > G
chr12:49747666:A:G:intergenic:DN	chr12	49747666	49747666	A	G	intergenic	DNAJC22,SP	dist=1708;dis	.	.	A	G	+	+	0.13333333	13,0,2,0	chr12,49747	Cytosol	A > G
chr12:49747683:A:G:intergenic:DN	chr12	49747683	49747683	A	G	intergenic	DNAJC22,SP	dist=1725;dis	.	.	A	G	+	+	0.41666667	7,0,5,0	chr12,49747	Cytosol	A > G
chr12:49749046:A:G:intergenic:DN	chr12	49749046	49749046	A	G	intergenic	DNAJC22,SP	dist=3088;dis	.	.	A	G	+	+	0.33333333	4,0,2,0	chr12,49749	Cytosol	A > G
chr12:49749059:A:G:intergenic:DN	chr12	49749059	49749059	A	G	intergenic	DNAJC22,SP	dist=3101;dis	.	.	A	G	+	+	0.5	3,0,3,0	chr12,49749	Cytosol	A > G
chr12:50689526:A:G:upstream:AC	chr12	50689526	50689526	A	G	upstream	AC140061.1	.	.	.	A	G	+		0.6	2,0,3,0	chr12,50689	Cytosol	A > G
chr12:51324085:A:G:UTR3:METTL	chr12	51324085	51324085	A	G	UTR3	METTL7A	ENST000005	.	.	A	G	+	+	0.02197802	89,0,2,0	chr12,51324	Cytosol	A > G
chr12:51324119:A:G:UTR3:METTL	chr12	51324119	51324119	A	G	UTR3	METTL7A	ENST000005	.	.	A	G	+	+	0.42622951	70,0,52,0	chr12,51324	Cytosol	A > G
chr12:51324122:A:G:UTR3:METTL	chr12	51324122	51324122	A	G	UTR3	METTL7A	ENST000005	.	.	A	G	+	+	0.56896552	49,1,66,0	chr12,51324	Cytosol	A > G
chr12:51324127:A:G:UTR3:METTL	chr12	51324127	51324127	A	G	UTR3	METTL7A	ENST000005	.	.	A	G	+	+	0.04310345	111,0,5,0	chr12,51324	Cytosol	A > G
chr12:51324163:A:G:UTR3:METTL	chr12	51324163	51324163	A	G	UTR3	METTL7A	ENST000005	.	.	A	G	+	+	0.22727273	51,0,15,0	chr12,51324	Cytosol	A > G
chr12:51324180:A:G:UTR3:METTL	chr12	51324180	51324180	A	G	UTR3	METTL7A	ENST000005	.	.	A	G	+	+	0.06521739	43,0,3,0	chr12,51324	Cytosol	A > G
chr12:51324192:A:G:UTR3:METTL	chr12	51324192	51324192	A	G	UTR3	METTL7A	ENST000005	.	.	A	G	+	+	0.25	39,0,13,0	chr12,51324	Cytosol	A > G
chr12:51324202:A:G:UTR3:METTL	chr12	51324202	51324202	A	G	UTR3	METTL7A	ENST000005	.	.	A	G	+	+	0.40952381	62,0,43,0	chr12,51324	Cytosol	A > G
chr12:51324241:A:G:UTR3:METTL	chr12	51324241	51324241	A	G	UTR3	METTL7A	ENST000005	.	.	A	G	+	+	0.035	193,0,7,0	chr12,51324	Cytosol	A > G
chr12:51324258:A:G:UTR3:METTL	chr12	51324258	51324258	A	G	UTR3	METTL7A	ENST000005	.	.	A	G	+	+	0.07172996	220,0,17,0	chr12,51324	Cytosol	A > G
chr12:51324308:A:G:UTR3:METTL	chr12	51324308	51324308	A	G	UTR3	METTL7A	ENST000005	.	.	A	G	+	+	0.05405405	140,0,8,0	chr12,51324	Cytosol	A > G
chr12:51324313:A:G:UTR3:METTL	chr12	51324313	51324313	A	G	UTR3	METTL7A	ENST000005	.	.	A	G	+	+	0.15116279	146,0,26,0	chr12,51324	Cytosol	A > G
chr12:51324322:A:G:UTR3:METTL	chr12	51324322	51324322	A	G	UTR3	METTL7A	ENST000005	.	.	A	G	+	+	0.02645503	183,0,5,1	chr12,51324	Cytosol	A > G
chr12:51324323:A:G:UTR3:METTL	chr12	51324323	51324323	A	G	UTR3	METTL7A	ENST000005	.	.	A	G	+	+	0.13978495	160,0,26,0	chr12,51324	Cytosol	A > G

chr12:51324384:A:G:UTR3:METTL	chr12	51324384	51324384	A	G	UTR3	METTL7A	ENST000005	.	.	A	G	+	+	0.01005025	197,0,2,0	chr12,51324	Cytosol	A > G
chr12:51324436:A:G:UTR3:METTL	chr12	51324436	51324436	A	G	UTR3	METTL7A	ENST000005	.	.	A	G	+	+	0.1712963	179,0,37,0	chr12,51324	Cytosol	A > G
chr12:51324447:A:G:UTR3:METTL	chr12	51324447	51324447	A	G	UTR3	METTL7A	ENST000005	.	.	A	G	+	+	0.04968944	153,0,8,0	chr12,51324	Cytosol	A > G
chr12:51324466:A:G:UTR3:METTL	chr12	51324466	51324466	A	G	UTR3	METTL7A	ENST000005	.	.	A	G	+	+	0.0625	150,0,10,0	chr12,51324	Cytosol	A > G
chr12:51324467:A:G:UTR3:METTL	chr12	51324467	51324467	A	G	UTR3	METTL7A	ENST000005	.	.	A	G	+	+	0.23602484	123,0,38,0	chr12,51324	Cytosol	A > G
chr12:51324496:A:G:UTR3:METTL	chr12	51324496	51324496	A	G	UTR3	METTL7A	ENST000005	.	.	A	G	+	+	0.22435897	120,0,35,1	chr12,51324	Cytosol	A > G
chr12:51324506:A:G:UTR3:METTL	chr12	51324506	51324506	A	G	UTR3	METTL7A	ENST000005	.	.	A	G	+	+	0.26153846	96,0,34,0	chr12,51324	Cytosol	A > G
chr12:51324508:A:G:UTR3:METTL	chr12	51324508	51324508	A	G	UTR3	METTL7A	ENST000005	.	.	A	G	+	+	0.04651163	123,0,6,0	chr12,51324	Cytosol	A > G
chr12:51324545:A:G:UTR3:METTL	chr12	51324545	51324545	A	G	UTR3	METTL7A	ENST000005	.	.	A	G	+	+	0.09574468	85,0,9,0	chr12,51324	Cytosol	A > G
chr12:51324627:A:G:UTR3:METTL	chr12	51324627	51324627	A	G	UTR3	METTL7A	ENST000005	.	.	A	G	+	+	0.39795918	118,0,78,0	chr12,51324	Cytosol	A > G
chr12:51324639:A:G:UTR3:METTL	chr12	51324639	51324639	A	G	UTR3	METTL7A	ENST000005	.	.	A	G	+	+	0.50207469	120,0,121,0	chr12,51324	Cytosol	A > G
chr12:51325229:A:G:UTR3:METTL	chr12	51325229	51325229	A	G	UTR3	METTL7A	ENST000005	.	.	A	G	+	+	0.08695652	105,0,10,0	chr12,51325	Cytosol	A > G
chr12:51325237:A:G:UTR3:METTL	chr12	51325237	51325237	A	G	UTR3	METTL7A	ENST000005	.	.	A	G	+	+	0.47933884	63,0,58,0	chr12,51325	Cytosol	A > G
chr12:51325238:A:G:UTR3:METTL	chr12	51325238	51325238	A	G	UTR3	METTL7A	ENST000005	.	.	A	G	+	+	0.34146341	81,0,42,0	chr12,51325	Cytosol	A > G
chr12:51325280:A:G:UTR3:METTL	chr12	51325280	51325280	A	G	UTR3	METTL7A	ENST000005	.	.	A	G	+	+	0.19463087	120,0,29,0	chr12,51325	Cytosol	A > G
chr12:51325287:A:G:UTR3:METTL	chr12	51325287	51325287	A	G	UTR3	METTL7A	ENST000005	.	.	A	G	+	+	0.17460317	104,0,22,0	chr12,51325	Cytosol	A > G
chr12:51325288:A:G:UTR3:METTL	chr12	51325288	51325288	A	G	UTR3	METTL7A	ENST000005	.	.	A	G	+	+	0.0610687	123,0,8,0	chr12,51325	Cytosol	A > G
chr12:51325306:A:G:UTR3:METTL	chr12	51325306	51325306	A	G	UTR3	METTL7A	ENST000005	.	.	A	G	+	+	0.08527132	118,0,11,0	chr12,51325	Cytosol	A > G
chr12:51325347:A:G:UTR3:METTL	chr12	51325347	51325347	A	G	UTR3	METTL7A	ENST000005	.	.	A	G	+	+	0.375	50,0,30,0	chr12,51325	Cytosol	A > G
chr12:51325351:A:G:UTR3:METTL	chr12	51325351	51325351	A	G	UTR3	METTL7A	ENST000005	.	.	A	G	+	+	0.13157895	66,0,10,0	chr12,51325	Cytosol	A > G
chr12:51325373:A:G:UTR3:METTL	chr12	51325373	51325373	A	G	UTR3	METTL7A	ENST000005	.	.	A	G	+	+	0.19327731	96,0,23,0	chr12,51325	Cytosol	A > G
chr12:51325374:A:G:UTR3:METTL	chr12	51325374	51325374	A	G	UTR3	METTL7A	ENST000005	.	.	A	G	+	+	0.12280702	100,0,14,0	chr12,51325	Cytosol	A > G
chr12:51325383:A:G:UTR3:METTL	chr12	51325383	51325383	A	G	UTR3	METTL7A	ENST000005	.	.	A	G	+	+	0.04237288	113,0,5,0	chr12,51325	Cytosol	A > G
chr12:51325389:A:G:UTR3:METTL	chr12	51325389	51325389	A	G	UTR3	METTL7A	ENST000005	.	.	A	G	+	+	0.03809524	101,0,4,0	chr12,51325	Cytosol	A > G
chr12:51325390:A:G:UTR3:METTL	chr12	51325390	51325390	A	G	UTR3	METTL7A	ENST000005	.	.	A	G	+	+	0.1980198	81,0,20,0	chr12,51325	Cytosol	A > G
chr12:51325419:A:G:UTR3:METTL	chr12	51325419	51325419	A	G	UTR3	METTL7A	ENST000005	.	.	A	G	+	+	0.20338983	47,0,12,0	chr12,51325	Cytosol	A > G
chr12:51325435:A:G:UTR3:METTL	chr12	51325435	51325435	A	G	UTR3	METTL7A	ENST000005	.	.	A	G	+	+	0.22727273	34,0,10,0	chr12,51325	Cytosol	A > G
chr12:51325440:A:G:UTR3:METTL	chr12	51325440	51325440	A	G	UTR3	METTL7A	ENST000005	.	.	A	G	+	+	0.35416667	31,0,17,0	chr12,51325	Cytosol	A > G
chr12:51325449:A:G:UTR3:METTL	chr12	51325449	51325449	A	G	UTR3	METTL7A	ENST000005	.	.	A	G	+	+	0.07692308	48,0,4,0	chr12,51325	Cytosol	A > G
chr12:51325535:A:G:UTR3:METTL	chr12	51325535	51325535	A	G	UTR3	METTL7A	ENST000005	.	.	A	G	+	+	0.01226994	161,0,2,0	chr12,51325	Cytosol	A > G
chr12:51325537:A:G:UTR3:METTL	chr12	51325537	51325537	A	G	UTR3	METTL7A	ENST000005	.	.	A	G	+	+	0.28658537	117,0,47,0	chr12,51325	Cytosol	A > G
chr12:53416839:A:G:ncRNA_intro	chr12	53416839	53416839	A	G	ncRNA_intro	RP11-983P1	.	.	.	A	G	+	+-	0.33333333	4,0,2,0	chr12,53416	Cytosol	A > G
chr12:53567832:T:C:intronic:CSAD	chr12	53567832	53567832	T	C	intronic	CSAD	.	.	.	A	G	-	-	0.33333333	4,0,2,0	chr12,53567	Cytosol	A > G
chr12:53567844:T:C:intronic:CSAD	chr12	53567844	53567844	T	C	intronic	CSAD	.	.	.	A	G	-	-	0.4	3,0,2,0	chr12,53567	Cytosol	A > G
chr12:53567845:T:C:intronic:CSAD	chr12	53567845	53567845	T	C	intronic	CSAD	.	.	.	A	G	-	-	0.8	1,0,4,0	chr12,53567	Cytosol	A > G
chr12:53568333:T:C:intronic:CSAD	chr12	53568333	53568333	T	C	intronic	CSAD	.	.	.	A	G	-	-	0.22222222	7,0,2,0	chr12,53568	Cytosol	A > G
chr12:53568334:T:C:intronic:CSAD	chr12	53568334	53568334	T	C	intronic	CSAD	.	.	.	A	G	-	-	0.22222222	7,0,2,0	chr12,53568	Cytosol	A > G
chr12:53568343:T:C:intronic:CSAD	chr12	53568343	53568343	T	C	intronic	CSAD	.	.	.	A	G	-	-	0.66666667	3,0,6,0	chr12,53568	Cytosol	A > G
chr12:53568361:T:C:intronic:CSAD	chr12	53568361	53568361	T	C	intronic	CSAD	.	.	.	A	G	-	-	0.33333333	6,0,3,0	chr12,53568	Cytosol	A > G
chr12:53570086:T:C:intronic:CSAD	chr12	53570086	53570086	T	C	intronic	CSAD	.	.	.	A	G	-	-	0.4	3,0,2,0	chr12,53570	Cytosol	A > G
chr12:53571355:T:C:intronic:CSAD	chr12	53571355	53571355	T	C	intronic	CSAD	.	.	.	A	G	-	-	0.33333333	4,0,2,0	chr12,53571	Cytosol	A > G
chr12:53572000:T:C:intronic:CSAD	chr12	53572000	53572000	T	C	intronic	CSAD	.	.	.	A	G	-	-	0.33333333	4,0,2,0	chr12,53571	Cytosol	A > G
chr12:53572006:T:C:intronic:CSAD	chr12	53572006	53572006	T	C	intronic	CSAD	.	.	.	A	G	-	-	0.83333333	1,0,5,0	chr12,53572	Cytosol	A > G
chr12:53879479:A:G:intronic:MAP	chr12	53879479	53879479	A	G	intronic	MAP3K12	.	.	.	A	G	+	-	0.33333333	4,0,2,0	chr12,53879	Cytosol	A > G
chr12:54627480:T:C:UTR3:CBX5	chr12	54627480	54627480	T	C	UTR3	CBX5	ENST000002	.	.	A	G	-	-	0.02214022	265,0,6,0	chr12,54627	Cytosol	A > G
chr12:54629209:T:C:UTR3:CBX5	chr12	54629209	54629209	T	C	UTR3	CBX5	ENST000002	.	.	A	G	-	-	0.13084112	93,0,14,0	chr12,54629	Cytosol	A > G
chr12:54631719:T:C:UTR3:CBX5	chr12	54631719	54631719	T	C	UTR3	CBX5	ENST000002	.	.	A	G	-	-	0.03529412	82,0,3,0	chr12,54631	Cytosol	A > G
chr12:54634283:T:C:UTR3:CBX5	chr12	54634283	54634283	T	C	UTR3	CBX5	ENST000002	.	.	A	G	-	-	0.22222222	77,0,22,0	chr12,54634	Cytosol	A > G
chr12:54634284:T:C:UTR3:CBX5	chr12	54634284	54634284	T	C	UTR3	CBX5	ENST000002	.	.	A	G	-	-	0.17307692	86,0,18,0	chr12,54634	Cytosol	A > G
chr12:54634300:T:C:UTR3:CBX5	chr12	54634300	54634300	T	C	UTR3	CBX5	ENST000002	.	.	A	G	-	-	0.08602151	85,0,8,0	chr12,54634	Cytosol	A > G
chr12:54634306:T:C:UTR3:CBX5	chr12	54634306	54634306	T	C	UTR3	CBX5	ENST000002	.	.	A	G	-	-	0.02105263	93,0,2,0	chr12,54634	Cytosol	A > G
chr12:56215627:A:G:UTR3:DNAJC	chr12	56215627	56215627	A	G	UTR3	DNAJC14,OR	ENST000003	.	.	A	G	+	+-	0.32258065	21,0,10,0	chr12,56215	Cytosol	A > G
chr12:56217537:A:G:intronic:DNA	chr12	56217537	56217537	A	G	intronic	DNAJC14,RP	.	.	.	A	G	+	-	0.88888889	1,0,8,0	chr12,56217	Cytosol	A > G
chr12:56218724:A:G:intronic:DNA	chr12	56218724	56218724	A	G	intronic	DNAJC14,RP	.	.	.	A	G	+	-	0.85714286	1,0,6,0	chr12,56218	Cytosol	A > G
chr12:56702088:T:C:ncRNA_intror	chr12	56702088	56702088	T	C	ncRNA_intro	RP11-977G1	.	.	.	A	G	-	-+	0.73913043	6,0,17,0	chr12,56702	Cytosol	A > G
chr12:56706520:T:C:ncRNA_intror	chr12	56706520	56706520	T	C	ncRNA_intro	RP11-977G1	.	.	.	A	G	-	+-	0.16666667	15,0,3,0	chr12,56706	Cytosol	A > G
chr12:56706628:T:C:ncRNA_intror	chr12	56706628	56706628	T	C	ncRNA_intro	RP11-977G1	.	.	.	A	G	-	+-	0.10526316	17,0,2,0	chr12,56706	Cytosol	A > G
chr12:57080296:T:C:intronic:PTGE	chr12	57080296	57080296	T	C	intronic	PTGES3	.	.	.	A	G	-	-	0.07692308	24,0,2,0	chr12,57080	Cytosol	A > G
chr12:57124262:T:C:intronic:NAC	chr12	57124262	57124262	T	C	intronic	NACA	.	.	.	A	G	-	-	0.13043478	20,0,3,0	chr12,57124	Cytosol	A > G
chr12:57124289:T:C:intronic:NAC	chr12	57124289	57124289	T	C	intronic	NACA	.	.	.	A	G	-	-	0.36666667	19,0,11,0	chr12,57124	Cytosol	A > G
chr12:57124316:T:C:intronic:NAC	chr12	57124316	57124316	T	C	intronic	NACA	.	.	.	A	G	-	-	0.52941176	16,0,18,0	chr12,57124	Cytosol	A > G
chr12:57124320:T:C:intronic:NAC	chr12	57124320	57124320	T	C	intronic	NACA	.	.	.	A	G	-	-	0.11627907	38,0,5,0	chr12,57124	Cytosol	A > G
chr12:57124324:T:C:intronic:NAC	chr12	57124324	57124324	T	C	intronic	NACA	.	.	.	A	G	-	-	0.175	33,0,7,0	chr12,57124	Cytosol	A > G
chr12:57124327:T:C:intronic:NAC	chr12	57124327	57124327	T	C	intronic	NACA	.	.	.	A	G	-	-	0.05	38,0,2,0	chr12,57124	Cytosol	A > G

chr12:57124347:T:C:intronic:NAC	chr12	57124347	57124347	T	C	intronic	NACA	.	.	.	A	G	-	-	0.32727273	37,0,18,0	chr12,57124	Cytosol	A > G
chr12:57124357:T:C:intronic:NAC	chr12	57124357	57124357	T	C	intronic	NACA	.	.	.	A	G	-	-	0.33783784	49,0,25,0	chr12,57124	Cytosol	A > G
chr12:57124358:T:C:intronic:NAC	chr12	57124358	57124358	T	C	intronic	NACA	.	.	.	A	G	-	-	0.23376623	59,0,18,0	chr12,57124	Cytosol	A > G
chr12:57124411:T:C:intronic:NAC	chr12	57124411	57124411	T	C	intronic	NACA	.	.	.	A	G	-	-	0.06153846	61,0,4,0	chr12,57124	Cytosol	A > G
chr12:57124413:T:C:intronic:NAC	chr12	57124413	57124413	T	C	intronic	NACA	.	.	.	A	G	-	-	0.10909091	49,0,6,0	chr12,57124	Cytosol	A > G
chr12:57124425:T:C:intronic:NAC	chr12	57124425	57124425	T	C	intronic	NACA	.	.	.	A	G	-	-	0.58928571	23,0,33,0	chr12,57124	Cytosol	A > G
chr12:57124426:T:C:intronic:NAC	chr12	57124426	57124426	T	C	intronic	NACA	.	.	.	A	G	-	-	0.31578947	39,0,18,0	chr12,57124	Cytosol	A > G
chr12:57124435:T:C:intronic:NAC	chr12	57124435	57124435	T	C	intronic	NACA	.	.	.	A	G	-	-	0.71929825	16,0,41,0	chr12,57124	Cytosol	A > G
chr12:57124476:T:C:intronic:NAC	chr12	57124476	57124476	T	C	intronic	NACA	.	.	.	A	G	-	-	0.07692308	36,0,3,0	chr12,57124	Cytosol	A > G
chr12:57124480:T:C:intronic:NAC	chr12	57124480	57124480	T	C	intronic	NACA	.	.	.	A	G	-	-	1	0,0,45,0	chr12,57124	Cytosol	A > G
chr12:57125135:T:C:intronic:NAC	chr12	57125135	57125135	T	C	intronic	NACA	.	.	.	A	G	-	-	0.63218391	32,0,55,0	chr12,57125	Cytosol	A > G
chr12:57125139:T:C:intronic:NAC	chr12	57125139	57125139	T	C	intronic	NACA	.	.	.	A	G	-	-	0.38372093	53,0,33,0	chr12,57125	Cytosol	A > G
chr12:57125180:T:C:intronic:NAC	chr12	57125180	57125180	T	C	intronic	NACA	.	.	.	A	G	-	-	0.54117647	39,0,46,0	chr12,57125	Cytosol	A > G
chr12:57125181:T:C:intronic:NAC	chr12	57125181	57125181	T	C	intronic	NACA	.	.	.	A	G	-	-	0.27058824	62,0,23,0	chr12,57125	Cytosol	A > G
chr12:57125246:T:C:intronic:NAC	chr12	57125246	57125246	T	C	intronic	NACA	.	.	.	A	G	-	-	0.33928571	37,0,19,0	chr12,57125	Cytosol	A > G
chr12:57715826:T:C:intronic:R3H	chr12	57715826	57715826	T	C	intronic	R3HDM2	.	.	.	A	G	-	-	0.375	5,0,3,0	chr12,57715	Cytosol	A > G
chr12:57822572:T:C:intronic:R3H	chr12	57822572	57822572	T	C	intronic	R3HDM2	.	.	.	A	G	-	-	0.33333333	4,0,2,0	chr12,57822	Cytosol	A > G
chr12:57822603:T:C:intronic:R3H	chr12	57822603	57822603	T	C	intronic	R3HDM2	.	.	.	A	G	-	-	0.66666667	2,0,4,0	chr12,57822	Cytosol	A > G
chr12:64585731:T:C:intronic:C12o	chr12	64585731	64585731	T	C	intronic	C12orf66	.	.	.	A	G	-	-	0.57142857	3,0,4,0	chr12,64585	Cytosol	A > G
chr12:64585972:T:C:intronic:C12o	chr12	64585972	64585972	T	C	intronic	C12orf66	.	.	.	A	G	-	-	0.44444444	5,0,4,0	chr12,64585	Cytosol	A > G
chr12:64585977:T:C:intronic:C12o	chr12	64585977	64585977	T	C	intronic	C12orf66	.	.	.	A	G	-	-	0.42857143	4,0,3,0	chr12,64585	Cytosol	A > G
chr12:64586016:T:C:intronic:C12o	chr12	64586016	64586016	T	C	intronic	C12orf66	.	.	.	A	G	-	-	0.4	9,0,6,0	chr12,64586	Cytosol	A > G
chr12:64586169:T:C:intronic:C12o	chr12	64586169	64586169	T	C	intronic	C12orf66	.	.	.	A	G	-	-	0.46666667	8,0,7,0	chr12,64586	Cytosol	A > G
chr12:64586177:T:C:intronic:C12o	chr12	64586177	64586177	T	C	intronic	C12orf66	.	.	.	A	G	-	-	0.4	9,0,6,0	chr12,64586	Cytosol	A > G
chr12:64586196:T:C:intronic:C12o	chr12	64586196	64586196	T	C	intronic	C12orf66	.	.	.	A	G	-	-	0.21428571	11,0,3,0	chr12,64586	Cytosol	A > G
chr12:64842759:A:G:UTR3:XPOT	chr12	64842759	64842759	A	G	UTR3	XPOT	ENST000003	.	.	A	G	+	+	0.125	35,0,5,0	chr12,64842	Cytosol	A > G
chr12:64842769:A:G:UTR3:XPOT	chr12	64842769	64842769	A	G	UTR3	XPOT	ENST000003	.	.	A	G	+	+	0.29508197	43,0,18,0	chr12,64842	Cytosol	A > G
chr12:64842793:A:G:UTR3:XPOT	chr12	64842793	64842793	A	G	UTR3	XPOT	ENST000003	.	.	A	G	+	+	0.171875	53,0,11,0	chr12,64842	Cytosol	A > G
chr12:64842802:A:G:UTR3:XPOT	chr12	64842802	64842802	A	G	UTR3	XPOT	ENST000003	.	.	A	G	+	+	0.11111111	56,0,7,0	chr12,64842	Cytosol	A > G
chr12:64842832:A:G:UTR3:XPOT	chr12	64842832	64842832	A	G	UTR3	XPOT	ENST000003	.	.	A	G	+	+	0.10416667	43,0,5,0	chr12,64842	Cytosol	A > G
chr12:64842854:A:G:UTR3:XPOT	chr12	64842854	64842854	A	G	UTR3	XPOT	ENST000003	.	.	A	G	+	+	0.75438596	14,0,43,0	chr12,64842	Cytosol	A > G
chr12:64842855:A:G:UTR3:XPOT	chr12	64842855	64842855	A	G	UTR3	XPOT	ENST000003	.	.	A	G	+	+	0.76923077	12,0,40,0	chr12,64842	Cytosol	A > G
chr12:64842864:A:G:UTR3:XPOT	chr12	64842864	64842864	A	G	UTR3	XPOT	ENST000003	.	.	A	G	+	+	0.27083333	35,0,13,0	chr12,64842	Cytosol	A > G
chr12:64842920:A:G:UTR3:XPOT	chr12	64842920	64842920	A	G	UTR3	XPOT	ENST000003	.	.	A	G	+	+	0.21875	25,0,7,0	chr12,64842	Cytosol	A > G
chr12:64844061:A:G:UTR3:XPOT	chr12	64844061	64844061	A	G	UTR3	XPOT	ENST000003	.	.	A	G	+	+	0.34042553	31,0,16,0	chr12,64844	Cytosol	A > G
chr12:64844071:A:G:UTR3:XPOT	chr12	64844071	64844071	A	G	UTR3	XPOT	ENST000003	.	.	A	G	+	+	0.86666667	6,0,39,0	chr12,64844	Cytosol	A > G
chr12:64844118:A:G:UTR3:XPOT	chr12	64844118	64844118	A	G	UTR3	XPOT	ENST000003	.	.	A	G	+	+	0.07692308	60,0,5,0	chr12,64844	Cytosol	A > G
chr12:64844134:A:G:UTR3:XPOT	chr12	64844134	64844134	A	G	UTR3	XPOT	ENST000003	.	.	A	G	+	+	0.57971014	29,0,40,0	chr12,64844	Cytosol	A > G
chr12:64844149:A:G:UTR3:XPOT	chr12	64844149	64844149	A	G	UTR3	XPOT	ENST000003	.	.	A	G	+	+	0.65789474	26,0,50,0	chr12,64844	Cytosol	A > G
chr12:64844167:A:G:UTR3:XPOT	chr12	64844167	64844167	A	G	UTR3	XPOT	ENST000003	.	.	A	G	+	+	0.13	87,0,13,0	chr12,64844	Cytosol	A > G
chr12:64844199:A:G:UTR3:XPOT	chr12	64844199	64844199	A	G	UTR3	XPOT	ENST000003	.	.	A	G	+	+	0.025	78,0,2,0	chr12,64844	Cytosol	A > G
chr12:66515569:T:C:intergenic:RN	chr12	66515569	66515569	T	C	intergenic	RNA5SP362,I	dist=55451;d	.	.	A	G	-		0.4	3,0,2,0	chr12,66515	Cytosol	A > G
chr12:66517184:T:C:UTR3:LLPH	chr12	66517184	66517184	T	C	UTR3	LLPH	ENST000002	.	.	A	G	-	-	0.01724138	171,0,3,0	chr12,66517	Cytosol	A > G
chr12:67712044:A:G:UTR3:CAND1	chr12	67712044	67712044	A	G	UTR3	CAND1	ENST000005	.	.	A	G	+	+	0.5	3,0,3,0	chr12,67712	Cytosol	A > G
chr12:69018475:A:G:UTR5:RAP1B	chr12	69018475	69018475	A	G	UTR5	RAP1B	ENST000004	.	.	A	G	+	+	0.23076923	10,0,3,0	chr12,69018	Cytosol	A > G
chr12:69018570:A:G:UTR5:RAP1B	chr12	69018570	69018570	A	G	UTR5	RAP1B	ENST000004	.	.	A	G	+	+	0.16666667	35,0,7,0	chr12,69018	Cytosol	A > G
chr12:69158117:A:G:intronic:SLC3	chr12	69158117	69158117	A	G	intronic	SLC35E3	.	.	.	A	G	+	+	0.6	2,0,3,0	chr12,69158	Cytosol	A > G
chr12:69158278:A:G:intronic:SLC3	chr12	69158278	69158278	A	G	intronic	SLC35E3	.	.	.	A	G	+	+	0.4	3,0,2,0	chr12,69158	Cytosol	A > G
chr12:69158289:A:G:intronic:SLC3	chr12	69158289	69158289	A	G	intronic	SLC35E3	.	.	.	A	G	+	+	0.375	5,0,3,0	chr12,69158	Cytosol	A > G
chr12:69158320:A:G:intronic:SLC3	chr12	69158320	69158320	A	G	intronic	SLC35E3	.	.	.	A	G	+	+	0.33333333	6,0,3,0	chr12,69158	Cytosol	A > G
chr12:69158881:A:G:UTR3:SLC35E	chr12	69158881	69158881	A	G	UTR3	SLC35E3	ENST000003	.	.	A	G	+	+	0.25	12,0,4,0	chr12,69158	Cytosol	A > G
chr12:69158951:A:G:UTR3:SLC35E	chr12	69158951	69158951	A	G	UTR3	SLC35E3	ENST000003	.	.	A	G	+	+	0.17647059	42,0,9,0	chr12,69158	Cytosol	A > G
chr12:69159247:A:G:UTR3:SLC35E	chr12	69159247	69159247	A	G	UTR3	SLC35E3	ENST000003	.	.	A	G	+	+	0.02272727	86,0,2,0	chr12,69159	Cytosol	A > G
chr12:69159309:A:G:UTR3:SLC35E	chr12	69159309	69159309	A	G	UTR3	SLC35E3	ENST000003	.	.	A	G	+	+	0.06451613	58,0,4,0	chr12,69159	Cytosol	A > G
chr12:69159324:A:G:UTR3:SLC35E	chr12	69159324	69159324	A	G	UTR3	SLC35E3	ENST000003	.	.	A	G	+	+	0.04166667	46,0,2,0	chr12,69159	Cytosol	A > G
chr12:69160065:A:G:UTR3:SLC35E	chr12	69160065	69160065	A	G	UTR3	SLC35E3	ENST000003	.	.	A	G	+	+	0.18181818	9,0,2,0	chr12,69160	Cytosol	A > G
chr12:69160066:A:G:UTR3:SLC35E	chr12	69160066	69160066	A	G	UTR3	SLC35E3	ENST000003	.	.	A	G	+	+	0.27272727	8,0,3,0	chr12,69160	Cytosol	A > G
chr12:69160102:A:G:UTR3:SLC35E	chr12	69160102	69160102	A	G	UTR3	SLC35E3	ENST000003	.	.	A	G	+	+	0.72222222	5,0,13,0	chr12,69160	Cytosol	A > G
chr12:69160105:A:G:UTR3:SLC35E	chr12	69160105	69160105	A	G	UTR3	SLC35E3	ENST000003	.	.	A	G	+	+	0.4	9,0,6,0	chr12,69160	Cytosol	A > G
chr12:69160108:A:G:UTR3:SLC35E	chr12	69160108	69160108	A	G	UTR3	SLC35E3	ENST000003	.	.	A	G	+	+	0.15789474	16,0,3,0	chr12,69160	Cytosol	A > G
chr12:69160109:A:G:UTR3:SLC35E	chr12	69160109	69160109	A	G	UTR3	SLC35E3	ENST000003	.	.	A	G	+	+	0.10526316	17,0,2,0	chr12,69160	Cytosol	A > G
chr12:69160146:A:G:UTR3:SLC35E	chr12	69160146	69160146	A	G	UTR3	SLC35E3	ENST000003	.	.	A	G	+	+	0.55555556	12,0,15,0	chr12,69160	Cytosol	A > G
chr12:69160187:A:G:UTR3:SLC35E	chr12	69160187	69160187	A	G	UTR3	SLC35E3	ENST000003	.	.	A	G	+	+	0.2962963	19,0,8,0	chr12,69160	Cytosol	A > G
chr12:69160398:A:G:UTR3:SLC35E	chr12	69160398	69160398	A	G	UTR3	SLC35E3	ENST000003	.	.	A	G	+	+	0.26315789	14,0,5,0	chr12,69160	Cytosol	A > G

chr12:69160416:A:G:exonic:SLC35	chr12	69160416	69160416	A	G	exonic	SLC35E3	.	synonymous	SLC35E3:ENS	A	G	+	+	0.35714286	9,0,5,0	chr12,69160	Cytosol	A > G
chr12:69160428:A:G:exonic:SLC35	chr12	69160428	69160428	A	G	exonic	SLC35E3	.	synonymous	SLC35E3:ENS	A	G	+	+	0.2	8,0,2,0	chr12,69160	Cytosol	A > G
chr12:69160464:A:G:exonic:SLC35	chr12	69160464	69160464	A	G	exonic	SLC35E3	.	synonymous	SLC35E3:ENS	A	G	+	+	0.27586207	21,0,8,0	chr12,69160	Cytosol	A > G
chr12:69160465:A:G:exonic:SLC35	chr12	69160465	69160465	A	G	exonic	SLC35E3	.	nonsynonym	SLC35E3:ENS	A	G	+	+	0.17241379	24,0,5,0	chr12,69160	Cytosol	A > G
chr12:69160468:A:G:exonic:SLC35	chr12	69160468	69160468	A	G	exonic	SLC35E3	.	nonsynonym	SLC35E3:ENS	A	G	+	+	0.5	15,0,15,0	chr12,69160	Cytosol	A > G
chr12:69160482:A:G:exonic:SLC35	chr12	69160482	69160482	A	G	exonic	SLC35E3	.	synonymous	SLC35E3:ENS	A	G	+	+	0.26666667	22,0,8,0	chr12,69160	Cytosol	A > G
chr12:69160483:A:G:exonic:SLC35	chr12	69160483	69160483	A	G	exonic	SLC35E3	.	nonsynonym	SLC35E3:ENS	A	G	+	+	0.6	12,0,18,0	chr12,69160	Cytosol	A > G
chr12:69160504:A:G:exonic:SLC35	chr12	69160504	69160504	A	G	exonic	SLC35E3	.	nonsynonym	SLC35E3:ENS	A	G	+	+	0.21875	25,0,7,0	chr12,69160	Cytosol	A > G
chr12:69160537:A:G:exonic:SLC35	chr12	69160537	69160537	A	G	exonic	SLC35E3	.	nonsynonym	SLC35E3:ENS	A	G	+	+	0.53846154	6,0,7,0	chr12,69160	Cytosol	A > G
chr12:69160539:A:G:exonic:SLC35	chr12	69160539	69160539	A	G	exonic	SLC35E3	.	nonsynonym	SLC35E3:ENS	A	G	+	+	0.15384615	11,0,2,0	chr12,69160	Cytosol	A > G
chr12:69160554:A:G:exonic:SLC35	chr12	69160554	69160554	A	G	exonic	SLC35E3	.	synonymous	SLC35E3:ENS	A	G	+	+	0.33333333	4,0,2,0	chr12,69160	Cytosol	A > G
chr12:69160599:A:G:exonic:SLC35	chr12	69160599	69160599	A	G	exonic	SLC35E3	.	synonymous	SLC35E3:ENS	A	G	+	+	0.125	21,0,3,0	chr12,69160	Cytosol	A > G
chr12:69160600:A:G:exonic:SLC35	chr12	69160600	69160600	A	G	exonic	SLC35E3	.	nonsynonym	SLC35E3:ENS	A	G	+	+	0.40909091	13,0,9,0	chr12,69160	Cytosol	A > G
chr12:69160639:A:G:exonic:SLC35	chr12	69160639	69160639	A	G	exonic	SLC35E3	.	nonsynonym	SLC35E3:ENS	A	G	+	+	0.05555556	34,0,2,0	chr12,69160	Cytosol	A > G
chr12:69161123:A:G:UTR3:SLC35E	chr12	69161123	69161123	A	G	UTR3	SLC35E3	ENST000003	.	.	A	G	+	+	0.5	5,0,5,0	chr12,69161	Cytosol	A > G
chr12:69161190:A:G:UTR3:SLC35E	chr12	69161190	69161190	A	G	UTR3	SLC35E3	ENST000003	.	.	A	G	+	+	0.18181818	9,0,2,0	chr12,69161	Cytosol	A > G
chr12:69161199:A:G:UTR3:SLC35E	chr12	69161199	69161199	A	G	UTR3	SLC35E3	ENST000003	.	.	A	G	+	+	0.3	7,0,3,0	chr12,69161	Cytosol	A > G
chr12:69237004:A:G:ncRNA_exoni	chr12	69237004	69237004	A	G	ncRNA_exon	RP11-611O2.	.	.	.	A	G	+	-+	0.23880597	51,0,16,0	chr12,69237	Cytosol	A > G
chr12:69237013:A:G:ncRNA_exoni	chr12	69237013	69237013	A	G	ncRNA_exon	RP11-611O2.	.	.	.	A	G	+	-+	0.16393443	51,0,10,0	chr12,69237	Cytosol	A > G
chr12:69237014:A:G:ncRNA_exoni	chr12	69237014	69237014	A	G	ncRNA_exon	RP11-611O2.	.	.	.	A	G	+	-+	0.15	51,0,9,0	chr12,69237	Cytosol	A > G
chr12:69237020:A:G:UTR3:MDM2	chr12	69237020	69237020	A	G	UTR3	MDM2	ENST000004	.	.	A	G	+	-+	0.078125	59,0,5,0	chr12,69237	Cytosol	A > G
chr12:69237021:A:G:UTR3:MDM2	chr12	69237021	69237021	A	G	UTR3	MDM2	ENST000004	.	.	A	G	+	-+	0.09230769	59,0,6,0	chr12,69237	Cytosol	A > G
chr12:69237043:A:G:UTR3:MDM2	chr12	69237043	69237043	A	G	UTR3	MDM2	ENST000004	.	.	A	G	+	-+	0.28301887	76,0,30,0	chr12,69237	Cytosol	A > G
chr12:69237053:A:G:UTR3:MDM2	chr12	69237053	69237053	A	G	UTR3	MDM2	ENST000004	.	.	A	G	+	-+	0.152	105,1,19,0	chr12,69237	Cytosol	A > G
chr12:69237056:A:G:UTR3:MDM2	chr12	69237056	69237056	A	G	UTR3	MDM2	ENST000004	.	.	A	G	+	-+	0.18181818	108,0,24,0	chr12,69237	Cytosol	A > G
chr12:69237073:A:G:UTR3:MDM2	chr12	69237073	69237073	A	G	UTR3	MDM2	ENST000004	.	.	A	G	+	-+	0.13432836	116,0,18,0	chr12,69237	Cytosol	A > G
chr12:69237509:A:G:UTR3:MDM2	chr12	69237509	69237509	A	G	UTR3	MDM2	ENST000004	.	.	A	G	+	-+	0.21428571	44,0,12,0	chr12,69237	Cytosol	A > G
chr12:69237510:A:G:UTR3:MDM2	chr12	69237510	69237510	A	G	UTR3	MDM2	ENST000004	.	.	A	G	+	-+	0.14035088	49,0,8,0	chr12,69237	Cytosol	A > G
chr12:69237519:A:G:UTR3:MDM2	chr12	69237519	69237519	A	G	UTR3	MDM2	ENST000004	.	.	A	G	+	-+	0.24528302	40,0,13,0	chr12,69237	Cytosol	A > G
chr12:69237526:A:G:UTR3:MDM2	chr12	69237526	69237526	A	G	UTR3	MDM2	ENST000004	.	.	A	G	+	-+	0.14	43,0,7,0	chr12,69237	Cytosol	A > G
chr12:69237534:A:G:UTR3:MDM2	chr12	69237534	69237534	A	G	UTR3	MDM2	ENST000004	.	.	A	G	+	-+	0.5	21,0,21,0	chr12,69237	Cytosol	A > G
chr12:69245141:T:C:UTR3:CPM	chr12	69245141	69245141	T	C	UTR3	CPM	ENST000005	.	.	A	G	-	-	0.16666667	20,0,4,0	chr12,69245	Cytosol	A > G
chr12:69245142:T:C:UTR3:CPM	chr12	69245142	69245142	T	C	UTR3	CPM	ENST000005	.	.	A	G	-	-	0.26923077	19,0,7,0	chr12,69245	Cytosol	A > G
chr12:69245202:T:C:UTR3:CPM	chr12	69245202	69245202	T	C	UTR3	CPM	ENST000005	.	.	A	G	-	-	0.88	3,0,22,0	chr12,69245	Cytosol	A > G
chr12:69245224:T:C:UTR3:CPM	chr12	69245224	69245224	T	C	UTR3	CPM	ENST000005	.	.	A	G	-	-	0.64285714	5,0,9,0	chr12,69245	Cytosol	A > G
chr12:69245269:T:C:UTR3:CPM	chr12	69245269	69245269	T	C	UTR3	CPM	ENST000005	.	.	A	G	-	-	0.66666667	4,0,8,0	chr12,69245	Cytosol	A > G
chr12:69245304:T:C:UTR3:CPM	chr12	69245304	69245304	T	C	UTR3	CPM	ENST000005	.	.	A	G	-	-	0.6	8,0,12,0	chr12,69245	Cytosol	A > G
chr12:69245305:T:C:UTR3:CPM	chr12	69245305	69245305	T	C	UTR3	CPM	ENST000005	.	.	A	G	-	-	0.39130435	14,0,9,0	chr12,69245	Cytosol	A > G
chr12:69245308:T:C:UTR3:CPM	chr12	69245308	69245308	T	C	UTR3	CPM	ENST000005	.	.	A	G	-	-	1	0,0,23,0	chr12,69245	Cytosol	A > G
chr12:69245309:T:C:UTR3:CPM	chr12	69245309	69245309	T	C	UTR3	CPM	ENST000005	.	.	A	G	-	-	0.63636364	8,0,14,0	chr12,69245	Cytosol	A > G
chr12:69245334:T:C:UTR3:CPM	chr12	69245334	69245334	T	C	UTR3	CPM	ENST000005	.	.	A	G	-	-	0.06896552	27,0,2,0	chr12,69245	Cytosol	A > G
chr12:69245371:T:C:UTR3:CPM	chr12	69245371	69245371	T	C	UTR3	CPM	ENST000005	.	.	A	G	-	-	0.41935484	18,0,13,0	chr12,69245	Cytosol	A > G
chr12:69245372:T:C:UTR3:CPM	chr12	69245372	69245372	T	C	UTR3	CPM	ENST000005	.	.	A	G	-	-	0.51612903	15,0,16,0	chr12,69245	Cytosol	A > G
chr12:69246367:T:C:UTR3:CPM	chr12	69246367	69246367	T	C	UTR3	CPM	ENST000005	.	.	A	G	-	-	0.28301887	38,0,15,0	chr12,69246	Cytosol	A > G
chr12:69246426:T:C:UTR3:CPM	chr12	69246426	69246426	T	C	UTR3	CPM	ENST000005	.	.	A	G	-	-	0.33333333	16,0,8,0	chr12,69246	Cytosol	A > G
chr12:69246429:T:C:UTR3:CPM	chr12	69246429	69246429	T	C	UTR3	CPM	ENST000005	.	.	A	G	-	-	0.5	12,0,12,0	chr12,69246	Cytosol	A > G
chr12:69246433:T:C:UTR3:CPM	chr12	69246433	69246433	T	C	UTR3	CPM	ENST000005	.	.	A	G	-	-	0.20833333	19,0,5,0	chr12,69246	Cytosol	A > G
chr12:69246475:T:C:UTR3:CPM	chr12	69246475	69246475	T	C	UTR3	CPM	ENST000005	.	.	A	G	-	-	0.54545455	10,0,12,0	chr12,69246	Cytosol	A > G
chr12:69246476:T:C:UTR3:CPM	chr12	69246476	69246476	T	C	UTR3	CPM	ENST000005	.	.	A	G	-	-	0.38095238	12,0,8,1	chr12,69246	Cytosol	A > G
chr12:69246508:T:C:UTR3:CPM	chr12	69246508	69246508	T	C	UTR3	CPM	ENST000005	.	.	A	G	-	-	0.375	5,0,3,0	chr12,69246	Cytosol	A > G
chr12:69246545:T:C:UTR3:CPM	chr12	69246545	69246545	T	C	UTR3	CPM	ENST000005	.	.	A	G	-	-	0.29411765	12,0,5,0	chr12,69246	Cytosol	A > G
chr12:69246546:T:C:UTR3:CPM	chr12	69246546	69246546	T	C	UTR3	CPM	ENST000005	.	.	A	G	-	-	0.625	6,0,10,0	chr12,69246	Cytosol	A > G
chr12:69246569:T:C:UTR3:CPM	chr12	69246569	69246569	T	C	UTR3	CPM	ENST000005	.	.	A	G	-	-	0.55555556	12,0,15,0	chr12,69246	Cytosol	A > G
chr12:69246614:T:C:UTR3:CPM	chr12	69246614	69246614	T	C	UTR3	CPM	ENST000005	.	.	A	G	-	-	0.08333333	33,0,3,0	chr12,69246	Cytosol	A > G
chr12:69249643:T:C:UTR3:CPM	chr12	69249643	69249643	T	C	UTR3	CPM	ENST000005	.	.	A	G	-	-	0.03278689	236,0,8,0	chr12,69249	Cytosol	A > G
chr12:69249656:T:C:UTR3:CPM	chr12	69249656	69249656	T	C	UTR3	CPM	ENST000005	.	.	A	G	-	-	0.02222222	220,0,5,0	chr12,69249	Cytosol	A > G
chr12:69249728:T:C:UTR3:CPM	chr12	69249728	69249728	T	C	UTR3	CPM	ENST000005	.	.	A	G	-	-	0.01310044	226,0,3,0	chr12,69249	Cytosol	A > G
chr12:72096123:A:G:UTR3:TMEM	chr12	72096123	72096123	A	G	UTR3	TMEM19	ENST000002	.	.	A	G	+	+	0.04477612	64,0,3,0	chr12,72096	Cytosol	A > G
chr12:72096125:A:G:UTR3:TMEM	chr12	72096125	72096125	A	G	UTR3	TMEM19	ENST000002	.	.	A	G	+	+	0.11111111	64,0,8,0	chr12,72096	Cytosol	A > G
chr12:72096200:A:G:UTR3:TMEM	chr12	72096200	72096200	A	G	UTR3	TMEM19	ENST000002	.	.	A	G	+	+	0.06	47,0,3,0	chr12,72096	Cytosol	A > G
chr12:72096206:A:G:UTR3:TMEM	chr12	72096206	72096206	A	G	UTR3	TMEM19	ENST000002	.	.	A	G	+	+	0.04347826	44,0,2,0	chr12,72096	Cytosol	A > G
chr12:72096213:A:G:UTR3:TMEM	chr12	72096213	72096213	A	G	UTR3	TMEM19	ENST000002	.	.	A	G	+	+	0.21428571	33,0,9,0	chr12,72096	Cytosol	A > G
chr12:72096221:A:G:UTR3:TMEM	chr12	72096221	72096221	A	G	UTR3	TMEM19	ENST000002	.	.	A	G	+	+	0.72727273	9,0,24,0	chr12,72096	Cytosol	A > G
chr12:72096222:A:G:UTR3:TMEM	chr12	72096222	72096222	A	G	UTR3	TMEM19	ENST000002	.	.	A	G	+	+	0.94285714	2,0,33,0	chr12,72096	Cytosol	A > G

chr12:72096225:A:G:UTR3:TMEM	chr12	72096225	72096225	A	G	UTR3	TMEM19	ENST000002	.	.	A	G	+	+	0.94285714	2,0,33,0	chr12,72096	Cytosol	A > G
chr12:72096237:A:G:UTR3:TMEM	chr12	72096237	72096237	A	G	UTR3	TMEM19	ENST000002	.	.	A	G	+	+	0.28	18,0,7,0	chr12,72096	Cytosol	A > G
chr12:72096276:A:G:UTR3:TMEM	chr12	72096276	72096276	A	G	UTR3	TMEM19	ENST000002	.	.	A	G	+	+	0.17241379	24,0,5,0	chr12,72096	Cytosol	A > G
chr12:72096279:A:G:UTR3:TMEM	chr12	72096279	72096279	A	G	UTR3	TMEM19	ENST000002	.	.	A	G	+	+	0.30769231	18,0,8,0	chr12,72096	Cytosol	A > G
chr12:72096285:A:G:UTR3:TMEM	chr12	72096285	72096285	A	G	UTR3	TMEM19	ENST000002	.	.	A	G	+	+	0.4	15,0,10,0	chr12,72096	Cytosol	A > G
chr12:72096318:A:G:UTR3:TMEM	chr12	72096318	72096318	A	G	UTR3	TMEM19	ENST000002	.	.	A	G	+	+	0.2	36,0,9,0	chr12,72096	Cytosol	A > G
chr12:72096330:A:G:UTR3:TMEM	chr12	72096330	72096330	A	G	UTR3	TMEM19	ENST000002	.	.	A	G	+	+	0.45652174	25,0,21,0	chr12,72096	Cytosol	A > G
chr12:72096331:A:G:UTR3:TMEM	chr12	72096331	72096331	A	G	UTR3	TMEM19	ENST000002	.	.	A	G	+	+	0.80851064	9,0,38,0	chr12,72096	Cytosol	A > G
chr12:72096348:A:G:UTR3:TMEM	chr12	72096348	72096348	A	G	UTR3	TMEM19	ENST000002	.	.	A	G	+	+	0.14	43,0,7,0	chr12,72096	Cytosol	A > G
chr12:72096367:A:G:UTR3:TMEM	chr12	72096367	72096367	A	G	UTR3	TMEM19	ENST000002	.	.	A	G	+	+	0.07142857	52,0,4,0	chr12,72096	Cytosol	A > G
chr12:72096370:A:G:UTR3:TMEM	chr12	72096370	72096370	A	G	UTR3	TMEM19	ENST000002	.	.	A	G	+	+	0.09090909	50,0,5,0	chr12,72096	Cytosol	A > G
chr12:72096579:A:G:UTR3:TMEM	chr12	72096579	72096579	A	G	UTR3	TMEM19	ENST000002	.	.	A	G	+	+	0.10416667	43,0,5,0	chr12,72096	Cytosol	A > G
chr12:72096727:A:G:UTR3:TMEM	chr12	72096727	72096727	A	G	UTR3	TMEM19	ENST000002	.	.	A	G	+	+	0.14814815	23,0,4,0	chr12,72096	Cytosol	A > G
chr12:72096740:A:G:UTR3:TMEM	chr12	72096740	72096740	A	G	UTR3	TMEM19	ENST000002	.	.	A	G	+	+	0.31578947	13,0,6,0	chr12,72096	Cytosol	A > G
chr12:72096750:A:G:UTR3:TMEM	chr12	72096750	72096750	A	G	UTR3	TMEM19	ENST000002	.	.	A	G	+	+	0.42857143	12,0,9,0	chr12,72096	Cytosol	A > G
chr12:72096776:A:G:UTR3:TMEM	chr12	72096776	72096776	A	G	UTR3	TMEM19	ENST000002	.	.	A	G	+	+	0.88888889	2,0,16,0	chr12,72096	Cytosol	A > G
chr12:72096782:A:G:UTR3:TMEM	chr12	72096782	72096782	A	G	UTR3	TMEM19	ENST000002	.	.	A	G	+	+	0.2	12,0,3,0	chr12,72096	Cytosol	A > G
chr12:72096784:A:G:UTR3:TMEM	chr12	72096784	72096784	A	G	UTR3	TMEM19	ENST000002	.	.	A	G	+	+	0.35294118	11,0,6,0	chr12,72096	Cytosol	A > G
chr12:72096795:A:G:UTR3:TMEM	chr12	72096795	72096795	A	G	UTR3	TMEM19	ENST000002	.	.	A	G	+	+	0.26666667	11,0,4,0	chr12,72096	Cytosol	A > G
chr12:72096798:A:G:UTR3:TMEM	chr12	72096798	72096798	A	G	UTR3	TMEM19	ENST000002	.	.	A	G	+	+	0.23529412	13,0,4,0	chr12,72096	Cytosol	A > G
chr12:72096836:A:G:UTR3:TMEM	chr12	72096836	72096836	A	G	UTR3	TMEM19	ENST000002	.	.	A	G	+	+	0.26315789	14,0,5,0	chr12,72096	Cytosol	A > G
chr12:72096842:A:G:UTR3:TMEM	chr12	72096842	72096842	A	G	UTR3	TMEM19	ENST000002	.	.	A	G	+	+	0.64705882	6,0,11,0	chr12,72096	Cytosol	A > G
chr12:72096850:A:G:UTR3:TMEM	chr12	72096850	72096850	A	G	UTR3	TMEM19	ENST000002	.	.	A	G	+	+	0.71428571	4,0,10,0	chr12,72096	Cytosol	A > G
chr12:72096863:A:G:UTR3:TMEM	chr12	72096863	72096863	A	G	UTR3	TMEM19	ENST000002	.	.	A	G	+	+	0.18181818	9,0,2,0	chr12,72096	Cytosol	A > G
chr12:75889814:T:C:intronic:GLIP	chr12	75889814	75889814	T	C	intronic	GLIPR1	.	.	.	A	G	-	+	0.2962963	19,0,8,0	chr12,75889	Cytosol	A > G
chr12:75889880:T:C:intronic:GLIP	chr12	75889880	75889880	T	C	intronic	GLIPR1	.	.	.	A	G	-	+	0.61904762	8,0,13,0	chr12,75889	Cytosol	A > G
chr12:75889881:T:C:intronic:GLIP	chr12	75889881	75889881	T	C	intronic	GLIPR1	.	.	.	A	G	-	+	0.95238095	1,0,20,0	chr12,75889	Cytosol	A > G
chr12:75889912:T:C:intronic:GLIP	chr12	75889912	75889912	T	C	intronic	GLIPR1	.	.	.	A	G	-	+	0.45238095	23,0,19,0	chr12,75889	Cytosol	A > G
chr12:75890052:T:C:intronic:GLIP	chr12	75890052	75890052	T	C	intronic	GLIPR1	.	.	.	A	G	-	+	0.89189189	4,0,33,0	chr12,75890	Cytosol	A > G
chr12:75890053:T:C:intronic:GLIP	chr12	75890053	75890053	T	C	intronic	GLIPR1	.	.	.	A	G	-	+	0.30555556	25,0,11,0	chr12,75890	Cytosol	A > G
chr12:75890088:T:C:intronic:GLIP	chr12	75890088	75890088	T	C	intronic	GLIPR1	.	.	.	A	G	-	+	0.17241379	24,0,5,0	chr12,75890	Cytosol	A > G
chr12:75890091:T:C:intronic:GLIP	chr12	75890091	75890091	T	C	intronic	GLIPR1	.	.	.	A	G	-	+	0.31034483	20,0,9,0	chr12,75890	Cytosol	A > G
chr12:75890121:T:C:intronic:GLIP	chr12	75890121	75890121	T	C	intronic	GLIPR1	.	.	.	A	G	-	+	0.37837838	23,0,14,0	chr12,75890	Cytosol	A > G
chr12:93894641:A:G:intronic:MRP	chr12	93894641	93894641	A	G	intronic	MRPL42	.	.	.	A	G	+	+	0.02564103	190,0,5,0	chr12,93894	Cytosol	A > G
chr12:93898060:A:G:downstream:	chr12	93898060	93898060	A	G	downstream	MRPL42	.	.	.	A	G	+		0.18181818	9,0,2,0	chr12,93898	Cytosol	A > G
chr12:93902722:A:G:upstream:RN	chr12	93902722	93902722	A	G	upstream	RN7SL737P	.	.	.	A	G	+		0.875	1,0,7,0	chr12,93902	Cytosol	A > G
chr12:93902773:A:G:upstream:RN	chr12	93902773	93902773	A	G	upstream	RN7SL737P	.	.	.	A	G	+		1	0,0,6,0	chr12,93902	Cytosol	A > G
chr12:93936836:T:C:ncRNA_exon	chr12	93936836	93936836	T	C	ncRNA_exon	SOCS2-AS1	.	.	.	A	G	-	-	0.4	3,0,2,0	chr12,93936	Cytosol	A > G
chr12:98942621:A:G:UTR3:TMPO	chr12	98942621	98942621	A	G	UTR3	TMPO	ENST000005	.	.	A	G	+	+	0.2033195	192,0,49,0	chr12,98942	Cytosol	A > G
chr12:98942643:A:G:UTR3:TMPO	chr12	98942643	98942643	A	G	UTR3	TMPO	ENST000005	.	.	A	G	+	+	0.07407407	225,0,18,0	chr12,98942	Cytosol	A > G
chr12:98942688:A:G:UTR3:TMPO	chr12	98942688	98942688	A	G	UTR3	TMPO	ENST000005	.	.	A	G	+	+	0.92307692	19,0,228,0	chr12,98942	Cytosol	A > G
chr12:98942690:A:G:UTR3:TMPO	chr12	98942690	98942690	A	G	UTR3	TMPO	ENST000005	.	.	A	G	+	+	0.17175573	217,0,45,0	chr12,98942	Cytosol	A > G
chr12:98942695:A:G:UTR3:TMPO	chr12	98942695	98942695	A	G	UTR3	TMPO	ENST000005	.	.	A	G	+	+	0.09747292	250,0,27,0	chr12,98942	Cytosol	A > G
chr12:98942696:A:G:UTR3:TMPO	chr12	98942696	98942696	A	G	UTR3	TMPO	ENST000005	.	.	A	G	+	+	0.4673913	147,0,129,0	chr12,98942	Cytosol	A > G
chr12:98942698:A:G:UTR3:TMPO	chr12	98942698	98942698	A	G	UTR3	TMPO	ENST000005	.	.	A	G	+	+	0.30107527	194,1,84,0	chr12,98942	Cytosol	A > G
chr12:98942805:A:G:UTR3:TMPO	chr12	98942805	98942805	A	G	UTR3	TMPO	ENST000005	.	.	A	G	+	+	0.04513889	275,0,13,0	chr12,98942	Cytosol	A > G
chr12:98942808:A:G:UTR3:TMPO	chr12	98942808	98942808	A	G	UTR3	TMPO	ENST000005	.	.	A	G	+	+	0.02657807	293,0,8,0	chr12,98942	Cytosol	A > G
chr12:98942819:A:G:UTR3:TMPO	chr12	98942819	98942819	A	G	UTR3	TMPO	ENST000005	.	.	A	G	+	+	0.06940063	295,0,22,0	chr12,98942	Cytosol	A > G
chr12:98942820:A:G:UTR3:TMPO	chr12	98942820	98942820	A	G	UTR3	TMPO	ENST000005	.	.	A	G	+	+	0.15755627	262,0,49,0	chr12,98942	Cytosol	A > G
chr12:98942824:A:G:UTR3:TMPO	chr12	98942824	98942824	A	G	UTR3	TMPO	ENST000005	.	.	A	G	+	+	0.48598131	165,0,156,0	chr12,98942	Cytosol	A > G
chr12:98942831:A:G:UTR3:TMPO	chr12	98942831	98942831	A	G	UTR3	TMPO	ENST000005	.	.	A	G	+	+	0.02527076	270,0,7,0	chr12,98942	Cytosol	A > G
chr12:98942847:A:G:UTR3:TMPO	chr12	98942847	98942847	A	G	UTR3	TMPO	ENST000005	.	.	A	G	+	+	0.03065134	253,0,8,0	chr12,98942	Cytosol	A > G
chr12:98942885:A:G:UTR3:TMPO	chr12	98942885	98942885	A	G	UTR3	TMPO	ENST000005	.	.	A	G	+	+	0.69198312	73,0,164,0	chr12,98942	Cytosol	A > G
chr12:98942886:A:G:UTR3:TMPO	chr12	98942886	98942886	A	G	UTR3	TMPO	ENST000005	.	.	A	G	+	+	0.3907563	145,0,93,0	chr12,98942	Cytosol	A > G
chr12:98943011:A:G:UTR3:TMPO	chr12	98943011	98943011	A	G	UTR3	TMPO	ENST000005	.	.	A	G	+	+	0.11538462	183,0,24,1	chr12,98943	Cytosol	A > G
chr12:98943018:A:G:UTR3:TMPO	chr12	98943018	98943018	A	G	UTR3	TMPO	ENST000005	.	.	A	G	+	+	0.01809955	217,0,4,0	chr12,98943	Cytosol	A > G
chr12:98943033:A:G:UTR3:TMPO	chr12	98943033	98943033	A	G	UTR3	TMPO	ENST000005	.	.	A	G	+	+	0.90601504	25,0,241,0	chr12,98943	Cytosol	A > G
chr12:98943083:A:G:UTR3:TMPO	chr12	98943083	98943083	A	G	UTR3	TMPO	ENST000005	.	.	A	G	+	+	0.04935065	366,0,19,0	chr12,98943	Cytosol	A > G
chr12:98943096:A:G:UTR3:TMPO	chr12	98943096	98943096	A	G	UTR3	TMPO	ENST000005	.	.	A	G	+	+	0.45061728	178,0,146,0	chr12,98943	Cytosol	A > G
chr12:98943102:A:G:UTR3:TMPO	chr12	98943102	98943102	A	G	UTR3	TMPO	ENST000005	.	.	A	G	+	+	0.15605096	265,0,49,0	chr12,98943	Cytosol	A > G
chr12:98943103:A:G:UTR3:TMPO	chr12	98943103	98943103	A	G	UTR3	TMPO	ENST000005	.	.	A	G	+	+	0.26315789	224,0,80,0	chr12,98943	Cytosol	A > G
chr12:98943140:A:G:UTR3:TMPO	chr12	98943140	98943140	A	G	UTR3	TMPO	ENST000005	.	.	A	G	+	+	0.01133144	349,0,4,0	chr12,98943	Cytosol	A > G
chr12:98943149:A:G:UTR3:TMPO	chr12	98943149	98943149	A	G	UTR3	TMPO	ENST000005	.	.	A	G	+	+	0.04516129	296,0,14,0	chr12,98943	Cytosol	A > G
chr12:98943182:A:G:UTR3:TMPO	chr12	98943182	98943182	A	G	UTR3	TMPO	ENST000005	.	.	A	G	+	+	0.03571429	270,0,10,0	chr12,98943	Cytosol	A > G

chr12:98943187:A:G:UTR3:TMPO	chr12	98943187	98943187	A	G	UTR3	TMPO	ENST000005	.	.	A	G	+	+	0.12152778	253,0,35,0	chr12,98943	Cytosol	A > G
chr12:98943201:A:G:UTR3:TMPO	chr12	98943201	98943201	A	G	UTR3	TMPO	ENST000005	.	.	A	G	+	+	0.05315615	285,0,16,0	chr12,98943	Cytosol	A > G
chr12:98996017:A:G:downstream:	chr12	98996017	98996017	A	G	downstream	SLC25A3	.	.	.	A	G	+		0.16666667	10,0,2,0	chr12,98996	Cytosol	A > G
chr12:98996023:A:G:downstream:	chr12	98996023	98996023	A	G	downstream	SLC25A3	.	.	.	A	G	+		0.22222222	7,0,2,0	chr12,98996	Cytosol	A > G
chr12:99127337:A:G:UTR3:APAF1	chr12	99127337	99127337	A	G	UTR3	APAF1	ENST000003	.	.	A	G	+	+-	0.04761905	40,0,2,0	chr12,99127	Cytosol	A > G
chr12:99127350:A:G:UTR3:APAF1	chr12	99127350	99127350	A	G	UTR3	APAF1	ENST000003	.	.	A	G	+	+-	0.05	38,0,2,0	chr12,99127	Cytosol	A > G
chr12:99127446:A:G:UTR3:APAF1	chr12	99127446	99127446	A	G	UTR3	APAF1	ENST000003	.	.	A	G	+	-+	0.12727273	48,0,7,0	chr12,99127	Cytosol	A > G
chr12:99127447:A:G:UTR3:APAF1	chr12	99127447	99127447	A	G	UTR3	APAF1	ENST000003	.	.	A	G	+	-+	0.09836066	55,0,6,0	chr12,99127	Cytosol	A > G
chr12:99127474:A:G:UTR3:APAF1	chr12	99127474	99127474	A	G	UTR3	APAF1	ENST000003	.	.	A	G	+	-+	0.08888889	41,0,4,0	chr12,99127	Cytosol	A > G
chr12:99128776:A:G:UTR3:ANKS1	chr12	99128776	99128776	A	G	UTR3	ANKS1B,APA	ENST000003	.	.	A	G	+	+-	0.04166667	46,0,2,0	chr12,99128	Cytosol	A > G
chr12:99128792:A:G:UTR3:ANKS1	chr12	99128792	99128792	A	G	UTR3	ANKS1B,APA	ENST000003	.	.	A	G	+	+-	0.40540541	22,0,15,0	chr12,99128	Cytosol	A > G
chr12:101020744:A:G:intronic:GA	chr12	101020744	101020744	A	G	intronic	GAS2L3	.	.	.	A	G	+	+	1	0,0,6,0	chr12,10102	Cytosol	A > G
chr12:101020746:A:G:intronic:GA	chr12	101020746	101020746	A	G	intronic	GAS2L3	.	.	.	A	G	+	+	0.57142857	3,0,4,0	chr12,10102	Cytosol	A > G
chr12:101021192:A:G:intronic:GA	chr12	101021192	101021192	A	G	intronic	GAS2L3	.	.	.	A	G	+	+	0.66666667	3,0,6,0	chr12,10102	Cytosol	A > G
chr12:109178972:T:C:UTR3:SSH1	chr12	109178972	109178972	T	C	UTR3	SSH1	ENST000003	.	.	A	G	-	-	0.10714286	25,0,3,0	chr12,10917	Cytosol	A > G
chr12:109180419:T:C:UTR3:SSH1	chr12	109180419	109180419	T	C	UTR3	SSH1	ENST000003	.	.	A	G	-	-	0.05882353	32,0,2,0	chr12,10918	Cytosol	A > G
chr12:110289484:T:C:UTR3:GLTP	chr12	110289484	110289484	T	C	UTR3	GLTP	ENST000003	.	.	A	G	-	-	0.03614458	80,0,3,0	chr12,11028	Cytosol	A > G
chr12:110289557:T:C:UTR3:GLTP	chr12	110289557	110289557	T	C	UTR3	GLTP	ENST000003	.	.	A	G	-	-	0.02040816	96,0,2,0	chr12,11028	Cytosol	A > G
chr12:110880327:T:C:ncRNA_intro	chr12	110880327	110880327	T	C	ncRNA_intro	RP11-478C1	.	.	.	A	G	-	-	0.4	3,0,2,0	chr12,11088	Cytosol	A > G
chr12:112278192:T:C:ncRNA_exor	chr12	112278192	112278192	T	C	ncRNA_exon	MAPKAPK5-A	.	.	.	A	G	-	-	0.05357143	53,0,3,0	chr12,11227	Cytosol	A > G
chr12:117152985:T:C:downstrean	chr12	117152985	117152985	T	C	downstream	C12orf49	.	.	.	A	G	-	-	0.03636364	53,0,2,0	chr12,11715	Cytosol	A > G
chr12:117288621:A:G:UTR3:RNFT	chr12	117288621	117288621	A	G	UTR3	RNFT2	ENST000002	.	.	A	G	+	+	0.5	3,0,3,0	chr12,11728	Cytosol	A > G
chr12:117289106:A:G:UTR3:RNFT	chr12	117289106	117289106	A	G	UTR3	RNFT2	ENST000002	.	.	A	G	+	+	0.28571429	5,0,2,0	chr12,11728	Cytosol	A > G
chr12:117289288:A:G:UTR3:RNFT	chr12	117289288	117289288	A	G	UTR3	RNFT2	ENST000002	.	.	A	G	+	+	0.875	1,0,7,0	chr12,11728	Cytosol	A > G
chr12:117289312:A:G:UTR3:RNFT	chr12	117289312	117289312	A	G	UTR3	RNFT2	ENST000002	.	.	A	G	+	+	1	0,0,7,0	chr12,11728	Cytosol	A > G
chr12:117289315:A:G:UTR3:RNFT	chr12	117289315	117289315	A	G	UTR3	RNFT2	ENST000002	.	.	A	G	+	+	0.5	3,0,3,0	chr12,11728	Cytosol	A > G
chr12:117289379:A:G:UTR3:RNFT	chr12	117289379	117289379	A	G	UTR3	RNFT2	ENST000002	.	.	A	G	+	+	0.33333333	4,0,2,0	chr12,11728	Cytosol	A > G
chr12:117289398:A:G:UTR3:RNFT	chr12	117289398	117289398	A	G	UTR3	RNFT2	ENST000002	.	.	A	G	+	+	0.28571429	5,0,2,0	chr12,11728	Cytosol	A > G
chr12:117289424:A:G:UTR3:RNFT	chr12	117289424	117289424	A	G	UTR3	RNFT2	ENST000002	.	.	A	G	+	+	0.33333333	4,0,2,0	chr12,11728	Cytosol	A > G
chr12:117289438:A:G:UTR3:RNFT	chr12	117289438	117289438	A	G	UTR3	RNFT2	ENST000002	.	.	A	G	+	+	1	0,0,5,0	chr12,11728	Cytosol	A > G
chr12:117289446:A:G:UTR3:RNFT	chr12	117289446	117289446	A	G	UTR3	RNFT2	ENST000002	.	.	A	G	+	+	0.4	3,0,2,0	chr12,11728	Cytosol	A > G
chr12:117467471:A:G:UTR3:FBXW	chr12	117467471	117467471	A	G	UTR3	FBXW8	ENST000004	.	.	A	G	+	+	0.72413793	8,0,21,0	chr12,11746	Cytosol	A > G
chr12:117467518:A:G:UTR3:FBXW	chr12	117467518	117467518	A	G	UTR3	FBXW8	ENST000004	.	.	A	G	+	+	0.38461538	8,0,5,0	chr12,11746	Cytosol	A > G
chr12:117467544:A:G:UTR3:FBXW	chr12	117467544	117467544	A	G	UTR3	FBXW8	ENST000004	.	.	A	G	+	+	0.15384615	11,0,2,0	chr12,11746	Cytosol	A > G
chr12:117467554:A:G:UTR3:FBXW	chr12	117467554	117467554	A	G	UTR3	FBXW8	ENST000004	.	.	A	G	+	+	0.2	8,0,2,0	chr12,11746	Cytosol	A > G
chr12:117467580:A:G:UTR3:FBXW	chr12	117467580	117467580	A	G	UTR3	FBXW8	ENST000004	.	.	A	G	+	+	0.36363636	7,0,4,0	chr12,11746	Cytosol	A > G
chr12:117467598:A:G:UTR3:FBXW	chr12	117467598	117467598	A	G	UTR3	FBXW8	ENST000004	.	.	A	G	+	+	0.38461538	8,0,5,0	chr12,11746	Cytosol	A > G
chr12:117467652:A:G:UTR3:FBXW	chr12	117467652	117467652	A	G	UTR3	FBXW8	ENST000004	.	.	A	G	+	+	0.5	4,0,4,0	chr12,11746	Cytosol	A > G
chr12:117467653:A:G:UTR3:FBXW	chr12	117467653	117467653	A	G	UTR3	FBXW8	ENST000004	.	.	A	G	+	+	0.45454545	6,0,5,0	chr12,11746	Cytosol	A > G
chr12:117467668:A:G:UTR3:FBXW	chr12	117467668	117467668	A	G	UTR3	FBXW8	ENST000004	.	.	A	G	+	+	0.5	4,0,4,0	chr12,11746	Cytosol	A > G
chr12:117469263:A:G:downstrean	chr12	117469263	117469263	A	G	downstream	FBXW8	.	.	.	A	G	+		0.1	18,0,2,0	chr12,11746	Cytosol	A > G
chr12:117469264:A:G:downstrean	chr12	117469264	117469264	A	G	downstream	FBXW8	.	.	.	A	G	+		0.15	17,0,3,0	chr12,11746	Cytosol	A > G
chr12:117469309:A:G:downstrean	chr12	117469309	117469309	A	G	downstream	FBXW8	.	.	.	A	G	+		0.63157895	7,0,12,0	chr12,11746	Cytosol	A > G
chr12:117469348:A:G:downstrean	chr12	117469348	117469348	A	G	downstream	FBXW8	.	.	.	A	G	+		0.25	9,0,3,0	chr12,11746	Cytosol	A > G
chr12:117469368:A:G:downstrean	chr12	117469368	117469368	A	G	downstream	FBXW8	.	.	.	A	G	+		0.22222222	7,0,2,0	chr12,11746	Cytosol	A > G
chr12:117469372:A:G:downstrean	chr12	117469372	117469372	A	G	downstream	FBXW8	.	.	.	A	G	+		0.33333333	4,0,2,0	chr12,11746	Cytosol	A > G
chr12:117469405:A:G:downstrean	chr12	117469405	117469405	A	G	downstream	FBXW8	.	.	.	A	G	+		0.4	6,0,4,0	chr12,11746	Cytosol	A > G
chr12:117469408:A:G:downstrean	chr12	117469408	117469408	A	G	downstream	FBXW8	.	.	.	A	G	+		0.77777778	2,0,7,0	chr12,11746	Cytosol	A > G
chr12:118554410:T:C:intergenic:V	chr12	118554410	118554410	T	C	intergenic	VSIG10,PEBP	dist=12657;d	.	.	A	G	-	-	0.6	2,0,3,0	chr12,11855	Cytosol	A > G
chr12:118575323:A:G:intronic:PEE	chr12	118575323	118575323	A	G	intronic	PEBP1	.	.	.	A	G	+	+	0.6	2,0,3,0	chr12,11857	Cytosol	A > G
chr12:120898553:A:G:UTR3:GATC	chr12	120898553	120898553	A	G	UTR3	GATC	ENST000005	.	.	A	G	+	+	0.52678571	53,0,59,0	chr12,12089	Cytosol	A > G
chr12:120898554:A:G:UTR3:GATC	chr12	120898554	120898554	A	G	UTR3	GATC	ENST000005	.	.	A	G	+	+	0.03571429	108,0,4,0	chr12,12089	Cytosol	A > G
chr12:120898631:A:G:UTR3:GATC	chr12	120898631	120898631	A	G	UTR3	GATC	ENST000005	.	.	A	G	+	+	0.02970297	98,0,3,0	chr12,12089	Cytosol	A > G
chr12:120898645:A:G:UTR3:GATC	chr12	120898645	120898645	A	G	UTR3	GATC	ENST000005	.	.	A	G	+	+	0.04132231	116,0,5,0	chr12,12089	Cytosol	A > G
chr12:120898656:A:G:UTR3:GATC	chr12	120898656	120898656	A	G	UTR3	GATC	ENST000005	.	.	A	G	+	+	0.01612903	122,0,2,0	chr12,12089	Cytosol	A > G
chr12:120898657:A:G:UTR3:GATC	chr12	120898657	120898657	A	G	UTR3	GATC	ENST000005	.	.	A	G	+	+	0.03007519	129,0,4,0	chr12,12089	Cytosol	A > G
chr12:120898658:A:G:UTR3:GATC	chr12	120898658	120898658	A	G	UTR3	GATC	ENST000005	.	.	A	G	+	+	0.03816794	126,0,5,0	chr12,12089	Cytosol	A > G
chr12:120898661:A:G:UTR3:GATC	chr12	120898661	120898661	A	G	UTR3	GATC	ENST000005	.	.	A	G	+	+	0.14285714	108,0,18,0	chr12,12089	Cytosol	A > G
chr12:120898687:A:G:UTR3:GATC	chr12	120898687	120898687	A	G	UTR3	GATC	ENST000005	.	.	A	G	+	+	0.05147059	129,0,7,0	chr12,12089	Cytosol	A > G
chr12:120898697:A:G:UTR3:GATC	chr12	120898697	120898697	A	G	UTR3	GATC	ENST000005	.	.	A	G	+	+	0.28275862	104,0,41,0	chr12,12089	Cytosol	A > G
chr12:120898714:A:G:UTR3:GATC	chr12	120898714	120898714	A	G	UTR3	GATC	ENST000005	.	.	A	G	+	+	0.94308943	7,0,116,0	chr12,12089	Cytosol	A > G
chr12:120898943:A:G:UTR3:GATC	chr12	120898943	120898943	A	G	UTR3	GATC	ENST000005	.	.	A	G	+	+	0.28658537	117,0,47,0	chr12,12089	Cytosol	A > G
chr12:120898978:A:G:UTR3:GATC	chr12	120898978	120898978	A	G	UTR3	GATC	ENST000005	.	.	A	G	+	+	0.12359551	156,0,22,0	chr12,12089	Cytosol	A > G
chr12:120898998:A:G:UTR3:GATC	chr12	120898998	120898998	A	G	UTR3	GATC	ENST000005	.	.	A	G	+	+	0.17142857	174,0,36,0	chr12,12089	Cytosol	A > G

chr12:120898999:A:G:UTR3:GATC	chr12	120898999	120898999	A	G	UTR3	GATC	ENST000005	.	.	A	G	+	+	0.25728155	153,0,53,0	chr12,12089	Cytosol	A > G
chr12:120899011:A:G:UTR3:GATC	chr12	120899011	120899011	A	G	UTR3	GATC	ENST000005	.	.	A	G	+	+	0.92571429	13,0,162,0	chr12,12089	Cytosol	A > G
chr12:120899029:A:G:UTR3:GATC	chr12	120899029	120899029	A	G	UTR3	GATC	ENST000005	.	.	A	G	+	+	0.04069767	165,0,7,0	chr12,12089	Cytosol	A > G
chr12:120899064:A:G:UTR3:GATC	chr12	120899064	120899064	A	G	UTR3	GATC	ENST000005	.	.	A	G	+	+	0.07352941	189,0,15,0	chr12,12089	Cytosol	A > G
chr12:120899075:A:G:UTR3:GATC	chr12	120899075	120899075	A	G	UTR3	GATC	ENST000005	.	.	A	G	+	+	0.736	65,0,184,1	chr12,12089	Cytosol	A > G
chr12:120899076:A:G:UTR3:GATC	chr12	120899076	120899076	A	G	UTR3	GATC	ENST000005	.	.	A	G	+	+	0.0204918	238,0,5,1	chr12,12089	Cytosol	A > G
chr12:120899079:A:G:UTR3:GATC	chr12	120899079	120899079	A	G	UTR3	GATC	ENST000005	.	.	A	G	+	+	0.01834862	214,0,4,0	chr12,12089	Cytosol	A > G
chr12:120899161:A:G:UTR3:GATC	chr12	120899161	120899161	A	G	UTR3	GATC	ENST000005	.	.	A	G	+	+	0.02352941	249,0,6,0	chr12,12089	Cytosol	A > G
chr12:120899168:A:G:UTR3:GATC	chr12	120899168	120899168	A	G	UTR3	GATC	ENST000005	.	.	A	G	+	+	0.04198473	251,0,11,0	chr12,12089	Cytosol	A > G
chr12:120926470:T:C:intronic:DYN	chr12	120926470	120926470	T	C	intronic	DYNLL1	.	.	.	A	G	-	+	0.11111111	16,0,2,0	chr12,12092	Cytosol	A > G
chr12:120926489:T:C:intronic:DYN	chr12	120926489	120926489	T	C	intronic	DYNLL1	.	.	.	A	G	-	+	0.60869565	9,0,14,0	chr12,12092	Cytosol	A > G
chr12:120926498:T:C:intronic:DYN	chr12	120926498	120926498	T	C	intronic	DYNLL1	.	.	.	A	G	-	+	0.25	15,0,5,0	chr12,12092	Cytosol	A > G
chr12:120926532:T:C:intronic:DYN	chr12	120926532	120926532	T	C	intronic	DYNLL1	.	.	.	A	G	-	+	0.36842105	12,0,7,0	chr12,12092	Cytosol	A > G
chr12:120926552:T:C:intronic:DYN	chr12	120926552	120926552	T	C	intronic	DYNLL1	.	.	.	A	G	-	+	0.16666667	15,0,3,0	chr12,12092	Cytosol	A > G
chr12:120926604:T:C:intronic:DYN	chr12	120926604	120926604	T	C	intronic	DYNLL1	.	.	.	A	G	-	+	0.11111111	24,0,3,0	chr12,12092	Cytosol	A > G
chr12:120926634:T:C:intronic:DYN	chr12	120926634	120926634	T	C	intronic	DYNLL1	.	.	.	A	G	-	+	0.10526316	17,0,2,0	chr12,12092	Cytosol	A > G
chr12:120926653:T:C:intronic:DYN	chr12	120926653	120926653	T	C	intronic	DYNLL1	.	.	.	A	G	-	+	0.33333333	8,0,4,0	chr12,12092	Cytosol	A > G
chr12:120926656:T:C:intronic:DYN	chr12	120926656	120926656	T	C	intronic	DYNLL1	.	.	.	A	G	-	+	0.46666667	8,0,7,0	chr12,12092	Cytosol	A > G
chr12:120926672:T:C:intronic:DYN	chr12	120926672	120926672	T	C	intronic	DYNLL1	.	.	.	A	G	-	+	0.16666667	10,0,2,0	chr12,12092	Cytosol	A > G
chr12:120926745:T:C:intronic:DYN	chr12	120926745	120926745	T	C	intronic	DYNLL1	.	.	.	A	G	-	+	0.29411765	12,0,5,0	chr12,12092	Cytosol	A > G
chr12:120927627:T:C:intronic:DYN	chr12	120927627	120927627	T	C	intronic	DYNLL1	.	.	.	A	G	-	+	0.23076923	10,0,3,0	chr12,12092	Cytosol	A > G
chr12:120927636:T:C:intronic:DYN	chr12	120927636	120927636	T	C	intronic	DYNLL1	.	.	.	A	G	-	+	0.53333333	7,0,8,0	chr12,12092	Cytosol	A > G
chr12:120927647:T:C:intronic:DYN	chr12	120927647	120927647	T	C	intronic	DYNLL1	.	.	.	A	G	-	+	0.33333333	12,0,6,0	chr12,12092	Cytosol	A > G
chr12:120927648:T:C:intronic:DYN	chr12	120927648	120927648	T	C	intronic	DYNLL1	.	.	.	A	G	-	+	0.47058824	9,0,8,0	chr12,12092	Cytosol	A > G
chr12:120927689:T:C:intronic:DYN	chr12	120927689	120927689	T	C	intronic	DYNLL1	.	.	.	A	G	-	+	0.28571429	5,0,2,0	chr12,12092	Cytosol	A > G
chr12:120927778:T:C:intronic:DYN	chr12	120927778	120927778	T	C	intronic	DYNLL1	.	.	.	A	G	-	+	0.82352941	3,0,14,0	chr12,12092	Cytosol	A > G
chr12:120927793:T:C:intronic:DYN	chr12	120927793	120927793	T	C	intronic	DYNLL1	.	.	.	A	G	-	+	0.83333333	3,0,15,0	chr12,12092	Cytosol	A > G
chr12:121862333:A:G:intronic:RN	chr12	121862333	121862333	A	G	intronic	RNF34	.	.	.	A	G	+	+	0.5	5,0,5,0	chr12,12186	Cytosol	A > G
chr12:121862334:A:G:intronic:RN	chr12	121862334	121862334	A	G	intronic	RNF34	.	.	.	A	G	+	+	0.5	5,0,5,0	chr12,12186	Cytosol	A > G
chr12:121865099:A:G:intronic:RN	chr12	121865099	121865099	A	G	intronic	RNF34	.	.	.	A	G	+	+	0.8	1,0,4,0	chr12,12186	Cytosol	A > G
chr12:121865174:A:G:intronic:RN	chr12	121865174	121865174	A	G	intronic	RNF34	.	.	.	A	G	+	+	0.28571429	5,0,2,0	chr12,12186	Cytosol	A > G
chr12:122214833:A:G:UTR3:TME	chr12	122214833	122214833	A	G	UTR3	TMEM120B	ENST000004	.	.	A	G	+	+	0.09677419	28,0,3,0	chr12,12221	Cytosol	A > G
chr12:122214955:A:G:UTR3:TME	chr12	122214955	122214955	A	G	UTR3	TMEM120B	ENST000004	.	.	A	G	+	+	0.3	14,0,6,0	chr12,12221	Cytosol	A > G
chr12:122214962:A:G:UTR3:TME	chr12	122214962	122214962	A	G	UTR3	TMEM120B	ENST000004	.	.	A	G	+	+	0.125	21,0,3,0	chr12,12221	Cytosol	A > G
chr12:122214991:A:G:UTR3:TME	chr12	122214991	122214991	A	G	UTR3	TMEM120B	ENST000004	.	.	A	G	+	+	0.11111111	16,0,2,0	chr12,12221	Cytosol	A > G
chr12:122214992:A:G:UTR3:TME	chr12	122214992	122214992	A	G	UTR3	TMEM120B	ENST000004	.	.	A	G	+	+	0.25	9,0,3,0	chr12,12221	Cytosol	A > G
chr12:122215001:A:G:UTR3:TME	chr12	122215001	122215001	A	G	UTR3	TMEM120B	ENST000004	.	.	A	G	+	+	0.07142857	26,0,2,0	chr12,12221	Cytosol	A > G
chr12:122215092:A:G:UTR3:TME	chr12	122215092	122215092	A	G	UTR3	TMEM120B	ENST000004	.	.	A	G	+	+	0.30434783	16,0,7,0	chr12,12221	Cytosol	A > G
chr12:122215150:A:G:UTR3:TME	chr12	122215150	122215150	A	G	UTR3	TMEM120B	ENST000004	.	.	A	G	+	+	0.08	23,0,2,0	chr12,12221	Cytosol	A > G
chr12:122215204:A:G:UTR3:TME	chr12	122215204	122215204	A	G	UTR3	TMEM120B	ENST000004	.	.	A	G	+	+	0.08	23,0,2,0	chr12,12221	Cytosol	A > G
chr12:122218225:A:G:UTR3:TME	chr12	122218225	122218225	A	G	UTR3	TMEM120B	ENST000004	.	.	A	G	+	+-	0.22222222	7,0,2,0	chr12,12221	Cytosol	A > G
chr12:122218226:A:G:UTR3:TME	chr12	122218226	122218226	A	G	UTR3	TMEM120B	ENST000004	.	.	A	G	+	+-	0.625	3,0,5,0	chr12,12221	Cytosol	A > G
chr12:123896983:T:C:intergenic:S	chr12	123896983	123896983	T	C	intergenic	SETD8,RILPL	dist=3078;dis	.	.	A	G	-		0.33333333	6,0,3,0	chr12,12389	Cytosol	A > G
chr12:123943586:A:G:intronic:SN	chr12	123943586	123943586	A	G	intronic	SNRNP35	.	.	.	A	G	+	+	0.57142857	3,0,4,0	chr12,12394	Cytosol	A > G
chr12:124002196:T:C:intronic:RIL	chr12	124002196	124002196	T	C	intronic	RILPL1	.	.	.	A	G	-	-	0.875	1,0,7,0	chr12,12400	Cytosol	A > G
chr12:124005030:T:C:intronic:RIL	chr12	124005030	124005030	T	C	intronic	RILPL1	.	.	.	A	G	-	-	1	0,0,5,0	chr12,12400	Cytosol	A > G
chr12:124146067:A:G:UTR3:GTF2	chr12	124146067	124146067	A	G	UTR3	GTF2H3	ENST000005	.	.	A	G	+	+	0.07017544	106,0,8,0	chr12,12414	Cytosol	A > G
chr12:124146077:A:G:UTR3:GTF2	chr12	124146077	124146077	A	G	UTR3	GTF2H3	ENST000005	.	.	A	G	+	+	0.17475728	85,0,18,0	chr12,12414	Cytosol	A > G
chr12:124146095:A:G:UTR3:GTF2	chr12	124146095	124146095	A	G	UTR3	GTF2H3	ENST000005	.	.	A	G	+	+	0.61428571	27,0,43,0	chr12,12414	Cytosol	A > G
chr12:124146315:A:G:UTR3:GTF2	chr12	124146315	124146315	A	G	UTR3	GTF2H3	ENST000005	.	.	A	G	+	+	0.0308642	157,0,5,0	chr12,12414	Cytosol	A > G
chr12:124146396:A:G:UTR3:GTF2	chr12	124146396	124146396	A	G	UTR3	GTF2H3	ENST000005	.	.	A	G	+	+	0.07361963	151,0,12,0	chr12,12414	Cytosol	A > G
chr12:125511226:A:G:UTR3:BRI3B	chr12	125511226	125511226	A	G	UTR3	BRI3BP	ENST000003	.	.	A	G	+	-+	0.19753086	65,0,16,0	chr12,12551	Cytosol	A > G
chr12:125511242:A:G:UTR3:BRI3B	chr12	125511242	125511242	A	G	UTR3	BRI3BP	ENST000003	.	.	A	G	+	-+	0.08045977	80,0,7,0	chr12,12551	Cytosol	A > G
chr12:125511311:A:G:UTR3:BRI3B	chr12	125511311	125511311	A	G	UTR3	BRI3BP	ENST000003	.	.	A	G	+	-+	0.05434783	87,0,5,0	chr12,12551	Cytosol	A > G
chr12:125511348:A:G:UTR3:BRI3B	chr12	125511348	125511348	A	G	UTR3	BRI3BP	ENST000003	.	.	A	G	+	-+	0.07142857	104,0,8,0	chr12,12551	Cytosol	A > G
chr12:125511958:A:G:UTR3:BRI3B	chr12	125511958	125511958	A	G	UTR3	BRI3BP	ENST000003	.	.	A	G	+	+-	0.78333333	13,0,47,0	chr12,12551	Cytosol	A > G
chr12:125511960:A:G:UTR3:BRI3B	chr12	125511960	125511960	A	G	UTR3	BRI3BP	ENST000003	.	.	A	G	+	+-	0.50909091	27,0,28,0	chr12,12551	Cytosol	A > G
chr12:125511967:A:G:UTR3:BRI3B	chr12	125511967	125511967	A	G	UTR3	BRI3BP	ENST000003	.	.	A	G	+	+-	0.60869565	18,0,28,0	chr12,12551	Cytosol	A > G
chr12:125512029:A:G:UTR3:BRI3B	chr12	125512029	125512029	A	G	UTR3	BRI3BP	ENST000003	.	.	A	G	+	+	0.75	15,0,45,0	chr12,12551	Cytosol	A > G
chr12:125512030:A:G:UTR3:BRI3B	chr12	125512030	125512030	A	G	UTR3	BRI3BP	ENST000003	.	.	A	G	+	+	0.65625	22,0,42,0	chr12,12551	Cytosol	A > G
chr12:125512032:A:G:UTR3:BRI3B	chr12	125512032	125512032	A	G	UTR3	BRI3BP	ENST000003	.	.	A	G	+	+	0.46666667	32,0,28,0	chr12,12551	Cytosol	A > G
chr12:125512063:A:G:UTR3:BRI3B	chr12	125512063	125512063	A	G	UTR3	BRI3BP	ENST000003	.	.	A	G	+	+	0.28846154	37,0,15,0	chr12,12551	Cytosol	A > G
chr12:125512069:A:G:UTR3:BRI3B	chr12	125512069	125512069	A	G	UTR3	BRI3BP	ENST000003	.	.	A	G	+	+	0.65853659	14,0,27,0	chr12,12551	Cytosol	A > G
chr12:125512073:A:G:UTR3:BRI3B	chr12	125512073	125512073	A	G	UTR3	BRI3BP	ENST000003	.	.	A	G	+	+	0.97435897	1,0,38,0	chr12,12551	Cytosol	A > G

chr12:125512096:A:G:UTR3:BRI3B	chr12	125512096	125512096	A	G	UTR3	BRI3BP	ENST000003	.	.	A	G	+	+	0.09589041	66,0,7,0	chr12,12551	Cytosol	A > G
chr12:125512101:A:G:UTR3:BRI3B	chr12	125512101	125512101	A	G	UTR3	BRI3BP	ENST000003	.	.	A	G	+	+	0.65333333	26,0,49,0	chr12,12551	Cytosol	A > G
chr12:125512128:A:G:UTR3:BRI3B	chr12	125512128	125512128	A	G	UTR3	BRI3BP	ENST000003	.	.	A	G	+	+	0.18085106	77,0,17,0	chr12,12551	Cytosol	A > G
chr12:125512173:A:G:UTR3:BRI3B	chr12	125512173	125512173	A	G	UTR3	BRI3BP	ENST000003	.	.	A	G	+	+	0.41935484	54,0,39,0	chr12,12551	Cytosol	A > G
chr12:125512179:A:G:UTR3:BRI3B	chr12	125512179	125512179	A	G	UTR3	BRI3BP	ENST000003	.	.	A	G	+	+	0.07865169	82,0,7,0	chr12,12551	Cytosol	A > G
chr12:125512217:A:G:UTR3:BRI3B	chr12	125512217	125512217	A	G	UTR3	BRI3BP	ENST000003	.	.	A	G	+	+	0.05434783	87,0,5,0	chr12,12551	Cytosol	A > G
chr12:125512218:A:G:UTR3:BRI3B	chr12	125512218	125512218	A	G	UTR3	BRI3BP	ENST000003	.	.	A	G	+	+	0.09195402	79,0,8,0	chr12,12551	Cytosol	A > G
chr12:125512289:A:G:UTR3:BRI3B	chr12	125512289	125512289	A	G	UTR3	BRI3BP	ENST000003	.	.	A	G	+	+	0.06944444	67,0,5,0	chr12,12551	Cytosol	A > G
chr12:125512295:A:G:UTR3:BRI3B	chr12	125512295	125512295	A	G	UTR3	BRI3BP	ENST000003	.	.	A	G	+	+	0.26666667	44,0,16,0	chr12,12551	Cytosol	A > G
chr12:125512298:A:G:UTR3:BRI3B	chr12	125512298	125512298	A	G	UTR3	BRI3BP	ENST000003	.	.	A	G	+	+	0.05970149	63,0,4,0	chr12,12551	Cytosol	A > G
chr12:125512314:A:G:UTR3:BRI3B	chr12	125512314	125512314	A	G	UTR3	BRI3BP	ENST000003	.	.	A	G	+	+	0.13043478	60,0,9,0	chr12,12551	Cytosol	A > G
chr12:125512360:A:G:UTR3:BRI3B	chr12	125512360	125512360	A	G	UTR3	BRI3BP	ENST000003	.	.	A	G	+	+	0.59649123	23,0,34,0	chr12,12551	Cytosol	A > G
chr12:125512363:A:G:UTR3:BRI3B	chr12	125512363	125512363	A	G	UTR3	BRI3BP	ENST000003	.	.	A	G	+	+	0.85964912	8,0,49,0	chr12,12551	Cytosol	A > G
chr12:125512375:A:G:UTR3:BRI3B	chr12	125512375	125512375	A	G	UTR3	BRI3BP	ENST000003	.	.	A	G	+	+	0.17241379	48,0,10,0	chr12,12551	Cytosol	A > G
chr12:125512399:A:G:UTR3:BRI3B	chr12	125512399	125512399	A	G	UTR3	BRI3BP	ENST000003	.	.	A	G	+	+	0.61111111	14,0,22,0	chr12,12551	Cytosol	A > G
chr12:125512400:A:G:UTR3:BRI3B	chr12	125512400	125512400	A	G	UTR3	BRI3BP	ENST000003	.	.	A	G	+	+	0.66666667	13,0,26,0	chr12,12551	Cytosol	A > G
chr12:125512407:A:G:UTR3:BRI3B	chr12	125512407	125512407	A	G	UTR3	BRI3BP	ENST000003	.	.	A	G	+	+	0.95121951	2,0,39,0	chr12,12551	Cytosol	A > G
chr12:125512440:A:G:UTR3:BRI3B	chr12	125512440	125512440	A	G	UTR3	BRI3BP	ENST000003	.	.	A	G	+	+	0.96	1,0,48,1	chr12,12551	Cytosol	A > G
chr12:125512494:A:G:UTR3:BRI3B	chr12	125512494	125512494	A	G	UTR3	BRI3BP	ENST000003	.	.	A	G	+	+	0.42727273	63,0,47,0	chr12,12551	Cytosol	A > G
chr12:125512495:A:G:UTR3:BRI3B	chr12	125512495	125512495	A	G	UTR3	BRI3BP	ENST000003	.	.	A	G	+	+	0.73394495	29,0,80,0	chr12,12551	Cytosol	A > G
chr12:125512507:A:G:UTR3:BRI3B	chr12	125512507	125512507	A	G	UTR3	BRI3BP	ENST000003	.	.	A	G	+	+	0.51612903	75,0,80,0	chr12,12551	Cytosol	A > G
chr12:125512509:A:G:UTR3:BRI3B	chr12	125512509	125512509	A	G	UTR3	BRI3BP	ENST000003	.	.	A	G	+	+	0.19631902	131,0,32,0	chr12,12551	Cytosol	A > G
chr12:125512516:A:G:UTR3:BRI3B	chr12	125512516	125512516	A	G	UTR3	BRI3BP	ENST000003	.	.	A	G	+	+	0.05442177	139,0,8,0	chr12,12551	Cytosol	A > G
chr12:125513957:A:G:UTR3:BRI3B	chr12	125513957	125513957	A	G	UTR3	BRI3BP	ENST000003	.	.	A	G	+	+	0.03731343	129,0,5,0	chr12,12551	Cytosol	A > G
chr12:125513971:A:G:UTR3:BRI3B	chr12	125513971	125513971	A	G	UTR3	BRI3BP	ENST000003	.	.	A	G	+	+	0.02484472	157,0,4,0	chr12,12551	Cytosol	A > G
chr12:133297904:A:G:UTR3:PGAN	chr12	133297904	133297904	A	G	UTR3	PGAM5	ENST000004	.	.	A	G	+	+	0.39087948	187,0,120,0	chr12,13329	Cytosol	A > G
chr12:133297956:A:G:UTR3:PGAN	chr12	133297956	133297956	A	G	UTR3	PGAM5	ENST000004	.	.	A	G	+	+	0.22929936	241,0,72,1	chr12,13329	Cytosol	A > G
chr12:133297974:A:G:UTR3:PGAN	chr12	133297974	133297974	A	G	UTR3	PGAM5	ENST000004	.	.	A	G	+	+	0.15503876	327,0,60,0	chr12,13329	Cytosol	A > G
chr12:133298019:A:G:UTR3:PGAN	chr12	133298019	133298019	A	G	UTR3	PGAM5	ENST000004	.	.	A	G	+	+	0.0878553	353,0,34,0	chr12,13329	Cytosol	A > G
chr12:133298095:A:G:UTR3:PGAN	chr12	133298095	133298095	A	G	UTR3	PGAM5	ENST000004	.	.	A	G	+	+	0.24034335	177,0,56,0	chr12,13329	Cytosol	A > G
chr12:133298339:A:G:UTR3:PGAN	chr12	133298339	133298339	A	G	UTR3	PGAM5	ENST000004	.	.	A	G	+	+	0.55345912	142,0,176,0	chr12,13329	Cytosol	A > G
chr12:133298349:A:G:UTR3:PGAN	chr12	133298349	133298349	A	G	UTR3	PGAM5	ENST000004	.	.	A	G	+	+	0.28869048	239,0,97,0	chr12,13329	Cytosol	A > G
chr12:133298417:A:G:UTR3:PGAN	chr12	133298417	133298417	A	G	UTR3	PGAM5	ENST000004	.	.	A	G	+	+	0.14521452	259,0,44,0	chr12,13329	Cytosol	A > G
chr12:133299058:A:G:UTR3:PGAN	chr12	133299058	133299058	A	G	UTR3	PGAM5	ENST000004	.	.	A	G	+	+	0.31168831	106,0,48,0	chr12,13329	Cytosol	A > G
chr12:133346564:T:C:UTR3:GOLG	chr12	133346564	133346564	T	C	UTR3	GOLGA3	ENST000002	.	.	A	G	-	-	0.2173913	36,0,10,0	chr12,13334	Cytosol	A > G
chr12:133346586:T:C:UTR3:GOLG	chr12	133346586	133346586	T	C	UTR3	GOLGA3	ENST000002	.	.	A	G	-	-	0.27777778	52,0,20,0	chr12,13334	Cytosol	A > G
chr12:133346684:T:C:UTR3:GOLG	chr12	133346684	133346684	T	C	UTR3	GOLGA3	ENST000002	.	.	A	G	-	-	0.09090909	30,0,3,0	chr12,13334	Cytosol	A > G
chr12:133346707:T:C:UTR3:GOLG	chr12	133346707	133346707	T	C	UTR3	GOLGA3	ENST000002	.	.	A	G	-	-	0.11764706	30,0,4,0	chr12,13334	Cytosol	A > G
chr12:133346736:T:C:UTR3:GOLG	chr12	133346736	133346736	T	C	UTR3	GOLGA3	ENST000002	.	.	A	G	-	-	0.1	27,0,3,0	chr12,13334	Cytosol	A > G
chr12:133346813:T:C:UTR3:GOLG	chr12	133346813	133346813	T	C	UTR3	GOLGA3	ENST000002	.	.	A	G	-	-	0.25	15,0,5,0	chr12,13334	Cytosol	A > G
chr12:133347372:T:C:UTR3:GOLG	chr12	133347372	133347372	T	C	UTR3	GOLGA3	ENST000002	.	.	A	G	-	-	1	0,0,7,0	chr12,13334	Cytosol	A > G
chr12:133347447:T:C:UTR3:GOLG	chr12	133347447	133347447	T	C	UTR3	GOLGA3	ENST000002	.	.	A	G	-	-	0.16129032	26,0,5,0	chr12,13334	Cytosol	A > G
chr12:133347494:T:C:UTR3:GOLG	chr12	133347494	133347494	T	C	UTR3	GOLGA3	ENST000002	.	.	A	G	-	-	0.13333333	13,0,2,0	chr12,13334	Cytosol	A > G
chr12:133347502:T:C:UTR3:GOLG	chr12	133347502	133347502	T	C	UTR3	GOLGA3	ENST000002	.	.	A	G	-	-	0.5	8,0,8,0	chr12,13334	Cytosol	A > G
chr13:20246341:A:G:UTR3:MPHOS	chr13	20246341	20246341	A	G	UTR3	MPHOSPH8	ENST000003	.	.	A	G	+	+	0.06666667	28,0,2,0	chr13,20246	Cytosol	A > G
chr13:20246359:A:G:UTR3:MPHOS	chr13	20246359	20246359	A	G	UTR3	MPHOSPH8	ENST000003	.	.	A	G	+	+	0.66	17,0,33,0	chr13,20246	Cytosol	A > G
chr13:20246385:A:G:UTR3:MPHOS	chr13	20246385	20246385	A	G	UTR3	MPHOSPH8	ENST000003	.	.	A	G	+	+	0.1025641	35,0,4,0	chr13,20246	Cytosol	A > G
chr13:20246402:A:G:UTR3:MPHOS	chr13	20246402	20246402	A	G	UTR3	MPHOSPH8	ENST000003	.	.	A	G	+	+	0.32653061	33,0,16,0	chr13,20246	Cytosol	A > G
chr13:20246518:A:G:UTR3:MPHOS	chr13	20246518	20246518	A	G	UTR3	MPHOSPH8	ENST000003	.	.	A	G	+	+	0.09090909	30,0,3,0	chr13,20246	Cytosol	A > G
chr13:20247023:A:G:UTR3:MPHOS	chr13	20247023	20247023	A	G	UTR3	MPHOSPH8	ENST000003	.	.	A	G	+	+	0.43548387	35,0,27,0	chr13,20247	Cytosol	A > G
chr13:20247024:A:G:UTR3:MPHOS	chr13	20247024	20247024	A	G	UTR3	MPHOSPH8	ENST000003	.	.	A	G	+	+	0.16129032	52,0,10,0	chr13,20247	Cytosol	A > G
chr13:20247029:A:G:UTR3:MPHOS	chr13	20247029	20247029	A	G	UTR3	MPHOSPH8	ENST000003	.	.	A	G	+	+	0.12244898	43,0,6,0	chr13,20247	Cytosol	A > G
chr13:20247075:A:G:UTR3:MPHOS	chr13	20247075	20247075	A	G	UTR3	MPHOSPH8	ENST000003	.	.	A	G	+	+-	0.22916667	37,0,11,0	chr13,20247	Cytosol	A > G
chr13:20247116:A:G:UTR3:MPHOS	chr13	20247116	20247116	A	G	UTR3	MPHOSPH8	ENST000003	.	.	A	G	+	+-	0.34375	21,0,11,0	chr13,20247	Cytosol	A > G
chr13:20247141:A:G:UTR3:MPHOS	chr13	20247141	20247141	A	G	UTR3	MPHOSPH8	ENST000003	.	.	A	G	+	+-	0.42857143	4,0,3,0	chr13,20247	Cytosol	A > G
chr13:21345170:T:C:intronic:N6AN	chr13	21345170	21345170	T	C	intronic	N6AMT2	.	.	.	A	G	-	-	0.6	2,0,3,0	chr13,21345	Cytosol	A > G
chr13:21948456:T:C:intergenic:RN	chr13	21948456	21948456	T	C	intergenic	RNA5SP25,ZI	dist=4418;dis	.	.	A	G	-	+-	0.09677419	28,0,3,0	chr13,21948	Cytosol	A > G
chr13:21948492:T:C:intergenic:RN	chr13	21948492	21948492	T	C	intergenic	RNA5SP25,ZI	dist=4454;dis	.	.	A	G	-	+-	0.21428571	77,0,21,0	chr13,21948	Cytosol	A > G
chr13:21948495:T:C:intergenic:RN	chr13	21948495	21948495	T	C	intergenic	RNA5SP25,ZI	dist=4457;dis	.	.	A	G	-	+-	0.13402062	84,0,13,0	chr13,21948	Cytosol	A > G
chr13:21948576:T:C:intergenic:RN	chr13	21948576	21948576	T	C	intergenic	RNA5SP25,ZI	dist=4538;dis	.	.	A	G	-	+-	0.29113924	112,0,46,0	chr13,21948	Cytosol	A > G
chr13:21948577:T:C:intergenic:RN	chr13	21948577	21948577	T	C	intergenic	RNA5SP25,ZI	dist=4539;dis	.	.	A	G	-	+-	0.30322581	108,0,47,0	chr13,21948	Cytosol	A > G
chr13:21948578:T:C:intergenic:RN	chr13	21948578	21948578	T	C	intergenic	RNA5SP25,ZI	dist=4540;dis	.	.	A	G	-	+-	0.15923567	132,0,25,0	chr13,21948	Cytosol	A > G
chr13:21948625:T:C:intergenic:RN	chr13	21948625	21948625	T	C	intergenic	RNA5SP25,ZI	dist=4587;dis	.	.	A	G	-	+-	0.09756098	74,0,8,0	chr13,21948	Cytosol	A > G
chr13:21949055:T:C:intergenic:RN	chr13	21949055	21949055	T	C	intergenic	RNA5SP25,ZI	dist=5017;dis	.	.	A	G	-	+-	0.13483146	77,0,12,0	chr13,21949	Cytosol	A > G

chr13:21949107:T:C:intergenic:RN	chr13	21949107	21949107	T	C	intergenic	RNA5SP25,ZI	dist=5069;dis	.	.	A	G	-	+-	0.49295775	72,0,70,0	chr13,21949	Cytosol	A > G
chr13:21949151:T:C:intergenic:RN	chr13	21949151	21949151	T	C	intergenic	RNA5SP25,ZI	dist=5113;dis	.	.	A	G	-	+-	0.22727273	68,0,20,0	chr13,21949	Cytosol	A > G
chr13:21949161:T:C:intergenic:RN	chr13	21949161	21949161	T	C	intergenic	RNA5SP25,ZI	dist=5123;dis	.	.	A	G	-	+-	0.6125	31,0,49,0	chr13,21949	Cytosol	A > G
chr13:21949165:T:C:intergenic:RN	chr13	21949165	21949165	T	C	intergenic	RNA5SP25,ZI	dist=5127;dis	.	.	A	G	-	+-	0.1369863	63,0,10,0	chr13,21949	Cytosol	A > G
chr13:25914977:A:G:UTR3:NUPL1	chr13	25914977	25914977	A	G	UTR3	NUPL1	ENST000003	.	.	A	G	+	+	0.01923077	102,0,2,0	chr13,25914	Cytosol	A > G
chr13:28121898:T:C:UTR3:LNX2	chr13	28121898	28121898	T	C	UTR3	LNX2	ENST000003	.	.	A	G	-	-	0.03846154	50,0,2,0	chr13,28121	Cytosol	A > G
chr13:32973183:A:G:UTR3:BRCA2	chr13	32973183	32973183	A	G	UTR3	BRCA2	ENST000003	.	.	A	G	+	+	0.1875	13,0,3,0	chr13,32973	Cytosol	A > G
chr13:32973189:A:G:UTR3:BRCA2	chr13	32973189	32973189	A	G	UTR3	BRCA2	ENST000003	.	.	A	G	+	+	0.125	14,0,2,0	chr13,32973	Cytosol	A > G
chr13:32973200:A:G:UTR3:BRCA2	chr13	32973200	32973200	A	G	UTR3	BRCA2	ENST000003	.	.	A	G	+	+	0.21052632	15,0,4,0	chr13,32973	Cytosol	A > G
chr13:32974133:A:G:downstream:	chr13	32974133	32974133	A	G	downstream	BRCA2,N4BP	.	.	.	A	G	+		0.3	7,0,3,0	chr13,32974	Cytosol	A > G
chr13:33089453:T:C:intronic:N4BP	chr13	33089453	33089453	T	C	intronic	N4BP2L2	.	.	.	A	G	-	-	0.6	2,0,3,0	chr13,33089	Cytosol	A > G
chr13:41384326:A:G:upstream;do	chr13	41384326	41384326	A	G	upstream;do	MIR621;SLC2	.	.	.	A	G	+	+-	0.85714286	1,0,6,0	chr13,41384	Cytosol	A > G
chr13:41384434:A:G:upstream;do	chr13	41384434	41384434	A	G	upstream;do	MIR621;SLC2	.	.	.	A	G	+	+-	1	0,0,5,0	chr13,41384	Cytosol	A > G
chr13:41384561:A:G:upstream;do	chr13	41384561	41384561	A	G	upstream;do	MIR621;SLC2	.	.	.	A	G	+	+-	0.36842105	12,0,7,0	chr13,41384	Cytosol	A > G
chr13:41385454:A:G:downstream:	chr13	41385454	41385454	A	G	downstream	MIR621	.	.	.	A	G	+	+-	0.66666667	2,0,4,0	chr13,41385	Cytosol	A > G
chr13:41790746:T:C:UTR3:MTRF1	chr13	41790746	41790746	T	C	UTR3	MTRF1	ENST000003	.	.	A	G	-	+-	0.3	7,0,3,0	chr13,41790	Cytosol	A > G
chr13:45604568:A:G:ncRNA_intro	chr13	45604568	45604568	A	G	ncRNA_intro	RP11-321C24	.	.	.	A	G	+	-+	0.6	2,0,3,0	chr13,45604	Cytosol	A > G
chr13:45604595:A:G:ncRNA_intro	chr13	45604595	45604595	A	G	ncRNA_intro	RP11-321C24	.	.	.	A	G	+	-+	0.33333333	6,0,3,0	chr13,45604	Cytosol	A > G
chr13:45604615:A:G:ncRNA_intro	chr13	45604615	45604615	A	G	ncRNA_intro	RP11-321C24	.	.	.	A	G	+	-+	0.5	5,0,5,0	chr13,45604	Cytosol	A > G
chr13:45604640:A:G:ncRNA_intro	chr13	45604640	45604640	A	G	ncRNA_intro	RP11-321C24	.	.	.	A	G	+	-+	0.63636364	4,0,7,0	chr13,45604	Cytosol	A > G
chr13:45604681:A:G:ncRNA_intro	chr13	45604681	45604681	A	G	ncRNA_intro	RP11-321C24	.	.	.	A	G	+	-+	0.66666667	2,0,4,0	chr13,45604	Cytosol	A > G
chr13:45604696:A:G:ncRNA_intro	chr13	45604696	45604696	A	G	ncRNA_intro	RP11-321C24	.	.	.	A	G	+	-+	0.33333333	4,0,2,0	chr13,45604	Cytosol	A > G
chr13:45604753:A:G:ncRNA_intro	chr13	45604753	45604753	A	G	ncRNA_intro	RP11-321C24	.	.	.	A	G	+	-+	0.28571429	10,0,4,0	chr13,45604	Cytosol	A > G
chr13:45605029:A:G:ncRNA_intro	chr13	45605029	45605029	A	G	ncRNA_intro	RP11-321C24	.	.	.	A	G	+	-+	1	0,0,5,0	chr13,45605	Cytosol	A > G
chr13:45605033:A:G:splicing:GPAL	chr13	45605033	45605033	A	G	splicing	GPALPP1	ENST000003	.	.	A	G	+	-+	0.4	3,0,2,0	chr13,45605	Cytosol	A > G
chr13:45605144:A:G:ncRNA_intro	chr13	45605144	45605144	A	G	ncRNA_intro	RP11-321C24	.	.	.	A	G	+	-+	0.57142857	3,0,4,0	chr13,45605	Cytosol	A > G
chr13:50000742:T:C:intronic:CAB3	chr13	50000742	50000742	T	C	intronic	CAB39L	.	.	.	A	G	-	-	0.6	2,0,3,0	chr13,50000	Cytosol	A > G
chr13:50487293:T:C:UTR3:SPRYD7	chr13	50487293	50487293	T	C	UTR3	SPRYD7	ENST000003	.	.	A	G	-	-	0.18181818	18,0,4,0	chr13,50487	Cytosol	A > G
chr13:50487313:T:C:UTR3:SPRYD7	chr13	50487313	50487313	T	C	UTR3	SPRYD7	ENST000003	.	.	A	G	-	-	0.52380952	10,0,11,0	chr13,50487	Cytosol	A > G
chr13:50487314:T:C:UTR3:SPRYD7	chr13	50487314	50487314	T	C	UTR3	SPRYD7	ENST000003	.	.	A	G	-	-	0.2	16,0,4,0	chr13,50487	Cytosol	A > G
chr13:50487339:T:C:UTR3:SPRYD7	chr13	50487339	50487339	T	C	UTR3	SPRYD7	ENST000003	.	.	A	G	-	-	0.33333333	10,0,5,0	chr13,50487	Cytosol	A > G
chr13:50487353:T:C:UTR3:SPRYD7	chr13	50487353	50487353	T	C	UTR3	SPRYD7	ENST000003	.	.	A	G	-	-	0.4375	9,0,7,0	chr13,50487	Cytosol	A > G
chr13:50487434:T:C:UTR3:SPRYD7	chr13	50487434	50487434	T	C	UTR3	SPRYD7	ENST000003	.	.	A	G	-	-	0.125	14,0,2,0	chr13,50487	Cytosol	A > G
chr13:50487444:T:C:UTR3:SPRYD7	chr13	50487444	50487444	T	C	UTR3	SPRYD7	ENST000003	.	.	A	G	-	-	0.75	3,0,9,0	chr13,50487	Cytosol	A > G
chr13:50487473:T:C:UTR3:SPRYD7	chr13	50487473	50487473	T	C	UTR3	SPRYD7	ENST000003	.	.	A	G	-	-	0.8	2,0,8,0	chr13,50487	Cytosol	A > G
chr13:50487480:T:C:UTR3:SPRYD7	chr13	50487480	50487480	T	C	UTR3	SPRYD7	ENST000003	.	.	A	G	-	-	0.91666667	1,0,11,0	chr13,50487	Cytosol	A > G
chr13:50487701:T:C:UTR3:SPRYD7	chr13	50487701	50487701	T	C	UTR3	SPRYD7	ENST000003	.	.	A	G	-	-	0.44444444	5,0,4,0	chr13,50487	Cytosol	A > G
chr13:50487787:T:C:UTR3:SPRYD7	chr13	50487787	50487787	T	C	UTR3	SPRYD7	ENST000003	.	.	A	G	-	-	0.5	6,0,6,0	chr13,50487	Cytosol	A > G
chr13:50487823:T:C:UTR3:SPRYD7	chr13	50487823	50487823	T	C	UTR3	SPRYD7	ENST000003	.	.	A	G	-	-	0.27777778	13,0,5,0	chr13,50487	Cytosol	A > G
chr13:50487857:T:C:UTR3:SPRYD7	chr13	50487857	50487857	T	C	UTR3	SPRYD7	ENST000003	.	.	A	G	-	-	0.56521739	10,0,13,0	chr13,50487	Cytosol	A > G
chr13:50487867:T:C:UTR3:SPRYD7	chr13	50487867	50487867	T	C	UTR3	SPRYD7	ENST000003	.	.	A	G	-	-	0.23809524	16,0,5,0	chr13,50487	Cytosol	A > G
chr13:50487934:T:C:UTR3:SPRYD7	chr13	50487934	50487934	T	C	UTR3	SPRYD7	ENST000003	.	.	A	G	-	-	0.63636364	4,0,7,0	chr13,50487	Cytosol	A > G
chr13:50487941:T:C:UTR3:SPRYD7	chr13	50487941	50487941	T	C	UTR3	SPRYD7	ENST000003	.	.	A	G	-	-	0.75	2,0,6,0	chr13,50487	Cytosol	A > G
chr13:50487943:T:C:UTR3:SPRYD7	chr13	50487943	50487943	T	C	UTR3	SPRYD7	ENST000003	.	.	A	G	-	-	0.71428571	2,0,5,0	chr13,50487	Cytosol	A > G
chr13:52035196:C:A:ncRNA_exoni	chr13	52035196	52035196	C	A	ncRNA_exon	RPS4XP16	.	.	.	C	A	+	+	0.66666667	4,2,0,0	chr13,52035	Cytosol	C > A
chr13:52035228:G:A:ncRNA_exoni	chr13	52035228	52035228	G	A	ncRNA_exon	RPS4XP16	.	.	.	G	A	+	+	0.83333333	5,0,1,0	chr13,52035	Cytosol	G > A
chr13:53203935:A:G:intronic:HNR	chr13	53203935	53203935	A	G	intronic	HNRNPA1L2	.	.	.	A	G	+	+	0.15789474	16,0,3,0	chr13,53203	Cytosol	A > G
chr13:53203942:A:G:intronic:HNR	chr13	53203942	53203942	A	G	intronic	HNRNPA1L2	.	.	.	A	G	+	+	0.27272727	16,0,6,0	chr13,53203	Cytosol	A > G
chr13:53203951:A:G:intronic:HNR	chr13	53203951	53203951	A	G	intronic	HNRNPA1L2	.	.	.	A	G	+	+	0.35294118	11,0,6,0	chr13,53203	Cytosol	A > G
chr13:53203983:A:G:intronic:HNR	chr13	53203983	53203983	A	G	intronic	HNRNPA1L2	.	.	.	A	G	+	+	0.56	11,0,14,0	chr13,53203	Cytosol	A > G
chr13:53204052:A:G:intronic:HNR	chr13	53204052	53204052	A	G	intronic	HNRNPA1L2	.	.	.	A	G	+	+	0.22222222	7,0,2,0	chr13,53204	Cytosol	A > G
chr13:53204103:A:G:intronic:HNR	chr13	53204103	53204103	A	G	intronic	HNRNPA1L2	.	.	.	A	G	+	+	0.1	18,0,2,0	chr13,53204	Cytosol	A > G
chr13:53204549:A:G:intronic:HNR	chr13	53204549	53204549	A	G	intronic	HNRNPA1L2	.	.	.	A	G	+	+	0.66666667	3,0,6,0	chr13,53204	Cytosol	A > G
chr13:53204590:A:G:intronic:HNR	chr13	53204590	53204590	A	G	intronic	HNRNPA1L2	.	.	.	A	G	+	+	0.54545455	5,0,6,0	chr13,53204	Cytosol	A > G
chr13:53204593:A:G:intronic:HNR	chr13	53204593	53204593	A	G	intronic	HNRNPA1L2	.	.	.	A	G	+	+	0.36363636	7,0,4,0	chr13,53204	Cytosol	A > G
chr13:53204599:A:G:intronic:HNR	chr13	53204599	53204599	A	G	intronic	HNRNPA1L2	.	.	.	A	G	+	+	0.66666667	3,0,6,0	chr13,53204	Cytosol	A > G
chr13:53204666:A:G:intronic:HNR	chr13	53204666	53204666	A	G	intronic	HNRNPA1L2	.	.	.	A	G	+	+	0.4	12,0,8,0	chr13,53204	Cytosol	A > G
chr13:53204680:A:G:intronic:HNR	chr13	53204680	53204680	A	G	intronic	HNRNPA1L2	.	.	.	A	G	+	+	0.44444444	10,0,8,0	chr13,53204	Cytosol	A > G
chr13:111549730:T:C:ncRNA_intro	chr13	111549730	111549730	T	C	ncRNA_intro	ANKRD10-IT1	.	.	.	A	G	-	-	0.8	1,0,4,0	chr13,11154	Cytosol	A > G
chr13:111552341:T:C:intronic:ANK	chr13	111552341	111552341	T	C	intronic	ANKRD10	.	.	.	A	G	-	-	0.6	2,0,3,0	chr13,11155	Cytosol	A > G
chr13:111552387:T:C:intronic:ANK	chr13	111552387	111552387	T	C	intronic	ANKRD10	.	.	.	A	G	-	-	0.6	2,0,3,0	chr13,11155	Cytosol	A > G
chr13:115049758:A:G:intronic:UPI	chr13	115049758	115049758	A	G	intronic	UPF3A	.	.	.	A	G	+	+	0.45454545	6,0,5,0	chr13,11504	Cytosol	A > G
chr13:115052945:A:G:intronic:UPI	chr13	115052945	115052945	A	G	intronic	UPF3A	.	.	.	A	G	+	+	0.11764706	15,0,2,0	chr13,11505	Cytosol	A > G
chr14:20835278:T:C:UTR3:TEP1	chr14	20835278	20835278	T	C	UTR3	TEP1	ENST000002	.	.	A	G	-	-	0.4	3,0,2,0	chr14,20835	Cytosol	A > G

chr14:20835288:T:C:UTR3:TEP1	chr14	20835288	20835288	T	C	UTR3	TEP1	ENST000002	.	.	A	G	-	-	0.5	3,0,3,0	chr14,20835	Cytosol	A > G
chr14:20835340:T:C:UTR3:TEP1	chr14	20835340	20835340	T	C	UTR3	TEP1	ENST000002	.	.	A	G	-	-	0.4	3,0,2,0	chr14,20835	Cytosol	A > G
chr14:20836040:T:C:UTR3:TEP1	chr14	20836040	20836040	T	C	UTR3	TEP1	ENST000002	.	.	A	G	-	-	0.33333333	4,0,2,0	chr14,20836	Cytosol	A > G
chr14:20836041:T:C:UTR3:TEP1	chr14	20836041	20836041	T	C	UTR3	TEP1	ENST000002	.	.	A	G	-	-	0.6	2,0,3,0	chr14,20836	Cytosol	A > G
chr14:21928490:T:C:UTR3:RAB2B	chr14	21928490	21928490	T	C	UTR3	RAB2B	ENST000003	.	.	A	G	-	-	0.13333333	13,0,2,0	chr14,21928	Cytosol	A > G
chr14:21928501:T:C:UTR3:RAB2B	chr14	21928501	21928501	T	C	UTR3	RAB2B	ENST000003	.	.	A	G	-	-	0.18181818	9,0,2,0	chr14,21928	Cytosol	A > G
chr14:21928578:T:C:UTR3:RAB2B	chr14	21928578	21928578	T	C	UTR3	RAB2B	ENST000003	.	.	A	G	-	-	0.33333333	6,0,3,0	chr14,21928	Cytosol	A > G
chr14:21928622:T:C:UTR3:RAB2B	chr14	21928622	21928622	T	C	UTR3	RAB2B	ENST000003	.	.	A	G	-	-	0.55555556	4,0,5,0	chr14,21928	Cytosol	A > G
chr14:21928632:T:C:UTR3:RAB2B	chr14	21928632	21928632	T	C	UTR3	RAB2B	ENST000003	.	.	A	G	-	-	0.84615385	2,0,11,0	chr14,21928	Cytosol	A > G
chr14:21928658:T:C:UTR3:RAB2B	chr14	21928658	21928658	T	C	UTR3	RAB2B	ENST000003	.	.	A	G	-	-	0.1875	13,0,3,0	chr14,21928	Cytosol	A > G
chr14:21928681:T:C:UTR3:RAB2B	chr14	21928681	21928681	T	C	UTR3	RAB2B	ENST000003	.	.	A	G	-	-	0.28571429	10,0,4,0	chr14,21928	Cytosol	A > G
chr14:21928682:T:C:UTR3:RAB2B	chr14	21928682	21928682	T	C	UTR3	RAB2B	ENST000003	.	.	A	G	-	-	0.15384615	11,0,2,0	chr14,21928	Cytosol	A > G
chr14:21928700:T:C:UTR3:RAB2B	chr14	21928700	21928700	T	C	UTR3	RAB2B	ENST000003	.	.	A	G	-	-	0.66666667	3,0,6,0	chr14,21928	Cytosol	A > G
chr14:21928846:T:C:UTR3:RAB2B	chr14	21928846	21928846	T	C	UTR3	RAB2B	ENST000003	.	.	A	G	-	-	0.13333333	26,0,4,0	chr14,21928	Cytosol	A > G
chr14:21928851:T:C:UTR3:RAB2B	chr14	21928851	21928851	T	C	UTR3	RAB2B	ENST000003	.	.	A	G	-	-	0.53333333	14,0,16,0	chr14,21928	Cytosol	A > G
chr14:21928910:T:C:UTR3:RAB2B	chr14	21928910	21928910	T	C	UTR3	RAB2B	ENST000003	.	.	A	G	-	-	0.47058824	9,0,8,0	chr14,21928	Cytosol	A > G
chr14:21928911:T:C:UTR3:RAB2B	chr14	21928911	21928911	T	C	UTR3	RAB2B	ENST000003	.	.	A	G	-	-	0.58823529	7,0,10,0	chr14,21928	Cytosol	A > G
chr14:21928941:T:C:UTR3:RAB2B	chr14	21928941	21928941	T	C	UTR3	RAB2B	ENST000003	.	.	A	G	-	-	0.0952381	19,0,2,0	chr14,21928	Cytosol	A > G
chr14:21928996:T:C:UTR3:RAB2B	chr14	21928996	21928996	T	C	UTR3	RAB2B	ENST000003	.	.	A	G	-	-	0.15	17,0,3,0	chr14,21928	Cytosol	A > G
chr14:21928998:T:C:UTR3:RAB2B	chr14	21928998	21928998	T	C	UTR3	RAB2B	ENST000003	.	.	A	G	-	-	0.45	11,0,9,0	chr14,21928	Cytosol	A > G
chr14:21928999:T:C:UTR3:RAB2B	chr14	21928999	21928999	T	C	UTR3	RAB2B	ENST000003	.	.	A	G	-	-	0.2	16,0,4,0	chr14,21928	Cytosol	A > G
chr14:21929044:T:C:UTR3:RAB2B	chr14	21929044	21929044	T	C	UTR3	RAB2B	ENST000003	.	.	A	G	-	-	0.42105263	11,0,8,0	chr14,21929	Cytosol	A > G
chr14:21929045:T:C:UTR3:RAB2B	chr14	21929045	21929045	T	C	UTR3	RAB2B	ENST000003	.	.	A	G	-	-	0.11111111	16,0,2,0	chr14,21929	Cytosol	A > G
chr14:21965805:A:G:UTR3:TOX4	chr14	21965805	21965805	A	G	UTR3	TOX4	ENST000004	.	.	A	G	+	+	0.33333333	4,0,2,0	chr14,21965	Cytosol	A > G
chr14:23441395:T:C:UTR3:AJUBA	chr14	23441395	23441395	T	C	UTR3	AJUBA	ENST000002	.	.	A	G	-	-	0.17621145	187,0,40,0	chr14,23441	Cytosol	A > G
chr14:23441439:T:C:UTR3:AJUBA	chr14	23441439	23441439	T	C	UTR3	AJUBA	ENST000002	.	.	A	G	-	-	0.10294118	183,0,21,0	chr14,23441	Cytosol	A > G
chr14:23441476:T:C:UTR3:AJUBA	chr14	23441476	23441476	T	C	UTR3	AJUBA	ENST000002	.	.	A	G	-	-	0.75	68,0,204,0	chr14,23441	Cytosol	A > G
chr14:23442051:T:C:UTR3:AJUBA	chr14	23442051	23442051	T	C	UTR3	AJUBA	ENST000002	.	.	A	G	-	-	0.08090615	284,0,25,0	chr14,23442	Cytosol	A > G
chr14:23442109:T:C:UTR3:AJUBA	chr14	23442109	23442109	T	C	UTR3	AJUBA	ENST000002	.	.	A	G	-	-	0.06355932	221,0,15,0	chr14,23442	Cytosol	A > G
chr14:23442122:T:C:UTR3:AJUBA	chr14	23442122	23442122	T	C	UTR3	AJUBA	ENST000002	.	.	A	G	-	-	0.16287879	221,0,43,0	chr14,23442	Cytosol	A > G
chr14:23442148:T:C:UTR3:AJUBA	chr14	23442148	23442148	T	C	UTR3	AJUBA	ENST000002	.	.	A	G	-	-	0.16513761	182,0,36,0	chr14,23442	Cytosol	A > G
chr14:23442149:T:C:UTR3:AJUBA	chr14	23442149	23442149	T	C	UTR3	AJUBA	ENST000002	.	.	A	G	-	-	0.48148148	112,0,104,0	chr14,23442	Cytosol	A > G
chr14:23442150:T:C:UTR3:AJUBA	chr14	23442150	23442150	T	C	UTR3	AJUBA	ENST000002	.	.	A	G	-	-	0.26794258	153,0,56,0	chr14,23442	Cytosol	A > G
chr14:23566025:A:G:intronic:C14c	chr14	23566025	23566025	A	G	intronic	C14orf119	.	.	.	A	G	+	+	0.25	6,0,2,0	chr14,23566	Cytosol	A > G
chr14:24455327:A:G:intronic:DHR:	chr14	24455327	24455327	A	G	intronic	DHRS4L2	.	.	.	A	G	+	-+	0.55555556	4,0,5,0	chr14,24455	Cytosol	A > G
chr14:24913829:A:G:intergenic:SD	chr14	24913829	24913829	A	G	intergenic	SDR39U1,RP	dist=1765;dis	.	.	A	G	+	+	0.38095238	13,0,8,0	chr14,24913	Cytosol	A > G
chr14:24913861:A:G:intergenic:SD	chr14	24913861	24913861	A	G	intergenic	SDR39U1,RP	dist=1797;dis	.	.	A	G	+	+	0.10526316	17,0,2,0	chr14,24913	Cytosol	A > G
chr14:31562756:A:G:UTR3:AP4S1	chr14	31562756	31562756	A	G	UTR3	AP4S1	ENST000005	.	.	A	G	+	+	0.3	7,0,3,0	chr14,31562	Cytosol	A > G
chr14:31562792:A:G:UTR3:AP4S1	chr14	31562792	31562792	A	G	UTR3	AP4S1	ENST000005	.	.	A	G	+	+	0.625	3,0,5,0	chr14,31562	Cytosol	A > G
chr14:31563591:A:G:downstream:	chr14	31563591	31563591	A	G	downstream	AP4S1	.	.	.	A	G	+	+	0.69230769	4,0,9,0	chr14,31563	Cytosol	A > G
chr14:31563655:A:G:downstream:	chr14	31563655	31563655	A	G	downstream	AP4S1	.	.	.	A	G	+	+	0.22222222	7,0,2,0	chr14,31563	Cytosol	A > G
chr14:31563681:A:G:downstream:	chr14	31563681	31563681	A	G	downstream	AP4S1	.	.	.	A	G	+	+	0.27272727	8,0,3,0	chr14,31563	Cytosol	A > G
chr14:31564294:A:G:intergenic:AF	chr14	31564294	31564294	A	G	intergenic	AP4S1,HECTI	dist=1476;dis	.	.	A	G	+	+	0.4	3,0,2,0	chr14,31564	Cytosol	A > G
chr14:31564650:A:G:intergenic:AF	chr14	31564650	31564650	A	G	intergenic	AP4S1,HECTI	dist=1832;dis	.	.	A	G	+	+	0.82352941	3,0,14,0	chr14,31564	Cytosol	A > G
chr14:31564700:A:G:intergenic:AF	chr14	31564700	31564700	A	G	intergenic	AP4S1,HECTI	dist=1882;dis	.	.	A	G	+	+	1	0,0,19,0	chr14,31564	Cytosol	A > G
chr14:31564722:A:G:intergenic:AF	chr14	31564722	31564722	A	G	intergenic	AP4S1,HECTI	dist=1904;dis	.	.	A	G	+	+	0.61538462	5,0,8,0	chr14,31564	Cytosol	A > G
chr14:31564764:A:G:intergenic:AF	chr14	31564764	31564764	A	G	intergenic	AP4S1,HECTI	dist=1946;dis	.	.	A	G	+	+	0.3	7,0,3,0	chr14,31564	Cytosol	A > G
chr14:31565088:A:G:intergenic:AF	chr14	31565088	31565088	A	G	intergenic	AP4S1,HECTI	dist=2270;dis	.	.	A	G	+	+	0.66666667	2,0,4,0	chr14,31565	Cytosol	A > G
chr14:31565089:A:G:intergenic:AF	chr14	31565089	31565089	A	G	intergenic	AP4S1,HECTI	dist=2271;dis	.	.	A	G	+	+	0.33333333	4,0,2,0	chr14,31565	Cytosol	A > G
chr14:31565104:A:G:intergenic:AF	chr14	31565104	31565104	A	G	intergenic	AP4S1,HECTI	dist=2286;dis	.	.	A	G	+	+	0.5	3,0,3,0	chr14,31565	Cytosol	A > G
chr14:31565144:A:G:intergenic:AF	chr14	31565144	31565144	A	G	intergenic	AP4S1,HECTI	dist=2326;dis	.	.	A	G	+	+	0.5	3,0,3,0	chr14,31565	Cytosol	A > G
chr14:31565211:A:G:intergenic:AF	chr14	31565211	31565211	A	G	intergenic	AP4S1,HECTI	dist=2393;dis	.	.	A	G	+	+	0.5	3,0,3,0	chr14,31565	Cytosol	A > G
chr14:31565212:A:G:intergenic:AF	chr14	31565212	31565212	A	G	intergenic	AP4S1,HECTI	dist=2394;dis	.	.	A	G	+	+	1	0,0,6,0	chr14,31565	Cytosol	A > G
chr14:31916461:T:C:ncRNA_intror	chr14	31916461	31916461	T	C	ncRNA_intro	CTD-2213F21	.	.	.	A	G	-	+-	0.46376812	37,0,32,0	chr14,31916	Cytosol	A > G
chr14:31916462:T:C:ncRNA_intror	chr14	31916462	31916462	T	C	ncRNA_intro	CTD-2213F21	.	.	.	A	G	-	+-	0.4057971	41,0,28,0	chr14,31916	Cytosol	A > G
chr14:31916464:T:C:ncRNA_intror	chr14	31916464	31916464	T	C	ncRNA_intro	CTD-2213F21	.	.	.	A	G	-	+-	0.59701493	27,0,40,0	chr14,31916	Cytosol	A > G
chr14:31916470:T:C:ncRNA_intror	chr14	31916470	31916470	T	C	ncRNA_intro	CTD-2213F21	.	.	.	A	G	-	+-	0.05882353	64,0,4,0	chr14,31916	Cytosol	A > G
chr14:31916782:T:C:ncRNA_intror	chr14	31916782	31916782	T	C	ncRNA_intro	CTD-2213F21	.	.	.	A	G	-	+-	0.53125	15,0,17,0	chr14,31916	Cytosol	A > G
chr14:35743447:A:G:UTR3:KIAA03	chr14	35743447	35743447	A	G	UTR3	KIAA0391	ENST000005	.	.	A	G	+	+	0.5	6,0,6,0	chr14,35743	Cytosol	A > G
chr14:35743453:A:G:UTR3:KIAA03	chr14	35743453	35743453	A	G	UTR3	KIAA0391	ENST000005	.	.	A	G	+	+	0.46153846	7,0,6,0	chr14,35743	Cytosol	A > G
chr14:35743458:A:G:UTR3:KIAA03	chr14	35743458	35743458	A	G	UTR3	KIAA0391	ENST000005	.	.	A	G	+	+	0.14285714	12,0,2,0	chr14,35743	Cytosol	A > G
chr14:35743466:A:G:UTR3:KIAA03	chr14	35743466	35743466	A	G	UTR3	KIAA0391	ENST000005	.	.	A	G	+	+	0.91666667	1,0,11,0	chr14,35743	Cytosol	A > G
chr14:35743556:A:G:intronic:KIAA	chr14	35743556	35743556	A	G	intronic	KIAA0391	.	.	.	A	G	+	+	0.25	6,0,2,0	chr14,35743	Cytosol	A > G
chr14:35743675:A:G:intronic:KIAA	chr14	35743675	35743675	A	G	intronic	KIAA0391	.	.	.	A	G	+	+	0.8	1,0,4,0	chr14,35743	Cytosol	A > G

chr14:35743689:A:G:intronic:KIAA	chr14	35743689	35743689	A	G	intronic	KIAA0391	.	.	.	A	G	+	+	0.83333333	1,0,5,0	chr14,35743	Cytosol	A > G
chr14:35743739:A:G:intronic:KIAA	chr14	35743739	35743739	A	G	intronic	KIAA0391	.	.	.	A	G	+	+	0.55555556	4,0,5,0	chr14,35743	Cytosol	A > G
chr14:35745251:A:G:intronic:KIAA	chr14	35745251	35745251	A	G	intronic	KIAA0391	.	.	.	A	G	+	+	0.15384615	11,0,2,0	chr14,35745	Cytosol	A > G
chr14:35745253:A:G:intronic:KIAA	chr14	35745253	35745253	A	G	intronic	KIAA0391	.	.	.	A	G	+	+	0.2	12,0,3,0	chr14,35745	Cytosol	A > G
chr14:35745259:A:G:intronic:KIAA	chr14	35745259	35745259	A	G	intronic	KIAA0391	.	.	.	A	G	+	+	0.2	12,0,3,0	chr14,35745	Cytosol	A > G
chr14:35745300:A:G:intronic:KIAA	chr14	35745300	35745300	A	G	intronic	KIAA0391	.	.	.	A	G	+	+	0.25	12,0,4,0	chr14,35745	Cytosol	A > G
chr14:35745304:A:G:intronic:KIAA	chr14	35745304	35745304	A	G	intronic	KIAA0391	.	.	.	A	G	+	+	0.125	14,0,2,0	chr14,35745	Cytosol	A > G
chr14:35745428:A:G:intronic:KIAA	chr14	35745428	35745428	A	G	intronic	KIAA0391	.	.	.	A	G	+	+	0.25	6,0,2,0	chr14,35745	Cytosol	A > G
chr14:35745452:A:G:intronic:KIAA	chr14	35745452	35745452	A	G	intronic	KIAA0391	.	.	.	A	G	+	+	0.5	4,0,4,0	chr14,35745	Cytosol	A > G
chr14:35745476:A:G:intronic:KIAA	chr14	35745476	35745476	A	G	intronic	KIAA0391	.	.	.	A	G	+	+	0.25	6,0,2,0	chr14,35745	Cytosol	A > G
chr14:35746288:A:G:intronic:KIAA	chr14	35746288	35746288	A	G	intronic	KIAA0391	.	.	.	A	G	+	+	0.625	6,0,10,0	chr14,35746	Cytosol	A > G
chr14:35746335:A:G:intronic:KIAA	chr14	35746335	35746335	A	G	intronic	KIAA0391	.	.	.	A	G	+	+	0.2	12,0,3,0	chr14,35746	Cytosol	A > G
chr14:35746361:A:G:intronic:KIAA	chr14	35746361	35746361	A	G	intronic	KIAA0391	.	.	.	A	G	+	+	0.11764706	15,0,2,0	chr14,35746	Cytosol	A > G
chr14:35746398:A:G:intronic:KIAA	chr14	35746398	35746398	A	G	intronic	KIAA0391	.	.	.	A	G	+	+	0.2	16,0,4,0	chr14,35746	Cytosol	A > G
chr14:35746412:A:G:intronic:KIAA	chr14	35746412	35746412	A	G	intronic	KIAA0391	.	.	.	A	G	+	+	0.1	18,0,2,0	chr14,35746	Cytosol	A > G
chr14:35746415:A:G:intronic:KIAA	chr14	35746415	35746415	A	G	intronic	KIAA0391	.	.	.	A	G	+	+	0.14285714	12,0,2,0	chr14,35746	Cytosol	A > G
chr14:35746479:A:G:intronic:KIAA	chr14	35746479	35746479	A	G	intronic	KIAA0391	.	.	.	A	G	+	+	0.17647059	14,0,3,0	chr14,35746	Cytosol	A > G
chr14:35746484:A:G:intronic:KIAA	chr14	35746484	35746484	A	G	intronic	KIAA0391	.	.	.	A	G	+	+	0.29411765	12,0,5,0	chr14,35746	Cytosol	A > G
chr14:35746494:A:G:intronic:KIAA	chr14	35746494	35746494	A	G	intronic	KIAA0391	.	.	.	A	G	+	+	0.35714286	9,0,5,0	chr14,35746	Cytosol	A > G
chr14:35786941:A:G:downstream:	chr14	35786941	35786941	A	G	downstream	KIAA0391,PS	.	.	.	A	G	+		0.03448276	56,0,2,0	chr14,35786	Cytosol	A > G
chr14:50051382:T:C:intronic:RPS2	chr14	50051382	50051382	T	C	intronic	RPS29	.	.	.	A	G	-	-	0.21428571	11,0,3,0	chr14,50051	Cytosol	A > G
chr14:50051454:T:C:intronic:RPS2	chr14	50051454	50051454	T	C	intronic	RPS29	.	.	.	A	G	-	-	0.33333333	4,0,2,0	chr14,50051	Cytosol	A > G
chr14:50066926:A:G:intronic:LRR1	chr14	50066926	50066926	A	G	intronic	LRR1	.	.	.	A	G	+	-+	0.33333333	8,0,4,0	chr14,50066	Cytosol	A > G
chr14:50712583:T:C:UTR3:L2HGD	chr14	50712583	50712583	T	C	UTR3	L2HGDH	ENST000002	.	.	A	G	-	-	0.05072464	131,0,7,0	chr14,50712	Cytosol	A > G
chr14:50712717:T:C:UTR3:L2HGD	chr14	50712717	50712717	T	C	UTR3	L2HGDH	ENST000002	.	.	A	G	-	-	0.04878049	78,0,4,0	chr14,50712	Cytosol	A > G
chr14:50712719:T:C:UTR3:L2HGD	chr14	50712719	50712719	T	C	UTR3	L2HGDH	ENST000002	.	.	A	G	-	-	0.22891566	64,0,19,0	chr14,50712	Cytosol	A > G
chr14:50712741:T:C:UTR3:L2HGD	chr14	50712741	50712741	T	C	UTR3	L2HGDH	ENST000002	.	.	A	G	-	-	0.1509434	45,0,8,0	chr14,50712	Cytosol	A > G
chr14:50712778:T:C:UTR3:L2HGD	chr14	50712778	50712778	T	C	UTR3	L2HGDH	ENST000002	.	.	A	G	-	-	0.13414634	71,0,11,0	chr14,50712	Cytosol	A > G
chr14:50713015:T:C:UTR3:L2HGD	chr14	50713015	50713015	T	C	UTR3	L2HGDH	ENST000002	.	.	A	G	-	-	0.35	26,0,14,0	chr14,50713	Cytosol	A > G
chr14:50713052:T:C:UTR3:L2HGD	chr14	50713052	50713052	T	C	UTR3	L2HGDH	ENST000002	.	.	A	G	-	-	0.225	31,0,9,0	chr14,50713	Cytosol	A > G
chr14:50713100:T:C:UTR3:L2HGD	chr14	50713100	50713100	T	C	UTR3	L2HGDH	ENST000002	.	.	A	G	-	-	0.33333333	4,0,2,0	chr14,50713	Cytosol	A > G
chr14:50713268:T:C:UTR3:L2HGD	chr14	50713268	50713268	T	C	UTR3	L2HGDH	ENST000002	.	.	A	G	-	-	0.46666667	16,0,14,0	chr14,50713	Cytosol	A > G
chr14:50713304:T:C:UTR3:L2HGD	chr14	50713304	50713304	T	C	UTR3	L2HGDH	ENST000002	.	.	A	G	-	-	0.4137931	17,0,12,0	chr14,50713	Cytosol	A > G
chr14:50713578:T:C:UTR3:L2HGD	chr14	50713578	50713578	T	C	UTR3	L2HGDH	ENST000002	.	.	A	G	-	-	0.3125	11,0,5,0	chr14,50713	Cytosol	A > G
chr14:50713579:T:C:UTR3:L2HGD	chr14	50713579	50713579	T	C	UTR3	L2HGDH	ENST000002	.	.	A	G	-	-	0.16666667	15,0,3,0	chr14,50713	Cytosol	A > G
chr14:50792676:A:G:UTR3:ATP5S	chr14	50792676	50792676	A	G	UTR3	ATP5S	ENST000002	.	.	A	G	+	+	0.23684211	29,0,9,0	chr14,50792	Cytosol	A > G
chr14:50792716:A:G:UTR3:ATP5S	chr14	50792716	50792716	A	G	UTR3	ATP5S	ENST000002	.	.	A	G	+	+	0.39130435	14,0,9,0	chr14,50792	Cytosol	A > G
chr14:50792723:A:G:UTR3:ATP5S	chr14	50792723	50792723	A	G	UTR3	ATP5S	ENST000002	.	.	A	G	+	+	0.4	15,0,10,0	chr14,50792	Cytosol	A > G
chr14:50792746:A:G:UTR3:ATP5S	chr14	50792746	50792746	A	G	UTR3	ATP5S	ENST000002	.	.	A	G	+	+	0.11538462	23,0,3,0	chr14,50792	Cytosol	A > G
chr14:50792747:A:G:UTR3:ATP5S	chr14	50792747	50792747	A	G	UTR3	ATP5S	ENST000002	.	.	A	G	+	+	0.23076923	20,0,6,0	chr14,50792	Cytosol	A > G
chr14:50792763:A:G:UTR3:ATP5S	chr14	50792763	50792763	A	G	UTR3	ATP5S	ENST000002	.	.	A	G	+	+	0.0625	30,0,2,0	chr14,50792	Cytosol	A > G
chr14:50792787:A:G:UTR3:ATP5S	chr14	50792787	50792787	A	G	UTR3	ATP5S	ENST000002	.	.	A	G	+	+	0.25	33,0,11,0	chr14,50792	Cytosol	A > G
chr14:50792788:A:G:UTR3:ATP5S	chr14	50792788	50792788	A	G	UTR3	ATP5S	ENST000002	.	.	A	G	+	+	0.17021277	39,0,8,0	chr14,50792	Cytosol	A > G
chr14:50792796:A:G:UTR3:ATP5S	chr14	50792796	50792796	A	G	UTR3	ATP5S	ENST000002	.	.	A	G	+	+	0.04081633	47,0,2,0	chr14,50792	Cytosol	A > G
chr14:50792827:A:G:UTR3:ATP5S	chr14	50792827	50792827	A	G	UTR3	ATP5S	ENST000002	.	.	A	G	+	+	0.17307692	43,0,9,0	chr14,50792	Cytosol	A > G
chr14:50792837:A:G:UTR3:ATP5S	chr14	50792837	50792837	A	G	UTR3	ATP5S	ENST000002	.	.	A	G	+	+	0.15686275	43,0,8,0	chr14,50792	Cytosol	A > G
chr14:50792863:A:G:UTR3:ATP5S	chr14	50792863	50792863	A	G	UTR3	ATP5S	ENST000002	.	.	A	G	+	+	0.11111111	48,0,6,0	chr14,50792	Cytosol	A > G
chr14:50792897:A:G:UTR3:ATP5S	chr14	50792897	50792897	A	G	UTR3	ATP5S	ENST000002	.	.	A	G	+	+	0.05405405	35,0,2,0	chr14,50792	Cytosol	A > G
chr14:50793577:A:G:ncRNA_intro	chr14	50793577	50793577	A	G	ncRNA_intro	RP11-247L2C	.	.	.	A	G	+	+-	0.08823529	31,0,3,0	chr14,50793	Cytosol	A > G
chr14:50793593:T:C:ncRNA_intro	chr14	50793593	50793593	T	C	ncRNA_intro	RP11-247L2C	.	.	.	A	G	-	+-	0.375	5,0,3,0	chr14,50793	Cytosol	A > G
chr14:50793612:A:G:ncRNA_intro	chr14	50793612	50793612	A	G	ncRNA_intro	RP11-247L2C	.	.	.	A	G	+	+-	0.04545455	42,0,2,0	chr14,50793	Cytosol	A > G
chr14:50793679:A:G:ncRNA_intro	chr14	50793679	50793679	A	G	ncRNA_intro	RP11-247L2C	.	.	.	A	G	+	+-	0.12820513	34,0,5,0	chr14,50793	Cytosol	A > G
chr14:50793684:A:G:ncRNA_intro	chr14	50793684	50793684	A	G	ncRNA_intro	RP11-247L2C	.	.	.	A	G	+	+-	0.09302326	39,0,4,0	chr14,50793	Cytosol	A > G
chr14:50793697:T:C:ncRNA_intro	chr14	50793697	50793697	T	C	ncRNA_intro	RP11-247L2C	.	.	.	A	G	-	+-	0.35294118	11,0,6,0	chr14,50793	Cytosol	A > G
chr14:50793703:A:G:ncRNA_intro	chr14	50793703	50793703	A	G	ncRNA_intro	RP11-247L2C	.	.	.	A	G	+	+-	0.2	36,0,9,0	chr14,50793	Cytosol	A > G
chr14:50793821:A:G:ncRNA_intro	chr14	50793821	50793821	A	G	ncRNA_intro	RP11-247L2C	.	.	.	A	G	+	+-	0.14705882	29,0,5,0	chr14,50793	Cytosol	A > G
chr14:50793867:A:G:ncRNA_intro	chr14	50793867	50793867	A	G	ncRNA_intro	RP11-247L2C	.	.	.	A	G	+	+-	0.13953488	37,0,6,0	chr14,50793	Cytosol	A > G
chr14:50793901:A:G:ncRNA_intro	chr14	50793901	50793901	A	G	ncRNA_intro	RP11-247L2C	.	.	.	A	G	+	+-	0.11428571	31,0,4,0	chr14,50793	Cytosol	A > G
chr14:50793916:A:G:ncRNA_intro	chr14	50793916	50793916	A	G	ncRNA_intro	RP11-247L2C	.	.	.	A	G	+	+-	0.61538462	10,0,16,0	chr14,50793	Cytosol	A > G
chr14:50793931:T:C:ncRNA_intro	chr14	50793931	50793931	T	C	ncRNA_intro	RP11-247L2C	.	.	.	A	G	-	+-	1	0,0,8,0	chr14,50793	Cytosol	A > G
chr14:50793932:A:G:ncRNA_intro	chr14	50793932	50793932	A	G	ncRNA_intro	RP11-247L2C	.	.	.	A	G	+	+-	0.26923077	19,0,7,0	chr14,50793	Cytosol	A > G
chr14:50793939:A:G:ncRNA_intro	chr14	50793939	50793939	A	G	ncRNA_intro	RP11-247L2C	.	.	.	A	G	+	+-	0.0952381	19,0,2,0	chr14,50793	Cytosol	A > G
chr14:50793968:A:G:ncRNA_intro	chr14	50793968	50793968	A	G	ncRNA_intro	RP11-247L2C	.	.	.	A	G	+	+-	0.21052632	15,0,4,0	chr14,50793	Cytosol	A > G
chr14:50793996:T:C:ncRNA_intro	chr14	50793996	50793996	T	C	ncRNA_intro	RP11-247L2C	.	.	.	A	G	-	+-	0.3	7,0,3,0	chr14,50793	Cytosol	A > G

chr14:50794040:A:G:ncRNA_intro	chr14	50794040	50794040	A	G	ncRNA_intro	RP11-247L2	.	.	.	A	G	+	+-	0.45833333	13,0,11,0	chr14,50794	Cytosol	A > G
chr14:50794215:A:G:ncRNA_intro	chr14	50794215	50794215	A	G	ncRNA_intro	RP11-247L2	.	.	.	A	G	+	+-	0.33333333	4,0,2,0	chr14,50794	Cytosol	A > G
chr14:50794253:A:G:ncRNA_intro	chr14	50794253	50794253	A	G	ncRNA_intro	RP11-247L2	.	.	.	A	G	+	+-	0.4	9,0,6,0	chr14,50794	Cytosol	A > G
chr14:50794280:T:C:ncRNA_intror	chr14	50794280	50794280	T	C	ncRNA_intro	RP11-247L2	.	.	.	A	G	-	+-	0.28571429	5,0,2,0	chr14,50794	Cytosol	A > G
chr14:50794284:A:G:ncRNA_intro	chr14	50794284	50794284	A	G	ncRNA_intro	RP11-247L2	.	.	.	A	G	+	+-	0.12903226	27,0,4,0	chr14,50794	Cytosol	A > G
chr14:50794308:A:G:ncRNA_intro	chr14	50794308	50794308	A	G	ncRNA_intro	RP11-247L2	.	.	.	A	G	+	+-	0.3125	22,0,10,0	chr14,50794	Cytosol	A > G
chr14:50794320:T:C:ncRNA_intror	chr14	50794320	50794320	T	C	ncRNA_intro	RP11-247L2	.	.	.	A	G	-	+-	0.57142857	3,0,4,0	chr14,50794	Cytosol	A > G
chr14:50794322:A:G:ncRNA_intro	chr14	50794322	50794322	A	G	ncRNA_intro	RP11-247L2	.	.	.	A	G	+	+-	0.6	12,0,18,0	chr14,50794	Cytosol	A > G
chr14:50794363:A:G:ncRNA_intro	chr14	50794363	50794363	A	G	ncRNA_intro	RP11-247L2	.	.	.	A	G	+	+-	0.45454545	12,0,10,0	chr14,50794	Cytosol	A > G
chr14:50794366:A:G:ncRNA_intro	chr14	50794366	50794366	A	G	ncRNA_intro	RP11-247L2	.	.	.	A	G	+	+-	0.21052632	15,0,4,0	chr14,50794	Cytosol	A > G
chr14:50794380:A:G:ncRNA_intro	chr14	50794380	50794380	A	G	ncRNA_intro	RP11-247L2	.	.	.	A	G	+	+-	0.31818182	15,0,7,0	chr14,50794	Cytosol	A > G
chr14:50794383:A:G:ncRNA_intro	chr14	50794383	50794383	A	G	ncRNA_intro	RP11-247L2	.	.	.	A	G	+	+-	0.0952381	19,0,2,0	chr14,50794	Cytosol	A > G
chr14:50794392:A:G:ncRNA_intro	chr14	50794392	50794392	A	G	ncRNA_intro	RP11-247L2	.	.	.	A	G	+	+-	0.23809524	16,0,5,0	chr14,50794	Cytosol	A > G
chr14:50794424:A:G:ncRNA_intro	chr14	50794424	50794424	A	G	ncRNA_intro	RP11-247L2	.	.	.	A	G	+	+-	0.38888889	11,0,7,0	chr14,50794	Cytosol	A > G
chr14:51095532:T:C:intronic:ATL1	chr14	51095532	51095532	T	C	intronic	ATL1	.	.	.	A	G	-	+	0.6	2,0,3,0	chr14,51095	Cytosol	A > G
chr14:53242354:T:C:ncRNA_exoni	chr14	53242354	53242354	T	C	ncRNA_exon	RP11-589M4	.	.	.	A	G	-	-+	0.22507123	272,0,79,0	chr14,53242	Cytosol	A > G
chr14:53242417:T:C:ncRNA_exoni	chr14	53242417	53242417	T	C	ncRNA_exon	RP11-589M4	.	.	.	A	G	-	-+	0.23295455	270,0,82,0	chr14,53242	Cytosol	A > G
chr14:53242425:T:C:ncRNA_exoni	chr14	53242425	53242425	T	C	ncRNA_exon	RP11-589M4	.	.	.	A	G	-	-+	0.08490566	388,0,36,0	chr14,53242	Cytosol	A > G
chr14:53242430:T:C:ncRNA_exoni	chr14	53242430	53242430	T	C	ncRNA_exon	RP11-589M4	.	.	.	A	G	-	-+	0.02905569	401,0,12,0	chr14,53242	Cytosol	A > G
chr14:53242478:T:C:ncRNA_exoni	chr14	53242478	53242478	T	C	ncRNA_exon	RP11-589M4	.	.	.	A	G	-	-+	0.38690476	206,0,130,0	chr14,53242	Cytosol	A > G
chr14:53242488:T:C:ncRNA_exoni	chr14	53242488	53242488	T	C	ncRNA_exon	RP11-589M4	.	.	.	A	G	-	-+	0.12539185	279,0,40,0	chr14,53242	Cytosol	A > G
chr14:53242500:T:C:ncRNA_exoni	chr14	53242500	53242500	T	C	ncRNA_exon	RP11-589M4	.	.	.	A	G	-	-+	0.06024096	312,0,20,0	chr14,53242	Cytosol	A > G
chr14:53242518:T:C:ncRNA_exoni	chr14	53242518	53242518	T	C	ncRNA_exon	RP11-589M4	.	.	.	A	G	-	-+	0.10144928	310,0,35,0	chr14,53242	Cytosol	A > G
chr14:53242519:T:C:ncRNA_exoni	chr14	53242519	53242519	T	C	ncRNA_exon	RP11-589M4	.	.	.	A	G	-	-+	0.02228412	351,0,8,0	chr14,53242	Cytosol	A > G
chr14:53242568:T:C:ncRNA_exoni	chr14	53242568	53242568	T	C	ncRNA_exon	RP11-589M4	.	.	.	A	G	-	-+	0.07539683	233,0,19,0	chr14,53242	Cytosol	A > G
chr14:53242639:T:C:ncRNA_exoni	chr14	53242639	53242639	T	C	ncRNA_exon	RP11-589M4	.	.	.	A	G	-	+-	0.27272727	48,0,18,0	chr14,53242	Cytosol	A > G
chr14:53242651:T:C:ncRNA_exoni	chr14	53242651	53242651	T	C	ncRNA_exon	RP11-589M4	.	.	.	A	G	-	+-	0.3592233	66,0,37,0	chr14,53242	Cytosol	A > G
chr14:53242674:T:C:ncRNA_intror	chr14	53242674	53242674	T	C	ncRNA_intro	RP11-589M4	.	.	.	A	G	-	+-	0.04790419	159,0,8,0	chr14,53242	Cytosol	A > G
chr14:53242833:T:C:ncRNA_intror	chr14	53242833	53242833	T	C	ncRNA_intro	RP11-589M4	.	.	.	A	G	-	+-	0.01256281	393,0,5,0	chr14,53242	Cytosol	A > G
chr14:53242859:T:C:ncRNA_intror	chr14	53242859	53242859	T	C	ncRNA_intro	RP11-589M4	.	.	.	A	G	-	+-	0.09192825	405,0,41,0	chr14,53242	Cytosol	A > G
chr14:53243394:T:C:ncRNA_intror	chr14	53243394	53243394	T	C	ncRNA_intro	RP11-589M4	.	.	.	A	G	-	+-	0.64539007	50,0,91,0	chr14,53243	Cytosol	A > G
chr14:53243402:T:C:ncRNA_intror	chr14	53243402	53243402	T	C	ncRNA_intro	RP11-589M4	.	.	.	A	G	-	+-	0.01438849	137,0,2,0	chr14,53243	Cytosol	A > G
chr14:53243406:T:C:ncRNA_intror	chr14	53243406	53243406	T	C	ncRNA_intro	RP11-589M4	.	.	.	A	G	-	+-	0.11971831	125,0,17,0	chr14,53243	Cytosol	A > G
chr14:53243411:T:C:ncRNA_intror	chr14	53243411	53243411	T	C	ncRNA_intro	RP11-589M4	.	.	.	A	G	-	+-	0.32748538	115,0,56,0	chr14,53243	Cytosol	A > G
chr14:53243412:T:C:ncRNA_intror	chr14	53243412	53243412	T	C	ncRNA_intro	RP11-589M4	.	.	.	A	G	-	+-	0.12068966	153,0,21,0	chr14,53243	Cytosol	A > G
chr14:53243434:T:C:ncRNA_intror	chr14	53243434	53243434	T	C	ncRNA_intro	RP11-589M4	.	.	.	A	G	-	+-	0.06467662	188,0,13,0	chr14,53243	Cytosol	A > G
chr14:53243438:T:C:ncRNA_intror	chr14	53243438	53243438	T	C	ncRNA_intro	RP11-589M4	.	.	.	A	G	-	+-	0.16289593	185,0,36,0	chr14,53243	Cytosol	A > G
chr14:53243488:T:C:ncRNA_intror	chr14	53243488	53243488	T	C	ncRNA_intro	RP11-589M4	.	.	.	A	G	-	+-	0.09395973	135,0,14,0	chr14,53243	Cytosol	A > G
chr14:53243545:T:C:ncRNA_intror	chr14	53243545	53243545	T	C	ncRNA_intro	RP11-589M4	.	.	.	A	G	-	+-	0.40594059	60,0,41,0	chr14,53243	Cytosol	A > G
chr14:53243546:T:C:ncRNA_intror	chr14	53243546	53243546	T	C	ncRNA_intro	RP11-589M4	.	.	.	A	G	-	+-	0.19417476	83,0,20,0	chr14,53243	Cytosol	A > G
chr14:53243554:T:C:ncRNA_intror	chr14	53243554	53243554	T	C	ncRNA_intro	RP11-589M4	.	.	.	A	G	-	+-	0.34693878	64,0,34,0	chr14,53243	Cytosol	A > G
chr14:55527599:A:G:intronic:MAP	chr14	55527599	55527599	A	G	intronic	MAPK1IP1L	.	.	.	A	G	+	+	0.2	8,0,2,0	chr14,55527	Cytosol	A > G
chr14:55527600:A:G:intronic:MAP	chr14	55527600	55527600	A	G	intronic	MAPK1IP1L	.	.	.	A	G	+	+	0.25	6,0,2,0	chr14,55527	Cytosol	A > G
chr14:55527788:A:G:intronic:MAP	chr14	55527788	55527788	A	G	intronic	MAPK1IP1L	.	.	.	A	G	+	+	0.4	3,0,2,0	chr14,55527	Cytosol	A > G
chr14:55601792:A:G:intronic:LGAL	chr14	55601792	55601792	A	G	intronic	LGALS3	.	.	.	A	G	+	+	0.57142857	3,0,4,0	chr14,55601	Cytosol	A > G
chr14:55834204:T:C:UTR3:ATG14	chr14	55834204	55834204	T	C	UTR3	ATG14	ENST000002	.	.	A	G	-	+-	0.22580645	24,0,7,0	chr14,55834	Cytosol	A > G
chr14:55834210:T:C:UTR3:ATG14	chr14	55834210	55834210	T	C	UTR3	ATG14	ENST000002	.	.	A	G	-	+-	0.87096774	4,0,27,0	chr14,55834	Cytosol	A > G
chr14:55834216:T:C:UTR3:ATG14	chr14	55834216	55834216	T	C	UTR3	ATG14	ENST000002	.	.	A	G	-	+-	0.40740741	16,0,11,0	chr14,55834	Cytosol	A > G
chr14:55834217:T:C:UTR3:ATG14	chr14	55834217	55834217	T	C	UTR3	ATG14	ENST000002	.	.	A	G	-	+-	0.33333333	20,0,10,0	chr14,55834	Cytosol	A > G
chr14:55834296:T:C:UTR3:ATG14	chr14	55834296	55834296	T	C	UTR3	ATG14	ENST000002	.	.	A	G	-	+-	0.8	4,0,16,0	chr14,55834	Cytosol	A > G
chr14:55834300:T:C:UTR3:ATG14	chr14	55834300	55834300	T	C	UTR3	ATG14	ENST000002	.	.	A	G	-	+-	0.13043478	20,0,3,0	chr14,55834	Cytosol	A > G
chr14:55834367:T:C:UTR3:ATG14	chr14	55834367	55834367	T	C	UTR3	ATG14	ENST000002	.	.	A	G	-	+-	0.05714286	33,0,2,0	chr14,55834	Cytosol	A > G
chr14:55834376:T:C:UTR3:ATG14	chr14	55834376	55834376	T	C	UTR3	ATG14	ENST000002	.	.	A	G	-	+-	0.29411765	24,0,10,0	chr14,55834	Cytosol	A > G
chr14:55834379:T:C:UTR3:ATG14	chr14	55834379	55834379	T	C	UTR3	ATG14	ENST000002	.	.	A	G	-	+-	0.12121212	29,0,4,0	chr14,55834	Cytosol	A > G
chr14:55834418:T:C:UTR3:ATG14	chr14	55834418	55834418	T	C	UTR3	ATG14	ENST000002	.	.	A	G	-	+-	0.28205128	28,0,11,0	chr14,55834	Cytosol	A > G
chr14:55834424:T:C:UTR3:ATG14	chr14	55834424	55834424	T	C	UTR3	ATG14	ENST000002	.	.	A	G	-	+-	0.05405405	35,0,2,0	chr14,55834	Cytosol	A > G
chr14:55834448:T:C:UTR3:ATG14	chr14	55834448	55834448	T	C	UTR3	ATG14	ENST000002	.	.	A	G	-	+-	0.48	13,0,12,0	chr14,55834	Cytosol	A > G
chr14:55834965:T:C:UTR3:ATG14	chr14	55834965	55834965	T	C	UTR3	ATG14	ENST000002	.	.	A	G	-	+-	0.10714286	50,0,6,0	chr14,55834	Cytosol	A > G
chr14:55834966:T:C:UTR3:ATG14	chr14	55834966	55834966	T	C	UTR3	ATG14	ENST000002	.	.	A	G	-	+-	0.23636364	42,0,13,0	chr14,55834	Cytosol	A > G
chr14:55835024:T:C:UTR3:ATG14	chr14	55835024	55835024	T	C	UTR3	ATG14	ENST000002	.	.	A	G	-	+-	0.14285714	12,0,2,0	chr14,55835	Cytosol	A > G
chr14:55835120:T:C:UTR3:ATG14	chr14	55835120	55835120	T	C	UTR3	ATG14	ENST000002	.	.	A	G	-	+-	0.36363636	7,0,4,0	chr14,55835	Cytosol	A > G
chr14:55835184:T:C:UTR3:ATG14	chr14	55835184	55835184	T	C	UTR3	ATG14	ENST000002	.	.	A	G	-	+-	0.23529412	13,0,4,0	chr14,55835	Cytosol	A > G
chr14:55835197:T:C:UTR3:ATG14	chr14	55835197	55835197	T	C	UTR3	ATG14	ENST000002	.	.	A	G	-	+-	0.58333333	10,0,14,0	chr14,55835	Cytosol	A > G
chr14:55835205:T:C:UTR3:ATG14	chr14	55835205	55835205	T	C	UTR3	ATG14	ENST000002	.	.	A	G	-	+-	0.34782609	15,0,8,0	chr14,55835	Cytosol	A > G

chr14:55835233:T:C:UTR3:ATG14	chr14	55835233	55835233	T	C	UTR3	ATG14	ENST000002	.	.	A	G	-	+-	0.41176471	10,0,7,0	chr14,55835	Cytosol	A > G
chr14:62262612:A:G:UTR3:SNAPC	chr14	62262612	62262612	A	G	UTR3	SNAPC1	ENST000002	.	.	A	G	+	+	0.9	1,0,9,0	chr14,62262	Cytosol	A > G
chr14:64948936:T:C:UTR3:ZBTB25	chr14	64948936	64948936	T	C	UTR3	ZBTB25	ENST0000006	.	.	A	G	-	-	0.1875	13,0,3,0	chr14,64948	Cytosol	A > G
chr14:64948977:T:C:UTR3:ZBTB25	chr14	64948977	64948977	T	C	UTR3	ZBTB25	ENST0000006	.	.	A	G	-	-	0.25	12,0,4,0	chr14,64948	Cytosol	A > G
chr14:64950170:T:C:UTR3:ZBTB25	chr14	64950170	64950170	T	C	UTR3	ZBTB25	ENST0000006	.	.	A	G	-	-	0.33333333	6,0,3,0	chr14,64950	Cytosol	A > G
chr14:64950172:T:C:UTR3:ZBTB25	chr14	64950172	64950172	T	C	UTR3	ZBTB25	ENST0000006	.	.	A	G	-	-	0.28571429	5,0,2,0	chr14,64950	Cytosol	A > G
chr14:64950173:T:C:UTR3:ZBTB25	chr14	64950173	64950173	T	C	UTR3	ZBTB25	ENST0000006	.	.	A	G	-	-	0.66666667	2,0,4,0	chr14,64950	Cytosol	A > G
chr14:64950174:T:C:UTR3:ZBTB25	chr14	64950174	64950174	T	C	UTR3	ZBTB25	ENST0000006	.	.	A	G	-	-	0.4	3,0,2,0	chr14,64950	Cytosol	A > G
chr14:64950176:T:C:UTR3:ZBTB25	chr14	64950176	64950176	T	C	UTR3	ZBTB25	ENST0000006	.	.	A	G	-	-	0.33333333	4,0,2,0	chr14,64950	Cytosol	A > G
chr14:64950196:T:C:UTR3:ZBTB25	chr14	64950196	64950196	T	C	UTR3	ZBTB25	ENST0000006	.	.	A	G	-	-	0.66666667	2,0,4,0	chr14,64950	Cytosol	A > G
chr14:64950221:T:C:UTR3:ZBTB25	chr14	64950221	64950221	T	C	UTR3	ZBTB25	ENST0000006	.	.	A	G	-	-	0.57142857	3,0,4,0	chr14,64950	Cytosol	A > G
chr14:64950224:T:C:UTR3:ZBTB25	chr14	64950224	64950224	T	C	UTR3	ZBTB25	ENST0000006	.	.	A	G	-	-	0.66666667	2,0,4,0	chr14,64950	Cytosol	A > G
chr14:64950461:T:C:UTR3:ZBTB25	chr14	64950461	64950461	T	C	UTR3	ZBTB25	ENST0000006	.	.	A	G	-	-	0.42857143	8,0,6,0	chr14,64950	Cytosol	A > G
chr14:64950495:T:C:UTR3:ZBTB25	chr14	64950495	64950495	T	C	UTR3	ZBTB25	ENST0000006	.	.	A	G	-	-	0.38461538	8,0,5,0	chr14,64950	Cytosol	A > G
chr14:64950506:T:C:UTR3:ZBTB25	chr14	64950506	64950506	T	C	UTR3	ZBTB25	ENST0000006	.	.	A	G	-	-	0.66666667	2,0,4,0	chr14,64950	Cytosol	A > G
chr14:64951277:T:C:UTR3:ZBTB25	chr14	64951277	64951277	T	C	UTR3	ZBTB25	ENST0000006	.	.	A	G	-	-	0.11111111	16,0,2,0	chr14,64951	Cytosol	A > G
chr14:64993719:A:G:ncRNA_intro	chr14	64993719	64993719	A	G	ncRNA_intro	RP11-973N1	.	.	.	A	G	+	-+	0.4	6,0,4,0	chr14,64993	Cytosol	A > G
chr14:67804220:T:C:UTR3:ATP6V1	chr14	67804220	67804220	T	C	UTR3	ATP6V1D	ENST000005	.	.	A	G	-	-	0.22222222	7,0,2,0	chr14,67804	Cytosol	A > G
chr14:70352471:C:T:intronic:SMO	chr14	70352471	70352471	C	T	intronic	SMOC1	.	.	.	G	A	-	+-	1	7,0,0,0	chr14,70352	Cytosol	G > A
chr14:74165276:A:G:ncRNA_exon	chr14	74165276	74165276	A	G	ncRNA_exon	RP4-693M11	.	.	.	A	G	+	+-	0.66666667	3,0,6,0	chr14,74165	Cytosol	A > G
chr14:74165327:A:G:ncRNA_intro	chr14	74165327	74165327	A	G	ncRNA_intro	RP4-693M11	.	.	.	A	G	+	+-	0.625	3,0,5,0	chr14,74165	Cytosol	A > G
chr14:74165330:A:G:ncRNA_intro	chr14	74165330	74165330	A	G	ncRNA_intro	RP4-693M11	.	.	.	A	G	+	+-	0.85714286	1,0,6,0	chr14,74165	Cytosol	A > G
chr14:74166845:A:G:ncRNA_intro	chr14	74166845	74166845	A	G	ncRNA_intro	RP4-693M11	.	.	.	A	G	+	+-	0.625	3,0,5,0	chr14,74166	Cytosol	A > G
chr14:74166927:A:G:ncRNA_exon	chr14	74166927	74166927	A	G	ncRNA_exon	RP4-693M11	.	.	.	A	G	+	+-	0.25	6,0,2,0	chr14,74166	Cytosol	A > G
chr14:74166935:A:G:ncRNA_exon	chr14	74166935	74166935	A	G	ncRNA_exon	RP4-693M11	.	.	.	A	G	+	+-	0.42857143	4,0,3,0	chr14,74166	Cytosol	A > G
chr14:74166936:A:G:ncRNA_exon	chr14	74166936	74166936	A	G	ncRNA_exon	RP4-693M11	.	.	.	A	G	+	+-	0.375	5,0,3,0	chr14,74166	Cytosol	A > G
chr14:74524774:T:C:ncRNA_intro	chr14	74524774	74524774	T	C	ncRNA_intro	AC005484.5	.	.	.	A	G	-	+-	0.45454545	6,0,5,0	chr14,74524	Cytosol	A > G
chr14:74524787:T:C:ncRNA_intro	chr14	74524787	74524787	T	C	ncRNA_intro	AC005484.5	.	.	.	A	G	-	+-	0.27272727	8,0,3,0	chr14,74524	Cytosol	A > G
chr14:74524944:T:C:ncRNA_intro	chr14	74524944	74524944	T	C	ncRNA_intro	AC005484.5	.	.	.	A	G	-	+-	0.4	12,0,8,0	chr14,74524	Cytosol	A > G
chr14:74524947:T:C:ncRNA_intro	chr14	74524947	74524947	T	C	ncRNA_intro	AC005484.5	.	.	.	A	G	-	+-	0.21052632	15,0,4,0	chr14,74524	Cytosol	A > G
chr14:74525544:T:C:ncRNA_intro	chr14	74525544	74525544	T	C	ncRNA_intro	AC005484.5	.	.	.	A	G	-	+-	0.13636364	19,0,3,0	chr14,74525	Cytosol	A > G
chr14:74526142:T:C:ncRNA_intro	chr14	74526142	74526142	T	C	ncRNA_intro	AC005484.5	.	.	.	A	G	-	+-	0.2	8,0,2,0	chr14,74526	Cytosol	A > G
chr14:74526154:T:C:ncRNA_intro	chr14	74526154	74526154	T	C	ncRNA_intro	AC005484.5	.	.	.	A	G	-	+-	0.25	6,0,2,0	chr14,74526	Cytosol	A > G
chr14:74526976:T:C:ncRNA_intro	chr14	74526976	74526976	T	C	ncRNA_intro	AC005484.5	.	.	.	A	G	-	+-	0.4	24,0,16,0	chr14,74526	Cytosol	A > G
chr14:75202288:C:A:UTR3:FCF1	chr14	75202288	75202288	C	A	UTR3	FCF1	ENST000003	.	.	C	A	+	+	0.03076923	2,63,0,0	chr14,75202	Cytosol	C > A
chr14:75202312:A:G:UTR3:FCF1	chr14	75202312	75202312	A	G	UTR3	FCF1	ENST000003	.	.	A	G	+	+	0.13513514	95,0,15,1	chr14,75202	Cytosol	A > G
chr14:75202372:A:G:UTR3:FCF1	chr14	75202372	75202372	A	G	UTR3	FCF1	ENST000003	.	.	A	G	+	+	0.28977273	125,0,51,0	chr14,75202	Cytosol	A > G
chr14:75202390:A:G:UTR3:FCF1	chr14	75202390	75202390	A	G	UTR3	FCF1	ENST000003	.	.	A	G	+	+	0.11111111	104,0,13,0	chr14,75202	Cytosol	A > G
chr14:75202402:A:G:UTR3:FCF1	chr14	75202402	75202402	A	G	UTR3	FCF1	ENST000003	.	.	A	G	+	+	0.86915888	14,0,93,0	chr14,75202	Cytosol	A > G
chr14:75202412:A:G:UTR3:FCF1	chr14	75202412	75202412	A	G	UTR3	FCF1	ENST000003	.	.	A	G	+	+	0.47445255	72,0,65,0	chr14,75202	Cytosol	A > G
chr14:75202438:A:G:UTR3:FCF1	chr14	75202438	75202438	A	G	UTR3	FCF1	ENST000003	.	.	A	G	+	+	0.0173913	113,0,2,0	chr14,75202	Cytosol	A > G
chr14:75202475:A:G:UTR3:FCF1	chr14	75202475	75202475	A	G	UTR3	FCF1	ENST000003	.	.	A	G	+	+	0.06428571	131,0,9,0	chr14,75202	Cytosol	A > G
chr14:75202531:A:G:UTR3:FCF1	chr14	75202531	75202531	A	G	UTR3	FCF1	ENST000003	.	.	A	G	+	+	0.05847953	161,0,10,0	chr14,75202	Cytosol	A > G
chr14:75202532:A:G:UTR3:FCF1	chr14	75202532	75202532	A	G	UTR3	FCF1	ENST000003	.	.	A	G	+	+	0.23529412	130,0,40,0	chr14,75202	Cytosol	A > G
chr14:75202540:A:G:UTR3:FCF1	chr14	75202540	75202540	A	G	UTR3	FCF1	ENST000003	.	.	A	G	+	+	0.01408451	140,0,2,0	chr14,75202	Cytosol	A > G
chr14:75202544:A:G:UTR3:FCF1	chr14	75202544	75202544	A	G	UTR3	FCF1	ENST000003	.	.	A	G	+	+	0.29104478	94,0,39,1	chr14,75202	Cytosol	A > G
chr14:75202553:A:G:UTR3:FCF1	chr14	75202553	75202553	A	G	UTR3	FCF1	ENST000003	.	.	A	G	+	+	0.048	119,0,6,0	chr14,75202	Cytosol	A > G
chr14:75202705:A:G:UTR3:FCF1	chr14	75202705	75202705	A	G	UTR3	FCF1	ENST000003	.	.	A	G	+	+	0.15151515	28,0,5,0	chr14,75202	Cytosol	A > G
chr14:75202722:A:G:UTR3:FCF1	chr14	75202722	75202722	A	G	UTR3	FCF1	ENST000003	.	.	A	G	+	+	0.03529412	82,0,3,0	chr14,75202	Cytosol	A > G
chr14:75202734:A:G:UTR3:FCF1	chr14	75202734	75202734	A	G	UTR3	FCF1	ENST000003	.	.	A	G	+	+	0.04385965	109,0,5,0	chr14,75202	Cytosol	A > G
chr14:75202834:A:G:UTR3:FCF1	chr14	75202834	75202834	A	G	UTR3	FCF1	ENST000003	.	.	A	G	+	+	0.02380952	82,0,2,0	chr14,75202	Cytosol	A > G
chr14:75202848:A:G:UTR3:FCF1	chr14	75202848	75202848	A	G	UTR3	FCF1	ENST000003	.	.	A	G	+	+	0.02222222	88,0,2,0	chr14,75202	Cytosol	A > G
chr14:75202856:A:G:UTR3:FCF1	chr14	75202856	75202856	A	G	UTR3	FCF1	ENST000003	.	.	A	G	+	+	0.37190083	76,0,45,0	chr14,75202	Cytosol	A > G
chr14:75202857:A:G:UTR3:FCF1	chr14	75202857	75202857	A	G	UTR3	FCF1	ENST000003	.	.	A	G	+	+	0.29464286	79,0,33,0	chr14,75202	Cytosol	A > G
chr14:75202952:A:G:UTR3:FCF1	chr14	75202952	75202952	A	G	UTR3	FCF1	ENST000003	.	.	A	G	+	+	0.03825137	176,0,7,0	chr14,75202	Cytosol	A > G
chr14:75202953:A:G:UTR3:FCF1	chr14	75202953	75202953	A	G	UTR3	FCF1	ENST000003	.	.	A	G	+	+	0.01604278	184,0,3,0	chr14,75202	Cytosol	A > G
chr14:75202963:A:G:UTR3:FCF1	chr14	75202963	75202963	A	G	UTR3	FCF1	ENST000003	.	.	A	G	+	+	0.03278689	177,0,6,0	chr14,75202	Cytosol	A > G
chr14:75202964:A:G:UTR3:FCF1	chr14	75202964	75202964	A	G	UTR3	FCF1	ENST000003	.	.	A	G	+	+	0.05586592	169,0,10,0	chr14,75202	Cytosol	A > G
chr14:75202965:A:G:UTR3:FCF1	chr14	75202965	75202965	A	G	UTR3	FCF1	ENST000003	.	.	A	G	+	+	0.03389831	171,0,6,0	chr14,75202	Cytosol	A > G
chr14:75202994:A:G:UTR3:FCF1	chr14	75202994	75202994	A	G	UTR3	FCF1	ENST000003	.	.	A	G	+	+	0.58585859	41,0,58,0	chr14,75202	Cytosol	A > G
chr14:75202995:A:G:UTR3:FCF1	chr14	75202995	75202995	A	G	UTR3	FCF1	ENST000003	.	.	A	G	+	+	0.43269231	59,0,45,0	chr14,75202	Cytosol	A > G
chr14:75203000:A:G:UTR3:FCF1	chr14	75203000	75203000	A	G	UTR3	FCF1	ENST000003	.	.	A	G	+	+	0.23469388	75,0,23,0	chr14,75202	Cytosol	A > G
chr14:75203004:A:G:UTR3:FCF1	chr14	75203004	75203004	A	G	UTR3	FCF1	ENST000003	.	.	A	G	+	+	0.81707317	15,0,67,0	chr14,75203	Cytosol	A > G
chr14:75203056:A:G:UTR3:FCF1	chr14	75203056	75203056	A	G	UTR3	FCF1	ENST000003	.	.	A	G	+	+	0.28571429	10,0,4,0	chr14,75203	Cytosol	A > G

chr14:75203067:A:G:UTR3:FCF1	chr14	75203067	75203067	A	G	UTR3	FCF1	ENST000003	.	.	A	G	+	+	0.5	10,0,10,0	chr14,75203	Cytosol	A > G
chr14:75203097:A:G:UTR3:FCF1	chr14	75203097	75203097	A	G	UTR3	FCF1	ENST000003	.	.	A	G	+	+	0.14634146	35,0,6,0	chr14,75203	Cytosol	A > G
chr14:90261928:T:C:ncRNA_intror	chr14	90261928	90261928	T	C	ncRNA_intro	RP11-33N16.	.	.	.	A	G	-	-	0.85714286	1,0,6,0	chr14,90261	Cytosol	A > G
chr14:90261974:T:C:ncRNA_intror	chr14	90261974	90261974	T	C	ncRNA_intro	RP11-33N16.	.	.	.	A	G	-	-	1	0,0,5,0	chr14,90261	Cytosol	A > G
chr14:90261996:T:C:ncRNA_intror	chr14	90261996	90261996	T	C	ncRNA_intro	RP11-33N16.	.	.	.	A	G	-	-	0.85714286	1,0,6,0	chr14,90261	Cytosol	A > G
chr14:100762714:T:C:UTR5:SLC25	chr14	100762714	100762714	T	C	UTR5	SLC25A29	ENST000005	.	.	A	G	-	-	0.42857143	4,0,3,0	chr14,10076	Cytosol	A > G
chr14:102807154:A:G:intronic:ZNF	chr14	102807154	102807154	A	G	intronic	ZNF839	.	.	.	A	G	+	+	0.6	2,0,3,0	chr14,10280	Cytosol	A > G
chr14:102811991:T:C:intronic:CINI	chr14	102811991	102811991	T	C	intronic	CINP	.	.	.	A	G	-	-	0.18181818	9,0,2,0	chr14,10281	Cytosol	A > G
chr14:102812062:T:C:intronic:CINI	chr14	102812062	102812062	T	C	intronic	CINP	.	.	.	A	G	-	-	0.44444444	5,0,4,0	chr14,10281	Cytosol	A > G
chr14:102812097:T:C:intronic:CINI	chr14	102812097	102812097	T	C	intronic	CINP	.	.	.	A	G	-	-	0.22222222	7,0,2,0	chr14,10281	Cytosol	A > G
chr14:102812259:T:C:intronic:CINI	chr14	102812259	102812259	T	C	intronic	CINP	.	.	.	A	G	-	-	0.4	3,0,2,0	chr14,10281	Cytosol	A > G
chr14:102812266:T:C:intronic:CINI	chr14	102812266	102812266	T	C	intronic	CINP	.	.	.	A	G	-	-	0.22222222	7,0,2,0	chr14,10281	Cytosol	A > G
chr14:102812353:T:C:intronic:CINI	chr14	102812353	102812353	T	C	intronic	CINP	.	.	.	A	G	-	-	0.5	5,0,5,0	chr14,10281	Cytosol	A > G
chr14:102812422:T:C:intronic:CINI	chr14	102812422	102812422	T	C	intronic	CINP	.	.	.	A	G	-	-	0.4	3,0,2,0	chr14,10281	Cytosol	A > G
chr14:102813118:T:C:intronic:CINI	chr14	102813118	102813118	T	C	intronic	CINP	.	.	.	A	G	-	-	0.83333333	1,0,5,0	chr14,10281	Cytosol	A > G
chr14:102813295:T:C:intronic:CINI	chr14	102813295	102813295	T	C	intronic	CINP	.	.	.	A	G	-	-	0.35714286	9,0,5,0	chr14,10281	Cytosol	A > G
chr14:102813316:T:C:intronic:CINI	chr14	102813316	102813316	T	C	intronic	CINP	.	.	.	A	G	-	-	0.46153846	7,0,6,0	chr14,10281	Cytosol	A > G
chr14:102813669:T:C:intronic:CINI	chr14	102813669	102813669	T	C	intronic	CINP	.	.	.	A	G	-	-	0.76923077	3,0,10,0	chr14,10281	Cytosol	A > G
chr14:102813676:T:C:intronic:CINI	chr14	102813676	102813676	T	C	intronic	CINP	.	.	.	A	G	-	-	0.14285714	12,0,2,0	chr14,10281	Cytosol	A > G
chr14:102813770:T:C:intronic:CINI	chr14	102813770	102813770	T	C	intronic	CINP	.	.	.	A	G	-	-	0.81818182	2,0,9,0	chr14,10281	Cytosol	A > G
chr14:102813796:T:C:intronic:CINI	chr14	102813796	102813796	T	C	intronic	CINP	.	.	.	A	G	-	-	0.5	7,0,7,0	chr14,10281	Cytosol	A > G
chr14:102813806:T:C:intronic:CINI	chr14	102813806	102813806	T	C	intronic	CINP	.	.	.	A	G	-	-	0.57894737	8,0,11,0	chr14,10281	Cytosol	A > G
chr14:102813836:T:C:intronic:CINI	chr14	102813836	102813836	T	C	intronic	CINP	.	.	.	A	G	-	-	0.5	4,0,4,0	chr14,10281	Cytosol	A > G
chr14:102814292:T:C:intronic:CINI	chr14	102814292	102814292	T	C	intronic	CINP	.	.	.	A	G	-	-	0.59375	13,0,19,0	chr14,10281	Cytosol	A > G
chr14:102814387:T:C:intronic:CINI	chr14	102814387	102814387	T	C	intronic	CINP	.	.	.	A	G	-	-	0.33333333	6,0,3,0	chr14,10281	Cytosol	A > G
chr14:105837171:A:G:intronic:PA(	chr14	105837171	105837171	A	G	intronic	PACS2	.	.	.	A	G	+	+	0.5	3,0,3,0	chr14,10583	Cytosol	A > G
chr14:105921342:A:G:intronic:MT	chr14	105921342	105921342	A	G	intronic	MTA1	.	.	.	A	G	+	+	0.14285714	12,0,2,0	chr14,10592	Cytosol	A > G
chr14:105921432:A:G:intronic:MT	chr14	105921432	105921432	A	G	intronic	MTA1	.	.	.	A	G	+	+	0.16666667	15,0,3,0	chr14,10592	Cytosol	A > G
chr14:105921463:A:G:intronic:MT	chr14	105921463	105921463	A	G	intronic	MTA1	.	.	.	A	G	+	+	0.35714286	9,0,5,0	chr14,10592	Cytosol	A > G
chr15:25230735:A:G:ncRNA_intro	chr15	25230735	25230735	A	G	ncRNA_intro	SNHG14	.	.	.	A	G	+	+	0.28571429	5,0,2,0	chr15,25230	Cytosol	A > G
chr15:34396594:A:G:downstream:	chr15	34396594	34396594	A	G	downstream	PGBD4	.	.	.	A	G	+		0.6	2,0,3,0	chr15,34396	Cytosol	A > G
chr15:34396618:A:G:downstream:	chr15	34396618	34396618	A	G	downstream	PGBD4	.	.	.	A	G	+		0.6	2,0,3,0	chr15,34396	Cytosol	A > G
chr15:34523297:T:C:downstream:	chr15	34523297	34523297	T	C	downstream	EMC4	.	.	.	A	G	-	-	0.64705882	6,0,11,0	chr15,34523	Cytosol	A > G
chr15:34523309:T:C:downstream:	chr15	34523309	34523309	T	C	downstream	EMC4	.	.	.	A	G	-	-	0.58333333	5,0,7,0	chr15,34523	Cytosol	A > G
chr15:34523310:T:C:downstream:	chr15	34523310	34523310	T	C	downstream	EMC4	.	.	.	A	G	-	-	0.53846154	6,0,7,0	chr15,34523	Cytosol	A > G
chr15:34523343:T:C:downstream:	chr15	34523343	34523343	T	C	downstream	EMC4	.	.	.	A	G	-	-	0.15384615	11,0,2,0	chr15,34523	Cytosol	A > G
chr15:34523438:T:C:intergenic:EM	chr15	34523438	34523438	T	C	intergenic	EMC4,SLC12	dist=1081;dis	.	.	A	G	-	-	0.82352941	3,0,14,0	chr15,34523	Cytosol	A > G
chr15:34523480:T:C:intergenic:EM	chr15	34523480	34523480	T	C	intergenic	EMC4,SLC12	dist=1123;dis	.	.	A	G	-	-	0.47826087	12,0,11,0	chr15,34523	Cytosol	A > G
chr15:34523481:T:C:intergenic:EM	chr15	34523481	34523481	T	C	intergenic	EMC4,SLC12	dist=1124;dis	.	.	A	G	-	-	0.18181818	18,0,4,0	chr15,34523	Cytosol	A > G
chr15:34523519:T:C:intergenic:EM	chr15	34523519	34523519	T	C	intergenic	EMC4,SLC12	dist=1162;dis	.	.	A	G	-	-	0.18181818	18,0,4,0	chr15,34523	Cytosol	A > G
chr15:34523556:T:C:intergenic:EM	chr15	34523556	34523556	T	C	intergenic	EMC4,SLC12	dist=1199;dis	.	.	A	G	-	-	0.27272727	16,0,6,0	chr15,34523	Cytosol	A > G
chr15:34523851:T:C:intergenic:EM	chr15	34523851	34523851	T	C	intergenic	EMC4,SLC12	dist=1494;dis	.	.	A	G	-	-	0.57142857	6,0,8,0	chr15,34523	Cytosol	A > G
chr15:34523867:T:C:intergenic:EM	chr15	34523867	34523867	T	C	intergenic	EMC4,SLC12	dist=1510;dis	.	.	A	G	-	-	0.28571429	5,0,2,0	chr15,34523	Cytosol	A > G
chr15:34523929:T:C:intergenic:EM	chr15	34523929	34523929	T	C	intergenic	EMC4,SLC12	dist=1572;dis	.	.	A	G	-	-	0.66666667	5,0,10,0	chr15,34523	Cytosol	A > G
chr15:34523955:T:C:intergenic:EM	chr15	34523955	34523955	T	C	intergenic	EMC4,SLC12	dist=1598;dis	.	.	A	G	-	-	0.2	12,0,3,0	chr15,34523	Cytosol	A > G
chr15:34524545:T:C:downstream:	chr15	34524545	34524545	T	C	downstream	SLC12A6	.	.	.	A	G	-	-	0.1	18,0,2,0	chr15,34524	Cytosol	A > G
chr15:34524561:T:C:downstream:	chr15	34524561	34524561	T	C	downstream	SLC12A6	.	.	.	A	G	-	-	0.13636364	19,0,3,0	chr15,34524	Cytosol	A > G
chr15:34524593:T:C:downstream:	chr15	34524593	34524593	T	C	downstream	SLC12A6	.	.	.	A	G	-	-	0.23809524	16,0,5,0	chr15,34524	Cytosol	A > G
chr15:34524656:T:C:downstream:	chr15	34524656	34524656	T	C	downstream	SLC12A6	.	.	.	A	G	-	-	0.41666667	7,0,5,0	chr15,34524	Cytosol	A > G
chr15:34524708:T:C:downstream:	chr15	34524708	34524708	T	C	downstream	SLC12A6	.	.	.	A	G	-	-	0.58333333	5,0,7,0	chr15,34524	Cytosol	A > G
chr15:40490236:A:G:intronic:BUB	chr15	40490236	40490236	A	G	intronic	BUB1B	.	.	.	A	G	+	+	0.57142857	3,0,4,0	chr15,40490	Cytosol	A > G
chr15:40686798:A:G:downstream:	chr15	40686798	40686798	A	G	downstream	KNSTRN	.	.	.	A	G	+		0.57142857	3,0,4,0	chr15,40686	Cytosol	A > G
chr15:40686844:A:G:downstream:	chr15	40686844	40686844	A	G	downstream	KNSTRN	.	.	.	A	G	+		0.35714286	9,0,5,0	chr15,40686	Cytosol	A > G
chr15:40687884:A:G:intergenic:KN	chr15	40687884	40687884	A	G	intergenic	KNSTRN,IVD	dist=1437;dis	.	.	A	G	+		0.65625	11,0,21,0	chr15,40687	Cytosol	A > G
chr15:40822246:T:C:ncRNA_intror	chr15	40822246	40822246	T	C	ncRNA_intro	RP11-111A2	.	.	.	A	G	-	+-	0.45454545	6,0,5,0	chr15,40822	Cytosol	A > G
chr15:40954956:A:G:UTR3:CASC5	chr15	40954956	40954956	A	G	UTR3	CASC5	ENST000003	.	.	A	G	+	+	0.35294118	22,0,12,0	chr15,40954	Cytosol	A > G
chr15:40954972:A:G:UTR3:CASC5	chr15	40954972	40954972	A	G	UTR3	CASC5	ENST000003	.	.	A	G	+	+	0.06666667	28,0,2,0	chr15,40954	Cytosol	A > G
chr15:40954979:A:G:UTR3:CASC5	chr15	40954979	40954979	A	G	UTR3	CASC5	ENST000003	.	.	A	G	+	+	0.05263158	36,0,2,0	chr15,40954	Cytosol	A > G
chr15:40954980:A:G:UTR3:CASC5	chr15	40954980	40954980	A	G	UTR3	CASC5	ENST000003	.	.	A	G	+	+	0.10810811	33,0,4,0	chr15,40954	Cytosol	A > G
chr15:40954982:A:G:UTR3:CASC5	chr15	40954982	40954982	A	G	UTR3	CASC5	ENST000003	.	.	A	G	+	+	0.14285714	30,0,5,0	chr15,40954	Cytosol	A > G
chr15:40954993:A:G:UTR3:CASC5	chr15	40954993	40954993	A	G	UTR3	CASC5	ENST000003	.	.	A	G	+	+	0.26315789	28,0,10,0	chr15,40954	Cytosol	A > G
chr15:40954998:A:G:UTR3:CASC5	chr15	40954998	40954998	A	G	UTR3	CASC5	ENST000003	.	.	A	G	+	+	0.15789474	32,0,6,0	chr15,40954	Cytosol	A > G
chr15:40954999:A:G:UTR3:CASC5	chr15	40954999	40954999	A	G	UTR3	CASC5	ENST000003	.	.	A	G	+	+	0.08	46,0,4,0	chr15,40954	Cytosol	A > G
chr15:40955011:A:G:UTR3:CASC5	chr15	40955011	40955011	A	G	UTR3	CASC5	ENST000003	.	.	A	G	+	+	0.14	43,0,7,0	chr15,40955	Cytosol	A > G

chr15:40955060:A:G:UTR3:CASC5	chr15	40955060	40955060	A	G	UTR3	CASC5	ENST000003	.	.	A	G	+	+	0.14285714	42,0,7,0	chr15,40955	Cytosol	A > G
chr15:40955064:A:G:UTR3:CASC5	chr15	40955064	40955064	A	G	UTR3	CASC5	ENST000003	.	.	A	G	+	+	0.10526316	34,0,4,0	chr15,40955	Cytosol	A > G
chr15:40955090:A:G:UTR3:CASC5	chr15	40955090	40955090	A	G	UTR3	CASC5	ENST000003	.	.	A	G	+	+	0.84444444	7,0,38,0	chr15,40955	Cytosol	A > G
chr15:40955102:A:G:UTR3:CASC5	chr15	40955102	40955102	A	G	UTR3	CASC5	ENST000003	.	.	A	G	+	+	0.97297297	1,0,36,0	chr15,40955	Cytosol	A > G
chr15:40955103:A:G:UTR3:CASC5	chr15	40955103	40955103	A	G	UTR3	CASC5	ENST000003	.	.	A	G	+	+	0.5	19,0,19,0	chr15,40955	Cytosol	A > G
chr15:40955108:A:G:UTR3:CASC5	chr15	40955108	40955108	A	G	UTR3	CASC5	ENST000003	.	.	A	G	+	+	0.23255814	33,0,10,0	chr15,40955	Cytosol	A > G
chr15:40955133:A:G:UTR3:CASC5	chr15	40955133	40955133	A	G	UTR3	CASC5	ENST000003	.	.	A	G	+	+	0.86486486	5,0,32,0	chr15,40955	Cytosol	A > G
chr15:40955146:A:G:UTR3:CASC5	chr15	40955146	40955146	A	G	UTR3	CASC5	ENST000003	.	.	A	G	+	+	0.07692308	24,0,2,0	chr15,40955	Cytosol	A > G
chr15:40955375:A:G:UTR3:CASC5	chr15	40955375	40955375	A	G	UTR3	CASC5	ENST000003	.	.	A	G	+	+	0.7	6,0,14,0	chr15,40955	Cytosol	A > G
chr15:40955439:A:G:UTR3:CASC5	chr15	40955439	40955439	A	G	UTR3	CASC5	ENST000003	.	.	A	G	+	+	0.38888889	11,0,7,0	chr15,40955	Cytosol	A > G
chr15:40955445:A:G:UTR3:CASC5	chr15	40955445	40955445	A	G	UTR3	CASC5	ENST000003	.	.	A	G	+	+	0.13333333	13,0,2,0	chr15,40955	Cytosol	A > G
chr15:40955456:A:G:UTR3:CASC5	chr15	40955456	40955456	A	G	UTR3	CASC5	ENST000003	.	.	A	G	+	+	0.46153846	7,0,6,0	chr15,40955	Cytosol	A > G
chr15:40955464:A:G:UTR3:CASC5	chr15	40955464	40955464	A	G	UTR3	CASC5	ENST000003	.	.	A	G	+	+	0.76923077	3,0,10,0	chr15,40955	Cytosol	A > G
chr15:41024200:A:G:UTR3:RAD51	chr15	41024200	41024200	A	G	UTR3	RAD51	ENST000002	.	.	A	G	+	+	0.23529412	13,0,4,0	chr15,41024	Cytosol	A > G
chr15:41579407:A:G:ncRNA_intro	chr15	41579407	41579407	A	G	ncRNA_intro	OIP5-AS1	.	.	.	A	G	+	+	0.71428571	2,0,5,0	chr15,41579	Cytosol	A > G
chr15:41580338:A:G:ncRNA_exoni	chr15	41580338	41580338	A	G	ncRNA_exon	OIP5-AS1	.	.	.	A	G	+	+	0.5	3,0,3,0	chr15,41580	Cytosol	A > G
chr15:41590030:A:G:ncRNA_intro	chr15	41590030	41590030	A	G	ncRNA_intro	OIP5-AS1	.	.	.	A	G	+	+	0.25	6,0,2,0	chr15,41590	Cytosol	A > G
chr15:41590838:A:G:ncRNA_exoni	chr15	41590838	41590838	A	G	ncRNA_exon	OIP5-AS1	.	.	.	A	G	+	+	0.05369128	141,0,8,0	chr15,41590	Cytosol	A > G
chr15:41590845:A:G:ncRNA_exoni	chr15	41590845	41590845	A	G	ncRNA_exon	OIP5-AS1	.	.	.	A	G	+	+	0.01694915	116,0,2,0	chr15,41590	Cytosol	A > G
chr15:41590932:A:G:ncRNA_exoni	chr15	41590932	41590932	A	G	ncRNA_exon	OIP5-AS1	.	.	.	A	G	+	+	0.10144928	186,0,21,0	chr15,41590	Cytosol	A > G
chr15:41590935:A:G:ncRNA_exoni	chr15	41590935	41590935	A	G	ncRNA_exon	OIP5-AS1	.	.	.	A	G	+	+	0.01724138	228,0,4,0	chr15,41590	Cytosol	A > G
chr15:41590938:A:G:ncRNA_exoni	chr15	41590938	41590938	A	G	ncRNA_exon	OIP5-AS1	.	.	.	A	G	+	+	0.08510638	215,0,20,0	chr15,41590	Cytosol	A > G
chr15:41590962:A:G:ncRNA_exoni	chr15	41590962	41590962	A	G	ncRNA_exon	OIP5-AS1	.	.	.	A	G	+	+	0.02489627	235,0,6,0	chr15,41590	Cytosol	A > G
chr15:41590972:A:G:ncRNA_exoni	chr15	41590972	41590972	A	G	ncRNA_exon	OIP5-AS1	.	.	.	A	G	+	+	0.04166667	253,0,11,0	chr15,41590	Cytosol	A > G
chr15:41590978:A:G:ncRNA_exoni	chr15	41590978	41590978	A	G	ncRNA_exon	OIP5-AS1	.	.	.	A	G	+	+	0.0969163	205,0,22,0	chr15,41590	Cytosol	A > G
chr15:41590982:A:G:ncRNA_exoni	chr15	41590982	41590982	A	G	ncRNA_exon	OIP5-AS1	.	.	.	A	G	+	+	0.06435644	189,0,13,0	chr15,41590	Cytosol	A > G
chr15:41591013:A:G:ncRNA_exoni	chr15	41591013	41591013	A	G	ncRNA_exon	OIP5-AS1	.	.	.	A	G	+	+	0.05442177	139,0,8,0	chr15,41591	Cytosol	A > G
chr15:41591021:A:G:ncRNA_exoni	chr15	41591021	41591021	A	G	ncRNA_exon	OIP5-AS1	.	.	.	A	G	+	+	0.13829787	81,0,13,0	chr15,41591	Cytosol	A > G
chr15:41591342:A:G:ncRNA_exoni	chr15	41591342	41591342	A	G	ncRNA_exon	OIP5-AS1	.	.	.	A	G	+	+	0.56976744	37,0,49,0	chr15,41591	Cytosol	A > G
chr15:41591397:A:G:ncRNA_exoni	chr15	41591397	41591397	A	G	ncRNA_exon	OIP5-AS1	.	.	.	A	G	+	+	0.08490566	97,0,9,0	chr15,41591	Cytosol	A > G
chr15:41591417:A:G:ncRNA_exoni	chr15	41591417	41591417	A	G	ncRNA_exon	OIP5-AS1	.	.	.	A	G	+	+	0.32989691	65,0,32,0	chr15,41591	Cytosol	A > G
chr15:41591418:A:G:ncRNA_exoni	chr15	41591418	41591418	A	G	ncRNA_exon	OIP5-AS1	.	.	.	A	G	+	+	0.41237113	57,0,40,0	chr15,41591	Cytosol	A > G
chr15:41591435:A:G:ncRNA_exoni	chr15	41591435	41591435	A	G	ncRNA_exon	OIP5-AS1	.	.	.	A	G	+	+	0.0375	77,0,3,0	chr15,41591	Cytosol	A > G
chr15:41591451:A:G:ncRNA_exoni	chr15	41591451	41591451	A	G	ncRNA_exon	OIP5-AS1	.	.	.	A	G	+	+	0.27826087	83,0,32,0	chr15,41591	Cytosol	A > G
chr15:41591457:A:G:ncRNA_exoni	chr15	41591457	41591457	A	G	ncRNA_exon	OIP5-AS1	.	.	.	A	G	+	+	0.05147059	129,0,7,0	chr15,41591	Cytosol	A > G
chr15:41593243:A:G:ncRNA_exoni	chr15	41593243	41593243	A	G	ncRNA_exon	OIP5-AS1	.	.	.	A	G	+	+	0.06122449	46,0,3,0	chr15,41593	Cytosol	A > G
chr15:41593268:A:G:ncRNA_exoni	chr15	41593268	41593268	A	G	ncRNA_exon	OIP5-AS1	.	.	.	A	G	+	+	0.10810811	33,0,4,0	chr15,41593	Cytosol	A > G
chr15:41593269:A:G:ncRNA_exoni	chr15	41593269	41593269	A	G	ncRNA_exon	OIP5-AS1	.	.	.	A	G	+	+	0.05263158	36,0,2,0	chr15,41593	Cytosol	A > G
chr15:41594436:A:G:ncRNA_exoni	chr15	41594436	41594436	A	G	ncRNA_exon	OIP5-AS1	.	.	.	A	G	+	+	0.28846154	37,0,15,0	chr15,41594	Cytosol	A > G
chr15:41594438:A:G:ncRNA_exoni	chr15	41594438	41594438	A	G	ncRNA_exon	OIP5-AS1	.	.	.	A	G	+	+	0.07692308	48,0,4,0	chr15,41594	Cytosol	A > G
chr15:41594439:A:G:ncRNA_exoni	chr15	41594439	41594439	A	G	ncRNA_exon	OIP5-AS1	.	.	.	A	G	+	+	0.0625	45,0,3,0	chr15,41594	Cytosol	A > G
chr15:41594483:A:G:ncRNA_exoni	chr15	41594483	41594483	A	G	ncRNA_exon	OIP5-AS1	.	.	.	A	G	+	+	0.19230769	42,0,10,0	chr15,41594	Cytosol	A > G
chr15:41594521:A:G:ncRNA_exoni	chr15	41594521	41594521	A	G	ncRNA_exon	OIP5-AS1	.	.	.	A	G	+	+	0.22857143	27,0,8,0	chr15,41594	Cytosol	A > G
chr15:41594530:A:G:ncRNA_exoni	chr15	41594530	41594530	A	G	ncRNA_exon	OIP5-AS1	.	.	.	A	G	+	+	0.05405405	35,0,2,0	chr15,41594	Cytosol	A > G
chr15:41594561:A:G:ncRNA_exoni	chr15	41594561	41594561	A	G	ncRNA_exon	OIP5-AS1	.	.	.	A	G	+	+	0.3	21,0,9,0	chr15,41594	Cytosol	A > G
chr15:41594585:A:G:ncRNA_exoni	chr15	41594585	41594585	A	G	ncRNA_exon	OIP5-AS1	.	.	.	A	G	+	+	0.20454545	35,0,9,0	chr15,41594	Cytosol	A > G
chr15:41594611:A:G:ncRNA_exoni	chr15	41594611	41594611	A	G	ncRNA_exon	OIP5-AS1	.	.	.	A	G	+	+	0.22857143	27,0,8,0	chr15,41594	Cytosol	A > G
chr15:41594612:A:G:ncRNA_exoni	chr15	41594612	41594612	A	G	ncRNA_exon	OIP5-AS1	.	.	.	A	G	+	+	0.04878049	39,0,2,0	chr15,41594	Cytosol	A > G
chr15:41594676:A:G:ncRNA_exoni	chr15	41594676	41594676	A	G	ncRNA_exon	OIP5-AS1	.	.	.	A	G	+	+	0.02158273	136,0,3,0	chr15,41594	Cytosol	A > G
chr15:41597476:A:G:ncRNA_exoni	chr15	41597476	41597476	A	G	ncRNA_exon	OIP5-AS1	.	.	.	A	G	+	+	0.01960784	100,0,2,0	chr15,41597	Cytosol	A > G
chr15:41597484:A:G:ncRNA_exoni	chr15	41597484	41597484	A	G	ncRNA_exon	OIP5-AS1	.	.	.	A	G	+	+	0.02678571	109,0,3,0	chr15,41597	Cytosol	A > G
chr15:41597485:A:G:ncRNA_exoni	chr15	41597485	41597485	A	G	ncRNA_exon	OIP5-AS1	.	.	.	A	G	+	+	0.06896552	108,0,8,0	chr15,41597	Cytosol	A > G
chr15:41597495:A:G:ncRNA_exoni	chr15	41597495	41597495	A	G	ncRNA_exon	OIP5-AS1	.	.	.	A	G	+	+	0.02608696	112,0,3,0	chr15,41597	Cytosol	A > G
chr15:41597523:A:G:ncRNA_exoni	chr15	41597523	41597523	A	G	ncRNA_exon	OIP5-AS1	.	.	.	A	G	+	+	0.08	115,0,10,0	chr15,41597	Cytosol	A > G
chr15:41597533:A:G:ncRNA_exoni	chr15	41597533	41597533	A	G	ncRNA_exon	OIP5-AS1	.	.	.	A	G	+	+	0.05882353	112,0,7,0	chr15,41597	Cytosol	A > G
chr15:41597543:A:G:ncRNA_exoni	chr15	41597543	41597543	A	G	ncRNA_exon	OIP5-AS1	.	.	.	A	G	+	+	0.07954545	81,0,7,0	chr15,41597	Cytosol	A > G
chr15:41597559:A:G:ncRNA_exoni	chr15	41597559	41597559	A	G	ncRNA_exon	OIP5-AS1	.	.	.	A	G	+	+	0.2	56,0,14,0	chr15,41597	Cytosol	A > G
chr15:41597568:A:G:ncRNA_exoni	chr15	41597568	41597568	A	G	ncRNA_exon	OIP5-AS1	.	.	.	A	G	+	+	0.03797468	76,0,3,0	chr15,41597	Cytosol	A > G
chr15:41597574:A:G:ncRNA_exoni	chr15	41597574	41597574	A	G	ncRNA_exon	OIP5-AS1	.	.	.	A	G	+	+	0.10843373	74,0,9,0	chr15,41597	Cytosol	A > G
chr15:41597577:A:G:ncRNA_exoni	chr15	41597577	41597577	A	G	ncRNA_exon	OIP5-AS1	.	.	.	A	G	+	+	0.53424658	34,0,39,0	chr15,41597	Cytosol	A > G
chr15:41597603:A:G:ncRNA_exoni	chr15	41597603	41597603	A	G	ncRNA_exon	OIP5-AS1	.	.	.	A	G	+	+	0.02597403	75,0,2,0	chr15,41597	Cytosol	A > G
chr15:41597616:A:G:ncRNA_exoni	chr15	41597616	41597616	A	G	ncRNA_exon	OIP5-AS1	.	.	.	A	G	+	+	0.02061856	95,0,2,0	chr15,41597	Cytosol	A > G
chr15:41597647:A:G:ncRNA_exoni	chr15	41597647	41597647	A	G	ncRNA_exon	OIP5-AS1	.	.	.	A	G	+	+	0.67213115	40,0,82,0	chr15,41597	Cytosol	A > G
chr15:41597657:A:G:ncRNA_exoni	chr15	41597657	41597657	A	G	ncRNA_exon	OIP5-AS1	.	.	.	A	G	+	+	0.14782609	98,0,17,0	chr15,41597	Cytosol	A > G

chr15:41597669:A:G:ncRNA_exoni	chr15	41597669	41597669	A	G	ncRNA_exon	OIP5-AS1	.	.	.	A	G	+	+	0.05263158	108,0,6,0	chr15,41597	Cytosol	A > G
chr15:41597671:A:G:ncRNA_exoni	chr15	41597671	41597671	A	G	ncRNA_exon	OIP5-AS1	.	.	.	A	G	+	+	0.04385965	109,0,5,0	chr15,41597	Cytosol	A > G
chr15:41599573:T:C:ncRNA_intror	chr15	41599573	41599573	T	C	ncRNA_intro	OIP5-AS1	.	.	.	A	G	-	+	0.10526316	17,0,2,0	chr15,41599	Cytosol	A > G
chr15:41600175:T:C:ncRNA_intror	chr15	41600175	41600175	T	C	ncRNA_intro	OIP5-AS1	.	.	.	A	G	-	+	0.3125	11,0,5,0	chr15,41600	Cytosol	A > G
chr15:41601026:A:G:ncRNA_exoni	chr15	41601026	41601026	A	G	ncRNA_exon	OIP5-AS1	.	.	.	A	G	+	+	0.14285714	12,0,2,0	chr15,41601	Cytosol	A > G
chr15:41682300:T:C:intronic:NDUI	chr15	41682300	41682300	T	C	intronic	NDUFAF1	.	.	.	A	G	-	-	0.5	3,0,3,0	chr15,41682	Cytosol	A > G
chr15:41682335:T:C:intronic:NDUI	chr15	41682335	41682335	T	C	intronic	NDUFAF1	.	.	.	A	G	-	-	0.28571429	5,0,2,0	chr15,41682	Cytosol	A > G
chr15:41682379:T:C:intronic:NDUI	chr15	41682379	41682379	T	C	intronic	NDUFAF1	.	.	.	A	G	-	-	0.28571429	5,0,2,0	chr15,41682	Cytosol	A > G
chr15:41683660:T:C:intronic:NDUI	chr15	41683660	41683660	T	C	intronic	NDUFAF1	.	.	.	A	G	-	-	0.5	4,0,4,0	chr15,41683	Cytosol	A > G
chr15:41683729:T:C:intronic:NDUI	chr15	41683729	41683729	T	C	intronic	NDUFAF1	.	.	.	A	G	-	-	0.44444444	5,0,4,0	chr15,41683	Cytosol	A > G
chr15:41684499:T:C:intronic:NDUI	chr15	41684499	41684499	T	C	intronic	NDUFAF1	.	.	.	A	G	-	-	0.25	6,0,2,0	chr15,41684	Cytosol	A > G
chr15:41686511:T:C:UTR3:NDUFAI	chr15	41686511	41686511	T	C	UTR3	NDUFAF1	ENST000005	.	.	A	G	-	-	0.3	7,0,3,0	chr15,41686	Cytosol	A > G
chr15:42824348:A:G:ncRNA_exoni	chr15	42824348	42824348	A	G	ncRNA_exon	RP11-265N6	.	.	.	A	G	+	+-	0.19178082	59,0,14,0	chr15,42824	Cytosol	A > G
chr15:42824393:A:G:ncRNA_exoni	chr15	42824393	42824393	A	G	ncRNA_exon	RP11-265N6	.	.	.	A	G	+	+-	0.03424658	141,0,5,0	chr15,42824	Cytosol	A > G
chr15:42824418:A:G:ncRNA_intro	chr15	42824418	42824418	A	G	ncRNA_intro	RP11-265N6	.	.	.	A	G	+	+-	0.02272727	172,0,4,0	chr15,42824	Cytosol	A > G
chr15:42824495:A:G:ncRNA_intro	chr15	42824495	42824495	A	G	ncRNA_intro	RP11-265N6	.	.	.	A	G	+	+-	0.03398058	199,0,7,0	chr15,42824	Cytosol	A > G
chr15:42824545:A:G:ncRNA_exoni	chr15	42824545	42824545	A	G	ncRNA_exon	RP11-265N6	.	.	.	A	G	+	+-	0.03333333	87,0,3,0	chr15,42824	Cytosol	A > G
chr15:42824548:A:G:ncRNA_exoni	chr15	42824548	42824548	A	G	ncRNA_exon	RP11-265N6	.	.	.	A	G	+	+-	0.05797101	65,0,4,0	chr15,42824	Cytosol	A > G
chr15:42831255:T:C:intronic:SNAP	chr15	42831255	42831255	T	C	intronic	SNAP23	.	.	.	A	G	-	+	0.13333333	13,0,2,0	chr15,42831	Cytosol	A > G
chr15:42831284:T:C:intronic:SNAP	chr15	42831284	42831284	T	C	intronic	SNAP23	.	.	.	A	G	-	+	0.16666667	10,0,2,0	chr15,42831	Cytosol	A > G
chr15:42831420:T:C:intronic:SNAP	chr15	42831420	42831420	T	C	intronic	SNAP23	.	.	.	A	G	-	+	0.14285714	12,0,2,0	chr15,42831	Cytosol	A > G
chr15:42831554:T:C:intronic:SNAP	chr15	42831554	42831554	T	C	intronic	SNAP23	.	.	.	A	G	-	+	0.22222222	7,0,2,0	chr15,42831	Cytosol	A > G
chr15:42831564:T:C:intronic:SNAP	chr15	42831564	42831564	T	C	intronic	SNAP23	.	.	.	A	G	-	+	0.22222222	7,0,2,0	chr15,42831	Cytosol	A > G
chr15:42833467:T:C:intronic:SNAP	chr15	42833467	42833467	T	C	intronic	SNAP23	.	.	.	A	G	-	+	0.4	3,0,2,0	chr15,42833	Cytosol	A > G
chr15:42833590:T:C:intronic:SNAP	chr15	42833590	42833590	T	C	intronic	SNAP23	.	.	.	A	G	-	+	0.375	5,0,3,0	chr15,42833	Cytosol	A > G
chr15:42835154:T:C:UTR3:LRRC57	chr15	42835154	42835154	T	C	UTR3	LRRC57	ENST000003	.	.	A	G	-	+-	0.07317073	38,0,3,0	chr15,42835	Cytosol	A > G
chr15:42835161:T:C:UTR3:LRRC57	chr15	42835161	42835161	T	C	UTR3	LRRC57	ENST000003	.	.	A	G	-	+-	0.07692308	36,0,3,0	chr15,42835	Cytosol	A > G
chr15:42835233:T:C:UTR3:LRRC57	chr15	42835233	42835233	T	C	UTR3	LRRC57	ENST000003	.	.	A	G	-	+-	0.03703704	52,0,2,0	chr15,42835	Cytosol	A > G
chr15:42835503:T:C:UTR3:LRRC57	chr15	42835503	42835503	T	C	UTR3	LRRC57	ENST000003	.	.	A	G	-	+-	0.05882353	32,0,2,0	chr15,42835	Cytosol	A > G
chr15:42835526:T:C:UTR3:LRRC57	chr15	42835526	42835526	T	C	UTR3	LRRC57	ENST000003	.	.	A	G	-	+-	0.03846154	50,0,2,0	chr15,42835	Cytosol	A > G
chr15:42859342:A:G:ncRNA_intro	chr15	42859342	42859342	A	G	ncRNA_intro	RP11-265N6	.	.	.	A	G	+	+-	0.07857143	129,0,11,0	chr15,42859	Cytosol	A > G
chr15:42859343:A:G:ncRNA_intro	chr15	42859343	42859343	A	G	ncRNA_intro	RP11-265N6	.	.	.	A	G	+	+-	0.05882353	128,0,8,0	chr15,42859	Cytosol	A > G
chr15:42859349:A:G:ncRNA_intro	chr15	42859349	42859349	A	G	ncRNA_intro	RP11-265N6	.	.	.	A	G	+	+-	0.04316547	133,0,6,0	chr15,42859	Cytosol	A > G
chr15:42859382:A:G:ncRNA_intro	chr15	42859382	42859382	A	G	ncRNA_intro	RP11-265N6	.	.	.	A	G	+	+-	0.30136986	51,0,22,0	chr15,42859	Cytosol	A > G
chr15:42859436:A:G:ncRNA_intro	chr15	42859436	42859436	A	G	ncRNA_intro	RP11-265N6	.	.	.	A	G	+	+-	0.01785714	110,0,2,0	chr15,42859	Cytosol	A > G
chr15:42859478:A:G:ncRNA_intro	chr15	42859478	42859478	A	G	ncRNA_intro	RP11-265N6	.	.	.	A	G	+	+-	0.04761905	80,0,4,0	chr15,42859	Cytosol	A > G
chr15:42859505:A:G:ncRNA_intro	chr15	42859505	42859505	A	G	ncRNA_intro	RP11-265N6	.	.	.	A	G	+	+-	0.17391304	57,0,12,0	chr15,42859	Cytosol	A > G
chr15:42859506:A:G:ncRNA_intro	chr15	42859506	42859506	A	G	ncRNA_intro	RP11-265N6	.	.	.	A	G	+	+-	0.17808219	60,0,13,0	chr15,42859	Cytosol	A > G
chr15:42859518:A:G:ncRNA_intro	chr15	42859518	42859518	A	G	ncRNA_intro	RP11-265N6	.	.	.	A	G	+	+-	0.11363636	78,0,10,0	chr15,42859	Cytosol	A > G
chr15:42859754:A:G:ncRNA_intro	chr15	42859754	42859754	A	G	ncRNA_intro	RP11-265N6	.	.	.	A	G	+	+-	0.02298851	85,0,2,0	chr15,42859	Cytosol	A > G
chr15:42859782:A:G:ncRNA_intro	chr15	42859782	42859782	A	G	ncRNA_intro	RP11-265N6	.	.	.	A	G	+	+-	0.06329114	74,0,5,0	chr15,42859	Cytosol	A > G
chr15:42859922:A:G:ncRNA_intro	chr15	42859922	42859922	A	G	ncRNA_intro	RP11-265N6	.	.	.	A	G	+	+-	0.16666667	35,0,7,0	chr15,42859	Cytosol	A > G
chr15:42859986:A:G:ncRNA_intro	chr15	42859986	42859986	A	G	ncRNA_intro	RP11-265N6	.	.	.	A	G	+	+-	0.08510638	43,0,4,0	chr15,42859	Cytosol	A > G
chr15:42860080:A:G:ncRNA_intro	chr15	42860080	42860080	A	G	ncRNA_intro	RP11-265N6	.	.	.	A	G	+	+-	0.07462687	62,0,5,0	chr15,42860	Cytosol	A > G
chr15:42860202:A:G:ncRNA_intro	chr15	42860202	42860202	A	G	ncRNA_intro	RP11-265N6	.	.	.	A	G	+	+-	0.02739726	71,0,2,0	chr15,42860	Cytosol	A > G
chr15:42861579:A:G:ncRNA_exoni	chr15	42861579	42861579	A	G	ncRNA_exon	RP11-265N6	.	.	.	A	G	+	+-	0.03488372	83,0,3,0	chr15,42861	Cytosol	A > G
chr15:42861584:A:G:ncRNA_exoni	chr15	42861584	42861584	A	G	ncRNA_exon	RP11-265N6	.	.	.	A	G	+	+-	0.04819277	79,0,4,0	chr15,42861	Cytosol	A > G
chr15:42861604:A:G:ncRNA_exoni	chr15	42861604	42861604	A	G	ncRNA_exon	RP11-265N6	.	.	.	A	G	+	+-	0.16949153	49,0,10,0	chr15,42861	Cytosol	A > G
chr15:42861605:A:G:ncRNA_exoni	chr15	42861605	42861605	A	G	ncRNA_exon	RP11-265N6	.	.	.	A	G	+	+-	0.29508197	43,0,18,0	chr15,42861	Cytosol	A > G
chr15:42861617:A:G:ncRNA_exoni	chr15	42861617	42861617	A	G	ncRNA_exon	RP11-265N6	.	.	.	A	G	+	+-	0.19512195	33,0,8,0	chr15,42861	Cytosol	A > G
chr15:42861630:A:G:ncRNA_exoni	chr15	42861630	42861630	A	G	ncRNA_exon	RP11-265N6	.	.	.	A	G	+	+-	0.19354839	25,0,6,0	chr15,42861	Cytosol	A > G
chr15:42861644:A:G:ncRNA_exoni	chr15	42861644	42861644	A	G	ncRNA_exon	RP11-265N6	.	.	.	A	G	+	+-	0.125	28,0,4,0	chr15,42861	Cytosol	A > G
chr15:42861705:A:G:ncRNA_intro	chr15	42861705	42861705	A	G	ncRNA_intro	RP11-265N6	.	.	.	A	G	+	+-	0.14457831	71,0,12,0	chr15,42861	Cytosol	A > G
chr15:42861739:A:G:ncRNA_intro	chr15	42861739	42861739	A	G	ncRNA_intro	RP11-265N6	.	.	.	A	G	+	+-	0.12903226	81,0,12,0	chr15,42861	Cytosol	A > G
chr15:42861740:A:G:ncRNA_intro	chr15	42861740	42861740	A	G	ncRNA_intro	RP11-265N6	.	.	.	A	G	+	+-	0.1547619	71,0,13,0	chr15,42861	Cytosol	A > G
chr15:44858978:A:G:intronic:SPG1	chr15	44858978	44858978	A	G	intronic	SPG11	.	.	.	A	G	+	-	0.4	3,0,2,0	chr15,44858	Cytosol	A > G
chr15:44873601:T:C:intronic:SPG1	chr15	44873601	44873601	T	C	intronic	SPG11	.	.	.	A	G	-	-	0.4	3,0,2,0	chr15,44873	Cytosol	A > G
chr15:44873624:T:C:intronic:SPG1	chr15	44873624	44873624	T	C	intronic	SPG11	.	.	.	A	G	-	-	0.42857143	4,0,3,0	chr15,44873	Cytosol	A > G
chr15:44873642:T:C:intronic:SPG1	chr15	44873642	44873642	T	C	intronic	SPG11	.	.	.	A	G	-	-	0.4	3,0,2,0	chr15,44873	Cytosol	A > G
chr15:44873651:T:C:intronic:SPG1	chr15	44873651	44873651	T	C	intronic	SPG11	.	.	.	A	G	-	-	0.6	2,0,3,0	chr15,44873	Cytosol	A > G
chr15:45025358:A:G:intronic:TRIM	chr15	45025358	45025358	A	G	intronic	TRIM69	.	.	.	A	G	+	+	0.28571429	5,0,2,0	chr15,45025	Cytosol	A > G
chr15:45025366:A:G:intronic:TRIM	chr15	45025366	45025366	A	G	intronic	TRIM69	.	.	.	A	G	+	+	0.66666667	3,0,6,0	chr15,45025	Cytosol	A > G
chr15:45025767:A:G:intronic:TRIM	chr15	45025767	45025767	A	G	intronic	TRIM69	.	.	.	A	G	+	+	0.22222222	7,0,2,0	chr15,45025	Cytosol	A > G
chr15:45025768:A:G:intronic:TRIM	chr15	45025768	45025768	A	G	intronic	TRIM69	.	.	.	A	G	+	+	0.66666667	3,0,6,0	chr15,45025	Cytosol	A > G

chr15:45775225:T:C:ncRNA_intror	chr15	45775225	45775225	T	C	ncRNA_intro	RP11-519G1(	.	.	.	A	G	-	+-	0.4	3,0,2,0	chr15,45775.	Cytosol	A > G
chr15:48022790:A:G:intronic:SEM.	chr15	48022790	48022790	A	G	intronic	SEMA6D	.	.	.	T	C	-	+-	1	0,12,0,0	chr15,48022'	Cytosol	T > C
chr15:50655724:A:G:ncRNA_intro	chr15	50655724	50655724	A	G	ncRNA_intro	GABPB1-AS1	.	.	.	A	G	+	+	0.6	2,0,3,0	chr15,50655'	Cytosol	A > G
chr15:50657927:A:G:ncRNA_intro	chr15	50657927	50657927	A	G	ncRNA_intro	GABPB1-AS1	.	.	.	A	G	+	+	0.71428571	2,0,5,0	chr15,50657!	Cytosol	A > G
chr15:50658624:A:G:ncRNA_intro	chr15	50658624	50658624	A	G	ncRNA_intro	GABPB1-AS1	.	.	.	A	G	+	+	0.6	2,0,3,0	chr15,50658(	Cytosol	A > G
chr15:50658860:A:G:ncRNA_intro	chr15	50658860	50658860	A	G	ncRNA_intro	GABPB1-AS1	.	.	.	A	G	+	+	0.5	3,0,3,0	chr15,50658:	Cytosol	A > G
chr15:50850687:T:C:ncRNA_intror	chr15	50850687	50850687	T	C	ncRNA_intro	RP11-120K9.	.	.	.	A	G	-	-+	0.42857143	4,0,3,0	chr15,50850(	Cytosol	A > G
chr15:50850813:T:C:ncRNA_intror	chr15	50850813	50850813	T	C	ncRNA_intro	RP11-120K9.	.	.	.	A	G	-	-+	0.5	4,0,4,0	chr15,50850:	Cytosol	A > G
chr15:50850844:T:C:ncRNA_intror	chr15	50850844	50850844	T	C	ncRNA_intro	RP11-120K9.	.	.	.	A	G	-	-+	0.44444444	5,0,4,0	chr15,50850:	Cytosol	A > G
chr15:50850846:T:C:ncRNA_intror	chr15	50850846	50850846	T	C	ncRNA_intro	RP11-120K9.	.	.	.	A	G	-	-+	0.22222222	7,0,2,0	chr15,50850:	Cytosol	A > G
chr15:50851228:T:C:ncRNA_intror	chr15	50851228	50851228	T	C	ncRNA_intro	RP11-120K9.	.	.	.	A	G	-	-+	0.33333333	6,0,3,0	chr15,50851:	Cytosol	A > G
chr15:50851578:T:C:ncRNA_intror	chr15	50851578	50851578	T	C	ncRNA_intro	RP11-120K9.	.	.	.	A	G	-	-+	0.375	5,0,3,0	chr15,50851!	Cytosol	A > G
chr15:50851640:T:C:ncRNA_intror	chr15	50851640	50851640	T	C	ncRNA_intro	RP11-120K9.	.	.	.	A	G	-	-+	0.8	1,0,4,0	chr15,50851(	Cytosol	A > G
chr15:50851641:T:C:ncRNA_intror	chr15	50851641	50851641	T	C	ncRNA_intro	RP11-120K9.	.	.	.	A	G	-	-+	0.5	3,0,3,0	chr15,50851(	Cytosol	A > G
chr15:50996377:T:C:intergenic:RN	chr15	50996377	50996377	T	C	intergenic	RN7SL354P,S	dist=14989;d	.	.	A	G	-		0.5	4,0,4,0	chr15,50996:	Cytosol	A > G
chr15:50996453:T:C:intergenic:RN	chr15	50996453	50996453	T	C	intergenic	RN7SL354P,S	dist=15065;d	.	.	A	G	-		0.90909091	1,0,10,0	chr15,50996	Cytosol	A > G
chr15:50996456:T:C:intergenic:RN	chr15	50996456	50996456	T	C	intergenic	RN7SL354P,S	dist=15068;d	.	.	A	G	-		0.81818182	2,0,9,0	chr15,50996	Cytosol	A > G
chr15:50996490:T:C:intergenic:RN	chr15	50996490	50996490	T	C	intergenic	RN7SL354P,S	dist=15102;d	.	.	A	G	-		0.23076923	10,0,3,0	chr15,50996	Cytosol	A > G
chr15:50996492:T:C:intergenic:RN	chr15	50996492	50996492	T	C	intergenic	RN7SL354P,S	dist=15104;d	.	.	A	G	-		0.38461538	8,0,5,0	chr15,50996	Cytosol	A > G
chr15:50996495:T:C:intergenic:RN	chr15	50996495	50996495	T	C	intergenic	RN7SL354P,S	dist=15107;d	.	.	A	G	-		0.72727273	3,0,8,0	chr15,50996	Cytosol	A > G
chr15:50997456:T:C:intergenic:RN	chr15	50997456	50997456	T	C	intergenic	RN7SL354P,S	dist=16068;d	.	.	A	G	-		0.19148936	38,0,9,0	chr15,50997	Cytosol	A > G
chr15:50997496:T:C:intergenic:RN	chr15	50997496	50997496	T	C	intergenic	RN7SL354P,S	dist=16108;d	.	.	A	G	-		0.17142857	29,0,6,0	chr15,50997	Cytosol	A > G
chr15:52204369:A:G:intronic:TMC	chr15	52204369	52204369	A	G	intronic	TMOD3	.	.	.	A	G	+	+	0.75	4,0,12,0	chr15,52204:	Cytosol	A > G
chr15:52204513:A:G:intronic:TMC	chr15	52204513	52204513	A	G	intronic	TMOD3	.	.	.	A	G	+	+	0.58333333	5,0,7,0	chr15,52204!	Cytosol	A > G
chr15:52205240:A:G:intronic:TMC	chr15	52205240	52205240	A	G	intronic	TMOD3	.	.	.	A	G	+	+	0.35294118	11,0,6,0	chr15,52205:	Cytosol	A > G
chr15:52205264:A:G:intronic:TMC	chr15	52205264	52205264	A	G	intronic	TMOD3	.	.	.	A	G	+	+	0.375	15,0,9,0	chr15,52205:	Cytosol	A > G
chr15:52205271:A:G:intronic:TMC	chr15	52205271	52205271	A	G	intronic	TMOD3	.	.	.	A	G	+	+	0.66666667	8,0,16,0	chr15,52205:	Cytosol	A > G
chr15:52205296:A:G:intronic:TMC	chr15	52205296	52205296	A	G	intronic	TMOD3	.	.	.	A	G	+	+	0.57692308	11,0,15,0	chr15,52205:	Cytosol	A > G
chr15:52205297:A:G:intronic:TMC	chr15	52205297	52205297	A	G	intronic	TMOD3	.	.	.	A	G	+	+	0.08	23,0,2,0	chr15,52205:	Cytosol	A > G
chr15:52205300:A:G:intronic:TMC	chr15	52205300	52205300	A	G	intronic	TMOD3	.	.	.	A	G	+	+	0.875	3,0,21,0	chr15,52205:	Cytosol	A > G
chr15:52205352:A:G:intronic:TMC	chr15	52205352	52205352	A	G	intronic	TMOD3	.	.	.	A	G	+	+	0.2	20,0,5,0	chr15,52205:	Cytosol	A > G
chr15:52205355:A:G:intronic:TMC	chr15	52205355	52205355	A	G	intronic	TMOD3	.	.	.	A	G	+	+	0.56	11,0,14,0	chr15,52205:	Cytosol	A > G
chr15:52205403:A:G:intronic:TMC	chr15	52205403	52205403	A	G	intronic	TMOD3	.	.	.	A	G	+	+	0.4	12,0,8,0	chr15,52205	Cytosol	A > G
chr15:52205412:A:G:intronic:TMC	chr15	52205412	52205412	A	G	intronic	TMOD3	.	.	.	A	G	+	+	0.14285714	18,0,3,0	chr15,52205	Cytosol	A > G
chr15:52205430:A:G:intronic:TMC	chr15	52205430	52205430	A	G	intronic	TMOD3	.	.	.	A	G	+	+	0.35	13,0,7,0	chr15,52205	Cytosol	A > G
chr15:52205431:A:G:intronic:TMC	chr15	52205431	52205431	A	G	intronic	TMOD3	.	.	.	A	G	+	+	0.47058824	9,0,8,0	chr15,52205	Cytosol	A > G
chr15:52205443:A:G:intronic:TMC	chr15	52205443	52205443	A	G	intronic	TMOD3	.	.	.	A	G	+	+	0.42857143	8,0,6,0	chr15,52205	Cytosol	A > G
chr15:52205954:A:G:intronic:TMC	chr15	52205954	52205954	A	G	intronic	TMOD3	.	.	.	A	G	+	+	0.0754717	49,0,4,0	chr15,52205!	Cytosol	A > G
chr15:52205955:A:G:intronic:TMC	chr15	52205955	52205955	A	G	intronic	TMOD3	.	.	.	A	G	+	+	0.07843137	47,0,4,0	chr15,52205!	Cytosol	A > G
chr15:52205971:A:G:intronic:TMC	chr15	52205971	52205971	A	G	intronic	TMOD3	.	.	.	A	G	+	+	0.07692308	48,0,4,0	chr15,52205!	Cytosol	A > G
chr15:52205972:A:G:intronic:TMC	chr15	52205972	52205972	A	G	intronic	TMOD3	.	.	.	A	G	+	+	0.21153846	41,0,11,0	chr15,52205!	Cytosol	A > G
chr15:52206009:A:G:intronic:TMC	chr15	52206009	52206009	A	G	intronic	TMOD3	.	.	.	A	G	+	+	0.11320755	47,0,6,0	chr15,52206(	Cytosol	A > G
chr15:52206015:A:G:intronic:TMC	chr15	52206015	52206015	A	G	intronic	TMOD3	.	.	.	A	G	+	+	0.11764706	45,0,6,0	chr15,52206(	Cytosol	A > G
chr15:52206050:A:G:intronic:TMC	chr15	52206050	52206050	A	G	intronic	TMOD3	.	.	.	A	G	+	+	0.10526316	34,0,4,0	chr15,52206(	Cytosol	A > G
chr15:52206051:A:G:intronic:TMC	chr15	52206051	52206051	A	G	intronic	TMOD3	.	.	.	A	G	+	+	0.10810811	33,0,4,0	chr15,52206(	Cytosol	A > G
chr15:52206057:A:G:intronic:TMC	chr15	52206057	52206057	A	G	intronic	TMOD3	.	.	.	A	G	+	+	0.05714286	33,0,2,0	chr15,52206(	Cytosol	A > G
chr15:52206063:A:G:intronic:TMC	chr15	52206063	52206063	A	G	intronic	TMOD3	.	.	.	A	G	+	+	0.1	27,0,3,0	chr15,52206(	Cytosol	A > G
chr15:52206089:A:G:intronic:TMC	chr15	52206089	52206089	A	G	intronic	TMOD3	.	.	.	A	G	+	+	0.48148148	14,0,13,0	chr15,52206(	Cytosol	A > G
chr15:52206116:A:G:intronic:TMC	chr15	52206116	52206116	A	G	intronic	TMOD3	.	.	.	A	G	+	+	0.06896552	27,0,2,0	chr15,52206:	Cytosol	A > G
chr15:52206130:A:G:intronic:TMC	chr15	52206130	52206130	A	G	intronic	TMOD3	.	.	.	A	G	+	+	0.11428571	31,0,4,0	chr15,52206:	Cytosol	A > G
chr15:52206135:A:G:intronic:TMC	chr15	52206135	52206135	A	G	intronic	TMOD3	.	.	.	A	G	+	+	0.71428571	10,0,25,0	chr15,52206:	Cytosol	A > G
chr15:52206154:A:G:intronic:TMC	chr15	52206154	52206154	A	G	intronic	TMOD3	.	.	.	A	G	+	+	0.52631579	18,0,20,0	chr15,52206:	Cytosol	A > G
chr15:52206155:A:G:intronic:TMC	chr15	52206155	52206155	A	G	intronic	TMOD3	.	.	.	A	G	+	+	0.14634146	35,0,6,0	chr15,52206:	Cytosol	A > G
chr15:52206312:A:G:intronic:TMC	chr15	52206312	52206312	A	G	intronic	TMOD3	.	.	.	A	G	+	+	0.15277778	61,0,11,0	chr15,52206:	Cytosol	A > G
chr15:52206590:A:G:intronic:TMC	chr15	52206590	52206590	A	G	intronic	TMOD3	.	.	.	A	G	+	+	0.09090909	30,0,3,0	chr15,52206!	Cytosol	A > G
chr15:52206606:A:G:intronic:TMC	chr15	52206606	52206606	A	G	intronic	TMOD3	.	.	.	A	G	+	+	0.5	27,0,27,0	chr15,52206(	Cytosol	A > G
chr15:52206607:A:G:intronic:TMC	chr15	52206607	52206607	A	G	intronic	TMOD3	.	.	.	A	G	+	+	0.67857143	18,0,38,0	chr15,52206(	Cytosol	A > G
chr15:52206699:A:G:intronic:TMC	chr15	52206699	52206699	A	G	intronic	TMOD3	.	.	.	A	G	+	+	0.72972973	10,0,27,0	chr15,52206(	Cytosol	A > G
chr15:52206742:A:G:intronic:TMC	chr15	52206742	52206742	A	G	intronic	TMOD3	.	.	.	A	G	+	+	0.375	25,0,15,0	chr15,52206'	Cytosol	A > G
chr15:52206827:A:G:intronic:TMC	chr15	52206827	52206827	A	G	intronic	TMOD3	.	.	.	A	G	+	+	0.15	17,0,3,0	chr15,52206:	Cytosol	A > G
chr15:52206946:A:G:intronic:TMC	chr15	52206946	52206946	A	G	intronic	TMOD3	.	.	.	A	G	+	+	0.46153846	7,0,6,0	chr15,52206!	Cytosol	A > G
chr15:52206998:A:G:intronic:TMC	chr15	52206998	52206998	A	G	intronic	TMOD3	.	.	.	A	G	+	+	0.83333333	4,0,20,0	chr15,52206!	Cytosol	A > G
chr15:52207046:A:G:intronic:TMC	chr15	52207046	52207046	A	G	intronic	TMOD3	.	.	.	A	G	+	+	0.4	12,0,8,0	chr15,52207(	Cytosol	A > G
chr15:52207058:A:G:intronic:TMC	chr15	52207058	52207058	A	G	intronic	TMOD3	.	.	.	A	G	+	+	0.58823529	7,0,10,0	chr15,52207(	Cytosol	A > G

chr15:52207068:A:G:intronic:TMO	chr15	52207068	52207068	A	G	intronic	TMOD3	.	.	.	A	G	+	+	0.65	7,0,13,0	chr15,52207	Cytosol	A > G
chr15:52207084:A:G:intronic:TMO	chr15	52207084	52207084	A	G	intronic	TMOD3	.	.	.	A	G	+	+	0.15	17,0,3,0	chr15,52207	Cytosol	A > G
chr15:52207094:A:G:intronic:TMO	chr15	52207094	52207094	A	G	intronic	TMOD3	.	.	.	A	G	+	+	0.26666667	11,0,4,0	chr15,52207	Cytosol	A > G
chr15:52207109:A:G:intronic:TMO	chr15	52207109	52207109	A	G	intronic	TMOD3	.	.	.	A	G	+	+	0.5	9,0,9,0	chr15,52207	Cytosol	A > G
chr15:52207126:A:G:intronic:TMO	chr15	52207126	52207126	A	G	intronic	TMOD3	.	.	.	A	G	+	+	0.5	10,0,10,0	chr15,52207	Cytosol	A > G
chr15:52207151:A:G:intronic:TMO	chr15	52207151	52207151	A	G	intronic	TMOD3	.	.	.	A	G	+	+	0.64705882	6,0,11,0	chr15,52207	Cytosol	A > G
chr15:52207201:A:G:intronic:TMO	chr15	52207201	52207201	A	G	intronic	TMOD3	.	.	.	A	G	+	+	0.4137931	17,0,12,0	chr15,52207	Cytosol	A > G
chr15:55609575:T:C:ncRNA_exoni	chr15	55609575	55609575	T	C	ncRNA_exon	RP11-139H1	.	.	.	A	G	-	-	0.03448276	84,0,3,0	chr15,55609	Cytosol	A > G
chr15:59414876:T:C:ncRNA_intror	chr15	59414876	59414876	T	C	ncRNA_intro	RP11-59H7.3	.	.	.	A	G	-	+	0.5	3,0,3,0	chr15,59414	Cytosol	A > G
chr15:59414895:T:C:ncRNA_intror	chr15	59414895	59414895	T	C	ncRNA_intro	RP11-59H7.3	.	.	.	A	G	-	+	0.33333333	4,0,2,0	chr15,59414	Cytosol	A > G
chr15:59414923:T:C:ncRNA_intror	chr15	59414923	59414923	T	C	ncRNA_intro	RP11-59H7.3	.	.	.	A	G	-	+	0.8	1,0,4,0	chr15,59414	Cytosol	A > G
chr15:60714387:T:C:UTR3:NARG2	chr15	60714387	60714387	T	C	UTR3	NARG2	ENST000002	.	.	A	G	-	-	0.08823529	31,0,3,0	chr15,60714	Cytosol	A > G
chr15:60714423:T:C:UTR3:NARG2	chr15	60714423	60714423	T	C	UTR3	NARG2	ENST000002	.	.	A	G	-	-	0.36666667	19,0,11,0	chr15,60714	Cytosol	A > G
chr15:60714470:T:C:UTR3:NARG2	chr15	60714470	60714470	T	C	UTR3	NARG2	ENST000002	.	.	A	G	-	-	0.2	16,0,4,0	chr15,60714	Cytosol	A > G
chr15:60714496:T:C:UTR3:NARG2	chr15	60714496	60714496	T	C	UTR3	NARG2	ENST000002	.	.	A	G	-	-	0.19047619	17,0,4,0	chr15,60714	Cytosol	A > G
chr15:60714500:T:C:UTR3:NARG2	chr15	60714500	60714500	T	C	UTR3	NARG2	ENST000002	.	.	A	G	-	-	0.63636364	8,0,14,0	chr15,60714	Cytosol	A > G
chr15:60714501:T:C:UTR3:NARG2	chr15	60714501	60714501	T	C	UTR3	NARG2	ENST000002	.	.	A	G	-	-	0.33333333	14,0,7,0	chr15,60714	Cytosol	A > G
chr15:60714502:T:C:UTR3:NARG2	chr15	60714502	60714502	T	C	UTR3	NARG2	ENST000002	.	.	A	G	-	-	0.125	21,0,3,0	chr15,60714	Cytosol	A > G
chr15:60714581:T:C:UTR3:NARG2	chr15	60714581	60714581	T	C	UTR3	NARG2	ENST000002	.	.	A	G	-	-	0.11111111	16,0,2,0	chr15,60714	Cytosol	A > G
chr15:60714916:T:C:UTR3:NARG2	chr15	60714916	60714916	T	C	UTR3	NARG2	ENST000002	.	.	A	G	-	-	0.39130435	14,0,9,0	chr15,60714	Cytosol	A > G
chr15:60714926:T:C:UTR3:NARG2	chr15	60714926	60714926	T	C	UTR3	NARG2	ENST000002	.	.	A	G	-	-	0.71428571	8,0,20,0	chr15,60714	Cytosol	A > G
chr15:60715012:T:C:UTR3:NARG2	chr15	60715012	60715012	T	C	UTR3	NARG2	ENST000002	.	.	A	G	-	-	0.23076923	10,0,3,0	chr15,60715	Cytosol	A > G
chr15:63421210:A:G:intronic:LACT	chr15	63421210	63421210	A	G	intronic	LACTB	.	.	.	A	G	+	+-	0.33333333	4,0,2,0	chr15,63421	Cytosol	A > G
chr15:63445841:T:C:UTR3:RPS27L	chr15	63445841	63445841	T	C	UTR3	RPS27L	ENST000003	.	.	A	G	-	-	0.1	27,0,3,0	chr15,63445	Cytosol	A > G
chr15:64432844:A:G:UTR3:SNX1	chr15	64432844	64432844	A	G	UTR3	SNX1	ENST000003	.	.	A	G	+	+	0.4	3,0,2,0	chr15,64432	Cytosol	A > G
chr15:64433036:A:G:UTR3:SNX1	chr15	64433036	64433036	A	G	UTR3	SNX1	ENST000003	.	.	A	G	+	+	0.42857143	4,0,3,0	chr15,64433	Cytosol	A > G
chr15:64434442:A:G:UTR3:SNX1	chr15	64434442	64434442	A	G	UTR3	SNX1	ENST000003	.	.	A	G	+	+	0.18181818	9,0,2,0	chr15,64434	Cytosol	A > G
chr15:64434502:A:G:UTR3:SNX1	chr15	64434502	64434502	A	G	UTR3	SNX1	ENST000003	.	.	A	G	+	+	0.33333333	10,0,5,0	chr15,64434	Cytosol	A > G
chr15:64446973:A:G:UTR3:SNX22	chr15	64446973	64446973	A	G	UTR3	SNX22	ENST000003	.	.	A	G	+	+	0.33333333	4,0,2,0	chr15,64446	Cytosol	A > G
chr15:64447128:A:G:UTR3:SNX22	chr15	64447128	64447128	A	G	UTR3	SNX22	ENST000003	.	.	A	G	+	+	0.57142857	3,0,4,0	chr15,64447	Cytosol	A > G
chr15:64447292:A:G:UTR3:SNX22	chr15	64447292	64447292	A	G	UTR3	SNX22	ENST000003	.	.	A	G	+	+	0.6	2,0,3,0	chr15,64447	Cytosol	A > G
chr15:64447424:A:G:UTR3:SNX22	chr15	64447424	64447424	A	G	UTR3	SNX22	ENST000003	.	.	A	G	+	+	0.4	3,0,2,0	chr15,64447	Cytosol	A > G
chr15:64447691:A:G:UTR3:SNX22	chr15	64447691	64447691	A	G	UTR3	SNX22	ENST000003	.	.	A	G	+	+	0.25	6,0,2,0	chr15,64447	Cytosol	A > G
chr15:64650904:T:C:intronic:CTD-	chr15	64650904	64650904	T	C	intronic	CTD-2116N1	.	.	.	A	G	-	-	0.77777778	2,0,7,0	chr15,64650	Cytosol	A > G
chr15:64651809:T:C:intronic:CTD-	chr15	64651809	64651809	T	C	intronic	CTD-2116N1	.	.	.	A	G	-	-	0.13043478	20,0,3,0	chr15,64651	Cytosol	A > G
chr15:64651880:T:C:intronic:CTD-	chr15	64651880	64651880	T	C	intronic	CTD-2116N1	.	.	.	A	G	-	-	0.70588235	5,0,12,0	chr15,64651	Cytosol	A > G
chr15:64652019:T:C:intronic:CTD-	chr15	64652019	64652019	T	C	intronic	CTD-2116N1	.	.	.	A	G	-	-	0.85714286	1,0,6,0	chr15,64652	Cytosol	A > G
chr15:64652147:T:C:intronic:CTD-	chr15	64652147	64652147	T	C	intronic	CTD-2116N1	.	.	.	A	G	-	-	0.5	3,0,3,0	chr15,64652	Cytosol	A > G
chr15:64653646:T:C:intronic:CTD-	chr15	64653646	64653646	T	C	intronic	CTD-2116N1	.	.	.	A	G	-	-	0.25925926	20,0,7,0	chr15,64653	Cytosol	A > G
chr15:64653662:T:C:intronic:CTD-	chr15	64653662	64653662	T	C	intronic	CTD-2116N1	.	.	.	A	G	-	-	0.16	21,0,4,0	chr15,64653	Cytosol	A > G
chr15:64653733:T:C:intronic:CTD-	chr15	64653733	64653733	T	C	intronic	CTD-2116N1	.	.	.	A	G	-	-	0.0952381	19,0,2,0	chr15,64653	Cytosol	A > G
chr15:64653737:T:C:intronic:CTD-	chr15	64653737	64653737	T	C	intronic	CTD-2116N1	.	.	.	A	G	-	-	0.29166667	17,0,7,0	chr15,64653	Cytosol	A > G
chr15:64653845:T:C:intronic:CTD-	chr15	64653845	64653845	T	C	intronic	CTD-2116N1	.	.	.	A	G	-	-	0.27777778	13,0,5,0	chr15,64653	Cytosol	A > G
chr15:64654562:T:C:intronic:CTD-	chr15	64654562	64654562	T	C	intronic	CTD-2116N1	.	.	.	A	G	-	-	0.47058824	9,0,8,0	chr15,64654	Cytosol	A > G
chr15:64654700:T:C:intronic:CTD-	chr15	64654700	64654700	T	C	intronic	CTD-2116N1	.	.	.	A	G	-	-	0.7	9,0,21,0	chr15,64654	Cytosol	A > G
chr15:64654733:T:C:intronic:CTD-	chr15	64654733	64654733	T	C	intronic	CTD-2116N1	.	.	.	A	G	-	-	0.82758621	5,0,24,0	chr15,64654	Cytosol	A > G
chr15:64655089:T:C:intronic:CTD-	chr15	64655089	64655089	T	C	intronic	CTD-2116N1	.	.	.	A	G	-	-	0.66666667	3,0,6,0	chr15,64655	Cytosol	A > G
chr15:64655099:T:C:intronic:CTD-	chr15	64655099	64655099	T	C	intronic	CTD-2116N1	.	.	.	A	G	-	-	0.55555556	4,0,5,0	chr15,64655	Cytosol	A > G
chr15:64655672:T:C:intronic:CTD-	chr15	64655672	64655672	T	C	intronic	CTD-2116N1	.	.	.	A	G	-	-	1	0,0,7,0	chr15,64655	Cytosol	A > G
chr15:64655674:T:C:intronic:CTD-	chr15	64655674	64655674	T	C	intronic	CTD-2116N1	.	.	.	A	G	-	-	1	0,0,5,0	chr15,64655	Cytosol	A > G
chr15:64656107:T:C:intronic:CTD-	chr15	64656107	64656107	T	C	intronic	CTD-2116N1	.	.	.	A	G	-	-	0.66666667	5,0,10,0	chr15,64656	Cytosol	A > G
chr15:64657290:T:C:UTR3:KIAA01	chr15	64657290	64657290	T	C	UTR3	KIAA0101	ENST000003	.	.	A	G	-	-	0.04761905	80,0,4,0	chr15,64657	Cytosol	A > G
chr15:64657366:T:C:UTR3:KIAA01	chr15	64657366	64657366	T	C	UTR3	KIAA0101	ENST000003	.	.	A	G	-	-	0.05	38,0,2,0	chr15,64657	Cytosol	A > G
chr15:64657403:T:C:UTR3:KIAA01	chr15	64657403	64657403	T	C	UTR3	KIAA0101	ENST000003	.	.	A	G	-	-	0.04761905	40,0,2,0	chr15,64657	Cytosol	A > G
chr15:64657473:T:C:UTR3:KIAA01	chr15	64657473	64657473	T	C	UTR3	KIAA0101	ENST000003	.	.	A	G	-	-	0.0625	75,0,5,0	chr15,64657	Cytosol	A > G
chr15:65441554:T:C:UTR3:CLPX	chr15	65441554	65441554	T	C	UTR3	CLPX	ENST000003	.	.	A	G	-	-	0.05263158	54,0,3,0	chr15,65441	Cytosol	A > G
chr15:65441566:T:C:UTR3:CLPX	chr15	65441566	65441566	T	C	UTR3	CLPX	ENST000003	.	.	A	G	-	-	0.20689655	46,0,12,0	chr15,65441	Cytosol	A > G
chr15:65441664:T:C:UTR3:CLPX	chr15	65441664	65441664	T	C	UTR3	CLPX	ENST000003	.	.	A	G	-	-	0.16666667	29,0,6,1	chr15,65441	Cytosol	A > G
chr15:65441713:T:C:UTR3:CLPX	chr15	65441713	65441713	T	C	UTR3	CLPX	ENST000003	.	.	A	G	-	-	0.10204082	44,0,5,0	chr15,65441	Cytosol	A > G
chr15:65441720:T:C:UTR3:CLPX	chr15	65441720	65441720	T	C	UTR3	CLPX	ENST000003	.	.	A	G	-	-	0.04545455	42,0,2,0	chr15,65441	Cytosol	A > G
chr15:65441730:T:C:UTR3:CLPX	chr15	65441730	65441730	T	C	UTR3	CLPX	ENST000003	.	.	A	G	-	-	0.06666667	42,0,3,0	chr15,65441	Cytosol	A > G
chr15:65441777:T:C:UTR3:CLPX	chr15	65441777	65441777	T	C	UTR3	CLPX	ENST000003	.	.	A	G	-	-	0.18918919	30,0,7,0	chr15,65441	Cytosol	A > G
chr15:65441934:T:C:UTR3:CLPX	chr15	65441934	65441934	T	C	UTR3	CLPX	ENST000003	.	.	A	G	-	-	0.1875	13,0,3,0	chr15,65441	Cytosol	A > G
chr15:65441935:T:C:UTR3:CLPX	chr15	65441935	65441935	T	C	UTR3	CLPX	ENST000003	.	.	A	G	-	-	0.125	14,0,2,0	chr15,65441	Cytosol	A > G

chr15:65441955:T:C:UTR3:CLPX	chr15	65441955	65441955	T	C	UTR3	CLPX	ENST000003	.	.	A	G	-	-	0.3	7,0,3,0	chr15,65441	Cytosol	A > G
chr15:65442001:T:C:UTR3:CLPX	chr15	65442001	65442001	T	C	UTR3	CLPX	ENST000003	.	.	A	G	-	-	0.44444444	5,0,4,0	chr15,65442	Cytosol	A > G
chr15:65442032:T:C:UTR3:CLPX	chr15	65442032	65442032	T	C	UTR3	CLPX	ENST000003	.	.	A	G	-	-	0.38461538	8,0,5,0	chr15,65442	Cytosol	A > G
chr15:65442086:T:C:UTR3:CLPX	chr15	65442086	65442086	T	C	UTR3	CLPX	ENST000003	.	.	A	G	-	-	0.16	21,0,4,0	chr15,65442	Cytosol	A > G
chr15:65442098:T:C:UTR3:CLPX	chr15	65442098	65442098	T	C	UTR3	CLPX	ENST000003	.	.	A	G	-	-	0.3	14,0,6,0	chr15,65442	Cytosol	A > G
chr15:65442112:T:C:UTR3:CLPX	chr15	65442112	65442112	T	C	UTR3	CLPX	ENST000003	.	.	A	G	-	-	0.57692308	11,0,15,0	chr15,65442	Cytosol	A > G
chr15:65442115:T:C:UTR3:CLPX	chr15	65442115	65442115	T	C	UTR3	CLPX	ENST000003	.	.	A	G	-	-	0.18518519	22,0,5,0	chr15,65442	Cytosol	A > G
chr15:65442116:T:C:UTR3:CLPX	chr15	65442116	65442116	T	C	UTR3	CLPX	ENST000003	.	.	A	G	-	-	0.07692308	24,0,2,0	chr15,65442	Cytosol	A > G
chr15:65442122:T:C:UTR3:CLPX	chr15	65442122	65442122	T	C	UTR3	CLPX	ENST000003	.	.	A	G	-	-	0.32	17,0,8,0	chr15,65442	Cytosol	A > G
chr15:65442476:T:C:UTR3:CLPX	chr15	65442476	65442476	T	C	UTR3	CLPX	ENST000003	.	.	A	G	-	-	0.25806452	23,0,8,0	chr15,65442	Cytosol	A > G
chr15:65442483:T:C:UTR3:CLPX	chr15	65442483	65442483	T	C	UTR3	CLPX	ENST000003	.	.	A	G	-	-	0.2972973	26,0,11,0	chr15,65442	Cytosol	A > G
chr15:65442535:T:C:UTR3:CLPX	chr15	65442535	65442535	T	C	UTR3	CLPX	ENST000003	.	.	A	G	-	-	0.15151515	28,0,5,0	chr15,65442	Cytosol	A > G
chr15:66625880:A:G:UTR3:DIS3L	chr15	66625880	66625880	A	G	UTR3	DIS3L	ENST000003	.	.	A	G	+	+	0.11764706	30,0,4,0	chr15,66625	Cytosol	A > G
chr15:66625881:A:G:UTR3:DIS3L	chr15	66625881	66625881	A	G	UTR3	DIS3L	ENST000003	.	.	A	G	+	+	0.06060606	31,0,2,0	chr15,66625	Cytosol	A > G
chr15:66625959:T:C:UTR3:DIS3L	chr15	66625959	66625959	T	C	UTR3	DIS3L	ENST000003	.	.	A	G	-	+	0.6	2,0,3,0	chr15,66625	Cytosol	A > G
chr15:66626889:T:C:downstream:	chr15	66626889	66626889	T	C	downstream	DIS3L	.	.	.	A	G	-		0.21052632	15,0,4,0	chr15,66626	Cytosol	A > G
chr15:66626948:T:C:downstream:	chr15	66626948	66626948	T	C	downstream	DIS3L	.	.	.	A	G	-		0.5	5,0,5,0	chr15,66626	Cytosol	A > G
chr15:81268265:T:C:UTR3:MESDC	chr15	81268265	81268265	T	C	UTR3	MESDC2	ENST000002	.	.	A	G	-	-	0.2745098	37,0,14,0	chr15,81268	Cytosol	A > G
chr15:81268275:T:C:UTR3:MESDC	chr15	81268275	81268275	T	C	UTR3	MESDC2	ENST000002	.	.	A	G	-	-	0.15686275	43,0,8,0	chr15,81268	Cytosol	A > G
chr15:83670792:T:C:ncRNA_intror	chr15	83670792	83670792	T	C	ncRNA_intro	RP11-382A2	.	.	.	A	G	-	+-	0.30769231	9,0,4,0	chr15,83670	Cytosol	A > G
chr15:83671641:T:C:ncRNA_intror	chr15	83671641	83671641	T	C	ncRNA_intro	RP11-382A2	.	.	.	A	G	-	+-	0.25	6,0,2,0	chr15,83671	Cytosol	A > G
chr15:83674084:T:C:ncRNA_intror	chr15	83674084	83674084	T	C	ncRNA_intro	RP11-382A2	.	.	.	A	G	-	+-	0.03333333	87,0,3,0	chr15,83674	Cytosol	A > G
chr15:83674110:T:C:ncRNA_intror	chr15	83674110	83674110	T	C	ncRNA_intro	RP11-382A2	.	.	.	A	G	-	+-	0.25925926	60,0,21,0	chr15,83674	Cytosol	A > G
chr15:83674152:T:C:ncRNA_intror	chr15	83674152	83674152	T	C	ncRNA_intro	RP11-382A2	.	.	.	A	G	-	+-	0.05319149	89,0,5,0	chr15,83674	Cytosol	A > G
chr15:83674161:T:C:ncRNA_intror	chr15	83674161	83674161	T	C	ncRNA_intro	RP11-382A2	.	.	.	A	G	-	+-	0.42	58,0,42,0	chr15,83674	Cytosol	A > G
chr15:83674165:T:C:ncRNA_intror	chr15	83674165	83674165	T	C	ncRNA_intro	RP11-382A2	.	.	.	A	G	-	+-	0.02752294	106,0,3,0	chr15,83674	Cytosol	A > G
chr15:83674174:T:C:ncRNA_intror	chr15	83674174	83674174	T	C	ncRNA_intro	RP11-382A2	.	.	.	A	G	-	+-	0.05426357	122,0,7,0	chr15,83674	Cytosol	A > G
chr15:83674201:T:C:ncRNA_intror	chr15	83674201	83674201	T	C	ncRNA_intro	RP11-382A2	.	.	.	A	G	-	+-	0.01538462	128,0,2,0	chr15,83674	Cytosol	A > G
chr15:83674224:T:C:ncRNA_intror	chr15	83674224	83674224	T	C	ncRNA_intro	RP11-382A2	.	.	.	A	G	-	+-	0.01587302	186,0,3,0	chr15,83674	Cytosol	A > G
chr15:83674237:T:C:ncRNA_intror	chr15	83674237	83674237	T	C	ncRNA_intro	RP11-382A2	.	.	.	A	G	-	+-	0.03910615	172,0,7,0	chr15,83674	Cytosol	A > G
chr15:83674263:T:C:ncRNA_intror	chr15	83674263	83674263	T	C	ncRNA_intro	RP11-382A2	.	.	.	A	G	-	+-	0.02272727	129,0,3,0	chr15,83674	Cytosol	A > G
chr15:83674273:T:C:ncRNA_intror	chr15	83674273	83674273	T	C	ncRNA_intro	RP11-382A2	.	.	.	A	G	-	+-	0.02409639	81,0,2,0	chr15,83674	Cytosol	A > G
chr15:83674325:T:C:ncRNA_intror	chr15	83674325	83674325	T	C	ncRNA_intro	RP11-382A2	.	.	.	A	G	-	+-	0.07079646	105,0,8,0	chr15,83674	Cytosol	A > G
chr15:85183875:T:C:ncRNA_exoni	chr15	85183875	85183875	T	C	ncRNA_exon	SCAND2P	.	.	.	A	G	-	+	0.25	12,0,4,0	chr15,85183	Cytosol	A > G
chr15:85183893:T:C:ncRNA_exoni	chr15	85183893	85183893	T	C	ncRNA_exon	SCAND2P	.	.	.	A	G	-	+	0.53846154	6,0,7,0	chr15,85183	Cytosol	A > G
chr15:85184830:T:C:ncRNA_exoni	chr15	85184830	85184830	T	C	ncRNA_exon	SCAND2P	.	.	.	A	G	-	+-	0.6875	10,0,22,0	chr15,85184	Cytosol	A > G
chr15:85184841:T:C:ncRNA_exoni	chr15	85184841	85184841	T	C	ncRNA_exon	SCAND2P	.	.	.	A	G	-	+-	0.29032258	22,0,9,0	chr15,85184	Cytosol	A > G
chr15:85184842:T:C:ncRNA_exoni	chr15	85184842	85184842	T	C	ncRNA_exon	SCAND2P	.	.	.	A	G	-	+-	0.06896552	27,0,2,0	chr15,85184	Cytosol	A > G
chr15:85184869:T:C:ncRNA_exoni	chr15	85184869	85184869	T	C	ncRNA_exon	SCAND2P	.	.	.	A	G	-	+-	0.06896552	27,0,2,0	chr15,85184	Cytosol	A > G
chr15:85184873:T:C:ncRNA_exoni	chr15	85184873	85184873	T	C	ncRNA_exon	SCAND2P	.	.	.	A	G	-	+-	0.10810811	33,0,4,0	chr15,85184	Cytosol	A > G
chr15:85185045:T:C:ncRNA_exoni	chr15	85185045	85185045	T	C	ncRNA_exon	SCAND2P	.	.	.	A	G	-	+-	0.37931034	18,0,11,0	chr15,85185	Cytosol	A > G
chr15:85185205:T:C:ncRNA_exoni	chr15	85185205	85185205	T	C	ncRNA_exon	SCAND2P	.	.	.	A	G	-	+-	0.35483871	20,0,11,0	chr15,85185	Cytosol	A > G
chr15:85185391:T:C:ncRNA_exoni	chr15	85185391	85185391	T	C	ncRNA_exon	SCAND2P	.	.	.	A	G	-	+-	0.05	38,0,2,0	chr15,85185	Cytosol	A > G
chr15:85185440:A:G:ncRNA_exoni	chr15	85185440	85185440	A	G	ncRNA_exon	SCAND2P	.	.	.	A	G	+	+-	0.33333333	4,0,2,0	chr15,85185	Cytosol	A > G
chr15:85185449:T:C:ncRNA_exoni	chr15	85185449	85185449	T	C	ncRNA_exon	SCAND2P	.	.	.	A	G	-	+-	0.42592593	30,0,23,1	chr15,85185	Cytosol	A > G
chr15:85185459:T:C:ncRNA_exoni	chr15	85185459	85185459	T	C	ncRNA_exon	SCAND2P	.	.	.	A	G	-	+-	0.05454545	52,0,3,0	chr15,85185	Cytosol	A > G
chr15:85185488:T:C:ncRNA_exoni	chr15	85185488	85185488	T	C	ncRNA_exon	SCAND2P	.	.	.	A	G	-	+-	0.36666667	38,0,22,0	chr15,85185	Cytosol	A > G
chr15:85185862:T:C:downstream:	chr15	85185862	85185862	T	C	downstream	SCAND2P,WI	.	.	.	A	G	-	-	0.07042254	66,0,5,0	chr15,85185	Cytosol	A > G
chr15:89021961:A:G:ncRNA_intro	chr15	89021961	89021961	A	G	ncRNA_intro	RP11-97O12.	.	.	.	A	G	+	-+	0.4	9,0,6,0	chr15,89021	Cytosol	A > G
chr15:89022073:A:G:ncRNA_intro	chr15	89022073	89022073	A	G	ncRNA_intro	RP11-97O12.	.	.	.	A	G	+	-+	0.30769231	9,0,4,0	chr15,89022	Cytosol	A > G
chr15:90375419:T:C:UTR3:AP3S2	chr15	90375419	90375419	T	C	UTR3	AP3S2	ENST000003	.	.	A	G	-	-	0.21621622	29,0,8,0	chr15,90375	Cytosol	A > G
chr15:90375494:T:C:UTR3:AP3S2	chr15	90375494	90375494	T	C	UTR3	AP3S2	ENST000003	.	.	A	G	-	-	0.87755102	6,0,43,0	chr15,90375	Cytosol	A > G
chr15:90375510:T:C:UTR3:AP3S2	chr15	90375510	90375510	T	C	UTR3	AP3S2	ENST000003	.	.	A	G	-	-	0.07894737	35,0,3,0	chr15,90375	Cytosol	A > G
chr15:90375568:T:C:UTR3:AP3S2	chr15	90375568	90375568	T	C	UTR3	AP3S2	ENST000003	.	.	A	G	-	-	0.63636364	16,0,28,0	chr15,90375	Cytosol	A > G
chr15:90375890:T:C:UTR3:AP3S2	chr15	90375890	90375890	T	C	UTR3	AP3S2	ENST000003	.	.	A	G	-	-	0.08695652	21,0,2,0	chr15,90375	Cytosol	A > G
chr15:90375921:T:C:UTR3:AP3S2	chr15	90375921	90375921	T	C	UTR3	AP3S2	ENST000003	.	.	A	G	-	-	0.29411765	12,0,5,0	chr15,90375	Cytosol	A > G
chr15:90375930:T:C:UTR3:AP3S2	chr15	90375930	90375930	T	C	UTR3	AP3S2	ENST000003	.	.	A	G	-	-	0.11764706	15,0,2,0	chr15,90375	Cytosol	A > G
chr15:90375992:T:C:UTR3:AP3S2	chr15	90375992	90375992	T	C	UTR3	AP3S2	ENST000003	.	.	A	G	-	-	0.30769231	9,0,4,0	chr15,90375	Cytosol	A > G
chr15:90376001:T:C:UTR3:AP3S2	chr15	90376001	90376001	T	C	UTR3	AP3S2	ENST000003	.	.	A	G	-	-	0.17647059	14,0,3,0	chr15,90376	Cytosol	A > G
chr15:90440482:T:C:intronic:C15o	chr15	90440482	90440482	T	C	intronic	C15orf38-AP	.	.	.	A	G	-	-	0.2962963	38,0,16,0	chr15,90440	Cytosol	A > G
chr15:90440499:T:C:intronic:C15o	chr15	90440499	90440499	T	C	intronic	C15orf38-AP	.	.	.	A	G	-	-	0.10909091	49,0,6,0	chr15,90440	Cytosol	A > G
chr15:90440554:T:C:intronic:C15o	chr15	90440554	90440554	T	C	intronic	C15orf38-AP	.	.	.	A	G	-	-	0.5	14,0,14,0	chr15,90440	Cytosol	A > G
chr15:90440560:T:C:intronic:C15o	chr15	90440560	90440560	T	C	intronic	C15orf38-AP	.	.	.	A	G	-	-	0.2173913	18,0,5,0	chr15,90440	Cytosol	A > G
chr15:90440569:T:C:intronic:C15o	chr15	90440569	90440569	T	C	intronic	C15orf38-AP	.	.	.	A	G	-	-	0.45454545	12,0,10,0	chr15,90440	Cytosol	A > G

chr15:90440595:T:C:intronic:C15o	chr15	90440595	90440595	T	C	intronic	C15orf38-AP	.	.	.	A	G	-	-	0.52	12,0,13,0	chr15,90440	Cytosol	A > G
chr15:90440654:T:C:intronic:C15o	chr15	90440654	90440654	T	C	intronic	C15orf38-AP	.	.	.	A	G	-	-	0.44	14,0,11,0	chr15,90440	Cytosol	A > G
chr15:90440655:T:C:intronic:C15o	chr15	90440655	90440655	T	C	intronic	C15orf38-AP	.	.	.	A	G	-	-	0.48275862	15,0,14,0	chr15,90440	Cytosol	A > G
chr15:90440660:T:C:intronic:C15o	chr15	90440660	90440660	T	C	intronic	C15orf38-AP	.	.	.	A	G	-	-	0.44827586	16,0,13,0	chr15,90440	Cytosol	A > G
chr15:90440678:T:C:intronic:C15o	chr15	90440678	90440678	T	C	intronic	C15orf38-AP	.	.	.	A	G	-	-	0.13157895	33,0,5,0	chr15,90440	Cytosol	A > G
chr15:90441105:T:C:intronic:C15o	chr15	90441105	90441105	T	C	intronic	C15orf38-AP	.	.	.	A	G	-	-	0.31428571	24,0,11,0	chr15,90441	Cytosol	A > G
chr15:90441148:T:C:intronic:C15o	chr15	90441148	90441148	T	C	intronic	C15orf38-AP	.	.	.	A	G	-	-	0.28571429	15,0,6,0	chr15,90441	Cytosol	A > G
chr15:90441168:T:C:intronic:C15o	chr15	90441168	90441168	T	C	intronic	C15orf38-AP	.	.	.	A	G	-	-	0.5	15,0,15,0	chr15,90441	Cytosol	A > G
chr15:90441208:T:C:intronic:C15o	chr15	90441208	90441208	T	C	intronic	C15orf38-AP	.	.	.	A	G	-	-	0.22727273	34,0,10,0	chr15,90441	Cytosol	A > G
chr15:90441221:T:C:intronic:C15o	chr15	90441221	90441221	T	C	intronic	C15orf38-AP	.	.	.	A	G	-	-	0.58823529	21,0,30,0	chr15,90441	Cytosol	A > G
chr15:90441262:T:C:intronic:C15o	chr15	90441262	90441262	T	C	intronic	C15orf38-AP	.	.	.	A	G	-	-	0.41666667	7,0,5,0	chr15,90441	Cytosol	A > G
chr15:90441263:T:C:intronic:C15o	chr15	90441263	90441263	T	C	intronic	C15orf38-AP	.	.	.	A	G	-	-	0.16666667	10,0,2,0	chr15,90441	Cytosol	A > G
chr15:90441269:T:C:intronic:C15o	chr15	90441269	90441269	T	C	intronic	C15orf38-AP	.	.	.	A	G	-	-	0.66666667	5,0,10,0	chr15,90441	Cytosol	A > G
chr15:90441337:T:C:intronic:C15o	chr15	90441337	90441337	T	C	intronic	C15orf38-AP	.	.	.	A	G	-	-	0.375	5,0,3,0	chr15,90441	Cytosol	A > G
chr15:90442412:T:C:intronic:C15o	chr15	90442412	90442412	T	C	intronic	C15orf38-AP	.	.	.	A	G	-	-	0.53846154	6,0,7,0	chr15,90442	Cytosol	A > G
chr15:90442413:T:C:intronic:C15o	chr15	90442413	90442413	T	C	intronic	C15orf38-AP	.	.	.	A	G	-	-	0.45454545	6,0,5,0	chr15,90442	Cytosol	A > G
chr15:90442454:T:C:intronic:C15o	chr15	90442454	90442454	T	C	intronic	C15orf38-AP	.	.	.	A	G	-	-	0.75	4,0,12,0	chr15,90442	Cytosol	A > G
chr15:90442464:T:C:intronic:C15o	chr15	90442464	90442464	T	C	intronic	C15orf38-AP	.	.	.	A	G	-	-	0.4	9,0,6,0	chr15,90442	Cytosol	A > G
chr15:90442472:T:C:intronic:C15o	chr15	90442472	90442472	T	C	intronic	C15orf38-AP	.	.	.	A	G	-	-	0.15789474	16,0,3,0	chr15,90442	Cytosol	A > G
chr15:90442480:T:C:intronic:C15o	chr15	90442480	90442480	T	C	intronic	C15orf38-AP	.	.	.	A	G	-	-	0.25	18,0,6,0	chr15,90442	Cytosol	A > G
chr15:90442495:T:C:intronic:C15o	chr15	90442495	90442495	T	C	intronic	C15orf38-AP	.	.	.	A	G	-	-	0.10714286	25,0,3,0	chr15,90442	Cytosol	A > G
chr15:90442499:T:C:intronic:C15o	chr15	90442499	90442499	T	C	intronic	C15orf38-AP	.	.	.	A	G	-	-	0.12	22,0,3,0	chr15,90442	Cytosol	A > G
chr15:90442510:T:C:intronic:C15o	chr15	90442510	90442510	T	C	intronic	C15orf38-AP	.	.	.	A	G	-	-	0.19230769	21,0,5,0	chr15,90442	Cytosol	A > G
chr15:90442516:T:C:intronic:C15o	chr15	90442516	90442516	T	C	intronic	C15orf38-AP	.	.	.	A	G	-	-	0.38461538	16,0,10,0	chr15,90442	Cytosol	A > G
chr15:90442533:T:C:intronic:C15o	chr15	90442533	90442533	T	C	intronic	C15orf38-AP	.	.	.	A	G	-	-	0.14705882	29,0,5,0	chr15,90442	Cytosol	A > G
chr15:90442534:T:C:intronic:C15o	chr15	90442534	90442534	T	C	intronic	C15orf38-AP	.	.	.	A	G	-	-	0.63636364	12,0,21,0	chr15,90442	Cytosol	A > G
chr15:90442544:T:C:intronic:C15o	chr15	90442544	90442544	T	C	intronic	C15orf38-AP	.	.	.	A	G	-	-	0.28	18,0,7,0	chr15,90442	Cytosol	A > G
chr15:90442549:T:C:intronic:C15o	chr15	90442549	90442549	T	C	intronic	C15orf38-AP	.	.	.	A	G	-	-	0.125	21,0,3,0	chr15,90442	Cytosol	A > G
chr15:90442554:T:C:intronic:C15o	chr15	90442554	90442554	T	C	intronic	C15orf38-AP	.	.	.	A	G	-	-	0.62068966	11,0,18,0	chr15,90442	Cytosol	A > G
chr15:90442590:T:C:intronic:C15o	chr15	90442590	90442590	T	C	intronic	C15orf38-AP	.	.	.	A	G	-	-	0.2	32,0,8,0	chr15,90442	Cytosol	A > G
chr15:90444685:T:C:UTR3:C15orf3	chr15	90444685	90444685	T	C	UTR3	C15orf38	ENST000003	.	.	A	G	-	-	0.1754386	47,0,10,0	chr15,90444	Cytosol	A > G
chr15:90444708:T:C:UTR3:C15orf3	chr15	90444708	90444708	T	C	UTR3	C15orf38	ENST000003	.	.	A	G	-	-	0.4	42,0,28,0	chr15,90444	Cytosol	A > G
chr15:90444728:T:C:UTR3:C15orf3	chr15	90444728	90444728	T	C	UTR3	C15orf38	ENST000003	.	.	A	G	-	-	0.40506329	47,0,32,0	chr15,90444	Cytosol	A > G
chr15:90816857:A:G:intergenic:RP	chr15	90816857	90816857	A	G	intergenic	RP11-697E2.	dist=1417;dis	.	.	A	G	+		0.72727273	3,0,8,0	chr15,90816	Cytosol	A > G
chr15:90816984:A:G:intergenic:RP	chr15	90816984	90816984	A	G	intergenic	RP11-697E2.	dist=1544;dis	.	.	A	G	+		0.2	8,0,2,0	chr15,90816	Cytosol	A > G
chr15:90816987:A:G:intergenic:RP	chr15	90816987	90816987	A	G	intergenic	RP11-697E2.	dist=1547;dis	.	.	A	G	+		0.4	6,0,4,0	chr15,90816	Cytosol	A > G
chr15:90817708:A:G:upstream:RP	chr15	90817708	90817708	A	G	upstream	RP11-697E2.	.	.	.	A	G	+		0.5	4,0,4,0	chr15,90817	Cytosol	A > G
chr15:90817844:A:G:upstream:RP	chr15	90817844	90817844	A	G	upstream	RP11-697E2.	.	.	.	A	G	+		0.71428571	2,0,5,0	chr15,90817	Cytosol	A > G
chr15:90817855:A:G:upstream:RP	chr15	90817855	90817855	A	G	upstream	RP11-697E2.	.	.	.	A	G	+		0.44444444	5,0,4,0	chr15,90817	Cytosol	A > G
chr15:90819418:A:G:ncRNA_exoni	chr15	90819418	90819418	A	G	ncRNA_exon	RP11-697E2.	.	.	.	A	G	+	+	0.30434783	16,0,7,0	chr15,90819	Cytosol	A > G
chr15:90819526:A:G:ncRNA_exoni	chr15	90819526	90819526	A	G	ncRNA_exon	RP11-697E2.	.	.	.	A	G	+	+	0.21052632	15,0,4,0	chr15,90819	Cytosol	A > G
chr15:90819530:A:G:ncRNA_exoni	chr15	90819530	90819530	A	G	ncRNA_exon	RP11-697E2.	.	.	.	A	G	+	+	0.375	10,0,6,0	chr15,90819	Cytosol	A > G
chr15:90819552:A:G:ncRNA_exoni	chr15	90819552	90819552	A	G	ncRNA_exon	RP11-697E2.	.	.	.	A	G	+	+	0.47826087	12,0,11,0	chr15,90819	Cytosol	A > G
chr15:90819579:A:G:ncRNA_exoni	chr15	90819579	90819579	A	G	ncRNA_exon	RP11-697E2.	.	.	.	A	G	+	+	0.76470588	4,0,13,0	chr15,90819	Cytosol	A > G
chr15:90819961:A:G:ncRNA_exoni	chr15	90819961	90819961	A	G	ncRNA_exon	RP11-697E2.	.	.	.	A	G	+	+	0.11538462	23,0,3,0	chr15,90819	Cytosol	A > G
chr15:90819997:A:G:ncRNA_exoni	chr15	90819997	90819997	A	G	ncRNA_exon	RP11-697E2.	.	.	.	A	G	+	+	0.33333333	14,0,7,0	chr15,90819	Cytosol	A > G
chr15:90820006:A:G:ncRNA_exoni	chr15	90820006	90820006	A	G	ncRNA_exon	RP11-697E2.	.	.	.	A	G	+	+	0.36842105	12,0,7,0	chr15,90820	Cytosol	A > G
chr15:90820012:A:G:ncRNA_exoni	chr15	90820012	90820012	A	G	ncRNA_exon	RP11-697E2.	.	.	.	A	G	+	+	0.26315789	14,0,5,0	chr15,90820	Cytosol	A > G
chr15:90820028:A:G:ncRNA_exoni	chr15	90820028	90820028	A	G	ncRNA_exon	RP11-697E2.	.	.	.	A	G	+	+	0.58823529	7,0,10,0	chr15,90820	Cytosol	A > G
chr15:90820073:A:G:ncRNA_exoni	chr15	90820073	90820073	A	G	ncRNA_exon	RP11-697E2.	.	.	.	A	G	+	+	0.92857143	1,0,13,0	chr15,90820	Cytosol	A > G
chr15:90820886:A:G:downstream:	chr15	90820886	90820886	A	G	downstream	RP11-697E2.	.	.	.	A	G	+		0.05714286	33,0,2,0	chr15,90820	Cytosol	A > G
chr15:90820887:A:G:downstream:	chr15	90820887	90820887	A	G	downstream	RP11-697E2.	.	.	.	A	G	+		0.11428571	31,0,4,0	chr15,90820	Cytosol	A > G
chr15:93255256:T:C:ncRNA_intror	chr15	93255256	93255256	T	C	ncRNA_intro	RP11-386M2	.	.	.	T	C	+	-+	1	0,20,0,0	chr15,93255	Cytosol	T > C
chr15:93323807:T:C:ncRNA_exoni	chr15	93323807	93323807	T	C	ncRNA_exon	CTD-2313J17	.	.	.	A	G	-	-	0.22222222	7,0,2,0	chr15,93323	Cytosol	A > G
chr16:592641:A:G:intronic:CAPN1	chr16	592641	592641	A	G	intronic	CAPN15	.	.	.	A	G	+	+	1	0,0,9,0	chr16,59264	Cytosol	A > G
chr16:592643:A:G:intronic:CAPN1	chr16	592643	592643	A	G	intronic	CAPN15	.	.	.	A	G	+	+	1	0,0,9,0	chr16,59264	Cytosol	A > G
chr16:592667:A:G:intronic:CAPN1	chr16	592667	592667	A	G	intronic	CAPN15	.	.	.	A	G	+	+	0.63636364	4,0,7,0	chr16,59266	Cytosol	A > G
chr16:933925:T:C:ncRNA_exonic:L	chr16	933925	933925	T	C	ncRNA_exon	LA16c-306A4	.	.	.	A	G	-	-+	0.6	2,0,3,0	chr16,93392	Cytosol	A > G
chr16:934013:T:C:ncRNA_exonic:L	chr16	934013	934013	T	C	ncRNA_exon	LA16c-306A4	.	.	.	A	G	-	-+	0.6	2,0,3,0	chr16,93401	Cytosol	A > G
chr16:1987693:T:C:downstream:M	chr16	1987693	1987693	T	C	downstream	MSRB1	.	.	.	A	G	-	-	0.4	3,0,2,0	chr16,19876	Cytosol	A > G
chr16:2290760:T:C:ncRNA_exonic:	chr16	2290760	2290760	T	C	ncRNA_exon	RP11-304L19	.	.	.	A	G	-	-+	0.54545455	5,0,6,0	chr16,22907	Cytosol	A > G
chr16:4512245:T:C:intronic:NMRA	chr16	4512245	4512245	T	C	intronic	NMRAL1	.	.	.	A	G	-	-	0.36842105	36,0,21,0	chr16,45122	Cytosol	A > G
chr16:4512262:T:C:intronic:NMRA	chr16	4512262	4512262	T	C	intronic	NMRAL1	.	.	.	A	G	-	-	0.42857143	28,0,21,0	chr16,45122	Cytosol	A > G
chr16:4512351:T:C:intronic:NMRA	chr16	4512351	4512351	T	C	intronic	NMRAL1	.	.	.	A	G	-	-	0.33333333	32,0,16,0	chr16,45123	Cytosol	A > G

chr16:4512357:T:C:intronic:NMRA	chr16	4512357	4512357	T	C	intronic	NMRAL1	.	.	.	A	G	-	-	0.35294118	33,0,18,0	chr16,45123	Cytosol	A > G
chr16:4512396:T:C:intronic:NMRA	chr16	4512396	4512396	T	C	intronic	NMRAL1	.	.	.	A	G	-	-	0.15625	27,0,5,0	chr16,45123	Cytosol	A > G
chr16:4512397:T:C:intronic:NMRA	chr16	4512397	4512397	T	C	intronic	NMRAL1	.	.	.	A	G	-	-	0.06451613	29,0,2,0	chr16,45123	Cytosol	A > G
chr16:4512410:T:C:intronic:NMRA	chr16	4512410	4512410	T	C	intronic	NMRAL1	.	.	.	A	G	-	-	0.29032258	22,0,9,0	chr16,45124	Cytosol	A > G
chr16:4512466:T:C:intronic:NMRA	chr16	4512466	4512466	T	C	intronic	NMRAL1	.	.	.	A	G	-	-	0.32352941	23,0,11,0	chr16,45124	Cytosol	A > G
chr16:4512803:T:C:intronic:NMRA	chr16	4512803	4512803	T	C	intronic	NMRAL1	.	.	.	A	G	-	-	0.11111111	40,0,5,0	chr16,45128	Cytosol	A > G
chr16:4512810:T:C:intronic:NMRA	chr16	4512810	4512810	T	C	intronic	NMRAL1	.	.	.	A	G	-	-	0.68627451	16,0,35,0	chr16,45128	Cytosol	A > G
chr16:4512941:T:C:intronic:NMRA	chr16	4512941	4512941	T	C	intronic	NMRAL1	.	.	.	A	G	-	-	0.21276596	37,0,10,0	chr16,45129	Cytosol	A > G
chr16:4513093:T:C:intronic:NMRA	chr16	4513093	4513093	T	C	intronic	NMRAL1	.	.	.	A	G	-	-	0.11111111	16,0,2,0	chr16,45130	Cytosol	A > G
chr16:4513128:T:C:intronic:NMRA	chr16	4513128	4513128	T	C	intronic	NMRAL1	.	.	.	A	G	-	-	0.16666667	25,0,5,0	chr16,45131	Cytosol	A > G
chr16:4513142:T:C:intronic:NMRA	chr16	4513142	4513142	T	C	intronic	NMRAL1	.	.	.	A	G	-	-	0.5862069	12,0,17,0	chr16,45131	Cytosol	A > G
chr16:4800610:T:C:ncRNA_introni	chr16	4800610	4800610	T	C	ncRNA_intro	RP11-127I20	.	.	.	A	G	-	-	0.23529412	13,0,4,0	chr16,48006	Cytosol	A > G
chr16:4800834:T:C:ncRNA_introni	chr16	4800834	4800834	T	C	ncRNA_intro	RP11-127I20	.	.	.	A	G	-	-	0.13636364	19,0,3,0	chr16,48008	Cytosol	A > G
chr16:4801826:T:C:ncRNA_introni	chr16	4801826	4801826	T	C	ncRNA_intro	RP11-127I20	.	.	.	A	G	-	-	0.16	21,0,4,0	chr16,48018	Cytosol	A > G
chr16:4801873:T:C:ncRNA_introni	chr16	4801873	4801873	T	C	ncRNA_intro	RP11-127I20	.	.	.	A	G	-	-	0.23076923	10,0,3,0	chr16,48018	Cytosol	A > G
chr16:4801875:T:C:ncRNA_introni	chr16	4801875	4801875	T	C	ncRNA_intro	RP11-127I20	.	.	.	A	G	-	-	0.15384615	11,0,2,0	chr16,48018	Cytosol	A > G
chr16:5136527:A:G:intronic:FAM8	chr16	5136527	5136527	A	G	intronic	FAM86A	.	.	.	A	G	+	+-	1	0,0,14,0	chr16,51365	Cytosol	A > G
chr16:5136529:A:G:intronic:FAM8	chr16	5136529	5136529	A	G	intronic	FAM86A	.	.	.	A	G	+	+-	0.93333333	1,0,14,0	chr16,51365	Cytosol	A > G
chr16:9198071:A:G:intronic:C16or	chr16	9198071	9198071	A	G	intronic	C16orf72	.	.	.	A	G	+	+	0.20689655	23,0,6,0	chr16,91980	Cytosol	A > G
chr16:9198100:A:G:intronic:C16or	chr16	9198100	9198100	A	G	intronic	C16orf72	.	.	.	A	G	+	+	0.18181818	9,0,2,0	chr16,91980	Cytosol	A > G
chr16:9204662:A:G:ncRNA_exonic	chr16	9204662	9204662	A	G	ncRNA_exon	RP11-473I1.1	.	.	.	A	G	+	+	0.11764706	15,0,2,0	chr16,92046	Cytosol	A > G
chr16:9204745:A:G:ncRNA_exonic	chr16	9204745	9204745	A	G	ncRNA_exon	RP11-473I1.1	.	.	.	A	G	+	+	0.07142857	39,0,3,0	chr16,92047	Cytosol	A > G
chr16:9204748:A:G:ncRNA_exonic	chr16	9204748	9204748	A	G	ncRNA_exon	RP11-473I1.1	.	.	.	A	G	+	+	0.13953488	37,0,6,0	chr16,92047	Cytosol	A > G
chr16:9204784:A:G:ncRNA_exonic	chr16	9204784	9204784	A	G	ncRNA_exon	RP11-473I1.1	.	.	.	A	G	+	+	0.12903226	27,0,4,0	chr16,92047	Cytosol	A > G
chr16:9204788:A:G:ncRNA_exonic	chr16	9204788	9204788	A	G	ncRNA_exon	RP11-473I1.1	.	.	.	A	G	+	+	0.41935484	18,0,13,0	chr16,92047	Cytosol	A > G
chr16:9204827:A:G:ncRNA_exonic	chr16	9204827	9204827	A	G	ncRNA_exon	RP11-473I1.1	.	.	.	A	G	+	+	0.15789474	32,0,6,0	chr16,92048	Cytosol	A > G
chr16:9204836:A:G:ncRNA_exonic	chr16	9204836	9204836	A	G	ncRNA_exon	RP11-473I1.1	.	.	.	A	G	+	+	0.05128205	37,0,2,0	chr16,92048	Cytosol	A > G
chr16:9204847:A:G:ncRNA_exonic	chr16	9204847	9204847	A	G	ncRNA_exon	RP11-473I1.1	.	.	.	A	G	+	+	0.1025641	35,0,4,0	chr16,92048	Cytosol	A > G
chr16:9204883:A:G:ncRNA_exonic	chr16	9204883	9204883	A	G	ncRNA_exon	RP11-473I1.1	.	.	.	A	G	+	+	0.0952381	38,0,4,0	chr16,92048	Cytosol	A > G
chr16:9204886:A:G:ncRNA_exonic	chr16	9204886	9204886	A	G	ncRNA_exon	RP11-473I1.1	.	.	.	A	G	+	+	0.04878049	39,0,2,0	chr16,92048	Cytosol	A > G
chr16:10623531:T:C:ncRNA_exoni	chr16	10623531	10623531	T	C	ncRNA_exon	RP11-27M24	.	.	.	A	G	-	-+	0.18604651	35,0,8,0	chr16,10623	Cytosol	A > G
chr16:10623544:T:C:ncRNA_exoni	chr16	10623544	10623544	T	C	ncRNA_exon	RP11-27M24	.	.	.	A	G	-	-+	0.41176471	30,0,21,0	chr16,10623	Cytosol	A > G
chr16:10623566:T:C:ncRNA_intror	chr16	10623566	10623566	T	C	ncRNA_intro	RP11-27M24	.	.	.	A	G	-	-+	0.3375	53,0,27,0	chr16,10623	Cytosol	A > G
chr16:10623576:T:C:ncRNA_intror	chr16	10623576	10623576	T	C	ncRNA_intro	RP11-27M24	.	.	.	A	G	-	-+	0.18055556	59,0,13,0	chr16,10623	Cytosol	A > G
chr16:10623622:T:C:ncRNA_intror	chr16	10623622	10623622	T	C	ncRNA_intro	RP11-27M24	.	.	.	A	G	-	-+	0.38461538	48,0,30,0	chr16,10623	Cytosol	A > G
chr16:10623631:T:C:ncRNA_intror	chr16	10623631	10623631	T	C	ncRNA_intro	RP11-27M24	.	.	.	A	G	-	-+	0.06666667	70,0,5,0	chr16,10623	Cytosol	A > G
chr16:10623644:T:C:ncRNA_intror	chr16	10623644	10623644	T	C	ncRNA_intro	RP11-27M24	.	.	.	A	G	-	-+	0.07936508	58,0,5,0	chr16,10623	Cytosol	A > G
chr16:10625266:T:C:ncRNA_intror	chr16	10625266	10625266	T	C	ncRNA_intro	RP11-27M24	.	.	.	A	G	-	-+	0.31746032	43,0,20,0	chr16,10625	Cytosol	A > G
chr16:10625313:T:C:ncRNA_intror	chr16	10625313	10625313	T	C	ncRNA_intro	RP11-27M24	.	.	.	A	G	-	-+	0.23595506	68,0,21,0	chr16,10625	Cytosol	A > G
chr16:10625325:T:C:ncRNA_intror	chr16	10625325	10625325	T	C	ncRNA_intro	RP11-27M24	.	.	.	A	G	-	-+	0.20481928	66,0,17,0	chr16,10625	Cytosol	A > G
chr16:10625352:T:C:ncRNA_intror	chr16	10625352	10625352	T	C	ncRNA_intro	RP11-27M24	.	.	.	A	G	-	-+	0.19512195	33,0,8,0	chr16,10625	Cytosol	A > G
chr16:10625356:T:C:ncRNA_intror	chr16	10625356	10625356	T	C	ncRNA_intro	RP11-27M24	.	.	.	A	G	-	-+	0.14285714	36,0,6,0	chr16,10625	Cytosol	A > G
chr16:10625382:T:C:ncRNA_intror	chr16	10625382	10625382	T	C	ncRNA_intro	RP11-27M24	.	.	.	A	G	-	-+	0.30769231	27,0,12,0	chr16,10625	Cytosol	A > G
chr16:10625388:T:C:ncRNA_intror	chr16	10625388	10625388	T	C	ncRNA_intro	RP11-27M24	.	.	.	A	G	-	-+	0.3125	22,0,10,0	chr16,10625	Cytosol	A > G
chr16:10625406:T:C:ncRNA_intror	chr16	10625406	10625406	T	C	ncRNA_intro	RP11-27M24	.	.	.	A	G	-	-+	0.40540541	22,0,15,0	chr16,10625	Cytosol	A > G
chr16:10625414:T:C:ncRNA_intror	chr16	10625414	10625414	T	C	ncRNA_intro	RP11-27M24	.	.	.	A	G	-	-+	0.55	18,0,22,0	chr16,10625	Cytosol	A > G
chr16:10625437:T:C:ncRNA_intror	chr16	10625437	10625437	T	C	ncRNA_intro	RP11-27M24	.	.	.	A	G	-	-+	0.06818182	41,0,3,0	chr16,10625	Cytosol	A > G
chr16:10625460:T:C:ncRNA_intror	chr16	10625460	10625460	T	C	ncRNA_intro	RP11-27M24	.	.	.	A	G	-	-+	0.26373626	67,0,24,0	chr16,10625	Cytosol	A > G
chr16:11121211:T:C:intronic:CLEC	chr16	11121211	11121211	T	C	intronic	CLEC16A	.	.	.	T	C	+	+	1	0,25,0,0	chr16,11121	Cytosol	T > C
chr16:11819898:T:C:intronic:TXND	chr16	11819898	11819898	T	C	intronic	TXNDC11	.	.	.	A	G	-	-	0.57142857	3,0,4,0	chr16,11819	Cytosol	A > G
chr16:11928725:T:C:downstream:	chr16	11928725	11928725	T	C	downstream	RSL1D1	.	.	.	A	G	-	-	0.08928571	51,0,5,0	chr16,11928	Cytosol	A > G
chr16:11928736:T:C:downstream:	chr16	11928736	11928736	T	C	downstream	RSL1D1	.	.	.	A	G	-	-	0.12307692	57,0,8,0	chr16,11928	Cytosol	A > G
chr16:11928846:T:C:downstream:	chr16	11928846	11928846	T	C	downstream	RSL1D1	.	.	.	A	G	-	-	0.44444444	25,0,20,0	chr16,11928	Cytosol	A > G
chr16:11928859:T:C:downstream:	chr16	11928859	11928859	T	C	downstream	RSL1D1	.	.	.	A	G	-	-	0.10416667	43,0,5,0	chr16,11928	Cytosol	A > G
chr16:11928951:T:C:downstream:	chr16	11928951	11928951	T	C	downstream	RSL1D1	.	.	.	A	G	-	-	0.40540541	22,0,15,0	chr16,11928	Cytosol	A > G
chr16:11929176:T:C:UTR3:RSL1D1	chr16	11929176	11929176	T	C	UTR3	RSL1D1	ENST000005	.	.	A	G	-	-	0.5	4,0,4,0	chr16,11929	Cytosol	A > G
chr16:11929177:T:C:UTR3:RSL1D1	chr16	11929177	11929177	T	C	UTR3	RSL1D1	ENST000005	.	.	A	G	-	-	0.42857143	4,0,3,0	chr16,11929	Cytosol	A > G
chr16:11929203:T:C:UTR3:RSL1D1	chr16	11929203	11929203	T	C	UTR3	RSL1D1	ENST000005	.	.	A	G	-	-	0.2	12,0,3,0	chr16,11929	Cytosol	A > G
chr16:11929207:T:C:UTR3:RSL1D1	chr16	11929207	11929207	T	C	UTR3	RSL1D1	ENST000005	.	.	A	G	-	-	0.0952381	19,0,2,0	chr16,11929	Cytosol	A > G
chr16:11929447:T:C:UTR3:RSL1D1	chr16	11929447	11929447	T	C	UTR3	RSL1D1	ENST000005	.	.	A	G	-	-	0.06382979	44,0,3,0	chr16,11929	Cytosol	A > G
chr16:11931345:T:C:UTR3:RSL1D1	chr16	11931345	11931345	T	C	UTR3	RSL1D1	ENST000005	.	.	A	G	-	-	0.01714286	688,0,12,0	chr16,11931	Cytosol	A > G
chr16:11931346:T:C:UTR3:RSL1D1	chr16	11931346	11931346	T	C	UTR3	RSL1D1	ENST000005	.	.	A	G	-	-	0.01007194	688,0,7,0	chr16,11931	Cytosol	A > G
chr16:11965240:T:C:ncRNA_intror	chr16	11965240	11965240	T	C	ncRNA_intro	RP11-166B2.	.	.	.	A	G	-	+-	0.0375	77,0,3,0	chr16,11965	Cytosol	A > G
chr16:11965252:T:C:ncRNA_intror	chr16	11965252	11965252	T	C	ncRNA_intro	RP11-166B2.	.	.	.	A	G	-	+-	0.02439024	80,0,2,0	chr16,11965	Cytosol	A > G

chr16:11965262:T:C:ncRNA_intror	chr16	11965262	11965262	T	C	ncRNA_intro	RP11-166B2.	.	.	.	A	G	-	+-	0.02325581	84,0,2,0	chr16,11965	Cytosol	A > G
chr16:11965277:T:C:ncRNA_intror	chr16	11965277	11965277	T	C	ncRNA_intro	RP11-166B2.	.	.	.	A	G	-	+-	0.02631579	74,0,2,0	chr16,11965	Cytosol	A > G
chr16:15794083:A:G:UTR3:NDE1	chr16	15794083	15794083	A	G	UTR3	NDE1	ENST000005	.	.	A	G	+	+	0.06521739	43,0,3,0	chr16,15794	Cytosol	A > G
chr16:15794109:A:G:UTR3:NDE1	chr16	15794109	15794109	A	G	UTR3	NDE1	ENST000005	.	.	A	G	+	+	0.04761905	40,0,2,0	chr16,15794	Cytosol	A > G
chr16:15794136:A:G:UTR3:NDE1	chr16	15794136	15794136	A	G	UTR3	NDE1	ENST000005	.	.	A	G	+	+	0.05714286	33,0,2,0	chr16,15794	Cytosol	A > G
chr16:15794191:A:G:UTR3:NDE1	chr16	15794191	15794191	A	G	UTR3	NDE1	ENST000005	.	.	A	G	+	+	0.08108108	34,0,3,0	chr16,15794	Cytosol	A > G
chr16:15794373:A:G:intronic:NDE	chr16	15794373	15794373	A	G	intronic	NDE1	.	.	.	A	G	+	+	0.09302326	39,0,4,0	chr16,15794	Cytosol	A > G
chr16:15794426:A:G:intronic:NDE	chr16	15794426	15794426	A	G	intronic	NDE1	.	.	.	A	G	+	+	0.0625	30,0,2,0	chr16,15794	Cytosol	A > G
chr16:15794438:A:G:intronic:NDE	chr16	15794438	15794438	A	G	intronic	NDE1	.	.	.	A	G	+	+	0.11538462	23,0,3,0	chr16,15794	Cytosol	A > G
chr16:15794464:A:G:intronic:NDE	chr16	15794464	15794464	A	G	intronic	NDE1	.	.	.	A	G	+	+	0.5	7,0,7,0	chr16,15794	Cytosol	A > G
chr16:15794530:A:G:intronic:NDE	chr16	15794530	15794530	A	G	intronic	NDE1	.	.	.	A	G	+	+	0.2	16,0,4,0	chr16,15794	Cytosol	A > G
chr16:15794542:A:G:intronic:NDE	chr16	15794542	15794542	A	G	intronic	NDE1	.	.	.	A	G	+	+	0.5	7,0,7,0	chr16,15794	Cytosol	A > G
chr16:15794543:A:G:intronic:NDE	chr16	15794543	15794543	A	G	intronic	NDE1	.	.	.	A	G	+	+	0.66666667	5,0,10,0	chr16,15794	Cytosol	A > G
chr16:15794582:A:G:intronic:NDE	chr16	15794582	15794582	A	G	intronic	NDE1	.	.	.	A	G	+	+	0.11111111	24,0,3,0	chr16,15794	Cytosol	A > G
chr16:15794883:A:G:intronic:NDE	chr16	15794883	15794883	A	G	intronic	NDE1	.	.	.	A	G	+	+	0.14285714	12,0,2,0	chr16,15794	Cytosol	A > G
chr16:15794947:A:G:intronic:NDE	chr16	15794947	15794947	A	G	intronic	NDE1	.	.	.	A	G	+	+	0.23529412	13,0,4,0	chr16,15794	Cytosol	A > G
chr16:15794957:A:G:intronic:NDE	chr16	15794957	15794957	A	G	intronic	NDE1	.	.	.	A	G	+	+	0.21052632	15,0,4,0	chr16,15794	Cytosol	A > G
chr16:15795035:A:G:intronic:NDE	chr16	15795035	15795035	A	G	intronic	NDE1	.	.	.	A	G	+	+	0.25925926	20,0,7,0	chr16,15795	Cytosol	A > G
chr16:15795039:A:G:intronic:NDE	chr16	15795039	15795039	A	G	intronic	NDE1	.	.	.	A	G	+	+	0.35	13,0,7,0	chr16,15795	Cytosol	A > G
chr16:15795043:A:G:intronic:NDE	chr16	15795043	15795043	A	G	intronic	NDE1	.	.	.	A	G	+	+	0.45	11,0,9,0	chr16,15795	Cytosol	A > G
chr16:15795044:A:G:intronic:NDE	chr16	15795044	15795044	A	G	intronic	NDE1	.	.	.	A	G	+	+	0.27272727	16,0,6,0	chr16,15795	Cytosol	A > G
chr16:15795046:A:G:intronic:NDE	chr16	15795046	15795046	A	G	intronic	NDE1	.	.	.	A	G	+	+	0.0952381	19,0,2,0	chr16,15795	Cytosol	A > G
chr16:15821965:A:G:ncRNA_intro	chr16	15821965	15821965	A	G	ncRNA_intro	AF001548.5	.	.	.	A	G	+	-+	0.4	3,0,2,0	chr16,15821	Cytosol	A > G
chr16:19705976:T:C:intronic:C16o	chr16	19705976	19705976	T	C	intronic	C16orf62	.	.	.	A	G	-	+	0.83333333	1,0,5,0	chr16,19705	Cytosol	A > G
chr16:19713404:T:C:intronic:C16o	chr16	19713404	19713404	T	C	intronic	C16orf62	.	.	.	A	G	-	+	0.66666667	2,0,4,0	chr16,19713	Cytosol	A > G
chr16:19714058:T:C:intronic:C16o	chr16	19714058	19714058	T	C	intronic	C16orf62	.	.	.	A	G	-	+	0.7254902	14,0,37,0	chr16,19714	Cytosol	A > G
chr16:19714080:T:C:intronic:C16o	chr16	19714080	19714080	T	C	intronic	C16orf62	.	.	.	A	G	-	+	0.46511628	23,0,20,0	chr16,19714	Cytosol	A > G
chr16:19714147:T:C:intronic:C16o	chr16	19714147	19714147	T	C	intronic	C16orf62	.	.	.	A	G	-	+	0.41666667	7,0,5,0	chr16,19714	Cytosol	A > G
chr16:21191548:C:T:UTR3:TMEM1	chr16	21191548	21191548	C	T	UTR3	TMEM159	ENST000002	.	.	C	T	+	+	0.20833333	0,19,0,5	chr16,21191	Cytosol	C > T
chr16:22296860:A:G:UTR3:EEF2K	chr16	22296860	22296860	A	G	UTR3	EEF2K	ENST000002	.	.	A	G	+	+	0.48	39,0,36,0	chr16,22296	Cytosol	A > G
chr16:22296891:A:G:UTR3:EEF2K	chr16	22296891	22296891	A	G	UTR3	EEF2K	ENST000002	.	.	A	G	+	+	0.05333333	71,0,4,0	chr16,22296	Cytosol	A > G
chr16:22296938:A:G:UTR3:EEF2K	chr16	22296938	22296938	A	G	UTR3	EEF2K	ENST000002	.	.	A	G	+	+	0.08247423	89,0,8,0	chr16,22296	Cytosol	A > G
chr16:22297236:A:G:UTR3:EEF2K	chr16	22297236	22297236	A	G	UTR3	EEF2K	ENST000002	.	.	A	G	+	+	0.13043478	20,0,3,0	chr16,22297	Cytosol	A > G
chr16:22297277:A:G:UTR3:EEF2K	chr16	22297277	22297277	A	G	UTR3	EEF2K	ENST000002	.	.	A	G	+	+	0.07894737	35,0,3,0	chr16,22297	Cytosol	A > G
chr16:22299174:A:G:ncRNA_intro	chr16	22299174	22299174	A	G	ncRNA_intro	RP11-141O1	.	.	.	A	G	+	+-	0.35294118	11,0,6,0	chr16,22299	Cytosol	A > G
chr16:22299403:A:G:ncRNA_intro	chr16	22299403	22299403	A	G	ncRNA_intro	RP11-141O1	.	.	.	A	G	+	+-	0.28571429	5,0,2,0	chr16,22299	Cytosol	A > G
chr16:22299410:A:G:ncRNA_intro	chr16	22299410	22299410	A	G	ncRNA_intro	RP11-141O1	.	.	.	A	G	+	+-	0.71428571	2,0,5,0	chr16,22299	Cytosol	A > G
chr16:22299697:A:G:ncRNA_intro	chr16	22299697	22299697	A	G	ncRNA_intro	RP11-141O1	.	.	.	A	G	+	+-	0.18181818	9,0,2,0	chr16,22299	Cytosol	A > G
chr16:22299698:A:G:ncRNA_intro	chr16	22299698	22299698	A	G	ncRNA_intro	RP11-141O1	.	.	.	A	G	+	+-	0.2	8,0,2,0	chr16,22299	Cytosol	A > G
chr16:23400102:T:C:UTR3:COG7	chr16	23400102	23400102	T	C	UTR3	COG7	ENST000003	.	.	A	G	-	-	0.04411765	65,0,3,0	chr16,23400	Cytosol	A > G
chr16:23475282:T:C:UTR3:GGA2	chr16	23475282	23475282	T	C	UTR3	GGA2	ENST000003	.	.	A	G	-	-	0.55555556	4,0,5,0	chr16,23475	Cytosol	A > G
chr16:23476506:T:C:UTR3:GGA2	chr16	23476506	23476506	T	C	UTR3	GGA2	ENST000003	.	.	A	G	-	-	0.03278689	59,0,2,0	chr16,23476	Cytosol	A > G
chr16:23476581:T:C:UTR3:GGA2	chr16	23476581	23476581	T	C	UTR3	GGA2	ENST000003	.	.	A	G	-	-	0.375	30,0,18,0	chr16,23476	Cytosol	A > G
chr16:23524908:T:C:intronic:GGA	chr16	23524908	23524908	T	C	intronic	GGA2	.	.	.	A	G	-	-	1	0,0,5,0	chr16,23524	Cytosol	A > G
chr16:23526051:T:C:intronic:GGA	chr16	23526051	23526051	T	C	intronic	GGA2	.	.	.	A	G	-	-	0.55555556	4,0,5,0	chr16,23526	Cytosol	A > G
chr16:23684186:A:G:ncRNA_exoni	chr16	23684186	23684186	A	G	ncRNA_exon	CTD-2196E1	.	.	.	A	G	+	+	0.2	12,0,3,0	chr16,23684	Cytosol	A > G
chr16:23684193:A:G:ncRNA_exoni	chr16	23684193	23684193	A	G	ncRNA_exon	CTD-2196E1	.	.	.	A	G	+	+	0.13333333	13,0,2,0	chr16,23684	Cytosol	A > G
chr16:23684636:A:G:ncRNA_intro	chr16	23684636	23684636	A	G	ncRNA_intro	CTD-2196E1	.	.	.	A	G	+	+	0.88235294	4,0,30,0	chr16,23684	Cytosol	A > G
chr16:23684702:A:G:ncRNA_intro	chr16	23684702	23684702	A	G	ncRNA_intro	CTD-2196E1	.	.	.	A	G	+	+	0.06060606	31,0,2,0	chr16,23684	Cytosol	A > G
chr16:28966447:A:G:intronic:NFA	chr16	28966447	28966447	A	G	intronic	NFATC2IP	.	.	.	A	G	+	-+	1	0,0,5,0	chr16,28966	Cytosol	A > G
chr16:28966937:A:G:intronic:NFA	chr16	28966937	28966937	A	G	intronic	NFATC2IP	.	.	.	A	G	+	-+	0.28571429	5,0,2,0	chr16,28966	Cytosol	A > G
chr16:28967005:A:G:intronic:NFA	chr16	28967005	28967005	A	G	intronic	NFATC2IP	.	.	.	A	G	+	-+	0.4	3,0,2,0	chr16,28967	Cytosol	A > G
chr16:28969583:A:G:ncRNA_intro	chr16	28969583	28969583	A	G	ncRNA_intro	RP11-264B1	.	.	.	A	G	+	+-	0.4	3,0,2,0	chr16,28969	Cytosol	A > G
chr16:29941173:A:G:downstream:	chr16	29941173	29941173	A	G	downstream	CTD-2574D2	.	.	.	A	G	+	-	0.57142857	3,0,4,0	chr16,29941	Cytosol	A > G
chr16:30590009:T:C:ncRNA_intror	chr16	30590009	30590009	T	C	ncRNA_intro	AC002310.7	.	.	.	A	G	-	+-	0.3	7,0,3,0	chr16,30590	Cytosol	A > G
chr16:30590292:T:C:ncRNA_intror	chr16	30590292	30590292	T	C	ncRNA_intro	AC002310.7	.	.	.	A	G	-	+-	0.15384615	11,0,2,0	chr16,30590	Cytosol	A > G
chr16:30590901:T:C:ncRNA_intror	chr16	30590901	30590901	T	C	ncRNA_intro	AC002310.7	.	.	.	A	G	-	+-	0.09090909	20,0,2,0	chr16,30590	Cytosol	A > G
chr16:30593164:T:C:ncRNA_exoni	chr16	30593164	30593164	T	C	ncRNA_exon	AC002310.7	.	.	.	A	G	-	+-	0.2	8,0,2,0	chr16,30593	Cytosol	A > G
chr16:30931745:T:C:ncRNA_exoni	chr16	30931745	30931745	T	C	ncRNA_exon	FBXL19-AS1	.	.	.	A	G	-	-	0.3	7,0,3,0	chr16,30931	Cytosol	A > G
chr16:30931756:T:C:ncRNA_exoni	chr16	30931756	30931756	T	C	ncRNA_exon	FBXL19-AS1	.	.	.	A	G	-	-	0.46153846	7,0,6,0	chr16,30931	Cytosol	A > G
chr16:30931810:T:C:ncRNA_exoni	chr16	30931810	30931810	T	C	ncRNA_exon	FBXL19-AS1	.	.	.	A	G	-	-	0.91666667	1,0,11,0	chr16,30931	Cytosol	A > G
chr16:30932016:T:C:ncRNA_exoni	chr16	30932016	30932016	T	C	ncRNA_exon	FBXL19-AS1	.	.	.	A	G	-	-	0.42857143	4,0,3,0	chr16,30932	Cytosol	A > G
chr16:30932118:T:C:ncRNA_exoni	chr16	30932118	30932118	T	C	ncRNA_exon	FBXL19-AS1	.	.	.	A	G	-	-	0.375	5,0,3,0	chr16,30932	Cytosol	A > G
chr16:30932829:T:C:ncRNA_exoni	chr16	30932829	30932829	T	C	ncRNA_exon	FBXL19-AS1	.	.	.	A	G	-	-	0.6	4,0,6,0	chr16,30932	Cytosol	A > G

chr16:31074633:T:C:intronic:ZNF6	chr16	31074633	31074633	T	C	intronic	ZNF668	.	.	.	A	G	-	-	0.42857143	4,0,3,0	chr16,31074	Cytosol	A > G
chr16:31132575:A:G:intronic:KAT8	chr16	31132575	31132575	A	G	intronic	KAT8	.	.	.	A	G	+	+	0.14754098	52,0,9,0	chr16,31132	Cytosol	A > G
chr16:31132581:A:G:intronic:KAT8	chr16	31132581	31132581	A	G	intronic	KAT8	.	.	.	A	G	+	+	0.03333333	58,0,2,0	chr16,31132	Cytosol	A > G
chr16:31132676:A:G:intronic:KAT8	chr16	31132676	31132676	A	G	intronic	KAT8	.	.	.	A	G	+	+	0.16666667	15,0,3,0	chr16,31132	Cytosol	A > G
chr16:31133202:A:G:intronic:KAT8	chr16	31133202	31133202	A	G	intronic	KAT8	.	.	.	A	G	+	+	0.12121212	29,0,4,0	chr16,31133	Cytosol	A > G
chr16:31133234:A:G:intronic:KAT8	chr16	31133234	31133234	A	G	intronic	KAT8	.	.	.	A	G	+	+	0.07692308	48,0,4,0	chr16,31133	Cytosol	A > G
chr16:31133246:A:G:intronic:KAT8	chr16	31133246	31133246	A	G	intronic	KAT8	.	.	.	A	G	+	+	0.04	48,0,2,0	chr16,31133	Cytosol	A > G
chr16:31133286:A:G:intronic:KAT8	chr16	31133286	31133286	A	G	intronic	KAT8	.	.	.	A	G	+	+	0.08510638	43,0,4,0	chr16,31133	Cytosol	A > G
chr16:31133324:A:G:intronic:KAT8	chr16	31133324	31133324	A	G	intronic	KAT8	.	.	.	A	G	+	+	0.41304348	27,0,19,0	chr16,31133	Cytosol	A > G
chr16:31133334:A:G:intronic:KAT8	chr16	31133334	31133334	A	G	intronic	KAT8	.	.	.	A	G	+	+	0.09090909	30,0,3,0	chr16,31133	Cytosol	A > G
chr16:31133839:A:G:intronic:KAT8	chr16	31133839	31133839	A	G	intronic	KAT8	.	.	.	A	G	+	+	0.51162791	21,0,22,0	chr16,31133	Cytosol	A > G
chr16:31133910:A:G:intronic:KAT8	chr16	31133910	31133910	A	G	intronic	KAT8	.	.	.	A	G	+	+	0.21428571	11,0,3,0	chr16,31133	Cytosol	A > G
chr16:31133919:A:G:intronic:KAT8	chr16	31133919	31133919	A	G	intronic	KAT8	.	.	.	A	G	+	+	0.58333333	5,0,7,0	chr16,31133	Cytosol	A > G
chr16:31133945:A:G:intronic:KAT8	chr16	31133945	31133945	A	G	intronic	KAT8	.	.	.	A	G	+	+	0.7	3,0,7,0	chr16,31133	Cytosol	A > G
chr16:31133946:A:G:intronic:KAT8	chr16	31133946	31133946	A	G	intronic	KAT8	.	.	.	A	G	+	+	0.5	5,0,5,0	chr16,31133	Cytosol	A > G
chr16:31133967:A:G:intronic:KAT8	chr16	31133967	31133967	A	G	intronic	KAT8	.	.	.	A	G	+	+	0.4	3,0,2,0	chr16,31133	Cytosol	A > G
chr16:31134013:A:G:intronic:KAT8	chr16	31134013	31134013	A	G	intronic	KAT8	.	.	.	A	G	+	+	0.15384615	11,0,2,0	chr16,31134	Cytosol	A > G
chr16:31134015:A:G:intronic:KAT8	chr16	31134015	31134015	A	G	intronic	KAT8	.	.	.	A	G	+	+	0.30769231	9,0,4,0	chr16,31134	Cytosol	A > G
chr16:31134055:A:G:intronic:KAT8	chr16	31134055	31134055	A	G	intronic	KAT8	.	.	.	A	G	+	+	0.33333333	8,0,4,0	chr16,31134	Cytosol	A > G
chr16:31134081:A:G:intronic:KAT8	chr16	31134081	31134081	A	G	intronic	KAT8	.	.	.	A	G	+	+	0.8	2,0,8,0	chr16,31134	Cytosol	A > G
chr16:31134087:A:G:intronic:KAT8	chr16	31134087	31134087	A	G	intronic	KAT8	.	.	.	A	G	+	+	0.44444444	5,0,4,0	chr16,31134	Cytosol	A > G
chr16:31134122:A:G:intronic:KAT8	chr16	31134122	31134122	A	G	intronic	KAT8	.	.	.	A	G	+	+	0.14285714	12,0,2,0	chr16,31134	Cytosol	A > G
chr16:31134123:A:G:intronic:KAT8	chr16	31134123	31134123	A	G	intronic	KAT8	.	.	.	A	G	+	+	0.54545455	5,0,6,0	chr16,31134	Cytosol	A > G
chr16:31134247:A:G:intronic:KAT8	chr16	31134247	31134247	A	G	intronic	KAT8	.	.	.	A	G	+	+	0.63636364	4,0,7,0	chr16,31134	Cytosol	A > G
chr16:31134254:A:G:intronic:KAT8	chr16	31134254	31134254	A	G	intronic	KAT8	.	.	.	A	G	+	+	0.2	8,0,2,0	chr16,31134	Cytosol	A > G
chr16:31134287:A:G:intronic:KAT8	chr16	31134287	31134287	A	G	intronic	KAT8	.	.	.	A	G	+	+	0.57142857	6,0,8,0	chr16,31134	Cytosol	A > G
chr16:31134317:A:G:intronic:KAT8	chr16	31134317	31134317	A	G	intronic	KAT8	.	.	.	A	G	+	+	0.55555556	4,0,5,0	chr16,31134	Cytosol	A > G
chr16:31134357:A:G:intronic:KAT8	chr16	31134357	31134357	A	G	intronic	KAT8	.	.	.	A	G	+	+	0.44444444	5,0,4,0	chr16,31134	Cytosol	A > G
chr16:31134561:A:G:intronic:KAT8	chr16	31134561	31134561	A	G	intronic	KAT8	.	.	.	A	G	+	+	0.15384615	11,0,2,0	chr16,31134	Cytosol	A > G
chr16:31134607:A:G:intronic:KAT8	chr16	31134607	31134607	A	G	intronic	KAT8	.	.	.	A	G	+	+	0.61538462	5,0,8,0	chr16,31134	Cytosol	A > G
chr16:31134631:A:G:intronic:KAT8	chr16	31134631	31134631	A	G	intronic	KAT8	.	.	.	A	G	+	+	0.66666667	8,0,16,0	chr16,31134	Cytosol	A > G
chr16:31134677:A:G:intronic:KAT8	chr16	31134677	31134677	A	G	intronic	KAT8	.	.	.	A	G	+	+	0.25	18,0,6,0	chr16,31134	Cytosol	A > G
chr16:31134703:A:G:intronic:KAT8	chr16	31134703	31134703	A	G	intronic	KAT8	.	.	.	A	G	+	+	0.4375	9,0,7,0	chr16,31134	Cytosol	A > G
chr16:31134727:A:G:intronic:KAT8	chr16	31134727	31134727	A	G	intronic	KAT8	.	.	.	A	G	+	+	0.33333333	18,0,9,0	chr16,31134	Cytosol	A > G
chr16:31134791:A:G:intronic:KAT8	chr16	31134791	31134791	A	G	intronic	KAT8	.	.	.	A	G	+	+	0.4	3,0,2,0	chr16,31134	Cytosol	A > G
chr16:31134792:A:G:intronic:KAT8	chr16	31134792	31134792	A	G	intronic	KAT8	.	.	.	A	G	+	+	0.83333333	1,0,5,0	chr16,31134	Cytosol	A > G
chr16:31134851:A:G:intronic:KAT8	chr16	31134851	31134851	A	G	intronic	KAT8	.	.	.	A	G	+	+	0.7	3,0,7,0	chr16,31134	Cytosol	A > G
chr16:31134881:A:G:intronic:KAT8	chr16	31134881	31134881	A	G	intronic	KAT8	.	.	.	A	G	+	+	0.22222222	14,0,4,0	chr16,31134	Cytosol	A > G
chr16:31134928:A:G:intronic:KAT8	chr16	31134928	31134928	A	G	intronic	KAT8	.	.	.	A	G	+	+	0.08695652	21,0,2,0	chr16,31134	Cytosol	A > G
chr16:31134932:A:G:intronic:KAT8	chr16	31134932	31134932	A	G	intronic	KAT8	.	.	.	A	G	+	+	0.17857143	23,0,5,0	chr16,31134	Cytosol	A > G
chr16:31134953:A:G:intronic:KAT8	chr16	31134953	31134953	A	G	intronic	KAT8	.	.	.	A	G	+	+	0.16666667	20,0,4,0	chr16,31134	Cytosol	A > G
chr16:31134954:A:G:intronic:KAT8	chr16	31134954	31134954	A	G	intronic	KAT8	.	.	.	A	G	+	+	0.16	21,0,4,0	chr16,31134	Cytosol	A > G
chr16:31134962:A:G:intronic:KAT8	chr16	31134962	31134962	A	G	intronic	KAT8	.	.	.	A	G	+	+	0.57894737	8,0,11,0	chr16,31134	Cytosol	A > G
chr16:31134967:A:G:intronic:KAT8	chr16	31134967	31134967	A	G	intronic	KAT8	.	.	.	A	G	+	+	0.45	11,0,9,0	chr16,31134	Cytosol	A > G
chr16:31135016:A:G:intronic:KAT8	chr16	31135016	31135016	A	G	intronic	KAT8	.	.	.	A	G	+	+	0.42857143	8,0,6,0	chr16,31135	Cytosol	A > G
chr16:31135042:A:G:intronic:KAT8	chr16	31135042	31135042	A	G	intronic	KAT8	.	.	.	A	G	+	+	0.21428571	11,0,3,0	chr16,31135	Cytosol	A > G
chr16:31135067:A:G:intronic:KAT8	chr16	31135067	31135067	A	G	intronic	KAT8	.	.	.	A	G	+	+	0.08695652	21,0,2,0	chr16,31135	Cytosol	A > G
chr16:31135068:A:G:intronic:KAT8	chr16	31135068	31135068	A	G	intronic	KAT8	.	.	.	A	G	+	+	0.14285714	18,0,3,0	chr16,31135	Cytosol	A > G
chr16:31135094:A:G:intronic:KAT8	chr16	31135094	31135094	A	G	intronic	KAT8	.	.	.	A	G	+	+	0.33333333	16,0,8,0	chr16,31135	Cytosol	A > G
chr16:31135095:A:G:intronic:KAT8	chr16	31135095	31135095	A	G	intronic	KAT8	.	.	.	A	G	+	+	0.44	14,0,11,0	chr16,31135	Cytosol	A > G
chr16:31135107:A:G:intronic:KAT8	chr16	31135107	31135107	A	G	intronic	KAT8	.	.	.	A	G	+	+	0.52380952	10,0,11,0	chr16,31135	Cytosol	A > G
chr16:31463648:T:C:ncRNA_intror	chr16	31463648	31463648	T	C	ncRNA_intro	RP11-452L6.	.	.	.	A	G	-	-	1	0,0,5,0	chr16,31463	Cytosol	A > G
chr16:31464383:T:C:ncRNA_intror	chr16	31464383	31464383	T	C	ncRNA_intro	RP11-452L6.	.	.	.	A	G	-	-	0.28571429	5,0,2,0	chr16,31464	Cytosol	A > G
chr16:46731906:A:G:UTR3:ORC6	chr16	46731906	46731906	A	G	UTR3	ORC6	ENST000002	.	.	A	G	+	+	0.02264151	259,0,6,0	chr16,46731	Cytosol	A > G
chr16:46831411:T:C:UTR3:C16orf8	chr16	46831411	46831411	T	C	UTR3	C16orf87	ENST000003	.	.	A	G	-	-	0.66666667	2,0,4,0	chr16,46831	Cytosol	A > G
chr16:46831434:T:C:UTR3:C16orf8	chr16	46831434	46831434	T	C	UTR3	C16orf87	ENST000003	.	.	A	G	-	-	0.38461538	8,0,5,0	chr16,46831	Cytosol	A > G
chr16:46831455:T:C:UTR3:C16orf8	chr16	46831455	46831455	T	C	UTR3	C16orf87	ENST000003	.	.	A	G	-	-	0.23529412	13,0,4,0	chr16,46831	Cytosol	A > G
chr16:46831493:T:C:UTR3:C16orf8	chr16	46831493	46831493	T	C	UTR3	C16orf87	ENST000003	.	.	A	G	-	-	0.28571429	10,0,4,0	chr16,46831	Cytosol	A > G
chr16:46831531:T:C:UTR3:C16orf8	chr16	46831531	46831531	T	C	UTR3	C16orf87	ENST000003	.	.	A	G	-	-	0.16666667	10,0,2,0	chr16,46831	Cytosol	A > G
chr16:47190102:T:C:ncRNA_intror	chr16	47190102	47190102	T	C	ncRNA_intro	RP11-329J18	.	.	.	A	G	-	+-	0.6	2,0,3,0	chr16,47190	Cytosol	A > G
chr16:47190193:T:C:ncRNA_intror	chr16	47190193	47190193	T	C	ncRNA_intro	RP11-329J18	.	.	.	A	G	-	+-	1	0,0,7,0	chr16,47190	Cytosol	A > G
chr16:48386295:A:G:UTR3:LONP2	chr16	48386295	48386295	A	G	UTR3	LONP2	ENST000002	.	.	A	G	+	+	0.2	76,0,19,0	chr16,48386	Cytosol	A > G
chr16:48386302:A:G:UTR3:LONP2	chr16	48386302	48386302	A	G	UTR3	LONP2	ENST000002	.	.	A	G	+	+	0.07843137	94,0,8,0	chr16,48386	Cytosol	A > G
chr16:48386424:A:G:UTR3:LONP2	chr16	48386424	48386424	A	G	UTR3	LONP2	ENST000002	.	.	A	G	+	+	0.30188679	37,0,16,0	chr16,48386	Cytosol	A > G

chr16:48388180:A:G:UTR3:LONP2	chr16	48388180	48388180	A	G	UTR3	LONP2	ENST000002	.	.	A	G	+	+	0.69565217	7,0,16,0	chr16,48388	Cytosol	A > G
chr16:48388218:A:G:UTR3:LONP2	chr16	48388218	48388218	A	G	UTR3	LONP2	ENST000002	.	.	A	G	+	+	0.07692308	24,0,2,0	chr16,48388	Cytosol	A > G
chr16:48388219:A:G:UTR3:LONP2	chr16	48388219	48388219	A	G	UTR3	LONP2	ENST000002	.	.	A	G	+	+	0.26923077	19,0,7,0	chr16,48388	Cytosol	A > G
chr16:48388245:A:G:UTR3:LONP2	chr16	48388245	48388245	A	G	UTR3	LONP2	ENST000002	.	.	A	G	+	+	0.625	6,0,10,0	chr16,48388	Cytosol	A > G
chr16:48388248:A:G:UTR3:LONP2	chr16	48388248	48388248	A	G	UTR3	LONP2	ENST000002	.	.	A	G	+	+	0.5	8,0,8,0	chr16,48388	Cytosol	A > G
chr16:48388258:A:G:UTR3:LONP2	chr16	48388258	48388258	A	G	UTR3	LONP2	ENST000002	.	.	A	G	+	+	0.42857143	12,0,9,0	chr16,48388	Cytosol	A > G
chr16:48388260:A:G:UTR3:LONP2	chr16	48388260	48388260	A	G	UTR3	LONP2	ENST000002	.	.	A	G	+	+	0.16666667	20,0,4,0	chr16,48388	Cytosol	A > G
chr16:48388303:A:G:UTR3:LONP2	chr16	48388303	48388303	A	G	UTR3	LONP2	ENST000002	.	.	A	G	+	+	0.07142857	52,0,4,0	chr16,48388	Cytosol	A > G
chr16:48388309:A:G:UTR3:LONP2	chr16	48388309	48388309	A	G	UTR3	LONP2	ENST000002	.	.	A	G	+	+	0.16981132	44,0,9,0	chr16,48388	Cytosol	A > G
chr16:48388317:A:G:UTR3:LONP2	chr16	48388317	48388317	A	G	UTR3	LONP2	ENST000002	.	.	A	G	+	+	0.28070175	41,0,16,0	chr16,48388	Cytosol	A > G
chr16:48388340:A:G:UTR3:LONP2	chr16	48388340	48388340	A	G	UTR3	LONP2	ENST000002	.	.	A	G	+	+	0.13513514	64,0,10,0	chr16,48388	Cytosol	A > G
chr16:48388348:A:G:UTR3:LONP2	chr16	48388348	48388348	A	G	UTR3	LONP2	ENST000002	.	.	A	G	+	+	0.03896104	74,0,3,0	chr16,48388	Cytosol	A > G
chr16:48388354:A:G:UTR3:LONP2	chr16	48388354	48388354	A	G	UTR3	LONP2	ENST000002	.	.	A	G	+	+	0.0625	60,0,4,0	chr16,48388	Cytosol	A > G
chr16:48388356:A:G:UTR3:LONP2	chr16	48388356	48388356	A	G	UTR3	LONP2	ENST000002	.	.	A	G	+	+	0.12903226	54,0,8,0	chr16,48388	Cytosol	A > G
chr16:48388380:A:G:UTR3:LONP2	chr16	48388380	48388380	A	G	UTR3	LONP2	ENST000002	.	.	A	G	+	+	0.34666667	49,0,26,0	chr16,48388	Cytosol	A > G
chr16:48388381:A:G:UTR3:LONP2	chr16	48388381	48388381	A	G	UTR3	LONP2	ENST000002	.	.	A	G	+	+	0.2027027	59,0,15,0	chr16,48388	Cytosol	A > G
chr16:48388382:A:G:UTR3:LONP2	chr16	48388382	48388382	A	G	UTR3	LONP2	ENST000002	.	.	A	G	+	+	0.31944444	49,0,23,0	chr16,48388	Cytosol	A > G
chr16:48388383:A:G:UTR3:LONP2	chr16	48388383	48388383	A	G	UTR3	LONP2	ENST000002	.	.	A	G	+	+	0.375	45,0,27,0	chr16,48388	Cytosol	A > G
chr16:48388394:A:G:UTR3:LONP2	chr16	48388394	48388394	A	G	UTR3	LONP2	ENST000002	.	.	A	G	+	+	0.34666667	49,0,26,0	chr16,48388	Cytosol	A > G
chr16:48388403:A:G:UTR3:LONP2	chr16	48388403	48388403	A	G	UTR3	LONP2	ENST000002	.	.	A	G	+	+	0.02777778	70,0,2,0	chr16,48388	Cytosol	A > G
chr16:50139117:A:G:ncRNA_intro	chr16	50139117	50139117	A	G	ncRNA_intro	RP11-429P3.	.	.	.	A	G	+	+-	0.18181818	9,0,2,0	chr16,50139	Cytosol	A > G
chr16:50139139:A:G:ncRNA_intro	chr16	50139139	50139139	A	G	ncRNA_intro	RP11-429P3.	.	.	.	A	G	+	+-	0.57142857	3,0,4,0	chr16,50139	Cytosol	A > G
chr16:50139194:A:G:ncRNA_intro	chr16	50139194	50139194	A	G	ncRNA_intro	RP11-429P3.	.	.	.	A	G	+	+-	0.71428571	2,0,5,0	chr16,50139	Cytosol	A > G
chr16:50139294:A:G:ncRNA_intro	chr16	50139294	50139294	A	G	ncRNA_intro	RP11-429P3.	.	.	.	A	G	+	+-	0.10447761	60,0,7,0	chr16,50139	Cytosol	A > G
chr16:50139328:A:G:ncRNA_intro	chr16	50139328	50139328	A	G	ncRNA_intro	RP11-429P3.	.	.	.	A	G	+	+-	0.5	15,0,15,0	chr16,50139	Cytosol	A > G
chr16:50139483:A:G:ncRNA_intro	chr16	50139483	50139483	A	G	ncRNA_intro	RP11-429P3.	.	.	.	A	G	+	+-	0.19512195	99,0,24,0	chr16,50139	Cytosol	A > G
chr16:50139515:A:G:ncRNA_intro	chr16	50139515	50139515	A	G	ncRNA_intro	RP11-429P3.	.	.	.	A	G	+	+-	0.53503185	73,0,84,0	chr16,50139	Cytosol	A > G
chr16:50139517:A:G:ncRNA_intro	chr16	50139517	50139517	A	G	ncRNA_intro	RP11-429P3.	.	.	.	A	G	+	+-	0.03289474	147,0,5,0	chr16,50139	Cytosol	A > G
chr16:50139562:A:G:ncRNA_intro	chr16	50139562	50139562	A	G	ncRNA_intro	RP11-429P3.	.	.	.	A	G	+	+-	0.23300971	79,0,24,0	chr16,50139	Cytosol	A > G
chr16:50139563:A:G:ncRNA_intro	chr16	50139563	50139563	A	G	ncRNA_intro	RP11-429P3.	.	.	.	A	G	+	+-	0.36734694	62,0,36,0	chr16,50139	Cytosol	A > G
chr16:50139580:A:G:ncRNA_intro	chr16	50139580	50139580	A	G	ncRNA_intro	RP11-429P3.	.	.	.	A	G	+	+-	0.26136364	65,0,23,0	chr16,50139	Cytosol	A > G
chr16:50139592:A:G:ncRNA_intro	chr16	50139592	50139592	A	G	ncRNA_intro	RP11-429P3.	.	.	.	A	G	+	+-	0.25301205	62,0,21,0	chr16,50139	Cytosol	A > G
chr16:50139702:A:G:ncRNA_intro	chr16	50139702	50139702	A	G	ncRNA_intro	RP11-429P3.	.	.	.	A	G	+	+-	0.76470588	4,0,13,0	chr16,50139	Cytosol	A > G
chr16:50139716:A:G:ncRNA_intro	chr16	50139716	50139716	A	G	ncRNA_intro	RP11-429P3.	.	.	.	A	G	+	+-	0.3125	11,0,5,0	chr16,50139	Cytosol	A > G
chr16:50139717:A:G:ncRNA_intro	chr16	50139717	50139717	A	G	ncRNA_intro	RP11-429P3.	.	.	.	A	G	+	+-	0.5625	7,0,9,0	chr16,50139	Cytosol	A > G
chr16:50139744:A:G:ncRNA_intro	chr16	50139744	50139744	A	G	ncRNA_intro	RP11-429P3.	.	.	.	A	G	+	+-	0.94117647	1,0,16,0	chr16,50139	Cytosol	A > G
chr16:50139756:A:G:ncRNA_intro	chr16	50139756	50139756	A	G	ncRNA_intro	RP11-429P3.	.	.	.	A	G	+	-+	0.58333333	10,0,14,0	chr16,50139	Cytosol	A > G
chr16:50139762:A:G:ncRNA_intro	chr16	50139762	50139762	A	G	ncRNA_intro	RP11-429P3.	.	.	.	A	G	+	-+	0.66666667	7,0,14,0	chr16,50139	Cytosol	A > G
chr16:54147789:A:G:UTR3:FTO	chr16	54147789	54147789	A	G	UTR3	FTO	ENST000004	.	.	A	G	+	+	0.125	28,0,4,0	chr16,54147	Cytosol	A > G
chr16:54147859:A:G:UTR3:FTO	chr16	54147859	54147859	A	G	UTR3	FTO	ENST000004	.	.	A	G	+	+	0.03773585	51,0,2,0	chr16,54147	Cytosol	A > G
chr16:57208123:T:C:UTR5:FAM19	chr16	57208123	57208123	T	C	UTR5	FAM192A	ENST000005	.	.	A	G	-	-	0.3	7,0,3,0	chr16,57208	Cytosol	A > G
chr16:66921587:A:G:UTR3:PDP2	chr16	66921587	66921587	A	G	UTR3	PDP2	ENST000003	.	.	A	G	+	+	0.46666667	16,0,14,0	chr16,66921	Cytosol	A > G
chr16:66921601:A:G:UTR3:PDP2	chr16	66921601	66921601	A	G	UTR3	PDP2	ENST000003	.	.	A	G	+	+	0.46153846	14,0,12,0	chr16,66921	Cytosol	A > G
chr16:66922201:A:G:ncRNA_exoni	chr16	66922201	66922201	A	G	ncRNA_exon	RP11-61A14.	.	.	.	A	G	+	+	0.6	4,0,6,0	chr16,66922	Cytosol	A > G
chr16:66922246:A:G:ncRNA_exoni	chr16	66922246	66922246	A	G	ncRNA_exon	RP11-61A14.	.	.	.	A	G	+	+	0.41666667	14,0,10,0	chr16,66922	Cytosol	A > G
chr16:66923235:A:G:ncRNA_exoni	chr16	66923235	66923235	A	G	ncRNA_exon	RP11-61A14.	.	.	.	A	G	+	+	0.58333333	5,0,7,0	chr16,66923	Cytosol	A > G
chr16:66923259:A:G:ncRNA_exoni	chr16	66923259	66923259	A	G	ncRNA_exon	RP11-61A14.	.	.	.	A	G	+	+	0.2173913	18,0,5,0	chr16,66923	Cytosol	A > G
chr16:66923263:A:G:ncRNA_exoni	chr16	66923263	66923263	A	G	ncRNA_exon	RP11-61A14.	.	.	.	A	G	+	+	0.17391304	19,0,4,0	chr16,66923	Cytosol	A > G
chr16:66923265:A:G:ncRNA_exoni	chr16	66923265	66923265	A	G	ncRNA_exon	RP11-61A14.	.	.	.	A	G	+	+	0.07407407	25,0,2,0	chr16,66923	Cytosol	A > G
chr16:66923267:A:G:ncRNA_exoni	chr16	66923267	66923267	A	G	ncRNA_exon	RP11-61A14.	.	.	.	A	G	+	+	0.42307692	15,0,11,0	chr16,66923	Cytosol	A > G
chr16:66923297:A:G:ncRNA_exoni	chr16	66923297	66923297	A	G	ncRNA_exon	RP11-61A14.	.	.	.	A	G	+	+	0.53333333	14,0,16,0	chr16,66923	Cytosol	A > G
chr16:66923303:A:G:ncRNA_exoni	chr16	66923303	66923303	A	G	ncRNA_exon	RP11-61A14.	.	.	.	A	G	+	+	0.32258065	21,0,10,0	chr16,66923	Cytosol	A > G
chr16:66923327:A:G:ncRNA_exoni	chr16	66923327	66923327	A	G	ncRNA_exon	RP11-61A14.	.	.	.	A	G	+	+	0.25806452	23,0,8,0	chr16,66923	Cytosol	A > G
chr16:66923332:A:G:ncRNA_exoni	chr16	66923332	66923332	A	G	ncRNA_exon	RP11-61A14.	.	.	.	A	G	+	+	0.56666667	13,0,17,0	chr16,66923	Cytosol	A > G
chr16:66923384:A:G:ncRNA_exoni	chr16	66923384	66923384	A	G	ncRNA_exon	RP11-61A14.	.	.	.	A	G	+	+	0.47058824	9,0,8,0	chr16,66923	Cytosol	A > G
chr16:66923387:A:G:ncRNA_exoni	chr16	66923387	66923387	A	G	ncRNA_exon	RP11-61A14.	.	.	.	A	G	+	+	0.45454545	6,0,5,0	chr16,66923	Cytosol	A > G
chr16:66923393:A:G:ncRNA_exoni	chr16	66923393	66923393	A	G	ncRNA_exon	RP11-61A14.	.	.	.	A	G	+	+	0.41666667	7,0,5,0	chr16,66923	Cytosol	A > G
chr16:66923401:A:G:ncRNA_exoni	chr16	66923401	66923401	A	G	ncRNA_exon	RP11-61A14.	.	.	.	A	G	+	+	0.33333333	10,0,5,0	chr16,66923	Cytosol	A > G
chr16:66923429:A:G:ncRNA_exoni	chr16	66923429	66923429	A	G	ncRNA_exon	RP11-61A14.	.	.	.	A	G	+	+	0.06666667	28,0,2,0	chr16,66923	Cytosol	A > G
chr16:66923435:A:G:ncRNA_exoni	chr16	66923435	66923435	A	G	ncRNA_exon	RP11-61A14.	.	.	.	A	G	+	+	0.14285714	30,0,5,0	chr16,66923	Cytosol	A > G
chr16:66923447:A:G:ncRNA_exoni	chr16	66923447	66923447	A	G	ncRNA_exon	RP11-61A14.	.	.	.	A	G	+	+	0.08571429	32,0,3,0	chr16,66923	Cytosol	A > G
chr16:66923452:A:G:ncRNA_exoni	chr16	66923452	66923452	A	G	ncRNA_exon	RP11-61A14.	.	.	.	A	G	+	+	0.09756098	37,0,4,0	chr16,66923	Cytosol	A > G
chr16:66923468:A:G:ncRNA_exoni	chr16	66923468	66923468	A	G	ncRNA_exon	RP11-61A14.	.	.	.	A	G	+	+	0.44444444	20,0,16,0	chr16,66923	Cytosol	A > G
chr16:66923759:A:G:ncRNA_exoni	chr16	66923759	66923759	A	G	ncRNA_exon	RP11-61A14.	.	.	.	A	G	+	+	0.10810811	33,0,4,0	chr16,66923	Cytosol	A > G

chr16:66923766:A:G:ncRNA_exoni	chr16	66923766	66923766	A	G	ncRNA_exon	RP11-61A14.	.	.	.	A	G	+	+	0.2972973	26,0,11,0	chr16,66923	Cytosol	A > G
chr16:66923767:A:G:ncRNA_exoni	chr16	66923767	66923767	A	G	ncRNA_exon	RP11-61A14.	.	.	.	A	G	+	+	0.08571429	32,0,3,0	chr16,66923	Cytosol	A > G
chr16:66923770:A:G:ncRNA_exoni	chr16	66923770	66923770	A	G	ncRNA_exon	RP11-61A14.	.	.	.	A	G	+	+	0.05714286	33,0,2,0	chr16,66923	Cytosol	A > G
chr16:66923950:A:G:ncRNA_exoni	chr16	66923950	66923950	A	G	ncRNA_exon	RP11-61A14.	.	.	.	A	G	+	+	0.06666667	28,0,2,0	chr16,66923	Cytosol	A > G
chr16:66924004:A:G:ncRNA_exoni	chr16	66924004	66924004	A	G	ncRNA_exon	RP11-61A14.	.	.	.	A	G	+	+	0.85	3,0,17,0	chr16,66924	Cytosol	A > G
chr16:66924005:A:G:ncRNA_exoni	chr16	66924005	66924005	A	G	ncRNA_exon	RP11-61A14.	.	.	.	A	G	+	+	0.5	10,0,10,0	chr16,66924	Cytosol	A > G
chr16:66924012:A:G:ncRNA_exoni	chr16	66924012	66924012	A	G	ncRNA_exon	RP11-61A14.	.	.	.	A	G	+	+	0.62962963	10,0,17,0	chr16,66924	Cytosol	A > G
chr16:66924072:A:G:ncRNA_exoni	chr16	66924072	66924072	A	G	ncRNA_exon	RP11-61A14.	.	.	.	A	G	+	+	0.51428571	17,0,18,0	chr16,66924	Cytosol	A > G
chr16:66924095:A:G:ncRNA_exoni	chr16	66924095	66924095	A	G	ncRNA_exon	RP11-61A14.	.	.	.	A	G	+	+	0.46153846	14,0,12,0	chr16,66924	Cytosol	A > G
chr16:66924103:A:G:ncRNA_exoni	chr16	66924103	66924103	A	G	ncRNA_exon	RP11-61A14.	.	.	.	A	G	+	+	0.14814815	23,0,4,0	chr16,66924	Cytosol	A > G
chr16:66924108:A:G:ncRNA_exoni	chr16	66924108	66924108	A	G	ncRNA_exon	RP11-61A14.	.	.	.	A	G	+	+	1	0,0,29,0	chr16,66924	Cytosol	A > G
chr16:66924111:A:G:ncRNA_exoni	chr16	66924111	66924111	A	G	ncRNA_exon	RP11-61A14.	.	.	.	A	G	+	+	0.57142857	12,0,16,0	chr16,66924	Cytosol	A > G
chr16:66924133:A:G:ncRNA_exoni	chr16	66924133	66924133	A	G	ncRNA_exon	RP11-61A14.	.	.	.	A	G	+	+	0.10714286	25,0,3,0	chr16,66924	Cytosol	A > G
chr16:67715404:T:C:intronic:GFOI	chr16	67715404	67715404	T	C	intronic	GFOD2	.	.	.	A	G	-	-	0.38461538	8,0,5,0	chr16,67715	Cytosol	A > G
chr16:67715585:T:C:intronic:GFOI	chr16	67715585	67715585	T	C	intronic	GFOD2	.	.	.	A	G	-	-	0.13333333	13,0,2,0	chr16,67715	Cytosol	A > G
chr16:67715917:T:C:intronic:GFOI	chr16	67715917	67715917	T	C	intronic	GFOD2	.	.	.	A	G	-	-	0.5	4,0,4,0	chr16,67715	Cytosol	A > G
chr16:69336876:A:G:UTR3:SNTB2	chr16	69336876	69336876	A	G	UTR3	SNTB2	ENST000003	.	.	A	G	+	+	0.05555556	34,0,2,0	chr16,69336	Cytosol	A > G
chr16:69340775:A:G:UTR3:SNTB2	chr16	69340775	69340775	A	G	UTR3	SNTB2	ENST000003	.	.	A	G	+	+	0.2	8,0,2,0	chr16,69340	Cytosol	A > G
chr16:69356716:T:C:intronic:VPS4	chr16	69356716	69356716	T	C	intronic	VPS4A	.	.	.	A	G	-	-+	0.11111111	32,0,4,0	chr16,69356	Cytosol	A > G
chr16:69356786:T:C:intronic:VPS4	chr16	69356786	69356786	T	C	intronic	VPS4A	.	.	.	A	G	-	-+	0.24324324	28,0,9,0	chr16,69356	Cytosol	A > G
chr16:69356888:T:C:intronic:VPS4	chr16	69356888	69356888	T	C	intronic	VPS4A	.	.	.	A	G	-	-+	0.625	6,0,10,0	chr16,69356	Cytosol	A > G
chr16:69356889:T:C:intronic:VPS4	chr16	69356889	69356889	T	C	intronic	VPS4A	.	.	.	A	G	-	-+	0.5	8,0,8,0	chr16,69356	Cytosol	A > G
chr16:69357218:T:C:intronic:VPS4	chr16	69357218	69357218	T	C	intronic	VPS4A	.	.	.	A	G	-	-+	0.41935484	18,0,13,0	chr16,69357	Cytosol	A > G
chr16:69357228:T:C:intronic:VPS4	chr16	69357228	69357228	T	C	intronic	VPS4A	.	.	.	A	G	-	-+	0.11538462	23,0,3,0	chr16,69357	Cytosol	A > G
chr16:69357326:T:C:intronic:VPS4	chr16	69357326	69357326	T	C	intronic	VPS4A	.	.	.	A	G	-	-+	0.11764706	15,0,2,0	chr16,69357	Cytosol	A > G
chr16:69359449:T:C:intronic:COG8	chr16	69359449	69359449	T	C	intronic	COG8	.	.	.	A	G	-	-	0.5	7,0,7,0	chr16,69359	Cytosol	A > G
chr16:69359473:T:C:intronic:COG8	chr16	69359473	69359473	T	C	intronic	COG8	.	.	.	A	G	-	-	0.57142857	3,0,4,0	chr16,69359	Cytosol	A > G
chr16:69361208:T:C:UTR3:COG8	chr16	69361208	69361208	T	C	UTR3	COG8	ENST000003	.	.	A	G	-	-	0.25	12,0,4,0	chr16,69361	Cytosol	A > G
chr16:69361219:T:C:UTR3:COG8	chr16	69361219	69361219	T	C	UTR3	COG8	ENST000003	.	.	A	G	-	-	0.11764706	15,0,2,0	chr16,69361	Cytosol	A > G
chr16:69361253:T:C:UTR3:COG8	chr16	69361253	69361253	T	C	UTR3	COG8	ENST000003	.	.	A	G	-	-	0.11764706	15,0,2,0	chr16,69361	Cytosol	A > G
chr16:69361290:T:C:UTR3:COG8	chr16	69361290	69361290	T	C	UTR3	COG8	ENST000003	.	.	A	G	-	-	0.13636364	19,0,3,0	chr16,69361	Cytosol	A > G
chr16:69361971:T:C:UTR3:COG8	chr16	69361971	69361971	T	C	UTR3	COG8	ENST000003	.	.	A	G	-	-	0.05555556	34,0,2,0	chr16,69361	Cytosol	A > G
chr16:69379799:A:G:intronic:RP11	chr16	69379799	69379799	A	G	intronic	RP11-343C2.	.	.	.	A	G	+	-	0.75	2,0,6,0	chr16,69379	Cytosol	A > G
chr16:69390514:T:C:UTR3:TERF2	chr16	69390514	69390514	T	C	UTR3	TERF2	ENST000002	.	.	A	G	-	-	0.16326531	41,0,8,0	chr16,69390	Cytosol	A > G
chr16:69399540:T:C:UTR3:TERF2	chr16	69399540	69399540	T	C	UTR3	TERF2	ENST000005	.	.	A	G	-	-	0.08108108	34,0,3,0	chr16,69399	Cytosol	A > G
chr16:69399567:T:C:UTR3:TERF2	chr16	69399567	69399567	T	C	UTR3	TERF2	ENST000005	.	.	A	G	-	-	0.24242424	25,0,8,0	chr16,69399	Cytosol	A > G
chr16:69399596:T:C:UTR3:TERF2	chr16	69399596	69399596	T	C	UTR3	TERF2	ENST000005	.	.	A	G	-	-	0.18181818	9,0,2,0	chr16,69399	Cytosol	A > G
chr16:70281075:T:C:intergenic:RP	chr16	70281075	70281075	T	C	intergenic	RP11-296I10	dist=11803;d	.	.	A	G	-		0.15384615	11,0,2,0	chr16,70281	Cytosol	A > G
chr16:70282577:T:C:intergenic:RP	chr16	70282577	70282577	T	C	intergenic	RP11-296I10	dist=13305;d	.	.	A	G	-		0.53333333	7,0,8,0	chr16,70282	Cytosol	A > G
chr16:70283218:T:C:downstream:	chr16	70283218	70283218	T	C	downstream	EXOSC6	.	.	.	A	G	-		0.36538462	33,0,19,0	chr16,70283	Cytosol	A > G
chr16:70283238:T:C:downstream:	chr16	70283238	70283238	T	C	downstream	EXOSC6	.	.	.	A	G	-		0.37037037	34,0,20,0	chr16,70283	Cytosol	A > G
chr16:70283239:T:C:downstream:	chr16	70283239	70283239	T	C	downstream	EXOSC6	.	.	.	A	G	-		0.33928571	37,0,19,0	chr16,70283	Cytosol	A > G
chr16:70283771:T:C:downstream:	chr16	70283771	70283771	T	C	downstream	EXOSC6	.	.	.	A	G	-		0.0952381	19,0,2,0	chr16,70283	Cytosol	A > G
chr16:70283780:T:C:downstream:	chr16	70283780	70283780	T	C	downstream	EXOSC6	.	.	.	A	G	-		0.2	16,0,4,0	chr16,70283	Cytosol	A > G
chr16:70283791:T:C:downstream:	chr16	70283791	70283791	T	C	downstream	EXOSC6	.	.	.	A	G	-		0.18181818	9,0,2,0	chr16,70283	Cytosol	A > G
chr16:70406878:A:G:UTR3:DDX19.	chr16	70406878	70406878	A	G	UTR3	DDX19A	ENST000003	.	.	A	G	+	+	0.12318841	121,0,17,0	chr16,70406	Cytosol	A > G
chr16:70406955:A:G:UTR3:DDX19.	chr16	70406955	70406955	A	G	UTR3	DDX19A	ENST000003	.	.	A	G	+	+	0.03940887	195,0,8,0	chr16,70406	Cytosol	A > G
chr16:70407018:A:G:UTR3:DDX19.	chr16	70407018	70407018	A	G	UTR3	DDX19A	ENST000003	.	.	A	G	+	+	0.05494505	172,0,10,0	chr16,70407	Cytosol	A > G
chr16:70608482:A:G:UTR3:SF3B3	chr16	70608482	70608482	A	G	UTR3	SF3B3	ENST000003	.	.	A	G	+	+	0.05769231	49,0,3,0	chr16,70608	Cytosol	A > G
chr16:70608643:A:G:UTR3:SF3B3	chr16	70608643	70608643	A	G	UTR3	SF3B3	ENST000003	.	.	A	G	+	+	0.05263158	36,0,2,0	chr16,70608	Cytosol	A > G
chr16:70610778:A:G:ncRNA_intro	chr16	70610778	70610778	A	G	ncRNA_intro	RP11-49C24.	.	.	.	A	G	+	+	0.25	9,0,3,0	chr16,70610	Cytosol	A > G
chr16:70610882:A:G:ncRNA_intro	chr16	70610882	70610882	A	G	ncRNA_intro	RP11-49C24.	.	.	.	A	G	+	+	0.125	21,0,3,0	chr16,70610	Cytosol	A > G
chr16:70610885:A:G:ncRNA_intro	chr16	70610885	70610885	A	G	ncRNA_intro	RP11-49C24.	.	.	.	A	G	+	+	0.22727273	17,0,5,0	chr16,70610	Cytosol	A > G
chr16:70610889:A:G:ncRNA_intro	chr16	70610889	70610889	A	G	ncRNA_intro	RP11-49C24.	.	.	.	A	G	+	+	0.19230769	21,0,5,0	chr16,70610	Cytosol	A > G
chr16:70610946:A:G:ncRNA_intro	chr16	70610946	70610946	A	G	ncRNA_intro	RP11-49C24.	.	.	.	A	G	+	+	0.38095238	13,0,8,0	chr16,70610	Cytosol	A > G
chr16:72058711:A:G:UTR3:DHODH	chr16	72058711	72058711	A	G	UTR3	DHODH	ENST000005	.	.	A	G	+	+	0.05882353	32,0,2,0	chr16,72058	Cytosol	A > G
chr16:72058748:A:G:UTR3:DHODH	chr16	72058748	72058748	A	G	UTR3	DHODH	ENST000005	.	.	A	G	+	+	0.06896552	27,0,2,0	chr16,72058	Cytosol	A > G
chr16:72058817:A:G:UTR3:DHODH	chr16	72058817	72058817	A	G	UTR3	DHODH	ENST000005	.	.	A	G	+	+	0.12	22,0,3,0	chr16,72058	Cytosol	A > G
chr16:72061147:A:G:intergenic:DH	chr16	72061147	72061147	A	G	intergenic	DHODH,TXNI	dist=2193;dis	.	.	A	G	+		1	0,0,5,0	chr16,72061	Cytosol	A > G
chr16:72061157:A:G:intergenic:DH	chr16	72061157	72061157	A	G	intergenic	DHODH,TXNI	dist=2203;dis	.	.	A	G	+		0.4	3,0,2,0	chr16,72061	Cytosol	A > G
chr16:72061265:A:G:intergenic:DH	chr16	72061265	72061265	A	G	intergenic	DHODH,TXNI	dist=2311;dis	.	.	A	G	+		0.5	3,0,3,0	chr16,72061	Cytosol	A > G
chr16:74361750:T:C:ncRNA_intror	chr16	74361750	74361750	T	C	ncRNA_intro	AC009120.6	.	.	.	A	G	-	-	0.4	3,0,2,0	chr16,74361	Cytosol	A > G
chr16:74364480:T:C:ncRNA_intror	chr16	74364480	74364480	T	C	ncRNA_intro	AC009120.6	.	.	.	A	G	-	-	0.33333333	4,0,2,0	chr16,74364	Cytosol	A > G
chr16:74364489:T:C:ncRNA_intror	chr16	74364489	74364489	T	C	ncRNA_intro	AC009120.6	.	.	.	A	G	-	-	0.8	1,0,4,0	chr16,74364	Cytosol	A > G

chr16:74655592:T:G:UTR3:RFWD3	chr16	74655592	74655592	T	G	UTR3	RFWD3	ENST000003(	.	.	A	C	-	-	0.84615385	4,22,0,0	chr16,74655!	Cytosol	A > C
chr16:74698525:T:C:UTR5:RFWD3	chr16	74698525	74698525	T	C	UTR5	RFWD3	ENST000005'	.	.	A	G	-	-	0.06976744	40,0,3,0	chr16,74698!	Cytosol	A > G
chr16:74698531:T:C:UTR5:RFWD3	chr16	74698531	74698531	T	C	UTR5	RFWD3	ENST000005'	.	.	A	G	-	-	0.04545455	42,0,2,0	chr16,74698!	Cytosol	A > G
chr16:74698535:T:C:UTR5:RFWD3	chr16	74698535	74698535	T	C	UTR5	RFWD3	ENST000005'	.	.	A	G	-	-	0.09302326	39,0,4,0	chr16,74698!	Cytosol	A > G
chr16:75477821:T:C:UTR3:TMEM1	chr16	75477821	75477821	T	C	UTR3	TMEM170A	ENST000003!	.	.	A	G	-	-	0.04444444	43,0,2,0	chr16,75477!	Cytosol	A > G
chr16:75477942:T:C:UTR3:TMEM1	chr16	75477942	75477942	T	C	UTR3	TMEM170A	ENST000003!	.	.	A	G	-	-	0.04651163	41,0,2,0	chr16,75477'	Cytosol	A > G
chr16:75479250:T:C:UTR3:TMEM1	chr16	75479250	75479250	T	C	UTR3	TMEM170A	ENST000003!	.	.	A	G	-	-	0.11764706	15,0,2,0	chr16,75479:	Cytosol	A > G
chr16:75479310:T:C:UTR3:TMEM1	chr16	75479310	75479310	T	C	UTR3	TMEM170A	ENST000003!	.	.	A	G	-	-	0.23529412	26,0,8,0	chr16,75479:	Cytosol	A > G
chr16:75479322:T:C:UTR3:TMEM1	chr16	75479322	75479322	T	C	UTR3	TMEM170A	ENST000003!	.	.	A	G	-	-	0.07407407	25,0,2,0	chr16,75479:	Cytosol	A > G
chr16:75479324:T:C:UTR3:TMEM1	chr16	75479324	75479324	T	C	UTR3	TMEM170A	ENST000003!	.	.	A	G	-	-	0.33333333	14,0,7,0	chr16,75479:	Cytosol	A > G
chr16:75479331:T:C:UTR3:TMEM1	chr16	75479331	75479331	T	C	UTR3	TMEM170A	ENST000003!	.	.	A	G	-	-	0.55555556	8,0,10,0	chr16,75479:	Cytosol	A > G
chr16:75479394:T:C:UTR3:TMEM1	chr16	75479394	75479394	T	C	UTR3	TMEM170A	ENST000003!	.	.	A	G	-	-	0.89473684	2,0,17,0	chr16,75479:	Cytosol	A > G
chr16:75479421:T:C:UTR3:TMEM1	chr16	75479421	75479421	T	C	UTR3	TMEM170A	ENST000003!	.	.	A	G	-	-	0.27272727	8,0,3,0	chr16,75479·	Cytosol	A > G
chr16:75479467:T:C:UTR3:TMEM1	chr16	75479467	75479467	T	C	UTR3	TMEM170A	ENST000003!	.	.	A	G	-	-	0.2	20,0,5,0	chr16,75479·	Cytosol	A > G
chr16:75479468:T:C:UTR3:TMEM1	chr16	75479468	75479468	T	C	UTR3	TMEM170A	ENST000003!	.	.	A	G	-	-	0.07692308	24,0,2,0	chr16,75479·	Cytosol	A > G
chr16:75479470:T:C:UTR3:TMEM1	chr16	75479470	75479470	T	C	UTR3	TMEM170A	ENST000003!	.	.	A	G	-	-	0.22222222	21,0,6,0	chr16,75479·	Cytosol	A > G
chr16:75479474:T:C:UTR3:TMEM1	chr16	75479474	75479474	T	C	UTR3	TMEM170A	ENST000003!	.	.	A	G	-	-	0.5	14,0,14,0	chr16,75479·	Cytosol	A > G
chr16:75479831:T:C:UTR3:TMEM1	chr16	75479831	75479831	T	C	UTR3	TMEM170A	ENST000003!	.	.	A	G	-	-	0.21428571	11,0,3,0	chr16,75479!	Cytosol	A > G
chr16:75479891:T:C:UTR3:TMEM1	chr16	75479891	75479891	T	C	UTR3	TMEM170A	ENST000003!	.	.	A	G	-	-	0.14285714	18,0,3,0	chr16,75479!	Cytosol	A > G
chr16:75479907:T:C:UTR3:TMEM1	chr16	75479907	75479907	T	C	UTR3	TMEM170A	ENST000003!	.	.	A	G	-	-	0.25	18,0,6,0	chr16,75479!	Cytosol	A > G
chr16:75479910:T:C:UTR3:TMEM1	chr16	75479910	75479910	T	C	UTR3	TMEM170A	ENST000003!	.	.	A	G	-	-	0.13043478	20,0,3,0	chr16,75479'	Cytosol	A > G
chr16:75480010:T:C:UTR3:TMEM1	chr16	75480010	75480010	T	C	UTR3	TMEM170A	ENST000003!	.	.	A	G	-	-	0.15384615	11,0,2,0	chr16,75480(	Cytosol	A > G
chr16:75632113:T:C:ncRNA_intror	chr16	75632113	75632113	T	C	ncRNA_intro	RP11-77K12.	.	.	.	A	G	-	-	0.04347826	66,0,3,0	chr16,75632:	Cytosol	A > G
chr16:75632690:T:C:ncRNA_intror	chr16	75632690	75632690	T	C	ncRNA_intro	RP11-77K12.	.	.	.	A	G	-	-	0.04347826	44,0,2,0	chr16,75632(	Cytosol	A > G
chr16:75633108:T:C:ncRNA_intror	chr16	75633108	75633108	T	C	ncRNA_intro	RP11-77K12.	.	.	.	A	G	-	-	0.04878049	39,0,2,0	chr16,75633:	Cytosol	A > G
chr16:75633144:T:C:ncRNA_intror	chr16	75633144	75633144	T	C	ncRNA_intro	RP11-77K12.	.	.	.	A	G	-	-	0.04878049	39,0,2,0	chr16,75633:	Cytosol	A > G
chr16:75633217:T:C:ncRNA_intror	chr16	75633217	75633217	T	C	ncRNA_intro	RP11-77K12.	.	.	.	A	G	-	-	0.17391304	19,0,4,0	chr16,75633:	Cytosol	A > G
chr16:75633235:T:C:ncRNA_intror	chr16	75633235	75633235	T	C	ncRNA_intro	RP11-77K12.	.	.	.	A	G	-	-	0.14285714	18,0,3,0	chr16,75633:	Cytosol	A > G
chr16:78155810:A:G:intronic:WW(	chr16	78155810	78155810	A	G	intronic	WWOX	.	.	.	A	G	+	+	0.42857143	4,0,3,0	chr16,78155!	Cytosol	A > G
chr16:81014384:T:C:UTR3:CMC2	chr16	81014384	81014384	T	C	UTR3	CMC2	ENST000005'	.	.	A	G	-	-	0.19195046	261,0,62,0	chr16,81014:	Cytosol	A > G
chr16:81063016:A:G:ncRNA_intro	chr16	81063016	81063016	A	G	ncRNA_intro	RP11-303E1(	.	.	.	A	G	+	+-	0.20289855	55,0,14,0	chr16,81063(	Cytosol	A > G
chr16:81063020:A:G:ncRNA_intro	chr16	81063020	81063020	A	G	ncRNA_intro	RP11-303E1(	.	.	.	A	G	+	+-	0.13043478	60,0,9,0	chr16,81063(	Cytosol	A > G
chr16:81063028:A:G:ncRNA_intro	chr16	81063028	81063028	A	G	ncRNA_intro	RP11-303E1(	.	.	.	A	G	+	+-	0.10909091	49,0,6,0	chr16,81063(	Cytosol	A > G
chr16:81063084:A:G:ncRNA_intro	chr16	81063084	81063084	A	G	ncRNA_intro	RP11-303E1(	.	.	.	A	G	+	+-	0.07142857	52,0,4,0	chr16,81063(	Cytosol	A > G
chr16:81063098:A:G:ncRNA_intro	chr16	81063098	81063098	A	G	ncRNA_intro	RP11-303E1(	.	.	.	A	G	+	+-	0.59259259	44,0,64,0	chr16,81063(	Cytosol	A > G
chr16:81063144:A:G:ncRNA_intro	chr16	81063144	81063144	A	G	ncRNA_intro	RP11-303E1(	.	.	.	A	G	+	+-	0.48466258	84,0,79,0	chr16,81063:	Cytosol	A > G
chr16:81063242:A:G:ncRNA_intro	chr16	81063242	81063242	A	G	ncRNA_intro	RP11-303E1(	.	.	.	A	G	+	+-	0.11864407	104,0,14,0	chr16,81063:	Cytosol	A > G
chr16:81063244:A:G:ncRNA_intro	chr16	81063244	81063244	A	G	ncRNA_intro	RP11-303E1(	.	.	.	A	G	+	+-	0.3553719	78,0,43,0	chr16,81063:	Cytosol	A > G
chr16:81063245:A:G:ncRNA_intro	chr16	81063245	81063245	A	G	ncRNA_intro	RP11-303E1(	.	.	.	A	G	+	+-	0.79487179	24,0,93,0	chr16,81063:	Cytosol	A > G
chr16:81063280:A:G:ncRNA_intro	chr16	81063280	81063280	A	G	ncRNA_intro	RP11-303E1(	.	.	.	A	G	+	+-	0.10958904	130,0,16,0	chr16,81063:	Cytosol	A > G
chr16:81063283:A:G:ncRNA_intro	chr16	81063283	81063283	A	G	ncRNA_intro	RP11-303E1(	.	.	.	A	G	+	+-	0.02777778	140,0,4,0	chr16,81063:	Cytosol	A > G
chr16:81063309:A:G:ncRNA_intro	chr16	81063309	81063309	A	G	ncRNA_intro	RP11-303E1(	.	.	.	A	G	+	+-	0.19117647	110,0,26,0	chr16,81063:	Cytosol	A > G
chr16:81063319:A:G:ncRNA_intro	chr16	81063319	81063319	A	G	ncRNA_intro	RP11-303E1(	.	.	.	A	G	+	+-	0.3880597	82,0,52,0	chr16,81063:	Cytosol	A > G
chr16:81063345:A:G:ncRNA_intro	chr16	81063345	81063345	A	G	ncRNA_intro	RP11-303E1(	.	.	.	A	G	+	+-	0.4625	43,0,37,0	chr16,81063:	Cytosol	A > G
chr16:81063360:A:G:ncRNA_intro	chr16	81063360	81063360	A	G	ncRNA_intro	RP11-303E1(	.	.	.	A	G	+	+-	0.12	88,0,12,0	chr16,81063:	Cytosol	A > G
chr16:81063363:A:G:ncRNA_intro	chr16	81063363	81063363	A	G	ncRNA_intro	RP11-303E1(	.	.	.	A	G	+	+-	0.21875	75,0,21,0	chr16,81063:	Cytosol	A > G
chr16:81063389:A:G:ncRNA_intro	chr16	81063389	81063389	A	G	ncRNA_intro	RP11-303E1(	.	.	.	A	G	+	+-	0.42553191	81,0,60,0	chr16,81063:	Cytosol	A > G
chr16:81063400:A:G:ncRNA_intro	chr16	81063400	81063400	A	G	ncRNA_intro	RP11-303E1(	.	.	.	A	G	+	+-	0.11875	141,0,19,0	chr16,81063:	Cytosol	A > G
chr16:81063432:A:G:ncRNA_intro	chr16	81063432	81063432	A	G	ncRNA_intro	RP11-303E1(	.	.	.	A	G	+	+-	0.15846995	154,0,29,0	chr16,81063·	Cytosol	A > G
chr16:81063443:A:G:ncRNA_intro	chr16	81063443	81063443	A	G	ncRNA_intro	RP11-303E1(	.	.	.	A	G	+	+-	0.08666667	137,0,13,0	chr16,81063·	Cytosol	A > G
chr16:81064163:A:G:ncRNA_intro	chr16	81064163	81064163	A	G	ncRNA_intro	RP11-303E1(	.	.	.	A	G	+	+-	0.62601626	46,0,77,0	chr16,81064:	Cytosol	A > G
chr16:81064189:A:G:ncRNA_intro	chr16	81064189	81064189	A	G	ncRNA_intro	RP11-303E1(	.	.	.	A	G	+	+-	0.30612245	102,0,45,0	chr16,81064:	Cytosol	A > G
chr16:81064207:A:G:ncRNA_intro	chr16	81064207	81064207	A	G	ncRNA_intro	RP11-303E1(	.	.	.	A	G	+	+-	0.04444444	129,0,6,0	chr16,81064:	Cytosol	A > G
chr16:81064233:A:G:ncRNA_intro	chr16	81064233	81064233	A	G	ncRNA_intro	RP11-303E1(	.	.	.	A	G	+	+-	0.19791667	154,0,38,0	chr16,81064:	Cytosol	A > G
chr16:81064234:A:G:ncRNA_intro	chr16	81064234	81064234	A	G	ncRNA_intro	RP11-303E1(	.	.	.	A	G	+	+-	0.4673913	98,0,86,0	chr16,81064:	Cytosol	A > G
chr16:81064295:A:G:ncRNA_intro	chr16	81064295	81064295	A	G	ncRNA_intro	RP11-303E1(	.	.	.	A	G	+	+-	0.06837607	109,0,8,0	chr16,81064:	Cytosol	A > G
chr16:81064303:A:G:ncRNA_intro	chr16	81064303	81064303	A	G	ncRNA_intro	RP11-303E1(	.	.	.	A	G	+	+-	0.08148148	124,0,11,0	chr16,81064:	Cytosol	A > G
chr16:81064304:A:G:ncRNA_intro	chr16	81064304	81064304	A	G	ncRNA_intro	RP11-303E1(	.	.	.	A	G	+	+-	0.33774834	100,0,51,0	chr16,81064:	Cytosol	A > G
chr16:81064309:A:G:ncRNA_intro	chr16	81064309	81064309	A	G	ncRNA_intro	RP11-303E1(	.	.	.	A	G	+	+-	0.30666667	104,0,46,0	chr16,81064:	Cytosol	A > G
chr16:81064336:A:G:ncRNA_intro	chr16	81064336	81064336	A	G	ncRNA_intro	RP11-303E1(	.	.	.	A	G	+	+-	0.31578947	104,0,48,0	chr16,81064:	Cytosol	A > G
chr16:81064343:A:G:ncRNA_intro	chr16	81064343	81064343	A	G	ncRNA_intro	RP11-303E1(	.	.	.	A	G	+	+-	0.16352201	133,0,26,0	chr16,81064:	Cytosol	A > G
chr16:81064373:A:G:ncRNA_intro	chr16	81064373	81064373	A	G	ncRNA_intro	RP11-303E1(	.	.	.	A	G	+	+-	0.02777778	175,0,5,0	chr16,81064:	Cytosol	A > G
chr16:81064396:A:G:ncRNA_exoni	chr16	81064396	81064396	A	G	ncRNA_exon	RP11-303E1(	.	.	.	A	G	+	-+	0.04524887	211,0,10,0	chr16,81064:	Cytosol	A > G
chr16:81064439:A:G:ncRNA_exoni	chr16	81064439	81064439	A	G	ncRNA_exon	RP11-303E1(	.	.	.	A	G	+	-+	0.14975845	176,0,31,0	chr16,81064·	Cytosol	A > G

chr16:81064440:A:G:ncRNA_exoni	chr16	81064440	81064440	A	G	ncRNA_exon	RP11-303E1	.	.	.	A	G	+	-+	0.49765258	107,0,106,0	chr16,81064	Cytosol	A > G
chr16:81064489:A:G:ncRNA_exoni	chr16	81064489	81064489	A	G	ncRNA_exon	RP11-303E1	.	.	.	A	G	+	-+	0.40131579	91,0,61,0	chr16,81064	Cytosol	A > G
chr16:81064492:A:G:ncRNA_exoni	chr16	81064492	81064492	A	G	ncRNA_exon	RP11-303E1	.	.	.	A	G	+	-+	0.27586207	105,0,40,0	chr16,81064	Cytosol	A > G
chr16:81064494:A:G:ncRNA_exoni	chr16	81064494	81064494	A	G	ncRNA_exon	RP11-303E1	.	.	.	A	G	+	-+	0.12318841	120,0,17,1	chr16,81064	Cytosol	A > G
chr16:81064507:A:G:ncRNA_exoni	chr16	81064507	81064507	A	G	ncRNA_exon	RP11-303E1	.	.	.	A	G	+	-+	0.51515152	64,0,68,0	chr16,81064	Cytosol	A > G
chr16:81064533:A:G:ncRNA_exoni	chr16	81064533	81064533	A	G	ncRNA_exon	RP11-303E1	.	.	.	A	G	+	-+	0.13821138	106,0,17,0	chr16,81064	Cytosol	A > G
chr16:81064534:A:G:ncRNA_exoni	chr16	81064534	81064534	A	G	ncRNA_exon	RP11-303E1	.	.	.	A	G	+	-+	0.69672131	37,0,85,0	chr16,81064	Cytosol	A > G
chr16:81064543:A:G:ncRNA_exoni	chr16	81064543	81064543	A	G	ncRNA_exon	RP11-303E1	.	.	.	A	G	+	-+	0.13559322	102,0,16,0	chr16,81064	Cytosol	A > G
chr16:81064560:A:G:ncRNA_exoni	chr16	81064560	81064560	A	G	ncRNA_exon	RP11-303E1	.	.	.	A	G	+	-+	0.43373494	47,0,36,0	chr16,81064	Cytosol	A > G
chr16:81064574:A:G:ncRNA_exoni	chr16	81064574	81064574	A	G	ncRNA_exon	RP11-303E1	.	.	.	A	G	+	-+	0.64444444	32,0,58,0	chr16,81064	Cytosol	A > G
chr16:81064575:A:G:ncRNA_exoni	chr16	81064575	81064575	A	G	ncRNA_exon	RP11-303E1	.	.	.	A	G	+	-+	0.50561798	44,0,45,0	chr16,81064	Cytosol	A > G
chr16:81064611:A:G:ncRNA_exoni	chr16	81064611	81064611	A	G	ncRNA_exon	RP11-303E1	.	.	.	A	G	+	-+	0.18965517	47,0,11,0	chr16,81064	Cytosol	A > G
chr16:81064629:A:G:ncRNA_exoni	chr16	81064629	81064629	A	G	ncRNA_exon	RP11-303E1	.	.	.	A	G	+	-+	0.38888889	44,0,28,0	chr16,81064	Cytosol	A > G
chr16:81064649:A:G:ncRNA_exoni	chr16	81064649	81064649	A	G	ncRNA_exon	RP11-303E1	.	.	.	A	G	+	-+	0.09473684	86,0,9,0	chr16,81064	Cytosol	A > G
chr16:81064656:A:G:ncRNA_exoni	chr16	81064656	81064656	A	G	ncRNA_exon	RP11-303E1	.	.	.	A	G	+	-+	0.01904762	102,0,2,1	chr16,81064	Cytosol	A > G
chr16:86568564:T:C:intronic:MTH	chr16	86568564	86568564	T	C	intronic	MTHFSD	.	.	.	A	G	-	-	0.625	3,0,5,0	chr16,86568	Cytosol	A > G
chr16:88726868:T:C:intronic:MVD	chr16	88726868	88726868	T	C	intronic	MVD	.	.	.	A	G	-	-	0.8	1,0,4,0	chr16,88726	Cytosol	A > G
chr16:88727226:T:C:exonic:MVD	chr16	88727226	88727226	T	C	exonic	MVD	.	nonsynonym	MVD:ENST0	A	G	-	-	1	0,0,6,0	chr16,88727	Cytosol	A > G
chr16:88742060:A:G:ncRNA_intro	chr16	88742060	88742060	A	G	ncRNA_intro	SNAI3-AS1	.	.	.	A	G	+	+	0.42857143	4,0,3,0	chr16,88742	Cytosol	A > G
chr16:88784379:T:C:intronic:PIEZ	chr16	88784379	88784379	T	C	intronic	PIEZO1	.	.	.	A	G	-	-	1	0,0,6,0	chr16,88784	Cytosol	A > G
chr16:88784415:T:C:intronic:PIEZ	chr16	88784415	88784415	T	C	intronic	PIEZO1	.	.	.	A	G	-	-	0.42857143	4,0,3,0	chr16,88784	Cytosol	A > G
chr16:88785336:T:C:ncRNA_exoni	chr16	88785336	88785336	T	C	ncRNA_exon	RP5-1142A6.	.	.	.	A	G	-	-+	0.2	12,0,3,0	chr16,88785	Cytosol	A > G
chr16:88785404:T:C:ncRNA_exoni	chr16	88785404	88785404	T	C	ncRNA_exon	RP5-1142A6.	.	.	.	A	G	-	+-	0.44444444	5,0,4,0	chr16,88785	Cytosol	A > G
chr16:89581174:A:G:intronic:SPG	chr16	89581174	89581174	A	G	intronic	SPG7	.	.	.	A	G	+	+	0.57142857	3,0,4,0	chr16,89581	Cytosol	A > G
chr16:89582444:A:G:intronic:SPG	chr16	89582444	89582444	A	G	intronic	SPG7	.	.	.	A	G	+	+	0.4	3,0,2,0	chr16,89582	Cytosol	A > G
chr16:89582838:A:G:intronic:SPG	chr16	89582838	89582838	A	G	intronic	SPG7	.	.	.	A	G	+	+	0.5	4,0,4,0	chr16,89582	Cytosol	A > G
chr16:89584084:A:G:intronic:SPG	chr16	89584084	89584084	A	G	intronic	SPG7	.	.	.	A	G	+	+	0.27272727	8,0,3,0	chr16,89584	Cytosol	A > G
chr16:89584109:A:G:intronic:SPG	chr16	89584109	89584109	A	G	intronic	SPG7	.	.	.	A	G	+	+	0.30769231	9,0,4,0	chr16,89584	Cytosol	A > G
chr16:89584128:A:G:intronic:SPG	chr16	89584128	89584128	A	G	intronic	SPG7	.	.	.	A	G	+	+	0.21428571	11,0,3,0	chr16,89584	Cytosol	A > G
chr16:89584131:A:G:intronic:SPG	chr16	89584131	89584131	A	G	intronic	SPG7	.	.	.	A	G	+	+	0.4	9,0,6,0	chr16,89584	Cytosol	A > G
chr16:89584158:A:G:intronic:SPG	chr16	89584158	89584158	A	G	intronic	SPG7	.	.	.	A	G	+	+	0.85714286	2,0,12,0	chr16,89584	Cytosol	A > G
chr16:89584168:A:G:intronic:SPG	chr16	89584168	89584168	A	G	intronic	SPG7	.	.	.	A	G	+	+	0.44444444	5,0,4,0	chr16,89584	Cytosol	A > G
chr16:89584169:A:G:intronic:SPG	chr16	89584169	89584169	A	G	intronic	SPG7	.	.	.	A	G	+	+	0.66666667	3,0,6,0	chr16,89584	Cytosol	A > G
chr16:89584209:A:G:intronic:SPG	chr16	89584209	89584209	A	G	intronic	SPG7	.	.	.	A	G	+	+	0.625	3,0,5,0	chr16,89584	Cytosol	A > G
chr16:89584212:A:G:intronic:SPG	chr16	89584212	89584212	A	G	intronic	SPG7	.	.	.	A	G	+	+	0.57142857	3,0,4,0	chr16,89584	Cytosol	A > G
chr16:89586884:A:G:intronic:SPG	chr16	89586884	89586884	A	G	intronic	SPG7	.	.	.	A	G	+	+	0.5	3,0,3,0	chr16,89586	Cytosol	A > G
chr16:89586885:A:G:intronic:SPG	chr16	89586885	89586885	A	G	intronic	SPG7	.	.	.	A	G	+	+	1	0,0,8,0	chr16,89586	Cytosol	A > G
chr16:89587968:A:G:intronic:SPG	chr16	89587968	89587968	A	G	intronic	SPG7	.	.	.	A	G	+	+	0.625	3,0,5,0	chr16,89587	Cytosol	A > G
chr16:89629906:A:G:UTR3:RPL13	chr16	89629906	89629906	A	G	UTR3	RPL13	ENST000003	.	.	A	G	+	+	0.375	25,0,15,0	chr16,89629	Cytosol	A > G
chr16:89754023:A:G:exonic:CDK1	chr16	89754023	89754023	A	G	exonic	CDK10	.	nonsynonym	CDK10:ENST	A	G	+	+	0.25	6,0,2,0	chr16,89754	Cytosol	A > G
chr16:89754046:A:G:exonic:CDK1	chr16	89754046	89754046	A	G	exonic	CDK10	.	nonsynonym	CDK10:ENST	A	G	+	+	1	0,0,5,0	chr16,89754	Cytosol	A > G
chr16:89754110:A:G:exonic:CDK1	chr16	89754110	89754110	A	G	exonic	CDK10	.	synonymous	CDK10:ENST	A	G	+	+	0.22222222	7,0,2,0	chr16,89754	Cytosol	A > G
chr16:89802044:T:C:intronic:ZNF2	chr16	89802044	89802044	T	C	intronic	ZNF276	.	.	.	A	G	-	+	0.25	6,0,2,0	chr16,89802	Cytosol	A > G
chr16:89802438:T:C:intronic:ZNF2	chr16	89802438	89802438	T	C	intronic	ZNF276	.	.	.	A	G	-	+	0.72727273	3,0,8,0	chr16,89802	Cytosol	A > G
chr16:89983818:A:G:ncRNA_intro	chr16	89983818	89983818	A	G	ncRNA_intro	RP11-566K11	.	.	.	A	G	+	+	0.4	3,0,2,0	chr16,89983	Cytosol	A > G
chr17:291773:T:C:UTR3:FAM101B	chr17	291773	291773	T	C	UTR3	FAM101B	ENST000003	.	.	A	G	-	-	0.07142857	195,0,15,0	chr17,29177	Cytosol	A > G
chr17:291783:T:C:UTR3:FAM101B	chr17	291783	291783	T	C	UTR3	FAM101B	ENST000003	.	.	A	G	-	-	0.01005025	197,0,2,0	chr17,29178	Cytosol	A > G
chr17:419172:T:C:UTR3:VPS53	chr17	419172	419172	T	C	UTR3	VPS53	ENST000004	.	.	A	G	-	-	0.14814815	23,0,4,0	chr17,41917	Cytosol	A > G
chr17:419173:T:C:UTR3:VPS53	chr17	419173	419173	T	C	UTR3	VPS53	ENST000004	.	.	A	G	-	-	0.15384615	22,0,4,0	chr17,41917	Cytosol	A > G
chr17:419630:T:C:UTR3:VPS53	chr17	419630	419630	T	C	UTR3	VPS53	ENST000004	.	.	A	G	-	-	0.07692308	35,1,3,0	chr17,41962	Cytosol	A > G
chr17:419669:T:C:UTR3:VPS53	chr17	419669	419669	T	C	UTR3	VPS53	ENST000004	.	.	A	G	-	-	0.21052632	15,0,4,0	chr17,41966	Cytosol	A > G
chr17:419691:T:C:UTR3:VPS53	chr17	419691	419691	T	C	UTR3	VPS53	ENST000004	.	.	A	G	-	-	0.41666667	7,0,5,0	chr17,41969	Cytosol	A > G
chr17:419692:T:C:UTR3:VPS53	chr17	419692	419692	T	C	UTR3	VPS53	ENST000004	.	.	A	G	-	-	0.66666667	4,0,8,0	chr17,41969	Cytosol	A > G
chr17:420820:T:C:exonic:VPS53	chr17	420820	420820	T	C	exonic	VPS53	.	nonsynonym	VPS53:ENST	A	G	-	-	0.06451613	29,0,2,0	chr17,42081	Cytosol	A > G
chr17:420828:T:C:exonic:VPS53	chr17	420828	420828	T	C	exonic	VPS53	.	synonymous	VPS53:ENST	A	G	-	-	0.33333333	18,0,9,0	chr17,42082	Cytosol	A > G
chr17:420857:T:C:exonic:VPS53	chr17	420857	420857	T	C	exonic	VPS53	.	nonsynonym	VPS53:ENST	A	G	-	-	0.23809524	16,0,5,0	chr17,42085	Cytosol	A > G
chr17:420858:T:C:exonic:VPS53	chr17	420858	420858	T	C	exonic	VPS53	.	synonymous	VPS53:ENST	A	G	-	-	0.66666667	6,0,12,0	chr17,42085	Cytosol	A > G
chr17:420862:T:C:exonic:VPS53	chr17	420862	420862	T	C	exonic	VPS53	.	nonsynonym	VPS53:ENST	A	G	-	-	0.27777778	13,0,5,0	chr17,42086	Cytosol	A > G
chr17:420897:T:C:UTR3:VPS53	chr17	420897	420897	T	C	UTR3	VPS53	ENST000004	.	.	A	G	-	-	0.05714286	33,0,2,0	chr17,42089	Cytosol	A > G
chr17:420898:T:C:UTR3:VPS53	chr17	420898	420898	T	C	UTR3	VPS53	ENST000004	.	.	A	G	-	-	0.23333333	23,0,7,0	chr17,42089	Cytosol	A > G
chr17:421034:T:C:UTR3:VPS53	chr17	421034	421034	T	C	UTR3	VPS53	ENST000004	.	.	A	G	-	-	0.07407407	50,0,4,0	chr17,42103	Cytosol	A > G
chr17:421159:T:C:UTR3:VPS53	chr17	421159	421159	T	C	UTR3	VPS53	ENST000004	.	.	A	G	-	-	0.03508772	55,0,2,0	chr17,42115	Cytosol	A > G
chr17:421887:T:C:UTR3:VPS53	chr17	421887	421887	T	C	UTR3	VPS53	ENST000004	.	.	A	G	-	-	0.13513514	32,0,5,0	chr17,42188	Cytosol	A > G
chr17:703190:T:C:UTR3:NXN	chr17	703190	703190	T	C	UTR3	NXN	ENST000005	.	.	A	G	-	-	0.04918033	58,0,3,0	chr17,70318	Cytosol	A > G

chr17:703280:T:C:UTR3:NXN	chr17	703280	703280	T	C	UTR3	NXN	ENST000005	.	.	A	G	-	-	0.015	197,0,3,0	chr17,70327	Cytosol	A > G
chr17:1368195:T:C:UTR3:MYO1C	chr17	1368195	1368195	T	C	UTR3	MYO1C	ENST000003	.	.	A	G	-	-	0.07843137	141,0,12,0	chr17,13681	Cytosol	A > G
chr17:1368203:T:C:UTR3:MYO1C	chr17	1368203	1368203	T	C	UTR3	MYO1C	ENST000003	.	.	A	G	-	-	0.01492537	132,0,2,0	chr17,13682	Cytosol	A > G
chr17:1368218:T:C:UTR3:MYO1C	chr17	1368218	1368218	T	C	UTR3	MYO1C	ENST000003	.	.	A	G	-	-	0.07563025	110,0,9,0	chr17,13682	Cytosol	A > G
chr17:1368279:T:C:UTR3:MYO1C	chr17	1368279	1368279	T	C	UTR3	MYO1C	ENST000003	.	.	A	G	-	-	0.06707317	306,0,22,0	chr17,13682	Cytosol	A > G
chr17:1368282:T:C:UTR3:MYO1C	chr17	1368282	1368282	T	C	UTR3	MYO1C	ENST000003	.	.	A	G	-	-	0.05230769	308,0,17,0	chr17,13682	Cytosol	A > G
chr17:1368283:T:C:UTR3:MYO1C	chr17	1368283	1368283	T	C	UTR3	MYO1C	ENST000003	.	.	A	G	-	-	0.05810398	308,0,19,0	chr17,13682	Cytosol	A > G
chr17:1368284:T:C:UTR3:MYO1C	chr17	1368284	1368284	T	C	UTR3	MYO1C	ENST000003	.	.	A	G	-	-	0.09667674	299,0,32,0	chr17,13682	Cytosol	A > G
chr17:1368288:T:C:UTR3:MYO1C	chr17	1368288	1368288	T	C	UTR3	MYO1C	ENST000003	.	.	A	G	-	-	0.05671642	316,0,19,0	chr17,13682	Cytosol	A > G
chr17:1606834:T:C:intergenic:PRP	chr17	1606834	1606834	T	C	intergenic	PRPF8,TLCD2	dist=18658;d	.	.	A	G	-	-	0.25	6,0,2,0	chr17,16068	Cytosol	A > G
chr17:1606838:T:C:intergenic:PRP	chr17	1606838	1606838	T	C	intergenic	PRPF8,TLCD2	dist=18662;d	.	.	A	G	-	-	0.42857143	4,0,3,0	chr17,16068	Cytosol	A > G
chr17:1606848:T:C:intergenic:PRP	chr17	1606848	1606848	T	C	intergenic	PRPF8,TLCD2	dist=18672;d	.	.	A	G	-	-	0.75	2,0,6,0	chr17,16068	Cytosol	A > G
chr17:1607665:T:C:intergenic:PRP	chr17	1607665	1607665	T	C	intergenic	PRPF8,TLCD2	dist=19489;d	.	.	A	G	-	-	0.22727273	17,0,5,0	chr17,16076	Cytosol	A > G
chr17:1608137:T:C:intergenic:PRP	chr17	1608137	1608137	T	C	intergenic	PRPF8,TLCD2	dist=19961;d	.	.	A	G	-	-	0.71428571	2,0,5,0	chr17,16081	Cytosol	A > G
chr17:1682445:T:C:downstream:S	chr17	1682445	1682445	T	C	downstream	SMYD4	.	.	.	A	G	-		0.5	3,0,3,0	chr17,16824	Cytosol	A > G
chr17:1683572:T:C:UTR3:SMYD4	chr17	1683572	1683572	T	C	UTR3	SMYD4	ENST000003	.	.	A	G	-	-	0.18604651	35,0,8,0	chr17,16835	Cytosol	A > G
chr17:1683601:T:C:UTR3:SMYD4	chr17	1683601	1683601	T	C	UTR3	SMYD4	ENST000003	.	.	A	G	-	-	0.08333333	44,0,4,0	chr17,16836	Cytosol	A > G
chr17:1683647:T:C:UTR3:SMYD4	chr17	1683647	1683647	T	C	UTR3	SMYD4	ENST000003	.	.	A	G	-	-	0.03448276	56,0,2,0	chr17,16836	Cytosol	A > G
chr17:1683738:T:C:UTR3:SMYD4	chr17	1683738	1683738	T	C	UTR3	SMYD4	ENST000003	.	.	A	G	-	-	0.10714286	25,0,3,0	chr17,16837	Cytosol	A > G
chr17:1683790:T:C:UTR3:SMYD4	chr17	1683790	1683790	T	C	UTR3	SMYD4	ENST000003	.	.	A	G	-	-	0.03921569	49,0,2,0	chr17,16837	Cytosol	A > G
chr17:1684001:T:C:UTR3:SMYD4	chr17	1684001	1684001	T	C	UTR3	SMYD4	ENST000003	.	.	A	G	-	-	0.11538462	23,0,3,0	chr17,16840	Cytosol	A > G
chr17:1684044:T:C:UTR3:SMYD4	chr17	1684044	1684044	T	C	UTR3	SMYD4	ENST000003	.	.	A	G	-	-	0.10526316	34,0,4,0	chr17,16840	Cytosol	A > G
chr17:1684054:T:C:UTR3:SMYD4	chr17	1684054	1684054	T	C	UTR3	SMYD4	ENST000003	.	.	A	G	-	-	0.48484848	17,0,16,0	chr17,16840	Cytosol	A > G
chr17:1684080:T:C:UTR3:SMYD4	chr17	1684080	1684080	T	C	UTR3	SMYD4	ENST000003	.	.	A	G	-	-	0.16666667	25,0,5,0	chr17,16840	Cytosol	A > G
chr17:1684091:T:C:UTR3:SMYD4	chr17	1684091	1684091	T	C	UTR3	SMYD4	ENST000003	.	.	A	G	-	-	0.17857143	23,0,5,0	chr17,16840	Cytosol	A > G
chr17:1684121:T:C:UTR3:SMYD4	chr17	1684121	1684121	T	C	UTR3	SMYD4	ENST000003	.	.	A	G	-	-	0.19354839	25,0,6,0	chr17,16841	Cytosol	A > G
chr17:1684306:T:C:UTR3:SMYD4	chr17	1684306	1684306	T	C	UTR3	SMYD4	ENST000003	.	.	A	G	-	-	0.29411765	12,0,5,0	chr17,16843	Cytosol	A > G
chr17:1684346:T:C:UTR3:SMYD4	chr17	1684346	1684346	T	C	UTR3	SMYD4	ENST000003	.	.	A	G	-	-	0.0625	30,0,2,0	chr17,16843	Cytosol	A > G
chr17:1684351:T:C:UTR3:SMYD4	chr17	1684351	1684351	T	C	UTR3	SMYD4	ENST000003	.	.	A	G	-	-	0.23076923	20,0,6,0	chr17,16843	Cytosol	A > G
chr17:1684352:T:C:UTR3:SMYD4	chr17	1684352	1684352	T	C	UTR3	SMYD4	ENST000003	.	.	A	G	-	-	0.22222222	21,0,6,0	chr17,16843	Cytosol	A > G
chr17:1684374:T:C:UTR3:SMYD4	chr17	1684374	1684374	T	C	UTR3	SMYD4	ENST000003	.	.	A	G	-	-	0.16666667	20,0,4,0	chr17,16843	Cytosol	A > G
chr17:1684403:T:C:UTR3:SMYD4	chr17	1684403	1684403	T	C	UTR3	SMYD4	ENST000003	.	.	A	G	-	-	0.10526316	17,0,2,0	chr17,16844	Cytosol	A > G
chr17:1684442:T:C:UTR3:SMYD4	chr17	1684442	1684442	T	C	UTR3	SMYD4	ENST000003	.	.	A	G	-	-	0.36363636	7,0,4,0	chr17,16844	Cytosol	A > G
chr17:1684496:T:C:UTR3:SMYD4	chr17	1684496	1684496	T	C	UTR3	SMYD4	ENST000003	.	.	A	G	-	-	0.16666667	15,0,3,0	chr17,16844	Cytosol	A > G
chr17:2320469:T:C:UTR3:METTL1	chr17	2320469	2320469	T	C	UTR3	METTL16	ENST000002	.	.	A	G	-	-	0.3	7,0,3,0	chr17,23204	Cytosol	A > G
chr17:2320486:T:C:UTR3:METTL1	chr17	2320486	2320486	T	C	UTR3	METTL16	ENST000002	.	.	A	G	-	-	0.57142857	6,0,8,0	chr17,23204	Cytosol	A > G
chr17:2320495:T:C:UTR3:METTL1	chr17	2320495	2320495	T	C	UTR3	METTL16	ENST000002	.	.	A	G	-	-	0.5	6,0,6,0	chr17,23204	Cytosol	A > G
chr17:2320496:T:C:UTR3:METTL1	chr17	2320496	2320496	T	C	UTR3	METTL16	ENST000002	.	.	A	G	-	-	0.66666667	4,0,8,0	chr17,23204	Cytosol	A > G
chr17:2320506:T:C:UTR3:METTL1	chr17	2320506	2320506	T	C	UTR3	METTL16	ENST000002	.	.	A	G	-	-	0.63636364	4,0,7,0	chr17,23205	Cytosol	A > G
chr17:2320509:T:C:UTR3:METTL1	chr17	2320509	2320509	T	C	UTR3	METTL16	ENST000002	.	.	A	G	-	-	1	0,0,12,0	chr17,23205	Cytosol	A > G
chr17:2320522:T:C:UTR3:METTL1	chr17	2320522	2320522	T	C	UTR3	METTL16	ENST000002	.	.	A	G	-	-	0.7	3,0,7,0	chr17,23205	Cytosol	A > G
chr17:2320531:T:C:UTR3:METTL1	chr17	2320531	2320531	T	C	UTR3	METTL16	ENST000002	.	.	A	G	-	-	0.21428571	11,0,3,0	chr17,23205	Cytosol	A > G
chr17:2320571:T:C:UTR3:METTL1	chr17	2320571	2320571	T	C	UTR3	METTL16	ENST000002	.	.	A	G	-	-	0.16666667	20,0,4,0	chr17,23205	Cytosol	A > G
chr17:2320606:T:C:UTR3:METTL1	chr17	2320606	2320606	T	C	UTR3	METTL16	ENST000002	.	.	A	G	-	-	0.08510638	43,0,4,0	chr17,23206	Cytosol	A > G
chr17:2320628:T:C:UTR3:METTL1	chr17	2320628	2320628	T	C	UTR3	METTL16	ENST000002	.	.	A	G	-	-	0.09302326	39,0,4,0	chr17,23206	Cytosol	A > G
chr17:2320645:T:C:UTR3:METTL1	chr17	2320645	2320645	T	C	UTR3	METTL16	ENST000002	.	.	A	G	-	-	0.04166667	46,0,2,0	chr17,23206	Cytosol	A > G
chr17:2320651:T:C:UTR3:METTL1	chr17	2320651	2320651	T	C	UTR3	METTL16	ENST000002	.	.	A	G	-	-	0.74137931	15,0,43,0	chr17,23206	Cytosol	A > G
chr17:2321606:T:C:UTR3:METTL1	chr17	2321606	2321606	T	C	UTR3	METTL16	ENST000002	.	.	A	G	-	-	0.140625	55,0,9,0	chr17,23216	Cytosol	A > G
chr17:2321628:T:C:UTR3:METTL1	chr17	2321628	2321628	T	C	UTR3	METTL16	ENST000002	.	.	A	G	-	-	0.05454545	52,0,3,0	chr17,23216	Cytosol	A > G
chr17:2321687:T:C:UTR3:METTL1	chr17	2321687	2321687	T	C	UTR3	METTL16	ENST000002	.	.	A	G	-	-	0.1627907	36,0,7,0	chr17,23216	Cytosol	A > G
chr17:2321688:T:C:UTR3:METTL1	chr17	2321688	2321688	T	C	UTR3	METTL16	ENST000002	.	.	A	G	-	-	0.34090909	29,0,15,0	chr17,23216	Cytosol	A > G
chr17:2321705:T:C:UTR3:METTL1	chr17	2321705	2321705	T	C	UTR3	METTL16	ENST000002	.	.	A	G	-	-	0.2	32,0,8,0	chr17,23217	Cytosol	A > G
chr17:2321719:T:C:UTR3:METTL1	chr17	2321719	2321719	T	C	UTR3	METTL16	ENST000002	.	.	A	G	-	-	0.2	16,0,4,0	chr17,23217	Cytosol	A > G
chr17:2321762:T:C:UTR3:METTL1	chr17	2321762	2321762	T	C	UTR3	METTL16	ENST000002	.	.	A	G	-	-	0.85714286	2,0,12,0	chr17,23217	Cytosol	A > G
chr17:2321772:T:C:UTR3:METTL1	chr17	2321772	2321772	T	C	UTR3	METTL16	ENST000002	.	.	A	G	-	-	0.73333333	4,0,11,0	chr17,23217	Cytosol	A > G
chr17:2321776:T:C:UTR3:METTL1	chr17	2321776	2321776	T	C	UTR3	METTL16	ENST000002	.	.	A	G	-	-	0.46666667	8,0,7,0	chr17,23217	Cytosol	A > G
chr17:2321801:T:C:UTR3:METTL1	chr17	2321801	2321801	T	C	UTR3	METTL16	ENST000002	.	.	A	G	-	-	0.85714286	2,0,12,0	chr17,23218	Cytosol	A > G
chr17:3512293:T:C:UTR3:SHPK	chr17	3512293	3512293	T	C	UTR3	SHPK	ENST000002	.	.	A	G	-	-	0.125	21,0,3,0	chr17,35122	Cytosol	A > G
chr17:3512323:T:C:UTR3:SHPK	chr17	3512323	3512323	T	C	UTR3	SHPK	ENST000002	.	.	A	G	-	-	0.05405405	35,0,2,0	chr17,35123	Cytosol	A > G
chr17:3512324:T:C:UTR3:SHPK	chr17	3512324	3512324	T	C	UTR3	SHPK	ENST000002	.	.	A	G	-	-	0.05128205	37,0,2,0	chr17,35123	Cytosol	A > G
chr17:3712086:T:C:ncRNA_exonic:	chr17	3712086	3712086	T	C	ncRNA_exon	CTD-3195I5.	.	.	.	A	G	-	-	0.1	36,0,4,0	chr17,37120	Cytosol	A > G
chr17:4928295:A:G:UTR3:KIF1C	chr17	4928295	4928295	A	G	UTR3	KIF1C	ENST000003	.	.	A	G	+	+	0.30769231	18,0,8,0	chr17,49282	Cytosol	A > G
chr17:4928305:A:G:UTR3:KIF1C	chr17	4928305	4928305	A	G	UTR3	KIF1C	ENST000003	.	.	A	G	+	+	0.36	16,0,9,0	chr17,49283	Cytosol	A > G
chr17:4928349:A:G:UTR3:KIF1C	chr17	4928349	4928349	A	G	UTR3	KIF1C	ENST000003	.	.	A	G	+	+	0.125	21,0,3,0	chr17,49283	Cytosol	A > G

chr17:4928363:A:G:UTR3:KIF1C	chr17	4928363	4928363	A	G	UTR3	KIF1C	ENST000003	.	.	A	G	+	+	0.125	21,0,3,0	chr17,49283	Cytosol	A > G
chr17:4928366:A:G:UTR3:KIF1C	chr17	4928366	4928366	A	G	UTR3	KIF1C	ENST000003	.	.	A	G	+	+	0.12	22,0,3,0	chr17,49283	Cytosol	A > G
chr17:4928416:A:G:UTR3:KIF1C	chr17	4928416	4928416	A	G	UTR3	KIF1C	ENST000003	.	.	A	G	+	+	0.17647059	14,0,3,0	chr17,49284	Cytosol	A > G
chr17:4928430:A:G:UTR3:KIF1C	chr17	4928430	4928430	A	G	UTR3	KIF1C	ENST000003	.	.	A	G	+	+	0.08823529	31,0,3,0	chr17,49284	Cytosol	A > G
chr17:4928835:A:G:UTR3:KIF1C	chr17	4928835	4928835	A	G	UTR3	KIF1C	ENST000003	.	.	A	G	+	+	0.32142857	19,0,9,0	chr17,49288	Cytosol	A > G
chr17:4928841:A:G:UTR3:KIF1C	chr17	4928841	4928841	A	G	UTR3	KIF1C	ENST000003	.	.	A	G	+	+	0.13333333	26,0,4,0	chr17,49288	Cytosol	A > G
chr17:4928994:A:G:UTR3:KIF1C	chr17	4928994	4928994	A	G	UTR3	KIF1C	ENST000003	.	.	A	G	+	+	0.5	10,0,10,0	chr17,49289	Cytosol	A > G
chr17:4929004:A:G:UTR3:KIF1C	chr17	4929004	4929004	A	G	UTR3	KIF1C	ENST000003	.	.	A	G	+	+	0.1	18,0,2,0	chr17,49290	Cytosol	A > G
chr17:4929776:A:G:UTR3:KIF1C	chr17	4929776	4929776	A	G	UTR3	KIF1C	ENST000003	.	.	A	G	+	+	0.06451613	29,0,2,0	chr17,49297	Cytosol	A > G
chr17:4929783:A:G:UTR3:KIF1C	chr17	4929783	4929783	A	G	UTR3	KIF1C	ENST000003	.	.	A	G	+	+	0.39393939	20,0,13,0	chr17,49297	Cytosol	A > G
chr17:4929789:A:G:UTR3:KIF1C	chr17	4929789	4929789	A	G	UTR3	KIF1C	ENST000003	.	.	A	G	+	+	0.10526316	34,0,4,0	chr17,49297	Cytosol	A > G
chr17:4929790:A:G:UTR3:KIF1C	chr17	4929790	4929790	A	G	UTR3	KIF1C	ENST000003	.	.	A	G	+	+	0.23684211	29,0,9,0	chr17,49297	Cytosol	A > G
chr17:4929816:A:G:UTR3:KIF1C	chr17	4929816	4929816	A	G	UTR3	KIF1C	ENST000003	.	.	A	G	+	+	0.27272727	16,0,6,0	chr17,49298	Cytosol	A > G
chr17:4929861:A:G:UTR3:KIF1C	chr17	4929861	4929861	A	G	UTR3	KIF1C	ENST000003	.	.	A	G	+	+	0.06060606	31,0,2,0	chr17,49298	Cytosol	A > G
chr17:4929863:A:G:UTR3:KIF1C	chr17	4929863	4929863	A	G	UTR3	KIF1C	ENST000003	.	.	A	G	+	+	0.32258065	21,0,10,0	chr17,49298	Cytosol	A > G
chr17:4929954:A:G:UTR3:KIF1C	chr17	4929954	4929954	A	G	UTR3	KIF1C	ENST000003	.	.	A	G	+	+	0.19565217	37,0,9,0	chr17,49299	Cytosol	A > G
chr17:4930105:A:G:UTR3:KIF1C	chr17	4930105	4930105	A	G	UTR3	KIF1C	ENST000003	.	.	A	G	+	+	0.23333333	23,0,7,0	chr17,49301	Cytosol	A > G
chr17:4930111:A:G:UTR3:KIF1C	chr17	4930111	4930111	A	G	UTR3	KIF1C	ENST000003	.	.	A	G	+	+	0.14285714	24,0,4,0	chr17,49301	Cytosol	A > G
chr17:4930121:A:G:UTR3:KIF1C	chr17	4930121	4930121	A	G	UTR3	KIF1C	ENST000003	.	.	A	G	+	+	0.24137931	22,0,7,0	chr17,49301	Cytosol	A > G
chr17:4930488:A:G:UTR3:KIF1C	chr17	4930488	4930488	A	G	UTR3	KIF1C	ENST000003	.	.	A	G	+	+	0.11111111	24,0,3,0	chr17,49304	Cytosol	A > G
chr17:4930501:A:G:UTR3:KIF1C	chr17	4930501	4930501	A	G	UTR3	KIF1C	ENST000003	.	.	A	G	+	+	0.08333333	22,0,2,0	chr17,49305	Cytosol	A > G
chr17:4930503:A:G:UTR3:KIF1C	chr17	4930503	4930503	A	G	UTR3	KIF1C	ENST000003	.	.	A	G	+	+	0.13636364	19,0,3,0	chr17,49305	Cytosol	A > G
chr17:4930504:A:G:UTR3:KIF1C	chr17	4930504	4930504	A	G	UTR3	KIF1C	ENST000003	.	.	A	G	+	+	0.42857143	12,0,9,0	chr17,49305	Cytosol	A > G
chr17:4930524:A:G:UTR3:KIF1C	chr17	4930524	4930524	A	G	UTR3	KIF1C	ENST000003	.	.	A	G	+	+	0.45454545	12,0,10,0	chr17,49305	Cytosol	A > G
chr17:4930557:A:G:UTR3:KIF1C	chr17	4930557	4930557	A	G	UTR3	KIF1C	ENST000003	.	.	A	G	+	+	0.625	6,0,10,0	chr17,49305	Cytosol	A > G
chr17:4930565:A:G:UTR3:KIF1C	chr17	4930565	4930565	A	G	UTR3	KIF1C	ENST000003	.	.	A	G	+	+	0.26315789	14,0,5,0	chr17,49305	Cytosol	A > G
chr17:4930590:A:G:UTR3:KIF1C	chr17	4930590	4930590	A	G	UTR3	KIF1C	ENST000003	.	.	A	G	+	+	0.10526316	17,0,2,0	chr17,49305	Cytosol	A > G
chr17:4930605:A:G:UTR3:KIF1C	chr17	4930605	4930605	A	G	UTR3	KIF1C	ENST000003	.	.	A	G	+	+	0.0952381	19,0,2,0	chr17,49306	Cytosol	A > G
chr17:4930625:A:G:UTR3:KIF1C	chr17	4930625	4930625	A	G	UTR3	KIF1C	ENST000003	.	.	A	G	+	+	0.23529412	13,0,4,0	chr17,49306	Cytosol	A > G
chr17:4930659:A:G:UTR3:KIF1C	chr17	4930659	4930659	A	G	UTR3	KIF1C	ENST000003	.	.	A	G	+	+	0.45454545	6,0,5,0	chr17,49306	Cytosol	A > G
chr17:4930687:A:G:UTR3:KIF1C	chr17	4930687	4930687	A	G	UTR3	KIF1C	ENST000003	.	.	A	G	+	+	0.36363636	14,0,8,0	chr17,49306	Cytosol	A > G
chr17:4930688:A:G:UTR3:KIF1C	chr17	4930688	4930688	A	G	UTR3	KIF1C	ENST000003	.	.	A	G	+	+	0.42857143	12,0,9,0	chr17,49306	Cytosol	A > G
chr17:4930699:A:G:UTR3:KIF1C	chr17	4930699	4930699	A	G	UTR3	KIF1C	ENST000003	.	.	A	G	+	+	0.375	15,0,9,0	chr17,49306	Cytosol	A > G
chr17:4930742:A:G:UTR3:KIF1C	chr17	4930742	4930742	A	G	UTR3	KIF1C	ENST000003	.	.	A	G	+	+	0.29032258	21,0,9,1	chr17,49307	Cytosol	A > G
chr17:4930745:A:G:UTR3:KIF1C	chr17	4930745	4930745	A	G	UTR3	KIF1C	ENST000003	.	.	A	G	+	+	0.11111111	24,0,3,0	chr17,49307	Cytosol	A > G
chr17:4930749:A:G:UTR3:KIF1C	chr17	4930749	4930749	A	G	UTR3	KIF1C	ENST000003	.	.	A	G	+	+	0.33333333	20,0,10,0	chr17,49307	Cytosol	A > G
chr17:4930751:A:G:UTR3:KIF1C	chr17	4930751	4930751	A	G	UTR3	KIF1C	ENST000003	.	.	A	G	+	+	0.1875	26,0,6,0	chr17,49307	Cytosol	A > G
chr17:4931018:A:G:UTR3:KIF1C	chr17	4931018	4931018	A	G	UTR3	KIF1C	ENST000003	.	.	A	G	+	+	0.27027027	27,0,10,0	chr17,49310	Cytosol	A > G
chr17:4931019:A:G:UTR3:KIF1C	chr17	4931019	4931019	A	G	UTR3	KIF1C	ENST000003	.	.	A	G	+	+	0.34285714	23,0,12,0	chr17,49310	Cytosol	A > G
chr17:4931064:A:G:UTR3:KIF1C	chr17	4931064	4931064	A	G	UTR3	KIF1C	ENST000003	.	.	A	G	+	+	0.4	9,0,6,0	chr17,49310	Cytosol	A > G
chr17:4931092:A:G:UTR3:KIF1C	chr17	4931092	4931092	A	G	UTR3	KIF1C	ENST000003	.	.	A	G	+	+	0.17647059	14,0,3,0	chr17,49310	Cytosol	A > G
chr17:4931093:A:G:UTR3:KIF1C	chr17	4931093	4931093	A	G	UTR3	KIF1C	ENST000003	.	.	A	G	+	+	0.41176471	10,0,7,0	chr17,49310	Cytosol	A > G
chr17:4931114:A:G:UTR3:KIF1C	chr17	4931114	4931114	A	G	UTR3	KIF1C	ENST000003	.	.	A	G	+	+	0.11538462	23,0,3,0	chr17,49311	Cytosol	A > G
chr17:4931117:A:G:UTR3:KIF1C	chr17	4931117	4931117	A	G	UTR3	KIF1C	ENST000003	.	.	A	G	+	+	0.36	16,0,9,0	chr17,49311	Cytosol	A > G
chr17:4931122:A:G:UTR3:KIF1C	chr17	4931122	4931122	A	G	UTR3	KIF1C	ENST000003	.	.	A	G	+	+	0.17391304	19,0,4,0	chr17,49311	Cytosol	A > G
chr17:4931126:A:G:UTR3:KIF1C	chr17	4931126	4931126	A	G	UTR3	KIF1C	ENST000003	.	.	A	G	+	+	0.08333333	22,0,2,0	chr17,49311	Cytosol	A > G
chr17:4931146:A:G:UTR3:KIF1C	chr17	4931146	4931146	A	G	UTR3	KIF1C	ENST000003	.	.	A	G	+	+	0.15384615	22,0,4,0	chr17,49311	Cytosol	A > G
chr17:5390516:A:G:intronic:MIS12	chr17	5390516	5390516	A	G	intronic	MIS12	.	.	.	A	G	+	+	0.14285714	12,0,2,0	chr17,53905	Cytosol	A > G
chr17:7572004:T:C:UTR3:TP53	chr17	7572004	7572004	T	C	UTR3	TP53	ENST0000002	.	.	A	G	-	-	0.06060606	124,0,8,0	chr17,75720	Cytosol	A > G
chr17:8173249:A:G:UTR3:PFAS	chr17	8173249	8173249	A	G	UTR3	PFAS	ENST000003	.	.	A	G	+	+	0.17117117	92,0,19,0	chr17,81732	Cytosol	A > G
chr17:8173252:A:G:UTR3:PFAS	chr17	8173252	8173252	A	G	UTR3	PFAS	ENST000003	.	.	A	G	+	+	0.09090909	110,0,11,0	chr17,81732	Cytosol	A > G
chr17:8173295:A:G:UTR3:PFAS	chr17	8173295	8173295	A	G	UTR3	PFAS	ENST000003	.	.	A	G	+	+	0.02702703	108,0,3,0	chr17,81732	Cytosol	A > G
chr17:8173296:A:G:UTR3:PFAS	chr17	8173296	8173296	A	G	UTR3	PFAS	ENST000003	.	.	A	G	+	+	0.05607477	101,0,6,0	chr17,81732	Cytosol	A > G
chr17:8173299:A:G:UTR3:PFAS	chr17	8173299	8173299	A	G	UTR3	PFAS	ENST000003	.	.	A	G	+	+	0.11504425	100,0,13,0	chr17,81732	Cytosol	A > G
chr17:8173300:A:G:UTR3:PFAS	chr17	8173300	8173300	A	G	UTR3	PFAS	ENST000003	.	.	A	G	+	+	0.05607477	101,0,6,0	chr17,81732	Cytosol	A > G
chr17:8173325:A:G:UTR3:PFAS	chr17	8173325	8173325	A	G	UTR3	PFAS	ENST000003	.	.	A	G	+	+	0.08	69,0,6,0	chr17,81733	Cytosol	A > G
chr17:8173376:A:G:UTR3:PFAS	chr17	8173376	8173376	A	G	UTR3	PFAS	ENST000003	.	.	A	G	+	+	0.13636364	57,0,9,0	chr17,81733	Cytosol	A > G
chr17:8173379:A:G:UTR3:PFAS	chr17	8173379	8173379	A	G	UTR3	PFAS	ENST000003	.	.	A	G	+	+	0.06849315	68,0,5,0	chr17,81733	Cytosol	A > G
chr17:8173396:A:G:UTR3:PFAS	chr17	8173396	8173396	A	G	UTR3	PFAS	ENST000003	.	.	A	G	+	+	0.04166667	115,0,5,0	chr17,81733	Cytosol	A > G
chr17:8173431:A:G:UTR3:PFAS	chr17	8173431	8173431	A	G	UTR3	PFAS	ENST000003	.	.	A	G	+	+	0.03389831	114,0,4,0	chr17,81734	Cytosol	A > G
chr17:8271330:T:C:ncRNA_intronic	chr17	8271330	8271330	T	C	ncRNA_intro	RP11-849F2.	.	.	.	A	G	-	+	0.33333333	4,0,2,0	chr17,82713	Cytosol	A > G
chr17:10576406:T:C:ncRNA_exoni	chr17	10576406	10576406	T	C	ncRNA_exon	CTC-297N7.8	.	.	.	A	G	-	-	0.83333333	1,0,5,0	chr17,10576	Cytosol	A > G
chr17:10576426:T:C:ncRNA_exoni	chr17	10576426	10576426	T	C	ncRNA_exon	CTC-297N7.8	.	.	.	A	G	-	-	0.28571429	5,0,2,0	chr17,10576	Cytosol	A > G
chr17:10576449:T:C:ncRNA_exoni	chr17	10576449	10576449	T	C	ncRNA_exon	CTC-297N7.8	.	.	.	A	G	-	-	0.83333333	1,0,5,0	chr17,10576	Cytosol	A > G

chr17:10576453:T:C:ncRNA_exoni	chr17	10576453	10576453	T	C	ncRNA_exon	CTC-297N7.8	.	.	.	A	G	-	-	0.4	3,0,2,0	chr17,10576	Cytosol	A > G
chr17:10576459:T:C:ncRNA_exoni	chr17	10576459	10576459	T	C	ncRNA_exon	CTC-297N7.8	.	.	.	A	G	-	-	0.5	3,0,3,0	chr17,10576	Cytosol	A > G
chr17:10576509:T:C:ncRNA_exoni	chr17	10576509	10576509	T	C	ncRNA_exon	CTC-297N7.8	.	.	.	A	G	-	-	0.26666667	11,0,4,0	chr17,10576	Cytosol	A > G
chr17:10576524:T:C:ncRNA_exoni	chr17	10576524	10576524	T	C	ncRNA_exon	CTC-297N7.8	.	.	.	A	G	-	-	0.13636364	19,0,3,0	chr17,10576	Cytosol	A > G
chr17:10576547:T:C:ncRNA_exoni	chr17	10576547	10576547	T	C	ncRNA_exon	CTC-297N7.8	.	.	.	A	G	-	-	0.70833333	7,0,17,0	chr17,10576	Cytosol	A > G
chr17:10577487:T:C:ncRNA_exoni	chr17	10577487	10577487	T	C	ncRNA_exon	CTC-297N7.8	.	.	.	A	G	-	-	0.125	14,0,2,0	chr17,10577	Cytosol	A > G
chr17:10577569:T:C:ncRNA_exoni	chr17	10577569	10577569	T	C	ncRNA_exon	CTC-297N7.8	.	.	.	A	G	-	-	0.22222222	7,0,2,0	chr17,10577	Cytosol	A > G
chr17:10577621:T:C:ncRNA_exoni	chr17	10577621	10577621	T	C	ncRNA_exon	CTC-297N7.8	.	.	.	A	G	-	-	0.85714286	2,0,12,0	chr17,10577	Cytosol	A > G
chr17:10577631:T:C:ncRNA_exoni	chr17	10577631	10577631	T	C	ncRNA_exon	CTC-297N7.8	.	.	.	A	G	-	-	1	0,0,7,0	chr17,10577	Cytosol	A > G
chr17:10577660:T:C:ncRNA_exoni	chr17	10577660	10577660	T	C	ncRNA_exon	CTC-297N7.8	.	.	.	A	G	-	-	0.33333333	6,0,3,0	chr17,10577	Cytosol	A > G
chr17:10577661:T:C:ncRNA_exoni	chr17	10577661	10577661	T	C	ncRNA_exon	CTC-297N7.8	.	.	.	A	G	-	-	0.22222222	7,0,2,0	chr17,10577	Cytosol	A > G
chr17:10577674:T:C:ncRNA_exoni	chr17	10577674	10577674	T	C	ncRNA_exon	CTC-297N7.8	.	.	.	A	G	-	-	0.8	2,0,8,0	chr17,10577	Cytosol	A > G
chr17:15890163:T:C:exonic:ZSWIN	chr17	15890163	15890163	T	C	exonic	ZSWIM7	.	stoploss	ZSWIM7:ENS	A	G	-	-	0.57142857	3,0,4,0	chr17,15890	Cytosol	A > G
chr17:16286322:A:G:upstream;do	chr17	16286322	16286322	A	G	upstream;do	RP11-138I1.4	.	.	.	A	G	+		0.17073171	34,0,7,0	chr17,16286	Cytosol	A > G
chr17:16286355:A:G:upstream;do	chr17	16286355	16286355	A	G	upstream;do	RP11-138I1.4	.	.	.	A	G	+		0.11764706	15,0,2,0	chr17,16286	Cytosol	A > G
chr17:16286366:A:G:upstream;do	chr17	16286366	16286366	A	G	upstream;do	RP11-138I1.4	.	.	.	A	G	+		0.33333333	10,0,5,0	chr17,16286	Cytosol	A > G
chr17:16286369:A:G:upstream;do	chr17	16286369	16286369	A	G	upstream;do	RP11-138I1.4	.	.	.	A	G	+		0.2	12,0,3,0	chr17,16286	Cytosol	A > G
chr17:16286409:A:G:upstream;do	chr17	16286409	16286409	A	G	upstream;do	RP11-138I1.4	.	.	.	A	G	+		0.85714286	2,0,12,0	chr17,16286	Cytosol	A > G
chr17:16287196:A:G:intergenic:UE	chr17	16287196	16287196	A	G	intergenic	UBB,RP11-13	dist=1142;dis	.	.	A	G	+		0.35714286	9,0,5,0	chr17,16287	Cytosol	A > G
chr17:18175151:T:C:intronic:TOP3	chr17	18175151	18175151	T	C	intronic	TOP3A	.	.	.	A	G	-	-	0.47619048	22,0,20,0	chr17,18175	Cytosol	A > G
chr17:18175173:T:C:intronic:TOP3	chr17	18175173	18175173	T	C	intronic	TOP3A	.	.	.	A	G	-	-	0.42857143	20,0,15,0	chr17,18175	Cytosol	A > G
chr17:18175174:T:C:intronic:TOP3	chr17	18175174	18175174	T	C	intronic	TOP3A	.	.	.	A	G	-	-	0.84848485	5,0,28,0	chr17,18175	Cytosol	A > G
chr17:18175209:T:C:intronic:TOP3	chr17	18175209	18175209	T	C	intronic	TOP3A	.	.	.	A	G	-	-	0.09090909	20,0,2,0	chr17,18175	Cytosol	A > G
chr17:18175210:T:C:intronic:TOP3	chr17	18175210	18175210	T	C	intronic	TOP3A	.	.	.	A	G	-	-	0.40909091	13,0,9,0	chr17,18175	Cytosol	A > G
chr17:18175211:T:C:intronic:TOP3	chr17	18175211	18175211	T	C	intronic	TOP3A	.	.	.	A	G	-	-	0.27272727	16,0,6,0	chr17,18175	Cytosol	A > G
chr17:18175396:T:C:intronic:TOP3	chr17	18175396	18175396	T	C	intronic	TOP3A	.	.	.	A	G	-	-	0.31428571	24,0,11,0	chr17,18175	Cytosol	A > G
chr17:18176067:T:C:intronic:TOP3	chr17	18176067	18176067	T	C	intronic	TOP3A	.	.	.	A	G	-	-	0.53571429	13,0,15,0	chr17,18176	Cytosol	A > G
chr17:18176090:T:C:intronic:TOP3	chr17	18176090	18176090	T	C	intronic	TOP3A	.	.	.	A	G	-	-	0.08333333	33,0,3,0	chr17,18176	Cytosol	A > G
chr17:18177375:T:C:UTR3:TOP3A	chr17	18177375	18177375	T	C	UTR3	TOP3A	ENST000003	.	.	A	G	-	-	0.0952381	19,0,2,0	chr17,18177	Cytosol	A > G
chr17:18177379:T:C:UTR3:TOP3A	chr17	18177379	18177379	T	C	UTR3	TOP3A	ENST000003	.	.	A	G	-	-	0.11538462	23,0,3,0	chr17,18177	Cytosol	A > G
chr17:18177389:T:C:UTR3:TOP3A	chr17	18177389	18177389	T	C	UTR3	TOP3A	ENST000003	.	.	A	G	-	-	0.10714286	25,0,3,0	chr17,18177	Cytosol	A > G
chr17:18177430:T:C:UTR3:TOP3A	chr17	18177430	18177430	T	C	UTR3	TOP3A	ENST000003	.	.	A	G	-	-	0.16666667	20,0,4,0	chr17,18177	Cytosol	A > G
chr17:19349456:T:C:ncRNA_intror	chr17	19349456	19349456	T	C	ncRNA_intro	AC004448.5	.	.	.	A	G	-	-	0.6	4,0,6,0	chr17,19349	Cytosol	A > G
chr17:20220954:A:G:ncRNA_intror	chr17	20220954	20220954	A	G	ncRNA_intro	AC004702.2	.	.	.	A	G	+	+	0.2	8,0,2,0	chr17,20220	Cytosol	A > G
chr17:25639871:A:G:UTR3:WSB1	chr17	25639871	25639871	A	G	UTR3	WSB1	ENST000002	.	.	A	G	+	+	0.03448276	56,0,2,0	chr17,25639	Cytosol	A > G
chr17:25640011:A:G:UTR3:WSB1	chr17	25640011	25640011	A	G	UTR3	WSB1	ENST000002	.	.	A	G	+	+	0.12765957	41,0,6,0	chr17,25640	Cytosol	A > G
chr17:25641831:A:G:ncRNA_exoni	chr17	25641831	25641831	A	G	ncRNA_exon	RP11-173M1	.	.	.	A	G	+	+	0.4	3,0,2,0	chr17,25641	Cytosol	A > G
chr17:28963253:A:G:ncRNA_exoni	chr17	28963253	28963253	A	G	ncRNA_exon	LRRC37BP1	.	.	.	A	G	+	+	0.13333333	13,0,2,0	chr17,28963	Cytosol	A > G
chr17:28963296:A:G:ncRNA_exoni	chr17	28963296	28963296	A	G	ncRNA_exon	LRRC37BP1	.	.	.	A	G	+	+	0.19047619	17,0,4,0	chr17,28963	Cytosol	A > G
chr17:29110384:T:C:ncRNA_intror	chr17	29110384	29110384	T	C	ncRNA_intro	SUZ12P	.	.	.	A	G	-	+-	0.02290076	128,0,3,0	chr17,29110	Cytosol	A > G
chr17:29225503:A:G:downstream:	chr17	29225503	29225503	A	G	downstream	TEFM	.	.	.	A	G	+	-	0.5	3,0,3,0	chr17,29225	Cytosol	A > G
chr17:29285710:A:G:UTR3:ADAP2	chr17	29285710	29285710	A	G	UTR3	ADAP2	ENST000005	.	.	A	G	+	+	0.02197802	89,0,2,0	chr17,29285	Cytosol	A > G
chr17:29285726:A:G:UTR3:ADAP2	chr17	29285726	29285726	A	G	UTR3	ADAP2	ENST000005	.	.	A	G	+	+	0.04166667	92,0,4,0	chr17,29285	Cytosol	A > G
chr17:29285743:A:G:UTR3:ADAP2	chr17	29285743	29285743	A	G	UTR3	ADAP2	ENST000005	.	.	A	G	+	+	0.01960784	100,0,2,0	chr17,29285	Cytosol	A > G
chr17:29285786:A:G:UTR3:ADAP2	chr17	29285786	29285786	A	G	UTR3	ADAP2	ENST000003	.	.	A	G	+	+	0.03	97,0,3,0	chr17,29285	Cytosol	A > G
chr17:29300997:A:G:intronic:RNF1	chr17	29300997	29300997	A	G	intronic	RNF135	.	.	.	A	G	+	+	0.77777778	2,0,7,0	chr17,29300	Cytosol	A > G
chr17:29301143:A:G:intronic:RNF1	chr17	29301143	29301143	A	G	intronic	RNF135	.	.	.	A	G	+	+	0.6	2,0,3,0	chr17,29301	Cytosol	A > G
chr17:29861528:A:G:UTR3:RAB11I	chr17	29861528	29861528	A	G	UTR3	RAB11FIP4	ENST000003	.	.	A	G	+	+	0.28571429	5,0,2,0	chr17,29861	Cytosol	A > G
chr17:30188603:T:C:UTR3:UTP6	chr17	30188603	30188603	T	C	UTR3	UTP6	ENST000002	.	.	A	G	-	-	0.43478261	13,0,10,0	chr17,30188	Cytosol	A > G
chr17:30189147:T:C:UTR3:UTP6	chr17	30189147	30189147	T	C	UTR3	UTP6	ENST000002	.	.	A	G	-	-	0.66666667	3,0,6,0	chr17,30189	Cytosol	A > G
chr17:33421181:T:C:intronic:RAD5	chr17	33421181	33421181	T	C	intronic	RAD51L3-RFI	.	.	.	A	G	-	-	0.28571429	5,0,2,0	chr17,33421	Cytosol	A > G
chr17:33423833:T:C:intronic:RAD5	chr17	33423833	33423833	T	C	intronic	RAD51L3-RFI	.	.	.	A	G	-	-	0.28571429	5,0,2,0	chr17,33423	Cytosol	A > G
chr17:33425377:T:C:intronic:RAD5	chr17	33425377	33425377	T	C	intronic	RAD51L3-RFI	.	.	.	A	G	-	-	0.06382979	44,0,3,0	chr17,33425	Cytosol	A > G
chr17:34886848:T:C:intronic:MYO	chr17	34886848	34886848	T	C	intronic	MYO19	.	.	.	A	G	-	-	0.14285714	12,0,2,0	chr17,34886	Cytosol	A > G
chr17:35837647:A:G:UTR3:TADA2	chr17	35837647	35837647	A	G	UTR3	TADA2A	ENST000003	.	.	A	G	+	+	0.125	14,0,2,0	chr17,35837	Cytosol	A > G
chr17:35837709:A:G:UTR3:TADA2	chr17	35837709	35837709	A	G	UTR3	TADA2A	ENST000003	.	.	A	G	+	+	0.06666667	28,0,2,0	chr17,35837	Cytosol	A > G
chr17:35837725:A:G:UTR3:TADA2	chr17	35837725	35837725	A	G	UTR3	TADA2A	ENST000003	.	.	A	G	+	+	0.13043478	20,0,3,0	chr17,35837	Cytosol	A > G
chr17:35837749:A:G:UTR3:TADA2	chr17	35837749	35837749	A	G	UTR3	TADA2A	ENST000003	.	.	A	G	+	+	0.2	20,0,5,0	chr17,35837	Cytosol	A > G
chr17:35837752:A:G:UTR3:TADA2	chr17	35837752	35837752	A	G	UTR3	TADA2A	ENST000003	.	.	A	G	+	+	0.66666667	8,0,16,0	chr17,35837	Cytosol	A > G
chr17:35837834:A:G:UTR3:TADA2	chr17	35837834	35837834	A	G	UTR3	TADA2A	ENST000003	.	.	A	G	+	+	0.15	34,0,6,0	chr17,35837	Cytosol	A > G
chr17:35837837:A:G:UTR3:TADA2	chr17	35837837	35837837	A	G	UTR3	TADA2A	ENST000003	.	.	A	G	+	+	0.58974359	16,0,23,0	chr17,35837	Cytosol	A > G
chr17:35837977:A:G:UTR3:TADA2	chr17	35837977	35837977	A	G	UTR3	TADA2A	ENST000003	.	.	A	G	+	+	0.2	24,0,6,0	chr17,35837	Cytosol	A > G
chr17:35838015:A:G:UTR3:TADA2	chr17	35838015	35838015	A	G	UTR3	TADA2A	ENST000003	.	.	A	G	+	+	0.78571429	3,0,11,0	chr17,35838	Cytosol	A > G
chr17:35838102:A:G:UTR3:TADA2	chr17	35838102	35838102	A	G	UTR3	TADA2A	ENST000003	.	.	A	G	+	+	0.1	27,0,3,0	chr17,35838	Cytosol	A > G

chr17:35838103:A:G:UTR3:TADA2	chr17	35838103	35838103	A	G	UTR3	TADA2A	ENST000003	.	.	A	G	+	+	0.5625	14,0,18,0	chr17,35838	Cytosol	A > G
chr17:35838116:A:G:UTR3:TADA2	chr17	35838116	35838116	A	G	UTR3	TADA2A	ENST000003	.	.	A	G	+	+	0.90909091	3,0,30,0	chr17,35838	Cytosol	A > G
chr17:35838222:C:A:UTR3:TADA2	chr17	35838222	35838222	C	A	UTR3	TADA2A	ENST000003	.	.	C	A	+	+	0.21428571	3,11,0,0	chr17,35838	Cytosol	C > A
chr17:35875690:T:C:UTR3:SYNRG	chr17	35875690	35875690	T	C	UTR3	SYNRG	ENST000003	.	.	A	G	-	-	0.35714286	9,0,5,0	chr17,35875	Cytosol	A > G
chr17:35875739:T:C:UTR3:SYNRG	chr17	35875739	35875739	T	C	UTR3	SYNRG	ENST000003	.	.	A	G	-	-	0.375	5,0,3,0	chr17,35875	Cytosol	A > G
chr17:35875740:T:C:UTR3:SYNRG	chr17	35875740	35875740	T	C	UTR3	SYNRG	ENST000003	.	.	A	G	-	-	0.5	4,0,4,0	chr17,35875	Cytosol	A > G
chr17:35875741:T:C:UTR3:SYNRG	chr17	35875741	35875741	T	C	UTR3	SYNRG	ENST000003	.	.	A	G	-	-	0.28571429	5,0,2,0	chr17,35875	Cytosol	A > G
chr17:35875744:T:C:UTR3:SYNRG	chr17	35875744	35875744	T	C	UTR3	SYNRG	ENST000003	.	.	A	G	-	-	0.55555556	4,0,5,0	chr17,35875	Cytosol	A > G
chr17:35877106:T:C:UTR3:SYNRG	chr17	35877106	35877106	T	C	UTR3	SYNRG	ENST000003	.	.	A	G	-	-	0.25	9,0,3,0	chr17,35877	Cytosol	A > G
chr17:35877155:T:C:UTR3:SYNRG	chr17	35877155	35877155	T	C	UTR3	SYNRG	ENST000003	.	.	A	G	-	-	0.8	2,0,8,0	chr17,35877	Cytosol	A > G
chr17:35877161:T:C:UTR3:SYNRG	chr17	35877161	35877161	T	C	UTR3	SYNRG	ENST000003	.	.	A	G	-	-	0.22222222	7,0,2,0	chr17,35877	Cytosol	A > G
chr17:35877195:T:C:UTR3:SYNRG	chr17	35877195	35877195	T	C	UTR3	SYNRG	ENST000003	.	.	A	G	-	-	0.33333333	4,0,2,0	chr17,35877	Cytosol	A > G
chr17:35877234:T:C:UTR3:SYNRG	chr17	35877234	35877234	T	C	UTR3	SYNRG	ENST000003	.	.	A	G	-	-	0.42857143	4,0,3,0	chr17,35877	Cytosol	A > G
chr17:35877235:T:C:UTR3:SYNRG	chr17	35877235	35877235	T	C	UTR3	SYNRG	ENST000003	.	.	A	G	-	-	0.71428571	2,0,5,0	chr17,35877	Cytosol	A > G
chr17:35877273:T:C:UTR3:SYNRG	chr17	35877273	35877273	T	C	UTR3	SYNRG	ENST000003	.	.	A	G	-	-	0.83333333	1,0,5,0	chr17,35877	Cytosol	A > G
chr17:35877278:T:C:UTR3:SYNRG	chr17	35877278	35877278	T	C	UTR3	SYNRG	ENST000003	.	.	A	G	-	-	0.83333333	1,0,5,0	chr17,35877	Cytosol	A > G
chr17:35971949:T:C:ncRNA_intror	chr17	35971949	35971949	T	C	ncRNA_intro	RP11-697E22	.	.	.	A	G	-	+-	0.33333333	16,0,8,0	chr17,35971	Cytosol	A > G
chr17:35972194:T:C:ncRNA_intror	chr17	35972194	35972194	T	C	ncRNA_intro	RP11-697E22	.	.	.	A	G	-	+-	0.23529412	13,0,4,0	chr17,35972	Cytosol	A > G
chr17:35972195:T:C:ncRNA_intror	chr17	35972195	35972195	T	C	ncRNA_intro	RP11-697E22	.	.	.	A	G	-	+-	0.66666667	6,0,12,0	chr17,35972	Cytosol	A > G
chr17:35972246:T:C:ncRNA_intror	chr17	35972246	35972246	T	C	ncRNA_intro	RP11-697E22	.	.	.	A	G	-	+-	0.51282051	19,0,20,0	chr17,35972	Cytosol	A > G
chr17:35972250:T:C:ncRNA_intror	chr17	35972250	35972250	T	C	ncRNA_intro	RP11-697E22	.	.	.	A	G	-	+-	0.1025641	35,0,4,0	chr17,35972	Cytosol	A > G
chr17:35972349:T:C:ncRNA_intror	chr17	35972349	35972349	T	C	ncRNA_intro	RP11-697E22	.	.	.	A	G	-	+-	0.05882353	32,0,2,0	chr17,35972	Cytosol	A > G
chr17:35972372:T:C:ncRNA_intror	chr17	35972372	35972372	T	C	ncRNA_intro	RP11-697E22	.	.	.	A	G	-	+-	0.26923077	19,0,7,0	chr17,35972	Cytosol	A > G
chr17:35972394:T:C:ncRNA_intror	chr17	35972394	35972394	T	C	ncRNA_intro	RP11-697E22	.	.	.	A	G	-	+-	0.34615385	17,0,9,0	chr17,35972	Cytosol	A > G
chr17:35972395:T:C:ncRNA_intror	chr17	35972395	35972395	T	C	ncRNA_intro	RP11-697E22	.	.	.	A	G	-	+-	0.12	22,0,3,0	chr17,35972	Cytosol	A > G
chr17:36884054:G:A:UTR3:MLLT6	chr17	36884054	36884054	G	A	UTR3	MLLT6	ENST000003	.	.	G	A	+	+	0.06315789	6,0,89,0	chr17,36884	Cytosol	G > A
chr17:36932495:T:C:intronic:PIP4	chr17	36932495	36932495	T	C	intronic	PIP4K2B	.	.	.	A	G	-	-	0.25	9,0,3,0	chr17,36932	Cytosol	A > G
chr17:37004629:T:C:UTR3:RPL23	chr17	37004629	37004629	T	C	UTR3	RPL23	ENST000004	.	.	A	G	-	-	0.10344828	26,0,3,0	chr17,37004	Cytosol	A > G
chr17:37005759:T:C:UTR3:RPL23	chr17	37005759	37005759	T	C	UTR3	RPL23	ENST000004	.	.	A	G	-	-	0.02597403	75,0,2,0	chr17,37005	Cytosol	A > G
chr17:37005764:T:C:UTR3:RPL23	chr17	37005764	37005764	T	C	UTR3	RPL23	ENST000004	.	.	A	G	-	-	0.02857143	68,0,2,0	chr17,37005	Cytosol	A > G
chr17:37005768:T:C:UTR3:RPL23	chr17	37005768	37005768	T	C	UTR3	RPL23	ENST000004	.	.	A	G	-	-	0.04285714	67,0,3,0	chr17,37005	Cytosol	A > G
chr17:37005839:T:C:UTR3:RPL23	chr17	37005839	37005839	T	C	UTR3	RPL23	ENST000004	.	.	A	G	-	-	0.41463415	48,0,34,0	chr17,37005	Cytosol	A > G
chr17:37005840:T:C:UTR3:RPL23	chr17	37005840	37005840	T	C	UTR3	RPL23	ENST000004	.	.	A	G	-	-	0.04597701	83,0,4,0	chr17,37005	Cytosol	A > G
chr17:37006214:T:C:UTR3:RPL23	chr17	37006214	37006214	T	C	UTR3	RPL23	ENST000004	.	.	A	G	-	-	0.04255319	90,0,4,0	chr17,37006	Cytosol	A > G
chr17:37006223:T:C:UTR3:RPL23	chr17	37006223	37006223	T	C	UTR3	RPL23	ENST000004	.	.	A	G	-	-	0.06122449	92,0,6,0	chr17,37006	Cytosol	A > G
chr17:37006249:T:C:UTR3:RPL23	chr17	37006249	37006249	T	C	UTR3	RPL23	ENST000004	.	.	A	G	-	-	0.11594203	61,0,8,0	chr17,37006	Cytosol	A > G
chr17:37412452:T:C:intergenic:RP	chr17	37412452	37412452	T	C	intergenic	RP11-690G1	dist=1902;dis	.	.	A	G	-	-	0.15384615	11,0,2,0	chr17,37412	Cytosol	A > G
chr17:37412491:T:C:intergenic:RP	chr17	37412491	37412491	T	C	intergenic	RP11-690G1	dist=1941;dis	.	.	A	G	-	-	0.375	5,0,3,0	chr17,37412	Cytosol	A > G
chr17:37412492:T:C:intergenic:RP	chr17	37412492	37412492	T	C	intergenic	RP11-690G1	dist=1942;dis	.	.	A	G	-	-	0.22222222	7,0,2,0	chr17,37412	Cytosol	A > G
chr17:37412517:T:C:intergenic:RP	chr17	37412517	37412517	T	C	intergenic	RP11-690G1	dist=1967;dis	.	.	A	G	-	-	0.22222222	7,0,2,0	chr17,37412	Cytosol	A > G
chr17:37412530:T:C:intergenic:RP	chr17	37412530	37412530	T	C	intergenic	RP11-690G1	dist=1980;dis	.	.	A	G	-	-	0.33333333	6,0,3,0	chr17,37412	Cytosol	A > G
chr17:37413440:T:C:intergenic:RP	chr17	37413440	37413440	T	C	intergenic	RP11-690G1	dist=2890;dis	.	.	A	G	-	-	0.33333333	4,0,2,0	chr17,37413	Cytosol	A > G
chr17:38439317:A:G:UTR3:WIPF2	chr17	38439317	38439317	A	G	UTR3	WIPF2	ENST000003	.	.	A	G	+	+	1	0,0,5,0	chr17,38439	Cytosol	A > G
chr17:38439597:A:G:UTR3:WIPF2	chr17	38439597	38439597	A	G	UTR3	WIPF2	ENST000003	.	.	A	G	+	+	0.15384615	11,0,2,0	chr17,38439	Cytosol	A > G
chr17:38439614:A:G:UTR3:WIPF2	chr17	38439614	38439614	A	G	UTR3	WIPF2	ENST000003	.	.	A	G	+	+	0.58333333	5,0,7,0	chr17,38439	Cytosol	A > G
chr17:38439683:A:G:UTR3:WIPF2	chr17	38439683	38439683	A	G	UTR3	WIPF2	ENST000003	.	.	A	G	+	+	0.33333333	6,0,3,0	chr17,38439	Cytosol	A > G
chr17:38439684:A:G:UTR3:WIPF2	chr17	38439684	38439684	A	G	UTR3	WIPF2	ENST000003	.	.	A	G	+	+	0.27272727	8,0,3,0	chr17,38439	Cytosol	A > G
chr17:38439688:A:G:UTR3:WIPF2	chr17	38439688	38439688	A	G	UTR3	WIPF2	ENST000003	.	.	A	G	+	+	0.41666667	7,0,5,0	chr17,38439	Cytosol	A > G
chr17:38439708:A:G:UTR3:WIPF2	chr17	38439708	38439708	A	G	UTR3	WIPF2	ENST000003	.	.	A	G	+	+	0.33333333	4,0,2,0	chr17,38439	Cytosol	A > G
chr17:38439719:A:G:UTR3:WIPF2	chr17	38439719	38439719	A	G	UTR3	WIPF2	ENST000003	.	.	A	G	+	+	0.375	5,0,3,0	chr17,38439	Cytosol	A > G
chr17:38439751:A:G:UTR3:WIPF2	chr17	38439751	38439751	A	G	UTR3	WIPF2	ENST000003	.	.	A	G	+	+	0.4	3,0,2,0	chr17,38439	Cytosol	A > G
chr17:38439773:A:G:UTR3:WIPF2	chr17	38439773	38439773	A	G	UTR3	WIPF2	ENST000003	.	.	A	G	+	+	0.55555556	4,0,5,0	chr17,38439	Cytosol	A > G
chr17:38458843:A:G:UTR3:CDC6	chr17	38458843	38458843	A	G	UTR3	CDC6	ENST000002	.	.	A	G	+	+	0.02008032	244,0,5,0	chr17,38458	Cytosol	A > G
chr17:39982199:T:C:intronic:NT5C	chr17	39982199	39982199	T	C	intronic	NT5C3B	.	.	.	A	G	-	-	0.33333333	4,0,2,0	chr17,39982	Cytosol	A > G
chr17:40008468:T:C:upstream:RP1	chr17	40008468	40008468	T	C	upstream	RP11-156E6.	.	.	.	A	G	-	-	0.57142857	12,0,16,0	chr17,40008	Cytosol	A > G
chr17:40008495:T:C:upstream:RP1	chr17	40008495	40008495	T	C	upstream	RP11-156E6.	.	.	.	A	G	-	-	0.28571429	15,0,6,0	chr17,40008	Cytosol	A > G
chr17:41370416:A:G:ncRNA_intro	chr17	41370416	41370416	A	G	ncRNA_intro	LINC00854	.	.	.	A	G	+	+-	0.22222222	7,0,2,0	chr17,41370	Cytosol	A > G
chr17:41370433:A:G:ncRNA_intro	chr17	41370433	41370433	A	G	ncRNA_intro	LINC00854	.	.	.	A	G	+	+-	0.375	5,0,3,0	chr17,41370	Cytosol	A > G
chr17:41370460:A:G:ncRNA_intro	chr17	41370460	41370460	A	G	ncRNA_intro	LINC00854	.	.	.	A	G	+	+-	0.25	6,0,2,0	chr17,41370	Cytosol	A > G
chr17:41370630:A:G:ncRNA_intro	chr17	41370630	41370630	A	G	ncRNA_intro	LINC00854	.	.	.	A	G	+	+-	0.22222222	7,0,2,0	chr17,41370	Cytosol	A > G
chr17:42876293:T:C:UTR3:GJC1	chr17	42876293	42876293	T	C	UTR3	GJC1	ENST000005	.	.	A	G	-	-	0.12121212	28,0,4,1	chr17,42876	Cytosol	A > G
chr17:42876325:T:C:UTR3:GJC1	chr17	42876325	42876325	T	C	UTR3	GJC1	ENST000005	.	.	A	G	-	-	0.12121212	29,0,4,0	chr17,42876	Cytosol	A > G
chr17:42876329:T:C:UTR3:GJC1	chr17	42876329	42876329	T	C	UTR3	GJC1	ENST000005	.	.	A	G	-	-	0.1875	26,0,6,0	chr17,42876	Cytosol	A > G
chr17:42876394:T:C:UTR3:GJC1	chr17	42876394	42876394	T	C	UTR3	GJC1	ENST000005	.	.	A	G	-	-	0.23529412	39,0,12,0	chr17,42876	Cytosol	A > G

chr17:42876659:T:C:UTR3:GJC1	chr17	42876659	42876659	T	C	UTR3	GJC1	ENST000005	.	.	A	G	-	-	0.075	37,0,3,0	chr17,42876	Cytosol	A > G
chr17:42876711:T:C:UTR3:GJC1	chr17	42876711	42876711	T	C	UTR3	GJC1	ENST000005	.	.	A	G	-	-	0.20930233	34,0,9,0	chr17,42876	Cytosol	A > G
chr17:42876712:T:C:UTR3:GJC1	chr17	42876712	42876712	T	C	UTR3	GJC1	ENST000005	.	.	A	G	-	-	0.11111111	40,0,5,0	chr17,42876	Cytosol	A > G
chr17:42876718:T:C:UTR3:GJC1	chr17	42876718	42876718	T	C	UTR3	GJC1	ENST000005	.	.	A	G	-	-	0.05128205	37,0,2,0	chr17,42876	Cytosol	A > G
chr17:42876738:T:C:UTR3:GJC1	chr17	42876738	42876738	T	C	UTR3	GJC1	ENST000005	.	.	A	G	-	-	0.09090909	30,0,3,0	chr17,42876	Cytosol	A > G
chr17:42876747:T:C:UTR3:GJC1	chr17	42876747	42876747	T	C	UTR3	GJC1	ENST000005	.	.	A	G	-	-	0.19565217	37,0,9,0	chr17,42876	Cytosol	A > G
chr17:42876753:T:C:UTR3:GJC1	chr17	42876753	42876753	T	C	UTR3	GJC1	ENST000005	.	.	A	G	-	-	0.13513514	32,0,5,0	chr17,42876	Cytosol	A > G
chr17:42876770:T:C:UTR3:GJC1	chr17	42876770	42876770	T	C	UTR3	GJC1	ENST000005	.	.	A	G	-	-	0.18421053	31,0,7,0	chr17,42876	Cytosol	A > G
chr17:42876777:T:C:UTR3:GJC1	chr17	42876777	42876777	T	C	UTR3	GJC1	ENST000005	.	.	A	G	-	-	0.11764706	30,0,4,0	chr17,42876	Cytosol	A > G
chr17:42876791:T:C:UTR3:GJC1	chr17	42876791	42876791	T	C	UTR3	GJC1	ENST000005	.	.	A	G	-	-	0.575	17,0,23,0	chr17,42876	Cytosol	A > G
chr17:42876808:T:C:UTR3:GJC1	chr17	42876808	42876808	T	C	UTR3	GJC1	ENST000005	.	.	A	G	-	-	0.20833333	19,0,5,0	chr17,42876	Cytosol	A > G
chr17:42876841:T:C:UTR3:GJC1	chr17	42876841	42876841	T	C	UTR3	GJC1	ENST000005	.	.	A	G	-	-	0.125	28,0,4,0	chr17,42876	Cytosol	A > G
chr17:42876846:T:C:UTR3:GJC1	chr17	42876846	42876846	T	C	UTR3	GJC1	ENST000005	.	.	A	G	-	-	0.21212121	26,0,7,0	chr17,42876	Cytosol	A > G
chr17:42876847:T:C:UTR3:GJC1	chr17	42876847	42876847	T	C	UTR3	GJC1	ENST000005	.	.	A	G	-	-	0.17142857	29,0,6,0	chr17,42876	Cytosol	A > G
chr17:42877044:T:C:UTR3:GJC1	chr17	42877044	42877044	T	C	UTR3	GJC1	ENST000005	.	.	A	G	-	-	0.13114754	53,0,8,0	chr17,42877	Cytosol	A > G
chr17:42877065:T:C:UTR3:GJC1	chr17	42877065	42877065	T	C	UTR3	GJC1	ENST000005	.	.	A	G	-	-	0.13114754	53,0,8,0	chr17,42877	Cytosol	A > G
chr17:42877107:T:C:UTR3:GJC1	chr17	42877107	42877107	T	C	UTR3	GJC1	ENST000005	.	.	A	G	-	-	0.10666667	67,0,8,0	chr17,42877	Cytosol	A > G
chr17:42877116:T:C:UTR3:GJC1	chr17	42877116	42877116	T	C	UTR3	GJC1	ENST000005	.	.	A	G	-	-	0.32857143	47,0,23,0	chr17,42877	Cytosol	A > G
chr17:42877139:T:C:UTR3:GJC1	chr17	42877139	42877139	T	C	UTR3	GJC1	ENST000005	.	.	A	G	-	-	0.07936508	58,0,5,0	chr17,42877	Cytosol	A > G
chr17:42877140:T:C:UTR3:GJC1	chr17	42877140	42877140	T	C	UTR3	GJC1	ENST000005	.	.	A	G	-	-	0.14754098	52,0,9,0	chr17,42877	Cytosol	A > G
chr17:42877148:T:C:UTR3:GJC1	chr17	42877148	42877148	T	C	UTR3	GJC1	ENST000005	.	.	A	G	-	-	0.35714286	36,0,20,0	chr17,42877	Cytosol	A > G
chr17:42877163:T:C:UTR3:GJC1	chr17	42877163	42877163	T	C	UTR3	GJC1	ENST000005	.	.	A	G	-	-	0.61333333	29,0,46,0	chr17,42877	Cytosol	A > G
chr17:42877178:T:C:UTR3:GJC1	chr17	42877178	42877178	T	C	UTR3	GJC1	ENST000005	.	.	A	G	-	-	0.13513514	64,0,10,0	chr17,42877	Cytosol	A > G
chr17:42877187:T:C:UTR3:GJC1	chr17	42877187	42877187	T	C	UTR3	GJC1	ENST000005	.	.	A	G	-	-	0.02564103	76,0,2,0	chr17,42877	Cytosol	A > G
chr17:42877204:T:C:UTR3:GJC1	chr17	42877204	42877204	T	C	UTR3	GJC1	ENST000005	.	.	A	G	-	-	0.41860465	50,0,36,0	chr17,42877	Cytosol	A > G
chr17:42877227:T:C:UTR3:GJC1	chr17	42877227	42877227	T	C	UTR3	GJC1	ENST000005	.	.	A	G	-	-	0.33333333	46,0,23,0	chr17,42877	Cytosol	A > G
chr17:42877230:T:C:UTR3:GJC1	chr17	42877230	42877230	T	C	UTR3	GJC1	ENST000005	.	.	A	G	-	-	0.11764706	60,0,8,0	chr17,42877	Cytosol	A > G
chr17:42877234:T:C:UTR3:GJC1	chr17	42877234	42877234	T	C	UTR3	GJC1	ENST000005	.	.	A	G	-	-	0.06097561	77,0,5,0	chr17,42877	Cytosol	A > G
chr17:42877235:T:C:UTR3:GJC1	chr17	42877235	42877235	T	C	UTR3	GJC1	ENST000005	.	.	A	G	-	-	0.27380952	61,0,23,0	chr17,42877	Cytosol	A > G
chr17:42877240:T:C:UTR3:GJC1	chr17	42877240	42877240	T	C	UTR3	GJC1	ENST000005	.	.	A	G	-	-	0.0326087	89,0,3,0	chr17,42877	Cytosol	A > G
chr17:42877256:T:C:UTR3:GJC1	chr17	42877256	42877256	T	C	UTR3	GJC1	ENST000005	.	.	A	G	-	-	0.62068966	33,0,54,0	chr17,42877	Cytosol	A > G
chr17:42877581:T:C:UTR3:GJC1	chr17	42877581	42877581	T	C	UTR3	GJC1	ENST000005	.	.	A	G	-	-	0.15625	27,0,5,0	chr17,42877	Cytosol	A > G
chr17:42877584:T:C:UTR3:GJC1	chr17	42877584	42877584	T	C	UTR3	GJC1	ENST000005	.	.	A	G	-	-	0.10714286	25,0,3,0	chr17,42877	Cytosol	A > G
chr17:42877601:T:C:UTR3:GJC1	chr17	42877601	42877601	T	C	UTR3	GJC1	ENST000005	.	.	A	G	-	-	0.125	28,0,4,0	chr17,42877	Cytosol	A > G
chr17:42877614:T:C:UTR3:GJC1	chr17	42877614	42877614	T	C	UTR3	GJC1	ENST000005	.	.	A	G	-	-	0.23529412	26,0,8,0	chr17,42877	Cytosol	A > G
chr17:42877615:T:C:UTR3:GJC1	chr17	42877615	42877615	T	C	UTR3	GJC1	ENST000005	.	.	A	G	-	-	0.09375	29,0,3,0	chr17,42877	Cytosol	A > G
chr17:42877621:T:C:UTR3:GJC1	chr17	42877621	42877621	T	C	UTR3	GJC1	ENST000005	.	.	A	G	-	-	0.60714286	11,0,17,0	chr17,42877	Cytosol	A > G
chr17:42877641:T:C:UTR3:GJC1	chr17	42877641	42877641	T	C	UTR3	GJC1	ENST000005	.	.	A	G	-	-	0.18181818	18,0,4,0	chr17,42877	Cytosol	A > G
chr17:42877644:T:C:UTR3:GJC1	chr17	42877644	42877644	T	C	UTR3	GJC1	ENST000005	.	.	A	G	-	-	0.13793103	25,0,4,0	chr17,42877	Cytosol	A > G
chr17:42877671:T:C:UTR3:GJC1	chr17	42877671	42877671	T	C	UTR3	GJC1	ENST000005	.	.	A	G	-	-	0.21621622	29,0,8,0	chr17,42877	Cytosol	A > G
chr17:42877713:T:C:UTR3:GJC1	chr17	42877713	42877713	T	C	UTR3	GJC1	ENST000005	.	.	A	G	-	-	0.06666667	28,0,2,0	chr17,42877	Cytosol	A > G
chr17:42877726:T:C:UTR3:GJC1	chr17	42877726	42877726	T	C	UTR3	GJC1	ENST000005	.	.	A	G	-	-	0.59259259	11,0,16,0	chr17,42877	Cytosol	A > G
chr17:42878387:T:C:UTR3:GJC1	chr17	42878387	42878387	T	C	UTR3	GJC1	ENST000004	.	.	A	G	-	-	0.33333333	18,0,9,0	chr17,42878	Cytosol	A > G
chr17:42878399:T:C:UTR3:GJC1	chr17	42878399	42878399	T	C	UTR3	GJC1	ENST000004	.	.	A	G	-	-	0.41666667	14,0,10,0	chr17,42878	Cytosol	A > G
chr17:42878400:T:C:UTR3:GJC1	chr17	42878400	42878400	T	C	UTR3	GJC1	ENST000004	.	.	A	G	-	-	0.66666667	9,0,18,0	chr17,42878	Cytosol	A > G
chr17:42878401:T:C:UTR3:GJC1	chr17	42878401	42878401	T	C	UTR3	GJC1	ENST000004	.	.	A	G	-	-	0.22222222	21,0,6,0	chr17,42878	Cytosol	A > G
chr17:42878486:T:C:UTR3:GJC1	chr17	42878486	42878486	T	C	UTR3	GJC1	ENST000004	.	.	A	G	-	-	0.61538462	5,0,8,0	chr17,42878	Cytosol	A > G
chr17:42878487:T:C:UTR3:GJC1	chr17	42878487	42878487	T	C	UTR3	GJC1	ENST000004	.	.	A	G	-	-	0.91666667	1,0,11,0	chr17,42878	Cytosol	A > G
chr17:42878528:T:C:UTR3:GJC1	chr17	42878528	42878528	T	C	UTR3	GJC1	ENST000004	.	.	A	G	-	-	0.28571429	5,0,2,0	chr17,42878	Cytosol	A > G
chr17:42878530:T:C:UTR3:GJC1	chr17	42878530	42878530	T	C	UTR3	GJC1	ENST000004	.	.	A	G	-	-	0.33333333	4,0,2,0	chr17,42878	Cytosol	A > G
chr17:42878540:T:C:UTR3:GJC1	chr17	42878540	42878540	T	C	UTR3	GJC1	ENST000004	.	.	A	G	-	-	0.44444444	5,0,4,0	chr17,42878	Cytosol	A > G
chr17:42878652:T:C:UTR3:GJC1	chr17	42878652	42878652	T	C	UTR3	GJC1	ENST000004	.	.	A	G	-	-	0.21052632	15,0,4,0	chr17,42878	Cytosol	A > G
chr17:42879068:T:C:UTR3:GJC1	chr17	42879068	42879068	T	C	UTR3	GJC1	ENST000004	.	.	A	G	-	-	0.33333333	8,0,4,0	chr17,42879	Cytosol	A > G
chr17:42879109:T:C:UTR3:GJC1	chr17	42879109	42879109	T	C	UTR3	GJC1	ENST000004	.	.	A	G	-	-	0.42857143	4,0,3,0	chr17,42879	Cytosol	A > G
chr17:42879177:T:C:UTR3:GJC1	chr17	42879177	42879177	T	C	UTR3	GJC1	ENST000004	.	.	A	G	-	-	0.25	12,0,4,0	chr17,42879	Cytosol	A > G
chr17:42879192:T:C:UTR3:GJC1	chr17	42879192	42879192	T	C	UTR3	GJC1	ENST000004	.	.	A	G	-	-	0.58333333	5,0,7,0	chr17,42879	Cytosol	A > G
chr17:42879232:T:C:UTR3:GJC1	chr17	42879232	42879232	T	C	UTR3	GJC1	ENST000004	.	.	A	G	-	-	0.69230769	4,0,9,0	chr17,42879	Cytosol	A > G
chr17:42879252:T:C:UTR3:GJC1	chr17	42879252	42879252	T	C	UTR3	GJC1	ENST000004	.	.	A	G	-	-	0.13636364	19,0,3,0	chr17,42879	Cytosol	A > G
chr17:42879268:T:C:UTR3:GJC1	chr17	42879268	42879268	T	C	UTR3	GJC1	ENST000004	.	.	A	G	-	-	0.16	21,0,4,0	chr17,42879	Cytosol	A > G
chr17:42880112:T:C:UTR3:GJC1	chr17	42880112	42880112	T	C	UTR3	GJC1	ENST000004	.	.	A	G	-	-	0.5	25,0,25,0	chr17,42880	Cytosol	A > G
chr17:42880118:T:C:UTR3:GJC1	chr17	42880118	42880118	T	C	UTR3	GJC1	ENST000004	.	.	A	G	-	-	0.25490196	38,0,13,0	chr17,42880	Cytosol	A > G
chr17:42880145:T:C:UTR3:GJC1	chr17	42880145	42880145	T	C	UTR3	GJC1	ENST000004	.	.	A	G	-	-	0.375	35,0,21,0	chr17,42880	Cytosol	A > G
chr17:42880146:T:C:UTR3:GJC1	chr17	42880146	42880146	T	C	UTR3	GJC1	ENST000004	.	.	A	G	-	-	0.16363636	46,0,9,0	chr17,42880	Cytosol	A > G
chr17:42880175:T:C:UTR3:GJC1	chr17	42880175	42880175	T	C	UTR3	GJC1	ENST000004	.	.	A	G	-	-	0.26666667	33,0,12,0	chr17,42880	Cytosol	A > G

chr17:42880190:T:C:UTR3:GJC1	chr17	42880190	42880190	T	C	UTR3	GJC1	ENST000004	.	.	A	G	-	-	0.06060606	31,0,2,0	chr17,42880	Cytosol	A > G
chr17:42880249:T:C:UTR3:GJC1	chr17	42880249	42880249	T	C	UTR3	GJC1	ENST000004	.	.	A	G	-	-	0.57142857	9,0,12,0	chr17,42880	Cytosol	A > G
chr17:42880259:T:C:UTR3:GJC1	chr17	42880259	42880259	T	C	UTR3	GJC1	ENST000004	.	.	A	G	-	-	0.07407407	25,0,2,0	chr17,42880	Cytosol	A > G
chr17:42880271:T:C:UTR3:GJC1	chr17	42880271	42880271	T	C	UTR3	GJC1	ENST000004	.	.	A	G	-	-	0.2	20,0,5,0	chr17,42880	Cytosol	A > G
chr17:42880295:T:C:UTR3:GJC1	chr17	42880295	42880295	T	C	UTR3	GJC1	ENST000004	.	.	A	G	-	-	0.27272727	16,0,6,0	chr17,42880	Cytosol	A > G
chr17:42880309:T:C:UTR3:GJC1	chr17	42880309	42880309	T	C	UTR3	GJC1	ENST000004	.	.	A	G	-	-	0.5	8,0,8,0	chr17,42880	Cytosol	A > G
chr17:42880314:T:C:UTR3:GJC1	chr17	42880314	42880314	T	C	UTR3	GJC1	ENST000004	.	.	A	G	-	-	0.55555556	8,0,10,0	chr17,42880	Cytosol	A > G
chr17:42880319:T:C:UTR3:GJC1	chr17	42880319	42880319	T	C	UTR3	GJC1	ENST000004	.	.	A	G	-	-	0.42105263	11,0,8,0	chr17,42880	Cytosol	A > G
chr17:43219992:A:G:UTR3:ACBD4	chr17	43219992	43219992	A	G	UTR3	ACBD4	ENST000003	.	.	A	G	+	+	0.85714286	1,0,6,0	chr17,43219	Cytosol	A > G
chr17:43220062:A:G:UTR3:ACBD4	chr17	43220062	43220062	A	G	UTR3	ACBD4	ENST000003	.	.	A	G	+	+	0.5	3,0,3,0	chr17,43220	Cytosol	A > G
chr17:43220064:A:G:UTR3:ACBD4	chr17	43220064	43220064	A	G	UTR3	ACBD4	ENST000003	.	.	A	G	+	+	0.28571429	5,0,2,0	chr17,43220	Cytosol	A > G
chr17:43220072:A:G:UTR3:ACBD4	chr17	43220072	43220072	A	G	UTR3	ACBD4	ENST000003	.	.	A	G	+	+	0.33333333	4,0,2,0	chr17,43220	Cytosol	A > G
chr17:43220185:A:G:UTR3:ACBD4	chr17	43220185	43220185	A	G	UTR3	ACBD4	ENST000003	.	.	A	G	+	+	0.42857143	4,0,3,0	chr17,43220	Cytosol	A > G
chr17:43294659:T:C:ncRNA_intro	chr17	43294659	43294659	T	C	ncRNA_intro	CTD-2020K1	.	.	.	A	G	-	-	0.75	2,0,6,0	chr17,43294	Cytosol	A > G
chr17:43294719:T:C:ncRNA_intro	chr17	43294719	43294719	T	C	ncRNA_intro	CTD-2020K1	.	.	.	A	G	-	-	0.4	3,0,2,0	chr17,43294	Cytosol	A > G
chr17:43294997:T:C:ncRNA_intro	chr17	43294997	43294997	T	C	ncRNA_intro	CTD-2020K1	.	.	.	A	G	-	-	0.25	15,0,5,0	chr17,43294	Cytosol	A > G
chr17:43294998:T:C:ncRNA_intro	chr17	43294998	43294998	T	C	ncRNA_intro	CTD-2020K1	.	.	.	A	G	-	-	0.19047619	17,0,4,0	chr17,43294	Cytosol	A > G
chr17:43295032:T:C:ncRNA_intro	chr17	43295032	43295032	T	C	ncRNA_intro	CTD-2020K1	.	.	.	A	G	-	-	0.35714286	9,0,5,0	chr17,43295	Cytosol	A > G
chr17:43295067:T:C:ncRNA_intro	chr17	43295067	43295067	T	C	ncRNA_intro	CTD-2020K1	.	.	.	A	G	-	-	0.27272727	8,0,3,0	chr17,43295	Cytosol	A > G
chr17:43295172:T:C:ncRNA_intro	chr17	43295172	43295172	T	C	ncRNA_intro	CTD-2020K1	.	.	.	A	G	-	-	0.33333333	10,0,5,0	chr17,43295	Cytosol	A > G
chr17:43295372:T:C:ncRNA_intro	chr17	43295372	43295372	T	C	ncRNA_intro	CTD-2020K1	.	.	.	A	G	-	-	0.14285714	12,0,2,0	chr17,43295	Cytosol	A > G
chr17:43295442:T:C:ncRNA_intro	chr17	43295442	43295442	T	C	ncRNA_intro	CTD-2020K1	.	.	.	A	G	-	-	0.4	3,0,2,0	chr17,43295	Cytosol	A > G
chr17:43295757:T:C:ncRNA_exoni	chr17	43295757	43295757	T	C	ncRNA_exon	CTD-2020K1	.	.	.	A	G	-	-	0.375	5,0,3,0	chr17,43295	Cytosol	A > G
chr17:43295766:T:C:ncRNA_exoni	chr17	43295766	43295766	T	C	ncRNA_exon	CTD-2020K1	.	.	.	A	G	-	-	0.5	3,0,3,0	chr17,43295	Cytosol	A > G
chr17:43295853:T:C:ncRNA_exoni	chr17	43295853	43295853	T	C	ncRNA_exon	CTD-2020K1	.	.	.	A	G	-	-	0.27272727	8,0,3,0	chr17,43295	Cytosol	A > G
chr17:43583007:T:C:ncRNA_intro	chr17	43583007	43583007	T	C	ncRNA_intro	RP11-798G7	.	.	.	A	G	-	-+	0.5	3,0,3,0	chr17,43583	Cytosol	A > G
chr17:43583070:T:C:ncRNA_intro	chr17	43583070	43583070	T	C	ncRNA_intro	RP11-798G7	.	.	.	A	G	-	-+	0.28571429	5,0,2,0	chr17,43583	Cytosol	A > G
chr17:43583373:T:C:ncRNA_intro	chr17	43583373	43583373	T	C	ncRNA_intro	RP11-798G7	.	.	.	A	G	-	+-	0.6	12,0,18,0	chr17,43583	Cytosol	A > G
chr17:45019172:A:G:intronic:GOS	chr17	45019172	45019172	A	G	intronic	GOSR2,RP11	.	.	.	A	G	+	+	0.02040816	96,0,2,0	chr17,45019	Cytosol	A > G
chr17:45019187:A:G:intronic:GOS	chr17	45019187	45019187	A	G	intronic	GOSR2,RP11	.	.	.	A	G	+	+	0.03296703	88,0,3,0	chr17,45019	Cytosol	A > G
chr17:46025078:A:G:ncRNA_intro	chr17	46025078	46025078	A	G	ncRNA_intro	RP11-6N17.6	.	.	.	A	G	+	+-	0.03937008	122,0,5,0	chr17,46025	Cytosol	A > G
chr17:46941503:A:G:UTR3:CALCO	chr17	46941503	46941503	A	G	UTR3	CALCOCO2	ENST000002	.	.	A	G	+	+	0.13043478	20,0,3,0	chr17,46941	Cytosol	A > G
chr17:46941519:A:G:UTR3:CALCO	chr17	46941519	46941519	A	G	UTR3	CALCOCO2	ENST000002	.	.	A	G	+	+	0.0952381	19,0,2,0	chr17,46941	Cytosol	A > G
chr17:46941526:A:G:UTR3:CALCO	chr17	46941526	46941526	A	G	UTR3	CALCOCO2	ENST000002	.	.	A	G	+	+	0.11764706	15,0,2,0	chr17,46941	Cytosol	A > G
chr17:46941551:A:G:UTR3:CALCO	chr17	46941551	46941551	A	G	UTR3	CALCOCO2	ENST000002	.	.	A	G	+	+	0.18181818	18,0,4,0	chr17,46941	Cytosol	A > G
chr17:46941609:A:G:UTR3:CALCO	chr17	46941609	46941609	A	G	UTR3	CALCOCO2	ENST000002	.	.	A	G	+	+	0.15	17,0,3,0	chr17,46941	Cytosol	A > G
chr17:46941615:A:G:UTR3:CALCO	chr17	46941615	46941615	A	G	UTR3	CALCOCO2	ENST000002	.	.	A	G	+	+	0.375	10,0,6,0	chr17,46941	Cytosol	A > G
chr17:46941648:A:G:UTR3:CALCO	chr17	46941648	46941648	A	G	UTR3	CALCOCO2	ENST000002	.	.	A	G	+	+	0.19047619	17,0,4,0	chr17,46941	Cytosol	A > G
chr17:46941678:A:G:UTR3:CALCO	chr17	46941678	46941678	A	G	UTR3	CALCOCO2	ENST000002	.	.	A	G	+	+	0.14814815	23,0,4,0	chr17,46941	Cytosol	A > G
chr17:46943469:A:G:downstream:	chr17	46943469	46943469	A	G	downstream	CALCOCO2	.	.	.	A	G	+	+	0.6	2,0,3,0	chr17,46943	Cytosol	A > G
chr17:47374028:T:C:UTR3:ZNF652	chr17	47374028	47374028	T	C	UTR3	ZNF652	ENST000003	.	.	A	G	-	-	0.2	16,0,4,0	chr17,47374	Cytosol	A > G
chr17:47374030:T:C:UTR3:ZNF652	chr17	47374030	47374030	T	C	UTR3	ZNF652	ENST000003	.	.	A	G	-	-	0.2173913	18,0,5,0	chr17,47374	Cytosol	A > G
chr17:47374102:T:C:UTR3:ZNF652	chr17	47374102	47374102	T	C	UTR3	ZNF652	ENST000003	.	.	A	G	-	-	0.33333333	13,0,7,1	chr17,47374	Cytosol	A > G
chr17:49041692:T:C:UTR3:SPAG9	chr17	49041692	49041692	T	C	UTR3	SPAG9	ENST000002	.	.	A	G	-	-	0.15384615	22,0,4,0	chr17,49041	Cytosol	A > G
chr17:49041732:T:C:UTR3:SPAG9	chr17	49041732	49041732	T	C	UTR3	SPAG9	ENST000002	.	.	A	G	-	-	0.14285714	12,0,2,0	chr17,49041	Cytosol	A > G
chr17:49041764:T:C:UTR3:SPAG9	chr17	49041764	49041764	T	C	UTR3	SPAG9	ENST000002	.	.	A	G	-	-	0.71428571	4,0,10,0	chr17,49041	Cytosol	A > G
chr17:49041769:T:C:UTR3:SPAG9	chr17	49041769	49041769	T	C	UTR3	SPAG9	ENST000002	.	.	A	G	-	-	0.57142857	6,0,8,0	chr17,49041	Cytosol	A > G
chr17:49041827:T:C:UTR3:SPAG9	chr17	49041827	49041827	T	C	UTR3	SPAG9	ENST000002	.	.	A	G	-	-	0.44444444	5,0,4,0	chr17,49041	Cytosol	A > G
chr17:49041837:T:C:UTR3:SPAG9	chr17	49041837	49041837	T	C	UTR3	SPAG9	ENST000002	.	.	A	G	-	-	0.83333333	1,0,5,0	chr17,49041	Cytosol	A > G
chr17:49041840:T:C:UTR3:SPAG9	chr17	49041840	49041840	T	C	UTR3	SPAG9	ENST000002	.	.	A	G	-	-	0.66666667	2,0,4,0	chr17,49041	Cytosol	A > G
chr17:49041866:T:C:UTR3:SPAG9	chr17	49041866	49041866	T	C	UTR3	SPAG9	ENST000002	.	.	A	G	-	-	0.83333333	1,0,5,0	chr17,49041	Cytosol	A > G
chr17:49041897:T:C:UTR3:SPAG9	chr17	49041897	49041897	T	C	UTR3	SPAG9	ENST000002	.	.	A	G	-	-	0.35714286	9,0,5,0	chr17,49041	Cytosol	A > G
chr17:49042216:T:C:UTR3:SPAG9	chr17	49042216	49042216	T	C	UTR3	SPAG9	ENST000002	.	.	A	G	-	-	0.35714286	9,0,5,0	chr17,49042	Cytosol	A > G
chr17:49042242:T:C:UTR3:SPAG9	chr17	49042242	49042242	T	C	UTR3	SPAG9	ENST000002	.	.	A	G	-	-	0.1875	13,0,3,0	chr17,49042	Cytosol	A > G
chr17:49042246:T:C:UTR3:SPAG9	chr17	49042246	49042246	T	C	UTR3	SPAG9	ENST000002	.	.	A	G	-	-	0.25	12,0,4,0	chr17,49042	Cytosol	A > G
chr17:49042252:T:C:UTR3:SPAG9	chr17	49042252	49042252	T	C	UTR3	SPAG9	ENST000002	.	.	A	G	-	-	0.84615385	2,0,11,0	chr17,49042	Cytosol	A > G
chr17:49042277:T:C:UTR3:SPAG9	chr17	49042277	49042277	T	C	UTR3	SPAG9	ENST000002	.	.	A	G	-	-	0.23076923	10,0,3,0	chr17,49042	Cytosol	A > G
chr17:49042278:T:C:UTR3:SPAG9	chr17	49042278	49042278	T	C	UTR3	SPAG9	ENST000002	.	.	A	G	-	-	0.92307692	1,0,12,0	chr17,49042	Cytosol	A > G
chr17:49042323:T:C:UTR3:SPAG9	chr17	49042323	49042323	T	C	UTR3	SPAG9	ENST000002	.	.	A	G	-	-	0.57142857	6,0,8,0	chr17,49042	Cytosol	A > G
chr17:49042341:T:C:UTR3:SPAG9	chr17	49042341	49042341	T	C	UTR3	SPAG9	ENST000002	.	.	A	G	-	-	0.33333333	4,0,2,0	chr17,49042	Cytosol	A > G
chr17:49042368:T:C:UTR3:SPAG9	chr17	49042368	49042368	T	C	UTR3	SPAG9	ENST000002	.	.	A	G	-	-	0.33333333	6,0,3,0	chr17,49042	Cytosol	A > G
chr17:49042388:T:C:UTR3:SPAG9	chr17	49042388	49042388	T	C	UTR3	SPAG9	ENST000002	.	.	A	G	-	-	0.48	13,0,12,0	chr17,49042	Cytosol	A > G
chr17:49042394:T:C:UTR3:SPAG9	chr17	49042394	49042394	T	C	UTR3	SPAG9	ENST000002	.	.	A	G	-	-	0.16	21,0,4,0	chr17,49042	Cytosol	A > G
chr17:49042395:T:C:UTR3:SPAG9	chr17	49042395	49042395	T	C	UTR3	SPAG9	ENST000002	.	.	A	G	-	-	0.09375	29,0,3,0	chr17,49042	Cytosol	A > G

chr17:53121931:A:G:UTR3:STXBP4	chr17	53121931	53121931	A	G	UTR3	STXBP4	ENST000004	.	.	A	G	+	+	0.4	3,0,2,0	chr17,53121	Cytosol	A > G
chr17:53121932:A:G:UTR3:STXBP4	chr17	53121932	53121932	A	G	UTR3	STXBP4	ENST000004	.	.	A	G	+	+	1	0,0,5,0	chr17,53121	Cytosol	A > G
chr17:54907515:T:C:intronic:C17o	chr17	54907515	54907515	T	C	intronic	C17orf67	.	.	.	A	G	-	-	0.25	6,0,2,0	chr17,54907	Cytosol	A > G
chr17:56175834:A:G:intergenic:D	chr17	56175834	56175834	A	G	intergenic	DYNLL2,OR4	dist=2937;dis	.	.	A	G	+		0.06666667	28,0,2,0	chr17,56175	Cytosol	A > G
chr17:56177488:A:G:intergenic:D	chr17	56177488	56177488	A	G	intergenic	DYNLL2,OR4	dist=4591;dis	.	.	A	G	+		0.16666667	10,0,2,0	chr17,56177	Cytosol	A > G
chr17:56177513:A:G:intergenic:D	chr17	56177513	56177513	A	G	intergenic	DYNLL2,OR4	dist=4616;dis	.	.	A	G	+		0.16666667	10,0,2,0	chr17,56177	Cytosol	A > G
chr17:56177514:A:G:intergenic:D	chr17	56177514	56177514	A	G	intergenic	DYNLL2,OR4	dist=4617;dis	.	.	A	G	+		0.15384615	11,0,2,0	chr17,56177	Cytosol	A > G
chr17:57279068:A:G:UTR3:PRR11	chr17	57279068	57279068	A	G	UTR3	PRR11	ENST000002	.	.	A	G	+	+	0.23333333	46,0,14,0	chr17,57279	Cytosol	A > G
chr17:57279070:A:G:UTR3:PRR11	chr17	57279070	57279070	A	G	UTR3	PRR11	ENST000002	.	.	A	G	+	+	0.50819672	30,0,31,0	chr17,57279	Cytosol	A > G
chr17:57279076:A:G:UTR3:PRR11	chr17	57279076	57279076	A	G	UTR3	PRR11	ENST000002	.	.	A	G	+	+	0.13888889	62,0,10,0	chr17,57279	Cytosol	A > G
chr17:57279077:A:G:UTR3:PRR11	chr17	57279077	57279077	A	G	UTR3	PRR11	ENST000002	.	.	A	G	+	+	0.14705882	58,0,10,0	chr17,57279	Cytosol	A > G
chr17:57279112:A:G:UTR3:PRR11	chr17	57279112	57279112	A	G	UTR3	PRR11	ENST000002	.	.	A	G	+	+	0.02197802	89,0,2,0	chr17,57279	Cytosol	A > G
chr17:57279156:A:G:UTR3:PRR11	chr17	57279156	57279156	A	G	UTR3	PRR11	ENST000002	.	.	A	G	+	+	0.03703704	104,0,4,0	chr17,57279	Cytosol	A > G
chr17:57279167:A:G:UTR3:PRR11	chr17	57279167	57279167	A	G	UTR3	PRR11	ENST000002	.	.	A	G	+	+	0.30252101	83,0,36,0	chr17,57279	Cytosol	A > G
chr17:57279170:A:G:UTR3:PRR11	chr17	57279170	57279170	A	G	UTR3	PRR11	ENST000002	.	.	A	G	+	+	0.1440678	100,1,17,0	chr17,57279	Cytosol	A > G
chr17:57279171:A:G:UTR3:PRR11	chr17	57279171	57279171	A	G	UTR3	PRR11	ENST000002	.	.	A	G	+	+	0.18699187	100,0,23,0	chr17,57279	Cytosol	A > G
chr17:57279185:A:G:UTR3:PRR11	chr17	57279185	57279185	A	G	UTR3	PRR11	ENST000002	.	.	A	G	+	+	0.15909091	111,0,21,0	chr17,57279	Cytosol	A > G
chr17:57279195:A:G:UTR3:PRR11	chr17	57279195	57279195	A	G	UTR3	PRR11	ENST000002	.	.	A	G	+	+	0.02	147,0,3,0	chr17,57279	Cytosol	A > G
chr17:57279211:A:G:UTR3:PRR11	chr17	57279211	57279211	A	G	UTR3	PRR11	ENST000002	.	.	A	G	+	+	0.01438849	137,0,2,0	chr17,57279	Cytosol	A > G
chr17:57279233:A:G:UTR3:PRR11	chr17	57279233	57279233	A	G	UTR3	PRR11	ENST000002	.	.	A	G	+	+	0.46153846	56,0,48,0	chr17,57279	Cytosol	A > G
chr17:57279234:A:G:UTR3:PRR11	chr17	57279234	57279234	A	G	UTR3	PRR11	ENST000002	.	.	A	G	+	+	0.15238095	89,0,16,0	chr17,57279	Cytosol	A > G
chr17:57279244:A:G:UTR3:PRR11	chr17	57279244	57279244	A	G	UTR3	PRR11	ENST000002	.	.	A	G	+	+	0.02857143	102,0,3,0	chr17,57279	Cytosol	A > G
chr17:57279252:A:G:UTR3:PRR11	chr17	57279252	57279252	A	G	UTR3	PRR11	ENST000002	.	.	A	G	+	+	0.01834862	107,0,2,0	chr17,57279	Cytosol	A > G
chr17:57279253:A:G:UTR3:PRR11	chr17	57279253	57279253	A	G	UTR3	PRR11	ENST000002	.	.	A	G	+	+	0.09174312	99,0,10,0	chr17,57279	Cytosol	A > G
chr17:57279257:A:G:UTR3:PRR11	chr17	57279257	57279257	A	G	UTR3	PRR11	ENST000002	.	.	A	G	+	+	0.19834711	97,0,24,0	chr17,57279	Cytosol	A > G
chr17:57279258:A:G:UTR3:PRR11	chr17	57279258	57279258	A	G	UTR3	PRR11	ENST000002	.	.	A	G	+	+	0.25641026	87,0,30,0	chr17,57279	Cytosol	A > G
chr17:57279264:A:G:UTR3:PRR11	chr17	57279264	57279264	A	G	UTR3	PRR11	ENST000002	.	.	A	G	+	+	0.89430894	13,0,110,0	chr17,57279	Cytosol	A > G
chr17:57279273:A:G:UTR3:PRR11	chr17	57279273	57279273	A	G	UTR3	PRR11	ENST000002	.	.	A	G	+	+	0.16339869	128,0,25,0	chr17,57279	Cytosol	A > G
chr17:57279281:A:G:UTR3:PRR11	chr17	57279281	57279281	A	G	UTR3	PRR11	ENST000002	.	.	A	G	+	+	0.54248366	70,0,83,0	chr17,57279	Cytosol	A > G
chr17:57279282:A:G:UTR3:PRR11	chr17	57279282	57279282	A	G	UTR3	PRR11	ENST000002	.	.	A	G	+	+	0.78612717	36,1,136,0	chr17,57279	Cytosol	A > G
chr17:57279284:A:G:UTR3:PRR11	chr17	57279284	57279284	A	G	UTR3	PRR11	ENST000002	.	.	A	G	+	+	0.48275862	105,0,98,0	chr17,57279	Cytosol	A > G
chr17:57279311:A:G:UTR3:PRR11	chr17	57279311	57279311	A	G	UTR3	PRR11	ENST000002	.	.	A	G	+	+	0.01030928	288,0,3,0	chr17,57279	Cytosol	A > G
chr17:57279323:A:G:UTR3:PRR11	chr17	57279323	57279323	A	G	UTR3	PRR11	ENST000002	.	.	A	G	+	+	0.01935484	304,0,6,0	chr17,57279	Cytosol	A > G
chr17:57279716:A:G:UTR3:PRR11	chr17	57279716	57279716	A	G	UTR3	PRR11	ENST000002	.	.	A	G	+	+	0.0106383	186,0,2,0	chr17,57279	Cytosol	A > G
chr17:57279806:A:G:UTR3:PRR11	chr17	57279806	57279806	A	G	UTR3	PRR11	ENST000002	.	.	A	G	+	+	0.01546392	191,0,3,0	chr17,57279	Cytosol	A > G
chr17:57279824:A:G:UTR3:PRR11	chr17	57279824	57279824	A	G	UTR3	PRR11	ENST000002	.	.	A	G	+	+	0.02403846	203,0,5,0	chr17,57279	Cytosol	A > G
chr17:57280292:A:G:ncRNA_intro	chr17	57280292	57280292	A	G	ncRNA_intro	CTD-2510F5.	.	.	.	A	G	+	+-	0.02419355	242,0,6,0	chr17,57280	Cytosol	A > G
chr17:57280605:A:G:ncRNA_intro	chr17	57280605	57280605	A	G	ncRNA_intro	CTD-2510F5.	.	.	.	A	G	+	+-	0.10619469	101,0,12,0	chr17,57280	Cytosol	A > G
chr17:57280617:A:G:ncRNA_intro	chr17	57280617	57280617	A	G	ncRNA_intro	CTD-2510F5.	.	.	.	A	G	+	+-	0.10869565	123,0,15,0	chr17,57280	Cytosol	A > G
chr17:57280625:A:G:ncRNA_intro	chr17	57280625	57280625	A	G	ncRNA_intro	CTD-2510F5.	.	.	.	A	G	+	+-	0.0733945	101,0,8,0	chr17,57280	Cytosol	A > G
chr17:57280649:A:G:ncRNA_intro	chr17	57280649	57280649	A	G	ncRNA_intro	CTD-2510F5.	.	.	.	A	G	+	+-	0.78181818	24,0,86,0	chr17,57280	Cytosol	A > G
chr17:57280650:A:G:ncRNA_intro	chr17	57280650	57280650	A	G	ncRNA_intro	CTD-2510F5.	.	.	.	A	G	+	+-	0.67857143	36,0,76,0	chr17,57280	Cytosol	A > G
chr17:57280660:A:G:ncRNA_intro	chr17	57280660	57280660	A	G	ncRNA_intro	CTD-2510F5.	.	.	.	A	G	+	+-	0.30909091	76,0,34,0	chr17,57280	Cytosol	A > G
chr17:57280672:A:G:ncRNA_intro	chr17	57280672	57280672	A	G	ncRNA_intro	CTD-2510F5.	.	.	.	A	G	+	+-	0.56756757	48,0,63,0	chr17,57280	Cytosol	A > G
chr17:57280679:A:G:ncRNA_intro	chr17	57280679	57280679	A	G	ncRNA_intro	CTD-2510F5.	.	.	.	A	G	+	+-	0.22680412	75,0,22,0	chr17,57280	Cytosol	A > G
chr17:57280703:A:G:ncRNA_intro	chr17	57280703	57280703	A	G	ncRNA_intro	CTD-2510F5.	.	.	.	A	G	+	+-	0.48780488	42,0,40,0	chr17,57280	Cytosol	A > G
chr17:57280725:A:G:ncRNA_intro	chr17	57280725	57280725	A	G	ncRNA_intro	CTD-2510F5.	.	.	.	A	G	+	+-	0.11940299	118,0,16,0	chr17,57280	Cytosol	A > G
chr17:57280741:A:G:ncRNA_intro	chr17	57280741	57280741	A	G	ncRNA_intro	CTD-2510F5.	.	.	.	A	G	+	+-	0.0661157	113,0,8,0	chr17,57280	Cytosol	A > G
chr17:57280751:A:G:ncRNA_intro	chr17	57280751	57280751	A	G	ncRNA_intro	CTD-2510F5.	.	.	.	A	G	+	+-	0.34126984	83,0,43,0	chr17,57280	Cytosol	A > G
chr17:57280752:A:G:ncRNA_intro	chr17	57280752	57280752	A	G	ncRNA_intro	CTD-2510F5.	.	.	.	A	G	+	+-	0.140625	110,0,18,0	chr17,57280	Cytosol	A > G
chr17:57280760:A:G:ncRNA_intro	chr17	57280760	57280760	A	G	ncRNA_intro	CTD-2510F5.	.	.	.	A	G	+	+-	0.26315789	98,0,35,0	chr17,57280	Cytosol	A > G
chr17:57280766:A:G:ncRNA_intro	chr17	57280766	57280766	A	G	ncRNA_intro	CTD-2510F5.	.	.	.	A	G	+	+-	0.25984252	94,0,33,0	chr17,57280	Cytosol	A > G
chr17:57280769:A:G:ncRNA_intro	chr17	57280769	57280769	A	G	ncRNA_intro	CTD-2510F5.	.	.	.	A	G	+	+-	0.86885246	16,0,106,0	chr17,57280	Cytosol	A > G
chr17:57280770:A:G:ncRNA_intro	chr17	57280770	57280770	A	G	ncRNA_intro	CTD-2510F5.	.	.	.	A	G	+	+-	0.77235772	28,0,95,0	chr17,57280	Cytosol	A > G
chr17:57280771:A:G:ncRNA_intro	chr17	57280771	57280771	A	G	ncRNA_intro	CTD-2510F5.	.	.	.	A	G	+	+-	0.43442623	69,0,53,0	chr17,57280	Cytosol	A > G
chr17:57280795:A:G:ncRNA_intro	chr17	57280795	57280795	A	G	ncRNA_intro	CTD-2510F5.	.	.	.	A	G	+	+-	0.08791209	83,0,8,0	chr17,57280	Cytosol	A > G
chr17:57280820:A:G:splicing:PRR1	chr17	57280820	57280820	A	G	splicing	PRR11	ENST000005	.	.	A	G	+	+-	0.28767123	52,0,21,0	chr17,57280	Cytosol	A > G
chr17:57280838:A:G:ncRNA_intro	chr17	57280838	57280838	A	G	ncRNA_intro	CTD-2510F5.	.	.	.	A	G	+	+-	0.65454545	19,0,36,0	chr17,57280	Cytosol	A > G
chr17:57280846:A:G:ncRNA_intro	chr17	57280846	57280846	A	G	ncRNA_intro	CTD-2510F5.	.	.	.	A	G	+	+-	0.03773585	51,0,2,0	chr17,57280	Cytosol	A > G
chr17:57280847:A:G:ncRNA_intro	chr17	57280847	57280847	A	G	ncRNA_intro	CTD-2510F5.	.	.	.	A	G	+	+-	0.16666667	45,0,9,0	chr17,57280	Cytosol	A > G
chr17:57280848:A:G:ncRNA_intro	chr17	57280848	57280848	A	G	ncRNA_intro	CTD-2510F5.	.	.	.	A	G	+	+-	0.22	39,0,11,0	chr17,57280	Cytosol	A > G
chr17:57280855:A:G:ncRNA_intro	chr17	57280855	57280855	A	G	ncRNA_intro	CTD-2510F5.	.	.	.	A	G	+	+-	0.109375	57,0,7,0	chr17,57280	Cytosol	A > G
chr17:57280860:A:G:ncRNA_intro	chr17	57280860	57280860	A	G	ncRNA_intro	CTD-2510F5.	.	.	.	A	G	+	+-	0.30769231	45,0,20,0	chr17,57280	Cytosol	A > G
chr17:57280965:A:G:ncRNA_intro	chr17	57280965	57280965	A	G	ncRNA_intro	CTD-2510F5.	.	.	.	A	G	+	+-	0.10344828	130,0,15,0	chr17,57280	Cytosol	A > G

chr17:57281010:A:G:ncRNA_intro	chr17	57281010	57281010	A	G	ncRNA_intro	CTD-2510F5.	.	.	.	A	G	+	+-	0.07627119	109,0,9,0	chr17,57281	Cytosol	A > G
chr17:57281021:A:G:ncRNA_intro	chr17	57281021	57281021	A	G	ncRNA_intro	CTD-2510F5.	.	.	.	A	G	+	+-	0.54	46,0,54,0	chr17,57281	Cytosol	A > G
chr17:57281034:A:G:ncRNA_intro	chr17	57281034	57281034	A	G	ncRNA_intro	CTD-2510F5.	.	.	.	A	G	+	+-	0.15789474	80,0,15,0	chr17,57281	Cytosol	A > G
chr17:57281042:A:G:ncRNA_intro	chr17	57281042	57281042	A	G	ncRNA_intro	CTD-2510F5.	.	.	.	A	G	+	+-	0.05982906	110,0,7,0	chr17,57281	Cytosol	A > G
chr17:57281043:A:G:ncRNA_intro	chr17	57281043	57281043	A	G	ncRNA_intro	CTD-2510F5.	.	.	.	A	G	+	+-	0.05454545	104,0,6,0	chr17,57281	Cytosol	A > G
chr17:57281055:A:G:ncRNA_intro	chr17	57281055	57281055	A	G	ncRNA_intro	CTD-2510F5.	.	.	.	A	G	+	+-	0.35353535	64,0,35,0	chr17,57281	Cytosol	A > G
chr17:57281058:A:G:ncRNA_intro	chr17	57281058	57281058	A	G	ncRNA_intro	CTD-2510F5.	.	.	.	A	G	+	+-	0.63917526	35,0,62,0	chr17,57281	Cytosol	A > G
chr17:57281059:A:G:ncRNA_intro	chr17	57281059	57281059	A	G	ncRNA_intro	CTD-2510F5.	.	.	.	A	G	+	+-	0.56435644	44,0,57,0	chr17,57281	Cytosol	A > G
chr17:57281061:A:G:ncRNA_intro	chr17	57281061	57281061	A	G	ncRNA_intro	CTD-2510F5.	.	.	.	A	G	+	+-	0.12149533	94,0,13,0	chr17,57281	Cytosol	A > G
chr17:57281067:A:G:ncRNA_intro	chr17	57281067	57281067	A	G	ncRNA_intro	CTD-2510F5.	.	.	.	A	G	+	+-	0.11340206	86,0,11,0	chr17,57281	Cytosol	A > G
chr17:57281069:A:G:ncRNA_intro	chr17	57281069	57281069	A	G	ncRNA_intro	CTD-2510F5.	.	.	.	A	G	+	+-	0.21568627	80,0,22,0	chr17,57281	Cytosol	A > G
chr17:57281076:A:G:ncRNA_intro	chr17	57281076	57281076	A	G	ncRNA_intro	CTD-2510F5.	.	.	.	A	G	+	+-	0.44329897	54,0,43,0	chr17,57281	Cytosol	A > G
chr17:57281092:A:G:ncRNA_splici	chr17	57281092	57281092	A	G	ncRNA_splici	CTD-2510F5.	ENST000005	.	.	A	G	+	+-	0.03061224	95,0,3,0	chr17,57281	Cytosol	A > G
chr17:57281098:A:G:ncRNA_exon	chr17	57281098	57281098	A	G	ncRNA_exon	CTD-2510F5.	.	.	.	A	G	+	+-	0.03703704	78,0,3,0	chr17,57281	Cytosol	A > G
chr17:57281114:A:G:ncRNA_exon	chr17	57281114	57281114	A	G	ncRNA_exon	CTD-2510F5.	.	.	.	A	G	+	+-	0.10344828	52,0,6,0	chr17,57281	Cytosol	A > G
chr17:57281125:A:G:ncRNA_exon	chr17	57281125	57281125	A	G	ncRNA_exon	CTD-2510F5.	.	.	.	A	G	+	+-	0.34782609	30,0,16,0	chr17,57281	Cytosol	A > G
chr17:57281150:A:G:ncRNA_exon	chr17	57281150	57281150	A	G	ncRNA_exon	CTD-2510F5.	.	.	.	A	G	+	+-	0.0952381	19,0,2,0	chr17,57281	Cytosol	A > G
chr17:57281513:A:G:UTR3:PRR11	chr17	57281513	57281513	A	G	UTR3	PRR11	ENST000005	.	.	A	G	+	+	0.07482993	136,0,11,0	chr17,57281	Cytosol	A > G
chr17:57281636:A:G:UTR3:PRR11	chr17	57281636	57281636	A	G	UTR3	PRR11	ENST000005	.	.	A	G	+	+	0.09134615	189,0,19,0	chr17,57281	Cytosol	A > G
chr17:57281689:A:G:UTR3:PRR11	chr17	57281689	57281689	A	G	UTR3	PRR11	ENST000005	.	.	A	G	+	+	0.07446809	87,0,7,0	chr17,57281	Cytosol	A > G
chr17:57282022:A:G:UTR3:PRR11	chr17	57282022	57282022	A	G	UTR3	PRR11	ENST000005	.	.	A	G	+	+	0.01538462	128,0,2,0	chr17,57282	Cytosol	A > G
chr17:57282132:A:G:intronic:CTD-	chr17	57282132	57282132	A	G	intronic	CTD-2510F5.	.	.	.	A	G	+	+	0.10526316	17,0,2,0	chr17,57282	Cytosol	A > G
chr17:57351504:A:G:UTR3:GDPD1	chr17	57351504	57351504	A	G	UTR3	GDPD1	ENST000002	.	.	A	G	+	+	0.5	4,0,4,0	chr17,57351	Cytosol	A > G
chr17:57351509:A:G:UTR3:GDPD1	chr17	57351509	57351509	A	G	UTR3	GDPD1	ENST000002	.	.	A	G	+	+	0.85714286	1,0,6,0	chr17,57351	Cytosol	A > G
chr17:57351690:A:G:UTR3:GDPD1	chr17	57351690	57351690	A	G	UTR3	GDPD1	ENST000002	.	.	A	G	+	+	0.13333333	13,0,2,0	chr17,57351	Cytosol	A > G
chr17:57351703:A:G:UTR3:GDPD1	chr17	57351703	57351703	A	G	UTR3	GDPD1	ENST000002	.	.	A	G	+	+	0.28571429	10,0,4,0	chr17,57351	Cytosol	A > G
chr17:60526183:A:G:UTR3:METTL	chr17	60526183	60526183	A	G	UTR3	METTL2A	ENST000003	.	.	A	G	+	+	0.02531646	77,0,2,0	chr17,60526	Cytosol	A > G
chr17:60526952:A:G:UTR3:METTL	chr17	60526952	60526952	A	G	UTR3	METTL2A	ENST000003	.	.	A	G	+	+	0.29411765	12,0,5,0	chr17,60526	Cytosol	A > G
chr17:60527232:A:G:UTR3:METTL	chr17	60527232	60527232	A	G	UTR3	METTL2A	ENST000003	.	.	A	G	+	+	0.0952381	19,0,2,0	chr17,60527	Cytosol	A > G
chr17:60527282:A:G:UTR3:METTL	chr17	60527282	60527282	A	G	UTR3	METTL2A	ENST000003	.	.	A	G	+	+	0.11764706	15,0,2,0	chr17,60527	Cytosol	A > G
chr17:60527298:A:G:UTR3:METTL	chr17	60527298	60527298	A	G	UTR3	METTL2A	ENST000003	.	.	A	G	+	+	0.13043478	20,0,3,0	chr17,60527	Cytosol	A > G
chr17:60553892:T:C:intronic:TLK2	chr17	60553892	60553892	T	C	intronic	TLK2	.	.	.	A	G	-	+	0.33333333	6,0,3,0	chr17,60553	Cytosol	A > G
chr17:62485178:T:C:intronic:POLG	chr17	62485178	62485178	T	C	intronic	POLG2	.	.	.	A	G	-	-	0.4	3,0,2,0	chr17,62485	Cytosol	A > G
chr17:62485822:T:C:intronic:POLG	chr17	62485822	62485822	T	C	intronic	POLG2	.	.	.	A	G	-	-	1	0,0,10,0	chr17,62485	Cytosol	A > G
chr17:62485855:T:C:intronic:POLG	chr17	62485855	62485855	T	C	intronic	POLG2	.	.	.	A	G	-	-	0.27272727	8,0,3,0	chr17,62485	Cytosol	A > G
chr17:65334753:T:C:UTR3:PSMD1	chr17	65334753	65334753	T	C	UTR3	PSMD12	ENST000003	.	.	A	G	-	-	0.6	33,0,51,1	chr17,65334	Cytosol	A > G
chr17:65334767:T:C:UTR3:PSMD1	chr17	65334767	65334767	T	C	UTR3	PSMD12	ENST000003	.	.	A	G	-	-	0.70666667	22,0,53,0	chr17,65334	Cytosol	A > G
chr17:65334802:T:C:UTR3:PSMD1	chr17	65334802	65334802	T	C	UTR3	PSMD12	ENST000003	.	.	A	G	-	-	0.73134328	18,0,49,0	chr17,65334	Cytosol	A > G
chr17:65334828:T:C:UTR3:PSMD1	chr17	65334828	65334828	T	C	UTR3	PSMD12	ENST000003	.	.	A	G	-	-	0.24242424	75,0,24,0	chr17,65334	Cytosol	A > G
chr17:65334856:T:C:UTR3:PSMD1	chr17	65334856	65334856	T	C	UTR3	PSMD12	ENST000003	.	.	A	G	-	-	0.41732283	74,0,53,0	chr17,65334	Cytosol	A > G
chr17:65334886:T:C:UTR3:PSMD1	chr17	65334886	65334886	T	C	UTR3	PSMD12	ENST000003	.	.	A	G	-	-	0.25609756	122,0,42,0	chr17,65334	Cytosol	A > G
chr17:65334930:T:C:UTR3:PSMD1	chr17	65334930	65334930	T	C	UTR3	PSMD12	ENST000003	.	.	A	G	-	-	0.16239316	98,0,19,0	chr17,65334	Cytosol	A > G
chr17:65335352:T:C:UTR3:PSMD1	chr17	65335352	65335352	T	C	UTR3	PSMD12	ENST000003	.	.	A	G	-	-	0.74757282	26,0,77,0	chr17,65335	Cytosol	A > G
chr17:65335356:T:C:UTR3:PSMD1	chr17	65335356	65335356	T	C	UTR3	PSMD12	ENST000003	.	.	A	G	-	-	0.08421053	87,0,8,0	chr17,65335	Cytosol	A > G
chr17:65335392:T:C:UTR3:PSMD1	chr17	65335392	65335392	T	C	UTR3	PSMD12	ENST000003	.	.	A	G	-	-	0.07792208	71,0,6,0	chr17,65335	Cytosol	A > G
chr17:65335394:T:C:UTR3:PSMD1	chr17	65335394	65335394	T	C	UTR3	PSMD12	ENST000003	.	.	A	G	-	-	0.14102564	67,0,11,0	chr17,65335	Cytosol	A > G
chr17:65335459:T:C:UTR3:PSMD1	chr17	65335459	65335459	T	C	UTR3	PSMD12	ENST000003	.	.	A	G	-	-	0.50666667	37,0,38,0	chr17,65335	Cytosol	A > G
chr17:65335460:T:C:UTR3:PSMD1	chr17	65335460	65335460	T	C	UTR3	PSMD12	ENST000003	.	.	A	G	-	-	0.34210526	50,0,26,0	chr17,65335	Cytosol	A > G
chr17:65335477:T:C:UTR3:PSMD1	chr17	65335477	65335477	T	C	UTR3	PSMD12	ENST000003	.	.	A	G	-	-	0.21348315	70,0,19,0	chr17,65335	Cytosol	A > G
chr17:65335491:T:C:UTR3:PSMD1	chr17	65335491	65335491	T	C	UTR3	PSMD12	ENST000003	.	.	A	G	-	-	0.06097561	77,0,5,0	chr17,65335	Cytosol	A > G
chr17:65335494:T:C:UTR3:PSMD1	chr17	65335494	65335494	T	C	UTR3	PSMD12	ENST000003	.	.	A	G	-	-	0.02469136	79,0,2,0	chr17,65335	Cytosol	A > G
chr17:66111289:A:G:ncRNA_exon	chr17	66111289	66111289	A	G	ncRNA_exon	LINC00674	.	.	.	A	G	+	+	0.2	8,0,2,0	chr17,66111	Cytosol	A > G
chr17:66111294:A:G:ncRNA_exon	chr17	66111294	66111294	A	G	ncRNA_exon	LINC00674	.	.	.	A	G	+	+	0.25	6,0,2,0	chr17,66111	Cytosol	A > G
chr17:67313320:T:C:intronic:ABCA	chr17	67313320	67313320	T	C	intronic	ABCA5	.	.	.	A	G	-	-	0.83333333	1,0,5,0	chr17,67313	Cytosol	A > G
chr17:73885274:T:C:UTR3:TRIM65	chr17	73885274	73885274	T	C	UTR3	TRIM65	ENST000002	.	.	A	G	-	-	0.06666667	28,0,2,0	chr17,73885	Cytosol	A > G
chr17:73885912:T:C:UTR3:TRIM65	chr17	73885912	73885912	T	C	UTR3	TRIM65	ENST000002	.	.	A	G	-	-	0.04273504	112,0,5,0	chr17,73885	Cytosol	A > G
chr17:73885934:T:C:UTR3:TRIM65	chr17	73885934	73885934	T	C	UTR3	TRIM65	ENST000002	.	.	A	G	-	-	0.0462963	103,0,5,0	chr17,73885	Cytosol	A > G
chr17:73885939:T:C:UTR3:TRIM65	chr17	73885939	73885939	T	C	UTR3	TRIM65	ENST000002	.	.	A	G	-	-	0.35779817	70,0,39,0	chr17,73885	Cytosol	A > G
chr17:73886004:T:C:UTR3:TRIM65	chr17	73886004	73886004	T	C	UTR3	TRIM65	ENST000002	.	.	A	G	-	-	0.24	76,0,24,0	chr17,73886	Cytosol	A > G
chr17:73886212:T:C:UTR3:TRIM65	chr17	73886212	73886212	T	C	UTR3	TRIM65	ENST000002	.	.	A	G	-	-	0.10294118	122,0,14,0	chr17,73886	Cytosol	A > G
chr17:73886252:T:C:UTR3:TRIM65	chr17	73886252	73886252	T	C	UTR3	TRIM65	ENST000002	.	.	A	G	-	-	0.07971014	127,0,11,0	chr17,73886	Cytosol	A > G
chr17:73940067:T:C:ncRNA_exon	chr17	73940067	73940067	T	C	ncRNA_exon	RP11-552F3.	.	.	.	A	G	-	-+	0.39534884	26,0,17,0	chr17,73940	Cytosol	A > G
chr17:73940077:T:C:ncRNA_intror	chr17	73940077	73940077	T	C	ncRNA_intro	RP11-552F3.	.	.	.	A	G	-	-+	0.67741935	20,0,42,0	chr17,73940	Cytosol	A > G
chr17:73940094:T:C:ncRNA_intror	chr17	73940094	73940094	T	C	ncRNA_intro	RP11-552F3.	.	.	.	A	G	-	-+	0.35416667	31,0,17,0	chr17,73940	Cytosol	A > G

chr17:73940109:T:C:ncRNA_intror	chr17	73940109	73940109	T	C	ncRNA_intro	RP11-552F3.	.	.	.	A	G	-	-+	0.34146341	27,0,14,0	chr17,73940	Cytosol	A > G
chr17:73940120:T:C:ncRNA_intror	chr17	73940120	73940120	T	C	ncRNA_intro	RP11-552F3.	.	.	.	A	G	-	-+	0.35135135	24,0,13,0	chr17,73940	Cytosol	A > G
chr17:73940157:T:C:ncRNA_intror	chr17	73940157	73940157	T	C	ncRNA_intro	RP11-552F3.	.	.	.	A	G	-	-+	0.2173913	18,0,5,0	chr17,73940	Cytosol	A > G
chr17:73940166:T:C:ncRNA_intror	chr17	73940166	73940166	T	C	ncRNA_intro	RP11-552F3.	.	.	.	A	G	-	-+	0.35	13,0,7,0	chr17,73940	Cytosol	A > G
chr17:73940167:T:C:ncRNA_intror	chr17	73940167	73940167	T	C	ncRNA_intro	RP11-552F3.	.	.	.	A	G	-	-+	0.5	11,0,11,0	chr17,73940	Cytosol	A > G
chr17:73940187:T:C:ncRNA_intror	chr17	73940187	73940187	T	C	ncRNA_intro	RP11-552F3.	.	.	.	A	G	-	-+	0.80769231	5,0,21,0	chr17,73940	Cytosol	A > G
chr17:73940193:T:C:ncRNA_intror	chr17	73940193	73940193	T	C	ncRNA_intro	RP11-552F3.	.	.	.	A	G	-	-+	0.83333333	4,0,20,0	chr17,73940	Cytosol	A > G
chr17:73940231:T:C:ncRNA_intror	chr17	73940231	73940231	T	C	ncRNA_intro	RP11-552F3.	.	.	.	A	G	-	-+	0.28	18,0,7,0	chr17,73940	Cytosol	A > G
chr17:73940232:T:C:ncRNA_intror	chr17	73940232	73940232	T	C	ncRNA_intro	RP11-552F3.	.	.	.	A	G	-	-+	0.10714286	25,0,3,0	chr17,73940	Cytosol	A > G
chr17:73940875:T:C:ncRNA_intror	chr17	73940875	73940875	T	C	ncRNA_intro	RP11-552F3.	.	.	.	A	G	-	-+	0.23728814	45,0,14,0	chr17,73940	Cytosol	A > G
chr17:73940888:T:C:ncRNA_intror	chr17	73940888	73940888	T	C	ncRNA_intro	RP11-552F3.	.	.	.	A	G	-	-+	0.175	33,0,7,0	chr17,73940	Cytosol	A > G
chr17:73940906:T:C:ncRNA_intror	chr17	73940906	73940906	T	C	ncRNA_intro	RP11-552F3.	.	.	.	A	G	-	-+	0.05405405	35,0,2,0	chr17,73940	Cytosol	A > G
chr17:73940917:T:C:ncRNA_intror	chr17	73940917	73940917	T	C	ncRNA_intro	RP11-552F3.	.	.	.	A	G	-	-+	0.23913043	35,0,11,0	chr17,73940	Cytosol	A > G
chr17:73940918:T:C:ncRNA_intror	chr17	73940918	73940918	T	C	ncRNA_intro	RP11-552F3.	.	.	.	A	G	-	-+	0.44680851	26,0,21,0	chr17,73940	Cytosol	A > G
chr17:73940932:T:C:ncRNA_intror	chr17	73940932	73940932	T	C	ncRNA_intro	RP11-552F3.	.	.	.	A	G	-	-+	0.23529412	26,0,8,0	chr17,73940	Cytosol	A > G
chr17:73940959:T:C:ncRNA_intror	chr17	73940959	73940959	T	C	ncRNA_intro	RP11-552F3.	.	.	.	A	G	-	-+	0.08333333	33,0,3,0	chr17,73940	Cytosol	A > G
chr17:73940988:T:C:ncRNA_intror	chr17	73940988	73940988	T	C	ncRNA_intro	RP11-552F3.	.	.	.	A	G	-	-+	0.14814815	23,0,4,0	chr17,73940	Cytosol	A > G
chr17:73941001:T:C:ncRNA_intror	chr17	73941001	73941001	T	C	ncRNA_intro	RP11-552F3.	.	.	.	A	G	-	-+	0.19047619	17,0,4,0	chr17,73941	Cytosol	A > G
chr17:73941003:T:C:ncRNA_intror	chr17	73941003	73941003	T	C	ncRNA_intro	RP11-552F3.	.	.	.	A	G	-	-+	0.08333333	22,0,2,0	chr17,73941	Cytosol	A > G
chr17:73941030:T:C:ncRNA_intror	chr17	73941030	73941030	T	C	ncRNA_intro	RP11-552F3.	.	.	.	A	G	-	-+	0.375	5,0,3,0	chr17,73941	Cytosol	A > G
chr17:73941064:T:C:ncRNA_intror	chr17	73941064	73941064	T	C	ncRNA_intro	RP11-552F3.	.	.	.	A	G	-	-+	0.5	4,0,4,0	chr17,73941	Cytosol	A > G
chr17:73941076:T:C:ncRNA_intror	chr17	73941076	73941076	T	C	ncRNA_intro	RP11-552F3.	.	.	.	A	G	-	-+	0.22222222	7,0,2,0	chr17,73941	Cytosol	A > G
chr17:73941094:T:C:ncRNA_intror	chr17	73941094	73941094	T	C	ncRNA_intro	RP11-552F3.	.	.	.	A	G	-	-+	0.2	16,0,4,0	chr17,73941	Cytosol	A > G
chr17:73941099:T:C:ncRNA_intror	chr17	73941099	73941099	T	C	ncRNA_intro	RP11-552F3.	.	.	.	A	G	-	-+	0.35	13,0,7,0	chr17,73941	Cytosol	A > G
chr17:73941108:T:C:ncRNA_intror	chr17	73941108	73941108	T	C	ncRNA_intro	RP11-552F3.	.	.	.	A	G	-	-+	0.08695652	21,0,2,0	chr17,73941	Cytosol	A > G
chr17:73941122:T:C:ncRNA_intror	chr17	73941122	73941122	T	C	ncRNA_intro	RP11-552F3.	.	.	.	A	G	-	-+	0.09090909	20,0,2,0	chr17,73941	Cytosol	A > G
chr17:74673246:T:C:UTR3:MXRA7	chr17	74673246	74673246	T	C	UTR3	MXRA7	ENST000003	.	.	A	G	-	-	0.06666667	28,0,2,0	chr17,74673	Cytosol	A > G
chr17:74673291:T:C:UTR3:MXRA7	chr17	74673291	74673291	T	C	UTR3	MXRA7	ENST000003	.	.	A	G	-	-	0.13333333	26,0,4,0	chr17,74673	Cytosol	A > G
chr17:74673292:T:C:UTR3:MXRA7	chr17	74673292	74673292	T	C	UTR3	MXRA7	ENST000003	.	.	A	G	-	-	0.125	28,0,4,0	chr17,74673	Cytosol	A > G
chr17:74723543:A:G:intronic:MET	chr17	74723543	74723543	A	G	intronic	METTL23	.	.	.	A	G	+	+	0.125	14,0,2,0	chr17,74723	Cytosol	A > G
chr17:74723544:A:G:intronic:MET	chr17	74723544	74723544	A	G	intronic	METTL23	.	.	.	A	G	+	+	0.17647059	14,0,3,0	chr17,74723	Cytosol	A > G
chr17:74723550:A:G:intronic:MET	chr17	74723550	74723550	A	G	intronic	METTL23	.	.	.	A	G	+	+	0.35294118	11,0,6,0	chr17,74723	Cytosol	A > G
chr17:74774917:A:G:UTR3:MFSD1	chr17	74774917	74774917	A	G	UTR3	MFSD11	ENST000003	.	.	A	G	+	+	0.05405405	35,0,2,0	chr17,74774	Cytosol	A > G
chr17:74775080:A:G:UTR3:MFSD1	chr17	74775080	74775080	A	G	UTR3	MFSD11	ENST000003	.	.	A	G	+	+	0.23529412	13,0,4,0	chr17,74775	Cytosol	A > G
chr17:74775215:A:G:UTR3:MFSD1	chr17	74775215	74775215	A	G	UTR3	MFSD11	ENST000003	.	.	A	G	+	+	0.2	12,0,3,0	chr17,74775	Cytosol	A > G
chr17:76203465:A:G:UTR3:AFMID	chr17	76203465	76203465	A	G	UTR3	AFMID	ENST000004	.	.	A	G	+	+	0.04123711	93,0,4,0	chr17,76203	Cytosol	A > G
chr17:78294253:A:G:UTR3:RNF21	chr17	78294253	78294253	A	G	UTR3	RNF213	ENST000003	.	.	A	G	+	+	0.57142857	3,0,4,0	chr17,78294	Cytosol	A > G
chr17:78294464:A:G:UTR3:RNF21	chr17	78294464	78294464	A	G	UTR3	RNF213	ENST000003	.	.	A	G	+	+	1	0,0,6,0	chr17,78294	Cytosol	A > G
chr17:78294728:A:G:UTR3:RNF21	chr17	78294728	78294728	A	G	UTR3	RNF213	ENST000003	.	.	A	G	+	+	0.61538462	5,0,8,0	chr17,78294	Cytosol	A > G
chr17:78294742:A:G:UTR3:RNF21	chr17	78294742	78294742	A	G	UTR3	RNF213	ENST000003	.	.	A	G	+	+	0.75	3,0,9,0	chr17,78294	Cytosol	A > G
chr17:78294758:A:G:UTR3:RNF21	chr17	78294758	78294758	A	G	UTR3	RNF213	ENST000003	.	.	A	G	+	+	0.13333333	13,0,2,0	chr17,78294	Cytosol	A > G
chr17:78294774:A:G:UTR3:RNF21	chr17	78294774	78294774	A	G	UTR3	RNF213	ENST000003	.	.	A	G	+	+	0.23529412	13,0,4,0	chr17,78294	Cytosol	A > G
chr17:78294775:A:G:UTR3:RNF21	chr17	78294775	78294775	A	G	UTR3	RNF213	ENST000003	.	.	A	G	+	+	0.11111111	16,0,2,0	chr17,78294	Cytosol	A > G
chr17:78976869:A:G:intronic:CHM	chr17	78976869	78976869	A	G	intronic	CHMP6	.	.	.	A	G	+	+	0.4	3,0,2,0	chr17,78976	Cytosol	A > G
chr17:79527657:T:C:splicing:NPLO	chr17	79527657	79527657	T	C	splicing	NPLOC4	ENST000005	.	.	A	G	-	-	0.33333333	5,0,3,1	chr17,79527	Cytosol	A > G
chr17:79529841:T:C:UTR3:NPLOC	chr17	79529841	79529841	T	C	UTR3	NPLOC4	ENST000003	.	.	A	G	-	-	0.28571429	5,0,2,0	chr17,79529	Cytosol	A > G
chr17:79530985:T:C:exonic:NPLO	chr17	79530985	79530985	T	C	exonic	NPLOC4	.	unknown	UNKNOWN	A	G	-	-	0.25	6,0,2,0	chr17,79530	Cytosol	A > G
chr17:79604839:A:G:upstream:NP	chr17	79604839	79604839	A	G	upstream	NPLOC4	.	.	.	A	G	+	-+	0.375	5,0,3,0	chr17,79604	Cytosol	A > G
chr17:79604840:A:G:upstream:NP	chr17	79604840	79604840	A	G	upstream	NPLOC4	.	.	.	A	G	+	-+	0.55555556	4,0,5,0	chr17,79604	Cytosol	A > G
chr17:79648328:T:C:UTR3:ARL16	chr17	79648328	79648328	T	C	UTR3	ARL16	ENST000003	.	.	A	G	-	-	0.02597403	75,0,2,0	chr17,79648	Cytosol	A > G
chr17:79648396:T:C:UTR3:ARL16	chr17	79648396	79648396	T	C	UTR3	ARL16	ENST000003	.	.	A	G	-	-	0.03658537	79,0,3,0	chr17,79648	Cytosol	A > G
chr17:79648434:T:C:UTR3:ARL16	chr17	79648434	79648434	T	C	UTR3	ARL16	ENST000003	.	.	A	G	-	-	0.01851852	106,0,2,0	chr17,79648	Cytosol	A > G
chr17:79838812:T:C:ncRNA_exoni	chr17	79838812	79838812	T	C	ncRNA_exon	RP11-498C9.	.	.	.	A	G	-	-	0.2	8,0,2,0	chr17,79838	Cytosol	A > G
chr17:80347528:T:C:UTR3:OGFOD	chr17	80347528	80347528	T	C	UTR3	OGFOD3	ENST000003	.	.	A	G	-	-	0.71428571	2,0,5,0	chr17,80347	Cytosol	A > G
chr17:80446517:A:G:UTR3:NARF	chr17	80446517	80446517	A	G	UTR3	NARF	ENST000003	.	.	A	G	+	+	0.11111111	16,0,2,0	chr17,80446	Cytosol	A > G
chr18:2581573:A:G:intronic:NDC8	chr18	2581573	2581573	A	G	intronic	NDC80	.	.	.	A	G	+	+	0.58333333	5,0,7,0	chr18,25815	Cytosol	A > G
chr18:2581583:A:G:intronic:NDC8	chr18	2581583	2581583	A	G	intronic	NDC80	.	.	.	A	G	+	+	0.30769231	9,0,4,0	chr18,25815	Cytosol	A > G
chr18:2581645:A:G:intronic:NDC8	chr18	2581645	2581645	A	G	intronic	NDC80	.	.	.	A	G	+	+	0.4	6,0,4,0	chr18,25816	Cytosol	A > G
chr18:9368300:A:G:intronic:TWSG	chr18	9368300	9368300	A	G	intronic	TWSG1	.	.	.	A	G	+	+	0.8	1,0,4,0	chr18,93682	Cytosol	A > G
chr18:12482347:T:C:intronic:SPIRE	chr18	12482347	12482347	T	C	intronic	SPIRE1	.	.	.	A	G	-	-	0.57142857	3,0,4,0	chr18,12482	Cytosol	A > G
chr18:19211233:A:G:ncRNA_intro	chr18	19211233	19211233	A	G	ncRNA_intro	RP11-13N13.	.	.	.	A	G	+	+	0.58333333	5,0,7,0	chr18,19211	Cytosol	A > G
chr18:19212375:A:G:ncRNA_intro	chr18	19212375	19212375	A	G	ncRNA_intro	RP11-13N13.	.	.	.	A	G	+	+	0.61111111	7,0,11,0	chr18,19212	Cytosol	A > G
chr18:19213073:A:G:ncRNA_intro	chr18	19213073	19213073	A	G	ncRNA_intro	RP11-13N13.	.	.	.	A	G	+	+	0.66666667	2,0,4,0	chr18,19213	Cytosol	A > G
chr18:20876322:T:C:UTR3:TMEM2	chr18	20876322	20876322	T	C	UTR3	TMEM241	ENST000003	.	.	A	G	-	-	0.03508772	55,0,2,0	chr18,20876	Cytosol	A > G

chr18:20876367:T:C:UTR3:TMEM2	chr18	20876367	20876367	T	C	UTR3	TMEM241	ENST000003	.	.	A	G	-	-	0.08695652	21,0,2,0	chr18,20876	Cytosol	A > G
chr18:20876381:T:C:UTR3:TMEM2	chr18	20876381	20876381	T	C	UTR3	TMEM241	ENST000003	.	.	A	G	-	-	0.32	17,0,8,0	chr18,20876	Cytosol	A > G
chr18:20876394:T:C:UTR3:TMEM2	chr18	20876394	20876394	T	C	UTR3	TMEM241	ENST000003	.	.	A	G	-	-	0.17948718	32,0,7,0	chr18,20876	Cytosol	A > G
chr18:20876567:T:C:UTR3:TMEM2	chr18	20876567	20876567	T	C	UTR3	TMEM241	ENST000003	.	.	A	G	-	-	0.07692308	36,0,3,0	chr18,20876	Cytosol	A > G
chr18:20877422:T:C:UTR3:TMEM2	chr18	20877422	20877422	T	C	UTR3	TMEM241	ENST000003	.	.	A	G	-	-	0.22222222	28,0,8,0	chr18,20877	Cytosol	A > G
chr18:20877468:T:C:UTR3:TMEM2	chr18	20877468	20877468	T	C	UTR3	TMEM241	ENST000003	.	.	A	G	-	-	0.10526316	17,0,2,0	chr18,20877	Cytosol	A > G
chr18:29648903:A:G:ncRNA_intro	chr18	29648903	29648903	A	G	ncRNA_intro	RP11-53I6.2	.	.	.	A	G	+	+	0.36363636	7,0,4,0	chr18,29648	Cytosol	A > G
chr18:29648939:A:G:ncRNA_intro	chr18	29648939	29648939	A	G	ncRNA_intro	RP11-53I6.2	.	.	.	A	G	+	+	0.4	3,0,2,0	chr18,29648	Cytosol	A > G
chr18:29648942:A:G:ncRNA_intro	chr18	29648942	29648942	A	G	ncRNA_intro	RP11-53I6.2	.	.	.	A	G	+	+	0.8	1,0,4,0	chr18,29648	Cytosol	A > G
chr18:29648947:A:G:ncRNA_intro	chr18	29648947	29648947	A	G	ncRNA_intro	RP11-53I6.2	.	.	.	A	G	+	+	1	0,0,5,0	chr18,29648	Cytosol	A > G
chr18:29649002:A:G:ncRNA_intro	chr18	29649002	29649002	A	G	ncRNA_intro	RP11-53I6.2	.	.	.	A	G	+	+	0.46153846	7,0,6,0	chr18,29649	Cytosol	A > G
chr18:29649004:A:G:ncRNA_intro	chr18	29649004	29649004	A	G	ncRNA_intro	RP11-53I6.2	.	.	.	A	G	+	+	0.1875	13,0,3,0	chr18,29649	Cytosol	A > G
chr18:29649008:A:G:ncRNA_intro	chr18	29649008	29649008	A	G	ncRNA_intro	RP11-53I6.2	.	.	.	A	G	+	+	0.11764706	15,0,2,0	chr18,29649	Cytosol	A > G
chr18:29649017:A:G:ncRNA_intro	chr18	29649017	29649017	A	G	ncRNA_intro	RP11-53I6.2	.	.	.	A	G	+	+	0.31578947	13,0,6,0	chr18,29649	Cytosol	A > G
chr18:29649043:A:G:ncRNA_intro	chr18	29649043	29649043	A	G	ncRNA_intro	RP11-53I6.2	.	.	.	A	G	+	+	0.4	6,0,4,0	chr18,29649	Cytosol	A > G
chr18:29649073:A:G:ncRNA_intro	chr18	29649073	29649073	A	G	ncRNA_intro	RP11-53I6.2	.	.	.	A	G	+	+	0.5	4,0,4,0	chr18,29649	Cytosol	A > G
chr18:29649096:A:G:ncRNA_intro	chr18	29649096	29649096	A	G	ncRNA_intro	RP11-53I6.2	.	.	.	A	G	+	+	0.5	4,0,4,0	chr18,29649	Cytosol	A > G
chr18:29649103:A:G:ncRNA_intro	chr18	29649103	29649103	A	G	ncRNA_intro	RP11-53I6.2	.	.	.	A	G	+	+	1	0,0,7,0	chr18,29649	Cytosol	A > G
chr18:29649121:A:G:ncRNA_intro	chr18	29649121	29649121	A	G	ncRNA_intro	RP11-53I6.2	.	.	.	A	G	+	+	0.57142857	3,0,4,0	chr18,29649	Cytosol	A > G
chr18:29649127:A:G:ncRNA_intro	chr18	29649127	29649127	A	G	ncRNA_intro	RP11-53I6.2	.	.	.	A	G	+	+	0.66666667	2,0,4,0	chr18,29649	Cytosol	A > G
chr18:29650083:A:G:ncRNA_intro	chr18	29650083	29650083	A	G	ncRNA_intro	RP11-53I6.2	.	.	.	A	G	+	+	0.75	2,0,6,0	chr18,29650	Cytosol	A > G
chr18:29650104:A:G:ncRNA_intro	chr18	29650104	29650104	A	G	ncRNA_intro	RP11-53I6.2	.	.	.	A	G	+	+	0.41666667	7,0,5,0	chr18,29650	Cytosol	A > G
chr18:29650107:A:G:ncRNA_intro	chr18	29650107	29650107	A	G	ncRNA_intro	RP11-53I6.2	.	.	.	A	G	+	+	0.4	6,0,4,0	chr18,29650	Cytosol	A > G
chr18:29650169:A:G:ncRNA_intro	chr18	29650169	29650169	A	G	ncRNA_intro	RP11-53I6.2	.	.	.	A	G	+	+	0.35714286	9,0,5,0	chr18,29650	Cytosol	A > G
chr18:29650729:A:G:ncRNA_intro	chr18	29650729	29650729	A	G	ncRNA_intro	RP11-53I6.2	.	.	.	A	G	+	+	0.64285714	5,0,9,0	chr18,29650	Cytosol	A > G
chr18:29651198:A:G:ncRNA_intro	chr18	29651198	29651198	A	G	ncRNA_intro	RP11-53I6.2	.	.	.	A	G	+	+	0.18181818	9,0,2,0	chr18,29651	Cytosol	A > G
chr18:29651263:A:G:ncRNA_intro	chr18	29651263	29651263	A	G	ncRNA_intro	RP11-53I6.2	.	.	.	A	G	+	+	0.14285714	18,0,3,0	chr18,29651	Cytosol	A > G
chr18:29651886:A:G:ncRNA_intro	chr18	29651886	29651886	A	G	ncRNA_intro	RP11-53I6.2	.	.	.	A	G	+	+	0.23076923	10,0,3,0	chr18,29651	Cytosol	A > G
chr18:29651890:A:G:ncRNA_intro	chr18	29651890	29651890	A	G	ncRNA_intro	RP11-53I6.2	.	.	.	A	G	+	+	0.2	12,0,3,0	chr18,29651	Cytosol	A > G
chr18:29651892:A:G:ncRNA_intro	chr18	29651892	29651892	A	G	ncRNA_intro	RP11-53I6.2	.	.	.	A	G	+	+	0.15789474	16,0,3,0	chr18,29651	Cytosol	A > G
chr18:29652057:A:G:ncRNA_intro	chr18	29652057	29652057	A	G	ncRNA_intro	RP11-53I6.2	.	.	.	A	G	+	+	0.4375	9,0,7,0	chr18,29652	Cytosol	A > G
chr18:29652079:A:G:ncRNA_intro	chr18	29652079	29652079	A	G	ncRNA_intro	RP11-53I6.2	.	.	.	A	G	+	+	0.125	14,0,2,0	chr18,29652	Cytosol	A > G
chr18:29652119:A:G:ncRNA_intro	chr18	29652119	29652119	A	G	ncRNA_intro	RP11-53I6.2	.	.	.	A	G	+	+	0.57142857	12,0,16,0	chr18,29652	Cytosol	A > G
chr18:32827764:A:G:intronic:ZNF3	chr18	32827764	32827764	A	G	intronic	ZNF397	.	.	.	A	G	+	+	0.22222222	7,0,2,0	chr18,32827	Cytosol	A > G
chr18:32828543:A:G:intronic:ZNF3	chr18	32828543	32828543	A	G	intronic	ZNF397	.	.	.	A	G	+	+	0.29411765	12,0,5,0	chr18,32828	Cytosol	A > G
chr18:32828547:A:G:intronic:ZNF3	chr18	32828547	32828547	A	G	intronic	ZNF397	.	.	.	A	G	+	+	0.10526316	17,0,2,0	chr18,32828	Cytosol	A > G
chr18:32828603:A:G:intronic:ZNF3	chr18	32828603	32828603	A	G	intronic	ZNF397	.	.	.	A	G	+	+	0.26923077	19,0,7,0	chr18,32828	Cytosol	A > G
chr18:32828623:A:G:intronic:ZNF3	chr18	32828623	32828623	A	G	intronic	ZNF397	.	.	.	A	G	+	+	0.26315789	14,0,5,0	chr18,32828	Cytosol	A > G
chr18:32828672:A:G:intronic:ZNF3	chr18	32828672	32828672	A	G	intronic	ZNF397	.	.	.	A	G	+	+	0.08695652	21,0,2,0	chr18,32828	Cytosol	A > G
chr18:32839403:T:C:intronic:ZSCA	chr18	32839403	32839403	T	C	intronic	ZSCAN30	.	.	.	A	G	-	+-	0.8	1,0,4,0	chr18,32839	Cytosol	A > G
chr18:32843089:T:C:UTR3:ZSCAN3	chr18	32843089	32843089	T	C	UTR3	ZSCAN30	ENST000005	.	.	A	G	-	+-	0.5	7,0,7,0	chr18,32843	Cytosol	A > G
chr18:33754753:A:G:UTR3:ELP2	chr18	33754753	33754753	A	G	UTR3	ELP2	ENST000003	.	.	A	G	+	+	0.06557377	57,0,4,0	chr18,33754	Cytosol	A > G
chr18:34465792:A:G:UTR3:KIAA13	chr18	34465792	34465792	A	G	UTR3	KIAA1328	ENST000005	.	.	A	G	+	+	0.5	3,0,3,0	chr18,34465	Cytosol	A > G
chr18:34465809:A:G:UTR3:KIAA13	chr18	34465809	34465809	A	G	UTR3	KIAA1328	ENST000005	.	.	A	G	+	+	0.33333333	4,0,2,0	chr18,34465	Cytosol	A > G
chr18:46915978:T:C:intronic:DYM	chr18	46915978	46915978	T	C	intronic	DYM	.	.	.	A	G	-	-	0.6	2,0,3,0	chr18,46915	Cytosol	A > G
chr18:47918813:A:G:UTR3:SKA1	chr18	47918813	47918813	A	G	UTR3	SKA1	ENST000002	.	.	A	G	+	+	0.05	38,0,2,0	chr18,47918	Cytosol	A > G
chr18:47919174:A:G:UTR3:SKA1	chr18	47919174	47919174	A	G	UTR3	SKA1	ENST000002	.	.	A	G	+	+	0.3125	33,0,15,0	chr18,47919	Cytosol	A > G
chr18:47919601:A:G:UTR3:SKA1	chr18	47919601	47919601	A	G	UTR3	SKA1	ENST000002	.	.	A	G	+	+	0.09090909	40,0,4,0	chr18,47919	Cytosol	A > G
chr18:56418421:A:G:ncRNA_intro	chr18	56418421	56418421	A	G	ncRNA_intro	RP11-126O1	.	.	.	A	G	+	-	0.33333333	4,0,2,0	chr18,56418	Cytosol	A > G
chr18:56418423:A:G:ncRNA_intro	chr18	56418423	56418423	A	G	ncRNA_intro	RP11-126O1	.	.	.	A	G	+	-	0.5	3,0,3,0	chr18,56418	Cytosol	A > G
chr18:56418477:A:G:ncRNA_intro	chr18	56418477	56418477	A	G	ncRNA_intro	RP11-126O1	.	.	.	A	G	+	-	0.42857143	8,0,6,0	chr18,56418	Cytosol	A > G
chr18:56418478:A:G:ncRNA_intro	chr18	56418478	56418478	A	G	ncRNA_intro	RP11-126O1	.	.	.	A	G	+	-	0.42857143	8,0,6,0	chr18,56418	Cytosol	A > G
chr18:56418542:A:G:ncRNA_intro	chr18	56418542	56418542	A	G	ncRNA_intro	RP11-126O1	.	.	.	A	G	+	-	0.17647059	14,0,3,0	chr18,56418	Cytosol	A > G
chr18:56419451:A:G:ncRNA_exon	chr18	56419451	56419451	A	G	ncRNA_exon	RP11-108P2	.	.	.	A	G	+	+-	0.14285714	18,0,3,0	chr18,56419	Cytosol	A > G
chr18:56419749:A:G:ncRNA_exon	chr18	56419749	56419749	A	G	ncRNA_exon	RP11-108P2	.	.	.	A	G	+	+-	0.57142857	3,0,4,0	chr18,56419	Cytosol	A > G
chr18:56419783:A:G:ncRNA_exon	chr18	56419783	56419783	A	G	ncRNA_exon	RP11-108P2	.	.	.	A	G	+	+-	0.13333333	13,0,2,0	chr18,56419	Cytosol	A > G
chr18:56419885:A:G:ncRNA_exon	chr18	56419885	56419885	A	G	ncRNA_exon	RP11-108P2	.	.	.	A	G	+	+-	0.10344828	26,0,3,0	chr18,56419	Cytosol	A > G
chr18:56419911:A:G:ncRNA_exon	chr18	56419911	56419911	A	G	ncRNA_exon	RP11-108P2	.	.	.	A	G	+	+-	0.11538462	23,0,3,0	chr18,56419	Cytosol	A > G
chr18:56419925:A:G:ncRNA_exon	chr18	56419925	56419925	A	G	ncRNA_exon	RP11-108P2	.	.	.	A	G	+	+-	0.35714286	18,0,10,0	chr18,56419	Cytosol	A > G
chr18:56419935:A:G:ncRNA_exon	chr18	56419935	56419935	A	G	ncRNA_exon	RP11-108P2	.	.	.	A	G	+	+-	0.23076923	20,0,6,0	chr18,56419	Cytosol	A > G
chr18:56419964:A:G:ncRNA_exon	chr18	56419964	56419964	A	G	ncRNA_exon	RP11-108P2	.	.	.	A	G	+	+-	0.24137931	22,0,7,0	chr18,56419	Cytosol	A > G
chr18:56419971:A:G:ncRNA_exon	chr18	56419971	56419971	A	G	ncRNA_exon	RP11-108P2	.	.	.	A	G	+	+-	0.34615385	17,0,9,0	chr18,56419	Cytosol	A > G
chr18:56420505:A:G:ncRNA_exon	chr18	56420505	56420505	A	G	ncRNA_exon	RP11-108P2	.	.	.	A	G	+	+-	0.3125	11,0,5,0	chr18,56420	Cytosol	A > G
chr18:56420506:A:G:ncRNA_exon	chr18	56420506	56420506	A	G	ncRNA_exon	RP11-108P2	.	.	.	A	G	+	+-	0.21428571	11,0,3,0	chr18,56420	Cytosol	A > G

chr18:56420519:A:G:ncRNA_exoni	chr18	56420519	56420519	A	G	ncRNA_exon	RP11-108P2	.	.	.	A	G	+	+-	0.25	12,0,4,0	chr18,56420	Cytosol	A > G
chr18:56420526:A:G:ncRNA_exoni	chr18	56420526	56420526	A	G	ncRNA_exon	RP11-108P2	.	.	.	A	G	+	+-	0.5	6,0,6,0	chr18,56420	Cytosol	A > G
chr18:56420565:A:G:ncRNA_exoni	chr18	56420565	56420565	A	G	ncRNA_exon	RP11-108P2	.	.	.	A	G	+	+-	0.23076923	10,0,3,0	chr18,56420	Cytosol	A > G
chr18:56420648:A:G:ncRNA_exoni	chr18	56420648	56420648	A	G	ncRNA_exon	RP11-108P2	.	.	.	A	G	+	+-	0.16666667	10,0,2,0	chr18,56420	Cytosol	A > G
chr18:56420659:A:G:ncRNA_exoni	chr18	56420659	56420659	A	G	ncRNA_exon	RP11-108P2	.	.	.	A	G	+	+-	0.5	6,0,6,0	chr18,56420	Cytosol	A > G
chr18:56420660:A:G:ncRNA_exoni	chr18	56420660	56420660	A	G	ncRNA_exon	RP11-108P2	.	.	.	A	G	+	+-	0.25	9,0,3,0	chr18,56420	Cytosol	A > G
chr18:56420661:A:G:ncRNA_exoni	chr18	56420661	56420661	A	G	ncRNA_exon	RP11-108P2	.	.	.	A	G	+	+-	0.18181818	9,0,2,0	chr18,56420	Cytosol	A > G
chr18:65174489:T:C:ncRNA_intror	chr18	65174489	65174489	T	C	ncRNA_intro	CTD-2541J13	.	.	.	A	G	-	-+	0.18367347	40,0,9,0	chr18,65174	Cytosol	A > G
chr18:65174501:T:C:ncRNA_intror	chr18	65174501	65174501	T	C	ncRNA_intro	CTD-2541J13	.	.	.	A	G	-	-+	0.34146341	27,0,14,0	chr18,65174	Cytosol	A > G
chr18:77916478:T:C:UTR3:PARD6	chr18	77916478	77916478	T	C	UTR3	PARD6G	ENST000003	.	.	A	G	-	+-	1	0,0,6,0	chr18,77916	Cytosol	A > G
chr18:77916484:T:C:UTR3:PARD6	chr18	77916484	77916484	T	C	UTR3	PARD6G	ENST000003	.	.	A	G	-	+-	0.625	3,0,5,0	chr18,77916	Cytosol	A > G
chr19:1032834:A:G:UTR3:CNN2	chr19	1032834	1032834	A	G	UTR3	CNN2	ENST000005	.	.	A	G	+	+	0.81818182	2,0,9,0	chr19,10328	Cytosol	A > G
chr19:1032842:A:G:UTR3:CNN2	chr19	1032842	1032842	A	G	UTR3	CNN2	ENST000005	.	.	A	G	+	+	0.36363636	7,0,4,0	chr19,10328	Cytosol	A > G
chr19:1033058:A:G:intronic:CNN2	chr19	1033058	1033058	A	G	intronic	CNN2	.	.	.	A	G	+	+	0.5	3,0,3,0	chr19,10330	Cytosol	A > G
chr19:1033449:A:G:intronic:CNN2	chr19	1033449	1033449	A	G	intronic	CNN2	.	.	.	A	G	+	+	0.41666667	7,0,5,0	chr19,10334	Cytosol	A > G
chr19:1033457:A:G:intronic:CNN2	chr19	1033457	1033457	A	G	intronic	CNN2	.	.	.	A	G	+	+	0.5	6,0,6,0	chr19,10334	Cytosol	A > G
chr19:1033487:A:G:intronic:CNN2	chr19	1033487	1033487	A	G	intronic	CNN2	.	.	.	A	G	+	+	0.2	8,0,2,0	chr19,10334	Cytosol	A > G
chr19:1414878:A:G:intronic:DAZAI	chr19	1414878	1414878	A	G	intronic	DAZAP1	.	.	.	A	G	+	+	0.5	3,0,3,0	chr19,14148	Cytosol	A > G
chr19:1457614:T:C:intronic:APC2	chr19	1457614	1457614	T	C	intronic	APC2	.	.	.	A	G	-	+	0.6	2,0,3,0	chr19,14576	Cytosol	A > G
chr19:1459302:T:C:ncRNA_introni	chr19	1459302	1459302	T	C	ncRNA_intro	CTB-25B13.1	.	.	.	A	G	-	+-	0.21195652	145,0,39,0	chr19,14593	Cytosol	A > G
chr19:1475513:T:C:exonic:C19orf2	chr19	1475513	1475513	T	C	exonic	C19orf25	.	nonsynonym	C19orf25:EN	A	G	-	-	0.33333333	4,0,2,0	chr19,14755	Cytosol	A > G
chr19:1586573:T:C:intronic:MBD3	chr19	1586573	1586573	T	C	intronic	MBD3,UQCR	.	.	.	A	G	-	-	0.08695652	42,0,4,0	chr19,15865	Cytosol	A > G
chr19:1586610:T:C:intronic:MBD3	chr19	1586610	1586610	T	C	intronic	MBD3,UQCR	.	.	.	A	G	-	-	0.06666667	42,0,3,0	chr19,15866	Cytosol	A > G
chr19:1586624:T:C:intronic:MBD3	chr19	1586624	1586624	T	C	intronic	MBD3,UQCR	.	.	.	A	G	-	-	0.08571429	32,0,3,0	chr19,15866	Cytosol	A > G
chr19:1586733:T:C:intronic:MBD3	chr19	1586733	1586733	T	C	intronic	MBD3,UQCR	.	.	.	A	G	-	-	0.08	23,0,2,0	chr19,15867	Cytosol	A > G
chr19:1586768:T:C:intronic:MBD3	chr19	1586768	1586768	T	C	intronic	MBD3,UQCR	.	.	.	A	G	-	-	0.61904762	8,0,13,0	chr19,15867	Cytosol	A > G
chr19:1586771:T:C:intronic:MBD3	chr19	1586771	1586771	T	C	intronic	MBD3,UQCR	.	.	.	A	G	-	-	0.2	16,0,4,0	chr19,15867	Cytosol	A > G
chr19:1586777:T:C:intronic:MBD3	chr19	1586777	1586777	T	C	intronic	MBD3,UQCR	.	.	.	A	G	-	-	0.33333333	10,0,5,0	chr19,15867	Cytosol	A > G
chr19:1586790:T:C:intronic:MBD3	chr19	1586790	1586790	T	C	intronic	MBD3,UQCR	.	.	.	A	G	-	-	0.64285714	5,0,9,0	chr19,15867	Cytosol	A > G
chr19:1587143:T:C:intronic:MBD3	chr19	1587143	1587143	T	C	intronic	MBD3,UQCR	.	.	.	A	G	-	-	0.25	9,0,3,0	chr19,15871	Cytosol	A > G
chr19:1587328:T:C:intronic:MBD3	chr19	1587328	1587328	T	C	intronic	MBD3,UQCR	.	.	.	A	G	-	-	0.16666667	10,0,2,0	chr19,15873	Cytosol	A > G
chr19:1587506:T:C:intronic:MBD3	chr19	1587506	1587506	T	C	intronic	MBD3,UQCR	.	.	.	A	G	-	-	0.17391304	19,0,4,0	chr19,15875	Cytosol	A > G
chr19:1587511:T:C:intronic:MBD3	chr19	1587511	1587511	T	C	intronic	MBD3,UQCR	.	.	.	A	G	-	-	0.15789474	16,0,3,0	chr19,15875	Cytosol	A > G
chr19:1587525:T:C:intronic:MBD3	chr19	1587525	1587525	T	C	intronic	MBD3,UQCR	.	.	.	A	G	-	-	0.13333333	13,0,2,0	chr19,15875	Cytosol	A > G
chr19:1587529:T:C:intronic:MBD3	chr19	1587529	1587529	T	C	intronic	MBD3,UQCR	.	.	.	A	G	-	-	0.15789474	16,0,3,0	chr19,15875	Cytosol	A > G
chr19:1587538:T:C:intronic:MBD3	chr19	1587538	1587538	T	C	intronic	MBD3,UQCR	.	.	.	A	G	-	-	0.57894737	8,0,11,0	chr19,15875	Cytosol	A > G
chr19:1587552:T:C:intronic:MBD3	chr19	1587552	1587552	T	C	intronic	MBD3,UQCR	.	.	.	A	G	-	-	0.33333333	13,0,7,1	chr19,15875	Cytosol	A > G
chr19:1587558:T:C:intronic:MBD3	chr19	1587558	1587558	T	C	intronic	MBD3,UQCR	.	.	.	A	G	-	-	0.22727273	17,0,5,0	chr19,15875	Cytosol	A > G
chr19:1587578:T:C:intronic:MBD3	chr19	1587578	1587578	T	C	intronic	MBD3,UQCR	.	.	.	A	G	-	-	0.32	17,0,8,0	chr19,15875	Cytosol	A > G
chr19:1587581:T:C:intronic:MBD3	chr19	1587581	1587581	T	C	intronic	MBD3,UQCR	.	.	.	A	G	-	-	0.08	23,0,2,0	chr19,15875	Cytosol	A > G
chr19:1587582:T:C:intronic:MBD3	chr19	1587582	1587582	T	C	intronic	MBD3,UQCR	.	.	.	A	G	-	-	0.16	21,0,4,0	chr19,15875	Cytosol	A > G
chr19:1587589:T:C:intronic:MBD3	chr19	1587589	1587589	T	C	intronic	MBD3,UQCR	.	.	.	A	G	-	-	0.125	21,0,3,0	chr19,15875	Cytosol	A > G
chr19:1587590:T:C:intronic:MBD3	chr19	1587590	1587590	T	C	intronic	MBD3,UQCR	.	.	.	A	G	-	-	0.22727273	17,0,5,0	chr19,15875	Cytosol	A > G
chr19:1587591:T:C:intronic:MBD3	chr19	1587591	1587591	T	C	intronic	MBD3,UQCR	.	.	.	A	G	-	-	0.28571429	15,0,6,0	chr19,15875	Cytosol	A > G
chr19:1587613:T:C:intronic:MBD3	chr19	1587613	1587613	T	C	intronic	MBD3,UQCR	.	.	.	A	G	-	-	0.45	11,0,9,0	chr19,15876	Cytosol	A > G
chr19:1587626:T:C:intronic:MBD3	chr19	1587626	1587626	T	C	intronic	MBD3,UQCR	.	.	.	A	G	-	-	0.33333333	12,0,6,0	chr19,15876	Cytosol	A > G
chr19:1587638:T:C:intronic:MBD3	chr19	1587638	1587638	T	C	intronic	MBD3,UQCR	.	.	.	A	G	-	-	0.2	16,0,4,0	chr19,15876	Cytosol	A > G
chr19:1587684:T:C:intronic:MBD3	chr19	1587684	1587684	T	C	intronic	MBD3,UQCR	.	.	.	A	G	-	-	0.25	12,0,4,0	chr19,15876	Cytosol	A > G
chr19:1589312:T:C:intronic:MBD3	chr19	1589312	1589312	T	C	intronic	MBD3,UQCR	.	.	.	A	G	-	-	0.16666667	10,0,2,0	chr19,15893	Cytosol	A > G
chr19:1589787:T:C:intronic:MBD3	chr19	1589787	1589787	T	C	intronic	MBD3,UQCR	.	.	.	A	G	-	-	0.1	18,0,2,0	chr19,15897	Cytosol	A > G
chr19:1590050:T:C:intronic:MBD3	chr19	1590050	1590050	T	C	intronic	MBD3,UQCR	.	.	.	A	G	-	-	0.075	37,0,3,0	chr19,15900	Cytosol	A > G
chr19:1590062:T:C:intronic:MBD3	chr19	1590062	1590062	T	C	intronic	MBD3,UQCR	.	.	.	A	G	-	-	0.09677419	28,0,3,0	chr19,15900	Cytosol	A > G
chr19:1590114:T:C:intronic:MBD3	chr19	1590114	1590114	T	C	intronic	MBD3,UQCR	.	.	.	A	G	-	-	0.58823529	7,0,10,0	chr19,15901	Cytosol	A > G
chr19:1590173:T:C:intronic:MBD3	chr19	1590173	1590173	T	C	intronic	MBD3,UQCR	.	.	.	A	G	-	-	0.4	9,0,6,0	chr19,15901	Cytosol	A > G
chr19:1590175:T:C:intronic:MBD3	chr19	1590175	1590175	T	C	intronic	MBD3,UQCR	.	.	.	A	G	-	-	0.3125	11,0,5,0	chr19,15901	Cytosol	A > G
chr19:1590197:T:C:intronic:MBD3	chr19	1590197	1590197	T	C	intronic	MBD3,UQCR	.	.	.	A	G	-	-	0.5	8,0,8,0	chr19,15901	Cytosol	A > G
chr19:1590210:T:C:intronic:MBD3	chr19	1590210	1590210	T	C	intronic	MBD3,UQCR	.	.	.	A	G	-	-	0.125	14,0,2,0	chr19,15902	Cytosol	A > G
chr19:1590214:T:C:intronic:MBD3	chr19	1590214	1590214	T	C	intronic	MBD3,UQCR	.	.	.	A	G	-	-	0.3125	11,0,5,0	chr19,15902	Cytosol	A > G
chr19:1590234:T:C:intronic:MBD3	chr19	1590234	1590234	T	C	intronic	MBD3,UQCR	.	.	.	A	G	-	-	0.2	12,0,3,0	chr19,15902	Cytosol	A > G
chr19:1590282:T:C:intronic:MBD3	chr19	1590282	1590282	T	C	intronic	MBD3,UQCR	.	.	.	A	G	-	-	0.4	6,0,4,0	chr19,15902	Cytosol	A > G
chr19:1590358:T:C:intronic:MBD3	chr19	1590358	1590358	T	C	intronic	MBD3,UQCR	.	.	.	A	G	-	-	0.29032258	22,0,9,0	chr19,15903	Cytosol	A > G
chr19:1590376:T:C:intronic:MBD3	chr19	1590376	1590376	T	C	intronic	MBD3,UQCR	.	.	.	A	G	-	-	0.27586207	21,0,8,0	chr19,15903	Cytosol	A > G
chr19:1590386:T:C:intronic:MBD3	chr19	1590386	1590386	T	C	intronic	MBD3,UQCR	.	.	.	A	G	-	-	0.31578947	13,0,6,0	chr19,15903	Cytosol	A > G
chr19:1590477:T:C:intronic:MBD3	chr19	1590477	1590477	T	C	intronic	MBD3,UQCR	.	.	.	A	G	-	-	0.0952381	19,0,2,0	chr19,15904	Cytosol	A > G

chr19:1590489:T:C:intronic:MBD3	chr19	1590489	1590489	T	C	intronic	MBD3,UQCR	.	.	.	A	G	-	-	0.09090909	20,0,2,0	chr19,15904	Cytosol	A > G
chr19:1590495:T:C:intronic:MBD3	chr19	1590495	1590495	T	C	intronic	MBD3,UQCR	.	.	.	A	G	-	-	0.36	16,0,9,0	chr19,15904	Cytosol	A > G
chr19:1590508:T:C:intronic:MBD3	chr19	1590508	1590508	T	C	intronic	MBD3,UQCR	.	.	.	A	G	-	-	0.13043478	20,0,3,0	chr19,15905	Cytosol	A > G
chr19:1590509:T:C:intronic:MBD3	chr19	1590509	1590509	T	C	intronic	MBD3,UQCR	.	.	.	A	G	-	-	0.08	23,0,2,0	chr19,15905	Cytosol	A > G
chr19:3540129:T:C:intronic:C19orf	chr19	3540129	3540129	T	C	intronic	C19orf71,MF	.	.	.	A	G	-	-+	0.04545455	63,0,3,0	chr19,35401	Cytosol	A > G
chr19:3540130:T:C:intronic:C19orf	chr19	3540130	3540130	T	C	intronic	C19orf71,MF	.	.	.	A	G	-	-+	0.06060606	62,0,4,0	chr19,35401	Cytosol	A > G
chr19:3540159:T:C:intronic:C19orf	chr19	3540159	3540159	T	C	intronic	C19orf71,MF	.	.	.	A	G	-	-+	0.13235294	59,0,9,0	chr19,35401	Cytosol	A > G
chr19:3540160:T:C:intronic:C19orf	chr19	3540160	3540160	T	C	intronic	C19orf71,MF	.	.	.	A	G	-	-+	0.41791045	39,0,28,0	chr19,35401	Cytosol	A > G
chr19:3540174:T:C:intronic:C19orf	chr19	3540174	3540174	T	C	intronic	C19orf71,MF	.	.	.	A	G	-	-+	0.56521739	30,0,39,0	chr19,35401	Cytosol	A > G
chr19:3540196:T:C:intronic:C19orf	chr19	3540196	3540196	T	C	intronic	C19orf71,MF	.	.	.	A	G	-	-+	0.25438596	85,0,29,0	chr19,35401	Cytosol	A > G
chr19:3540199:T:C:intronic:C19orf	chr19	3540199	3540199	T	C	intronic	C19orf71,MF	.	.	.	A	G	-	-+	0.11214953	95,0,12,0	chr19,35401	Cytosol	A > G
chr19:3540213:T:C:intronic:C19orf	chr19	3540213	3540213	T	C	intronic	C19orf71,MF	.	.	.	A	G	-	-+	0.18627451	83,0,19,0	chr19,35402	Cytosol	A > G
chr19:3540216:T:C:intronic:C19orf	chr19	3540216	3540216	T	C	intronic	C19orf71,MF	.	.	.	A	G	-	-+	0.37068966	73,0,43,0	chr19,35402	Cytosol	A > G
chr19:3540230:T:C:intronic:C19orf	chr19	3540230	3540230	T	C	intronic	C19orf71,MF	.	.	.	A	G	-	-+	0.29710145	97,0,41,0	chr19,35402	Cytosol	A > G
chr19:3540285:T:C:intronic:C19orf	chr19	3540285	3540285	T	C	intronic	C19orf71,MF	.	.	.	A	G	-	-+	0.05	76,0,4,0	chr19,35402	Cytosol	A > G
chr19:3540289:T:C:intronic:C19orf	chr19	3540289	3540289	T	C	intronic	C19orf71,MF	.	.	.	A	G	-	-+	0.03529412	82,0,3,0	chr19,35402	Cytosol	A > G
chr19:3540323:T:C:intronic:C19orf	chr19	3540323	3540323	T	C	intronic	C19orf71,MF	.	.	.	A	G	-	-+	0.03508772	55,0,2,0	chr19,35403	Cytosol	A > G
chr19:3540328:T:C:intronic:C19orf	chr19	3540328	3540328	T	C	intronic	C19orf71,MF	.	.	.	A	G	-	-+	0.12820513	34,0,5,0	chr19,35403	Cytosol	A > G
chr19:3540332:T:C:intronic:C19orf	chr19	3540332	3540332	T	C	intronic	C19orf71,MF	.	.	.	A	G	-	-+	0.09302326	39,0,4,0	chr19,35403	Cytosol	A > G
chr19:3540349:T:C:intronic:C19orf	chr19	3540349	3540349	T	C	intronic	C19orf71,MF	.	.	.	A	G	-	-+	0.18518519	22,0,5,0	chr19,35403	Cytosol	A > G
chr19:3540352:T:C:intronic:C19orf	chr19	3540352	3540352	T	C	intronic	C19orf71,MF	.	.	.	A	G	-	-+	0.33333333	18,0,9,0	chr19,35403	Cytosol	A > G
chr19:3540353:T:C:intronic:C19orf	chr19	3540353	3540353	T	C	intronic	C19orf71,MF	.	.	.	A	G	-	-+	0.14705882	29,0,5,0	chr19,35403	Cytosol	A > G
chr19:3540367:T:C:intronic:C19orf	chr19	3540367	3540367	T	C	intronic	C19orf71,MF	.	.	.	A	G	-	-+	0.16666667	25,0,5,0	chr19,35403	Cytosol	A > G
chr19:3540377:T:C:intronic:C19orf	chr19	3540377	3540377	T	C	intronic	C19orf71,MF	.	.	.	A	G	-	-+	0.13513514	32,0,5,0	chr19,35403	Cytosol	A > G
chr19:3540384:T:C:intronic:C19orf	chr19	3540384	3540384	T	C	intronic	C19orf71,MF	.	.	.	A	G	-	-+	0.83783784	6,0,31,0	chr19,35403	Cytosol	A > G
chr19:3540388:T:C:intronic:C19orf	chr19	3540388	3540388	T	C	intronic	C19orf71,MF	.	.	.	A	G	-	-+	0.09375	28,0,3,1	chr19,35403	Cytosol	A > G
chr19:3540393:T:C:intronic:C19orf	chr19	3540393	3540393	T	C	intronic	C19orf71,MF	.	.	.	A	G	-	-+	0.39393939	20,0,13,0	chr19,35403	Cytosol	A > G
chr19:3540394:T:C:intronic:C19orf	chr19	3540394	3540394	T	C	intronic	C19orf71,MF	.	.	.	A	G	-	-+	0.64705882	12,0,22,0	chr19,35403	Cytosol	A > G
chr19:3540407:T:C:intronic:C19orf	chr19	3540407	3540407	T	C	intronic	C19orf71,MF	.	.	.	A	G	-	-+	1	0,0,35,0	chr19,35404	Cytosol	A > G
chr19:3540420:T:C:intronic:C19orf	chr19	3540420	3540420	T	C	intronic	C19orf71,MF	.	.	.	A	G	-	-+	0.27586207	21,0,8,0	chr19,35404	Cytosol	A > G
chr19:3540423:T:C:intronic:C19orf	chr19	3540423	3540423	T	C	intronic	C19orf71,MF	.	.	.	A	G	-	-+	0.07407407	25,0,2,0	chr19,35404	Cytosol	A > G
chr19:3540429:T:C:intronic:C19orf	chr19	3540429	3540429	T	C	intronic	C19orf71,MF	.	.	.	A	G	-	-+	0.23333333	23,0,7,0	chr19,35404	Cytosol	A > G
chr19:3540434:T:C:intronic:C19orf	chr19	3540434	3540434	T	C	intronic	C19orf71,MF	.	.	.	A	G	-	-+	0.14814815	23,0,4,0	chr19,35404	Cytosol	A > G
chr19:3540449:T:C:intronic:C19orf	chr19	3540449	3540449	T	C	intronic	C19orf71,MF	.	.	.	A	G	-	-+	0.48387097	16,0,15,0	chr19,35404	Cytosol	A > G
chr19:3540450:T:C:intronic:C19orf	chr19	3540450	3540450	T	C	intronic	C19orf71,MF	.	.	.	A	G	-	-+	0.28125	23,0,9,0	chr19,35404	Cytosol	A > G
chr19:3540451:T:C:intronic:C19orf	chr19	3540451	3540451	T	C	intronic	C19orf71,MF	.	.	.	A	G	-	-+	0.125	28,0,4,0	chr19,35404	Cytosol	A > G
chr19:3540457:T:C:intronic:C19orf	chr19	3540457	3540457	T	C	intronic	C19orf71,MF	.	.	.	A	G	-	-+	0.1025641	35,0,4,0	chr19,35404	Cytosol	A > G
chr19:3540459:T:C:intronic:C19orf	chr19	3540459	3540459	T	C	intronic	C19orf71,MF	.	.	.	A	G	-	-+	0.32432432	25,0,12,0	chr19,35404	Cytosol	A > G
chr19:3540483:T:C:intronic:C19orf	chr19	3540483	3540483	T	C	intronic	C19orf71,MF	.	.	.	A	G	-	-+	0.05882353	32,0,2,0	chr19,35404	Cytosol	A > G
chr19:3540496:T:C:intronic:C19orf	chr19	3540496	3540496	T	C	intronic	C19orf71,MF	.	.	.	A	G	-	-+	0.37837838	23,0,14,0	chr19,35404	Cytosol	A > G
chr19:3540521:T:C:intronic:C19orf	chr19	3540521	3540521	T	C	intronic	C19orf71,MF	.	.	.	A	G	-	-+	0.4137931	17,0,12,0	chr19,35405	Cytosol	A > G
chr19:3540526:T:C:intronic:C19orf	chr19	3540526	3540526	T	C	intronic	C19orf71,MF	.	.	.	A	G	-	-+	0.66666667	10,0,20,0	chr19,35405	Cytosol	A > G
chr19:3540527:T:C:intronic:C19orf	chr19	3540527	3540527	T	C	intronic	C19orf71,MF	.	.	.	A	G	-	-+	0.64285714	10,0,18,0	chr19,35405	Cytosol	A > G
chr19:3540621:T:C:intronic:C19orf	chr19	3540621	3540621	T	C	intronic	C19orf71,MF	.	.	.	A	G	-	-+	0.72727273	6,0,16,0	chr19,35406	Cytosol	A > G
chr19:3540698:T:C:intronic:C19orf	chr19	3540698	3540698	T	C	intronic	C19orf71,MF	.	.	.	A	G	-	-+	0.25	9,0,3,0	chr19,35406	Cytosol	A > G
chr19:3540712:T:C:intronic:C19orf	chr19	3540712	3540712	T	C	intronic	C19orf71,MF	.	.	.	A	G	-	-+	0.3	7,0,3,0	chr19,35407	Cytosol	A > G
chr19:3540715:T:C:intronic:C19orf	chr19	3540715	3540715	T	C	intronic	C19orf71,MF	.	.	.	A	G	-	-+	0.45454545	6,0,5,0	chr19,35407	Cytosol	A > G
chr19:3540755:T:C:intronic:C19orf	chr19	3540755	3540755	T	C	intronic	C19orf71,MF	.	.	.	A	G	-	-+	0.90909091	1,0,10,0	chr19,35407	Cytosol	A > G
chr19:3540765:T:C:intronic:C19orf	chr19	3540765	3540765	T	C	intronic	C19orf71,MF	.	.	.	A	G	-	-+	0.83333333	2,0,10,0	chr19,35407	Cytosol	A > G
chr19:3540794:T:C:intronic:C19orf	chr19	3540794	3540794	T	C	intronic	C19orf71,MF	.	.	.	A	G	-	-+	0.40909091	13,0,9,0	chr19,35407	Cytosol	A > G
chr19:3540795:T:C:intronic:C19orf	chr19	3540795	3540795	T	C	intronic	C19orf71,MF	.	.	.	A	G	-	-+	0.80952381	4,0,17,0	chr19,35407	Cytosol	A > G
chr19:3540801:T:C:intronic:C19orf	chr19	3540801	3540801	T	C	intronic	C19orf71,MF	.	.	.	A	G	-	-+	0.13636364	19,0,3,0	chr19,35408	Cytosol	A > G
chr19:3541591:T:C:intronic:C19orf	chr19	3541591	3541591	T	C	intronic	C19orf71,MF	.	.	.	A	G	-	-+	0.15384615	11,0,2,0	chr19,35415	Cytosol	A > G
chr19:3541592:T:C:intronic:C19orf	chr19	3541592	3541592	T	C	intronic	C19orf71,MF	.	.	.	A	G	-	-+	0.30769231	9,0,4,0	chr19,35415	Cytosol	A > G
chr19:3541879:T:C:intronic:C19orf	chr19	3541879	3541879	T	C	intronic	C19orf71,MF	.	.	.	A	G	-	-+	0.13333333	13,0,2,0	chr19,35418	Cytosol	A > G
chr19:3541903:T:C:intronic:C19orf	chr19	3541903	3541903	T	C	intronic	C19orf71,MF	.	.	.	A	G	-	-+	0.41666667	7,0,5,0	chr19,35419	Cytosol	A > G
chr19:3541904:T:C:intronic:C19orf	chr19	3541904	3541904	T	C	intronic	C19orf71,MF	.	.	.	A	G	-	-+	0.28571429	10,0,4,0	chr19,35419	Cytosol	A > G
chr19:3541919:T:C:intronic:C19orf	chr19	3541919	3541919	T	C	intronic	C19orf71,MF	.	.	.	A	G	-	-+	0.15384615	11,0,2,0	chr19,35419	Cytosol	A > G
chr19:3541928:T:C:intronic:C19orf	chr19	3541928	3541928	T	C	intronic	C19orf71,MF	.	.	.	A	G	-	-+	0.16	21,0,4,0	chr19,35419	Cytosol	A > G
chr19:3541936:T:C:intronic:C19orf	chr19	3541936	3541936	T	C	intronic	C19orf71,MF	.	.	.	A	G	-	-+	0.22222222	21,0,6,0	chr19,35419	Cytosol	A > G
chr19:3541940:T:C:intronic:C19orf	chr19	3541940	3541940	T	C	intronic	C19orf71,MF	.	.	.	A	G	-	-+	0.12	22,0,3,0	chr19,35419	Cytosol	A > G
chr19:3541987:T:C:intronic:C19orf	chr19	3541987	3541987	T	C	intronic	C19orf71,MF	.	.	.	A	G	-	-+	0.1	27,0,3,0	chr19,35419	Cytosol	A > G
chr19:3542001:T:C:intronic:C19orf	chr19	3542001	3542001	T	C	intronic	C19orf71,MF	.	.	.	A	G	-	-+	0.44827586	16,0,13,0	chr19,35420	Cytosol	A > G
chr19:3542002:T:C:intronic:C19orf	chr19	3542002	3542002	T	C	intronic	C19orf71,MF	.	.	.	A	G	-	-+	0.22580645	24,0,7,0	chr19,35420	Cytosol	A > G

chr19:3542050:T:C:intronic:C19orf	chr19	3542050	3542050	T	C	intronic	C19orf71,MF	.	.	.	A	G	-	-+	0.36363636	14,0,8,0	chr19,35420	Cytosol	A > G
chr19:3542058:T:C:intronic:C19orf	chr19	3542058	3542058	T	C	intronic	C19orf71,MF	.	.	.	A	G	-	-+	0.09090909	20,0,2,0	chr19,35420	Cytosol	A > G
chr19:3542059:T:C:intronic:C19orf	chr19	3542059	3542059	T	C	intronic	C19orf71,MF	.	.	.	A	G	-	-+	0.08333333	22,0,2,0	chr19,35420	Cytosol	A > G
chr19:3542080:T:C:intronic:C19orf	chr19	3542080	3542080	T	C	intronic	C19orf71,MF	.	.	.	A	G	-	-+	0.34883721	28,0,15,0	chr19,35420	Cytosol	A > G
chr19:3542081:T:C:intronic:C19orf	chr19	3542081	3542081	T	C	intronic	C19orf71,MF	.	.	.	A	G	-	-+	0.36585366	26,0,15,0	chr19,35420	Cytosol	A > G
chr19:3542097:T:C:intronic:C19orf	chr19	3542097	3542097	T	C	intronic	C19orf71,MF	.	.	.	A	G	-	-+	0.07142857	39,0,3,0	chr19,35420	Cytosol	A > G
chr19:3542545:T:C:intronic:C19orf	chr19	3542545	3542545	T	C	intronic	C19orf71,MF	.	.	.	A	G	-	-+	0.26829268	60,0,22,0	chr19,35425	Cytosol	A > G
chr19:3542596:T:C:intronic:C19orf	chr19	3542596	3542596	T	C	intronic	C19orf71,MF	.	.	.	A	G	-	-+	0.29787234	33,0,14,0	chr19,35425	Cytosol	A > G
chr19:3758349:T:C:intronic:APBA3	chr19	3758349	3758349	T	C	intronic	APBA3	.	.	.	A	G	-	+-	0.18181818	9,0,2,0	chr19,37583	Cytosol	A > G
chr19:3758395:T:C:intronic:APBA3	chr19	3758395	3758395	T	C	intronic	APBA3	.	.	.	A	G	-	+-	0.7	3,0,7,0	chr19,37583	Cytosol	A > G
chr19:3758536:T:C:intronic:APBA3	chr19	3758536	3758536	T	C	intronic	APBA3	.	.	.	A	G	-	+-	0.28571429	10,0,4,0	chr19,37585	Cytosol	A > G
chr19:3759151:T:C:intronic:APBA3	chr19	3759151	3759151	T	C	intronic	APBA3	.	.	.	A	G	-	+-	0.41666667	7,0,5,0	chr19,37591	Cytosol	A > G
chr19:4653139:A:G:UTR3:TNFAIP8	chr19	4653139	4653139	A	G	UTR3	TNFAIP8L1	ENST000003	.	.	A	G	+	+-	0.22727273	17,0,5,0	chr19,46531	Cytosol	A > G
chr19:4653238:A:G:UTR3:TNFAIP8	chr19	4653238	4653238	A	G	UTR3	TNFAIP8L1	ENST000003	.	.	A	G	+	+-	0.57142857	3,0,4,0	chr19,46532	Cytosol	A > G
chr19:4653300:A:G:UTR3:TNFAIP8	chr19	4653300	4653300	A	G	UTR3	TNFAIP8L1	ENST000003	.	.	A	G	+	+-	0.14285714	18,0,3,0	chr19,46532	Cytosol	A > G
chr19:4653399:A:G:UTR3:TNFAIP8	chr19	4653399	4653399	A	G	UTR3	TNFAIP8L1	ENST000003	.	.	A	G	+	+-	0.2	8,0,2,0	chr19,46533	Cytosol	A > G
chr19:4653426:A:G:UTR3:TNFAIP8	chr19	4653426	4653426	A	G	UTR3	TNFAIP8L1	ENST000003	.	.	A	G	+	+-	0.4	9,0,6,0	chr19,46534	Cytosol	A > G
chr19:4653427:A:G:UTR3:TNFAIP8	chr19	4653427	4653427	A	G	UTR3	TNFAIP8L1	ENST000003	.	.	A	G	+	+-	0.26666667	11,0,4,0	chr19,46534	Cytosol	A > G
chr19:4653673:A:G:UTR3:TNFAIP8	chr19	4653673	4653673	A	G	UTR3	TNFAIP8L1	ENST000003	.	.	A	G	+	+-	0.68181818	7,0,15,0	chr19,46536	Cytosol	A > G
chr19:4654380:A:G:UTR3:TNFAIP8	chr19	4654380	4654380	A	G	UTR3	TNFAIP8L1	ENST000003	.	.	A	G	+	+-	0.6	6,0,9,0	chr19,46543	Cytosol	A > G
chr19:4654381:A:G:UTR3:TNFAIP8	chr19	4654381	4654381	A	G	UTR3	TNFAIP8L1	ENST000003	.	.	A	G	+	+-	0.6	6,0,9,0	chr19,46543	Cytosol	A > G
chr19:4654420:A:G:UTR3:TNFAIP8	chr19	4654420	4654420	A	G	UTR3	TNFAIP8L1	ENST000003	.	.	A	G	+	+-	0.07142857	26,0,2,0	chr19,46544	Cytosol	A > G
chr19:4654468:A:G:UTR3:TNFAIP8	chr19	4654468	4654468	A	G	UTR3	TNFAIP8L1	ENST000003	.	.	A	G	+	+-	0.44444444	10,0,8,0	chr19,46544	Cytosol	A > G
chr19:4654473:A:G:UTR3:TNFAIP8	chr19	4654473	4654473	A	G	UTR3	TNFAIP8L1	ENST000003	.	.	A	G	+	+-	0.21052632	15,0,4,0	chr19,46544	Cytosol	A > G
chr19:4654511:A:G:UTR3:TNFAIP8	chr19	4654511	4654511	A	G	UTR3	TNFAIP8L1	ENST000003	.	.	A	G	+	+-	0.06896552	27,0,2,0	chr19,46545	Cytosol	A > G
chr19:4654537:A:G:UTR3:TNFAIP8	chr19	4654537	4654537	A	G	UTR3	TNFAIP8L1	ENST000003	.	.	A	G	+	+-	0.17857143	23,0,5,0	chr19,46545	Cytosol	A > G
chr19:4654551:A:G:UTR3:TNFAIP8	chr19	4654551	4654551	A	G	UTR3	TNFAIP8L1	ENST000003	.	.	A	G	+	+-	0.09677419	28,0,3,0	chr19,46545	Cytosol	A > G
chr19:4654554:A:G:UTR3:TNFAIP8	chr19	4654554	4654554	A	G	UTR3	TNFAIP8L1	ENST000003	.	.	A	G	+	+-	0.16216216	31,0,6,0	chr19,46545	Cytosol	A > G
chr19:4654597:A:G:UTR3:TNFAIP8	chr19	4654597	4654597	A	G	UTR3	TNFAIP8L1	ENST000003	.	.	A	G	+	+-	0.14893617	40,0,7,0	chr19,46545	Cytosol	A > G
chr19:4654601:A:G:UTR3:TNFAIP8	chr19	4654601	4654601	A	G	UTR3	TNFAIP8L1	ENST000003	.	.	A	G	+	+-	0.2	36,0,9,0	chr19,46546	Cytosol	A > G
chr19:5709308:T:C:intronic:LONP1	chr19	5709308	5709308	T	C	intronic	LONP1	.	.	.	A	G	-	-	0.33333333	4,0,2,0	chr19,57093	Cytosol	A > G
chr19:5710925:T:C:intronic:LONP1	chr19	5710925	5710925	T	C	intronic	LONP1	.	.	.	A	G	-	-	0.22222222	7,0,2,0	chr19,57109	Cytosol	A > G
chr19:5710968:T:C:intronic:LONP1	chr19	5710968	5710968	T	C	intronic	LONP1	.	.	.	A	G	-	-	0.33333333	4,0,2,0	chr19,57109	Cytosol	A > G
chr19:6379046:T:C:UTR5:PSPN	chr19	6379046	6379046	T	C	UTR5	PSPN	ENST000005	.	.	A	G	-	-	0.5	8,0,8,0	chr19,63790	Cytosol	A > G
chr19:6379682:T:C:UTR3:GTF2F1	chr19	6379682	6379682	T	C	UTR3	GTF2F1	ENST000003	.	.	A	G	-	-	0.04237288	113,0,5,0	chr19,63796	Cytosol	A > G
chr19:6583633:T:C:intronic:CD70	chr19	6583633	6583633	T	C	intronic	CD70	.	.	.	A	G	-	-	0.15384615	11,0,2,0	chr19,65836	Cytosol	A > G
chr19:6583707:T:C:intronic:CD70	chr19	6583707	6583707	T	C	intronic	CD70	.	.	.	A	G	-	-	0.07692308	24,0,2,0	chr19,65837	Cytosol	A > G
chr19:6583734:T:C:intronic:CD70	chr19	6583734	6583734	T	C	intronic	CD70	.	.	.	A	G	-	-	0.11538462	23,0,3,0	chr19,65837	Cytosol	A > G
chr19:6583872:T:C:intronic:CD70	chr19	6583872	6583872	T	C	intronic	CD70	.	.	.	A	G	-	-	0.29411765	12,0,5,0	chr19,65838	Cytosol	A > G
chr19:6583902:T:C:intronic:CD70	chr19	6583902	6583902	T	C	intronic	CD70	.	.	.	A	G	-	-	0.16666667	10,0,2,0	chr19,65839	Cytosol	A > G
chr19:6583941:T:C:intronic:CD70	chr19	6583941	6583941	T	C	intronic	CD70	.	.	.	A	G	-	-	0.71428571	2,0,5,0	chr19,65839	Cytosol	A > G
chr19:6583942:T:C:intronic:CD70	chr19	6583942	6583942	T	C	intronic	CD70	.	.	.	A	G	-	-	0.5	3,0,3,0	chr19,65839	Cytosol	A > G
chr19:6584077:T:C:intronic:CD70	chr19	6584077	6584077	T	C	intronic	CD70	.	.	.	A	G	-	-	0.6	2,0,3,0	chr19,65840	Cytosol	A > G
chr19:6584396:T:C:intronic:CD70	chr19	6584396	6584396	T	C	intronic	CD70	.	.	.	A	G	-	-	0.63636364	4,0,7,0	chr19,65843	Cytosol	A > G
chr19:6584430:T:C:intronic:CD70	chr19	6584430	6584430	T	C	intronic	CD70	.	.	.	A	G	-	-	0.125	14,0,2,0	chr19,65844	Cytosol	A > G
chr19:6584433:T:C:intronic:CD70	chr19	6584433	6584433	T	C	intronic	CD70	.	.	.	A	G	-	-	0.64285714	5,0,9,0	chr19,65844	Cytosol	A > G
chr19:6584434:T:C:intronic:CD70	chr19	6584434	6584434	T	C	intronic	CD70	.	.	.	A	G	-	-	0.53333333	7,0,8,0	chr19,65844	Cytosol	A > G
chr19:6584440:T:C:intronic:CD70	chr19	6584440	6584440	T	C	intronic	CD70	.	.	.	A	G	-	-	0.21052632	15,0,4,0	chr19,65844	Cytosol	A > G
chr19:6584471:T:C:intronic:CD70	chr19	6584471	6584471	T	C	intronic	CD70	.	.	.	A	G	-	-	0.57142857	6,0,8,0	chr19,65844	Cytosol	A > G
chr19:6584626:T:C:intronic:CD70	chr19	6584626	6584626	T	C	intronic	CD70	.	.	.	A	G	-	-	0.28571429	5,0,2,0	chr19,65846	Cytosol	A > G
chr19:6584691:T:C:intronic:CD70	chr19	6584691	6584691	T	C	intronic	CD70	.	.	.	A	G	-	-	0.3	7,0,3,0	chr19,65846	Cytosol	A > G
chr19:6585065:T:C:intronic:CD70	chr19	6585065	6585065	T	C	intronic	CD70	.	.	.	A	G	-	-	0.14285714	12,0,2,0	chr19,65850	Cytosol	A > G
chr19:6585471:T:C:intronic:CD70	chr19	6585471	6585471	T	C	intronic	CD70	.	.	.	A	G	-	-	0.2	8,0,2,0	chr19,65854	Cytosol	A > G
chr19:6585719:T:C:intronic:CD70	chr19	6585719	6585719	T	C	intronic	CD70	.	.	.	A	G	-	-	0.10526316	17,0,2,0	chr19,65857	Cytosol	A > G
chr19:6585783:T:C:intronic:CD70	chr19	6585783	6585783	T	C	intronic	CD70	.	.	.	A	G	-	-	0.33333333	6,0,3,0	chr19,65857	Cytosol	A > G
chr19:6585823:T:C:intronic:CD70	chr19	6585823	6585823	T	C	intronic	CD70	.	.	.	A	G	-	-	0.4	3,0,2,0	chr19,65858	Cytosol	A > G
chr19:7084380:A:G:UTR3:ZNF557	chr19	7084380	7084380	A	G	UTR3	ZNF557	ENST000004	.	.	A	G	+	+	0.33333333	6,0,3,0	chr19,70843	Cytosol	A > G
chr19:7084393:A:G:UTR3:ZNF557	chr19	7084393	7084393	A	G	UTR3	ZNF557	ENST000004	.	.	A	G	+	+	0.5	4,0,4,0	chr19,70843	Cytosol	A > G
chr19:7084465:A:G:UTR3:ZNF557	chr19	7084465	7084465	A	G	UTR3	ZNF557	ENST000004	.	.	A	G	+	+	0.28571429	5,0,2,0	chr19,70844	Cytosol	A > G
chr19:7085277:A:G:UTR3:ZNF557	chr19	7085277	7085277	A	G	UTR3	ZNF557	ENST000004	.	.	A	G	+	+	0.25	6,0,2,0	chr19,70852	Cytosol	A > G
chr19:7086421:A:G:UTR3:ZNF557	chr19	7086421	7086421	A	G	UTR3	ZNF557	ENST000004	.	.	A	G	+	+	0.5	3,0,3,0	chr19,70864	Cytosol	A > G
chr19:7086512:A:G:UTR3:ZNF557	chr19	7086512	7086512	A	G	UTR3	ZNF557	ENST000004	.	.	A	G	+	+	0.4	3,0,2,0	chr19,70865	Cytosol	A > G
chr19:7086557:A:G:UTR3:ZNF557	chr19	7086557	7086557	A	G	UTR3	ZNF557	ENST000004	.	.	A	G	+	+	0.4	3,0,2,0	chr19,70865	Cytosol	A > G
chr19:7086850:A:G:UTR3:ZNF557	chr19	7086850	7086850	A	G	UTR3	ZNF557	ENST000004	.	.	A	G	+	+	0.66666667	2,0,4,0	chr19,70868	Cytosol	A > G

chr19:7086851:A:G:UTR3:ZNF557	chr19	7086851	7086851	A	G	UTR3	ZNF557	ENST000004	.	.	A	G	+	+	0.33333333	4,0,2,0	chr19,70868	Cytosol	A > G
chr19:7086920:A:G:UTR3:ZNF557	chr19	7086920	7086920	A	G	UTR3	ZNF557	ENST000004	.	.	A	G	+	+	0.8	1,0,4,0	chr19,70869	Cytosol	A > G
chr19:8483486:A:G:UTR5:37316	chr19	8483486	8483486	A	G	UTR5	3月-02	ENST000005	.	.	A	G	+	+	0.11111111	16,0,2,0	chr19,84834	Cytosol	A > G
chr19:9672360:T:C:intergenic:ZNF	chr19	9672360	9672360	T	C	intergenic	ZNF426,ZNF1	dist=23057;d	.	.	A	G	-	-	0.06451613	29,0,2,0	chr19,96723	Cytosol	A > G
chr19:9672908:T:C:intergenic:ZNF	chr19	9672908	9672908	T	C	intergenic	ZNF426,ZNF1	dist=23605;d	.	.	A	G	-	-	0.07692308	36,0,3,0	chr19,96729	Cytosol	A > G
chr19:9672919:T:C:intergenic:ZNF	chr19	9672919	9672919	T	C	intergenic	ZNF426,ZNF1	dist=23616;d	.	.	A	G	-	-	0.15151515	28,0,5,0	chr19,96729	Cytosol	A > G
chr19:9672943:T:C:intergenic:ZNF	chr19	9672943	9672943	T	C	intergenic	ZNF426,ZNF1	dist=23640;d	.	.	A	G	-	-	0.125	28,0,4,0	chr19,96729	Cytosol	A > G
chr19:9672979:T:C:intergenic:ZNF	chr19	9672979	9672979	T	C	intergenic	ZNF426,ZNF1	dist=23676;d	.	.	A	G	-	-	0.10526316	17,0,2,0	chr19,96729	Cytosol	A > G
chr19:9673471:T:C:intergenic:ZNF	chr19	9673471	9673471	T	C	intergenic	ZNF426,ZNF1	dist=24168;d	.	.	A	G	-		0.075	37,0,3,0	chr19,96734	Cytosol	A > G
chr19:9673510:T:C:intergenic:ZNF	chr19	9673510	9673510	T	C	intergenic	ZNF426,ZNF1	dist=24207;d	.	.	A	G	-		0.11111111	24,0,3,0	chr19,96735	Cytosol	A > G
chr19:9674351:T:C:intergenic:ZNF	chr19	9674351	9674351	T	C	intergenic	ZNF426,ZNF1	dist=25048;d	.	.	A	G	-		0.075	37,0,3,0	chr19,96743	Cytosol	A > G
chr19:9761462:T:C:UTR3:ZNF562	chr19	9761462	9761462	T	C	UTR3	ZNF562	ENST000002	.	.	A	G	-	-	0.26315789	14,0,5,0	chr19,97614	Cytosol	A > G
chr19:11040806:A:G:UTR3:C19orf	chr19	11040806	11040806	A	G	UTR3	C19orf52	ENST000002	.	.	A	G	+	+	0.11188811	127,0,16,0	chr19,11040	Cytosol	A > G
chr19:11043739:A:G:intronic:C19	chr19	11043739	11043739	A	G	intronic	C19orf52	.	.	.	A	G	+	+	0.25	9,0,3,0	chr19,11043	Cytosol	A > G
chr19:11256689:T:C:UTR3:SPC24	chr19	11256689	11256689	T	C	UTR3	SPC24	ENST000005	.	.	A	G	-	-	0.25543478	137,0,47,0	chr19,11256	Cytosol	A > G
chr19:11256725:T:C:UTR3:SPC24	chr19	11256725	11256725	T	C	UTR3	SPC24	ENST000005	.	.	A	G	-	-	0.31818182	90,0,42,0	chr19,11256	Cytosol	A > G
chr19:11257185:T:C:UTR3:SPC24	chr19	11257185	11257185	T	C	UTR3	SPC24	ENST000005	.	.	A	G	-	-	0.3877551	30,0,19,0	chr19,11257	Cytosol	A > G
chr19:11257509:T:C:UTR3:SPC24	chr19	11257509	11257509	T	C	UTR3	SPC24	ENST000005	.	.	A	G	-	-	0.05479452	138,0,8,0	chr19,11257	Cytosol	A > G
chr19:11257634:T:C:UTR3:SPC24	chr19	11257634	11257634	T	C	UTR3	SPC24	ENST000005	.	.	A	G	-	-	0.22619048	65,0,19,0	chr19,11257	Cytosol	A > G
chr19:11257644:T:C:UTR3:SPC24	chr19	11257644	11257644	T	C	UTR3	SPC24	ENST000005	.	.	A	G	-	-	0.1547619	71,0,13,0	chr19,11257	Cytosol	A > G
chr19:11257704:T:C:UTR3:SPC24	chr19	11257704	11257704	T	C	UTR3	SPC24	ENST000005	.	.	A	G	-	-	0.45132743	62,0,51,0	chr19,11257	Cytosol	A > G
chr19:11257709:T:C:UTR3:SPC24	chr19	11257709	11257709	T	C	UTR3	SPC24	ENST000005	.	.	A	G	-	-	0.23364486	82,0,25,0	chr19,11257	Cytosol	A > G
chr19:11257717:T:C:UTR3:SPC24	chr19	11257717	11257717	T	C	UTR3	SPC24	ENST000005	.	.	A	G	-	-	0.27192982	81,0,31,2	chr19,11257	Cytosol	A > G
chr19:11560427:A:G:UTR3:PRKCSI	chr19	11560427	11560427	A	G	UTR3	PRKCSH	ENST000005	.	.	A	G	+	+	0.65789474	39,0,75,0	chr19,11560	Cytosol	A > G
chr19:11560461:A:G:UTR3:PRKCSI	chr19	11560461	11560461	A	G	UTR3	PRKCSH	ENST000005	.	.	A	G	+	+	0.06315789	89,0,6,0	chr19,11560	Cytosol	A > G
chr19:11560483:A:G:UTR3:PRKCSI	chr19	11560483	11560483	A	G	UTR3	PRKCSH	ENST000005	.	.	A	G	+	+	0.02439024	80,0,2,0	chr19,11560	Cytosol	A > G
chr19:11560530:A:G:UTR3:PRKCSI	chr19	11560530	11560530	A	G	UTR3	PRKCSH	ENST000005	.	.	A	G	+	+	0.08139535	79,0,7,0	chr19,11560	Cytosol	A > G
chr19:11560533:A:G:UTR3:PRKCSI	chr19	11560533	11560533	A	G	UTR3	PRKCSH	ENST000005	.	.	A	G	+	+	0.12195122	72,0,10,0	chr19,11560	Cytosol	A > G
chr19:11560535:A:G:UTR3:PRKCSI	chr19	11560535	11560535	A	G	UTR3	PRKCSH	ENST000005	.	.	A	G	+	+	0.02702703	72,0,2,0	chr19,11560	Cytosol	A > G
chr19:11560584:A:G:UTR3:PRKCSI	chr19	11560584	11560584	A	G	UTR3	PRKCSH	ENST000005	.	.	A	G	+	+	0.02105263	93,0,2,0	chr19,11560	Cytosol	A > G
chr19:11560716:A:G:intronic:PRK	chr19	11560716	11560716	A	G	intronic	PRKCSH	.	.	.	A	G	+	+	0.64835165	32,0,59,0	chr19,11560	Cytosol	A > G
chr19:11560763:A:G:intronic:PRK	chr19	11560763	11560763	A	G	intronic	PRKCSH	.	.	.	A	G	+	+	0.79245283	22,0,84,0	chr19,11560	Cytosol	A > G
chr19:11560825:A:G:intronic:PRK	chr19	11560825	11560825	A	G	intronic	PRKCSH	.	.	.	A	G	+	+	0.44186047	24,0,19,0	chr19,11560	Cytosol	A > G
chr19:11560835:A:G:intronic:PRK	chr19	11560835	11560835	A	G	intronic	PRKCSH	.	.	.	A	G	+	+	0.27027027	27,0,10,0	chr19,11560	Cytosol	A > G
chr19:11560841:A:G:intronic:PRK	chr19	11560841	11560841	A	G	intronic	PRKCSH	.	.	.	A	G	+	+	0.18181818	27,0,6,0	chr19,11560	Cytosol	A > G
chr19:11560866:A:G:intronic:PRK	chr19	11560866	11560866	A	G	intronic	PRKCSH	.	.	.	A	G	+	+	0.45454545	6,0,5,0	chr19,11560	Cytosol	A > G
chr19:11560911:A:G:intronic:PRK	chr19	11560911	11560911	A	G	intronic	PRKCSH	.	.	.	A	G	+	+	0.30769231	9,0,4,0	chr19,11560	Cytosol	A > G
chr19:11560921:A:G:intronic:PRK	chr19	11560921	11560921	A	G	intronic	PRKCSH	.	.	.	A	G	+	+	0.66666667	5,0,10,0	chr19,11560	Cytosol	A > G
chr19:11561032:A:G:intronic:PRK	chr19	11561032	11561032	A	G	intronic	PRKCSH	.	.	.	A	G	+	+	1	0,0,5,0	chr19,11561	Cytosol	A > G
chr19:11561033:A:G:intronic:PRK	chr19	11561033	11561033	A	G	intronic	PRKCSH	.	.	.	A	G	+	+	0.4	3,0,2,0	chr19,11561	Cytosol	A > G
chr19:11561078:A:G:intronic:PRK	chr19	11561078	11561078	A	G	intronic	PRKCSH	.	.	.	A	G	+	+	0.3125	11,0,5,0	chr19,11561	Cytosol	A > G
chr19:11561101:A:G:intronic:PRK	chr19	11561101	11561101	A	G	intronic	PRKCSH	.	.	.	A	G	+	+	0.65384615	9,0,17,0	chr19,11561	Cytosol	A > G
chr19:11561102:A:G:intronic:PRK	chr19	11561102	11561102	A	G	intronic	PRKCSH	.	.	.	A	G	+	+	0.81481481	5,0,22,0	chr19,11561	Cytosol	A > G
chr19:11561138:A:G:intronic:PRK	chr19	11561138	11561138	A	G	intronic	PRKCSH	.	.	.	A	G	+	+	0.2173913	18,0,5,0	chr19,11561	Cytosol	A > G
chr19:11561139:A:G:intronic:PRK	chr19	11561139	11561139	A	G	intronic	PRKCSH	.	.	.	A	G	+	+	0.66666667	8,0,16,0	chr19,11561	Cytosol	A > G
chr19:11561140:A:G:intronic:PRK	chr19	11561140	11561140	A	G	intronic	PRKCSH	.	.	.	A	G	+	+	0.38095238	13,0,8,0	chr19,11561	Cytosol	A > G
chr19:11561141:A:G:intronic:PRK	chr19	11561141	11561141	A	G	intronic	PRKCSH	.	.	.	A	G	+	+	0.45454545	12,0,10,0	chr19,11561	Cytosol	A > G
chr19:11561142:A:G:intronic:PRK	chr19	11561142	11561142	A	G	intronic	PRKCSH	.	.	.	A	G	+	+	0.43478261	13,0,10,0	chr19,11561	Cytosol	A > G
chr19:11561145:A:G:intronic:PRK	chr19	11561145	11561145	A	G	intronic	PRKCSH	.	.	.	A	G	+	+	0.38461538	16,0,10,0	chr19,11561	Cytosol	A > G
chr19:11561161:A:G:intronic:PRK	chr19	11561161	11561161	A	G	intronic	PRKCSH	.	.	.	A	G	+	+	0.42307692	15,0,11,0	chr19,11561	Cytosol	A > G
chr19:11561171:A:G:intronic:PRK	chr19	11561171	11561171	A	G	intronic	PRKCSH	.	.	.	A	G	+	+	0.7027027	11,0,26,0	chr19,11561	Cytosol	A > G
chr19:11561186:A:G:intronic:PRK	chr19	11561186	11561186	A	G	intronic	PRKCSH	.	.	.	A	G	+	+	0.51111111	22,0,23,0	chr19,11561	Cytosol	A > G
chr19:11561207:A:G:intronic:PRK	chr19	11561207	11561207	A	G	intronic	PRKCSH	.	.	.	A	G	+	+	0.56818182	19,0,25,0	chr19,11561	Cytosol	A > G
chr19:11561217:A:G:intronic:PRK	chr19	11561217	11561217	A	G	intronic	PRKCSH	.	.	.	A	G	+	+	0.14814815	46,0,8,0	chr19,11561	Cytosol	A > G
chr19:11561224:A:G:intronic:PRK	chr19	11561224	11561224	A	G	intronic	PRKCSH	.	.	.	A	G	+	+	0.90909091	5,0,50,0	chr19,11561	Cytosol	A > G
chr19:11561241:A:G:intronic:PRK	chr19	11561241	11561241	A	G	intronic	PRKCSH	.	.	.	A	G	+	+	0.36363636	28,0,16,0	chr19,11561	Cytosol	A > G
chr19:11561242:A:G:splicing:PRK	chr19	11561242	11561242	A	G	splicing	PRKCSH	ENST000005	.	.	A	G	+	+	0.04255319	45,0,2,0	chr19,11561	Cytosol	A > G
chr19:11561359:A:G:UTR3:PRKCSI	chr19	11561359	11561359	A	G	UTR3	PRKCSH	ENST000005	.	.	A	G	+	+	0.06837607	109,0,8,0	chr19,11561	Cytosol	A > G
chr19:11944968:A:G:UTR3:ZNF44	chr19	11944968	11944968	A	G	UTR3	ZNF440	ENST000003	.	.	A	G	+	+	0.66666667	2,0,4,0	chr19,11944	Cytosol	A > G
chr19:11945003:A:G:UTR3:ZNF44	chr19	11945003	11945003	A	G	UTR3	ZNF440	ENST000003	.	.	A	G	+	+	0.57142857	3,0,4,0	chr19,11945	Cytosol	A > G
chr19:11945011:A:G:UTR3:ZNF44	chr19	11945011	11945011	A	G	UTR3	ZNF440	ENST000003	.	.	A	G	+	+	0.83333333	1,0,5,0	chr19,11945	Cytosol	A > G
chr19:11945117:A:G:UTR3:ZNF44	chr19	11945117	11945117	A	G	UTR3	ZNF440	ENST000003	.	.	A	G	+	+	0.21428571	11,0,3,0	chr19,11945	Cytosol	A > G
chr19:11945124:A:G:UTR3:ZNF44	chr19	11945124	11945124	A	G	UTR3	ZNF440	ENST000003	.	.	A	G	+	+	0.25	9,0,3,0	chr19,11945	Cytosol	A > G
chr19:11945127:A:G:UTR3:ZNF44	chr19	11945127	11945127	A	G	UTR3	ZNF440	ENST000003	.	.	A	G	+	+	0.4	6,0,4,0	chr19,11945	Cytosol	A > G

chr19:11945146:A:G:UTR3:ZNF44	chr19	11945146	11945146	A	G	UTR3	ZNF440	ENST000003	.	.	A	G	+	+	0.4	3,0,2,0	chr19,11945	Cytosol	A > G
chr19:11945216:A:G:UTR3:ZNF44	chr19	11945216	11945216	A	G	UTR3	ZNF440	ENST000003	.	.	A	G	+	+	0.39130435	14,0,9,0	chr19,11945	Cytosol	A > G
chr19:11945225:A:G:UTR3:ZNF44	chr19	11945225	11945225	A	G	UTR3	ZNF440	ENST000003	.	.	A	G	+	+	0.1	18,0,2,0	chr19,11945	Cytosol	A > G
chr19:11945707:A:G:UTR3:ZNF44	chr19	11945707	11945707	A	G	UTR3	ZNF440	ENST000003	.	.	A	G	+	+	0.5	3,0,3,0	chr19,11945	Cytosol	A > G
chr19:11945748:A:G:UTR3:ZNF44	chr19	11945748	11945748	A	G	UTR3	ZNF440	ENST000003	.	.	A	G	+	+	0.13333333	13,0,2,0	chr19,11945	Cytosol	A > G
chr19:11945749:A:G:UTR3:ZNF44	chr19	11945749	11945749	A	G	UTR3	ZNF440	ENST000003	.	.	A	G	+	+	0.53333333	6,0,8,1	chr19,11945	Cytosol	A > G
chr19:11945798:A:G:UTR3:ZNF44	chr19	11945798	11945798	A	G	UTR3	ZNF440	ENST000003	.	.	A	G	+	+	0.15384615	11,0,2,0	chr19,11945	Cytosol	A > G
chr19:11945923:A:G:UTR3:ZNF44	chr19	11945923	11945923	A	G	UTR3	ZNF440	ENST000003	.	.	A	G	+	+	0.36363636	7,0,4,0	chr19,11945	Cytosol	A > G
chr19:11946002:A:G:UTR3:ZNF44	chr19	11946002	11946002	A	G	UTR3	ZNF440	ENST000003	.	.	A	G	+	+	0.28571429	5,0,2,0	chr19,11946	Cytosol	A > G
chr19:11946003:A:G:UTR3:ZNF44	chr19	11946003	11946003	A	G	UTR3	ZNF440	ENST000003	.	.	A	G	+	+	0.28571429	5,0,2,0	chr19,11946	Cytosol	A > G
chr19:12743409:A:G:downstream:	chr19	12743409	12743409	A	G	downstream	ZNF791	.	.	.	A	G	+	-	0.5	3,0,3,0	chr19,12743	Cytosol	A > G
chr19:12743410:A:G:downstream:	chr19	12743410	12743410	A	G	downstream	ZNF791	.	.	.	A	G	+	-	0.5	4,0,4,0	chr19,12743	Cytosol	A > G
chr19:12743476:A:G:downstream:	chr19	12743476	12743476	A	G	downstream	ZNF791	.	.	.	A	G	+	-	0.25	6,0,2,0	chr19,12743	Cytosol	A > G
chr19:13878243:A:G:intronic:MRI	chr19	13878243	13878243	A	G	intronic	MRI1	.	.	.	A	G	+	+	0.28571429	5,0,2,0	chr19,13878	Cytosol	A > G
chr19:13883381:A:G:ncRNA_exoni	chr19	13883381	13883381	A	G	ncRNA_exon	CTB-5E10.3	.	.	.	A	G	+	+-	0.81896552	21,0,95,0	chr19,13883	Cytosol	A > G
chr19:13883387:A:G:ncRNA_exoni	chr19	13883387	13883387	A	G	ncRNA_exon	CTB-5E10.3	.	.	.	A	G	+	+-	0.22307692	101,0,29,0	chr19,13883	Cytosol	A > G
chr19:13883388:A:G:ncRNA_exoni	chr19	13883388	13883388	A	G	ncRNA_exon	CTB-5E10.3	.	.	.	A	G	+	+-	0.515625	62,0,66,0	chr19,13883	Cytosol	A > G
chr19:13883426:A:G:ncRNA_exoni	chr19	13883426	13883426	A	G	ncRNA_exon	CTB-5E10.3	.	.	.	A	G	+	+-	0.02255639	130,0,3,0	chr19,13883	Cytosol	A > G
chr19:13883431:A:G:ncRNA_exoni	chr19	13883431	13883431	A	G	ncRNA_exon	CTB-5E10.3	.	.	.	A	G	+	+-	0.23287671	112,0,34,0	chr19,13883	Cytosol	A > G
chr19:13883434:A:G:ncRNA_exoni	chr19	13883434	13883434	A	G	ncRNA_exon	CTB-5E10.3	.	.	.	A	G	+	+-	0.11464968	139,0,18,0	chr19,13883	Cytosol	A > G
chr19:13883476:A:G:ncRNA_intro	chr19	13883476	13883476	A	G	ncRNA_intro	CTB-5E10.3	.	.	.	A	G	+	+-	0.36134454	76,0,43,0	chr19,13883	Cytosol	A > G
chr19:13883479:A:G:ncRNA_intro	chr19	13883479	13883479	A	G	ncRNA_intro	CTB-5E10.3	.	.	.	A	G	+	+-	0.54545455	55,0,66,0	chr19,13883	Cytosol	A > G
chr19:13883480:A:G:ncRNA_intro	chr19	13883480	13883480	A	G	ncRNA_intro	CTB-5E10.3	.	.	.	A	G	+	+-	0.04065041	118,0,5,0	chr19,13883	Cytosol	A > G
chr19:13883481:A:G:ncRNA_intro	chr19	13883481	13883481	A	G	ncRNA_intro	CTB-5E10.3	.	.	.	A	G	+	+-	0.04424779	108,0,5,0	chr19,13883	Cytosol	A > G
chr19:13883482:A:G:ncRNA_intro	chr19	13883482	13883482	A	G	ncRNA_intro	CTB-5E10.3	.	.	.	A	G	+	+-	0.09322034	107,0,11,0	chr19,13883	Cytosol	A > G
chr19:13883485:A:G:ncRNA_intro	chr19	13883485	13883485	A	G	ncRNA_intro	CTB-5E10.3	.	.	.	A	G	+	+-	0.06956522	107,0,8,0	chr19,13883	Cytosol	A > G
chr19:13883488:A:G:ncRNA_intro	chr19	13883488	13883488	A	G	ncRNA_intro	CTB-5E10.3	.	.	.	A	G	+	+-	0.04201681	114,0,5,0	chr19,13883	Cytosol	A > G
chr19:13883492:A:G:ncRNA_intro	chr19	13883492	13883492	A	G	ncRNA_intro	CTB-5E10.3	.	.	.	A	G	+	+-	0.1	108,0,12,0	chr19,13883	Cytosol	A > G
chr19:13883495:A:G:ncRNA_intro	chr19	13883495	13883495	A	G	ncRNA_intro	CTB-5E10.3	.	.	.	A	G	+	+-	0.21	79,0,21,0	chr19,13883	Cytosol	A > G
chr19:13883521:A:G:ncRNA_intro	chr19	13883521	13883521	A	G	ncRNA_intro	CTB-5E10.3	.	.	.	A	G	+	+-	0.56756757	32,0,42,0	chr19,13883	Cytosol	A > G
chr19:13883531:A:G:ncRNA_intro	chr19	13883531	13883531	A	G	ncRNA_intro	CTB-5E10.3	.	.	.	A	G	+	+-	0.31884058	47,0,22,0	chr19,13883	Cytosol	A > G
chr19:13883548:A:G:ncRNA_intro	chr19	13883548	13883548	A	G	ncRNA_intro	CTB-5E10.3	.	.	.	A	G	+	+-	0.45098039	28,0,23,0	chr19,13883	Cytosol	A > G
chr19:13883554:A:G:ncRNA_intro	chr19	13883554	13883554	A	G	ncRNA_intro	CTB-5E10.3	.	.	.	A	G	+	+-	0.2	32,0,8,0	chr19,13883	Cytosol	A > G
chr19:13883562:A:G:ncRNA_intro	chr19	13883562	13883562	A	G	ncRNA_intro	CTB-5E10.3	.	.	.	A	G	+	+-	0.09756098	37,0,4,0	chr19,13883	Cytosol	A > G
chr19:13883574:A:G:ncRNA_intro	chr19	13883574	13883574	A	G	ncRNA_intro	CTB-5E10.3	.	.	.	A	G	+	+-	0.2	24,0,6,0	chr19,13883	Cytosol	A > G
chr19:13883596:A:G:ncRNA_intro	chr19	13883596	13883596	A	G	ncRNA_intro	CTB-5E10.3	.	.	.	A	G	+	+-	0.13636364	19,0,3,0	chr19,13883	Cytosol	A > G
chr19:13883852:A:G:ncRNA_intro	chr19	13883852	13883852	A	G	ncRNA_intro	CTB-5E10.3	.	.	.	A	G	+	+-	0.02923977	166,0,5,0	chr19,13883	Cytosol	A > G
chr19:13883855:A:G:ncRNA_intro	chr19	13883855	13883855	A	G	ncRNA_intro	CTB-5E10.3	.	.	.	A	G	+	+-	0.12195122	144,0,20,0	chr19,13883	Cytosol	A > G
chr19:13883862:A:G:ncRNA_intro	chr19	13883862	13883862	A	G	ncRNA_intro	CTB-5E10.3	.	.	.	A	G	+	+-	0.23853211	166,0,52,0	chr19,13883	Cytosol	A > G
chr19:13883874:A:G:ncRNA_intro	chr19	13883874	13883874	A	G	ncRNA_intro	CTB-5E10.3	.	.	.	A	G	+	+-	0.18695652	187,0,43,0	chr19,13883	Cytosol	A > G
chr19:13883892:A:G:ncRNA_intro	chr19	13883892	13883892	A	G	ncRNA_intro	CTB-5E10.3	.	.	.	A	G	+	+-	0.71180556	83,0,205,0	chr19,13883	Cytosol	A > G
chr19:13883922:A:G:ncRNA_intro	chr19	13883922	13883922	A	G	ncRNA_intro	CTB-5E10.3	.	.	.	A	G	+	+-	0.01869159	210,0,4,0	chr19,13883	Cytosol	A > G
chr19:13883923:A:G:ncRNA_intro	chr19	13883923	13883923	A	G	ncRNA_intro	CTB-5E10.3	.	.	.	A	G	+	+-	0.10824742	173,0,21,0	chr19,13883	Cytosol	A > G
chr19:13883927:A:G:ncRNA_intro	chr19	13883927	13883927	A	G	ncRNA_intro	CTB-5E10.3	.	.	.	A	G	+	+-	0.19021739	149,0,35,0	chr19,13883	Cytosol	A > G
chr19:13883938:A:G:ncRNA_intro	chr19	13883938	13883938	A	G	ncRNA_intro	CTB-5E10.3	.	.	.	A	G	+	+-	0.08633094	127,0,12,0	chr19,13883	Cytosol	A > G
chr19:13883940:A:G:ncRNA_intro	chr19	13883940	13883940	A	G	ncRNA_intro	CTB-5E10.3	.	.	.	A	G	+	+-	0.05737705	115,0,7,0	chr19,13883	Cytosol	A > G
chr19:13883962:A:G:ncRNA_intro	chr19	13883962	13883962	A	G	ncRNA_intro	CTB-5E10.3	.	.	.	A	G	+	+-	0.1122449	87,0,11,0	chr19,13883	Cytosol	A > G
chr19:13883968:A:G:ncRNA_intro	chr19	13883968	13883968	A	G	ncRNA_intro	CTB-5E10.3	.	.	.	A	G	+	+-	0.12121212	87,0,12,0	chr19,13883	Cytosol	A > G
chr19:13883978:A:G:ncRNA_intro	chr19	13883978	13883978	A	G	ncRNA_intro	CTB-5E10.3	.	.	.	A	G	+	+-	0.03409091	85,0,3,0	chr19,13883	Cytosol	A > G
chr19:13883984:A:G:ncRNA_intro	chr19	13883984	13883984	A	G	ncRNA_intro	CTB-5E10.3	.	.	.	A	G	+	+-	0.14678899	93,0,16,0	chr19,13883	Cytosol	A > G
chr19:13883988:A:G:ncRNA_intro	chr19	13883988	13883988	A	G	ncRNA_intro	CTB-5E10.3	.	.	.	A	G	+	+-	0.18867925	86,0,20,0	chr19,13883	Cytosol	A > G
chr19:13883997:A:G:ncRNA_intro	chr19	13883997	13883997	A	G	ncRNA_intro	CTB-5E10.3	.	.	.	A	G	+	+-	0.01449275	136,0,2,0	chr19,13883	Cytosol	A > G
chr19:13884003:A:G:ncRNA_intro	chr19	13884003	13884003	A	G	ncRNA_intro	CTB-5E10.3	.	.	.	A	G	+	+-	0.12179487	137,0,19,0	chr19,13884	Cytosol	A > G
chr19:13884006:A:G:ncRNA_intro	chr19	13884006	13884006	A	G	ncRNA_intro	CTB-5E10.3	.	.	.	A	G	+	+-	0.234375	98,0,30,0	chr19,13884	Cytosol	A > G
chr19:13884020:A:G:ncRNA_intro	chr19	13884020	13884020	A	G	ncRNA_intro	CTB-5E10.3	.	.	.	A	G	+	+-	0.11392405	140,0,18,0	chr19,13884	Cytosol	A > G
chr19:13884023:A:G:ncRNA_intro	chr19	13884023	13884023	A	G	ncRNA_intro	CTB-5E10.3	.	.	.	A	G	+	+-	0.17751479	139,0,30,0	chr19,13884	Cytosol	A > G
chr19:13884032:A:G:ncRNA_intro	chr19	13884032	13884032	A	G	ncRNA_intro	CTB-5E10.3	.	.	.	A	G	+	+-	0.03267974	148,0,5,0	chr19,13884	Cytosol	A > G
chr19:13884074:A:G:ncRNA_exoni	chr19	13884074	13884074	A	G	ncRNA_exon	CTB-5E10.3	.	.	.	A	G	+	+-	0.51219512	40,0,42,0	chr19,13884	Cytosol	A > G
chr19:13884083:A:G:ncRNA_exoni	chr19	13884083	13884083	A	G	ncRNA_exon	CTB-5E10.3	.	.	.	A	G	+	+-	0.47916667	25,0,23,0	chr19,13884	Cytosol	A > G
chr19:13884084:A:G:ncRNA_exoni	chr19	13884084	13884084	A	G	ncRNA_exon	CTB-5E10.3	.	.	.	A	G	+	+-	0.82	9,0,41,0	chr19,13884	Cytosol	A > G
chr19:13884096:A:G:ncRNA_exoni	chr19	13884096	13884096	A	G	ncRNA_exon	CTB-5E10.3	.	.	.	A	G	+	+-	0.4	33,0,22,0	chr19,13884	Cytosol	A > G
chr19:13884102:A:G:ncRNA_exoni	chr19	13884102	13884102	A	G	ncRNA_exon	CTB-5E10.3	.	.	.	A	G	+	+-	0.2295082	47,0,14,0	chr19,13884	Cytosol	A > G
chr19:13884176:A:G:ncRNA_exoni	chr19	13884176	13884176	A	G	ncRNA_exon	CTB-5E10.3	.	.	.	A	G	+	+-	0.13861386	87,0,14,0	chr19,13884	Cytosol	A > G
chr19:13884198:A:G:ncRNA_exoni	chr19	13884198	13884198	A	G	ncRNA_exon	CTB-5E10.3	.	.	.	A	G	+	+-	0.40123457	97,0,65,0	chr19,13884	Cytosol	A > G

chr19:13884199:A:G:ncRNA_exoni	chr19	13884199	13884199	A	G	ncRNA_exon	CTB-5E10.3	.	.	.	A	G	+	+-	0.4	96,0,64,0	chr19,13884	Cytosol	A > G
chr19:13884259:A:G:ncRNA_exoni	chr19	13884259	13884259	A	G	ncRNA_exon	CTB-5E10.3	.	.	.	A	G	+	+-	0.41921397	133,0,96,0	chr19,13884	Cytosol	A > G
chr19:13884308:A:G:ncRNA_exoni	chr19	13884308	13884308	A	G	ncRNA_exon	CTB-5E10.3	.	.	.	A	G	+	+-	0.02293578	213,0,5,0	chr19,13884	Cytosol	A > G
chr19:13884319:A:G:ncRNA_exoni	chr19	13884319	13884319	A	G	ncRNA_exon	CTB-5E10.3	.	.	.	A	G	+	+-	0.11417323	225,0,29,0	chr19,13884	Cytosol	A > G
chr19:13884325:A:G:ncRNA_exoni	chr19	13884325	13884325	A	G	ncRNA_exon	CTB-5E10.3	.	.	.	A	G	+	+-	0.05761317	229,0,14,0	chr19,13884	Cytosol	A > G
chr19:13884339:A:G:ncRNA_exoni	chr19	13884339	13884339	A	G	ncRNA_exon	CTB-5E10.3	.	.	.	A	G	+	+-	0.1	189,0,21,0	chr19,13884	Cytosol	A > G
chr19:13884345:A:G:ncRNA_exoni	chr19	13884345	13884345	A	G	ncRNA_exon	CTB-5E10.3	.	.	.	A	G	+	+-	0.02727273	214,0,6,0	chr19,13884	Cytosol	A > G
chr19:13884346:A:G:ncRNA_exoni	chr19	13884346	13884346	A	G	ncRNA_exon	CTB-5E10.3	.	.	.	A	G	+	+-	0.82673267	35,0,167,0	chr19,13884	Cytosol	A > G
chr19:13884377:A:G:ncRNA_exoni	chr19	13884377	13884377	A	G	ncRNA_exon	CTB-5E10.3	.	.	.	A	G	+	+-	0.0104712	189,0,2,0	chr19,13884	Cytosol	A > G
chr19:13884384:A:G:ncRNA_exoni	chr19	13884384	13884384	A	G	ncRNA_exon	CTB-5E10.3	.	.	.	A	G	+	+-	0.07236842	141,0,11,0	chr19,13884	Cytosol	A > G
chr19:13884408:A:G:ncRNA_exoni	chr19	13884408	13884408	A	G	ncRNA_exon	CTB-5E10.3	.	.	.	A	G	+	+-	0.62348178	93,0,154,0	chr19,13884	Cytosol	A > G
chr19:13884418:A:G:ncRNA_exoni	chr19	13884418	13884418	A	G	ncRNA_exon	CTB-5E10.3	.	.	.	A	G	+	+-	0.93548387	12,0,174,0	chr19,13884	Cytosol	A > G
chr19:13884419:A:G:ncRNA_exoni	chr19	13884419	13884419	A	G	ncRNA_exon	CTB-5E10.3	.	.	.	A	G	+	+-	0.77314815	49,0,167,0	chr19,13884	Cytosol	A > G
chr19:13884515:A:G:ncRNA_intro	chr19	13884515	13884515	A	G	ncRNA_intro	CTB-5E10.3	.	.	.	A	G	+	+-	0.30769231	18,0,8,0	chr19,13884	Cytosol	A > G
chr19:13884525:A:G:ncRNA_intro	chr19	13884525	13884525	A	G	ncRNA_intro	CTB-5E10.3	.	.	.	A	G	+	+-	1	0,0,33,0	chr19,13884	Cytosol	A > G
chr19:13884536:A:G:ncRNA_intro	chr19	13884536	13884536	A	G	ncRNA_intro	CTB-5E10.3	.	.	.	A	G	+	+-	0.08823529	30,1,3,0	chr19,13884	Cytosol	A > G
chr19:13884541:A:G:ncRNA_intro	chr19	13884541	13884541	A	G	ncRNA_intro	CTB-5E10.3	.	.	.	A	G	+	+-	0.41935484	18,0,13,0	chr19,13884	Cytosol	A > G
chr19:13884552:A:G:ncRNA_intro	chr19	13884552	13884552	A	G	ncRNA_intro	CTB-5E10.3	.	.	.	A	G	+	+-	0.09677419	28,0,3,0	chr19,13884	Cytosol	A > G
chr19:13884572:A:G:ncRNA_intro	chr19	13884572	13884572	A	G	ncRNA_intro	CTB-5E10.3	.	.	.	A	G	+	+-	0.43333333	17,0,13,0	chr19,13884	Cytosol	A > G
chr19:13884601:A:G:ncRNA_intro	chr19	13884601	13884601	A	G	ncRNA_intro	CTB-5E10.3	.	.	.	A	G	+	+-	0.08641975	74,0,7,0	chr19,13884	Cytosol	A > G
chr19:13884611:A:G:ncRNA_intro	chr19	13884611	13884611	A	G	ncRNA_intro	CTB-5E10.3	.	.	.	A	G	+	+-	0.37179487	49,0,29,0	chr19,13884	Cytosol	A > G
chr19:13884613:A:G:ncRNA_intro	chr19	13884613	13884613	A	G	ncRNA_intro	CTB-5E10.3	.	.	.	A	G	+	+-	0.04878049	78,0,4,0	chr19,13884	Cytosol	A > G
chr19:13884669:A:G:ncRNA_exoni	chr19	13884669	13884669	A	G	ncRNA_exon	CTB-5E10.3	.	.	.	A	G	+	+-	0.36507937	40,0,23,0	chr19,13884	Cytosol	A > G
chr19:13884725:A:G:ncRNA_exoni	chr19	13884725	13884725	A	G	ncRNA_exon	CTB-5E10.3	.	.	.	A	G	+	+-	0.17307692	172,0,36,0	chr19,13884	Cytosol	A > G
chr19:13884727:A:G:ncRNA_exoni	chr19	13884727	13884727	A	G	ncRNA_exon	CTB-5E10.3	.	.	.	A	G	+	+-	0.48356808	110,0,103,0	chr19,13884	Cytosol	A > G
chr19:13884739:A:G:ncRNA_exoni	chr19	13884739	13884739	A	G	ncRNA_exon	CTB-5E10.3	.	.	.	A	G	+	+-	0.33678756	128,0,65,0	chr19,13884	Cytosol	A > G
chr19:13884742:A:G:ncRNA_exoni	chr19	13884742	13884742	A	G	ncRNA_exon	CTB-5E10.3	.	.	.	A	G	+	+-	0.28415301	131,0,52,0	chr19,13884	Cytosol	A > G
chr19:14680184:T:C:intronic:NDUI	chr19	14680184	14680184	T	C	intronic	NDUFB7	.	.	.	A	G	-	-	0.28571429	5,0,2,0	chr19,14680	Cytosol	A > G
chr19:14680189:T:C:intronic:NDUI	chr19	14680189	14680189	T	C	intronic	NDUFB7	.	.	.	A	G	-	-	0.33333333	4,0,2,0	chr19,14680	Cytosol	A > G
chr19:14682327:T:C:intronic:NDUI	chr19	14682327	14682327	T	C	intronic	NDUFB7	.	.	.	A	G	-	-	0.2	8,0,2,0	chr19,14682	Cytosol	A > G
chr19:14845123:T:C:UTR3:EMR2	chr19	14845123	14845123	T	C	UTR3	EMR2	ENST000003	.	.	A	G	-	-	0.4	3,0,2,0	chr19,14845	Cytosol	A > G
chr19:16655400:A:G:ncRNA_exoni	chr19	16655400	16655400	A	G	ncRNA_exon	CTD-3222D1	.	.	.	A	G	+	-	0.3	7,0,3,0	chr19,16655	Cytosol	A > G
chr19:16655411:A:G:ncRNA_exoni	chr19	16655411	16655411	A	G	ncRNA_exon	CTD-3222D1	.	.	.	A	G	+	-	0.22222222	7,0,2,0	chr19,16655	Cytosol	A > G
chr19:16661291:T:C:UTR3:SLC35E	chr19	16661291	16661291	T	C	UTR3	SLC35E1	ENST000005	.	.	A	G	-	-	0.67741935	10,0,21,0	chr19,16661	Cytosol	A > G
chr19:16661297:T:C:UTR3:SLC35E	chr19	16661297	16661297	T	C	UTR3	SLC35E1	ENST000005	.	.	A	G	-	-	0.5	16,0,16,0	chr19,16661	Cytosol	A > G
chr19:16661359:T:C:UTR3:SLC35E	chr19	16661359	16661359	T	C	UTR3	SLC35E1	ENST000005	.	.	A	G	-	-	0.64285714	10,0,18,0	chr19,16661	Cytosol	A > G
chr19:16661376:T:C:UTR3:SLC35E	chr19	16661376	16661376	T	C	UTR3	SLC35E1	ENST000005	.	.	A	G	-	-	0.41935484	18,0,13,0	chr19,16661	Cytosol	A > G
chr19:16661379:T:C:UTR3:SLC35E	chr19	16661379	16661379	T	C	UTR3	SLC35E1	ENST000005	.	.	A	G	-	-	0.64705882	12,0,22,0	chr19,16661	Cytosol	A > G
chr19:16661421:T:C:UTR3:SLC35E	chr19	16661421	16661421	T	C	UTR3	SLC35E1	ENST000005	.	.	A	G	-	-	0.6875	10,0,22,0	chr19,16661	Cytosol	A > G
chr19:16661443:T:C:UTR3:SLC35E	chr19	16661443	16661443	T	C	UTR3	SLC35E1	ENST000005	.	.	A	G	-	-	0.17073171	34,0,7,0	chr19,16661	Cytosol	A > G
chr19:16661461:T:C:UTR3:SLC35E	chr19	16661461	16661461	T	C	UTR3	SLC35E1	ENST000005	.	.	A	G	-	-	0.47058824	27,0,24,0	chr19,16661	Cytosol	A > G
chr19:16661486:T:C:UTR3:SLC35E	chr19	16661486	16661486	T	C	UTR3	SLC35E1	ENST000005	.	.	A	G	-	-	0.42696629	51,0,38,0	chr19,16661	Cytosol	A > G
chr19:16661491:T:C:UTR3:SLC35E	chr19	16661491	16661491	T	C	UTR3	SLC35E1	ENST000005	.	.	A	G	-	-	0.25	66,0,22,0	chr19,16661	Cytosol	A > G
chr19:16662658:T:C:ncRNA_exoni	chr19	16662658	16662658	T	C	ncRNA_exon	CTD-3222D1	.	.	.	A	G	-	-+	0.14754098	52,0,9,0	chr19,16662	Cytosol	A > G
chr19:16662704:T:C:ncRNA_exoni	chr19	16662704	16662704	T	C	ncRNA_exon	CTD-3222D1	.	.	.	A	G	-	-+	0.225	62,0,18,0	chr19,16662	Cytosol	A > G
chr19:16662714:T:C:ncRNA_intror	chr19	16662714	16662714	T	C	ncRNA_intro	CTD-3222D1	.	.	.	A	G	-	-+	0.10447761	60,0,7,0	chr19,16662	Cytosol	A > G
chr19:16662827:T:C:ncRNA_intror	chr19	16662827	16662827	T	C	ncRNA_intro	CTD-3222D1	.	.	.	A	G	-	-+	0.14666667	64,0,11,0	chr19,16662	Cytosol	A > G
chr19:16663345:T:C:UTR3:SLC35E	chr19	16663345	16663345	T	C	UTR3	SLC35E1	ENST000005	.	.	A	G	-	-	0.17391304	133,0,28,0	chr19,16663	Cytosol	A > G
chr19:16663411:T:C:UTR3:SLC35E	chr19	16663411	16663411	T	C	UTR3	SLC35E1	ENST000005	.	.	A	G	-	-	0.10958904	65,0,8,0	chr19,16663	Cytosol	A > G
chr19:16663416:T:C:UTR3:SLC35E	chr19	16663416	16663416	T	C	UTR3	SLC35E1	ENST000005	.	.	A	G	-	-	0.10344828	78,0,9,0	chr19,16663	Cytosol	A > G
chr19:16663423:T:C:UTR3:SLC35E	chr19	16663423	16663423	T	C	UTR3	SLC35E1	ENST000005	.	.	A	G	-	-	0.08602151	85,0,8,0	chr19,16663	Cytosol	A > G
chr19:16663433:T:C:UTR3:SLC35E	chr19	16663433	16663433	T	C	UTR3	SLC35E1	ENST000005	.	.	A	G	-	-	0.08130081	113,0,10,0	chr19,16663	Cytosol	A > G
chr19:16674551:T:C:intronic:CTD-	chr19	16674551	16674551	T	C	intronic	CTD-3222D1	.	.	.	A	G	-	-	0.9	1,0,9,0	chr19,16674	Cytosol	A > G
chr19:16745385:T:C:ncRNA_exoni	chr19	16745385	16745385	T	C	ncRNA_exon	CTC-429P9.3	.	.	.	A	G	-	+-	0.3	7,0,3,0	chr19,16745	Cytosol	A > G
chr19:16745494:T:C:ncRNA_exoni	chr19	16745494	16745494	T	C	ncRNA_exon	CTC-429P9.3	.	.	.	A	G	-	+-	0.33333333	4,0,2,0	chr19,16745	Cytosol	A > G
chr19:16747241:T:C:ncRNA_exoni	chr19	16747241	16747241	T	C	ncRNA_exon	CTC-429P9.2	.	.	.	A	G	-	+-	0.14285714	12,0,2,0	chr19,16747	Cytosol	A > G
chr19:16747301:T:C:ncRNA_exoni	chr19	16747301	16747301	T	C	ncRNA_exon	CTC-429P9.2	.	.	.	A	G	-	+-	0.85714286	1,0,6,0	chr19,16747	Cytosol	A > G
chr19:16747642:T:C:ncRNA_intror	chr19	16747642	16747642	T	C	ncRNA_intro	CTC-429P9.1	.	.	.	A	G	-	+-	0.41666667	7,0,5,0	chr19,16747	Cytosol	A > G
chr19:16748082:T:C:ncRNA_intror	chr19	16748082	16748082	T	C	ncRNA_intro	CTC-429P9.1	.	.	.	A	G	-	+-	0.25	6,0,2,0	chr19,16748	Cytosol	A > G
chr19:16748234:T:C:ncRNA_intror	chr19	16748234	16748234	T	C	ncRNA_intro	CTC-429P9.1	.	.	.	A	G	-	+-	0.15384615	11,0,2,0	chr19,16748	Cytosol	A > G
chr19:16749026:T:C:ncRNA_intror	chr19	16749026	16749026	T	C	ncRNA_intro	CTC-429P9.1	.	.	.	A	G	-	+-	0.8	1,0,4,0	chr19,16749	Cytosol	A > G
chr19:16751070:T:C:ncRNA_intror	chr19	16751070	16751070	T	C	ncRNA_intro	CTC-429P9.1	.	.	.	A	G	-	+-	0.4	3,0,2,0	chr19,16751	Cytosol	A > G
chr19:16753539:T:C:ncRNA_intror	chr19	16753539	16753539	T	C	ncRNA_intro	CTC-429P9.1	.	.	.	A	G	-	+-	0.55555556	4,0,5,0	chr19,16753	Cytosol	A > G
chr19:16754310:T:C:ncRNA_intror	chr19	16754310	16754310	T	C	ncRNA_intro	CTC-429P9.1	.	.	.	A	G	-	+-	0.28571429	5,0,2,0	chr19,16754	Cytosol	A > G

chr19:16755175:T:C:intronic:CTC-4	chr19	16755175	16755175	T	C	intronic	CTC-429P9.4	.	.	.	A	G	-	-	0.33333333	4,0,2,0	chr19,16755	Cytosol	A > G
chr19:16794138:A:G:intronic:TME	chr19	16794138	16794138	A	G	intronic	TMEM38A	.	.	.	A	G	+	+	0.57142857	3,0,4,0	chr19,16794	Cytosol	A > G
chr19:16966484:A:G:UTR3:SIN3B	chr19	16966484	16966484	A	G	UTR3	SIN3B	ENST000005	.	.	A	G	+	+	0.30769231	9,0,4,0	chr19,16966	Cytosol	A > G
chr19:16966487:A:G:UTR3:SIN3B	chr19	16966487	16966487	A	G	UTR3	SIN3B	ENST000005	.	.	A	G	+	+	0.73333333	4,0,11,0	chr19,16966	Cytosol	A > G
chr19:16966605:A:G:intronic:SIN3	chr19	16966605	16966605	A	G	intronic	SIN3B	.	.	.	A	G	+	+	0.13043478	20,0,3,0	chr19,16966	Cytosol	A > G
chr19:16966612:A:G:intronic:SIN3	chr19	16966612	16966612	A	G	intronic	SIN3B	.	.	.	A	G	+	+	0.15625	27,0,5,0	chr19,16966	Cytosol	A > G
chr19:16966618:A:G:intronic:SIN3	chr19	16966618	16966618	A	G	intronic	SIN3B	.	.	.	A	G	+	+	0.13333333	26,0,4,0	chr19,16966	Cytosol	A > G
chr19:16966739:A:G:intronic:SIN3	chr19	16966739	16966739	A	G	intronic	SIN3B	.	.	.	A	G	+	+	0.47826087	12,0,11,0	chr19,16967	Cytosol	A > G
chr19:16967463:A:G:intronic:SIN3	chr19	16967463	16967463	A	G	intronic	SIN3B	.	.	.	A	G	+	+	0.23529412	13,0,4,0	chr19,16967	Cytosol	A > G
chr19:16967476:A:G:intronic:SIN3	chr19	16967476	16967476	A	G	intronic	SIN3B	.	.	.	A	G	+	+	0.4375	9,0,7,0	chr19,16967	Cytosol	A > G
chr19:16967512:A:G:intronic:SIN3	chr19	16967512	16967512	A	G	intronic	SIN3B	.	.	.	A	G	+	+	0.63636364	8,0,14,0	chr19,16967	Cytosol	A > G
chr19:16967522:A:G:intronic:SIN3	chr19	16967522	16967522	A	G	intronic	SIN3B	.	.	.	A	G	+	+	0.70833333	7,0,17,0	chr19,16967	Cytosol	A > G
chr19:16967548:A:G:intronic:SIN3	chr19	16967548	16967548	A	G	intronic	SIN3B	.	.	.	A	G	+	+	0.6	10,0,15,0	chr19,16967	Cytosol	A > G
chr19:16967563:A:G:intronic:SIN3	chr19	16967563	16967563	A	G	intronic	SIN3B	.	.	.	A	G	+	+	0.68965517	9,0,20,0	chr19,16967	Cytosol	A > G
chr19:16967566:A:G:intronic:SIN3	chr19	16967566	16967566	A	G	intronic	SIN3B	.	.	.	A	G	+	+	0.55172414	13,0,16,0	chr19,16967	Cytosol	A > G
chr19:16967592:A:G:intronic:SIN3	chr19	16967592	16967592	A	G	intronic	SIN3B	.	.	.	A	G	+	+	0.13636364	19,0,3,0	chr19,16967	Cytosol	A > G
chr19:16967618:A:G:intronic:SIN3	chr19	16967618	16967618	A	G	intronic	SIN3B	.	.	.	A	G	+	+	0.81481481	5,0,22,0	chr19,16967	Cytosol	A > G
chr19:16967635:A:G:intronic:SIN3	chr19	16967635	16967635	A	G	intronic	SIN3B	.	.	.	A	G	+	+	0.77777778	6,0,21,0	chr19,16967	Cytosol	A > G
chr19:17214604:A:G:intronic:MYO	chr19	17214604	17214604	A	G	intronic	MYO9B	.	.	.	A	G	+	+	0.83333333	1,0,5,0	chr19,17214	Cytosol	A > G
chr19:17214617:A:G:intronic:MYO	chr19	17214617	17214617	A	G	intronic	MYO9B	.	.	.	A	G	+	+	0.8	1,0,4,0	chr19,17214	Cytosol	A > G
chr19:17215499:A:G:intronic:MYO	chr19	17215499	17215499	A	G	intronic	MYO9B	.	.	.	A	G	+	+	0.35714286	9,0,5,0	chr19,17215	Cytosol	A > G
chr19:17388273:A:G:UTR3:BABAM	chr19	17388273	17388273	A	G	UTR3	BABAM1	ENST000006	.	.	A	G	+	-+	0.4	3,0,2,0	chr19,17388	Cytosol	A > G
chr19:17524943:A:G:ncRNA_splici	chr19	17524943	17524943	A	G	ncRNA_splici	CTD-2521M2	ENST000005	.	.	A	G	+	+	0.28571429	5,0,2,0	chr19,17524	Cytosol	A > G
chr19:17524986:A:G:ncRNA_exoni	chr19	17524986	17524986	A	G	ncRNA_exon	CTD-2521M2	.	.	.	A	G	+	+	0.6	2,0,3,0	chr19,17524	Cytosol	A > G
chr19:17525422:A:G:ncRNA_exoni	chr19	17525422	17525422	A	G	ncRNA_exon	CTD-2521M2	.	.	.	A	G	+	+-	0.05714286	33,0,2,0	chr19,17525	Cytosol	A > G
chr19:17525423:A:G:ncRNA_exoni	chr19	17525423	17525423	A	G	ncRNA_exon	CTD-2521M2	.	.	.	A	G	+	+-	0.25	27,0,9,0	chr19,17525	Cytosol	A > G
chr19:17525434:A:G:ncRNA_exoni	chr19	17525434	17525434	A	G	ncRNA_exon	CTD-2521M2	.	.	.	A	G	+	+-	0.27586207	21,0,8,0	chr19,17525	Cytosol	A > G
chr19:17525449:A:G:ncRNA_exoni	chr19	17525449	17525449	A	G	ncRNA_exon	CTD-2521M2	.	.	.	A	G	+	+-	0.31818182	15,0,7,0	chr19,17525	Cytosol	A > G
chr19:17525496:A:G:ncRNA_exoni	chr19	17525496	17525496	A	G	ncRNA_exon	CTD-2521M2	.	.	.	A	G	+	+-	0.37037037	17,0,10,0	chr19,17525	Cytosol	A > G
chr19:17525617:A:G:ncRNA_exoni	chr19	17525617	17525617	A	G	ncRNA_exon	CTD-2521M2	.	.	.	A	G	+	+-	0.10526316	17,0,2,0	chr19,17525	Cytosol	A > G
chr19:17525742:A:G:ncRNA_exoni	chr19	17525742	17525742	A	G	ncRNA_exon	CTD-2521M2	.	.	.	A	G	+	+-	0.06896552	27,0,2,0	chr19,17525	Cytosol	A > G
chr19:17525816:A:G:ncRNA_exoni	chr19	17525816	17525816	A	G	ncRNA_exon	CTD-2521M2	.	.	.	A	G	+	+-	0.13953488	37,0,6,0	chr19,17525	Cytosol	A > G
chr19:17525842:A:G:ncRNA_exoni	chr19	17525842	17525842	A	G	ncRNA_exon	CTD-2521M2	.	.	.	A	G	+	+-	0.04	48,0,2,0	chr19,17525	Cytosol	A > G
chr19:17525847:A:G:ncRNA_exoni	chr19	17525847	17525847	A	G	ncRNA_exon	CTD-2521M2	.	.	.	A	G	+	+-	0.06122449	46,0,3,0	chr19,17525	Cytosol	A > G
chr19:17525848:A:G:ncRNA_exoni	chr19	17525848	17525848	A	G	ncRNA_exon	CTD-2521M2	.	.	.	A	G	+	+-	0.10416667	43,0,5,0	chr19,17525	Cytosol	A > G
chr19:17525851:A:G:ncRNA_exoni	chr19	17525851	17525851	A	G	ncRNA_exon	CTD-2521M2	.	.	.	A	G	+	+-	0.16	42,0,8,0	chr19,17525	Cytosol	A > G
chr19:17525852:A:G:ncRNA_exoni	chr19	17525852	17525852	A	G	ncRNA_exon	CTD-2521M2	.	.	.	A	G	+	+-	0.20930233	34,0,9,0	chr19,17525	Cytosol	A > G
chr19:17532489:A:G:ncRNA_intro	chr19	17532489	17532489	A	G	ncRNA_intro	CTD-2521M2	.	.	.	A	G	+	-+	1	0,0,7,0	chr19,17532	Cytosol	A > G
chr19:17936521:T:C:UTR3:JAK3	chr19	17936521	17936521	T	C	UTR3	JAK3	ENST000004	.	.	A	G	-	-	0.66666667	3,0,6,0	chr19,17936	Cytosol	A > G
chr19:18045317:A:G:intronic:CCD	chr19	18045317	18045317	A	G	intronic	CCDC124	.	.	.	A	G	+	+	0.4	3,0,2,0	chr19,18045	Cytosol	A > G
chr19:18045824:A:G:intronic:CCD	chr19	18045824	18045824	A	G	intronic	CCDC124	.	.	.	A	G	+	+	0.66666667	2,0,4,0	chr19,18045	Cytosol	A > G
chr19:18475222:A:G:UTR3:PGPEP	chr19	18475222	18475222	A	G	UTR3	PGPEP1	ENST000002	.	.	A	G	+	+	0.11764706	15,0,2,0	chr19,18475	Cytosol	A > G
chr19:18475223:A:G:UTR3:PGPEP	chr19	18475223	18475223	A	G	UTR3	PGPEP1	ENST000002	.	.	A	G	+	+	0.23529412	13,0,4,0	chr19,18475	Cytosol	A > G
chr19:18475303:A:G:UTR3:PGPEP	chr19	18475303	18475303	A	G	UTR3	PGPEP1	ENST000002	.	.	A	G	+	+	0.10526316	17,0,2,0	chr19,18475	Cytosol	A > G
chr19:18475345:A:G:UTR3:PGPEP	chr19	18475345	18475345	A	G	UTR3	PGPEP1	ENST000002	.	.	A	G	+	+	0.16666667	15,0,3,0	chr19,18475	Cytosol	A > G
chr19:18476176:A:G:UTR3:PGPEP	chr19	18476176	18476176	A	G	UTR3	PGPEP1	ENST000002	.	.	A	G	+	+	0.23076923	20,0,6,0	chr19,18476	Cytosol	A > G
chr19:18476206:A:G:UTR3:PGPEP	chr19	18476206	18476206	A	G	UTR3	PGPEP1	ENST000002	.	.	A	G	+	+	0.2173913	18,0,5,0	chr19,18476	Cytosol	A > G
chr19:18476232:A:G:UTR3:PGPEP	chr19	18476232	18476232	A	G	UTR3	PGPEP1	ENST000002	.	.	A	G	+	+	0.0952381	17,0,2,0	chr19,18476	Cytosol	A > G
chr19:18476239:A:G:UTR3:PGPEP	chr19	18476239	18476239	A	G	UTR3	PGPEP1	ENST000002	.	.	A	G	+	+	0.36363636	14,0,8,0	chr19,18476	Cytosol	A > G
chr19:18476252:A:G:UTR3:PGPEP	chr19	18476252	18476252	A	G	UTR3	PGPEP1	ENST000002	.	.	A	G	+	+	0.0952381	19,0,2,0	chr19,18476	Cytosol	A > G
chr19:18476282:A:G:UTR3:PGPEP	chr19	18476282	18476282	A	G	UTR3	PGPEP1	ENST000002	.	.	A	G	+	+	0.17647059	14,0,3,0	chr19,18476	Cytosol	A > G
chr19:18476292:A:G:UTR3:PGPEP	chr19	18476292	18476292	A	G	UTR3	PGPEP1	ENST000002	.	.	A	G	+	+	0.35714286	9,0,5,0	chr19,18476	Cytosol	A > G
chr19:18476294:A:G:UTR3:PGPEP	chr19	18476294	18476294	A	G	UTR3	PGPEP1	ENST000002	.	.	A	G	+	+	0.13333333	13,0,2,0	chr19,18476	Cytosol	A > G
chr19:18476325:A:G:UTR3:PGPEP	chr19	18476325	18476325	A	G	UTR3	PGPEP1	ENST000002	.	.	A	G	+	+	0.25	9,0,3,0	chr19,18476	Cytosol	A > G
chr19:18476415:A:G:UTR3:PGPEP	chr19	18476415	18476415	A	G	UTR3	PGPEP1	ENST000002	.	.	A	G	+	+	0.08695652	21,0,2,0	chr19,18476	Cytosol	A > G
chr19:18476416:A:G:UTR3:PGPEP	chr19	18476416	18476416	A	G	UTR3	PGPEP1	ENST000002	.	.	A	G	+	+	0.18181818	18,0,4,0	chr19,18476	Cytosol	A > G
chr19:18477320:A:G:UTR3:PGPEP	chr19	18477320	18477320	A	G	UTR3	PGPEP1	ENST000002	.	.	A	G	+	+	0.17647059	14,0,3,0	chr19,18477	Cytosol	A > G
chr19:18477329:A:G:UTR3:PGPEP	chr19	18477329	18477329	A	G	UTR3	PGPEP1	ENST000002	.	.	A	G	+	+	0.75	4,0,12,0	chr19,18477	Cytosol	A > G
chr19:18477337:A:G:UTR3:PGPEP	chr19	18477337	18477337	A	G	UTR3	PGPEP1	ENST000002	.	.	A	G	+	+	0.26666667	11,0,4,0	chr19,18477	Cytosol	A > G
chr19:18477410:A:G:UTR3:PGPEP	chr19	18477410	18477410	A	G	UTR3	PGPEP1	ENST000002	.	.	A	G	+	+	0.46666667	8,0,7,0	chr19,18477	Cytosol	A > G
chr19:18477420:A:G:UTR3:PGPEP	chr19	18477420	18477420	A	G	UTR3	PGPEP1	ENST000002	.	.	A	G	+	+	0.26666667	11,0,4,0	chr19,18477	Cytosol	A > G
chr19:18477446:A:G:UTR3:PGPEP	chr19	18477446	18477446	A	G	UTR3	PGPEP1	ENST000002	.	.	A	G	+	+	0.33333333	4,0,2,0	chr19,18477	Cytosol	A > G
chr19:18477461:A:G:UTR3:PGPEP	chr19	18477461	18477461	A	G	UTR3	PGPEP1	ENST000002	.	.	A	G	+	+	1	0,0,5,0	chr19,18477	Cytosol	A > G
chr19:18477696:A:G:UTR3:PGPEP	chr19	18477696	18477696	A	G	UTR3	PGPEP1	ENST000002	.	.	A	G	+	+	0.06666667	28,0,2,0	chr19,18477	Cytosol	A > G

chr19:18477702:A:G:UTR3:PGPEP	chr19	18477702	18477702	A	G	UTR3	PGPEP1	ENST000002	.	.	A	G	+	+	0.12	22,0,3,0	chr19,18477	Cytosol	A > G
chr19:18477708:A:G:UTR3:PGPEP	chr19	18477708	18477708	A	G	UTR3	PGPEP1	ENST000002	.	.	A	G	+	+	0.12903226	27,0,4,0	chr19,18477	Cytosol	A > G
chr19:18477709:A:G:UTR3:PGPEP	chr19	18477709	18477709	A	G	UTR3	PGPEP1	ENST000002	.	.	A	G	+	+	0.36666667	19,0,11,0	chr19,18477	Cytosol	A > G
chr19:18477761:A:G:UTR3:PGPEP	chr19	18477761	18477761	A	G	UTR3	PGPEP1	ENST000002	.	.	A	G	+	+	0.16666667	20,0,4,0	chr19,18477	Cytosol	A > G
chr19:18477800:A:G:UTR3:PGPEP	chr19	18477800	18477800	A	G	UTR3	PGPEP1	ENST000002	.	.	A	G	+	+	0.3125	11,0,5,0	chr19,18477	Cytosol	A > G
chr19:18477803:A:G:UTR3:PGPEP	chr19	18477803	18477803	A	G	UTR3	PGPEP1	ENST000002	.	.	A	G	+	+	0.35294118	11,0,6,0	chr19,18477	Cytosol	A > G
chr19:18477819:A:G:UTR3:PGPEP	chr19	18477819	18477819	A	G	UTR3	PGPEP1	ENST000002	.	.	A	G	+	+	0.23076923	10,0,3,0	chr19,18477	Cytosol	A > G
chr19:18477851:A:G:UTR3:PGPEP	chr19	18477851	18477851	A	G	UTR3	PGPEP1	ENST000002	.	.	A	G	+	+	0.44444444	5,0,4,0	chr19,18477	Cytosol	A > G
chr19:18477859:A:G:UTR3:PGPEP	chr19	18477859	18477859	A	G	UTR3	PGPEP1	ENST000002	.	.	A	G	+	+	0.61538462	5,0,8,0	chr19,18477	Cytosol	A > G
chr19:18477901:A:G:UTR3:PGPEP	chr19	18477901	18477901	A	G	UTR3	PGPEP1	ENST000002	.	.	A	G	+	+	0.22727273	17,0,5,0	chr19,18477	Cytosol	A > G
chr19:18477915:A:G:UTR3:PGPEP	chr19	18477915	18477915	A	G	UTR3	PGPEP1	ENST000002	.	.	A	G	+	+	0.13043478	20,0,3,0	chr19,18477	Cytosol	A > G
chr19:18477921:A:G:UTR3:PGPEP	chr19	18477921	18477921	A	G	UTR3	PGPEP1	ENST000002	.	.	A	G	+	+	0.06896552	27,0,2,0	chr19,18477	Cytosol	A > G
chr19:18477923:A:G:UTR3:PGPEP	chr19	18477923	18477923	A	G	UTR3	PGPEP1	ENST000002	.	.	A	G	+	+	0.20689655	23,0,6,0	chr19,18477	Cytosol	A > G
chr19:18478822:A:G:UTR3:PGPEP	chr19	18478822	18478822	A	G	UTR3	PGPEP1	ENST000002	.	.	A	G	+	+	0.64285714	5,0,9,0	chr19,18478	Cytosol	A > G
chr19:18478885:A:G:UTR3:PGPEP	chr19	18478885	18478885	A	G	UTR3	PGPEP1	ENST000002	.	.	A	G	+	+	0.40909091	13,0,9,0	chr19,18478	Cytosol	A > G
chr19:18478908:A:G:UTR3:PGPEP	chr19	18478908	18478908	A	G	UTR3	PGPEP1	ENST000002	.	.	A	G	+	+	0.0952381	19,0,2,0	chr19,18478	Cytosol	A > G
chr19:18479128:A:G:UTR3:PGPEP	chr19	18479128	18479128	A	G	UTR3	PGPEP1	ENST000002	.	.	A	G	+	+	0.1	18,0,2,0	chr19,18479	Cytosol	A > G
chr19:18479155:A:G:UTR3:PGPEP	chr19	18479155	18479155	A	G	UTR3	PGPEP1	ENST000002	.	.	A	G	+	+	0.15789474	16,0,3,0	chr19,18479	Cytosol	A > G
chr19:18671080:A:G:ncRNA_intro	chr19	18671080	18671080	A	G	ncRNA_intro	AC005253.2	.	.	.	A	G	+	-+	0.22222222	7,0,2,0	chr19,18671	Cytosol	A > G
chr19:18671086:A:G:ncRNA_intro	chr19	18671086	18671086	A	G	ncRNA_intro	AC005253.2	.	.	.	A	G	+	-+	0.3	7,0,3,0	chr19,18671	Cytosol	A > G
chr19:19019750:T:C:intronic:AC00	chr19	19019750	19019750	T	C	intronic	AC002985.3,	.	.	.	A	G	-	-	0.33333333	4,0,2,0	chr19,19019	Cytosol	A > G
chr19:19623698:T:C:intronic:TSSK	chr19	19623698	19623698	T	C	intronic	TSSK6	.	.	.	A	G	-	-	0.10526316	17,0,2,0	chr19,19623	Cytosol	A > G
chr19:19624590:T:C:intronic:TSSK	chr19	19624590	19624590	T	C	intronic	TSSK6	.	.	.	A	G	-	-	0.47368421	10,0,9,0	chr19,19624	Cytosol	A > G
chr19:19791273:A:G:UTR3:ZNF101	chr19	19791273	19791273	A	G	UTR3	ZNF101	ENST000003	.	.	A	G	+	+	0.3	7,0,3,0	chr19,19791	Cytosol	A > G
chr19:19791274:A:G:UTR3:ZNF101	chr19	19791274	19791274	A	G	UTR3	ZNF101	ENST000003	.	.	A	G	+	+	0.36363636	7,0,4,0	chr19,19791	Cytosol	A > G
chr19:19791275:A:G:UTR3:ZNF101	chr19	19791275	19791275	A	G	UTR3	ZNF101	ENST000003	.	.	A	G	+	+	0.5	4,0,4,0	chr19,19791	Cytosol	A > G
chr19:19791276:A:G:UTR3:ZNF101	chr19	19791276	19791276	A	G	UTR3	ZNF101	ENST000003	.	.	A	G	+	+	0.5	4,0,4,0	chr19,19791	Cytosol	A > G
chr19:19791493:A:G:UTR3:ZNF101	chr19	19791493	19791493	A	G	UTR3	ZNF101	ENST000005	.	.	A	G	+	+	0.21428571	11,0,3,0	chr19,19791	Cytosol	A > G
chr19:19791511:A:G:UTR3:ZNF101	chr19	19791511	19791511	A	G	UTR3	ZNF101	ENST000005	.	.	A	G	+	+	0.22222222	7,0,2,0	chr19,19791	Cytosol	A > G
chr19:19791618:A:G:UTR3:ZNF101	chr19	19791618	19791618	A	G	UTR3	ZNF101	ENST000005	.	.	A	G	+	+	0.375	5,0,3,0	chr19,19791	Cytosol	A > G
chr19:19792335:A:G:UTR3:ZNF101	chr19	19792335	19792335	A	G	UTR3	ZNF101	ENST000005	.	.	A	G	+	+	0.46666667	8,0,7,0	chr19,19792	Cytosol	A > G
chr19:19792448:A:G:UTR3:ZNF101	chr19	19792448	19792448	A	G	UTR3	ZNF101	ENST000005	.	.	A	G	+	+	0.21052632	15,0,4,0	chr19,19792	Cytosol	A > G
chr19:19792456:A:G:UTR3:ZNF101	chr19	19792456	19792456	A	G	UTR3	ZNF101	ENST000005	.	.	A	G	+	+	0.16666667	15,0,3,0	chr19,19792	Cytosol	A > G
chr19:19793154:A:G:UTR3:ZNF101	chr19	19793154	19793154	A	G	UTR3	ZNF101	ENST000005	.	.	A	G	+	+	0.19047619	17,0,4,0	chr19,19793	Cytosol	A > G
chr19:19793780:A:G:UTR3:ZNF101	chr19	19793780	19793780	A	G	UTR3	ZNF101	ENST000005	.	.	A	G	+	+	0.875	2,0,14,0	chr19,19793	Cytosol	A > G
chr19:19793875:A:G:UTR3:ZNF101	chr19	19793875	19793875	A	G	UTR3	ZNF101	ENST000005	.	.	A	G	+	+	0.28	18,0,7,0	chr19,19793	Cytosol	A > G
chr19:19793876:A:G:UTR3:ZNF101	chr19	19793876	19793876	A	G	UTR3	ZNF101	ENST000005	.	.	A	G	+	+	0.52173913	11,0,12,0	chr19,19793	Cytosol	A > G
chr19:20823833:T:C:ncRNA_intror	chr19	20823833	20823833	T	C	ncRNA_intro	CTC-513N18.	.	.	.	A	G	-	-	0.21052632	15,0,4,0	chr19,20823	Cytosol	A > G
chr19:20823928:T:C:ncRNA_intror	chr19	20823928	20823928	T	C	ncRNA_intro	CTC-513N18.	.	.	.	A	G	-	-	0.25	9,0,3,0	chr19,20823	Cytosol	A > G
chr19:20824450:T:C:ncRNA_intror	chr19	20824450	20824450	T	C	ncRNA_intro	CTC-513N18.	.	.	.	A	G	-	-	0.30769231	9,0,4,0	chr19,20824	Cytosol	A > G
chr19:20824633:T:C:ncRNA_intror	chr19	20824633	20824633	T	C	ncRNA_intro	CTC-513N18.	.	.	.	A	G	-	-	0.29411765	12,0,5,0	chr19,20824	Cytosol	A > G
chr19:20824634:T:C:ncRNA_intror	chr19	20824634	20824634	T	C	ncRNA_intro	CTC-513N18.	.	.	.	A	G	-	-	0.38888889	11,0,7,0	chr19,20824	Cytosol	A > G
chr19:21302045:A:G:UTR3:ZNF71	chr19	21302045	21302045	A	G	UTR3	ZNF714	ENST000005	.	.	A	G	+	+	0.14285714	12,0,2,0	chr19,21302	Cytosol	A > G
chr19:21302094:A:G:UTR3:ZNF71	chr19	21302094	21302094	A	G	UTR3	ZNF714	ENST000005	.	.	A	G	+	+	0.18181818	9,0,2,0	chr19,21302	Cytosol	A > G
chr19:21302099:A:G:UTR3:ZNF71	chr19	21302099	21302099	A	G	UTR3	ZNF714	ENST000005	.	.	A	G	+	+	0.45454545	6,0,5,0	chr19,21302	Cytosol	A > G
chr19:21302938:A:G:UTR3:ZNF71	chr19	21302938	21302938	A	G	UTR3	ZNF714	ENST000005	.	.	A	G	+	+	0.11538462	23,0,3,0	chr19,21302	Cytosol	A > G
chr19:21302944:A:G:UTR3:ZNF71	chr19	21302944	21302944	A	G	UTR3	ZNF714	ENST000005	.	.	A	G	+	+	0.28	18,0,7,0	chr19,21302	Cytosol	A > G
chr19:21302945:A:G:UTR3:ZNF71	chr19	21302945	21302945	A	G	UTR3	ZNF714	ENST000005	.	.	A	G	+	+	0.39130435	14,0,9,0	chr19,21302	Cytosol	A > G
chr19:21302962:A:G:UTR3:ZNF71	chr19	21302962	21302962	A	G	UTR3	ZNF714	ENST000005	.	.	A	G	+	+	0.16666667	20,0,4,0	chr19,21302	Cytosol	A > G
chr19:21303010:A:G:UTR3:ZNF71	chr19	21303010	21303010	A	G	UTR3	ZNF714	ENST000005	.	.	A	G	+	+	0.39130435	14,0,9,0	chr19,21303	Cytosol	A > G
chr19:21303017:A:G:UTR3:ZNF71	chr19	21303017	21303017	A	G	UTR3	ZNF714	ENST000005	.	.	A	G	+	+	0.54166667	11,0,13,0	chr19,21303	Cytosol	A > G
chr19:21303025:A:G:UTR3:ZNF71	chr19	21303025	21303025	A	G	UTR3	ZNF714	ENST000005	.	.	A	G	+	+	0.09090909	20,0,2,0	chr19,21303	Cytosol	A > G
chr19:21303034:A:G:UTR3:ZNF71	chr19	21303034	21303034	A	G	UTR3	ZNF714	ENST000005	.	.	A	G	+	+	0.25	15,0,5,0	chr19,21303	Cytosol	A > G
chr19:21303090:A:G:UTR3:ZNF71	chr19	21303090	21303090	A	G	UTR3	ZNF714	ENST000005	.	.	A	G	+	+	0.5	5,0,5,0	chr19,21303	Cytosol	A > G
chr19:21303968:A:G:UTR3:ZNF71	chr19	21303968	21303968	A	G	UTR3	ZNF714	ENST000005	.	.	A	G	+	+	0.54545455	5,0,6,0	chr19,21303	Cytosol	A > G
chr19:21303974:A:G:UTR3:ZNF71	chr19	21303974	21303974	A	G	UTR3	ZNF714	ENST000005	.	.	A	G	+	+	0.18181818	9,0,2,0	chr19,21303	Cytosol	A > G
chr19:21304041:A:G:UTR3:ZNF71	chr19	21304041	21304041	A	G	UTR3	ZNF714	ENST000005	.	.	A	G	+	+	0.17647059	14,0,3,0	chr19,21304	Cytosol	A > G
chr19:21305292:A:G:UTR3:ZNF71	chr19	21305292	21305292	A	G	UTR3	ZNF714	ENST000005	.	.	A	G	+	+	0.15384615	11,0,2,0	chr19,21305	Cytosol	A > G
chr19:21305301:A:G:UTR3:ZNF71	chr19	21305301	21305301	A	G	UTR3	ZNF714	ENST000005	.	.	A	G	+	+	0.30769231	9,0,4,0	chr19,21305	Cytosol	A > G
chr19:21305781:A:G:UTR3:ZNF71	chr19	21305781	21305781	A	G	UTR3	ZNF714	ENST000005	.	.	A	G	+	+	0.4	3,0,2,0	chr19,21305	Cytosol	A > G
chr19:21305836:A:G:UTR3:ZNF71	chr19	21305836	21305836	A	G	UTR3	ZNF714	ENST000005	.	.	A	G	+	+	0.28571429	5,0,2,0	chr19,21305	Cytosol	A > G
chr19:21305837:A:G:UTR3:ZNF71	chr19	21305837	21305837	A	G	UTR3	ZNF714	ENST000005	.	.	A	G	+	+	0.4	6,0,4,0	chr19,21305	Cytosol	A > G
chr19:21305863:A:G:UTR3:ZNF71	chr19	21305863	21305863	A	G	UTR3	ZNF714	ENST000005	.	.	A	G	+	+	0.42857143	4,0,3,0	chr19,21305	Cytosol	A > G
chr19:21306206:A:G:intronic:ZNF7	chr19	21306206	21306206	A	G	intronic	ZNF714	.	.	.	A	G	+	+	1	0,0,5,0	chr19,21306	Cytosol	A > G

chr19:21306365:A:G:intronic:ZNF7	chr19	21306365	21306365	A	G	intronic	ZNF714	.	.	.	A	G	+	+	0.83333333	1,0,5,0	chr19,21306	Cytosol	A > G
chr19:21366944:A:G:UTR3:ZNF431	chr19	21366944	21366944	A	G	UTR3	ZNF431	ENST000003	.	.	A	G	+	+	0.19230769	21,0,5,0	chr19,21366	Cytosol	A > G
chr19:21367088:A:G:UTR3:ZNF431	chr19	21367088	21367088	A	G	UTR3	ZNF431	ENST000003	.	.	A	G	+	+	0.0952381	19,0,2,0	chr19,21367	Cytosol	A > G
chr19:21367103:A:G:UTR3:ZNF431	chr19	21367103	21367103	A	G	UTR3	ZNF431	ENST000003	.	.	A	G	+	+	0.0952381	19,0,2,0	chr19,21367	Cytosol	A > G
chr19:21368295:A:G:UTR3:ZNF431	chr19	21368295	21368295	A	G	UTR3	ZNF431	ENST000003	.	.	A	G	+	+	0.25	6,0,2,0	chr19,21368	Cytosol	A > G
chr19:21368333:A:G:UTR3:ZNF431	chr19	21368333	21368333	A	G	UTR3	ZNF431	ENST000003	.	.	A	G	+	+	0.2	8,0,2,0	chr19,21368	Cytosol	A > G
chr19:21368341:A:G:UTR3:ZNF431	chr19	21368341	21368341	A	G	UTR3	ZNF431	ENST000003	.	.	A	G	+	+	0.18181818	9,0,2,0	chr19,21368	Cytosol	A > G
chr19:21368367:A:G:UTR3:ZNF431	chr19	21368367	21368367	A	G	UTR3	ZNF431	ENST000003	.	.	A	G	+	+	0.25	6,0,2,0	chr19,21368	Cytosol	A > G
chr19:21368402:A:G:UTR3:ZNF431	chr19	21368402	21368402	A	G	UTR3	ZNF431	ENST000003	.	.	A	G	+	+	0.28571429	5,0,2,0	chr19,21368	Cytosol	A > G
chr19:21372861:A:G:UTR3:ZNF431	chr19	21372861	21372861	A	G	UTR3	ZNF431	ENST000005	.	.	A	G	+	+	0.11111111	16,0,2,0	chr19,21372	Cytosol	A > G
chr19:21372918:A:G:UTR3:ZNF431	chr19	21372918	21372918	A	G	UTR3	ZNF431	ENST000005	.	.	A	G	+	+	0.14285714	18,0,3,0	chr19,21372	Cytosol	A > G
chr19:21373522:A:G:downstream:	chr19	21373522	21373522	A	G	downstream	ZNF431	.	.	.	A	G	+		0.88888889	1,0,8,0	chr19,21373	Cytosol	A > G
chr19:21373529:A:G:downstream:	chr19	21373529	21373529	A	G	downstream	ZNF431	.	.	.	A	G	+		0.5	5,0,5,0	chr19,21373	Cytosol	A > G
chr19:21373666:A:G:downstream:	chr19	21373666	21373666	A	G	downstream	ZNF431	.	.	.	A	G	+		0.4	3,0,2,0	chr19,21373	Cytosol	A > G
chr19:23438402:T:C:ncRNA_exoni	chr19	23438402	23438402	T	C	ncRNA_exon	RP11-15H20.	.	.	.	A	G	-	-	0.27272727	8,0,3,0	chr19,23438	Cytosol	A > G
chr19:23440320:T:C:ncRNA_exoni	chr19	23440320	23440320	T	C	ncRNA_exon	RP11-15H20.	.	.	.	A	G	-	-	0.44444444	5,0,4,0	chr19,23440	Cytosol	A > G
chr19:23440364:T:C:ncRNA_exoni	chr19	23440364	23440364	T	C	ncRNA_exon	RP11-15H20.	.	.	.	A	G	-	-	0.6	2,0,3,0	chr19,23440	Cytosol	A > G
chr19:23441299:T:C:ncRNA_intror	chr19	23441299	23441299	T	C	ncRNA_intro	RP11-15H20.	.	.	.	A	G	-	-	0.28571429	5,0,2,0	chr19,23441	Cytosol	A > G
chr19:23441302:T:C:ncRNA_intror	chr19	23441302	23441302	T	C	ncRNA_intro	RP11-15H20.	.	.	.	A	G	-	-	0.28571429	5,0,2,0	chr19,23441	Cytosol	A > G
chr19:23441304:T:C:ncRNA_intror	chr19	23441304	23441304	T	C	ncRNA_intro	RP11-15H20.	.	.	.	A	G	-	-	0.42857143	4,0,3,0	chr19,23441	Cytosol	A > G
chr19:23441343:T:C:ncRNA_intror	chr19	23441343	23441343	T	C	ncRNA_intro	RP11-15H20.	.	.	.	A	G	-	-	0.28571429	5,0,2,0	chr19,23441	Cytosol	A > G
chr19:23441380:T:C:ncRNA_intror	chr19	23441380	23441380	T	C	ncRNA_intro	RP11-15H20.	.	.	.	A	G	-	-	0.66666667	3,0,6,0	chr19,23441	Cytosol	A > G
chr19:24010482:G:A:exonic:RPSAF	chr19	24010482	24010482	G	A	exonic	RPSAP58	.	synonymous	RPSAP58:EN	G	A	+	-+	0.6228275	2401,0,1454,	chr19,24010	Cytosol	G > A
chr19:24103276:A:G:ncRNA_intro	chr19	24103276	24103276	A	G	ncRNA_intro	CTB-92J24.3	.	.	.	A	G	+	+-	0.6875	5,0,11,0	chr19,24103	Cytosol	A > G
chr19:24103349:A:G:ncRNA_intro	chr19	24103349	24103349	A	G	ncRNA_intro	CTB-92J24.3	.	.	.	A	G	+	+-	0.5625	7,0,9,0	chr19,24103	Cytosol	A > G
chr19:24249581:A:G:ncRNA_exoni	chr19	24249581	24249581	A	G	ncRNA_exon	CTD-2017D1	.	.	.	A	G	+	+	0.41666667	14,0,10,0	chr19,24249	Cytosol	A > G
chr19:30191863:T:C:UTR3:C19orf1	chr19	30191863	30191863	T	C	UTR3	C19orf12	ENST000003	.	.	A	G	-	-	0.27272727	8,0,3,0	chr19,30191	Cytosol	A > G
chr19:33461875:T:C:intronic:CEP8	chr19	33461875	33461875	T	C	intronic	CEP89	.	.	.	A	G	-	-	0.36363636	14,0,8,0	chr19,33461	Cytosol	A > G
chr19:33665031:T:C:intronic:WDR	chr19	33665031	33665031	T	C	intronic	WDR88	.	.	.	A	G	-	+	0.4	3,0,2,0	chr19,33665	Cytosol	A > G
chr19:34860009:A:G:intronic:GPI	chr19	34860009	34860009	A	G	intronic	GPI	.	.	.	A	G	+	+	0.9	1,0,9,0	chr19,34860	Cytosol	A > G
chr19:34860018:A:G:intronic:GPI	chr19	34860018	34860018	A	G	intronic	GPI	.	.	.	A	G	+	+	0.5	3,0,3,0	chr19,34860	Cytosol	A > G
chr19:34860066:A:G:intronic:GPI	chr19	34860066	34860066	A	G	intronic	GPI	.	.	.	A	G	+	+	1	0,0,6,0	chr19,34860	Cytosol	A > G
chr19:36260415:A:G:ncRNA_intro	chr19	36260415	36260415	A	G	ncRNA_intro	AC002398.1	.	.	.	A	G	+	+-	0.69230769	4,0,9,0	chr19,36260	Cytosol	A > G
chr19:36260451:A:G:ncRNA_intro	chr19	36260451	36260451	A	G	ncRNA_intro	AC002398.1	.	.	.	A	G	+	+-	0.52941176	8,0,9,0	chr19,36260	Cytosol	A > G
chr19:36260469:A:G:ncRNA_intro	chr19	36260469	36260469	A	G	ncRNA_intro	AC002398.1	.	.	.	A	G	+	+-	0.17647059	14,0,3,0	chr19,36260	Cytosol	A > G
chr19:36260470:A:G:ncRNA_intro	chr19	36260470	36260470	A	G	ncRNA_intro	AC002398.1	.	.	.	A	G	+	+-	0.26315789	14,0,5,0	chr19,36260	Cytosol	A > G
chr19:36260762:A:G:ncRNA_intro	chr19	36260762	36260762	A	G	ncRNA_intro	AC002398.1	.	.	.	A	G	+	+-	0.44444444	5,0,4,0	chr19,36260	Cytosol	A > G
chr19:36260906:A:G:ncRNA_intro	chr19	36260906	36260906	A	G	ncRNA_intro	AC002398.1	.	.	.	A	G	+	+-	0.29411765	24,0,10,0	chr19,36260	Cytosol	A > G
chr19:36260907:A:G:ncRNA_intro	chr19	36260907	36260907	A	G	ncRNA_intro	AC002398.1	.	.	.	A	G	+	+-	0.37142857	22,0,13,0	chr19,36260	Cytosol	A > G
chr19:36260936:A:G:ncRNA_intro	chr19	36260936	36260936	A	G	ncRNA_intro	AC002398.1	.	.	.	A	G	+	+-	0.1025641	35,0,4,0	chr19,36260	Cytosol	A > G
chr19:36260987:A:G:ncRNA_intro	chr19	36260987	36260987	A	G	ncRNA_intro	AC002398.1	.	.	.	A	G	+	+-	0.1372549	44,0,7,0	chr19,36260	Cytosol	A > G
chr19:36261009:A:G:ncRNA_intro	chr19	36261009	36261009	A	G	ncRNA_intro	AC002398.1	.	.	.	A	G	+	+-	0.525	19,0,21,0	chr19,36261	Cytosol	A > G
chr19:36261168:A:G:ncRNA_intro	chr19	36261168	36261168	A	G	ncRNA_intro	AC002398.1	.	.	.	A	G	+	+-	0.31428571	24,0,11,0	chr19,36261	Cytosol	A > G
chr19:36261208:A:G:ncRNA_intro	chr19	36261208	36261208	A	G	ncRNA_intro	AC002398.1	.	.	.	A	G	+	+-	0.67741935	10,0,21,0	chr19,36261	Cytosol	A > G
chr19:36261524:A:G:ncRNA_intro	chr19	36261524	36261524	A	G	ncRNA_intro	AC002398.1	.	.	.	A	G	+	+-	0.48	13,0,12,0	chr19,36261	Cytosol	A > G
chr19:36261533:A:G:ncRNA_intro	chr19	36261533	36261533	A	G	ncRNA_intro	AC002398.1	.	.	.	A	G	+	+-	0.22727273	17,0,5,0	chr19,36261	Cytosol	A > G
chr19:36261570:A:G:ncRNA_intro	chr19	36261570	36261570	A	G	ncRNA_intro	AC002398.1	.	.	.	A	G	+	+-	1	0,0,10,0	chr19,36261	Cytosol	A > G
chr19:36261578:A:G:ncRNA_intro	chr19	36261578	36261578	A	G	ncRNA_intro	AC002398.1	.	.	.	A	G	+	+-	0.54545455	5,0,6,0	chr19,36261	Cytosol	A > G
chr19:36261612:A:G:ncRNA_intro	chr19	36261612	36261612	A	G	ncRNA_intro	AC002398.1	.	.	.	A	G	+	+-	0.3	14,0,6,0	chr19,36261	Cytosol	A > G
chr19:36261657:A:G:ncRNA_exoni	chr19	36261657	36261657	A	G	ncRNA_exon	AC002398.1	.	.	.	A	G	+	+-	0.46153846	14,0,12,0	chr19,36261	Cytosol	A > G
chr19:36261658:A:G:ncRNA_exoni	chr19	36261658	36261658	A	G	ncRNA_exon	AC002398.1	.	.	.	A	G	+	+-	0.67857143	9,0,19,0	chr19,36261	Cytosol	A > G
chr19:36261671:A:G:ncRNA_exoni	chr19	36261671	36261671	A	G	ncRNA_exon	AC002398.1	.	.	.	A	G	+	+-	0.42307692	15,0,11,0	chr19,36261	Cytosol	A > G
chr19:36810797:T:C:ncRNA_exoni	chr19	36810797	36810797	T	C	ncRNA_exon	LINC00665	.	.	.	A	G	-	-	0.15151515	28,0,5,0	chr19,36810	Cytosol	A > G
chr19:36810823:T:C:ncRNA_exoni	chr19	36810823	36810823	T	C	ncRNA_exon	LINC00665	.	.	.	A	G	-	-	0.85714286	3,0,18,0	chr19,36810	Cytosol	A > G
chr19:36810840:T:C:ncRNA_exoni	chr19	36810840	36810840	T	C	ncRNA_exon	LINC00665	.	.	.	A	G	-	-	0.5	7,0,7,0	chr19,36810	Cytosol	A > G
chr19:36810844:T:C:ncRNA_exoni	chr19	36810844	36810844	T	C	ncRNA_exon	LINC00665	.	.	.	A	G	-	-	0.78571429	3,0,11,0	chr19,36810	Cytosol	A > G
chr19:36810850:T:C:ncRNA_exoni	chr19	36810850	36810850	T	C	ncRNA_exon	LINC00665	.	.	.	A	G	-	-	0.78571429	3,0,11,0	chr19,36810	Cytosol	A > G
chr19:36810876:T:C:ncRNA_exoni	chr19	36810876	36810876	T	C	ncRNA_exon	LINC00665	.	.	.	A	G	-	-	0.4	9,0,6,0	chr19,36810	Cytosol	A > G
chr19:36810880:T:C:ncRNA_exoni	chr19	36810880	36810880	T	C	ncRNA_exon	LINC00665	.	.	.	A	G	-	-	0.33333333	10,0,5,0	chr19,36810	Cytosol	A > G
chr19:36810886:T:C:ncRNA_exoni	chr19	36810886	36810886	T	C	ncRNA_exon	LINC00665	.	.	.	A	G	-	-	0.27777778	13,0,5,0	chr19,36810	Cytosol	A > G
chr19:36810916:T:C:ncRNA_exoni	chr19	36810916	36810916	T	C	ncRNA_exon	LINC00665	.	.	.	A	G	-	-	0.8	4,0,16,0	chr19,36810	Cytosol	A > G
chr19:36810917:T:C:ncRNA_exoni	chr19	36810917	36810917	T	C	ncRNA_exon	LINC00665	.	.	.	A	G	-	-	0.63157895	7,0,12,0	chr19,36810	Cytosol	A > G
chr19:36810940:T:C:ncRNA_exoni	chr19	36810940	36810940	T	C	ncRNA_exon	LINC00665	.	.	.	A	G	-	-	0.33333333	10,0,5,0	chr19,36810	Cytosol	A > G
chr19:36810945:T:C:ncRNA_exoni	chr19	36810945	36810945	T	C	ncRNA_exon	LINC00665	.	.	.	A	G	-	-	0.23076923	10,0,3,0	chr19,36810	Cytosol	A > G

chr19:36810948:T:C:ncRNA_exoni	chr19	36810948	36810948	T	C	ncRNA_exon	LINC00665	.	.	.	A	G	-	-	0.3	7,0,3,0	chr19,36810	Cytosol	A > G
chr19:36811000:T:C:ncRNA_exoni	chr19	36811000	36811000	T	C	ncRNA_exon	LINC00665	.	.	.	A	G	-	-	0.70833333	7,0,17,0	chr19,36810	Cytosol	A > G
chr19:36811015:T:C:ncRNA_exoni	chr19	36811015	36811015	T	C	ncRNA_exon	LINC00665	.	.	.	A	G	-	-	0.25	21,0,7,0	chr19,36811	Cytosol	A > G
chr19:36811417:T:C:ncRNA_exoni	chr19	36811417	36811417	T	C	ncRNA_exon	LINC00665	.	.	.	A	G	-	-	0.54285714	16,0,19,0	chr19,36811	Cytosol	A > G
chr19:36811418:T:C:ncRNA_exoni	chr19	36811418	36811418	T	C	ncRNA_exon	LINC00665	.	.	.	A	G	-	-	0.33333333	22,0,11,0	chr19,36811	Cytosol	A > G
chr19:36811443:T:C:ncRNA_exoni	chr19	36811443	36811443	T	C	ncRNA_exon	LINC00665	.	.	.	A	G	-	-	0.87179487	5,0,34,0	chr19,36811	Cytosol	A > G
chr19:36811497:T:C:ncRNA_exoni	chr19	36811497	36811497	T	C	ncRNA_exon	LINC00665	.	.	.	A	G	-	-	0.29787234	32,0,14,1	chr19,36811	Cytosol	A > G
chr19:36811543:T:C:ncRNA_exoni	chr19	36811543	36811543	T	C	ncRNA_exon	LINC00665	.	.	.	A	G	-	-	0.75925926	13,0,41,0	chr19,36811	Cytosol	A > G
chr19:36811553:T:C:ncRNA_exoni	chr19	36811553	36811553	T	C	ncRNA_exon	LINC00665	.	.	.	A	G	-	-	0.27272727	40,0,15,0	chr19,36811	Cytosol	A > G
chr19:36811570:T:C:ncRNA_exoni	chr19	36811570	36811570	T	C	ncRNA_exon	LINC00665	.	.	.	A	G	-	-	0.75471698	13,0,40,0	chr19,36811	Cytosol	A > G
chr19:36811589:T:C:ncRNA_exoni	chr19	36811589	36811589	T	C	ncRNA_exon	LINC00665	.	.	.	A	G	-	-	0.60465116	17,0,26,0	chr19,36811	Cytosol	A > G
chr19:36811619:T:C:ncRNA_exoni	chr19	36811619	36811619	T	C	ncRNA_exon	LINC00665	.	.	.	A	G	-	-	0.46666667	24,0,21,0	chr19,36811	Cytosol	A > G
chr19:36811658:T:C:ncRNA_exoni	chr19	36811658	36811658	T	C	ncRNA_exon	LINC00665	.	.	.	A	G	-	-	0.09677419	28,0,3,0	chr19,36811	Cytosol	A > G
chr19:36936556:T:C:UTR3:ZNF566	chr19	36936556	36936556	T	C	UTR3	ZNF566	ENST000005	.	.	A	G	-	-	0.5	3,0,3,0	chr19,36936	Cytosol	A > G
chr19:36937362:T:C:UTR3:ZNF566	chr19	36937362	36937362	T	C	UTR3	ZNF566	ENST000004	.	.	A	G	-	-	0.4	3,0,2,0	chr19,36937	Cytosol	A > G
chr19:36937373:T:C:UTR3:ZNF566	chr19	36937373	36937373	T	C	UTR3	ZNF566	ENST000004	.	.	A	G	-	-	0.8	1,0,4,0	chr19,36937	Cytosol	A > G
chr19:36937420:T:C:UTR3:ZNF566	chr19	36937420	36937420	T	C	UTR3	ZNF566	ENST000004	.	.	A	G	-	-	0.27272727	8,0,3,0	chr19,36937	Cytosol	A > G
chr19:37036881:T:C:UTR3:ZNF529	chr19	37036881	37036881	T	C	UTR3	ZNF529	ENST000003	.	.	A	G	-	-	0.375	5,0,3,0	chr19,37036	Cytosol	A > G
chr19:37037174:T:C:UTR3:ZNF529	chr19	37037174	37037174	T	C	UTR3	ZNF529	ENST000003	.	.	A	G	-	-	0.6	2,0,3,0	chr19,37037	Cytosol	A > G
chr19:37815778:A:G:UTR5:HKR1	chr19	37815778	37815778	A	G	UTR5	HKR1	ENST000005	.	.	A	G	+	+	0.15384615	11,0,2,0	chr19,37815	Cytosol	A > G
chr19:37815816:A:G:UTR5:HKR1	chr19	37815816	37815816	A	G	UTR5	HKR1	ENST000005	.	.	A	G	+	+	0.83333333	1,0,5,0	chr19,37815	Cytosol	A > G
chr19:37815865:A:G:intronic:HKR	chr19	37815865	37815865	A	G	intronic	HKR1	.	.	.	A	G	+	+	0.8	2,0,8,0	chr19,37815	Cytosol	A > G
chr19:37815932:A:G:intronic:HKR	chr19	37815932	37815932	A	G	intronic	HKR1	.	.	.	A	G	+	+	0.75	2,0,6,0	chr19,37815	Cytosol	A > G
chr19:37817805:A:G:intronic:HKR	chr19	37817805	37817805	A	G	intronic	HKR1	.	.	.	A	G	+	+	0.88888889	1,0,8,0	chr19,37817	Cytosol	A > G
chr19:37817822:A:G:intronic:HKR	chr19	37817822	37817822	A	G	intronic	HKR1	.	.	.	A	G	+	+	0.44444444	5,0,4,0	chr19,37817	Cytosol	A > G
chr19:37819027:A:G:intronic:HKR	chr19	37819027	37819027	A	G	intronic	HKR1	.	.	.	A	G	+	+	0.57142857	3,0,4,0	chr19,37819	Cytosol	A > G
chr19:37819085:A:G:intronic:HKR	chr19	37819085	37819085	A	G	intronic	HKR1	.	.	.	A	G	+	+	0.55555556	4,0,5,0	chr19,37819	Cytosol	A > G
chr19:37881070:A:G:UTR3:ZNF52	chr19	37881070	37881070	A	G	UTR3	ZNF527	ENST000004	.	.	A	G	+	+	0.2	8,0,2,0	chr19,37881	Cytosol	A > G
chr19:38123816:T:C:UTR3:ZFP30	chr19	38123816	38123816	T	C	UTR3	ZFP30	ENST000003	.	.	A	G	-	-	0.3	7,0,3,0	chr19,38123	Cytosol	A > G
chr19:38123879:T:C:UTR3:ZFP30	chr19	38123879	38123879	T	C	UTR3	ZFP30	ENST000003	.	.	A	G	-	-	0.6	4,0,6,0	chr19,38123	Cytosol	A > G
chr19:38123906:T:C:UTR3:ZFP30	chr19	38123906	38123906	T	C	UTR3	ZFP30	ENST000003	.	.	A	G	-	-	1	0,0,10,0	chr19,38123	Cytosol	A > G
chr19:38124347:T:C:UTR3:ZFP30	chr19	38124347	38124347	T	C	UTR3	ZFP30	ENST000003	.	.	A	G	-	-	0.92857143	1,0,13,0	chr19,38124	Cytosol	A > G
chr19:38124352:T:C:UTR3:ZFP30	chr19	38124352	38124352	T	C	UTR3	ZFP30	ENST000003	.	.	A	G	-	-	0.18181818	9,0,2,0	chr19,38124	Cytosol	A > G
chr19:38897188:A:G:intronic:FAM	chr19	38897188	38897188	A	G	intronic	FAM98C	.	.	.	A	G	+	+	0.4	3,0,2,0	chr19,38897	Cytosol	A > G
chr19:38897366:A:G:intronic:FAM	chr19	38897366	38897366	A	G	intronic	FAM98C	.	.	.	A	G	+	+	0.4	3,0,2,0	chr19,38897	Cytosol	A > G
chr19:39121713:A:G:intronic:EIF3I	chr19	39121713	39121713	A	G	intronic	EIF3K	.	.	.	A	G	+	+	0.55555556	4,0,5,0	chr19,39121	Cytosol	A > G
chr19:39122807:A:G:intronic:EIF3I	chr19	39122807	39122807	A	G	intronic	EIF3K	.	.	.	A	G	+	+	0.4	6,0,4,0	chr19,39122	Cytosol	A > G
chr19:39122831:A:G:intronic:EIF3I	chr19	39122831	39122831	A	G	intronic	EIF3K	.	.	.	A	G	+	+	0.71428571	2,0,5,0	chr19,39122	Cytosol	A > G
chr19:39122937:A:G:intronic:EIF3I	chr19	39122937	39122937	A	G	intronic	EIF3K	.	.	.	A	G	+	+	0.4	6,0,4,0	chr19,39122	Cytosol	A > G
chr19:39122941:A:G:intronic:EIF3I	chr19	39122941	39122941	A	G	intronic	EIF3K	.	.	.	A	G	+	+	0.25	6,0,2,0	chr19,39122	Cytosol	A > G
chr19:39122956:A:G:intronic:EIF3I	chr19	39122956	39122956	A	G	intronic	EIF3K	.	.	.	A	G	+	+	0.28571429	5,0,2,0	chr19,39122	Cytosol	A > G
chr19:39122960:A:G:intronic:EIF3I	chr19	39122960	39122960	A	G	intronic	EIF3K	.	.	.	A	G	+	+	0.66666667	2,0,4,0	chr19,39122	Cytosol	A > G
chr19:39320672:T:C:intronic:AC10	chr19	39320672	39320672	T	C	intronic	AC104534.3,	.	.	.	A	G	-	-	0.8	1,0,4,0	chr19,39320	Cytosol	A > G
chr19:39320745:T:C:intronic:AC10	chr19	39320745	39320745	T	C	intronic	AC104534.3,	.	.	.	A	G	-	-	0.57142857	3,0,4,0	chr19,39320	Cytosol	A > G
chr19:39358724:T:C:UTR3:RINL	chr19	39358724	39358724	T	C	UTR3	RINL	ENST000003	.	.	A	G	-	-	0.5	8,0,8,0	chr19,39358	Cytosol	A > G
chr19:39358803:T:C:UTR3:RINL	chr19	39358803	39358803	T	C	UTR3	RINL	ENST000003	.	.	A	G	-	-	0.08	23,0,2,0	chr19,39358	Cytosol	A > G
chr19:39358804:T:C:UTR3:RINL	chr19	39358804	39358804	T	C	UTR3	RINL	ENST000003	.	.	A	G	-	-	0.24	19,0,6,0	chr19,39358	Cytosol	A > G
chr19:39358857:T:C:UTR3:RINL	chr19	39358857	39358857	T	C	UTR3	RINL	ENST000003	.	.	A	G	-	-	0.11111111	24,0,3,0	chr19,39358	Cytosol	A > G
chr19:39358866:T:C:UTR3:RINL	chr19	39358866	39358866	T	C	UTR3	RINL	ENST000003	.	.	A	G	-	-	0.08	23,0,2,0	chr19,39358	Cytosol	A > G
chr19:39359028:T:C:UTR3:RINL	chr19	39359028	39359028	T	C	UTR3	RINL	ENST000003	.	.	A	G	-	-	0.20833333	19,0,5,0	chr19,39359	Cytosol	A > G
chr19:39359051:T:C:UTR3:RINL	chr19	39359051	39359051	T	C	UTR3	RINL	ENST000003	.	.	A	G	-	-	0.12903226	27,0,4,0	chr19,39359	Cytosol	A > G
chr19:39359055:T:C:UTR3:RINL	chr19	39359055	39359055	T	C	UTR3	RINL	ENST000003	.	.	A	G	-	-	0.48571429	18,0,17,0	chr19,39359	Cytosol	A > G
chr19:39359100:T:C:UTR3:RINL	chr19	39359100	39359100	T	C	UTR3	RINL	ENST000003	.	.	A	G	-	-	0.25	21,0,7,0	chr19,39359	Cytosol	A > G
chr19:39359152:T:C:UTR3:RINL	chr19	39359152	39359152	T	C	UTR3	RINL	ENST000003	.	.	A	G	-	-	0.06896552	27,0,2,0	chr19,39359	Cytosol	A > G
chr19:39359162:T:C:UTR3:RINL	chr19	39359162	39359162	T	C	UTR3	RINL	ENST000003	.	.	A	G	-	-	0.06060606	31,0,2,0	chr19,39359	Cytosol	A > G
chr19:39359229:T:C:UTR3:RINL	chr19	39359229	39359229	T	C	UTR3	RINL	ENST000003	.	.	A	G	-	-	0.19230769	21,0,5,0	chr19,39359	Cytosol	A > G
chr19:39359707:T:C:UTR3:RINL	chr19	39359707	39359707	T	C	UTR3	RINL	ENST000003	.	.	A	G	-	-	0.32	17,0,8,0	chr19,39359	Cytosol	A > G
chr19:39359713:T:C:UTR3:RINL	chr19	39359713	39359713	T	C	UTR3	RINL	ENST000003	.	.	A	G	-	-	0.08108108	34,0,3,0	chr19,39359	Cytosol	A > G
chr19:39917874:A:G:ncRNA_exoni	chr19	39917874	39917874	A	G	ncRNA_exon	CTB-60E11.4	.	.	.	A	G	+	+	0.22222222	7,0,2,0	chr19,39917	Cytosol	A > G
chr19:39918031:A:G:ncRNA_exoni	chr19	39918031	39918031	A	G	ncRNA_exon	CTB-60E11.4	.	.	.	A	G	+	+	0.5	4,0,4,0	chr19,39918	Cytosol	A > G
chr19:39918033:A:G:ncRNA_exoni	chr19	39918033	39918033	A	G	ncRNA_exon	CTB-60E11.4	.	.	.	A	G	+	+	1	0,0,7,0	chr19,39918	Cytosol	A > G
chr19:39918145:A:G:ncRNA_exoni	chr19	39918145	39918145	A	G	ncRNA_exon	CTB-60E11.4	.	.	.	A	G	+	+	1	0,0,10,0	chr19,39918	Cytosol	A > G
chr19:39918148:A:G:ncRNA_exoni	chr19	39918148	39918148	A	G	ncRNA_exon	CTB-60E11.4	.	.	.	A	G	+	+	0.625	3,0,5,0	chr19,39918	Cytosol	A > G
chr19:39918204:A:G:ncRNA_exoni	chr19	39918204	39918204	A	G	ncRNA_exon	CTB-60E11.4	.	.	.	A	G	+	+	0.5	3,0,3,0	chr19,39918	Cytosol	A > G

chr19:39918209:A:G:ncRNA_exoni	chr19	39918209	39918209	A	G	ncRNA_exon	CTB-60E11.4	.	.	.	A	G	+	+	0.5	3,0,3,0	chr19,39918	Cytosol	A > G
chr19:39918217:A:G:ncRNA_exoni	chr19	39918217	39918217	A	G	ncRNA_exon	CTB-60E11.4	.	.	.	A	G	+	+	0.8	1,0,4,0	chr19,39918	Cytosol	A > G
chr19:39918230:A:G:ncRNA_exoni	chr19	39918230	39918230	A	G	ncRNA_exon	CTB-60E11.4	.	.	.	A	G	+	+	0.5	4,0,4,0	chr19,39918	Cytosol	A > G
chr19:39981091:A:G:intronic:TIM	chr19	39981091	39981091	A	G	intronic	TIMM50	.	.	.	A	G	+	+	0.15217391	39,0,7,0	chr19,39981	Cytosol	A > G
chr19:39981168:A:G:intronic:TIM	chr19	39981168	39981168	A	G	intronic	TIMM50	.	.	.	A	G	+	+	0.44067797	33,0,26,0	chr19,39981	Cytosol	A > G
chr19:39981170:A:G:intronic:TIM	chr19	39981170	39981170	A	G	intronic	TIMM50	.	.	.	A	G	+	+	0.36666667	38,0,22,0	chr19,39981	Cytosol	A > G
chr19:39981250:A:G:intronic:TIM	chr19	39981250	39981250	A	G	intronic	TIMM50	.	.	.	A	G	+	+	0.4047619	25,0,17,0	chr19,39981	Cytosol	A > G
chr19:39981265:A:G:intronic:TIM	chr19	39981265	39981265	A	G	intronic	TIMM50	.	.	.	A	G	+	+	0.45	22,0,18,0	chr19,39981	Cytosol	A > G
chr19:39981291:A:G:intronic:TIM	chr19	39981291	39981291	A	G	intronic	TIMM50	.	.	.	A	G	+	+	0.65116279	15,0,28,0	chr19,39981	Cytosol	A > G
chr19:39981292:A:G:intronic:TIM	chr19	39981292	39981292	A	G	intronic	TIMM50	.	.	.	A	G	+	+	0.92857143	3,0,39,0	chr19,39981	Cytosol	A > G
chr19:39981313:A:G:intronic:TIM	chr19	39981313	39981313	A	G	intronic	TIMM50	.	.	.	A	G	+	+	0.06451613	29,0,2,0	chr19,39981	Cytosol	A > G
chr19:39981412:A:G:intronic:TIM	chr19	39981412	39981412	A	G	intronic	TIMM50	.	.	.	A	G	+	+	0.125	14,0,2,0	chr19,39981	Cytosol	A > G
chr19:39981442:A:G:intronic:TIM	chr19	39981442	39981442	A	G	intronic	TIMM50	.	.	.	A	G	+	+	0.4	6,0,4,0	chr19,39981	Cytosol	A > G
chr19:39981461:A:G:intronic:TIM	chr19	39981461	39981461	A	G	intronic	TIMM50	.	.	.	A	G	+	+	0.33333333	6,0,3,0	chr19,39981	Cytosol	A > G
chr19:39981551:A:G:intronic:TIM	chr19	39981551	39981551	A	G	intronic	TIMM50	.	.	.	A	G	+	+	0.33333333	4,0,2,0	chr19,39981	Cytosol	A > G
chr19:39981615:A:G:intronic:TIM	chr19	39981615	39981615	A	G	intronic	TIMM50	.	.	.	A	G	+	+	0.4	3,0,2,0	chr19,39981	Cytosol	A > G
chr19:39981907:A:G:intronic:TIM	chr19	39981907	39981907	A	G	intronic	TIMM50	.	.	.	A	G	+	+	0.33333333	4,0,2,0	chr19,39981	Cytosol	A > G
chr19:39981924:A:G:intronic:TIM	chr19	39981924	39981924	A	G	intronic	TIMM50	.	.	.	A	G	+	+	0.4	3,0,2,0	chr19,39981	Cytosol	A > G
chr19:39982204:A:G:intronic:TIM	chr19	39982204	39982204	A	G	intronic	TIMM50	.	.	.	A	G	+	+	0.31428571	48,0,22,0	chr19,39982	Cytosol	A > G
chr19:39982205:A:G:intronic:TIM	chr19	39982205	39982205	A	G	intronic	TIMM50	.	.	.	A	G	+	+	0.28358209	48,0,19,0	chr19,39982	Cytosol	A > G
chr19:39982228:A:G:intronic:TIM	chr19	39982228	39982228	A	G	intronic	TIMM50	.	.	.	A	G	+	+	0.24528302	40,0,13,0	chr19,39982	Cytosol	A > G
chr19:39982231:A:G:intronic:TIM	chr19	39982231	39982231	A	G	intronic	TIMM50	.	.	.	A	G	+	+	0.38	31,0,19,0	chr19,39982	Cytosol	A > G
chr19:39982256:A:G:intronic:TIM	chr19	39982256	39982256	A	G	intronic	TIMM50	.	.	.	A	G	+	+	0.21875	50,0,14,0	chr19,39982	Cytosol	A > G
chr19:39982430:A:G:intronic:TIM	chr19	39982430	39982430	A	G	intronic	TIMM50	.	.	.	A	G	+	+	0.5	6,0,6,0	chr19,39982	Cytosol	A > G
chr19:39982592:A:G:UTR3:TIMM5	chr19	39982592	39982592	A	G	UTR3	TIMM50	ENST0000006	.	.	A	G	+	+	0.53571429	13,0,15,0	chr19,39982	Cytosol	A > G
chr19:39982593:A:G:UTR3:TIMM5	chr19	39982593	39982593	A	G	UTR3	TIMM50	ENST0000006	.	.	A	G	+	+	0.30769231	18,0,8,0	chr19,39982	Cytosol	A > G
chr19:39982619:A:G:UTR3:TIMM5	chr19	39982619	39982619	A	G	UTR3	TIMM50	ENST0000006	.	.	A	G	+	+	0.31884058	47,0,22,0	chr19,39982	Cytosol	A > G
chr19:39982626:A:G:UTR3:TIMM5	chr19	39982626	39982626	A	G	UTR3	TIMM50	ENST0000006	.	.	A	G	+	+	0.05263158	72,0,4,0	chr19,39982	Cytosol	A > G
chr19:39983251:A:G:UTR3:TIMM5	chr19	39983251	39983251	A	G	UTR3	TIMM50	ENST0000006	.	.	A	G	+	+	0.10526316	17,0,2,0	chr19,39983	Cytosol	A > G
chr19:39983252:A:G:UTR3:TIMM5	chr19	39983252	39983252	A	G	UTR3	TIMM50	ENST0000006	.	.	A	G	+	+	0.31578947	13,0,6,0	chr19,39983	Cytosol	A > G
chr19:39983321:A:G:UTR3:TIMM5	chr19	39983321	39983321	A	G	UTR3	TIMM50	ENST0000006	.	.	A	G	+	+	0.18181818	18,0,4,0	chr19,39983	Cytosol	A > G
chr19:39983329:A:G:UTR3:TIMM5	chr19	39983329	39983329	A	G	UTR3	TIMM50	ENST0000006	.	.	A	G	+	+	0.16666667	20,0,4,0	chr19,39983	Cytosol	A > G
chr19:39983331:A:G:UTR3:TIMM5	chr19	39983331	39983331	A	G	UTR3	TIMM50	ENST0000006	.	.	A	G	+	+	0.09090909	20,0,2,0	chr19,39983	Cytosol	A > G
chr19:39983360:A:G:UTR3:TIMM5	chr19	39983360	39983360	A	G	UTR3	TIMM50	ENST0000006	.	.	A	G	+	+	0.10526316	17,0,2,0	chr19,39983	Cytosol	A > G
chr19:39983364:A:G:downstream:	chr19	39983364	39983364	A	G	downstream	TIMM50	.	.	.	A	G	+	+	0.25	12,0,4,0	chr19,39983	Cytosol	A > G
chr19:39983390:A:G:downstream:	chr19	39983390	39983390	A	G	downstream	TIMM50	.	.	.	A	G	+	+	0.20833333	19,0,5,0	chr19,39983	Cytosol	A > G
chr19:39983391:A:G:downstream:	chr19	39983391	39983391	A	G	downstream	TIMM50	.	.	.	A	G	+	+	0.41666667	14,0,10,0	chr19,39983	Cytosol	A > G
chr19:39983396:A:G:downstream:	chr19	39983396	39983396	A	G	downstream	TIMM50	.	.	.	A	G	+	+	0.07407407	25,0,2,0	chr19,39983	Cytosol	A > G
chr19:39983452:A:G:downstream:	chr19	39983452	39983452	A	G	downstream	TIMM50	.	.	.	A	G	+	+	0.3125	11,0,5,0	chr19,39983	Cytosol	A > G
chr19:39983466:A:G:downstream:	chr19	39983466	39983466	A	G	downstream	TIMM50	.	.	.	A	G	+	+	0.5	6,0,6,0	chr19,39983	Cytosol	A > G
chr19:39983989:A:G:downstream:	chr19	39983989	39983989	A	G	downstream	TIMM50	.	.	.	A	G	+	+	0.33333333	4,0,2,0	chr19,39983	Cytosol	A > G
chr19:39984007:A:G:downstream:	chr19	39984007	39984007	A	G	downstream	TIMM50	.	.	.	A	G	+	+	0.26666667	11,0,4,0	chr19,39984	Cytosol	A > G
chr19:39984016:A:G:downstream:	chr19	39984016	39984016	A	G	downstream	TIMM50	.	.	.	A	G	+	+	0.31578947	13,0,6,0	chr19,39984	Cytosol	A > G
chr19:39984019:A:G:downstream:	chr19	39984019	39984019	A	G	downstream	TIMM50	.	.	.	A	G	+	+	0.35294118	11,0,6,0	chr19,39984	Cytosol	A > G
chr19:40537162:T:C:UTR3:ZNF780	chr19	40537162	40537162	T	C	UTR3	ZNF780B	ENST000004	.	.	A	G	-	-	0.21428571	11,0,3,0	chr19,40537	Cytosol	A > G
chr19:40537163:T:C:UTR3:ZNF780	chr19	40537163	40537163	T	C	UTR3	ZNF780B	ENST000004	.	.	A	G	-	-	0.92857143	1,0,13,0	chr19,40537	Cytosol	A > G
chr19:40537196:T:C:UTR3:ZNF780	chr19	40537196	40537196	T	C	UTR3	ZNF780B	ENST000004	.	.	A	G	-	-	1	0,0,12,0	chr19,40537	Cytosol	A > G
chr19:40537305:T:C:UTR3:ZNF780	chr19	40537305	40537305	T	C	UTR3	ZNF780B	ENST000004	.	.	A	G	-	-	0.25	6,0,2,0	chr19,40537	Cytosol	A > G
chr19:40537306:T:C:UTR3:ZNF780	chr19	40537306	40537306	T	C	UTR3	ZNF780B	ENST000004	.	.	A	G	-	-	0.57142857	3,0,4,0	chr19,40537	Cytosol	A > G
chr19:40537308:T:C:UTR3:ZNF780	chr19	40537308	40537308	T	C	UTR3	ZNF780B	ENST000004	.	.	A	G	-	-	0.75	2,0,6,0	chr19,40537	Cytosol	A > G
chr19:40537317:T:C:UTR3:ZNF780	chr19	40537317	40537317	T	C	UTR3	ZNF780B	ENST000004	.	.	A	G	-	-	0.33333333	4,0,2,0	chr19,40537	Cytosol	A > G
chr19:41336165:A:G:intronic:CTC-	chr19	41336165	41336165	A	G	intronic	CTC-490E21.	.	.	.	A	G	+	+	0.4	3,0,2,0	chr19,41336	Cytosol	A > G
chr19:41336219:A:G:intronic:CTC-	chr19	41336219	41336219	A	G	intronic	CTC-490E21.	.	.	.	A	G	+	+	0.71428571	2,0,5,0	chr19,41336	Cytosol	A > G
chr19:42375554:A:G:UTR3:RPS19	chr19	42375554	42375554	A	G	UTR3	RPS19	ENST000005	.	.	A	G	+	+	0.19318182	71,0,17,0	chr19,42375	Cytosol	A > G
chr19:42375584:A:G:UTR3:RPS19	chr19	42375584	42375584	A	G	UTR3	RPS19	ENST000005	.	.	A	G	+	+	0.61111111	35,0,55,0	chr19,42375	Cytosol	A > G
chr19:42375585:A:G:UTR3:RPS19	chr19	42375585	42375585	A	G	UTR3	RPS19	ENST000005	.	.	A	G	+	+	0.0212766	92,0,2,0	chr19,42375	Cytosol	A > G
chr19:42375602:A:G:UTR3:RPS19	chr19	42375602	42375602	A	G	UTR3	RPS19	ENST000005	.	.	A	G	+	+	0.38095238	52,0,32,0	chr19,42375	Cytosol	A > G
chr19:42375612:A:G:UTR3:RPS19	chr19	42375612	42375612	A	G	UTR3	RPS19	ENST000005	.	.	A	G	+	+	0.35227273	57,0,31,0	chr19,42375	Cytosol	A > G
chr19:42375624:A:G:UTR3:RPS19	chr19	42375624	42375624	A	G	UTR3	RPS19	ENST000005	.	.	A	G	+	+	0.64516129	33,0,60,0	chr19,42375	Cytosol	A > G
chr19:42375627:A:G:UTR3:RPS19	chr19	42375627	42375627	A	G	UTR3	RPS19	ENST000005	.	.	A	G	+	+	0.43137255	58,0,44,0	chr19,42375	Cytosol	A > G
chr19:42376702:A:G:UTR3:RPS19	chr19	42376702	42376702	A	G	UTR3	RPS19	ENST000005	.	.	A	G	+	+	0.2260274	113,0,33,0	chr19,42376	Cytosol	A > G
chr19:42376710:A:G:UTR3:RPS19	chr19	42376710	42376710	A	G	UTR3	RPS19	ENST000005	.	.	A	G	+	+	0.37671233	91,0,55,0	chr19,42376	Cytosol	A > G
chr19:42376711:A:G:UTR3:RPS19	chr19	42376711	42376711	A	G	UTR3	RPS19	ENST000005	.	.	A	G	+	+	0.35172414	94,0,51,0	chr19,42376	Cytosol	A > G
chr19:42376717:A:G:UTR3:RPS19	chr19	42376717	42376717	A	G	UTR3	RPS19	ENST000005	.	.	A	G	+	+	0.07751938	119,0,10,0	chr19,42376	Cytosol	A > G

chr19:42376842:A:G:UTR3:RPS19	chr19	42376842	42376842	A	G	UTR3	RPS19	ENST000005	.	.	A	G	+	+	0.33783784	49,0,25,0	chr19,42376	Cytosol	A > G
chr19:42376843:A:G:UTR3:RPS19	chr19	42376843	42376843	A	G	UTR3	RPS19	ENST000005	.	.	A	G	+	+	0.65217391	24,0,45,0	chr19,42376	Cytosol	A > G
chr19:42731763:A:G:UTR3:ZNF526	chr19	42731763	42731763	A	G	UTR3	ZNF526	ENST000003	.	.	A	G	+	+	0.28571429	5,0,2,0	chr19,42731	Cytosol	A > G
chr19:42732827:A:G:downstream:	chr19	42732827	42732827	A	G	downstream	ZNF526	.	.	.	A	G	+		0.2	8,0,2,0	chr19,42732	Cytosol	A > G
chr19:42732933:A:G:downstream:	chr19	42732933	42732933	A	G	downstream	ZNF526	.	.	.	A	G	+		0.6	2,0,3,0	chr19,42732	Cytosol	A > G
chr19:42732939:A:G:downstream:	chr19	42732939	42732939	A	G	downstream	ZNF526	.	.	.	A	G	+		0.33333333	4,0,2,0	chr19,42732	Cytosol	A > G
chr19:42735437:A:G:intronic:GSK3	chr19	42735437	42735437	A	G	intronic	GSK3A	.	.	.	A	G	+	-	0.21428571	11,0,3,0	chr19,42735	Cytosol	A > G
chr19:42881818:A:G:UTR3:MEGF8	chr19	42881818	42881818	A	G	UTR3	MEGF8	ENST000003	.	.	A	G	+	+	0.05714286	33,0,2,0	chr19,42881	Cytosol	A > G
chr19:44354791:A:G:intergenic:ZN	chr19	44354791	44354791	A	G	intergenic	ZNF283,ZNF4	dist=1484;dis	.	.	A	G	+		0.66666667	2,0,4,0	chr19,44354	Cytosol	A > G
chr19:44663395:A:G:downstream:	chr19	44663395	44663395	A	G	downstream	ZNF234	.	.	.	A	G	+	+	0.5	3,0,3,0	chr19,44663	Cytosol	A > G
chr19:44663410:A:G:downstream:	chr19	44663410	44663410	A	G	downstream	ZNF234	.	.	.	A	G	+	+	0.33333333	4,0,2,0	chr19,44663	Cytosol	A > G
chr19:44663424:A:G:downstream:	chr19	44663424	44663424	A	G	downstream	ZNF234	.	.	.	A	G	+	+	0.5	4,0,4,0	chr19,44663	Cytosol	A > G
chr19:44663469:A:G:downstream:	chr19	44663469	44663469	A	G	downstream	ZNF234	.	.	.	A	G	+	+	0.8	1,0,4,0	chr19,44663	Cytosol	A > G
chr19:44663518:A:G:downstream:	chr19	44663518	44663518	A	G	downstream	ZNF234	.	.	.	A	G	+	+	0.8	1,0,4,0	chr19,44663	Cytosol	A > G
chr19:44663519:A:G:downstream:	chr19	44663519	44663519	A	G	downstream	ZNF234	.	.	.	A	G	+	+	0.5	3,0,3,0	chr19,44663	Cytosol	A > G
chr19:44732262:T:C:intronic:ZNF2	chr19	44732262	44732262	T	C	intronic	ZNF227	.	.	.	A	G	-	+	0.33333333	4,0,2,0	chr19,44732	Cytosol	A > G
chr19:45167078:A:G:ncRNA_intro	chr19	45167078	45167078	A	G	ncRNA_intro	CTB-171A8.1	.	.	.	A	G	+	-+	0.13333333	26,0,4,0	chr19,45167	Cytosol	A > G
chr19:45167091:A:G:ncRNA_intro	chr19	45167091	45167091	A	G	ncRNA_intro	CTB-171A8.1	.	.	.	A	G	+	-+	0.58823529	14,0,20,0	chr19,45167	Cytosol	A > G
chr19:45167122:A:G:ncRNA_intro	chr19	45167122	45167122	A	G	ncRNA_intro	CTB-171A8.1	.	.	.	A	G	+	-+	0.3125	22,0,10,0	chr19,45167	Cytosol	A > G
chr19:45167742:A:G:ncRNA_intro	chr19	45167742	45167742	A	G	ncRNA_intro	CTB-171A8.1	.	.	.	A	G	+	-+	0.14285714	18,0,3,0	chr19,45167	Cytosol	A > G
chr19:45167748:A:G:ncRNA_intro	chr19	45167748	45167748	A	G	ncRNA_intro	CTB-171A8.1	.	.	.	A	G	+	-+	0.31818182	15,0,7,0	chr19,45167	Cytosol	A > G
chr19:45168725:A:G:ncRNA_intro	chr19	45168725	45168725	A	G	ncRNA_intro	CTB-171A8.1	.	.	.	A	G	+	-+	0.08333333	22,0,2,0	chr19,45168	Cytosol	A > G
chr19:45168780:A:G:ncRNA_intro	chr19	45168780	45168780	A	G	ncRNA_intro	CTB-171A8.1	.	.	.	A	G	+	-+	0.75	5,0,15,0	chr19,45168	Cytosol	A > G
chr19:45168781:A:G:ncRNA_intro	chr19	45168781	45168781	A	G	ncRNA_intro	CTB-171A8.1	.	.	.	A	G	+	-+	0.52173913	11,0,12,0	chr19,45168	Cytosol	A > G
chr19:45168790:A:G:ncRNA_intro	chr19	45168790	45168790	A	G	ncRNA_intro	CTB-171A8.1	.	.	.	A	G	+	-+	0.42857143	16,0,12,0	chr19,45168	Cytosol	A > G
chr19:45168822:A:G:ncRNA_intro	chr19	45168822	45168822	A	G	ncRNA_intro	CTB-171A8.1	.	.	.	A	G	+	-+	0.33333333	18,0,9,0	chr19,45168	Cytosol	A > G
chr19:45168850:A:G:ncRNA_intro	chr19	45168850	45168850	A	G	ncRNA_intro	CTB-171A8.1	.	.	.	A	G	+	-+	0.12	22,0,3,0	chr19,45168	Cytosol	A > G
chr19:46052831:T:C:UTR3:OPA3	chr19	46052831	46052831	T	C	UTR3	OPA3	ENST000002	.	.	A	G	-	-	0.24390244	31,0,10,0	chr19,46052	Cytosol	A > G
chr19:46052915:T:C:UTR3:OPA3	chr19	46052915	46052915	T	C	UTR3	OPA3	ENST000002	.	.	A	G	-	-	0.06349206	59,0,4,0	chr19,46052	Cytosol	A > G
chr19:46052917:T:C:UTR3:OPA3	chr19	46052917	46052917	T	C	UTR3	OPA3	ENST000002	.	.	A	G	-	-	0.04761905	60,0,3,0	chr19,46052	Cytosol	A > G
chr19:46052931:T:C:UTR3:OPA3	chr19	46052931	46052931	T	C	UTR3	OPA3	ENST000002	.	.	A	G	-	-	0.28333333	43,0,17,0	chr19,46052	Cytosol	A > G
chr19:46052975:T:C:UTR3:OPA3	chr19	46052975	46052975	T	C	UTR3	OPA3	ENST000002	.	.	A	G	-	-	0.25641026	58,0,20,0	chr19,46052	Cytosol	A > G
chr19:46053159:T:C:UTR3:OPA3	chr19	46053159	46053159	T	C	UTR3	OPA3	ENST000002	.	.	A	G	-	-	0.2	20,0,5,0	chr19,46053	Cytosol	A > G
chr19:46053172:T:C:UTR3:OPA3	chr19	46053172	46053172	T	C	UTR3	OPA3	ENST000002	.	.	A	G	-	-	0.23809524	16,0,5,0	chr19,46053	Cytosol	A > G
chr19:46053194:T:C:UTR3:OPA3	chr19	46053194	46053194	T	C	UTR3	OPA3	ENST000002	.	.	A	G	-	-	0.26666667	22,0,8,0	chr19,46053	Cytosol	A > G
chr19:46053195:T:C:UTR3:OPA3	chr19	46053195	46053195	T	C	UTR3	OPA3	ENST000002	.	.	A	G	-	-	0.42307692	15,0,11,0	chr19,46053	Cytosol	A > G
chr19:46053232:T:C:UTR3:OPA3	chr19	46053232	46053232	T	C	UTR3	OPA3	ENST000002	.	.	A	G	-	-	0.63829787	17,0,30,0	chr19,46053	Cytosol	A > G
chr19:46053253:T:C:UTR3:OPA3	chr19	46053253	46053253	T	C	UTR3	OPA3	ENST000002	.	.	A	G	-	-	0.12280702	50,0,7,0	chr19,46053	Cytosol	A > G
chr19:46053266:T:C:UTR3:OPA3	chr19	46053266	46053266	T	C	UTR3	OPA3	ENST000002	.	.	A	G	-	-	0.2295082	47,0,14,0	chr19,46053	Cytosol	A > G
chr19:46053267:T:C:UTR3:OPA3	chr19	46053267	46053267	T	C	UTR3	OPA3	ENST000002	.	.	A	G	-	-	0.66129032	21,0,41,0	chr19,46053	Cytosol	A > G
chr19:46053294:T:C:UTR3:OPA3	chr19	46053294	46053294	T	C	UTR3	OPA3	ENST000002	.	.	A	G	-	-	0.06493506	72,0,5,0	chr19,46053	Cytosol	A > G
chr19:46053316:T:C:UTR3:OPA3	chr19	46053316	46053316	T	C	UTR3	OPA3	ENST000002	.	.	A	G	-	-	0.31884058	47,0,22,0	chr19,46053	Cytosol	A > G
chr19:46053340:T:C:UTR3:OPA3	chr19	46053340	46053340	T	C	UTR3	OPA3	ENST000002	.	.	A	G	-	-	0.3625	51,0,29,0	chr19,46053	Cytosol	A > G
chr19:46053359:T:C:UTR3:OPA3	chr19	46053359	46053359	T	C	UTR3	OPA3	ENST000002	.	.	A	G	-	-	0.2125	63,0,17,0	chr19,46053	Cytosol	A > G
chr19:46053369:T:C:UTR3:OPA3	chr19	46053369	46053369	T	C	UTR3	OPA3	ENST000002	.	.	A	G	-	-	0.17948718	64,0,14,0	chr19,46053	Cytosol	A > G
chr19:46053370:T:C:UTR3:OPA3	chr19	46053370	46053370	T	C	UTR3	OPA3	ENST000002	.	.	A	G	-	-	0.02739726	71,0,2,0	chr19,46053	Cytosol	A > G
chr19:46054357:T:C:UTR3:OPA3	chr19	46054357	46054357	T	C	UTR3	OPA3	ENST000002	.	.	A	G	-	-	0.17647059	28,0,6,0	chr19,46054	Cytosol	A > G
chr19:46054421:T:C:UTR3:OPA3	chr19	46054421	46054421	T	C	UTR3	OPA3	ENST000002	.	.	A	G	-	-	0.09230769	59,0,6,0	chr19,46054	Cytosol	A > G
chr19:46054503:T:C:UTR3:OPA3	chr19	46054503	46054503	T	C	UTR3	OPA3	ENST000002	.	.	A	G	-	-	0.09090909	50,0,5,0	chr19,46054	Cytosol	A > G
chr19:46055761:T:C:UTR3:OPA3	chr19	46055761	46055761	T	C	UTR3	OPA3	ENST000002	.	.	A	G	-	-	0.12121212	29,0,4,0	chr19,46055	Cytosol	A > G
chr19:46055912:T:C:UTR3:OPA3	chr19	46055912	46055912	T	C	UTR3	OPA3	ENST000002	.	.	A	G	-	-	0.06666667	28,0,2,0	chr19,46055	Cytosol	A > G
chr19:46123227:T:C:intronic:EML2	chr19	46123227	46123227	T	C	intronic	EML2	.	.	.	A	G	-	-	0.4	3,0,2,0	chr19,46123	Cytosol	A > G
chr19:46192675:T:C:intronic:SNRP	chr19	46192675	46192675	T	C	intronic	SNRPD2	.	.	.	A	G	-	-	0.66666667	2,0,4,0	chr19,46192	Cytosol	A > G
chr19:46192746:T:C:intronic:SNRP	chr19	46192746	46192746	T	C	intronic	SNRPD2	.	.	.	A	G	-	-	0.8	1,0,4,0	chr19,46192	Cytosol	A > G
chr19:46206728:A:G:UTR3:QPCTL	chr19	46206728	46206728	A	G	UTR3	QPCTL	ENST000000	.	.	A	G	+	+	0.88888889	2,0,16,0	chr19,46206	Cytosol	A > G
chr19:46206753:A:G:UTR3:QPCTL	chr19	46206753	46206753	A	G	UTR3	QPCTL	ENST000000	.	.	A	G	+	+	0.77777778	4,0,14,0	chr19,46206	Cytosol	A > G
chr19:46206769:A:G:UTR3:QPCTL	chr19	46206769	46206769	A	G	UTR3	QPCTL	ENST000000	.	.	A	G	+	+	0.52380952	10,0,11,0	chr19,46206	Cytosol	A > G
chr19:46206794:A:G:UTR3:QPCTL	chr19	46206794	46206794	A	G	UTR3	QPCTL	ENST000000	.	.	A	G	+	+	1	0,0,29,0	chr19,46206	Cytosol	A > G
chr19:46206848:A:G:UTR3:QPCTL	chr19	46206848	46206848	A	G	UTR3	QPCTL	ENST000000	.	.	A	G	+	+	0.35294118	22,0,12,0	chr19,46206	Cytosol	A > G
chr19:46206849:A:G:UTR3:QPCTL	chr19	46206849	46206849	A	G	UTR3	QPCTL	ENST000000	.	.	A	G	+	+	0.58064516	13,0,18,0	chr19,46206	Cytosol	A > G
chr19:46206861:A:G:UTR3:QPCTL	chr19	46206861	46206861	A	G	UTR3	QPCTL	ENST000000	.	.	A	G	+	+	0.55555556	12,0,15,0	chr19,46206	Cytosol	A > G
chr19:46206955:A:G:UTR3:QPCTL	chr19	46206955	46206955	A	G	UTR3	QPCTL	ENST000000	.	.	A	G	+	+	0.91358025	7,0,74,0	chr19,46206	Cytosol	A > G
chr19:46206956:A:G:UTR3:QPCTL	chr19	46206956	46206956	A	G	UTR3	QPCTL	ENST000000	.	.	A	G	+	+	0.28947368	54,0,22,0	chr19,46206	Cytosol	A > G
chr19:46206962:A:G:UTR3:QPCTL	chr19	46206962	46206962	A	G	UTR3	QPCTL	ENST000000	.	.	A	G	+	+	0.15068493	62,0,11,0	chr19,46206	Cytosol	A > G

chr19:46207024:A:G:UTR3:QPCTL	chr19	46207024	46207024	A	G	UTR3	QPCTL	ENST000000	.	.	A	G	+	+	0.33333333	42,0,21,0	chr19,46207	Cytosol	A > G
chr19:46207025:A:G:UTR3:QPCTL	chr19	46207025	46207025	A	G	UTR3	QPCTL	ENST000000	.	.	A	G	+	+	0.82539683	11,0,52,0	chr19,46207	Cytosol	A > G
chr19:46207034:A:G:UTR3:QPCTL	chr19	46207034	46207034	A	G	UTR3	QPCTL	ENST000000	.	.	A	G	+	+	0.34210526	25,0,13,0	chr19,46207	Cytosol	A > G
chr19:46207035:A:G:UTR3:QPCTL	chr19	46207035	46207035	A	G	UTR3	QPCTL	ENST000000	.	.	A	G	+	+	0.21875	25,0,7,0	chr19,46207	Cytosol	A > G
chr19:46207049:A:G:UTR3:QPCTL	chr19	46207049	46207049	A	G	UTR3	QPCTL	ENST000000	.	.	A	G	+	+	0.27777778	13,0,5,0	chr19,46207	Cytosol	A > G
chr19:46207064:A:G:UTR3:QPCTL	chr19	46207064	46207064	A	G	UTR3	QPCTL	ENST000000	.	.	A	G	+	+	0.63636364	4,0,7,0	chr19,46207	Cytosol	A > G
chr19:46207090:A:G:UTR3:QPCTL	chr19	46207090	46207090	A	G	UTR3	QPCTL	ENST000000	.	.	A	G	+	+	1	0,0,8,0	chr19,46207	Cytosol	A > G
chr19:46207091:A:G:UTR3:QPCTL	chr19	46207091	46207091	A	G	UTR3	QPCTL	ENST000000	.	.	A	G	+	+	0.75	2,0,6,0	chr19,46207	Cytosol	A > G
chr19:46207160:A:G:UTR3:QPCTL	chr19	46207160	46207160	A	G	UTR3	QPCTL	ENST000000	.	.	A	G	+	+	0.8	1,0,4,0	chr19,46207	Cytosol	A > G
chr19:46207161:A:G:UTR3:QPCTL	chr19	46207161	46207161	A	G	UTR3	QPCTL	ENST000000	.	.	A	G	+	+	0.6	2,0,3,0	chr19,46207	Cytosol	A > G
chr19:46207166:A:G:UTR3:QPCTL	chr19	46207166	46207166	A	G	UTR3	QPCTL	ENST000000	.	.	A	G	+	+	1	0,0,5,0	chr19,46207	Cytosol	A > G
chr19:48753098:T:C:UTR5:CARD8	chr19	48753098	48753098	T	C	UTR5	CARD8	ENST000005	.	.	A	G	-	+-	0.10714286	25,0,3,0	chr19,48753	Cytosol	A > G
chr19:48956653:A:G:UTR3:GRWD1	chr19	48956653	48956653	A	G	UTR3	GRWD1	ENST000002	.	.	A	G	+	+	0.19277108	67,0,16,0	chr19,48956	Cytosol	A > G
chr19:48961806:A:G:intronic:KCNJ	chr19	48961806	48961806	A	G	intronic	KCNJ14	.	.	.	A	G	+	+	0.22222222	14,0,4,0	chr19,48961	Cytosol	A > G
chr19:48962032:A:G:intronic:KCNJ	chr19	48962032	48962032	A	G	intronic	KCNJ14	.	.	.	A	G	+	+	0.53333333	7,0,8,0	chr19,48962	Cytosol	A > G
chr19:48962073:A:G:intronic:KCNJ	chr19	48962073	48962073	A	G	intronic	KCNJ14	.	.	.	A	G	+	+	0.44444444	5,0,4,0	chr19,48962	Cytosol	A > G
chr19:48962700:A:G:intronic:KCNJ	chr19	48962700	48962700	A	G	intronic	KCNJ14	.	.	.	A	G	+	+	0.33333333	10,0,5,0	chr19,48962	Cytosol	A > G
chr19:48962764:A:G:intronic:KCNJ	chr19	48962764	48962764	A	G	intronic	KCNJ14	.	.	.	A	G	+	+	0.14285714	18,0,3,0	chr19,48962	Cytosol	A > G
chr19:48962770:A:G:intronic:KCNJ	chr19	48962770	48962770	A	G	intronic	KCNJ14	.	.	.	A	G	+	+	0.46153846	14,0,12,0	chr19,48962	Cytosol	A > G
chr19:48962889:A:G:intronic:KCNJ	chr19	48962889	48962889	A	G	intronic	KCNJ14	.	.	.	A	G	+	+	0.22727273	17,0,5,0	chr19,48962	Cytosol	A > G
chr19:48962921:A:G:intronic:KCNJ	chr19	48962921	48962921	A	G	intronic	KCNJ14	.	.	.	A	G	+	+	0.5	12,0,12,0	chr19,48962	Cytosol	A > G
chr19:48963165:A:G:intronic:KCNJ	chr19	48963165	48963165	A	G	intronic	KCNJ14	.	.	.	A	G	+	+	0.75	3,0,9,0	chr19,48963	Cytosol	A > G
chr19:48963166:A:G:intronic:KCNJ	chr19	48963166	48963166	A	G	intronic	KCNJ14	.	.	.	A	G	+	+	0.81818182	2,0,9,0	chr19,48963	Cytosol	A > G
chr19:48963218:A:G:intronic:KCNJ	chr19	48963218	48963218	A	G	intronic	KCNJ14	.	.	.	A	G	+	+	0.41176471	10,0,7,0	chr19,48963	Cytosol	A > G
chr19:49426814:A:G:downstream:	chr19	49426814	49426814	A	G	downstream	NUCB1	.	.	.	A	G	+		0.8	1,0,4,0	chr19,49426	Cytosol	A > G
chr19:49426934:A:G:downstream:	chr19	49426934	49426934	A	G	downstream	NUCB1	.	.	.	A	G	+		0.5	3,0,3,0	chr19,49426	Cytosol	A > G
chr19:49426949:A:G:downstream:	chr19	49426949	49426949	A	G	downstream	NUCB1	.	.	.	A	G	+		0.57142857	3,0,4,0	chr19,49426	Cytosol	A > G
chr19:49426954:A:G:downstream:	chr19	49426954	49426954	A	G	downstream	NUCB1	.	.	.	A	G	+		1	0,0,8,0	chr19,49426	Cytosol	A > G
chr19:49427113:A:G:downstream:	chr19	49427113	49427113	A	G	downstream	NUCB1	.	.	.	A	G	+		0.36363636	7,0,4,0	chr19,49427	Cytosol	A > G
chr19:49428376:A:G:intergenic:NU	chr19	49428376	49428376	A	G	intergenic	NUCB1,CTD-	dist=1747;dis	.	.	A	G	+		0.6	2,0,3,0	chr19,49428	Cytosol	A > G
chr19:50024985:A:G:UTR3:FCGRT	chr19	50024985	50024985	A	G	UTR3	FCGRT	ENST000005	.	.	A	G	+	+	0.4	3,0,2,0	chr19,50024	Cytosol	A > G
chr19:50025055:A:G:UTR3:FCGRT	chr19	50025055	50025055	A	G	UTR3	FCGRT	ENST000005	.	.	A	G	+	+	0.33333333	4,0,2,0	chr19,50025	Cytosol	A > G
chr19:51305854:T:C:UTR5:C19orf4	chr19	51305854	51305854	T	C	UTR5	C19orf48	ENST000005	.	.	A	G	-	-	0.55555556	4,0,5,0	chr19,51305	Cytosol	A > G
chr19:51305859:T:C:UTR5:C19orf4	chr19	51305859	51305859	T	C	UTR5	C19orf48	ENST000005	.	.	A	G	-	-	0.33333333	4,0,2,0	chr19,51305	Cytosol	A > G
chr19:52535088:T:C:UTR3:ZNF432	chr19	52535088	52535088	T	C	UTR3	ZNF432	ENST000005	.	.	A	G	-	-	0.28571429	5,0,2,0	chr19,52535	Cytosol	A > G
chr19:52535151:T:C:UTR3:ZNF432	chr19	52535151	52535151	T	C	UTR3	ZNF432	ENST000005	.	.	A	G	-	-	0.26666667	11,0,4,0	chr19,52535	Cytosol	A > G
chr19:52535206:T:C:UTR3:ZNF432	chr19	52535206	52535206	T	C	UTR3	ZNF432	ENST000005	.	.	A	G	-	-	0.42857143	4,0,3,0	chr19,52535	Cytosol	A > G
chr19:52535209:T:C:UTR3:ZNF432	chr19	52535209	52535209	T	C	UTR3	ZNF432	ENST000005	.	.	A	G	-	-	0.875	1,0,7,0	chr19,52535	Cytosol	A > G
chr19:52535804:T:C:UTR3:ZNF432	chr19	52535804	52535804	T	C	UTR3	ZNF432	ENST000005	.	.	A	G	-	-	0.375	5,0,3,0	chr19,52535	Cytosol	A > G
chr19:52535805:T:C:UTR3:ZNF432	chr19	52535805	52535805	T	C	UTR3	ZNF432	ENST000005	.	.	A	G	-	-	0.57142857	3,0,4,0	chr19,52535	Cytosol	A > G
chr19:52535865:T:C:UTR3:ZNF432	chr19	52535865	52535865	T	C	UTR3	ZNF432	ENST000005	.	.	A	G	-	-	0.25	6,0,2,0	chr19,52535	Cytosol	A > G
chr19:52535866:T:C:UTR3:ZNF432	chr19	52535866	52535866	T	C	UTR3	ZNF432	ENST000005	.	.	A	G	-	-	0.3	7,0,3,0	chr19,52535	Cytosol	A > G
chr19:52535867:T:C:UTR3:ZNF432	chr19	52535867	52535867	T	C	UTR3	ZNF432	ENST000005	.	.	A	G	-	-	0.25	6,0,2,0	chr19,52535	Cytosol	A > G
chr19:52535870:T:C:UTR3:ZNF432	chr19	52535870	52535870	T	C	UTR3	ZNF432	ENST000005	.	.	A	G	-	-	0.88888889	1,0,8,0	chr19,52535	Cytosol	A > G
chr19:53065005:A:G:intronic:ZNF8	chr19	53065005	53065005	A	G	intronic	ZNF808	.	.	.	A	G	+	+	0.85714286	1,0,6,0	chr19,53065	Cytosol	A > G
chr19:53065338:A:G:intronic:ZNF8	chr19	53065338	53065338	A	G	intronic	ZNF808	.	.	.	A	G	+	+	0.4	3,0,2,0	chr19,53065	Cytosol	A > G
chr19:53065540:A:G:intronic:ZNF8	chr19	53065540	53065540	A	G	intronic	ZNF808	.	.	.	A	G	+	+	0.28571429	5,0,2,0	chr19,53065	Cytosol	A > G
chr19:53065580:A:G:intronic:ZNF8	chr19	53065580	53065580	A	G	intronic	ZNF808	.	.	.	A	G	+	+	0.28571429	5,0,2,0	chr19,53065	Cytosol	A > G
chr19:53087633:A:G:ncRNA_intro	chr19	53087633	53087633	A	G	ncRNA_intro	CTD-3099C6.	.	.	.	A	G	+	+-	0.71428571	2,0,5,0	chr19,53087	Cytosol	A > G
chr19:53102126:A:G:ncRNA_exon	chr19	53102126	53102126	A	G	ncRNA_exon	CTD-3099C6.	.	.	.	A	G	+	+-	0.15384615	11,0,2,0	chr19,53102	Cytosol	A > G
chr19:53146331:T:C:intronic:ZNF8	chr19	53146331	53146331	T	C	intronic	ZNF83	.	.	.	A	G	-	-	0.4	6,0,4,0	chr19,53146	Cytosol	A > G
chr19:53149278:T:C:intronic:ZNF8	chr19	53149278	53149278	T	C	intronic	ZNF83	.	.	.	A	G	-	-	0.36363636	7,0,4,0	chr19,53149	Cytosol	A > G
chr19:53149331:T:C:intronic:ZNF8	chr19	53149331	53149331	T	C	intronic	ZNF83	.	.	.	A	G	-	-	0.57142857	3,0,4,0	chr19,53149	Cytosol	A > G
chr19:53149411:T:C:intronic:ZNF8	chr19	53149411	53149411	T	C	intronic	ZNF83	.	.	.	A	G	-	-	0.25	6,0,2,0	chr19,53149	Cytosol	A > G
chr19:53149412:T:C:intronic:ZNF8	chr19	53149412	53149412	T	C	intronic	ZNF83	.	.	.	A	G	-	-	0.42857143	4,0,3,0	chr19,53149	Cytosol	A > G
chr19:53149550:T:C:intronic:ZNF8	chr19	53149550	53149550	T	C	intronic	ZNF83	.	.	.	A	G	-	-	0.46666667	8,0,7,0	chr19,53149	Cytosol	A > G
chr19:53149553:T:C:intronic:ZNF8	chr19	53149553	53149553	T	C	intronic	ZNF83	.	.	.	A	G	-	-	0.58333333	5,0,7,0	chr19,53149	Cytosol	A > G
chr19:53149616:T:C:intronic:ZNF8	chr19	53149616	53149616	T	C	intronic	ZNF83	.	.	.	A	G	-	-	0.21428571	11,0,3,0	chr19,53149	Cytosol	A > G
chr19:53149690:T:C:intronic:ZNF8	chr19	53149690	53149690	T	C	intronic	ZNF83	.	.	.	A	G	-	-	0.27272727	8,0,3,0	chr19,53149	Cytosol	A > G
chr19:53149707:T:C:intronic:ZNF8	chr19	53149707	53149707	T	C	intronic	ZNF83	.	.	.	A	G	-	-	0.72727273	3,0,8,0	chr19,53149	Cytosol	A > G
chr19:53150734:T:C:intronic:ZNF8	chr19	53150734	53150734	T	C	intronic	ZNF83	.	.	.	A	G	-	-	0.36363636	7,0,4,0	chr19,53150	Cytosol	A > G
chr19:53150739:T:C:intronic:ZNF8	chr19	53150739	53150739	T	C	intronic	ZNF83	.	.	.	A	G	-	-	0.16666667	10,0,2,0	chr19,53150	Cytosol	A > G
chr19:53150794:T:C:intronic:ZNF8	chr19	53150794	53150794	T	C	intronic	ZNF83	.	.	.	A	G	-	-	0.4375	9,0,7,0	chr19,53150	Cytosol	A > G
chr19:53153595:T:C:intronic:ZNF8	chr19	53153595	53153595	T	C	intronic	ZNF83	.	.	.	A	G	-	-	0.28571429	5,0,2,0	chr19,53153	Cytosol	A > G

chr19:53155526:T:C:ncRNA_exoni	chr19	53155526	53155526	T	C	ncRNA_exon	CTD-3099C6.	.	.	.	A	G	-	-	0.22222222	7,0,2,0	chr19,53155	Cytosol	A > G
chr19:53155534:T:C:ncRNA_exoni	chr19	53155534	53155534	T	C	ncRNA_exon	CTD-3099C6.	.	.	.	A	G	-	-	0.33333333	6,0,3,0	chr19,53155	Cytosol	A > G
chr19:53155564:T:C:ncRNA_exoni	chr19	53155564	53155564	T	C	ncRNA_exon	CTD-3099C6.	.	.	.	A	G	-	-	0.33333333	10,0,5,0	chr19,53155	Cytosol	A > G
chr19:53155674:T:C:ncRNA_exoni	chr19	53155674	53155674	T	C	ncRNA_exon	CTD-3099C6.	.	.	.	A	G	-	-	0.46666667	8,0,7,0	chr19,53155	Cytosol	A > G
chr19:53205471:T:C:downstream:	chr19	53205471	53205471	T	C	downstream	ZNF611	.	.	.	A	G	-		0.14285714	12,0,2,0	chr19,53205	Cytosol	A > G
chr19:53205494:T:C:downstream:	chr19	53205494	53205494	T	C	downstream	ZNF611	.	.	.	A	G	-		0.14285714	12,0,2,0	chr19,53205	Cytosol	A > G
chr19:53205800:T:C:downstream:	chr19	53205800	53205800	T	C	downstream	ZNF611	.	.	.	A	G	-		0.14285714	12,0,2,0	chr19,53205	Cytosol	A > G
chr19:53205835:T:C:downstream:	chr19	53205835	53205835	T	C	downstream	ZNF611	.	.	.	A	G	-		0.2	8,0,2,0	chr19,53205	Cytosol	A > G
chr19:53206633:T:C:UTR3:ZNF611	chr19	53206633	53206633	T	C	UTR3	ZNF611	ENST000005	.	.	A	G	-	-	0.42105263	11,0,8,0	chr19,53206	Cytosol	A > G
chr19:53206673:T:C:UTR3:ZNF611	chr19	53206673	53206673	T	C	UTR3	ZNF611	ENST000005	.	.	A	G	-	-	0.15384615	11,0,2,0	chr19,53206	Cytosol	A > G
chr19:53206983:T:C:UTR3:ZNF611	chr19	53206983	53206983	T	C	UTR3	ZNF611	ENST000005	.	.	A	G	-	-	0.25	6,0,2,0	chr19,53206	Cytosol	A > G
chr19:53207012:T:C:UTR3:ZNF611	chr19	53207012	53207012	T	C	UTR3	ZNF611	ENST000005	.	.	A	G	-	-	0.22222222	7,0,2,0	chr19,53207	Cytosol	A > G
chr19:53207019:T:C:UTR3:ZNF611	chr19	53207019	53207019	T	C	UTR3	ZNF611	ENST000005	.	.	A	G	-	-	0.22222222	7,0,2,0	chr19,53207	Cytosol	A > G
chr19:53207049:T:C:UTR3:ZNF611	chr19	53207049	53207049	T	C	UTR3	ZNF611	ENST000005	.	.	A	G	-	-	0.33333333	6,0,3,0	chr19,53207	Cytosol	A > G
chr19:53379862:T:C:UTR3:ZNF320	chr19	53379862	53379862	T	C	UTR3	ZNF320	ENST000003	.	.	A	G	-	-	0.13333333	13,0,2,0	chr19,53379	Cytosol	A > G
chr19:53380244:T:C:UTR3:ZNF320	chr19	53380244	53380244	T	C	UTR3	ZNF320	ENST000003	.	.	A	G	-	-	0.23529412	13,0,4,0	chr19,53380	Cytosol	A > G
chr19:53380277:T:C:UTR3:ZNF320	chr19	53380277	53380277	T	C	UTR3	ZNF320	ENST000003	.	.	A	G	-	-	0.1	18,0,2,0	chr19,53380	Cytosol	A > G
chr19:53381641:T:C:UTR3:ZNF320	chr19	53381641	53381641	T	C	UTR3	ZNF320	ENST000003	.	.	A	G	-	-	0.5	7,0,7,0	chr19,53381	Cytosol	A > G
chr19:53381683:T:C:UTR3:ZNF320	chr19	53381683	53381683	T	C	UTR3	ZNF320	ENST000003	.	.	A	G	-	-	0.23529412	13,0,4,0	chr19,53381	Cytosol	A > G
chr19:53381774:T:C:UTR3:ZNF320	chr19	53381774	53381774	T	C	UTR3	ZNF320	ENST000003	.	.	A	G	-	-	0.05	38,0,2,0	chr19,53381	Cytosol	A > G
chr19:53381781:T:C:UTR3:ZNF320	chr19	53381781	53381781	T	C	UTR3	ZNF320	ENST000003	.	.	A	G	-	-	0.13157895	33,0,5,0	chr19,53381	Cytosol	A > G
chr19:53382030:T:C:UTR3:ZNF320	chr19	53382030	53382030	T	C	UTR3	ZNF320	ENST000003	.	.	A	G	-	-	0.08333333	55,0,5,0	chr19,53382	Cytosol	A > G
chr19:53382102:T:C:UTR3:ZNF320	chr19	53382102	53382102	T	C	UTR3	ZNF320	ENST000003	.	.	A	G	-	-	0.06122449	46,0,3,0	chr19,53382	Cytosol	A > G
chr19:53382111:T:C:UTR3:ZNF320	chr19	53382111	53382111	T	C	UTR3	ZNF320	ENST000003	.	.	A	G	-	-	0.1	45,0,5,0	chr19,53382	Cytosol	A > G
chr19:53382117:T:C:UTR3:ZNF320	chr19	53382117	53382117	T	C	UTR3	ZNF320	ENST000003	.	.	A	G	-	-	0.09302326	39,0,4,0	chr19,53382	Cytosol	A > G
chr19:53382120:T:C:UTR3:ZNF320	chr19	53382120	53382120	T	C	UTR3	ZNF320	ENST000003	.	.	A	G	-	-	0.04347826	44,0,2,0	chr19,53382	Cytosol	A > G
chr19:53383327:T:C:UTR3:ZNF320	chr19	53383327	53383327	T	C	UTR3	ZNF320	ENST000003	.	.	A	G	-	-	0.06666667	42,0,3,0	chr19,53383	Cytosol	A > G
chr19:53383345:T:C:UTR3:ZNF320	chr19	53383345	53383345	T	C	UTR3	ZNF320	ENST000003	.	.	A	G	-	-	0.22641509	41,0,12,0	chr19,53383	Cytosol	A > G
chr19:53383357:T:C:UTR3:ZNF320	chr19	53383357	53383357	T	C	UTR3	ZNF320	ENST000003	.	.	A	G	-	-	0.1	27,0,3,0	chr19,53383	Cytosol	A > G
chr19:53383412:T:C:UTR3:ZNF320	chr19	53383412	53383412	T	C	UTR3	ZNF320	ENST000003	.	.	A	G	-	-	0.33333333	12,0,6,0	chr19,53383	Cytosol	A > G
chr19:53383592:T:C:UTR3:ZNF320	chr19	53383592	53383592	T	C	UTR3	ZNF320	ENST000003	.	.	A	G	-	-	0.0952381	19,0,2,0	chr19,53383	Cytosol	A > G
chr19:53383696:T:C:UTR3:ZNF320	chr19	53383696	53383696	T	C	UTR3	ZNF320	ENST000003	.	.	A	G	-	-	0.05405405	35,0,2,0	chr19,53383	Cytosol	A > G
chr19:53383726:T:C:UTR3:ZNF320	chr19	53383726	53383726	T	C	UTR3	ZNF320	ENST000003	.	.	A	G	-	-	0.04878049	39,0,2,0	chr19,53383	Cytosol	A > G
chr19:53383733:T:C:UTR3:ZNF320	chr19	53383733	53383733	T	C	UTR3	ZNF320	ENST000003	.	.	A	G	-	-	0.18918919	30,0,7,0	chr19,53383	Cytosol	A > G
chr19:53383749:T:C:UTR3:ZNF320	chr19	53383749	53383749	T	C	UTR3	ZNF320	ENST000003	.	.	A	G	-	-	0.04761905	40,0,2,0	chr19,53383	Cytosol	A > G
chr19:53399862:T:C:intronic:ZNF3	chr19	53399862	53399862	T	C	intronic	ZNF320	.	.	.	A	G	-	-	0.28571429	5,0,2,0	chr19,53399	Cytosol	A > G
chr19:53914411:A:G:UTR3:ZNF76	chr19	53914411	53914411	A	G	UTR3	ZNF765	ENST000003	.	.	A	G	+	+	0.54545455	10,0,12,0	chr19,53914	Cytosol	A > G
chr19:53998061:A:G:UTR3:ZNF81	chr19	53998061	53998061	A	G	UTR3	ZNF813	ENST000003	.	.	A	G	+	+	0.25	6,0,2,0	chr19,53998	Cytosol	A > G
chr19:53998737:A:G:UTR3:ZNF81	chr19	53998737	53998737	A	G	UTR3	ZNF813	ENST000003	.	.	A	G	+	+	0.11764706	15,0,2,0	chr19,53998	Cytosol	A > G
chr19:53998738:A:G:UTR3:ZNF81	chr19	53998738	53998738	A	G	UTR3	ZNF813	ENST000003	.	.	A	G	+	+	0.11764706	15,0,2,0	chr19,53998	Cytosol	A > G
chr19:53998944:A:G:UTR3:ZNF81	chr19	53998944	53998944	A	G	UTR3	ZNF813	ENST000003	.	.	A	G	+	+	0.26666667	11,0,4,0	chr19,53998	Cytosol	A > G
chr19:53998945:A:G:UTR3:ZNF81	chr19	53998945	53998945	A	G	UTR3	ZNF813	ENST000003	.	.	A	G	+	+	0.53333333	7,0,8,0	chr19,53998	Cytosol	A > G
chr19:54707516:A:G:intronic:RPS	chr19	54707516	54707516	A	G	intronic	RPS9	.	.	.	A	G	+	+	0.2	8,0,2,0	chr19,54707	Cytosol	A > G
chr19:55550641:T:C:ncRNA_intror	chr19	55550641	55550641	T	C	ncRNA_intro	CTC-550B14.	.	.	.	A	G	-	+-	0.17647059	14,0,3,0	chr19,55550	Cytosol	A > G
chr19:55550643:T:C:ncRNA_intror	chr19	55550643	55550643	T	C	ncRNA_intro	CTC-550B14.	.	.	.	A	G	-	+-	0.5	8,0,8,0	chr19,55550	Cytosol	A > G
chr19:55550711:T:C:ncRNA_intror	chr19	55550711	55550711	T	C	ncRNA_intro	CTC-550B14.	.	.	.	A	G	-	+-	0.42105263	11,0,8,0	chr19,55550	Cytosol	A > G
chr19:55550712:T:C:ncRNA_intror	chr19	55550712	55550712	T	C	ncRNA_intro	CTC-550B14.	.	.	.	A	G	-	+-	0.6	8,0,12,0	chr19,55550	Cytosol	A > G
chr19:55550717:T:C:ncRNA_intror	chr19	55550717	55550717	T	C	ncRNA_intro	CTC-550B14.	.	.	.	A	G	-	+-	0.36363636	14,0,8,0	chr19,55550	Cytosol	A > G
chr19:55550804:T:C:ncRNA_intror	chr19	55550804	55550804	T	C	ncRNA_intro	CTC-550B14.	.	.	.	A	G	-	+-	0.4375	9,0,7,0	chr19,55550	Cytosol	A > G
chr19:55550959:T:C:ncRNA_intror	chr19	55550959	55550959	T	C	ncRNA_intro	CTC-550B14.	.	.	.	A	G	-	+-	0.25	21,0,7,0	chr19,55550	Cytosol	A > G
chr19:55550962:T:C:ncRNA_intror	chr19	55550962	55550962	T	C	ncRNA_intro	CTC-550B14.	.	.	.	A	G	-	+-	0.08333333	22,0,2,0	chr19,55550	Cytosol	A > G
chr19:55551790:T:C:ncRNA_intror	chr19	55551790	55551790	T	C	ncRNA_intro	CTC-550B14.	.	.	.	A	G	-	+-	0.4375	9,0,7,0	chr19,55551	Cytosol	A > G
chr19:55552238:T:C:ncRNA_intror	chr19	55552238	55552238	T	C	ncRNA_intro	CTC-550B14.	.	.	.	A	G	-	+-	0.23076923	10,0,3,0	chr19,55552	Cytosol	A > G
chr19:55552312:T:C:ncRNA_intror	chr19	55552312	55552312	T	C	ncRNA_intro	CTC-550B14.	.	.	.	A	G	-	+-	0.41666667	7,0,5,0	chr19,55552	Cytosol	A > G
chr19:55554741:T:C:ncRNA_intror	chr19	55554741	55554741	T	C	ncRNA_intro	CTC-550B14.	.	.	.	A	G	-	+-	0.25925926	20,0,7,0	chr19,55554	Cytosol	A > G
chr19:55554755:T:C:ncRNA_intror	chr19	55554755	55554755	T	C	ncRNA_intro	CTC-550B14.	.	.	.	A	G	-	+-	0.07407407	25,0,2,0	chr19,55554	Cytosol	A > G
chr19:55554984:T:C:ncRNA_intror	chr19	55554984	55554984	T	C	ncRNA_intro	CTC-550B14.	.	.	.	A	G	-	+-	0.30769231	9,0,4,0	chr19,55554	Cytosol	A > G
chr19:55900533:A:G:UTR3:RPL28	chr19	55900533	55900533	A	G	UTR3	RPL28	ENST000003	.	.	A	G	+	+	0.91666667	2,0,22,0	chr19,55900	Cytosol	A > G
chr19:55900648:A:G:UTR3:RPL28	chr19	55900648	55900648	A	G	UTR3	RPL28	ENST000003	.	.	A	G	+	+	0.21428571	33,0,9,0	chr19,55900	Cytosol	A > G
chr19:55900653:A:G:UTR3:RPL28	chr19	55900653	55900653	A	G	UTR3	RPL28	ENST000003	.	.	A	G	+	+	0.11111111	40,0,5,0	chr19,55900	Cytosol	A > G
chr19:56150126:A:G:intronic:ZNF	chr19	56150126	56150126	A	G	intronic	ZNF580,ZNF	.	.	.	A	G	+	+	0.4	3,0,2,0	chr19,56150	Cytosol	A > G
chr19:57815547:A:G:upstream:CT	chr19	57815547	57815547	A	G	upstream	CTC-444N24.	.	.	.	A	G	+		0.15789474	16,0,3,0	chr19,57815	Cytosol	A > G
chr19:57816153:A:G:ncRNA_exoni	chr19	57816153	57816153	A	G	ncRNA_exon	CTC-444N24.	.	.	.	A	G	+	+	0.71428571	2,0,5,0	chr19,57816	Cytosol	A > G
chr19:57816287:A:G:ncRNA_exoni	chr19	57816287	57816287	A	G	ncRNA_exon	CTC-444N24.	.	.	.	A	G	+	+	0.77777778	2,0,7,0	chr19,57816	Cytosol	A > G

chr19:57816924:A:G:ncRNA_exoni	chr19	57816924	57816924	A	G	ncRNA_exon	CTC-444N24.	.	.	.	A	G	+	+	0.47058824	9,0,8,0	chr19,57816	Cytosol	A > G
chr19:57817005:A:G:ncRNA_exoni	chr19	57817005	57817005	A	G	ncRNA_exon	CTC-444N24.	.	.	.	A	G	+	+	0.66666667	7,0,14,0	chr19,57817	Cytosol	A > G
chr19:57817016:A:G:ncRNA_exoni	chr19	57817016	57817016	A	G	ncRNA_exon	CTC-444N24.	.	.	.	A	G	+	+	0.64705882	6,0,11,0	chr19,57817	Cytosol	A > G
chr19:57818348:A:G:ncRNA_exoni	chr19	57818348	57818348	A	G	ncRNA_exon	CTC-444N24.	.	.	.	A	G	+	+	0.27272727	8,0,3,0	chr19,57818	Cytosol	A > G
chr19:57818376:A:G:ncRNA_exoni	chr19	57818376	57818376	A	G	ncRNA_exon	CTC-444N24.	.	.	.	A	G	+	+	0.14285714	12,0,2,0	chr19,57818	Cytosol	A > G
chr19:57818777:A:G:ncRNA_exoni	chr19	57818777	57818777	A	G	ncRNA_exon	CTC-444N24.	.	.	.	A	G	+	+	0.10526316	17,0,2,0	chr19,57818	Cytosol	A > G
chr19:58120322:A:G:intronic:ZNF5	chr19	58120322	58120322	A	G	intronic	ZNF530	.	.	.	A	G	+	+	0.4	3,0,2,0	chr19,58120	Cytosol	A > G
chr19:58318762:T:C:UTR3:ZNF552	chr19	58318762	58318762	T	C	UTR3	ZNF552	ENST000003	.	.	A	G	-	+-	0.25	6,0,2,0	chr19,58318	Cytosol	A > G
chr19:58319085:T:C:UTR3:ZNF552	chr19	58319085	58319085	T	C	UTR3	ZNF552	ENST000003	.	.	A	G	-	+-	0.35294118	11,0,6,0	chr19,58319	Cytosol	A > G
chr19:58354433:A:G:intronic:CTD-	chr19	58354433	58354433	A	G	intronic	CTD-2583A1	.	.	.	A	G	+	+	0.13333333	13,0,2,0	chr19,58354	Cytosol	A > G
chr19:58354558:A:G:intronic:CTD-	chr19	58354558	58354558	A	G	intronic	CTD-2583A1	.	.	.	A	G	+	+	0.5	9,0,9,0	chr19,58354	Cytosol	A > G
chr19:58354622:A:G:intronic:CTD-	chr19	58354622	58354622	A	G	intronic	CTD-2583A1	.	.	.	A	G	+	+	0.15384615	11,0,2,0	chr19,58354	Cytosol	A > G
chr19:58355049:A:G:intronic:CTD-	chr19	58355049	58355049	A	G	intronic	CTD-2583A1	.	.	.	A	G	+	+	0.11764706	15,0,2,0	chr19,58355	Cytosol	A > G
chr19:58355050:A:G:intronic:CTD-	chr19	58355050	58355050	A	G	intronic	CTD-2583A1	.	.	.	A	G	+	+	0.29411765	12,0,5,0	chr19,58355	Cytosol	A > G
chr19:58355069:A:G:intronic:CTD-	chr19	58355069	58355069	A	G	intronic	CTD-2583A1	.	.	.	A	G	+	+	0.46153846	7,0,6,0	chr19,58355	Cytosol	A > G
chr19:58355070:A:G:intronic:CTD-	chr19	58355070	58355070	A	G	intronic	CTD-2583A1	.	.	.	A	G	+	+	0.57142857	6,0,8,0	chr19,58355	Cytosol	A > G
chr19:58355073:A:G:intronic:CTD-	chr19	58355073	58355073	A	G	intronic	CTD-2583A1	.	.	.	A	G	+	+	0.38461538	8,0,5,0	chr19,58355	Cytosol	A > G
chr19:58355074:A:G:intronic:CTD-	chr19	58355074	58355074	A	G	intronic	CTD-2583A1	.	.	.	A	G	+	+	0.42857143	8,0,6,0	chr19,58355	Cytosol	A > G
chr19:58355150:A:G:intronic:CTD-	chr19	58355150	58355150	A	G	intronic	CTD-2583A1	.	.	.	A	G	+	+	0.21428571	11,0,3,0	chr19,58355	Cytosol	A > G
chr19:58355170:A:G:intronic:CTD-	chr19	58355170	58355170	A	G	intronic	CTD-2583A1	.	.	.	A	G	+	+	0.16666667	10,0,2,0	chr19,58355	Cytosol	A > G
chr19:58355620:A:G:intronic:CTD-	chr19	58355620	58355620	A	G	intronic	CTD-2583A1	.	.	.	A	G	+	+	0.66666667	5,0,10,0	chr19,58355	Cytosol	A > G
chr19:58355622:A:G:splicing:ZNF5	chr19	58355622	58355622	A	G	splicing	ZNF587B	ENST000004	.	.	A	G	+	+	0.58823529	7,0,10,0	chr19,58355	Cytosol	A > G
chr19:58355670:A:G:exonic:ZNF58	chr19	58355670	58355670	A	G	exonic	ZNF587B	.	nonsynonym	ZNF587B:EN	A	G	+	+	0.27272727	8,0,3,0	chr19,58355	Cytosol	A > G
chr19:58355689:A:G:exonic:ZNF58	chr19	58355689	58355689	A	G	exonic	ZNF587B	.	nonsynonym	ZNF587B:EN	A	G	+	+	0.63636364	4,0,7,0	chr19,58355	Cytosol	A > G
chr19:58355713:A:G:UTR3:ZNF587	chr19	58355713	58355713	A	G	UTR3	ZNF587B	ENST000004	.	.	A	G	+	+	0.36363636	7,0,4,0	chr19,58355	Cytosol	A > G
chr19:58355748:A:G:UTR3:ZNF587	chr19	58355748	58355748	A	G	UTR3	ZNF587B	ENST000004	.	.	A	G	+	+	0.66666667	3,0,6,0	chr19,58355	Cytosol	A > G
chr19:58355779:A:G:UTR3:ZNF587	chr19	58355779	58355779	A	G	UTR3	ZNF587B	ENST000004	.	.	A	G	+	+	0.2	8,0,2,0	chr19,58355	Cytosol	A > G
chr19:58355790:A:G:UTR3:ZNF587	chr19	58355790	58355790	A	G	UTR3	ZNF587B	ENST000004	.	.	A	G	+	+	0.25	9,0,3,0	chr19,58355	Cytosol	A > G
chr19:58356393:A:G:UTR3:ZNF587	chr19	58356393	58356393	A	G	UTR3	ZNF587B	ENST000004	.	.	A	G	+	+	0.54545455	5,0,6,0	chr19,58356	Cytosol	A > G
chr19:58356445:A:G:UTR3:ZNF587	chr19	58356445	58356445	A	G	UTR3	ZNF587B	ENST000004	.	.	A	G	+	+	0.27272727	16,0,6,0	chr19,58356	Cytosol	A > G
chr19:58356470:A:G:UTR3:ZNF587	chr19	58356470	58356470	A	G	UTR3	ZNF587B	ENST000004	.	.	A	G	+	+	0.08	23,0,2,0	chr19,58356	Cytosol	A > G
chr19:58356524:A:G:UTR3:ZNF587	chr19	58356524	58356524	A	G	UTR3	ZNF587B	ENST000004	.	.	A	G	+	+	0.29166667	17,0,7,0	chr19,58356	Cytosol	A > G
chr19:58357111:A:G:UTR3:ZNF587	chr19	58357111	58357111	A	G	UTR3	ZNF587B	ENST000004	.	.	A	G	+	+	0.06896552	27,0,2,0	chr19,58357	Cytosol	A > G
chr19:58357158:A:G:UTR3:ZNF587	chr19	58357158	58357158	A	G	UTR3	ZNF587B	ENST000004	.	.	A	G	+	+	0.16129032	26,0,5,0	chr19,58357	Cytosol	A > G
chr19:58357160:A:G:UTR3:ZNF587	chr19	58357160	58357160	A	G	UTR3	ZNF587B	ENST000004	.	.	A	G	+	+	0.10344828	26,0,3,0	chr19,58357	Cytosol	A > G
chr19:58357256:A:G:UTR3:ZNF587	chr19	58357256	58357256	A	G	UTR3	ZNF587B	ENST000004	.	.	A	G	+	+	0.25	6,0,2,0	chr19,58357	Cytosol	A > G
chr19:58357268:A:G:UTR3:ZNF587	chr19	58357268	58357268	A	G	UTR3	ZNF587B	ENST000004	.	.	A	G	+	+	0.83333333	1,0,5,0	chr19,58357	Cytosol	A > G
chr19:58357271:A:G:UTR3:ZNF587	chr19	58357271	58357271	A	G	UTR3	ZNF587B	ENST000004	.	.	A	G	+	+	0.42857143	4,0,3,0	chr19,58357	Cytosol	A > G
chr19:58372048:A:G:UTR3:ZNF587	chr19	58372048	58372048	A	G	UTR3	ZNF587	ENST000003	.	.	A	G	+	+-	0.85714286	2,0,12,0	chr19,58372	Cytosol	A > G
chr19:58372069:A:G:UTR3:ZNF587	chr19	58372069	58372069	A	G	UTR3	ZNF587	ENST000003	.	.	A	G	+	+-	0.5	8,0,8,0	chr19,58372	Cytosol	A > G
chr19:58372249:A:G:UTR3:ZNF587	chr19	58372249	58372249	A	G	UTR3	ZNF587	ENST000003	.	.	A	G	+	+-	0.0952381	19,0,2,0	chr19,58372	Cytosol	A > G
chr19:58372320:A:G:UTR3:ZNF587	chr19	58372320	58372320	A	G	UTR3	ZNF587	ENST000003	.	.	A	G	+	+-	0.06060606	31,0,2,0	chr19,58372	Cytosol	A > G
chr19:58372365:A:G:UTR3:ZNF587	chr19	58372365	58372365	A	G	UTR3	ZNF587	ENST000003	.	.	A	G	+	+-	0.11538462	23,0,3,0	chr19,58372	Cytosol	A > G
chr19:58372747:A:G:UTR3:ZNF587	chr19	58372747	58372747	A	G	UTR3	ZNF587	ENST000003	.	.	A	G	+	-+	0.22727273	17,0,5,0	chr19,58372	Cytosol	A > G
chr19:58372752:A:G:UTR3:ZNF587	chr19	58372752	58372752	A	G	UTR3	ZNF587	ENST000003	.	.	A	G	+	-+	0.38888889	11,0,7,0	chr19,58372	Cytosol	A > G
chr19:58372768:A:G:UTR3:ZNF587	chr19	58372768	58372768	A	G	UTR3	ZNF587	ENST000003	.	.	A	G	+	-+	0.33333333	6,0,3,0	chr19,58372	Cytosol	A > G
chr19:58373255:A:G:UTR3:ZNF587	chr19	58373255	58373255	A	G	UTR3	ZNF587	ENST000003	.	.	A	G	+	-+	0.14285714	12,0,2,0	chr19,58373	Cytosol	A > G
chr19:58373325:A:G:UTR3:ZNF587	chr19	58373325	58373325	A	G	UTR3	ZNF587	ENST000003	.	.	A	G	+	-+	0.125	14,0,2,0	chr19,58373	Cytosol	A > G
chr19:58375157:A:G:UTR3:ZNF587	chr19	58375157	58375157	A	G	UTR3	ZNF587	ENST000003	.	.	A	G	+	+-	0.54545455	5,0,6,0	chr19,58375	Cytosol	A > G
chr19:58375158:A:G:UTR3:ZNF587	chr19	58375158	58375158	A	G	UTR3	ZNF587	ENST000003	.	.	A	G	+	+-	0.5	5,0,5,0	chr19,58375	Cytosol	A > G
chr19:58375195:A:G:UTR3:ZNF587	chr19	58375195	58375195	A	G	UTR3	ZNF587	ENST000003	.	.	A	G	+	+-	0.28571429	5,0,2,0	chr19,58375	Cytosol	A > G
chr19:58375196:A:G:UTR3:ZNF587	chr19	58375196	58375196	A	G	UTR3	ZNF587	ENST000003	.	.	A	G	+	+-	0.42857143	4,0,3,0	chr19,58375	Cytosol	A > G
chr19:58375321:A:G:UTR3:ZNF587	chr19	58375321	58375321	A	G	UTR3	ZNF587	ENST000003	.	.	A	G	+	+-	0.5625	7,0,9,0	chr19,58375	Cytosol	A > G
chr19:58375360:A:G:UTR3:ZNF587	chr19	58375360	58375360	A	G	UTR3	ZNF587	ENST000003	.	.	A	G	+	+-	0.22580645	24,0,7,0	chr19,58375	Cytosol	A > G
chr19:58375722:A:G:UTR3:ZNF587	chr19	58375722	58375722	A	G	UTR3	ZNF587	ENST000003	.	.	A	G	+	+-	0.375	5,0,3,0	chr19,58375	Cytosol	A > G
chr19:58375736:A:G:UTR3:ZNF587	chr19	58375736	58375736	A	G	UTR3	ZNF587	ENST000003	.	.	A	G	+	+-	0.625	3,0,5,0	chr19,58375	Cytosol	A > G
chr19:58375743:A:G:UTR3:ZNF587	chr19	58375743	58375743	A	G	UTR3	ZNF587	ENST000003	.	.	A	G	+	+-	0.8	2,0,8,0	chr19,58375	Cytosol	A > G
chr19:58376237:A:G:UTR3:ZNF587	chr19	58376237	58376237	A	G	UTR3	ZNF587,ZNF8	ENST000003	.	.	A	G	+	+-	0.11764706	15,0,2,0	chr19,58376	Cytosol	A > G
chr19:58377006:A:G:intronic:ZNF8	chr19	58377006	58377006	A	G	intronic	ZNF814	.	.	.	A	G	+	-	0.125	14,0,2,0	chr19,58377	Cytosol	A > G
chr19:58377379:A:G:intronic:ZNF8	chr19	58377379	58377379	A	G	intronic	ZNF814	.	.	.	A	G	+	-	0.71428571	2,0,5,0	chr19,58377	Cytosol	A > G
chr19:58377386:A:G:intronic:ZNF8	chr19	58377386	58377386	A	G	intronic	ZNF814	.	.	.	A	G	+	-	1	0,0,6,0	chr19,58377	Cytosol	A > G
chr19:58377448:A:G:intronic:ZNF8	chr19	58377448	58377448	A	G	intronic	ZNF814	.	.	.	A	G	+	-	0.31578947	13,0,6,0	chr19,58377	Cytosol	A > G
chr19:58377528:A:G:intronic:ZNF8	chr19	58377528	58377528	A	G	intronic	ZNF814	.	.	.	A	G	+	-	0.35714286	9,0,5,0	chr19,58377	Cytosol	A > G
chr19:58377868:A:G:intronic:ZNF8	chr19	58377868	58377868	A	G	intronic	ZNF814	.	.	.	A	G	+	-	0.28571429	5,0,2,0	chr19,58377	Cytosol	A > G

chr19:58378001:A:G:intronic:ZNF8	chr19	58378001	58378001	A	G	intronic	ZNF814	.	.	.	A	G	+	-	0.42857143	4,0,3,0	chr19,58378	Cytosol	A > G
chr19:58378174:A:G:intronic:ZNF8	chr19	58378174	58378174	A	G	intronic	ZNF814	.	.	.	A	G	+	-	0.27777778	13,0,5,0	chr19,58378	Cytosol	A > G
chr19:58378175:A:G:intronic:ZNF8	chr19	58378175	58378175	A	G	intronic	ZNF814	.	.	.	A	G	+	-	0.1	18,0,2,0	chr19,58378	Cytosol	A > G
chr19:58378231:A:G:intronic:ZNF8	chr19	58378231	58378231	A	G	intronic	ZNF814	.	.	.	A	G	+	-	0.125	14,0,2,0	chr19,58378	Cytosol	A > G
chr19:58378232:A:G:intronic:ZNF8	chr19	58378232	58378232	A	G	intronic	ZNF814	.	.	.	A	G	+	-	0.11111111	16,0,2,0	chr19,58378	Cytosol	A > G
chr19:58378245:A:G:intronic:ZNF8	chr19	58378245	58378245	A	G	intronic	ZNF814	.	.	.	A	G	+	-	0.1	18,0,2,0	chr19,58378	Cytosol	A > G
chr19:58378253:A:G:intronic:ZNF8	chr19	58378253	58378253	A	G	intronic	ZNF814	.	.	.	A	G	+	-	0.09090909	20,0,2,0	chr19,58378	Cytosol	A > G
chr19:58378256:A:G:intronic:ZNF8	chr19	58378256	58378256	A	G	intronic	ZNF814	.	.	.	A	G	+	-	0.1	18,0,2,0	chr19,58378	Cytosol	A > G
chr19:58378285:A:G:intronic:ZNF8	chr19	58378285	58378285	A	G	intronic	ZNF814	.	.	.	A	G	+	-	0.40740741	16,0,11,0	chr19,58378	Cytosol	A > G
chr19:58378286:A:G:intronic:ZNF8	chr19	58378286	58378286	A	G	intronic	ZNF814	.	.	.	A	G	+	-	0.2962963	19,0,8,0	chr19,58378	Cytosol	A > G
chr19:58378337:A:G:intronic:ZNF8	chr19	58378337	58378337	A	G	intronic	ZNF814	.	.	.	A	G	+	-	0.36363636	7,0,4,0	chr19,58378	Cytosol	A > G
chr19:58378349:A:G:intronic:ZNF8	chr19	58378349	58378349	A	G	intronic	ZNF814	.	.	.	A	G	+	-	0.76923077	3,0,10,0	chr19,58378	Cytosol	A > G
chr19:58378350:A:G:intronic:ZNF8	chr19	58378350	58378350	A	G	intronic	ZNF814	.	.	.	A	G	+	-	0.28571429	10,0,4,0	chr19,58378	Cytosol	A > G
chr19:58378360:A:G:intronic:ZNF8	chr19	58378360	58378360	A	G	intronic	ZNF814	.	.	.	A	G	+	-	0.2	12,0,3,0	chr19,58378	Cytosol	A > G
chr19:58378380:A:G:intronic:ZNF8	chr19	58378380	58378380	A	G	intronic	ZNF814	.	.	.	A	G	+	-	0.21428571	11,0,3,0	chr19,58378	Cytosol	A > G
chr19:58418492:T:C:UTR3:ZNF417	chr19	58418492	58418492	T	C	UTR3	ZNF417	ENST000003	.	.	A	G	-	-	0.4	6,0,4,0	chr19,58418	Cytosol	A > G
chr19:58418493:T:C:UTR3:ZNF417	chr19	58418493	58418493	T	C	UTR3	ZNF417	ENST000003	.	.	A	G	-	-	0.7	3,0,7,0	chr19,58418	Cytosol	A > G
chr19:58638141:T:C:UTR3:ZNF329	chr19	58638141	58638141	T	C	UTR3	ZNF329	ENST000005	.	.	A	G	-	-	0.16666667	10,0,2,0	chr19,58638	Cytosol	A > G
chr19:58638153:T:C:UTR3:ZNF329	chr19	58638153	58638153	T	C	UTR3	ZNF329	ENST000005	.	.	A	G	-	-	0.16666667	10,0,2,0	chr19,58638	Cytosol	A > G
chr19:58638172:T:C:UTR3:ZNF329	chr19	58638172	58638172	T	C	UTR3	ZNF329	ENST000005	.	.	A	G	-	-	0.22222222	7,0,2,0	chr19,58638	Cytosol	A > G
chr19:58638293:T:C:UTR3:ZNF329	chr19	58638293	58638293	T	C	UTR3	ZNF329	ENST000005	.	.	A	G	-	-	0.26666667	11,0,4,0	chr19,58638	Cytosol	A > G
chr19:58638705:T:C:UTR3:ZNF329	chr19	58638705	58638705	T	C	UTR3	ZNF329	ENST000005	.	.	A	G	-	-	0.5	3,0,3,0	chr19,58638	Cytosol	A > G
chr19:58771146:A:G:ncRNA_intro	chr19	58771146	58771146	A	G	ncRNA_intro	CTD-3138B1	.	.	.	A	G	+	+-	0.8	1,0,4,0	chr19,58771	Cytosol	A > G
chr19:58771195:A:G:ncRNA_intro	chr19	58771195	58771195	A	G	ncRNA_intro	CTD-3138B1	.	.	.	A	G	+	+-	0.6	2,0,3,0	chr19,58771	Cytosol	A > G
chr19:59093997:A:G:ncRNA_exoni	chr19	59093997	59093997	A	G	ncRNA_exon	RPL23AP79	.	.	.	A	G	+	+	0.125	28,0,4,0	chr19,59093	Cytosol	A > G
chr19:59093998:A:G:ncRNA_exoni	chr19	59093998	59093998	A	G	ncRNA_exon	RPL23AP79	.	.	.	A	G	+	+	0.125	28,0,4,0	chr19,59093	Cytosol	A > G
chr19:59093999:A:G:ncRNA_exoni	chr19	59093999	59093999	A	G	ncRNA_exon	RPL23AP79	.	.	.	A	G	+	+	0.16129032	26,0,5,0	chr19,59093	Cytosol	A > G
chr19:59095456:A:G:ncRNA_exoni	chr19	59095456	59095456	A	G	ncRNA_exon	RPL23AP79	.	.	.	A	G	+	+	0.05263158	36,0,2,0	chr19,59095	Cytosol	A > G
chr19:59095469:A:G:ncRNA_exoni	chr19	59095469	59095469	A	G	ncRNA_exon	RPL23AP79	.	.	.	A	G	+	+	0.48648649	19,0,18,0	chr19,59095	Cytosol	A > G
chr19:59095482:A:G:ncRNA_exoni	chr19	59095482	59095482	A	G	ncRNA_exon	RPL23AP79	.	.	.	A	G	+	+	0.40540541	22,0,15,0	chr19,59095	Cytosol	A > G
chr19:59095573:A:G:ncRNA_exoni	chr19	59095573	59095573	A	G	ncRNA_exon	RPL23AP79	.	.	.	A	G	+	+	0.09677419	28,0,3,0	chr19,59095	Cytosol	A > G
chr2:3589498:T:C:intronic:RP13-5	chr2	3589498	3589498	T	C	intronic	RP13-512J5.	.	.	.	A	G	-	-	0.22666667	58,0,17,0	chr2,358949	Cytosol	A > G
chr2:3589499:T:C:intronic:RP13-5	chr2	3589499	3589499	T	C	intronic	RP13-512J5.	.	.	.	A	G	-	-	0.07894737	70,0,6,0	chr2,358949	Cytosol	A > G
chr2:3589500:T:C:intronic:RP13-5	chr2	3589500	3589500	T	C	intronic	RP13-512J5.	.	.	.	A	G	-	-	0.03947368	73,0,3,0	chr2,358949	Cytosol	A > G
chr2:3589521:T:C:intronic:RP13-5	chr2	3589521	3589521	T	C	intronic	RP13-512J5.	.	.	.	A	G	-	-	0.0625	75,0,5,0	chr2,358952	Cytosol	A > G
chr2:3589525:T:C:intronic:RP13-5	chr2	3589525	3589525	T	C	intronic	RP13-512J5.	.	.	.	A	G	-	-	0.26136364	65,0,23,0	chr2,358952	Cytosol	A > G
chr2:3589526:T:C:intronic:RP13-5	chr2	3589526	3589526	T	C	intronic	RP13-512J5.	.	.	.	A	G	-	-	0.02912621	100,0,3,0	chr2,358952	Cytosol	A > G
chr2:3589551:T:C:intronic:RP13-5	chr2	3589551	3589551	T	C	intronic	RP13-512J5.	.	.	.	A	G	-	-	0.2	88,0,22,0	chr2,358955	Cytosol	A > G
chr2:3589577:T:C:intronic:RP13-5	chr2	3589577	3589577	T	C	intronic	RP13-512J5.	.	.	.	A	G	-	-	0.02830189	103,0,3,0	chr2,358957	Cytosol	A > G
chr2:3591441:T:C:intronic:RP13-5	chr2	3591441	3591441	T	C	intronic	RP13-512J5.	.	.	.	A	G	-	-	0.28378378	53,0,21,0	chr2,359144	Cytosol	A > G
chr2:3592602:T:C:UTR3:RNASEH1	chr2	3592602	3592602	T	C	UTR3	RNASEH1	ENST000004	.	.	A	G	-	-	0.43137255	29,0,22,0	chr2,359260	Cytosol	A > G
chr2:3592607:T:C:UTR3:RNASEH1	chr2	3592607	3592607	T	C	UTR3	RNASEH1	ENST000004	.	.	A	G	-	-	0.25806452	46,0,16,0	chr2,359260	Cytosol	A > G
chr2:3592615:T:C:UTR3:RNASEH1	chr2	3592615	3592615	T	C	UTR3	RNASEH1	ENST000004	.	.	A	G	-	-	0.04878049	78,0,4,0	chr2,359261	Cytosol	A > G
chr2:3592638:T:C:UTR3:RNASEH1	chr2	3592638	3592638	T	C	UTR3	RNASEH1	ENST000004	.	.	A	G	-	-	0.13636364	76,0,12,0	chr2,359263	Cytosol	A > G
chr2:3618712:T:C:intergenic:AC10	chr2	3618712	3618712	T	C	intergenic	AC108488.3,	dist=9388;dis	.	.	A	G	-		0.1875	13,0,3,0	chr2,361871	Cytosol	A > G
chr2:10270959:A:G:UTR3:RRM2	chr2	10270959	10270959	A	G	UTR3	RRM2	ENST000003	.	.	A	G	+	+	0.024	366,0,9,0	chr2,102709	Cytosol	A > G
chr2:20492628:T:C:intronic:PUM2	chr2	20492628	20492628	T	C	intronic	PUM2	.	.	.	A	G	-	-	0.33333333	4,0,2,0	chr2,204926	Cytosol	A > G
chr2:24223159:A:G:UTR3:UBXN2A	chr2	24223159	24223159	A	G	UTR3	UBXN2A	ENST000003	.	.	A	G	+	+	0.13533835	114,0,18,1	chr2,242231	Cytosol	A > G
chr2:24223166:A:G:UTR3:UBXN2A	chr2	24223166	24223166	A	G	UTR3	UBXN2A	ENST000003	.	.	A	G	+	+	0.02702703	144,0,4,0	chr2,242231	Cytosol	A > G
chr2:24223167:A:G:UTR3:UBXN2A	chr2	24223167	24223167	A	G	UTR3	UBXN2A	ENST000003	.	.	A	G	+	+	0.05844156	145,0,9,0	chr2,242231	Cytosol	A > G
chr2:24223189:A:G:UTR3:UBXN2A	chr2	24223189	24223189	A	G	UTR3	UBXN2A	ENST000003	.	.	A	G	+	+	0.02564103	152,0,4,0	chr2,242231	Cytosol	A > G
chr2:24223241:A:G:UTR3:UBXN2A	chr2	24223241	24223241	A	G	UTR3	UBXN2A	ENST000003	.	.	A	G	+	+	0.06493506	72,0,5,0	chr2,242232	Cytosol	A > G
chr2:24223247:A:G:UTR3:UBXN2A	chr2	24223247	24223247	A	G	UTR3	UBXN2A	ENST000003	.	.	A	G	+	+	0.04938272	77,0,4,0	chr2,242232	Cytosol	A > G
chr2:24223251:A:G:UTR3:UBXN2A	chr2	24223251	24223251	A	G	UTR3	UBXN2A	ENST000003	.	.	A	G	+	+	0.06666667	70,0,5,0	chr2,242232	Cytosol	A > G
chr2:24223252:A:G:UTR3:UBXN2A	chr2	24223252	24223252	A	G	UTR3	UBXN2A	ENST000003	.	.	A	G	+	+	0.04166667	69,0,3,0	chr2,242232	Cytosol	A > G
chr2:24223256:A:G:UTR3:UBXN2A	chr2	24223256	24223256	A	G	UTR3	UBXN2A	ENST000003	.	.	A	G	+	+	0.19298246	46,0,11,0	chr2,242232	Cytosol	A > G
chr2:24223265:A:G:UTR3:UBXN2A	chr2	24223265	24223265	A	G	UTR3	UBXN2A	ENST000003	.	.	A	G	+	+	0.05172414	55,0,3,0	chr2,242232	Cytosol	A > G
chr2:24223341:A:G:UTR3:UBXN2A	chr2	24223341	24223341	A	G	UTR3	UBXN2A	ENST000003	.	.	A	G	+	+	0.04672897	102,0,5,0	chr2,242233	Cytosol	A > G
chr2:24223353:A:G:UTR3:UBXN2A	chr2	24223353	24223353	A	G	UTR3	UBXN2A	ENST000003	.	.	A	G	+	+	0.08823529	93,0,9,0	chr2,242233	Cytosol	A > G
chr2:24223463:A:G:UTR3:UBXN2A	chr2	24223463	24223463	A	G	UTR3	UBXN2A	ENST000003	.	.	A	G	+	+	0.02	98,0,2,0	chr2,242234	Cytosol	A > G
chr2:24223931:A:G:UTR3:UBXN2A	chr2	24223931	24223931	A	G	UTR3	UBXN2A	ENST000003	.	.	A	G	+	+	0.5	3,0,3,0	chr2,242239	Cytosol	A > G
chr2:24223986:A:G:UTR3:UBXN2A	chr2	24223986	24223986	A	G	UTR3	UBXN2A	ENST000003	.	.	A	G	+	+	0.4	6,0,4,0	chr2,242239	Cytosol	A > G
chr2:24224013:A:G:UTR3:UBXN2A	chr2	24224013	24224013	A	G	UTR3	UBXN2A	ENST000003	.	.	A	G	+	+	0.2	12,0,3,0	chr2,242240	Cytosol	A > G
chr2:24224040:A:G:UTR3:UBXN2A	chr2	24224040	24224040	A	G	UTR3	UBXN2A	ENST000003	.	.	A	G	+	+	0.17391304	19,0,4,0	chr2,242240	Cytosol	A > G

chr2:24224134:A:G:UTR3:UBXN2A	chr2	24224134	24224134	A	G	UTR3	UBXN2A	ENST000003	.	.	A	G	+	+	0.175	33,0,7,0	chr2,242241	Cytosol	A > G
chr2:24224642:A:G:UTR3:UBXN2A	chr2	24224642	24224642	A	G	UTR3	UBXN2A	ENST000003	.	.	A	G	+	+	0.35714286	9,0,5,0	chr2,242246	Cytosol	A > G
chr2:24224710:A:G:UTR3:UBXN2A	chr2	24224710	24224710	A	G	UTR3	UBXN2A	ENST000003	.	.	A	G	+	+	0.33333333	8,0,4,0	chr2,242247	Cytosol	A > G
chr2:24224883:A:G:UTR3:UBXN2A	chr2	24224883	24224883	A	G	UTR3	UBXN2A	ENST000003	.	.	A	G	+	+	0.26666667	11,0,4,0	chr2,242248	Cytosol	A > G
chr2:24225006:A:G:UTR3:UBXN2A	chr2	24225006	24225006	A	G	UTR3	UBXN2A	ENST000003	.	.	A	G	+	+	0.37837838	23,0,14,0	chr2,242250	Cytosol	A > G
chr2:24225013:A:G:UTR3:UBXN2A	chr2	24225013	24225013	A	G	UTR3	UBXN2A	ENST000003	.	.	A	G	+	+	0.09090909	30,0,3,0	chr2,242250	Cytosol	A > G
chr2:24225050:A:G:UTR3:UBXN2A	chr2	24225050	24225050	A	G	UTR3	UBXN2A	ENST000003	.	.	A	G	+	+	0.19444444	29,0,7,0	chr2,242250	Cytosol	A > G
chr2:24225065:A:G:UTR3:UBXN2A	chr2	24225065	24225065	A	G	UTR3	UBXN2A	ENST000003	.	.	A	G	+	+	0.20689655	23,0,6,0	chr2,242250	Cytosol	A > G
chr2:24225114:A:G:UTR3:UBXN2A	chr2	24225114	24225114	A	G	UTR3	UBXN2A	ENST000003	.	.	A	G	+	+	0.13636364	19,0,3,0	chr2,242251	Cytosol	A > G
chr2:24225177:A:G:UTR3:UBXN2A	chr2	24225177	24225177	A	G	UTR3	UBXN2A	ENST000003	.	.	A	G	+	+	0.11111111	16,0,2,0	chr2,242251	Cytosol	A > G
chr2:24225329:A:G:UTR3:UBXN2A	chr2	24225329	24225329	A	G	UTR3	UBXN2A	ENST000003	.	.	A	G	+	+	0.92307692	2,0,24,0	chr2,242253	Cytosol	A > G
chr2:24225331:A:G:UTR3:UBXN2A	chr2	24225331	24225331	A	G	UTR3	UBXN2A	ENST000003	.	.	A	G	+	+	0.35714286	18,0,10,0	chr2,242253	Cytosol	A > G
chr2:24225336:A:G:UTR3:UBXN2A	chr2	24225336	24225336	A	G	UTR3	UBXN2A	ENST000003	.	.	A	G	+	+	0.05882353	32,0,2,0	chr2,242253	Cytosol	A > G
chr2:24225337:A:G:UTR3:UBXN2A	chr2	24225337	24225337	A	G	UTR3	UBXN2A	ENST000003	.	.	A	G	+	+	0.72222222	9,0,26,1	chr2,242253	Cytosol	A > G
chr2:24225379:A:G:UTR3:UBXN2A	chr2	24225379	24225379	A	G	UTR3	UBXN2A	ENST000003	.	.	A	G	+	+	0.07894737	35,0,3,0	chr2,242253	Cytosol	A > G
chr2:24225380:A:G:UTR3:UBXN2A	chr2	24225380	24225380	A	G	UTR3	UBXN2A	ENST000003	.	.	A	G	+	+	0.07894737	35,0,3,0	chr2,242253	Cytosol	A > G
chr2:24225445:A:G:UTR3:UBXN2A	chr2	24225445	24225445	A	G	UTR3	UBXN2A	ENST000003	.	.	A	G	+	+	0.16666667	15,0,3,0	chr2,242254	Cytosol	A > G
chr2:24225460:A:G:UTR3:UBXN2A	chr2	24225460	24225460	A	G	UTR3	UBXN2A	ENST000003	.	.	A	G	+	+	0.31578947	13,0,6,0	chr2,242254	Cytosol	A > G
chr2:24225514:A:G:UTR3:UBXN2A	chr2	24225514	24225514	A	G	UTR3	UBXN2A	ENST000003	.	.	A	G	+	+	0.05714286	33,0,2,0	chr2,242255	Cytosol	A > G
chr2:24225542:A:G:UTR3:UBXN2A	chr2	24225542	24225542	A	G	UTR3	UBXN2A	ENST000003	.	.	A	G	+	+	0.55	9,0,11,0	chr2,242255	Cytosol	A > G
chr2:24225548:A:G:UTR3:UBXN2A	chr2	24225548	24225548	A	G	UTR3	UBXN2A	ENST000003	.	.	A	G	+	+	0.77777778	4,0,14,0	chr2,242255	Cytosol	A > G
chr2:24227377:A:G:UTR3:UBXN2A	chr2	24227377	24227377	A	G	UTR3	UBXN2A	ENST000003	.	.	A	G	+	+	0.11111111	16,0,2,0	chr2,242273	Cytosol	A > G
chr2:24227387:A:G:UTR3:UBXN2A	chr2	24227387	24227387	A	G	UTR3	UBXN2A	ENST000003	.	.	A	G	+	+	0.16666667	10,0,2,0	chr2,242273	Cytosol	A > G
chr2:24227417:A:G:UTR3:UBXN2A	chr2	24227417	24227417	A	G	UTR3	UBXN2A	ENST000003	.	.	A	G	+	+	0.5	4,0,4,0	chr2,242274	Cytosol	A > G
chr2:25047637:A:G:intronic:ADCY	chr2	25047637	25047637	A	G	intronic	ADCY3	.	.	.	A	G	+	-	0.875	1,0,7,0	chr2,250476	Cytosol	A > G
chr2:25047638:A:G:intronic:ADCY	chr2	25047638	25047638	A	G	intronic	ADCY3	.	.	.	A	G	+	-	0.85714286	1,0,6,0	chr2,250476	Cytosol	A > G
chr2:25047684:A:G:intronic:ADCY	chr2	25047684	25047684	A	G	intronic	ADCY3	.	.	.	A	G	+	-	0.8	1,0,4,0	chr2,250476	Cytosol	A > G
chr2:25047716:A:G:intronic:ADCY	chr2	25047716	25047716	A	G	intronic	ADCY3	.	.	.	A	G	+	-	0.5	3,0,3,0	chr2,250477	Cytosol	A > G
chr2:25048333:A:G:intronic:ADCY	chr2	25048333	25048333	A	G	intronic	ADCY3	.	.	.	A	G	+	-	0.25	9,0,3,0	chr2,250483	Cytosol	A > G
chr2:25048407:A:G:intronic:ADCY	chr2	25048407	25048407	A	G	intronic	ADCY3	.	.	.	A	G	+	-	0.16666667	10,0,2,0	chr2,250484	Cytosol	A > G
chr2:27001810:C:T:UTR3:SLC35F6	chr2	27001810	27001810	C	T	UTR3	SLC35F6	ENST000004	.	.	C	T	+	+	0.02857143	0,68,0,2	chr2,270018	Cytosol	C > T
chr2:27255484:T:C:upstream:TME	chr2	27255484	27255484	T	C	upstream	TMEM214	.	.	.	A	G	-		0.5	3,0,3,0	chr2,272554	Cytosol	A > G
chr2:27255495:T:C:upstream:TME	chr2	27255495	27255495	T	C	upstream	TMEM214	.	.	.	A	G	-		0.66666667	2,0,4,0	chr2,272554	Cytosol	A > G
chr2:27580247:A:G:ncRNA_introni	chr2	27580247	27580247	A	G	ncRNA_intro	AC074117.1	.	.	.	A	G	+	+	0.5	6,0,6,0	chr2,275802	Cytosol	A > G
chr2:27580260:A:G:ncRNA_introni	chr2	27580260	27580260	A	G	ncRNA_intro	AC074117.1	.	.	.	A	G	+	+	0.4	6,0,4,0	chr2,275802	Cytosol	A > G
chr2:27580312:A:G:ncRNA_introni	chr2	27580312	27580312	A	G	ncRNA_intro	AC074117.1	.	.	.	A	G	+	+	0.27272727	8,0,3,0	chr2,275803	Cytosol	A > G
chr2:32532725:A:G:UTR3:YIPF4	chr2	32532725	32532725	A	G	UTR3	YIPF4	ENST000002	.	.	A	G	+	+	0.08333333	22,0,2,0	chr2,325327	Cytosol	A > G
chr2:32532741:A:G:UTR3:YIPF4	chr2	32532741	32532741	A	G	UTR3	YIPF4	ENST000002	.	.	A	G	+	+	0.12820513	34,0,5,0	chr2,325327	Cytosol	A > G
chr2:32532742:A:G:UTR3:YIPF4	chr2	32532742	32532742	A	G	UTR3	YIPF4	ENST000002	.	.	A	G	+	+	0.05555556	34,0,2,0	chr2,325327	Cytosol	A > G
chr2:32532809:A:G:UTR3:YIPF4	chr2	32532809	32532809	A	G	UTR3	YIPF4	ENST000002	.	.	A	G	+	+	0.16666667	30,0,6,0	chr2,325328	Cytosol	A > G
chr2:32532810:A:G:UTR3:YIPF4	chr2	32532810	32532810	A	G	UTR3	YIPF4	ENST000002	.	.	A	G	+	+	0.35135135	24,0,13,0	chr2,325328	Cytosol	A > G
chr2:32532819:A:G:UTR3:YIPF4	chr2	32532819	32532819	A	G	UTR3	YIPF4	ENST000002	.	.	A	G	+	+	0.10344828	26,0,3,0	chr2,325328	Cytosol	A > G
chr2:32532820:A:G:UTR3:YIPF4	chr2	32532820	32532820	A	G	UTR3	YIPF4	ENST000002	.	.	A	G	+	+	0.22222222	21,0,6,0	chr2,325328	Cytosol	A > G
chr2:32532834:A:G:UTR3:YIPF4	chr2	32532834	32532834	A	G	UTR3	YIPF4	ENST000002	.	.	A	G	+	+	0.11111111	16,0,2,0	chr2,325328	Cytosol	A > G
chr2:32532840:A:G:UTR3:YIPF4	chr2	32532840	32532840	A	G	UTR3	YIPF4	ENST000002	.	.	A	G	+	+	0.14285714	18,0,3,0	chr2,325328	Cytosol	A > G
chr2:32532845:A:G:UTR3:YIPF4	chr2	32532845	32532845	A	G	UTR3	YIPF4	ENST000002	.	.	A	G	+	+	0.18181818	18,0,4,0	chr2,325328	Cytosol	A > G
chr2:32532849:A:G:UTR3:YIPF4	chr2	32532849	32532849	A	G	UTR3	YIPF4	ENST000002	.	.	A	G	+	+	0.36842105	12,0,7,0	chr2,325328	Cytosol	A > G
chr2:32532878:A:G:UTR3:YIPF4	chr2	32532878	32532878	A	G	UTR3	YIPF4	ENST000002	.	.	A	G	+	+	0.34782609	15,0,8,0	chr2,325328	Cytosol	A > G
chr2:32532887:A:G:UTR3:YIPF4	chr2	32532887	32532887	A	G	UTR3	YIPF4	ENST000002	.	.	A	G	+	+	0.31818182	15,0,7,0	chr2,325328	Cytosol	A > G
chr2:32533223:A:G:UTR3:YIPF4	chr2	32533223	32533223	A	G	UTR3	YIPF4	ENST000002	.	.	A	G	+	+	0.66666667	3,0,6,0	chr2,325332	Cytosol	A > G
chr2:32533235:A:G:UTR3:YIPF4	chr2	32533235	32533235	A	G	UTR3	YIPF4	ENST000002	.	.	A	G	+	+	0.54545455	5,0,6,0	chr2,325332	Cytosol	A > G
chr2:32533240:A:G:UTR3:YIPF4	chr2	32533240	32533240	A	G	UTR3	YIPF4	ENST000002	.	.	A	G	+	+	0.7	3,0,7,0	chr2,325332	Cytosol	A > G
chr2:32533243:A:G:UTR3:YIPF4	chr2	32533243	32533243	A	G	UTR3	YIPF4	ENST000002	.	.	A	G	+	+	0.4	6,0,4,0	chr2,325332	Cytosol	A > G
chr2:32533308:A:G:UTR3:YIPF4	chr2	32533308	32533308	A	G	UTR3	YIPF4	ENST000002	.	.	A	G	+	+	0.33333333	4,0,2,0	chr2,325333	Cytosol	A > G
chr2:32533318:A:G:UTR3:YIPF4	chr2	32533318	32533318	A	G	UTR3	YIPF4	ENST000002	.	.	A	G	+	+	0.66666667	2,0,4,0	chr2,325333	Cytosol	A > G
chr2:32533533:A:G:UTR3:YIPF4	chr2	32533533	32533533	A	G	UTR3	YIPF4	ENST000002	.	.	A	G	+	+	0.8	2,0,8,0	chr2,325335	Cytosol	A > G
chr2:32533596:A:G:UTR3:YIPF4	chr2	32533596	32533596	A	G	UTR3	YIPF4	ENST000002	.	.	A	G	+	+	0.78571429	3,0,11,0	chr2,325335	Cytosol	A > G
chr2:32533618:A:G:UTR3:YIPF4	chr2	32533618	32533618	A	G	UTR3	YIPF4	ENST000002	.	.	A	G	+	+	0.38461538	8,0,5,0	chr2,325336	Cytosol	A > G
chr2:32533627:A:G:UTR3:YIPF4	chr2	32533627	32533627	A	G	UTR3	YIPF4	ENST000002	.	.	A	G	+	+	0.71428571	4,0,10,0	chr2,325336	Cytosol	A > G
chr2:32533630:A:G:UTR3:YIPF4	chr2	32533630	32533630	A	G	UTR3	YIPF4	ENST000002	.	.	A	G	+	+	0.30769231	9,0,4,0	chr2,325336	Cytosol	A > G
chr2:32533661:A:G:UTR3:YIPF4	chr2	32533661	32533661	A	G	UTR3	YIPF4	ENST000002	.	.	A	G	+	+	0.4	9,0,6,0	chr2,325336	Cytosol	A > G
chr2:32533662:A:G:UTR3:YIPF4	chr2	32533662	32533662	A	G	UTR3	YIPF4	ENST000002	.	.	A	G	+	+	0.66666667	5,0,10,0	chr2,325336	Cytosol	A > G
chr2:32533679:A:G:UTR3:YIPF4	chr2	32533679	32533679	A	G	UTR3	YIPF4	ENST000002	.	.	A	G	+	+	0.46666667	7,0,7,1	chr2,325336	Cytosol	A > G
chr2:32533707:A:G:UTR3:YIPF4	chr2	32533707	32533707	A	G	UTR3	YIPF4	ENST000002	.	.	A	G	+	+	0.54545455	10,0,12,0	chr2,325337	Cytosol	A > G

chr2:32534821:A:G:UTR3:YIPF4	chr2	32534821	32534821	A	G	UTR3	YIPF4	ENST000002	.	.	A	G	+	+	0.32	17,0,8,0	chr2,325348	Cytosol	A > G
chr2:32534827:A:G:UTR3:YIPF4	chr2	32534827	32534827	A	G	UTR3	YIPF4	ENST000002	.	.	A	G	+	+	0.34782609	15,0,8,0	chr2,325348	Cytosol	A > G
chr2:32534838:A:G:UTR3:YIPF4	chr2	32534838	32534838	A	G	UTR3	YIPF4	ENST000002	.	.	A	G	+	+	0.55	9,0,11,0	chr2,325348	Cytosol	A > G
chr2:32534858:A:G:UTR3:YIPF4	chr2	32534858	32534858	A	G	UTR3	YIPF4	ENST000002	.	.	A	G	+	+	0.4	15,0,10,0	chr2,325348	Cytosol	A > G
chr2:32534861:A:G:UTR3:YIPF4	chr2	32534861	32534861	A	G	UTR3	YIPF4	ENST000002	.	.	A	G	+	+	0.17857143	23,0,5,0	chr2,325348	Cytosol	A > G
chr2:32534875:A:G:UTR3:YIPF4	chr2	32534875	32534875	A	G	UTR3	YIPF4	ENST000002	.	.	A	G	+	+	0.43478261	13,0,10,0	chr2,325348	Cytosol	A > G
chr2:32535691:A:G:UTR3:YIPF4	chr2	32535691	32535691	A	G	UTR3	YIPF4	ENST000002	.	.	A	G	+	+	0.35849057	34,0,19,0	chr2,325356	Cytosol	A > G
chr2:32535692:A:G:UTR3:YIPF4	chr2	32535692	32535692	A	G	UTR3	YIPF4	ENST000002	.	.	A	G	+	+	0.36538462	33,0,19,0	chr2,325356	Cytosol	A > G
chr2:32535746:A:G:UTR3:YIPF4	chr2	32535746	32535746	A	G	UTR3	YIPF4	ENST000002	.	.	A	G	+	+	0.26	37,0,13,0	chr2,325357	Cytosol	A > G
chr2:32535747:A:G:UTR3:YIPF4	chr2	32535747	32535747	A	G	UTR3	YIPF4	ENST000002	.	.	A	G	+	+	0.31914894	32,0,15,0	chr2,325357	Cytosol	A > G
chr2:32535748:A:G:UTR3:YIPF4	chr2	32535748	32535748	A	G	UTR3	YIPF4	ENST000002	.	.	A	G	+	+	0.4	30,0,20,0	chr2,325357	Cytosol	A > G
chr2:32535766:A:G:UTR3:YIPF4	chr2	32535766	32535766	A	G	UTR3	YIPF4	ENST000002	.	.	A	G	+	+	0.17021277	39,0,8,0	chr2,325357	Cytosol	A > G
chr2:32535797:A:G:UTR3:YIPF4	chr2	32535797	32535797	A	G	UTR3	YIPF4	ENST000002	.	.	A	G	+	+	0.15909091	37,0,7,0	chr2,325357	Cytosol	A > G
chr2:32535836:A:G:UTR3:YIPF4	chr2	32535836	32535836	A	G	UTR3	YIPF4	ENST000002	.	.	A	G	+	+	0.44897959	27,0,22,0	chr2,325358	Cytosol	A > G
chr2:32535865:A:G:UTR3:YIPF4	chr2	32535865	32535865	A	G	UTR3	YIPF4	ENST000002	.	.	A	G	+	+	0.28301887	38,0,15,0	chr2,325358	Cytosol	A > G
chr2:32535902:A:G:UTR3:YIPF4	chr2	32535902	32535902	A	G	UTR3	YIPF4	ENST000002	.	.	A	G	+	+	0.24390244	31,0,10,0	chr2,325359	Cytosol	A > G
chr2:37329180:T:C:UTR3:EIF2AK2	chr2	37329180	37329180	T	C	UTR3	EIF2AK2	ENST000002	.	.	A	G	-	-	0.04255319	90,0,4,0	chr2,373291	Cytosol	A > G
chr2:37329213:T:C:UTR3:EIF2AK2	chr2	37329213	37329213	T	C	UTR3	EIF2AK2	ENST000002	.	.	A	G	-	-	0.02564103	76,0,2,0	chr2,373292	Cytosol	A > G
chr2:37329214:T:C:UTR3:EIF2AK2	chr2	37329214	37329214	T	C	UTR3	EIF2AK2	ENST000002	.	.	A	G	-	-	0.30769231	54,0,24,0	chr2,373292	Cytosol	A > G
chr2:37329217:T:C:UTR3:EIF2AK2	chr2	37329217	37329217	T	C	UTR3	EIF2AK2	ENST000002	.	.	A	G	-	-	0.06666667	70,0,5,0	chr2,373292	Cytosol	A > G
chr2:37329235:T:C:UTR3:EIF2AK2	chr2	37329235	37329235	T	C	UTR3	EIF2AK2	ENST000002	.	.	A	G	-	-	0.09677419	56,0,6,0	chr2,373292	Cytosol	A > G
chr2:37329238:T:C:UTR3:EIF2AK2	chr2	37329238	37329238	T	C	UTR3	EIF2AK2	ENST000002	.	.	A	G	-	-	0.28985507	49,0,20,0	chr2,373292	Cytosol	A > G
chr2:37329261:T:C:UTR3:EIF2AK2	chr2	37329261	37329261	T	C	UTR3	EIF2AK2	ENST000002	.	.	A	G	-	-	0.21348315	70,0,19,0	chr2,373292	Cytosol	A > G
chr2:37329283:T:C:UTR3:EIF2AK2	chr2	37329283	37329283	T	C	UTR3	EIF2AK2	ENST000002	.	.	A	G	-	-	0.28865979	69,0,28,0	chr2,373292	Cytosol	A > G
chr2:37329300:T:C:UTR3:EIF2AK2	chr2	37329300	37329300	T	C	UTR3	EIF2AK2	ENST000002	.	.	A	G	-	-	0.12765957	82,0,12,0	chr2,373292	Cytosol	A > G
chr2:37329301:T:C:UTR3:EIF2AK2	chr2	37329301	37329301	T	C	UTR3	EIF2AK2	ENST000002	.	.	A	G	-	-	0.07368421	88,0,7,0	chr2,373293	Cytosol	A > G
chr2:37329307:T:C:UTR3:EIF2AK2	chr2	37329307	37329307	T	C	UTR3	EIF2AK2	ENST000002	.	.	A	G	-	-	0.46464646	53,0,46,0	chr2,373293	Cytosol	A > G
chr2:37329313:T:C:UTR3:EIF2AK2	chr2	37329313	37329313	T	C	UTR3	EIF2AK2	ENST000002	.	.	A	G	-	-	0.03030303	96,0,3,0	chr2,373293	Cytosol	A > G
chr2:37329314:T:C:UTR3:EIF2AK2	chr2	37329314	37329314	T	C	UTR3	EIF2AK2	ENST000002	.	.	A	G	-	-	0.08510638	86,0,8,0	chr2,373293	Cytosol	A > G
chr2:37329320:T:C:UTR3:EIF2AK2	chr2	37329320	37329320	T	C	UTR3	EIF2AK2	ENST000002	.	.	A	G	-	-	0.08536585	75,0,7,0	chr2,373293	Cytosol	A > G
chr2:37329331:T:C:UTR3:EIF2AK2	chr2	37329331	37329331	T	C	UTR3	EIF2AK2	ENST000002	.	.	A	G	-	-	0.1627907	72,0,14,0	chr2,373293	Cytosol	A > G
chr2:37329347:T:C:UTR3:EIF2AK2	chr2	37329347	37329347	T	C	UTR3	EIF2AK2	ENST000002	.	.	A	G	-	-	0.32098765	55,0,26,0	chr2,373293	Cytosol	A > G
chr2:37329352:T:C:UTR3:EIF2AK2	chr2	37329352	37329352	T	C	UTR3	EIF2AK2	ENST000002	.	.	A	G	-	-	0.15189873	67,0,12,0	chr2,373293	Cytosol	A > G
chr2:37330368:T:C:UTR3:EIF2AK2	chr2	37330368	37330368	T	C	UTR3	EIF2AK2	ENST000002	.	.	A	G	-	-	0.4	6,0,4,0	chr2,373303	Cytosol	A > G
chr2:37330431:T:C:UTR3:EIF2AK2	chr2	37330431	37330431	T	C	UTR3	EIF2AK2	ENST000002	.	.	A	G	-	-	0.66666667	2,0,4,0	chr2,373304	Cytosol	A > G
chr2:37330435:T:C:UTR3:EIF2AK2	chr2	37330435	37330435	T	C	UTR3	EIF2AK2	ENST000002	.	.	A	G	-	-	0.83333333	1,0,5,0	chr2,373304	Cytosol	A > G
chr2:37330436:T:C:UTR3:EIF2AK2	chr2	37330436	37330436	T	C	UTR3	EIF2AK2	ENST000002	.	.	A	G	-	-	0.83333333	1,0,5,0	chr2,373304	Cytosol	A > G
chr2:37330503:T:C:UTR3:EIF2AK2	chr2	37330503	37330503	T	C	UTR3	EIF2AK2	ENST000002	.	.	A	G	-	-	0.73333333	4,0,11,0	chr2,373305	Cytosol	A > G
chr2:37330506:T:C:UTR3:EIF2AK2	chr2	37330506	37330506	T	C	UTR3	EIF2AK2	ENST000002	.	.	A	G	-	-	0.21428571	11,0,3,0	chr2,373305	Cytosol	A > G
chr2:37330518:T:C:UTR3:EIF2AK2	chr2	37330518	37330518	T	C	UTR3	EIF2AK2	ENST000002	.	.	A	G	-	-	0.21428571	11,0,3,0	chr2,373305	Cytosol	A > G
chr2:37330540:T:C:UTR3:EIF2AK2	chr2	37330540	37330540	T	C	UTR3	EIF2AK2	ENST000002	.	.	A	G	-	-	0.11111111	16,0,2,0	chr2,373305	Cytosol	A > G
chr2:37330542:T:C:UTR3:EIF2AK2	chr2	37330542	37330542	T	C	UTR3	EIF2AK2	ENST000002	.	.	A	G	-	-	0.22222222	14,0,4,0	chr2,373305	Cytosol	A > G
chr2:37330566:T:C:UTR3:EIF2AK2	chr2	37330566	37330566	T	C	UTR3	EIF2AK2	ENST000002	.	.	A	G	-	-	0.33333333	10,0,5,0	chr2,373305	Cytosol	A > G
chr2:37330611:T:C:UTR3:EIF2AK2	chr2	37330611	37330611	T	C	UTR3	EIF2AK2	ENST000002	.	.	A	G	-	-	0.13043478	20,0,3,0	chr2,373306	Cytosol	A > G
chr2:37330612:T:C:UTR3:EIF2AK2	chr2	37330612	37330612	T	C	UTR3	EIF2AK2	ENST000002	.	.	A	G	-	-	0.20833333	19,0,5,0	chr2,373306	Cytosol	A > G
chr2:37330620:T:C:UTR3:EIF2AK2	chr2	37330620	37330620	T	C	UTR3	EIF2AK2	ENST000002	.	.	A	G	-	-	0.13043478	20,0,3,0	chr2,373306	Cytosol	A > G
chr2:37331019:T:C:UTR3:EIF2AK2	chr2	37331019	37331019	T	C	UTR3	EIF2AK2	ENST000002	.	.	A	G	-	-	0.06451613	58,0,4,0	chr2,373310	Cytosol	A > G
chr2:37331023:T:C:UTR3:EIF2AK2	chr2	37331023	37331023	T	C	UTR3	EIF2AK2	ENST000002	.	.	A	G	-	-	0.05084746	56,0,3,0	chr2,373310	Cytosol	A > G
chr2:37331025:T:C:UTR3:EIF2AK2	chr2	37331025	37331025	T	C	UTR3	EIF2AK2	ENST000002	.	.	A	G	-	-	0.15789474	48,0,9,0	chr2,373310	Cytosol	A > G
chr2:37331037:T:C:UTR3:EIF2AK2	chr2	37331037	37331037	T	C	UTR3	EIF2AK2	ENST000002	.	.	A	G	-	-	0.06	47,0,3,0	chr2,373310	Cytosol	A > G
chr2:37331038:T:C:UTR3:EIF2AK2	chr2	37331038	37331038	T	C	UTR3	EIF2AK2	ENST000002	.	.	A	G	-	-	0.20689655	46,0,12,0	chr2,373310	Cytosol	A > G
chr2:37331065:T:C:UTR3:EIF2AK2	chr2	37331065	37331065	T	C	UTR3	EIF2AK2	ENST000002	.	.	A	G	-	-	0.27272727	32,0,12,0	chr2,373310	Cytosol	A > G
chr2:37331070:T:C:UTR3:EIF2AK2	chr2	37331070	37331070	T	C	UTR3	EIF2AK2	ENST000002	.	.	A	G	-	-	0.05405405	35,0,2,0	chr2,373310	Cytosol	A > G
chr2:37331077:T:C:UTR3:EIF2AK2	chr2	37331077	37331077	T	C	UTR3	EIF2AK2	ENST000002	.	.	A	G	-	-	0.06976744	40,0,3,0	chr2,373310	Cytosol	A > G
chr2:37331089:T:C:UTR3:EIF2AK2	chr2	37331089	37331089	T	C	UTR3	EIF2AK2	ENST000002	.	.	A	G	-	-	0.0952381	38,0,4,0	chr2,373310	Cytosol	A > G
chr2:37331116:T:C:UTR3:EIF2AK2	chr2	37331116	37331116	T	C	UTR3	EIF2AK2	ENST000002	.	.	A	G	-	-	0.35135135	24,0,13,0	chr2,373311	Cytosol	A > G
chr2:37331119:T:C:UTR3:EIF2AK2	chr2	37331119	37331119	T	C	UTR3	EIF2AK2	ENST000002	.	.	A	G	-	-	0.19444444	29,0,7,0	chr2,373311	Cytosol	A > G
chr2:37331125:T:C:UTR3:EIF2AK2	chr2	37331125	37331125	T	C	UTR3	EIF2AK2	ENST000002	.	.	A	G	-	-	0.05128205	37,0,2,0	chr2,373311	Cytosol	A > G
chr2:37331161:T:C:UTR3:EIF2AK2	chr2	37331161	37331161	T	C	UTR3	EIF2AK2	ENST000002	.	.	A	G	-	-	0.15	17,0,3,0	chr2,373311	Cytosol	A > G
chr2:37331171:T:C:UTR3:EIF2AK2	chr2	37331171	37331171	T	C	UTR3	EIF2AK2	ENST000002	.	.	A	G	-	-	0.92857143	1,0,13,0	chr2,373311	Cytosol	A > G
chr2:37331200:T:C:UTR3:EIF2AK2	chr2	37331200	37331200	T	C	UTR3	EIF2AK2	ENST000002	.	.	A	G	-	-	0.44444444	5,0,4,0	chr2,373311	Cytosol	A > G
chr2:37331207:T:C:UTR3:EIF2AK2	chr2	37331207	37331207	T	C	UTR3	EIF2AK2	ENST000002	.	.	A	G	-	-	0.5	6,0,6,0	chr2,373312	Cytosol	A > G
chr2:37331220:T:C:UTR3:EIF2AK2	chr2	37331220	37331220	T	C	UTR3	EIF2AK2	ENST000002	.	.	A	G	-	-	0.2	12,0,3,0	chr2,373312	Cytosol	A > G
chr2:37331221:T:C:UTR3:EIF2AK2	chr2	37331221	37331221	T	C	UTR3	EIF2AK2	ENST000002	.	.	A	G	-	-	0.46666667	8,0,7,0	chr2,373312	Cytosol	A > G

chr2:37331236:T:C:UTR3:EIF2AK2	chr2	37331236	37331236	T	C	UTR3	EIF2AK2	ENST000002	.	.	A	G	-	-	0.27777778	13,0,5,0	chr2,373312	Cytosol	A > G
chr2:37331239:T:C:UTR3:EIF2AK2	chr2	37331239	37331239	T	C	UTR3	EIF2AK2	ENST000002	.	.	A	G	-	-	0.23529412	13,0,4,0	chr2,373312	Cytosol	A > G
chr2:37331269:T:C:UTR3:EIF2AK2	chr2	37331269	37331269	T	C	UTR3	EIF2AK2	ENST000002	.	.	A	G	-	-	0.11111111	16,0,2,0	chr2,373312	Cytosol	A > G
chr2:37331525:T:C:UTR3:EIF2AK2	chr2	37331525	37331525	T	C	UTR3	EIF2AK2	ENST000002	.	.	A	G	-	-	0.3	21,0,9,0	chr2,373315	Cytosol	A > G
chr2:37331531:T:C:UTR3:EIF2AK2	chr2	37331531	37331531	T	C	UTR3	EIF2AK2	ENST000002	.	.	A	G	-	-	0.06451613	29,0,2,0	chr2,373315	Cytosol	A > G
chr2:37331547:T:C:UTR3:EIF2AK2	chr2	37331547	37331547	T	C	UTR3	EIF2AK2	ENST000002	.	.	A	G	-	-	0.21428571	22,0,6,0	chr2,373315	Cytosol	A > G
chr2:37331555:T:C:UTR3:EIF2AK2	chr2	37331555	37331555	T	C	UTR3	EIF2AK2	ENST000002	.	.	A	G	-	-	0.07407407	25,0,2,0	chr2,373315	Cytosol	A > G
chr2:37331578:T:C:UTR3:EIF2AK2	chr2	37331578	37331578	T	C	UTR3	EIF2AK2	ENST000002	.	.	A	G	-	-	0.125	14,0,2,0	chr2,373315	Cytosol	A > G
chr2:37331614:T:C:UTR3:EIF2AK2	chr2	37331614	37331614	T	C	UTR3	EIF2AK2	ENST000002	.	.	A	G	-	-	0.2962963	19,0,8,0	chr2,373316	Cytosol	A > G
chr2:37331627:T:C:UTR3:EIF2AK2	chr2	37331627	37331627	T	C	UTR3	EIF2AK2	ENST000002	.	.	A	G	-	-	0.05405405	35,0,2,0	chr2,373316	Cytosol	A > G
chr2:37331640:T:C:UTR3:EIF2AK2	chr2	37331640	37331640	T	C	UTR3	EIF2AK2	ENST000002	.	.	A	G	-	-	0.14634146	35,0,6,0	chr2,373316	Cytosol	A > G
chr2:37331642:T:C:UTR3:EIF2AK2	chr2	37331642	37331642	T	C	UTR3	EIF2AK2	ENST000002	.	.	A	G	-	-	0.19565217	37,0,9,0	chr2,373316	Cytosol	A > G
chr2:37331645:T:C:UTR3:EIF2AK2	chr2	37331645	37331645	T	C	UTR3	EIF2AK2	ENST000002	.	.	A	G	-	-	0.28888889	32,0,13,0	chr2,373316	Cytosol	A > G
chr2:37331745:T:C:UTR3:EIF2AK2	chr2	37331745	37331745	T	C	UTR3	EIF2AK2	ENST000002	.	.	A	G	-	-	0.19117647	55,0,13,0	chr2,373317	Cytosol	A > G
chr2:37331750:T:C:UTR3:EIF2AK2	chr2	37331750	37331750	T	C	UTR3	EIF2AK2	ENST000002	.	.	A	G	-	-	0.171875	53,0,11,0	chr2,373317	Cytosol	A > G
chr2:37429526:A:G:UTR3:AC00739	chr2	37429526	37429526	A	G	UTR3	AC007390.5	ENST000004	.	.	A	G	+	+-	0.66829268	68,0,137,0	chr2,374295	Cytosol	A > G
chr2:37429533:A:G:UTR3:AC00739	chr2	37429533	37429533	A	G	UTR3	AC007390.5	ENST000004	.	.	A	G	+	+-	0.12149533	188,0,26,0	chr2,374295	Cytosol	A > G
chr2:37429578:A:G:UTR3:AC00739	chr2	37429578	37429578	A	G	UTR3	AC007390.5	ENST000004	.	.	A	G	+	+-	0.15183246	162,0,29,0	chr2,374295	Cytosol	A > G
chr2:37429589:A:G:UTR3:AC00739	chr2	37429589	37429589	A	G	UTR3	AC007390.5	ENST000004	.	.	A	G	+	+-	0.02564103	190,0,5,0	chr2,374295	Cytosol	A > G
chr2:37429612:A:G:UTR3:AC00739	chr2	37429612	37429612	A	G	UTR3	AC007390.5	ENST000004	.	.	A	G	+	+-	0.09027778	131,0,13,0	chr2,374296	Cytosol	A > G
chr2:37429626:A:G:UTR3:AC00739	chr2	37429626	37429626	A	G	UTR3	AC007390.5	ENST000004	.	.	A	G	+	+-	0.50757576	65,0,67,0	chr2,374296	Cytosol	A > G
chr2:37429627:A:G:UTR3:AC00739	chr2	37429627	37429627	A	G	UTR3	AC007390.5	ENST000004	.	.	A	G	+	+-	0.57462687	57,0,77,0	chr2,374296	Cytosol	A > G
chr2:37429628:A:G:UTR3:AC00739	chr2	37429628	37429628	A	G	UTR3	AC007390.5	ENST000004	.	.	A	G	+	+-	0.23308271	102,0,31,0	chr2,374296	Cytosol	A > G
chr2:37429629:A:G:UTR3:AC00739	chr2	37429629	37429629	A	G	UTR3	AC007390.5	ENST000004	.	.	A	G	+	+-	0.1048951	128,0,15,0	chr2,374296	Cytosol	A > G
chr2:37429632:A:G:UTR3:AC00739	chr2	37429632	37429632	A	G	UTR3	AC007390.5	ENST000004	.	.	A	G	+	+-	0.23129252	113,0,34,0	chr2,374296	Cytosol	A > G
chr2:37429634:A:G:UTR3:AC00739	chr2	37429634	37429634	A	G	UTR3	AC007390.5	ENST000004	.	.	A	G	+	+-	0.01333333	148,0,2,0	chr2,374296	Cytosol	A > G
chr2:37429635:A:G:UTR3:AC00739	chr2	37429635	37429635	A	G	UTR3	AC007390.5	ENST000004	.	.	A	G	+	+-	0.22147651	116,0,33,0	chr2,374296	Cytosol	A > G
chr2:37429667:A:G:UTR3:AC00739	chr2	37429667	37429667	A	G	UTR3	AC007390.5	ENST000004	.	.	A	G	+	+-	0.10160428	168,0,19,0	chr2,374296	Cytosol	A > G
chr2:37429668:A:G:UTR3:AC00739	chr2	37429668	37429668	A	G	UTR3	AC007390.5	ENST000004	.	.	A	G	+	+-	0.17741935	153,0,33,0	chr2,374296	Cytosol	A > G
chr2:37429677:A:G:UTR3:AC00739	chr2	37429677	37429677	A	G	UTR3	AC007390.5	ENST000004	.	.	A	G	+	+-	0.41758242	106,0,76,0	chr2,374296	Cytosol	A > G
chr2:37429690:A:G:UTR3:AC00739	chr2	37429690	37429690	A	G	UTR3	AC007390.5	ENST000004	.	.	A	G	+	+-	0.03296703	176,0,6,0	chr2,374296	Cytosol	A > G
chr2:37429729:A:G:UTR3:AC00739	chr2	37429729	37429729	A	G	UTR3	AC007390.5	ENST000004	.	.	A	G	+	+-	0.05797101	130,0,8,0	chr2,374297	Cytosol	A > G
chr2:37429733:A:G:UTR3:AC00739	chr2	37429733	37429733	A	G	UTR3	AC007390.5	ENST000004	.	.	A	G	+	+-	0.62307692	49,0,81,0	chr2,374297	Cytosol	A > G
chr2:37429737:A:G:UTR3:AC00739	chr2	37429737	37429737	A	G	UTR3	AC007390.5	ENST000004	.	.	A	G	+	+-	0.2238806	104,0,30,0	chr2,374297	Cytosol	A > G
chr2:37429738:A:G:UTR3:AC00739	chr2	37429738	37429738	A	G	UTR3	AC007390.5	ENST000004	.	.	A	G	+	+-	0.33076923	87,0,43,0	chr2,374297	Cytosol	A > G
chr2:37431474:A:G:UTR3:AC00739	chr2	37431474	37431474	A	G	UTR3	AC007390.5	ENST000004	.	.	A	G	+	+-	0.11931818	155,0,21,0	chr2,374314	Cytosol	A > G
chr2:37431485:A:G:UTR3:AC00739	chr2	37431485	37431485	A	G	UTR3	AC007390.5	ENST000004	.	.	A	G	+	+-	0.28292683	147,0,58,0	chr2,374314	Cytosol	A > G
chr2:37431547:A:G:UTR3:AC00739	chr2	37431547	37431547	A	G	UTR3	AC007390.5	ENST000004	.	.	A	G	+	+-	0.02631579	407,0,11,0	chr2,374315	Cytosol	A > G
chr2:37431681:A:G:UTR3:AC00739	chr2	37431681	37431681	A	G	UTR3	AC007390.5	ENST000004	.	.	A	G	+	+-	0.40725806	147,0,101,0	chr2,374316	Cytosol	A > G
chr2:38961546:G:A:UTR3:GALM	chr2	38961546	38961546	G	A	UTR3	GALM	ENST000002	.	.	G	A	+	+	0.125	3,0,21,0	chr2,389615	Cytosol	G > A
chr2:39006934:A:G:intronic:GEMII	chr2	39006934	39006934	A	G	intronic	GEMIN6	.	.	.	A	G	+	+	0.2	8,0,2,0	chr2,390069	Cytosol	A > G
chr2:39007632:A:G:intronic:GEMII	chr2	39007632	39007632	A	G	intronic	GEMIN6	.	.	.	A	G	+	+	0.5	3,0,3,0	chr2,390076	Cytosol	A > G
chr2:39007633:A:G:intronic:GEMII	chr2	39007633	39007633	A	G	intronic	GEMIN6	.	.	.	A	G	+	+	0.28571429	5,0,2,0	chr2,390076	Cytosol	A > G
chr2:39009355:A:G:UTR3:GEMIN6	chr2	39009355	39009355	A	G	UTR3	GEMIN6	ENST000002	.	.	A	G	+	+	0.11827957	82,0,11,0	chr2,390093	Cytosol	A > G
chr2:39701980:A:G:ncRNA_introni	chr2	39701980	39701980	A	G	ncRNA_intro	AC007246.3	.	.	.	A	G	+	+	0.1	18,0,2,0	chr2,397019	Cytosol	A > G
chr2:39701981:A:G:ncRNA_introni	chr2	39701981	39701981	A	G	ncRNA_intro	AC007246.3	.	.	.	A	G	+	+	0.21052632	15,0,4,0	chr2,397019	Cytosol	A > G
chr2:39702019:A:G:ncRNA_introni	chr2	39702019	39702019	A	G	ncRNA_intro	AC007246.3	.	.	.	A	G	+	+	0.07692308	24,0,2,0	chr2,397020	Cytosol	A > G
chr2:39702024:A:G:ncRNA_introni	chr2	39702024	39702024	A	G	ncRNA_intro	AC007246.3	.	.	.	A	G	+	+	0.08333333	33,0,3,0	chr2,397020	Cytosol	A > G
chr2:39702058:A:G:ncRNA_introni	chr2	39702058	39702058	A	G	ncRNA_intro	AC007246.3	.	.	.	A	G	+	+	0.03508772	55,0,2,0	chr2,397020	Cytosol	A > G
chr2:39702088:A:G:ncRNA_introni	chr2	39702088	39702088	A	G	ncRNA_intro	AC007246.3	.	.	.	A	G	+	+	0.17391304	38,0,8,0	chr2,397020	Cytosol	A > G
chr2:39702115:A:G:ncRNA_introni	chr2	39702115	39702115	A	G	ncRNA_intro	AC007246.3	.	.	.	A	G	+	+	0.13793103	25,0,4,0	chr2,397021	Cytosol	A > G
chr2:39702128:A:G:ncRNA_introni	chr2	39702128	39702128	A	G	ncRNA_intro	AC007246.3	.	.	.	A	G	+	+	0.22222222	21,0,6,0	chr2,397021	Cytosol	A > G
chr2:39702141:A:G:ncRNA_introni	chr2	39702141	39702141	A	G	ncRNA_intro	AC007246.3	.	.	.	A	G	+	+	0.07692308	24,0,2,0	chr2,397021	Cytosol	A > G
chr2:39702147:A:G:ncRNA_introni	chr2	39702147	39702147	A	G	ncRNA_intro	AC007246.3	.	.	.	A	G	+	+	0.07407407	25,0,2,0	chr2,397021	Cytosol	A > G
chr2:39702161:A:G:ncRNA_introni	chr2	39702161	39702161	A	G	ncRNA_intro	AC007246.3	.	.	.	A	G	+	+	0.25	21,0,7,0	chr2,397021	Cytosol	A > G
chr2:39702489:A:G:ncRNA_introni	chr2	39702489	39702489	A	G	ncRNA_intro	AC007246.3	.	.	.	A	G	+	+	0.5	3,0,3,0	chr2,397024	Cytosol	A > G
chr2:39702576:A:G:ncRNA_introni	chr2	39702576	39702576	A	G	ncRNA_intro	AC007246.3	.	.	.	A	G	+	+	0.6	4,0,6,0	chr2,397025	Cytosol	A > G
chr2:39704156:A:G:ncRNA_introni	chr2	39704156	39704156	A	G	ncRNA_intro	AC007246.3	.	.	.	A	G	+	+	0.18181818	9,0,2,0	chr2,397041	Cytosol	A > G
chr2:39704221:A:G:ncRNA_introni	chr2	39704221	39704221	A	G	ncRNA_intro	AC007246.3	.	.	.	A	G	+	+	0.53846154	6,0,7,0	chr2,397042	Cytosol	A > G
chr2:39704257:A:G:ncRNA_introni	chr2	39704257	39704257	A	G	ncRNA_intro	AC007246.3	.	.	.	A	G	+	+	0.25	9,0,3,0	chr2,397042	Cytosol	A > G
chr2:39704258:A:G:ncRNA_introni	chr2	39704258	39704258	A	G	ncRNA_intro	AC007246.3	.	.	.	A	G	+	+	0.18181818	9,0,2,0	chr2,397042	Cytosol	A > G
chr2:39704266:A:G:ncRNA_introni	chr2	39704266	39704266	A	G	ncRNA_intro	AC007246.3	.	.	.	A	G	+	+	0.27272727	8,0,3,0	chr2,397042	Cytosol	A > G
chr2:39704292:A:G:ncRNA_introni	chr2	39704292	39704292	A	G	ncRNA_intro	AC007246.3	.	.	.	A	G	+	+	0.25	6,0,2,0	chr2,397042	Cytosol	A > G
chr2:39704327:A:G:ncRNA_introni	chr2	39704327	39704327	A	G	ncRNA_intro	AC007246.3	.	.	.	A	G	+	+	0.83333333	1,0,5,0	chr2,397043	Cytosol	A > G

chr2:39704366:A:G:ncRNA_introni	chr2	39704366	39704366	A	G	ncRNA_intro	AC007246.3	.	.	.	A	G	+	+	0.66666667	2,0,4,0	chr2,397043	Cytosol	A > G
chr2:39704371:A:G:ncRNA_introni	chr2	39704371	39704371	A	G	ncRNA_intro	AC007246.3	.	.	.	A	G	+	+	0.5	3,0,3,0	chr2,397043	Cytosol	A > G
chr2:44639539:A:G:intronic:CAMK	chr2	44639539	44639539	A	G	intronic	CAMKMT	.	.	.	A	G	+	+	0.6	2,0,3,0	chr2,446395	Cytosol	A > G
chr2:44639568:A:G:intronic:CAMK	chr2	44639568	44639568	A	G	intronic	CAMKMT	.	.	.	A	G	+	+	1	0,0,5,0	chr2,446395	Cytosol	A > G
chr2:54060305:A:G:intronic:ASB3,	chr2	54060305	54060305	A	G	intronic	ASB3,GPR75-	.	.	.	A	G	+	-	0.8	1,0,4,0	chr2,540603	Cytosol	A > G
chr2:55752537:A:G:intronic:CCDC	chr2	55752537	55752537	A	G	intronic	CCDC104	.	.	.	A	G	+	+	1	0,0,5,0	chr2,557525	Cytosol	A > G
chr2:55781286:T:C:intronic:SMEK	chr2	55781286	55781286	T	C	intronic	SMEK2	.	.	.	A	G	-	-	0.6	2,0,3,0	chr2,557812	Cytosol	A > G
chr2:55838938:T:C:intronic:SMEK	chr2	55838938	55838938	T	C	intronic	SMEK2	.	.	.	A	G	-	-	0.8	1,0,4,0	chr2,558389	Cytosol	A > G
chr2:61251106:A:G:intronic:PEX13	chr2	61251106	61251106	A	G	intronic	PEX13	.	.	.	A	G	+	+	0.8	1,0,4,0	chr2,612511	Cytosol	A > G
chr2:61251115:A:G:intronic:PEX13	chr2	61251115	61251115	A	G	intronic	PEX13	.	.	.	A	G	+	+	0.6	2,0,3,0	chr2,612511	Cytosol	A > G
chr2:61251179:A:G:intronic:PEX13	chr2	61251179	61251179	A	G	intronic	PEX13	.	.	.	A	G	+	+	0.25	6,0,2,0	chr2,612511	Cytosol	A > G
chr2:61251183:A:G:intronic:PEX13	chr2	61251183	61251183	A	G	intronic	PEX13	.	.	.	A	G	+	+	0.22222222	7,0,2,0	chr2,612511	Cytosol	A > G
chr2:61251194:A:G:intronic:PEX13	chr2	61251194	61251194	A	G	intronic	PEX13	.	.	.	A	G	+	+	0.625	3,0,5,0	chr2,612511	Cytosol	A > G
chr2:61252343:A:G:intronic:PEX13	chr2	61252343	61252343	A	G	intronic	PEX13	.	.	.	A	G	+	+	0.4	3,0,2,0	chr2,612523	Cytosol	A > G
chr2:61276631:A:G:UTR3:PEX13	chr2	61276631	61276631	A	G	UTR3	PEX13	ENST000002	.	.	A	G	+	+	0.33333333	6,0,3,0	chr2,612766	Cytosol	A > G
chr2:61276787:A:G:UTR3:PEX13	chr2	61276787	61276787	A	G	UTR3	PEX13	ENST000002	.	.	A	G	+	+	0.34375	21,0,11,0	chr2,612767	Cytosol	A > G
chr2:61276788:A:G:UTR3:PEX13	chr2	61276788	61276788	A	G	UTR3	PEX13	ENST000002	.	.	A	G	+	+	0.51724138	14,0,15,0	chr2,612767	Cytosol	A > G
chr2:61277325:A:G:UTR3:PEX13	chr2	61277325	61277325	A	G	UTR3	PEX13	ENST000002	.	.	A	G	+	+	0.1627907	36,0,7,0	chr2,612773	Cytosol	A > G
chr2:61277384:A:G:UTR3:PEX13	chr2	61277384	61277384	A	G	UTR3	PEX13	ENST000002	.	.	A	G	+	+	0.15789474	16,0,3,0	chr2,612773	Cytosol	A > G
chr2:61277390:A:G:UTR3:PEX13	chr2	61277390	61277390	A	G	UTR3	PEX13	ENST000002	.	.	A	G	+	+	0.2	16,0,4,0	chr2,612773	Cytosol	A > G
chr2:61277404:A:G:UTR3:PEX13	chr2	61277404	61277404	A	G	UTR3	PEX13	ENST000002	.	.	A	G	+	+	0.36363636	14,0,8,0	chr2,612774	Cytosol	A > G
chr2:61277443:A:G:UTR3:PEX13	chr2	61277443	61277443	A	G	UTR3	PEX13	ENST000002	.	.	A	G	+	+	0.3255814	29,0,14,0	chr2,612774	Cytosol	A > G
chr2:61277466:A:G:UTR3:PEX13	chr2	61277466	61277466	A	G	UTR3	PEX13	ENST000002	.	.	A	G	+	+	0.06557377	57,0,4,0	chr2,612774	Cytosol	A > G
chr2:61277486:A:G:UTR3:PEX13	chr2	61277486	61277486	A	G	UTR3	PEX13	ENST000002	.	.	A	G	+	+	0.15053763	79,0,14,0	chr2,612774	Cytosol	A > G
chr2:61277775:A:G:UTR3:PEX13	chr2	61277775	61277775	A	G	UTR3	PEX13	ENST000002	.	.	A	G	+	+	0.25	15,0,5,0	chr2,612777	Cytosol	A > G
chr2:61277938:A:G:UTR3:PEX13	chr2	61277938	61277938	A	G	UTR3	PEX13	ENST000002	.	.	A	G	+	+	0.16666667	10,0,2,0	chr2,612779	Cytosol	A > G
chr2:61277998:A:G:UTR3:PEX13	chr2	61277998	61277998	A	G	UTR3	PEX13	ENST000002	.	.	A	G	+	+	0.14285714	24,0,4,0	chr2,612779	Cytosol	A > G
chr2:61278017:A:G:UTR3:PEX13	chr2	61278017	61278017	A	G	UTR3	PEX13	ENST000002	.	.	A	G	+	+	0.25	27,0,9,0	chr2,612780	Cytosol	A > G
chr2:61278018:A:G:UTR3:PEX13	chr2	61278018	61278018	A	G	UTR3	PEX13	ENST000002	.	.	A	G	+	+	0.36842105	24,0,14,0	chr2,612780	Cytosol	A > G
chr2:61278030:A:G:UTR3:PEX13	chr2	61278030	61278030	A	G	UTR3	PEX13	ENST000002	.	.	A	G	+	+	0.10810811	32,1,4,0	chr2,612780	Cytosol	A > G
chr2:61315686:A:G:intronic:KIAA1	chr2	61315686	61315686	A	G	intronic	KIAA1841	.	.	.	A	G	+	+	0.72413793	8,0,21,0	chr2,613156	Cytosol	A > G
chr2:61315734:A:G:intronic:KIAA1	chr2	61315734	61315734	A	G	intronic	KIAA1841	.	.	.	A	G	+	+	0.16666667	25,0,5,0	chr2,613157	Cytosol	A > G
chr2:61315735:A:G:intronic:KIAA1	chr2	61315735	61315735	A	G	intronic	KIAA1841	.	.	.	A	G	+	+	0.26666667	22,0,8,0	chr2,613157	Cytosol	A > G
chr2:61315749:A:G:intronic:KIAA1	chr2	61315749	61315749	A	G	intronic	KIAA1841	.	.	.	A	G	+	+	0.36363636	14,0,8,0	chr2,613157	Cytosol	A > G
chr2:61315795:A:G:intronic:KIAA1	chr2	61315795	61315795	A	G	intronic	KIAA1841	.	.	.	A	G	+	+	0.66666667	4,0,8,0	chr2,613157	Cytosol	A > G
chr2:61315839:A:G:intronic:KIAA1	chr2	61315839	61315839	A	G	intronic	KIAA1841	.	.	.	A	G	+	+	0.8	1,0,4,0	chr2,613158	Cytosol	A > G
chr2:61315919:A:G:intronic:KIAA1	chr2	61315919	61315919	A	G	intronic	KIAA1841	.	.	.	A	G	+	+	0.38095238	13,0,8,0	chr2,613159	Cytosol	A > G
chr2:61315928:A:G:intronic:KIAA1	chr2	61315928	61315928	A	G	intronic	KIAA1841	.	.	.	A	G	+	+	0.28571429	15,0,6,0	chr2,613159	Cytosol	A > G
chr2:61316390:A:G:intronic:KIAA1	chr2	61316390	61316390	A	G	intronic	KIAA1841	.	.	.	A	G	+	+	0.3	7,0,3,0	chr2,613163	Cytosol	A > G
chr2:61316399:A:G:intronic:KIAA1	chr2	61316399	61316399	A	G	intronic	KIAA1841	.	.	.	A	G	+	+	0.25	6,0,2,0	chr2,613163	Cytosol	A > G
chr2:61316417:A:G:intronic:KIAA1	chr2	61316417	61316417	A	G	intronic	KIAA1841	.	.	.	A	G	+	+	0.5	4,0,4,0	chr2,613164	Cytosol	A > G
chr2:61316450:A:G:intronic:KIAA1	chr2	61316450	61316450	A	G	intronic	KIAA1841	.	.	.	A	G	+	+	0.16666667	15,0,3,0	chr2,613164	Cytosol	A > G
chr2:61316456:A:G:intronic:KIAA1	chr2	61316456	61316456	A	G	intronic	KIAA1841	.	.	.	A	G	+	+	0.11764706	15,0,2,0	chr2,613164	Cytosol	A > G
chr2:61316502:A:G:intronic:KIAA1	chr2	61316502	61316502	A	G	intronic	KIAA1841	.	.	.	A	G	+	+	0.06060606	31,0,2,0	chr2,613165	Cytosol	A > G
chr2:61316542:A:G:intronic:KIAA1	chr2	61316542	61316542	A	G	intronic	KIAA1841	.	.	.	A	G	+	+	0.13513514	32,0,5,0	chr2,613165	Cytosol	A > G
chr2:61316562:A:G:intronic:KIAA1	chr2	61316562	61316562	A	G	intronic	KIAA1841	.	.	.	A	G	+	+	0.08	23,0,2,0	chr2,613165	Cytosol	A > G
chr2:61316566:A:G:intronic:KIAA1	chr2	61316566	61316566	A	G	intronic	KIAA1841	.	.	.	A	G	+	+	0.12	22,0,3,0	chr2,613165	Cytosol	A > G
chr2:61316725:A:G:intronic:KIAA1	chr2	61316725	61316725	A	G	intronic	KIAA1841	.	.	.	A	G	+	+	0.3	7,0,3,0	chr2,613167	Cytosol	A > G
chr2:61318137:A:G:intronic:KIAA1	chr2	61318137	61318137	A	G	intronic	KIAA1841	.	.	.	A	G	+	+	0.28571429	5,0,2,0	chr2,613181	Cytosol	A > G
chr2:61318811:A:G:intronic:KIAA1	chr2	61318811	61318811	A	G	intronic	KIAA1841	.	.	.	A	G	+	+	0.57142857	3,0,4,0	chr2,613188	Cytosol	A > G
chr2:61336868:A:G:intronic:KIAA1	chr2	61336868	61336868	A	G	intronic	KIAA1841	.	.	.	A	G	+	+	0.6	2,0,3,0	chr2,613368	Cytosol	A > G
chr2:61338273:A:G:intronic:KIAA1	chr2	61338273	61338273	A	G	intronic	KIAA1841	.	.	.	A	G	+	+	0.42857143	4,0,3,0	chr2,613382	Cytosol	A > G
chr2:61338274:A:G:intronic:KIAA1	chr2	61338274	61338274	A	G	intronic	KIAA1841	.	.	.	A	G	+	+	0.33333333	4,0,2,0	chr2,613382	Cytosol	A > G
chr2:61369025:T:C:ncRNA_exonic:	chr2	61369025	61369025	T	C	ncRNA_exon	AC016747.3	.	.	.	A	G	-	+-	0.46341463	22,0,19,0	chr2,613690	Cytosol	A > G
chr2:61369034:T:C:ncRNA_exonic:	chr2	61369034	61369034	T	C	ncRNA_exon	AC016747.3	.	.	.	A	G	-	+-	0.26315789	28,0,10,0	chr2,613690	Cytosol	A > G
chr2:61369048:T:C:ncRNA_exonic:	chr2	61369048	61369048	T	C	ncRNA_exon	AC016747.3	.	.	.	A	G	-	+-	0.38235294	21,0,13,0	chr2,613690	Cytosol	A > G
chr2:61369085:T:C:ncRNA_exonic:	chr2	61369085	61369085	T	C	ncRNA_exon	AC016747.3	.	.	.	A	G	-	+-	0.04761905	40,0,2,0	chr2,613690	Cytosol	A > G
chr2:61369151:T:C:ncRNA_exonic:	chr2	61369151	61369151	T	C	ncRNA_exon	AC016747.3	.	.	.	A	G	-	+-	0.06976744	40,0,3,0	chr2,613691	Cytosol	A > G
chr2:61369175:T:C:ncRNA_exonic:	chr2	61369175	61369175	T	C	ncRNA_exon	AC016747.3	.	.	.	A	G	-	+-	0.16666667	30,0,6,0	chr2,613691	Cytosol	A > G
chr2:61369237:T:C:ncRNA_exonic:	chr2	61369237	61369237	T	C	ncRNA_exon	AC016747.3	.	.	.	A	G	-	+-	0.34782609	30,0,16,0	chr2,613692	Cytosol	A > G
chr2:61369822:T:C:ncRNA_exonic:	chr2	61369822	61369822	T	C	ncRNA_exon	AC016747.3	.	.	.	A	G	-	+-	0.02469136	79,0,2,0	chr2,613698	Cytosol	A > G
chr2:61369840:T:C:ncRNA_exonic:	chr2	61369840	61369840	T	C	ncRNA_exon	AC016747.3	.	.	.	A	G	-	+-	0.05970149	63,0,4,0	chr2,613698	Cytosol	A > G
chr2:61369929:T:C:ncRNA_exonic:	chr2	61369929	61369929	T	C	ncRNA_exon	AC016747.3	.	.	.	A	G	-	+-	0.10843373	74,0,9,0	chr2,613699	Cytosol	A > G
chr2:61369944:T:C:ncRNA_exonic:	chr2	61369944	61369944	T	C	ncRNA_exon	AC016747.3	.	.	.	A	G	-	+-	0.16666667	65,0,13,0	chr2,613699	Cytosol	A > G

chr2:69547754:T:C:UTR3:GFPT1	chr2	69547754	69547754	T	C	UTR3	GFPT1	ENST000003	.	.	A	G	-	-	0.22222222	14,0,4,0	chr2,695477	Cytosol	A > G
chr2:69547792:T:C:UTR3:GFPT1	chr2	69547792	69547792	T	C	UTR3	GFPT1	ENST000003	.	.	A	G	-	-	0.06060606	31,0,2,0	chr2,695477	Cytosol	A > G
chr2:69688754:T:C:ncRNA_exonic:	chr2	69688754	69688754	T	C	ncRNA_exon	RP11-427H3.	.	.	.	A	G	-	-	0.22727273	17,0,5,0	chr2,696887	Cytosol	A > G
chr2:69688849:T:C:ncRNA_exonic:	chr2	69688849	69688849	T	C	ncRNA_exon	RP11-427H3.	.	.	.	A	G	-	-	0.25	18,0,6,0	chr2,696888	Cytosol	A > G
chr2:69688901:T:C:ncRNA_exonic:	chr2	69688901	69688901	T	C	ncRNA_exon	RP11-427H3.	.	.	.	A	G	-	-	0.75	4,0,12,0	chr2,696889	Cytosol	A > G
chr2:69688902:T:C:ncRNA_exonic:	chr2	69688902	69688902	T	C	ncRNA_exon	RP11-427H3.	.	.	.	A	G	-	-	0.375	10,0,6,0	chr2,696889	Cytosol	A > G
chr2:69688903:T:C:ncRNA_exonic:	chr2	69688903	69688903	T	C	ncRNA_exon	RP11-427H3.	.	.	.	A	G	-	-	0.4375	9,0,7,0	chr2,696889	Cytosol	A > G
chr2:69688906:T:C:ncRNA_exonic:	chr2	69688906	69688906	T	C	ncRNA_exon	RP11-427H3.	.	.	.	A	G	-	-	0.1875	13,0,3,0	chr2,696889	Cytosol	A > G
chr2:69688991:T:C:ncRNA_exonic:	chr2	69688991	69688991	T	C	ncRNA_exon	RP11-427H3.	.	.	.	A	G	-	-	0.375	5,0,3,0	chr2,696889	Cytosol	A > G
chr2:69688997:T:C:ncRNA_exonic:	chr2	69688997	69688997	T	C	ncRNA_exon	RP11-427H3.	.	.	.	A	G	-	-	0.22222222	7,0,2,0	chr2,696889	Cytosol	A > G
chr2:69689648:T:C:ncRNA_exonic:	chr2	69689648	69689648	T	C	ncRNA_exon	RP11-427H3.	.	.	.	A	G	-	-	0.8	1,0,4,0	chr2,696896	Cytosol	A > G
chr2:69689699:T:C:ncRNA_exonic:	chr2	69689699	69689699	T	C	ncRNA_exon	RP11-427H3.	.	.	.	A	G	-	-	0.14285714	12,0,2,0	chr2,696896	Cytosol	A > G
chr2:69689711:T:C:ncRNA_exonic:	chr2	69689711	69689711	T	C	ncRNA_exon	RP11-427H3.	.	.	.	A	G	-	-	0.1875	13,0,3,0	chr2,696897	Cytosol	A > G
chr2:69689725:T:C:ncRNA_exonic:	chr2	69689725	69689725	T	C	ncRNA_exon	RP11-427H3.	.	.	.	A	G	-	-	0.16666667	15,0,3,0	chr2,696897	Cytosol	A > G
chr2:69689806:T:C:ncRNA_exonic:	chr2	69689806	69689806	T	C	ncRNA_exon	RP11-427H3.	.	.	.	A	G	-	-	0.10526316	17,0,2,0	chr2,696898	Cytosol	A > G
chr2:74645427:A:G:intronic:C2orf8	chr2	74645427	74645427	A	G	intronic	C2orf81	.	.	.	T	C	-	-	0.94736842	0,18,0,1	chr2,746454	Cytosol	T > C
chr2:75890776:A:G:UTR3:GCFC2	chr2	75890776	75890776	A	G	UTR3	GCFC2	ENST000005	.	.	A	G	+	+-	0.42857143	4,0,3,0	chr2,758907	Cytosol	A > G
chr2:75894097:A:G:intronic:GCFC	chr2	75894097	75894097	A	G	intronic	GCFC2,MRPL	.	.	.	A	G	+	+-	0.375	5,0,3,0	chr2,758940	Cytosol	A > G
chr2:75894137:A:G:intronic:GCFC	chr2	75894137	75894137	A	G	intronic	GCFC2,MRPL	.	.	.	A	G	+	+-	0.66666667	4,0,8,0	chr2,758941	Cytosol	A > G
chr2:75894144:A:G:intronic:GCFC	chr2	75894144	75894144	A	G	intronic	GCFC2,MRPL	.	.	.	A	G	+	+-	0.91666667	1,0,11,0	chr2,758941	Cytosol	A > G
chr2:75894154:A:G:intronic:GCFC	chr2	75894154	75894154	A	G	intronic	GCFC2,MRPL	.	.	.	A	G	+	+-	0.88888889	1,0,8,0	chr2,758941	Cytosol	A > G
chr2:75894155:A:G:intronic:GCFC	chr2	75894155	75894155	A	G	intronic	GCFC2,MRPL	.	.	.	A	G	+	+-	0.375	5,0,3,0	chr2,758941	Cytosol	A > G
chr2:75894215:A:G:intronic:GCFC	chr2	75894215	75894215	A	G	intronic	GCFC2,MRPL	.	.	.	A	G	+	+-	0.375	5,0,3,0	chr2,758942	Cytosol	A > G
chr2:75894229:A:G:intronic:GCFC	chr2	75894229	75894229	A	G	intronic	GCFC2,MRPL	.	.	.	A	G	+	+-	0.71428571	3,1,10,0	chr2,758942	Cytosol	A > G
chr2:75894237:A:G:intronic:GCFC	chr2	75894237	75894237	A	G	intronic	GCFC2,MRPL	.	.	.	A	G	+	+-	0.73333333	4,0,11,0	chr2,758942	Cytosol	A > G
chr2:75894244:A:G:intronic:GCFC	chr2	75894244	75894244	A	G	intronic	GCFC2,MRPL	.	.	.	A	G	+	+-	0.75	4,0,12,0	chr2,758942	Cytosol	A > G
chr2:75894252:A:G:intronic:GCFC	chr2	75894252	75894252	A	G	intronic	GCFC2,MRPL	.	.	.	A	G	+	+-	0.47058824	9,0,8,0	chr2,758942	Cytosol	A > G
chr2:75894273:A:G:intronic:GCFC	chr2	75894273	75894273	A	G	intronic	GCFC2,MRPL	.	.	.	A	G	+	+-	0.40909091	13,0,9,0	chr2,758942	Cytosol	A > G
chr2:75894331:A:G:intronic:GCFC	chr2	75894331	75894331	A	G	intronic	GCFC2,MRPL	.	.	.	A	G	+	+-	0.61538462	5,0,8,0	chr2,758943	Cytosol	A > G
chr2:75894338:A:G:intronic:GCFC	chr2	75894338	75894338	A	G	intronic	GCFC2,MRPL	.	.	.	A	G	+	+-	0.7	3,0,7,0	chr2,758943	Cytosol	A > G
chr2:75894362:A:G:intronic:GCFC	chr2	75894362	75894362	A	G	intronic	GCFC2,MRPL	.	.	.	A	G	+	+-	0.75	3,0,9,0	chr2,758943	Cytosol	A > G
chr2:75894365:A:G:intronic:GCFC	chr2	75894365	75894365	A	G	intronic	GCFC2,MRPL	.	.	.	A	G	+	+-	0.8	2,0,8,0	chr2,758943	Cytosol	A > G
chr2:75895106:A:G:intronic:GCFC	chr2	75895106	75895106	A	G	intronic	GCFC2,MRPL	.	.	.	A	G	+	+-	0.42857143	4,0,3,0	chr2,758951	Cytosol	A > G
chr2:75895174:A:G:intronic:GCFC	chr2	75895174	75895174	A	G	intronic	GCFC2,MRPL	.	.	.	A	G	+	+-	1	0,0,9,0	chr2,758951	Cytosol	A > G
chr2:75895175:A:G:intronic:GCFC	chr2	75895175	75895175	A	G	intronic	GCFC2,MRPL	.	.	.	A	G	+	+-	0.88888889	1,0,8,0	chr2,758951	Cytosol	A > G
chr2:75895182:A:G:intronic:GCFC	chr2	75895182	75895182	A	G	intronic	GCFC2,MRPL	.	.	.	A	G	+	+-	1	0,0,11,0	chr2,758951	Cytosol	A > G
chr2:75895187:A:G:intronic:GCFC	chr2	75895187	75895187	A	G	intronic	GCFC2,MRPL	.	.	.	A	G	+	+-	0.4	9,0,6,0	chr2,758951	Cytosol	A > G
chr2:75895188:A:G:intronic:GCFC	chr2	75895188	75895188	A	G	intronic	GCFC2,MRPL	.	.	.	A	G	+	+-	0.30769231	9,0,4,0	chr2,758951	Cytosol	A > G
chr2:75895202:A:G:intronic:GCFC	chr2	75895202	75895202	A	G	intronic	GCFC2,MRPL	.	.	.	A	G	+	+-	0.38461538	8,0,5,0	chr2,758952	Cytosol	A > G
chr2:75895214:A:G:intronic:GCFC	chr2	75895214	75895214	A	G	intronic	GCFC2,MRPL	.	.	.	A	G	+	+-	0.69230769	4,0,9,0	chr2,758952	Cytosol	A > G
chr2:75895225:A:G:intronic:GCFC	chr2	75895225	75895225	A	G	intronic	GCFC2,MRPL	.	.	.	A	G	+	+-	0.38461538	8,0,5,0	chr2,758952	Cytosol	A > G
chr2:75895233:A:G:intronic:GCFC	chr2	75895233	75895233	A	G	intronic	GCFC2,MRPL	.	.	.	A	G	+	+-	0.91666667	1,0,11,0	chr2,758952	Cytosol	A > G
chr2:75895234:A:G:intronic:GCFC	chr2	75895234	75895234	A	G	intronic	GCFC2,MRPL	.	.	.	A	G	+	+-	0.44444444	5,0,4,0	chr2,758952	Cytosol	A > G
chr2:75895270:A:G:intronic:GCFC	chr2	75895270	75895270	A	G	intronic	GCFC2,MRPL	.	.	.	A	G	+	+-	0.22222222	14,0,4,0	chr2,758952	Cytosol	A > G
chr2:75895273:A:G:intronic:GCFC	chr2	75895273	75895273	A	G	intronic	GCFC2,MRPL	.	.	.	A	G	+	+-	0.25	12,0,4,0	chr2,758952	Cytosol	A > G
chr2:75895274:A:G:intronic:GCFC	chr2	75895274	75895274	A	G	intronic	GCFC2,MRPL	.	.	.	A	G	+	+-	0.17647059	14,0,3,0	chr2,758952	Cytosol	A > G
chr2:75895275:A:G:intronic:GCFC	chr2	75895275	75895275	A	G	intronic	GCFC2,MRPL	.	.	.	A	G	+	+-	0.52941176	8,0,9,0	chr2,758952	Cytosol	A > G
chr2:75895277:A:G:intronic:GCFC	chr2	75895277	75895277	A	G	intronic	GCFC2,MRPL	.	.	.	A	G	+	+-	0.41176471	10,0,7,0	chr2,758952	Cytosol	A > G
chr2:75895278:A:G:intronic:GCFC	chr2	75895278	75895278	A	G	intronic	GCFC2,MRPL	.	.	.	A	G	+	+-	0.82352941	3,0,14,0	chr2,758952	Cytosol	A > G
chr2:75895292:A:G:intronic:GCFC	chr2	75895292	75895292	A	G	intronic	GCFC2,MRPL	.	.	.	A	G	+	+-	0.17647059	14,0,3,0	chr2,758952	Cytosol	A > G
chr2:75895321:A:G:intronic:GCFC	chr2	75895321	75895321	A	G	intronic	GCFC2,MRPL	.	.	.	A	G	+	+-	0.33333333	6,0,3,0	chr2,758953	Cytosol	A > G
chr2:75895327:A:G:intronic:GCFC	chr2	75895327	75895327	A	G	intronic	GCFC2,MRPL	.	.	.	A	G	+	+-	0.66666667	3,0,6,0	chr2,758953	Cytosol	A > G
chr2:75895328:A:G:intronic:GCFC	chr2	75895328	75895328	A	G	intronic	GCFC2,MRPL	.	.	.	A	G	+	+-	0.375	5,0,3,0	chr2,758953	Cytosol	A > G
chr2:75895329:A:G:intronic:GCFC	chr2	75895329	75895329	A	G	intronic	GCFC2,MRPL	.	.	.	A	G	+	+-	0.375	5,0,3,0	chr2,758953	Cytosol	A > G
chr2:75895340:A:G:intronic:GCFC	chr2	75895340	75895340	A	G	intronic	GCFC2,MRPL	.	.	.	A	G	+	+-	0.5	5,0,5,0	chr2,758953	Cytosol	A > G
chr2:75895352:A:G:intronic:GCFC	chr2	75895352	75895352	A	G	intronic	GCFC2,MRPL	.	.	.	A	G	+	+-	0.3	7,0,3,0	chr2,758953	Cytosol	A > G
chr2:75895395:A:G:intronic:GCFC	chr2	75895395	75895395	A	G	intronic	GCFC2,MRPL	.	.	.	A	G	+	+-	0.17647059	14,0,3,0	chr2,758953	Cytosol	A > G
chr2:75895432:A:G:intronic:GCFC	chr2	75895432	75895432	A	G	intronic	GCFC2,MRPL	.	.	.	A	G	+	+-	0.2	12,0,3,0	chr2,758954	Cytosol	A > G
chr2:75895444:A:G:intronic:GCFC	chr2	75895444	75895444	A	G	intronic	GCFC2,MRPL	.	.	.	A	G	+	+-	0.25	9,0,3,0	chr2,758954	Cytosol	A > G
chr2:85546398:T:C:UTR3:TGOLN2	chr2	85546398	85546398	T	C	UTR3	TGOLN2	ENST000002	.	.	A	G	-	-	0.02439024	80,0,2,0	chr2,855463	Cytosol	A > G
chr2:85546457:T:C:UTR3:TGOLN2	chr2	85546457	85546457	T	C	UTR3	TGOLN2	ENST000002	.	.	A	G	-	-	0.0776699	95,0,8,0	chr2,855464	Cytosol	A > G
chr2:85546465:T:C:UTR3:TGOLN2	chr2	85546465	85546465	T	C	UTR3	TGOLN2	ENST000002	.	.	A	G	-	-	0.07317073	76,0,6,0	chr2,855464	Cytosol	A > G
chr2:85546474:T:C:UTR3:TGOLN2	chr2	85546474	85546474	T	C	UTR3	TGOLN2	ENST000002	.	.	A	G	-	-	0.10891089	90,0,11,0	chr2,855464	Cytosol	A > G
chr2:85546517:T:C:UTR3:TGOLN2	chr2	85546517	85546517	T	C	UTR3	TGOLN2	ENST000002	.	.	A	G	-	-	0.15686275	43,0,8,0	chr2,855465	Cytosol	A > G

chr2:85546571:T:C:UTR3:TGOLN2	chr2	85546571	85546571	T	C	UTR3	TGOLN2	ENST000002	.	.	A	G	-	-	0.04615385	124,0,6,0	chr2,855465	Cytosol	A > G
chr2:85546572:T:C:UTR3:TGOLN2	chr2	85546572	85546572	T	C	UTR3	TGOLN2	ENST000002	.	.	A	G	-	-	0.0530303	125,0,7,0	chr2,855465	Cytosol	A > G
chr2:85773368:T:C:UTR3:GGCX	chr2	85773368	85773368	T	C	UTR3	GGCX	ENST000002	.	.	A	G	-	-	0.28571429	5,0,2,0	chr2,857733	Cytosol	A > G
chr2:85773390:T:C:UTR3:GGCX	chr2	85773390	85773390	T	C	UTR3	GGCX	ENST000002	.	.	A	G	-	-	0.5	4,0,4,0	chr2,857733	Cytosol	A > G
chr2:85773401:T:C:UTR3:GGCX	chr2	85773401	85773401	T	C	UTR3	GGCX	ENST000002	.	.	A	G	-	-	0.5	3,0,3,0	chr2,857734	Cytosol	A > G
chr2:85773708:T:C:UTR3:GGCX	chr2	85773708	85773708	T	C	UTR3	GGCX	ENST000002	.	.	A	G	-	-	0.66666667	2,0,4,0	chr2,857737	Cytosol	A > G
chr2:85773711:T:C:UTR3:GGCX	chr2	85773711	85773711	T	C	UTR3	GGCX	ENST000002	.	.	A	G	-	-	0.66666667	2,0,4,0	chr2,857737	Cytosol	A > G
chr2:85775118:T:C:UTR3:GGCX	chr2	85775118	85775118	T	C	UTR3	GGCX	ENST000002	.	.	A	G	-	-	0.38461538	8,0,5,0	chr2,857751	Cytosol	A > G
chr2:85775136:T:C:UTR3:GGCX	chr2	85775136	85775136	T	C	UTR3	GGCX	ENST000002	.	.	A	G	-	-	0.33333333	10,0,5,0	chr2,857751	Cytosol	A > G
chr2:85776590:T:C:UTR3:GGCX	chr2	85776590	85776590	T	C	UTR3	GGCX	ENST000002	.	.	A	G	-	-	0.05882353	48,0,3,0	chr2,857765	Cytosol	A > G
chr2:85776621:T:C:UTR3:GGCX	chr2	85776621	85776621	T	C	UTR3	GGCX	ENST000002	.	.	A	G	-	-	0.24528302	40,0,13,0	chr2,857766	Cytosol	A > G
chr2:85776677:T:C:UTR3:GGCX	chr2	85776677	85776677	T	C	UTR3	GGCX	ENST000002	.	.	A	G	-	-	0.10144928	62,0,7,0	chr2,857766	Cytosol	A > G
chr2:85776685:T:C:UTR3:GGCX	chr2	85776685	85776685	T	C	UTR3	GGCX	ENST000002	.	.	A	G	-	-	0.07228916	77,0,6,0	chr2,857766	Cytosol	A > G
chr2:85776716:T:C:UTR3:GGCX	chr2	85776716	85776716	T	C	UTR3	GGCX	ENST000002	.	.	A	G	-	-	0.10204082	44,0,5,0	chr2,857767	Cytosol	A > G
chr2:86247543:T:C:UTR3:POLR1A	chr2	86247543	86247543	T	C	UTR3	POLR1A	ENST000002	.	.	A	G	-	-	0.13333333	13,0,2,0	chr2,862475	Cytosol	A > G
chr2:86247549:T:C:UTR3:POLR1A	chr2	86247549	86247549	T	C	UTR3	POLR1A	ENST000002	.	.	A	G	-	-	0.22222222	14,0,4,0	chr2,862475	Cytosol	A > G
chr2:86247558:T:C:UTR3:POLR1A	chr2	86247558	86247558	T	C	UTR3	POLR1A	ENST000002	.	.	A	G	-	-	0.17647059	14,0,3,0	chr2,862475	Cytosol	A > G
chr2:86247559:T:C:UTR3:POLR1A	chr2	86247559	86247559	T	C	UTR3	POLR1A	ENST000002	.	.	A	G	-	-	0.375	10,0,6,0	chr2,862475	Cytosol	A > G
chr2:86247562:T:C:UTR3:POLR1A	chr2	86247562	86247562	T	C	UTR3	POLR1A	ENST000002	.	.	A	G	-	-	0.26315789	14,0,5,0	chr2,862475	Cytosol	A > G
chr2:86247595:T:C:UTR3:POLR1A	chr2	86247595	86247595	T	C	UTR3	POLR1A	ENST000002	.	.	A	G	-	-	0.23809524	16,0,5,0	chr2,862475	Cytosol	A > G
chr2:86247624:T:C:UTR3:POLR1A	chr2	86247624	86247624	T	C	UTR3	POLR1A	ENST000002	.	.	A	G	-	-	0.15789474	16,0,3,0	chr2,862476	Cytosol	A > G
chr2:86249872:T:C:UTR3:POLR1A	chr2	86249872	86249872	T	C	UTR3	POLR1A	ENST000002	.	.	A	G	-	-	0.04255319	45,0,2,0	chr2,862498	Cytosol	A > G
chr2:86249876:T:C:UTR3:POLR1A	chr2	86249876	86249876	T	C	UTR3	POLR1A	ENST000002	.	.	A	G	-	-	0.03571429	54,0,2,0	chr2,862498	Cytosol	A > G
chr2:86249930:T:C:UTR3:POLR1A	chr2	86249930	86249930	T	C	UTR3	POLR1A	ENST000002	.	.	A	G	-	-	0.10714286	50,0,6,0	chr2,862499	Cytosol	A > G
chr2:86249961:T:C:UTR3:POLR1A	chr2	86249961	86249961	T	C	UTR3	POLR1A	ENST000002	.	.	A	G	-	-	0.15555556	38,0,7,0	chr2,862499	Cytosol	A > G
chr2:86249962:T:C:UTR3:POLR1A	chr2	86249962	86249962	T	C	UTR3	POLR1A	ENST000002	.	.	A	G	-	-	0.30232558	30,0,13,0	chr2,862499	Cytosol	A > G
chr2:86250101:T:C:UTR3:POLR1A	chr2	86250101	86250101	T	C	UTR3	POLR1A	ENST000002	.	.	A	G	-	-	0.05555556	51,0,3,0	chr2,862501	Cytosol	A > G
chr2:86250128:T:C:UTR3:POLR1A	chr2	86250128	86250128	T	C	UTR3	POLR1A	ENST000002	.	.	A	G	-	-	0.0625	30,0,2,0	chr2,862501	Cytosol	A > G
chr2:86250947:T:C:UTR3:POLR1A	chr2	86250947	86250947	T	C	UTR3	POLR1A	ENST000002	.	.	A	G	-	-	0.44444444	5,0,4,0	chr2,862509	Cytosol	A > G
chr2:86250967:T:C:UTR3:POLR1A	chr2	86250967	86250967	T	C	UTR3	POLR1A	ENST000002	.	.	A	G	-	-	0.625	3,0,5,0	chr2,862509	Cytosol	A > G
chr2:86250971:T:C:UTR3:POLR1A	chr2	86250971	86250971	T	C	UTR3	POLR1A	ENST000002	.	.	A	G	-	-	0.28571429	5,0,2,0	chr2,862509	Cytosol	A > G
chr2:86251005:T:C:UTR3:POLR1A	chr2	86251005	86251005	T	C	UTR3	POLR1A	ENST000002	.	.	A	G	-	-	0.4	3,0,2,0	chr2,862510	Cytosol	A > G
chr2:86251011:T:C:UTR3:POLR1A	chr2	86251011	86251011	T	C	UTR3	POLR1A	ENST000002	.	.	A	G	-	-	0.83333333	1,0,5,0	chr2,862510	Cytosol	A > G
chr2:86251013:T:C:UTR3:POLR1A	chr2	86251013	86251013	T	C	UTR3	POLR1A	ENST000002	.	.	A	G	-	-	0.8	1,0,4,0	chr2,862510	Cytosol	A > G
chr2:86251041:T:C:UTR3:POLR1A	chr2	86251041	86251041	T	C	UTR3	POLR1A	ENST000002	.	.	A	G	-	-	0.66666667	2,0,4,0	chr2,862510	Cytosol	A > G
chr2:86251048:T:C:UTR3:POLR1A	chr2	86251048	86251048	T	C	UTR3	POLR1A	ENST000002	.	.	A	G	-	-	0.42857143	4,0,3,0	chr2,862510	Cytosol	A > G
chr2:86251069:T:C:UTR3:POLR1A	chr2	86251069	86251069	T	C	UTR3	POLR1A	ENST000002	.	.	A	G	-	-	0.15789474	16,0,3,0	chr2,862510	Cytosol	A > G
chr2:86251099:T:C:UTR3:POLR1A	chr2	86251099	86251099	T	C	UTR3	POLR1A	ENST000002	.	.	A	G	-	-	0.11428571	31,0,4,0	chr2,862510	Cytosol	A > G
chr2:86251145:T:C:UTR3:POLR1A	chr2	86251145	86251145	T	C	UTR3	POLR1A	ENST000002	.	.	A	G	-	-	0.1	27,0,3,0	chr2,862511	Cytosol	A > G
chr2:86251151:T:C:UTR3:POLR1A	chr2	86251151	86251151	T	C	UTR3	POLR1A	ENST000002	.	.	A	G	-	-	0.25	21,0,7,0	chr2,862511	Cytosol	A > G
chr2:95811092:T:C:intergenic:MRI	chr2	95811092	95811092	T	C	intergenic	MRPS5,ZNF5	dist=23347;d	.	.	A	G	-	-	0.57142857	3,0,4,0	chr2,958110	Cytosol	A > G
chr2:95811118:T:C:intergenic:MRI	chr2	95811118	95811118	T	C	intergenic	MRPS5,ZNF5	dist=23373;d	.	.	A	G	-	-	0.55555556	4,0,5,0	chr2,958111	Cytosol	A > G
chr2:95811605:T:C:intergenic:MRI	chr2	95811605	95811605	T	C	intergenic	MRPS5,ZNF5	dist=23860;d	.	.	A	G	-	-	0.88888889	1,0,8,0	chr2,958116	Cytosol	A > G
chr2:95811606:T:C:intergenic:MRI	chr2	95811606	95811606	T	C	intergenic	MRPS5,ZNF5	dist=23861;d	.	.	A	G	-	-	0.54545455	5,0,6,0	chr2,958116	Cytosol	A > G
chr2:95811616:T:C:intergenic:MRI	chr2	95811616	95811616	T	C	intergenic	MRPS5,ZNF5	dist=23871;d	.	.	A	G	-	-	0.4	6,0,4,0	chr2,958116	Cytosol	A > G
chr2:95811840:T:C:intergenic:MRI	chr2	95811840	95811840	T	C	intergenic	MRPS5,ZNF5	dist=24095;d	.	.	A	G	-	-	0.8	1,0,4,0	chr2,958118	Cytosol	A > G
chr2:99812273:A:G:UTR3:C2orf15,	chr2	99812273	99812273	A	G	UTR3	C2orf15,MRI	ENST000005	.	.	A	G	+	+	0.04225352	68,0,3,0	chr2,998122	Cytosol	A > G
chr2:99812289:A:G:UTR3:C2orf15,	chr2	99812289	99812289	A	G	UTR3	C2orf15,MRI	ENST000005	.	.	A	G	+	+	0.14705882	87,0,15,0	chr2,998122	Cytosol	A > G
chr2:99812290:A:G:UTR3:C2orf15,	chr2	99812290	99812290	A	G	UTR3	C2orf15,MRI	ENST000005	.	.	A	G	+	+	0.12149533	94,0,13,0	chr2,998122	Cytosol	A > G
chr2:99812311:A:G:UTR3:C2orf15,	chr2	99812311	99812311	A	G	UTR3	C2orf15,MRI	ENST000005	.	.	A	G	+	+	0.04964539	134,0,7,0	chr2,998123	Cytosol	A > G
chr2:99812336:A:G:UTR3:C2orf15,	chr2	99812336	99812336	A	G	UTR3	C2orf15,MRI	ENST000005	.	.	A	G	+	+	0.76111111	43,0,137,0	chr2,998123	Cytosol	A > G
chr2:99812398:A:G:UTR3:C2orf15,	chr2	99812398	99812398	A	G	UTR3	C2orf15,MRI	ENST000005	.	.	A	G	+	+	0.06289308	149,0,10,0	chr2,998123	Cytosol	A > G
chr2:99812414:A:G:UTR3:C2orf15,	chr2	99812414	99812414	A	G	UTR3	C2orf15,MRI	ENST000005	.	.	A	G	+	+	0.1741573	147,0,31,0	chr2,998124	Cytosol	A > G
chr2:99812416:A:G:UTR3:C2orf15,	chr2	99812416	99812416	A	G	UTR3	C2orf15,MRI	ENST000005	.	.	A	G	+	+	0.01081081	183,0,2,0	chr2,998124	Cytosol	A > G
chr2:99812429:A:G:UTR3:C2orf15,	chr2	99812429	99812429	A	G	UTR3	C2orf15,MRI	ENST000005	.	.	A	G	+	+	0.20207254	154,0,39,0	chr2,998124	Cytosol	A > G
chr2:99812447:A:G:UTR3:C2orf15,	chr2	99812447	99812447	A	G	UTR3	C2orf15,MRI	ENST000005	.	.	A	G	+	+	0.5862069	72,0,102,0	chr2,998124	Cytosol	A > G
chr2:99812450:A:G:UTR3:C2orf15,	chr2	99812450	99812450	A	G	UTR3	C2orf15,MRI	ENST000005	.	.	A	G	+	+	0.22857143	135,0,40,0	chr2,998124	Cytosol	A > G
chr2:99812459:A:G:UTR3:C2orf15,	chr2	99812459	99812459	A	G	UTR3	C2orf15,MRI	ENST000005	.	.	A	G	+	+	0.03333333	174,0,6,0	chr2,998124	Cytosol	A > G
chr2:99812464:A:G:UTR3:C2orf15,	chr2	99812464	99812464	A	G	UTR3	C2orf15,MRI	ENST000005	.	.	A	G	+	+	0.26815642	131,0,48,0	chr2,998124	Cytosol	A > G
chr2:99812465:A:G:UTR3:C2orf15,	chr2	99812465	99812465	A	G	UTR3	C2orf15,MRI	ENST000005	.	.	A	G	+	+	0.26946108	122,0,45,0	chr2,998124	Cytosol	A > G
chr2:99812494:A:G:UTR3:C2orf15,	chr2	99812494	99812494	A	G	UTR3	C2orf15,MRI	ENST000005	.	.	A	G	+	+	0.04564315	230,0,11,0	chr2,998124	Cytosol	A > G
chr2:99812509:A:G:UTR3:C2orf15,	chr2	99812509	99812509	A	G	UTR3	C2orf15,MRI	ENST000005	.	.	A	G	+	+	0.03305785	234,0,8,0	chr2,998125	Cytosol	A > G
chr2:99812787:A:G:UTR3:C2orf15,	chr2	99812787	99812787	A	G	UTR3	C2orf15,MRI	ENST000005	.	.	A	G	+	+	0.02659574	183,0,5,0	chr2,998127	Cytosol	A > G
chr2:99812808:A:G:UTR3:C2orf15,	chr2	99812808	99812808	A	G	UTR3	C2orf15,MRI	ENST000005	.	.	A	G	+	+	0.25380711	147,0,50,0	chr2,998128	Cytosol	A > G

chr2:99812815:A:G:UTR3:C2orf15,	chr2	99812815	99812815	A	G	UTR3	C2orf15,MRP	ENST000005	.	.	A	G	+	+	0.3368984	124,0,63,0	chr2,998128	Cytosol	A > G
chr2:99812817:A:G:UTR3:C2orf15,	chr2	99812817	99812817	A	G	UTR3	C2orf15,MRP	ENST000005	.	.	A	G	+	+	0.26966292	130,0,48,0	chr2,998128	Cytosol	A > G
chr2:99812838:A:G:UTR3:C2orf15,	chr2	99812838	99812838	A	G	UTR3	C2orf15,MRP	ENST000005	.	.	A	G	+	+	0.07096774	144,0,11,0	chr2,998128	Cytosol	A > G
chr2:99812843:A:G:UTR3:C2orf15,	chr2	99812843	99812843	A	G	UTR3	C2orf15,MRP	ENST000005	.	.	A	G	+	+	0.71895425	43,0,110,0	chr2,998128	Cytosol	A > G
chr2:99812857:A:G:UTR3:C2orf15,	chr2	99812857	99812857	A	G	UTR3	C2orf15,MRP	ENST000005	.	.	A	G	+	+	0.48598131	55,0,52,0	chr2,998128	Cytosol	A > G
chr2:99812864:A:G:UTR3:C2orf15,	chr2	99812864	99812864	A	G	UTR3	C2orf15,MRP	ENST000005	.	.	A	G	+	+	0.17699115	93,0,20,0	chr2,998128	Cytosol	A > G
chr2:99812893:A:G:UTR3:C2orf15,	chr2	99812893	99812893	A	G	UTR3	C2orf15,MRP	ENST000005	.	.	A	G	+	+	0.0516129	147,0,8,0	chr2,998128	Cytosol	A > G
chr2:99812897:A:G:UTR3:C2orf15,	chr2	99812897	99812897	A	G	UTR3	C2orf15,MRP	ENST000005	.	.	A	G	+	+	0.76047904	39,1,127,0	chr2,998128	Cytosol	A > G
chr2:99812903:A:G:UTR3:C2orf15,	chr2	99812903	99812903	A	G	UTR3	C2orf15,MRP	ENST000005	.	.	A	G	+	+	0.08045977	160,0,14,0	chr2,998129	Cytosol	A > G
chr2:99812951:A:G:UTR3:C2orf15,	chr2	99812951	99812951	A	G	UTR3	C2orf15,MRP	ENST000005	.	.	A	G	+	+	0.02247191	261,0,6,0	chr2,998129	Cytosol	A > G
chr2:99812978:A:G:UTR3:C2orf15,	chr2	99812978	99812978	A	G	UTR3	C2orf15,MRP	ENST000005	.	.	A	G	+	+	0.2734375	186,0,70,0	chr2,998129	Cytosol	A > G
chr2:99812988:A:G:UTR3:C2orf15,	chr2	99812988	99812988	A	G	UTR3	C2orf15,MRP	ENST000005	.	.	A	G	+	+	0.19502075	194,0,47,0	chr2,998129	Cytosol	A > G
chr2:99813003:A:G:UTR3:C2orf15,	chr2	99813003	99813003	A	G	UTR3	C2orf15,MRP	ENST000005	.	.	A	G	+	+	0.14035088	147,0,24,0	chr2,998130	Cytosol	A > G
chr2:99813015:A:G:UTR3:C2orf15,	chr2	99813015	99813015	A	G	UTR3	C2orf15,MRP	ENST000005	.	.	A	G	+	+	0.09734513	102,0,11,0	chr2,998130	Cytosol	A > G
chr2:99813020:A:G:splicing:MRPL	chr2	99813020	99813020	A	G	splicing	MRPL30	ENST000004	.	.	A	G	+	+	0.20720721	88,0,23,0	chr2,998130	Cytosol	A > G
chr2:99813031:A:G:UTR3:C2orf15,	chr2	99813031	99813031	A	G	UTR3	C2orf15,MRP	ENST000005	.	.	A	G	+	+	0.1147541	54,0,7,0	chr2,998130	Cytosol	A > G
chr2:99813042:A:G:UTR3:C2orf15,	chr2	99813042	99813042	A	G	UTR3	C2orf15,MRP	ENST000005	.	.	A	G	+	+	0.05	38,0,2,0	chr2,998130	Cytosol	A > G
chr2:99814181:A:G:intronic:C2orf	chr2	99814181	99814181	A	G	intronic	C2orf15	.	.	.	A	G	+	+	0.34090909	29,0,15,0	chr2,998141	Cytosol	A > G
chr2:99814184:A:G:intronic:C2orf	chr2	99814184	99814184	A	G	intronic	C2orf15	.	.	.	A	G	+	+	0.11627907	38,0,5,0	chr2,998141	Cytosol	A > G
chr2:99814204:A:G:intronic:C2orf	chr2	99814204	99814204	A	G	intronic	C2orf15	.	.	.	A	G	+	+	0.07692308	36,0,3,0	chr2,998142	Cytosol	A > G
chr2:99814216:A:G:intronic:C2orf	chr2	99814216	99814216	A	G	intronic	C2orf15	.	.	.	A	G	+	+	0.10714286	25,0,3,0	chr2,998142	Cytosol	A > G
chr2:99814221:A:G:intronic:C2orf	chr2	99814221	99814221	A	G	intronic	C2orf15	.	.	.	A	G	+	+	0.08823529	31,0,3,0	chr2,998142	Cytosol	A > G
chr2:99814237:A:G:intronic:C2orf	chr2	99814237	99814237	A	G	intronic	C2orf15	.	.	.	A	G	+	+	0.28947368	27,0,11,0	chr2,998142	Cytosol	A > G
chr2:99814335:A:G:intronic:C2orf	chr2	99814335	99814335	A	G	intronic	C2orf15	.	.	.	A	G	+	+	0.04081633	47,0,2,0	chr2,998143	Cytosol	A > G
chr2:99814342:A:G:intronic:C2orf	chr2	99814342	99814342	A	G	intronic	C2orf15	.	.	.	A	G	+	+	0.05660377	50,0,3,0	chr2,998143	Cytosol	A > G
chr2:99814343:A:G:intronic:C2orf	chr2	99814343	99814343	A	G	intronic	C2orf15	.	.	.	A	G	+	+	0.05882353	48,0,3,0	chr2,998143	Cytosol	A > G
chr2:99814359:A:G:intronic:C2orf	chr2	99814359	99814359	A	G	intronic	C2orf15	.	.	.	A	G	+	+	0.06	47,0,3,0	chr2,998143	Cytosol	A > G
chr2:99814369:A:G:intronic:C2orf	chr2	99814369	99814369	A	G	intronic	C2orf15	.	.	.	A	G	+	+	0.075	37,0,3,0	chr2,998143	Cytosol	A > G
chr2:99814381:A:G:intronic:C2orf	chr2	99814381	99814381	A	G	intronic	C2orf15	.	.	.	A	G	+	+	0.03125	62,0,2,0	chr2,998143	Cytosol	A > G
chr2:99816862:A:G:intronic:C2orf	chr2	99816862	99816862	A	G	intronic	C2orf15	.	.	.	A	G	+	+	0.83333333	1,0,5,0	chr2,998168	Cytosol	A > G
chr2:99816863:A:G:intronic:C2orf	chr2	99816863	99816863	A	G	intronic	C2orf15	.	.	.	A	G	+	+	0.75	2,0,6,0	chr2,998168	Cytosol	A > G
chr2:99816994:A:G:intronic:C2orf	chr2	99816994	99816994	A	G	intronic	C2orf15	.	.	.	A	G	+	+	0.66666667	2,0,4,0	chr2,998169	Cytosol	A > G
chr2:99817007:A:G:intronic:C2orf	chr2	99817007	99817007	A	G	intronic	C2orf15	.	.	.	A	G	+	+	0.5	4,0,4,0	chr2,998170	Cytosol	A > G
chr2:99819322:A:G:intronic:C2orf	chr2	99819322	99819322	A	G	intronic	C2orf15	.	.	.	A	G	+	+	0.28571429	5,0,2,0	chr2,998193	Cytosol	A > G
chr2:99820639:A:G:intronic:C2orf	chr2	99820639	99820639	A	G	intronic	C2orf15	.	.	.	A	G	+	+	0.6	2,0,3,0	chr2,998206	Cytosol	A > G
chr2:99820665:A:G:intronic:C2orf	chr2	99820665	99820665	A	G	intronic	C2orf15	.	.	.	A	G	+	+	0.33333333	4,0,2,0	chr2,998206	Cytosol	A > G
chr2:99820686:A:G:intronic:C2orf	chr2	99820686	99820686	A	G	intronic	C2orf15	.	.	.	A	G	+	+	0.33333333	6,0,3,0	chr2,998206	Cytosol	A > G
chr2:100018089:A:G:intronic:REV1	chr2	100018089	100018089	A	G	intronic	REV1	.	.	.	A	G	+	-	0.15384615	11,0,2,0	chr2,100018	Cytosol	A > G
chr2:100018333:A:G:intronic:REV1	chr2	100018333	100018333	A	G	intronic	REV1	.	.	.	A	G	+	-	0.28571429	5,0,2,0	chr2,100018	Cytosol	A > G
chr2:101887860:T:C:UTR3:RNF149	chr2	101887860	101887860	T	C	UTR3	RNF149	ENST000004	.	.	A	G	-	-	0.15384615	11,0,2,0	chr2,101887	Cytosol	A > G
chr2:101887879:T:C:UTR3:RNF149	chr2	101887879	101887879	T	C	UTR3	RNF149	ENST000004	.	.	A	G	-	-	0.16666667	20,0,4,0	chr2,101887	Cytosol	A > G
chr2:101887982:T:C:UTR3:RNF149	chr2	101887982	101887982	T	C	UTR3	RNF149	ENST000004	.	.	A	G	-	-	0.08823529	62,0,6,0	chr2,101887	Cytosol	A > G
chr2:101887994:T:C:UTR3:RNF149	chr2	101887994	101887994	T	C	UTR3	RNF149	ENST000004	.	.	A	G	-	-	0.06666667	56,0,4,0	chr2,101887	Cytosol	A > G
chr2:101887999:T:C:UTR3:RNF149	chr2	101887999	101887999	T	C	UTR3	RNF149	ENST000004	.	.	A	G	-	-	0.0877193	52,0,5,0	chr2,101887	Cytosol	A > G
chr2:101888008:T:C:UTR3:RNF149	chr2	101888008	101888008	T	C	UTR3	RNF149	ENST000004	.	.	A	G	-	-	0.07017544	53,0,4,0	chr2,101888	Cytosol	A > G
chr2:101888012:T:C:UTR3:RNF149	chr2	101888012	101888012	T	C	UTR3	RNF149	ENST000004	.	.	A	G	-	-	0.0483871	59,0,3,0	chr2,101888	Cytosol	A > G
chr2:101891386:T:C:intronic:RNF1	chr2	101891386	101891386	T	C	intronic	RNF149	.	.	.	A	G	-	-	0.25	15,0,5,0	chr2,101891	Cytosol	A > G
chr2:101891387:T:C:intronic:RNF1	chr2	101891387	101891387	T	C	intronic	RNF149	.	.	.	A	G	-	-	0.26315789	14,0,5,0	chr2,101891	Cytosol	A > G
chr2:101891718:T:C:intronic:RNF1	chr2	101891718	101891718	T	C	intronic	RNF149	.	.	.	A	G	-	-	0.18181818	9,0,2,0	chr2,101891	Cytosol	A > G
chr2:109434615:A:G:intronic:CCD	chr2	109434615	109434615	A	G	intronic	CCDC138	.	.	.	A	G	+	+	0.28571429	5,0,2,0	chr2,109434	Cytosol	A > G
chr2:109434639:A:G:intronic:CCD	chr2	109434639	109434639	A	G	intronic	CCDC138	.	.	.	A	G	+	+	0.4	6,0,4,0	chr2,109434	Cytosol	A > G
chr2:113318378:A:G:intronic:POLR	chr2	113318378	113318378	A	G	intronic	POLR1B	.	.	.	A	G	+	+	0.6	2,0,3,0	chr2,113318	Cytosol	A > G
chr2:113318396:A:G:intronic:POLR	chr2	113318396	113318396	A	G	intronic	POLR1B	.	.	.	A	G	+	+	0.4	3,0,2,0	chr2,113318	Cytosol	A > G
chr2:113318527:A:G:intronic:POLR	chr2	113318527	113318527	A	G	intronic	POLR1B	.	.	.	A	G	+	+	0.4	3,0,2,0	chr2,113318	Cytosol	A > G
chr2:113318535:A:G:intronic:POLR	chr2	113318535	113318535	A	G	intronic	POLR1B	.	.	.	A	G	+	+	0.6	2,0,3,0	chr2,113318	Cytosol	A > G
chr2:113334414:A:G:UTR3:POLR1	chr2	113334414	113334414	A	G	UTR3	POLR1B	ENST000004	.	.	A	G	+	+	0.05555556	51,0,3,0	chr2,113334	Cytosol	A > G
chr2:113962780:A:G:UTR3:PSD4	chr2	113962780	113962780	A	G	UTR3	PSD4	ENST000004	.	.	A	G	+	+	0.3	7,0,3,0	chr2,113962	Cytosol	A > G
chr2:113962819:A:G:UTR3:PSD4	chr2	113962819	113962819	A	G	UTR3	PSD4	ENST000004	.	.	A	G	+	+	0.30769231	9,0,4,0	chr2,113962	Cytosol	A > G
chr2:114224494:A:G:intronic:CBW	chr2	114224494	114224494	A	G	intronic	CBWD2	.	.	.	A	G	+	+	1	0,0,7,0	chr2,114224	Cytosol	A > G
chr2:114226765:A:G:intronic:CBW	chr2	114226765	114226765	A	G	intronic	CBWD2	.	.	.	A	G	+	+	1	0,0,6,0	chr2,114226	Cytosol	A > G
chr2:114226849:A:G:intronic:CBW	chr2	114226849	114226849	A	G	intronic	CBWD2	.	.	.	A	G	+	+	0.4	3,0,2,0	chr2,114226	Cytosol	A > G
chr2:114465138:T:C:intergenic:AC	chr2	114465138	114465138	T	C	intergenic	AC017074.2, dist=3483;dis		.	.	A	G	-	-	0.03896104	74,0,3,0	chr2,114465	Cytosol	A > G
chr2:114465166:T:C:intergenic:AC	chr2	114465166	114465166	T	C	intergenic	AC017074.2, dist=3511;dis		.	.	A	G	-	-	0.08	46,0,4,0	chr2,114465	Cytosol	A > G
chr2:114465172:T:C:intergenic:AC	chr2	114465172	114465172	T	C	intergenic	AC017074.2, dist=3517;dis		.	.	A	G	-	-	0.23255814	33,0,10,0	chr2,114465	Cytosol	A > G

chr2:114465197:T:C:intergenic:AC	chr2	114465197	114465197	T	C	intergenic	AC017074.2, dist=3542;dis		.	.	A	G	-	-	0.12195122	36,0,5,0	chr2,114465	Cytosol	A > G
chr2:114465199:T:C:intergenic:AC	chr2	114465199	114465199	T	C	intergenic	AC017074.2, dist=3544;dis		.	.	A	G	-	-	0.19047619	34,0,8,0	chr2,114465	Cytosol	A > G
chr2:114465204:T:C:intergenic:AC	chr2	114465204	114465204	T	C	intergenic	AC017074.2, dist=3549;dis		.	.	A	G	-	-	0.05555556	34,0,2,0	chr2,114465	Cytosol	A > G
chr2:114465209:T:C:intergenic:AC	chr2	114465209	114465209	T	C	intergenic	AC017074.2, dist=3554;dis		.	.	A	G	-	-	0.61290323	12,0,19,0	chr2,114465	Cytosol	A > G
chr2:114465228:T:C:intergenic:AC	chr2	114465228	114465228	T	C	intergenic	AC017074.2, dist=3573;dis		.	.	A	G	-	-	0.84375	5,0,27,0	chr2,114465	Cytosol	A > G
chr2:114465233:T:C:intergenic:AC	chr2	114465233	114465233	T	C	intergenic	AC017074.2, dist=3578;dis		.	.	A	G	-	-	0.25925926	20,0,7,0	chr2,114465	Cytosol	A > G
chr2:114465234:T:C:intergenic:AC	chr2	114465234	114465234	T	C	intergenic	AC017074.2, dist=3579;dis		.	.	A	G	-	-	0.36666667	19,0,11,0	chr2,114465	Cytosol	A > G
chr2:114465242:T:C:intergenic:AC	chr2	114465242	114465242	T	C	intergenic	AC017074.2, dist=3587;dis		.	.	A	G	-	-	0.08	23,0,2,0	chr2,114465	Cytosol	A > G
chr2:114465248:T:C:intergenic:AC	chr2	114465248	114465248	T	C	intergenic	AC017074.2, dist=3593;dis		.	.	A	G	-	-	0.17391304	19,0,4,0	chr2,114465	Cytosol	A > G
chr2:114465252:T:C:intergenic:AC	chr2	114465252	114465252	T	C	intergenic	AC017074.2, dist=3597;dis		.	.	A	G	-	-	0.06451613	29,0,2,0	chr2,114465	Cytosol	A > G
chr2:114465265:T:C:intergenic:AC	chr2	114465265	114465265	T	C	intergenic	AC017074.2, dist=3610;dis		.	.	A	G	-	-	0.13793103	25,0,4,0	chr2,114465	Cytosol	A > G
chr2:114465269:T:C:intergenic:AC	chr2	114465269	114465269	T	C	intergenic	AC017074.2, dist=3614;dis		.	.	A	G	-	-	0.2	20,0,5,0	chr2,114465	Cytosol	A > G
chr2:114465297:T:C:intergenic:AC	chr2	114465297	114465297	T	C	intergenic	AC017074.2, dist=3642;dis		.	.	A	G	-	-	0.15789474	16,0,3,0	chr2,114465	Cytosol	A > G
chr2:114465301:T:C:intergenic:AC	chr2	114465301	114465301	T	C	intergenic	AC017074.2, dist=3646;dis		.	.	A	G	-	-	0.61904762	8,0,13,0	chr2,114465	Cytosol	A > G
chr2:114465305:T:C:intergenic:AC	chr2	114465305	114465305	T	C	intergenic	AC017074.2, dist=3650;dis		.	.	A	G	-	-	0.07407407	25,0,2,0	chr2,114465	Cytosol	A > G
chr2:114465306:T:C:intergenic:AC	chr2	114465306	114465306	T	C	intergenic	AC017074.2, dist=3651;dis		.	.	A	G	-	-	0.07407407	25,0,2,0	chr2,114465	Cytosol	A > G
chr2:114465308:T:C:intergenic:AC	chr2	114465308	114465308	T	C	intergenic	AC017074.2, dist=3653;dis		.	.	A	G	-	-	0.15384615	22,0,4,0	chr2,114465	Cytosol	A > G
chr2:114465316:T:C:intergenic:AC	chr2	114465316	114465316	T	C	intergenic	AC017074.2, dist=3661;dis		.	.	A	G	-	-	0.30434783	16,0,7,0	chr2,114465	Cytosol	A > G
chr2:114465317:T:C:intergenic:AC	chr2	114465317	114465317	T	C	intergenic	AC017074.2, dist=3662;dis		.	.	A	G	-	-	0.21052632	15,0,4,0	chr2,114465	Cytosol	A > G
chr2:114465341:T:C:intergenic:AC	chr2	114465341	114465341	T	C	intergenic	AC017074.2, dist=3686;dis		.	.	A	G	-	-	0.14285714	18,0,3,0	chr2,114465	Cytosol	A > G
chr2:114465345:T:C:intergenic:AC	chr2	114465345	114465345	T	C	intergenic	AC017074.2, dist=3690;dis		.	.	A	G	-	-	0.13043478	20,0,3,0	chr2,114465	Cytosol	A > G
chr2:114465355:T:C:intergenic:AC	chr2	114465355	114465355	T	C	intergenic	AC017074.2, dist=3700;dis		.	.	A	G	-	-	0.84	4,0,21,0	chr2,114465	Cytosol	A > G
chr2:114465356:T:C:intergenic:AC	chr2	114465356	114465356	T	C	intergenic	AC017074.2, dist=3701;dis		.	.	A	G	-	-	0.20833333	19,0,5,0	chr2,114465	Cytosol	A > G
chr2:114465370:T:C:intergenic:AC	chr2	114465370	114465370	T	C	intergenic	AC017074.2, dist=3715;dis		.	.	A	G	-	-	0.33333333	16,0,8,0	chr2,114465	Cytosol	A > G
chr2:114465410:T:C:intergenic:AC	chr2	114465410	114465410	T	C	intergenic	AC017074.2, dist=3755;dis		.	.	A	G	-	-	0.22222222	21,0,6,0	chr2,114465	Cytosol	A > G
chr2:114466064:T:C:intergenic:AC	chr2	114466064	114466064	T	C	intergenic	AC017074.2, dist=4409;dis		.	.	A	G	-	-	0.71428571	4,0,10,0	chr2,114466	Cytosol	A > G
chr2:114466090:T:C:intergenic:AC	chr2	114466090	114466090	T	C	intergenic	AC017074.2, dist=4435;dis		.	.	A	G	-	-	0.32	17,0,8,0	chr2,114466	Cytosol	A > G
chr2:114466099:T:C:intergenic:AC	chr2	114466099	114466099	T	C	intergenic	AC017074.2, dist=4444;dis		.	.	A	G	-	-	0.14285714	30,0,5,0	chr2,114466	Cytosol	A > G
chr2:114466121:T:C:intergenic:AC	chr2	114466121	114466121	T	C	intergenic	AC017074.2, dist=4466;dis		.	.	A	G	-	-	0.33333333	28,0,14,0	chr2,114466	Cytosol	A > G
chr2:114466125:T:C:intergenic:AC	chr2	114466125	114466125	T	C	intergenic	AC017074.2, dist=4470;dis		.	.	A	G	-	-	0.13043478	40,0,6,0	chr2,114466	Cytosol	A > G
chr2:114467441:T:C:intergenic:AC	chr2	114467441	114467441	T	C	intergenic	AC017074.2, dist=5786;dis		.	.	A	G	-	-	0.02222222	88,0,2,0	chr2,114467	Cytosol	A > G
chr2:114467527:T:C:intergenic:AC	chr2	114467527	114467527	T	C	intergenic	AC017074.2, dist=5872;dis		.	.	A	G	-	-	0.15789474	80,0,15,0	chr2,114467	Cytosol	A > G
chr2:114467533:T:C:intergenic:AC	chr2	114467533	114467533	T	C	intergenic	AC017074.2, dist=5878;dis		.	.	A	G	-	-	0.44444444	50,0,40,0	chr2,114467	Cytosol	A > G
chr2:114467556:T:C:intergenic:AC	chr2	114467556	114467556	T	C	intergenic	AC017074.2, dist=5901;dis		.	.	A	G	-	-	0.23863636	67,0,21,0	chr2,114467	Cytosol	A > G
chr2:114467557:T:C:intergenic:AC	chr2	114467557	114467557	T	C	intergenic	AC017074.2, dist=5902;dis		.	.	A	G	-	-	0.10638298	84,0,10,0	chr2,114467	Cytosol	A > G
chr2:114467630:T:C:intergenic:AC	chr2	114467630	114467630	T	C	intergenic	AC017074.2, dist=5975;dis		.	.	A	G	-	-	0.34090909	29,0,15,0	chr2,114467	Cytosol	A > G
chr2:114468062:T:C:intergenic:AC	chr2	114468062	114468062	T	C	intergenic	AC017074.2, dist=6407;dis		.	.	A	G	-	-	0.55555556	16,0,20,0	chr2,114468	Cytosol	A > G
chr2:114468063:T:C:intergenic:AC	chr2	114468063	114468063	T	C	intergenic	AC017074.2, dist=6408;dis		.	.	A	G	-	-	0.33333333	26,0,13,0	chr2,114468	Cytosol	A > G
chr2:114468124:T:C:intergenic:AC	chr2	114468124	114468124	T	C	intergenic	AC017074.2, dist=6469;dis		.	.	A	G	-	-	0.375	25,0,15,0	chr2,114468	Cytosol	A > G
chr2:114468205:T:C:intergenic:AC	chr2	114468205	114468205	T	C	intergenic	AC017074.2, dist=6550;dis		.	.	A	G	-	-	0.19047619	34,0,8,0	chr2,114468	Cytosol	A > G
chr2:114468231:T:C:intergenic:AC	chr2	114468231	114468231	T	C	intergenic	AC017074.2, dist=6576;dis		.	.	A	G	-	-	0.25	39,0,13,0	chr2,114468	Cytosol	A > G
chr2:114468258:T:C:intergenic:AC	chr2	114468258	114468258	T	C	intergenic	AC017074.2, dist=6603;dis		.	.	A	G	-	-	0.275	29,0,11,0	chr2,114468	Cytosol	A > G
chr2:114470088:T:C:downstream:	chr2	114470088	114470088	T	C	downstream	SLC35F5	.	.	.	A	G	-	-	0.16666667	20,0,4,0	chr2,114470	Cytosol	A > G
chr2:114470093:T:C:downstream:	chr2	114470093	114470093	T	C	downstream	SLC35F5	.	.	.	A	G	-	-	0.54166667	11,0,13,0	chr2,114470	Cytosol	A > G
chr2:114470165:T:C:downstream:	chr2	114470165	114470165	T	C	downstream	SLC35F5	.	.	.	A	G	-	-	0.6	8,0,12,0	chr2,114470	Cytosol	A > G
chr2:122483093:T:C:ncRNA_intror	chr2	122483093	122483093	T	C	ncRNA_intro	AC018737.1	.	.	.	A	G	-	+	0.4	3,0,2,0	chr2,122483	Cytosol	A > G
chr2:122483138:T:C:ncRNA_intror	chr2	122483138	122483138	T	C	ncRNA_intro	AC018737.1	.	.	.	A	G	-	+	0.23529412	13,0,4,0	chr2,122483	Cytosol	A > G
chr2:122483140:T:C:ncRNA_intror	chr2	122483140	122483140	T	C	ncRNA_intro	AC018737.1	.	.	.	A	G	-	+	0.15789474	16,0,3,0	chr2,122483	Cytosol	A > G
chr2:122483231:T:C:ncRNA_intror	chr2	122483231	122483231	T	C	ncRNA_intro	AC018737.1	.	.	.	A	G	-	+	0.4375	9,0,7,0	chr2,122483	Cytosol	A > G
chr2:122483240:T:C:ncRNA_intror	chr2	122483240	122483240	T	C	ncRNA_intro	AC018737.1	.	.	.	A	G	-	+	0.14285714	12,0,2,0	chr2,122483	Cytosol	A > G
chr2:122483250:T:C:ncRNA_intror	chr2	122483250	122483250	T	C	ncRNA_intro	AC018737.1	.	.	.	A	G	-	+	0.53333333	7,0,8,0	chr2,122483	Cytosol	A > G
chr2:122483813:T:C:ncRNA_intror	chr2	122483813	122483813	T	C	ncRNA_intro	AC018737.1	.	.	.	A	G	-	+	0.44444444	5,0,4,0	chr2,122483	Cytosol	A > G
chr2:128601420:T:C:ncRNA_exoni	chr2	128601420	128601420	T	C	ncRNA_exon	RP5-935K16.	.	.	.	A	G	-	-	0.02352941	83,0,2,0	chr2,128601	Cytosol	A > G
chr2:128601424:T:C:ncRNA_exoni	chr2	128601424	128601424	T	C	ncRNA_exon	RP5-935K16.	.	.	.	A	G	-	-	0.03703704	78,0,3,0	chr2,128601	Cytosol	A > G
chr2:128601426:T:C:ncRNA_exoni	chr2	128601426	128601426	T	C	ncRNA_exon	RP5-935K16.	.	.	.	A	G	-	-	0.1025641	70,0,8,0	chr2,128601	Cytosol	A > G
chr2:128601438:T:C:ncRNA_exoni	chr2	128601438	128601438	T	C	ncRNA_exon	RP5-935K16.	.	.	.	A	G	-	-	0.04761905	100,0,5,0	chr2,128601	Cytosol	A > G
chr2:128601461:T:C:ncRNA_exoni	chr2	128601461	128601461	T	C	ncRNA_exon	RP5-935K16.	.	.	.	A	G	-	-	0.05681818	83,0,5,0	chr2,128601	Cytosol	A > G
chr2:128601464:T:C:ncRNA_exoni	chr2	128601464	128601464	T	C	ncRNA_exon	RP5-935K16.	.	.	.	A	G	-	-	0.70408163	29,0,69,0	chr2,128601	Cytosol	A > G
chr2:128601556:T:C:ncRNA_exoni	chr2	128601556	128601556	T	C	ncRNA_exon	RP5-935K16.	.	.	.	A	G	-	-	0.07142857	78,0,6,0	chr2,128601	Cytosol	A > G
chr2:128601578:T:C:ncRNA_exoni	chr2	128601578	128601578	T	C	ncRNA_exon	RP5-935K16.	.	.	.	A	G	-	-	0.03947368	73,0,3,0	chr2,128601	Cytosol	A > G
chr2:128602290:T:C:ncRNA_exoni	chr2	128602290	128602290	T	C	ncRNA_exon	RP5-935K16.	.	.	.	A	G	-	-	0.04651163	41,0,2,0	chr2,128602	Cytosol	A > G
chr2:128602339:T:C:ncRNA_exoni	chr2	128602339	128602339	T	C	ncRNA_exon	RP5-935K16.	.	.	.	A	G	-	-	0.1	27,0,3,0	chr2,128602	Cytosol	A > G
chr2:128602348:T:C:ncRNA_exoni	chr2	128602348	128602348	T	C	ncRNA_exon	RP5-935K16.	.	.	.	A	G	-	-	0.10810811	33,0,4,0	chr2,128602	Cytosol	A > G
chr2:128602365:T:C:ncRNA_exoni	chr2	128602365	128602365	T	C	ncRNA_exon	RP5-935K16.	.	.	.	A	G	-	-	0.15625	27,0,5,0	chr2,128602	Cytosol	A > G

chr2:128602366:T:C:ncRNA_exoni	chr2	128602366	128602366	T	C	ncRNA_exon	RP5-935K16.	.	.	.	A	G	-	-	0.44444444	20,0,16,0	chr2,128602	Cytosol	A > G
chr2:128602431:T:C:ncRNA_exoni	chr2	128602431	128602431	T	C	ncRNA_exon	RP5-935K16.	.	.	.	A	G	-	-	0.16666667	20,0,4,0	chr2,128602	Cytosol	A > G
chr2:128602441:T:C:ncRNA_exoni	chr2	128602441	128602441	T	C	ncRNA_exon	RP5-935K16.	.	.	.	A	G	-	-	0.53571429	13,0,15,0	chr2,128602	Cytosol	A > G
chr2:128602452:T:C:ncRNA_exoni	chr2	128602452	128602452	T	C	ncRNA_exon	RP5-935K16.	.	.	.	A	G	-	-	0.19047619	17,0,4,0	chr2,128602	Cytosol	A > G
chr2:128602455:T:C:ncRNA_exoni	chr2	128602455	128602455	T	C	ncRNA_exon	RP5-935K16.	.	.	.	A	G	-	-	0.26086957	17,0,6,0	chr2,128602	Cytosol	A > G
chr2:128602462:T:C:ncRNA_exoni	chr2	128602462	128602462	T	C	ncRNA_exon	RP5-935K16.	.	.	.	A	G	-	-	0.28	18,0,7,0	chr2,128602	Cytosol	A > G
chr2:128602465:T:C:ncRNA_exoni	chr2	128602465	128602465	T	C	ncRNA_exon	RP5-935K16.	.	.	.	A	G	-	-	0.08333333	22,0,2,0	chr2,128602	Cytosol	A > G
chr2:128602500:T:C:ncRNA_exoni	chr2	128602500	128602500	T	C	ncRNA_exon	RP5-935K16.	.	.	.	A	G	-	-	0.21052632	30,0,8,0	chr2,128602	Cytosol	A > G
chr2:128602501:T:C:ncRNA_exoni	chr2	128602501	128602501	T	C	ncRNA_exon	RP5-935K16.	.	.	.	A	G	-	-	0.08108108	34,0,3,0	chr2,128602	Cytosol	A > G
chr2:128602992:T:C:ncRNA_exoni	chr2	128602992	128602992	T	C	ncRNA_exon	RP5-935K16.	.	.	.	A	G	-	-	0.04444444	43,0,2,0	chr2,128602	Cytosol	A > G
chr2:128603035:T:C:ncRNA_exoni	chr2	128603035	128603035	T	C	ncRNA_exon	RP5-935K16.	.	.	.	A	G	-	-	0.07246377	64,0,5,0	chr2,128603	Cytosol	A > G
chr2:128603046:T:C:ncRNA_exoni	chr2	128603046	128603046	T	C	ncRNA_exon	RP5-935K16.	.	.	.	A	G	-	-	0.08955224	61,0,6,0	chr2,128603	Cytosol	A > G
chr2:128603047:T:C:ncRNA_exoni	chr2	128603047	128603047	T	C	ncRNA_exon	RP5-935K16.	.	.	.	A	G	-	-	0.03174603	61,0,2,0	chr2,128603	Cytosol	A > G
chr2:128603078:T:C:ncRNA_exoni	chr2	128603078	128603078	T	C	ncRNA_exon	RP5-935K16.	.	.	.	A	G	-	-	0.1	27,0,3,0	chr2,128603	Cytosol	A > G
chr2:128603087:T:C:ncRNA_exoni	chr2	128603087	128603087	T	C	ncRNA_exon	RP5-935K16.	.	.	.	A	G	-	-	0.15625	27,0,5,0	chr2,128603	Cytosol	A > G
chr2:128603088:T:C:ncRNA_exoni	chr2	128603088	128603088	T	C	ncRNA_exon	RP5-935K16.	.	.	.	A	G	-	-	0.09677419	28,0,3,0	chr2,128603	Cytosol	A > G
chr2:128603119:T:C:ncRNA_exoni	chr2	128603119	128603119	T	C	ncRNA_exon	RP5-935K16.	.	.	.	A	G	-	-	0.25	15,0,5,0	chr2,128603	Cytosol	A > G
chr2:128603131:T:C:ncRNA_exoni	chr2	128603131	128603131	T	C	ncRNA_exon	RP5-935K16.	.	.	.	A	G	-	-	0.57894737	8,0,11,0	chr2,128603	Cytosol	A > G
chr2:128603150:T:C:ncRNA_exoni	chr2	128603150	128603150	T	C	ncRNA_exon	RP5-935K16.	.	.	.	A	G	-	-	0.71428571	2,0,5,0	chr2,128603	Cytosol	A > G
chr2:128603152:T:C:ncRNA_exoni	chr2	128603152	128603152	T	C	ncRNA_exon	RP5-935K16.	.	.	.	A	G	-	-	0.375	5,0,3,0	chr2,128603	Cytosol	A > G
chr2:128603205:T:C:ncRNA_exoni	chr2	128603205	128603205	T	C	ncRNA_exon	RP5-935K16.	.	.	.	A	G	-	-	0.05172414	55,0,3,0	chr2,128603	Cytosol	A > G
chr2:128603207:T:C:ncRNA_exoni	chr2	128603207	128603207	T	C	ncRNA_exon	RP5-935K16.	.	.	.	A	G	-	-	0.04918033	58,0,3,0	chr2,128603	Cytosol	A > G
chr2:128603654:T:C:upstream;do	chr2	128603654	128603654	T	C	upstream;do	RP5-935K16.	.	.	.	A	G	-		0.64102564	14,0,25,0	chr2,128603	Cytosol	A > G
chr2:128603660:T:C:upstream;do	chr2	128603660	128603660	T	C	upstream;do	RP5-935K16.	.	.	.	A	G	-		0.14893617	40,0,7,0	chr2,128603	Cytosol	A > G
chr2:128603705:T:C:upstream;do	chr2	128603705	128603705	T	C	upstream;do	RP5-935K16.	.	.	.	A	G	-		0.60377358	21,0,32,0	chr2,128603	Cytosol	A > G
chr2:128603715:T:C:upstream;do	chr2	128603715	128603715	T	C	upstream;do	RP5-935K16.	.	.	.	A	G	-		0.23529412	39,0,12,0	chr2,128603	Cytosol	A > G
chr2:128603744:T:C:upstream;do	chr2	128603744	128603744	T	C	upstream;do	RP5-935K16.	.	.	.	A	G	-		0.48979592	25,0,24,0	chr2,128603	Cytosol	A > G
chr2:128603745:T:C:upstream;do	chr2	128603745	128603745	T	C	upstream;do	RP5-935K16.	.	.	.	A	G	-		0.35849057	34,0,19,0	chr2,128603	Cytosol	A > G
chr2:128603770:T:C:upstream;do	chr2	128603770	128603770	T	C	upstream;do	RP5-935K16.	.	.	.	A	G	-		0.27906977	31,0,12,0	chr2,128603	Cytosol	A > G
chr2:128949178:A:G:UTR3:UGGT1	chr2	128949178	128949178	A	G	UTR3	UGGT1	ENST000003	.	.	A	G	+	+	0.11724138	128,0,17,0	chr2,128949	Cytosol	A > G
chr2:128949284:A:G:UTR3:UGGT1	chr2	128949284	128949284	A	G	UTR3	UGGT1	ENST000003	.	.	A	G	+	+	0.05128205	111,0,6,0	chr2,128949	Cytosol	A > G
chr2:128949355:A:G:UTR3:UGGT1	chr2	128949355	128949355	A	G	UTR3	UGGT1	ENST000003	.	.	A	G	+	+	0.18333333	49,0,11,0	chr2,128949	Cytosol	A > G
chr2:128950475:A:G:UTR3:UGGT1	chr2	128950475	128950475	A	G	UTR3	UGGT1	ENST000003	.	.	A	G	+	+	0.1625	67,0,13,0	chr2,128950	Cytosol	A > G
chr2:128950481:A:G:UTR3:UGGT1	chr2	128950481	128950481	A	G	UTR3	UGGT1	ENST000003	.	.	A	G	+	+	0.21428571	55,0,15,0	chr2,128950	Cytosol	A > G
chr2:128950489:A:G:UTR3:UGGT1	chr2	128950489	128950489	A	G	UTR3	UGGT1	ENST000003	.	.	A	G	+	+	0.04411765	65,0,3,0	chr2,128950	Cytosol	A > G
chr2:128950491:A:G:UTR3:UGGT1	chr2	128950491	128950491	A	G	UTR3	UGGT1	ENST000003	.	.	A	G	+	+	0.70769231	19,0,46,0	chr2,128950	Cytosol	A > G
chr2:128950497:A:G:UTR3:UGGT1	chr2	128950497	128950497	A	G	UTR3	UGGT1	ENST000003	.	.	A	G	+	+	0.93548387	4,0,58,0	chr2,128950	Cytosol	A > G
chr2:128950498:A:G:UTR3:UGGT1	chr2	128950498	128950498	A	G	UTR3	UGGT1	ENST000003	.	.	A	G	+	+	0.71186441	17,0,42,0	chr2,128950	Cytosol	A > G
chr2:128950515:A:G:UTR3:UGGT1	chr2	128950515	128950515	A	G	UTR3	UGGT1	ENST000003	.	.	A	G	+	+	0.38888889	33,0,21,0	chr2,128950	Cytosol	A > G
chr2:128950542:A:G:UTR3:UGGT1	chr2	128950542	128950542	A	G	UTR3	UGGT1	ENST000003	.	.	A	G	+	+	0.85294118	5,0,29,0	chr2,128950	Cytosol	A > G
chr2:128950548:A:G:UTR3:UGGT1	chr2	128950548	128950548	A	G	UTR3	UGGT1	ENST000003	.	.	A	G	+	+	0.75	7,0,21,0	chr2,128950	Cytosol	A > G
chr2:128950595:A:G:UTR3:UGGT1	chr2	128950595	128950595	A	G	UTR3	UGGT1	ENST000003	.	.	A	G	+	+	0.77777778	2,0,7,0	chr2,128950	Cytosol	A > G
chr2:128950598:A:G:UTR3:UGGT1	chr2	128950598	128950598	A	G	UTR3	UGGT1	ENST000003	.	.	A	G	+	+	0.44444444	5,0,4,0	chr2,128950	Cytosol	A > G
chr2:128950604:A:G:UTR3:UGGT1	chr2	128950604	128950604	A	G	UTR3	UGGT1	ENST000003	.	.	A	G	+	+	1	0,0,7,0	chr2,128950	Cytosol	A > G
chr2:128950630:A:G:UTR3:UGGT1	chr2	128950630	128950630	A	G	UTR3	UGGT1	ENST000003	.	.	A	G	+	+	1	0,0,12,0	chr2,128950	Cytosol	A > G
chr2:128950636:A:G:UTR3:UGGT1	chr2	128950636	128950636	A	G	UTR3	UGGT1	ENST000003	.	.	A	G	+	+	0.5	6,0,6,0	chr2,128950	Cytosol	A > G
chr2:128950857:A:G:UTR3:UGGT1	chr2	128950857	128950857	A	G	UTR3	UGGT1	ENST000003	.	.	A	G	+	+	0.16216216	31,0,6,0	chr2,128950	Cytosol	A > G
chr2:128950890:A:G:UTR3:UGGT1	chr2	128950890	128950890	A	G	UTR3	UGGT1	ENST000003	.	.	A	G	+	+	0.22222222	28,0,8,0	chr2,128950	Cytosol	A > G
chr2:128950919:A:G:UTR3:UGGT1	chr2	128950919	128950919	A	G	UTR3	UGGT1	ENST000003	.	.	A	G	+	+	0.44	14,0,11,0	chr2,128950	Cytosol	A > G
chr2:128950920:A:G:UTR3:UGGT1	chr2	128950920	128950920	A	G	UTR3	UGGT1	ENST000003	.	.	A	G	+	+	0.68	8,0,17,0	chr2,128950	Cytosol	A > G
chr2:128950925:A:G:UTR3:UGGT1	chr2	128950925	128950925	A	G	UTR3	UGGT1	ENST000003	.	.	A	G	+	+	0.10344828	26,0,3,0	chr2,128950	Cytosol	A > G
chr2:128950926:A:G:UTR3:UGGT1	chr2	128950926	128950926	A	G	UTR3	UGGT1	ENST000003	.	.	A	G	+	+	0.15384615	22,0,4,0	chr2,128950	Cytosol	A > G
chr2:128950955:A:G:UTR3:UGGT1	chr2	128950955	128950955	A	G	UTR3	UGGT1	ENST000003	.	.	A	G	+	+	0.95918367	2,0,47,0	chr2,128950	Cytosol	A > G
chr2:128950965:A:G:UTR3:UGGT1	chr2	128950965	128950965	A	G	UTR3	UGGT1	ENST000003	.	.	A	G	+	+	0.79166667	10,0,38,0	chr2,128950	Cytosol	A > G
chr2:128950967:A:G:UTR3:UGGT1	chr2	128950967	128950967	A	G	UTR3	UGGT1	ENST000003	.	.	A	G	+	+	0.11363636	39,0,5,0	chr2,128950	Cytosol	A > G
chr2:128950991:A:G:UTR3:UGGT1	chr2	128950991	128950991	A	G	UTR3	UGGT1	ENST000003	.	.	A	G	+	+	0.43333333	17,0,13,0	chr2,128950	Cytosol	A > G
chr2:128950992:A:G:UTR3:UGGT1	chr2	128950992	128950992	A	G	UTR3	UGGT1	ENST000003	.	.	A	G	+	+	0.5625	14,0,18,0	chr2,128950	Cytosol	A > G
chr2:128951000:A:G:UTR3:UGGT1	chr2	128951000	128951000	A	G	UTR3	UGGT1	ENST000003	.	.	A	G	+	+	0.70967742	9,0,22,0	chr2,128950	Cytosol	A > G
chr2:128951006:A:G:UTR3:UGGT1	chr2	128951006	128951006	A	G	UTR3	UGGT1	ENST000003	.	.	A	G	+	+	0.52	12,0,13,0	chr2,128951	Cytosol	A > G
chr2:128951026:A:G:UTR3:UGGT1	chr2	128951026	128951026	A	G	UTR3	UGGT1	ENST000003	.	.	A	G	+	+	0.30555556	25,0,11,0	chr2,128951	Cytosol	A > G
chr2:128951080:A:G:UTR3:UGGT1	chr2	128951080	128951080	A	G	UTR3	UGGT1	ENST000003	.	.	A	G	+	+	0.50666667	37,0,38,0	chr2,128951	Cytosol	A > G
chr2:128951094:A:G:UTR3:UGGT1	chr2	128951094	128951094	A	G	UTR3	UGGT1	ENST000003	.	.	A	G	+	+	0.36231884	44,0,25,0	chr2,128951	Cytosol	A > G
chr2:128951095:A:G:UTR3:UGGT1	chr2	128951095	128951095	A	G	UTR3	UGGT1	ENST000003	.	.	A	G	+	+	0.52238806	32,0,35,0	chr2,128951	Cytosol	A > G
chr2:128951107:A:G:UTR3:UGGT1	chr2	128951107	128951107	A	G	UTR3	UGGT1	ENST000003	.	.	A	G	+	+	0.65217391	24,0,45,0	chr2,128951	Cytosol	A > G

chr2:128951123:A:G:UTR3:UGGT1	chr2	128951123	128951123	A	G	UTR3	UGGT1	ENST000003	.	.	A	G	+	+	0.11940299	59,0,8,0	chr2,128951	Cytosol	A > G
chr2:128951720:A:G:UTR3:UGGT1	chr2	128951720	128951720	A	G	UTR3	UGGT1	ENST000003	.	.	A	G	+	+	0.64583333	17,0,31,0	chr2,128951	Cytosol	A > G
chr2:128951723:A:G:UTR3:UGGT1	chr2	128951723	128951723	A	G	UTR3	UGGT1	ENST000003	.	.	A	G	+	+	0.48780488	21,0,20,0	chr2,128951	Cytosol	A > G
chr2:128951741:A:G:UTR3:UGGT1	chr2	128951741	128951741	A	G	UTR3	UGGT1	ENST000003	.	.	A	G	+	+	0.11538462	23,0,3,0	chr2,128951	Cytosol	A > G
chr2:128951747:A:G:UTR3:UGGT1	chr2	128951747	128951747	A	G	UTR3	UGGT1	ENST000003	.	.	A	G	+	+	0.09090909	20,0,2,0	chr2,128951	Cytosol	A > G
chr2:128951766:A:G:UTR3:UGGT1	chr2	128951766	128951766	A	G	UTR3	UGGT1	ENST000003	.	.	A	G	+	+	0.875	2,0,14,0	chr2,128951	Cytosol	A > G
chr2:128951771:A:G:UTR3:UGGT1	chr2	128951771	128951771	A	G	UTR3	UGGT1	ENST000003	.	.	A	G	+	+	0.29411765	12,0,5,0	chr2,128951	Cytosol	A > G
chr2:128951784:A:G:UTR3:UGGT1	chr2	128951784	128951784	A	G	UTR3	UGGT1	ENST000003	.	.	A	G	+	+	0.33333333	8,0,4,0	chr2,128951	Cytosol	A > G
chr2:128951806:A:G:UTR3:UGGT1	chr2	128951806	128951806	A	G	UTR3	UGGT1	ENST000003	.	.	A	G	+	+	0.25	15,0,5,0	chr2,128951	Cytosol	A > G
chr2:128951904:A:G:UTR3:UGGT1	chr2	128951904	128951904	A	G	UTR3	UGGT1	ENST000003	.	.	A	G	+	+	0.15909091	37,0,7,0	chr2,128951	Cytosol	A > G
chr2:128951941:A:G:UTR3:UGGT1	chr2	128951941	128951941	A	G	UTR3	UGGT1	ENST000003	.	.	A	G	+	+	0.19607843	41,0,10,0	chr2,128951	Cytosol	A > G
chr2:128952007:A:G:UTR3:UGGT1	chr2	128952007	128952007	A	G	UTR3	UGGT1	ENST000003	.	.	A	G	+	+	0.15384615	22,0,4,0	chr2,128952	Cytosol	A > G
chr2:128952041:A:G:UTR3:UGGT1	chr2	128952041	128952041	A	G	UTR3	UGGT1	ENST000003	.	.	A	G	+	+	0.23809524	32,0,10,0	chr2,128952	Cytosol	A > G
chr2:128952050:A:G:UTR3:UGGT1	chr2	128952050	128952050	A	G	UTR3	UGGT1	ENST000003	.	.	A	G	+	+	0.15	34,0,6,0	chr2,128952	Cytosol	A > G
chr2:128952084:A:G:UTR3:UGGT1	chr2	128952084	128952084	A	G	UTR3	UGGT1	ENST000003	.	.	A	G	+	+	0.06382979	44,0,3,0	chr2,128952	Cytosol	A > G
chr2:128952103:A:G:UTR3:UGGT1	chr2	128952103	128952103	A	G	UTR3	UGGT1	ENST000003	.	.	A	G	+	+	0.30434783	32,0,14,0	chr2,128952	Cytosol	A > G
chr2:128952115:A:G:UTR3:UGGT1	chr2	128952115	128952115	A	G	UTR3	UGGT1	ENST000003	.	.	A	G	+	+	0.10526316	34,0,4,0	chr2,128952	Cytosol	A > G
chr2:128952677:A:G:UTR3:UGGT1	chr2	128952677	128952677	A	G	UTR3	UGGT1	ENST000003	.	.	A	G	+	+	0.05434783	87,0,5,0	chr2,128952	Cytosol	A > G
chr2:128952678:A:G:UTR3:UGGT1	chr2	128952678	128952678	A	G	UTR3	UGGT1	ENST000003	.	.	A	G	+	+	0.04347826	88,0,4,0	chr2,128952	Cytosol	A > G
chr2:128952699:A:G:UTR3:UGGT1	chr2	128952699	128952699	A	G	UTR3	UGGT1	ENST000003	.	.	A	G	+	+	0.14117647	73,0,12,0	chr2,128952	Cytosol	A > G
chr2:128952700:A:G:UTR3:UGGT1	chr2	128952700	128952700	A	G	UTR3	UGGT1	ENST000003	.	.	A	G	+	+	0.02439024	80,0,2,0	chr2,128952	Cytosol	A > G
chr2:130916608:T:C:intronic:SMP	chr2	130916608	130916608	T	C	intronic	SMPD4	.	.	.	A	G	-	-	0.5	3,0,3,0	chr2,130916	Cytosol	A > G
chr2:130916696:T:C:intronic:SMP	chr2	130916696	130916696	T	C	intronic	SMPD4	.	.	.	A	G	-	-	0.6	2,0,3,0	chr2,130916	Cytosol	A > G
chr2:130917142:T:C:UTR3:SMPD4	chr2	130917142	130917142	T	C	UTR3	SMPD4	ENST000004	.	.	A	G	-	-	0.6	2,0,3,0	chr2,130917	Cytosol	A > G
chr2:131846622:T:C:intronic:FAM	chr2	131846622	131846622	T	C	intronic	FAM168B	.	.	.	A	G	-	-	0.23076923	10,0,3,0	chr2,131846	Cytosol	A > G
chr2:131846811:T:C:intronic:FAM	chr2	131846811	131846811	T	C	intronic	FAM168B	.	.	.	A	G	-	-	0.11764706	15,0,2,0	chr2,131846	Cytosol	A > G
chr2:131846812:T:C:intronic:FAM	chr2	131846812	131846812	T	C	intronic	FAM168B	.	.	.	A	G	-	-	0.23529412	13,0,4,0	chr2,131846	Cytosol	A > G
chr2:131849458:T:C:intronic:FAM	chr2	131849458	131849458	T	C	intronic	FAM168B	.	.	.	A	G	-	-	0.10344828	26,0,3,0	chr2,131849	Cytosol	A > G
chr2:131849459:T:C:intronic:FAM	chr2	131849459	131849459	T	C	intronic	FAM168B	.	.	.	A	G	-	-	0.07692308	24,0,2,0	chr2,131849	Cytosol	A > G
chr2:152334009:A:G:UTR3:RIF1	chr2	152334009	152334009	A	G	UTR3	RIF1	ENST000002	.	.	A	G	+	+	0.22222222	14,0,4,0	chr2,152334	Cytosol	A > G
chr2:152334058:A:G:UTR3:RIF1	chr2	152334058	152334058	A	G	UTR3	RIF1	ENST000002	.	.	A	G	+	+	0.54545455	5,0,6,0	chr2,152334	Cytosol	A > G
chr2:152334068:A:G:UTR3:RIF1	chr2	152334068	152334068	A	G	UTR3	RIF1	ENST000002	.	.	A	G	+	+	0.3	7,0,3,0	chr2,152334	Cytosol	A > G
chr2:152334279:A:G:UTR3:RIF1	chr2	152334279	152334279	A	G	UTR3	RIF1	ENST000002	.	.	A	G	+	+	0.57142857	9,0,12,0	chr2,152334	Cytosol	A > G
chr2:152334349:A:G:UTR3:RIF1	chr2	152334349	152334349	A	G	UTR3	RIF1	ENST000002	.	.	A	G	+	+	0.38235294	21,0,13,0	chr2,152334	Cytosol	A > G
chr2:152334389:A:G:UTR3:RIF1	chr2	152334389	152334389	A	G	UTR3	RIF1	ENST000002	.	.	A	G	+	+	0.15625	27,0,5,0	chr2,152334	Cytosol	A > G
chr2:152334421:A:G:UTR3:RIF1	chr2	152334421	152334421	A	G	UTR3	RIF1	ENST000002	.	.	A	G	+	+	0.08333333	22,0,2,0	chr2,152334	Cytosol	A > G
chr2:152334823:A:G:UTR3:RIF1	chr2	152334823	152334823	A	G	UTR3	RIF1	ENST000002	.	.	A	G	+	+	0.23076923	20,0,6,0	chr2,152334	Cytosol	A > G
chr2:152334857:A:G:UTR3:RIF1	chr2	152334857	152334857	A	G	UTR3	RIF1	ENST000002	.	.	A	G	+	+	0.17647059	14,0,3,0	chr2,152334	Cytosol	A > G
chr2:152334879:A:G:UTR3:RIF1	chr2	152334879	152334879	A	G	UTR3	RIF1	ENST000002	.	.	A	G	+	+	0.6	8,0,12,0	chr2,152334	Cytosol	A > G
chr2:152334880:A:G:UTR3:RIF1	chr2	152334880	152334880	A	G	UTR3	RIF1	ENST000002	.	.	A	G	+	+	0.78947368	4,0,15,0	chr2,152334	Cytosol	A > G
chr2:152334893:A:G:UTR3:RIF1	chr2	152334893	152334893	A	G	UTR3	RIF1	ENST000002	.	.	A	G	+	+	0.75	4,0,12,0	chr2,152334	Cytosol	A > G
chr2:160587494:A:G:intronic:3914	chr2	160587494	160587494	A	G	intronic	3月-07	.	.	.	A	G	+	+	0.3	7,0,3,0	chr2,160587	Cytosol	A > G
chr2:160587537:A:G:intronic:3914	chr2	160587537	160587537	A	G	intronic	3月-07	.	.	.	A	G	+	+	0.41666667	7,0,5,0	chr2,160587	Cytosol	A > G
chr2:160591403:A:G:intronic:3914	chr2	160591403	160591403	A	G	intronic	3月-07	.	.	.	A	G	+	+	1	0,0,5,0	chr2,160591	Cytosol	A > G
chr2:170558876:A:G:intronic:KLHL	chr2	170558876	170558876	A	G	intronic	KLHL23	.	.	.	A	G	+	+	1	0,0,6,0	chr2,170558	Cytosol	A > G
chr2:170559003:A:G:intronic:KLHL	chr2	170559003	170559003	A	G	intronic	KLHL23	.	.	.	A	G	+	+	0.6	2,0,3,0	chr2,170559	Cytosol	A > G
chr2:172178501:T:C:UTR3:METTL	chr2	172178501	172178501	T	C	UTR3	METTL8	ENST000003	.	.	A	G	-	-	0.2962963	19,0,8,0	chr2,172178	Cytosol	A > G
chr2:172178533:T:C:UTR3:METTL	chr2	172178533	172178533	T	C	UTR3	METTL8	ENST000003	.	.	A	G	-	-	0.76190476	5,0,16,0	chr2,172178	Cytosol	A > G
chr2:172178540:T:C:UTR3:METTL	chr2	172178540	172178540	T	C	UTR3	METTL8	ENST000003	.	.	A	G	-	-	0.25	12,0,4,0	chr2,172178	Cytosol	A > G
chr2:172178558:T:C:UTR3:METTL	chr2	172178558	172178558	T	C	UTR3	METTL8	ENST000003	.	.	A	G	-	-	0.55555556	8,0,10,0	chr2,172178	Cytosol	A > G
chr2:172178610:T:C:UTR3:METTL	chr2	172178610	172178610	T	C	UTR3	METTL8	ENST000003	.	.	A	G	-	-	0.69230769	8,0,18,0	chr2,172178	Cytosol	A > G
chr2:172178637:T:C:UTR3:METTL	chr2	172178637	172178637	T	C	UTR3	METTL8	ENST000003	.	.	A	G	-	-	0.16666667	20,0,4,0	chr2,172178	Cytosol	A > G
chr2:172178669:T:C:UTR3:METTL	chr2	172178669	172178669	T	C	UTR3	METTL8	ENST000003	.	.	A	G	-	-	0.52941176	8,0,9,0	chr2,172178	Cytosol	A > G
chr2:172178670:T:C:UTR3:METTL	chr2	172178670	172178670	T	C	UTR3	METTL8	ENST000003	.	.	A	G	-	-	0.25	12,0,4,0	chr2,172178	Cytosol	A > G
chr2:172178679:T:C:UTR3:METTL	chr2	172178679	172178679	T	C	UTR3	METTL8	ENST000003	.	.	A	G	-	-	0.72222222	5,0,13,0	chr2,172178	Cytosol	A > G
chr2:172178680:T:C:UTR3:METTL	chr2	172178680	172178680	T	C	UTR3	METTL8	ENST000003	.	.	A	G	-	-	0.41176471	10,0,7,0	chr2,172178	Cytosol	A > G
chr2:172178729:T:C:UTR3:METTL	chr2	172178729	172178729	T	C	UTR3	METTL8	ENST000003	.	.	A	G	-	-	0.36842105	12,0,7,0	chr2,172178	Cytosol	A > G
chr2:172178745:T:C:UTR3:METTL	chr2	172178745	172178745	T	C	UTR3	METTL8	ENST000003	.	.	A	G	-	-	0.57894737	8,0,11,0	chr2,172178	Cytosol	A > G
chr2:172178746:T:C:UTR3:METTL	chr2	172178746	172178746	T	C	UTR3	METTL8	ENST000003	.	.	A	G	-	-	0.57894737	8,0,11,0	chr2,172178	Cytosol	A > G
chr2:172178753:T:C:UTR3:METTL	chr2	172178753	172178753	T	C	UTR3	METTL8	ENST000003	.	.	A	G	-	-	0.26315789	14,0,5,0	chr2,172178	Cytosol	A > G
chr2:172178754:T:C:UTR3:METTL	chr2	172178754	172178754	T	C	UTR3	METTL8	ENST000003	.	.	A	G	-	-	0.10526316	17,0,2,0	chr2,172178	Cytosol	A > G
chr2:172178762:T:C:UTR3:METTL	chr2	172178762	172178762	T	C	UTR3	METTL8	ENST000003	.	.	A	G	-	-	0.4375	9,0,7,0	chr2,172178	Cytosol	A > G
chr2:172178988:T:C:UTR3:METTL	chr2	172178988	172178988	T	C	UTR3	METTL8	ENST000003	.	.	A	G	-	-	0.65853659	14,0,27,0	chr2,172178	Cytosol	A > G
chr2:172178990:T:C:UTR3:METTL	chr2	172178990	172178990	T	C	UTR3	METTL8	ENST000003	.	.	A	G	-	-	0.28571429	30,0,12,0	chr2,172178	Cytosol	A > G

chr2:172179003:T:C:UTR3:METTL8	chr2	172179003	172179003	T	C	UTR3	METTL8	ENST000003	.	.	A	G	-	-	0.39130435	14,0,9,0	chr2,172179	Cytosol	A > G
chr2:172179129:T:C:UTR3:METTL8	chr2	172179129	172179129	T	C	UTR3	METTL8	ENST000003	.	.	A	G	-	-	1	0,0,12,0	chr2,172179	Cytosol	A > G
chr2:172179132:T:C:UTR3:METTL8	chr2	172179132	172179132	T	C	UTR3	METTL8	ENST000003	.	.	A	G	-	-	0.82352941	3,0,14,0	chr2,172179	Cytosol	A > G
chr2:172179142:T:C:UTR3:METTL8	chr2	172179142	172179142	T	C	UTR3	METTL8	ENST000003	.	.	A	G	-	-	0.52631579	9,0,10,0	chr2,172179	Cytosol	A > G
chr2:172179191:T:C:UTR3:METTL8	chr2	172179191	172179191	T	C	UTR3	METTL8	ENST000003	.	.	A	G	-	-	0.07142857	26,0,2,0	chr2,172179	Cytosol	A > G
chr2:172179197:T:C:UTR3:METTL8	chr2	172179197	172179197	T	C	UTR3	METTL8	ENST000003	.	.	A	G	-	-	0.22222222	21,0,6,0	chr2,172179	Cytosol	A > G
chr2:172179202:T:C:UTR3:METTL8	chr2	172179202	172179202	T	C	UTR3	METTL8	ENST000003	.	.	A	G	-	-	0.81481481	5,0,22,0	chr2,172179	Cytosol	A > G
chr2:172179841:T:C:UTR3:METTL8	chr2	172179841	172179841	T	C	UTR3	METTL8	ENST000003	.	.	A	G	-	-	0.13793103	25,0,4,0	chr2,172179	Cytosol	A > G
chr2:172179922:T:C:UTR3:METTL8	chr2	172179922	172179922	T	C	UTR3	METTL8	ENST000003	.	.	A	G	-	-	0.13793103	25,0,4,0	chr2,172179	Cytosol	A > G
chr2:172179937:T:C:UTR3:METTL8	chr2	172179937	172179937	T	C	UTR3	METTL8	ENST000003	.	.	A	G	-	-	0.34782609	15,0,8,0	chr2,172179	Cytosol	A > G
chr2:172179940:T:C:UTR3:METTL8	chr2	172179940	172179940	T	C	UTR3	METTL8	ENST000003	.	.	A	G	-	-	0.20833333	19,0,5,0	chr2,172179	Cytosol	A > G
chr2:172179947:T:C:UTR3:METTL8	chr2	172179947	172179947	T	C	UTR3	METTL8	ENST000003	.	.	A	G	-	-	0.2173913	18,0,5,0	chr2,172179	Cytosol	A > G
chr2:172179986:T:C:UTR3:METTL8	chr2	172179986	172179986	T	C	UTR3	METTL8	ENST000003	.	.	A	G	-	-	0.375	5,0,3,0	chr2,172179	Cytosol	A > G
chr2:172180032:T:C:UTR3:METTL8	chr2	172180032	172180032	T	C	UTR3	METTL8	ENST000003	.	.	A	G	-	-	0.09375	29,0,3,0	chr2,172180	Cytosol	A > G
chr2:172180039:T:C:UTR3:METTL8	chr2	172180039	172180039	T	C	UTR3	METTL8	ENST000003	.	.	A	G	-	-	0.05555556	34,0,2,0	chr2,172180	Cytosol	A > G
chr2:176809724:T:C:intronic:KIAA1	chr2	176809724	176809724	T	C	intronic	KIAA1715	.	.	.	A	G	-	-	0.25	6,0,2,0	chr2,176809	Cytosol	A > G
chr2:176809755:T:C:intronic:KIAA1	chr2	176809755	176809755	T	C	intronic	KIAA1715	.	.	.	A	G	-	-	0.28571429	5,0,2,0	chr2,176809	Cytosol	A > G
chr2:176809803:T:C:intronic:KIAA1	chr2	176809803	176809803	T	C	intronic	KIAA1715	.	.	.	A	G	-	-	0.55555556	4,0,5,0	chr2,176809	Cytosol	A > G
chr2:176809804:T:C:intronic:KIAA1	chr2	176809804	176809804	T	C	intronic	KIAA1715	.	.	.	A	G	-	-	0.22222222	7,0,2,0	chr2,176809	Cytosol	A > G
chr2:176809806:T:C:intronic:KIAA1	chr2	176809806	176809806	T	C	intronic	KIAA1715	.	.	.	A	G	-	-	0.9	1,0,9,0	chr2,176809	Cytosol	A > G
chr2:176809846:T:C:intronic:KIAA1	chr2	176809846	176809846	T	C	intronic	KIAA1715	.	.	.	A	G	-	-	0.4	3,0,2,0	chr2,176809	Cytosol	A > G
chr2:176810067:T:C:intronic:KIAA1	chr2	176810067	176810067	T	C	intronic	KIAA1715	.	.	.	A	G	-	-	0.5	3,0,3,0	chr2,176810	Cytosol	A > G
chr2:176810133:T:C:intronic:KIAA1	chr2	176810133	176810133	T	C	intronic	KIAA1715	.	.	.	A	G	-	-	0.27272727	8,0,3,0	chr2,176810	Cytosol	A > G
chr2:176810146:T:C:intronic:KIAA1	chr2	176810146	176810146	T	C	intronic	KIAA1715	.	.	.	A	G	-	-	0.5	6,0,6,0	chr2,176810	Cytosol	A > G
chr2:176810150:T:C:intronic:KIAA1	chr2	176810150	176810150	T	C	intronic	KIAA1715	.	.	.	A	G	-	-	0.83333333	2,0,10,0	chr2,176810	Cytosol	A > G
chr2:176810151:T:C:intronic:KIAA1	chr2	176810151	176810151	T	C	intronic	KIAA1715	.	.	.	A	G	-	-	0.5	6,0,6,0	chr2,176810	Cytosol	A > G
chr2:178184846:T:C:ncRNA_intron	chr2	178184846	178184846	T	C	ncRNA_intro	AC074286.1	.	.	.	A	G	-	-	0.44444444	5,0,4,0	chr2,178184	Cytosol	A > G
chr2:178186007:T:C:ncRNA_intron	chr2	178186007	178186007	T	C	ncRNA_intro	AC074286.1	.	.	.	A	G	-	-	0.42857143	8,0,6,0	chr2,178186	Cytosol	A > G
chr2:178186008:T:C:ncRNA_intron	chr2	178186008	178186008	T	C	ncRNA_intro	AC074286.1	.	.	.	A	G	-	-	0.38461538	8,0,5,0	chr2,178186	Cytosol	A > G
chr2:178186718:T:C:ncRNA_exoni	chr2	178186718	178186718	T	C	ncRNA_exon	AC074286.1	.	.	.	A	G	-	-	0.4	9,0,6,0	chr2,178186	Cytosol	A > G
chr2:179299758:A:G:ncRNA_intro	chr2	179299758	179299758	A	G	ncRNA_intro	AC009948.5	.	.	.	A	G	+	+-	0.3	7,0,3,0	chr2,179299	Cytosol	A > G
chr2:179299864:A:G:ncRNA_intro	chr2	179299864	179299864	A	G	ncRNA_intro	AC009948.5	.	.	.	A	G	+	+-	0.375	5,0,3,0	chr2,179299	Cytosol	A > G
chr2:179299876:A:G:ncRNA_intro	chr2	179299876	179299876	A	G	ncRNA_intro	AC009948.5	.	.	.	A	G	+	+-	0.15384615	11,0,2,0	chr2,179299	Cytosol	A > G
chr2:179299883:A:G:ncRNA_intro	chr2	179299883	179299883	A	G	ncRNA_intro	AC009948.5	.	.	.	A	G	+	+-	0.42857143	8,0,6,0	chr2,179299	Cytosol	A > G
chr2:179299963:A:G:ncRNA_intro	chr2	179299963	179299963	A	G	ncRNA_intro	AC009948.5	.	.	.	A	G	+	+-	0.15384615	11,0,2,0	chr2,179299	Cytosol	A > G
chr2:179299989:A:G:ncRNA_intro	chr2	179299989	179299989	A	G	ncRNA_intro	AC009948.5	.	.	.	A	G	+	+-	0.46666667	8,0,7,0	chr2,179299	Cytosol	A > G
chr2:179300016:A:G:ncRNA_intro	chr2	179300016	179300016	A	G	ncRNA_intro	AC009948.5	.	.	.	A	G	+	+-	0.23529412	13,0,4,0	chr2,179300	Cytosol	A > G
chr2:179300030:A:G:ncRNA_intro	chr2	179300030	179300030	A	G	ncRNA_intro	AC009948.5	.	.	.	A	G	+	+-	0.28571429	10,0,4,0	chr2,179300	Cytosol	A > G
chr2:179300031:A:G:ncRNA_intro	chr2	179300031	179300031	A	G	ncRNA_intro	AC009948.5	.	.	.	A	G	+	+-	0.14285714	12,0,2,0	chr2,179300	Cytosol	A > G
chr2:179300070:A:G:ncRNA_intro	chr2	179300070	179300070	A	G	ncRNA_intro	AC009948.5	.	.	.	A	G	+	+-	0.15789474	16,0,3,0	chr2,179300	Cytosol	A > G
chr2:179303617:A:G:ncRNA_exoni	chr2	179303617	179303617	A	G	ncRNA_exon	AC009948.5	.	.	.	A	G	+	+-	0.5	3,0,3,0	chr2,179303	Cytosol	A > G
chr2:190611982:T:C:UTR3:OSGEPL	chr2	190611982	190611982	T	C	UTR3	OSGEPL1	ENST000005	.	.	A	G	-	+-	0.0483871	59,0,3,0	chr2,190611	Cytosol	A > G
chr2:190612029:T:C:UTR3:OSGEPL	chr2	190612029	190612029	T	C	UTR3	OSGEPL1	ENST000005	.	.	A	G	-	+-	0.13333333	13,0,2,0	chr2,190612	Cytosol	A > G
chr2:198357113:T:C:intronic:HSPD	chr2	198357113	198357113	T	C	intronic	HSPD1	.	.	.	A	G	-	-	0.8	1,0,4,0	chr2,198357	Cytosol	A > G
chr2:198357193:T:C:intronic:HSPD	chr2	198357193	198357193	T	C	intronic	HSPD1	.	.	.	A	G	-	-	0.4	3,0,2,0	chr2,198357	Cytosol	A > G
chr2:200802276:T:C:intronic:TYW5	chr2	200802276	200802276	T	C	intronic	TYW5	.	.	.	A	G	-	+-	1	0,0,5,0	chr2,200802	Cytosol	A > G
chr2:201755743:A:G:intronic:NIF3	chr2	201755743	201755743	A	G	intronic	NIF3L1	.	.	.	A	G	+	+	0.66666667	2,0,4,0	chr2,201755	Cytosol	A > G
chr2:201755799:A:G:intronic:NIF3	chr2	201755799	201755799	A	G	intronic	NIF3L1	.	.	.	A	G	+	+	0.4	3,0,2,0	chr2,201755	Cytosol	A > G
chr2:201774173:T:C:UTR3:ORC2	chr2	201774173	201774173	T	C	UTR3	ORC2	ENST000002	.	.	A	G	-	-	0.22222222	21,0,6,0	chr2,201774	Cytosol	A > G
chr2:201774235:T:C:UTR3:ORC2	chr2	201774235	201774235	T	C	UTR3	ORC2	ENST000002	.	.	A	G	-	-	0.1875	13,0,3,0	chr2,201774	Cytosol	A > G
chr2:201774244:T:C:UTR3:ORC2	chr2	201774244	201774244	T	C	UTR3	ORC2	ENST000002	.	.	A	G	-	-	0.61111111	7,0,11,0	chr2,201774	Cytosol	A > G
chr2:201774261:T:C:UTR3:ORC2	chr2	201774261	201774261	T	C	UTR3	ORC2	ENST000002	.	.	A	G	-	-	0.1875	13,0,3,0	chr2,201774	Cytosol	A > G
chr2:201774264:T:C:UTR3:ORC2	chr2	201774264	201774264	T	C	UTR3	ORC2	ENST000002	.	.	A	G	-	-	0.40909091	13,0,9,0	chr2,201774	Cytosol	A > G
chr2:201774341:T:C:UTR3:ORC2	chr2	201774341	201774341	T	C	UTR3	ORC2	ENST000002	.	.	A	G	-	-	0.31578947	13,0,6,0	chr2,201774	Cytosol	A > G
chr2:201774393:T:C:UTR3:ORC2	chr2	201774393	201774393	T	C	UTR3	ORC2	ENST000002	.	.	A	G	-	-	0.64285714	5,0,9,0	chr2,201774	Cytosol	A > G
chr2:201774600:T:C:UTR3:ORC2	chr2	201774600	201774600	T	C	UTR3	ORC2	ENST000002	.	.	A	G	-	-	0.30555556	25,0,11,0	chr2,201774	Cytosol	A > G
chr2:201774625:T:C:UTR3:ORC2	chr2	201774625	201774625	T	C	UTR3	ORC2	ENST000002	.	.	A	G	-	-	0.04444444	43,0,2,0	chr2,201774	Cytosol	A > G
chr2:201774641:T:C:UTR3:ORC2	chr2	201774641	201774641	T	C	UTR3	ORC2	ENST000002	.	.	A	G	-	-	0.04761905	40,0,2,0	chr2,201774	Cytosol	A > G
chr2:201774668:T:C:UTR3:ORC2	chr2	201774668	201774668	T	C	UTR3	ORC2	ENST000002	.	.	A	G	-	-	0.2	28,0,7,0	chr2,201774	Cytosol	A > G
chr2:201774685:T:C:UTR3:ORC2	chr2	201774685	201774685	T	C	UTR3	ORC2	ENST000002	.	.	A	G	-	-	0.96	1,0,24,0	chr2,201774	Cytosol	A > G
chr2:201774687:T:C:UTR3:ORC2	chr2	201774687	201774687	T	C	UTR3	ORC2	ENST000002	.	.	A	G	-	-	0.17857143	23,0,5,0	chr2,201774	Cytosol	A > G
chr2:201774704:T:C:UTR3:ORC2	chr2	201774704	201774704	T	C	UTR3	ORC2	ENST000002	.	.	A	G	-	-	0.07142857	26,0,2,0	chr2,201774	Cytosol	A > G
chr2:201774774:T:C:UTR3:ORC2	chr2	201774774	201774774	T	C	UTR3	ORC2	ENST000002	.	.	A	G	-	-	0.26923077	19,0,7,0	chr2,201774	Cytosol	A > G
chr2:201774775:T:C:UTR3:ORC2	chr2	201774775	201774775	T	C	UTR3	ORC2	ENST000002	.	.	A	G	-	-	0.08695652	21,0,2,0	chr2,201774	Cytosol	A > G

chr2:201842323:T:C:ncRNA_intro	chr2	201842323	201842323	T	C	ncRNA_intro	AC005037.3	.	.	.	A	G	-	+-	0.41666667	7,0,5,0	chr2,201842	Cytosol	A > G
chr2:201842408:T:C:ncRNA_intro	chr2	201842408	201842408	T	C	ncRNA_intro	AC005037.3	.	.	.	A	G	-	+-	0.4	3,0,2,0	chr2,201842	Cytosol	A > G
chr2:201842479:T:C:ncRNA_intro	chr2	201842479	201842479	T	C	ncRNA_intro	AC005037.3	.	.	.	A	G	-	+-	0.36363636	7,0,4,0	chr2,201842	Cytosol	A > G
chr2:201842492:T:C:ncRNA_intro	chr2	201842492	201842492	T	C	ncRNA_intro	AC005037.3	.	.	.	A	G	-	+-	0.27272727	8,0,3,0	chr2,201842	Cytosol	A > G
chr2:201842561:T:C:ncRNA_intro	chr2	201842561	201842561	T	C	ncRNA_intro	AC005037.3	.	.	.	A	G	-	+-	0.3	7,0,3,0	chr2,201842	Cytosol	A > G
chr2:201842569:T:C:ncRNA_intro	chr2	201842569	201842569	T	C	ncRNA_intro	AC005037.3	.	.	.	A	G	-	+-	0.75	3,0,9,0	chr2,201842	Cytosol	A > G
chr2:201843373:T:C:ncRNA_exoni	chr2	201843373	201843373	T	C	ncRNA_exon	AC005037.3	.	.	.	A	G	-	+-	0.57142857	3,0,4,0	chr2,201843	Cytosol	A > G
chr2:201843379:T:C:ncRNA_exoni	chr2	201843379	201843379	T	C	ncRNA_exon	AC005037.3	.	.	.	A	G	-	+-	0.8	1,0,4,0	chr2,201843	Cytosol	A > G
chr2:202028867:A:G:UTR3:CFLAR	chr2	202028867	202028867	A	G	UTR3	CFLAR	ENST000003	.	.	A	G	+	+	0.14285714	12,0,2,0	chr2,202028	Cytosol	A > G
chr2:202151362:A:G:UTR3:CASP8	chr2	202151362	202151362	A	G	UTR3	CASP8	ENST000003	.	.	A	G	+	+	0.25	30,0,10,0	chr2,202151	Cytosol	A > G
chr2:202152062:A:G:UTR3:CASP8	chr2	202152062	202152062	A	G	UTR3	CASP8	ENST000003	.	.	A	G	+	+	0.07894737	35,0,3,0	chr2,202152	Cytosol	A > G
chr2:202152078:A:G:UTR3:CASP8	chr2	202152078	202152078	A	G	UTR3	CASP8	ENST000003	.	.	A	G	+	+	0.0625	30,0,2,0	chr2,202152	Cytosol	A > G
chr2:202152106:A:G:UTR3:CASP8	chr2	202152106	202152106	A	G	UTR3	CASP8	ENST000003	.	.	A	G	+	+	0.26470588	25,0,9,0	chr2,202152	Cytosol	A > G
chr2:203745036:T:C:UTR3:WDR12	chr2	203745036	203745036	T	C	UTR3	WDR12	ENST000002	.	.	A	G	-	-	0.23333333	23,0,7,0	chr2,203745	Cytosol	A > G
chr2:203745054:T:C:UTR3:WDR12	chr2	203745054	203745054	T	C	UTR3	WDR12	ENST000002	.	.	A	G	-	-	0.125	21,0,3,0	chr2,203745	Cytosol	A > G
chr2:203745105:T:C:UTR3:WDR12	chr2	203745105	203745105	T	C	UTR3	WDR12	ENST000002	.	.	A	G	-	-	0.31578947	13,0,6,0	chr2,203745	Cytosol	A > G
chr2:203745149:T:C:UTR3:WDR12	chr2	203745149	203745149	T	C	UTR3	WDR12	ENST000002	.	.	A	G	-	-	0.15789474	16,0,3,0	chr2,203745	Cytosol	A > G
chr2:203745170:T:C:UTR3:WDR12	chr2	203745170	203745170	T	C	UTR3	WDR12	ENST000002	.	.	A	G	-	-	0.31578947	13,0,6,0	chr2,203745	Cytosol	A > G
chr2:203745171:T:C:UTR3:WDR12	chr2	203745171	203745171	T	C	UTR3	WDR12	ENST000002	.	.	A	G	-	-	0.55	9,0,11,0	chr2,203745	Cytosol	A > G
chr2:203745195:T:C:UTR3:WDR12	chr2	203745195	203745195	T	C	UTR3	WDR12	ENST000002	.	.	A	G	-	-	0.63157895	7,0,12,0	chr2,203745	Cytosol	A > G
chr2:203745218:T:C:UTR3:WDR12	chr2	203745218	203745218	T	C	UTR3	WDR12	ENST000002	.	.	A	G	-	-	0.58823529	7,0,10,0	chr2,203745	Cytosol	A > G
chr2:203745224:T:C:UTR3:WDR12	chr2	203745224	203745224	T	C	UTR3	WDR12	ENST000002	.	.	A	G	-	-	0.1875	13,0,3,0	chr2,203745	Cytosol	A > G
chr2:204162515:A:G:UTR3:CYP20	chr2	204162515	204162515	A	G	UTR3	CYP20A1	ENST000004	.	.	A	G	+	+	0.375	5,0,3,0	chr2,204162	Cytosol	A > G
chr2:204162587:A:G:UTR3:CYP20	chr2	204162587	204162587	A	G	UTR3	CYP20A1	ENST000004	.	.	A	G	+	+	0.4	3,0,2,0	chr2,204162	Cytosol	A > G
chr2:204162588:A:G:UTR3:CYP20	chr2	204162588	204162588	A	G	UTR3	CYP20A1	ENST000004	.	.	A	G	+	+	0.33333333	4,0,2,0	chr2,204162	Cytosol	A > G
chr2:204162597:A:G:UTR3:CYP20	chr2	204162597	204162597	A	G	UTR3	CYP20A1	ENST000004	.	.	A	G	+	+	0.5	3,0,3,0	chr2,204162	Cytosol	A > G
chr2:204162601:A:G:UTR3:CYP20	chr2	204162601	204162601	A	G	UTR3	CYP20A1	ENST000004	.	.	A	G	+	+	0.4	3,0,2,0	chr2,204162	Cytosol	A > G
chr2:204163581:A:G:downstream:	chr2	204163581	204163581	A	G	downstream	CYP20A1	.	.	.	A	G	+	+	0.4	3,0,2,0	chr2,204163	Cytosol	A > G
chr2:204166024:A:G:intergenic:CY	chr2	204166024	204166024	A	G	intergenic	CYP20A1,ABI	dist=3015;dis	.	.	A	G	+	+	0.33333333	4,0,2,0	chr2,204166	Cytosol	A > G
chr2:204166049:A:G:intergenic:CY	chr2	204166049	204166049	A	G	intergenic	CYP20A1,ABI	dist=3040;dis	.	.	A	G	+	+	0.27272727	8,0,3,0	chr2,204166	Cytosol	A > G
chr2:204167136:A:G:intergenic:CY	chr2	204167136	204167136	A	G	intergenic	CYP20A1,ABI	dist=4127;dis	.	.	A	G	+	+	0.33333333	4,0,2,0	chr2,204167	Cytosol	A > G
chr2:204167467:A:G:intergenic:CY	chr2	204167467	204167467	A	G	intergenic	CYP20A1,ABI	dist=4458;dis	.	.	A	G	+	+	0.83333333	1,0,5,0	chr2,204167	Cytosol	A > G
chr2:204167496:A:G:intergenic:CY	chr2	204167496	204167496	A	G	intergenic	CYP20A1,ABI	dist=4487;dis	.	.	A	G	+	+	0.28571429	5,0,2,0	chr2,204167	Cytosol	A > G
chr2:204167515:A:G:intergenic:CY	chr2	204167515	204167515	A	G	intergenic	CYP20A1,ABI	dist=4506;dis	.	.	A	G	+	+	0.4	3,0,2,0	chr2,204167	Cytosol	A > G
chr2:204167516:A:G:intergenic:CY	chr2	204167516	204167516	A	G	intergenic	CYP20A1,ABI	dist=4507;dis	.	.	A	G	+	+	0.4	3,0,2,0	chr2,204167	Cytosol	A > G
chr2:204167863:A:G:intergenic:CY	chr2	204167863	204167863	A	G	intergenic	CYP20A1,ABI	dist=4854;dis	.	.	A	G	+	+	0.5	4,0,4,0	chr2,204167	Cytosol	A > G
chr2:204167911:A:G:intergenic:CY	chr2	204167911	204167911	A	G	intergenic	CYP20A1,ABI	dist=4902;dis	.	.	A	G	+	+	0.33333333	4,0,2,0	chr2,204167	Cytosol	A > G
chr2:204168008:A:G:intergenic:CY	chr2	204168008	204168008	A	G	intergenic	CYP20A1,ABI	dist=4999;dis	.	.	A	G	+	+	0.22222222	7,0,2,0	chr2,204168	Cytosol	A > G
chr2:204168044:A:G:intergenic:CY	chr2	204168044	204168044	A	G	intergenic	CYP20A1,ABI	dist=5035;dis	.	.	A	G	+	+	0.625	3,0,5,0	chr2,204168	Cytosol	A > G
chr2:204168053:A:G:intergenic:CY	chr2	204168053	204168053	A	G	intergenic	CYP20A1,ABI	dist=5044;dis	.	.	A	G	+	+	0.28571429	5,0,2,0	chr2,204168	Cytosol	A > G
chr2:204168575:A:G:intergenic:CY	chr2	204168575	204168575	A	G	intergenic	CYP20A1,ABI	dist=5566;dis	.	.	A	G	+	+	0.4	3,0,2,0	chr2,204168	Cytosol	A > G
chr2:204168964:A:G:intergenic:CY	chr2	204168964	204168964	A	G	intergenic	CYP20A1,ABI	dist=5955;dis	.	.	A	G	+	+	0.22222222	7,0,2,0	chr2,204168	Cytosol	A > G
chr2:204168982:A:G:intergenic:CY	chr2	204168982	204168982	A	G	intergenic	CYP20A1,ABI	dist=5973;dis	.	.	A	G	+	+	0.71428571	2,0,5,0	chr2,204168	Cytosol	A > G
chr2:204168998:A:G:intergenic:CY	chr2	204168998	204168998	A	G	intergenic	CYP20A1,ABI	dist=5989;dis	.	.	A	G	+	+	0.6	2,0,3,0	chr2,204168	Cytosol	A > G
chr2:204169060:A:G:intergenic:CY	chr2	204169060	204169060	A	G	intergenic	CYP20A1,ABI	dist=6051;dis	.	.	A	G	+	+	0.875	1,0,7,0	chr2,204169	Cytosol	A > G
chr2:204169105:A:G:intergenic:CY	chr2	204169105	204169105	A	G	intergenic	CYP20A1,ABI	dist=6096;dis	.	.	A	G	+	+	0.4	6,0,4,0	chr2,204169	Cytosol	A > G
chr2:204169127:A:G:intergenic:CY	chr2	204169127	204169127	A	G	intergenic	CYP20A1,ABI	dist=6118;dis	.	.	A	G	+	+	0.83333333	1,0,5,0	chr2,204169	Cytosol	A > G
chr2:204169194:A:G:intergenic:CY	chr2	204169194	204169194	A	G	intergenic	CYP20A1,ABI	dist=6185;dis	.	.	A	G	+	+	0.375	5,0,3,0	chr2,204169	Cytosol	A > G
chr2:204169468:A:G:intergenic:CY	chr2	204169468	204169468	A	G	intergenic	CYP20A1,ABI	dist=6459;dis	.	.	A	G	+	+	0.6	4,0,6,0	chr2,204169	Cytosol	A > G
chr2:204169634:A:G:intergenic:CY	chr2	204169634	204169634	A	G	intergenic	CYP20A1,ABI	dist=6625;dis	.	.	A	G	+	+	0.15384615	11,0,2,0	chr2,204169	Cytosol	A > G
chr2:204169993:A:G:intergenic:CY	chr2	204169993	204169993	A	G	intergenic	CYP20A1,ABI	dist=6984;dis	.	.	A	G	+	+	0.8	1,0,4,0	chr2,204169	Cytosol	A > G
chr2:204170017:A:G:intergenic:CY	chr2	204170017	204170017	A	G	intergenic	CYP20A1,ABI	dist=7008;dis	.	.	A	G	+	+	0.4	3,0,2,0	chr2,204170	Cytosol	A > G
chr2:204170023:A:G:intergenic:CY	chr2	204170023	204170023	A	G	intergenic	CYP20A1,ABI	dist=7014;dis	.	.	A	G	+	+	0.4	3,0,2,0	chr2,204170	Cytosol	A > G
chr2:204170028:A:G:intergenic:CY	chr2	204170028	204170028	A	G	intergenic	CYP20A1,ABI	dist=7019;dis	.	.	A	G	+	+	0.5	3,0,3,0	chr2,204170	Cytosol	A > G
chr2:204170063:A:G:intergenic:CY	chr2	204170063	204170063	A	G	intergenic	CYP20A1,ABI	dist=7054;dis	.	.	A	G	+	+	0.42857143	4,0,3,0	chr2,204170	Cytosol	A > G
chr2:204170105:A:G:intergenic:CY	chr2	204170105	204170105	A	G	intergenic	CYP20A1,ABI	dist=7096;dis	.	.	A	G	+	+	0.57142857	3,0,4,0	chr2,204170	Cytosol	A > G
chr2:204170108:A:G:intergenic:CY	chr2	204170108	204170108	A	G	intergenic	CYP20A1,ABI	dist=7099;dis	.	.	A	G	+	+	0.4	3,0,2,0	chr2,204170	Cytosol	A > G
chr2:204170161:A:G:intergenic:CY	chr2	204170161	204170161	A	G	intergenic	CYP20A1,ABI	dist=7152;dis	.	.	A	G	+	+	0.25	6,0,2,0	chr2,204170	Cytosol	A > G
chr2:204170170:A:G:intergenic:CY	chr2	204170170	204170170	A	G	intergenic	CYP20A1,ABI	dist=7161;dis	.	.	A	G	+	+	0.5	3,0,3,0	chr2,204170	Cytosol	A > G
chr2:204170185:A:G:intergenic:CY	chr2	204170185	204170185	A	G	intergenic	CYP20A1,ABI	dist=7176;dis	.	.	A	G	+	+	0.375	5,0,3,0	chr2,204170	Cytosol	A > G
chr2:204170197:A:G:intergenic:CY	chr2	204170197	204170197	A	G	intergenic	CYP20A1,ABI	dist=7188;dis	.	.	A	G	+	+	0.3	7,0,3,0	chr2,204170	Cytosol	A > G
chr2:206981516:T:C:ncRNA_intro	chr2	206981516	206981516	T	C	ncRNA_intro	AC007383.4	.	.	.	A	G	-	-+	0.52173913	11,0,12,0	chr2,206981	Cytosol	A > G
chr2:206981539:T:C:ncRNA_intro	chr2	206981539	206981539	T	C	ncRNA_intro	AC007383.4	.	.	.	A	G	-	-+	0.19047619	17,0,4,0	chr2,206981	Cytosol	A > G
chr2:206981581:T:C:ncRNA_intro	chr2	206981581	206981581	T	C	ncRNA_intro	AC007383.4	.	.	.	A	G	-	-+	0.35714286	9,0,5,0	chr2,206981	Cytosol	A > G

chr2:206981600:T:C:ncRNA_intror	chr2	206981600	206981600	T	C	ncRNA_intro	AC007383.4	.	.	.	A	G	-	-+	0.59090909	9,0,13,0	chr2,206981	Cytosol	A > G
chr2:206981622:T:C:ncRNA_intror	chr2	206981622	206981622	T	C	ncRNA_intro	AC007383.4	.	.	.	A	G	-	-+	0.5	12,0,12,0	chr2,206981	Cytosol	A > G
chr2:206981628:T:C:ncRNA_intror	chr2	206981628	206981628	T	C	ncRNA_intro	AC007383.4	.	.	.	A	G	-	-+	0.9047619	2,0,19,0	chr2,206981	Cytosol	A > G
chr2:206981674:T:C:ncRNA_intror	chr2	206981674	206981674	T	C	ncRNA_intro	AC007383.4	.	.	.	A	G	-	-+	0.05882353	32,0,2,0	chr2,206981	Cytosol	A > G
chr2:206981691:T:C:ncRNA_intror	chr2	206981691	206981691	T	C	ncRNA_intro	AC007383.4	.	.	.	A	G	-	-+	0.75862069	7,0,22,0	chr2,206981	Cytosol	A > G
chr2:206981705:T:C:ncRNA_intror	chr2	206981705	206981705	T	C	ncRNA_intro	AC007383.4	.	.	.	A	G	-	-+	0.48275862	15,0,14,0	chr2,206981	Cytosol	A > G
chr2:206981729:T:C:ncRNA_intror	chr2	206981729	206981729	T	C	ncRNA_intro	AC007383.4	.	.	.	A	G	-	-+	0.33333333	16,0,8,0	chr2,206981	Cytosol	A > G
chr2:206981759:T:C:ncRNA_intror	chr2	206981759	206981759	T	C	ncRNA_intro	AC007383.4	.	.	.	A	G	-	-+	0.35483871	19,0,11,1	chr2,206981	Cytosol	A > G
chr2:206981866:T:C:ncRNA_intror	chr2	206981866	206981866	T	C	ncRNA_intro	AC007383.4	.	.	.	A	G	-	-+	0.09459459	67,0,7,0	chr2,206981	Cytosol	A > G
chr2:206982257:T:C:ncRNA_intror	chr2	206982257	206982257	T	C	ncRNA_intro	AC007383.4	.	.	.	A	G	-	-+	0.84	4,0,21,0	chr2,206982	Cytosol	A > G
chr2:206982295:T:C:ncRNA_intror	chr2	206982295	206982295	T	C	ncRNA_intro	AC007383.4	.	.	.	A	G	-	-+	0.35714286	18,0,10,0	chr2,206982	Cytosol	A > G
chr2:206982323:T:C:ncRNA_intror	chr2	206982323	206982323	T	C	ncRNA_intro	AC007383.4	.	.	.	A	G	-	-+	0.39130435	14,0,9,0	chr2,206982	Cytosol	A > G
chr2:206982334:T:C:ncRNA_intror	chr2	206982334	206982334	T	C	ncRNA_intro	AC007383.4	.	.	.	A	G	-	-+	0.08333333	22,0,2,0	chr2,206982	Cytosol	A > G
chr2:206982338:T:C:ncRNA_intror	chr2	206982338	206982338	T	C	ncRNA_intro	AC007383.4	.	.	.	A	G	-	-+	0.26086957	17,0,6,0	chr2,206982	Cytosol	A > G
chr2:206982341:T:C:ncRNA_intror	chr2	206982341	206982341	T	C	ncRNA_intro	AC007383.4	.	.	.	A	G	-	-+	0.40909091	13,0,9,0	chr2,206982	Cytosol	A > G
chr2:206982358:T:C:ncRNA_intror	chr2	206982358	206982358	T	C	ncRNA_intro	AC007383.4	.	.	.	A	G	-	-+	0.83333333	5,0,25,0	chr2,206982	Cytosol	A > G
chr2:206982377:T:C:ncRNA_intror	chr2	206982377	206982377	T	C	ncRNA_intro	AC007383.4	.	.	.	A	G	-	-+	0.1875	26,0,6,0	chr2,206982	Cytosol	A > G
chr2:206982392:T:C:ncRNA_intror	chr2	206982392	206982392	T	C	ncRNA_intro	AC007383.4	.	.	.	A	G	-	-+	0.24137931	22,0,7,0	chr2,206982	Cytosol	A > G
chr2:206983093:T:C:ncRNA_intror	chr2	206983093	206983093	T	C	ncRNA_intro	AC007383.4	.	.	.	A	G	-	-+	0.20833333	19,0,5,0	chr2,206983	Cytosol	A > G
chr2:206983115:T:C:ncRNA_intror	chr2	206983115	206983115	T	C	ncRNA_intro	AC007383.4	.	.	.	A	G	-	-+	0.42307692	15,0,11,0	chr2,206983	Cytosol	A > G
chr2:206983132:T:C:ncRNA_intror	chr2	206983132	206983132	T	C	ncRNA_intro	AC007383.4	.	.	.	A	G	-	-+	0.37037037	16,0,10,1	chr2,206983	Cytosol	A > G
chr2:206983138:T:C:ncRNA_intror	chr2	206983138	206983138	T	C	ncRNA_intro	AC007383.4	.	.	.	A	G	-	-+	0.14285714	24,0,4,0	chr2,206983	Cytosol	A > G
chr2:206983162:T:C:ncRNA_intror	chr2	206983162	206983162	T	C	ncRNA_intro	AC007383.4	.	.	.	A	G	-	-+	0.15625	27,0,5,0	chr2,206983	Cytosol	A > G
chr2:206983193:T:C:ncRNA_intror	chr2	206983193	206983193	T	C	ncRNA_intro	AC007383.4	.	.	.	A	G	-	-+	0.0877193	52,0,5,0	chr2,206983	Cytosol	A > G
chr2:206983201:T:C:ncRNA_intror	chr2	206983201	206983201	T	C	ncRNA_intro	AC007383.4	.	.	.	A	G	-	-+	0.11111111	48,0,6,0	chr2,206983	Cytosol	A > G
chr2:206983441:T:C:ncRNA_intror	chr2	206983441	206983441	T	C	ncRNA_intro	AC007383.4	.	.	.	A	G	-	+-	0.35294118	11,0,6,0	chr2,206983	Cytosol	A > G
chr2:206983491:T:C:ncRNA_intror	chr2	206983491	206983491	T	C	ncRNA_intro	AC007383.4	.	.	.	A	G	-	+-	0.61538462	5,0,8,0	chr2,206983	Cytosol	A > G
chr2:206983510:T:C:ncRNA_intror	chr2	206983510	206983510	T	C	ncRNA_intro	AC007383.4	.	.	.	A	G	-	+-	0.29411765	12,0,5,0	chr2,206983	Cytosol	A > G
chr2:206983513:T:C:ncRNA_intror	chr2	206983513	206983513	T	C	ncRNA_intro	AC007383.4	.	.	.	A	G	-	+-	0.38888889	11,0,7,0	chr2,206983	Cytosol	A > G
chr2:206983532:T:C:ncRNA_intror	chr2	206983532	206983532	T	C	ncRNA_intro	AC007383.4	.	.	.	A	G	-	+-	0.42105263	11,0,8,0	chr2,206983	Cytosol	A > G
chr2:206983555:T:C:ncRNA_intror	chr2	206983555	206983555	T	C	ncRNA_intro	AC007383.4	.	.	.	A	G	-	+-	0.13636364	18,1,3,0	chr2,206983	Cytosol	A > G
chr2:206983576:T:C:ncRNA_intror	chr2	206983576	206983576	T	C	ncRNA_intro	AC007383.4	.	.	.	A	G	-	+-	0.10526316	17,0,2,0	chr2,206983	Cytosol	A > G
chr2:206983577:T:C:ncRNA_intror	chr2	206983577	206983577	T	C	ncRNA_intro	AC007383.4	.	.	.	A	G	-	+-	0.3	14,0,6,0	chr2,206983	Cytosol	A > G
chr2:206983586:T:C:ncRNA_intror	chr2	206983586	206983586	T	C	ncRNA_intro	AC007383.4	.	.	.	A	G	-	+-	0.3125	11,0,5,0	chr2,206983	Cytosol	A > G
chr2:206983608:T:C:ncRNA_intror	chr2	206983608	206983608	T	C	ncRNA_intro	AC007383.4	.	.	.	A	G	-	+-	0.16666667	10,0,2,0	chr2,206983	Cytosol	A > G
chr2:206983646:T:C:ncRNA_intror	chr2	206983646	206983646	T	C	ncRNA_intro	AC007383.4	.	.	.	A	G	-	+-	0.18181818	9,0,2,0	chr2,206983	Cytosol	A > G
chr2:206983649:T:C:ncRNA_intror	chr2	206983649	206983649	T	C	ncRNA_intro	AC007383.4	.	.	.	A	G	-	+-	0.33333333	8,0,4,0	chr2,206983	Cytosol	A > G
chr2:206983668:T:C:ncRNA_intror	chr2	206983668	206983668	T	C	ncRNA_intro	AC007383.4	.	.	.	A	G	-	+-	0.15384615	11,0,2,0	chr2,206983	Cytosol	A > G
chr2:206983688:T:C:ncRNA_intror	chr2	206983688	206983688	T	C	ncRNA_intro	AC007383.4	.	.	.	A	G	-	+-	0.25	12,0,4,0	chr2,206983	Cytosol	A > G
chr2:206983690:T:C:ncRNA_intror	chr2	206983690	206983690	T	C	ncRNA_intro	AC007383.4	.	.	.	A	G	-	+-	0.3125	11,0,5,0	chr2,206983	Cytosol	A > G
chr2:206983712:T:C:ncRNA_intror	chr2	206983712	206983712	T	C	ncRNA_intro	AC007383.4	.	.	.	A	G	-	+-	0.5	10,0,10,0	chr2,206983	Cytosol	A > G
chr2:206983729:T:C:ncRNA_intror	chr2	206983729	206983729	T	C	ncRNA_intro	AC007383.4	.	.	.	A	G	-	+-	0.70967742	9,0,22,0	chr2,206983	Cytosol	A > G
chr2:206983730:T:C:ncRNA_intror	chr2	206983730	206983730	T	C	ncRNA_intro	AC007383.4	.	.	.	A	G	-	+-	0.90322581	3,0,28,0	chr2,206983	Cytosol	A > G
chr2:206983743:T:C:ncRNA_intror	chr2	206983743	206983743	T	C	ncRNA_intro	AC007383.4	.	.	.	A	G	-	+-	0.06451613	29,0,2,0	chr2,206983	Cytosol	A > G
chr2:206983749:T:C:ncRNA_intror	chr2	206983749	206983749	T	C	ncRNA_intro	AC007383.4	.	.	.	A	G	-	+-	0.15151515	28,0,5,0	chr2,206983	Cytosol	A > G
chr2:206983755:T:C:ncRNA_intror	chr2	206983755	206983755	T	C	ncRNA_intro	AC007383.4	.	.	.	A	G	-	+-	0.08823529	31,0,3,0	chr2,206983	Cytosol	A > G
chr2:206983760:T:C:ncRNA_intror	chr2	206983760	206983760	T	C	ncRNA_intro	AC007383.4	.	.	.	A	G	-	+-	0.44444444	20,0,16,0	chr2,206983	Cytosol	A > G
chr2:206983765:T:C:ncRNA_intror	chr2	206983765	206983765	T	C	ncRNA_intro	AC007383.4	.	.	.	A	G	-	+-	0.37837838	23,0,14,0	chr2,206983	Cytosol	A > G
chr2:206983766:T:C:ncRNA_intror	chr2	206983766	206983766	T	C	ncRNA_intro	AC007383.4	.	.	.	A	G	-	+-	0.13513514	32,0,5,0	chr2,206983	Cytosol	A > G
chr2:206983797:T:C:ncRNA_intror	chr2	206983797	206983797	T	C	ncRNA_intro	AC007383.4	.	.	.	A	G	-	+-	0.32142857	19,0,9,0	chr2,206983	Cytosol	A > G
chr2:206984298:T:C:ncRNA_intror	chr2	206984298	206984298	T	C	ncRNA_intro	AC007383.4	.	.	.	A	G	-	-+	0.18181818	9,0,2,0	chr2,206984	Cytosol	A > G
chr2:206984411:T:C:ncRNA_intror	chr2	206984411	206984411	T	C	ncRNA_intro	AC007383.4	.	.	.	A	G	-	-+	0.42857143	12,0,9,0	chr2,206984	Cytosol	A > G
chr2:206984412:T:C:ncRNA_intror	chr2	206984412	206984412	T	C	ncRNA_intro	AC007383.4	.	.	.	A	G	-	-+	0.55	9,0,11,0	chr2,206984	Cytosol	A > G
chr2:206984425:T:C:ncRNA_intror	chr2	206984425	206984425	T	C	ncRNA_intro	AC007383.4	.	.	.	A	G	-	-+	0.93333333	1,0,14,0	chr2,206984	Cytosol	A > G
chr2:206985625:T:C:ncRNA_intror	chr2	206985625	206985625	T	C	ncRNA_intro	AC007383.4	.	.	.	A	G	-	-+	0.075	37,0,3,0	chr2,206985	Cytosol	A > G
chr2:206986314:T:C:ncRNA_intror	chr2	206986314	206986314	T	C	ncRNA_intro	AC007383.4	.	.	.	A	G	-	-+	0.4516129	17,0,14,0	chr2,206986	Cytosol	A > G
chr2:206986341:T:C:ncRNA_intror	chr2	206986341	206986341	T	C	ncRNA_intro	AC007383.4	.	.	.	A	G	-	-+	0.6	8,0,12,0	chr2,206986	Cytosol	A > G
chr2:206986350:T:C:ncRNA_intror	chr2	206986350	206986350	T	C	ncRNA_intro	AC007383.4	.	.	.	A	G	-	-+	0.40909091	13,0,9,0	chr2,206986	Cytosol	A > G
chr2:206986356:T:C:ncRNA_intror	chr2	206986356	206986356	T	C	ncRNA_intro	AC007383.4	.	.	.	A	G	-	-+	0.66666667	6,0,12,0	chr2,206986	Cytosol	A > G
chr2:206986359:T:C:ncRNA_intror	chr2	206986359	206986359	T	C	ncRNA_intro	AC007383.4	.	.	.	A	G	-	-+	0.43478261	13,0,10,0	chr2,206986	Cytosol	A > G
chr2:206987108:T:C:UTR3:NDUFS	chr2	206987108	206987108	T	C	UTR3	NDUFS1	ENST000002	.	.	A	G	-	-	0.74418605	22,0,64,0	chr2,206987	Cytosol	A > G
chr2:206987228:T:C:UTR3:NDUFS	chr2	206987228	206987228	T	C	UTR3	NDUFS1	ENST000002	.	.	A	G	-	-	0.85981308	15,0,92,0	chr2,206987	Cytosol	A > G
chr2:206987229:T:C:UTR3:NDUFS	chr2	206987229	206987229	T	C	UTR3	NDUFS1	ENST000002	.	.	A	G	-	-	0.42727273	63,0,47,0	chr2,206987	Cytosol	A > G
chr2:206987231:T:C:UTR3:NDUFS	chr2	206987231	206987231	T	C	UTR3	NDUFS1	ENST000002	.	.	A	G	-	-	0.34862385	71,0,38,0	chr2,206987	Cytosol	A > G

chr2:206987253:T:C:UTR3:NDUFS	chr2	206987253	206987253	T	C	UTR3	NDUFS1	ENST000002	.	.	A	G	-	-	0.28455285	88,0,35,0	chr2,206987	Cytosol	A > G
chr2:206987270:T:C:UTR3:NDUFS	chr2	206987270	206987270	T	C	UTR3	NDUFS1	ENST000002	.	.	A	G	-	-	0.74637681	35,0,103,0	chr2,206987	Cytosol	A > G
chr2:206987271:T:C:UTR3:NDUFS	chr2	206987271	206987271	T	C	UTR3	NDUFS1	ENST000002	.	.	A	G	-	-	0.15384615	121,0,22,0	chr2,206987	Cytosol	A > G
chr2:206987309:T:C:UTR3:NDUFS	chr2	206987309	206987309	T	C	UTR3	NDUFS1	ENST000002	.	.	A	G	-	-	0.13913043	99,0,16,0	chr2,206987	Cytosol	A > G
chr2:206987315:T:C:UTR3:NDUFS	chr2	206987315	206987315	T	C	UTR3	NDUFS1	ENST000002	.	.	A	G	-	-	0.11428571	93,0,12,0	chr2,206987	Cytosol	A > G
chr2:206987319:T:C:UTR3:NDUFS	chr2	206987319	206987319	T	C	UTR3	NDUFS1	ENST000002	.	.	A	G	-	-	0.17821782	83,0,18,0	chr2,206987	Cytosol	A > G
chr2:206987340:T:C:UTR3:NDUFS	chr2	206987340	206987340	T	C	UTR3	NDUFS1	ENST000002	.	.	A	G	-	-	0.8015873	25,0,101,0	chr2,206987	Cytosol	A > G
chr2:206987536:T:C:UTR3:NDUFS	chr2	206987536	206987536	T	C	UTR3	NDUFS1	ENST000002	.	.	A	G	-	-	0.024	122,0,3,0	chr2,206987	Cytosol	A > G
chr2:206987561:T:C:UTR3:NDUFS	chr2	206987561	206987561	T	C	UTR3	NDUFS1	ENST000002	.	.	A	G	-	-	0.4673913	49,0,43,0	chr2,206987	Cytosol	A > G
chr2:206987597:T:C:UTR3:NDUFS	chr2	206987597	206987597	T	C	UTR3	NDUFS1	ENST000002	.	.	A	G	-	-	0.42028986	40,0,29,0	chr2,206987	Cytosol	A > G
chr2:206987599:T:C:UTR3:NDUFS	chr2	206987599	206987599	T	C	UTR3	NDUFS1	ENST000002	.	.	A	G	-	-	0.68115942	22,0,47,0	chr2,206987	Cytosol	A > G
chr2:206987605:T:C:UTR3:NDUFS	chr2	206987605	206987605	T	C	UTR3	NDUFS1	ENST000002	.	.	A	G	-	-	0.48484848	34,0,32,0	chr2,206987	Cytosol	A > G
chr2:206987626:T:C:UTR3:NDUFS	chr2	206987626	206987626	T	C	UTR3	NDUFS1	ENST000002	.	.	A	G	-	-	0.125	49,0,7,0	chr2,206987	Cytosol	A > G
chr2:206987627:T:C:UTR3:NDUFS	chr2	206987627	206987627	T	C	UTR3	NDUFS1	ENST000002	.	.	A	G	-	-	0.10169492	53,0,6,0	chr2,206987	Cytosol	A > G
chr2:206987628:T:C:UTR3:NDUFS	chr2	206987628	206987628	T	C	UTR3	NDUFS1	ENST000002	.	.	A	G	-	-	0.08196721	56,0,5,0	chr2,206987	Cytosol	A > G
chr2:206987629:T:C:UTR3:NDUFS	chr2	206987629	206987629	T	C	UTR3	NDUFS1	ENST000002	.	.	A	G	-	-	0.37037037	34,0,20,0	chr2,206987	Cytosol	A > G
chr2:206987632:T:C:UTR3:NDUFS	chr2	206987632	206987632	T	C	UTR3	NDUFS1	ENST000002	.	.	A	G	-	-	0.43636364	31,0,24,0	chr2,206987	Cytosol	A > G
chr2:206987634:T:C:UTR3:NDUFS	chr2	206987634	206987634	T	C	UTR3	NDUFS1	ENST000002	.	.	A	G	-	-	0.33928571	37,0,19,0	chr2,206987	Cytosol	A > G
chr2:206987635:T:C:UTR3:NDUFS	chr2	206987635	206987635	T	C	UTR3	NDUFS1	ENST000002	.	.	A	G	-	-	0.07407407	50,0,4,0	chr2,206987	Cytosol	A > G
chr2:206987637:T:C:UTR3:NDUFS	chr2	206987637	206987637	T	C	UTR3	NDUFS1	ENST000002	.	.	A	G	-	-	0.27777778	39,0,15,0	chr2,206987	Cytosol	A > G
chr2:206987638:T:C:UTR3:NDUFS	chr2	206987638	206987638	T	C	UTR3	NDUFS1	ENST000002	.	.	A	G	-	-	0.125	49,0,7,0	chr2,206987	Cytosol	A > G
chr2:206987641:T:C:UTR3:NDUFS	chr2	206987641	206987641	T	C	UTR3	NDUFS1	ENST000002	.	.	A	G	-	-	0.15151515	56,0,10,0	chr2,206987	Cytosol	A > G
chr2:206987652:T:C:UTR3:NDUFS	chr2	206987652	206987652	T	C	UTR3	NDUFS1	ENST000002	.	.	A	G	-	-	0.43137255	29,0,22,0	chr2,206987	Cytosol	A > G
chr2:206987725:T:C:UTR3:NDUFS	chr2	206987725	206987725	T	C	UTR3	NDUFS1	ENST000002	.	.	A	G	-	-	0.30927835	67,0,30,0	chr2,206987	Cytosol	A > G
chr2:206987731:T:C:UTR3:NDUFS	chr2	206987731	206987731	T	C	UTR3	NDUFS1	ENST000002	.	.	A	G	-	-	0.17272727	91,0,19,0	chr2,206987	Cytosol	A > G
chr2:206987732:T:C:UTR3:NDUFS	chr2	206987732	206987732	T	C	UTR3	NDUFS1	ENST000002	.	.	A	G	-	-	0.1509434	90,0,16,0	chr2,206987	Cytosol	A > G
chr2:206987738:T:C:UTR3:NDUFS	chr2	206987738	206987738	T	C	UTR3	NDUFS1	ENST000002	.	.	A	G	-	-	0.13684211	82,0,13,0	chr2,206987	Cytosol	A > G
chr2:208476909:T:C:UTR3:METTL	chr2	208476909	208476909	T	C	UTR3	METTL21A	ENST000004	.	.	A	G	-	-	0.125	14,0,2,0	chr2,208476	Cytosol	A > G
chr2:208476948:T:C:UTR3:METTL	chr2	208476948	208476948	T	C	UTR3	METTL21A	ENST000004	.	.	A	G	-	-	0.09090909	20,0,2,0	chr2,208476	Cytosol	A > G
chr2:208477105:T:C:UTR3:METTL	chr2	208477105	208477105	T	C	UTR3	METTL21A	ENST000004	.	.	A	G	-	-	0.05263158	36,0,2,0	chr2,208477	Cytosol	A > G
chr2:208619377:A:G:UTR3:CCNYL	chr2	208619377	208619377	A	G	UTR3	CCNYL1	ENST000002	.	.	A	G	+	+	0.13513514	32,0,5,0	chr2,208619	Cytosol	A > G
chr2:208619461:A:G:UTR3:CCNYL	chr2	208619461	208619461	A	G	UTR3	CCNYL1	ENST000002	.	.	A	G	+	+	0.06060606	31,0,2,0	chr2,208619	Cytosol	A > G
chr2:208619479:A:G:UTR3:CCNYL	chr2	208619479	208619479	A	G	UTR3	CCNYL1	ENST000002	.	.	A	G	+	+	0.67647059	11,0,23,0	chr2,208619	Cytosol	A > G
chr2:208619491:A:G:UTR3:CCNYL	chr2	208619491	208619491	A	G	UTR3	CCNYL1	ENST000002	.	.	A	G	+	+	0.21621622	29,0,8,0	chr2,208619	Cytosol	A > G
chr2:208619585:A:G:ncRNA_exon	chr2	208619585	208619585	A	G	ncRNA_exon	MIR4775	.	.	.	A	G	+	+	0.32142857	19,0,9,0	chr2,208619	Cytosol	A > G
chr2:208619650:A:G:UTR3:CCNYL	chr2	208619650	208619650	A	G	UTR3	CCNYL1	ENST000002	.	.	A	G	+	+	0.36363636	7,0,4,0	chr2,208619	Cytosol	A > G
chr2:208619675:A:G:UTR3:CCNYL	chr2	208619675	208619675	A	G	UTR3	CCNYL1	ENST000002	.	.	A	G	+	+	0.25	6,0,2,0	chr2,208619	Cytosol	A > G
chr2:208619742:A:G:UTR3:CCNYL	chr2	208619742	208619742	A	G	UTR3	CCNYL1	ENST000002	.	.	A	G	+	+	0.44444444	15,0,12,0	chr2,208619	Cytosol	A > G
chr2:208619749:A:G:UTR3:CCNYL	chr2	208619749	208619749	A	G	UTR3	CCNYL1	ENST000002	.	.	A	G	+	+	0.57575758	14,0,19,0	chr2,208619	Cytosol	A > G
chr2:216808308:T:C:downstream	chr2	216808308	216808308	T	C	downstream	MREG	.	.	.	A	G	-	-	0.5	13,0,13,0	chr2,216808	Cytosol	A > G
chr2:216808320:T:C:downstream	chr2	216808320	216808320	T	C	downstream	MREG	.	.	.	A	G	-	-	0.53846154	12,0,14,0	chr2,216808	Cytosol	A > G
chr2:216808321:T:C:downstream	chr2	216808321	216808321	T	C	downstream	MREG	.	.	.	A	G	-	-	0.5	12,0,12,0	chr2,216808	Cytosol	A > G
chr2:216808330:T:C:downstream	chr2	216808330	216808330	T	C	downstream	MREG	.	.	.	A	G	-	-	0.22222222	14,0,4,0	chr2,216808	Cytosol	A > G
chr2:216808375:T:C:downstream	chr2	216808375	216808375	T	C	downstream	MREG	.	.	.	A	G	-	-	0.9047619	2,0,19,0	chr2,216808	Cytosol	A > G
chr2:216808400:T:C:downstream	chr2	216808400	216808400	T	C	downstream	MREG	.	.	.	A	G	-	-	0.45454545	12,0,10,0	chr2,216808	Cytosol	A > G
chr2:216808406:T:C:downstream	chr2	216808406	216808406	T	C	downstream	MREG	.	.	.	A	G	-	-	0.22727273	17,0,5,0	chr2,216808	Cytosol	A > G
chr2:216808416:T:C:downstream	chr2	216808416	216808416	T	C	downstream	MREG	.	.	.	A	G	-	-	0.11538462	23,0,3,0	chr2,216808	Cytosol	A > G
chr2:216808440:T:C:downstream	chr2	216808440	216808440	T	C	downstream	MREG	.	.	.	A	G	-	-	0.15	17,0,3,0	chr2,216808	Cytosol	A > G
chr2:216808442:T:C:downstream	chr2	216808442	216808442	T	C	downstream	MREG	.	.	.	A	G	-	-	0.85714286	3,0,18,0	chr2,216808	Cytosol	A > G
chr2:216808452:T:C:downstream	chr2	216808452	216808452	T	C	downstream	MREG	.	.	.	A	G	-	-	0.5	7,0,7,0	chr2,216808	Cytosol	A > G
chr2:216808840:T:C:downstream	chr2	216808840	216808840	T	C	downstream	MREG	.	.	.	A	G	-	-	0.375	5,0,3,0	chr2,216808	Cytosol	A > G
chr2:216808841:T:C:downstream	chr2	216808841	216808841	T	C	downstream	MREG	.	.	.	A	G	-	-	0.25	6,0,2,0	chr2,216808	Cytosol	A > G
chr2:216808863:T:C:downstream	chr2	216808863	216808863	T	C	downstream	MREG	.	.	.	A	G	-	-	0.16666667	10,0,2,0	chr2,216808	Cytosol	A > G
chr2:216808867:T:C:downstream	chr2	216808867	216808867	T	C	downstream	MREG	.	.	.	A	G	-	-	0.78571429	3,0,11,0	chr2,216808	Cytosol	A > G
chr2:216808873:T:C:downstream	chr2	216808873	216808873	T	C	downstream	MREG	.	.	.	A	G	-	-	0.15384615	11,0,2,0	chr2,216808	Cytosol	A > G
chr2:216808880:T:C:downstream	chr2	216808880	216808880	T	C	downstream	MREG	.	.	.	A	G	-	-	0.25	9,0,3,0	chr2,216808	Cytosol	A > G
chr2:216808881:T:C:downstream	chr2	216808881	216808881	T	C	downstream	MREG	.	.	.	A	G	-	-	0.25	9,0,3,0	chr2,216808	Cytosol	A > G
chr2:216808906:T:C:downstream	chr2	216808906	216808906	T	C	downstream	MREG	.	.	.	A	G	-	-	0.53846154	6,0,7,0	chr2,216808	Cytosol	A > G
chr2:216808907:T:C:downstream	chr2	216808907	216808907	T	C	downstream	MREG	.	.	.	A	G	-	-	0.33333333	8,0,4,0	chr2,216808	Cytosol	A > G
chr2:216808951:T:C:downstream	chr2	216808951	216808951	T	C	downstream	MREG	.	.	.	A	G	-	-	0.30769231	9,0,4,0	chr2,216808	Cytosol	A > G
chr2:216808952:T:C:downstream	chr2	216808952	216808952	T	C	downstream	MREG	.	.	.	A	G	-	-	0.15384615	11,0,2,0	chr2,216808	Cytosol	A > G
chr2:216808974:T:C:downstream	chr2	216808974	216808974	T	C	downstream	MREG	.	.	.	A	G	-	-	0.58333333	5,0,7,0	chr2,216808	Cytosol	A > G
chr2:216808982:T:C:downstream	chr2	216808982	216808982	T	C	downstream	MREG	.	.	.	A	G	-	-	0.23529412	13,0,4,0	chr2,216808	Cytosol	A > G
chr2:216903419:T:C:UTR3:PECR	chr2	216903419	216903419	T	C	UTR3	PECR	ENST000002	.	.	A	G	-	-	0.04651163	41,0,2,0	chr2,216903	Cytosol	A > G

chr2:216903426:T:C:UTR3:PECR	chr2	216903426	216903426	T	C	UTR3	PECR	ENST000002	.	.	A	G	-	-	0.06521739	43,0,3,0	chr2,216903	Cytosol	A > G
chr2:216903472:T:C:UTR3:PECR	chr2	216903472	216903472	T	C	UTR3	PECR	ENST000002	.	.	A	G	-	-	0.08988764	81,0,8,0	chr2,216903	Cytosol	A > G
chr2:216903482:T:C:UTR3:PECR	chr2	216903482	216903482	T	C	UTR3	PECR	ENST000002	.	.	A	G	-	-	0.05813953	81,0,5,0	chr2,216903	Cytosol	A > G
chr2:216903498:T:C:UTR3:PECR	chr2	216903498	216903498	T	C	UTR3	PECR	ENST000002	.	.	A	G	-	-	0.06060606	93,0,6,0	chr2,216903	Cytosol	A > G
chr2:216903544:T:C:UTR3:PECR	chr2	216903544	216903544	T	C	UTR3	PECR	ENST000002	.	.	A	G	-	-	0.1047619	94,0,11,0	chr2,216903	Cytosol	A > G
chr2:216903551:T:C:UTR3:PECR	chr2	216903551	216903551	T	C	UTR3	PECR	ENST000002	.	.	A	G	-	-	0.04166667	92,0,4,0	chr2,216903	Cytosol	A > G
chr2:216903561:T:C:UTR3:PECR	chr2	216903561	216903561	T	C	UTR3	PECR	ENST000002	.	.	A	G	-	-	0.05050505	94,0,5,0	chr2,216903	Cytosol	A > G
chr2:216903620:T:C:UTR3:PECR	chr2	216903620	216903620	T	C	UTR3	PECR	ENST000002	.	.	A	G	-	-	0.03448276	56,0,2,0	chr2,216903	Cytosol	A > G
chr2:216903621:T:C:UTR3:PECR	chr2	216903621	216903621	T	C	UTR3	PECR	ENST000002	.	.	A	G	-	-	0.03389831	57,0,2,0	chr2,216903	Cytosol	A > G
chr2:217366480:A:G:UTR3:RPL37A	chr2	217366480	217366480	A	G	UTR3	RPL37A	ENST000004	.	.	A	G	+	+	0.01086957	182,0,2,0	chr2,217366	Cytosol	A > G
chr2:217366496:A:G:UTR3:RPL37A	chr2	217366496	217366496	A	G	UTR3	RPL37A	ENST000004	.	.	A	G	+	+	0.0308642	156,0,5,1	chr2,217366	Cytosol	A > G
chr2:217366499:A:G:UTR3:RPL37A	chr2	217366499	217366499	A	G	UTR3	RPL37A	ENST000004	.	.	A	G	+	+	0.23404255	144,0,44,0	chr2,217366	Cytosol	A > G
chr2:217366515:A:G:UTR3:RPL37A	chr2	217366515	217366515	A	G	UTR3	RPL37A	ENST000004	.	.	A	G	+	+	0.48186528	100,0,93,0	chr2,217366	Cytosol	A > G
chr2:217366521:A:G:UTR3:RPL37A	chr2	217366521	217366521	A	G	UTR3	RPL37A	ENST000004	.	.	A	G	+	+	0.13772455	144,0,23,0	chr2,217366	Cytosol	A > G
chr2:217366540:A:G:UTR3:RPL37A	chr2	217366540	217366540	A	G	UTR3	RPL37A	ENST000004	.	.	A	G	+	+	0.19371728	154,0,37,0	chr2,217366	Cytosol	A > G
chr2:217366541:A:G:UTR3:RPL37A	chr2	217366541	217366541	A	G	UTR3	RPL37A	ENST000004	.	.	A	G	+	+	0.37349398	104,0,62,0	chr2,217366	Cytosol	A > G
chr2:217366570:A:G:UTR3:RPL37A	chr2	217366570	217366570	A	G	UTR3	RPL37A	ENST000004	.	.	A	G	+	+	0.42207792	89,0,65,0	chr2,217366	Cytosol	A > G
chr2:217366580:A:G:UTR3:RPL37A	chr2	217366580	217366580	A	G	UTR3	RPL37A	ENST000004	.	.	A	G	+	+	0.88435374	17,0,130,0	chr2,217366	Cytosol	A > G
chr2:217366590:A:G:UTR3:RPL37A	chr2	217366590	217366590	A	G	UTR3	RPL37A	ENST000004	.	.	A	G	+	+	0.53642384	70,0,81,0	chr2,217366	Cytosol	A > G
chr2:217366606:A:G:UTR3:RPL37A	chr2	217366606	217366606	A	G	UTR3	RPL37A	ENST000004	.	.	A	G	+	+	0.11510791	123,0,16,0	chr2,217366	Cytosol	A > G
chr2:217366607:A:G:UTR3:RPL37A	chr2	217366607	217366607	A	G	UTR3	RPL37A	ENST000004	.	.	A	G	+	+	0.0141844	139,0,2,0	chr2,217366	Cytosol	A > G
chr2:217366615:A:G:UTR3:RPL37A	chr2	217366615	217366615	A	G	UTR3	RPL37A	ENST000004	.	.	A	G	+	+	0.1503268	130,0,23,0	chr2,217366	Cytosol	A > G
chr2:217366624:A:G:UTR3:RPL37A	chr2	217366624	217366624	A	G	UTR3	RPL37A	ENST000004	.	.	A	G	+	+	0.15483871	131,0,24,0	chr2,217366	Cytosol	A > G
chr2:217366626:A:G:UTR3:RPL37A	chr2	217366626	217366626	A	G	UTR3	RPL37A	ENST000004	.	.	A	G	+	+	0.11578947	168,0,22,0	chr2,217366	Cytosol	A > G
chr2:217366639:A:G:UTR3:RPL37A	chr2	217366639	217366639	A	G	UTR3	RPL37A	ENST000004	.	.	A	G	+	+	0.065	187,0,13,0	chr2,217366	Cytosol	A > G
chr2:217366642:A:G:UTR3:RPL37A	chr2	217366642	217366642	A	G	UTR3	RPL37A	ENST000004	.	.	A	G	+	+	0.05527638	187,0,11,1	chr2,217366	Cytosol	A > G
chr2:217366644:A:G:UTR3:RPL37A	chr2	217366644	217366644	A	G	UTR3	RPL37A	ENST000004	.	.	A	G	+	+	0.30769231	135,0,60,0	chr2,217366	Cytosol	A > G
chr2:217366673:A:G:UTR3:RPL37A	chr2	217366673	217366673	A	G	UTR3	RPL37A	ENST000004	.	.	A	G	+	+	0.16	126,0,24,0	chr2,217366	Cytosol	A > G
chr2:217366674:A:G:UTR3:RPL37A	chr2	217366674	217366674	A	G	UTR3	RPL37A	ENST000004	.	.	A	G	+	+	0.15172414	123,0,22,0	chr2,217366	Cytosol	A > G
chr2:217366699:A:G:UTR3:RPL37A	chr2	217366699	217366699	A	G	UTR3	RPL37A	ENST000004	.	.	A	G	+	+	0.43283582	37,1,29,0	chr2,217366	Cytosol	A > G
chr2:217366713:A:G:UTR3:RPL37A	chr2	217366713	217366713	A	G	UTR3	RPL37A	ENST000004	.	.	A	G	+	+	0.88	6,0,44,0	chr2,217366	Cytosol	A > G
chr2:217366722:A:G:UTR3:RPL37A	chr2	217366722	217366722	A	G	UTR3	RPL37A	ENST000004	.	.	A	G	+	+	0.26190476	31,0,11,0	chr2,217366	Cytosol	A > G
chr2:217366729:A:G:UTR3:RPL37A	chr2	217366729	217366729	A	G	UTR3	RPL37A	ENST000004	.	.	A	G	+	+	0.08823529	31,0,3,0	chr2,217366	Cytosol	A > G
chr2:217366786:A:G:UTR3:RPL37A	chr2	217366786	217366786	A	G	UTR3	RPL37A	ENST000004	.	.	A	G	+	+	0.171875	53,0,11,0	chr2,217366	Cytosol	A > G
chr2:217366800:A:G:UTR3:RPL37A	chr2	217366800	217366800	A	G	UTR3	RPL37A	ENST000004	.	.	A	G	+	+	0.03896104	74,0,3,0	chr2,217366	Cytosol	A > G
chr2:217366803:A:G:UTR3:RPL37A	chr2	217366803	217366803	A	G	UTR3	RPL37A	ENST000004	.	.	A	G	+	+	0.17808219	60,0,13,0	chr2,217366	Cytosol	A > G
chr2:217366821:A:G:UTR3:RPL37A	chr2	217366821	217366821	A	G	UTR3	RPL37A	ENST000004	.	.	A	G	+	+	0.15151515	84,0,15,0	chr2,217366	Cytosol	A > G
chr2:217366866:A:G:UTR3:RPL37A	chr2	217366866	217366866	A	G	UTR3	RPL37A	ENST000004	.	.	A	G	+	+	0.25477707	117,0,40,0	chr2,217366	Cytosol	A > G
chr2:217366868:A:G:UTR3:RPL37A	chr2	217366868	217366868	A	G	UTR3	RPL37A	ENST000004	.	.	A	G	+	+	0.38064516	96,0,59,0	chr2,217366	Cytosol	A > G
chr2:217366871:A:G:UTR3:RPL37A	chr2	217366871	217366871	A	G	UTR3	RPL37A	ENST000004	.	.	A	G	+	+	0.02564103	152,0,4,0	chr2,217366	Cytosol	A > G
chr2:217366874:A:G:UTR3:RPL37A	chr2	217366874	217366874	A	G	UTR3	RPL37A	ENST000004	.	.	A	G	+	+	0.09803922	138,0,15,0	chr2,217366	Cytosol	A > G
chr2:217366875:A:G:UTR3:RPL37A	chr2	217366875	217366875	A	G	UTR3	RPL37A	ENST000004	.	.	A	G	+	+	0.1171875	113,0,15,0	chr2,217366	Cytosol	A > G
chr2:217366885:A:G:UTR3:RPL37A	chr2	217366885	217366885	A	G	UTR3	RPL37A	ENST000004	.	.	A	G	+	+	0.07801418	130,0,11,0	chr2,217366	Cytosol	A > G
chr2:217366897:A:G:UTR3:RPL37A	chr2	217366897	217366897	A	G	UTR3	RPL37A	ENST000004	.	.	A	G	+	+	0.4015748	76,0,51,0	chr2,217366	Cytosol	A > G
chr2:217366898:A:G:UTR3:RPL37A	chr2	217366898	217366898	A	G	UTR3	RPL37A	ENST000004	.	.	A	G	+	+	0.234375	98,0,30,0	chr2,217366	Cytosol	A > G
chr2:217366900:A:G:UTR3:RPL37A	chr2	217366900	217366900	A	G	UTR3	RPL37A	ENST000004	.	.	A	G	+	+	0.18604651	105,0,24,0	chr2,217366	Cytosol	A > G
chr2:217366903:A:G:UTR3:RPL37A	chr2	217366903	217366903	A	G	UTR3	RPL37A	ENST000004	.	.	A	G	+	+	0.12698413	110,0,16,0	chr2,217366	Cytosol	A > G
chr2:217366929:A:G:UTR3:RPL37A	chr2	217366929	217366929	A	G	UTR3	RPL37A	ENST000004	.	.	A	G	+	+	0.37894737	59,0,36,0	chr2,217366	Cytosol	A > G
chr2:217366930:A:G:UTR3:RPL37A	chr2	217366930	217366930	A	G	UTR3	RPL37A	ENST000004	.	.	A	G	+	+	0.27472527	66,0,25,0	chr2,217366	Cytosol	A > G
chr2:217366935:A:G:UTR3:RPL37A	chr2	217366935	217366935	A	G	UTR3	RPL37A	ENST000004	.	.	A	G	+	+	0.02970297	98,0,3,0	chr2,217366	Cytosol	A > G
chr2:217366939:A:G:UTR3:RPL37A	chr2	217366939	217366939	A	G	UTR3	RPL37A	ENST000004	.	.	A	G	+	+	0.14018692	92,0,15,0	chr2,217366	Cytosol	A > G
chr2:217366958:A:G:UTR3:RPL37A	chr2	217366958	217366958	A	G	UTR3	RPL37A	ENST000004	.	.	A	G	+	+	0.05384615	122,0,7,1	chr2,217366	Cytosol	A > G
chr2:217366959:A:G:UTR3:RPL37A	chr2	217366959	217366959	A	G	UTR3	RPL37A	ENST000004	.	.	A	G	+	+	0.19565217	111,0,27,0	chr2,217366	Cytosol	A > G
chr2:217366965:A:G:UTR3:RPL37A	chr2	217366965	217366965	A	G	UTR3	RPL37A	ENST000004	.	.	A	G	+	+	0.22580645	120,0,35,0	chr2,217366	Cytosol	A > G
chr2:217366970:A:G:UTR3:RPL37A	chr2	217366970	217366970	A	G	UTR3	RPL37A	ENST000004	.	.	A	G	+	+	0.05294118	161,0,9,0	chr2,217366	Cytosol	A > G
chr2:217366971:A:G:UTR3:RPL37A	chr2	217366971	217366971	A	G	UTR3	RPL37A	ENST000004	.	.	A	G	+	+	0.14705882	145,0,25,0	chr2,217366	Cytosol	A > G
chr2:217366975:A:G:UTR3:RPL37A	chr2	217366975	217366975	A	G	UTR3	RPL37A	ENST000004	.	.	A	G	+	+	0.01081081	183,0,2,0	chr2,217366	Cytosol	A > G
chr2:217366978:A:G:UTR3:RPL37A	chr2	217366978	217366978	A	G	UTR3	RPL37A	ENST000004	.	.	A	G	+	+	0.04395604	174,0,8,0	chr2,217366	Cytosol	A > G
chr2:219499939:T:C:intronic:PLCD	chr2	219499939	219499939	T	C	intronic	PLCD4	.	.	.	A	G	-	+	0.94117647	1,0,16,0	chr2,219499	Cytosol	A > G
chr2:219500093:T:C:intronic:PLCD	chr2	219500093	219500093	T	C	intronic	PLCD4	.	.	.	A	G	-	+	0.14285714	18,0,3,0	chr2,219500	Cytosol	A > G
chr2:219500158:T:C:intronic:PLCD	chr2	219500158	219500158	T	C	intronic	PLCD4	.	.	.	A	G	-	+	0.38461538	8,0,5,0	chr2,219500	Cytosol	A > G
chr2:219500159:T:C:intronic:PLCD	chr2	219500159	219500159	T	C	intronic	PLCD4	.	.	.	A	G	-	+	0.70588235	5,0,12,0	chr2,219500	Cytosol	A > G
chr2:219500167:T:C:intronic:PLCD	chr2	219500167	219500167	T	C	intronic	PLCD4	.	.	.	A	G	-	+	0.41666667	7,0,5,0	chr2,219500	Cytosol	A > G
chr2:219500194:T:C:intronic:PLCD	chr2	219500194	219500194	T	C	intronic	PLCD4	.	.	.	A	G	-	+	0.4	9,0,6,0	chr2,219500	Cytosol	A > G

chr2:219500240:T:C:intronic:PLCD	chr2	219500240	219500240	T	C	intronic	PLCD4	.	.	.	A	G	-	+	0.25	9,0,3,0	chr2,219500:	Cytosol	A > G
chr2:219500241:T:C:intronic:PLCD	chr2	219500241	219500241	T	C	intronic	PLCD4	.	.	.	A	G	-	+	0.30769231	9,0,4,0	chr2,219500:	Cytosol	A > G
chr2:219500244:T:C:intronic:PLCD	chr2	219500244	219500244	T	C	intronic	PLCD4	.	.	.	A	G	-	+	0.6	4,0,6,0	chr2,219500:	Cytosol	A > G
chr2:219519729:T:C:intronic:ZNF1	chr2	219519729	219519729	T	C	intronic	ZNF142	.	.	.	A	G	-	-	0.42857143	4,0,3,0	chr2,219519	Cytosol	A > G
chr2:219940233:T:C:UTR3:NHEJ1	chr2	219940233	219940233	T	C	UTR3	NHEJ1	ENST000004(	.	.	A	G	-	-	0.06410256	73,0,5,0	chr2,219940:	Cytosol	A > G
chr2:219940239:T:C:UTR3:NHEJ1	chr2	219940239	219940239	T	C	UTR3	NHEJ1	ENST000004(	.	.	A	G	-	-	0.0375	77,0,3,0	chr2,219940:	Cytosol	A > G
chr2:220238178:T:C:downstream:	chr2	220238178	220238178	T	C	downstream	DNPEP	.	.	.	A	G	-	-	0.07407407	25,0,2,0	chr2,220238:	Cytosol	A > G
chr2:220238246:T:C:downstream:	chr2	220238246	220238246	T	C	downstream	DNPEP	.	.	.	A	G	-	-	0.11764706	15,0,2,0	chr2,220238:	Cytosol	A > G
chr2:224618710:T:C:intronic:AP1S	chr2	224618710	224618710	T	C	intronic	AP1S3	.	.	.	A	G	-	-	0.25	6,0,2,0	chr2,224618	Cytosol	A > G
chr2:224618725:T:C:intronic:AP1S	chr2	224618725	224618725	T	C	intronic	AP1S3	.	.	.	A	G	-	-	0.3	7,0,3,0	chr2,224618	Cytosol	A > G
chr2:224618737:T:C:intronic:AP1S	chr2	224618737	224618737	T	C	intronic	AP1S3	.	.	.	A	G	-	-	0.375	5,0,3,0	chr2,224618	Cytosol	A > G
chr2:224618738:T:C:intronic:AP1S	chr2	224618738	224618738	T	C	intronic	AP1S3	.	.	.	A	G	-	-	0.25	6,0,2,0	chr2,224618	Cytosol	A > G
chr2:224618813:T:C:intronic:AP1S	chr2	224618813	224618813	T	C	intronic	AP1S3	.	.	.	A	G	-	-	0.33333333	10,0,5,0	chr2,224618	Cytosol	A > G
chr2:224618819:T:C:intronic:AP1S	chr2	224618819	224618819	T	C	intronic	AP1S3	.	.	.	A	G	-	-	0.15384615	11,0,2,0	chr2,224618	Cytosol	A > G
chr2:224618846:T:C:intronic:AP1S	chr2	224618846	224618846	T	C	intronic	AP1S3	.	.	.	A	G	-	-	0.5	6,0,6,0	chr2,224618	Cytosol	A > G
chr2:224618859:T:C:intronic:AP1S	chr2	224618859	224618859	T	C	intronic	AP1S3	.	.	.	A	G	-	-	0.10526316	17,0,2,0	chr2,224618	Cytosol	A > G
chr2:224618924:T:C:intronic:AP1S	chr2	224618924	224618924	T	C	intronic	AP1S3	.	.	.	A	G	-	-	0.08695652	21,0,2,0	chr2,224618	Cytosol	A > G
chr2:224619461:T:C:intronic:AP1S	chr2	224619461	224619461	T	C	intronic	AP1S3	.	.	.	A	G	-	-	0.33333333	6,0,3,0	chr2,224619	Cytosol	A > G
chr2:224620372:T:C:intronic:AP1S	chr2	224620372	224620372	T	C	intronic	AP1S3	.	.	.	A	G	-	-	0.66666667	4,0,8,0	chr2,224620:	Cytosol	A > G
chr2:224620376:T:C:intronic:AP1S	chr2	224620376	224620376	T	C	intronic	AP1S3	.	.	.	A	G	-	-	0.61538462	5,0,8,0	chr2,224620:	Cytosol	A > G
chr2:224620412:T:C:intronic:AP1S	chr2	224620412	224620412	T	C	intronic	AP1S3	.	.	.	A	G	-	-	0.77777778	2,0,7,0	chr2,224620	Cytosol	A > G
chr2:224620423:T:C:intronic:AP1S	chr2	224620423	224620423	T	C	intronic	AP1S3	.	.	.	A	G	-	-	0.16666667	10,0,2,0	chr2,224620	Cytosol	A > G
chr2:224620424:T:C:intronic:AP1S	chr2	224620424	224620424	T	C	intronic	AP1S3	.	.	.	A	G	-	-	0.63636364	4,0,7,0	chr2,224620	Cytosol	A > G
chr2:224620490:T:C:intronic:AP1S	chr2	224620490	224620490	T	C	intronic	AP1S3	.	.	.	A	G	-	-	0.28571429	10,0,4,0	chr2,224620	Cytosol	A > G
chr2:224620519:T:C:intronic:AP1S	chr2	224620519	224620519	T	C	intronic	AP1S3	.	.	.	A	G	-	-	0.08695652	21,0,2,0	chr2,224620!	Cytosol	A > G
chr2:224620520:T:C:intronic:AP1S	chr2	224620520	224620520	T	C	intronic	AP1S3	.	.	.	A	G	-	-	0.16	21,0,4,0	chr2,224620!	Cytosol	A > G
chr2:224620793:T:C:intronic:AP1S	chr2	224620793	224620793	T	C	intronic	AP1S3	.	.	.	A	G	-	-	0.04347826	44,0,2,0	chr2,224620	Cytosol	A > G
chr2:224620811:T:C:intronic:AP1S	chr2	224620811	224620811	T	C	intronic	AP1S3	.	.	.	A	G	-	-	0.12820513	34,0,5,0	chr2,224620	Cytosol	A > G
chr2:224620812:T:C:intronic:AP1S	chr2	224620812	224620812	T	C	intronic	AP1S3	.	.	.	A	G	-	-	0.1627907	36,0,7,0	chr2,224620	Cytosol	A > G
chr2:224620889:T:C:intronic:AP1S	chr2	224620889	224620889	T	C	intronic	AP1S3	.	.	.	A	G	-	-	0.25	9,0,3,0	chr2,224620	Cytosol	A > G
chr2:224620942:T:C:intronic:AP1S	chr2	224620942	224620942	T	C	intronic	AP1S3	.	.	.	A	G	-	-	0.7	3,0,7,0	chr2,224620!	Cytosol	A > G
chr2:224620970:T:C:intronic:AP1S	chr2	224620970	224620970	T	C	intronic	AP1S3	.	.	.	A	G	-	-	0.73333333	4,0,11,0	chr2,224620!	Cytosol	A > G
chr2:224620971:T:C:intronic:AP1S	chr2	224620971	224620971	T	C	intronic	AP1S3	.	.	.	A	G	-	-	0.625	6,0,10,0	chr2,224620!	Cytosol	A > G
chr2:224620972:T:C:intronic:AP1S	chr2	224620972	224620972	T	C	intronic	AP1S3	.	.	.	A	G	-	-	0.88235294	2,0,15,0	chr2,224620!	Cytosol	A > G
chr2:224620992:T:C:intronic:AP1S	chr2	224620992	224620992	T	C	intronic	AP1S3	.	.	.	A	G	-	-	0.33333333	10,0,5,0	chr2,224620!	Cytosol	A > G
chr2:224621002:T:C:intronic:AP1S	chr2	224621002	224621002	T	C	intronic	AP1S3	.	.	.	A	G	-	-	0.11764706	15,0,2,0	chr2,224621(	Cytosol	A > G
chr2:224621015:T:C:intronic:AP1S	chr2	224621015	224621015	T	C	intronic	AP1S3	.	.	.	A	G	-	-	0.47619048	11,0,10,0	chr2,224621(	Cytosol	A > G
chr2:224621028:T:C:intronic:AP1S	chr2	224621028	224621028	T	C	intronic	AP1S3	.	.	.	A	G	-	-	0.27777778	13,0,5,0	chr2,224621(	Cytosol	A > G
chr2:224621844:T:C:UTR3:AP1S3	chr2	224621844	224621844	T	C	UTR3	AP1S3	ENST000004	.	.	A	G	-	-	0.05714286	33,0,2,0	chr2,224621	Cytosol	A > G
chr2:224622071:T:C:UTR3:AP1S3	chr2	224622071	224622071	T	C	UTR3	AP1S3	ENST000004	.	.	A	G	-	-	0.14705882	29,0,5,0	chr2,224622(	Cytosol	A > G
chr2:224622193:T:C:UTR3:AP1S3	chr2	224622193	224622193	T	C	UTR3	AP1S3	ENST000004	.	.	A	G	-	-	0.04545455	42,0,2,0	chr2,224622	Cytosol	A > G
chr2:224622278:T:C:UTR3:AP1S3	chr2	224622278	224622278	T	C	UTR3	AP1S3	ENST000004	.	.	A	G	-	-	0.05769231	49,0,3,0	chr2,224622	Cytosol	A > G
chr2:224622284:T:C:UTR3:AP1S3	chr2	224622284	224622284	T	C	UTR3	AP1S3	ENST000004	.	.	A	G	-	-	0.06666667	42,0,3,0	chr2,224622	Cytosol	A > G
chr2:228550750:T:C:UTR3:SLC19A	chr2	228550750	228550750	T	C	UTR3	SLC19A3	ENST000004	.	.	A	G	-	-	0.25	12,0,4,0	chr2,228550	Cytosol	A > G
chr2:228550765:T:C:UTR3:SLC19A	chr2	228550765	228550765	T	C	UTR3	SLC19A3	ENST000004	.	.	A	G	-	-	0.25	12,0,4,0	chr2,228550	Cytosol	A > G
chr2:228550804:T:C:UTR3:SLC19A	chr2	228550804	228550804	T	C	UTR3	SLC19A3	ENST000004	.	.	A	G	-	-	0.3	14,0,6,0	chr2,228550	Cytosol	A > G
chr2:228550850:T:C:UTR3:SLC19A	chr2	228550850	228550850	T	C	UTR3	SLC19A3	ENST000004	.	.	A	G	-	-	0.625	3,0,5,0	chr2,228550	Cytosol	A > G
chr2:228550879:T:C:exonic:SLC19	chr2	228550879	228550879	T	C	exonic	SLC19A3	.	nonsynonym	SLC19A3:ENS	A	G	-	-	0.625	3,0,5,0	chr2,228550	Cytosol	A > G
chr2:239267102:A:G:intronic:TRAF	chr2	239267102	239267102	A	G	intronic	TRAF3IP1	.	.	.	A	G	+	+	0.83333333	1,0,5,0	chr2,239267	Cytosol	A > G
chr2:242263986:A:G:exonic:3750C	chr2	242263986	242263986	A	G	exonic	9月-02	.	nonsynonym	SEPT2:ENST0	A	G	+	+	0.13333333	13,0,2,0	chr2,242263!	Cytosol	A > G
chr2:242696037:A:G:intronic:D2H(	chr2	242696037	242696037	A	G	intronic	D2HGDH	.	.	.	A	G	+	+	0.4	3,0,2,0	chr2,242696(	Cytosol	A > G
chr2:242696040:A:G:intronic:D2H(	chr2	242696040	242696040	A	G	intronic	D2HGDH	.	.	.	A	G	+	+	0.33333333	4,0,2,0	chr2,242696(	Cytosol	A > G
chr2:242696120:A:G:intronic:D2H(	chr2	242696120	242696120	A	G	intronic	D2HGDH	.	.	.	A	G	+	+	1	0,0,7,0	chr2,242696	Cytosol	A > G
chr2:242696146:A:G:intronic:D2H(	chr2	242696146	242696146	A	G	intronic	D2HGDH	.	.	.	A	G	+	+	0.85714286	1,0,6,0	chr2,242696	Cytosol	A > G
chr20:1331063:A:G:ncRNA_introni	chr20	1331063	1331063	A	G	ncRNA_intro	SDCBP2-AS1	.	.	.	A	G	+	-+	0.27272727	8,0,3,0	chr20,13310(	Cytosol	A > G
chr20:1331066:A:G:ncRNA_introni	chr20	1331066	1331066	A	G	ncRNA_intro	SDCBP2-AS1	.	.	.	A	G	+	-+	0.27272727	8,0,3,0	chr20,13310(	Cytosol	A > G
chr20:2467381:T:C:intronic:RP4-7:	chr20	2467381	2467381	T	C	intronic	RP4-734P14.	.	.	.	A	G	-	-	0.2	8,0,2,0	chr20,24673	Cytosol	A > G
chr20:3088980:T:C:ncRNA_introni	chr20	3088980	3088980	T	C	ncRNA_intro	UBOX5-AS1	.	.	.	A	G	-	+-	0.8	1,0,4,0	chr20,30889	Cytosol	A > G
chr20:3089496:T:C:ncRNA_introni	chr20	3089496	3089496	T	C	ncRNA_intro	UBOX5-AS1	.	.	.	A	G	-	+-	0.8	1,0,4,0	chr20,30894!	Cytosol	A > G
chr20:3805378:A:G:UTR3:AP5S1	chr20	3805378	3805378	A	G	UTR3	AP5S1	ENST000003	.	.	A	G	+	+	0.05263158	54,0,3,0	chr20,38053	Cytosol	A > G
chr20:3805394:A:G:UTR3:AP5S1	chr20	3805394	3805394	A	G	UTR3	AP5S1	ENST000003	.	.	A	G	+	+	0.03278689	59,0,2,0	chr20,38053!	Cytosol	A > G
chr20:3805409:A:G:UTR3:AP5S1	chr20	3805409	3805409	A	G	UTR3	AP5S1	ENST000003	.	.	A	G	+	+	0.0754717	49,0,4,0	chr20,38054(	Cytosol	A > G
chr20:3805458:A:G:UTR3:AP5S1	chr20	3805458	3805458	A	G	UTR3	AP5S1	ENST000003	.	.	A	G	+	+	0.09333333	68,0,7,0	chr20,38054!	Cytosol	A > G
chr20:3805459:A:G:UTR3:AP5S1	chr20	3805459	3805459	A	G	UTR3	AP5S1	ENST000003	.	.	A	G	+	+	0.13888889	62,0,10,0	chr20,38054!	Cytosol	A > G

chr20:3848102:A:G:UTR3:MAVS	chr20	3848102	3848102	A	G	UTR3	MAVS	ENST000004	.	.	A	G	+	+	0.10526316	17,0,2,0	chr20,38481	Cytosol	A > G
chr20:3848132:A:G:UTR3:MAVS	chr20	3848132	3848132	A	G	UTR3	MAVS	ENST000004	.	.	A	G	+	+	0.23076923	10,0,3,0	chr20,38481	Cytosol	A > G
chr20:3848158:A:G:UTR3:MAVS	chr20	3848158	3848158	A	G	UTR3	MAVS	ENST000004	.	.	A	G	+	+	0.33333333	12,0,6,0	chr20,38481	Cytosol	A > G
chr20:3848163:A:G:UTR3:MAVS	chr20	3848163	3848163	A	G	UTR3	MAVS	ENST000004	.	.	A	G	+	+	0.2	16,0,4,0	chr20,38481	Cytosol	A > G
chr20:3848168:A:G:UTR3:MAVS	chr20	3848168	3848168	A	G	UTR3	MAVS	ENST000004	.	.	A	G	+	+	0.35	13,0,7,0	chr20,38481	Cytosol	A > G
chr20:3848171:A:G:UTR3:MAVS	chr20	3848171	3848171	A	G	UTR3	MAVS	ENST000004	.	.	A	G	+	+	0.21052632	15,0,4,0	chr20,38481	Cytosol	A > G
chr20:3848178:A:G:UTR3:MAVS	chr20	3848178	3848178	A	G	UTR3	MAVS	ENST000004	.	.	A	G	+	+	0.17391304	19,0,4,0	chr20,38481	Cytosol	A > G
chr20:3848192:A:G:UTR3:MAVS	chr20	3848192	3848192	A	G	UTR3	MAVS	ENST000004	.	.	A	G	+	+	0.125	21,0,3,0	chr20,38481	Cytosol	A > G
chr20:3848225:A:G:UTR3:MAVS	chr20	3848225	3848225	A	G	UTR3	MAVS	ENST000004	.	.	A	G	+	+	0.3125	22,0,10,0	chr20,38482	Cytosol	A > G
chr20:3849010:A:G:UTR3:MAVS	chr20	3849010	3849010	A	G	UTR3	MAVS	ENST000004	.	.	A	G	+	+	0.5	6,0,6,0	chr20,38490	Cytosol	A > G
chr20:3849018:A:G:UTR3:MAVS	chr20	3849018	3849018	A	G	UTR3	MAVS	ENST000004	.	.	A	G	+	+	0.4	6,0,4,0	chr20,38490	Cytosol	A > G
chr20:3849026:A:G:UTR3:MAVS	chr20	3849026	3849026	A	G	UTR3	MAVS	ENST000004	.	.	A	G	+	+	0.42857143	8,0,6,0	chr20,38490	Cytosol	A > G
chr20:3849027:A:G:UTR3:MAVS	chr20	3849027	3849027	A	G	UTR3	MAVS	ENST000004	.	.	A	G	+	+	0.53846154	6,0,7,0	chr20,38490	Cytosol	A > G
chr20:3849057:A:G:UTR3:MAVS	chr20	3849057	3849057	A	G	UTR3	MAVS	ENST000004	.	.	A	G	+	+	0.33333333	4,0,2,0	chr20,38490	Cytosol	A > G
chr20:3849080:A:G:UTR3:MAVS	chr20	3849080	3849080	A	G	UTR3	MAVS	ENST000004	.	.	A	G	+	+	0.375	5,0,3,0	chr20,38490	Cytosol	A > G
chr20:3849095:A:G:UTR3:MAVS	chr20	3849095	3849095	A	G	UTR3	MAVS	ENST000004	.	.	A	G	+	+	0.44444444	5,0,4,0	chr20,38490	Cytosol	A > G
chr20:3849096:A:G:UTR3:MAVS	chr20	3849096	3849096	A	G	UTR3	MAVS	ENST000004	.	.	A	G	+	+	0.22222222	7,0,2,0	chr20,38490	Cytosol	A > G
chr20:3849098:A:G:UTR3:MAVS	chr20	3849098	3849098	A	G	UTR3	MAVS	ENST000004	.	.	A	G	+	+	0.36363636	7,0,4,0	chr20,38490	Cytosol	A > G
chr20:3849125:A:G:UTR3:MAVS	chr20	3849125	3849125	A	G	UTR3	MAVS	ENST000004	.	.	A	G	+	+	0.15384615	11,0,2,0	chr20,38491	Cytosol	A > G
chr20:3849126:A:G:UTR3:MAVS	chr20	3849126	3849126	A	G	UTR3	MAVS	ENST000004	.	.	A	G	+	+	0.38461538	8,0,5,0	chr20,38491	Cytosol	A > G
chr20:3849134:A:G:UTR3:MAVS	chr20	3849134	3849134	A	G	UTR3	MAVS	ENST000004	.	.	A	G	+	+	0.5	7,0,7,0	chr20,38491	Cytosol	A > G
chr20:3849138:A:G:UTR3:MAVS	chr20	3849138	3849138	A	G	UTR3	MAVS	ENST000004	.	.	A	G	+	+	0.14285714	12,0,2,0	chr20,38491	Cytosol	A > G
chr20:3849144:A:G:UTR3:MAVS	chr20	3849144	3849144	A	G	UTR3	MAVS	ENST000004	.	.	A	G	+	+	0.76923077	3,0,10,0	chr20,38491	Cytosol	A > G
chr20:3849150:A:G:UTR3:MAVS	chr20	3849150	3849150	A	G	UTR3	MAVS	ENST000004	.	.	A	G	+	+	0.33333333	8,0,4,0	chr20,38491	Cytosol	A > G
chr20:3849160:A:G:UTR3:MAVS	chr20	3849160	3849160	A	G	UTR3	MAVS	ENST000004	.	.	A	G	+	+	0.41666667	7,0,5,0	chr20,38491	Cytosol	A > G
chr20:3849161:A:G:UTR3:MAVS	chr20	3849161	3849161	A	G	UTR3	MAVS	ENST000004	.	.	A	G	+	+	0.58333333	5,0,7,0	chr20,38491	Cytosol	A > G
chr20:3849166:A:G:UTR3:MAVS	chr20	3849166	3849166	A	G	UTR3	MAVS	ENST000004	.	.	A	G	+	+	0.18181818	9,0,2,0	chr20,38491	Cytosol	A > G
chr20:3849174:A:G:UTR3:MAVS	chr20	3849174	3849174	A	G	UTR3	MAVS	ENST000004	.	.	A	G	+	+	0.2	8,0,2,0	chr20,38491	Cytosol	A > G
chr20:3849202:A:G:UTR3:MAVS	chr20	3849202	3849202	A	G	UTR3	MAVS	ENST000004	.	.	A	G	+	+	0.375	5,0,3,0	chr20,38492	Cytosol	A > G
chr20:3849206:A:G:UTR3:MAVS	chr20	3849206	3849206	A	G	UTR3	MAVS	ENST000004	.	.	A	G	+	+	0.22222222	7,0,2,0	chr20,38492	Cytosol	A > G
chr20:3849226:A:G:UTR3:MAVS	chr20	3849226	3849226	A	G	UTR3	MAVS	ENST000004	.	.	A	G	+	+	0.33333333	8,0,4,0	chr20,38492	Cytosol	A > G
chr20:3849263:A:G:UTR3:MAVS	chr20	3849263	3849263	A	G	UTR3	MAVS	ENST000004	.	.	A	G	+	+	0.27272727	8,0,3,0	chr20,38492	Cytosol	A > G
chr20:3849409:A:G:downstream:M	chr20	3849409	3849409	A	G	downstream	MAVS	.	.	.	A	G	+	+	0.25	6,0,2,0	chr20,38494	Cytosol	A > G
chr20:3849481:A:G:downstream:M	chr20	3849481	3849481	A	G	downstream	MAVS	.	.	.	A	G	+	+	0.22222222	7,0,2,0	chr20,38494	Cytosol	A > G
chr20:3849488:A:G:downstream:M	chr20	3849488	3849488	A	G	downstream	MAVS	.	.	.	A	G	+	+	0.5	3,0,3,0	chr20,38494	Cytosol	A > G
chr20:3849774:A:G:downstream:M	chr20	3849774	3849774	A	G	downstream	MAVS	.	.	.	A	G	+	+	0.2	20,0,5,0	chr20,38497	Cytosol	A > G
chr20:3849777:A:G:downstream:M	chr20	3849777	3849777	A	G	downstream	MAVS	.	.	.	A	G	+	+	0.86956522	3,0,20,0	chr20,38497	Cytosol	A > G
chr20:3850027:A:G:downstream:M	chr20	3850027	3850027	A	G	downstream	MAVS	.	.	.	A	G	+	+	0.75	2,0,6,0	chr20,38500	Cytosol	A > G
chr20:3850081:A:G:downstream:M	chr20	3850081	3850081	A	G	downstream	MAVS	.	.	.	A	G	+	+	0.42857143	8,0,6,0	chr20,38500	Cytosol	A > G
chr20:3850082:A:G:downstream:M	chr20	3850082	3850082	A	G	downstream	MAVS	.	.	.	A	G	+	+	0.41666667	7,0,5,0	chr20,38500	Cytosol	A > G
chr20:3850094:A:G:downstream:M	chr20	3850094	3850094	A	G	downstream	MAVS	.	.	.	A	G	+	+	0.6	6,0,9,0	chr20,38500	Cytosol	A > G
chr20:3850134:A:G:downstream:M	chr20	3850134	3850134	A	G	downstream	MAVS	.	.	.	A	G	+	+	0.3	14,0,6,0	chr20,38501	Cytosol	A > G
chr20:3850191:A:G:downstream:M	chr20	3850191	3850191	A	G	downstream	MAVS	.	.	.	A	G	+	+	0.54285714	16,0,19,0	chr20,38501	Cytosol	A > G
chr20:3850199:A:G:downstream:M	chr20	3850199	3850199	A	G	downstream	MAVS	.	.	.	A	G	+	+	0.05882353	32,0,2,0	chr20,38501	Cytosol	A > G
chr20:3850200:A:G:downstream:M	chr20	3850200	3850200	A	G	downstream	MAVS	.	.	.	A	G	+	+	0.05714286	33,0,2,0	chr20,38501	Cytosol	A > G
chr20:3850201:A:G:downstream:M	chr20	3850201	3850201	A	G	downstream	MAVS	.	.	.	A	G	+	+	0.17142857	29,0,6,0	chr20,38502	Cytosol	A > G
chr20:3850273:A:G:downstream:M	chr20	3850273	3850273	A	G	downstream	MAVS	.	.	.	A	G	+	+	0.125	21,0,3,0	chr20,38502	Cytosol	A > G
chr20:3850277:A:G:downstream:M	chr20	3850277	3850277	A	G	downstream	MAVS	.	.	.	A	G	+	+	0.10526316	17,0,2,0	chr20,38502	Cytosol	A > G
chr20:3850283:A:G:intergenic:MA	chr20	3850283	3850283	A	G	intergenic	MAVS,RP11-	dist=1003;dis	.	.	A	G	+	+	0.16666667	15,0,3,0	chr20,38502	Cytosol	A > G
chr20:3850321:A:G:intergenic:MA	chr20	3850321	3850321	A	G	intergenic	MAVS,RP11-	dist=1041;dis	.	.	A	G	+	+	0.54166667	11,0,13,0	chr20,38503	Cytosol	A > G
chr20:3850332:A:G:intergenic:MA	chr20	3850332	3850332	A	G	intergenic	MAVS,RP11-	dist=1052;dis	.	.	A	G	+	+	0.2	20,0,5,0	chr20,38503	Cytosol	A > G
chr20:3850351:A:G:intergenic:MA	chr20	3850351	3850351	A	G	intergenic	MAVS,RP11-	dist=1071;dis	.	.	A	G	+	+	0.39130435	14,0,9,0	chr20,38503	Cytosol	A > G
chr20:3850352:A:G:intergenic:MA	chr20	3850352	3850352	A	G	intergenic	MAVS,RP11-	dist=1072;dis	.	.	A	G	+	+	0.82608696	4,0,19,0	chr20,38503	Cytosol	A > G
chr20:3850355:A:G:intergenic:MA	chr20	3850355	3850355	A	G	intergenic	MAVS,RP11-	dist=1075;dis	.	.	A	G	+	+	0.2173913	18,0,5,0	chr20,38503	Cytosol	A > G
chr20:3850369:A:G:intergenic:MA	chr20	3850369	3850369	A	G	intergenic	MAVS,RP11-	dist=1089;dis	.	.	A	G	+	+	0.59090909	9,0,13,0	chr20,38503	Cytosol	A > G
chr20:3850391:A:G:intergenic:MA	chr20	3850391	3850391	A	G	intergenic	MAVS,RP11-	dist=1111;dis	.	.	A	G	+	+	0.65517241	10,0,19,0	chr20,38503	Cytosol	A > G
chr20:3850432:A:G:intergenic:MA	chr20	3850432	3850432	A	G	intergenic	MAVS,RP11-	dist=1152;dis	.	.	A	G	+	+	0.1	18,0,2,0	chr20,38504	Cytosol	A > G
chr20:3850435:A:G:intergenic:MA	chr20	3850435	3850435	A	G	intergenic	MAVS,RP11-	dist=1155;dis	.	.	A	G	+	+	0.15	17,0,3,0	chr20,38504	Cytosol	A > G
chr20:3850461:A:G:intergenic:MA	chr20	3850461	3850461	A	G	intergenic	MAVS,RP11-	dist=1181;dis	.	.	A	G	+	+	0.13043478	20,0,3,0	chr20,38504	Cytosol	A > G
chr20:3850487:A:G:intergenic:MA	chr20	3850487	3850487	A	G	intergenic	MAVS,RP11-	dist=1207;dis	.	.	A	G	+	+	0.18181818	27,0,6,0	chr20,38504	Cytosol	A > G
chr20:3850513:A:G:intergenic:MA	chr20	3850513	3850513	A	G	intergenic	MAVS,RP11-	dist=1233;dis	.	.	A	G	+	+	0.26666667	22,0,8,0	chr20,38505	Cytosol	A > G
chr20:3850514:A:G:intergenic:MA	chr20	3850514	3850514	A	G	intergenic	MAVS,RP11-	dist=1234;dis	.	.	A	G	+	+	0.26666667	22,0,8,0	chr20,38505	Cytosol	A > G
chr20:3851191:A:G:intergenic:MA	chr20	3851191	3851191	A	G	intergenic	MAVS,RP11-	dist=1911;dis	.	.	A	G	+	+	0.23076923	10,0,3,0	chr20,38511	Cytosol	A > G
chr20:3851192:A:G:intergenic:MA	chr20	3851192	3851192	A	G	intergenic	MAVS,RP11-	dist=1912;dis	.	.	A	G	+	+	0.30769231	9,0,4,0	chr20,38511	Cytosol	A > G

chr20:3851209:A:G:intergenic:MA	chr20	3851209	3851209	A	G	intergenic	MAVS,RP11-	dist=1929;dis	.	.	A	G	+	+	0.75	4,0,12,0	chr20,38512	Cytosol	A > G
chr20:3851621:A:G:intergenic:MA	chr20	3851621	3851621	A	G	intergenic	MAVS,RP11-	dist=2341;dis	.	.	A	G	+	+	0.35294118	11,0,6,0	chr20,38516	Cytosol	A > G
chr20:3851628:A:G:intergenic:MA	chr20	3851628	3851628	A	G	intergenic	MAVS,RP11-	dist=2348;dis	.	.	A	G	+	+	0.47058824	9,0,8,0	chr20,38516	Cytosol	A > G
chr20:3851645:A:G:intergenic:MA	chr20	3851645	3851645	A	G	intergenic	MAVS,RP11-	dist=2365;dis	.	.	A	G	+	+	0.38888889	11,0,7,0	chr20,38516	Cytosol	A > G
chr20:3851657:A:G:intergenic:MA	chr20	3851657	3851657	A	G	intergenic	MAVS,RP11-	dist=2377;dis	.	.	A	G	+	+	0.22580645	24,0,7,0	chr20,38516	Cytosol	A > G
chr20:3851667:A:G:intergenic:MA	chr20	3851667	3851667	A	G	intergenic	MAVS,RP11-	dist=2387;dis	.	.	A	G	+	+	0.22857143	27,0,8,0	chr20,38516	Cytosol	A > G
chr20:3851711:A:G:intergenic:MA	chr20	3851711	3851711	A	G	intergenic	MAVS,RP11-	dist=2431;dis	.	.	A	G	+	+	0.05263158	36,0,2,0	chr20,38517	Cytosol	A > G
chr20:3851762:A:G:intergenic:MA	chr20	3851762	3851762	A	G	intergenic	MAVS,RP11-	dist=2482;dis	.	.	A	G	+	+	0.12121212	29,0,4,0	chr20,38517	Cytosol	A > G
chr20:3851763:A:G:intergenic:MA	chr20	3851763	3851763	A	G	intergenic	MAVS,RP11-	dist=2483;dis	.	.	A	G	+	+	0.13513514	32,0,5,0	chr20,38517	Cytosol	A > G
chr20:3851791:A:G:intergenic:MA	chr20	3851791	3851791	A	G	intergenic	MAVS,RP11-	dist=2511;dis	.	.	A	G	+	+	0.24242424	25,0,8,0	chr20,38517	Cytosol	A > G
chr20:3852823:A:G:intergenic:MA	chr20	3852823	3852823	A	G	intergenic	MAVS,RP11-	dist=3543;dis	.	.	A	G	+	+	0.14814815	46,0,8,0	chr20,38528	Cytosol	A > G
chr20:3852829:A:G:intergenic:MA	chr20	3852829	3852829	A	G	intergenic	MAVS,RP11-	dist=3549;dis	.	.	A	G	+	+	0.42857143	32,0,24,0	chr20,38528	Cytosol	A > G
chr20:3852854:A:G:intergenic:MA	chr20	3852854	3852854	A	G	intergenic	MAVS,RP11-	dist=3574;dis	.	.	A	G	+	+	0.17241379	48,0,10,0	chr20,38528	Cytosol	A > G
chr20:3852858:A:G:intergenic:MA	chr20	3852858	3852858	A	G	intergenic	MAVS,RP11-	dist=3578;dis	.	.	A	G	+	+	0.32307692	44,0,21,0	chr20,38528	Cytosol	A > G
chr20:3852861:A:G:intergenic:MA	chr20	3852861	3852861	A	G	intergenic	MAVS,RP11-	dist=3581;dis	.	.	A	G	+	+	0.20634921	50,0,13,0	chr20,38528	Cytosol	A > G
chr20:3852885:A:G:intergenic:MA	chr20	3852885	3852885	A	G	intergenic	MAVS,RP11-	dist=3605;dis	.	.	A	G	+	+	0.08196721	56,0,5,0	chr20,38528	Cytosol	A > G
chr20:3852896:A:G:intergenic:MA	chr20	3852896	3852896	A	G	intergenic	MAVS,RP11-	dist=3616;dis	.	.	A	G	+	+	0.06349206	59,0,4,0	chr20,38528	Cytosol	A > G
chr20:3852901:A:G:intergenic:MA	chr20	3852901	3852901	A	G	intergenic	MAVS,RP11-	dist=3621;dis	.	.	A	G	+	+	0.10169492	53,0,6,0	chr20,38529	Cytosol	A > G
chr20:3852922:A:G:intergenic:MA	chr20	3852922	3852922	A	G	intergenic	MAVS,RP11-	dist=3642;dis	.	.	A	G	+	+	0.08333333	44,0,4,0	chr20,38529	Cytosol	A > G
chr20:3852923:A:G:intergenic:MA	chr20	3852923	3852923	A	G	intergenic	MAVS,RP11-	dist=3643;dis	.	.	A	G	+	+	0.125	42,0,6,0	chr20,38529	Cytosol	A > G
chr20:3852924:A:G:intergenic:MA	chr20	3852924	3852924	A	G	intergenic	MAVS,RP11-	dist=3644;dis	.	.	A	G	+	+	0.10638298	42,0,5,0	chr20,38529	Cytosol	A > G
chr20:3852925:A:G:intergenic:MA	chr20	3852925	3852925	A	G	intergenic	MAVS,RP11-	dist=3645;dis	.	.	A	G	+	+	0.08510638	43,0,4,0	chr20,38529	Cytosol	A > G
chr20:3852926:A:G:intergenic:MA	chr20	3852926	3852926	A	G	intergenic	MAVS,RP11-	dist=3646;dis	.	.	A	G	+	+	0.09803922	46,0,5,0	chr20,38529	Cytosol	A > G
chr20:3852927:A:G:intergenic:MA	chr20	3852927	3852927	A	G	intergenic	MAVS,RP11-	dist=3647;dis	.	.	A	G	+	+	0.05454545	52,0,3,0	chr20,38529	Cytosol	A > G
chr20:3852938:A:G:intergenic:MA	chr20	3852938	3852938	A	G	intergenic	MAVS,RP11-	dist=3658;dis	.	.	A	G	+	+	0.06	47,0,3,0	chr20,38529	Cytosol	A > G
chr20:3852978:A:G:intergenic:MA	chr20	3852978	3852978	A	G	intergenic	MAVS,RP11-	dist=3698;dis	.	.	A	G	+	+	0.12727273	48,0,7,0	chr20,38529	Cytosol	A > G
chr20:3852987:A:G:intergenic:MA	chr20	3852987	3852987	A	G	intergenic	MAVS,RP11-	dist=3707;dis	.	.	A	G	+	+	0.0877193	52,0,5,0	chr20,38529	Cytosol	A > G
chr20:3853000:A:G:intergenic:MA	chr20	3853000	3853000	A	G	intergenic	MAVS,RP11-	dist=3720;dis	.	.	A	G	+	+	0.32786885	41,0,20,0	chr20,38529	Cytosol	A > G
chr20:3853005:A:G:intergenic:MA	chr20	3853005	3853005	A	G	intergenic	MAVS,RP11-	dist=3725;dis	.	.	A	G	+	+	0.1641791	56,0,11,0	chr20,38530	Cytosol	A > G
chr20:3853024:A:G:intergenic:MA	chr20	3853024	3853024	A	G	intergenic	MAVS,RP11-	dist=3744;dis	.	.	A	G	+	+	0.09090909	60,0,6,0	chr20,38530	Cytosol	A > G
chr20:3853060:A:G:intergenic:MA	chr20	3853060	3853060	A	G	intergenic	MAVS,RP11-	dist=3780;dis	.	.	A	G	+	+	0.08235294	78,0,7,0	chr20,38530	Cytosol	A > G
chr20:3853067:A:G:intergenic:MA	chr20	3853067	3853067	A	G	intergenic	MAVS,RP11-	dist=3787;dis	.	.	A	G	+	+	0.15384615	66,0,12,0	chr20,38530	Cytosol	A > G
chr20:3853956:A:G:intergenic:MA	chr20	3853956	3853956	A	G	intergenic	MAVS,RP11-	dist=4676;dis	.	.	A	G	+	+	0.22222222	28,0,8,0	chr20,38539	Cytosol	A > G
chr20:3853967:A:G:intergenic:MA	chr20	3853967	3853967	A	G	intergenic	MAVS,RP11-	dist=4687;dis	.	.	A	G	+	+	0.13333333	26,0,4,0	chr20,38539	Cytosol	A > G
chr20:3853980:A:G:intergenic:MA	chr20	3853980	3853980	A	G	intergenic	MAVS,RP11-	dist=4700;dis	.	.	A	G	+	+	0.14285714	24,0,4,0	chr20,38539	Cytosol	A > G
chr20:3854019:A:G:intergenic:MA	chr20	3854019	3854019	A	G	intergenic	MAVS,RP11-	dist=4739;dis	.	.	A	G	+	+	0.11111111	32,0,4,0	chr20,38540	Cytosol	A > G
chr20:3854021:A:G:intergenic:MA	chr20	3854021	3854021	A	G	intergenic	MAVS,RP11-	dist=4741;dis	.	.	A	G	+	+	0.14634146	35,0,6,0	chr20,38540	Cytosol	A > G
chr20:3854022:A:G:intergenic:MA	chr20	3854022	3854022	A	G	intergenic	MAVS,RP11-	dist=4742;dis	.	.	A	G	+	+	0.08695652	42,0,4,0	chr20,38540	Cytosol	A > G
chr20:3854025:A:G:intergenic:MA	chr20	3854025	3854025	A	G	intergenic	MAVS,RP11-	dist=4745;dis	.	.	A	G	+	+	0.06521739	43,0,3,0	chr20,38540	Cytosol	A > G
chr20:3854027:A:G:intergenic:MA	chr20	3854027	3854027	A	G	intergenic	MAVS,RP11-	dist=4747;dis	.	.	A	G	+	+	0.0625	45,0,3,0	chr20,38540	Cytosol	A > G
chr20:3854061:A:G:intergenic:MA	chr20	3854061	3854061	A	G	intergenic	MAVS,RP11-	dist=4781;dis	.	.	A	G	+	+	0.12727273	48,0,7,0	chr20,38540	Cytosol	A > G
chr20:3854106:A:G:intergenic:MA	chr20	3854106	3854106	A	G	intergenic	MAVS,RP11-	dist=4826;dis	.	.	A	G	+	+	0.17021277	39,0,8,0	chr20,38541	Cytosol	A > G
chr20:3854163:A:G:intergenic:MA	chr20	3854163	3854163	A	G	intergenic	MAVS,RP11-	dist=4883;dis	.	.	A	G	+	+	0.11764706	30,0,4,0	chr20,38541	Cytosol	A > G
chr20:3855844:A:G:intergenic:MA	chr20	3855844	3855844	A	G	intergenic	MAVS,RP11-	dist=6564;dis	.	.	A	G	+	+	0.10869565	41,0,5,0	chr20,38558	Cytosol	A > G
chr20:3855847:A:G:intergenic:MA	chr20	3855847	3855847	A	G	intergenic	MAVS,RP11-	dist=6567;dis	.	.	A	G	+	+	0.06666667	42,0,3,0	chr20,38558	Cytosol	A > G
chr20:3907469:A:G:UTR3:PANK2	chr20	3907469	3907469	A	G	UTR3	PANK2	ENST000004	.	.	A	G	+	+	0.4	3,0,2,0	chr20,39074	Cytosol	A > G
chr20:3907470:A:G:UTR3:PANK2	chr20	3907470	3907470	A	G	UTR3	PANK2	ENST000004	.	.	A	G	+	+	0.6	2,0,3,0	chr20,39074	Cytosol	A > G
chr20:5975434:A:G:UTR3:MCM8	chr20	5975434	5975434	A	G	UTR3	MCM8	ENST000003	.	.	A	G	+	+-	0.06097561	77,0,5,0	chr20,59754	Cytosol	A > G
chr20:5976734:A:G:downstream:M	chr20	5976734	5976734	A	G	downstream	MCM8	.	.	.	A	G	+	-	0.1875	13,0,3,0	chr20,59767	Cytosol	A > G
chr20:5976774:A:G:downstream:M	chr20	5976774	5976774	A	G	downstream	MCM8	.	.	.	A	G	+	-	0.25	9,0,3,0	chr20,59767	Cytosol	A > G
chr20:5976798:A:G:downstream:M	chr20	5976798	5976798	A	G	downstream	MCM8	.	.	.	A	G	+	-	0.28571429	10,0,4,0	chr20,59767	Cytosol	A > G
chr20:5976824:A:G:downstream:M	chr20	5976824	5976824	A	G	downstream	MCM8	.	.	.	A	G	+	-	0.45454545	6,0,5,0	chr20,59768	Cytosol	A > G
chr20:5976840:A:G:downstream:M	chr20	5976840	5976840	A	G	downstream	MCM8	.	.	.	A	G	+	-	0.58333333	5,0,7,0	chr20,59768	Cytosol	A > G
chr20:5976859:A:G:intergenic:MC	chr20	5976859	5976859	A	G	intergenic	MCM8,RN7S	dist=1007;dis	.	.	A	G	+	-	0.41666667	7,0,5,0	chr20,59768	Cytosol	A > G
chr20:5977051:A:G:intergenic:MC	chr20	5977051	5977051	A	G	intergenic	MCM8,RN7S	dist=1199;dis	.	.	A	G	+	-	0.6	2,0,3,0	chr20,59770	Cytosol	A > G
chr20:5977911:A:G:intergenic:MC	chr20	5977911	5977911	A	G	intergenic	MCM8,RN7S	dist=2059;dis	.	.	A	G	+	-	0.4	3,0,2,0	chr20,59779	Cytosol	A > G
chr20:5977960:A:G:intergenic:MC	chr20	5977960	5977960	A	G	intergenic	MCM8,RN7S	dist=2108;dis	.	.	A	G	+	-	0.66666667	3,0,6,0	chr20,59779	Cytosol	A > G
chr20:5978362:A:G:intergenic:MC	chr20	5978362	5978362	A	G	intergenic	MCM8,RN7S	dist=2510;dis	.	.	A	G	+	-	0.5	5,0,5,0	chr20,59783	Cytosol	A > G
chr20:5978379:A:G:intergenic:MC	chr20	5978379	5978379	A	G	intergenic	MCM8,RN7S	dist=2527;dis	.	.	A	G	+	-	0.25	12,0,4,0	chr20,59783	Cytosol	A > G
chr20:5978386:A:G:intergenic:MC	chr20	5978386	5978386	A	G	intergenic	MCM8,RN7S	dist=2534;dis	.	.	A	G	+	-	0.1875	13,0,3,0	chr20,59783	Cytosol	A > G
chr20:5978389:A:G:intergenic:MC	chr20	5978389	5978389	A	G	intergenic	MCM8,RN7S	dist=2537;dis	.	.	A	G	+	-	0.25	12,0,4,0	chr20,59783	Cytosol	A > G
chr20:5978391:A:G:intergenic:MC	chr20	5978391	5978391	A	G	intergenic	MCM8,RN7S	dist=2539;dis	.	.	A	G	+	-	0.5625	7,0,9,0	chr20,59783	Cytosol	A > G
chr20:5978428:A:G:intergenic:MC	chr20	5978428	5978428	A	G	intergenic	MCM8,RN7S	dist=2576;dis	.	.	A	G	+	-	0.25	9,0,3,0	chr20,59784	Cytosol	A > G
chr20:5979776:A:G:intergenic:MC	chr20	5979776	5979776	A	G	intergenic	MCM8,RN7S	dist=3924;dis	.	.	A	G	+	-	0.88888889	1,0,8,0	chr20,59797	Cytosol	A > G

chr20:5980697:A:G:intergenic:MC	chr20	5980697	5980697	A	G	intergenic	MCM8,RN7S	dist=4845;dis	.	.	A	G	+	-	0.38461538	8,0,5,0	chr20,59806	Cytosol	A > G
chr20:5981201:A:G:intergenic:MC	chr20	5981201	5981201	A	G	intergenic	MCM8,RN7S	dist=5349;dis	.	.	A	G	+	-	0.28571429	5,0,2,0	chr20,59812	Cytosol	A > G
chr20:5981214:A:G:intergenic:MC	chr20	5981214	5981214	A	G	intergenic	MCM8,RN7S	dist=5362;dis	.	.	A	G	+	-	0.22222222	7,0,2,0	chr20,59812	Cytosol	A > G
chr20:10036978:A:G:ncRNA_exoni	chr20	10036978	10036978	A	G	ncRNA_exon	AL109754.1	.	.	.	A	G	+	-+	1	0,0,8,0	chr20,10036	Cytosol	A > G
chr20:10037034:A:G:ncRNA_exoni	chr20	10037034	10037034	A	G	ncRNA_exon	AL109754.1	.	.	.	A	G	+	-+	0.36363636	14,0,8,0	chr20,10037	Cytosol	A > G
chr20:17942938:T:C:intronic:SNX5	chr20	17942938	17942938	T	C	intronic	SNX5	.	.	.	A	G	-	-	0.08	23,0,2,0	chr20,17942	Cytosol	A > G
chr20:17942976:T:C:intronic:SNX5	chr20	17942976	17942976	T	C	intronic	SNX5	.	.	.	A	G	-	-	0.15384615	11,0,2,0	chr20,17942	Cytosol	A > G
chr20:17942980:T:C:intronic:SNX5	chr20	17942980	17942980	T	C	intronic	SNX5	.	.	.	A	G	-	-	0.14285714	12,0,2,0	chr20,17942	Cytosol	A > G
chr20:17942985:T:C:intronic:SNX5	chr20	17942985	17942985	T	C	intronic	SNX5	.	.	.	A	G	-	-	0.25	9,0,3,0	chr20,17942	Cytosol	A > G
chr20:17943068:T:C:intronic:SNX5	chr20	17943068	17943068	T	C	intronic	SNX5	.	.	.	A	G	-	-	0.28571429	10,0,4,0	chr20,17943	Cytosol	A > G
chr20:17943118:T:C:intronic:SNX5	chr20	17943118	17943118	T	C	intronic	SNX5	.	.	.	A	G	-	-	0.16666667	15,0,3,0	chr20,17943	Cytosol	A > G
chr20:17943119:T:C:intronic:SNX5	chr20	17943119	17943119	T	C	intronic	SNX5	.	.	.	A	G	-	-	0.55555556	8,0,10,0	chr20,17943	Cytosol	A > G
chr20:17943121:T:C:intronic:SNX5	chr20	17943121	17943121	T	C	intronic	SNX5	.	.	.	A	G	-	-	0.65	7,0,13,0	chr20,17943	Cytosol	A > G
chr20:18468339:T:C:UTR3:RBBP9	chr20	18468339	18468339	T	C	UTR3	RBBP9	ENST000003	.	.	A	G	-	-	0.40298507	40,0,27,0	chr20,18468	Cytosol	A > G
chr20:18468351:T:C:UTR3:RBBP9	chr20	18468351	18468351	T	C	UTR3	RBBP9	ENST000003	.	.	A	G	-	-	0.31034483	40,0,18,0	chr20,18468	Cytosol	A > G
chr20:18468352:T:C:UTR3:RBBP9	chr20	18468352	18468352	T	C	UTR3	RBBP9	ENST000003	.	.	A	G	-	-	0.09677419	56,0,6,0	chr20,18468	Cytosol	A > G
chr20:18468361:T:C:UTR3:RBBP9	chr20	18468361	18468361	T	C	UTR3	RBBP9	ENST000003	.	.	A	G	-	-	0.17741935	51,0,11,0	chr20,18468	Cytosol	A > G
chr20:18468378:T:C:UTR3:RBBP9	chr20	18468378	18468378	T	C	UTR3	RBBP9	ENST000003	.	.	A	G	-	-	0.13043478	40,0,6,0	chr20,18468	Cytosol	A > G
chr20:18468468:T:C:UTR3:RBBP9	chr20	18468468	18468468	T	C	UTR3	RBBP9	ENST000003	.	.	A	G	-	-	0.07526882	86,0,7,0	chr20,18468	Cytosol	A > G
chr20:18468472:T:C:UTR3:RBBP9	chr20	18468472	18468472	T	C	UTR3	RBBP9	ENST000003	.	.	A	G	-	-	0.06666667	84,0,6,0	chr20,18468	Cytosol	A > G
chr20:18468473:T:C:UTR3:RBBP9	chr20	18468473	18468473	T	C	UTR3	RBBP9	ENST000003	.	.	A	G	-	-	0.06666667	84,0,6,0	chr20,18468	Cytosol	A > G
chr20:18468474:T:C:UTR3:RBBP9	chr20	18468474	18468474	T	C	UTR3	RBBP9	ENST000003	.	.	A	G	-	-	0.12087912	79,0,11,1	chr20,18468	Cytosol	A > G
chr20:18468511:T:C:UTR3:RBBP9	chr20	18468511	18468511	T	C	UTR3	RBBP9	ENST000003	.	.	A	G	-	-	0.30588235	59,0,26,0	chr20,18468	Cytosol	A > G
chr20:18468512:T:C:UTR3:RBBP9	chr20	18468512	18468512	T	C	UTR3	RBBP9	ENST000003	.	.	A	G	-	-	0.04597701	83,0,4,0	chr20,18468	Cytosol	A > G
chr20:18468537:T:C:UTR3:RBBP9	chr20	18468537	18468537	T	C	UTR3	RBBP9	ENST000003	.	.	A	G	-	-	0.04761905	80,0,4,0	chr20,18468	Cytosol	A > G
chr20:18469048:T:C:UTR3:RBBP9	chr20	18469048	18469048	T	C	UTR3	RBBP9	ENST000003	.	.	A	G	-	-	0.45454545	30,0,25,0	chr20,18469	Cytosol	A > G
chr20:18469054:T:C:UTR3:RBBP9	chr20	18469054	18469054	T	C	UTR3	RBBP9	ENST000003	.	.	A	G	-	-	0.20408163	39,0,10,0	chr20,18469	Cytosol	A > G
chr20:18469085:T:C:UTR3:RBBP9	chr20	18469085	18469085	T	C	UTR3	RBBP9	ENST000003	.	.	A	G	-	-	0.10294118	61,0,7,0	chr20,18469	Cytosol	A > G
chr20:18469087:T:C:UTR3:RBBP9	chr20	18469087	18469087	T	C	UTR3	RBBP9	ENST000003	.	.	A	G	-	-	0.21538462	51,0,14,0	chr20,18469	Cytosol	A > G
chr20:18469089:T:C:UTR3:RBBP9	chr20	18469089	18469089	T	C	UTR3	RBBP9	ENST000003	.	.	A	G	-	-	0.03076923	63,0,2,0	chr20,18469	Cytosol	A > G
chr20:18469137:T:C:UTR3:RBBP9	chr20	18469137	18469137	T	C	UTR3	RBBP9	ENST000003	.	.	A	G	-	-	0.05	57,0,3,0	chr20,18469	Cytosol	A > G
chr20:18469182:T:C:UTR3:RBBP9	chr20	18469182	18469182	T	C	UTR3	RBBP9	ENST000003	.	.	A	G	-	-	0.32258065	21,0,10,0	chr20,18469	Cytosol	A > G
chr20:18469183:T:C:UTR3:RBBP9	chr20	18469183	18469183	T	C	UTR3	RBBP9	ENST000003	.	.	A	G	-	-	0.2	24,0,6,0	chr20,18469	Cytosol	A > G
chr20:25426905:A:G:UTR3:GINS1	chr20	25426905	25426905	A	G	UTR3	GINS1	ENST000002	.	.	A	G	+	+	0.03030303	160,0,5,0	chr20,25426	Cytosol	A > G
chr20:25427756:A:G:UTR3:GINS1	chr20	25427756	25427756	A	G	UTR3	GINS1	ENST000002	.	.	A	G	+	+	0.06306306	104,0,7,0	chr20,25427	Cytosol	A > G
chr20:25427805:A:G:UTR3:GINS1	chr20	25427805	25427805	A	G	UTR3	GINS1	ENST000002	.	.	A	G	+	+	0.05357143	106,0,6,0	chr20,25427	Cytosol	A > G
chr20:25427815:A:G:UTR3:GINS1	chr20	25427815	25427815	A	G	UTR3	GINS1	ENST000002	.	.	A	G	+	+	0.30357143	78,0,34,0	chr20,25427	Cytosol	A > G
chr20:25428294:A:G:UTR3:GINS1	chr20	25428294	25428294	A	G	UTR3	GINS1	ENST000002	.	.	A	G	+	+	0.33846154	86,0,44,0	chr20,25428	Cytosol	A > G
chr20:25428308:A:G:UTR3:GINS1	chr20	25428308	25428308	A	G	UTR3	GINS1	ENST000002	.	.	A	G	+	+	0.19875776	129,0,32,0	chr20,25428	Cytosol	A > G
chr20:25428320:A:G:UTR3:GINS1	chr20	25428320	25428320	A	G	UTR3	GINS1	ENST000002	.	.	A	G	+	+	0.04666667	143,0,7,0	chr20,25428	Cytosol	A > G
chr20:25428348:A:G:UTR3:GINS1	chr20	25428348	25428348	A	G	UTR3	GINS1	ENST000002	.	.	A	G	+	+	0.05633803	134,0,8,0	chr20,25428	Cytosol	A > G
chr20:25428384:A:G:UTR3:GINS1	chr20	25428384	25428384	A	G	UTR3	GINS1	ENST000002	.	.	A	G	+	+	0.04794521	139,0,7,0	chr20,25428	Cytosol	A > G
chr20:25428503:A:G:UTR3:GINS1	chr20	25428503	25428503	A	G	UTR3	GINS1	ENST000002	.	.	A	G	+	+	0.17605634	117,0,25,0	chr20,25428	Cytosol	A > G
chr20:25428511:A:G:UTR3:GINS1	chr20	25428511	25428511	A	G	UTR3	GINS1	ENST000002	.	.	A	G	+	+	0.11510791	123,0,16,0	chr20,25428	Cytosol	A > G
chr20:25428512:A:G:UTR3:GINS1	chr20	25428512	25428512	A	G	UTR3	GINS1	ENST000002	.	.	A	G	+	+	0.03546099	136,0,5,0	chr20,25428	Cytosol	A > G
chr20:25428547:A:G:UTR3:GINS1	chr20	25428547	25428547	A	G	UTR3	GINS1	ENST000002	.	.	A	G	+	+	0.08108108	136,0,12,0	chr20,25428	Cytosol	A > G
chr20:25428587:A:G:UTR3:GINS1	chr20	25428587	25428587	A	G	UTR3	GINS1	ENST000002	.	.	A	G	+	+	0.26666667	121,0,44,0	chr20,25428	Cytosol	A > G
chr20:25428588:A:G:UTR3:GINS1	chr20	25428588	25428588	A	G	UTR3	GINS1	ENST000002	.	.	A	G	+	+	0.19526627	136,0,33,0	chr20,25428	Cytosol	A > G
chr20:25428590:A:G:UTR3:GINS1	chr20	25428590	25428590	A	G	UTR3	GINS1	ENST000002	.	.	A	G	+	+	0.05521472	154,0,9,0	chr20,25428	Cytosol	A > G
chr20:25428601:A:G:UTR3:GINS1	chr20	25428601	25428601	A	G	UTR3	GINS1	ENST000002	.	.	A	G	+	+	0.07638889	133,0,11,0	chr20,25428	Cytosol	A > G
chr20:25428606:A:G:UTR3:GINS1	chr20	25428606	25428606	A	G	UTR3	GINS1	ENST000002	.	.	A	G	+	+	0.04635762	144,0,7,0	chr20,25428	Cytosol	A > G
chr20:25428620:A:G:UTR3:GINS1	chr20	25428620	25428620	A	G	UTR3	GINS1	ENST000002	.	.	A	G	+	+	0.01333333	148,0,2,0	chr20,25428	Cytosol	A > G
chr20:25428624:A:G:UTR3:GINS1	chr20	25428624	25428624	A	G	UTR3	GINS1	ENST000002	.	.	A	G	+	+	0.03597122	134,0,5,0	chr20,25428	Cytosol	A > G
chr20:25428646:A:G:UTR3:GINS1	chr20	25428646	25428646	A	G	UTR3	GINS1	ENST000002	.	.	A	G	+	+	0.19565217	111,0,27,0	chr20,25428	Cytosol	A > G
chr20:25428669:A:G:UTR3:GINS1	chr20	25428669	25428669	A	G	UTR3	GINS1	ENST000002	.	.	A	G	+	+	0.08064516	114,0,10,0	chr20,25428	Cytosol	A > G
chr20:25428695:A:G:UTR3:GINS1	chr20	25428695	25428695	A	G	UTR3	GINS1	ENST000002	.	.	A	G	+	+	0.02631579	148,0,4,0	chr20,25428	Cytosol	A > G
chr20:25428722:A:G:UTR3:GINS1	chr20	25428722	25428722	A	G	UTR3	GINS1	ENST000002	.	.	A	G	+	+	0.07189542	142,0,11,0	chr20,25428	Cytosol	A > G
chr20:25428724:A:G:UTR3:GINS1	chr20	25428724	25428724	A	G	UTR3	GINS1	ENST000002	.	.	A	G	+	+	0.27659574	102,0,39,0	chr20,25428	Cytosol	A > G
chr20:25428750:A:G:UTR3:GINS1	chr20	25428750	25428750	A	G	UTR3	GINS1	ENST000002	.	.	A	G	+	+	0.14285714	132,0,22,0	chr20,25428	Cytosol	A > G
chr20:25659310:A:G:intronic:ZNF3	chr20	25659310	25659310	A	G	intronic	ZNF337	.	.	.	A	G	+	-	0.25	9,0,3,0	chr20,25659	Cytosol	A > G
chr20:25659503:A:G:intronic:ZNF3	chr20	25659503	25659503	A	G	intronic	ZNF337	.	.	.	A	G	+	-	0.42857143	4,0,3,0	chr20,25659	Cytosol	A > G
chr20:25659566:A:G:intronic:ZNF3	chr20	25659566	25659566	A	G	intronic	ZNF337	.	.	.	A	G	+	-	0.30769231	9,0,4,0	chr20,25659	Cytosol	A > G
chr20:25663205:A:G:intronic:ZNF3	chr20	25663205	25663205	A	G	intronic	ZNF337	.	.	.	A	G	+	-	0.42857143	4,0,3,0	chr20,25663	Cytosol	A > G
chr20:30736266:A:G:intronic:TM9	chr20	30736266	30736266	A	G	intronic	TM9SF4	.	.	.	A	G	+	+	0.8	1,0,4,0	chr20,30736	Cytosol	A > G

chr20:30824220:A:G:UTR3:POFUT	chr20	30824220	30824220	A	G	UTR3	POFUT1	ENST000003	.	.	A	G	+	+	0.02439024	200,0,5,0	chr20,30824	Cytosol	A > G
chr20:30921278:A:G:UTR3:KIF3B	chr20	30921278	30921278	A	G	UTR3	KIF3B	ENST000003	.	.	A	G	+	+	0.02739726	71,0,2,0	chr20,30921	Cytosol	A > G
chr20:30921315:A:G:UTR3:KIF3B	chr20	30921315	30921315	A	G	UTR3	KIF3B	ENST000003	.	.	A	G	+	+	0.04166667	69,0,3,0	chr20,30921	Cytosol	A > G
chr20:30921351:A:G:UTR3:KIF3B	chr20	30921351	30921351	A	G	UTR3	KIF3B	ENST000003	.	.	A	G	+	+	0.03448276	56,0,2,0	chr20,30921	Cytosol	A > G
chr20:32000837:A:G:intronic:SNT	chr20	32000837	32000837	A	G	intronic	SNTA1	.	.	.	A	G	+	-	0.26666667	11,0,4,0	chr20,32000	Cytosol	A > G
chr20:32000840:A:G:intronic:SNT	chr20	32000840	32000840	A	G	intronic	SNTA1	.	.	.	A	G	+	-	0.66666667	4,0,8,0	chr20,32000	Cytosol	A > G
chr20:32001136:A:G:intronic:SNT	chr20	32001136	32001136	A	G	intronic	SNTA1	.	.	.	A	G	+	-	0.375	5,0,3,0	chr20,32001	Cytosol	A > G
chr20:32001640:A:G:intronic:SNT	chr20	32001640	32001640	A	G	intronic	SNTA1	.	.	.	A	G	+	-	0.6	4,0,6,0	chr20,32001	Cytosol	A > G
chr20:32001861:A:G:intronic:SNT	chr20	32001861	32001861	A	G	intronic	SNTA1	.	.	.	A	G	+	-	0.33333333	6,0,3,0	chr20,32001	Cytosol	A > G
chr20:32002208:A:G:intronic:SNT	chr20	32002208	32002208	A	G	intronic	SNTA1	.	.	.	A	G	+	-	0.5	3,0,3,0	chr20,32002	Cytosol	A > G
chr20:32234407:A:G:UTR3:CBFA2	chr20	32234407	32234407	A	G	UTR3	CBFA2T2	ENST000003	.	.	A	G	+	+	0.16666667	20,0,4,0	chr20,32234	Cytosol	A > G
chr20:32234467:A:G:UTR3:CBFA2	chr20	32234467	32234467	A	G	UTR3	CBFA2T2	ENST000003	.	.	A	G	+	+	0.44444444	5,0,4,0	chr20,32234	Cytosol	A > G
chr20:32234560:A:G:UTR3:CBFA2	chr20	32234560	32234560	A	G	UTR3	CBFA2T2	ENST000003	.	.	A	G	+	+	0.5	5,0,5,0	chr20,32234	Cytosol	A > G
chr20:32234574:A:G:UTR3:CBFA2	chr20	32234574	32234574	A	G	UTR3	CBFA2T2	ENST000003	.	.	A	G	+	+	0.81818182	2,0,9,0	chr20,32234	Cytosol	A > G
chr20:32234587:A:G:UTR3:CBFA2	chr20	32234587	32234587	A	G	UTR3	CBFA2T2	ENST000003	.	.	A	G	+	+	0.3	7,0,3,0	chr20,32234	Cytosol	A > G
chr20:32235190:A:G:UTR3:CBFA2	chr20	32235190	32235190	A	G	UTR3	CBFA2T2	ENST000003	.	.	A	G	+	+	0.13333333	13,0,2,0	chr20,32235	Cytosol	A > G
chr20:32235191:A:G:UTR3:CBFA2	chr20	32235191	32235191	A	G	UTR3	CBFA2T2	ENST000003	.	.	A	G	+	+	0.15384615	11,0,2,0	chr20,32235	Cytosol	A > G
chr20:32235220:A:G:UTR3:CBFA2	chr20	32235220	32235220	A	G	UTR3	CBFA2T2	ENST000003	.	.	A	G	+	+	0.25	12,0,4,0	chr20,32235	Cytosol	A > G
chr20:32235230:A:G:UTR3:CBFA2	chr20	32235230	32235230	A	G	UTR3	CBFA2T2	ENST000003	.	.	A	G	+	+	0.125	14,0,2,0	chr20,32235	Cytosol	A > G
chr20:32235273:A:G:UTR3:CBFA2	chr20	32235273	32235273	A	G	UTR3	CBFA2T2	ENST000003	.	.	A	G	+	+	0.54545455	5,0,6,0	chr20,32235	Cytosol	A > G
chr20:32292831:T:C:intergenic:E2	chr20	32292831	32292831	T	C	intergenic	E2F1,PXMP4	dist=18621;d	.	.	A	G	-	-	0.18181818	18,0,4,0	chr20,32292	Cytosol	A > G
chr20:32292856:T:C:intergenic:E2	chr20	32292856	32292856	T	C	intergenic	E2F1,PXMP4	dist=18646;d	.	.	A	G	-	-	0.68965517	9,0,20,0	chr20,32292	Cytosol	A > G
chr20:32292924:T:C:intergenic:E2	chr20	32292924	32292924	T	C	intergenic	E2F1,PXMP4	dist=18714;d	.	.	A	G	-	-	0.16666667	20,0,4,0	chr20,32292	Cytosol	A > G
chr20:32292934:T:C:intergenic:E2	chr20	32292934	32292934	T	C	intergenic	E2F1,PXMP4	dist=18724;d	.	.	A	G	-	-	0.26666667	11,0,4,0	chr20,32292	Cytosol	A > G
chr20:32292944:T:C:intergenic:E2	chr20	32292944	32292944	T	C	intergenic	E2F1,PXMP4	dist=18734;d	.	.	A	G	-	-	0.83333333	2,0,10,0	chr20,32292	Cytosol	A > G
chr20:32294119:T:C:downstream:	chr20	32294119	32294119	T	C	downstream	PXMP4	.	.	.	A	G	-	-	0.2	20,0,5,0	chr20,32294	Cytosol	A > G
chr20:32294141:T:C:downstream:	chr20	32294141	32294141	T	C	downstream	PXMP4	.	.	.	A	G	-	-	0.5	10,0,10,0	chr20,32294	Cytosol	A > G
chr20:32294153:T:C:downstream:	chr20	32294153	32294153	T	C	downstream	PXMP4	.	.	.	A	G	-	-	0.3	14,0,6,0	chr20,32294	Cytosol	A > G
chr20:32294180:T:C:downstream:	chr20	32294180	32294180	T	C	downstream	PXMP4	.	.	.	A	G	-	-	0.67741935	10,0,21,0	chr20,32294	Cytosol	A > G
chr20:32294181:T:C:downstream:	chr20	32294181	32294181	T	C	downstream	PXMP4	.	.	.	A	G	-	-	0.41935484	18,0,13,0	chr20,32294	Cytosol	A > G
chr20:32294187:T:C:downstream:	chr20	32294187	32294187	T	C	downstream	PXMP4	.	.	.	A	G	-	-	0.3125	22,0,10,0	chr20,32294	Cytosol	A > G
chr20:32294201:T:C:downstream:	chr20	32294201	32294201	T	C	downstream	PXMP4	.	.	.	A	G	-	-	0.27272727	24,0,9,0	chr20,32294	Cytosol	A > G
chr20:32862496:T:C:ncRNA_intror	chr20	32862496	32862496	T	C	ncRNA_intro	RP4-785G19.	.	.	.	A	G	-	-	0.75	5,0,15,0	chr20,32862	Cytosol	A > G
chr20:32862499:T:C:ncRNA_intror	chr20	32862499	32862499	T	C	ncRNA_intro	RP4-785G19.	.	.	.	A	G	-	-	0.38095238	13,0,8,0	chr20,32862	Cytosol	A > G
chr20:32862508:T:C:ncRNA_intror	chr20	32862508	32862508	T	C	ncRNA_intro	RP4-785G19.	.	.	.	A	G	-	-	0.52941176	8,0,9,0	chr20,32862	Cytosol	A > G
chr20:32862509:T:C:ncRNA_intror	chr20	32862509	32862509	T	C	ncRNA_intro	RP4-785G19.	.	.	.	A	G	-	-	0.27777778	13,0,5,0	chr20,32862	Cytosol	A > G
chr20:32862532:T:C:ncRNA_intror	chr20	32862532	32862532	T	C	ncRNA_intro	RP4-785G19.	.	.	.	A	G	-	-	0.22222222	7,0,2,0	chr20,32862	Cytosol	A > G
chr20:32862535:T:C:ncRNA_intror	chr20	32862535	32862535	T	C	ncRNA_intro	RP4-785G19.	.	.	.	A	G	-	-	0.5	4,0,4,0	chr20,32862	Cytosol	A > G
chr20:32862536:T:C:ncRNA_intror	chr20	32862536	32862536	T	C	ncRNA_intro	RP4-785G19.	.	.	.	A	G	-	-	0.42857143	4,0,3,0	chr20,32862	Cytosol	A > G
chr20:32862638:T:C:ncRNA_intror	chr20	32862638	32862638	T	C	ncRNA_intro	RP4-785G19.	.	.	.	A	G	-	-	1	0,0,16,0	chr20,32862	Cytosol	A > G
chr20:32862643:T:C:ncRNA_intror	chr20	32862643	32862643	T	C	ncRNA_intro	RP4-785G19.	.	.	.	A	G	-	-	0.46666667	8,0,7,0	chr20,32862	Cytosol	A > G
chr20:32862650:T:C:ncRNA_intror	chr20	32862650	32862650	T	C	ncRNA_intro	RP4-785G19.	.	.	.	A	G	-	-	0.57142857	6,0,8,0	chr20,32862	Cytosol	A > G
chr20:32862651:T:C:ncRNA_intror	chr20	32862651	32862651	T	C	ncRNA_intro	RP4-785G19.	.	.	.	A	G	-	-	0.4375	9,0,7,0	chr20,32862	Cytosol	A > G
chr20:32862652:T:C:ncRNA_intror	chr20	32862652	32862652	T	C	ncRNA_intro	RP4-785G19.	.	.	.	A	G	-	-	0.17647059	14,0,3,0	chr20,32862	Cytosol	A > G
chr20:32862677:T:C:ncRNA_intror	chr20	32862677	32862677	T	C	ncRNA_intro	RP4-785G19.	.	.	.	A	G	-	-	0.71428571	6,0,15,0	chr20,32862	Cytosol	A > G
chr20:32862678:T:C:ncRNA_intror	chr20	32862678	32862678	T	C	ncRNA_intro	RP4-785G19.	.	.	.	A	G	-	-	0.66666667	7,0,14,0	chr20,32862	Cytosol	A > G
chr20:32862683:T:C:ncRNA_intror	chr20	32862683	32862683	T	C	ncRNA_intro	RP4-785G19.	.	.	.	A	G	-	-	0.13043478	20,0,3,0	chr20,32862	Cytosol	A > G
chr20:32862698:T:C:ncRNA_intror	chr20	32862698	32862698	T	C	ncRNA_intro	RP4-785G19.	.	.	.	A	G	-	-	0.375	10,0,6,0	chr20,32862	Cytosol	A > G
chr20:32862744:T:C:ncRNA_intror	chr20	32862744	32862744	T	C	ncRNA_intro	RP4-785G19.	.	.	.	A	G	-	-	0.17647059	14,0,3,0	chr20,32862	Cytosol	A > G
chr20:32862750:T:C:ncRNA_intror	chr20	32862750	32862750	T	C	ncRNA_intro	RP4-785G19.	.	.	.	A	G	-	-	0.5	8,0,8,0	chr20,32862	Cytosol	A > G
chr20:32862856:T:C:ncRNA_intror	chr20	32862856	32862856	T	C	ncRNA_intro	RP4-785G19.	.	.	.	A	G	-	-	0.81818182	4,0,18,0	chr20,32862	Cytosol	A > G
chr20:32863175:T:C:ncRNA_intror	chr20	32863175	32863175	T	C	ncRNA_intro	RP4-785G19.	.	.	.	A	G	-	-	0.4	6,0,4,0	chr20,32863	Cytosol	A > G
chr20:34301191:T:C:intronic:RBM	chr20	34301191	34301191	T	C	intronic	RBM39	.	.	.	A	G	-	-	0.44444444	5,0,4,0	chr20,34301	Cytosol	A > G
chr20:34301192:T:C:intronic:RBM	chr20	34301192	34301192	T	C	intronic	RBM39	.	.	.	A	G	-	-	0.5	5,0,5,0	chr20,34301	Cytosol	A > G
chr20:35409773:T:C:UTR3:SOGA1	chr20	35409773	35409773	T	C	UTR3	SOGA1	ENST000002	.	.	A	G	-	-	0.88888889	1,0,8,0	chr20,35409	Cytosol	A > G
chr20:35409776:T:C:UTR3:SOGA1	chr20	35409776	35409776	T	C	UTR3	SOGA1	ENST000002	.	.	A	G	-	-	0.75	2,0,9,1	chr20,35409	Cytosol	A > G
chr20:35409817:T:C:UTR3:SOGA1	chr20	35409817	35409817	T	C	UTR3	SOGA1	ENST000002	.	.	A	G	-	-	0.7	6,0,14,0	chr20,35409	Cytosol	A > G
chr20:35409826:T:C:UTR3:SOGA1	chr20	35409826	35409826	T	C	UTR3	SOGA1	ENST000002	.	.	A	G	-	-	0.45454545	12,0,10,0	chr20,35409	Cytosol	A > G
chr20:35409838:T:C:UTR3:SOGA1	chr20	35409838	35409838	T	C	UTR3	SOGA1	ENST000002	.	.	A	G	-	-	0.27777778	13,0,5,0	chr20,35409	Cytosol	A > G
chr20:35409853:T:C:UTR3:SOGA1	chr20	35409853	35409853	T	C	UTR3	SOGA1	ENST000002	.	.	A	G	-	-	0.33333333	16,0,8,0	chr20,35409	Cytosol	A > G
chr20:35409856:T:C:UTR3:SOGA1	chr20	35409856	35409856	T	C	UTR3	SOGA1	ENST000002	.	.	A	G	-	-	0.32	17,0,8,0	chr20,35409	Cytosol	A > G
chr20:35410314:T:C:UTR3:SOGA1	chr20	35410314	35410314	T	C	UTR3	SOGA1	ENST000002	.	.	A	G	-	-	0.4	21,0,14,0	chr20,35410	Cytosol	A > G
chr20:35410369:T:C:UTR3:SOGA1	chr20	35410369	35410369	T	C	UTR3	SOGA1	ENST000002	.	.	A	G	-	-	0.4	9,0,6,0	chr20,35410	Cytosol	A > G
chr20:35410378:T:C:UTR3:SOGA1	chr20	35410378	35410378	T	C	UTR3	SOGA1	ENST000002	.	.	A	G	-	-	0.26666667	11,0,4,0	chr20,35410	Cytosol	A > G

chr20:35410379:T:C:UTR3:SOGA1	chr20	35410379	35410379	T	C	UTR3	SOGA1	ENST000002	.	.	A	G	-	-	0.5	8,0,8,0	chr20,35410	Cytosol	A > G
chr20:35410401:T:C:UTR3:SOGA1	chr20	35410401	35410401	T	C	UTR3	SOGA1	ENST000002	.	.	A	G	-	-	0.5	7,0,7,0	chr20,35410	Cytosol	A > G
chr20:35410405:T:C:UTR3:SOGA1	chr20	35410405	35410405	T	C	UTR3	SOGA1	ENST000002	.	.	A	G	-	-	0.46153846	7,0,6,0	chr20,35410	Cytosol	A > G
chr20:35410409:T:C:UTR3:SOGA1	chr20	35410409	35410409	T	C	UTR3	SOGA1	ENST000002	.	.	A	G	-	-	0.4	9,0,6,0	chr20,35410	Cytosol	A > G
chr20:35410419:T:C:UTR3:SOGA1	chr20	35410419	35410419	T	C	UTR3	SOGA1	ENST000002	.	.	A	G	-	-	0.125	14,0,2,0	chr20,35410	Cytosol	A > G
chr20:35410515:T:C:UTR3:SOGA1	chr20	35410515	35410515	T	C	UTR3	SOGA1	ENST000002	.	.	A	G	-	-	0.66666667	6,0,12,0	chr20,35410	Cytosol	A > G
chr20:35520977:T:C:UTR3:SAMHD	chr20	35520977	35520977	T	C	UTR3	SAMHD1	ENST000002	.	.	A	G	-	+-	0.65972222	49,0,95,0	chr20,35520	Cytosol	A > G
chr20:37257077:A:G:intronic:ARH	chr20	37257077	37257077	A	G	intronic	ARHGAP40	.	.	.	A	G	+	+	0.6	2,0,3,0	chr20,37257	Cytosol	A > G
chr20:42934260:T:C:UTR3:FITM2	chr20	42934260	42934260	T	C	UTR3	FITM2	ENST000003	.	.	A	G	-	-	0.6	2,0,3,0	chr20,42934	Cytosol	A > G
chr20:42934749:T:C:UTR3:FITM2	chr20	42934749	42934749	T	C	UTR3	FITM2	ENST000003	.	.	A	G	-	-	0.28571429	5,0,2,0	chr20,42934	Cytosol	A > G
chr20:42934768:T:C:UTR3:FITM2	chr20	42934768	42934768	T	C	UTR3	FITM2	ENST000003	.	.	A	G	-	-	0.25	6,0,2,0	chr20,42934	Cytosol	A > G
chr20:43706098:A:G:UTR3:STK4	chr20	43706098	43706098	A	G	UTR3	STK4	ENST000003	.	.	A	G	+	+	0.52173913	22,0,24,0	chr20,43706	Cytosol	A > G
chr20:43706101:A:G:UTR3:STK4	chr20	43706101	43706101	A	G	UTR3	STK4	ENST000003	.	.	A	G	+	+	0.06818182	41,0,3,0	chr20,43706	Cytosol	A > G
chr20:43706870:A:G:UTR3:STK4	chr20	43706870	43706870	A	G	UTR3	STK4	ENST000003	.	.	A	G	+	+	0.4	24,0,16,0	chr20,43706	Cytosol	A > G
chr20:43706940:A:G:UTR3:STK4	chr20	43706940	43706940	A	G	UTR3	STK4	ENST000003	.	.	A	G	+	+	0.24657534	55,0,18,0	chr20,43706	Cytosol	A > G
chr20:43706946:A:G:UTR3:STK4	chr20	43706946	43706946	A	G	UTR3	STK4	ENST000003	.	.	A	G	+	+	0.04347826	66,0,3,0	chr20,43706	Cytosol	A > G
chr20:43706947:A:G:UTR3:STK4	chr20	43706947	43706947	A	G	UTR3	STK4	ENST000003	.	.	A	G	+	+	0.63636364	24,0,42,0	chr20,43706	Cytosol	A > G
chr20:43706951:A:G:UTR3:STK4	chr20	43706951	43706951	A	G	UTR3	STK4	ENST000003	.	.	A	G	+	+	0.1969697	53,0,13,0	chr20,43706	Cytosol	A > G
chr20:43706988:A:G:UTR3:STK4	chr20	43706988	43706988	A	G	UTR3	STK4	ENST000003	.	.	A	G	+	+	0.05982906	110,0,7,0	chr20,43706	Cytosol	A > G
chr20:43707709:A:G:UTR3:STK4	chr20	43707709	43707709	A	G	UTR3	STK4	ENST000003	.	.	A	G	+	+	0.25925926	60,0,21,0	chr20,43707	Cytosol	A > G
chr20:43707759:A:G:UTR3:STK4	chr20	43707759	43707759	A	G	UTR3	STK4	ENST000003	.	.	A	G	+	+	0.05555556	102,0,6,0	chr20,43707	Cytosol	A > G
chr20:43707771:A:G:UTR3:STK4	chr20	43707771	43707771	A	G	UTR3	STK4	ENST000003	.	.	A	G	+	+	0.13333333	91,0,14,0	chr20,43707	Cytosol	A > G
chr20:43707776:A:G:UTR3:STK4	chr20	43707776	43707776	A	G	UTR3	STK4	ENST000003	.	.	A	G	+	+	0.73394495	29,0,80,0	chr20,43707	Cytosol	A > G
chr20:43707785:A:G:UTR3:STK4	chr20	43707785	43707785	A	G	UTR3	STK4	ENST000003	.	.	A	G	+	+	0.08	92,0,8,0	chr20,43707	Cytosol	A > G
chr20:43707790:A:G:UTR3:STK4	chr20	43707790	43707790	A	G	UTR3	STK4	ENST000003	.	.	A	G	+	+	0.03846154	100,0,4,0	chr20,43707	Cytosol	A > G
chr20:43707839:A:G:UTR3:STK4	chr20	43707839	43707839	A	G	UTR3	STK4	ENST000003	.	.	A	G	+	+	0.04716981	101,0,5,0	chr20,43707	Cytosol	A > G
chr20:43707842:A:G:UTR3:STK4	chr20	43707842	43707842	A	G	UTR3	STK4	ENST000003	.	.	A	G	+	+	0.48113208	55,0,51,0	chr20,43707	Cytosol	A > G
chr20:44513024:A:G:UTR3:ZSWIM	chr20	44513024	44513024	A	G	UTR3	ZSWIM1	ENST000003	.	.	A	G	+	+	0.76923077	3,0,10,0	chr20,44513	Cytosol	A > G
chr20:44513168:A:G:UTR3:ZSWIM	chr20	44513168	44513168	A	G	UTR3	ZSWIM1	ENST000003	.	.	A	G	+	+	0.33333333	4,0,2,0	chr20,44513	Cytosol	A > G
chr20:44513263:A:G:UTR3:ZSWIM	chr20	44513263	44513263	A	G	UTR3	ZSWIM1	ENST000003	.	.	A	G	+	+	0.875	1,0,7,0	chr20,44513	Cytosol	A > G
chr20:44513395:A:G:UTR3:ZSWIM	chr20	44513395	44513395	A	G	UTR3	ZSWIM1	ENST000003	.	.	A	G	+	+	0.38461538	8,0,5,0	chr20,44513	Cytosol	A > G
chr20:44513463:A:G:UTR3:ZSWIM	chr20	44513463	44513463	A	G	UTR3	ZSWIM1	ENST000003	.	.	A	G	+	+	0.57142857	3,0,4,0	chr20,44513	Cytosol	A > G
chr20:44513464:A:G:UTR3:ZSWIM	chr20	44513464	44513464	A	G	UTR3	ZSWIM1	ENST000003	.	.	A	G	+	+	0.42857143	4,0,3,0	chr20,44513	Cytosol	A > G
chr20:44513503:A:G:UTR3:ZSWIM	chr20	44513503	44513503	A	G	UTR3	ZSWIM1	ENST000003	.	.	A	G	+	+	0.75	2,0,6,0	chr20,44513	Cytosol	A > G
chr20:44513530:A:G:UTR3:ZSWIM	chr20	44513530	44513530	A	G	UTR3	ZSWIM1	ENST000003	.	.	A	G	+	+	0.9	1,0,9,0	chr20,44513	Cytosol	A > G
chr20:44513531:A:G:UTR3:ZSWIM	chr20	44513531	44513531	A	G	UTR3	ZSWIM1	ENST000003	.	.	A	G	+	+	0.7	3,0,7,0	chr20,44513	Cytosol	A > G
chr20:44513595:A:G:UTR3:ZSWIM	chr20	44513595	44513595	A	G	UTR3	ZSWIM1	ENST000003	.	.	A	G	+	+	0.96153846	1,0,25,0	chr20,44513	Cytosol	A > G
chr20:44524313:A:G:intronic:CTSA	chr20	44524313	44524313	A	G	intronic	CTSA	.	.	.	A	G	+	+	0.375	5,0,3,0	chr20,44524	Cytosol	A > G
chr20:48735434:T:C:intronic:TMEM	chr20	48735434	48735434	T	C	intronic	TMEM189,T	.	.	.	A	G	-	-	0.71428571	2,0,5,0	chr20,48735	Cytosol	A > G
chr20:48736686:T:C:intronic:TMEM	chr20	48736686	48736686	T	C	intronic	TMEM189,T	.	.	.	A	G	-	-	0.5	3,0,3,0	chr20,48736	Cytosol	A > G
chr20:48789787:A:G:ncRNA_exoni	chr20	48789787	48789787	A	G	ncRNA_exon	RP11-112L6.	.	.	.	A	G	+	+	0.27272727	8,0,3,0	chr20,48789	Cytosol	A > G
chr20:49494415:A:G:downstream:	chr20	49494415	49494415	A	G	downstream	BCAS4	.	.	.	A	G	+		0.28571429	5,0,2,0	chr20,49494	Cytosol	A > G
chr20:49494416:A:G:downstream:	chr20	49494416	49494416	A	G	downstream	BCAS4	.	.	.	A	G	+		0.33333333	4,0,2,0	chr20,49494	Cytosol	A > G
chr20:49498267:A:G:intergenic:BC	chr20	49498267	49498267	A	G	intergenic	BCAS4,ADNP	dist=4553;dis	.	.	A	G	+		0.6	2,0,3,0	chr20,49498	Cytosol	A > G
chr20:49498297:A:G:intergenic:BC	chr20	49498297	49498297	A	G	intergenic	BCAS4,ADNP	dist=4583;dis	.	.	A	G	+		0.6	2,0,3,0	chr20,49498	Cytosol	A > G
chr20:54963756:T:C:UTR5:AURKA	chr20	54963756	54963756	T	C	UTR5	AURKA	ENST000003	.	.	A	G	-	-	0.06521739	43,0,3,0	chr20,54963	Cytosol	A > G
chr20:54963785:T:C:UTR5:AURKA	chr20	54963785	54963785	T	C	UTR5	AURKA	ENST000003	.	.	A	G	-	-	0.07692308	24,0,2,0	chr20,54963	Cytosol	A > G
chr20:54963786:T:C:UTR5:AURKA	chr20	54963786	54963786	T	C	UTR5	AURKA	ENST000003	.	.	A	G	-	-	0.07407407	25,0,2,0	chr20,54963	Cytosol	A > G
chr20:54963816:T:C:UTR5:AURKA	chr20	54963816	54963816	T	C	UTR5	AURKA	ENST000003	.	.	A	G	-	-	0.26923077	57,0,21,0	chr20,54963	Cytosol	A > G
chr20:61483299:T:C:intronic:TCFL	chr20	61483299	61483299	T	C	intronic	TCFL5	.	.	.	A	G	-	-	0.13793103	25,0,4,0	chr20,61483	Cytosol	A > G
chr20:61483327:T:C:intronic:TCFL	chr20	61483327	61483327	T	C	intronic	TCFL5	.	.	.	A	G	-	-	0.04651163	41,0,2,0	chr20,61483	Cytosol	A > G
chr21:34636204:A:G:ncRNA_exoni	chr21	34636204	34636204	A	G	ncRNA_exon	IL10RB-AS1	.	.	.	A	G	+	+-	0.3030303	23,0,10,0	chr21,34636	Cytosol	A > G
chr21:34636253:A:G:ncRNA_exoni	chr21	34636253	34636253	A	G	ncRNA_exon	IL10RB-AS1	.	.	.	A	G	+	+-	0.74074074	7,0,20,0	chr21,34636	Cytosol	A > G
chr21:34636260:A:G:ncRNA_exoni	chr21	34636260	34636260	A	G	ncRNA_exon	IL10RB-AS1	.	.	.	A	G	+	+-	0.33333333	18,0,9,0	chr21,34636	Cytosol	A > G
chr21:34636293:A:G:ncRNA_exoni	chr21	34636293	34636293	A	G	ncRNA_exon	IL10RB-AS1	.	.	.	A	G	+	+-	0.17142857	29,0,6,0	chr21,34636	Cytosol	A > G
chr21:34636303:A:G:ncRNA_exoni	chr21	34636303	34636303	A	G	ncRNA_exon	IL10RB-AS1	.	.	.	A	G	+	+-	0.22222222	28,0,8,0	chr21,34636	Cytosol	A > G
chr21:34636347:A:G:ncRNA_exoni	chr21	34636347	34636347	A	G	ncRNA_exon	IL10RB-AS1	.	.	.	A	G	+	+-	0.15625	27,0,5,0	chr21,34636	Cytosol	A > G
chr21:34636361:A:G:ncRNA_exoni	chr21	34636361	34636361	A	G	ncRNA_exon	IL10RB-AS1	.	.	.	A	G	+	+-	0.66666667	12,0,24,0	chr21,34636	Cytosol	A > G
chr21:34636384:A:G:ncRNA_exoni	chr21	34636384	34636384	A	G	ncRNA_exon	IL10RB-AS1	.	.	.	A	G	+	+-	0.90243902	4,0,37,0	chr21,34636	Cytosol	A > G
chr21:34636385:A:G:ncRNA_exoni	chr21	34636385	34636385	A	G	ncRNA_exon	IL10RB-AS1	.	.	.	A	G	+	+-	0.34146341	27,0,14,0	chr21,34636	Cytosol	A > G
chr21:34637023:A:G:ncRNA_exoni	chr21	34637023	34637023	A	G	ncRNA_exon	IL10RB-AS1	.	.	.	A	G	+	+-	0.14285714	12,0,2,0	chr21,34637	Cytosol	A > G
chr21:34637146:A:G:ncRNA_exoni	chr21	34637146	34637146	A	G	ncRNA_exon	IL10RB-AS1	.	.	.	A	G	+	+-	0.11290323	55,0,7,0	chr21,34637	Cytosol	A > G
chr21:34637149:A:G:ncRNA_exoni	chr21	34637149	34637149	A	G	ncRNA_exon	IL10RB-AS1	.	.	.	A	G	+	+-	0.234375	49,0,15,0	chr21,34637	Cytosol	A > G
chr21:34637159:A:G:ncRNA_exoni	chr21	34637159	34637159	A	G	ncRNA_exon	IL10RB-AS1	.	.	.	A	G	+	+-	0.31818182	30,0,14,0	chr21,34637	Cytosol	A > G

chr21:34637208:A:G:ncRNA_exoni	chr21	34637208	34637208	A	G	ncRNA_exon	IL10RB-AS1	.	.	.	A	G	+	+-	0.28125	23,0,9,0	chr21,34637	Cytosol	A > G
chr21:34637216:A:G:ncRNA_exoni	chr21	34637216	34637216	A	G	ncRNA_exon	IL10RB-AS1	.	.	.	A	G	+	+-	0.37142857	22,0,13,0	chr21,34637	Cytosol	A > G
chr21:34637221:A:G:ncRNA_exoni	chr21	34637221	34637221	A	G	ncRNA_exon	IL10RB-AS1	.	.	.	A	G	+	+-	0.86842105	5,0,33,0	chr21,34637	Cytosol	A > G
chr21:34637225:A:G:ncRNA_exoni	chr21	34637225	34637225	A	G	ncRNA_exon	IL10RB-AS1	.	.	.	A	G	+	+-	0.80555556	7,0,29,0	chr21,34637	Cytosol	A > G
chr21:34728136:A:G:UTR3:IFNAR1	chr21	34728136	34728136	A	G	UTR3	IFNAR1	ENST000002	.	.	A	G	+	+	0.22916667	37,0,11,0	chr21,34728	Cytosol	A > G
chr21:34729906:A:G:UTR3:IFNAR1	chr21	34729906	34729906	A	G	UTR3	IFNAR1	ENST000002	.	.	A	G	+	+	0.28571429	5,0,2,0	chr21,34729	Cytosol	A > G
chr21:34729931:A:G:UTR3:IFNAR1	chr21	34729931	34729931	A	G	UTR3	IFNAR1	ENST000002	.	.	A	G	+	+	0.31578947	13,0,6,0	chr21,34729	Cytosol	A > G
chr21:34729934:A:G:UTR3:IFNAR1	chr21	34729934	34729934	A	G	UTR3	IFNAR1	ENST000002	.	.	A	G	+	+	0.27777778	13,0,5,0	chr21,34729	Cytosol	A > G
chr21:34729959:A:G:UTR3:IFNAR1	chr21	34729959	34729959	A	G	UTR3	IFNAR1	ENST000002	.	.	A	G	+	+	0.61904762	16,0,26,0	chr21,34729	Cytosol	A > G
chr21:34729969:A:G:UTR3:IFNAR1	chr21	34729969	34729969	A	G	UTR3	IFNAR1	ENST000002	.	.	A	G	+	+	0.475	21,0,19,0	chr21,34729	Cytosol	A > G
chr21:34729971:A:G:UTR3:IFNAR1	chr21	34729971	34729971	A	G	UTR3	IFNAR1	ENST000002	.	.	A	G	+	+	0.31707317	28,0,13,0	chr21,34729	Cytosol	A > G
chr21:34730010:A:G:UTR3:IFNAR1	chr21	34730010	34730010	A	G	UTR3	IFNAR1	ENST000002	.	.	A	G	+	+	0.06060606	31,0,2,0	chr21,34730	Cytosol	A > G
chr21:34730016:A:G:UTR3:IFNAR1	chr21	34730016	34730016	A	G	UTR3	IFNAR1	ENST000002	.	.	A	G	+	+	0.22580645	24,0,7,0	chr21,34730	Cytosol	A > G
chr21:34730019:A:G:UTR3:IFNAR1	chr21	34730019	34730019	A	G	UTR3	IFNAR1	ENST000002	.	.	A	G	+	+	0.32142857	19,0,9,0	chr21,34730	Cytosol	A > G
chr21:34730033:A:G:UTR3:IFNAR1	chr21	34730033	34730033	A	G	UTR3	IFNAR1	ENST000002	.	.	A	G	+	+	0.33333333	16,0,8,0	chr21,34730	Cytosol	A > G
chr21:34730065:A:G:UTR3:IFNAR1	chr21	34730065	34730065	A	G	UTR3	IFNAR1	ENST000002	.	.	A	G	+	+	0.08333333	33,0,3,0	chr21,34730	Cytosol	A > G
chr21:34730077:A:G:UTR3:IFNAR1	chr21	34730077	34730077	A	G	UTR3	IFNAR1	ENST000002	.	.	A	G	+	+	0.17948718	32,0,7,0	chr21,34730	Cytosol	A > G
chr21:34730095:A:G:UTR3:IFNAR1	chr21	34730095	34730095	A	G	UTR3	IFNAR1	ENST000002	.	.	A	G	+	+	0.33333333	34,0,17,0	chr21,34730	Cytosol	A > G
chr21:34730096:A:G:UTR3:IFNAR1	chr21	34730096	34730096	A	G	UTR3	IFNAR1	ENST000002	.	.	A	G	+	+	0.5	24,0,24,0	chr21,34730	Cytosol	A > G
chr21:34730108:A:G:UTR3:IFNAR1	chr21	34730108	34730108	A	G	UTR3	IFNAR1	ENST000002	.	.	A	G	+	+	0.54901961	23,0,28,0	chr21,34730	Cytosol	A > G
chr21:34731077:A:G:UTR3:IFNAR1	chr21	34731077	34731077	A	G	UTR3	IFNAR1	ENST000002	.	.	A	G	+	+	0.0862069	53,0,5,0	chr21,34731	Cytosol	A > G
chr21:34805286:A:G:intronic:IFNG	chr21	34805286	34805286	A	G	intronic	IFNGR2,TME	.	.	.	A	G	+	+-	0.4	3,0,2,0	chr21,34805	Cytosol	A > G
chr21:34923319:A:G:exonic:SON	chr21	34923319	34923319	A	G	exonic	SON	.	synonymous	SON:ENST00	A	G	+	+	0.09170306	208,0,21,0	chr21,34923	Cytosol	A > G
chr21:34965101:T:C:intronic:AP00	chr21	34965101	34965101	T	C	intronic	AP000304.12	.	.	.	A	G	-	-	0.57142857	3,0,4,0	chr21,34965	Cytosol	A > G
chr21:34965112:T:C:intronic:AP00	chr21	34965112	34965112	T	C	intronic	AP000304.12	.	.	.	A	G	-	-	0.28571429	5,0,2,0	chr21,34965	Cytosol	A > G
chr21:34965113:T:C:intronic:AP00	chr21	34965113	34965113	T	C	intronic	AP000304.12	.	.	.	A	G	-	-	0.71428571	2,0,5,0	chr21,34965	Cytosol	A > G
chr21:37789515:A:G:UTR3:CHAF1I	chr21	37789515	37789515	A	G	UTR3	CHAF1B	ENST000003	.	.	A	G	+	+	0.14285714	24,0,4,0	chr21,37789	Cytosol	A > G
chr21:44263581:T:C:intronic:WDR	chr21	44263581	44263581	T	C	intronic	WDR4	.	.	.	A	G	-	-	0.41818182	32,0,23,0	chr21,44263	Cytosol	A > G
chr21:44263593:T:C:intronic:WDR	chr21	44263593	44263593	T	C	intronic	WDR4	.	.	.	A	G	-	-	0.08510638	43,0,4,0	chr21,44263	Cytosol	A > G
chr21:44263594:T:C:intronic:WDR	chr21	44263594	44263594	T	C	intronic	WDR4	.	.	.	A	G	-	-	0.29411765	36,0,15,0	chr21,44263	Cytosol	A > G
chr21:44263623:T:C:intronic:WDR	chr21	44263623	44263623	T	C	intronic	WDR4	.	.	.	A	G	-	-	0.16	21,0,4,0	chr21,44263	Cytosol	A > G
chr21:44263659:T:C:intronic:WDR	chr21	44263659	44263659	T	C	intronic	WDR4	.	.	.	A	G	-	-	0.46153846	7,0,6,0	chr21,44263	Cytosol	A > G
chr21:44263714:T:C:intronic:WDR	chr21	44263714	44263714	T	C	intronic	WDR4	.	.	.	A	G	-	-	0.44444444	5,0,4,0	chr21,44263	Cytosol	A > G
chr21:44263798:T:C:intronic:WDR	chr21	44263798	44263798	T	C	intronic	WDR4	.	.	.	A	G	-	-	0.53846154	6,0,7,0	chr21,44263	Cytosol	A > G
chr21:44263800:T:C:intronic:WDR	chr21	44263800	44263800	T	C	intronic	WDR4	.	.	.	A	G	-	-	0.4375	9,0,7,0	chr21,44263	Cytosol	A > G
chr21:44263856:T:C:intronic:WDR	chr21	44263856	44263856	T	C	intronic	WDR4	.	.	.	A	G	-	-	0.04255319	45,0,2,0	chr21,44263	Cytosol	A > G
chr21:44263859:T:C:intronic:WDR	chr21	44263859	44263859	T	C	intronic	WDR4	.	.	.	A	G	-	-	0.18604651	35,0,8,0	chr21,44263	Cytosol	A > G
chr21:44263870:T:C:intronic:WDR	chr21	44263870	44263870	T	C	intronic	WDR4	.	.	.	A	G	-	-	0.4047619	25,0,17,0	chr21,44263	Cytosol	A > G
chr21:44263909:T:C:intronic:WDR	chr21	44263909	44263909	T	C	intronic	WDR4	.	.	.	A	G	-	-	0.09803922	46,0,5,0	chr21,44263	Cytosol	A > G
chr21:44263941:T:C:intronic:WDR	chr21	44263941	44263941	T	C	intronic	WDR4	.	.	.	A	G	-	-	0.05172414	55,0,3,0	chr21,44263	Cytosol	A > G
chr21:44264056:T:C:intronic:WDR	chr21	44264056	44264056	T	C	intronic	WDR4	.	.	.	A	G	-	-	0.57142857	21,0,28,0	chr21,44264	Cytosol	A > G
chr21:44265998:T:C:intronic:WDR	chr21	44265998	44265998	T	C	intronic	WDR4	.	.	.	A	G	-	-	0.12765957	41,0,6,0	chr21,44265	Cytosol	A > G
chr21:44265999:T:C:intronic:WDR	chr21	44265999	44265999	T	C	intronic	WDR4	.	.	.	A	G	-	-	0.17391304	38,0,8,0	chr21,44265	Cytosol	A > G
chr21:44266022:T:C:intronic:WDR	chr21	44266022	44266022	T	C	intronic	WDR4	.	.	.	A	G	-	-	0.15686275	43,0,8,0	chr21,44266	Cytosol	A > G
chr21:44266026:T:C:intronic:WDR	chr21	44266026	44266026	T	C	intronic	WDR4	.	.	.	A	G	-	-	0.10416667	43,0,5,0	chr21,44266	Cytosol	A > G
chr21:44266142:T:C:intronic:WDR	chr21	44266142	44266142	T	C	intronic	WDR4	.	.	.	A	G	-	-	0.48571429	18,0,17,0	chr21,44266	Cytosol	A > G
chr21:44266235:T:C:intronic:WDR	chr21	44266235	44266235	T	C	intronic	WDR4	.	.	.	A	G	-	-	0.09375	29,0,3,0	chr21,44266	Cytosol	A > G
chr21:44266887:T:C:intronic:WDR	chr21	44266887	44266887	T	C	intronic	WDR4	.	.	.	A	G	-	-	0.33333333	26,0,13,0	chr21,44266	Cytosol	A > G
chr21:44266940:T:C:intronic:WDR	chr21	44266940	44266940	T	C	intronic	WDR4	.	.	.	A	G	-	-	0.29411765	24,0,10,0	chr21,44266	Cytosol	A > G
chr21:44266949:T:C:intronic:WDR	chr21	44266949	44266949	T	C	intronic	WDR4	.	.	.	A	G	-	-	0.175	33,0,7,0	chr21,44266	Cytosol	A > G
chr21:44266969:T:C:intronic:WDR	chr21	44266969	44266969	T	C	intronic	WDR4	.	.	.	A	G	-	-	0.125	21,0,3,0	chr21,44266	Cytosol	A > G
chr21:44267010:T:C:intronic:WDR	chr21	44267010	44267010	T	C	intronic	WDR4	.	.	.	A	G	-	-	0.19230769	21,0,5,0	chr21,44267	Cytosol	A > G
chr21:44267036:T:C:intronic:WDR	chr21	44267036	44267036	T	C	intronic	WDR4	.	.	.	A	G	-	-	0.33333333	12,0,6,0	chr21,44267	Cytosol	A > G
chr21:44267048:T:C:intronic:WDR	chr21	44267048	44267048	T	C	intronic	WDR4	.	.	.	A	G	-	-	0.58823529	7,0,10,0	chr21,44267	Cytosol	A > G
chr21:44267102:T:C:intronic:WDR	chr21	44267102	44267102	T	C	intronic	WDR4	.	.	.	A	G	-	-	0.375	5,0,3,0	chr21,44267	Cytosol	A > G
chr21:44267104:T:C:intronic:WDR	chr21	44267104	44267104	T	C	intronic	WDR4	.	.	.	A	G	-	-	0.75	2,0,6,0	chr21,44267	Cytosol	A > G
chr21:44329283:A:G:UTR3:NDUFV	chr21	44329283	44329283	A	G	UTR3	NDUFV3	ENST000003	.	.	A	G	+	+	0.11111111	16,0,2,0	chr21,44329	Cytosol	A > G
chr21:44329364:A:G:UTR3:NDUFV	chr21	44329364	44329364	A	G	UTR3	NDUFV3	ENST000003	.	.	A	G	+	+	0.06451613	29,0,2,0	chr21,44329	Cytosol	A > G
chr21:44329377:A:G:UTR3:NDUFV	chr21	44329377	44329377	A	G	UTR3	NDUFV3	ENST000003	.	.	A	G	+	+	0.07894737	35,0,3,0	chr21,44329	Cytosol	A > G
chr21:44329378:A:G:UTR3:NDUFV	chr21	44329378	44329378	A	G	UTR3	NDUFV3	ENST000003	.	.	A	G	+	+	0.20588235	27,0,7,0	chr21,44329	Cytosol	A > G
chr21:44329381:A:G:UTR3:NDUFV	chr21	44329381	44329381	A	G	UTR3	NDUFV3	ENST000003	.	.	A	G	+	+	0.05714286	33,0,2,0	chr21,44329	Cytosol	A > G
chr21:44329391:A:G:UTR3:NDUFV	chr21	44329391	44329391	A	G	UTR3	NDUFV3	ENST000003	.	.	A	G	+	+	0.04878049	39,0,2,0	chr21,44329	Cytosol	A > G
chr21:44329417:A:G:UTR3:NDUFV	chr21	44329417	44329417	A	G	UTR3	NDUFV3	ENST000003	.	.	A	G	+	+	0.06060606	31,0,2,0	chr21,44329	Cytosol	A > G
chr21:44329432:A:G:UTR3:NDUFV	chr21	44329432	44329432	A	G	UTR3	NDUFV3	ENST000003	.	.	A	G	+	+	0.11111111	32,0,4,0	chr21,44329	Cytosol	A > G

chr21:44329435:A:G:UTR3:NDUFV	chr21	44329435	44329435	A	G	UTR3	NDUFV3	ENST000003	.	.	A	G	+	+	0.06451613	29,0,2,0	chr21,44329	Cytosol	A > G
chr21:44329526:A:G:UTR3:NDUFV	chr21	44329526	44329526	A	G	UTR3	NDUFV3	ENST000003	.	.	A	G	+	+	0.04545455	42,0,2,0	chr21,44329	Cytosol	A > G
chr21:44329853:A:G:UTR3:NDUFV	chr21	44329853	44329853	A	G	UTR3	NDUFV3	ENST000003	.	.	A	G	+	+	0.125	28,0,4,0	chr21,44329	Cytosol	A > G
chr21:44329870:A:G:UTR3:NDUFV	chr21	44329870	44329870	A	G	UTR3	NDUFV3	ENST000003	.	.	A	G	+	+	0.36666667	19,0,11,0	chr21,44329	Cytosol	A > G
chr21:44329889:A:G:UTR3:NDUFV	chr21	44329889	44329889	A	G	UTR3	NDUFV3	ENST000003	.	.	A	G	+	+	0.06451613	29,0,2,0	chr21,44329	Cytosol	A > G
chr21:44329933:A:G:UTR3:NDUFV	chr21	44329933	44329933	A	G	UTR3	NDUFV3	ENST000003	.	.	A	G	+	+	0.15789474	16,0,3,0	chr21,44329	Cytosol	A > G
chr21:44329997:A:G:UTR3:NDUFV	chr21	44329997	44329997	A	G	UTR3	NDUFV3	ENST000003	.	.	A	G	+	+	0.35714286	9,0,5,0	chr21,44329	Cytosol	A > G
chr21:44330047:A:G:UTR3:NDUFV	chr21	44330047	44330047	A	G	UTR3	NDUFV3	ENST000003	.	.	A	G	+	+	0.28571429	15,0,6,0	chr21,44330	Cytosol	A > G
chr21:44330181:A:G:UTR3:NDUFV	chr21	44330181	44330181	A	G	UTR3	NDUFV3	ENST000003	.	.	A	G	+	+	0.29032258	22,0,9,0	chr21,44330	Cytosol	A > G
chr21:44330350:A:G:UTR3:NDUFV	chr21	44330350	44330350	A	G	UTR3	NDUFV3	ENST000003	.	.	A	G	+	+	0.41666667	7,0,5,0	chr21,44330	Cytosol	A > G
chr21:44331297:A:G:UTR3:NDUFV	chr21	44331297	44331297	A	G	UTR3	NDUFV3	ENST000003	.	.	A	G	+	+	0.76923077	3,0,10,0	chr21,44331	Cytosol	A > G
chr21:44331621:A:G:UTR3:NDUFV	chr21	44331621	44331621	A	G	UTR3	NDUFV3	ENST000003	.	.	A	G	+	+	0.85714286	2,0,12,0	chr21,44331	Cytosol	A > G
chr21:44331645:A:G:UTR3:NDUFV	chr21	44331645	44331645	A	G	UTR3	NDUFV3	ENST000003	.	.	A	G	+	+	0.85714286	2,0,12,0	chr21,44331	Cytosol	A > G
chr21:44331652:A:G:UTR3:NDUFV	chr21	44331652	44331652	A	G	UTR3	NDUFV3	ENST000003	.	.	A	G	+	+	0.30769231	9,0,4,0	chr21,44331	Cytosol	A > G
chr21:44331655:A:G:UTR3:NDUFV	chr21	44331655	44331655	A	G	UTR3	NDUFV3	ENST000003	.	.	A	G	+	+	0.6	6,0,9,0	chr21,44331	Cytosol	A > G
chr21:44331717:A:G:UTR3:NDUFV	chr21	44331717	44331717	A	G	UTR3	NDUFV3	ENST000003	.	.	A	G	+	+	0.375	10,0,6,0	chr21,44331	Cytosol	A > G
chr21:45643801:T:C:UTR3:ICOSLG	chr21	45643801	45643801	T	C	UTR3	ICOSLG	ENST000004	.	.	A	G	-	-	0.17647059	14,0,3,0	chr21,45643	Cytosol	A > G
chr21:45643865:T:C:UTR3:ICOSLG	chr21	45643865	45643865	T	C	UTR3	ICOSLG	ENST000004	.	.	A	G	-	-	0.33333333	4,0,2,0	chr21,45643	Cytosol	A > G
chr21:45643901:T:C:UTR3:ICOSLG	chr21	45643901	45643901	T	C	UTR3	ICOSLG	ENST000004	.	.	A	G	-	-	0.21428571	11,0,3,0	chr21,45643	Cytosol	A > G
chr21:45643911:T:C:UTR3:ICOSLG	chr21	45643911	45643911	T	C	UTR3	ICOSLG	ENST000004	.	.	A	G	-	-	0.8125	3,0,13,0	chr21,45643	Cytosol	A > G
chr21:45643975:T:C:UTR3:ICOSLG	chr21	45643975	45643975	T	C	UTR3	ICOSLG	ENST000004	.	.	A	G	-	-	0.5	5,0,5,0	chr21,45643	Cytosol	A > G
chr21:45644102:T:C:UTR3:ICOSLG	chr21	45644102	45644102	T	C	UTR3	ICOSLG	ENST000004	.	.	A	G	-	-	0.25	9,0,3,0	chr21,45644	Cytosol	A > G
chr21:45644103:T:C:UTR3:ICOSLG	chr21	45644103	45644103	T	C	UTR3	ICOSLG	ENST000004	.	.	A	G	-	-	0.45454545	6,0,5,0	chr21,45644	Cytosol	A > G
chr21:45644164:T:C:UTR3:ICOSLG	chr21	45644164	45644164	T	C	UTR3	ICOSLG	ENST000004	.	.	A	G	-	-	0.375	5,0,3,0	chr21,45644	Cytosol	A > G
chr21:45644177:T:C:UTR3:ICOSLG	chr21	45644177	45644177	T	C	UTR3	ICOSLG	ENST000004	.	.	A	G	-	-	0.44444444	5,0,4,0	chr21,45644	Cytosol	A > G
chr21:45644330:T:C:UTR3:ICOSLG	chr21	45644330	45644330	T	C	UTR3	ICOSLG	ENST000004	.	.	A	G	-	-	0.5	4,0,4,0	chr21,45644	Cytosol	A > G
chr21:45644342:T:C:UTR3:ICOSLG	chr21	45644342	45644342	T	C	UTR3	ICOSLG	ENST000004	.	.	A	G	-	-	0.375	5,0,3,0	chr21,45644	Cytosol	A > G
chr21:45644343:T:C:UTR3:ICOSLG	chr21	45644343	45644343	T	C	UTR3	ICOSLG	ENST000004	.	.	A	G	-	-	0.25	6,0,2,0	chr21,45644	Cytosol	A > G
chr21:45644368:T:C:UTR3:ICOSLG	chr21	45644368	45644368	T	C	UTR3	ICOSLG	ENST000004	.	.	A	G	-	-	0.28571429	5,0,2,0	chr21,45644	Cytosol	A > G
chr21:45644369:T:C:UTR3:ICOSLG	chr21	45644369	45644369	T	C	UTR3	ICOSLG	ENST000004	.	.	A	G	-	-	0.83333333	1,0,5,0	chr21,45644	Cytosol	A > G
chr21:45644370:T:C:UTR3:ICOSLG	chr21	45644370	45644370	T	C	UTR3	ICOSLG	ENST000004	.	.	A	G	-	-	0.42857143	4,0,3,0	chr21,45644	Cytosol	A > G
chr21:45644384:T:C:UTR3:ICOSLG	chr21	45644384	45644384	T	C	UTR3	ICOSLG	ENST000004	.	.	A	G	-	-	0.5	3,0,3,0	chr21,45644	Cytosol	A > G
chr21:45644407:T:C:UTR3:ICOSLG	chr21	45644407	45644407	T	C	UTR3	ICOSLG	ENST000004	.	.	A	G	-	-	0.4	3,0,2,0	chr21,45644	Cytosol	A > G
chr21:45644472:T:C:UTR3:ICOSLG	chr21	45644472	45644472	T	C	UTR3	ICOSLG	ENST000004	.	.	A	G	-	-	0.90909091	1,0,10,0	chr21,45644	Cytosol	A > G
chr21:45644475:T:C:UTR3:ICOSLG	chr21	45644475	45644475	T	C	UTR3	ICOSLG	ENST000004	.	.	A	G	-	-	0.22222222	7,0,2,0	chr21,45644	Cytosol	A > G
chr21:45644518:T:C:UTR3:ICOSLG	chr21	45644518	45644518	T	C	UTR3	ICOSLG	ENST000004	.	.	A	G	-	-	0.13333333	13,0,2,0	chr21,45644	Cytosol	A > G
chr21:45644519:T:C:UTR3:ICOSLG	chr21	45644519	45644519	T	C	UTR3	ICOSLG	ENST000004	.	.	A	G	-	-	0.21428571	11,0,3,0	chr21,45644	Cytosol	A > G
chr21:45644562:T:C:UTR3:ICOSLG	chr21	45644562	45644562	T	C	UTR3	ICOSLG	ENST000004	.	.	A	G	-	-	0.91666667	1,0,11,0	chr21,45644	Cytosol	A > G
chr21:45644567:T:C:UTR3:ICOSLG	chr21	45644567	45644567	T	C	UTR3	ICOSLG	ENST000004	.	.	A	G	-	-	0.38461538	8,0,5,0	chr21,45644	Cytosol	A > G
chr21:47667742:A:G:ncRNA_intro	chr21	47667742	47667742	A	G	ncRNA_intro	MCM3AP-AS	.	.	.	A	G	+	-+	0.66666667	2,0,4,0	chr21,47667	Cytosol	A > G
chr21:47669202:A:G:ncRNA_intro	chr21	47669202	47669202	A	G	ncRNA_intro	MCM3AP-AS	.	.	.	A	G	+	-+	0.45454545	6,0,5,0	chr21,47669	Cytosol	A > G
chr21:47669206:A:G:ncRNA_intro	chr21	47669206	47669206	A	G	ncRNA_intro	MCM3AP-AS	.	.	.	A	G	+	-+	0.27272727	8,0,3,0	chr21,47669	Cytosol	A > G
chr21:47669235:A:G:ncRNA_intro	chr21	47669235	47669235	A	G	ncRNA_intro	MCM3AP-AS	.	.	.	A	G	+	-+	0.16666667	10,0,2,0	chr21,47669	Cytosol	A > G
chr21:47671910:A:G:ncRNA_exoni	chr21	47671910	47671910	A	G	ncRNA_exon	AP001469.9	.	.	.	A	G	+	+-	0.5	5,0,5,0	chr21,47671	Cytosol	A > G
chr21:47672001:A:G:ncRNA_exoni	chr21	47672001	47672001	A	G	ncRNA_exon	AP001469.9	.	.	.	A	G	+	+-	0.4	3,0,2,0	chr21,47672	Cytosol	A > G
chr21:47672483:A:G:ncRNA_exoni	chr21	47672483	47672483	A	G	ncRNA_exon	AP001469.9	.	.	.	A	G	+	+-	0.4	3,0,2,0	chr21,47672	Cytosol	A > G
chr21:47672558:A:G:ncRNA_exoni	chr21	47672558	47672558	A	G	ncRNA_exon	AP001469.9	.	.	.	A	G	+	+-	0.16666667	20,0,4,0	chr21,47672	Cytosol	A > G
chr21:47672832:A:G:ncRNA_exoni	chr21	47672832	47672832	A	G	ncRNA_exon	AP001469.9	.	.	.	A	G	+	+-	0.22222222	7,0,2,0	chr21,47672	Cytosol	A > G
chr21:47672855:A:G:ncRNA_exoni	chr21	47672855	47672855	A	G	ncRNA_exon	AP001469.9	.	.	.	A	G	+	+-	0.36363636	7,0,4,0	chr21,47672	Cytosol	A > G
chr21:47672856:A:G:ncRNA_exoni	chr21	47672856	47672856	A	G	ncRNA_exon	AP001469.9	.	.	.	A	G	+	+-	0.3	7,0,3,0	chr21,47672	Cytosol	A > G
chr21:47672932:A:G:ncRNA_exoni	chr21	47672932	47672932	A	G	ncRNA_exon	AP001469.9	.	.	.	A	G	+	+-	0.57142857	3,0,4,0	chr21,47672	Cytosol	A > G
chr21:47688836:T:C:intronic:MCM	chr21	47688836	47688836	T	C	intronic	MCM3AP	.	.	.	A	G	-	-	1	0,0,5,0	chr21,47688	Cytosol	A > G
chr21:47741179:T:C:intronic:C21o	chr21	47741179	47741179	T	C	intronic	C21orf58	.	.	.	A	G	-	-	0.07692308	24,0,2,0	chr21,47741	Cytosol	A > G
chr21:47741211:T:C:intronic:C21o	chr21	47741211	47741211	T	C	intronic	C21orf58	.	.	.	A	G	-	-	0.0625	30,0,2,0	chr21,47741	Cytosol	A > G
chr21:47741221:T:C:intronic:C21o	chr21	47741221	47741221	T	C	intronic	C21orf58	.	.	.	A	G	-	-	0.10810811	33,0,4,0	chr21,47741	Cytosol	A > G
chr21:47741227:T:C:intronic:C21o	chr21	47741227	47741227	T	C	intronic	C21orf58	.	.	.	A	G	-	-	0.07894737	35,0,3,0	chr21,47741	Cytosol	A > G
chr22:17591267:A:G:UTR3:IL17RA	chr22	17591267	17591267	A	G	UTR3	IL17RA	ENST000003	.	.	A	G	+	+	0.33333333	10,0,5,0	chr22,17591	Cytosol	A > G
chr22:17594105:A:G:UTR3:IL17RA	chr22	17594105	17594105	A	G	UTR3	IL17RA	ENST000003	.	.	A	G	+	+	0.21052632	15,0,4,0	chr22,17594	Cytosol	A > G
chr22:17594137:A:G:UTR3:IL17RA	chr22	17594137	17594137	A	G	UTR3	IL17RA	ENST000003	.	.	A	G	+	+	0.625	6,0,10,0	chr22,17594	Cytosol	A > G
chr22:17594148:A:G:UTR3:IL17RA	chr22	17594148	17594148	A	G	UTR3	IL17RA	ENST000003	.	.	A	G	+	+	0.35294118	11,0,6,0	chr22,17594	Cytosol	A > G
chr22:17594151:A:G:UTR3:IL17RA	chr22	17594151	17594151	A	G	UTR3	IL17RA	ENST000003	.	.	A	G	+	+	0.26666667	11,0,4,0	chr22,17594	Cytosol	A > G
chr22:17594158:A:G:UTR3:IL17RA	chr22	17594158	17594158	A	G	UTR3	IL17RA	ENST000003	.	.	A	G	+	+	0.3125	11,0,5,0	chr22,17594	Cytosol	A > G
chr22:17594222:A:G:UTR3:IL17RA	chr22	17594222	17594222	A	G	UTR3	IL17RA	ENST000003	.	.	A	G	+	+	0.6	8,0,12,0	chr22,17594	Cytosol	A > G
chr22:17594224:A:G:UTR3:IL17RA	chr22	17594224	17594224	A	G	UTR3	IL17RA	ENST000003	.	.	A	G	+	+	0.1	18,0,2,0	chr22,17594	Cytosol	A > G

chr22:17594270:A:G:UTR3:IL17RA	chr22	17594270	17594270	A	G	UTR3	IL17RA	ENST000003	.	.	A	G	+	+	0.6875	5,0,11,0	chr22,17594	Cytosol	A > G
chr22:17594273:A:G:UTR3:IL17RA	chr22	17594273	17594273	A	G	UTR3	IL17RA	ENST000003	.	.	A	G	+	+	0.38461538	8,0,5,0	chr22,17594	Cytosol	A > G
chr22:18185842:A:G:UTR3:BCL2L1	chr22	18185842	18185842	A	G	UTR3	BCL2L13	ENST000004	.	.	A	G	+	+	0.28571429	5,0,2,0	chr22,18185	Cytosol	A > G
chr22:18571277:A:G:UTR3:PEX26	chr22	18571277	18571277	A	G	UTR3	PEX26	ENST000003	.	.	A	G	+	+	0.0625	45,0,3,0	chr22,18571	Cytosol	A > G
chr22:18571281:A:G:UTR3:PEX26	chr22	18571281	18571281	A	G	UTR3	PEX26	ENST000003	.	.	A	G	+	+	0.06122449	46,0,3,0	chr22,18571	Cytosol	A > G
chr22:18571289:A:G:UTR3:PEX26	chr22	18571289	18571289	A	G	UTR3	PEX26	ENST000003	.	.	A	G	+	+	0.03448276	56,0,2,0	chr22,18571	Cytosol	A > G
chr22:18571338:A:G:UTR3:PEX26	chr22	18571338	18571338	A	G	UTR3	PEX26	ENST000003	.	.	A	G	+	+	0.21153846	41,0,11,0	chr22,18571	Cytosol	A > G
chr22:18571343:A:G:UTR3:PEX26	chr22	18571343	18571343	A	G	UTR3	PEX26	ENST000003	.	.	A	G	+	+	0.22222222	42,0,12,0	chr22,18571	Cytosol	A > G
chr22:18571345:A:G:UTR3:PEX26	chr22	18571345	18571345	A	G	UTR3	PEX26	ENST000003	.	.	A	G	+	+	0.17241379	48,0,10,0	chr22,18571	Cytosol	A > G
chr22:18571367:A:G:UTR3:PEX26	chr22	18571367	18571367	A	G	UTR3	PEX26	ENST000003	.	.	A	G	+	+	0.04	48,0,2,0	chr22,18571	Cytosol	A > G
chr22:18571368:A:G:UTR3:PEX26	chr22	18571368	18571368	A	G	UTR3	PEX26	ENST000003	.	.	A	G	+	+	0.31372549	35,0,16,0	chr22,18571	Cytosol	A > G
chr22:18571369:A:G:UTR3:PEX26	chr22	18571369	18571369	A	G	UTR3	PEX26	ENST000003	.	.	A	G	+	+	0.34	33,0,17,0	chr22,18571	Cytosol	A > G
chr22:18571383:A:G:UTR3:PEX26	chr22	18571383	18571383	A	G	UTR3	PEX26	ENST000003	.	.	A	G	+	+	0.03571429	54,0,2,0	chr22,18571	Cytosol	A > G
chr22:18571385:A:G:UTR3:PEX26	chr22	18571385	18571385	A	G	UTR3	PEX26	ENST000003	.	.	A	G	+	+	0.04918033	58,0,3,0	chr22,18571	Cytosol	A > G
chr22:18571389:A:G:UTR3:PEX26	chr22	18571389	18571389	A	G	UTR3	PEX26	ENST000003	.	.	A	G	+	+	0.03125	62,0,2,0	chr22,18571	Cytosol	A > G
chr22:18571421:A:G:UTR3:PEX26	chr22	18571421	18571421	A	G	UTR3	PEX26	ENST000003	.	.	A	G	+	+	0.53333333	42,0,48,0	chr22,18571	Cytosol	A > G
chr22:18571431:A:G:UTR3:PEX26	chr22	18571431	18571431	A	G	UTR3	PEX26	ENST000003	.	.	A	G	+	+	0.34375	63,0,33,0	chr22,18571	Cytosol	A > G
chr22:18571454:A:G:UTR3:PEX26	chr22	18571454	18571454	A	G	UTR3	PEX26	ENST000003	.	.	A	G	+	+	0.02247191	87,0,2,0	chr22,18571	Cytosol	A > G
chr22:18571474:A:G:UTR3:PEX26	chr22	18571474	18571474	A	G	UTR3	PEX26	ENST000003	.	.	A	G	+	+	0.04854369	98,0,5,0	chr22,18571	Cytosol	A > G
chr22:18571475:A:G:UTR3:PEX26	chr22	18571475	18571475	A	G	UTR3	PEX26	ENST000003	.	.	A	G	+	+	0.01941748	101,0,2,0	chr22,18571	Cytosol	A > G
chr22:18571477:A:G:UTR3:PEX26	chr22	18571477	18571477	A	G	UTR3	PEX26	ENST000003	.	.	A	G	+	+	0.04761905	100,0,5,0	chr22,18571	Cytosol	A > G
chr22:18572572:A:G:UTR3:PEX26	chr22	18572572	18572572	A	G	UTR3	PEX26	ENST000003	.	.	A	G	+	+	0.11666667	53,0,7,0	chr22,18572	Cytosol	A > G
chr22:18572597:A:G:UTR3:PEX26	chr22	18572597	18572597	A	G	UTR3	PEX26	ENST000003	.	.	A	G	+	+	0.60869565	18,0,28,0	chr22,18572	Cytosol	A > G
chr22:18572633:A:G:UTR3:PEX26	chr22	18572633	18572633	A	G	UTR3	PEX26	ENST000003	.	.	A	G	+	+	0.06382979	44,0,3,0	chr22,18572	Cytosol	A > G
chr22:18572650:A:G:UTR3:PEX26	chr22	18572650	18572650	A	G	UTR3	PEX26	ENST000003	.	.	A	G	+	+	0.30357143	39,0,17,0	chr22,18572	Cytosol	A > G
chr22:18572653:A:G:UTR3:PEX26	chr22	18572653	18572653	A	G	UTR3	PEX26	ENST000003	.	.	A	G	+	+	0.10909091	49,0,6,0	chr22,18572	Cytosol	A > G
chr22:18572675:A:G:UTR3:PEX26	chr22	18572675	18572675	A	G	UTR3	PEX26	ENST000003	.	.	A	G	+	+	0.71428571	16,0,40,0	chr22,18572	Cytosol	A > G
chr22:18572728:A:G:UTR3:PEX26	chr22	18572728	18572728	A	G	UTR3	PEX26	ENST000003	.	.	A	G	+	+	0.08928571	51,0,5,0	chr22,18572	Cytosol	A > G
chr22:18572729:A:G:UTR3:PEX26	chr22	18572729	18572729	A	G	UTR3	PEX26	ENST000003	.	.	A	G	+	+	0.16666667	45,0,9,0	chr22,18572	Cytosol	A > G
chr22:18572730:A:G:UTR3:PEX26	chr22	18572730	18572730	A	G	UTR3	PEX26	ENST000003	.	.	A	G	+	+	0.37037037	34,0,20,0	chr22,18572	Cytosol	A > G
chr22:18572742:A:G:UTR3:PEX26	chr22	18572742	18572742	A	G	UTR3	PEX26	ENST000003	.	.	A	G	+	+	0.12676056	62,0,9,0	chr22,18572	Cytosol	A > G
chr22:18574284:A:G:UTR3:PEX26	chr22	18574284	18574284	A	G	UTR3	PEX26	ENST000003	.	.	A	G	+	+	0.83333333	1,0,5,0	chr22,18574	Cytosol	A > G
chr22:18574297:A:G:UTR3:PEX26	chr22	18574297	18574297	A	G	UTR3	PEX26	ENST000003	.	.	A	G	+	+	0.8	1,0,4,0	chr22,18574	Cytosol	A > G
chr22:18574395:A:G:UTR3:PEX26	chr22	18574395	18574395	A	G	UTR3	PEX26	ENST000003	.	.	A	G	+	+	0.19047619	17,0,4,0	chr22,18574	Cytosol	A > G
chr22:18580814:A:G:UTR3:PEX26	chr22	18580814	18580814	A	G	UTR3	PEX26	ENST000003	.	.	A	G	+	+	0.4	3,0,2,0	chr22,18580	Cytosol	A > G
chr22:18581208:A:G:UTR3:PEX26	chr22	18581208	18581208	A	G	UTR3	PEX26	ENST000003	.	.	A	G	+	+	0.28571429	5,0,2,0	chr22,18581	Cytosol	A > G
chr22:18581219:A:G:UTR3:PEX26	chr22	18581219	18581219	A	G	UTR3	PEX26	ENST000003	.	.	A	G	+	+	0.6	2,0,3,0	chr22,18581	Cytosol	A > G
chr22:20052926:A:G:ncRNA_intro	chr22	20052926	20052926	A	G	ncRNA_intro	AC006547.1	.	.	.	A	G	+	+-	0.20408163	39,0,10,0	chr22,20052	Cytosol	A > G
chr22:20052958:A:G:ncRNA_intro	chr22	20052958	20052958	A	G	ncRNA_intro	AC006547.1	.	.	.	A	G	+	+-	0.18644068	48,0,11,0	chr22,20052	Cytosol	A > G
chr22:20053125:A:G:ncRNA_exon	chr22	20053125	20053125	A	G	ncRNA_exon	AC006547.1	.	.	.	A	G	+	+-	0.10144928	62,0,7,0	chr22,20053	Cytosol	A > G
chr22:24081601:T:C:upstream:LL2	chr22	24081601	24081601	T	C	upstream	LL22NC03-N	.	.	.	A	G	-		1	0,0,6,0	chr22,24081	Cytosol	A > G
chr22:24968476:A:G:UTR3:SNRPD	chr22	24968476	24968476	A	G	UTR3	SNRPD3	ENST000002	.	.	A	G	+	+	0.04812834	178,0,9,0	chr22,24968	Cytosol	A > G
chr22:24968574:A:G:UTR3:SNRPD	chr22	24968574	24968574	A	G	UTR3	SNRPD3	ENST000002	.	.	A	G	+	+	0.0942029	125,0,13,0	chr22,24968	Cytosol	A > G
chr22:24968577:A:G:UTR3:SNRPD	chr22	24968577	24968577	A	G	UTR3	SNRPD3	ENST000002	.	.	A	G	+	+	0.07246377	128,0,10,0	chr22,24968	Cytosol	A > G
chr22:24968578:A:G:UTR3:SNRPD	chr22	24968578	24968578	A	G	UTR3	SNRPD3	ENST000002	.	.	A	G	+	+	0.11678832	121,0,16,0	chr22,24968	Cytosol	A > G
chr22:24968579:A:G:UTR3:SNRPD	chr22	24968579	24968579	A	G	UTR3	SNRPD3	ENST000002	.	.	A	G	+	+	0.12142857	123,0,17,0	chr22,24968	Cytosol	A > G
chr22:24968582:A:G:UTR3:SNRPD	chr22	24968582	24968582	A	G	UTR3	SNRPD3	ENST000002	.	.	A	G	+	+	0.07692308	120,0,10,0	chr22,24968	Cytosol	A > G
chr22:24968608:A:G:UTR3:SNRPD	chr22	24968608	24968608	A	G	UTR3	SNRPD3	ENST000002	.	.	A	G	+	+	0.15584416	130,0,24,0	chr22,24968	Cytosol	A > G
chr22:24968614:A:G:UTR3:SNRPD	chr22	24968614	24968614	A	G	UTR3	SNRPD3	ENST000002	.	.	A	G	+	+	0.15606936	146,0,27,0	chr22,24968	Cytosol	A > G
chr22:24968635:A:G:UTR3:SNRPD	chr22	24968635	24968635	A	G	UTR3	SNRPD3	ENST000002	.	.	A	G	+	+	0.10204082	132,0,15,0	chr22,24968	Cytosol	A > G
chr22:24968971:A:G:intronic:SNR	chr22	24968971	24968971	A	G	intronic	SNRPD3	.	.	.	A	G	+	+	0.34210526	100,0,52,0	chr22,24968	Cytosol	A > G
chr22:24968989:A:G:intronic:SNR	chr22	24968989	24968989	A	G	intronic	SNRPD3	.	.	.	A	G	+	+	0.75961538	25,0,79,0	chr22,24968	Cytosol	A > G
chr22:24969021:A:G:intronic:SNR	chr22	24969021	24969021	A	G	intronic	SNRPD3	.	.	.	A	G	+	+	0.38541667	59,0,37,0	chr22,24969	Cytosol	A > G
chr22:24969025:A:G:intronic:SNR	chr22	24969025	24969025	A	G	intronic	SNRPD3	.	.	.	A	G	+	+	0.47777778	47,0,43,0	chr22,24969	Cytosol	A > G
chr22:24969026:A:G:intronic:SNR	chr22	24969026	24969026	A	G	intronic	SNRPD3	.	.	.	A	G	+	+	0.06896552	81,0,6,0	chr22,24969	Cytosol	A > G
chr22:24969051:A:G:intronic:SNR	chr22	24969051	24969051	A	G	intronic	SNRPD3	.	.	.	A	G	+	+	0.13592233	89,0,14,0	chr22,24969	Cytosol	A > G
chr22:24969061:A:G:intronic:SNR	chr22	24969061	24969061	A	G	intronic	SNRPD3	.	.	.	A	G	+	+	0.33673469	65,0,33,0	chr22,24969	Cytosol	A > G
chr22:24969064:A:G:intronic:SNR	chr22	24969064	24969064	A	G	intronic	SNRPD3	.	.	.	A	G	+	+	0.30107527	65,0,28,0	chr22,24969	Cytosol	A > G
chr22:24969087:A:G:intronic:SNR	chr22	24969087	24969087	A	G	intronic	SNRPD3	.	.	.	A	G	+	+	0.13709677	107,0,17,0	chr22,24969	Cytosol	A > G
chr22:24969092:A:G:intronic:SNR	chr22	24969092	24969092	A	G	intronic	SNRPD3	.	.	.	A	G	+	+	0.39130435	98,0,63,0	chr22,24969	Cytosol	A > G
chr22:24969623:A:G:intronic:SNR	chr22	24969623	24969623	A	G	intronic	SNRPD3	.	.	.	A	G	+	+	0.1352459	211,0,33,0	chr22,24969	Cytosol	A > G
chr22:24969628:A:G:intronic:SNR	chr22	24969628	24969628	A	G	intronic	SNRPD3	.	.	.	A	G	+	+	0.16129032	208,0,40,0	chr22,24969	Cytosol	A > G
chr22:24969629:A:G:intronic:SNR	chr22	24969629	24969629	A	G	intronic	SNRPD3	.	.	.	A	G	+	+	0.34468085	154,0,81,0	chr22,24969	Cytosol	A > G
chr22:24969691:A:G:intronic:SNR	chr22	24969691	24969691	A	G	intronic	SNRPD3	.	.	.	A	G	+	+	0.04046243	166,0,7,0	chr22,24969	Cytosol	A > G

chr22:24969694:A:G:intronic:SNRI	chr22	24969694	24969694	A	G	intronic	SNRPD3	.	.	.	A	G	+	+	0.06435644	189,0,13,0	chr22,24969	Cytosol	A > G
chr22:24969702:A:G:intronic:SNRI	chr22	24969702	24969702	A	G	intronic	SNRPD3	.	.	.	A	G	+	+	0.01809955	217,0,4,0	chr22,24969	Cytosol	A > G
chr22:24969705:A:G:intronic:SNRI	chr22	24969705	24969705	A	G	intronic	SNRPD3	.	.	.	A	G	+	+	0.01293103	229,0,3,0	chr22,24969	Cytosol	A > G
chr22:24969734:A:G:intronic:SNRI	chr22	24969734	24969734	A	G	intronic	SNRPD3	.	.	.	A	G	+	+	0.01239669	239,0,3,0	chr22,24969	Cytosol	A > G
chr22:24969755:A:G:intronic:SNRI	chr22	24969755	24969755	A	G	intronic	SNRPD3	.	.	.	A	G	+	+	0.19444444	145,0,35,0	chr22,24969	Cytosol	A > G
chr22:24969782:A:G:intronic:SNRI	chr22	24969782	24969782	A	G	intronic	SNRPD3	.	.	.	A	G	+	+	0.39416058	83,0,54,0	chr22,24969	Cytosol	A > G
chr22:24969800:A:G:intronic:SNRI	chr22	24969800	24969800	A	G	intronic	SNRPD3	.	.	.	A	G	+	+	0.23178808	116,0,35,0	chr22,24969	Cytosol	A > G
chr22:24969801:A:G:intronic:SNRI	chr22	24969801	24969801	A	G	intronic	SNRPD3	.	.	.	A	G	+	+	0.21710526	119,0,33,0	chr22,24969	Cytosol	A > G
chr22:24969806:A:G:intronic:SNRI	chr22	24969806	24969806	A	G	intronic	SNRPD3	.	.	.	A	G	+	+	0.05696203	149,0,9,0	chr22,24969	Cytosol	A > G
chr22:24970542:A:G:intronic:SNRI	chr22	24970542	24970542	A	G	intronic	SNRPD3	.	.	.	A	G	+	+	0.28113879	202,0,79,0	chr22,24970	Cytosol	A > G
chr22:24970576:A:G:intronic:SNRI	chr22	24970576	24970576	A	G	intronic	SNRPD3	.	.	.	A	G	+	+	0.22699387	126,0,37,0	chr22,24970	Cytosol	A > G
chr22:24970609:A:G:intronic:SNRI	chr22	24970609	24970609	A	G	intronic	SNRPD3	.	.	.	A	G	+	+	0.84431138	26,0,141,0	chr22,24970	Cytosol	A > G
chr22:24970614:A:G:intronic:SNRI	chr22	24970614	24970614	A	G	intronic	SNRPD3	.	.	.	A	G	+	+	0.16981132	132,0,27,0	chr22,24970	Cytosol	A > G
chr22:24970622:A:G:intronic:SNRI	chr22	24970622	24970622	A	G	intronic	SNRPD3	.	.	.	A	G	+	+	0.24050633	120,0,38,0	chr22,24970	Cytosol	A > G
chr22:24970639:A:G:intronic:SNRI	chr22	24970639	24970639	A	G	intronic	SNRPD3	.	.	.	A	G	+	+	0.67213115	60,0,123,0	chr22,24970	Cytosol	A > G
chr22:24970643:A:G:intronic:SNRI	chr22	24970643	24970643	A	G	intronic	SNRPD3	.	.	.	A	G	+	+	0.39779006	109,0,72,0	chr22,24970	Cytosol	A > G
chr22:24970646:A:G:intronic:SNRI	chr22	24970646	24970646	A	G	intronic	SNRPD3	.	.	.	A	G	+	+	0.24858757	133,0,44,0	chr22,24970	Cytosol	A > G
chr22:24970662:A:G:intronic:SNRI	chr22	24970662	24970662	A	G	intronic	SNRPD3	.	.	.	A	G	+	+	0.07746479	131,0,11,0	chr22,24970	Cytosol	A > G
chr22:24970671:A:G:intronic:SNRI	chr22	24970671	24970671	A	G	intronic	SNRPD3	.	.	.	A	G	+	+	0.11564626	130,0,17,0	chr22,24970	Cytosol	A > G
chr22:24970675:A:G:intronic:SNRI	chr22	24970675	24970675	A	G	intronic	SNRPD3	.	.	.	A	G	+	+	0.3	98,0,42,0	chr22,24970	Cytosol	A > G
chr22:24970676:A:G:intronic:SNRI	chr22	24970676	24970676	A	G	intronic	SNRPD3	.	.	.	A	G	+	+	0.13669065	120,0,19,0	chr22,24970	Cytosol	A > G
chr22:24970693:A:G:intronic:SNRI	chr22	24970693	24970693	A	G	intronic	SNRPD3	.	.	.	A	G	+	+	0.07692308	108,0,9,0	chr22,24970	Cytosol	A > G
chr22:24970710:A:G:intronic:SNRI	chr22	24970710	24970710	A	G	intronic	SNRPD3	.	.	.	A	G	+	+	0.44537815	66,0,53,0	chr22,24970	Cytosol	A > G
chr22:24970720:A:G:intronic:SNRI	chr22	24970720	24970720	A	G	intronic	SNRPD3	.	.	.	A	G	+	+	0.68907563	37,0,82,0	chr22,24970	Cytosol	A > G
chr22:24970744:A:G:intronic:SNRI	chr22	24970744	24970744	A	G	intronic	SNRPD3	.	.	.	A	G	+	+	0.16546763	116,0,23,0	chr22,24970	Cytosol	A > G
chr22:24970789:A:G:intronic:SNRI	chr22	24970789	24970789	A	G	intronic	SNRPD3	.	.	.	A	G	+	+	0.01492537	198,0,3,0	chr22,24970	Cytosol	A > G
chr22:24972364:A:G:intronic:SNRI	chr22	24972364	24972364	A	G	intronic	SNRPD3	.	.	.	A	G	+	+	0.26923077	19,0,7,0	chr22,24972	Cytosol	A > G
chr22:24972482:A:G:intronic:SNRI	chr22	24972482	24972482	A	G	intronic	SNRPD3	.	.	.	A	G	+	+	1	0,0,7,0	chr22,24972	Cytosol	A > G
chr22:26120881:A:G:UTR3:ADRBK	chr22	26120881	26120881	A	G	UTR3	ADRBK2	ENST000003	.	.	A	G	+	+	0.5	3,0,3,0	chr22,26120	Cytosol	A > G
chr22:26120907:A:G:UTR3:ADRBK	chr22	26120907	26120907	A	G	UTR3	ADRBK2	ENST000003	.	.	A	G	+	+	0.66666667	2,0,4,0	chr22,26120	Cytosol	A > G
chr22:26121065:A:G:UTR3:ADRBK	chr22	26121065	26121065	A	G	UTR3	ADRBK2	ENST000003	.	.	A	G	+	+	0.4	3,0,2,0	chr22,26121	Cytosol	A > G
chr22:26121077:A:G:UTR3:ADRBK	chr22	26121077	26121077	A	G	UTR3	ADRBK2	ENST000003	.	.	A	G	+	+	0.4	3,0,2,0	chr22,26121	Cytosol	A > G
chr22:26121801:A:G:UTR3:ADRBK	chr22	26121801	26121801	A	G	UTR3	ADRBK2	ENST000003	.	.	A	G	+	+	0.8	1,0,4,0	chr22,26121	Cytosol	A > G
chr22:26890997:A:G:intronic:TFIP	chr22	26890997	26890997	A	G	intronic	TFIP11	.	.	.	A	G	+	-	0.625	3,0,5,0	chr22,26890	Cytosol	A > G
chr22:29143321:A:G:intronic:HSC	chr22	29143321	29143321	A	G	intronic	HSCB	.	.	.	A	G	+	+	0.42857143	4,0,3,0	chr22,29143	Cytosol	A > G
chr22:29143518:A:G:intronic:HSC	chr22	29143518	29143518	A	G	intronic	HSCB	.	.	.	A	G	+	+	0.75	2,0,6,0	chr22,29143	Cytosol	A > G
chr22:29145054:A:G:intronic:HSC	chr22	29145054	29145054	A	G	intronic	HSCB	.	.	.	A	G	+	+	0.6	2,0,3,0	chr22,29145	Cytosol	A > G
chr22:29153392:A:G:UTR3:HSCB	chr22	29153392	29153392	A	G	UTR3	HSCB	ENST000002	.	.	A	G	+	+	0.03896104	74,0,3,0	chr22,29153	Cytosol	A > G
chr22:29657351:T:C:exonic:RHBDI	chr22	29657351	29657351	T	C	exonic	RHBDD3	.	nonsynonym	RHBDD3:ENS	A	G	-	-+	0.6	2,0,3,0	chr22,29657	Cytosol	A > G
chr22:29836603:T:C:ncRNA_exoni	chr22	29836603	29836603	T	C	ncRNA_exon	RFPL1S	.	.	.	A	G	-	-+	0.44444444	5,0,4,0	chr22,29836	Cytosol	A > G
chr22:30885279:T:C:ncRNA_intror	chr22	30885279	30885279	T	C	ncRNA_intro	RP4-539M6.	.	.	.	A	G	-	+-	0.15384615	22,0,4,0	chr22,30885	Cytosol	A > G
chr22:30885303:T:C:ncRNA_intror	chr22	30885303	30885303	T	C	ncRNA_intro	RP4-539M6.	.	.	.	A	G	-	+-	0.7	3,0,7,0	chr22,30885	Cytosol	A > G
chr22:30885319:T:C:ncRNA_intror	chr22	30885319	30885319	T	C	ncRNA_intro	RP4-539M6.	.	.	.	A	G	-	+-	0.73333333	4,0,11,0	chr22,30885	Cytosol	A > G
chr22:30885332:T:C:ncRNA_intror	chr22	30885332	30885332	T	C	ncRNA_intro	RP4-539M6.	.	.	.	A	G	-	+-	0.22222222	14,0,4,0	chr22,30885	Cytosol	A > G
chr22:30885338:T:C:ncRNA_intror	chr22	30885338	30885338	T	C	ncRNA_intro	RP4-539M6.	.	.	.	A	G	-	+-	0.78571429	3,0,11,0	chr22,30885	Cytosol	A > G
chr22:30885342:T:C:ncRNA_intror	chr22	30885342	30885342	T	C	ncRNA_intro	RP4-539M6.	.	.	.	A	G	-	+-	0.33333333	10,0,5,0	chr22,30885	Cytosol	A > G
chr22:30885351:T:C:ncRNA_intror	chr22	30885351	30885351	T	C	ncRNA_intro	RP4-539M6.	.	.	.	A	G	-	+-	0.78947368	4,0,15,0	chr22,30885	Cytosol	A > G
chr22:30885355:T:C:ncRNA_intror	chr22	30885355	30885355	T	C	ncRNA_intro	RP4-539M6.	.	.	.	A	G	-	+-	0.22222222	14,0,4,0	chr22,30885	Cytosol	A > G
chr22:30885364:T:C:ncRNA_intror	chr22	30885364	30885364	T	C	ncRNA_intro	RP4-539M6.	.	.	.	A	G	-	+-	0.4	9,0,6,0	chr22,30885	Cytosol	A > G
chr22:30885365:T:C:ncRNA_intror	chr22	30885365	30885365	T	C	ncRNA_intro	RP4-539M6.	.	.	.	A	G	-	+-	0.4	9,0,6,0	chr22,30885	Cytosol	A > G
chr22:30885403:T:C:ncRNA_intror	chr22	30885403	30885403	T	C	ncRNA_intro	RP4-539M6.	.	.	.	A	G	-	+-	0.13043478	20,0,3,0	chr22,30885	Cytosol	A > G
chr22:30885404:T:C:ncRNA_intror	chr22	30885404	30885404	T	C	ncRNA_intro	RP4-539M6.	.	.	.	A	G	-	+-	0.12	22,0,3,0	chr22,30885	Cytosol	A > G
chr22:30885405:T:C:ncRNA_intror	chr22	30885405	30885405	T	C	ncRNA_intro	RP4-539M6.	.	.	.	A	G	-	+-	0.28571429	15,0,6,0	chr22,30885	Cytosol	A > G
chr22:30885406:T:C:ncRNA_intror	chr22	30885406	30885406	T	C	ncRNA_intro	RP4-539M6.	.	.	.	A	G	-	+-	0.61904762	8,0,13,0	chr22,30885	Cytosol	A > G
chr22:30885409:T:C:ncRNA_intror	chr22	30885409	30885409	T	C	ncRNA_intro	RP4-539M6.	.	.	.	A	G	-	+-	0.12	22,0,3,0	chr22,30885	Cytosol	A > G
chr22:30885428:T:C:ncRNA_intror	chr22	30885428	30885428	T	C	ncRNA_intro	RP4-539M6.	.	.	.	A	G	-	+-	0.05128205	37,0,2,0	chr22,30885	Cytosol	A > G
chr22:30885454:T:C:ncRNA_intror	chr22	30885454	30885454	T	C	ncRNA_intro	RP4-539M6.	.	.	.	A	G	-	+-	0.33333333	26,0,13,0	chr22,30885	Cytosol	A > G
chr22:30885464:T:C:ncRNA_intror	chr22	30885464	30885464	T	C	ncRNA_intro	RP4-539M6.	.	.	.	A	G	-	+-	0.23076923	20,0,6,0	chr22,30885	Cytosol	A > G
chr22:30885477:T:C:ncRNA_intror	chr22	30885477	30885477	T	C	ncRNA_intro	RP4-539M6.	.	.	.	A	G	-	+-	0.05405405	35,0,2,0	chr22,30885	Cytosol	A > G
chr22:30885499:T:C:ncRNA_intror	chr22	30885499	30885499	T	C	ncRNA_intro	RP4-539M6.	.	.	.	A	G	-	+-	0.16216216	31,0,6,0	chr22,30885	Cytosol	A > G
chr22:30885500:T:C:ncRNA_intror	chr22	30885500	30885500	T	C	ncRNA_intro	RP4-539M6.	.	.	.	A	G	-	+-	0.55263158	17,0,21,0	chr22,30885	Cytosol	A > G
chr22:30885502:T:C:ncRNA_intror	chr22	30885502	30885502	T	C	ncRNA_intro	RP4-539M6.	.	.	.	A	G	-	+-	0.56756757	16,0,21,0	chr22,30885	Cytosol	A > G
chr22:30885508:T:C:ncRNA_intror	chr22	30885508	30885508	T	C	ncRNA_intro	RP4-539M6.	.	.	.	A	G	-	+-	0.35483871	20,0,11,0	chr22,30885	Cytosol	A > G
chr22:31058634:T:C:UTR3:DUSP1	chr22	31058634	31058634	T	C	UTR3	DUSP18,SLC3	ENST0000004	.	.	A	G	-	+-	0.33333333	4,0,2,0	chr22,31058	Cytosol	A > G

chr22:31831465:A:G:intronic:DRG	chr22	31831465	31831465	A	G	intronic	DRG1	.	.	.	A	G	+	+	0.8	4,0,16,0	chr22,31831	Cytosol	A > G
chr22:31831552:A:G:intronic:DRG	chr22	31831552	31831552	A	G	intronic	DRG1	.	.	.	A	G	+	+	0.80952381	4,0,17,0	chr22,31831	Cytosol	A > G
chr22:31831589:A:G:intronic:DRG	chr22	31831589	31831589	A	G	intronic	DRG1	.	.	.	A	G	+	+	0.46666667	8,0,7,0	chr22,31831	Cytosol	A > G
chr22:31831615:A:G:intronic:DRG	chr22	31831615	31831615	A	G	intronic	DRG1	.	.	.	A	G	+	+	0.47058824	9,0,8,0	chr22,31831	Cytosol	A > G
chr22:31831654:A:G:intronic:DRG	chr22	31831654	31831654	A	G	intronic	DRG1	.	.	.	A	G	+	+	0.63636364	4,0,7,0	chr22,31831	Cytosol	A > G
chr22:31831675:A:G:intronic:DRG	chr22	31831675	31831675	A	G	intronic	DRG1	.	.	.	A	G	+	+	0.8	1,0,4,0	chr22,31831	Cytosol	A > G
chr22:36055907:A:G:UTR3:APOL6	chr22	36055907	36055907	A	G	UTR3	APOL6	ENST000004	.	.	A	G	+	+	0.63636364	4,0,7,0	chr22,36055	Cytosol	A > G
chr22:36055913:A:G:UTR3:APOL6	chr22	36055913	36055913	A	G	UTR3	APOL6	ENST000004	.	.	A	G	+	+	0.45454545	6,0,5,0	chr22,36055	Cytosol	A > G
chr22:36055916:A:G:UTR3:APOL6	chr22	36055916	36055916	A	G	UTR3	APOL6	ENST000004	.	.	A	G	+	+	0.83333333	2,0,10,0	chr22,36055	Cytosol	A > G
chr22:36055938:A:G:UTR3:APOL6	chr22	36055938	36055938	A	G	UTR3	APOL6	ENST000004	.	.	A	G	+	+	0.23076923	10,0,3,0	chr22,36055	Cytosol	A > G
chr22:36055952:A:G:UTR3:APOL6	chr22	36055952	36055952	A	G	UTR3	APOL6	ENST000004	.	.	A	G	+	+	0.14285714	12,0,2,0	chr22,36055	Cytosol	A > G
chr22:36056011:A:G:UTR3:APOL6	chr22	36056011	36056011	A	G	UTR3	APOL6	ENST000004	.	.	A	G	+	+	0.15789474	16,0,3,0	chr22,36056	Cytosol	A > G
chr22:36056598:A:G:UTR3:APOL6	chr22	36056598	36056598	A	G	UTR3	APOL6	ENST000004	.	.	A	G	+	+	0.33333333	18,0,9,0	chr22,36056	Cytosol	A > G
chr22:36057206:A:G:UTR3:APOL6	chr22	36057206	36057206	A	G	UTR3	APOL6	ENST000004	.	.	A	G	+	+	0.40740741	16,0,11,0	chr22,36057	Cytosol	A > G
chr22:36057248:A:G:UTR3:APOL6	chr22	36057248	36057248	A	G	UTR3	APOL6	ENST000004	.	.	A	G	+	+	0.24390244	31,0,10,0	chr22,36057	Cytosol	A > G
chr22:36057251:A:G:UTR3:APOL6	chr22	36057251	36057251	A	G	UTR3	APOL6	ENST000004	.	.	A	G	+	+	0.23076923	30,0,9,0	chr22,36057	Cytosol	A > G
chr22:36057254:A:G:UTR3:APOL6	chr22	36057254	36057254	A	G	UTR3	APOL6	ENST000004	.	.	A	G	+	+	0.10526316	34,0,4,0	chr22,36057	Cytosol	A > G
chr22:36057261:A:G:UTR3:APOL6	chr22	36057261	36057261	A	G	UTR3	APOL6	ENST000004	.	.	A	G	+	+	0.05128205	37,0,2,0	chr22,36057	Cytosol	A > G
chr22:36057267:A:G:UTR3:APOL6	chr22	36057267	36057267	A	G	UTR3	APOL6	ENST000004	.	.	A	G	+	+	0.075	37,0,3,0	chr22,36057	Cytosol	A > G
chr22:36057284:A:G:UTR3:APOL6	chr22	36057284	36057284	A	G	UTR3	APOL6	ENST000004	.	.	A	G	+	+	0.6875	10,0,22,0	chr22,36057	Cytosol	A > G
chr22:36057285:A:G:UTR3:APOL6	chr22	36057285	36057285	A	G	UTR3	APOL6	ENST000004	.	.	A	G	+	+	0.3030303	23,0,10,0	chr22,36057	Cytosol	A > G
chr22:36057294:A:G:UTR3:APOL6	chr22	36057294	36057294	A	G	UTR3	APOL6	ENST000004	.	.	A	G	+	+	0.21428571	22,0,6,0	chr22,36057	Cytosol	A > G
chr22:36057354:A:G:UTR3:APOL6	chr22	36057354	36057354	A	G	UTR3	APOL6	ENST000004	.	.	A	G	+	+	0.5	4,0,4,0	chr22,36057	Cytosol	A > G
chr22:36057355:A:G:UTR3:APOL6	chr22	36057355	36057355	A	G	UTR3	APOL6	ENST000004	.	.	A	G	+	+	0.66666667	2,0,4,0	chr22,36057	Cytosol	A > G
chr22:36057400:A:G:UTR3:APOL6	chr22	36057400	36057400	A	G	UTR3	APOL6	ENST000004	.	.	A	G	+	+	0.25	6,0,2,0	chr22,36057	Cytosol	A > G
chr22:36662181:A:G:UTR3:APOL1	chr22	36662181	36662181	A	G	UTR3	APOL1	ENST000003	.	.	A	G	+	+	0.08571429	64,0,6,0	chr22,36662	Cytosol	A > G
chr22:36662193:A:G:UTR3:APOL1	chr22	36662193	36662193	A	G	UTR3	APOL1	ENST000003	.	.	A	G	+	+	0.58227848	33,0,46,0	chr22,36662	Cytosol	A > G
chr22:36662206:A:G:UTR3:APOL1	chr22	36662206	36662206	A	G	UTR3	APOL1	ENST000003	.	.	A	G	+	+	0.78313253	17,0,65,1	chr22,36662	Cytosol	A > G
chr22:36662217:A:G:UTR3:APOL1	chr22	36662217	36662217	A	G	UTR3	APOL1	ENST000003	.	.	A	G	+	+	0.05714286	66,0,4,0	chr22,36662	Cytosol	A > G
chr22:36662236:A:G:UTR3:APOL1	chr22	36662236	36662236	A	G	UTR3	APOL1	ENST000003	.	.	A	G	+	+	0.04411765	65,0,3,0	chr22,36662	Cytosol	A > G
chr22:36662237:A:G:UTR3:APOL1	chr22	36662237	36662237	A	G	UTR3	APOL1	ENST000003	.	.	A	G	+	+	0.22058824	53,0,15,0	chr22,36662	Cytosol	A > G
chr22:36662266:A:G:UTR3:APOL1	chr22	36662266	36662266	A	G	UTR3	APOL1	ENST000003	.	.	A	G	+	+	0.52830189	25,0,28,0	chr22,36662	Cytosol	A > G
chr22:36662276:A:G:UTR3:APOL1	chr22	36662276	36662276	A	G	UTR3	APOL1	ENST000003	.	.	A	G	+	+	0.58928571	23,0,33,0	chr22,36662	Cytosol	A > G
chr22:36662278:A:G:UTR3:APOL1	chr22	36662278	36662278	A	G	UTR3	APOL1	ENST000003	.	.	A	G	+	+	0.19642857	45,0,11,0	chr22,36662	Cytosol	A > G
chr22:36662298:A:G:UTR3:APOL1	chr22	36662298	36662298	A	G	UTR3	APOL1	ENST000003	.	.	A	G	+	+	0.1875	13,0,3,0	chr22,36662	Cytosol	A > G
chr22:36662302:A:G:UTR3:APOL1	chr22	36662302	36662302	A	G	UTR3	APOL1	ENST000003	.	.	A	G	+	+	0.17647059	14,0,3,0	chr22,36662	Cytosol	A > G
chr22:36662311:A:G:UTR3:APOL1	chr22	36662311	36662311	A	G	UTR3	APOL1	ENST000003	.	.	A	G	+	+	0.28571429	10,0,4,0	chr22,36662	Cytosol	A > G
chr22:36662317:A:G:UTR3:APOL1	chr22	36662317	36662317	A	G	UTR3	APOL1	ENST000003	.	.	A	G	+	+	0.375	10,0,6,0	chr22,36662	Cytosol	A > G
chr22:36662318:A:G:UTR3:APOL1	chr22	36662318	36662318	A	G	UTR3	APOL1	ENST000003	.	.	A	G	+	+	0.64285714	5,0,9,0	chr22,36662	Cytosol	A > G
chr22:36662337:A:G:UTR3:APOL1	chr22	36662337	36662337	A	G	UTR3	APOL1	ENST000003	.	.	A	G	+	+	0.76923077	3,0,10,0	chr22,36662	Cytosol	A > G
chr22:36662366:A:G:UTR3:APOL1	chr22	36662366	36662366	A	G	UTR3	APOL1	ENST000003	.	.	A	G	+	+	0.28571429	10,0,4,0	chr22,36662	Cytosol	A > G
chr22:36662374:A:G:UTR3:APOL1	chr22	36662374	36662374	A	G	UTR3	APOL1	ENST000003	.	.	A	G	+	+	0.22727273	17,0,5,0	chr22,36662	Cytosol	A > G
chr22:36662382:A:G:UTR3:APOL1	chr22	36662382	36662382	A	G	UTR3	APOL1	ENST000003	.	.	A	G	+	+	0.0952381	19,0,2,0	chr22,36662	Cytosol	A > G
chr22:36662396:A:G:UTR3:APOL1	chr22	36662396	36662396	A	G	UTR3	APOL1	ENST000003	.	.	A	G	+	+	0.42857143	12,0,9,0	chr22,36662	Cytosol	A > G
chr22:36662397:A:G:UTR3:APOL1	chr22	36662397	36662397	A	G	UTR3	APOL1	ENST000003	.	.	A	G	+	+	0.66666667	7,0,14,0	chr22,36662	Cytosol	A > G
chr22:36662409:A:G:UTR3:APOL1	chr22	36662409	36662409	A	G	UTR3	APOL1	ENST000003	.	.	A	G	+	+	0.07692308	24,0,2,0	chr22,36662	Cytosol	A > G
chr22:36662696:A:G:UTR3:APOL1	chr22	36662696	36662696	A	G	UTR3	APOL1	ENST000003	.	.	A	G	+	+	0.71428571	6,0,15,0	chr22,36662	Cytosol	A > G
chr22:36662697:A:G:UTR3:APOL1	chr22	36662697	36662697	A	G	UTR3	APOL1	ENST000003	.	.	A	G	+	+	0.42105263	11,0,8,0	chr22,36662	Cytosol	A > G
chr22:36662735:A:G:UTR3:APOL1	chr22	36662735	36662735	A	G	UTR3	APOL1	ENST000003	.	.	A	G	+	+	0.4	3,0,2,0	chr22,36662	Cytosol	A > G
chr22:36662804:A:G:UTR3:APOL1	chr22	36662804	36662804	A	G	UTR3	APOL1	ENST000003	.	.	A	G	+	+	0.81818182	2,0,9,0	chr22,36662	Cytosol	A > G
chr22:36662830:A:G:UTR3:APOL1	chr22	36662830	36662830	A	G	UTR3	APOL1	ENST000003	.	.	A	G	+	+	0.31428571	24,0,11,0	chr22,36662	Cytosol	A > G
chr22:36662840:A:G:UTR3:APOL1	chr22	36662840	36662840	A	G	UTR3	APOL1	ENST000003	.	.	A	G	+	+	0.07317073	38,0,3,0	chr22,36662	Cytosol	A > G
chr22:36662841:A:G:UTR3:APOL1	chr22	36662841	36662841	A	G	UTR3	APOL1	ENST000003	.	.	A	G	+	+	0.07692308	36,0,3,0	chr22,36662	Cytosol	A > G
chr22:36662857:A:G:UTR3:APOL1	chr22	36662857	36662857	A	G	UTR3	APOL1	ENST000003	.	.	A	G	+	+	0.04255319	45,0,2,0	chr22,36662	Cytosol	A > G
chr22:36662863:A:G:UTR3:APOL1	chr22	36662863	36662863	A	G	UTR3	APOL1	ENST000003	.	.	A	G	+	+	0.05633803	67,0,4,0	chr22,36662	Cytosol	A > G
chr22:36662871:A:G:UTR3:APOL1	chr22	36662871	36662871	A	G	UTR3	APOL1	ENST000003	.	.	A	G	+	+	0.42307692	45,0,33,0	chr22,36662	Cytosol	A > G
chr22:36662872:A:G:UTR3:APOL1	chr22	36662872	36662872	A	G	UTR3	APOL1	ENST000003	.	.	A	G	+	+	0.58974359	32,0,46,0	chr22,36662	Cytosol	A > G
chr22:36662879:A:G:UTR3:APOL1	chr22	36662879	36662879	A	G	UTR3	APOL1	ENST000003	.	.	A	G	+	+	0.02564103	76,0,2,0	chr22,36662	Cytosol	A > G
chr22:36662901:A:G:UTR3:APOL1	chr22	36662901	36662901	A	G	UTR3	APOL1	ENST000003	.	.	A	G	+	+	0.1097561	73,0,9,0	chr22,36662	Cytosol	A > G
chr22:36662903:A:G:UTR3:APOL1	chr22	36662903	36662903	A	G	UTR3	APOL1	ENST000003	.	.	A	G	+	+	0.04878049	78,0,4,0	chr22,36662	Cytosol	A > G
chr22:36662933:A:G:splicing:APO	chr22	36662933	36662933	A	G	splicing	APOL1	ENST000003	.	.	A	G	+	+	0.05970149	63,0,4,0	chr22,36662	Cytosol	A > G
chr22:36662946:A:G:UTR3:APOL1	chr22	36662946	36662946	A	G	UTR3	APOL1	ENST000003	.	.	A	G	+	+	0.03797468	76,0,3,0	chr22,36662	Cytosol	A > G
chr22:36662950:C:A:UTR3:APOL1	chr22	36662950	36662950	C	A	UTR3	APOL1	ENST000003	.	.	C	A	+	+	0.03030303	2,64,0,0	chr22,36662	Cytosol	C > A
chr22:36883882:T:C:UTR3:FOXRE	chr22	36883882	36883882	T	C	UTR3	FOXRED2	ENST000003	.	.	A	G	-	-	0.01980198	99,0,2,0	chr22,36883	Cytosol	A > G

chr22:36883895:T:C:UTR3:FOXRED	chr22	36883895	36883895	T	C	UTR3	FOXRED2	ENST000003	.	.	A	G	-	-	0.11688312	68,0,9,0	chr22,36883	Cytosol	A > G
chr22:36883990:T:C:UTR3:FOXRED	chr22	36883990	36883990	T	C	UTR3	FOXRED2	ENST000003	.	.	A	G	-	-	0.45945946	20,0,17,0	chr22,36883	Cytosol	A > G
chr22:36884039:T:C:UTR3:FOXRED	chr22	36884039	36884039	T	C	UTR3	FOXRED2	ENST000003	.	.	A	G	-	-	0.34615385	34,0,18,0	chr22,36884	Cytosol	A > G
chr22:36884055:T:C:UTR3:FOXRED	chr22	36884055	36884055	T	C	UTR3	FOXRED2	ENST000003	.	.	A	G	-	-	0.24615385	49,0,16,0	chr22,36884	Cytosol	A > G
chr22:36884076:T:C:UTR3:FOXRED	chr22	36884076	36884076	T	C	UTR3	FOXRED2	ENST000003	.	.	A	G	-	-	0.33333333	32,0,16,0	chr22,36884	Cytosol	A > G
chr22:36884095:T:C:UTR3:FOXRED	chr22	36884095	36884095	T	C	UTR3	FOXRED2	ENST000003	.	.	A	G	-	-	0.92857143	3,0,39,0	chr22,36884	Cytosol	A > G
chr22:36884100:T:C:UTR3:FOXRED	chr22	36884100	36884100	T	C	UTR3	FOXRED2	ENST000003	.	.	A	G	-	-	0.56521739	20,0,26,0	chr22,36884	Cytosol	A > G
chr22:36884348:T:C:UTR3:FOXRED	chr22	36884348	36884348	T	C	UTR3	FOXRED2	ENST000003	.	.	A	G	-	-	0.04320988	155,0,7,0	chr22,36884	Cytosol	A > G
chr22:36884403:T:C:UTR3:FOXRED	chr22	36884403	36884403	T	C	UTR3	FOXRED2	ENST000003	.	.	A	G	-	-	0.07826087	106,0,9,0	chr22,36884	Cytosol	A > G
chr22:36884426:T:C:UTR3:FOXRED	chr22	36884426	36884426	T	C	UTR3	FOXRED2	ENST000003	.	.	A	G	-	-	0.1559633	92,0,17,0	chr22,36884	Cytosol	A > G
chr22:36884486:T:C:UTR3:FOXRED	chr22	36884486	36884486	T	C	UTR3	FOXRED2	ENST000003	.	.	A	G	-	-	0.11111111	80,0,10,0	chr22,36884	Cytosol	A > G
chr22:36884489:T:C:UTR3:FOXRED	chr22	36884489	36884489	T	C	UTR3	FOXRED2	ENST000003	.	.	A	G	-	-	0.30188679	74,0,32,0	chr22,36884	Cytosol	A > G
chr22:36884506:T:C:UTR3:FOXRED	chr22	36884506	36884506	T	C	UTR3	FOXRED2	ENST000003	.	.	A	G	-	-	0.1754386	94,0,20,0	chr22,36884	Cytosol	A > G
chr22:36884530:T:C:UTR3:FOXRED	chr22	36884530	36884530	T	C	UTR3	FOXRED2	ENST000003	.	.	A	G	-	-	0.08256881	100,0,9,0	chr22,36884	Cytosol	A > G
chr22:36884539:T:C:UTR3:FOXRED	chr22	36884539	36884539	T	C	UTR3	FOXRED2	ENST000003	.	.	A	G	-	-	0.1559633	92,0,17,0	chr22,36884	Cytosol	A > G
chr22:36884544:T:C:UTR3:FOXRED	chr22	36884544	36884544	T	C	UTR3	FOXRED2	ENST000003	.	.	A	G	-	-	0.19191919	80,0,19,0	chr22,36884	Cytosol	A > G
chr22:36884556:T:C:UTR3:FOXRED	chr22	36884556	36884556	T	C	UTR3	FOXRED2	ENST000003	.	.	A	G	-	-	0.08333333	88,0,8,0	chr22,36884	Cytosol	A > G
chr22:36884586:T:C:UTR3:FOXRED	chr22	36884586	36884586	T	C	UTR3	FOXRED2	ENST000003	.	.	A	G	-	-	0.04	72,0,3,0	chr22,36884	Cytosol	A > G
chr22:36884671:T:C:UTR3:FOXRED	chr22	36884671	36884671	T	C	UTR3	FOXRED2	ENST000003	.	.	A	G	-	-	0.34375	21,0,11,0	chr22,36884	Cytosol	A > G
chr22:36884701:T:C:UTR3:FOXRED	chr22	36884701	36884701	T	C	UTR3	FOXRED2	ENST000003	.	.	A	G	-	-	0.375	15,0,9,0	chr22,36884	Cytosol	A > G
chr22:36884726:T:C:UTR3:FOXRED	chr22	36884726	36884726	T	C	UTR3	FOXRED2	ENST000003	.	.	A	G	-	-	0.34375	21,0,11,0	chr22,36884	Cytosol	A > G
chr22:36884727:T:C:UTR3:FOXRED	chr22	36884727	36884727	T	C	UTR3	FOXRED2	ENST000003	.	.	A	G	-	-	0.59259259	11,0,16,0	chr22,36884	Cytosol	A > G
chr22:36884767:T:C:UTR3:FOXRED	chr22	36884767	36884767	T	C	UTR3	FOXRED2	ENST000003	.	.	A	G	-	-	0.45945946	20,0,17,0	chr22,36884	Cytosol	A > G
chr22:36884780:T:C:UTR3:FOXRED	chr22	36884780	36884780	T	C	UTR3	FOXRED2	ENST000003	.	.	A	G	-	-	0.54545455	15,0,18,0	chr22,36884	Cytosol	A > G
chr22:36884789:T:C:UTR3:FOXRED	chr22	36884789	36884789	T	C	UTR3	FOXRED2	ENST000003	.	.	A	G	-	-	0.43589744	22,0,17,0	chr22,36884	Cytosol	A > G
chr22:36884793:T:C:UTR3:FOXRED	chr22	36884793	36884793	T	C	UTR3	FOXRED2	ENST000003	.	.	A	G	-	-	0.1627907	36,0,7,0	chr22,36884	Cytosol	A > G
chr22:36884801:T:C:UTR3:FOXRED	chr22	36884801	36884801	T	C	UTR3	FOXRED2	ENST000003	.	.	A	G	-	-	0.5	17,0,17,0	chr22,36884	Cytosol	A > G
chr22:36884807:T:C:UTR3:FOXRED	chr22	36884807	36884807	T	C	UTR3	FOXRED2	ENST000003	.	.	A	G	-	-	0.59375	13,0,19,0	chr22,36884	Cytosol	A > G
chr22:36884816:T:C:UTR3:FOXRED	chr22	36884816	36884816	T	C	UTR3	FOXRED2	ENST000003	.	.	A	G	-	-	0.81081081	7,0,30,0	chr22,36884	Cytosol	A > G
chr22:36884829:T:C:UTR3:FOXRED	chr22	36884829	36884829	T	C	UTR3	FOXRED2	ENST000003	.	.	A	G	-	-	0.41860465	25,0,18,0	chr22,36884	Cytosol	A > G
chr22:36884855:T:C:UTR3:FOXRED	chr22	36884855	36884855	T	C	UTR3	FOXRED2	ENST000003	.	.	A	G	-	-	0.22222222	49,0,14,0	chr22,36884	Cytosol	A > G
chr22:36884865:T:C:UTR3:FOXRED	chr22	36884865	36884865	T	C	UTR3	FOXRED2	ENST000003	.	.	A	G	-	-	0.30357143	39,0,17,0	chr22,36884	Cytosol	A > G
chr22:36884878:T:C:UTR3:FOXRED	chr22	36884878	36884878	T	C	UTR3	FOXRED2	ENST000003	.	.	A	G	-	-	0.0952381	57,0,6,0	chr22,36884	Cytosol	A > G
chr22:36884895:T:C:UTR3:FOXRED	chr22	36884895	36884895	T	C	UTR3	FOXRED2	ENST000003	.	.	A	G	-	-	0.07017544	53,0,4,0	chr22,36884	Cytosol	A > G
chr22:38007975:A:G:intronic:GGA	chr22	38007975	38007975	A	G	intronic	GGA1	.	.	.	A	G	+	+	0.4	3,0,2,0	chr22,38007	Cytosol	A > G
chr22:38007998:A:G:intronic:GGA	chr22	38007998	38007998	A	G	intronic	GGA1	.	.	.	A	G	+	+	0.4	3,0,2,0	chr22,38007	Cytosol	A > G
chr22:38284876:A:G:UTR3:EIF3L	chr22	38284876	38284876	A	G	UTR3	EIF3L	ENST000004	.	.	A	G	+	+	0.0952381	19,0,2,0	chr22,38284	Cytosol	A > G
chr22:38340786:A:G:intronic:C22c	chr22	38340786	38340786	A	G	intronic	C22orf23	.	.	.	A	G	+	-	0.83333333	1,0,5,0	chr22,38340	Cytosol	A > G
chr22:38342761:A:G:intronic:C22c	chr22	38342761	38342761	A	G	intronic	C22orf23	.	.	.	A	G	+	-	0.33333333	4,0,2,0	chr22,38342	Cytosol	A > G
chr22:39414754:A:G:UTR3:APOBE	chr22	39414754	39414754	A	G	UTR3	APOBEC3C	ENST000003	.	.	A	G	+	+	0.04672897	102,0,5,0	chr22,39414	Cytosol	A > G
chr22:39414772:A:G:UTR3:APOBE	chr22	39414772	39414772	A	G	UTR3	APOBEC3C	ENST000003	.	.	A	G	+	+	0.0952381	95,0,10,0	chr22,39414	Cytosol	A > G
chr22:39414900:A:G:UTR3:APOBE	chr22	39414900	39414900	A	G	UTR3	APOBEC3C	ENST000003	.	.	A	G	+	+	0.01818182	162,0,3,0	chr22,39414	Cytosol	A > G
chr22:39414912:A:G:UTR3:APOBE	chr22	39414912	39414912	A	G	UTR3	APOBEC3C	ENST000003	.	.	A	G	+	+	0.25974026	114,0,40,0	chr22,39414	Cytosol	A > G
chr22:39414923:A:G:UTR3:APOBE	chr22	39414923	39414923	A	G	UTR3	APOBEC3C	ENST000003	.	.	A	G	+	+	0.02162162	181,0,4,0	chr22,39414	Cytosol	A > G
chr22:39415872:A:G:UTR3:APOBE	chr22	39415872	39415872	A	G	UTR3	APOBEC3C	ENST000003	.	.	A	G	+	+	0.19	81,0,19,0	chr22,39415	Cytosol	A > G
chr22:39415875:A:G:UTR3:APOBE	chr22	39415875	39415875	A	G	UTR3	APOBEC3C	ENST000003	.	.	A	G	+	+	0.01769912	111,0,2,0	chr22,39415	Cytosol	A > G
chr22:39415891:A:G:UTR3:APOBE	chr22	39415891	39415891	A	G	UTR3	APOBEC3C	ENST000003	.	.	A	G	+	+	0.15322581	105,0,19,0	chr22,39415	Cytosol	A > G
chr22:39415905:A:G:UTR3:APOBE	chr22	39415905	39415905	A	G	UTR3	APOBEC3C	ENST000003	.	.	A	G	+	+	0.47328244	69,0,62,0	chr22,39415	Cytosol	A > G
chr22:39415910:A:G:UTR3:APOBE	chr22	39415910	39415910	A	G	UTR3	APOBEC3C	ENST000003	.	.	A	G	+	+	0.125	119,0,17,0	chr22,39415	Cytosol	A > G
chr22:39415911:A:G:UTR3:APOBE	chr22	39415911	39415911	A	G	UTR3	APOBEC3C	ENST000003	.	.	A	G	+	+	0.4469697	73,0,59,0	chr22,39415	Cytosol	A > G
chr22:39416023:A:G:UTR3:APOBE	chr22	39416023	39416023	A	G	UTR3	APOBEC3C	ENST000003	.	.	A	G	+	+	0.03030303	64,0,2,0	chr22,39416	Cytosol	A > G
chr22:39416035:A:G:UTR3:APOBE	chr22	39416035	39416035	A	G	UTR3	APOBEC3C	ENST000003	.	.	A	G	+	+	0.17333333	62,0,13,0	chr22,39416	Cytosol	A > G
chr22:39416063:A:G:UTR3:APOBE	chr22	39416063	39416063	A	G	UTR3	APOBEC3C	ENST000003	.	.	A	G	+	+	0.02439024	120,0,3,0	chr22,39416	Cytosol	A > G
chr22:39416077:A:G:UTR3:APOBE	chr22	39416077	39416077	A	G	UTR3	APOBEC3C	ENST000003	.	.	A	G	+	+	0.032	121,0,4,0	chr22,39416	Cytosol	A > G
chr22:40763189:A:G:intronic:ADSL	chr22	40763189	40763189	A	G	intronic	ADSL	.	.	.	A	G	+	+	0.52380952	10,0,11,0	chr22,40763	Cytosol	A > G
chr22:40763199:A:G:intronic:ADSL	chr22	40763199	40763199	A	G	intronic	ADSL	.	.	.	A	G	+	+	0.52941176	8,0,9,0	chr22,40763	Cytosol	A > G
chr22:40763282:A:G:intronic:ADSL	chr22	40763282	40763282	A	G	intronic	ADSL	.	.	.	A	G	+	+	0.6	2,0,3,0	chr22,40763	Cytosol	A > G
chr22:40763376:A:G:intronic:ADSL	chr22	40763376	40763376	A	G	intronic	ADSL	.	.	.	A	G	+	+	0.53846154	12,0,14,0	chr22,40763	Cytosol	A > G
chr22:41323223:A:G:UTR3:XPNPEI	chr22	41323223	41323223	A	G	UTR3	XPNPEP3	ENST000003	.	.	A	G	+	+	0.1875	13,0,3,0	chr22,41323	Cytosol	A > G
chr22:41323292:A:G:UTR3:XPNPEI	chr22	41323292	41323292	A	G	UTR3	XPNPEP3	ENST000003	.	.	A	G	+	+	0.14285714	12,0,2,0	chr22,41323	Cytosol	A > G
chr22:41323357:A:G:UTR3:XPNPEI	chr22	41323357	41323357	A	G	UTR3	XPNPEP3	ENST000003	.	.	A	G	+	+	0.14285714	12,0,2,0	chr22,41323	Cytosol	A > G
chr22:41323358:A:G:UTR3:XPNPEI	chr22	41323358	41323358	A	G	UTR3	XPNPEP3	ENST000003	.	.	A	G	+	+	0.5	7,0,7,0	chr22,41323	Cytosol	A > G
chr22:41323465:A:G:UTR3:XPNPEI	chr22	41323465	41323465	A	G	UTR3	XPNPEP3	ENST000003	.	.	A	G	+	+	0.27272727	8,0,3,0	chr22,41323	Cytosol	A > G
chr22:41324310:A:G:UTR3:XPNPEI	chr22	41324310	41324310	A	G	UTR3	XPNPEP3	ENST000003	.	.	A	G	+	+	0.5	3,0,3,0	chr22,41324	Cytosol	A > G

chr22:41325295:A:G:UTR3:XPNPEI	chr22	41325295	41325295	A	G	UTR3	XPNPEP3	ENST000003	.	.	A	G	+	+	0.42857143	4,0,3,0	chr22,41325	Cytosol	A > G
chr22:41326340:A:G:UTR3:XPNPEI	chr22	41326340	41326340	A	G	UTR3	XPNPEP3	ENST000003	.	.	A	G	+	+	0.5	3,0,3,0	chr22,41326	Cytosol	A > G
chr22:41326407:A:G:UTR3:XPNPEI	chr22	41326407	41326407	A	G	UTR3	XPNPEP3	ENST000003	.	.	A	G	+	+	0.41666667	7,0,5,0	chr22,41326	Cytosol	A > G
chr22:41326593:A:G:UTR3:XPNPEI	chr22	41326593	41326593	A	G	UTR3	XPNPEP3	ENST000003	.	.	A	G	+	+	0.66666667	4,0,8,0	chr22,41326	Cytosol	A > G
chr22:41326673:A:G:UTR3:XPNPEI	chr22	41326673	41326673	A	G	UTR3	XPNPEP3	ENST000003	.	.	A	G	+	+	0.1875	13,0,3,0	chr22,41326	Cytosol	A > G
chr22:41327456:A:G:UTR3:XPNPEI	chr22	41327456	41327456	A	G	UTR3	XPNPEP3	ENST000003	.	.	A	G	+	+	0.64285714	5,0,9,0	chr22,41327	Cytosol	A > G
chr22:41327460:A:G:UTR3:XPNPEI	chr22	41327460	41327460	A	G	UTR3	XPNPEP3	ENST000003	.	.	A	G	+	+	0.71428571	4,0,10,0	chr22,41327	Cytosol	A > G
chr22:41327536:A:G:UTR3:XPNPEI	chr22	41327536	41327536	A	G	UTR3	XPNPEP3	ENST000003	.	.	A	G	+	+	0.5	5,0,5,0	chr22,41327	Cytosol	A > G
chr22:41327575:A:G:UTR3:XPNPEI	chr22	41327575	41327575	A	G	UTR3	XPNPEP3	ENST000003	.	.	A	G	+	+	0.30769231	9,0,4,0	chr22,41327	Cytosol	A > G
chr22:41327591:A:G:UTR3:XPNPEI	chr22	41327591	41327591	A	G	UTR3	XPNPEP3	ENST000003	.	.	A	G	+	+	0.45	11,0,9,0	chr22,41327	Cytosol	A > G
chr22:41327595:A:G:UTR3:XPNPEI	chr22	41327595	41327595	A	G	UTR3	XPNPEP3	ENST000003	.	.	A	G	+	+	0.66666667	7,0,14,0	chr22,41327	Cytosol	A > G
chr22:41327597:A:G:UTR3:XPNPEI	chr22	41327597	41327597	A	G	UTR3	XPNPEP3	ENST000003	.	.	A	G	+	+	0.56521739	10,0,13,0	chr22,41327	Cytosol	A > G
chr22:41327603:A:G:UTR3:XPNPEI	chr22	41327603	41327603	A	G	UTR3	XPNPEP3	ENST000003	.	.	A	G	+	+	0.11111111	16,0,2,0	chr22,41327	Cytosol	A > G
chr22:41327611:A:G:UTR3:XPNPEI	chr22	41327611	41327611	A	G	UTR3	XPNPEP3	ENST000003	.	.	A	G	+	+	0.47619048	11,0,10,0	chr22,41327	Cytosol	A > G
chr22:41327643:A:G:UTR3:XPNPEI	chr22	41327643	41327643	A	G	UTR3	XPNPEP3	ENST000003	.	.	A	G	+	+	0.25	15,0,5,0	chr22,41327	Cytosol	A > G
chr22:41327651:A:G:UTR3:XPNPEI	chr22	41327651	41327651	A	G	UTR3	XPNPEP3	ENST000003	.	.	A	G	+	+	0.13333333	13,0,2,0	chr22,41327	Cytosol	A > G
chr22:41351338:A:G:UTR3:XPNPEI	chr22	41351338	41351338	A	G	UTR3	XPNPEP3	ENST000005	.	.	A	G	+	+	0.21052632	15,0,4,0	chr22,41351	Cytosol	A > G
chr22:41995599:T:C:UTR3:DESI1	chr22	41995599	41995599	T	C	UTR3	DESI1	ENST000002	.	.	A	G	-	-	0.016	123,0,2,0	chr22,41995	Cytosol	A > G
chr22:41995624:T:C:UTR3:DESI1	chr22	41995624	41995624	T	C	UTR3	DESI1	ENST000002	.	.	A	G	-	-	0.05	57,0,3,0	chr22,41995	Cytosol	A > G
chr22:41995636:T:C:UTR3:DESI1	chr22	41995636	41995636	T	C	UTR3	DESI1	ENST000002	.	.	A	G	-	-	0.11538462	23,0,3,0	chr22,41995	Cytosol	A > G
chr22:41995660:T:C:UTR3:DESI1	chr22	41995660	41995660	T	C	UTR3	DESI1	ENST000002	.	.	A	G	-	-	0.11538462	23,0,3,0	chr22,41995	Cytosol	A > G
chr22:41995672:T:C:UTR3:DESI1	chr22	41995672	41995672	T	C	UTR3	DESI1	ENST000002	.	.	A	G	-	-	0.33333333	12,0,6,0	chr22,41995	Cytosol	A > G
chr22:41995675:T:C:UTR3:DESI1	chr22	41995675	41995675	T	C	UTR3	DESI1	ENST000002	.	.	A	G	-	-	0.88888889	2,0,16,0	chr22,41995	Cytosol	A > G
chr22:41995679:T:C:UTR3:DESI1	chr22	41995679	41995679	T	C	UTR3	DESI1	ENST000002	.	.	A	G	-	-	0.84210526	3,0,16,0	chr22,41995	Cytosol	A > G
chr22:41995692:T:C:UTR3:DESI1	chr22	41995692	41995692	T	C	UTR3	DESI1	ENST000002	.	.	A	G	-	-	0.57894737	8,0,11,0	chr22,41995	Cytosol	A > G
chr22:41995696:T:C:UTR3:DESI1	chr22	41995696	41995696	T	C	UTR3	DESI1	ENST000002	.	.	A	G	-	-	0.26315789	14,0,5,0	chr22,41995	Cytosol	A > G
chr22:41995702:T:C:UTR3:DESI1	chr22	41995702	41995702	T	C	UTR3	DESI1	ENST000002	.	.	A	G	-	-	0.61904762	8,0,13,0	chr22,41995	Cytosol	A > G
chr22:41995728:T:C:UTR3:DESI1	chr22	41995728	41995728	T	C	UTR3	DESI1	ENST000002	.	.	A	G	-	-	0.61538462	5,0,8,0	chr22,41995	Cytosol	A > G
chr22:41995733:T:C:UTR3:DESI1	chr22	41995733	41995733	T	C	UTR3	DESI1	ENST000002	.	.	A	G	-	-	0.23076923	10,0,3,0	chr22,41995	Cytosol	A > G
chr22:41995742:T:C:UTR3:DESI1	chr22	41995742	41995742	T	C	UTR3	DESI1	ENST000002	.	.	A	G	-	-	1	0,0,12,0	chr22,41995	Cytosol	A > G
chr22:41995926:T:C:UTR3:DESI1	chr22	41995926	41995926	T	C	UTR3	DESI1	ENST000002	.	.	A	G	-	-	0.42857143	4,0,3,0	chr22,41995	Cytosol	A > G
chr22:41995957:T:C:UTR3:DESI1	chr22	41995957	41995957	T	C	UTR3	DESI1	ENST000002	.	.	A	G	-	-	0.6	2,0,3,0	chr22,41995	Cytosol	A > G
chr22:41996011:T:C:UTR3:DESI1	chr22	41996011	41996011	T	C	UTR3	DESI1	ENST000002	.	.	A	G	-	-	0.8	1,0,4,0	chr22,41996	Cytosol	A > G
chr22:41996017:T:C:UTR3:DESI1	chr22	41996017	41996017	T	C	UTR3	DESI1	ENST000002	.	.	A	G	-	-	0.77777778	2,0,7,0	chr22,41996	Cytosol	A > G
chr22:41996026:T:C:UTR3:DESI1	chr22	41996026	41996026	T	C	UTR3	DESI1	ENST000002	.	.	A	G	-	-	0.7	3,0,7,0	chr22,41996	Cytosol	A > G
chr22:41996061:T:C:UTR3:DESI1	chr22	41996061	41996061	T	C	UTR3	DESI1	ENST000002	.	.	A	G	-	-	0.75	2,0,6,0	chr22,41996	Cytosol	A > G
chr22:41996071:T:C:UTR3:DESI1	chr22	41996071	41996071	T	C	UTR3	DESI1	ENST000002	.	.	A	G	-	-	0.75	2,0,6,0	chr22,41996	Cytosol	A > G
chr22:41996101:T:C:UTR3:DESI1	chr22	41996101	41996101	T	C	UTR3	DESI1	ENST000002	.	.	A	G	-	-	0.4	6,0,4,0	chr22,41996	Cytosol	A > G
chr22:41996121:T:C:UTR3:DESI1	chr22	41996121	41996121	T	C	UTR3	DESI1	ENST000002	.	.	A	G	-	-	0.4	12,0,8,0	chr22,41996	Cytosol	A > G
chr22:41996157:T:C:UTR3:DESI1	chr22	41996157	41996157	T	C	UTR3	DESI1	ENST000002	.	.	A	G	-	-	0.25641026	29,0,10,0	chr22,41996	Cytosol	A > G
chr22:41996161:T:C:UTR3:DESI1	chr22	41996161	41996161	T	C	UTR3	DESI1	ENST000002	.	.	A	G	-	-	0.56097561	18,0,23,0	chr22,41996	Cytosol	A > G
chr22:41996164:T:C:UTR3:DESI1	chr22	41996164	41996164	T	C	UTR3	DESI1	ENST000002	.	.	A	G	-	-	0.07142857	39,0,3,0	chr22,41996	Cytosol	A > G
chr22:42975019:T:C:ncRNA_intror	chr22	42975019	42975019	T	C	ncRNA_intro	RRP7B	.	.	.	A	G	-	-	0.83333333	1,0,5,0	chr22,42975	Cytosol	A > G
chr22:42975021:T:C:ncRNA_intror	chr22	42975021	42975021	T	C	ncRNA_intro	RRP7B	.	.	.	A	G	-	-	0.66666667	2,0,4,0	chr22,42975	Cytosol	A > G
chr22:44343196:A:G:UTR3:PNPLA	chr22	44343196	44343196	A	G	UTR3	PNPLA3	ENST000002	.	.	A	G	+	+	0.23076923	10,0,3,0	chr22,44343	Cytosol	A > G
chr22:44345316:A:G:intronic:PNPI	chr22	44345316	44345316	A	G	intronic	PNPLA3	.	.	.	A	G	+	+	0.125	14,0,2,0	chr22,44345	Cytosol	A > G
chr22:44345406:A:G:intronic:PNPI	chr22	44345406	44345406	A	G	intronic	PNPLA3	.	.	.	A	G	+	+	0.47368421	10,0,9,0	chr22,44345	Cytosol	A > G
chr22:44345412:A:G:intronic:PNPI	chr22	44345412	44345412	A	G	intronic	PNPLA3	.	.	.	A	G	+	+	0.53333333	7,0,8,0	chr22,44345	Cytosol	A > G
chr22:44345742:A:G:intronic:PNPI	chr22	44345742	44345742	A	G	intronic	PNPLA3	.	.	.	A	G	+	+	0.66666667	8,0,16,0	chr22,44345	Cytosol	A > G
chr22:44346105:A:G:intronic:PNPI	chr22	44346105	44346105	A	G	intronic	PNPLA3	.	.	.	A	G	+	+	0.35483871	20,0,11,0	chr22,44346	Cytosol	A > G
chr22:44346545:A:G:intronic:PNPI	chr22	44346545	44346545	A	G	intronic	PNPLA3	.	.	.	A	G	+	+	0.55555556	4,0,5,0	chr22,44346	Cytosol	A > G
chr22:44346622:A:G:intronic:PNPI	chr22	44346622	44346622	A	G	intronic	PNPLA3	.	.	.	A	G	+	+	0.4	12,0,8,0	chr22,44346	Cytosol	A > G
chr22:44346623:A:G:intronic:PNPI	chr22	44346623	44346623	A	G	intronic	PNPLA3	.	.	.	A	G	+	+	0.15789474	16,0,3,0	chr22,44346	Cytosol	A > G
chr22:44346636:A:G:intronic:PNPI	chr22	44346636	44346636	A	G	intronic	PNPLA3	.	.	.	A	G	+	+	0.4	9,0,6,0	chr22,44346	Cytosol	A > G
chr22:44346974:A:G:intronic:PNPI	chr22	44346974	44346974	A	G	intronic	PNPLA3	.	.	.	A	G	+	+	0.6	4,0,6,0	chr22,44346	Cytosol	A > G
chr22:44346981:A:G:intronic:PNPI	chr22	44346981	44346981	A	G	intronic	PNPLA3	.	.	.	A	G	+	+	0.94117647	1,0,16,0	chr22,44346	Cytosol	A > G
chr22:44347050:A:G:intronic:PNPI	chr22	44347050	44347050	A	G	intronic	PNPLA3	.	.	.	A	G	+	+	0.35714286	9,0,5,0	chr22,44347	Cytosol	A > G
chr22:44347068:A:G:intronic:PNPI	chr22	44347068	44347068	A	G	intronic	PNPLA3	.	.	.	A	G	+	+	0.72727273	3,0,8,0	chr22,44347	Cytosol	A > G
chr22:44565950:A:G:UTR3:PARVB	chr22	44565950	44565950	A	G	UTR3	PARVB	ENST000003	.	.	A	G	+	+	0.4	15,0,10,0	chr22,44565	Cytosol	A > G
chr22:44565974:A:G:UTR3:PARVB	chr22	44565974	44565974	A	G	UTR3	PARVB	ENST000003	.	.	A	G	+	+	0.21875	25,0,7,0	chr22,44565	Cytosol	A > G
chr22:44565993:A:G:UTR3:PARVB	chr22	44565993	44565993	A	G	UTR3	PARVB	ENST000003	.	.	A	G	+	+	0.15	17,0,3,0	chr22,44565	Cytosol	A > G
chr22:45558936:T:C:ncRNA_intror	chr22	45558936	45558936	T	C	ncRNA_intro	CTA-217C2.1	.	.	.	A	G	-	-	0.4	3,0,2,0	chr22,45558	Cytosol	A > G
chr22:45736722:A:G:UTR3:FAM11	chr22	45736722	45736722	A	G	UTR3	FAM118A	ENST000002	.	.	A	G	+	+	0.1025641	35,0,4,0	chr22,45736	Cytosol	A > G
chr22:45736763:A:G:UTR3:FAM11	chr22	45736763	45736763	A	G	UTR3	FAM118A	ENST000002	.	.	A	G	+	+	0.05769231	49,0,3,0	chr22,45736	Cytosol	A > G

chr22:45736783:A:G:UTR3:FAM11	chr22	45736783	45736783	A	G	UTR3	FAM118A	ENST000002	.	.	A	G	+	+	0.17948718	32,0,7,0	chr22,45736	Cytosol	A > G
chr22:45736784:A:G:UTR3:FAM11	chr22	45736784	45736784	A	G	UTR3	FAM118A	ENST000002	.	.	A	G	+	+	0.21052632	30,0,8,0	chr22,45736	Cytosol	A > G
chr22:45736801:A:G:UTR3:FAM11	chr22	45736801	45736801	A	G	UTR3	FAM118A	ENST000002	.	.	A	G	+	+	0.3030303	23,0,10,0	chr22,45736	Cytosol	A > G
chr22:45736867:A:G:UTR3:FAM11	chr22	45736867	45736867	A	G	UTR3	FAM118A	ENST000002	.	.	A	G	+	+	0.3030303	23,0,10,0	chr22,45736	Cytosol	A > G
chr22:45736946:A:G:UTR3:FAM11	chr22	45736946	45736946	A	G	UTR3	FAM118A	ENST000002	.	.	A	G	+	+	0.03571429	54,0,2,0	chr22,45736	Cytosol	A > G
chr22:45736952:A:G:UTR3:FAM11	chr22	45736952	45736952	A	G	UTR3	FAM118A	ENST000002	.	.	A	G	+	+	0.14754098	52,0,9,0	chr22,45736	Cytosol	A > G
chr22:46616464:A:G:intronic:PPAI	chr22	46616464	46616464	A	G	intronic	PPARA	.	.	.	A	G	+	+	0.42857143	4,0,3,0	chr22,46616	Cytosol	A > G
chr22:46616720:A:G:intronic:PPAI	chr22	46616720	46616720	A	G	intronic	PPARA	.	.	.	A	G	+	+	0.66666667	3,0,6,0	chr22,46616	Cytosol	A > G
chr22:46688132:A:G:exonic:TTC38	chr22	46688132	46688132	A	G	exonic	TTC38	.	unknown	UNKNOWN	A	G	+	+	0.8	1,0,4,0	chr22,46688	Cytosol	A > G
chr22:51062283:T:C:UTR3:ARSA	chr22	51062283	51062283	T	C	UTR3	ARSA	ENST000006	.	.	A	G	-	-	1	0,0,5,0	chr22,51062	Cytosol	A > G
chr3:10036456:A:G:ncRNA_introni	chr3	10036456	10036456	A	G	ncRNA_intro	AC034193.5	.	.	.	A	G	+	-+	0.25	6,0,2,0	chr3,100364	Cytosol	A > G
chr3:10038997:A:G:ncRNA_introni	chr3	10038997	10038997	A	G	ncRNA_intro	AC034193.5	.	.	.	A	G	+	-+	0.4	3,0,2,0	chr3,100389	Cytosol	A > G
chr3:10040632:A:G:ncRNA_introni	chr3	10040632	10040632	A	G	ncRNA_intro	AC034193.5	.	.	.	A	G	+	-+	0.30434783	16,0,7,0	chr3,100406	Cytosol	A > G
chr3:10041175:A:G:ncRNA_introni	chr3	10041175	10041175	A	G	ncRNA_intro	AC034193.5	.	.	.	A	G	+	-+	0.18181818	9,0,2,0	chr3,100411	Cytosol	A > G
chr3:10041862:A:G:ncRNA_introni	chr3	10041862	10041862	A	G	ncRNA_intro	AC034193.5	.	.	.	A	G	+	-+	0.22222222	7,0,2,0	chr3,100418	Cytosol	A > G
chr3:10143270:A:G:UTR3:FANCD2	chr3	10143270	10143270	A	G	UTR3	FANCD2	ENST000003	.	.	A	G	+	+-	0.04411765	65,0,3,0	chr3,101432	Cytosol	A > G
chr3:10192608:A:G:UTR3:VHL	chr3	10192608	10192608	A	G	UTR3	VHL	ENST000002	.	.	A	G	+	+	0.59615385	21,0,31,0	chr3,101926	Cytosol	A > G
chr3:10192617:A:G:UTR3:VHL	chr3	10192617	10192617	A	G	UTR3	VHL	ENST000002	.	.	A	G	+	+	0.27586207	42,0,16,0	chr3,101926	Cytosol	A > G
chr3:10192633:A:G:UTR3:VHL	chr3	10192633	10192633	A	G	UTR3	VHL	ENST000002	.	.	A	G	+	+	0.06896552	54,0,4,0	chr3,101926	Cytosol	A > G
chr3:10192642:A:G:UTR3:VHL	chr3	10192642	10192642	A	G	UTR3	VHL	ENST000002	.	.	A	G	+	+	0.234375	49,0,15,0	chr3,101926	Cytosol	A > G
chr3:10192652:A:G:UTR3:VHL	chr3	10192652	10192652	A	G	UTR3	VHL	ENST000002	.	.	A	G	+	+	0.08139535	79,0,7,0	chr3,101926	Cytosol	A > G
chr3:10192658:A:G:UTR3:VHL	chr3	10192658	10192658	A	G	UTR3	VHL	ENST000002	.	.	A	G	+	+	0.10377358	95,0,11,0	chr3,101926	Cytosol	A > G
chr3:10192694:A:G:UTR3:VHL	chr3	10192694	10192694	A	G	UTR3	VHL	ENST000002	.	.	A	G	+	+	0.11258278	134,0,17,0	chr3,101926	Cytosol	A > G
chr3:10192704:A:G:UTR3:VHL	chr3	10192704	10192704	A	G	UTR3	VHL	ENST000002	.	.	A	G	+	+	0.16788321	114,0,23,0	chr3,101927	Cytosol	A > G
chr3:10192717:A:G:UTR3:VHL	chr3	10192717	10192717	A	G	UTR3	VHL	ENST000002	.	.	A	G	+	+	0.07826087	106,0,9,0	chr3,101927	Cytosol	A > G
chr3:10192720:A:G:UTR3:VHL	chr3	10192720	10192720	A	G	UTR3	VHL	ENST000002	.	.	A	G	+	+	0.04716981	101,0,5,0	chr3,101927	Cytosol	A > G
chr3:10192744:A:G:UTR3:VHL	chr3	10192744	10192744	A	G	UTR3	VHL	ENST000002	.	.	A	G	+	+	0.20833333	57,0,15,0	chr3,101927	Cytosol	A > G
chr3:10192747:A:G:UTR3:VHL	chr3	10192747	10192747	A	G	UTR3	VHL	ENST000002	.	.	A	G	+	+	0.47058824	18,0,16,0	chr3,101927	Cytosol	A > G
chr3:10192753:A:G:UTR3:VHL	chr3	10192753	10192753	A	G	UTR3	VHL	ENST000002	.	.	A	G	+	+	0.23333333	23,0,7,0	chr3,101927	Cytosol	A > G
chr3:10192784:A:G:UTR3:VHL	chr3	10192784	10192784	A	G	UTR3	VHL	ENST000002	.	.	A	G	+	+	0.19047619	17,0,4,0	chr3,101927	Cytosol	A > G
chr3:10192811:A:G:UTR3:VHL	chr3	10192811	10192811	A	G	UTR3	VHL	ENST000002	.	.	A	G	+	+	0.25	15,0,5,0	chr3,101928	Cytosol	A > G
chr3:10192816:A:G:UTR3:VHL	chr3	10192816	10192816	A	G	UTR3	VHL	ENST000002	.	.	A	G	+	+	0.37931034	18,0,11,0	chr3,101928	Cytosol	A > G
chr3:10192825:A:G:UTR3:VHL	chr3	10192825	10192825	A	G	UTR3	VHL	ENST000002	.	.	A	G	+	+	0.05555556	34,0,2,0	chr3,101928	Cytosol	A > G
chr3:10192827:A:G:UTR3:VHL	chr3	10192827	10192827	A	G	UTR3	VHL	ENST000002	.	.	A	G	+	+	0.08108108	34,0,3,0	chr3,101928	Cytosol	A > G
chr3:10193475:A:G:UTR3:VHL	chr3	10193475	10193475	A	G	UTR3	VHL	ENST000002	.	.	A	G	+	+	0.11764706	120,0,16,0	chr3,101934	Cytosol	A > G
chr3:10193566:A:G:UTR3:VHL	chr3	10193566	10193566	A	G	UTR3	VHL	ENST000002	.	.	A	G	+	+	0.01111111	178,0,2,0	chr3,101935	Cytosol	A > G
chr3:10193732:A:G:UTR3:VHL	chr3	10193732	10193732	A	G	UTR3	VHL	ENST000002	.	.	A	G	+	+	0.10606061	59,0,7,0	chr3,101937	Cytosol	A > G
chr3:10193796:A:G:UTR3:VHL	chr3	10193796	10193796	A	G	UTR3	VHL	ENST000002	.	.	A	G	+	+	0.125	21,0,3,0	chr3,101937	Cytosol	A > G
chr3:10193866:A:G:UTR3:VHL	chr3	10193866	10193866	A	G	UTR3	VHL	ENST000002	.	.	A	G	+	+	0.11111111	16,0,2,0	chr3,101938	Cytosol	A > G
chr3:10193873:A:G:UTR3:VHL	chr3	10193873	10193873	A	G	UTR3	VHL	ENST000002	.	.	A	G	+	+	0.33333333	16,0,8,0	chr3,101938	Cytosol	A > G
chr3:10193876:A:G:UTR3:VHL	chr3	10193876	10193876	A	G	UTR3	VHL	ENST000002	.	.	A	G	+	+	0.13636364	19,0,3,0	chr3,101938	Cytosol	A > G
chr3:10193978:A:G:downstream:V	chr3	10193978	10193978	A	G	downstream	VHL	.	.	.	A	G	+	+	0.34375	84,0,44,0	chr3,101939	Cytosol	A > G
chr3:10194254:A:G:downstream:V	chr3	10194254	10194254	A	G	downstream	VHL	.	.	.	A	G	+	+	0.80530973	22,0,91,0	chr3,101942	Cytosol	A > G
chr3:10194555:A:G:downstream:V	chr3	10194555	10194555	A	G	downstream	VHL	.	.	.	A	G	+	+	0.23170732	63,0,19,0	chr3,101945	Cytosol	A > G
chr3:10194571:A:G:downstream:V	chr3	10194571	10194571	A	G	downstream	VHL	.	.	.	A	G	+	+	0.11956522	81,0,11,0	chr3,101945	Cytosol	A > G
chr3:10194593:A:G:downstream:V	chr3	10194593	10194593	A	G	downstream	VHL	.	.	.	A	G	+	+	0.36666667	38,0,22,0	chr3,101945	Cytosol	A > G
chr3:10194617:A:G:downstream:V	chr3	10194617	10194617	A	G	downstream	VHL	.	.	.	A	G	+	+	0.17142857	58,0,12,0	chr3,101946	Cytosol	A > G
chr3:10194641:A:G:downstream:V	chr3	10194641	10194641	A	G	downstream	VHL	.	.	.	A	G	+	+	0.18181818	63,0,14,0	chr3,101946	Cytosol	A > G
chr3:10194650:A:G:downstream:V	chr3	10194650	10194650	A	G	downstream	VHL	.	.	.	A	G	+	+	0.05333333	71,0,4,0	chr3,101946	Cytosol	A > G
chr3:10194841:A:G:downstream:V	chr3	10194841	10194841	A	G	downstream	VHL	.	.	.	A	G	+	+	0.11392405	70,0,9,0	chr3,101948	Cytosol	A > G
chr3:10194851:A:G:downstream:V	chr3	10194851	10194851	A	G	downstream	VHL	.	.	.	A	G	+	+	0.06756757	69,0,5,0	chr3,101948	Cytosol	A > G
chr3:10194882:A:G:downstream:V	chr3	10194882	10194882	A	G	downstream	VHL	.	.	.	A	G	+	+	0.20481928	66,0,17,0	chr3,101948	Cytosol	A > G
chr3:10194883:A:G:downstream:V	chr3	10194883	10194883	A	G	downstream	VHL	.	.	.	A	G	+	+	0.13978495	80,0,13,0	chr3,101948	Cytosol	A > G
chr3:10194890:A:G:downstream:V	chr3	10194890	10194890	A	G	downstream	VHL	.	.	.	A	G	+	+	0.43809524	59,0,46,0	chr3,101948	Cytosol	A > G
chr3:10194903:A:G:downstream:V	chr3	10194903	10194903	A	G	downstream	VHL	.	.	.	A	G	+	+	0.07476636	99,0,8,0	chr3,101949	Cytosol	A > G
chr3:10194984:A:G:intergenic:VHL	chr3	10194984	10194984	A	G	intergenic	VHL,IRAK2	dist=1080;dis	.	.	A	G	+	+	0.21198157	171,0,46,0	chr3,101949	Cytosol	A > G
chr3:10195092:A:G:intergenic:VHL	chr3	10195092	10195092	A	G	intergenic	VHL,IRAK2	dist=1188;dis	.	.	A	G	+	+	0.07633588	121,0,10,0	chr3,101950	Cytosol	A > G
chr3:10195096:A:G:intergenic:VHL	chr3	10195096	10195096	A	G	intergenic	VHL,IRAK2	dist=1192;dis	.	.	A	G	+	+	0.568	54,0,71,0	chr3,101950	Cytosol	A > G
chr3:10195103:A:G:intergenic:VHL	chr3	10195103	10195103	A	G	intergenic	VHL,IRAK2	dist=1199;dis	.	.	A	G	+	+	0.11811024	112,0,15,0	chr3,101951	Cytosol	A > G
chr3:11858118:T:C:intronic:TAMN	chr3	11858118	11858118	T	C	intronic	TAMM41	.	.	.	A	G	-	-	0.2	8,0,2,0	chr3,118581	Cytosol	A > G
chr3:12580816:A:G:exonic:TSEN2	chr3	12580816	12580816	A	G	exonic	TSEN2	.	nonsynonym	TSEN2:ENST(	A	G	+	-+	0.125	42,0,6,0	chr3,125808	Cytosol	A > G
chr3:12580867:A:G:exonic:TSEN2	chr3	12580867	12580867	A	G	exonic	TSEN2	.	nonsynonym	TSEN2:ENST(	A	G	+	-+	0.04255319	45,0,2,0	chr3,125808	Cytosol	A > G
chr3:12580868:A:G:exonic:TSEN2	chr3	12580868	12580868	A	G	exonic	TSEN2	.	nonsynonym	TSEN2:ENST(	A	G	+	-+	0.04545455	42,0,2,0	chr3,125808	Cytosol	A > G
chr3:14986080:T:C:ncRNA_exonic:	chr3	14986080	14986080	T	C	ncRNA_exon	FGD5-AS1	.	.	.	A	G	-	-	0.05777778	212,0,13,0	chr3,149860	Cytosol	A > G

chr3:14986098:T:C:ncRNA_exonic:	chr3	14986098	14986098	T	C	ncRNA_exon	FGD5-AS1	.	.	.	A	G	-	-	0.3515625	166,0,90,0	chr3,149860	Cytosol	A > G
chr3:14986104:T:C:ncRNA_exonic:	chr3	14986104	14986104	T	C	ncRNA_exon	FGD5-AS1	.	.	.	A	G	-	-	0.91629956	18,1,208,0	chr3,149861	Cytosol	A > G
chr3:14986110:T:C:ncRNA_exonic:	chr3	14986110	14986110	T	C	ncRNA_exon	FGD5-AS1	.	.	.	A	G	-	-	0.52702703	105,0,117,0	chr3,149861	Cytosol	A > G
chr3:14986111:T:C:ncRNA_exonic:	chr3	14986111	14986111	T	C	ncRNA_exon	FGD5-AS1	.	.	.	A	G	-	-	0.2394958	181,0,57,0	chr3,149861	Cytosol	A > G
chr3:14986137:T:C:ncRNA_exonic:	chr3	14986137	14986137	T	C	ncRNA_exon	FGD5-AS1	.	.	.	A	G	-	-	0.83661972	58,0,297,0	chr3,149861	Cytosol	A > G
chr3:14986138:T:C:ncRNA_exonic:	chr3	14986138	14986138	T	C	ncRNA_exon	FGD5-AS1	.	.	.	A	G	-	-	0.20338983	282,0,72,0	chr3,149861	Cytosol	A > G
chr3:14986150:T:C:ncRNA_exonic:	chr3	14986150	14986150	T	C	ncRNA_exon	FGD5-AS1	.	.	.	A	G	-	-	0.04189944	343,0,15,0	chr3,149861	Cytosol	A > G
chr3:14986398:T:C:ncRNA_exonic:	chr3	14986398	14986398	T	C	ncRNA_exon	FGD5-AS1	.	.	.	A	G	-	-	0.01709402	115,0,2,0	chr3,149863	Cytosol	A > G
chr3:14986399:T:C:ncRNA_exonic:	chr3	14986399	14986399	T	C	ncRNA_exon	FGD5-AS1	.	.	.	A	G	-	-	0.32743363	76,0,37,0	chr3,149863	Cytosol	A > G
chr3:14986417:T:C:ncRNA_exonic:	chr3	14986417	14986417	T	C	ncRNA_exon	FGD5-AS1	.	.	.	A	G	-	-	0.03361345	115,0,4,0	chr3,149864	Cytosol	A > G
chr3:14986447:T:C:ncRNA_exonic:	chr3	14986447	14986447	T	C	ncRNA_exon	FGD5-AS1	.	.	.	A	G	-	-	0.184	102,0,23,0	chr3,149864	Cytosol	A > G
chr3:14986452:T:C:ncRNA_exonic:	chr3	14986452	14986452	T	C	ncRNA_exon	FGD5-AS1	.	.	.	A	G	-	-	0.14367816	149,0,25,0	chr3,149864	Cytosol	A > G
chr3:14986453:T:C:ncRNA_exonic:	chr3	14986453	14986453	T	C	ncRNA_exon	FGD5-AS1	.	.	.	A	G	-	-	0.38251366	113,0,70,0	chr3,149864	Cytosol	A > G
chr3:14986461:T:C:ncRNA_exonic:	chr3	14986461	14986461	T	C	ncRNA_exon	FGD5-AS1	.	.	.	A	G	-	-	0.046875	183,0,9,0	chr3,149864	Cytosol	A > G
chr3:15112612:T:C:UTR3:ZFYVE20	chr3	15112612	15112612	T	C	UTR3	ZFYVE20	ENST000002	.	.	A	G	-	-	0.25	18,0,6,0	chr3,151126	Cytosol	A > G
chr3:15113655:T:C:UTR3:ZFYVE20	chr3	15113655	15113655	T	C	UTR3	ZFYVE20	ENST000002	.	.	A	G	-	-	0.38	30,0,19,1	chr3,151136	Cytosol	A > G
chr3:15113656:T:C:UTR3:ZFYVE20	chr3	15113656	15113656	T	C	UTR3	ZFYVE20	ENST000002	.	.	A	G	-	-	0.11764706	45,0,6,0	chr3,151136	Cytosol	A > G
chr3:15113716:T:C:UTR3:ZFYVE20	chr3	15113716	15113716	T	C	UTR3	ZFYVE20	ENST000002	.	.	A	G	-	-	0.05454545	52,0,3,0	chr3,151137	Cytosol	A > G
chr3:15113733:T:C:UTR3:ZFYVE20	chr3	15113733	15113733	T	C	UTR3	ZFYVE20	ENST000002	.	.	A	G	-	-	0.13953488	37,0,6,0	chr3,151137	Cytosol	A > G
chr3:15113736:T:C:UTR3:ZFYVE20	chr3	15113736	15113736	T	C	UTR3	ZFYVE20	ENST000002	.	.	A	G	-	-	0.12	44,0,6,0	chr3,151137	Cytosol	A > G
chr3:15113777:T:C:UTR3:ZFYVE20	chr3	15113777	15113777	T	C	UTR3	ZFYVE20	ENST000002	.	.	A	G	-	-	0.11764706	45,0,6,0	chr3,151137	Cytosol	A > G
chr3:15113799:T:C:UTR3:ZFYVE20	chr3	15113799	15113799	T	C	UTR3	ZFYVE20	ENST000002	.	.	A	G	-	-	0.18518519	44,0,10,0	chr3,151137	Cytosol	A > G
chr3:15113816:T:C:UTR3:ZFYVE20	chr3	15113816	15113816	T	C	UTR3	ZFYVE20	ENST000002	.	.	A	G	-	-	0.15254237	50,0,9,0	chr3,151138	Cytosol	A > G
chr3:15113830:T:C:UTR3:ZFYVE20	chr3	15113830	15113830	T	C	UTR3	ZFYVE20	ENST000002	.	.	A	G	-	-	0.03448276	56,0,2,0	chr3,151138	Cytosol	A > G
chr3:15113842:T:C:UTR3:ZFYVE20	chr3	15113842	15113842	T	C	UTR3	ZFYVE20	ENST000002	.	.	A	G	-	-	0.18965517	47,0,11,0	chr3,151138	Cytosol	A > G
chr3:15113847:T:C:UTR3:ZFYVE20	chr3	15113847	15113847	T	C	UTR3	ZFYVE20	ENST000002	.	.	A	G	-	-	0.8245614	10,0,47,0	chr3,151138	Cytosol	A > G
chr3:15113855:T:C:UTR3:ZFYVE20	chr3	15113855	15113855	T	C	UTR3	ZFYVE20	ENST000002	.	.	A	G	-	-	0.04444444	43,0,2,0	chr3,151138	Cytosol	A > G
chr3:15113893:T:C:UTR3:ZFYVE20	chr3	15113893	15113893	T	C	UTR3	ZFYVE20	ENST000002	.	.	A	G	-	-	0.50980392	25,0,26,0	chr3,151138	Cytosol	A > G
chr3:15114293:T:C:UTR3:ZFYVE20	chr3	15114293	15114293	T	C	UTR3	ZFYVE20	ENST000002	.	.	A	G	-	-	0.47619048	22,0,20,0	chr3,151142	Cytosol	A > G
chr3:15114306:T:C:UTR3:ZFYVE20	chr3	15114306	15114306	T	C	UTR3	ZFYVE20	ENST000002	.	.	A	G	-	-	0.125	35,0,5,0	chr3,151143	Cytosol	A > G
chr3:15114317:T:C:UTR3:ZFYVE20	chr3	15114317	15114317	T	C	UTR3	ZFYVE20	ENST000002	.	.	A	G	-	-	0.2195122	32,0,9,0	chr3,151143	Cytosol	A > G
chr3:15114359:T:C:UTR3:ZFYVE20	chr3	15114359	15114359	T	C	UTR3	ZFYVE20	ENST000002	.	.	A	G	-	-	0.50819672	30,0,31,0	chr3,151143	Cytosol	A > G
chr3:15114385:T:C:UTR3:ZFYVE20	chr3	15114385	15114385	T	C	UTR3	ZFYVE20	ENST000002	.	.	A	G	-	-	0.046875	61,0,3,0	chr3,151143	Cytosol	A > G
chr3:15114395:T:C:UTR3:ZFYVE20	chr3	15114395	15114395	T	C	UTR3	ZFYVE20	ENST000002	.	.	A	G	-	-	0.21666667	47,0,13,0	chr3,151143	Cytosol	A > G
chr3:15114397:T:C:UTR3:ZFYVE20	chr3	15114397	15114397	T	C	UTR3	ZFYVE20	ENST000002	.	.	A	G	-	-	0.07142857	52,0,4,0	chr3,151143	Cytosol	A > G
chr3:15114399:T:C:UTR3:ZFYVE20	chr3	15114399	15114399	T	C	UTR3	ZFYVE20	ENST000002	.	.	A	G	-	-	0.05263158	54,0,3,0	chr3,151143	Cytosol	A > G
chr3:15114400:T:C:UTR3:ZFYVE20	chr3	15114400	15114400	T	C	UTR3	ZFYVE20	ENST000002	.	.	A	G	-	-	0.24561404	43,0,14,0	chr3,151143	Cytosol	A > G
chr3:15114401:T:C:UTR3:ZFYVE20	chr3	15114401	15114401	T	C	UTR3	ZFYVE20	ENST000002	.	.	A	G	-	-	0.58181818	23,0,32,0	chr3,151144	Cytosol	A > G
chr3:15114404:T:C:UTR3:ZFYVE20	chr3	15114404	15114404	T	C	UTR3	ZFYVE20	ENST000002	.	.	A	G	-	-	0.32692308	35,0,17,0	chr3,151144	Cytosol	A > G
chr3:15114421:T:C:UTR3:ZFYVE20	chr3	15114421	15114421	T	C	UTR3	ZFYVE20	ENST000002	.	.	A	G	-	-	0.07894737	35,0,3,0	chr3,151144	Cytosol	A > G
chr3:15114454:T:C:UTR3:ZFYVE20	chr3	15114454	15114454	T	C	UTR3	ZFYVE20	ENST000002	.	.	A	G	-	-	0.11538462	23,0,3,0	chr3,151144	Cytosol	A > G
chr3:15114486:T:C:UTR3:ZFYVE20	chr3	15114486	15114486	T	C	UTR3	ZFYVE20	ENST000002	.	.	A	G	-	-	0.29411765	24,0,10,0	chr3,151144	Cytosol	A > G
chr3:15114493:T:C:UTR3:ZFYVE20	chr3	15114493	15114493	T	C	UTR3	ZFYVE20	ENST000002	.	.	A	G	-	-	0.04761905	40,0,2,0	chr3,151144	Cytosol	A > G
chr3:15451897:T:C:intronic:METTL	chr3	15451897	15451897	T	C	intronic	METTL6	.	.	.	A	G	-	-	0.13559322	51,0,8,0	chr3,154518	Cytosol	A > G
chr3:15451902:T:C:intronic:METTL	chr3	15451902	15451902	T	C	intronic	METTL6	.	.	.	A	G	-	-	0.38709677	38,0,24,0	chr3,154519	Cytosol	A > G
chr3:15451903:T:C:intronic:METTL	chr3	15451903	15451903	T	C	intronic	METTL6	.	.	.	A	G	-	-	0.12903226	54,0,8,0	chr3,154519	Cytosol	A > G
chr3:15451982:T:C:intronic:METTL	chr3	15451982	15451982	T	C	intronic	METTL6	.	.	.	A	G	-	-	0.01851852	106,0,2,0	chr3,154519	Cytosol	A > G
chr3:15452037:T:C:intronic:METTL	chr3	15452037	15452037	T	C	intronic	METTL6	.	.	.	A	G	-	-	0.02083333	94,0,2,0	chr3,154520	Cytosol	A > G
chr3:15452260:T:C:intronic:METTL	chr3	15452260	15452260	T	C	intronic	METTL6	.	.	.	A	G	-	-	0.18292683	67,0,15,0	chr3,154522	Cytosol	A > G
chr3:15452276:T:C:intronic:METTL	chr3	15452276	15452276	T	C	intronic	METTL6	.	.	.	A	G	-	-	0.05263158	72,0,4,0	chr3,154522	Cytosol	A > G
chr3:15452290:T:C:intronic:METTL	chr3	15452290	15452290	T	C	intronic	METTL6	.	.	.	A	G	-	-	0.28571429	55,0,22,0	chr3,154522	Cytosol	A > G
chr3:15452293:T:C:intronic:METTL	chr3	15452293	15452293	T	C	intronic	METTL6	.	.	.	A	G	-	-	0.27848101	57,0,22,0	chr3,154522	Cytosol	A > G
chr3:15452294:T:C:intronic:METTL	chr3	15452294	15452294	T	C	intronic	METTL6	.	.	.	A	G	-	-	0.29761905	59,0,25,0	chr3,154522	Cytosol	A > G
chr3:15452410:T:C:intronic:METTL	chr3	15452410	15452410	T	C	intronic	METTL6	.	.	.	A	G	-	-	0.125	28,0,4,0	chr3,154524	Cytosol	A > G
chr3:15452496:T:C:intronic:METTL	chr3	15452496	15452496	T	C	intronic	METTL6	.	.	.	A	G	-	-	0.18181818	18,0,4,0	chr3,154524	Cytosol	A > G
chr3:15452537:T:C:UTR3:METTL6	chr3	15452537	15452537	T	C	UTR3	METTL6	ENST000003	.	.	A	G	-	-	0.64912281	20,0,37,0	chr3,154525	Cytosol	A > G
chr3:15452538:T:C:UTR3:METTL6	chr3	15452538	15452538	T	C	UTR3	METTL6	ENST000003	.	.	A	G	-	-	0.25	45,0,15,0	chr3,154525	Cytosol	A > G
chr3:15452554:T:C:UTR3:METTL6	chr3	15452554	15452554	T	C	UTR3	METTL6	ENST000003	.	.	A	G	-	-	0.89090909	6,0,49,0	chr3,154525	Cytosol	A > G
chr3:15453421:T:C:intronic:METTL	chr3	15453421	15453421	T	C	intronic	METTL6	.	.	.	A	G	-	-	0.4	3,0,2,0	chr3,154534	Cytosol	A > G
chr3:15456003:T:C:UTR3:METTL6	chr3	15456003	15456003	T	C	UTR3	METTL6	ENST000003	.	.	A	G	-	-	0.28571429	5,0,2,0	chr3,154560	Cytosol	A > G
chr3:15456245:T:C:UTR3:METTL6	chr3	15456245	15456245	T	C	UTR3	METTL6	ENST000003	.	.	A	G	-	-	0.33333333	4,0,2,0	chr3,154562	Cytosol	A > G
chr3:15456407:T:C:exonic:METTL6	chr3	15456407	15456407	T	C	exonic	METTL6	.	nonsynonym	METTL6:ENS	A	G	-	-	0.28571429	5,0,2,0	chr3,154564	Cytosol	A > G
chr3:16352819:A:G:intronic:OXNA	chr3	16352819	16352819	A	G	intronic	OXNAD1	.	.	.	A	G	+	+	0.6	2,0,3,0	chr3,163528	Cytosol	A > G
chr3:16352831:A:G:intronic:OXNA	chr3	16352831	16352831	A	G	intronic	OXNAD1	.	.	.	A	G	+	+	0.5	3,0,3,0	chr3,163528	Cytosol	A > G

chr3:16382770:A:G:intronic:OXNA	chr3	16382770	16382770	A	G	intronic	OXNAD1,RFT	.	.	.	A	G	+	+-	0.33333333	8,0,4,0	chr3,163827	Cytosol	A > G
chr3:20211661:T:C:UTR3:SGOL1	chr3	20211661	20211661	T	C	UTR3	SGOL1	ENST000004	.	.	A	G	-	-	0.08823529	31,0,3,0	chr3,202116	Cytosol	A > G
chr3:20211814:T:C:UTR3:SGOL1	chr3	20211814	20211814	T	C	UTR3	SGOL1	ENST000004	.	.	A	G	-	-	0.15	17,0,3,0	chr3,202118	Cytosol	A > G
chr3:20211838:T:C:UTR3:SGOL1	chr3	20211838	20211838	T	C	UTR3	SGOL1	ENST000004	.	.	A	G	-	-	0.11111111	16,0,2,0	chr3,202118	Cytosol	A > G
chr3:33164517:A:G:intronic:CRTA	chr3	33164517	33164517	A	G	intronic	CRTAP	.	.	.	A	G	+	+	0.6	2,0,3,0	chr3,331645	Cytosol	A > G
chr3:37212559:T:C:intronic:LRRFI	chr3	37212559	37212559	T	C	intronic	LRRFIP2	.	.	.	A	G	-	-	0.11111111	16,0,2,0	chr3,372125	Cytosol	A > G
chr3:38457652:A:G:intergenic:XYL	chr3	38457652	38457652	A	G	intergenic	XYLB,ACVR2	dist=1185;di	.	.	A	G	+	+	0.92857143	1,0,13,0	chr3,384576	Cytosol	A > G
chr3:38458352:A:G:intergenic:XYL	chr3	38458352	38458352	A	G	intergenic	XYLB,ACVR2	dist=1885;di	.	.	A	G	+	+	1	0,0,8,0	chr3,384583	Cytosol	A > G
chr3:38458364:A:G:intergenic:XYL	chr3	38458364	38458364	A	G	intergenic	XYLB,ACVR2	dist=1897;di	.	.	A	G	+	+	0.38461538	8,0,5,0	chr3,384583	Cytosol	A > G
chr3:38461571:A:G:intergenic:XYL	chr3	38461571	38461571	A	G	intergenic	XYLB,ACVR2	dist=5104;di	.	.	A	G	+	+	0.6	2,0,3,0	chr3,384615	Cytosol	A > G
chr3:38566026:A:G:UTR3:EXOG	chr3	38566026	38566026	A	G	UTR3	EXOG	ENST000004	.	.	A	G	+	+	0.05	38,0,2,0	chr3,385660	Cytosol	A > G
chr3:38567314:A:G:UTR3:EXOG	chr3	38567314	38567314	A	G	UTR3	EXOG	ENST000002	.	.	A	G	+	+	0.08333333	22,0,2,0	chr3,385673	Cytosol	A > G
chr3:38581751:A:G:intronic:EXOG	chr3	38581751	38581751	A	G	intronic	EXOG	.	.	.	A	G	+	+	0.16666667	10,0,2,0	chr3,385817	Cytosol	A > G
chr3:38582255:A:G:intronic:EXOG	chr3	38582255	38582255	A	G	intronic	EXOG	.	.	.	A	G	+	+	0.61538462	5,0,8,0	chr3,385822	Cytosol	A > G
chr3:39450786:A:G:intronic:RPSA	chr3	39450786	39450786	A	G	intronic	RPSA	.	.	.	A	G	+	+	0.16666667	10,0,2,0	chr3,394507	Cytosol	A > G
chr3:40503932:A:G:downstream:	chr3	40503932	40503932	A	G	downstream	RPL14	.	.	.	A	G	+		0.08	23,0,2,0	chr3,405039	Cytosol	A > G
chr3:40504877:A:G:intergenic:RPL	chr3	40504877	40504877	A	G	intergenic	RPL14,RP11-	dist=1016;di	.	.	A	G	+		0.04761905	40,0,2,0	chr3,405048	Cytosol	A > G
chr3:40504894:A:G:intergenic:RPL	chr3	40504894	40504894	A	G	intergenic	RPL14,RP11-	dist=1033;di	.	.	A	G	+		0.13043478	20,0,3,0	chr3,405048	Cytosol	A > G
chr3:40504958:A:G:intergenic:RPL	chr3	40504958	40504958	A	G	intergenic	RPL14,RP11-	dist=1097;di	.	.	A	G	+		0.4375	9,0,7,0	chr3,405049	Cytosol	A > G
chr3:40504959:A:G:intergenic:RPL	chr3	40504959	40504959	A	G	intergenic	RPL14,RP11-	dist=1098;di	.	.	A	G	+		0.4	9,0,6,0	chr3,405049	Cytosol	A > G
chr3:40505512:A:G:intergenic:RPL	chr3	40505512	40505512	A	G	intergenic	RPL14,RP11-	dist=1651;di	.	.	A	G	+		0.04081633	47,0,2,0	chr3,405055	Cytosol	A > G
chr3:40505530:A:G:intergenic:RPL	chr3	40505530	40505530	A	G	intergenic	RPL14,RP11-	dist=1669;di	.	.	A	G	+		0.42424242	38,0,28,0	chr3,405055	Cytosol	A > G
chr3:40505535:A:G:intergenic:RPL	chr3	40505535	40505535	A	G	intergenic	RPL14,RP11-	dist=1674;di	.	.	A	G	+		0.47692308	34,0,31,0	chr3,405055	Cytosol	A > G
chr3:40505554:A:G:intergenic:RPL	chr3	40505554	40505554	A	G	intergenic	RPL14,RP11-	dist=1693;di	.	.	A	G	+		0.27586207	42,0,16,0	chr3,405055	Cytosol	A > G
chr3:40505597:A:G:intergenic:RPL	chr3	40505597	40505597	A	G	intergenic	RPL14,RP11-	dist=1736;di	.	.	A	G	+		0.39215686	31,0,20,0	chr3,405055	Cytosol	A > G
chr3:40505660:A:G:intergenic:RPL	chr3	40505660	40505660	A	G	intergenic	RPL14,RP11-	dist=1799;di	.	.	A	G	+		0.25806452	23,0,8,0	chr3,405056	Cytosol	A > G
chr3:40505685:A:G:intergenic:RPL	chr3	40505685	40505685	A	G	intergenic	RPL14,RP11-	dist=1824;di	.	.	A	G	+		0.37037037	17,0,10,0	chr3,405056	Cytosol	A > G
chr3:40505712:A:G:intergenic:RPL	chr3	40505712	40505712	A	G	intergenic	RPL14,RP11-	dist=1851;di	.	.	A	G	+		0.5	12,0,12,0	chr3,405057	Cytosol	A > G
chr3:40505713:A:G:intergenic:RPL	chr3	40505713	40505713	A	G	intergenic	RPL14,RP11-	dist=1852;di	.	.	A	G	+		0.08	23,0,2,0	chr3,405057	Cytosol	A > G
chr3:40507420:A:G:upstream:RP1	chr3	40507420	40507420	A	G	upstream	RP11-391M1	.	.	.	A	G	+		0.64705882	6,0,11,0	chr3,405074	Cytosol	A > G
chr3:40507426:A:G:upstream:RP1	chr3	40507426	40507426	A	G	upstream	RP11-391M1	.	.	.	A	G	+		0.11111111	16,0,2,0	chr3,405074	Cytosol	A > G
chr3:40507438:A:G:upstream:RP1	chr3	40507438	40507438	A	G	upstream	RP11-391M1	.	.	.	A	G	+		0.5	7,0,7,0	chr3,405074	Cytosol	A > G
chr3:40507922:A:G:upstream:RP1	chr3	40507922	40507922	A	G	upstream	RP11-391M1	.	.	.	A	G	+		0.22413793	45,0,13,0	chr3,405079	Cytosol	A > G
chr3:40507925:A:G:upstream:RP1	chr3	40507925	40507925	A	G	upstream	RP11-391M1	.	.	.	A	G	+		0.47457627	31,0,28,0	chr3,405079	Cytosol	A > G
chr3:40507943:A:G:upstream:RP1	chr3	40507943	40507943	A	G	upstream	RP11-391M1	.	.	.	A	G	+		0.20408163	39,0,10,0	chr3,405079	Cytosol	A > G
chr3:40577649:A:G:UTR3:ZNF621	chr3	40577649	40577649	A	G	UTR3	ZNF621	ENST000003	.	.	A	G	+	+	0.4	3,0,2,0	chr3,405776	Cytosol	A > G
chr3:40577684:A:G:UTR3:ZNF621	chr3	40577684	40577684	A	G	UTR3	ZNF621	ENST000003	.	.	A	G	+	+	0.18181818	9,0,2,0	chr3,405776	Cytosol	A > G
chr3:40577685:A:G:UTR3:ZNF621	chr3	40577685	40577685	A	G	UTR3	ZNF621	ENST000003	.	.	A	G	+	+	0.54545455	5,0,6,0	chr3,405776	Cytosol	A > G
chr3:40577737:A:G:UTR3:ZNF621	chr3	40577737	40577737	A	G	UTR3	ZNF621	ENST000003	.	.	A	G	+	+	0.42857143	8,0,6,0	chr3,405777	Cytosol	A > G
chr3:40577738:A:G:UTR3:ZNF621	chr3	40577738	40577738	A	G	UTR3	ZNF621	ENST000003	.	.	A	G	+	+	0.6875	5,0,11,0	chr3,405777	Cytosol	A > G
chr3:40577745:A:G:UTR3:ZNF621	chr3	40577745	40577745	A	G	UTR3	ZNF621	ENST000003	.	.	A	G	+	+	0.73333333	4,0,11,0	chr3,405777	Cytosol	A > G
chr3:40577746:A:G:UTR3:ZNF621	chr3	40577746	40577746	A	G	UTR3	ZNF621	ENST000003	.	.	A	G	+	+	0.13333333	13,0,2,0	chr3,405777	Cytosol	A > G
chr3:40577752:A:G:UTR3:ZNF621	chr3	40577752	40577752	A	G	UTR3	ZNF621	ENST000003	.	.	A	G	+	+	0.35294118	11,0,6,0	chr3,405777	Cytosol	A > G
chr3:40577755:A:G:UTR3:ZNF621	chr3	40577755	40577755	A	G	UTR3	ZNF621	ENST000003	.	.	A	G	+	+	0.16666667	10,0,2,0	chr3,405777	Cytosol	A > G
chr3:40577781:A:G:UTR3:ZNF621	chr3	40577781	40577781	A	G	UTR3	ZNF621	ENST000003	.	.	A	G	+	+	0.25	6,0,2,0	chr3,405777	Cytosol	A > G
chr3:40578831:A:G:UTR3:ZNF621	chr3	40578831	40578831	A	G	UTR3	ZNF621	ENST000003	.	.	A	G	+	+	0.28571429	20,0,8,0	chr3,405788	Cytosol	A > G
chr3:40578843:A:G:UTR3:ZNF621	chr3	40578843	40578843	A	G	UTR3	ZNF621	ENST000003	.	.	A	G	+	+	0.25	24,0,8,0	chr3,405788	Cytosol	A > G
chr3:40578931:A:G:UTR3:ZNF621	chr3	40578931	40578931	A	G	UTR3	ZNF621	ENST000003	.	.	A	G	+	+	0.39285714	17,0,11,0	chr3,405789	Cytosol	A > G
chr3:40578932:A:G:UTR3:ZNF621	chr3	40578932	40578932	A	G	UTR3	ZNF621	ENST000003	.	.	A	G	+	+	0.53571429	13,0,15,0	chr3,405789	Cytosol	A > G
chr3:40578933:A:G:UTR3:ZNF621	chr3	40578933	40578933	A	G	UTR3	ZNF621	ENST000003	.	.	A	G	+	+	0.39285714	17,0,11,0	chr3,405789	Cytosol	A > G
chr3:40578934:A:G:UTR3:ZNF621	chr3	40578934	40578934	A	G	UTR3	ZNF621	ENST000003	.	.	A	G	+	+	0.33333333	18,0,9,0	chr3,405789	Cytosol	A > G
chr3:40578935:A:G:UTR3:ZNF621	chr3	40578935	40578935	A	G	UTR3	ZNF621	ENST000003	.	.	A	G	+	+	0.13333333	26,0,4,0	chr3,405789	Cytosol	A > G
chr3:40578939:A:G:UTR3:ZNF621	chr3	40578939	40578939	A	G	UTR3	ZNF621	ENST000003	.	.	A	G	+	+	0.90322581	3,0,28,0	chr3,405789	Cytosol	A > G
chr3:40578940:A:G:UTR3:ZNF621	chr3	40578940	40578940	A	G	UTR3	ZNF621	ENST000003	.	.	A	G	+	+	0.20588235	27,0,7,0	chr3,405789	Cytosol	A > G
chr3:40578941:A:G:UTR3:ZNF621	chr3	40578941	40578941	A	G	UTR3	ZNF621	ENST000003	.	.	A	G	+	+	0.17647059	28,0,6,0	chr3,405789	Cytosol	A > G
chr3:40578942:A:G:UTR3:ZNF621	chr3	40578942	40578942	A	G	UTR3	ZNF621	ENST000003	.	.	A	G	+	+	0.11764706	30,0,4,0	chr3,405789	Cytosol	A > G
chr3:40578997:A:G:UTR3:ZNF621	chr3	40578997	40578997	A	G	UTR3	ZNF621	ENST000003	.	.	A	G	+	+	0.3	21,0,9,0	chr3,405789	Cytosol	A > G
chr3:40579748:A:G:intronic:ZNF62	chr3	40579748	40579748	A	G	intronic	ZNF621	.	.	.	A	G	+	+	0.09375	29,0,3,0	chr3,405797	Cytosol	A > G
chr3:40579749:A:G:intronic:ZNF62	chr3	40579749	40579749	A	G	intronic	ZNF621	.	.	.	A	G	+	+	0.09375	29,0,3,0	chr3,405797	Cytosol	A > G
chr3:40579843:A:G:downstream:Z	chr3	40579843	40579843	A	G	downstream	ZNF621	.	.	.	A	G	+	+	0.2	8,0,2,0	chr3,405798	Cytosol	A > G
chr3:44536430:T:C:intergenic:ZNF	chr3	44536430	44536430	T	C	intergenic	ZNF445,ZNF	dist=17268;d	.	.	A	G	-	-	0.6	2,0,3,0	chr3,445364	Cytosol	A > G
chr3:44536486:T:C:intergenic:ZNF	chr3	44536486	44536486	T	C	intergenic	ZNF445,ZNF	dist=17324;d	.	.	A	G	-	-	1	0,0,6,0	chr3,445364	Cytosol	A > G
chr3:44537725:T:C:intergenic:ZNF	chr3	44537725	44537725	T	C	intergenic	ZNF445,ZNF	dist=18563;d	.	.	A	G	-	-	0.28571429	5,0,2,0	chr3,445377	Cytosol	A > G
chr3:44537731:T:C:intergenic:ZNF	chr3	44537731	44537731	T	C	intergenic	ZNF445,ZNF	dist=18569;d	.	.	A	G	-	-	0.28571429	5,0,2,0	chr3,445377	Cytosol	A > G

chr3:44790636:T:C:UTR3:KIAA114	chr3	44790636	44790636	T	C	UTR3	KIAA1143	ENST000002	.	.	A	G	-	-	0.125	35,0,5,0	chr3,447906	Cytosol	A > G
chr3:44790691:T:C:UTR3:KIAA114	chr3	44790691	44790691	T	C	UTR3	KIAA1143	ENST000002	.	.	A	G	-	-	0.06060606	31,0,2,0	chr3,447906	Cytosol	A > G
chr3:44790759:T:C:UTR3:KIAA114	chr3	44790759	44790759	T	C	UTR3	KIAA1143	ENST000002	.	.	A	G	-	-	0.05357143	53,0,3,0	chr3,447907	Cytosol	A > G
chr3:44790773:T:C:UTR3:KIAA114	chr3	44790773	44790773	T	C	UTR3	KIAA1143	ENST000002	.	.	A	G	-	-	0.32075472	36,0,17,0	chr3,447907	Cytosol	A > G
chr3:44790781:T:C:UTR3:KIAA114	chr3	44790781	44790781	T	C	UTR3	KIAA1143	ENST000002	.	.	A	G	-	-	0.50877193	28,0,29,0	chr3,447907	Cytosol	A > G
chr3:44790859:T:C:UTR3:KIAA114	chr3	44790859	44790859	T	C	UTR3	KIAA1143	ENST000002	.	.	A	G	-	-	0.18367347	40,0,9,0	chr3,447908	Cytosol	A > G
chr3:44791351:T:C:UTR3:KIAA114	chr3	44791351	44791351	T	C	UTR3	KIAA1143	ENST000002	.	.	A	G	-	-	0.40740741	16,0,11,0	chr3,447913	Cytosol	A > G
chr3:44791355:T:C:UTR3:KIAA114	chr3	44791355	44791355	T	C	UTR3	KIAA1143	ENST000002	.	.	A	G	-	-	0.14285714	18,0,3,0	chr3,447913	Cytosol	A > G
chr3:44791394:T:C:UTR3:KIAA114	chr3	44791394	44791394	T	C	UTR3	KIAA1143	ENST000002	.	.	A	G	-	-	0.13333333	13,0,2,0	chr3,447913	Cytosol	A > G
chr3:44791397:T:C:UTR3:KIAA114	chr3	44791397	44791397	T	C	UTR3	KIAA1143	ENST000002	.	.	A	G	-	-	0.52631579	9,0,10,0	chr3,447913	Cytosol	A > G
chr3:45721874:A:G:ncRNA_introni	chr3	45721874	45721874	A	G	ncRNA_intro	LIMD1-AS1	.	.	.	A	G	+	+-	0.94444444	2,0,34,0	chr3,457218	Cytosol	A > G
chr3:45721891:A:G:ncRNA_introni	chr3	45721891	45721891	A	G	ncRNA_intro	LIMD1-AS1	.	.	.	A	G	+	+-	0.64583333	17,0,31,0	chr3,457218	Cytosol	A > G
chr3:45721922:A:G:ncRNA_introni	chr3	45721922	45721922	A	G	ncRNA_intro	LIMD1-AS1	.	.	.	A	G	+	+-	0.98507463	1,0,66,0	chr3,457219	Cytosol	A > G
chr3:45721934:A:G:ncRNA_introni	chr3	45721934	45721934	A	G	ncRNA_intro	LIMD1-AS1	.	.	.	A	G	+	+-	0.41176471	50,0,35,0	chr3,457219	Cytosol	A > G
chr3:45721989:A:G:ncRNA_introni	chr3	45721989	45721989	A	G	ncRNA_intro	LIMD1-AS1	.	.	.	A	G	+	+-	0.18867925	43,0,10,0	chr3,457219	Cytosol	A > G
chr3:45721990:A:G:ncRNA_introni	chr3	45721990	45721990	A	G	ncRNA_intro	LIMD1-AS1	.	.	.	A	G	+	+-	0.44	28,0,22,0	chr3,457219	Cytosol	A > G
chr3:45721992:A:G:ncRNA_introni	chr3	45721992	45721992	A	G	ncRNA_intro	LIMD1-AS1	.	.	.	A	G	+	+-	0.38095238	26,0,16,0	chr3,457219	Cytosol	A > G
chr3:45722042:A:G:ncRNA_introni	chr3	45722042	45722042	A	G	ncRNA_intro	LIMD1-AS1	.	.	.	A	G	+	+-	0.25333333	56,0,19,0	chr3,457220	Cytosol	A > G
chr3:45722043:A:G:ncRNA_introni	chr3	45722043	45722043	A	G	ncRNA_intro	LIMD1-AS1	.	.	.	A	G	+	+-	0.375	45,0,27,0	chr3,457220	Cytosol	A > G
chr3:45722047:A:G:ncRNA_introni	chr3	45722047	45722047	A	G	ncRNA_intro	LIMD1-AS1	.	.	.	A	G	+	+-	0.52173913	33,0,36,0	chr3,457220	Cytosol	A > G
chr3:45722100:A:G:ncRNA_introni	chr3	45722100	45722100	A	G	ncRNA_intro	LIMD1-AS1	.	.	.	A	G	+	+-	0.74324324	19,0,55,0	chr3,457220	Cytosol	A > G
chr3:45722287:A:G:ncRNA_introni	chr3	45722287	45722287	A	G	ncRNA_intro	LIMD1-AS1	.	.	.	A	G	+	+-	0.57142857	12,0,16,0	chr3,457222	Cytosol	A > G
chr3:45722300:A:G:ncRNA_introni	chr3	45722300	45722300	A	G	ncRNA_intro	LIMD1-AS1	.	.	.	A	G	+	+-	0.41176471	20,0,14,0	chr3,457222	Cytosol	A > G
chr3:45722327:A:G:ncRNA_introni	chr3	45722327	45722327	A	G	ncRNA_intro	LIMD1-AS1	.	.	.	A	G	+	+-	0.14634146	35,0,6,0	chr3,457223	Cytosol	A > G
chr3:45722341:A:G:ncRNA_introni	chr3	45722341	45722341	A	G	ncRNA_intro	LIMD1-AS1	.	.	.	A	G	+	+-	0.21153846	41,0,11,0	chr3,457223	Cytosol	A > G
chr3:45722342:A:G:ncRNA_introni	chr3	45722342	45722342	A	G	ncRNA_intro	LIMD1-AS1	.	.	.	A	G	+	+-	0.19298246	46,0,11,0	chr3,457223	Cytosol	A > G
chr3:45722381:A:G:ncRNA_introni	chr3	45722381	45722381	A	G	ncRNA_intro	LIMD1-AS1	.	.	.	A	G	+	+-	0.48	26,0,24,0	chr3,457223	Cytosol	A > G
chr3:45722387:A:G:ncRNA_introni	chr3	45722387	45722387	A	G	ncRNA_intro	LIMD1-AS1	.	.	.	A	G	+	+-	0.18367347	40,0,9,0	chr3,457223	Cytosol	A > G
chr3:45722408:A:G:ncRNA_introni	chr3	45722408	45722408	A	G	ncRNA_intro	LIMD1-AS1	.	.	.	A	G	+	+-	0.4	15,0,10,0	chr3,457224	Cytosol	A > G
chr3:45722422:A:G:ncRNA_introni	chr3	45722422	45722422	A	G	ncRNA_intro	LIMD1-AS1	.	.	.	A	G	+	+-	0.51851852	13,0,14,0	chr3,457224	Cytosol	A > G
chr3:45722423:A:G:ncRNA_introni	chr3	45722423	45722423	A	G	ncRNA_intro	LIMD1-AS1	.	.	.	A	G	+	+-	0.44444444	15,0,12,0	chr3,457224	Cytosol	A > G
chr3:45722430:A:G:ncRNA_introni	chr3	45722430	45722430	A	G	ncRNA_intro	LIMD1-AS1	.	.	.	A	G	+	+-	0.41666667	14,0,10,0	chr3,457224	Cytosol	A > G
chr3:45722434:A:G:ncRNA_introni	chr3	45722434	45722434	A	G	ncRNA_intro	LIMD1-AS1	.	.	.	A	G	+	+-	0.85714286	3,0,18,0	chr3,457224	Cytosol	A > G
chr3:45722452:A:G:ncRNA_introni	chr3	45722452	45722452	A	G	ncRNA_intro	LIMD1-AS1	.	.	.	A	G	+	+-	0.08571429	32,0,3,0	chr3,457224	Cytosol	A > G
chr3:45722491:A:G:ncRNA_introni	chr3	45722491	45722491	A	G	ncRNA_intro	LIMD1-AS1	.	.	.	A	G	+	+-	0.09433962	48,0,5,0	chr3,457224	Cytosol	A > G
chr3:47553978:T:C:intronic:ELP6	chr3	47553978	47553978	T	C	intronic	ELP6	.	.	.	A	G	-	-	0.57142857	3,0,4,0	chr3,475539	Cytosol	A > G
chr3:48714021:T:C:intronic:NCKIP	chr3	48714021	48714021	T	C	intronic	NCKIPSD	.	.	.	A	G	-	-	0.4	3,0,2,0	chr3,487140	Cytosol	A > G
chr3:48714045:T:C:intronic:NCKIP	chr3	48714045	48714045	T	C	intronic	NCKIPSD	.	.	.	A	G	-	-	0.28571429	5,0,2,0	chr3,487140	Cytosol	A > G
chr3:48714051:T:C:intronic:NCKIP	chr3	48714051	48714051	T	C	intronic	NCKIPSD	.	.	.	A	G	-	-	0.5	4,0,4,0	chr3,487140	Cytosol	A > G
chr3:48714341:T:C:intronic:NCKIP	chr3	48714341	48714341	T	C	intronic	NCKIPSD	.	.	.	A	G	-	-	0.5	3,0,3,0	chr3,487143	Cytosol	A > G
chr3:48714342:T:C:intronic:NCKIP	chr3	48714342	48714342	T	C	intronic	NCKIPSD	.	.	.	A	G	-	-	1	0,0,6,0	chr3,487143	Cytosol	A > G
chr3:48714368:T:C:intronic:NCKIP	chr3	48714368	48714368	T	C	intronic	NCKIPSD	.	.	.	A	G	-	-	0.8	1,0,4,0	chr3,487143	Cytosol	A > G
chr3:48786945:T:C:UTR3:PRKAR2	chr3	48786945	48786945	T	C	UTR3	PRKAR2A	ENST000002	.	.	A	G	-	-	0.01657459	178,0,3,0	chr3,487869	Cytosol	A > G
chr3:48786991:T:C:UTR3:PRKAR2	chr3	48786991	48786991	T	C	UTR3	PRKAR2A	ENST000002	.	.	A	G	-	-	0.6474359	55,0,101,0	chr3,487869	Cytosol	A > G
chr3:48787011:T:C:UTR3:PRKAR2	chr3	48787011	48787011	T	C	UTR3	PRKAR2A	ENST000002	.	.	A	G	-	-	0.13709677	107,0,17,0	chr3,487870	Cytosol	A > G
chr3:48787035:T:C:UTR3:PRKAR2	chr3	48787035	48787035	T	C	UTR3	PRKAR2A	ENST000002	.	.	A	G	-	-	0.06666667	56,0,4,0	chr3,487870	Cytosol	A > G
chr3:48787042:T:C:UTR3:PRKAR2	chr3	48787042	48787042	T	C	UTR3	PRKAR2A	ENST000002	.	.	A	G	-	-	0.42307692	45,0,33,0	chr3,487870	Cytosol	A > G
chr3:48787051:T:C:UTR3:PRKAR2	chr3	48787051	48787051	T	C	UTR3	PRKAR2A	ENST000002	.	.	A	G	-	-	0.21	79,0,21,0	chr3,487870	Cytosol	A > G
chr3:48787088:T:C:UTR3:PRKAR2	chr3	48787088	48787088	T	C	UTR3	PRKAR2A	ENST000002	.	.	A	G	-	-	0.06329114	148,0,10,0	chr3,487870	Cytosol	A > G
chr3:48787108:T:C:UTR3:PRKAR2	chr3	48787108	48787108	T	C	UTR3	PRKAR2A	ENST000002	.	.	A	G	-	-	0.15454545	93,0,17,0	chr3,487871	Cytosol	A > G
chr3:48787109:T:C:UTR3:PRKAR2	chr3	48787109	48787109	T	C	UTR3	PRKAR2A	ENST000002	.	.	A	G	-	-	0.13821138	106,0,17,0	chr3,487871	Cytosol	A > G
chr3:48787114:T:C:UTR3:PRKAR2	chr3	48787114	48787114	T	C	UTR3	PRKAR2A	ENST000002	.	.	A	G	-	-	0.128	109,0,16,0	chr3,487871	Cytosol	A > G
chr3:48787118:T:C:UTR3:PRKAR2	chr3	48787118	48787118	T	C	UTR3	PRKAR2A	ENST000002	.	.	A	G	-	-	0.33663366	67,0,34,0	chr3,487871	Cytosol	A > G
chr3:48787452:T:C:UTR3:PRKAR2	chr3	48787452	48787452	T	C	UTR3	PRKAR2A	ENST000002	.	.	A	G	-	-	0.11538462	92,0,12,0	chr3,487874	Cytosol	A > G
chr3:48787466:T:C:UTR3:PRKAR2	chr3	48787466	48787466	T	C	UTR3	PRKAR2A	ENST000002	.	.	A	G	-	-	0.08791209	83,0,8,0	chr3,487874	Cytosol	A > G
chr3:48787562:T:C:UTR3:PRKAR2	chr3	48787562	48787562	T	C	UTR3	PRKAR2A	ENST000002	.	.	A	G	-	-	0.30434783	64,0,28,0	chr3,487875	Cytosol	A > G
chr3:48787580:T:C:UTR3:PRKAR2	chr3	48787580	48787580	T	C	UTR3	PRKAR2A	ENST000002	.	.	A	G	-	-	0.05454545	104,0,6,0	chr3,487875	Cytosol	A > G
chr3:48787634:T:C:UTR3:PRKAR2	chr3	48787634	48787634	T	C	UTR3	PRKAR2A	ENST000002	.	.	A	G	-	-	0.20212766	75,0,19,0	chr3,487876	Cytosol	A > G
chr3:48787674:T:C:UTR3:PRKAR2	chr3	48787674	48787674	T	C	UTR3	PRKAR2A	ENST000002	.	.	A	G	-	-	0.38541667	59,0,37,0	chr3,487876	Cytosol	A > G
chr3:48787684:T:C:UTR3:PRKAR2	chr3	48787684	48787684	T	C	UTR3	PRKAR2A	ENST000002	.	.	A	G	-	-	0.61864407	45,0,73,0	chr3,487876	Cytosol	A > G
chr3:48787715:T:C:UTR3:PRKAR2	chr3	48787715	48787715	T	C	UTR3	PRKAR2A	ENST000002	.	.	A	G	-	-	0.11464968	139,0,18,0	chr3,487877	Cytosol	A > G
chr3:48787733:T:C:UTR3:PRKAR2	chr3	48787733	48787733	T	C	UTR3	PRKAR2A	ENST000002	.	.	A	G	-	-	0.31944444	98,0,46,0	chr3,487877	Cytosol	A > G
chr3:48787734:T:C:UTR3:PRKAR2	chr3	48787734	48787734	T	C	UTR3	PRKAR2A	ENST000002	.	.	A	G	-	-	0.09459459	134,0,14,0	chr3,487877	Cytosol	A > G
chr3:48787757:T:C:UTR3:PRKAR2	chr3	48787757	48787757	T	C	UTR3	PRKAR2A	ENST000002	.	.	A	G	-	-	0.03278689	118,0,4,0	chr3,487877	Cytosol	A > G

chr3:48787856:T:C:UTR3:PRKAR2A	chr3	48787856	48787856	T	C	UTR3	PRKAR2A	ENST000002	.	.	A	G	-	-	0.37333333	47,0,28,0	chr3,487878	Cytosol	A > G
chr3:48787909:T:C:UTR3:PRKAR2A	chr3	48787909	48787909	T	C	UTR3	PRKAR2A	ENST000002	.	.	A	G	-	-	0.26315789	98,0,35,0	chr3,487879	Cytosol	A > G
chr3:49452695:A:G:UTR3:TCTA	chr3	49452695	49452695	A	G	UTR3	TCTA	ENST000002	.	.	A	G	+	+	0.02222222	88,0,2,0	chr3,494526	Cytosol	A > G
chr3:49452787:A:G:UTR3:TCTA	chr3	49452787	49452787	A	G	UTR3	TCTA	ENST000002	.	.	A	G	+	+	0.06122449	92,0,6,0	chr3,494527	Cytosol	A > G
chr3:49452802:A:G:UTR3:TCTA	chr3	49452802	49452802	A	G	UTR3	TCTA	ENST000002	.	.	A	G	+	+	0.03157895	92,0,3,0	chr3,494528	Cytosol	A > G
chr3:49452829:A:G:UTR3:TCTA	chr3	49452829	49452829	A	G	UTR3	TCTA	ENST000002	.	.	A	G	+	+	0.01666667	118,0,2,0	chr3,494528	Cytosol	A > G
chr3:49452852:A:G:UTR3:TCTA	chr3	49452852	49452852	A	G	UTR3	TCTA	ENST000002	.	.	A	G	+	+	0.02531646	77,0,2,0	chr3,494528	Cytosol	A > G
chr3:49460945:T:C:ncRNA_exonic:	chr3	49460945	49460945	T	C	ncRNA_exon	NICN1-AS1	.	.	.	A	G	-	+-	0.83333333	1,0,5,0	chr3,494609	Cytosol	A > G
chr3:49460946:T:C:ncRNA_exonic:	chr3	49460946	49460946	T	C	ncRNA_exon	NICN1-AS1	.	.	.	A	G	-	+-	0.83333333	1,0,5,0	chr3,494609	Cytosol	A > G
chr3:49460973:T:C:ncRNA_introni	chr3	49460973	49460973	T	C	ncRNA_intro	NICN1-AS1	.	.	.	A	G	-	+-	0.83333333	1,0,5,0	chr3,494609	Cytosol	A > G
chr3:49461067:T:C:ncRNA_introni	chr3	49461067	49461067	T	C	ncRNA_intro	NICN1-AS1	.	.	.	A	G	-	+-	0.3	7,0,3,0	chr3,494610	Cytosol	A > G
chr3:49461089:T:C:ncRNA_introni	chr3	49461089	49461089	T	C	ncRNA_intro	NICN1-AS1	.	.	.	A	G	-	+-	0.46666667	8,0,7,0	chr3,494610	Cytosol	A > G
chr3:49461106:T:C:ncRNA_introni	chr3	49461106	49461106	T	C	ncRNA_intro	NICN1-AS1	.	.	.	A	G	-	+-	0.78571429	3,0,11,0	chr3,494611	Cytosol	A > G
chr3:49461113:T:C:ncRNA_introni	chr3	49461113	49461113	T	C	ncRNA_intro	NICN1-AS1	.	.	.	A	G	-	+-	0.70588235	5,0,12,0	chr3,494611	Cytosol	A > G
chr3:49461379:T:C:ncRNA_introni	chr3	49461379	49461379	T	C	ncRNA_intro	NICN1-AS1	.	.	.	A	G	-	+-	0.78571429	3,0,11,0	chr3,494613	Cytosol	A > G
chr3:49461421:T:C:ncRNA_introni	chr3	49461421	49461421	T	C	ncRNA_intro	NICN1-AS1	.	.	.	A	G	-	+-	0.57142857	3,0,4,0	chr3,494614	Cytosol	A > G
chr3:52449313:A:G:intronic:PHF7	chr3	52449313	52449313	A	G	intronic	PHF7	.	.	.	A	G	+	+	0.5	3,0,3,0	chr3,524493	Cytosol	A > G
chr3:53897910:T:C:intronic:IL17RE	chr3	53897910	53897910	T	C	intronic	IL17RB	.	.	.	A	G	-	+	0.75862069	7,0,22,0	chr3,538979	Cytosol	A > G
chr3:53897915:T:C:intronic:IL17RE	chr3	53897915	53897915	T	C	intronic	IL17RB	.	.	.	A	G	-	+	0.24137931	22,0,7,0	chr3,538979	Cytosol	A > G
chr3:53897935:T:C:intronic:IL17RE	chr3	53897935	53897935	T	C	intronic	IL17RB	.	.	.	A	G	-	+	0.76470588	8,0,26,0	chr3,538979	Cytosol	A > G
chr3:53897974:T:C:intronic:IL17RE	chr3	53897974	53897974	T	C	intronic	IL17RB	.	.	.	A	G	-	+	0.71428571	12,0,30,0	chr3,538979	Cytosol	A > G
chr3:53897998:T:C:intronic:IL17RE	chr3	53897998	53897998	T	C	intronic	IL17RB	.	.	.	A	G	-	+	0.38888889	22,0,14,0	chr3,538979	Cytosol	A > G
chr3:53898014:T:C:intronic:IL17RE	chr3	53898014	53898014	T	C	intronic	IL17RB	.	.	.	A	G	-	+	0.15909091	37,0,7,0	chr3,538980	Cytosol	A > G
chr3:53898023:T:C:intronic:IL17RE	chr3	53898023	53898023	T	C	intronic	IL17RB	.	.	.	A	G	-	+	0.08	46,0,4,0	chr3,538980	Cytosol	A > G
chr3:53898038:T:C:intronic:IL17RE	chr3	53898038	53898038	T	C	intronic	IL17RB	.	.	.	A	G	-	+	0.22222222	28,0,8,0	chr3,538980	Cytosol	A > G
chr3:53898066:T:C:intronic:IL17RE	chr3	53898066	53898066	T	C	intronic	IL17RB	.	.	.	A	G	-	+	0.08823529	31,0,3,0	chr3,538980	Cytosol	A > G
chr3:53898092:T:C:intronic:IL17RE	chr3	53898092	53898092	T	C	intronic	IL17RB	.	.	.	A	G	-	+	0.06451613	29,0,2,0	chr3,538980	Cytosol	A > G
chr3:53898129:T:C:intronic:IL17RE	chr3	53898129	53898129	T	C	intronic	IL17RB	.	.	.	A	G	-	+	0.63636364	12,0,21,0	chr3,538981	Cytosol	A > G
chr3:53898382:T:C:intronic:IL17RE	chr3	53898382	53898382	T	C	intronic	IL17RB	.	.	.	A	G	-	+	0.6	4,0,6,0	chr3,538983	Cytosol	A > G
chr3:53898399:T:C:intronic:IL17RE	chr3	53898399	53898399	T	C	intronic	IL17RB	.	.	.	A	G	-	+	0.55555556	8,0,10,0	chr3,538983	Cytosol	A > G
chr3:53898405:T:C:intronic:IL17RE	chr3	53898405	53898405	T	C	intronic	IL17RB	.	.	.	A	G	-	+	0.45	11,0,9,0	chr3,538984	Cytosol	A > G
chr3:53898502:T:C:intronic:IL17RE	chr3	53898502	53898502	T	C	intronic	IL17RB	.	.	.	A	G	-	+	0.82142857	5,0,23,0	chr3,538985	Cytosol	A > G
chr3:53900289:T:C:downstream:A	chr3	53900289	53900289	T	C	downstream	ACTR8,IL17R	.	.	.	A	G	-		0.25925926	20,0,7,0	chr3,539002	Cytosol	A > G
chr3:56603924:A:G:UTR3:CCDC66	chr3	56603924	56603924	A	G	UTR3	CCDC66	ENST000004	.	.	A	G	+	+	0.6	2,0,3,0	chr3,566039	Cytosol	A > G
chr3:56603962:A:G:UTR3:CCDC66	chr3	56603962	56603962	A	G	UTR3	CCDC66	ENST000004	.	.	A	G	+	+	0.375	5,0,3,0	chr3,566039	Cytosol	A > G
chr3:56603963:A:G:UTR3:CCDC66	chr3	56603963	56603963	A	G	UTR3	CCDC66	ENST000004	.	.	A	G	+	+	0.625	3,0,5,0	chr3,566039	Cytosol	A > G
chr3:56603992:A:G:UTR3:CCDC66	chr3	56603992	56603992	A	G	UTR3	CCDC66	ENST000004	.	.	A	G	+	+	0.84615385	2,0,11,0	chr3,566039	Cytosol	A > G
chr3:56604044:A:G:UTR3:CCDC66	chr3	56604044	56604044	A	G	UTR3	CCDC66	ENST000004	.	.	A	G	+	+	0.3	7,0,3,0	chr3,566040	Cytosol	A > G
chr3:56604047:A:G:UTR3:CCDC66	chr3	56604047	56604047	A	G	UTR3	CCDC66	ENST000004	.	.	A	G	+	+	1	0,0,8,0	chr3,566040	Cytosol	A > G
chr3:56604092:A:G:UTR3:CCDC66	chr3	56604092	56604092	A	G	UTR3	CCDC66	ENST000004	.	.	A	G	+	+	1	0,0,6,0	chr3,566040	Cytosol	A > G
chr3:56604524:A:G:UTR3:CCDC66	chr3	56604524	56604524	A	G	UTR3	CCDC66	ENST000004	.	.	A	G	+	+	0.27777778	13,0,5,0	chr3,566045	Cytosol	A > G
chr3:56604568:A:G:UTR3:CCDC66	chr3	56604568	56604568	A	G	UTR3	CCDC66	ENST000004	.	.	A	G	+	+	0.25	6,0,2,0	chr3,566045	Cytosol	A > G
chr3:56604655:A:G:UTR3:CCDC66	chr3	56604655	56604655	A	G	UTR3	CCDC66	ENST000004	.	.	A	G	+	+	0.8	1,0,4,0	chr3,566046	Cytosol	A > G
chr3:56604665:A:G:UTR3:CCDC66	chr3	56604665	56604665	A	G	UTR3	CCDC66	ENST000004	.	.	A	G	+	+	0.4	3,0,2,0	chr3,566046	Cytosol	A > G
chr3:56604669:A:G:UTR3:CCDC66	chr3	56604669	56604669	A	G	UTR3	CCDC66	ENST000004	.	.	A	G	+	+	0.6	2,0,3,0	chr3,566046	Cytosol	A > G
chr3:57124571:T:C:UTR3:IL17RD	chr3	57124571	57124571	T	C	UTR3	IL17RD	ENST000002	.	.	A	G	-	-	0.25	6,0,2,0	chr3,571245	Cytosol	A > G
chr3:57124572:T:C:UTR3:IL17RD	chr3	57124572	57124572	T	C	UTR3	IL17RD	ENST000002	.	.	A	G	-	-	0.25	6,0,2,0	chr3,571245	Cytosol	A > G
chr3:57126452:T:C:UTR3:IL17RD	chr3	57126452	57126452	T	C	UTR3	IL17RD	ENST000002	.	.	A	G	-	-	0.33333333	4,0,2,0	chr3,571264	Cytosol	A > G
chr3:57548917:A:G:UTR3:PDE12	chr3	57548917	57548917	A	G	UTR3	PDE12	ENST000003	.	.	A	G	+	+	0.2	8,0,2,0	chr3,575489	Cytosol	A > G
chr3:57548924:A:G:UTR3:PDE12	chr3	57548924	57548924	A	G	UTR3	PDE12	ENST000003	.	.	A	G	+	+	0.33333333	6,0,3,0	chr3,575489	Cytosol	A > G
chr3:57548976:A:G:UTR3:PDE12	chr3	57548976	57548976	A	G	UTR3	PDE12	ENST000003	.	.	A	G	+	+	0.5	5,0,5,0	chr3,575489	Cytosol	A > G
chr3:57549504:A:G:UTR3:PDE12	chr3	57549504	57549504	A	G	UTR3	PDE12	ENST000003	.	.	A	G	+	+	0.6	6,0,9,0	chr3,575495	Cytosol	A > G
chr3:57612180:T:C:UTR3:DENND6	chr3	57612180	57612180	T	C	UTR3	DENND6A	ENST000003	.	.	A	G	-	-	0.48148148	28,0,26,0	chr3,576121	Cytosol	A > G
chr3:57612192:T:C:UTR3:DENND6	chr3	57612192	57612192	T	C	UTR3	DENND6A	ENST000003	.	.	A	G	-	-	0.44897959	27,0,22,0	chr3,576121	Cytosol	A > G
chr3:57612193:T:C:UTR3:DENND6	chr3	57612193	57612193	T	C	UTR3	DENND6A	ENST000003	.	.	A	G	-	-	0.14	43,0,7,0	chr3,576121	Cytosol	A > G
chr3:57612271:T:C:UTR3:DENND6	chr3	57612271	57612271	T	C	UTR3	DENND6A	ENST000003	.	.	A	G	-	-	0.44444444	25,0,20,0	chr3,576122	Cytosol	A > G
chr3:57612274:T:C:UTR3:DENND6	chr3	57612274	57612274	T	C	UTR3	DENND6A	ENST000003	.	.	A	G	-	-	0.12	44,0,6,0	chr3,576122	Cytosol	A > G
chr3:57612287:T:C:UTR3:DENND6	chr3	57612287	57612287	T	C	UTR3	DENND6A	ENST000003	.	.	A	G	-	-	0.10869565	41,0,5,0	chr3,576122	Cytosol	A > G
chr3:57612314:T:C:UTR3:DENND6	chr3	57612314	57612314	T	C	UTR3	DENND6A	ENST000003	.	.	A	G	-	-	0.15789474	48,0,9,0	chr3,576123	Cytosol	A > G
chr3:57612324:T:C:UTR3:DENND6	chr3	57612324	57612324	T	C	UTR3	DENND6A	ENST000003	.	.	A	G	-	-	0.03636364	53,0,2,0	chr3,576123	Cytosol	A > G
chr3:57612389:T:C:UTR3:DENND6	chr3	57612389	57612389	T	C	UTR3	DENND6A	ENST000003	.	.	A	G	-	-	0.22222222	70,0,20,0	chr3,576123	Cytosol	A > G
chr3:57612931:T:C:UTR3:DENND6	chr3	57612931	57612931	T	C	UTR3	DENND6A	ENST000003	.	.	A	G	-	-	0.08888889	41,0,4,0	chr3,576129	Cytosol	A > G
chr3:57612933:T:C:UTR3:DENND6	chr3	57612933	57612933	T	C	UTR3	DENND6A	ENST000003	.	.	A	G	-	-	0.31914894	32,0,15,0	chr3,576129	Cytosol	A > G
chr3:57612967:T:C:UTR3:DENND6	chr3	57612967	57612967	T	C	UTR3	DENND6A	ENST000003	.	.	A	G	-	-	0.0483871	59,0,3,0	chr3,576129	Cytosol	A > G

chr3:57612968:T:C:UTR3:DENND6	chr3	57612968	57612968	T	C	UTR3	DENND6A	ENST000003	.	.	A	G	-	-	0.04545455	63,0,3,0	chr3,576129	Cytosol	A > G
chr3:57612977:T:C:UTR3:DENND6	chr3	57612977	57612977	T	C	UTR3	DENND6A	ENST000003	.	.	A	G	-	-	0.05970149	63,0,4,0	chr3,576129	Cytosol	A > G
chr3:57613008:T:C:UTR3:DENND6	chr3	57613008	57613008	T	C	UTR3	DENND6A	ENST000003	.	.	A	G	-	-	0.20930233	34,0,9,0	chr3,576130	Cytosol	A > G
chr3:57613035:T:C:UTR3:DENND6	chr3	57613035	57613035	T	C	UTR3	DENND6A	ENST000003	.	.	A	G	-	-	0.36363636	28,0,16,0	chr3,576130	Cytosol	A > G
chr3:57613044:T:C:UTR3:DENND6	chr3	57613044	57613044	T	C	UTR3	DENND6A	ENST000003	.	.	A	G	-	-	0.03921569	49,0,2,0	chr3,576130	Cytosol	A > G
chr3:57613046:T:C:UTR3:DENND6	chr3	57613046	57613046	T	C	UTR3	DENND6A	ENST000003	.	.	A	G	-	-	0.06382979	44,0,3,0	chr3,576130	Cytosol	A > G
chr3:57613047:T:C:UTR3:DENND6	chr3	57613047	57613047	T	C	UTR3	DENND6A	ENST000003	.	.	A	G	-	-	0.0625	45,0,3,0	chr3,576130	Cytosol	A > G
chr3:57613049:T:C:UTR3:DENND6	chr3	57613049	57613049	T	C	UTR3	DENND6A	ENST000003	.	.	A	G	-	-	0.11111111	40,0,5,0	chr3,576130	Cytosol	A > G
chr3:57613053:T:C:UTR3:DENND6	chr3	57613053	57613053	T	C	UTR3	DENND6A	ENST000003	.	.	A	G	-	-	0.34782609	30,0,16,0	chr3,576130	Cytosol	A > G
chr3:57613064:T:C:UTR3:DENND6	chr3	57613064	57613064	T	C	UTR3	DENND6A	ENST000003	.	.	A	G	-	-	0.08108108	34,0,3,0	chr3,576130	Cytosol	A > G
chr3:57613113:T:C:UTR3:DENND6	chr3	57613113	57613113	T	C	UTR3	DENND6A	ENST000003	.	.	A	G	-	-	0.24242424	25,0,8,0	chr3,576131	Cytosol	A > G
chr3:57613122:T:C:UTR3:DENND6	chr3	57613122	57613122	T	C	UTR3	DENND6A	ENST000003	.	.	A	G	-	-	0.2195122	32,0,9,0	chr3,576131	Cytosol	A > G
chr3:57613127:T:C:UTR3:DENND6	chr3	57613127	57613127	T	C	UTR3	DENND6A	ENST000003	.	.	A	G	-	-	0.10526316	34,0,4,0	chr3,576131	Cytosol	A > G
chr3:57613144:T:C:UTR3:DENND6	chr3	57613144	57613144	T	C	UTR3	DENND6A	ENST000003	.	.	A	G	-	-	0.35294118	22,0,12,0	chr3,576131	Cytosol	A > G
chr3:58304129:A:G:UTR3:RPP14	chr3	58304129	58304129	A	G	UTR3	RPP14	ENST000004	.	.	A	G	+	+	0.01941748	101,0,2,0	chr3,583041	Cytosol	A > G
chr3:58304338:A:G:UTR3:RPP14	chr3	58304338	58304338	A	G	UTR3	RPP14	ENST000004	.	.	A	G	+	+	0.03149606	123,0,4,0	chr3,583043	Cytosol	A > G
chr3:58306587:A:G:UTR3:RPP14	chr3	58306587	58306587	A	G	UTR3	RPP14	ENST000004	.	.	A	G	+	+	0.3	7,0,3,0	chr3,583065	Cytosol	A > G
chr3:58306666:A:G:UTR3:RPP14	chr3	58306666	58306666	A	G	UTR3	RPP14	ENST000004	.	.	A	G	+	+	0.16666667	10,0,2,0	chr3,583066	Cytosol	A > G
chr3:58306671:A:G:UTR3:RPP14	chr3	58306671	58306671	A	G	UTR3	RPP14	ENST000004	.	.	A	G	+	+	0.3	7,0,3,0	chr3,583066	Cytosol	A > G
chr3:58306728:A:G:UTR3:RPP14	chr3	58306728	58306728	A	G	UTR3	RPP14	ENST000004	.	.	A	G	+	+	0.5	11,0,11,0	chr3,583067	Cytosol	A > G
chr3:58306729:A:G:UTR3:RPP14	chr3	58306729	58306729	A	G	UTR3	RPP14	ENST000004	.	.	A	G	+	+	0.7826087	5,0,18,0	chr3,583067	Cytosol	A > G
chr3:58306797:A:G:UTR3:RPP14	chr3	58306797	58306797	A	G	UTR3	RPP14	ENST000004	.	.	A	G	+	+	0.25	15,0,5,0	chr3,583067	Cytosol	A > G
chr3:61728164:C:T:intronic:PTPRG	chr3	61728164	61728164	C	T	intronic	PTPRG	.	.	.	G	A	-	+-	1	10,0,0,0	chr3,617281	Cytosol	G > A
chr3:69070907:T:C:ncRNA_introni	chr3	69070907	69070907	T	C	ncRNA_intro	CTD-2013N2	.	.	.	A	G	-	+-	0.03636364	53,0,2,0	chr3,690709	Cytosol	A > G
chr3:69070910:T:C:ncRNA_introni	chr3	69070910	69070910	T	C	ncRNA_intro	CTD-2013N2	.	.	.	A	G	-	+-	0.08	46,0,4,0	chr3,690709	Cytosol	A > G
chr3:75465964:T:C:intergenic:AC1	chr3	75465964	75465964	T	C	intergenic	AC139453.1,	dist=47467;d	.	.	A	G	-	+	0.8	1,0,4,0	chr3,754659	Cytosol	A > G
chr3:93778214:T:C:UTR3:DHFRL1	chr3	93778214	93778214	T	C	UTR3	DHFRL1	ENST000003	.	.	A	G	-	-	0.19230769	21,0,5,0	chr3,937782	Cytosol	A > G
chr3:93846809:A:G:UTR3:NSUN3	chr3	93846809	93846809	A	G	UTR3	NSUN3	ENST000003	.	.	A	G	+	+	0.17391304	19,0,4,0	chr3,938468	Cytosol	A > G
chr3:93846810:A:G:UTR3:NSUN3	chr3	93846810	93846810	A	G	UTR3	NSUN3	ENST000003	.	.	A	G	+	+	0.27272727	16,0,6,0	chr3,938468	Cytosol	A > G
chr3:93846961:A:G:UTR3:NSUN3	chr3	93846961	93846961	A	G	UTR3	NSUN3	ENST000003	.	.	A	G	+	+	0.13636364	19,0,3,0	chr3,938469	Cytosol	A > G
chr3:93847017:A:G:UTR3:NSUN3	chr3	93847017	93847017	A	G	UTR3	NSUN3	ENST000003	.	.	A	G	+	+	0.33333333	6,0,3,0	chr3,938470	Cytosol	A > G
chr3:93848667:A:G:intergenic:NSU	chr3	93848667	93848667	A	G	intergenic	NSUN3,LINC	dist=1278;di	.	.	A	G	+		0.85714286	1,0,6,0	chr3,938486	Cytosol	A > G
chr3:101487418:A:G:UTR3:CEP97	chr3	101487418	101487418	A	G	UTR3	CEP97	ENST000003	.	.	A	G	+	+	0.38461538	8,0,5,0	chr3,101487	Cytosol	A > G
chr3:101487424:A:G:UTR3:CEP97	chr3	101487424	101487424	A	G	UTR3	CEP97	ENST000003	.	.	A	G	+	+	0.16666667	10,0,2,0	chr3,101487	Cytosol	A > G
chr3:101521765:A:G:intronic:NXPE	chr3	101521765	101521765	A	G	intronic	NXPE3	.	.	.	A	G	+	+	0.72727273	3,0,8,0	chr3,101521	Cytosol	A > G
chr3:101521811:A:G:intronic:NXPE	chr3	101521811	101521811	A	G	intronic	NXPE3	.	.	.	A	G	+	+	0.66666667	2,0,4,0	chr3,101521	Cytosol	A > G
chr3:101522726:A:G:intronic:NXPE	chr3	101522726	101522726	A	G	intronic	NXPE3	.	.	.	A	G	+	+	0.33333333	14,0,7,0	chr3,101522	Cytosol	A > G
chr3:101542350:A:G:UTR3:NXPE3	chr3	101542350	101542350	A	G	UTR3	NXPE3	ENST000002	.	.	A	G	+	+	0.71428571	4,0,10,0	chr3,101542	Cytosol	A > G
chr3:101542408:A:G:UTR3:NXPE3	chr3	101542408	101542408	A	G	UTR3	NXPE3	ENST000002	.	.	A	G	+	+	0.33333333	6,0,3,0	chr3,101542	Cytosol	A > G
chr3:101542436:A:G:UTR3:NXPE3	chr3	101542436	101542436	A	G	UTR3	NXPE3	ENST000002	.	.	A	G	+	+	0.23529412	13,0,4,0	chr3,101542	Cytosol	A > G
chr3:101542453:A:G:UTR3:NXPE3	chr3	101542453	101542453	A	G	UTR3	NXPE3	ENST000002	.	.	A	G	+	+	0.125	28,0,4,0	chr3,101542	Cytosol	A > G
chr3:101543436:A:G:ncRNA_intro	chr3	101543436	101543436	A	G	ncRNA_intro	RP11-49I4.3	.	.	.	A	G	+	+-	0.4	6,0,4,0	chr3,101543	Cytosol	A > G
chr3:101543453:A:G:ncRNA_intro	chr3	101543453	101543453	A	G	ncRNA_intro	RP11-49I4.3	.	.	.	A	G	+	+-	0.53846154	6,0,7,0	chr3,101543	Cytosol	A > G
chr3:101543508:A:G:ncRNA_intro	chr3	101543508	101543508	A	G	ncRNA_intro	RP11-49I4.3	.	.	.	A	G	+	+-	0.44	14,0,11,0	chr3,101543	Cytosol	A > G
chr3:101545622:A:G:UTR3:NXPE3	chr3	101545622	101545622	A	G	UTR3	NXPE3	ENST000004	.	.	A	G	+	+	0.25	6,0,2,0	chr3,101545	Cytosol	A > G
chr3:101545727:A:G:UTR3:NXPE3	chr3	101545727	101545727	A	G	UTR3	NXPE3	ENST000004	.	.	A	G	+	+	0.25	9,0,3,0	chr3,101545	Cytosol	A > G
chr3:101545797:A:G:UTR3:NXPE3	chr3	101545797	101545797	A	G	UTR3	NXPE3	ENST000004	.	.	A	G	+	+	0.13333333	13,0,2,0	chr3,101545	Cytosol	A > G
chr3:113433216:T:C:intergenic:KI	chr3	113433216	113433216	T	C	intergenic	KIAA2018,N	dist=17723;d	.	.	A	G	-		0.54545455	5,0,6,0	chr3,113433	Cytosol	A > G
chr3:113433252:T:C:intergenic:KI	chr3	113433252	113433252	T	C	intergenic	KIAA2018,N	dist=17759;d	.	.	A	G	-		0.2	12,0,3,0	chr3,113433	Cytosol	A > G
chr3:113433254:T:C:intergenic:KI	chr3	113433254	113433254	T	C	intergenic	KIAA2018,N	dist=17761;d	.	.	A	G	-		0.14285714	12,0,2,0	chr3,113433	Cytosol	A > G
chr3:119191725:A:G:intronic:POG	chr3	119191725	119191725	A	G	intronic	POGLUT1	.	.	.	A	G	+	+	0.6	2,0,3,0	chr3,119191	Cytosol	A > G
chr3:119191731:A:G:intronic:POG	chr3	119191731	119191731	A	G	intronic	POGLUT1	.	.	.	A	G	+	+	0.4	3,0,2,0	chr3,119191	Cytosol	A > G
chr3:120046873:T:C:UTR3:LRRC58	chr3	120046873	120046873	T	C	UTR3	LRRC58	ENST000002	.	.	A	G	-	-	0.02898551	134,0,4,0	chr3,120046	Cytosol	A > G
chr3:121552063:T:C:intronic:IQCB	chr3	121552063	121552063	T	C	intronic	IQCB1	.	.	.	A	G	-	-	0.71428571	2,0,5,0	chr3,121552	Cytosol	A > G
chr3:121552073:T:C:intronic:IQCB	chr3	121552073	121552073	T	C	intronic	IQCB1	.	.	.	A	G	-	-	0.57142857	3,0,4,0	chr3,121552	Cytosol	A > G
chr3:122290868:A:G:UTR3:DTX3L	chr3	122290868	122290868	A	G	UTR3	DTX3L	ENST000002	.	.	A	G	+	+	0.03125	62,0,2,0	chr3,122290	Cytosol	A > G
chr3:126174841:T:C:intronic:ZXDC	chr3	126174841	126174841	T	C	intronic	ZXDC	.	.	.	A	G	-	-	0.33333333	4,0,2,0	chr3,126174	Cytosol	A > G
chr3:126177217:T:C:intronic:ZXDC	chr3	126177217	126177217	T	C	intronic	ZXDC	.	.	.	A	G	-	-	0.6	2,0,3,0	chr3,126177	Cytosol	A > G
chr3:132397691:A:G:intronic:NPH	chr3	132397691	132397691	A	G	intronic	NPHP3	.	.	.	A	G	+	-+	0.8	1,0,4,0	chr3,132397	Cytosol	A > G
chr3:132397703:A:G:intronic:NPH	chr3	132397703	132397703	A	G	intronic	NPHP3	.	.	.	A	G	+	-+	0.66666667	2,0,4,0	chr3,132397	Cytosol	A > G
chr3:132397737:A:G:intronic:NPH	chr3	132397737	132397737	A	G	intronic	NPHP3	.	.	.	A	G	+	-+	0.83333333	1,0,5,0	chr3,132397	Cytosol	A > G
chr3:132397859:A:G:intronic:NPH	chr3	132397859	132397859	A	G	intronic	NPHP3	.	.	.	A	G	+	-+	0.88888889	1,0,8,0	chr3,132397	Cytosol	A > G
chr3:136050244:A:G:intronic:PCCE	chr3	136050244	136050244	A	G	intronic	PCCB	.	.	.	A	G	+	+	0.36666667	19,0,11,0	chr3,136050	Cytosol	A > G
chr3:136050375:A:G:intronic:PCCE	chr3	136050375	136050375	A	G	intronic	PCCB	.	.	.	A	G	+	+	0.75	4,0,12,0	chr3,136050	Cytosol	A > G

chr3:136050377:A:G:intronic:PCCB	chr3	136050377	136050377	A	G	intronic	PCCB	.	.	.	A	G	+	+	0.63636364	12,0,21,0	chr3,136050	Cytosol	A > G
chr3:136050686:A:G:intronic:PCCB	chr3	136050686	136050686	A	G	intronic	PCCB	.	.	.	A	G	+	+	0.58333333	5,0,7,0	chr3,136050	Cytosol	A > G
chr3:136050687:A:G:intronic:PCCB	chr3	136050687	136050687	A	G	intronic	PCCB	.	.	.	A	G	+	+	0.90909091	1,0,10,0	chr3,136050	Cytosol	A > G
chr3:136050820:A:G:intronic:PCCB	chr3	136050820	136050820	A	G	intronic	PCCB	.	.	.	A	G	+	+	0.83870968	5,0,26,0	chr3,136050	Cytosol	A > G
chr3:136050825:A:G:intronic:PCCB	chr3	136050825	136050825	A	G	intronic	PCCB	.	.	.	A	G	+	+	1	0,0,34,0	chr3,136050	Cytosol	A > G
chr3:136050873:A:G:intronic:PCCB	chr3	136050873	136050873	A	G	intronic	PCCB	.	.	.	A	G	+	+	0.06060606	62,0,4,0	chr3,136050	Cytosol	A > G
chr3:136050883:A:G:intronic:PCCB	chr3	136050883	136050883	A	G	intronic	PCCB	.	.	.	A	G	+	+	0.19402985	54,0,13,0	chr3,136050	Cytosol	A > G
chr3:136054551:A:G:UTR3:PCCB	chr3	136054551	136054551	A	G	UTR3	PCCB	ENST000004	.	.	A	G	+	+	0.85714286	1,0,6,0	chr3,136054	Cytosol	A > G
chr3:138218827:T:C:UTR3:CEP70	chr3	138218827	138218827	T	C	UTR3	CEP70	ENST000004	.	.	A	G	-	-	0.4	3,0,2,0	chr3,138218	Cytosol	A > G
chr3:138218833:T:C:UTR3:CEP70	chr3	138218833	138218833	T	C	UTR3	CEP70	ENST000004	.	.	A	G	-	-	0.33333333	6,0,3,0	chr3,138218	Cytosol	A > G
chr3:141664039:T:C:ncRNA_exoni	chr3	141664039	141664039	T	C	ncRNA_exon	RP11-271K21	.	.	.	A	G	-	-	0.6	4,0,6,0	chr3,141664	Cytosol	A > G
chr3:141664054:T:C:ncRNA_exoni	chr3	141664054	141664054	T	C	ncRNA_exon	RP11-271K21	.	.	.	A	G	-	-	0.5	3,0,3,0	chr3,141664	Cytosol	A > G
chr3:141664056:T:C:ncRNA_exoni	chr3	141664056	141664056	T	C	ncRNA_exon	RP11-271K21	.	.	.	A	G	-	-	0.33333333	4,0,2,0	chr3,141664	Cytosol	A > G
chr3:141664064:T:C:ncRNA_exoni	chr3	141664064	141664064	T	C	ncRNA_exon	RP11-271K21	.	.	.	A	G	-	-	0.57142857	3,0,4,0	chr3,141664	Cytosol	A > G
chr3:141664077:T:C:ncRNA_exoni	chr3	141664077	141664077	T	C	ncRNA_exon	RP11-271K21	.	.	.	A	G	-	-	0.375	5,0,3,0	chr3,141664	Cytosol	A > G
chr3:141664083:T:C:ncRNA_exoni	chr3	141664083	141664083	T	C	ncRNA_exon	RP11-271K21	.	.	.	A	G	-	-	0.5	5,0,5,0	chr3,141664	Cytosol	A > G
chr3:141664110:T:C:ncRNA_exoni	chr3	141664110	141664110	T	C	ncRNA_exon	RP11-271K21	.	.	.	A	G	-	-	0.28571429	5,0,2,0	chr3,141664	Cytosol	A > G
chr3:141665627:T:C:ncRNA_exoni	chr3	141665627	141665627	T	C	ncRNA_exon	RP11-271K21	.	.	.	A	G	-	-	0.375	5,0,3,0	chr3,141665	Cytosol	A > G
chr3:141665628:T:C:ncRNA_exoni	chr3	141665628	141665628	T	C	ncRNA_exon	RP11-271K21	.	.	.	A	G	-	-	0.375	5,0,3,0	chr3,141665	Cytosol	A > G
chr3:141665748:T:C:ncRNA_exoni	chr3	141665748	141665748	T	C	ncRNA_exon	RP11-271K21	.	.	.	A	G	-	-	1	0,0,5,0	chr3,141665	Cytosol	A > G
chr3:141666421:T:C:UTR3:TFDP2	chr3	141666421	141666421	T	C	UTR3	TFDP2	ENST000004	.	.	A	G	-	-	0.5	3,0,3,0	chr3,141666	Cytosol	A > G
chr3:141667236:T:C:UTR3:TFDP2	chr3	141667236	141667236	T	C	UTR3	TFDP2	ENST000004	.	.	A	G	-	-	0.4	3,0,2,0	chr3,141667	Cytosol	A > G
chr3:141667690:T:C:UTR3:TFDP2	chr3	141667690	141667690	T	C	UTR3	TFDP2	ENST000004	.	.	A	G	-	-	0.26666667	11,0,4,0	chr3,141667	Cytosol	A > G
chr3:141667757:T:C:UTR3:TFDP2	chr3	141667757	141667757	T	C	UTR3	TFDP2	ENST000004	.	.	A	G	-	-	0.8	1,0,4,0	chr3,141667	Cytosol	A > G
chr3:141668820:T:C:UTR3:TFDP2	chr3	141668820	141668820	T	C	UTR3	TFDP2	ENST000004	.	.	A	G	-	-	0.36363636	7,0,4,0	chr3,141668	Cytosol	A > G
chr3:141668831:T:C:UTR3:TFDP2	chr3	141668831	141668831	T	C	UTR3	TFDP2	ENST000004	.	.	A	G	-	-	0.22222222	7,0,2,0	chr3,141668	Cytosol	A > G
chr3:141668868:T:C:UTR3:TFDP2	chr3	141668868	141668868	T	C	UTR3	TFDP2	ENST000004	.	.	A	G	-	-	0.66666667	3,0,6,0	chr3,141668	Cytosol	A > G
chr3:141668876:T:C:UTR3:TFDP2	chr3	141668876	141668876	T	C	UTR3	TFDP2	ENST000004	.	.	A	G	-	-	0.33333333	4,0,2,0	chr3,141668	Cytosol	A > G
chr3:141668921:T:C:UTR3:TFDP2	chr3	141668921	141668921	T	C	UTR3	TFDP2	ENST000004	.	.	A	G	-	-	0.15	17,0,3,0	chr3,141668	Cytosol	A > G
chr3:141668922:T:C:UTR3:TFDP2	chr3	141668922	141668922	T	C	UTR3	TFDP2	ENST000004	.	.	A	G	-	-	0.61904762	8,0,13,0	chr3,141668	Cytosol	A > G
chr3:141668938:T:C:UTR3:TFDP2	chr3	141668938	141668938	T	C	UTR3	TFDP2	ENST000004	.	.	A	G	-	-	0.65	7,0,13,0	chr3,141668	Cytosol	A > G
chr3:141879040:T:C:ncRNA_exoni	chr3	141879040	141879040	T	C	ncRNA_exon	RP11-485G4.	.	.	.	A	G	-	-	0.11538462	23,0,3,0	chr3,141879	Cytosol	A > G
chr3:141879846:T:C:ncRNA_exoni	chr3	141879846	141879846	T	C	ncRNA_exon	RP11-485G4.	.	.	.	A	G	-	-	0.125	14,0,2,0	chr3,141879	Cytosol	A > G
chr3:141879856:T:C:ncRNA_exoni	chr3	141879856	141879856	T	C	ncRNA_exon	RP11-485G4.	.	.	.	A	G	-	-	0.16	21,0,4,0	chr3,141879	Cytosol	A > G
chr3:141880749:T:C:ncRNA_exoni	chr3	141880749	141880749	T	C	ncRNA_exon	RP11-485G4.	.	.	.	A	G	-	-	0.23529412	13,0,4,0	chr3,141880	Cytosol	A > G
chr3:141880766:T:C:ncRNA_exoni	chr3	141880766	141880766	T	C	ncRNA_exon	RP11-485G4.	.	.	.	A	G	-	-	0.27272727	8,0,3,0	chr3,141880	Cytosol	A > G
chr3:141880818:T:C:ncRNA_exoni	chr3	141880818	141880818	T	C	ncRNA_exon	RP11-485G4.	.	.	.	A	G	-	-	0.0952381	19,0,2,0	chr3,141880	Cytosol	A > G
chr3:141880921:T:C:upstream:RP1	chr3	141880921	141880921	T	C	upstream	RP11-485G4.	.	.	.	A	G	-	-	0.54545455	5,0,6,0	chr3,141880	Cytosol	A > G
chr3:141880931:T:C:upstream:RP1	chr3	141880931	141880931	T	C	upstream	RP11-485G4.	.	.	.	A	G	-	-	0.13333333	13,0,2,0	chr3,141880	Cytosol	A > G
chr3:141881589:T:C:upstream;dow	chr3	141881589	141881589	T	C	upstream;do	RP11-485G4.	.	.	.	A	G	-	-	1	0,0,10,0	chr3,141881	Cytosol	A > G
chr3:141881596:T:C:upstream;dow	chr3	141881596	141881596	T	C	upstream;do	RP11-485G4.	.	.	.	A	G	-	-	0.85714286	1,0,6,0	chr3,141881	Cytosol	A > G
chr3:141881614:T:C:upstream;dow	chr3	141881614	141881614	T	C	upstream;do	RP11-485G4.	.	.	.	A	G	-	-	0.66666667	2,0,4,0	chr3,141881	Cytosol	A > G
chr3:141881672:T:C:upstream;dow	chr3	141881672	141881672	T	C	upstream;do	RP11-485G4.	.	.	.	A	G	-	-	0.83333333	1,0,5,0	chr3,141881	Cytosol	A > G
chr3:141881681:T:C:upstream;dow	chr3	141881681	141881681	T	C	upstream;do	RP11-485G4.	.	.	.	A	G	-	-	0.6	2,0,3,0	chr3,141881	Cytosol	A > G
chr3:141881708:T:C:upstream;dow	chr3	141881708	141881708	T	C	upstream;do	RP11-485G4.	.	.	.	A	G	-	-	0.4	3,0,2,0	chr3,141881	Cytosol	A > G
chr3:141881738:T:C:upstream;dow	chr3	141881738	141881738	T	C	upstream;do	RP11-485G4.	.	.	.	A	G	-	-	0.625	3,0,5,0	chr3,141881	Cytosol	A > G
chr3:141881752:T:C:upstream;dow	chr3	141881752	141881752	T	C	upstream;do	RP11-485G4.	.	.	.	A	G	-	-	0.5	5,0,5,0	chr3,141881	Cytosol	A > G
chr3:141881755:T:C:upstream;dow	chr3	141881755	141881755	T	C	upstream;do	RP11-485G4.	.	.	.	A	G	-	-	0.77777778	2,0,7,0	chr3,141881	Cytosol	A > G
chr3:141881756:T:C:upstream;dow	chr3	141881756	141881756	T	C	upstream;do	RP11-485G4.	.	.	.	A	G	-	-	0.88888889	1,0,8,0	chr3,141881	Cytosol	A > G
chr3:141882184:T:C:downstream:	chr3	141882184	141882184	T	C	downstream	GK5	.	.	.	A	G	-	-	0.33333333	4,0,2,0	chr3,141882	Cytosol	A > G
chr3:141882185:T:C:downstream:	chr3	141882185	141882185	T	C	downstream	GK5	.	.	.	A	G	-	-	0.4	3,0,2,0	chr3,141882	Cytosol	A > G
chr3:141882199:T:C:downstream:	chr3	141882199	141882199	T	C	downstream	GK5	.	.	.	A	G	-	-	0.6	2,0,3,0	chr3,141882	Cytosol	A > G
chr3:141882262:T:C:downstream:	chr3	141882262	141882262	T	C	downstream	GK5	.	.	.	A	G	-	-	0.8	2,0,8,0	chr3,141882	Cytosol	A > G
chr3:141882295:T:C:downstream:	chr3	141882295	141882295	T	C	downstream	GK5	.	.	.	A	G	-	-	0.25	6,0,2,0	chr3,141882	Cytosol	A > G
chr3:141882296:T:C:downstream:	chr3	141882296	141882296	T	C	downstream	GK5	.	.	.	A	G	-	-	0.71428571	2,0,5,0	chr3,141882	Cytosol	A > G
chr3:141882310:T:C:downstream:	chr3	141882310	141882310	T	C	downstream	GK5	.	.	.	A	G	-	-	0.35714286	9,0,5,0	chr3,141882	Cytosol	A > G
chr3:141882330:T:C:downstream:	chr3	141882330	141882330	T	C	downstream	GK5	.	.	.	A	G	-	-	0.375	10,0,6,0	chr3,141882	Cytosol	A > G
chr3:141882379:T:C:downstream:	chr3	141882379	141882379	T	C	downstream	GK5	.	.	.	A	G	-	-	0.16666667	15,0,3,0	chr3,141882	Cytosol	A > G
chr3:141882620:T:C:UTR3:GK5	chr3	141882620	141882620	T	C	UTR3	GK5	ENST000004	.	.	A	G	-	-	0.22222222	7,0,2,0	chr3,141882	Cytosol	A > G
chr3:141882646:T:C:UTR3:GK5	chr3	141882646	141882646	T	C	UTR3	GK5	ENST000004	.	.	A	G	-	-	0.3	7,0,3,0	chr3,141882	Cytosol	A > G
chr3:141882672:T:C:intronic:GK5	chr3	141882672	141882672	T	C	intronic	GK5	.	.	.	A	G	-	-	0.80952381	4,0,17,0	chr3,141882	Cytosol	A > G
chr3:141903562:T:C:UTR3:GK5	chr3	141903562	141903562	T	C	UTR3	GK5	ENST000004	.	.	A	G	-	-	1	0,0,6,0	chr3,141903	Cytosol	A > G
chr3:141903566:T:C:UTR3:GK5	chr3	141903566	141903566	T	C	UTR3	GK5	ENST000004	.	.	A	G	-	-	0.33333333	4,0,2,0	chr3,141903	Cytosol	A > G
chr3:155659901:A:G:UTR3:GMPS	chr3	155659901	155659901	A	G	UTR3	GMPS	ENST000004	.	.	A	G	+	+	0.71428571	4,0,10,0	chr3,155659	Cytosol	A > G

chr3:155659937:A:G:UTR3:GMPS	chr3	155659937	155659937	A	G	UTR3	GMPS	ENST000004	.	.	A	G	+	+	0.36363636	7,0,4,0	chr3,155659	Cytosol	A > G
chr3:155659956:A:G:UTR3:GMPS	chr3	155659956	155659956	A	G	UTR3	GMPS	ENST000004	.	.	A	G	+	+	0.63636364	4,0,7,0	chr3,155659	Cytosol	A > G
chr3:155660005:A:G:UTR3:GMPS	chr3	155660005	155660005	A	G	UTR3	GMPS	ENST000004	.	.	A	G	+	+	1	0,0,6,0	chr3,155660	Cytosol	A > G
chr3:155660015:A:G:UTR3:GMPS	chr3	155660015	155660015	A	G	UTR3	GMPS	ENST000004	.	.	A	G	+	+	0.71428571	2,0,5,0	chr3,155660	Cytosol	A > G
chr3:155660765:A:G:UTR3:GMPS	chr3	155660765	155660765	A	G	UTR3	GMPS	ENST000004	.	.	A	G	+	+	0.75	9,0,27,0	chr3,155660	Cytosol	A > G
chr3:155660810:A:G:UTR3:GMPS	chr3	155660810	155660810	A	G	UTR3	GMPS	ENST000004	.	.	A	G	+	+	0.88888889	2,0,16,0	chr3,155660	Cytosol	A > G
chr3:156258205:T:C:UTR3:SSR3	chr3	156258205	156258205	T	C	UTR3	SSR3	ENST000002	.	.	A	G	-	-	0.46929825	121,0,107,0	chr3,156258	Cytosol	A > G
chr3:156258273:T:C:UTR3:SSR3	chr3	156258273	156258273	T	C	UTR3	SSR3	ENST000002	.	.	A	G	-	-	0.93951613	15,0,233,0	chr3,156258	Cytosol	A > G
chr3:156258283:T:C:UTR3:SSR3	chr3	156258283	156258283	T	C	UTR3	SSR3	ENST000002	.	.	A	G	-	-	0.79130435	47,0,182,1	chr3,156258	Cytosol	A > G
chr3:156258295:T:C:UTR3:SSR3	chr3	156258295	156258295	T	C	UTR3	SSR3	ENST000002	.	.	A	G	-	-	0.28019324	149,0,58,0	chr3,156258	Cytosol	A > G
chr3:156258308:T:C:UTR3:SSR3	chr3	156258308	156258308	T	C	UTR3	SSR3	ENST000002	.	.	A	G	-	-	0.4159292	132,0,94,0	chr3,156258	Cytosol	A > G
chr3:156258389:T:C:UTR3:SSR3	chr3	156258389	156258389	T	C	UTR3	SSR3	ENST000002	.	.	A	G	-	-	0.46666667	8,0,7,0	chr3,156258	Cytosol	A > G
chr3:156258393:T:C:UTR3:SSR3	chr3	156258393	156258393	T	C	UTR3	SSR3	ENST000002	.	.	A	G	-	-	0.73333333	4,0,11,0	chr3,156258	Cytosol	A > G
chr3:156259229:T:C:UTR3:SSR3	chr3	156259229	156259229	T	C	UTR3	SSR3	ENST000002	.	.	A	G	-	-	0.09694794	503,0,54,0	chr3,156259	Cytosol	A > G
chr3:156259288:T:C:UTR3:SSR3	chr3	156259288	156259288	T	C	UTR3	SSR3	ENST000002	.	.	A	G	-	-	0.59530026	155,0,228,0	chr3,156259	Cytosol	A > G
chr3:156259295:T:C:UTR3:SSR3	chr3	156259295	156259295	T	C	UTR3	SSR3	ENST000002	.	.	A	G	-	-	0.544	169,0,204,2	chr3,156259	Cytosol	A > G
chr3:156259299:T:C:UTR3:SSR3	chr3	156259299	156259299	T	C	UTR3	SSR3	ENST000002	.	.	A	G	-	-	0.05698006	331,0,20,0	chr3,156259	Cytosol	A > G
chr3:156259317:T:C:UTR3:SSR3	chr3	156259317	156259317	T	C	UTR3	SSR3	ENST000002	.	.	A	G	-	-	0.25541126	172,0,59,0	chr3,156259	Cytosol	A > G
chr3:156259324:T:C:UTR3:SSR3	chr3	156259324	156259324	T	C	UTR3	SSR3	ENST000002	.	.	A	G	-	-	0.08056872	194,0,17,0	chr3,156259	Cytosol	A > G
chr3:156259340:T:C:UTR3:SSR3	chr3	156259340	156259340	T	C	UTR3	SSR3	ENST000002	.	.	A	G	-	-	0.77837838	41,0,144,0	chr3,156259	Cytosol	A > G
chr3:156259341:T:C:UTR3:SSR3	chr3	156259341	156259341	T	C	UTR3	SSR3	ENST000002	.	.	A	G	-	-	0.52406417	89,0,98,0	chr3,156259	Cytosol	A > G
chr3:156259363:T:C:UTR3:SSR3	chr3	156259363	156259363	T	C	UTR3	SSR3	ENST000002	.	.	A	G	-	-	0.4389313	147,0,115,0	chr3,156259	Cytosol	A > G
chr3:156259367:T:C:UTR3:SSR3	chr3	156259367	156259367	T	C	UTR3	SSR3	ENST000002	.	.	A	G	-	-	0.3814433	180,0,111,0	chr3,156259	Cytosol	A > G
chr3:156259376:T:C:UTR3:SSR3	chr3	156259376	156259376	T	C	UTR3	SSR3	ENST000002	.	.	A	G	-	-	0.04237288	338,0,15,1	chr3,156259	Cytosol	A > G
chr3:156259396:T:C:UTR3:SSR3	chr3	156259396	156259396	T	C	UTR3	SSR3	ENST000002	.	.	A	G	-	-	0.03274559	384,0,13,0	chr3,156259	Cytosol	A > G
chr3:156259406:T:C:UTR3:SSR3	chr3	156259406	156259406	T	C	UTR3	SSR3	ENST000002	.	.	A	G	-	-	0.67561983	157,0,327,0	chr3,156259	Cytosol	A > G
chr3:160154941:A:G:UTR3:TRIM5	chr3	160154941	160154941	A	G	UTR3	TRIM59	ENST000003	.	.	A	G	+	-	0.28571429	5,0,2,0	chr3,160154	Cytosol	A > G
chr3:160154955:T:C:UTR3:TRIM59	chr3	160154955	160154955	T	C	UTR3	TRIM59	ENST000003	.	.	A	G	-	-	0.0952381	19,0,2,0	chr3,160154	Cytosol	A > G
chr3:169806736:T:C:ncRNA_exoni	chr3	169806736	169806736	T	C	ncRNA_exon	RP11-379K17	.	.	.	A	G	-	-+	0.09090909	20,0,2,0	chr3,169806	Cytosol	A > G
chr3:169806879:T:C:ncRNA_exoni	chr3	169806879	169806879	T	C	ncRNA_exon	RP11-379K17	.	.	.	A	G	-	-+	0.04	48,0,2,0	chr3,169806	Cytosol	A > G
chr3:169807623:T:C:UTR3:PHC3	chr3	169807623	169807623	T	C	UTR3	PHC3	ENST000004	.	.	A	G	-	-	0.08	23,0,2,0	chr3,169807	Cytosol	A > G
chr3:179042910:A:G:UTR5:ZNF63	chr3	179042910	179042910	A	G	UTR5	ZNF639	ENST000004	.	.	A	G	+	+	0.12307692	57,0,8,0	chr3,179042	Cytosol	A > G
chr3:179042949:A:G:UTR5:ZNF63	chr3	179042949	179042949	A	G	UTR5	ZNF639	ENST000004	.	.	A	G	+	+	0.07142857	26,0,2,0	chr3,179042	Cytosol	A > G
chr3:179043023:A:G:UTR5:ZNF63	chr3	179043023	179043023	A	G	UTR5	ZNF639	ENST000004	.	.	A	G	+	+	0.06060606	62,0,4,0	chr3,179043	Cytosol	A > G
chr3:179055180:A:G:upstream:RP	chr3	179055180	179055180	A	G	upstream	RP11-255C1	.	.	.	A	G	+		0.55555556	4,0,5,0	chr3,179055	Cytosol	A > G
chr3:179111820:A:G:UTR3:MFN1	chr3	179111820	179111820	A	G	UTR3	MFN1	ENST000004	.	.	A	G	+	+	0.0375	77,0,3,0	chr3,179111	Cytosol	A > G
chr3:179115621:T:C:ncRNA_exoni	chr3	179115621	179115621	T	C	ncRNA_exon	AC007620.3,	.	.	.	A	G	-	-+	0.16086957	193,0,37,0	chr3,179115	Cytosol	A > G
chr3:179115627:T:C:ncRNA_exoni	chr3	179115627	179115627	T	C	ncRNA_exon	AC007620.3,	.	.	.	A	G	-	-+	0.95081967	9,0,174,0	chr3,179115	Cytosol	A > G
chr3:179115630:T:C:ncRNA_exoni	chr3	179115630	179115630	T	C	ncRNA_exon	AC007620.3,	.	.	.	A	G	-	-+	0.44642857	93,0,75,0	chr3,179115	Cytosol	A > G
chr3:179115698:T:C:ncRNA_exoni	chr3	179115698	179115698	T	C	ncRNA_exon	AC007620.3,	.	.	.	A	G	-	-+	0.73195876	26,0,71,0	chr3,179115	Cytosol	A > G
chr3:179115701:T:C:ncRNA_exoni	chr3	179115701	179115701	T	C	ncRNA_exon	AC007620.3,	.	.	.	A	G	-	-+	0.81730769	19,0,85,0	chr3,179115	Cytosol	A > G
chr3:179116285:T:C:ncRNA_exoni	chr3	179116285	179116285	T	C	ncRNA_exon	AC007620.3	.	.	.	A	G	-	-+	0.94805195	4,0,73,0	chr3,179116	Cytosol	A > G
chr3:179117033:T:C:UTR3:GNB4	chr3	179117033	179117033	T	C	UTR3	GNB4	ENST000002	.	.	A	G	-	-	0.35833333	77,0,43,0	chr3,179117	Cytosol	A > G
chr3:179117128:T:C:UTR3:GNB4	chr3	179117128	179117128	T	C	UTR3	GNB4	ENST000002	.	.	A	G	-	-	0.60526316	15,0,23,0	chr3,179117	Cytosol	A > G
chr3:179117168:T:C:UTR3:GNB4	chr3	179117168	179117168	T	C	UTR3	GNB4	ENST000002	.	.	A	G	-	-	0.04615385	62,0,3,0	chr3,179117	Cytosol	A > G
chr3:179117233:T:C:UTR3:GNB4	chr3	179117233	179117233	T	C	UTR3	GNB4	ENST000002	.	.	A	G	-	-	0.26984127	46,0,17,0	chr3,179117	Cytosol	A > G
chr3:179117241:T:C:UTR3:GNB4	chr3	179117241	179117241	T	C	UTR3	GNB4	ENST000002	.	.	A	G	-	-	0.09876543	73,0,8,0	chr3,179117	Cytosol	A > G
chr3:179117242:T:C:UTR3:GNB4	chr3	179117242	179117242	T	C	UTR3	GNB4	ENST000002	.	.	A	G	-	-	0.37349398	52,0,31,0	chr3,179117	Cytosol	A > G
chr3:179117264:T:C:UTR3:GNB4	chr3	179117264	179117264	T	C	UTR3	GNB4	ENST000002	.	.	A	G	-	-	0.11111111	80,0,10,0	chr3,179117	Cytosol	A > G
chr3:179117266:T:C:UTR3:GNB4	chr3	179117266	179117266	T	C	UTR3	GNB4	ENST000002	.	.	A	G	-	-	0.19318182	71,0,17,0	chr3,179117	Cytosol	A > G
chr3:183912931:A:G:intronic:EIF2	chr3	183912931	183912931	A	G	intronic	EIF2B5	.	.	.	A	G	+	+	0.5	3,0,3,0	chr3,183912	Cytosol	A > G
chr3:183915256:A:G:intronic:EIF2	chr3	183915256	183915256	A	G	intronic	EIF2B5	.	.	.	A	G	+	+	0.8	1,0,4,0	chr3,183915	Cytosol	A > G
chr3:185208781:C:T:UTR3:TMEM4	chr3	185208781	185208781	C	T	UTR3	TMEM41A	ENST000004	.	.	G	A	-	-	0.02439024	2,0,80,0	chr3,185208	Cytosol	G > A
chr3:188600755:A:G:UTR3:LPP	chr3	188600755	188600755	A	G	UTR3	LPP	ENST000003	.	.	A	G	+	+	0.5	3,0,3,0	chr3,188600	Cytosol	A > G
chr3:194309846:A:G:ncRNA_exoni	chr3	194309846	194309846	A	G	ncRNA_exon	TMEM44-AS	.	.	.	A	G	+	+-	0.22222222	7,0,2,0	chr3,194309	Cytosol	A > G
chr3:196079280:T:C:UTR3:UBXN7	chr3	196079280	196079280	T	C	UTR3	UBXN7	ENST000002	.	.	A	G	-	-	0.08333333	22,0,2,0	chr3,196079	Cytosol	A > G
chr3:196079284:T:C:UTR3:UBXN7	chr3	196079284	196079284	T	C	UTR3	UBXN7	ENST000002	.	.	A	G	-	-	0.12	22,0,3,0	chr3,196079	Cytosol	A > G
chr3:196079289:T:C:UTR3:UBXN7	chr3	196079289	196079289	T	C	UTR3	UBXN7	ENST000002	.	.	A	G	-	-	0.16	21,0,4,0	chr3,196079	Cytosol	A > G
chr3:196079293:T:C:UTR3:UBXN7	chr3	196079293	196079293	T	C	UTR3	UBXN7	ENST000002	.	.	A	G	-	-	0.28571429	15,0,6,0	chr3,196079	Cytosol	A > G
chr3:196079305:T:C:UTR3:UBXN7	chr3	196079305	196079305	T	C	UTR3	UBXN7	ENST000002	.	.	A	G	-	-	0.33333333	8,0,4,0	chr3,196079	Cytosol	A > G
chr3:196079390:T:C:UTR3:UBXN7	chr3	196079390	196079390	T	C	UTR3	UBXN7	ENST000002	.	.	A	G	-	-	0.14285714	12,0,2,0	chr3,196079	Cytosol	A > G
chr3:196079412:T:C:UTR3:UBXN7	chr3	196079412	196079412	T	C	UTR3	UBXN7	ENST000002	.	.	A	G	-	-	0.16666667	10,0,2,0	chr3,196079	Cytosol	A > G
chr3:196079608:T:C:UTR3:UBXN7	chr3	196079608	196079608	T	C	UTR3	UBXN7	ENST000002	.	.	A	G	-	-	0.10526316	17,0,2,0	chr3,196079	Cytosol	A > G
chr3:196079620:T:C:UTR3:UBXN7	chr3	196079620	196079620	T	C	UTR3	UBXN7	ENST000002	.	.	A	G	-	-	0.53333333	7,0,8,0	chr3,196079	Cytosol	A > G

chr3:196079691:T:C:UTR3:UBXN7	chr3	196079691	196079691	T	C	UTR3	UBXN7	ENST000002	.	.	A	G	-	-	0.625	3,0,5,0	chr3,196079	Cytosol	A > G
chr3:196079697:T:C:UTR3:UBXN7	chr3	196079697	196079697	T	C	UTR3	UBXN7	ENST000002	.	.	A	G	-	-	0.2	12,0,3,0	chr3,196079	Cytosol	A > G
chr3:196080531:T:C:UTR3:UBXN7	chr3	196080531	196080531	T	C	UTR3	UBXN7	ENST000002	.	.	A	G	-	-	0.15384615	11,0,2,0	chr3,196080	Cytosol	A > G
chr3:196080553:T:C:UTR3:UBXN7	chr3	196080553	196080553	T	C	UTR3	UBXN7	ENST000002	.	.	A	G	-	-	0.15384615	11,0,2,0	chr3,196080	Cytosol	A > G
chr3:196080600:T:C:UTR3:UBXN7	chr3	196080600	196080600	T	C	UTR3	UBXN7	ENST000002	.	.	A	G	-	-	0.22222222	7,0,2,0	chr3,196080	Cytosol	A > G
chr3:196461234:A:G:UTR3:PIGX	chr3	196461234	196461234	A	G	UTR3	PIGX	ENST000003	.	.	A	G	+	+	0.04166667	46,0,2,0	chr3,196461	Cytosol	A > G
chr3:197339583:T:C:ncRNA_intror	chr3	197339583	197339583	T	C	ncRNA_intro	AC024560.3	.	.	.	A	G	-	-	0.66666667	9,0,18,0	chr3,197339	Cytosol	A > G
chr3:197339633:T:C:ncRNA_intror	chr3	197339633	197339633	T	C	ncRNA_intro	AC024560.3	.	.	.	A	G	-	-	0.91666667	2,0,22,0	chr3,197339	Cytosol	A > G
chr3:197339634:T:C:ncRNA_intror	chr3	197339634	197339634	T	C	ncRNA_intro	AC024560.3	.	.	.	A	G	-	-	0.2	20,0,5,0	chr3,197339	Cytosol	A > G
chr3:197339658:T:C:ncRNA_intror	chr3	197339658	197339658	T	C	ncRNA_intro	AC024560.3	.	.	.	A	G	-	-	0.5	13,0,13,0	chr3,197339	Cytosol	A > G
chr3:197339888:T:C:ncRNA_intror	chr3	197339888	197339888	T	C	ncRNA_intro	AC024560.3	.	.	.	A	G	-	-	0.47368421	10,0,9,0	chr3,197339	Cytosol	A > G
chr3:197339891:T:C:ncRNA_intror	chr3	197339891	197339891	T	C	ncRNA_intro	AC024560.3	.	.	.	A	G	-	-	0.08695652	21,0,2,0	chr3,197339	Cytosol	A > G
chr3:197339992:T:C:ncRNA_intror	chr3	197339992	197339992	T	C	ncRNA_intro	AC024560.3	.	.	.	A	G	-	-	0.57142857	3,0,4,0	chr3,197339	Cytosol	A > G
chr3:197611048:A:G:UTR3:LRCH3	chr3	197611048	197611048	A	G	UTR3	LRCH3	ENST000004	.	.	A	G	+	+	0.33333333	8,0,4,0	chr3,197611	Cytosol	A > G
chr3:197611192:A:G:UTR3:LRCH3	chr3	197611192	197611192	A	G	UTR3	LRCH3	ENST000004	.	.	A	G	+	+	0.83333333	1,0,5,0	chr3,197611	Cytosol	A > G
chr3:197611262:A:G:UTR3:LRCH3	chr3	197611262	197611262	A	G	UTR3	LRCH3	ENST000004	.	.	A	G	+	+	0.23076923	10,0,3,0	chr3,197611	Cytosol	A > G
chr3:197611263:A:G:UTR3:LRCH3	chr3	197611263	197611263	A	G	UTR3	LRCH3	ENST000004	.	.	A	G	+	+	0.64285714	5,0,9,0	chr3,197611	Cytosol	A > G
chr3:197611289:A:G:UTR3:LRCH3	chr3	197611289	197611289	A	G	UTR3	LRCH3	ENST000004	.	.	A	G	+	+	0.11764706	15,0,2,0	chr3,197611	Cytosol	A > G
chr3:197611666:A:G:UTR3:LRCH3	chr3	197611666	197611666	A	G	UTR3	LRCH3	ENST000004	.	.	A	G	+	+	0.0625	30,0,2,0	chr3,197611	Cytosol	A > G
chr3:197611667:A:G:UTR3:LRCH3	chr3	197611667	197611667	A	G	UTR3	LRCH3	ENST000004	.	.	A	G	+	+	0.05714286	33,0,2,0	chr3,197611	Cytosol	A > G
chr3:197611745:A:G:UTR3:LRCH3	chr3	197611745	197611745	A	G	UTR3	LRCH3	ENST000004	.	.	A	G	+	+	0.06896552	27,0,2,0	chr3,197611	Cytosol	A > G
chr3:197611746:A:G:UTR3:LRCH3	chr3	197611746	197611746	A	G	UTR3	LRCH3	ENST000004	.	.	A	G	+	+	0.14285714	24,0,4,0	chr3,197611	Cytosol	A > G
chr3:197611758:A:G:UTR3:LRCH3	chr3	197611758	197611758	A	G	UTR3	LRCH3	ENST000004	.	.	A	G	+	+	0.0625	30,0,2,0	chr3,197611	Cytosol	A > G
chr3:197612226:A:G:UTR3:LRCH3	chr3	197612226	197612226	A	G	UTR3	LRCH3	ENST000004	.	.	A	G	+	+	0.22222222	14,0,4,0	chr3,197612	Cytosol	A > G
chr3:197612267:A:G:UTR3:LRCH3	chr3	197612267	197612267	A	G	UTR3	LRCH3	ENST000004	.	.	A	G	+	+	0.5	7,0,7,0	chr3,197612	Cytosol	A > G
chr3:197612268:A:G:UTR3:LRCH3	chr3	197612268	197612268	A	G	UTR3	LRCH3	ENST000004	.	.	A	G	+	+	0.30769231	9,0,4,0	chr3,197612	Cytosol	A > G
chr3:197612360:A:G:UTR3:LRCH3	chr3	197612360	197612360	A	G	UTR3	LRCH3	ENST000004	.	.	A	G	+	+	0.33333333	4,0,2,0	chr3,197612	Cytosol	A > G
chr3:197612361:A:G:UTR3:LRCH3	chr3	197612361	197612361	A	G	UTR3	LRCH3	ENST000004	.	.	A	G	+	+	0.28571429	5,0,2,0	chr3,197612	Cytosol	A > G
chr3:197612370:A:G:UTR3:LRCH3	chr3	197612370	197612370	A	G	UTR3	LRCH3	ENST000004	.	.	A	G	+	+	0.44444444	5,0,4,0	chr3,197612	Cytosol	A > G
chr3:197612393:A:G:UTR3:LRCH3	chr3	197612393	197612393	A	G	UTR3	LRCH3	ENST000004	.	.	A	G	+	+	0.57142857	3,0,4,0	chr3,197612	Cytosol	A > G
chr3:197612402:A:G:UTR3:LRCH3	chr3	197612402	197612402	A	G	UTR3	LRCH3	ENST000004	.	.	A	G	+	+	0.5	3,0,3,0	chr3,197612	Cytosol	A > G
chr4:126849:A:G:ncRNA_exonic:Zl	chr4	126849	126849	A	G	ncRNA_exon	ZNF718	.	.	.	A	G	+	+	0.6	2,0,3,0	chr4,126848,	Cytosol	A > G
chr4:491473:T:C:intronic:ZNF721	chr4	491473	491473	T	C	intronic	ZNF721	.	.	.	A	G	-	-	0.5	3,0,3,0	chr4,491472,	Cytosol	A > G
chr4:491488:T:C:intronic:ZNF721	chr4	491488	491488	T	C	intronic	ZNF721	.	.	.	A	G	-	-	0.8	1,0,4,0	chr4,491487,	Cytosol	A > G
chr4:771586:T:C:ncRNA_intronic:R	chr4	771586	771586	T	C	ncRNA_intro	RP11-440L14	.	.	.	A	G	-	-	0.28571429	5,0,2,0	chr4,771585,	Cytosol	A > G
chr4:771590:T:C:ncRNA_intronic:R	chr4	771590	771590	T	C	ncRNA_intro	RP11-440L14	.	.	.	A	G	-	-	0.44444444	5,0,4,0	chr4,771589,	Cytosol	A > G
chr4:774074:T:C:ncRNA_exonic:RF	chr4	774074	774074	T	C	ncRNA_exon	RP11-440L14	.	.	.	A	G	-	-	0.4	6,0,4,0	chr4,774073,	Cytosol	A > G
chr4:774098:T:C:ncRNA_exonic:RF	chr4	774098	774098	T	C	ncRNA_exon	RP11-440L14	.	.	.	A	G	-	-	0.2	8,0,2,0	chr4,774097,	Cytosol	A > G
chr4:774115:T:C:ncRNA_exonic:RF	chr4	774115	774115	T	C	ncRNA_exon	RP11-440L14	.	.	.	A	G	-	-	0.38461538	8,0,5,0	chr4,774114,	Cytosol	A > G
chr4:774138:T:C:ncRNA_exonic:RF	chr4	774138	774138	T	C	ncRNA_exon	RP11-440L14	.	.	.	A	G	-	-	0.44444444	5,0,4,0	chr4,774137,	Cytosol	A > G
chr4:774147:T:C:ncRNA_exonic:RF	chr4	774147	774147	T	C	ncRNA_exon	RP11-440L14	.	.	.	A	G	-	-	0.2	8,0,2,0	chr4,774146,	Cytosol	A > G
chr4:774253:T:C:ncRNA_exonic:RF	chr4	774253	774253	T	C	ncRNA_exon	RP11-440L14	.	.	.	A	G	-	-	0.25	6,0,2,0	chr4,774252,	Cytosol	A > G
chr4:1383237:A:G:intergenic:UVSS	chr4	1383237	1383237	A	G	intergenic	UVSSA,CRIPA	dist=1400;dis	.	.	A	G	+		0.23529412	13,0,4,0	chr4,138323	Cytosol	A > G
chr4:1387263:A:G:UTR5:CRIPAK	chr4	1387263	1387263	A	G	UTR5	CRIPAK	ENST000003	.	.	A	G	+	+	0.25	6,0,2,0	chr4,138726	Cytosol	A > G
chr4:1387264:A:G:UTR5:CRIPAK	chr4	1387264	1387264	A	G	UTR5	CRIPAK	ENST000003	.	.	A	G	+	+	0.2	8,0,2,0	chr4,138726	Cytosol	A > G
chr4:2231939:T:C:UTR3:HAUS3	chr4	2231939	2231939	T	C	UTR3	HAUS3	ENST000002	.	.	A	G	-	-	0.28571429	5,0,2,0	chr4,223193	Cytosol	A > G
chr4:2841320:A:G:UTR3:SH3BP2	chr4	2841320	2841320	A	G	UTR3	SH3BP2	ENST000004	.	.	A	G	+	+	0.58333333	5,0,7,0	chr4,284131	Cytosol	A > G
chr4:2841352:A:G:UTR3:SH3BP2	chr4	2841352	2841352	A	G	UTR3	SH3BP2	ENST000004	.	.	A	G	+	+	0.8	3,0,12,0	chr4,284135	Cytosol	A > G
chr4:2841359:A:G:UTR3:SH3BP2	chr4	2841359	2841359	A	G	UTR3	SH3BP2	ENST000004	.	.	A	G	+	+	0.14285714	12,0,2,0	chr4,284135	Cytosol	A > G
chr4:2841360:A:G:UTR3:SH3BP2	chr4	2841360	2841360	A	G	UTR3	SH3BP2	ENST000004	.	.	A	G	+	+	0.46153846	7,0,6,0	chr4,284135	Cytosol	A > G
chr4:2841388:A:G:UTR3:SH3BP2	chr4	2841388	2841388	A	G	UTR3	SH3BP2	ENST000004	.	.	A	G	+	+	0.6	6,0,9,0	chr4,284138	Cytosol	A > G
chr4:2841396:A:G:UTR3:SH3BP2	chr4	2841396	2841396	A	G	UTR3	SH3BP2	ENST000004	.	.	A	G	+	+	0.71428571	4,0,10,0	chr4,284139	Cytosol	A > G
chr4:2841421:A:G:UTR3:SH3BP2	chr4	2841421	2841421	A	G	UTR3	SH3BP2	ENST000004	.	.	A	G	+	+	0.25	12,0,4,0	chr4,284142	Cytosol	A > G
chr4:2841424:A:G:UTR3:SH3BP2	chr4	2841424	2841424	A	G	UTR3	SH3BP2	ENST000004	.	.	A	G	+	+	0.4375	9,0,7,0	chr4,284142	Cytosol	A > G
chr4:2841434:A:G:UTR3:SH3BP2	chr4	2841434	2841434	A	G	UTR3	SH3BP2	ENST000004	.	.	A	G	+	+	0.33333333	8,0,4,0	chr4,284143	Cytosol	A > G
chr4:2841456:A:G:UTR3:SH3BP2	chr4	2841456	2841456	A	G	UTR3	SH3BP2	ENST000004	.	.	A	G	+	+	0.25	6,0,2,0	chr4,284145	Cytosol	A > G
chr4:2841552:A:G:UTR3:SH3BP2	chr4	2841552	2841552	A	G	UTR3	SH3BP2	ENST000004	.	.	A	G	+	+	0.28571429	10,0,4,0	chr4,284155	Cytosol	A > G
chr4:2841691:A:G:UTR3:SH3BP2	chr4	2841691	2841691	A	G	UTR3	SH3BP2	ENST000004	.	.	A	G	+	+	0.23076923	10,0,3,0	chr4,284169	Cytosol	A > G
chr4:2841799:A:G:UTR3:SH3BP2	chr4	2841799	2841799	A	G	UTR3	SH3BP2	ENST000004	.	.	A	G	+	+	0.13333333	13,0,2,0	chr4,284179	Cytosol	A > G
chr4:2841801:A:G:UTR3:SH3BP2	chr4	2841801	2841801	A	G	UTR3	SH3BP2	ENST000004	.	.	A	G	+	+	0.2	12,0,3,0	chr4,284180	Cytosol	A > G
chr4:2841805:A:G:UTR3:SH3BP2	chr4	2841805	2841805	A	G	UTR3	SH3BP2	ENST000004	.	.	A	G	+	+	0.45454545	6,0,5,0	chr4,284180	Cytosol	A > G
chr4:2841831:A:G:UTR3:SH3BP2	chr4	2841831	2841831	A	G	UTR3	SH3BP2	ENST000004	.	.	A	G	+	+	0.63636364	4,0,7,0	chr4,284183	Cytosol	A > G
chr4:2841837:A:G:UTR3:SH3BP2	chr4	2841837	2841837	A	G	UTR3	SH3BP2	ENST000004	.	.	A	G	+	+	0.72727273	3,0,8,0	chr4,284183	Cytosol	A > G
chr4:2841841:A:G:UTR3:SH3BP2	chr4	2841841	2841841	A	G	UTR3	SH3BP2	ENST000004	.	.	A	G	+	+	0.64285714	5,0,9,0	chr4,284184	Cytosol	A > G

chr4:2841845:A:G:UTR3:SH3BP2	chr4	2841845	2841845	A	G	UTR3	SH3BP2	ENST000004	.	.	A	G	+	+	1	0,0,13,0	chr4,284184	Cytosol	A > G
chr4:2841893:A:G:UTR3:SH3BP2	chr4	2841893	2841893	A	G	UTR3	SH3BP2	ENST000004	.	.	A	G	+	+	0.25	12,0,4,0	chr4,284189	Cytosol	A > G
chr4:2940026:T:C:exonic:NOP14	chr4	2940026	2940026	T	C	exonic	NOP14	.	nonsynonym	NOP14:ENST	A	G	-	+-	0.30769231	27,0,12,0	chr4,294002	Cytosol	A > G
chr4:2940085:T:C:ncRNA_intronic:	chr4	2940085	2940085	T	C	ncRNA_intro	NOP14-AS1	.	.	.	A	G	-	+-	0.6741573	58,0,120,0	chr4,294008	Cytosol	A > G
chr4:2940098:T:C:ncRNA_intronic:	chr4	2940098	2940098	T	C	ncRNA_intro	NOP14-AS1	.	.	.	A	G	-	+-	0.18817204	151,0,35,0	chr4,294009	Cytosol	A > G
chr4:2940125:T:C:ncRNA_intronic:	chr4	2940125	2940125	T	C	ncRNA_intro	NOP14-AS1	.	.	.	A	G	-	+-	0.14090909	189,0,31,0	chr4,294012	Cytosol	A > G
chr4:2940265:T:C:ncRNA_intronic:	chr4	2940265	2940265	T	C	ncRNA_intro	NOP14-AS1	.	.	.	A	G	-	+-	0.19130435	93,0,22,0	chr4,294026	Cytosol	A > G
chr4:2940347:T:C:ncRNA_intronic:	chr4	2940347	2940347	T	C	ncRNA_intro	NOP14-AS1	.	.	.	A	G	-	+-	0.20394737	121,0,31,0	chr4,294034	Cytosol	A > G
chr4:2940371:T:C:ncRNA_intronic:	chr4	2940371	2940371	T	C	ncRNA_intro	NOP14-AS1	.	.	.	A	G	-	+-	0.91160221	16,0,165,0	chr4,294037	Cytosol	A > G
chr4:2940397:T:C:ncRNA_intronic:	chr4	2940397	2940397	T	C	ncRNA_intro	NOP14-AS1	.	.	.	A	G	-	+-	0.71604938	69,0,174,0	chr4,294039	Cytosol	A > G
chr4:2940413:T:C:ncRNA_intronic:	chr4	2940413	2940413	T	C	ncRNA_intro	NOP14-AS1	.	.	.	A	G	-	+-	0.08118081	249,0,22,0	chr4,294041	Cytosol	A > G
chr4:2940414:T:C:ncRNA_intronic:	chr4	2940414	2940414	T	C	ncRNA_intro	NOP14-AS1	.	.	.	A	G	-	+-	0.09363296	242,0,25,0	chr4,294041	Cytosol	A > G
chr4:2940430:T:C:ncRNA_exonic:N	chr4	2940430	2940430	T	C	ncRNA_exon	NOP14-AS1	.	.	.	A	G	-	+-	0.05990783	204,0,13,0	chr4,294042	Cytosol	A > G
chr4:2940434:T:C:ncRNA_exonic:N	chr4	2940434	2940434	T	C	ncRNA_exon	NOP14-AS1	.	.	.	A	G	-	+-	0.03864734	199,0,8,0	chr4,294043	Cytosol	A > G
chr4:2940462:T:C:ncRNA_exonic:N	chr4	2940462	2940462	T	C	ncRNA_exon	NOP14-AS1	.	.	.	A	G	-	+-	0.46296296	29,0,25,0	chr4,294046	Cytosol	A > G
chr4:2940485:T:C:ncRNA_exonic:N	chr4	2940485	2940485	T	C	ncRNA_exon	NOP14-AS1	.	.	.	A	G	-	+-	0.10810811	66,0,8,0	chr4,294048	Cytosol	A > G
chr4:3509795:T:C:UTR3:LRPAP1	chr4	3509795	3509795	T	C	UTR3	LRPAP1	ENST000005	.	.	A	G	-	-	0.33333333	6,0,3,0	chr4,350979	Cytosol	A > G
chr4:3509829:T:C:UTR3:LRPAP1	chr4	3509829	3509829	T	C	UTR3	LRPAP1	ENST000005	.	.	A	G	-	-	0.57142857	3,0,4,0	chr4,350982	Cytosol	A > G
chr4:3509835:T:C:UTR3:LRPAP1	chr4	3509835	3509835	T	C	UTR3	LRPAP1	ENST000005	.	.	A	G	-	-	0.875	1,0,7,0	chr4,350983	Cytosol	A > G
chr4:3509844:T:C:UTR3:LRPAP1	chr4	3509844	3509844	T	C	UTR3	LRPAP1	ENST000005	.	.	A	G	-	-	0.33333333	4,0,2,0	chr4,350984	Cytosol	A > G
chr4:17627717:A:G:UTR3:MED28	chr4	17627717	17627717	A	G	UTR3	MED28	ENST000002	.	.	A	G	+	+	0.4	9,0,6,0	chr4,176277	Cytosol	A > G
chr4:17628548:A:G:UTR3:MED28	chr4	17628548	17628548	A	G	UTR3	MED28	ENST000002	.	.	A	G	+	+	0.38888889	11,0,7,0	chr4,176285	Cytosol	A > G
chr4:17628551:A:G:UTR3:MED28	chr4	17628551	17628551	A	G	UTR3	MED28	ENST000002	.	.	A	G	+	+	0.44444444	10,0,8,0	chr4,176285	Cytosol	A > G
chr4:17628635:A:G:UTR3:MED28	chr4	17628635	17628635	A	G	UTR3	MED28	ENST000002	.	.	A	G	+	+	0.36363636	7,0,4,0	chr4,176286	Cytosol	A > G
chr4:17628661:A:G:UTR3:MED28	chr4	17628661	17628661	A	G	UTR3	MED28	ENST000002	.	.	A	G	+	+	0.13333333	13,0,2,0	chr4,176286	Cytosol	A > G
chr4:17628667:A:G:UTR3:MED28	chr4	17628667	17628667	A	G	UTR3	MED28	ENST000002	.	.	A	G	+	+	0.17647059	14,0,3,0	chr4,176286	Cytosol	A > G
chr4:17628691:A:G:UTR3:MED28	chr4	17628691	17628691	A	G	UTR3	MED28	ENST000002	.	.	A	G	+	+	0.30769231	9,0,4,0	chr4,176286	Cytosol	A > G
chr4:17628699:A:G:UTR3:MED28	chr4	17628699	17628699	A	G	UTR3	MED28	ENST000002	.	.	A	G	+	+	0.25	9,0,3,0	chr4,176286	Cytosol	A > G
chr4:17628978:A:G:UTR3:MED28	chr4	17628978	17628978	A	G	UTR3	MED28	ENST000002	.	.	A	G	+	+	0.61538462	5,0,8,0	chr4,176289	Cytosol	A > G
chr4:17629372:A:G:UTR3:MED28	chr4	17629372	17629372	A	G	UTR3	MED28	ENST000002	.	.	A	G	+	+	0.46153846	7,0,6,0	chr4,176293	Cytosol	A > G
chr4:17629377:A:G:UTR3:MED28	chr4	17629377	17629377	A	G	UTR3	MED28	ENST000002	.	.	A	G	+	+	0.91666667	1,0,11,0	chr4,176293	Cytosol	A > G
chr4:17629434:A:G:UTR3:MED28	chr4	17629434	17629434	A	G	UTR3	MED28	ENST000002	.	.	A	G	+	+	0.27777778	13,0,5,0	chr4,176294	Cytosol	A > G
chr4:17629440:A:G:UTR3:MED28	chr4	17629440	17629440	A	G	UTR3	MED28	ENST000002	.	.	A	G	+	+	0.08	23,0,2,0	chr4,176294	Cytosol	A > G
chr4:17630685:A:G:UTR3:MED28	chr4	17630685	17630685	A	G	UTR3	MED28	ENST000002	.	.	A	G	+	+	0.27272727	8,0,3,0	chr4,176306	Cytosol	A > G
chr4:17630699:A:G:UTR3:MED28	chr4	17630699	17630699	A	G	UTR3	MED28	ENST000002	.	.	A	G	+	+	0.38461538	8,0,5,0	chr4,176306	Cytosol	A > G
chr4:17630735:A:G:UTR3:MED28	chr4	17630735	17630735	A	G	UTR3	MED28	ENST000002	.	.	A	G	+	+	0.33333333	10,0,5,0	chr4,176307	Cytosol	A > G
chr4:17630762:A:G:UTR3:MED28	chr4	17630762	17630762	A	G	UTR3	MED28	ENST000002	.	.	A	G	+	+	0.46153846	7,0,6,0	chr4,176307	Cytosol	A > G
chr4:17630776:A:G:UTR3:MED28	chr4	17630776	17630776	A	G	UTR3	MED28	ENST000002	.	.	A	G	+	+	0.4	6,0,4,0	chr4,176307	Cytosol	A > G
chr4:17802783:T:C:UTR3:DCAF16	chr4	17802783	17802783	T	C	UTR3	DCAF16	ENST000003	.	.	A	G	-	-	0.56	22,0,28,0	chr4,178027	Cytosol	A > G
chr4:17802819:T:C:UTR3:DCAF16	chr4	17802819	17802819	T	C	UTR3	DCAF16	ENST000003	.	.	A	G	-	-	0.61403509	22,0,35,0	chr4,178028	Cytosol	A > G
chr4:17802824:T:C:UTR3:DCAF16	chr4	17802824	17802824	T	C	UTR3	DCAF16	ENST000003	.	.	A	G	-	-	0.42	29,0,21,0	chr4,178028	Cytosol	A > G
chr4:17802827:T:C:UTR3:DCAF16	chr4	17802827	17802827	T	C	UTR3	DCAF16	ENST000003	.	.	A	G	-	-	0.80357143	11,0,45,0	chr4,178028	Cytosol	A > G
chr4:17802867:T:C:UTR3:DCAF16	chr4	17802867	17802867	T	C	UTR3	DCAF16	ENST000003	.	.	A	G	-	-	0.25641026	29,0,10,0	chr4,178028	Cytosol	A > G
chr4:17802889:T:C:UTR3:DCAF16	chr4	17802889	17802889	T	C	UTR3	DCAF16	ENST000003	.	.	A	G	-	-	0.57142857	15,0,20,0	chr4,178028	Cytosol	A > G
chr4:17802890:T:C:UTR3:DCAF16	chr4	17802890	17802890	T	C	UTR3	DCAF16	ENST000003	.	.	A	G	-	-	0.34285714	23,0,12,0	chr4,178028	Cytosol	A > G
chr4:17802930:T:C:UTR3:DCAF16	chr4	17802930	17802930	T	C	UTR3	DCAF16	ENST000003	.	.	A	G	-	-	0.4	12,0,8,0	chr4,178029	Cytosol	A > G
chr4:17802931:T:C:UTR3:DCAF16	chr4	17802931	17802931	T	C	UTR3	DCAF16	ENST000003	.	.	A	G	-	-	0.15789474	16,0,3,0	chr4,178029	Cytosol	A > G
chr4:17802934:T:C:UTR3:DCAF16	chr4	17802934	17802934	T	C	UTR3	DCAF16	ENST000003	.	.	A	G	-	-	0.18181818	18,0,4,0	chr4,178029	Cytosol	A > G
chr4:17802940:T:C:UTR3:DCAF16	chr4	17802940	17802940	T	C	UTR3	DCAF16	ENST000003	.	.	A	G	-	-	0.25	18,0,6,0	chr4,178029	Cytosol	A > G
chr4:17802945:T:C:UTR3:DCAF16	chr4	17802945	17802945	T	C	UTR3	DCAF16	ENST000003	.	.	A	G	-	-	0.09677419	28,0,3,0	chr4,178029	Cytosol	A > G
chr4:17802956:T:C:UTR3:DCAF16	chr4	17802956	17802956	T	C	UTR3	DCAF16	ENST000003	.	.	A	G	-	-	0.15686275	43,0,8,0	chr4,178029	Cytosol	A > G
chr4:17802976:T:C:UTR3:DCAF16	chr4	17802976	17802976	T	C	UTR3	DCAF16	ENST000003	.	.	A	G	-	-	0.13333333	52,0,8,0	chr4,178029	Cytosol	A > G
chr4:17802979:T:C:UTR3:DCAF16	chr4	17802979	17802979	T	C	UTR3	DCAF16	ENST000003	.	.	A	G	-	-	0.49152542	30,0,29,0	chr4,178029	Cytosol	A > G
chr4:17802982:T:C:UTR3:DCAF16	chr4	17802982	17802982	T	C	UTR3	DCAF16	ENST000003	.	.	A	G	-	-	0.03703704	52,0,2,0	chr4,178029	Cytosol	A > G
chr4:17803009:T:C:UTR3:DCAF16	chr4	17803009	17803009	T	C	UTR3	DCAF16	ENST000003	.	.	A	G	-	-	0.16049383	68,0,13,0	chr4,178030	Cytosol	A > G
chr4:17803019:T:C:UTR3:DCAF16	chr4	17803019	17803019	T	C	UTR3	DCAF16	ENST000003	.	.	A	G	-	-	0.79012346	17,0,64,0	chr4,178030	Cytosol	A > G
chr4:17803537:T:C:UTR3:DCAF16	chr4	17803537	17803537	T	C	UTR3	DCAF16	ENST000003	.	.	A	G	-	-	0.94545455	3,0,52,0	chr4,178035	Cytosol	A > G
chr4:17803553:T:C:UTR3:DCAF16	chr4	17803553	17803553	T	C	UTR3	DCAF16	ENST000003	.	.	A	G	-	-	0.08510638	43,0,4,0	chr4,178035	Cytosol	A > G
chr4:17803559:T:C:UTR3:DCAF16	chr4	17803559	17803559	T	C	UTR3	DCAF16	ENST000003	.	.	A	G	-	-	0.85365854	6,0,35,0	chr4,178035	Cytosol	A > G
chr4:17803562:T:C:UTR3:DCAF16	chr4	17803562	17803562	T	C	UTR3	DCAF16	ENST000003	.	.	A	G	-	-	0.5106383	23,0,24,0	chr4,178035	Cytosol	A > G
chr4:17803604:T:C:UTR3:DCAF16	chr4	17803604	17803604	T	C	UTR3	DCAF16	ENST000003	.	.	A	G	-	-	0.3559322	38,0,21,0	chr4,178036	Cytosol	A > G
chr4:17803614:T:C:UTR3:DCAF16	chr4	17803614	17803614	T	C	UTR3	DCAF16	ENST000003	.	.	A	G	-	-	0.21311475	48,0,13,0	chr4,178036	Cytosol	A > G
chr4:17803662:T:C:UTR3:DCAF16	chr4	17803662	17803662	T	C	UTR3	DCAF16	ENST000003	.	.	A	G	-	-	0.5	30,0,30,0	chr4,178036	Cytosol	A > G
chr4:17803663:T:C:UTR3:DCAF16	chr4	17803663	17803663	T	C	UTR3	DCAF16	ENST000003	.	.	A	G	-	-	0.03703704	52,0,2,0	chr4,178036	Cytosol	A > G

chr4:17803673:T:C:UTR3:DCAF16	chr4	17803673	17803673	T	C	UTR3	DCAF16	ENST000003	.	.	A	G	-	-	0.30434783	32,0,14,0	chr4,178036	Cytosol	A > G
chr4:17803681:T:C:UTR3:DCAF16	chr4	17803681	17803681	T	C	UTR3	DCAF16	ENST000003	.	.	A	G	-	-	0.38461538	24,0,15,0	chr4,178036	Cytosol	A > G
chr4:17804710:T:C:UTR3:DCAF16	chr4	17804710	17804710	T	C	UTR3	DCAF16	ENST000003	.	.	A	G	-	-	0.08474576	54,0,5,0	chr4,178047	Cytosol	A > G
chr4:17804735:T:C:UTR3:DCAF16	chr4	17804735	17804735	T	C	UTR3	DCAF16	ENST000003	.	.	A	G	-	-	0.03703704	52,0,2,0	chr4,178047	Cytosol	A > G
chr4:17804740:T:C:UTR3:DCAF16	chr4	17804740	17804740	T	C	UTR3	DCAF16	ENST000003	.	.	A	G	-	-	0.15	51,0,9,0	chr4,178047	Cytosol	A > G
chr4:17804785:T:C:UTR3:DCAF16	chr4	17804785	17804785	T	C	UTR3	DCAF16	ENST000003	.	.	A	G	-	-	0.25	39,0,13,0	chr4,178047	Cytosol	A > G
chr4:17804799:T:C:UTR3:DCAF16	chr4	17804799	17804799	T	C	UTR3	DCAF16	ENST000003	.	.	A	G	-	-	0.06382979	44,0,3,0	chr4,178047	Cytosol	A > G
chr4:17804847:T:C:UTR3:DCAF16	chr4	17804847	17804847	T	C	UTR3	DCAF16	ENST000003	.	.	A	G	-	-	0.29166667	51,0,21,0	chr4,178048	Cytosol	A > G
chr4:17804851:T:C:UTR3:DCAF16	chr4	17804851	17804851	T	C	UTR3	DCAF16	ENST000003	.	.	A	G	-	-	0.13513514	64,0,10,0	chr4,178048	Cytosol	A > G
chr4:17804852:T:C:UTR3:DCAF16	chr4	17804852	17804852	T	C	UTR3	DCAF16	ENST000003	.	.	A	G	-	-	0.17105263	63,0,13,0	chr4,178048	Cytosol	A > G
chr4:17804872:T:C:UTR3:DCAF16	chr4	17804872	17804872	T	C	UTR3	DCAF16	ENST000003	.	.	A	G	-	-	0.07142857	65,0,5,0	chr4,178048	Cytosol	A > G
chr4:25280872:A:G:downstream:P	chr4	25280872	25280872	A	G	downstream	PI4K2B	.	.	.	A	G	+		0.55555556	4,0,5,0	chr4,252808	Cytosol	A > G
chr4:25280957:A:G:downstream:P	chr4	25280957	25280957	A	G	downstream	PI4K2B	.	.	.	A	G	+		0.88461538	3,0,23,0	chr4,252809	Cytosol	A > G
chr4:25281423:A:G:downstream:P	chr4	25281423	25281423	A	G	downstream	PI4K2B	.	.	.	A	G	+		0.57142857	6,0,8,0	chr4,252814	Cytosol	A > G
chr4:25281445:A:G:downstream:P	chr4	25281445	25281445	A	G	downstream	PI4K2B	.	.	.	A	G	+		0.53846154	6,0,7,0	chr4,252814	Cytosol	A > G
chr4:25281459:A:G:downstream:P	chr4	25281459	25281459	A	G	downstream	PI4K2B	.	.	.	A	G	+		0.1875	13,0,3,0	chr4,252814	Cytosol	A > G
chr4:25281460:A:G:downstream:P	chr4	25281460	25281460	A	G	downstream	PI4K2B	.	.	.	A	G	+		0.33333333	10,0,5,0	chr4,252814	Cytosol	A > G
chr4:25281527:A:G:downstream:P	chr4	25281527	25281527	A	G	downstream	PI4K2B	.	.	.	A	G	+		0.35	13,0,7,0	chr4,252815	Cytosol	A > G
chr4:37866273:A:G:intergenic:PGM	chr4	37866273	37866273	A	G	intergenic	PGM2,TBC1D	dist=1715;dis	.	.	A	G	+		0.6	2,0,3,0	chr4,378662	Cytosol	A > G
chr4:39534647:A:G:ncRNA_intronic	chr4	39534647	39534647	A	G	ncRNA_intro	UGDH-AS1	.	.	.	A	G	+	+	0.5	3,0,3,0	chr4,395346	Cytosol	A > G
chr4:39536407:A:G:ncRNA_intronic	chr4	39536407	39536407	A	G	ncRNA_intro	UGDH-AS1	.	.	.	A	G	+	+	0.33333333	6,0,3,0	chr4,395364	Cytosol	A > G
chr4:39536462:A:G:ncRNA_intronic	chr4	39536462	39536462	A	G	ncRNA_intro	UGDH-AS1	.	.	.	A	G	+	+	0.2	8,0,2,0	chr4,395364	Cytosol	A > G
chr4:39536475:A:G:ncRNA_intronic	chr4	39536475	39536475	A	G	ncRNA_intro	UGDH-AS1	.	.	.	A	G	+	+	0.42857143	4,0,3,0	chr4,395364	Cytosol	A > G
chr4:39536485:A:G:ncRNA_intronic	chr4	39536485	39536485	A	G	ncRNA_intro	UGDH-AS1	.	.	.	A	G	+	+	0.4	3,0,2,0	chr4,395364	Cytosol	A > G
chr4:39536490:A:G:ncRNA_intronic	chr4	39536490	39536490	A	G	ncRNA_intro	UGDH-AS1	.	.	.	A	G	+	+	0.4	3,0,2,0	chr4,395364	Cytosol	A > G
chr4:39550558:T:C:ncRNA_intronic	chr4	39550558	39550558	T	C	ncRNA_intro	UGDH-AS1	.	.	.	A	G	-	+-	0.53333333	7,0,8,0	chr4,395505	Cytosol	A > G
chr4:39550564:T:C:ncRNA_intronic	chr4	39550564	39550564	T	C	ncRNA_intro	UGDH-AS1	.	.	.	A	G	-	+-	0.75	3,0,9,0	chr4,395505	Cytosol	A > G
chr4:39550570:T:C:ncRNA_intronic	chr4	39550570	39550570	T	C	ncRNA_intro	UGDH-AS1	.	.	.	A	G	-	+-	0.66666667	3,0,6,0	chr4,395505	Cytosol	A > G
chr4:39550612:T:C:ncRNA_intronic	chr4	39550612	39550612	T	C	ncRNA_intro	UGDH-AS1	.	.	.	A	G	-	+-	0.72727273	3,0,8,0	chr4,395506	Cytosol	A > G
chr4:39550623:T:C:ncRNA_intronic	chr4	39550623	39550623	T	C	ncRNA_intro	UGDH-AS1	.	.	.	A	G	-	+-	0.36363636	7,0,4,0	chr4,395506	Cytosol	A > G
chr4:39550671:T:C:ncRNA_intronic	chr4	39550671	39550671	T	C	ncRNA_intro	UGDH-AS1	.	.	.	A	G	-	+-	0.66666667	4,0,8,0	chr4,395506	Cytosol	A > G
chr4:39550757:T:C:ncRNA_intronic	chr4	39550757	39550757	T	C	ncRNA_intro	UGDH-AS1	.	.	.	A	G	-	+-	0.12903226	27,0,4,0	chr4,395507	Cytosol	A > G
chr4:39550978:T:C:ncRNA_intronic	chr4	39550978	39550978	T	C	ncRNA_intro	UGDH-AS1	.	.	.	A	G	-	+-	0.68421053	6,0,13,0	chr4,395509	Cytosol	A > G
chr4:39551008:T:C:ncRNA_intronic	chr4	39551008	39551008	T	C	ncRNA_intro	UGDH-AS1	.	.	.	A	G	-	+-	0.4375	18,0,14,0	chr4,395510	Cytosol	A > G
chr4:39551018:T:C:ncRNA_intronic	chr4	39551018	39551018	T	C	ncRNA_intro	UGDH-AS1	.	.	.	A	G	-	+-	0.13157895	33,0,5,0	chr4,395510	Cytosol	A > G
chr4:39551140:T:C:ncRNA_intronic	chr4	39551140	39551140	T	C	ncRNA_intro	UGDH-AS1	.	.	.	A	G	-	+-	0.18181818	18,0,4,0	chr4,395511	Cytosol	A > G
chr4:39551167:T:C:ncRNA_intronic	chr4	39551167	39551167	T	C	ncRNA_intro	UGDH-AS1	.	.	.	A	G	-	+-	0.26086957	17,0,6,0	chr4,395511	Cytosol	A > G
chr4:39551179:T:C:ncRNA_intronic	chr4	39551179	39551179	T	C	ncRNA_intro	UGDH-AS1	.	.	.	A	G	-	+-	0.67857143	9,0,19,0	chr4,395511	Cytosol	A > G
chr4:39552022:T:C:ncRNA_intronic	chr4	39552022	39552022	T	C	ncRNA_intro	UGDH-AS1	.	.	.	A	G	-	+-	0.05714286	33,0,2,0	chr4,395520	Cytosol	A > G
chr4:39552062:T:C:ncRNA_intronic	chr4	39552062	39552062	T	C	ncRNA_intro	UGDH-AS1	.	.	.	A	G	-	+-	0.10526316	17,0,2,0	chr4,395520	Cytosol	A > G
chr4:39552066:T:C:ncRNA_intronic	chr4	39552066	39552066	T	C	ncRNA_intro	UGDH-AS1	.	.	.	A	G	-	+-	0.21052632	15,0,4,0	chr4,395520	Cytosol	A > G
chr4:39552183:T:C:ncRNA_intronic	chr4	39552183	39552183	T	C	ncRNA_intro	UGDH-AS1	.	.	.	A	G	-	+-	0.2	16,0,4,0	chr4,395521	Cytosol	A > G
chr4:42100231:A:G:intergenic:SLC	chr4	42100231	42100231	A	G	intergenic	SLC30A9,BEN	dist=7757;dis	.	.	A	G	+		0.25	6,0,2,0	chr4,421002	Cytosol	A > G
chr4:56236837:A:G:ncRNA_intronic	chr4	56236837	56236837	A	G	ncRNA_intro	SRD5A3-AS1	.	.	.	A	G	+	+-	0.55555556	8,0,10,0	chr4,562368	Cytosol	A > G
chr4:57326078:A:G:UTR3:PAICS	chr4	57326078	57326078	A	G	UTR3	PAICS	ENST000005	.	.	A	G	+	+	0.08888889	328,0,32,0	chr4,573260	Cytosol	A > G
chr4:57326082:A:G:UTR3:PAICS	chr4	57326082	57326082	A	G	UTR3	PAICS	ENST000005	.	.	A	G	+	+	0.23972603	222,0,70,0	chr4,573260	Cytosol	A > G
chr4:57326121:A:G:UTR3:PAICS	chr4	57326121	57326121	A	G	UTR3	PAICS	ENST000005	.	.	A	G	+	+	0.03076923	567,0,18,0	chr4,573261	Cytosol	A > G
chr4:57326126:A:G:UTR3:PAICS	chr4	57326126	57326126	A	G	UTR3	PAICS	ENST000005	.	.	A	G	+	+	0.22404372	568,0,164,0	chr4,573261	Cytosol	A > G
chr4:57326131:A:G:UTR3:PAICS	chr4	57326131	57326131	A	G	UTR3	PAICS	ENST000005	.	.	A	G	+	+	0.08365508	712,0,65,0	chr4,573261	Cytosol	A > G
chr4:57326155:A:G:UTR3:PAICS	chr4	57326155	57326155	A	G	UTR3	PAICS	ENST000005	.	.	A	G	+	+	0.01212121	978,0,12,0	chr4,573261	Cytosol	A > G
chr4:57326156:A:G:UTR3:PAICS	chr4	57326156	57326156	A	G	UTR3	PAICS	ENST000005	.	.	A	G	+	+	0.03072349	978,0,31,0	chr4,573261	Cytosol	A > G
chr4:57326159:A:G:UTR3:PAICS	chr4	57326159	57326159	A	G	UTR3	PAICS	ENST000005	.	.	A	G	+	+	0.08613861	923,0,87,0	chr4,573261	Cytosol	A > G
chr4:57326186:A:G:UTR3:PAICS	chr4	57326186	57326186	A	G	UTR3	PAICS	ENST000005	.	.	A	G	+	+	0.01682692	818,0,14,0	chr4,573261	Cytosol	A > G
chr4:57326250:A:G:UTR3:PAICS	chr4	57326250	57326250	A	G	UTR3	PAICS	ENST000005	.	.	A	G	+	+	0.01175318	1009,0,12,0	chr4,573262	Cytosol	A > G
chr4:57326262:A:G:UTR3:PAICS	chr4	57326262	57326262	A	G	UTR3	PAICS	ENST000005	.	.	A	G	+	+	0.0383536	1028,0,41,0	chr4,573262	Cytosol	A > G
chr4:57326291:A:G:UTR3:PAICS	chr4	57326291	57326291	A	G	UTR3	PAICS	ENST000005	.	.	A	G	+	+	0.4490532	610,1,498,0	chr4,573262	Cytosol	A > G
chr4:57326292:A:G:UTR3:PAICS	chr4	57326292	57326292	A	G	UTR3	PAICS	ENST000005	.	.	A	G	+	+	0.01459854	1080,0,16,0	chr4,573262	Cytosol	A > G
chr4:57326313:A:G:UTR3:PAICS	chr4	57326313	57326313	A	G	UTR3	PAICS	ENST000005	.	.	A	G	+	+	0.07401177	1100,1,88,0	chr4,573263	Cytosol	A > G
chr4:57326315:A:G:UTR3:PAICS	chr4	57326315	57326315	A	G	UTR3	PAICS	ENST000005	.	.	A	G	+	+	0.10009268	971,0,108,0	chr4,573263	Cytosol	A > G
chr4:57326321:A:G:UTR3:PAICS	chr4	57326321	57326321	A	G	UTR3	PAICS	ENST000005	.	.	A	G	+	+	0.03743842	977,0,38,0	chr4,573263	Cytosol	A > G
chr4:57326329:A:G:UTR3:PAICS	chr4	57326329	57326329	A	G	UTR3	PAICS	ENST000005	.	.	A	G	+	+	0.02112029	1066,0,23,0	chr4,573263	Cytosol	A > G
chr4:57326333:A:G:UTR3:PAICS	chr4	57326333	57326333	A	G	UTR3	PAICS	ENST000005	.	.	A	G	+	+	0.04583723	1020,0,49,0	chr4,573263	Cytosol	A > G
chr4:57326873:A:G:UTR3:PAICS	chr4	57326873	57326873	A	G	UTR3	PAICS	ENST000005	.	.	A	G	+	+	0.01034929	765,0,8,0	chr4,573268	Cytosol	A > G
chr4:57326875:A:G:UTR3:PAICS	chr4	57326875	57326875	A	G	UTR3	PAICS	ENST000005	.	.	A	G	+	+	0.11832061	693,0,93,0	chr4,573268	Cytosol	A > G

chr4:57326879:A:G:UTR3:PAICS	chr4	57326879	57326879	A	G	UTR3	PAICS	ENST000005	.	.	A	G	+	+	0.08633094	762,0,72,0	chr4,573268	Cytosol	A > G
chr4:57326917:A:G:UTR3:PAICS	chr4	57326917	57326917	A	G	UTR3	PAICS	ENST000005	.	.	A	G	+	+	0.01458333	473,0,7,0	chr4,573269	Cytosol	A > G
chr4:57326960:A:G:UTR3:PAICS	chr4	57326960	57326960	A	G	UTR3	PAICS	ENST000005	.	.	A	G	+	+	0.0254372	613,0,16,0	chr4,573269	Cytosol	A > G
chr4:57327017:A:G:UTR3:PAICS	chr4	57327017	57327017	A	G	UTR3	PAICS	ENST000005	.	.	A	G	+	+	0.10169492	530,0,60,0	chr4,573270	Cytosol	A > G
chr4:57327022:A:G:UTR3:PAICS	chr4	57327022	57327022	A	G	UTR3	PAICS	ENST000005	.	.	A	G	+	+	0.02893309	537,0,16,0	chr4,573270	Cytosol	A > G
chr4:57327049:A:G:UTR3:PAICS	chr4	57327049	57327049	A	G	UTR3	PAICS	ENST000005	.	.	A	G	+	+	0.01265823	312,0,4,0	chr4,573270	Cytosol	A > G
chr4:57327057:A:G:UTR3:PAICS	chr4	57327057	57327057	A	G	UTR3	PAICS	ENST000005	.	.	A	G	+	+	0.03584229	269,0,10,0	chr4,573270	Cytosol	A > G
chr4:57327058:A:G:UTR3:PAICS	chr4	57327058	57327058	A	G	UTR3	PAICS	ENST000005	.	.	A	G	+	+	0.3089172	217,0,97,0	chr4,573270	Cytosol	A > G
chr4:57327078:A:G:UTR3:PAICS	chr4	57327078	57327078	A	G	UTR3	PAICS	ENST000005	.	.	A	G	+	+	0.08653846	475,0,45,0	chr4,573270	Cytosol	A > G
chr4:57327082:A:G:UTR3:PAICS	chr4	57327082	57327082	A	G	UTR3	PAICS	ENST000005	.	.	A	G	+	+	0.29072165	344,0,141,0	chr4,573270	Cytosol	A > G
chr4:57327087:A:G:UTR3:PAICS	chr4	57327087	57327087	A	G	UTR3	PAICS	ENST000005	.	.	A	G	+	+	0.8812616	64,0,475,0	chr4,573270	Cytosol	A > G
chr4:57327092:A:G:UTR3:PAICS	chr4	57327092	57327092	A	G	UTR3	PAICS	ENST000005	.	.	A	G	+	+	0.05799649	536,0,33,0	chr4,573270	Cytosol	A > G
chr4:57327097:A:G:UTR3:PAICS	chr4	57327097	57327097	A	G	UTR3	PAICS	ENST000005	.	.	A	G	+	+	0.06034483	654,0,42,0	chr4,573270	Cytosol	A > G
chr4:57327108:A:G:UTR3:PAICS	chr4	57327108	57327108	A	G	UTR3	PAICS	ENST000005	.	.	A	G	+	+	0.02173913	765,0,17,0	chr4,573271	Cytosol	A > G
chr4:57327116:A:G:UTR3:PAICS	chr4	57327116	57327116	A	G	UTR3	PAICS	ENST000005	.	.	A	G	+	+	0.0369515	834,0,32,0	chr4,573271	Cytosol	A > G
chr4:57327125:A:G:UTR3:PAICS	chr4	57327125	57327125	A	G	UTR3	PAICS	ENST000005	.	.	A	G	+	+	0.01557286	885,0,14,0	chr4,573271	Cytosol	A > G
chr4:57976234:T:C:exonic;exonic:I	chr4	57976234	57976234	T	C	exonic;exoni	IGFBP7	.	nonsynonym	IGFBP7:ENST	A	G	-	-+	0.1674277	547,0,110,0	chr4,579762	Cytosol	A > G
chr4:57976234:T:C:exonic;exonic:I	chr4	57976234	57976234	T	C	exonic;exoni	IGFBP7	.	nonsynonym	IGFBP7:ENST	T	C	+	-+	0.5	0,4,0,4	chr4,579762	Cytosol	T > C
chr4:71681967:T:C:UTR3:GRSF1	chr4	71681967	71681967	T	C	UTR3	GRSF1	ENST000002	.	.	A	G	-	-	0.18181818	27,0,6,0	chr4,716819	Cytosol	A > G
chr4:71681984:T:C:UTR3:GRSF1	chr4	71681984	71681984	T	C	UTR3	GRSF1	ENST000002	.	.	A	G	-	-	0.125	21,0,3,0	chr4,716819	Cytosol	A > G
chr4:71681998:T:C:UTR3:GRSF1	chr4	71681998	71681998	T	C	UTR3	GRSF1	ENST000002	.	.	A	G	-	-	0.44827586	16,0,13,0	chr4,716819	Cytosol	A > G
chr4:71682004:T:C:UTR3:GRSF1	chr4	71682004	71682004	T	C	UTR3	GRSF1	ENST000002	.	.	A	G	-	-	0.08	23,0,2,0	chr4,716820	Cytosol	A > G
chr4:71682010:T:C:UTR3:GRSF1	chr4	71682010	71682010	T	C	UTR3	GRSF1	ENST000002	.	.	A	G	-	-	0.44827586	16,0,13,0	chr4,716820	Cytosol	A > G
chr4:71682036:T:C:UTR3:GRSF1	chr4	71682036	71682036	T	C	UTR3	GRSF1	ENST000002	.	.	A	G	-	-	0.4375	9,0,7,0	chr4,716820	Cytosol	A > G
chr4:71682275:T:C:UTR3:GRSF1	chr4	71682275	71682275	T	C	UTR3	GRSF1	ENST000002	.	.	A	G	-	-	0.42105263	11,0,8,0	chr4,716822	Cytosol	A > G
chr4:71682379:T:C:UTR3:GRSF1	chr4	71682379	71682379	T	C	UTR3	GRSF1	ENST000002	.	.	A	G	-	-	0.41558442	45,0,32,0	chr4,716823	Cytosol	A > G
chr4:71682381:T:C:UTR3:GRSF1	chr4	71682381	71682381	T	C	UTR3	GRSF1	ENST000002	.	.	A	G	-	-	0.64473684	27,0,49,0	chr4,716823	Cytosol	A > G
chr4:71683041:T:C:UTR3:GRSF1	chr4	71683041	71683041	T	C	UTR3	GRSF1	ENST000002	.	.	A	G	-	-	0.26666667	33,0,12,0	chr4,716830	Cytosol	A > G
chr4:71683087:T:C:UTR3:GRSF1	chr4	71683087	71683087	T	C	UTR3	GRSF1	ENST000002	.	.	A	G	-	-	0.14893617	40,0,7,0	chr4,716830	Cytosol	A > G
chr4:71683841:T:C:UTR3:GRSF1	chr4	71683841	71683841	T	C	UTR3	GRSF1	ENST000002	.	.	A	G	-	-	0.62162162	14,0,23,0	chr4,716838	Cytosol	A > G
chr4:73922581:T:C:UTR3:COX18	chr4	73922581	73922581	T	C	UTR3	COX18	ENST000002	.	.	A	G	-	-	0.25806452	23,0,8,0	chr4,739225	Cytosol	A > G
chr4:73922593:T:C:UTR3:COX18	chr4	73922593	73922593	T	C	UTR3	COX18	ENST000002	.	.	A	G	-	-	0.26923077	19,0,7,0	chr4,739225	Cytosol	A > G
chr4:73922594:T:C:UTR3:COX18	chr4	73922594	73922594	T	C	UTR3	COX18	ENST000002	.	.	A	G	-	-	0.07142857	26,0,2,0	chr4,739225	Cytosol	A > G
chr4:73922620:T:C:UTR3:COX18	chr4	73922620	73922620	T	C	UTR3	COX18	ENST000002	.	.	A	G	-	-	0.23076923	20,0,6,0	chr4,739226	Cytosol	A > G
chr4:73922672:T:C:UTR3:COX18	chr4	73922672	73922672	T	C	UTR3	COX18	ENST000002	.	.	A	G	-	-	0.26315789	14,0,5,0	chr4,739226	Cytosol	A > G
chr4:73922743:T:C:UTR3:COX18	chr4	73922743	73922743	T	C	UTR3	COX18	ENST000002	.	.	A	G	-	-	0.32653061	33,0,16,0	chr4,739227	Cytosol	A > G
chr4:73922755:T:C:UTR3:COX18	chr4	73922755	73922755	T	C	UTR3	COX18	ENST000002	.	.	A	G	-	-	0.12280702	50,0,7,0	chr4,739227	Cytosol	A > G
chr4:73922756:T:C:UTR3:COX18	chr4	73922756	73922756	T	C	UTR3	COX18	ENST000002	.	.	A	G	-	-	0.06896552	54,0,4,0	chr4,739227	Cytosol	A > G
chr4:73922899:T:C:UTR3:COX18	chr4	73922899	73922899	T	C	UTR3	COX18	ENST000002	.	.	A	G	-	-	0.28571429	35,0,14,0	chr4,739228	Cytosol	A > G
chr4:73922926:T:C:UTR3:COX18	chr4	73922926	73922926	T	C	UTR3	COX18	ENST000002	.	.	A	G	-	-	0.06666667	70,0,5,0	chr4,739229	Cytosol	A > G
chr4:73922964:T:C:UTR3:COX18	chr4	73922964	73922964	T	C	UTR3	COX18	ENST000002	.	.	A	G	-	-	0.09589041	66,0,7,0	chr4,739229	Cytosol	A > G
chr4:73922981:T:C:UTR3:COX18	chr4	73922981	73922981	T	C	UTR3	COX18	ENST000002	.	.	A	G	-	-	0.34883721	28,0,15,0	chr4,739229	Cytosol	A > G
chr4:73922996:T:C:UTR3:COX18	chr4	73922996	73922996	T	C	UTR3	COX18	ENST000002	.	.	A	G	-	-	0.13157895	33,0,5,0	chr4,739229	Cytosol	A > G
chr4:73923043:T:C:UTR3:COX18	chr4	73923043	73923043	T	C	UTR3	COX18	ENST000002	.	.	A	G	-	-	0.22222222	35,0,10,0	chr4,739230	Cytosol	A > G
chr4:73923091:T:C:UTR3:COX18	chr4	73923091	73923091	T	C	UTR3	COX18	ENST000002	.	.	A	G	-	-	0.03846154	50,0,2,0	chr4,739230	Cytosol	A > G
chr4:73923096:T:C:UTR3:COX18	chr4	73923096	73923096	T	C	UTR3	COX18	ENST000002	.	.	A	G	-	-	0.07142857	52,0,4,0	chr4,739230	Cytosol	A > G
chr4:73923097:T:C:UTR3:COX18	chr4	73923097	73923097	T	C	UTR3	COX18	ENST000002	.	.	A	G	-	-	0.36	32,0,18,0	chr4,739230	Cytosol	A > G
chr4:73923105:T:C:UTR3:COX18	chr4	73923105	73923105	T	C	UTR3	COX18	ENST000002	.	.	A	G	-	-	0.0625	45,0,3,0	chr4,739231	Cytosol	A > G
chr4:73923414:T:C:UTR3:COX18	chr4	73923414	73923414	T	C	UTR3	COX18	ENST000002	.	.	A	G	-	-	0.05102041	93,0,5,0	chr4,739234	Cytosol	A > G
chr4:73923449:T:C:UTR3:COX18	chr4	73923449	73923449	T	C	UTR3	COX18	ENST000002	.	.	A	G	-	-	0.33663366	67,0,34,0	chr4,739234	Cytosol	A > G
chr4:73923452:T:C:UTR3:COX18	chr4	73923452	73923452	T	C	UTR3	COX18	ENST000002	.	.	A	G	-	-	0.03370787	86,0,3,0	chr4,739234	Cytosol	A > G
chr4:73923498:T:C:UTR3:COX18	chr4	73923498	73923498	T	C	UTR3	COX18	ENST000002	.	.	A	G	-	-	0.10810811	33,0,4,0	chr4,739234	Cytosol	A > G
chr4:73923524:T:C:UTR3:COX18	chr4	73923524	73923524	T	C	UTR3	COX18	ENST000002	.	.	A	G	-	-	0.0952381	38,0,4,0	chr4,739235	Cytosol	A > G
chr4:73923541:T:C:UTR3:COX18	chr4	73923541	73923541	T	C	UTR3	COX18	ENST000002	.	.	A	G	-	-	0.15151515	28,0,5,0	chr4,739235	Cytosol	A > G
chr4:73923551:T:C:UTR3:COX18	chr4	73923551	73923551	T	C	UTR3	COX18	ENST000002	.	.	A	G	-	-	0.125	28,0,4,0	chr4,739235	Cytosol	A > G
chr4:73923567:T:C:UTR3:COX18	chr4	73923567	73923567	T	C	UTR3	COX18	ENST000002	.	.	A	G	-	-	0.08823529	31,0,3,0	chr4,739235	Cytosol	A > G
chr4:73923568:T:C:UTR3:COX18	chr4	73923568	73923568	T	C	UTR3	COX18	ENST000002	.	.	A	G	-	-	0.14705882	29,0,5,0	chr4,739235	Cytosol	A > G
chr4:73923592:T:C:UTR3:COX18	chr4	73923592	73923592	T	C	UTR3	COX18	ENST000002	.	.	A	G	-	-	0.43333333	17,0,13,0	chr4,739235	Cytosol	A > G
chr4:73923607:T:C:UTR3:COX18	chr4	73923607	73923607	T	C	UTR3	COX18	ENST000002	.	.	A	G	-	-	0.13793103	25,0,4,0	chr4,739236	Cytosol	A > G
chr4:73923661:T:C:UTR3:COX18	chr4	73923661	73923661	T	C	UTR3	COX18	ENST000002	.	.	A	G	-	-	0.23529412	26,0,8,0	chr4,739236	Cytosol	A > G
chr4:73923662:T:C:UTR3:COX18	chr4	73923662	73923662	T	C	UTR3	COX18	ENST000002	.	.	A	G	-	-	0.23529412	26,0,8,0	chr4,739236	Cytosol	A > G
chr4:84215350:T:C:UTR3:HPSE	chr4	84215350	84215350	T	C	UTR3	HPSE	ENST000003	.	.	A	G	-	-	0.35714286	9,0,5,0	chr4,842153	Cytosol	A > G
chr4:84215354:T:C:UTR3:HPSE	chr4	84215354	84215354	T	C	UTR3	HPSE	ENST000003	.	.	A	G	-	-	0.23529412	13,0,4,0	chr4,842153	Cytosol	A > G
chr4:84215360:T:C:UTR3:HPSE	chr4	84215360	84215360	T	C	UTR3	HPSE	ENST000003	.	.	A	G	-	-	0.23529412	13,0,4,0	chr4,842153	Cytosol	A > G

chr4:84215384:T:C:UTR3:HPSE	chr4	84215384	84215384	T	C	UTR3	HPSE	ENST000003	.	.	A	G	-	-	0.58823529	7,0,10,0	chr4,842153	Cytosol	A > G
chr4:84215387:T:C:UTR3:HPSE	chr4	84215387	84215387	T	C	UTR3	HPSE	ENST000003	.	.	A	G	-	-	0.3125	11,0,5,0	chr4,842153	Cytosol	A > G
chr4:84215391:T:C:UTR3:HPSE	chr4	84215391	84215391	T	C	UTR3	HPSE	ENST000003	.	.	A	G	-	-	0.125	14,0,2,0	chr4,842153	Cytosol	A > G
chr4:84215392:T:C:UTR3:HPSE	chr4	84215392	84215392	T	C	UTR3	HPSE	ENST000003	.	.	A	G	-	-	0.11111111	16,0,2,0	chr4,842153	Cytosol	A > G
chr4:84215416:T:C:UTR3:HPSE	chr4	84215416	84215416	T	C	UTR3	HPSE	ENST000003	.	.	A	G	-	-	0.36842105	12,0,7,0	chr4,842154	Cytosol	A > G
chr4:84215438:T:C:UTR3:HPSE	chr4	84215438	84215438	T	C	UTR3	HPSE	ENST000003	.	.	A	G	-	-	0.21052632	15,0,4,0	chr4,842154	Cytosol	A > G
chr4:84215479:T:C:UTR3:HPSE	chr4	84215479	84215479	T	C	UTR3	HPSE	ENST000003	.	.	A	G	-	-	0.22727273	17,0,5,0	chr4,842154	Cytosol	A > G
chr4:84215718:T:C:UTR3:HPSE	chr4	84215718	84215718	T	C	UTR3	HPSE	ENST000003	.	.	A	G	-	-	0.07142857	26,0,2,0	chr4,842157	Cytosol	A > G
chr4:84215723:T:C:UTR3:HPSE	chr4	84215723	84215723	T	C	UTR3	HPSE	ENST000003	.	.	A	G	-	-	0.08823529	31,0,3,0	chr4,842157	Cytosol	A > G
chr4:84215797:T:C:UTR3:HPSE	chr4	84215797	84215797	T	C	UTR3	HPSE	ENST000003	.	.	A	G	-	-	0.09302326	39,0,4,0	chr4,842157	Cytosol	A > G
chr4:84215851:T:C:UTR3:HPSE	chr4	84215851	84215851	T	C	UTR3	HPSE	ENST000003	.	.	A	G	-	-	0.1	36,0,4,0	chr4,842158	Cytosol	A > G
chr4:84216169:T:C:UTR3:HPSE	chr4	84216169	84216169	T	C	UTR3	HPSE	ENST000003	.	.	A	G	-	-	0.36	16,0,9,0	chr4,842161	Cytosol	A > G
chr4:87844320:T:C:intronic:C4orf3	chr4	87844320	87844320	T	C	intronic	C4orf36	.	.	.	A	G	-	-	0.23076923	10,0,3,0	chr4,878443	Cytosol	A > G
chr4:87844340:T:C:intronic:C4orf3	chr4	87844340	87844340	T	C	intronic	C4orf36	.	.	.	A	G	-	-	0.125	14,0,2,0	chr4,878443	Cytosol	A > G
chr4:87844347:T:C:intronic:C4orf3	chr4	87844347	87844347	T	C	intronic	C4orf36	.	.	.	A	G	-	-	0.38461538	8,0,5,0	chr4,878443	Cytosol	A > G
chr4:87844382:T:C:intronic:C4orf3	chr4	87844382	87844382	T	C	intronic	C4orf36	.	.	.	A	G	-	-	0.4	6,0,4,0	chr4,878443	Cytosol	A > G
chr4:87844480:T:C:intronic:C4orf3	chr4	87844480	87844480	T	C	intronic	C4orf36	.	.	.	A	G	-	-	0.42857143	4,0,3,0	chr4,878444	Cytosol	A > G
chr4:87844502:T:C:intronic:C4orf3	chr4	87844502	87844502	T	C	intronic	C4orf36	.	.	.	A	G	-	-	0.55555556	4,0,5,0	chr4,878445	Cytosol	A > G
chr4:87844666:T:C:intronic:C4orf3	chr4	87844666	87844666	T	C	intronic	C4orf36	.	.	.	A	G	-	-	0.57142857	3,0,4,0	chr4,878446	Cytosol	A > G
chr4:87844738:T:C:intronic:C4orf3	chr4	87844738	87844738	T	C	intronic	C4orf36	.	.	.	A	G	-	-	0.85714286	1,0,6,0	chr4,878447	Cytosol	A > G
chr4:87844744:T:C:intronic:C4orf3	chr4	87844744	87844744	T	C	intronic	C4orf36	.	.	.	A	G	-	-	0.75	2,0,6,0	chr4,878447	Cytosol	A > G
chr4:87844747:T:C:intronic:C4orf3	chr4	87844747	87844747	T	C	intronic	C4orf36	.	.	.	A	G	-	-	0.55555556	4,0,5,0	chr4,878447	Cytosol	A > G
chr4:87844760:T:C:intronic:C4orf3	chr4	87844760	87844760	T	C	intronic	C4orf36	.	.	.	A	G	-	-	0.6	4,0,6,0	chr4,878447	Cytosol	A > G
chr4:87844855:T:C:intronic:C4orf3	chr4	87844855	87844855	T	C	intronic	C4orf36	.	.	.	A	G	-	-	0.4	3,0,2,0	chr4,878448	Cytosol	A > G
chr4:87845235:T:C:intronic:C4orf3	chr4	87845235	87845235	T	C	intronic	C4orf36	.	.	.	A	G	-	-	0.18181818	9,0,2,0	chr4,878452	Cytosol	A > G
chr4:87845414:T:C:intronic:C4orf3	chr4	87845414	87845414	T	C	intronic	C4orf36	.	.	.	A	G	-	-	0.5	5,0,5,0	chr4,878454	Cytosol	A > G
chr4:87845417:T:C:intronic:C4orf3	chr4	87845417	87845417	T	C	intronic	C4orf36	.	.	.	A	G	-	-	0.16666667	10,0,2,0	chr4,878454	Cytosol	A > G
chr4:87845419:T:C:intronic:C4orf3	chr4	87845419	87845419	T	C	intronic	C4orf36	.	.	.	A	G	-	-	0.2	8,0,2,0	chr4,878454	Cytosol	A > G
chr4:87845428:T:C:intronic:C4orf3	chr4	87845428	87845428	T	C	intronic	C4orf36	.	.	.	A	G	-	-	0.375	5,0,3,0	chr4,878454	Cytosol	A > G
chr4:87845531:T:C:intronic:C4orf3	chr4	87845531	87845531	T	C	intronic	C4orf36	.	.	.	A	G	-	-	0.6	2,0,3,0	chr4,878455	Cytosol	A > G
chr4:87845541:T:C:intronic:C4orf3	chr4	87845541	87845541	T	C	intronic	C4orf36	.	.	.	A	G	-	-	0.6	2,0,3,0	chr4,878455	Cytosol	A > G
chr4:87845770:T:C:intronic:C4orf3	chr4	87845770	87845770	T	C	intronic	C4orf36	.	.	.	A	G	-	-	0.27272727	8,0,3,0	chr4,878457	Cytosol	A > G
chr4:87846538:T:C:intronic:C4orf3	chr4	87846538	87846538	T	C	intronic	C4orf36	.	.	.	A	G	-	-	0.10526316	17,0,2,0	chr4,878465	Cytosol	A > G
chr4:87847105:T:C:intronic:C4orf3	chr4	87847105	87847105	T	C	intronic	C4orf36	.	.	.	A	G	-	-	0.375	5,0,3,0	chr4,878471	Cytosol	A > G
chr4:87847157:T:C:intronic:C4orf3	chr4	87847157	87847157	T	C	intronic	C4orf36	.	.	.	A	G	-	-	0.2	8,0,2,0	chr4,878471	Cytosol	A > G
chr4:87847166:T:C:intronic:C4orf3	chr4	87847166	87847166	T	C	intronic	C4orf36	.	.	.	A	G	-	-	0.4	6,0,4,0	chr4,878471	Cytosol	A > G
chr4:87847167:T:C:intronic:C4orf3	chr4	87847167	87847167	T	C	intronic	C4orf36	.	.	.	A	G	-	-	0.22222222	7,0,2,0	chr4,878471	Cytosol	A > G
chr4:87847258:T:C:intronic:C4orf3	chr4	87847258	87847258	T	C	intronic	C4orf36	.	.	.	A	G	-	-	0.6	2,0,3,0	chr4,878472	Cytosol	A > G
chr4:87847273:T:C:intronic:C4orf3	chr4	87847273	87847273	T	C	intronic	C4orf36	.	.	.	A	G	-	-	0.33333333	6,0,3,0	chr4,878472	Cytosol	A > G
chr4:87853387:T:C:UTR5:C4orf36	chr4	87853387	87853387	T	C	UTR5	C4orf36	ENST000004	.	.	A	G	-	-	0.26315789	14,0,5,0	chr4,878533	Cytosol	A > G
chr4:87853430:T:C:UTR5:C4orf36	chr4	87853430	87853430	T	C	UTR5	C4orf36	ENST000004	.	.	A	G	-	-	0.375	5,0,3,0	chr4,878534	Cytosol	A > G
chr4:87853463:T:C:UTR5:C4orf36	chr4	87853463	87853463	T	C	UTR5	C4orf36	ENST000004	.	.	A	G	-	-	0.22222222	14,0,4,0	chr4,878534	Cytosol	A > G
chr4:87916278:A:G:intronic:AFF1	chr4	87916278	87916278	A	G	intronic	AFF1	.	.	.	A	G	+	+	1	0,0,6,0	chr4,879162	Cytosol	A > G
chr4:87918133:A:G:intronic:AFF1	chr4	87918133	87918133	A	G	intronic	AFF1	.	.	.	A	G	+	+	0.625	3,0,5,0	chr4,879181	Cytosol	A > G
chr4:87918325:A:G:intronic:AFF1	chr4	87918325	87918325	A	G	intronic	AFF1	.	.	.	A	G	+	+	0.35714286	9,0,5,0	chr4,879183	Cytosol	A > G
chr4:87918326:A:G:intronic:AFF1	chr4	87918326	87918326	A	G	intronic	AFF1	.	.	.	A	G	+	+	0.66666667	5,0,10,0	chr4,879183	Cytosol	A > G
chr4:87918338:A:G:intronic:AFF1	chr4	87918338	87918338	A	G	intronic	AFF1	.	.	.	A	G	+	+	0.23076923	10,0,3,0	chr4,879183	Cytosol	A > G
chr4:87919005:A:G:intronic:AFF1	chr4	87919005	87919005	A	G	intronic	AFF1	.	.	.	A	G	+	+	0.15	17,0,3,0	chr4,879190	Cytosol	A > G
chr4:87919006:A:G:intronic:AFF1	chr4	87919006	87919006	A	G	intronic	AFF1	.	.	.	A	G	+	+	0.14285714	18,0,3,0	chr4,879190	Cytosol	A > G
chr4:87919130:A:G:intronic:AFF1	chr4	87919130	87919130	A	G	intronic	AFF1	.	.	.	A	G	+	+	0.10526316	17,0,2,0	chr4,879191	Cytosol	A > G
chr4:87919151:A:G:intronic:AFF1	chr4	87919151	87919151	A	G	intronic	AFF1	.	.	.	A	G	+	+	0.4	9,0,6,0	chr4,879191	Cytosol	A > G
chr4:87919175:A:G:intronic:AFF1	chr4	87919175	87919175	A	G	intronic	AFF1	.	.	.	A	G	+	+	0.33333333	10,0,5,0	chr4,879191	Cytosol	A > G
chr4:89179595:T:C:UTR3:PPM1K	chr4	89179595	89179595	T	C	UTR3	PPM1K	ENST000006	.	.	A	G	-	-	0.30769231	9,0,4,0	chr4,891795	Cytosol	A > G
chr4:89180296:T:C:UTR3:PPM1K	chr4	89180296	89180296	T	C	UTR3	PPM1K	ENST000006	.	.	A	G	-	-	0.4	3,0,2,0	chr4,891802	Cytosol	A > G
chr4:89180334:T:C:UTR3:PPM1K	chr4	89180334	89180334	T	C	UTR3	PPM1K	ENST000006	.	.	A	G	-	-	0.33333333	6,0,3,0	chr4,891803	Cytosol	A > G
chr4:99806882:T:C:intronic:EIF4E	chr4	99806882	99806882	T	C	intronic	EIF4E	.	.	.	A	G	-	-	0.2	8,0,2,0	chr4,998068	Cytosol	A > G
chr4:100801747:T:C:UTR3:LAMTO	chr4	100801747	100801747	T	C	UTR3	LAMTOR3	ENST000004	.	.	A	G	-	-	0.46153846	14,0,12,0	chr4,100801	Cytosol	A > G
chr4:100802179:T:C:UTR3:LAMTO	chr4	100802179	100802179	T	C	UTR3	LAMTOR3	ENST000004	.	.	A	G	-	-	0.11538462	23,0,3,0	chr4,100802	Cytosol	A > G
chr4:103983892:T:C:intronic:SLC9	chr4	103983892	103983892	T	C	intronic	SLC9B2	.	.	.	A	G	-	-	0.4	3,0,2,0	chr4,103983	Cytosol	A > G
chr4:103983905:T:C:intronic:SLC9	chr4	103983905	103983905	T	C	intronic	SLC9B2	.	.	.	A	G	-	-	0.6	2,0,3,0	chr4,103983	Cytosol	A > G
chr4:103983968:T:C:intronic:SLC9	chr4	103983968	103983968	T	C	intronic	SLC9B2	.	.	.	A	G	-	-	0.71428571	2,0,5,0	chr4,103983	Cytosol	A > G
chr4:103984039:T:C:intronic:SLC9	chr4	103984039	103984039	T	C	intronic	SLC9B2	.	.	.	A	G	-	-	0.5	4,0,4,0	chr4,103984	Cytosol	A > G
chr4:103984058:T:C:intronic:SLC9	chr4	103984058	103984058	T	C	intronic	SLC9B2	.	.	.	A	G	-	-	0.55555556	4,0,5,0	chr4,103984	Cytosol	A > G
chr4:106341267:T:C:intronic:PPA2	chr4	106341267	106341267	T	C	intronic	PPA2	.	.	.	A	G	-	-	0.6	2,0,3,0	chr4,106341	Cytosol	A > G

chr4:106341294:T:C:intronic:PPA2	chr4	106341294	106341294	T	C	intronic	PPA2	.	.	.	A	G	-	-	1	0,0,7,0	chr4,106341	Cytosol	A > G
chr4:106341302:T:C:intronic:PPA2	chr4	106341302	106341302	T	C	intronic	PPA2	.	.	.	A	G	-	-	0.66666667	2,0,4,0	chr4,106341	Cytosol	A > G
chr4:106341506:T:C:intronic:PPA2	chr4	106341506	106341506	T	C	intronic	PPA2	.	.	.	A	G	-	-	0.27272727	8,0,3,0	chr4,106341	Cytosol	A > G
chr4:106341510:T:C:intronic:PPA2	chr4	106341510	106341510	T	C	intronic	PPA2	.	.	.	A	G	-	-	0.18181818	9,0,2,0	chr4,106341	Cytosol	A > G
chr4:106341528:T:C:intronic:PPA2	chr4	106341528	106341528	T	C	intronic	PPA2	.	.	.	A	G	-	-	0.25	9,0,3,0	chr4,106341	Cytosol	A > G
chr4:106341535:T:C:intronic:PPA2	chr4	106341535	106341535	T	C	intronic	PPA2	.	.	.	A	G	-	-	0.5	6,0,6,0	chr4,106341	Cytosol	A > G
chr4:106341536:T:C:intronic:PPA2	chr4	106341536	106341536	T	C	intronic	PPA2	.	.	.	A	G	-	-	0.30769231	9,0,4,0	chr4,106341	Cytosol	A > G
chr4:106342952:T:C:intronic:PPA2	chr4	106342952	106342952	T	C	intronic	PPA2	.	.	.	A	G	-	-	0.55555556	4,0,5,0	chr4,106342	Cytosol	A > G
chr4:106343042:T:C:intronic:PPA2	chr4	106343042	106343042	T	C	intronic	PPA2	.	.	.	A	G	-	-	0.28571429	5,0,2,0	chr4,106343	Cytosol	A > G
chr4:106343051:T:C:intronic:PPA2	chr4	106343051	106343051	T	C	intronic	PPA2	.	.	.	A	G	-	-	1	0,0,5,0	chr4,106343	Cytosol	A > G
chr4:106343078:T:C:intronic:PPA2	chr4	106343078	106343078	T	C	intronic	PPA2	.	.	.	A	G	-	-	0.83333333	1,0,5,0	chr4,106343	Cytosol	A > G
chr4:106343088:T:C:intronic:PPA2	chr4	106343088	106343088	T	C	intronic	PPA2	.	.	.	A	G	-	-	0.5	3,0,3,0	chr4,106343	Cytosol	A > G
chr4:106343124:T:C:intronic:PPA2	chr4	106343124	106343124	T	C	intronic	PPA2	.	.	.	A	G	-	-	0.8	1,0,4,0	chr4,106343	Cytosol	A > G
chr4:106767318:A:G:UTR3:GSTCD	chr4	106767318	106767318	A	G	UTR3	GSTCD	ENST000003	.	.	A	G	+	-+	0.05785124	114,0,7,0	chr4,106767	Cytosol	A > G
chr4:106767321:A:G:UTR3:GSTCD	chr4	106767321	106767321	A	G	UTR3	GSTCD	ENST000003	.	.	A	G	+	-+	0.03149606	123,0,4,0	chr4,106767	Cytosol	A > G
chr4:106767880:A:G:UTR3:GSTCD	chr4	106767880	106767880	A	G	UTR3	GSTCD	ENST000003	.	.	A	G	+	-+	0.03703704	156,0,6,0	chr4,106767	Cytosol	A > G
chr4:106767930:A:G:UTR3:GSTCD	chr4	106767930	106767930	A	G	UTR3	GSTCD	ENST000003	.	.	A	G	+	-+	0.01626016	121,0,2,0	chr4,106767	Cytosol	A > G
chr4:106767946:A:G:UTR3:GSTCD	chr4	106767946	106767946	A	G	UTR3	GSTCD	ENST000003	.	.	A	G	+	-+	0.16666667	105,0,21,0	chr4,106767	Cytosol	A > G
chr4:106767952:A:G:UTR3:GSTCD	chr4	106767952	106767952	A	G	UTR3	GSTCD	ENST000003	.	.	A	G	+	-+	0.11764706	105,0,14,0	chr4,106767	Cytosol	A > G
chr4:106768068:A:G:UTR3:GSTCD	chr4	106768068	106768068	A	G	UTR3	GSTCD	ENST000003	.	.	A	G	+	-+	0.03448276	84,0,3,0	chr4,106768	Cytosol	A > G
chr4:106768078:A:G:UTR3:GSTCD	chr4	106768078	106768078	A	G	UTR3	GSTCD	ENST000003	.	.	A	G	+	-+	0.19117647	55,0,13,0	chr4,106768	Cytosol	A > G
chr4:109547684:A:G:intronic:RPL3	chr4	109547684	109547684	A	G	intronic	RPL34	.	.	.	A	G	+	+	1	0,0,5,0	chr4,109547	Cytosol	A > G
chr4:109548872:A:G:intronic:RPL3	chr4	109548872	109548872	A	G	intronic	RPL34	.	.	.	A	G	+	+	0.75	2,0,6,0	chr4,109548	Cytosol	A > G
chr4:109548950:A:G:intronic:RPL3	chr4	109548950	109548950	A	G	intronic	RPL34	.	.	.	A	G	+	+	0.6	2,0,3,0	chr4,109548	Cytosol	A > G
chr4:109550174:A:G:intronic:RPL3	chr4	109550174	109550174	A	G	intronic	RPL34	.	.	.	A	G	+	+	0.4	3,0,2,0	chr4,109550	Cytosol	A > G
chr4:109550248:A:G:intronic:RPL3	chr4	109550248	109550248	A	G	intronic	RPL34	.	.	.	A	G	+	+	0.25	6,0,2,0	chr4,109550	Cytosol	A > G
chr4:110610260:T:C:UTR3:CASP6	chr4	110610260	110610260	T	C	UTR3	CASP6	ENST000003	.	.	A	G	-	-	0.0462963	103,0,5,0	chr4,110610	Cytosol	A > G
chr4:110610264:T:C:UTR3:CASP6	chr4	110610264	110610264	T	C	UTR3	CASP6	ENST000003	.	.	A	G	-	-	0.0754717	98,0,8,0	chr4,110610	Cytosol	A > G
chr4:110610269:T:C:UTR3:CASP6	chr4	110610269	110610269	T	C	UTR3	CASP6	ENST000003	.	.	A	G	-	-	0.02272727	129,0,3,0	chr4,110610	Cytosol	A > G
chr4:110610296:T:C:UTR3:CASP6	chr4	110610296	110610296	T	C	UTR3	CASP6	ENST000003	.	.	A	G	-	-	0.01169591	169,0,2,0	chr4,110610	Cytosol	A > G
chr4:110631902:T:C:UTR3:PLA2G1	chr4	110631902	110631902	T	C	UTR3	PLA2G12A	ENST000002	.	.	A	G	-	-	0.01941748	101,0,2,0	chr4,110631	Cytosol	A > G
chr4:113525161:T:C:intronic:C4or	chr4	113525161	113525161	T	C	intronic	C4orf21	.	.	.	A	G	-	-	0.33333333	8,0,4,0	chr4,113525	Cytosol	A > G
chr4:113525199:T:C:intronic:C4or	chr4	113525199	113525199	T	C	intronic	C4orf21	.	.	.	A	G	-	-	0.33333333	4,0,2,0	chr4,113525	Cytosol	A > G
chr4:120423369:A:G:ncRNA_intro	chr4	120423369	120423369	A	G	ncRNA_intro	RP11-33B1.1	.	.	.	A	G	+	+-	0.2	12,0,3,0	chr4,120423	Cytosol	A > G
chr4:120423371:A:G:ncRNA_intro	chr4	120423371	120423371	A	G	ncRNA_intro	RP11-33B1.1	.	.	.	A	G	+	+-	0.53333333	7,0,8,0	chr4,120423	Cytosol	A > G
chr4:124237067:A:G:UTR3:SPATA	chr4	124237067	124237067	A	G	UTR3	SPATA5	ENST000002	.	.	A	G	+	+	0.375	15,0,9,0	chr4,124237	Cytosol	A > G
chr4:124237075:A:G:UTR3:SPATA	chr4	124237075	124237075	A	G	UTR3	SPATA5	ENST000002	.	.	A	G	+	+	0.30769231	18,0,8,0	chr4,124237	Cytosol	A > G
chr4:124237124:A:G:UTR3:SPATA	chr4	124237124	124237124	A	G	UTR3	SPATA5	ENST000002	.	.	A	G	+	+	0.11764706	15,0,2,0	chr4,124237	Cytosol	A > G
chr4:124239811:A:G:UTR3:SPATA	chr4	124239811	124239811	A	G	UTR3	SPATA5	ENST000002	.	.	A	G	+	+	0.11111111	24,0,3,0	chr4,124239	Cytosol	A > G
chr4:124239847:A:G:UTR3:SPATA	chr4	124239847	124239847	A	G	UTR3	SPATA5	ENST000002	.	.	A	G	+	+	0.05882353	32,0,2,0	chr4,124239	Cytosol	A > G
chr4:128758981:A:G:UTR3:HSPA4	chr4	128758981	128758981	A	G	UTR3	HSPA4L	ENST000002	.	.	A	G	+	+	0.07692308	36,0,3,0	chr4,128758	Cytosol	A > G
chr4:128758990:A:G:UTR3:HSPA4	chr4	128758990	128758990	A	G	UTR3	HSPA4L	ENST000002	.	.	A	G	+	+	0.51219512	20,0,21,0	chr4,128758	Cytosol	A > G
chr4:128759014:A:G:UTR3:HSPA4	chr4	128759014	128759014	A	G	UTR3	HSPA4L	ENST000002	.	.	A	G	+	+	0.33333333	28,0,14,0	chr4,128759	Cytosol	A > G
chr4:128759016:A:G:UTR3:HSPA4	chr4	128759016	128759016	A	G	UTR3	HSPA4L	ENST000002	.	.	A	G	+	+	0.38636364	27,0,17,0	chr4,128759	Cytosol	A > G
chr4:128759017:A:G:UTR3:HSPA4	chr4	128759017	128759017	A	G	UTR3	HSPA4L	ENST000002	.	.	A	G	+	+	0.54347826	21,0,25,0	chr4,128759	Cytosol	A > G
chr4:128759027:A:G:UTR3:HSPA4	chr4	128759027	128759027	A	G	UTR3	HSPA4L	ENST000002	.	.	A	G	+	+	0.43478261	26,0,20,0	chr4,128759	Cytosol	A > G
chr4:128759032:A:G:UTR3:HSPA4	chr4	128759032	128759032	A	G	UTR3	HSPA4L	ENST000002	.	.	A	G	+	+	0.30952381	29,0,13,0	chr4,128759	Cytosol	A > G
chr4:128759033:A:G:UTR3:HSPA4	chr4	128759033	128759033	A	G	UTR3	HSPA4L	ENST000002	.	.	A	G	+	+	0.2972973	26,0,11,0	chr4,128759	Cytosol	A > G
chr4:128759039:A:G:UTR3:HSPA4	chr4	128759039	128759039	A	G	UTR3	HSPA4L	ENST000002	.	.	A	G	+	+	0.13888889	31,0,5,0	chr4,128759	Cytosol	A > G
chr4:128759085:A:G:UTR3:HSPA4	chr4	128759085	128759085	A	G	UTR3	HSPA4L	ENST000002	.	.	A	G	+	+	0.38095238	13,0,8,0	chr4,128759	Cytosol	A > G
chr4:128759086:A:G:UTR3:HSPA4	chr4	128759086	128759086	A	G	UTR3	HSPA4L	ENST000002	.	.	A	G	+	+	0.22222222	14,0,4,0	chr4,128759	Cytosol	A > G
chr4:128759089:A:G:UTR3:HSPA4	chr4	128759089	128759089	A	G	UTR3	HSPA4L	ENST000002	.	.	A	G	+	+	0.58823529	7,0,10,0	chr4,128759	Cytosol	A > G
chr4:128759099:A:G:UTR3:HSPA4	chr4	128759099	128759099	A	G	UTR3	HSPA4L	ENST000002	.	.	A	G	+	+	0.70833333	7,0,17,0	chr4,128759	Cytosol	A > G
chr4:128759104:A:G:UTR3:HSPA4	chr4	128759104	128759104	A	G	UTR3	HSPA4L	ENST000002	.	.	A	G	+	+	0.08333333	22,0,2,0	chr4,128759	Cytosol	A > G
chr4:128759105:A:G:UTR3:HSPA4	chr4	128759105	128759105	A	G	UTR3	HSPA4L	ENST000002	.	.	A	G	+	+	0.41666667	14,0,10,0	chr4,128759	Cytosol	A > G
chr4:128759109:A:G:UTR3:HSPA4	chr4	128759109	128759109	A	G	UTR3	HSPA4L	ENST000002	.	.	A	G	+	+	0.15	17,0,3,0	chr4,128759	Cytosol	A > G
chr4:128759140:A:G:UTR3:HSPA4	chr4	128759140	128759140	A	G	UTR3	HSPA4L	ENST000002	.	.	A	G	+	+	0.6	8,0,12,0	chr4,128759	Cytosol	A > G
chr4:128759143:A:G:UTR3:HSPA4	chr4	128759143	128759143	A	G	UTR3	HSPA4L	ENST000002	.	.	A	G	+	+	0.625	6,0,10,0	chr4,128759	Cytosol	A > G
chr4:128759158:A:G:UTR3:HSPA4	chr4	128759158	128759158	A	G	UTR3	HSPA4L	ENST000002	.	.	A	G	+	+	0.09090909	20,0,2,0	chr4,128759	Cytosol	A > G
chr4:128759167:A:G:UTR3:HSPA4	chr4	128759167	128759167	A	G	UTR3	HSPA4L	ENST000002	.	.	A	G	+	+	0.25	12,0,4,0	chr4,128759	Cytosol	A > G
chr4:128759218:A:G:UTR3:HSPA4	chr4	128759218	128759218	A	G	UTR3	HSPA4L	ENST000002	.	.	A	G	+	+	0.3	21,0,9,0	chr4,128759	Cytosol	A > G
chr4:128759232:A:G:UTR3:HSPA4	chr4	128759232	128759232	A	G	UTR3	HSPA4L	ENST000002	.	.	A	G	+	+	0.11111111	24,0,3,0	chr4,128759	Cytosol	A > G
chr4:128760470:A:G:UTR3:HSPA4	chr4	128760470	128760470	A	G	UTR3	HSPA4L	ENST000002	.	.	A	G	+	+	0.34615385	17,0,9,0	chr4,128760	Cytosol	A > G
chr4:128760586:A:G:UTR3:HSPA4	chr4	128760586	128760586	A	G	UTR3	HSPA4L	ENST000002	.	.	A	G	+	+	0.16	21,0,4,0	chr4,128760	Cytosol	A > G

chr4:128760622:A:G:UTR3:HSPA4I	chr4	128760622	128760622	A	G	UTR3	HSPA4L	ENST000002	.	.	A	G	+	+	0.12	22,0,3,0	chr4,128760	Cytosol	A > G
chr4:128760632:A:G:UTR3:HSPA4I	chr4	128760632	128760632	A	G	UTR3	HSPA4L	ENST000002	.	.	A	G	+	+	0.11538462	23,0,3,0	chr4,128760	Cytosol	A > G
chr4:128760725:A:G:UTR3:HSPA4I	chr4	128760725	128760725	A	G	UTR3	HSPA4L	ENST000002	.	.	A	G	+	+	0.33333333	8,0,4,0	chr4,128760	Cytosol	A > G
chr4:128760727:A:G:UTR3:HSPA4I	chr4	128760727	128760727	A	G	UTR3	HSPA4L	ENST000002	.	.	A	G	+	+	0.25	12,0,4,0	chr4,128760	Cytosol	A > G
chr4:128760769:A:G:UTR3:HSPA4I	chr4	128760769	128760769	A	G	UTR3	HSPA4L	ENST000002	.	.	A	G	+	+	0.13636364	19,0,3,0	chr4,128760	Cytosol	A > G
chr4:128761255:A:G:UTR3:HSPA4I	chr4	128761255	128761255	A	G	UTR3	HSPA4L	ENST000002	.	.	A	G	+	+	0.30769231	27,0,12,0	chr4,128761	Cytosol	A > G
chr4:128761256:A:G:UTR3:HSPA4I	chr4	128761256	128761256	A	G	UTR3	HSPA4L	ENST000002	.	.	A	G	+	+	0.19047619	34,0,8,0	chr4,128761	Cytosol	A > G
chr4:128957671:A:G:UTR3:C4orf2	chr4	128957671	128957671	A	G	UTR3	C4orf29	ENST000003	.	.	A	G	+	+	0.15384615	11,0,2,0	chr4,128957	Cytosol	A > G
chr4:128957684:A:G:UTR3:C4orf2	chr4	128957684	128957684	A	G	UTR3	C4orf29	ENST000003	.	.	A	G	+	+	0.22222222	7,0,2,0	chr4,128957	Cytosol	A > G
chr4:128957704:A:G:UTR3:C4orf2	chr4	128957704	128957704	A	G	UTR3	C4orf29	ENST000003	.	.	A	G	+	+	0.36363636	7,0,4,0	chr4,128957	Cytosol	A > G
chr4:128957730:A:G:UTR3:C4orf2	chr4	128957730	128957730	A	G	UTR3	C4orf29	ENST000003	.	.	A	G	+	+	0.17647059	14,0,3,0	chr4,128957	Cytosol	A > G
chr4:128957740:A:G:UTR3:C4orf2	chr4	128957740	128957740	A	G	UTR3	C4orf29	ENST000003	.	.	A	G	+	+	0.11764706	15,0,2,0	chr4,128957	Cytosol	A > G
chr4:128958383:A:G:UTR3:C4orf2	chr4	128958383	128958383	A	G	UTR3	C4orf29	ENST000003	.	.	A	G	+	+	0.3125	11,0,5,0	chr4,128958	Cytosol	A > G
chr4:128958589:A:G:UTR3:C4orf2	chr4	128958589	128958589	A	G	UTR3	C4orf29	ENST000003	.	.	A	G	+	+	0.25	15,0,5,0	chr4,128958	Cytosol	A > G
chr4:128958595:A:G:UTR3:C4orf2	chr4	128958595	128958595	A	G	UTR3	C4orf29	ENST000003	.	.	A	G	+	+	0.1	18,0,2,0	chr4,128958	Cytosol	A > G
chr4:128958622:A:G:UTR3:C4orf2	chr4	128958622	128958622	A	G	UTR3	C4orf29	ENST000003	.	.	A	G	+	+	0.52631579	9,0,10,0	chr4,128958	Cytosol	A > G
chr4:128958627:A:G:UTR3:C4orf2	chr4	128958627	128958627	A	G	UTR3	C4orf29	ENST000003	.	.	A	G	+	+	0.7	6,0,14,0	chr4,128958	Cytosol	A > G
chr4:128958632:A:G:UTR3:C4orf2	chr4	128958632	128958632	A	G	UTR3	C4orf29	ENST000003	.	.	A	G	+	+	0.6	8,0,12,0	chr4,128958	Cytosol	A > G
chr4:128958634:A:G:UTR3:C4orf2	chr4	128958634	128958634	A	G	UTR3	C4orf29	ENST000003	.	.	A	G	+	+	0.14285714	18,0,3,0	chr4,128958	Cytosol	A > G
chr4:128958673:A:G:UTR3:C4orf2	chr4	128958673	128958673	A	G	UTR3	C4orf29	ENST000003	.	.	A	G	+	+	0.31578947	13,0,6,0	chr4,128958	Cytosol	A > G
chr4:128958674:A:G:UTR3:C4orf2	chr4	128958674	128958674	A	G	UTR3	C4orf29	ENST000003	.	.	A	G	+	+	0.15789474	16,0,3,0	chr4,128958	Cytosol	A > G
chr4:128958676:A:G:UTR3:C4orf2	chr4	128958676	128958676	A	G	UTR3	C4orf29	ENST000003	.	.	A	G	+	+	0.41176471	10,0,7,0	chr4,128958	Cytosol	A > G
chr4:128958683:A:G:UTR3:C4orf2	chr4	128958683	128958683	A	G	UTR3	C4orf29	ENST000003	.	.	A	G	+	+	0.35714286	9,0,5,0	chr4,128958	Cytosol	A > G
chr4:128958703:A:G:UTR3:C4orf2	chr4	128958703	128958703	A	G	UTR3	C4orf29	ENST000003	.	.	A	G	+	+	0.76923077	3,0,10,0	chr4,128958	Cytosol	A > G
chr4:128958728:A:G:UTR3:C4orf2	chr4	128958728	128958728	A	G	UTR3	C4orf29	ENST000003	.	.	A	G	+	+	0.35714286	9,0,5,0	chr4,128958	Cytosol	A > G
chr4:139966801:A:G:UTR3:CCRN4	chr4	139966801	139966801	A	G	UTR3	CCRN4L	ENST000002	.	.	A	G	+	+-	0.05714286	33,0,2,0	chr4,139966	Cytosol	A > G
chr4:139966877:A:G:UTR3:CCRN4	chr4	139966877	139966877	A	G	UTR3	CCRN4L	ENST000002	.	.	A	G	+	+-	0.34090909	29,0,15,0	chr4,139966	Cytosol	A > G
chr4:139967804:A:G:downstream:	chr4	139967804	139967804	A	G	downstream	CCRN4L	.	.	.	A	G	+	-	0.33333333	4,0,2,0	chr4,139967	Cytosol	A > G
chr4:139968398:A:G:intergenic:CC	chr4	139968398	139968398	A	G	intergenic	CCRN4L,RNU	dist=1492;dis	.	.	A	G	+	-	0.11538462	46,0,6,0	chr4,139968	Cytosol	A > G
chr4:139968467:A:G:intergenic:CC	chr4	139968467	139968467	A	G	intergenic	CCRN4L,RNU	dist=1561;dis	.	.	A	G	+	-	0.45	11,0,9,0	chr4,139968	Cytosol	A > G
chr4:139968493:A:G:intergenic:CC	chr4	139968493	139968493	A	G	intergenic	CCRN4L,RNU	dist=1587;dis	.	.	A	G	+	-	0.21052632	15,0,4,0	chr4,139968	Cytosol	A > G
chr4:139968506:A:G:intergenic:CC	chr4	139968506	139968506	A	G	intergenic	CCRN4L,RNU	dist=1600;dis	.	.	A	G	+	-	0.54545455	10,0,12,0	chr4,139968	Cytosol	A > G
chr4:139968532:A:G:intergenic:CC	chr4	139968532	139968532	A	G	intergenic	CCRN4L,RNU	dist=1626;dis	.	.	A	G	+	-	0.73684211	5,0,14,0	chr4,139968	Cytosol	A > G
chr4:139968533:A:G:intergenic:CC	chr4	139968533	139968533	A	G	intergenic	CCRN4L,RNU	dist=1627;dis	.	.	A	G	+	-	0.68421053	6,0,13,0	chr4,139968	Cytosol	A > G
chr4:139968542:A:G:intergenic:CC	chr4	139968542	139968542	A	G	intergenic	CCRN4L,RNU	dist=1636;dis	.	.	A	G	+	-	0.22222222	14,0,4,0	chr4,139968	Cytosol	A > G
chr4:139968556:A:G:intergenic:CC	chr4	139968556	139968556	A	G	intergenic	CCRN4L,RNU	dist=1650;dis	.	.	A	G	+	-	0.4	6,0,4,0	chr4,139968	Cytosol	A > G
chr4:139973053:T:C:intergenic:CC	chr4	139973053	139973053	T	C	intergenic	CCRN4L,RNU	dist=6147;dis	.	.	A	G	-	-	0.22222222	7,0,2,0	chr4,139973	Cytosol	A > G
chr4:153898310:A:G:UTR3:FHDC1	chr4	153898310	153898310	A	G	UTR3	FHDC1	ENST000005	.	.	A	G	+	+	0.4	3,0,2,0	chr4,153898	Cytosol	A > G
chr4:153898353:A:G:UTR3:FHDC1	chr4	153898353	153898353	A	G	UTR3	FHDC1	ENST000005	.	.	A	G	+	+	0.22222222	7,0,2,0	chr4,153898	Cytosol	A > G
chr4:153898397:A:G:UTR3:FHDC1	chr4	153898397	153898397	A	G	UTR3	FHDC1	ENST000005	.	.	A	G	+	+	0.27272727	8,0,3,0	chr4,153898	Cytosol	A > G
chr4:153898402:A:G:UTR3:FHDC1	chr4	153898402	153898402	A	G	UTR3	FHDC1	ENST000005	.	.	A	G	+	+	0.2	8,0,2,0	chr4,153898	Cytosol	A > G
chr4:153898454:A:G:UTR3:FHDC1	chr4	153898454	153898454	A	G	UTR3	FHDC1	ENST000005	.	.	A	G	+	+	0.16666667	10,0,2,0	chr4,153898	Cytosol	A > G
chr4:153898469:A:G:UTR3:FHDC1	chr4	153898469	153898469	A	G	UTR3	FHDC1	ENST000005	.	.	A	G	+	+	0.15384615	11,0,2,0	chr4,153898	Cytosol	A > G
chr4:165996287:T:C:UTR3:TMEM1	chr4	165996287	165996287	T	C	UTR3	TMEM192	ENST000003	.	.	A	G	-	-	0.71428571	8,0,20,0	chr4,165996	Cytosol	A > G
chr4:165997104:T:C:UTR3:TMEM1	chr4	165997104	165997104	T	C	UTR3	TMEM192	ENST000003	.	.	A	G	-	-	0.51851852	13,0,14,0	chr4,165997	Cytosol	A > G
chr4:165997105:T:C:UTR3:TMEM1	chr4	165997105	165997105	T	C	UTR3	TMEM192	ENST000003	.	.	A	G	-	-	0.43333333	17,0,13,0	chr4,165997	Cytosol	A > G
chr4:165997114:T:C:UTR3:TMEM1	chr4	165997114	165997114	T	C	UTR3	TMEM192	ENST000003	.	.	A	G	-	-	0.5625	14,0,18,0	chr4,165997	Cytosol	A > G
chr4:165997124:T:C:UTR3:TMEM1	chr4	165997124	165997124	T	C	UTR3	TMEM192	ENST000003	.	.	A	G	-	-	0.15	17,0,3,0	chr4,165997	Cytosol	A > G
chr4:165998019:T:C:UTR3:TMEM1	chr4	165998019	165998019	T	C	UTR3	TMEM192	ENST000003	.	.	A	G	-	-	0.25	24,0,8,0	chr4,165998	Cytosol	A > G
chr4:165998039:T:C:UTR3:TMEM1	chr4	165998039	165998039	T	C	UTR3	TMEM192	ENST000003	.	.	A	G	-	-	0.34285714	23,0,12,0	chr4,165998	Cytosol	A > G
chr4:165998078:T:C:UTR3:TMEM1	chr4	165998078	165998078	T	C	UTR3	TMEM192	ENST000003	.	.	A	G	-	-	0.35714286	18,0,10,0	chr4,165998	Cytosol	A > G
chr4:165998082:T:C:UTR3:TMEM1	chr4	165998082	165998082	T	C	UTR3	TMEM192	ENST000003	.	.	A	G	-	-	0.46428571	15,0,13,0	chr4,165998	Cytosol	A > G
chr4:165998092:T:C:UTR3:TMEM1	chr4	165998092	165998092	T	C	UTR3	TMEM192	ENST000003	.	.	A	G	-	-	0.35714286	18,0,10,0	chr4,165998	Cytosol	A > G
chr4:165998642:T:C:UTR3:TMEM1	chr4	165998642	165998642	T	C	UTR3	TMEM192	ENST000003	.	.	A	G	-	-	0.28787879	47,0,19,0	chr4,165998	Cytosol	A > G
chr4:165998654:T:C:UTR3:TMEM1	chr4	165998654	165998654	T	C	UTR3	TMEM192	ENST000003	.	.	A	G	-	-	0.16666667	35,0,7,0	chr4,165998	Cytosol	A > G
chr4:165998717:T:C:UTR3:TMEM1	chr4	165998717	165998717	T	C	UTR3	TMEM192	ENST000003	.	.	A	G	-	-	0.17857143	23,0,5,0	chr4,165998	Cytosol	A > G
chr4:165998785:T:C:UTR3:TMEM1	chr4	165998785	165998785	T	C	UTR3	TMEM192	ENST000003	.	.	A	G	-	-	0.30769231	9,0,4,0	chr4,165998	Cytosol	A > G
chr4:165998802:T:C:UTR3:TMEM1	chr4	165998802	165998802	T	C	UTR3	TMEM192	ENST000003	.	.	A	G	-	-	0.54545455	5,0,6,0	chr4,165998	Cytosol	A > G
chr4:165998856:T:C:UTR3:TMEM1	chr4	165998856	165998856	T	C	UTR3	TMEM192	ENST000003	.	.	A	G	-	-	0.33333333	4,0,2,0	chr4,165998	Cytosol	A > G
chr4:165998950:T:C:UTR3:TMEM1	chr4	165998950	165998950	T	C	UTR3	TMEM192	ENST000003	.	.	A	G	-	-	0.10714286	25,0,3,0	chr4,165998	Cytosol	A > G
chr4:165998958:T:C:UTR3:TMEM1	chr4	165998958	165998958	T	C	UTR3	TMEM192	ENST000003	.	.	A	G	-	-	0.24242424	25,0,8,0	chr4,165998	Cytosol	A > G
chr4:165999143:T:C:UTR3:TMEM1	chr4	165999143	165999143	T	C	UTR3	TMEM192	ENST000003	.	.	A	G	-	-	0.0754717	49,0,4,0	chr4,165999	Cytosol	A > G
chr4:165999188:T:C:UTR3:TMEM1	chr4	165999188	165999188	T	C	UTR3	TMEM192	ENST000003	.	.	A	G	-	-	0.08108108	68,0,6,0	chr4,165999	Cytosol	A > G
chr4:165999189:T:C:UTR3:TMEM1	chr4	165999189	165999189	T	C	UTR3	TMEM192	ENST000003	.	.	A	G	-	-	0.05555556	68,0,4,0	chr4,165999	Cytosol	A > G

chr4:166000080:T:C:UTR3:TMEM1	chr4	166000080	166000080	T	C	UTR3	TMEM192	ENST000003	.	.	A	G	-	-	0.06818182	41,0,3,0	chr4,166000	Cytosol	A > G
chr4:166000148:T:C:UTR3:TMEM1	chr4	166000148	166000148	T	C	UTR3	TMEM192	ENST000003	.	.	A	G	-	-	0.34042553	31,0,16,0	chr4,166000	Cytosol	A > G
chr4:166263518:A:T:UTR3:MSMO	chr4	166263518	166263518	A	T	UTR3	MSMO1	ENST000002	.	.	A	T	+	+	0.03743316	180,0,0,7	chr4,166263	Cytosol	A > T
chr4:186233886:A:G:intronic:SNX2	chr4	186233886	186233886	A	G	intronic	SNX25	.	.	.	A	G	+	+	0.625	3,0,5,0	chr4,186233	Cytosol	A > G
chr4:186236389:A:G:intronic:SNX2	chr4	186236389	186236389	A	G	intronic	SNX25	.	.	.	A	G	+	+	0.2	8,0,2,0	chr4,186236	Cytosol	A > G
chr4:186236390:A:G:intronic:SNX2	chr4	186236390	186236390	A	G	intronic	SNX25	.	.	.	A	G	+	+	0.44444444	5,0,4,0	chr4,186236	Cytosol	A > G
chr4:186236722:A:G:intronic:SNX2	chr4	186236722	186236722	A	G	intronic	SNX25	.	.	.	A	G	+	+	0.77777778	2,0,7,0	chr4,186236	Cytosol	A > G
chr4:186236728:A:G:intronic:SNX2	chr4	186236728	186236728	A	G	intronic	SNX25	.	.	.	A	G	+	+	0.25	6,0,2,0	chr4,186236	Cytosol	A > G
chr5:14715305:A:G:intronic:ANKH	chr5	14715305	14715305	A	G	intronic	ANKH	.	.	.	A	G	+	-+	0.22727273	17,0,5,0	chr5,147153	Cytosol	A > G
chr5:14715380:A:G:intronic:ANKH	chr5	14715380	14715380	A	G	intronic	ANKH	.	.	.	A	G	+	-+	0.1875	13,0,3,0	chr5,147153	Cytosol	A > G
chr5:14715416:A:G:intronic:ANKH	chr5	14715416	14715416	A	G	intronic	ANKH	.	.	.	A	G	+	-+	0.72727273	3,0,8,0	chr5,147154	Cytosol	A > G
chr5:14715420:A:G:intronic:ANKH	chr5	14715420	14715420	A	G	intronic	ANKH	.	.	.	A	G	+	-+	0.33333333	8,0,4,0	chr5,147154	Cytosol	A > G
chr5:14715423:A:G:intronic:ANKH	chr5	14715423	14715423	A	G	intronic	ANKH	.	.	.	A	G	+	-+	0.30769231	9,0,4,0	chr5,147154	Cytosol	A > G
chr5:14715449:A:G:intronic:ANKH	chr5	14715449	14715449	A	G	intronic	ANKH	.	.	.	A	G	+	-+	0.28571429	20,0,8,0	chr5,147154	Cytosol	A > G
chr5:14715516:A:G:intronic:ANKH	chr5	14715516	14715516	A	G	intronic	ANKH	.	.	.	A	G	+	-+	0.07407407	25,0,2,0	chr5,147155	Cytosol	A > G
chr5:14716333:A:G:intronic:ANKH	chr5	14716333	14716333	A	G	intronic	ANKH	.	.	.	A	G	+	-+	0.0952381	19,0,2,0	chr5,147163	Cytosol	A > G
chr5:14716392:A:G:intronic:ANKH	chr5	14716392	14716392	A	G	intronic	ANKH	.	.	.	A	G	+	-+	0.1	18,0,2,0	chr5,147163	Cytosol	A > G
chr5:14716400:A:G:intronic:ANKH	chr5	14716400	14716400	A	G	intronic	ANKH	.	.	.	A	G	+	-+	0.17647059	14,0,3,0	chr5,147163	Cytosol	A > G
chr5:14716455:A:G:intronic:ANKH	chr5	14716455	14716455	A	G	intronic	ANKH	.	.	.	A	G	+	-+	0.4	3,0,2,0	chr5,147164	Cytosol	A > G
chr5:34904163:T:C:intergenic:TTC	chr5	34904163	34904163	T	C	intergenic	TTC23L,RAD1	dist=4602;dis	.	.	A	G	-		0.6	2,0,3,0	chr5,349041	Cytosol	A > G
chr5:34906127:T:C:UTR3:RAD1	chr5	34906127	34906127	T	C	UTR3	RAD1	ENST000003	.	.	A	G	-	-	0.07894737	35,0,3,0	chr5,349061	Cytosol	A > G
chr5:34906162:T:C:UTR3:RAD1	chr5	34906162	34906162	T	C	UTR3	RAD1	ENST000003	.	.	A	G	-	-	0.2	28,0,7,0	chr5,349061	Cytosol	A > G
chr5:34906197:T:C:UTR3:RAD1	chr5	34906197	34906197	T	C	UTR3	RAD1	ENST000003	.	.	A	G	-	-	0.15384615	22,0,4,0	chr5,349061	Cytosol	A > G
chr5:34906198:T:C:UTR3:RAD1	chr5	34906198	34906198	T	C	UTR3	RAD1	ENST000003	.	.	A	G	-	-	0.08	23,0,2,0	chr5,349061	Cytosol	A > G
chr5:34906233:T:C:UTR3:RAD1	chr5	34906233	34906233	T	C	UTR3	RAD1	ENST000003	.	.	A	G	-	-	0.05263158	36,0,2,0	chr5,349062	Cytosol	A > G
chr5:34906239:T:C:UTR3:RAD1	chr5	34906239	34906239	T	C	UTR3	RAD1	ENST000003	.	.	A	G	-	-	0.05	38,0,2,0	chr5,349062	Cytosol	A > G
chr5:34906242:T:C:UTR3:RAD1	chr5	34906242	34906242	T	C	UTR3	RAD1	ENST000003	.	.	A	G	-	-	0.08333333	44,0,4,0	chr5,349062	Cytosol	A > G
chr5:34906274:T:C:UTR3:RAD1	chr5	34906274	34906274	T	C	UTR3	RAD1	ENST000003	.	.	A	G	-	-	0.04	48,0,2,0	chr5,349062	Cytosol	A > G
chr5:34906329:T:C:UTR3:RAD1	chr5	34906329	34906329	T	C	UTR3	RAD1	ENST000003	.	.	A	G	-	-	0.09433962	48,0,5,0	chr5,349063	Cytosol	A > G
chr5:34906334:T:C:UTR3:RAD1	chr5	34906334	34906334	T	C	UTR3	RAD1	ENST000003	.	.	A	G	-	-	0.57142857	24,0,32,0	chr5,349063	Cytosol	A > G
chr5:34906335:T:C:UTR3:RAD1	chr5	34906335	34906335	T	C	UTR3	RAD1	ENST000003	.	.	A	G	-	-	0.40384615	31,0,21,0	chr5,349063	Cytosol	A > G
chr5:34906341:T:C:UTR3:RAD1	chr5	34906341	34906341	T	C	UTR3	RAD1	ENST000003	.	.	A	G	-	-	0.33333333	30,0,15,0	chr5,349063	Cytosol	A > G
chr5:34906487:T:C:UTR3:RAD1	chr5	34906487	34906487	T	C	UTR3	RAD1	ENST000003	.	.	A	G	-	-	0.24390244	31,0,10,0	chr5,349064	Cytosol	A > G
chr5:34906499:T:C:UTR3:RAD1	chr5	34906499	34906499	T	C	UTR3	RAD1	ENST000003	.	.	A	G	-	-	0.09756098	37,0,4,0	chr5,349064	Cytosol	A > G
chr5:34906526:T:C:UTR3:RAD1	chr5	34906526	34906526	T	C	UTR3	RAD1	ENST000003	.	.	A	G	-	-	0.15555556	76,0,14,0	chr5,349065	Cytosol	A > G
chr5:34906547:T:C:UTR3:RAD1	chr5	34906547	34906547	T	C	UTR3	RAD1	ENST000003	.	.	A	G	-	-	0.21505376	73,0,20,0	chr5,349065	Cytosol	A > G
chr5:34906634:T:C:UTR3:RAD1	chr5	34906634	34906634	T	C	UTR3	RAD1	ENST000003	.	.	A	G	-	-	0.04225352	68,0,3,0	chr5,349066	Cytosol	A > G
chr5:34906657:T:C:UTR3:RAD1	chr5	34906657	34906657	T	C	UTR3	RAD1	ENST000003	.	.	A	G	-	-	0.2	76,0,19,0	chr5,349066	Cytosol	A > G
chr5:34906658:T:C:UTR3:RAD1	chr5	34906658	34906658	T	C	UTR3	RAD1	ENST000003	.	.	A	G	-	-	0.34693878	64,0,34,0	chr5,349066	Cytosol	A > G
chr5:34906661:T:C:UTR3:RAD1	chr5	34906661	34906661	T	C	UTR3	RAD1	ENST000003	.	.	A	G	-	-	0.41414141	58,0,41,0	chr5,349066	Cytosol	A > G
chr5:34906662:T:C:UTR3:RAD1	chr5	34906662	34906662	T	C	UTR3	RAD1	ENST000003	.	.	A	G	-	-	0.30208333	67,0,29,0	chr5,349066	Cytosol	A > G
chr5:34906700:T:C:UTR3:RAD1	chr5	34906700	34906700	T	C	UTR3	RAD1	ENST000003	.	.	A	G	-	-	0.06976744	80,0,6,0	chr5,349066	Cytosol	A > G
chr5:34906708:T:C:UTR3:RAD1	chr5	34906708	34906708	T	C	UTR3	RAD1	ENST000003	.	.	A	G	-	-	0.07042254	66,0,5,0	chr5,349067	Cytosol	A > G
chr5:34906737:T:C:UTR3:RAD1	chr5	34906737	34906737	T	C	UTR3	RAD1	ENST000003	.	.	A	G	-	-	0.29545455	31,0,13,0	chr5,349067	Cytosol	A > G
chr5:34906738:T:C:UTR3:RAD1	chr5	34906738	34906738	T	C	UTR3	RAD1	ENST000003	.	.	A	G	-	-	0.23255814	33,0,10,0	chr5,349067	Cytosol	A > G
chr5:34906750:T:C:UTR3:RAD1	chr5	34906750	34906750	T	C	UTR3	RAD1	ENST000003	.	.	A	G	-	-	0.14	43,0,7,0	chr5,349067	Cytosol	A > G
chr5:34908375:T:C:UTR3:RAD1	chr5	34908375	34908375	T	C	UTR3	RAD1	ENST000003	.	.	A	G	-	-	0.2	44,0,11,0	chr5,349083	Cytosol	A > G
chr5:34908443:T:C:UTR3:RAD1	chr5	34908443	34908443	T	C	UTR3	RAD1	ENST000003	.	.	A	G	-	-	0.10769231	58,0,7,0	chr5,349084	Cytosol	A > G
chr5:34908462:T:C:UTR3:RAD1	chr5	34908462	34908462	T	C	UTR3	RAD1	ENST000003	.	.	A	G	-	-	0.02105263	93,0,2,0	chr5,349084	Cytosol	A > G
chr5:34908507:T:C:UTR3:RAD1	chr5	34908507	34908507	T	C	UTR3	RAD1	ENST000003	.	.	A	G	-	-	0.02912621	100,0,3,0	chr5,349085	Cytosol	A > G
chr5:34908512:T:C:UTR3:RAD1	chr5	34908512	34908512	T	C	UTR3	RAD1	ENST000003	.	.	A	G	-	-	0.11392405	70,0,9,0	chr5,349085	Cytosol	A > G
chr5:34908517:T:C:UTR3:RAD1	chr5	34908517	34908517	T	C	UTR3	RAD1	ENST000003	.	.	A	G	-	-	0.02222222	88,0,2,0	chr5,349085	Cytosol	A > G
chr5:34953000:A:G:intronic:DNAJ	chr5	34953000	34953000	A	G	intronic	DNAJC21	.	.	.	A	G	+	+	0.25714286	26,0,9,0	chr5,349529	Cytosol	A > G
chr5:34953006:A:G:intronic:DNAJ	chr5	34953006	34953006	A	G	intronic	DNAJC21	.	.	.	A	G	+	+	0.09090909	30,0,3,0	chr5,349530	Cytosol	A > G
chr5:34953018:A:G:intronic:DNAJ	chr5	34953018	34953018	A	G	intronic	DNAJC21	.	.	.	A	G	+	+	0.17857143	23,0,5,0	chr5,349530	Cytosol	A > G
chr5:34953075:A:G:intronic:DNAJ	chr5	34953075	34953075	A	G	intronic	DNAJC21	.	.	.	A	G	+	+	0.26923077	19,0,7,0	chr5,349530	Cytosol	A > G
chr5:34953105:A:G:intronic:DNAJ	chr5	34953105	34953105	A	G	intronic	DNAJC21	.	.	.	A	G	+	+	0.09375	29,0,3,0	chr5,349531	Cytosol	A > G
chr5:34953108:A:G:intronic:DNAJ	chr5	34953108	34953108	A	G	intronic	DNAJC21	.	.	.	A	G	+	+	0.21212121	26,0,7,0	chr5,349531	Cytosol	A > G
chr5:34953187:A:G:intronic:DNAJ	chr5	34953187	34953187	A	G	intronic	DNAJC21	.	.	.	A	G	+	+	0.86666667	2,0,13,0	chr5,349531	Cytosol	A > G
chr5:34953418:A:G:intronic:DNAJ	chr5	34953418	34953418	A	G	intronic	DNAJC21	.	.	.	A	G	+	+	0.23255814	33,0,10,0	chr5,349534	Cytosol	A > G
chr5:34953446:A:G:intronic:DNAJ	chr5	34953446	34953446	A	G	intronic	DNAJC21	.	.	.	A	G	+	+	0.06818182	41,0,3,0	chr5,349534	Cytosol	A > G
chr5:34953453:A:G:intronic:DNAJ	chr5	34953453	34953453	A	G	intronic	DNAJC21	.	.	.	A	G	+	+	0.43478261	26,0,20,0	chr5,349534	Cytosol	A > G
chr5:34953505:A:G:intronic:DNAJ	chr5	34953505	34953505	A	G	intronic	DNAJC21	.	.	.	A	G	+	+	0.96	1,0,24,0	chr5,349535	Cytosol	A > G
chr5:34953547:A:G:intronic:DNAJ	chr5	34953547	34953547	A	G	intronic	DNAJC21	.	.	.	A	G	+	+	0.26315789	14,0,5,0	chr5,349535	Cytosol	A > G

chr5:34953578:A:G:intronic:DNAJ	chr5	34953578	34953578	A	G	intronic	DNAJC21	.	.	.	A	G	+	+	0.9	2,0,18,0	chr5,349535	Cytosol	A > G
chr5:37288767:T:C:UTR3:NUP155	chr5	37288767	37288767	T	C	UTR3	NUP155	ENST000002	.	.	A	G	-	-	0.15037594	113,0,20,0	chr5,372887	Cytosol	A > G
chr5:37288784:T:C:UTR3:NUP155	chr5	37288784	37288784	T	C	UTR3	NUP155	ENST000002	.	.	A	G	-	-	0.04807692	99,0,5,0	chr5,372887	Cytosol	A > G
chr5:37289051:T:C:UTR3:NUP155	chr5	37289051	37289051	T	C	UTR3	NUP155	ENST000002	.	.	A	G	-	-	0.53488372	20,0,23,0	chr5,372890	Cytosol	A > G
chr5:37289086:T:C:UTR3:NUP155	chr5	37289086	37289086	T	C	UTR3	NUP155	ENST000002	.	.	A	G	-	-	0.28169014	51,0,20,0	chr5,372890	Cytosol	A > G
chr5:37289087:T:C:UTR3:NUP155	chr5	37289087	37289087	T	C	UTR3	NUP155	ENST000002	.	.	A	G	-	-	0.39130435	42,0,27,0	chr5,372890	Cytosol	A > G
chr5:37289129:T:C:UTR3:NUP155	chr5	37289129	37289129	T	C	UTR3	NUP155	ENST000002	.	.	A	G	-	-	0.04950495	96,0,5,0	chr5,372891	Cytosol	A > G
chr5:37290323:T:C:UTR3:NUP155	chr5	37290323	37290323	T	C	UTR3	NUP155	ENST000002	.	.	A	G	-	-	0.17730496	232,0,50,0	chr5,372903	Cytosol	A > G
chr5:37290335:T:C:UTR3:NUP155	chr5	37290335	37290335	T	C	UTR3	NUP155	ENST000002	.	.	A	G	-	-	0.21126761	224,0,60,0	chr5,372903	Cytosol	A > G
chr5:37290336:T:C:UTR3:NUP155	chr5	37290336	37290336	T	C	UTR3	NUP155	ENST000002	.	.	A	G	-	-	0.12956811	262,0,39,0	chr5,372903	Cytosol	A > G
chr5:37290400:T:C:UTR3:NUP155	chr5	37290400	37290400	T	C	UTR3	NUP155	ENST000002	.	.	A	G	-	-	0.25735294	101,0,35,0	chr5,372903	Cytosol	A > G
chr5:37290416:T:C:UTR3:NUP155	chr5	37290416	37290416	T	C	UTR3	NUP155	ENST000002	.	.	A	G	-	-	0.50819672	60,0,62,0	chr5,372904	Cytosol	A > G
chr5:37290435:T:C:UTR3:NUP155	chr5	37290435	37290435	T	C	UTR3	NUP155	ENST000002	.	.	A	G	-	-	0.03061224	95,0,3,0	chr5,372904	Cytosol	A > G
chr5:37290467:T:C:UTR3:NUP155	chr5	37290467	37290467	T	C	UTR3	NUP155	ENST000002	.	.	A	G	-	-	0.91005291	17,0,172,0	chr5,372904	Cytosol	A > G
chr5:37290492:T:C:UTR3:NUP155	chr5	37290492	37290492	T	C	UTR3	NUP155	ENST000002	.	.	A	G	-	-	0.62612613	83,0,139,0	chr5,372904	Cytosol	A > G
chr5:37290493:T:C:UTR3:NUP155	chr5	37290493	37290493	T	C	UTR3	NUP155	ENST000002	.	.	A	G	-	-	0.68075117	68,0,145,0	chr5,372904	Cytosol	A > G
chr5:37290523:T:C:UTR3:NUP155	chr5	37290523	37290523	T	C	UTR3	NUP155	ENST000002	.	.	A	G	-	-	0.05	152,0,8,0	chr5,372905	Cytosol	A > G
chr5:37290554:T:C:UTR3:NUP155	chr5	37290554	37290554	T	C	UTR3	NUP155	ENST000002	.	.	A	G	-	-	0.07246377	128,0,10,0	chr5,372905	Cytosol	A > G
chr5:37290566:T:C:UTR3:NUP155	chr5	37290566	37290566	T	C	UTR3	NUP155	ENST000002	.	.	A	G	-	-	0.87179487	20,0,136,0	chr5,372905	Cytosol	A > G
chr5:37290571:T:C:UTR3:NUP155	chr5	37290571	37290571	T	C	UTR3	NUP155	ENST000002	.	.	A	G	-	-	0.09937888	145,0,16,0	chr5,372905	Cytosol	A > G
chr5:37291386:T:C:UTR3:NUP155	chr5	37291386	37291386	T	C	UTR3	NUP155	ENST000002	.	.	A	G	-	-	0.21428571	220,0,60,0	chr5,372913	Cytosol	A > G
chr5:37291446:T:C:UTR3:NUP155	chr5	37291446	37291446	T	C	UTR3	NUP155	ENST000002	.	.	A	G	-	-	0.63186813	67,0,115,0	chr5,372914	Cytosol	A > G
chr5:37291472:T:C:UTR3:NUP155	chr5	37291472	37291472	T	C	UTR3	NUP155	ENST000002	.	.	A	G	-	-	0.27857143	101,0,39,0	chr5,372914	Cytosol	A > G
chr5:37291482:T:C:UTR3:NUP155	chr5	37291482	37291482	T	C	UTR3	NUP155	ENST000002	.	.	A	G	-	-	0.22222222	70,0,20,0	chr5,372914	Cytosol	A > G
chr5:37291508:T:C:UTR3:NUP155	chr5	37291508	37291508	T	C	UTR3	NUP155	ENST000002	.	.	A	G	-	-	0.16438356	61,0,12,0	chr5,372915	Cytosol	A > G
chr5:37291526:T:C:UTR3:NUP155	chr5	37291526	37291526	T	C	UTR3	NUP155	ENST000002	.	.	A	G	-	-	0.05063291	75,0,4,0	chr5,372915	Cytosol	A > G
chr5:37291529:T:C:UTR3:NUP155	chr5	37291529	37291529	T	C	UTR3	NUP155	ENST000002	.	.	A	G	-	-	0.04938272	77,0,4,0	chr5,372915	Cytosol	A > G
chr5:37291540:T:C:UTR3:NUP155	chr5	37291540	37291540	T	C	UTR3	NUP155	ENST000002	.	.	A	G	-	-	0.45882353	46,0,39,0	chr5,372915	Cytosol	A > G
chr5:37291541:T:C:UTR3:NUP155	chr5	37291541	37291541	T	C	UTR3	NUP155	ENST000002	.	.	A	G	-	-	0.21505376	73,0,20,0	chr5,372915	Cytosol	A > G
chr5:37291542:T:C:UTR3:NUP155	chr5	37291542	37291542	T	C	UTR3	NUP155	ENST000002	.	.	A	G	-	-	0.23232323	76,0,23,0	chr5,372915	Cytosol	A > G
chr5:37291550:T:C:UTR3:NUP155	chr5	37291550	37291550	T	C	UTR3	NUP155	ENST000002	.	.	A	G	-	-	0.37719298	71,0,43,0	chr5,372915	Cytosol	A > G
chr5:37291559:T:C:UTR3:NUP155	chr5	37291559	37291559	T	C	UTR3	NUP155	ENST000002	.	.	A	G	-	-	0.67647059	33,0,69,0	chr5,372915	Cytosol	A > G
chr5:37291573:T:C:UTR3:NUP155	chr5	37291573	37291573	T	C	UTR3	NUP155	ENST000002	.	.	A	G	-	-	0.78095238	23,0,82,0	chr5,372915	Cytosol	A > G
chr5:37291574:T:C:UTR3:NUP155	chr5	37291574	37291574	T	C	UTR3	NUP155	ENST000002	.	.	A	G	-	-	0.12037037	95,0,13,0	chr5,372915	Cytosol	A > G
chr5:37291579:T:C:UTR3:NUP155	chr5	37291579	37291579	T	C	UTR3	NUP155	ENST000002	.	.	A	G	-	-	0.02941176	99,0,3,0	chr5,372915	Cytosol	A > G
chr5:37291618:T:C:UTR3:NUP155	chr5	37291618	37291618	T	C	UTR3	NUP155	ENST000002	.	.	A	G	-	-	0.30097087	72,0,31,0	chr5,372916	Cytosol	A > G
chr5:37291624:T:C:UTR3:NUP155	chr5	37291624	37291624	T	C	UTR3	NUP155	ENST000002	.	.	A	G	-	-	0.13684211	82,0,13,0	chr5,372916	Cytosol	A > G
chr5:37291630:T:C:UTR3:NUP155	chr5	37291630	37291630	T	C	UTR3	NUP155	ENST000002	.	.	A	G	-	-	0.12765957	82,0,12,0	chr5,372916	Cytosol	A > G
chr5:37398308:A:G:intronic:WDR7	chr5	37398308	37398308	A	G	intronic	WDR70	.	.	.	A	G	+	+	0.4	3,0,2,0	chr5,373983	Cytosol	A > G
chr5:37398320:A:G:intronic:WDR7	chr5	37398320	37398320	A	G	intronic	WDR70	.	.	.	A	G	+	+	0.6	2,0,3,0	chr5,373983	Cytosol	A > G
chr5:40728227:T:C:intronic:TTC33	chr5	40728227	40728227	T	C	intronic	TTC33	.	.	.	A	G	-	-	0.28571429	5,0,2,0	chr5,407282	Cytosol	A > G
chr5:40762119:T:C:UTR3:PRKAA1	chr5	40762119	40762119	T	C	UTR3	PRKAA1	ENST000003	.	.	A	G	-	-	0.02364865	289,0,7,0	chr5,407621	Cytosol	A > G
chr5:40825936:T:C:UTR3:RPL37	chr5	40825936	40825936	T	C	UTR3	RPL37	ENST000002	.	.	A	G	-	-	0.01960784	100,0,2,0	chr5,408259	Cytosol	A > G
chr5:40825945:T:C:UTR3:RPL37	chr5	40825945	40825945	T	C	UTR3	RPL37	ENST000002	.	.	A	G	-	-	0.02105263	93,0,2,0	chr5,408259	Cytosol	A > G
chr5:40830050:T:C:UTR3:RPL37	chr5	40830050	40830050	T	C	UTR3	RPL37	ENST000002	.	.	A	G	-	-	0.11538462	23,0,3,0	chr5,408300	Cytosol	A > G
chr5:40830052:T:C:UTR3:RPL37	chr5	40830052	40830052	T	C	UTR3	RPL37	ENST000002	.	.	A	G	-	-	0.23333333	23,0,7,0	chr5,408300	Cytosol	A > G
chr5:40830064:T:C:UTR3:RPL37	chr5	40830064	40830064	T	C	UTR3	RPL37	ENST000002	.	.	A	G	-	-	0.35714286	18,0,10,0	chr5,408300	Cytosol	A > G
chr5:40830452:T:C:UTR3:RPL37	chr5	40830452	40830452	T	C	UTR3	RPL37	ENST000002	.	.	A	G	-	-	0.12121212	29,0,4,0	chr5,408304	Cytosol	A > G
chr5:40830464:T:C:UTR3:RPL37	chr5	40830464	40830464	T	C	UTR3	RPL37	ENST000002	.	.	A	G	-	-	0.16666667	20,0,4,0	chr5,408304	Cytosol	A > G
chr5:40830465:T:C:UTR3:RPL37	chr5	40830465	40830465	T	C	UTR3	RPL37	ENST000002	.	.	A	G	-	-	0.14814815	23,0,4,0	chr5,408304	Cytosol	A > G
chr5:40830491:T:C:UTR3:RPL37	chr5	40830491	40830491	T	C	UTR3	RPL37	ENST000002	.	.	A	G	-	-	0.1875	26,0,6,0	chr5,408304	Cytosol	A > G
chr5:40830695:T:C:UTR3:RPL37	chr5	40830695	40830695	T	C	UTR3	RPL37	ENST000002	.	.	A	G	-	-	0.16666667	25,0,5,0	chr5,408306	Cytosol	A > G
chr5:40830696:T:C:UTR3:RPL37	chr5	40830696	40830696	T	C	UTR3	RPL37	ENST000002	.	.	A	G	-	-	0.125	28,0,4,0	chr5,408306	Cytosol	A > G
chr5:40830727:T:C:UTR3:RPL37	chr5	40830727	40830727	T	C	UTR3	RPL37	ENST000002	.	.	A	G	-	-	0.3030303	23,0,10,0	chr5,408307	Cytosol	A > G
chr5:40830737:T:C:UTR3:RPL37	chr5	40830737	40830737	T	C	UTR3	RPL37	ENST000002	.	.	A	G	-	-	0.05714286	33,0,2,0	chr5,408307	Cytosol	A > G
chr5:40830769:T:C:UTR3:RPL37	chr5	40830769	40830769	T	C	UTR3	RPL37	ENST000002	.	.	A	G	-	-	0.08	23,0,2,0	chr5,408307	Cytosol	A > G
chr5:40830777:T:C:UTR3:RPL37	chr5	40830777	40830777	T	C	UTR3	RPL37	ENST000002	.	.	A	G	-	-	0.66666667	7,0,14,0	chr5,408307	Cytosol	A > G
chr5:40830778:T:C:UTR3:RPL37	chr5	40830778	40830778	T	C	UTR3	RPL37	ENST000002	.	.	A	G	-	-	0.36363636	14,0,8,0	chr5,408307	Cytosol	A > G
chr5:40830794:T:C:UTR3:RPL37	chr5	40830794	40830794	T	C	UTR3	RPL37	ENST000002	.	.	A	G	-	-	0.15	17,0,3,0	chr5,408307	Cytosol	A > G
chr5:40830869:T:C:UTR3:RPL37	chr5	40830869	40830869	T	C	UTR3	RPL37	ENST000002	.	.	A	G	-	-	0.6	2,0,3,0	chr5,408308	Cytosol	A > G
chr5:40830870:T:C:UTR3:RPL37	chr5	40830870	40830870	T	C	UTR3	RPL37	ENST000002	.	.	A	G	-	-	0.5	3,0,3,0	chr5,408308	Cytosol	A > G
chr5:40831092:T:C:UTR3:RPL37	chr5	40831092	40831092	T	C	UTR3	RPL37	ENST000002	.	.	A	G	-	-	0.22222222	7,0,2,0	chr5,408310	Cytosol	A > G
chr5:40831181:T:C:UTR3:RPL37	chr5	40831181	40831181	T	C	UTR3	RPL37	ENST000002	.	.	A	G	-	-	0.22222222	21,0,6,0	chr5,408311	Cytosol	A > G
chr5:40831265:T:C:UTR3:RPL37	chr5	40831265	40831265	T	C	UTR3	RPL37	ENST000002	.	.	A	G	-	-	0.57894737	16,0,22,0	chr5,408312	Cytosol	A > G

chr5:40831275:T:C:UTR3:RPL37	chr5	40831275	40831275	T	C	UTR3	RPL37	ENST000002	.	.	A	G	-	-	0.13333333	26,0,4,0	chr5,408312	Cytosol	A > G
chr5:40831330:T:C:UTR3:RPL37	chr5	40831330	40831330	T	C	UTR3	RPL37	ENST000002	.	.	A	G	-	-	0.05405405	35,0,2,0	chr5,408313	Cytosol	A > G
chr5:43380817:T:C:UTR3:CCL28	chr5	43380817	43380817	T	C	UTR3	CCL28	ENST000003	.	.	A	G	-	-	0.33333333	4,0,2,0	chr5,433808	Cytosol	A > G
chr5:43380821:T:C:UTR3:CCL28	chr5	43380821	43380821	T	C	UTR3	CCL28	ENST000003	.	.	A	G	-	-	0.33333333	4,0,2,0	chr5,433808	Cytosol	A > G
chr5:43381599:T:C:UTR3:CCL28	chr5	43381599	43381599	T	C	UTR3	CCL28	ENST000003	.	.	A	G	-	-	0.2	8,0,2,0	chr5,433815	Cytosol	A > G
chr5:43381602:T:C:UTR3:CCL28	chr5	43381602	43381602	T	C	UTR3	CCL28	ENST000003	.	.	A	G	-	-	0.22222222	7,0,2,0	chr5,433816	Cytosol	A > G
chr5:43381613:T:C:UTR3:CCL28	chr5	43381613	43381613	T	C	UTR3	CCL28	ENST000003	.	.	A	G	-	-	0.4	3,0,2,0	chr5,433816	Cytosol	A > G
chr5:43381689:T:C:UTR3:CCL28	chr5	43381689	43381689	T	C	UTR3	CCL28	ENST000003	.	.	A	G	-	-	0.28571429	5,0,2,0	chr5,433816	Cytosol	A > G
chr5:43381708:T:C:UTR3:CCL28	chr5	43381708	43381708	T	C	UTR3	CCL28	ENST000003	.	.	A	G	-	-	0.6	2,0,3,0	chr5,433817	Cytosol	A > G
chr5:43381741:T:C:UTR3:CCL28	chr5	43381741	43381741	T	C	UTR3	CCL28	ENST000003	.	.	A	G	-	-	0.38461538	8,0,5,0	chr5,433817	Cytosol	A > G
chr5:43574975:T:C:ncRNA_exonic:	chr5	43574975	43574975	T	C	ncRNA_exon	NNT-AS1	.	.	.	A	G	-	-	0.10294118	61,0,7,0	chr5,435749	Cytosol	A > G
chr5:43574976:T:C:ncRNA_exonic:	chr5	43574976	43574976	T	C	ncRNA_exon	NNT-AS1	.	.	.	A	G	-	-	0.05714286	66,0,4,0	chr5,435749	Cytosol	A > G
chr5:43575020:T:C:ncRNA_exonic:	chr5	43575020	43575020	T	C	ncRNA_exon	NNT-AS1	.	.	.	A	G	-	-	0.16393443	51,0,10,0	chr5,435750	Cytosol	A > G
chr5:43575786:T:C:ncRNA_exonic:	chr5	43575786	43575786	T	C	ncRNA_exon	NNT-AS1	.	.	.	A	G	-	-	0.03448276	84,0,3,0	chr5,435757	Cytosol	A > G
chr5:43575804:T:C:ncRNA_exonic:	chr5	43575804	43575804	T	C	ncRNA_exon	NNT-AS1	.	.	.	A	G	-	-	0.05797101	65,0,4,0	chr5,435758	Cytosol	A > G
chr5:43575840:T:C:ncRNA_exonic:	chr5	43575840	43575840	T	C	ncRNA_exon	NNT-AS1	.	.	.	A	G	-	-	0.13636364	38,0,6,0	chr5,435758	Cytosol	A > G
chr5:43576329:T:C:ncRNA_introni	chr5	43576329	43576329	T	C	ncRNA_intro	NNT-AS1	.	.	.	A	G	-	-	0.4375	9,0,7,0	chr5,435763	Cytosol	A > G
chr5:43576380:T:C:ncRNA_introni	chr5	43576380	43576380	T	C	ncRNA_intro	NNT-AS1	.	.	.	A	G	-	-	0.125	14,0,2,0	chr5,435763	Cytosol	A > G
chr5:43576398:T:C:ncRNA_introni	chr5	43576398	43576398	T	C	ncRNA_intro	NNT-AS1	.	.	.	A	G	-	-	0.30769231	9,0,4,0	chr5,435763	Cytosol	A > G
chr5:44815668:A:G:downstream:	chr5	44815668	44815668	A	G	downstream	MRPS30	.	.	.	A	G	+	+	0.19047619	17,0,4,0	chr5,448156	Cytosol	A > G
chr5:44815669:A:G:downstream:	chr5	44815669	44815669	A	G	downstream	MRPS30	.	.	.	A	G	+	+	0.17647059	14,0,3,0	chr5,448156	Cytosol	A > G
chr5:44815675:A:G:downstream:	chr5	44815675	44815675	A	G	downstream	MRPS30	.	.	.	A	G	+	+	0.4	12,0,8,0	chr5,448156	Cytosol	A > G
chr5:44815725:A:G:downstream:	chr5	44815725	44815725	A	G	downstream	MRPS30	.	.	.	A	G	+	+	0.25531915	35,0,12,0	chr5,448157	Cytosol	A > G
chr5:44815768:A:G:downstream:	chr5	44815768	44815768	A	G	downstream	MRPS30	.	.	.	A	G	+	+	0.10126582	71,0,8,0	chr5,448157	Cytosol	A > G
chr5:44815777:A:G:downstream:	chr5	44815777	44815777	A	G	downstream	MRPS30	.	.	.	A	G	+	+	0.37974684	49,0,30,0	chr5,448157	Cytosol	A > G
chr5:44815813:A:G:downstream:	chr5	44815813	44815813	A	G	downstream	MRPS30	.	.	.	A	G	+	+	0.04672897	102,0,5,0	chr5,448158	Cytosol	A > G
chr5:44815839:A:G:downstream:	chr5	44815839	44815839	A	G	downstream	MRPS30	.	.	.	A	G	+	+	0.06870229	122,0,9,0	chr5,448158	Cytosol	A > G
chr5:44816354:A:G:downstream:	chr5	44816354	44816354	A	G	downstream	MRPS30	.	.	.	A	G	+	+	0.2254902	79,0,23,0	chr5,448163	Cytosol	A > G
chr5:44816406:A:G:downstream:	chr5	44816406	44816406	A	G	downstream	MRPS30	.	.	.	A	G	+	+	0.25	78,0,26,0	chr5,448164	Cytosol	A > G
chr5:44816583:A:G:downstream:	chr5	44816583	44816583	A	G	downstream	MRPS30	.	.	.	A	G	+	+	0.55555556	28,0,35,0	chr5,448165	Cytosol	A > G
chr5:60452579:T:C:downstream:S	chr5	60452579	60452579	T	C	downstream	SMIM15	.	.	.	A	G	-		0.5	4,0,4,0	chr5,604525	Cytosol	A > G
chr5:64847015:T:C:exonic:CENPK	chr5	64847015	64847015	T	C	exonic	CENPK	.	nonsynonym	CENPK:ENST	A	G	-	-	0.4	3,0,2,0	chr5,648470	Cytosol	A > G
chr5:64964623:T:C:UTR3:SGTB	chr5	64964623	64964623	T	C	UTR3	SGTB	ENST000003	.	.	A	G	-	-	0.4	3,0,2,0	chr5,649646	Cytosol	A > G
chr5:64964626:T:C:UTR3:SGTB	chr5	64964626	64964626	T	C	UTR3	SGTB	ENST000003	.	.	A	G	-	-	0.8	1,0,4,0	chr5,649646	Cytosol	A > G
chr5:64964627:T:C:UTR3:SGTB	chr5	64964627	64964627	T	C	UTR3	SGTB	ENST000003	.	.	A	G	-	-	0.33333333	4,0,2,0	chr5,649646	Cytosol	A > G
chr5:64964702:T:C:UTR3:SGTB	chr5	64964702	64964702	T	C	UTR3	SGTB	ENST000003	.	.	A	G	-	-	0.5	4,0,4,0	chr5,649647	Cytosol	A > G
chr5:64964718:T:C:UTR3:SGTB	chr5	64964718	64964718	T	C	UTR3	SGTB	ENST000003	.	.	A	G	-	-	1	0,0,8,0	chr5,649647	Cytosol	A > G
chr5:68505967:A:G:UTR3:CENPH	chr5	68505967	68505967	A	G	UTR3	CENPH	ENST000002	.	.	A	G	+	+	0.07058824	79,0,6,0	chr5,685059	Cytosol	A > G
chr5:68506009:A:G:UTR3:CENPH	chr5	68506009	68506009	A	G	UTR3	CENPH	ENST000002	.	.	A	G	+	+	0.03773585	51,0,2,0	chr5,685060	Cytosol	A > G
chr5:68506094:A:G:UTR3:CENPH	chr5	68506094	68506094	A	G	UTR3	CENPH	ENST000002	.	.	A	G	+	+	0.10714286	50,0,6,0	chr5,685060	Cytosol	A > G
chr5:68576744:T:C:UTR3:CCDC125	chr5	68576744	68576744	T	C	UTR3	CCDC125	ENST000003	.	.	A	G	-	-	0.1	36,0,4,0	chr5,685767	Cytosol	A > G
chr5:68576752:T:C:UTR3:CCDC125	chr5	68576752	68576752	T	C	UTR3	CCDC125	ENST000003	.	.	A	G	-	-	0.04761905	40,0,2,0	chr5,685767	Cytosol	A > G
chr5:68577105:T:C:UTR3:CCDC125	chr5	68577105	68577105	T	C	UTR3	CCDC125	ENST000003	.	.	A	G	-	-	0.375	15,0,9,0	chr5,685771	Cytosol	A > G
chr5:68577119:T:C:UTR3:CCDC125	chr5	68577119	68577119	T	C	UTR3	CCDC125	ENST000003	.	.	A	G	-	-	0.0952381	19,0,2,0	chr5,685771	Cytosol	A > G
chr5:68577791:T:C:UTR3:CCDC125	chr5	68577791	68577791	T	C	UTR3	CCDC125	ENST000003	.	.	A	G	-	-	0.93333333	1,0,14,0	chr5,685777	Cytosol	A > G
chr5:68577815:T:C:UTR3:CCDC125	chr5	68577815	68577815	T	C	UTR3	CCDC125	ENST000003	.	.	A	G	-	-	0.22222222	14,0,4,0	chr5,685778	Cytosol	A > G
chr5:68577823:T:C:UTR3:CCDC125	chr5	68577823	68577823	T	C	UTR3	CCDC125	ENST000003	.	.	A	G	-	-	0.35714286	9,0,5,0	chr5,685778	Cytosol	A > G
chr5:68577846:T:C:UTR3:CCDC125	chr5	68577846	68577846	T	C	UTR3	CCDC125	ENST000003	.	.	A	G	-	-	0.53333333	7,0,8,0	chr5,685778	Cytosol	A > G
chr5:68577878:T:C:UTR3:CCDC125	chr5	68577878	68577878	T	C	UTR3	CCDC125	ENST000003	.	.	A	G	-	-	0.875	1,0,7,0	chr5,685778	Cytosol	A > G
chr5:68577882:T:C:UTR3:CCDC125	chr5	68577882	68577882	T	C	UTR3	CCDC125	ENST000003	.	.	A	G	-	-	0.875	1,0,7,0	chr5,685778	Cytosol	A > G
chr5:68577907:T:C:UTR3:CCDC125	chr5	68577907	68577907	T	C	UTR3	CCDC125	ENST000003	.	.	A	G	-	-	0.41666667	7,0,5,0	chr5,685779	Cytosol	A > G
chr5:68577912:T:C:UTR3:CCDC125	chr5	68577912	68577912	T	C	UTR3	CCDC125	ENST000003	.	.	A	G	-	-	0.33333333	8,0,4,0	chr5,685779	Cytosol	A > G
chr5:68577920:T:C:UTR3:CCDC125	chr5	68577920	68577920	T	C	UTR3	CCDC125	ENST000003	.	.	A	G	-	-	0.23076923	10,0,3,0	chr5,685779	Cytosol	A > G
chr5:68577922:T:C:UTR3:CCDC125	chr5	68577922	68577922	T	C	UTR3	CCDC125	ENST000003	.	.	A	G	-	-	0.35714286	9,0,5,0	chr5,685779	Cytosol	A > G
chr5:68577937:T:C:UTR3:CCDC125	chr5	68577937	68577937	T	C	UTR3	CCDC125	ENST000003	.	.	A	G	-	-	0.33333333	8,0,4,0	chr5,685779	Cytosol	A > G
chr5:68577950:T:C:UTR3:CCDC125	chr5	68577950	68577950	T	C	UTR3	CCDC125	ENST000003	.	.	A	G	-	-	0.38888889	11,0,7,0	chr5,685779	Cytosol	A > G
chr5:68578241:T:C:UTR3:CCDC125	chr5	68578241	68578241	T	C	UTR3	CCDC125	ENST000003	.	.	A	G	-	-	0.4375	9,0,7,0	chr5,685782	Cytosol	A > G
chr5:68578245:T:C:UTR3:CCDC125	chr5	68578245	68578245	T	C	UTR3	CCDC125	ENST000003	.	.	A	G	-	-	0.38888889	11,0,7,0	chr5,685782	Cytosol	A > G
chr5:68578267:T:C:UTR3:CCDC125	chr5	68578267	68578267	T	C	UTR3	CCDC125	ENST000003	.	.	A	G	-	-	0.2	12,0,3,0	chr5,685782	Cytosol	A > G
chr5:68578276:T:C:UTR3:CCDC125	chr5	68578276	68578276	T	C	UTR3	CCDC125	ENST000003	.	.	A	G	-	-	0.6	6,0,9,0	chr5,685782	Cytosol	A > G
chr5:68578277:T:C:UTR3:CCDC125	chr5	68578277	68578277	T	C	UTR3	CCDC125	ENST000003	.	.	A	G	-	-	0.3125	11,0,5,0	chr5,685782	Cytosol	A > G
chr5:68578302:T:C:UTR3:CCDC125	chr5	68578302	68578302	T	C	UTR3	CCDC125	ENST000003	.	.	A	G	-	-	0.3	7,0,3,0	chr5,685783	Cytosol	A > G
chr5:68578306:T:C:UTR3:CCDC125	chr5	68578306	68578306	T	C	UTR3	CCDC125	ENST000003	.	.	A	G	-	-	0.625	3,0,5,0	chr5,685783	Cytosol	A > G
chr5:68578307:T:C:UTR3:CCDC125	chr5	68578307	68578307	T	C	UTR3	CCDC125	ENST000003	.	.	A	G	-	-	0.44444444	5,0,4,0	chr5,685783	Cytosol	A > G

chr5:68578331:T:C:UTR3:CCDC125	chr5	68578331	68578331	T	C	UTR3	CCDC125	ENST000003	.	.	A	G	-	-	0.28571429	5,0,2,0	chr5,685783	Cytosol	A > G
chr5:70859238:A:G:intronic:BDP1	chr5	70859238	70859238	A	G	intronic	BDP1	.	.	.	A	G	+	+	0.4	3,0,2,0	chr5,708592	Cytosol	A > G
chr5:70859561:A:G:intronic:BDP1	chr5	70859561	70859561	A	G	intronic	BDP1	.	.	.	A	G	+	+	0.6	2,0,3,0	chr5,708595	Cytosol	A > G
chr5:70859618:A:G:intronic:BDP1	chr5	70859618	70859618	A	G	intronic	BDP1	.	.	.	A	G	+	+	0.33333333	4,0,2,0	chr5,708596	Cytosol	A > G
chr5:74673831:T:C:intronic:COL4A	chr5	74673831	74673831	T	C	intronic	COL4A3BP	.	.	.	A	G	-	-	0.22222222	7,0,2,0	chr5,746738	Cytosol	A > G
chr5:74673872:T:C:intronic:COL4A	chr5	74673872	74673872	T	C	intronic	COL4A3BP	.	.	.	A	G	-	-	0.3	7,0,3,0	chr5,746738	Cytosol	A > G
chr5:74673879:T:C:intronic:COL4A	chr5	74673879	74673879	T	C	intronic	COL4A3BP	.	.	.	A	G	-	-	0.3	7,0,3,0	chr5,746738	Cytosol	A > G
chr5:74674169:T:C:intronic:COL4A	chr5	74674169	74674169	T	C	intronic	COL4A3BP	.	.	.	A	G	-	-	0.28571429	5,0,2,0	chr5,746741	Cytosol	A > G
chr5:74674816:T:C:intronic:COL4A	chr5	74674816	74674816	T	C	intronic	COL4A3BP	.	.	.	A	G	-	-	0.4	3,0,2,0	chr5,746748	Cytosol	A > G
chr5:77655393:T:C:ncRNA_exonic:	chr5	77655393	77655393	T	C	ncRNA_exon	CTD-2037K2	.	.	.	A	G	-	-	0.3	7,0,3,0	chr5,776553	Cytosol	A > G
chr5:79923329:T:C:UTR3:DHFR	chr5	79923329	79923329	T	C	UTR3	DHFR	ENST000004	.	.	A	G	-	-	0.5	6,0,6,0	chr5,799233	Cytosol	A > G
chr5:79923334:T:C:UTR3:DHFR	chr5	79923334	79923334	T	C	UTR3	DHFR	ENST000004	.	.	A	G	-	-	0.16666667	10,0,2,0	chr5,799233	Cytosol	A > G
chr5:79923378:T:C:UTR3:DHFR	chr5	79923378	79923378	T	C	UTR3	DHFR	ENST000004	.	.	A	G	-	-	0.5	6,0,6,0	chr5,799233	Cytosol	A > G
chr5:79923397:T:C:UTR3:DHFR	chr5	79923397	79923397	T	C	UTR3	DHFR	ENST000004	.	.	A	G	-	-	0.33333333	4,0,2,0	chr5,799233	Cytosol	A > G
chr5:79923402:T:C:UTR3:DHFR	chr5	79923402	79923402	T	C	UTR3	DHFR	ENST000004	.	.	A	G	-	-	0.66666667	3,0,6,0	chr5,799234	Cytosol	A > G
chr5:79923430:T:C:UTR3:DHFR	chr5	79923430	79923430	T	C	UTR3	DHFR	ENST000004	.	.	A	G	-	-	0.7	3,0,7,0	chr5,799234	Cytosol	A > G
chr5:79923826:T:C:UTR3:DHFR	chr5	79923826	79923826	T	C	UTR3	DHFR	ENST000004	.	.	A	G	-	-	1	0,0,17,0	chr5,799238	Cytosol	A > G
chr5:79923843:T:C:UTR3:DHFR	chr5	79923843	79923843	T	C	UTR3	DHFR	ENST000004	.	.	A	G	-	-	0.11111111	16,0,2,0	chr5,799238	Cytosol	A > G
chr5:79923855:T:C:UTR3:DHFR	chr5	79923855	79923855	T	C	UTR3	DHFR	ENST000004	.	.	A	G	-	-	0.06976744	40,0,3,0	chr5,799238	Cytosol	A > G
chr5:93018302:G:C:intronic:FAM1	chr5	93018302	93018302	G	C	intronic	FAM172A	.	.	.	C	G	-	-	1	0,0,19,0	chr5,930183	Cytosol	C > G
chr5:96110544:T:C:UTR3:ERAP1	chr5	96110544	96110544	T	C	UTR3	ERAP1	ENST000004	.	.	A	G	-	+-	0.16666667	25,0,5,0	chr5,961105	Cytosol	A > G
chr5:112228865:A:G:intronic:REEF	chr5	112228865	112228865	A	G	intronic	REEP5	.	.	.	A	G	+	-	0.66666667	2,0,4,0	chr5,112228	Cytosol	A > G
chr5:115165563:T:C:UTR3:ATG12	chr5	115165563	115165563	T	C	UTR3	ATG12	ENST000002	.	.	A	G	-	-	0.22222222	35,0,10,0	chr5,115165	Cytosol	A > G
chr5:115165591:T:C:UTR3:ATG12	chr5	115165591	115165591	T	C	UTR3	ATG12	ENST000002	.	.	A	G	-	-	0.67307692	17,0,35,0	chr5,115165	Cytosol	A > G
chr5:115165632:T:C:UTR3:ATG12	chr5	115165632	115165632	T	C	UTR3	ATG12	ENST000002	.	.	A	G	-	-	0.5060241	41,0,42,0	chr5,115165	Cytosol	A > G
chr5:115165731:T:C:UTR3:ATG12	chr5	115165731	115165731	T	C	UTR3	ATG12	ENST000002	.	.	A	G	-	-	0.13888889	62,0,10,0	chr5,115165	Cytosol	A > G
chr5:115166252:C:T:UTR3:ATG12	chr5	115166252	115166252	C	T	UTR3	ATG12	ENST000002	.	.	G	A	-	-	0.03286385	7,0,206,0	chr5,115166	Cytosol	G > A
chr5:118172032:T:C:downstream:	chr5	118172032	118172032	T	C	downstream	DTWD2	.	.	.	A	G	-	-	0.45833333	13,0,11,0	chr5,118172	Cytosol	A > G
chr5:118172058:T:C:downstream:	chr5	118172058	118172058	T	C	downstream	DTWD2	.	.	.	A	G	-	-	0.36	16,0,9,0	chr5,118172	Cytosol	A > G
chr5:118172152:T:C:downstream:	chr5	118172152	118172152	T	C	downstream	DTWD2	.	.	.	A	G	-	-	0.36842105	12,0,7,0	chr5,118172	Cytosol	A > G
chr5:118172169:T:C:downstream:	chr5	118172169	118172169	T	C	downstream	DTWD2	.	.	.	A	G	-	-	0.33333333	8,0,4,0	chr5,118172	Cytosol	A > G
chr5:118173470:T:C:UTR3:DTWD2	chr5	118173470	118173470	T	C	UTR3	DTWD2	ENST000003	.	.	A	G	-	-	0.05405405	35,0,2,0	chr5,118173	Cytosol	A > G
chr5:118173498:T:C:UTR3:DTWD2	chr5	118173498	118173498	T	C	UTR3	DTWD2	ENST000003	.	.	A	G	-	-	0.48275862	30,0,28,0	chr5,118173	Cytosol	A > G
chr5:118173506:T:C:UTR3:DTWD2	chr5	118173506	118173506	T	C	UTR3	DTWD2	ENST000003	.	.	A	G	-	-	0.19642857	45,0,11,0	chr5,118173	Cytosol	A > G
chr5:118173514:T:C:UTR3:DTWD2	chr5	118173514	118173514	T	C	UTR3	DTWD2	ENST000003	.	.	A	G	-	-	0.10606061	59,0,7,0	chr5,118173	Cytosol	A > G
chr5:118173531:T:C:UTR3:DTWD2	chr5	118173531	118173531	T	C	UTR3	DTWD2	ENST000003	.	.	A	G	-	-	0.11864407	52,0,7,0	chr5,118173	Cytosol	A > G
chr5:118173552:T:C:UTR3:DTWD2	chr5	118173552	118173552	T	C	UTR3	DTWD2	ENST000003	.	.	A	G	-	-	0.06382979	44,0,3,0	chr5,118173	Cytosol	A > G
chr5:118173592:T:C:UTR3:DTWD2	chr5	118173592	118173592	T	C	UTR3	DTWD2	ENST000003	.	.	A	G	-	-	0.05555556	34,0,2,0	chr5,118173	Cytosol	A > G
chr5:118174668:T:C:UTR3:DTWD2	chr5	118174668	118174668	T	C	UTR3	DTWD2	ENST000003	.	.	A	G	-	-	0.86486486	5,0,32,0	chr5,118174	Cytosol	A > G
chr5:118174733:T:C:UTR3:DTWD2	chr5	118174733	118174733	T	C	UTR3	DTWD2	ENST000003	.	.	A	G	-	-	0.7173913	13,0,33,0	chr5,118174	Cytosol	A > G
chr5:118174735:T:C:UTR3:DTWD2	chr5	118174735	118174735	T	C	UTR3	DTWD2	ENST000003	.	.	A	G	-	-	0.77272727	10,0,34,0	chr5,118174	Cytosol	A > G
chr5:118174745:T:C:UTR3:DTWD2	chr5	118174745	118174745	T	C	UTR3	DTWD2	ENST000003	.	.	A	G	-	-	0.21276596	37,0,10,0	chr5,118174	Cytosol	A > G
chr5:118174757:T:C:UTR3:DTWD2	chr5	118174757	118174757	T	C	UTR3	DTWD2	ENST000003	.	.	A	G	-	-	0.46153846	13,0,12,1	chr5,118174	Cytosol	A > G
chr5:118175165:T:C:UTR3:DTWD2	chr5	118175165	118175165	T	C	UTR3	DTWD2	ENST000003	.	.	A	G	-	-	0.51428571	17,0,18,0	chr5,118175	Cytosol	A > G
chr5:118175166:T:C:UTR3:DTWD2	chr5	118175166	118175166	T	C	UTR3	DTWD2	ENST000003	.	.	A	G	-	-	0.76315789	9,0,29,0	chr5,118175	Cytosol	A > G
chr5:118175182:T:C:UTR3:DTWD2	chr5	118175182	118175182	T	C	UTR3	DTWD2	ENST000003	.	.	A	G	-	-	0.0625	45,0,3,0	chr5,118175	Cytosol	A > G
chr5:118175188:T:C:UTR3:DTWD2	chr5	118175188	118175188	T	C	UTR3	DTWD2	ENST000003	.	.	A	G	-	-	0.0625	45,0,3,0	chr5,118175	Cytosol	A > G
chr5:118175210:T:C:UTR3:DTWD2	chr5	118175210	118175210	T	C	UTR3	DTWD2	ENST000003	.	.	A	G	-	-	0.43181818	25,0,19,0	chr5,118175	Cytosol	A > G
chr5:118175214:T:C:UTR3:DTWD2	chr5	118175214	118175214	T	C	UTR3	DTWD2	ENST000003	.	.	A	G	-	-	0.32608696	31,0,15,0	chr5,118175	Cytosol	A > G
chr5:118175258:T:C:UTR3:DTWD2	chr5	118175258	118175258	T	C	UTR3	DTWD2	ENST000003	.	.	A	G	-	-	0.10638298	42,0,5,0	chr5,118175	Cytosol	A > G
chr5:121362874:A:G:UTR3:SRFBP1	chr5	121362874	121362874	A	G	UTR3	SRFBP1	ENST000003	.	.	A	G	+	+	0.44117647	76,0,60,0	chr5,121362	Cytosol	A > G
chr5:121362875:A:G:UTR3:SRFBP1	chr5	121362875	121362875	A	G	UTR3	SRFBP1	ENST000003	.	.	A	G	+	+	0.39416058	83,0,54,0	chr5,121362	Cytosol	A > G
chr5:121362879:A:G:UTR3:SRFBP1	chr5	121362879	121362879	A	G	UTR3	SRFBP1	ENST000003	.	.	A	G	+	+	0.06617647	127,0,9,0	chr5,121362	Cytosol	A > G
chr5:121362904:A:G:UTR3:SRFBP1	chr5	121362904	121362904	A	G	UTR3	SRFBP1	ENST000003	.	.	A	G	+	+	0.14166667	103,0,17,0	chr5,121362	Cytosol	A > G
chr5:121362909:A:G:UTR3:SRFBP1	chr5	121362909	121362909	A	G	UTR3	SRFBP1	ENST000003	.	.	A	G	+	+	0.03252033	119,0,4,0	chr5,121362	Cytosol	A > G
chr5:121362912:A:G:UTR3:SRFBP1	chr5	121362912	121362912	A	G	UTR3	SRFBP1	ENST000003	.	.	A	G	+	+	0.01492537	132,0,2,0	chr5,121362	Cytosol	A > G
chr5:121364222:A:G:UTR3:SRFBP1	chr5	121364222	121364222	A	G	UTR3	SRFBP1	ENST000003	.	.	A	G	+	+	0.11111111	16,0,2,0	chr5,121364	Cytosol	A > G
chr5:121366385:A:G:intergenic:SR	chr5	121366385	121366385	A	G	intergenic	SRFBP1,LOX	dist=2071;dis	.	.	A	G	+	+	0.11904762	37,0,5,0	chr5,121366	Cytosol	A > G
chr5:121366410:A:G:intergenic:SR	chr5	121366410	121366410	A	G	intergenic	SRFBP1,LOX	dist=2096;dis	.	.	A	G	+	+	0.27272727	24,0,9,0	chr5,121366	Cytosol	A > G
chr5:121366418:A:G:intergenic:SR	chr5	121366418	121366418	A	G	intergenic	SRFBP1,LOX	dist=2104;dis	.	.	A	G	+	+	0.16326531	41,0,8,0	chr5,121366	Cytosol	A > G
chr5:121367092:A:G:intergenic:SR	chr5	121367092	121367092	A	G	intergenic	SRFBP1,LOX	dist=2778;dis	.	.	A	G	+	+	0.22727273	34,0,10,0	chr5,121367	Cytosol	A > G
chr5:121367093:A:G:intergenic:SR	chr5	121367093	121367093	A	G	intergenic	SRFBP1,LOX	dist=2779;dis	.	.	A	G	+	+	0.19565217	37,0,9,0	chr5,121367	Cytosol	A > G
chr5:121367099:A:G:intergenic:SR	chr5	121367099	121367099	A	G	intergenic	SRFBP1,LOX	dist=2785;dis	.	.	A	G	+	+	0.47169811	28,0,25,0	chr5,121367	Cytosol	A > G
chr5:121367102:A:G:intergenic:SR	chr5	121367102	121367102	A	G	intergenic	SRFBP1,LOX	dist=2788;dis	.	.	A	G	+	+	0.17307692	43,0,9,0	chr5,121367	Cytosol	A > G

chr5:121367133:A:G:intergenic:SR	chr5	121367133	121367133	A	G	intergenic	SRFBP1,LOX	dist=2819;dis	.	.	A	G	+	+	0.06849315	68,0,5,0	chr5,121367	Cytosol	A > G
chr5:121367139:A:G:intergenic:SR	chr5	121367139	121367139	A	G	intergenic	SRFBP1,LOX	dist=2825;dis	.	.	A	G	+	+	0.175	66,0,14,0	chr5,121367	Cytosol	A > G
chr5:121367140:A:G:intergenic:SR	chr5	121367140	121367140	A	G	intergenic	SRFBP1,LOX	dist=2826;dis	.	.	A	G	+	+	0.29487179	55,0,23,0	chr5,121367	Cytosol	A > G
chr5:121367142:A:G:intergenic:SR	chr5	121367142	121367142	A	G	intergenic	SRFBP1,LOX	dist=2828;dis	.	.	A	G	+	+	0.12658228	69,0,10,0	chr5,121367	Cytosol	A > G
chr5:125961040:A:G:UTR3:PHAX	chr5	125961040	125961040	A	G	UTR3	PHAX	ENST000002	.	.	A	G	+	+	0.07142857	78,0,6,0	chr5,125961	Cytosol	A > G
chr5:125961047:A:G:UTR3:PHAX	chr5	125961047	125961047	A	G	UTR3	PHAX	ENST000002	.	.	A	G	+	+	0.47916667	50,0,46,0	chr5,125961	Cytosol	A > G
chr5:125961079:A:G:UTR3:PHAX	chr5	125961079	125961079	A	G	UTR3	PHAX	ENST000002	.	.	A	G	+	+	0.1744186	142,0,30,0	chr5,125961	Cytosol	A > G
chr5:125961127:A:G:UTR3:PHAX	chr5	125961127	125961127	A	G	UTR3	PHAX	ENST000002	.	.	A	G	+	+	0.01136364	174,0,2,0	chr5,125961	Cytosol	A > G
chr5:125961130:A:G:UTR3:PHAX	chr5	125961130	125961130	A	G	UTR3	PHAX	ENST000002	.	.	A	G	+	+	0.06111111	169,0,11,0	chr5,125961	Cytosol	A > G
chr5:125961146:A:G:UTR3:PHAX	chr5	125961146	125961146	A	G	UTR3	PHAX	ENST000002	.	.	A	G	+	+	0.03418803	113,0,4,0	chr5,125961	Cytosol	A > G
chr5:125961166:A:G:UTR3:PHAX	chr5	125961166	125961166	A	G	UTR3	PHAX	ENST000002	.	.	A	G	+	+	0.36538462	99,0,57,0	chr5,125961	Cytosol	A > G
chr5:125961168:A:G:UTR3:PHAX	chr5	125961168	125961168	A	G	UTR3	PHAX	ENST000002	.	.	A	G	+	+	0.30769231	108,0,48,0	chr5,125961	Cytosol	A > G
chr5:125961169:A:G:UTR3:PHAX	chr5	125961169	125961169	A	G	UTR3	PHAX	ENST000002	.	.	A	G	+	+	0.0974026	139,0,15,0	chr5,125961	Cytosol	A > G
chr5:125961204:A:G:UTR3:PHAX	chr5	125961204	125961204	A	G	UTR3	PHAX	ENST000002	.	.	A	G	+	+	0.08264463	111,0,10,0	chr5,125961	Cytosol	A > G
chr5:125961209:A:G:UTR3:PHAX	chr5	125961209	125961209	A	G	UTR3	PHAX	ENST000002	.	.	A	G	+	+	0.10344828	130,0,15,0	chr5,125961	Cytosol	A > G
chr5:125961210:A:G:UTR3:PHAX	chr5	125961210	125961210	A	G	UTR3	PHAX	ENST000002	.	.	A	G	+	+	0.20300752	106,0,27,0	chr5,125961	Cytosol	A > G
chr5:125961239:A:G:UTR3:PHAX	chr5	125961239	125961239	A	G	UTR3	PHAX	ENST000002	.	.	A	G	+	+	0.06451613	116,0,8,0	chr5,125961	Cytosol	A > G
chr5:125961246:A:G:UTR3:PHAX	chr5	125961246	125961246	A	G	UTR3	PHAX	ENST000002	.	.	A	G	+	+	0.10869565	123,0,15,0	chr5,125961	Cytosol	A > G
chr5:125961433:A:G:UTR3:PHAX	chr5	125961433	125961433	A	G	UTR3	PHAX	ENST000002	.	.	A	G	+	+	0.07352941	126,0,10,0	chr5,125961	Cytosol	A > G
chr5:125961447:A:G:UTR3:PHAX	chr5	125961447	125961447	A	G	UTR3	PHAX	ENST000002	.	.	A	G	+	+	0.56521739	50,0,65,0	chr5,125961	Cytosol	A > G
chr5:125961455:A:G:UTR3:PHAX	chr5	125961455	125961455	A	G	UTR3	PHAX	ENST000002	.	.	A	G	+	+	0.21186441	93,0,25,0	chr5,125961	Cytosol	A > G
chr5:125961463:A:G:UTR3:PHAX	chr5	125961463	125961463	A	G	UTR3	PHAX	ENST000002	.	.	A	G	+	+	0.24637681	104,0,34,0	chr5,125961	Cytosol	A > G
chr5:125961468:A:G:UTR3:PHAX	chr5	125961468	125961468	A	G	UTR3	PHAX	ENST000002	.	.	A	G	+	+	0.17647059	126,0,27,0	chr5,125961	Cytosol	A > G
chr5:125961509:A:G:UTR3:PHAX	chr5	125961509	125961509	A	G	UTR3	PHAX	ENST000002	.	.	A	G	+	+	0.02419355	121,0,3,0	chr5,125961	Cytosol	A > G
chr5:125961510:A:G:UTR3:PHAX	chr5	125961510	125961510	A	G	UTR3	PHAX	ENST000002	.	.	A	G	+	+	0.04347826	110,0,5,0	chr5,125961	Cytosol	A > G
chr5:125961532:A:G:UTR3:PHAX	chr5	125961532	125961532	A	G	UTR3	PHAX	ENST000002	.	.	A	G	+	+	0.25242718	77,0,26,0	chr5,125961	Cytosol	A > G
chr5:125961534:A:G:UTR3:PHAX	chr5	125961534	125961534	A	G	UTR3	PHAX	ENST000002	.	.	A	G	+	+	0.14851485	86,0,15,0	chr5,125961	Cytosol	A > G
chr5:125961548:A:G:UTR3:PHAX	chr5	125961548	125961548	A	G	UTR3	PHAX	ENST000002	.	.	A	G	+	+	0.23364486	82,0,25,0	chr5,125961	Cytosol	A > G
chr5:125961554:A:G:UTR3:PHAX	chr5	125961554	125961554	A	G	UTR3	PHAX	ENST000002	.	.	A	G	+	+	0.15151515	112,0,20,0	chr5,125961	Cytosol	A > G
chr5:125961623:A:G:UTR3:PHAX	chr5	125961623	125961623	A	G	UTR3	PHAX	ENST000002	.	.	A	G	+	+	0.10843373	148,0,18,0	chr5,125961	Cytosol	A > G
chr5:125961649:A:G:UTR3:PHAX	chr5	125961649	125961649	A	G	UTR3	PHAX	ENST000002	.	.	A	G	+	+	0.23529412	130,0,40,0	chr5,125961	Cytosol	A > G
chr5:125961810:A:G:UTR3:PHAX	chr5	125961810	125961810	A	G	UTR3	PHAX	ENST000002	.	.	A	G	+	+	0.05517241	137,0,8,0	chr5,125961	Cytosol	A > G
chr5:125961816:A:G:UTR3:PHAX	chr5	125961816	125961816	A	G	UTR3	PHAX	ENST000002	.	.	A	G	+	+	0.02366864	165,0,4,0	chr5,125961	Cytosol	A > G
chr5:125962022:A:G:UTR3:PHAX	chr5	125962022	125962022	A	G	UTR3	PHAX	ENST000002	.	.	A	G	+	+	0.03351955	173,0,6,0	chr5,125962	Cytosol	A > G
chr5:125962030:A:G:UTR3:PHAX	chr5	125962030	125962030	A	G	UTR3	PHAX	ENST000002	.	.	A	G	+	+	0.16	147,0,28,0	chr5,125962	Cytosol	A > G
chr5:125962079:A:G:UTR3:PHAX	chr5	125962079	125962079	A	G	UTR3	PHAX	ENST000002	.	.	A	G	+	+	0.07317073	114,0,9,0	chr5,125962	Cytosol	A > G
chr5:125962084:A:G:UTR3:PHAX	chr5	125962084	125962084	A	G	UTR3	PHAX	ENST000002	.	.	A	G	+	+	0.04580153	125,0,6,0	chr5,125962	Cytosol	A > G
chr5:125962085:A:G:UTR3:PHAX	chr5	125962085	125962085	A	G	UTR3	PHAX	ENST000002	.	.	A	G	+	+	0.06015038	125,0,8,0	chr5,125962	Cytosol	A > G
chr5:125962086:A:G:UTR3:PHAX	chr5	125962086	125962086	A	G	UTR3	PHAX	ENST000002	.	.	A	G	+	+	0.04895105	136,0,7,0	chr5,125962	Cytosol	A > G
chr5:125962142:A:G:UTR3:PHAX	chr5	125962142	125962142	A	G	UTR3	PHAX	ENST000002	.	.	A	G	+	+	0.03030303	128,0,4,0	chr5,125962	Cytosol	A > G
chr5:125962175:A:G:UTR3:PHAX	chr5	125962175	125962175	A	G	UTR3	PHAX	ENST000002	.	.	A	G	+	+	0.30769231	54,0,24,0	chr5,125962	Cytosol	A > G
chr5:125962176:A:G:UTR3:PHAX	chr5	125962176	125962176	A	G	UTR3	PHAX	ENST000002	.	.	A	G	+	+	0.02564103	76,0,2,0	chr5,125962	Cytosol	A > G
chr5:125962308:A:G:UTR3:PHAX	chr5	125962308	125962308	A	G	UTR3	PHAX	ENST000002	.	.	A	G	+	+	0.11111111	48,0,6,0	chr5,125962	Cytosol	A > G
chr5:125962482:A:G:UTR3:PHAX	chr5	125962482	125962482	A	G	UTR3	PHAX	ENST000002	.	.	A	G	+	+	0.05785124	114,0,7,0	chr5,125962	Cytosol	A > G
chr5:125962528:A:G:UTR3:PHAX	chr5	125962528	125962528	A	G	UTR3	PHAX	ENST000002	.	.	A	G	+	+	0.16923077	54,0,11,0	chr5,125962	Cytosol	A > G
chr5:125962568:A:G:UTR3:PHAX	chr5	125962568	125962568	A	G	UTR3	PHAX	ENST000002	.	.	A	G	+	+	0.10655738	109,0,13,0	chr5,125962	Cytosol	A > G
chr5:130495621:T:C:intronic:HINT	chr5	130495621	130495621	T	C	intronic	HINT1	.	.	.	A	G	-	-	0.30434783	16,0,7,0	chr5,130495	Cytosol	A > G
chr5:130495647:T:C:intronic:HINT	chr5	130495647	130495647	T	C	intronic	HINT1	.	.	.	A	G	-	-	0.08	23,0,2,0	chr5,130495	Cytosol	A > G
chr5:130495683:T:C:intronic:HINT	chr5	130495683	130495683	T	C	intronic	HINT1	.	.	.	A	G	-	-	0.81818182	6,0,27,0	chr5,130495	Cytosol	A > G
chr5:130495709:T:C:intronic:HINT	chr5	130495709	130495709	T	C	intronic	HINT1	.	.	.	A	G	-	-	0.08571429	32,0,3,0	chr5,130495	Cytosol	A > G
chr5:130495718:T:C:intronic:HINT	chr5	130495718	130495718	T	C	intronic	HINT1	.	.	.	A	G	-	-	0.05	38,0,2,0	chr5,130495	Cytosol	A > G
chr5:130495740:T:C:intronic:HINT	chr5	130495740	130495740	T	C	intronic	HINT1	.	.	.	A	G	-	-	0.07894737	35,0,3,0	chr5,130495	Cytosol	A > G
chr5:130495760:T:C:intronic:HINT	chr5	130495760	130495760	T	C	intronic	HINT1	.	.	.	A	G	-	-	0.03921569	49,0,2,0	chr5,130495	Cytosol	A > G
chr5:130495813:T:C:intronic:HINT	chr5	130495813	130495813	T	C	intronic	HINT1	.	.	.	A	G	-	-	0.06896552	27,0,2,0	chr5,130495	Cytosol	A > G
chr5:130495835:T:C:intronic:HINT	chr5	130495835	130495835	T	C	intronic	HINT1	.	.	.	A	G	-	-	0.45833333	13,0,11,0	chr5,130495	Cytosol	A > G
chr5:130495837:T:C:intronic:HINT	chr5	130495837	130495837	T	C	intronic	HINT1	.	.	.	A	G	-	-	0.30434783	16,0,7,0	chr5,130495	Cytosol	A > G
chr5:130497071:T:C:intronic:HINT	chr5	130497071	130497071	T	C	intronic	HINT1	.	.	.	A	G	-	-	0.1875	13,0,3,0	chr5,130497	Cytosol	A > G
chr5:130497100:T:C:intronic:HINT	chr5	130497100	130497100	T	C	intronic	HINT1	.	.	.	A	G	-	-	0.34615385	17,0,9,0	chr5,130497	Cytosol	A > G
chr5:130497131:T:C:intronic:HINT	chr5	130497131	130497131	T	C	intronic	HINT1	.	.	.	A	G	-	-	0.2	20,0,5,0	chr5,130497	Cytosol	A > G
chr5:130497140:T:C:intronic:HINT	chr5	130497140	130497140	T	C	intronic	HINT1	.	.	.	A	G	-	-	0.06060606	31,0,2,0	chr5,130497	Cytosol	A > G
chr5:130497171:T:C:intronic:HINT	chr5	130497171	130497171	T	C	intronic	HINT1	.	.	.	A	G	-	-	0.06666667	28,0,2,0	chr5,130497	Cytosol	A > G
chr5:130497225:T:C:intronic:HINT	chr5	130497225	130497225	T	C	intronic	HINT1	.	.	.	A	G	-	-	0.39473684	23,0,15,0	chr5,130497	Cytosol	A > G
chr5:130497226:T:C:intronic:HINT	chr5	130497226	130497226	T	C	intronic	HINT1	.	.	.	A	G	-	-	0.15789474	32,0,6,0	chr5,130497	Cytosol	A > G
chr5:130497249:T:C:intronic:HINT	chr5	130497249	130497249	T	C	intronic	HINT1	.	.	.	A	G	-	-	0.34782609	15,0,8,0	chr5,130497	Cytosol	A > G

chr5:130497953:T:C:UTR3:HINT1	chr5	130497953	130497953	T	C	UTR3	HINT1	ENST000005	.	.	A	G	-	-	0.10169492	53,0,6,0	chr5,1304979	Cytosol	A > G
chr5:130536748:A:G:UTR3:LYRM7	chr5	130536748	130536748	A	G	UTR3	LYRM7	ENST000003	.	.	A	G	+	+	0.23809524	32,0,10,0	chr5,1305367	Cytosol	A > G
chr5:130536754:A:G:UTR3:LYRM7	chr5	130536754	130536754	A	G	UTR3	LYRM7	ENST000003	.	.	A	G	+	+	0.28571429	30,0,12,0	chr5,1305367	Cytosol	A > G
chr5:130536755:A:G:UTR3:LYRM7	chr5	130536755	130536755	A	G	UTR3	LYRM7	ENST000003	.	.	A	G	+	+	0.22727273	34,0,10,0	chr5,1305367	Cytosol	A > G
chr5:130536797:A:G:UTR3:LYRM7	chr5	130536797	130536797	A	G	UTR3	LYRM7	ENST000003	.	.	A	G	+	+	0.03921569	49,0,2,0	chr5,1305367	Cytosol	A > G
chr5:130536806:A:G:UTR3:LYRM7	chr5	130536806	130536806	A	G	UTR3	LYRM7	ENST000003	.	.	A	G	+	+	0.04081633	47,0,2,0	chr5,1305368	Cytosol	A > G
chr5:130536830:A:G:UTR3:LYRM7	chr5	130536830	130536830	A	G	UTR3	LYRM7	ENST000003	.	.	A	G	+	+	0.05882353	48,0,3,0	chr5,1305368	Cytosol	A > G
chr5:130536836:A:G:UTR3:LYRM7	chr5	130536836	130536836	A	G	UTR3	LYRM7	ENST000003	.	.	A	G	+	+	0.1	45,0,5,0	chr5,1305368	Cytosol	A > G
chr5:130536842:A:G:UTR3:LYRM7	chr5	130536842	130536842	A	G	UTR3	LYRM7	ENST000003	.	.	A	G	+	+	0.2037037	43,0,11,0	chr5,1305368	Cytosol	A > G
chr5:130536845:A:G:UTR3:LYRM7	chr5	130536845	130536845	A	G	UTR3	LYRM7	ENST000003	.	.	A	G	+	+	0.34146341	27,0,14,0	chr5,1305368	Cytosol	A > G
chr5:130536846:A:G:UTR3:LYRM7	chr5	130536846	130536846	A	G	UTR3	LYRM7	ENST000003	.	.	A	G	+	+	0.09615385	47,0,5,0	chr5,1305368	Cytosol	A > G
chr5:130536847:A:G:UTR3:LYRM7	chr5	130536847	130536847	A	G	UTR3	LYRM7	ENST000003	.	.	A	G	+	+	0.10204082	44,0,5,0	chr5,1305368	Cytosol	A > G
chr5:130536848:A:G:UTR3:LYRM7	chr5	130536848	130536848	A	G	UTR3	LYRM7	ENST000003	.	.	A	G	+	+	0.05263158	54,0,3,0	chr5,1305368	Cytosol	A > G
chr5:130536868:A:G:UTR3:LYRM7	chr5	130536868	130536868	A	G	UTR3	LYRM7	ENST000003	.	.	A	G	+	+	0.35294118	22,0,12,0	chr5,1305368	Cytosol	A > G
chr5:130536886:A:G:UTR3:LYRM7	chr5	130536886	130536886	A	G	UTR3	LYRM7	ENST000003	.	.	A	G	+	+	0.3	21,0,9,0	chr5,1305368	Cytosol	A > G
chr5:130537154:A:G:UTR3:LYRM7	chr5	130537154	130537154	A	G	UTR3	LYRM7	ENST000003	.	.	A	G	+	+	0.37931034	36,0,22,0	chr5,1305371	Cytosol	A > G
chr5:130537155:A:G:UTR3:LYRM7	chr5	130537155	130537155	A	G	UTR3	LYRM7	ENST000003	.	.	A	G	+	+	0.53333333	28,0,32,0	chr5,1305371	Cytosol	A > G
chr5:130537181:A:G:UTR3:LYRM7	chr5	130537181	130537181	A	G	UTR3	LYRM7	ENST000003	.	.	A	G	+	+	0.51612903	15,0,16,0	chr5,1305371	Cytosol	A > G
chr5:130537200:A:G:UTR3:LYRM7	chr5	130537200	130537200	A	G	UTR3	LYRM7	ENST000003	.	.	A	G	+	+	0.13157895	33,0,5,0	chr5,1305371	Cytosol	A > G
chr5:130537220:A:G:UTR3:LYRM7	chr5	130537220	130537220	A	G	UTR3	LYRM7	ENST000003	.	.	A	G	+	+	0.35135135	24,0,13,0	chr5,1305372	Cytosol	A > G
chr5:130537223:A:G:UTR3:LYRM7	chr5	130537223	130537223	A	G	UTR3	LYRM7	ENST000003	.	.	A	G	+	+	0.28571429	30,0,12,0	chr5,1305372	Cytosol	A > G
chr5:130537224:A:G:UTR3:LYRM7	chr5	130537224	130537224	A	G	UTR3	LYRM7	ENST000003	.	.	A	G	+	+	0.40909091	26,0,18,0	chr5,1305372	Cytosol	A > G
chr5:130537226:A:G:UTR3:LYRM7	chr5	130537226	130537226	A	G	UTR3	LYRM7	ENST000003	.	.	A	G	+	+	0.07142857	39,0,3,0	chr5,1305372	Cytosol	A > G
chr5:130537233:A:G:UTR3:LYRM7	chr5	130537233	130537233	A	G	UTR3	LYRM7	ENST000003	.	.	A	G	+	+	0.0952381	38,0,4,0	chr5,1305372	Cytosol	A > G
chr5:130537253:A:G:UTR3:LYRM7	chr5	130537253	130537253	A	G	UTR3	LYRM7	ENST000003	.	.	A	G	+	+	0.15909091	37,0,7,0	chr5,1305372	Cytosol	A > G
chr5:130537281:A:G:UTR3:LYRM7	chr5	130537281	130537281	A	G	UTR3	LYRM7	ENST000003	.	.	A	G	+	+	0.25	18,0,6,0	chr5,1305372	Cytosol	A > G
chr5:130537291:A:G:UTR3:LYRM7	chr5	130537291	130537291	A	G	UTR3	LYRM7	ENST000003	.	.	A	G	+	+	0.11627907	38,0,5,0	chr5,1305372	Cytosol	A > G
chr5:130537292:A:G:UTR3:LYRM7	chr5	130537292	130537292	A	G	UTR3	LYRM7	ENST000003	.	.	A	G	+	+	1	0,0,45,0	chr5,1305372	Cytosol	A > G
chr5:130537357:A:G:UTR3:LYRM7	chr5	130537357	130537357	A	G	UTR3	LYRM7	ENST000003	.	.	A	G	+	+	0.41666667	14,0,10,0	chr5,1305373	Cytosol	A > G
chr5:130537387:A:G:UTR3:LYRM7	chr5	130537387	130537387	A	G	UTR3	LYRM7	ENST000003	.	.	A	G	+	+	0.39622642	32,0,21,0	chr5,1305373	Cytosol	A > G
chr5:130537403:A:G:UTR3:LYRM7	chr5	130537403	130537403	A	G	UTR3	LYRM7	ENST000003	.	.	A	G	+	+	0.16666667	50,0,10,0	chr5,1305374	Cytosol	A > G
chr5:130537531:A:G:UTR3:LYRM7	chr5	130537531	130537531	A	G	UTR3	LYRM7	ENST000003	.	.	A	G	+	+	0.08196721	56,0,5,0	chr5,1305375	Cytosol	A > G
chr5:130537567:A:G:UTR3:LYRM7	chr5	130537567	130537567	A	G	UTR3	LYRM7	ENST000003	.	.	A	G	+	+	0.09375	58,0,6,0	chr5,1305375	Cytosol	A > G
chr5:130537602:A:G:UTR3:LYRM7	chr5	130537602	130537602	A	G	UTR3	LYRM7	ENST000003	.	.	A	G	+	+	0.02380952	82,0,2,0	chr5,1305376	Cytosol	A > G
chr5:130537644:A:G:UTR3:LYRM7	chr5	130537644	130537644	A	G	UTR3	LYRM7	ENST000003	.	.	A	G	+	+	0.08536585	75,0,7,0	chr5,1305376	Cytosol	A > G
chr5:130537652:A:G:UTR3:LYRM7	chr5	130537652	130537652	A	G	UTR3	LYRM7	ENST000003	.	.	A	G	+	+	0.22222222	63,0,18,0	chr5,1305376	Cytosol	A > G
chr5:130537654:A:G:UTR3:LYRM7	chr5	130537654	130537654	A	G	UTR3	LYRM7	ENST000003	.	.	A	G	+	+	0.40506329	47,0,32,0	chr5,1305376	Cytosol	A > G
chr5:130537656:A:G:UTR3:LYRM7	chr5	130537656	130537656	A	G	UTR3	LYRM7	ENST000003	.	.	A	G	+	+	0.67105263	25,0,51,0	chr5,1305376	Cytosol	A > G
chr5:130537661:A:G:UTR3:LYRM7	chr5	130537661	130537661	A	G	UTR3	LYRM7	ENST000003	.	.	A	G	+	+	0.92307692	6,0,72,0	chr5,1305376	Cytosol	A > G
chr5:130537666:A:G:UTR3:LYRM7	chr5	130537666	130537666	A	G	UTR3	LYRM7	ENST000003	.	.	A	G	+	+	0.95	4,0,76,0	chr5,1305376	Cytosol	A > G
chr5:130537710:A:G:UTR3:LYRM7	chr5	130537710	130537710	A	G	UTR3	LYRM7	ENST000003	.	.	A	G	+	+	0.72340426	13,0,34,0	chr5,1305377	Cytosol	A > G
chr5:130537711:A:G:UTR3:LYRM7	chr5	130537711	130537711	A	G	UTR3	LYRM7	ENST000003	.	.	A	G	+	+	0.83333333	8,0,40,0	chr5,1305377	Cytosol	A > G
chr5:130537736:A:G:UTR3:LYRM7	chr5	130537736	130537736	A	G	UTR3	LYRM7	ENST000003	.	.	A	G	+	+	0.79069767	9,0,34,0	chr5,1305377	Cytosol	A > G
chr5:130537788:A:G:UTR3:LYRM7	chr5	130537788	130537788	A	G	UTR3	LYRM7	ENST000003	.	.	A	G	+	+	0.21212121	26,0,7,0	chr5,1305377	Cytosol	A > G
chr5:130537932:A:G:UTR3:LYRM7	chr5	130537932	130537932	A	G	UTR3	LYRM7	ENST000003	.	.	A	G	+	+	0.25925926	40,0,14,0	chr5,1305379	Cytosol	A > G
chr5:130537933:A:G:UTR3:LYRM7	chr5	130537933	130537933	A	G	UTR3	LYRM7	ENST000003	.	.	A	G	+	+	0.14545455	47,0,8,0	chr5,1305379	Cytosol	A > G
chr5:130537959:A:G:UTR3:LYRM7	chr5	130537959	130537959	A	G	UTR3	LYRM7	ENST000003	.	.	A	G	+	+	0.43396226	30,0,23,0	chr5,1305379	Cytosol	A > G
chr5:130537968:A:G:UTR3:LYRM7	chr5	130537968	130537968	A	G	UTR3	LYRM7	ENST000003	.	.	A	G	+	+	0.24590164	46,0,15,0	chr5,1305379	Cytosol	A > G
chr5:130537973:A:G:UTR3:LYRM7	chr5	130537973	130537973	A	G	UTR3	LYRM7	ENST000003	.	.	A	G	+	+	0.03125	62,0,2,0	chr5,1305379	Cytosol	A > G
chr5:130537981:A:G:UTR3:LYRM7	chr5	130537981	130537981	A	G	UTR3	LYRM7	ENST000003	.	.	A	G	+	+	0.02469136	79,0,2,0	chr5,1305379	Cytosol	A > G
chr5:130537987:A:G:UTR3:LYRM7	chr5	130537987	130537987	A	G	UTR3	LYRM7	ENST000003	.	.	A	G	+	+	0.02531646	77,0,2,0	chr5,1305379	Cytosol	A > G
chr5:130537997:A:G:UTR3:LYRM7	chr5	130537997	130537997	A	G	UTR3	LYRM7	ENST000003	.	.	A	G	+	+	0.14772727	75,0,13,0	chr5,1305379	Cytosol	A > G
chr5:130537998:A:G:UTR3:LYRM7	chr5	130537998	130537998	A	G	UTR3	LYRM7	ENST000003	.	.	A	G	+	+	0.08235294	78,0,7,0	chr5,1305379	Cytosol	A > G
chr5:130538002:A:G:UTR3:LYRM7	chr5	130538002	130538002	A	G	UTR3	LYRM7	ENST000003	.	.	A	G	+	+	0.04705882	81,0,4,0	chr5,1305380	Cytosol	A > G
chr5:130538011:A:G:UTR3:LYRM7	chr5	130538011	130538011	A	G	UTR3	LYRM7	ENST000003	.	.	A	G	+	+	0.09459459	67,0,7,0	chr5,1305380	Cytosol	A > G
chr5:130538043:A:G:UTR3:LYRM7	chr5	130538043	130538043	A	G	UTR3	LYRM7	ENST000003	.	.	A	G	+	+	0.02777778	70,0,2,0	chr5,1305380	Cytosol	A > G
chr5:130538066:A:G:UTR3:LYRM7	chr5	130538066	130538066	A	G	UTR3	LYRM7	ENST000003	.	.	A	G	+	+	0.43269231	59,0,45,0	chr5,1305380	Cytosol	A > G
chr5:130538067:A:G:UTR3:LYRM7	chr5	130538067	130538067	A	G	UTR3	LYRM7	ENST000003	.	.	A	G	+	+	0.75	25,0,75,0	chr5,1305380	Cytosol	A > G
chr5:130538072:A:G:UTR3:LYRM7	chr5	130538072	130538072	A	G	UTR3	LYRM7	ENST000003	.	.	A	G	+	+	0.66666667	35,0,70,0	chr5,1305380	Cytosol	A > G
chr5:130538076:A:G:UTR3:LYRM7	chr5	130538076	130538076	A	G	UTR3	LYRM7	ENST000003	.	.	A	G	+	+	0.51401869	52,0,55,0	chr5,1305380	Cytosol	A > G
chr5:130538080:A:G:UTR3:LYRM7	chr5	130538080	130538080	A	G	UTR3	LYRM7	ENST000003	.	.	A	G	+	+	0.45833333	52,0,44,0	chr5,1305380	Cytosol	A > G
chr5:130538102:A:G:UTR3:LYRM7	chr5	130538102	130538102	A	G	UTR3	LYRM7	ENST000003	.	.	A	G	+	+	0.21212121	78,0,21,0	chr5,1305381	Cytosol	A > G
chr5:130538136:A:G:UTR3:LYRM7	chr5	130538136	130538136	A	G	UTR3	LYRM7	ENST000003	.	.	A	G	+	+	0.38636364	54,0,34,0	chr5,1305381	Cytosol	A > G
chr5:130538137:A:G:UTR3:LYRM7	chr5	130538137	130538137	A	G	UTR3	LYRM7	ENST000003	.	.	A	G	+	+	0.72222222	25,0,65,0	chr5,1305381	Cytosol	A > G

chr5:130538268:A:G:UTR3:LYRM7	chr5	130538268	130538268	A	G	UTR3	LYRM7	ENST000003	.	.	A	G	+	+	0.01869159	105,0,2,0	chr5,130538	Cytosol	A > G
chr5:130538274:A:G:UTR3:LYRM7	chr5	130538274	130538274	A	G	UTR3	LYRM7	ENST000003	.	.	A	G	+	+	0.31372549	70,0,32,0	chr5,130538	Cytosol	A > G
chr5:130538304:A:G:UTR3:LYRM7	chr5	130538304	130538304	A	G	UTR3	LYRM7	ENST000003	.	.	A	G	+	+	0.10416667	86,0,10,0	chr5,130538	Cytosol	A > G
chr5:130538305:A:G:UTR3:LYRM7	chr5	130538305	130538305	A	G	UTR3	LYRM7	ENST000003	.	.	A	G	+	+	0.42553191	54,0,40,0	chr5,130538	Cytosol	A > G
chr5:130538308:A:G:UTR3:LYRM7	chr5	130538308	130538308	A	G	UTR3	LYRM7	ENST000003	.	.	A	G	+	+	0.22340426	73,0,21,0	chr5,130538	Cytosol	A > G
chr5:130538309:A:G:UTR3:LYRM7	chr5	130538309	130538309	A	G	UTR3	LYRM7	ENST000003	.	.	A	G	+	+	0.6043956	36,0,55,0	chr5,130538	Cytosol	A > G
chr5:130538322:A:G:UTR3:LYRM7	chr5	130538322	130538322	A	G	UTR3	LYRM7	ENST000003	.	.	A	G	+	+	0.18181818	54,0,12,0	chr5,130538	Cytosol	A > G
chr5:130538330:A:G:UTR3:LYRM7	chr5	130538330	130538330	A	G	UTR3	LYRM7	ENST000003	.	.	A	G	+	+	0.42372881	34,0,25,0	chr5,130538	Cytosol	A > G
chr5:130538331:A:G:UTR3:LYRM7	chr5	130538331	130538331	A	G	UTR3	LYRM7	ENST000003	.	.	A	G	+	+	0.18965517	47,0,11,0	chr5,130538	Cytosol	A > G
chr5:130538338:A:G:UTR3:LYRM7	chr5	130538338	130538338	A	G	UTR3	LYRM7	ENST000003	.	.	A	G	+	+	0.76363636	13,0,42,0	chr5,130538	Cytosol	A > G
chr5:130538344:A:G:UTR3:LYRM7	chr5	130538344	130538344	A	G	UTR3	LYRM7	ENST000003	.	.	A	G	+	+	0.26923077	38,0,14,0	chr5,130538	Cytosol	A > G
chr5:130538362:A:G:UTR3:LYRM7	chr5	130538362	130538362	A	G	UTR3	LYRM7	ENST000003	.	.	A	G	+	+	0.0862069	53,0,5,0	chr5,130538	Cytosol	A > G
chr5:130539816:A:G:UTR3:LYRM7	chr5	130539816	130539816	A	G	UTR3	LYRM7	ENST000003	.	.	A	G	+	+	0.17808219	60,0,13,0	chr5,130539	Cytosol	A > G
chr5:130539835:A:G:UTR3:LYRM7	chr5	130539835	130539835	A	G	UTR3	LYRM7	ENST000003	.	.	A	G	+	+	0.27173913	67,0,25,0	chr5,130539	Cytosol	A > G
chr5:130539846:A:G:UTR3:LYRM7	chr5	130539846	130539846	A	G	UTR3	LYRM7	ENST000003	.	.	A	G	+	+	0.14285714	96,0,16,0	chr5,130539	Cytosol	A > G
chr5:130539861:A:G:UTR3:LYRM7	chr5	130539861	130539861	A	G	UTR3	LYRM7	ENST000003	.	.	A	G	+	+	0.02985075	130,0,4,0	chr5,130539	Cytosol	A > G
chr5:130539890:A:G:UTR3:LYRM7	chr5	130539890	130539890	A	G	UTR3	LYRM7	ENST000003	.	.	A	G	+	+	0.05925926	127,0,8,0	chr5,130539	Cytosol	A > G
chr5:130539895:A:G:UTR3:LYRM7	chr5	130539895	130539895	A	G	UTR3	LYRM7	ENST000003	.	.	A	G	+	+	0.0962963	122,0,13,0	chr5,130539	Cytosol	A > G
chr5:131698966:T:C:ncRNA_intror	chr5	131698966	131698966	T	C	ncRNA_intro	AC034220.3	.	.	.	A	G	-	-	0.33333333	4,0,2,0	chr5,131698	Cytosol	A > G
chr5:131980596:A:G:ncRNA_intro	chr5	131980596	131980596	A	G	ncRNA_intro	AC004041.2	.	.	.	A	G	+	-	0.25	18,0,6,0	chr5,131980	Cytosol	A > G
chr5:131980608:A:G:ncRNA_intro	chr5	131980608	131980608	A	G	ncRNA_intro	AC004041.2	.	.	.	A	G	+	-	0.08695652	21,0,2,0	chr5,131980	Cytosol	A > G
chr5:131981653:A:G:ncRNA_intro	chr5	131981653	131981653	A	G	ncRNA_intro	AC004041.2	.	.	.	A	G	+	-	0.66666667	2,0,4,0	chr5,131981	Cytosol	A > G
chr5:131981659:A:G:ncRNA_intro	chr5	131981659	131981659	A	G	ncRNA_intro	AC004041.2	.	.	.	A	G	+	-	1	0,0,5,0	chr5,131981	Cytosol	A > G
chr5:133729218:A:G:intergenic:UE	chr5	133729218	133729218	A	G	intergenic	UBE2B,RP11-	dist=1535;dis	.	.	A	G	+		0.6	2,0,3,0	chr5,133729	Cytosol	A > G
chr5:133737952:T:C:UTR3:CDKN2	chr5	133737952	133737952	T	C	UTR3	CDKN2AIPNL	ENST000004	.	.	A	G	-	-	0.03194888	303,0,10,0	chr5,133737	Cytosol	A > G
chr5:133737970:T:C:UTR3:CDKN2	chr5	133737970	133737970	T	C	UTR3	CDKN2AIPNL	ENST000004	.	.	A	G	-	-	0.01123596	352,0,4,0	chr5,133737	Cytosol	A > G
chr5:133738270:T:C:UTR3:CDKN2	chr5	133738270	133738270	T	C	UTR3	CDKN2AIPNL	ENST000004	.	.	A	G	-	-	0.0412844	209,0,9,0	chr5,133738	Cytosol	A > G
chr5:134078529:A:G:intronic:CAM	chr5	134078529	134078529	A	G	intronic	CAMLG	.	.	.	A	G	+	+	0.42857143	4,0,3,0	chr5,134078	Cytosol	A > G
chr5:134078635:A:G:intronic:CAM	chr5	134078635	134078635	A	G	intronic	CAMLG	.	.	.	A	G	+	+	0.4	3,0,2,0	chr5,134078	Cytosol	A > G
chr5:134078765:A:G:intronic:CAM	chr5	134078765	134078765	A	G	intronic	CAMLG	.	.	.	A	G	+	+	0.27272727	8,0,3,0	chr5,134078	Cytosol	A > G
chr5:134079280:A:G:intronic:CAM	chr5	134079280	134079280	A	G	intronic	CAMLG	.	.	.	A	G	+	+	0.25	6,0,2,0	chr5,134079	Cytosol	A > G
chr5:134079296:A:G:intronic:CAM	chr5	134079296	134079296	A	G	intronic	CAMLG	.	.	.	A	G	+	+	0.28571429	5,0,2,0	chr5,134079	Cytosol	A > G
chr5:134165394:A:G:UTR3:DDX46	chr5	134165394	134165394	A	G	UTR3	DDX46	ENST000004	.	.	A	G	+	+	0.04166667	46,0,2,0	chr5,134165	Cytosol	A > G
chr5:134165528:A:G:UTR3:DDX46	chr5	134165528	134165528	A	G	UTR3	DDX46	ENST000004	.	.	A	G	+	+	0.06521739	43,0,3,0	chr5,134165	Cytosol	A > G
chr5:134236107:A:G:UTR3:TXNDC	chr5	134236107	134236107	A	G	UTR3	TXNDC15	ENST000003	.	.	A	G	+	+	0.75	8,0,24,0	chr5,134236	Cytosol	A > G
chr5:134236108:A:G:UTR3:TXNDC	chr5	134236108	134236108	A	G	UTR3	TXNDC15	ENST000003	.	.	A	G	+	+	0.48484848	17,0,16,0	chr5,134236	Cytosol	A > G
chr5:134236113:A:G:UTR3:TXNDC	chr5	134236113	134236113	A	G	UTR3	TXNDC15	ENST000003	.	.	A	G	+	+	0.06896552	27,0,2,0	chr5,134236	Cytosol	A > G
chr5:134236129:A:G:UTR3:TXNDC	chr5	134236129	134236129	A	G	UTR3	TXNDC15	ENST000003	.	.	A	G	+	+	0.55555556	8,0,10,0	chr5,134236	Cytosol	A > G
chr5:134236178:A:G:UTR3:TXNDC	chr5	134236178	134236178	A	G	UTR3	TXNDC15	ENST000003	.	.	A	G	+	+	0.19444444	29,0,7,0	chr5,134236	Cytosol	A > G
chr5:134236188:A:G:UTR3:TXNDC	chr5	134236188	134236188	A	G	UTR3	TXNDC15	ENST000003	.	.	A	G	+	+	0.45	22,0,18,0	chr5,134236	Cytosol	A > G
chr5:134236199:A:G:UTR3:TXNDC	chr5	134236199	134236199	A	G	UTR3	TXNDC15	ENST000003	.	.	A	G	+	+	0.25641026	29,0,10,0	chr5,134236	Cytosol	A > G
chr5:134236203:A:G:UTR3:TXNDC	chr5	134236203	134236203	A	G	UTR3	TXNDC15	ENST000003	.	.	A	G	+	+	0.44186047	24,0,19,0	chr5,134236	Cytosol	A > G
chr5:134236204:A:G:UTR3:TXNDC	chr5	134236204	134236204	A	G	UTR3	TXNDC15	ENST000003	.	.	A	G	+	+	0.04761905	40,0,2,0	chr5,134236	Cytosol	A > G
chr5:134236205:A:G:UTR3:TXNDC	chr5	134236205	134236205	A	G	UTR3	TXNDC15	ENST000003	.	.	A	G	+	+	0.26666667	33,0,12,0	chr5,134236	Cytosol	A > G
chr5:134236206:A:G:UTR3:TXNDC	chr5	134236206	134236206	A	G	UTR3	TXNDC15	ENST000003	.	.	A	G	+	+	0.04	48,0,2,0	chr5,134236	Cytosol	A > G
chr5:134236218:A:G:UTR3:TXNDC	chr5	134236218	134236218	A	G	UTR3	TXNDC15	ENST000003	.	.	A	G	+	+	0.32	34,0,16,0	chr5,134236	Cytosol	A > G
chr5:134236245:A:G:UTR3:TXNDC	chr5	134236245	134236245	A	G	UTR3	TXNDC15	ENST000003	.	.	A	G	+	+	0.45098039	28,0,23,0	chr5,134236	Cytosol	A > G
chr5:134236275:A:G:UTR3:TXNDC	chr5	134236275	134236275	A	G	UTR3	TXNDC15	ENST000003	.	.	A	G	+	+	0.08510638	43,0,4,0	chr5,134236	Cytosol	A > G
chr5:134236278:A:G:UTR3:TXNDC	chr5	134236278	134236278	A	G	UTR3	TXNDC15	ENST000003	.	.	A	G	+	+	0.10416667	43,0,5,0	chr5,134236	Cytosol	A > G
chr5:134236281:A:G:UTR3:TXNDC	chr5	134236281	134236281	A	G	UTR3	TXNDC15	ENST000003	.	.	A	G	+	+	0.52	24,0,26,0	chr5,134236	Cytosol	A > G
chr5:134236286:A:G:UTR3:TXNDC	chr5	134236286	134236286	A	G	UTR3	TXNDC15	ENST000003	.	.	A	G	+	+	0.47916667	25,0,23,0	chr5,134236	Cytosol	A > G
chr5:134236287:A:G:UTR3:TXNDC	chr5	134236287	134236287	A	G	UTR3	TXNDC15	ENST000003	.	.	A	G	+	+	0.28	36,0,14,0	chr5,134236	Cytosol	A > G
chr5:134236318:A:G:UTR3:TXNDC	chr5	134236318	134236318	A	G	UTR3	TXNDC15	ENST000003	.	.	A	G	+	+	0.11267606	63,0,8,0	chr5,134236	Cytosol	A > G
chr5:134236321:A:G:UTR3:TXNDC	chr5	134236321	134236321	A	G	UTR3	TXNDC15	ENST000003	.	.	A	G	+	+	0.04411765	65,0,3,0	chr5,134236	Cytosol	A > G
chr5:134236552:A:G:UTR3:TXNDC	chr5	134236552	134236552	A	G	UTR3	TXNDC15	ENST000003	.	.	A	G	+	+	0.14285714	18,0,3,0	chr5,134236	Cytosol	A > G
chr5:134236578:A:G:UTR3:TXNDC	chr5	134236578	134236578	A	G	UTR3	TXNDC15	ENST000003	.	.	A	G	+	+	0.15789474	16,0,3,0	chr5,134236	Cytosol	A > G
chr5:134236595:A:G:UTR3:TXNDC	chr5	134236595	134236595	A	G	UTR3	TXNDC15	ENST000003	.	.	A	G	+	+	0.16666667	20,0,4,0	chr5,134236	Cytosol	A > G
chr5:134236607:A:G:UTR3:TXNDC	chr5	134236607	134236607	A	G	UTR3	TXNDC15	ENST000003	.	.	A	G	+	+	0.12195122	36,0,5,0	chr5,134236	Cytosol	A > G
chr5:134236617:A:G:UTR3:TXNDC	chr5	134236617	134236617	A	G	UTR3	TXNDC15	ENST000003	.	.	A	G	+	+	0.27906977	31,0,12,0	chr5,134236	Cytosol	A > G
chr5:134236635:A:G:UTR3:TXNDC	chr5	134236635	134236635	A	G	UTR3	TXNDC15	ENST000003	.	.	A	G	+	+	0.41666667	21,0,15,0	chr5,134236	Cytosol	A > G
chr5:134236643:A:G:UTR3:TXNDC	chr5	134236643	134236643	A	G	UTR3	TXNDC15	ENST000003	.	.	A	G	+	+	0.27027027	27,0,10,0	chr5,134236	Cytosol	A > G
chr5:134236644:A:G:UTR3:TXNDC	chr5	134236644	134236644	A	G	UTR3	TXNDC15	ENST000003	.	.	A	G	+	+	0.06818182	41,0,3,0	chr5,134236	Cytosol	A > G
chr5:134236658:A:G:UTR3:TXNDC	chr5	134236658	134236658	A	G	UTR3	TXNDC15	ENST000003	.	.	A	G	+	+	0.43589744	22,0,17,0	chr5,134236	Cytosol	A > G
chr5:134236661:A:G:UTR3:TXNDC	chr5	134236661	134236661	A	G	UTR3	TXNDC15	ENST000003	.	.	A	G	+	+	0.32432432	25,0,12,0	chr5,134236	Cytosol	A > G

chr5:134236698:A:G:UTR3:TXNDC	chr5	134236698	134236698	A	G	UTR3	TXNDC15	ENST000003	.	.	A	G	+	+	0.2	16,0,4,0	chr5,134236	Cytosol	A > G
chr5:134236712:A:G:UTR3:TXNDC	chr5	134236712	134236712	A	G	UTR3	TXNDC15	ENST000003	.	.	A	G	+	+	0.22222222	14,0,4,0	chr5,134236	Cytosol	A > G
chr5:134236730:A:G:UTR3:TXNDC	chr5	134236730	134236730	A	G	UTR3	TXNDC15	ENST000003	.	.	A	G	+	+	0.68181818	7,0,15,0	chr5,134236	Cytosol	A > G
chr5:134236739:A:G:UTR3:TXNDC	chr5	134236739	134236739	A	G	UTR3	TXNDC15	ENST000003	.	.	A	G	+	+	0.15	17,0,3,0	chr5,134236	Cytosol	A > G
chr5:134236740:A:G:UTR3:TXNDC	chr5	134236740	134236740	A	G	UTR3	TXNDC15	ENST000003	.	.	A	G	+	+	0.57142857	9,0,12,0	chr5,134236	Cytosol	A > G
chr5:134236752:A:G:UTR3:TXNDC	chr5	134236752	134236752	A	G	UTR3	TXNDC15	ENST000003	.	.	A	G	+	+	0.8	4,0,16,0	chr5,134236	Cytosol	A > G
chr5:134299649:A:G:intronic:PCBI	chr5	134299649	134299649	A	G	intronic	PCBD2	.	.	.	A	G	+	+	0.6	2,0,3,0	chr5,134299	Cytosol	A > G
chr5:138618538:A:G:intronic:MAT	chr5	138618538	138618538	A	G	intronic	MATR3	.	.	.	A	G	+	-+	0.91970803	11,0,126,0	chr5,138618	Cytosol	A > G
chr5:138618584:A:G:intronic:MAT	chr5	138618584	138618584	A	G	intronic	MATR3	.	.	.	A	G	+	-+	0.05882353	144,0,9,0	chr5,138618	Cytosol	A > G
chr5:138618619:A:G:intronic:MAT	chr5	138618619	138618619	A	G	intronic	MATR3	.	.	.	A	G	+	-+	0.45054945	50,0,41,0	chr5,138618	Cytosol	A > G
chr5:138618620:A:G:intronic:MAT	chr5	138618620	138618620	A	G	intronic	MATR3	.	.	.	A	G	+	-+	0.44318182	49,0,39,0	chr5,138618	Cytosol	A > G
chr5:138620176:A:G:intronic:MAT	chr5	138620176	138620176	A	G	intronic	MATR3	.	.	.	A	G	+	-+	0.08571429	126,0,12,2	chr5,138620	Cytosol	A > G
chr5:138620177:A:G:intronic:MAT	chr5	138620177	138620177	A	G	intronic	MATR3	.	.	.	A	G	+	-+	0.0962963	122,0,13,0	chr5,138620	Cytosol	A > G
chr5:138620183:A:G:intronic:MAT	chr5	138620183	138620183	A	G	intronic	MATR3	.	.	.	A	G	+	-+	0.1221374	115,0,16,0	chr5,138620	Cytosol	A > G
chr5:138620209:A:G:intronic:MAT	chr5	138620209	138620209	A	G	intronic	MATR3	.	.	.	A	G	+	-+	0.09473684	86,0,9,0	chr5,138620	Cytosol	A > G
chr5:138620217:A:G:intronic:MAT	chr5	138620217	138620217	A	G	intronic	MATR3	.	.	.	A	G	+	-+	0.10091743	98,0,11,0	chr5,138620	Cytosol	A > G
chr5:138620218:A:G:intronic:MAT	chr5	138620218	138620218	A	G	intronic	MATR3	.	.	.	A	G	+	-+	0.13207547	92,0,14,0	chr5,138620	Cytosol	A > G
chr5:138620224:A:G:intronic:MAT	chr5	138620224	138620224	A	G	intronic	MATR3	.	.	.	A	G	+	-+	0.22916667	74,0,22,0	chr5,138620	Cytosol	A > G
chr5:138620225:A:G:intronic:MAT	chr5	138620225	138620225	A	G	intronic	MATR3	.	.	.	A	G	+	-+	0.94736842	5,0,90,0	chr5,138620	Cytosol	A > G
chr5:138620229:A:G:intronic:MAT	chr5	138620229	138620229	A	G	intronic	MATR3	.	.	.	A	G	+	-+	0.41052632	56,0,39,0	chr5,138620	Cytosol	A > G
chr5:145137359:T:C:intergenic:NC	chr5	145137359	145137359	T	C	intergenic	NONE,PRELI	dist=NONE;d	.	.	A	G	-	-	0.54545455	5,0,6,0	chr5,145137	Cytosol	A > G
chr5:148872833:T:C:UTR3:CSNK1A	chr5	148872833	148872833	T	C	UTR3	CSNK1A1	ENST000002	.	.	A	G	-	-	0.02898551	67,0,2,0	chr5,148872	Cytosol	A > G
chr5:148872853:T:C:UTR3:CSNK1A	chr5	148872853	148872853	T	C	UTR3	CSNK1A1	ENST000002	.	.	A	G	-	-	0.03125	62,0,2,0	chr5,148872	Cytosol	A > G
chr5:148927661:T:C:intronic:CSNK	chr5	148927661	148927661	T	C	intronic	CSNK1A1	.	.	.	A	G	-	-	0.28571429	5,0,2,0	chr5,148927	Cytosol	A > G
chr5:148927685:T:C:intronic:CSNK	chr5	148927685	148927685	T	C	intronic	CSNK1A1	.	.	.	A	G	-	-	0.28571429	5,0,2,0	chr5,148927	Cytosol	A > G
chr5:148927701:T:C:intronic:CSNK	chr5	148927701	148927701	T	C	intronic	CSNK1A1	.	.	.	A	G	-	-	0.57142857	3,0,4,0	chr5,148927	Cytosol	A > G
chr5:149363214:A:G:UTR3:SLC26A	chr5	149363214	149363214	A	G	UTR3	SLC26A2	ENST000002	.	.	A	G	+	+	0.496	63,0,62,0	chr5,149363	Cytosol	A > G
chr5:149363218:A:G:UTR3:SLC26A	chr5	149363218	149363218	A	G	UTR3	SLC26A2	ENST000002	.	.	A	G	+	+	0.27777778	91,0,35,0	chr5,149363	Cytosol	A > G
chr5:149363273:A:G:UTR3:SLC26A	chr5	149363273	149363273	A	G	UTR3	SLC26A2	ENST000002	.	.	A	G	+	+	0.0546875	121,0,7,0	chr5,149363	Cytosol	A > G
chr5:149363330:A:G:UTR3:SLC26A	chr5	149363330	149363330	A	G	UTR3	SLC26A2	ENST000002	.	.	A	G	+	+	0.11594203	61,0,8,0	chr5,149363	Cytosol	A > G
chr5:149363392:A:G:UTR3:SLC26A	chr5	149363392	149363392	A	G	UTR3	SLC26A2	ENST000002	.	.	A	G	+	+	0.376	78,0,47,0	chr5,149363	Cytosol	A > G
chr5:149363414:A:G:UTR3:SLC26A	chr5	149363414	149363414	A	G	UTR3	SLC26A2	ENST000002	.	.	A	G	+	+	0.28368794	101,0,40,0	chr5,149363	Cytosol	A > G
chr5:149363416:A:G:UTR3:SLC26A	chr5	149363416	149363416	A	G	UTR3	SLC26A2	ENST000002	.	.	A	G	+	+	0.02597403	150,0,4,0	chr5,149363	Cytosol	A > G
chr5:149363967:A:G:UTR3:SLC26A	chr5	149363967	149363967	A	G	UTR3	SLC26A2	ENST000002	.	.	A	G	+	+	0.42105263	44,0,32,0	chr5,149363	Cytosol	A > G
chr5:149363968:A:G:UTR3:SLC26A	chr5	149363968	149363968	A	G	UTR3	SLC26A2	ENST000002	.	.	A	G	+	+	0.32	51,0,24,0	chr5,149363	Cytosol	A > G
chr5:149364103:A:G:UTR3:SLC26A	chr5	149364103	149364103	A	G	UTR3	SLC26A2	ENST000002	.	.	A	G	+	+	0.12345679	71,0,10,0	chr5,149364	Cytosol	A > G
chr5:149364145:A:G:UTR3:SLC26A	chr5	149364145	149364145	A	G	UTR3	SLC26A2	ENST000002	.	.	A	G	+	+	0.16049383	68,0,13,0	chr5,149364	Cytosol	A > G
chr5:150647737:A:G:UTR3:GM2A	chr5	150647737	150647737	A	G	UTR3	GM2A	ENST000003	.	.	A	G	+	+	0.13571429	121,0,19,0	chr5,150647	Cytosol	A > G
chr5:150647739:A:G:UTR3:GM2A	chr5	150647739	150647739	A	G	UTR3	GM2A	ENST000003	.	.	A	G	+	+	0.06034483	109,0,7,0	chr5,150647	Cytosol	A > G
chr5:150647741:A:G:UTR3:GM2A	chr5	150647741	150647741	A	G	UTR3	GM2A	ENST000003	.	.	A	G	+	+	0.06569343	128,0,9,0	chr5,150647	Cytosol	A > G
chr5:150647744:A:G:UTR3:GM2A	chr5	150647744	150647744	A	G	UTR3	GM2A	ENST000003	.	.	A	G	+	+	0.25185185	101,0,34,0	chr5,150647	Cytosol	A > G
chr5:150647749:A:G:UTR3:GM2A	chr5	150647749	150647749	A	G	UTR3	GM2A	ENST000003	.	.	A	G	+	+	0.04494382	85,0,4,0	chr5,150647	Cytosol	A > G
chr5:150647817:A:G:UTR3:GM2A	chr5	150647817	150647817	A	G	UTR3	GM2A	ENST000003	.	.	A	G	+	+	0.03571429	135,0,5,0	chr5,150647	Cytosol	A > G
chr5:150647820:A:G:UTR3:GM2A	chr5	150647820	150647820	A	G	UTR3	GM2A	ENST000003	.	.	A	G	+	+	0.02564103	152,0,4,0	chr5,150647	Cytosol	A > G
chr5:150647856:A:G:UTR3:GM2A	chr5	150647856	150647856	A	G	UTR3	GM2A	ENST000003	.	.	A	G	+	+	0.021875	313,0,7,0	chr5,150647	Cytosol	A > G
chr5:150647896:A:G:UTR3:GM2A	chr5	150647896	150647896	A	G	UTR3	GM2A	ENST000003	.	.	A	G	+	+	0.03041825	255,0,8,0	chr5,150647	Cytosol	A > G
chr5:150647899:A:G:UTR3:GM2A	chr5	150647899	150647899	A	G	UTR3	GM2A	ENST000003	.	.	A	G	+	+	0.03797468	228,0,9,0	chr5,150647	Cytosol	A > G
chr5:150651380:A:G:intergenic:GI	chr5	150651380	150651380	A	G	intergenic	GM2A,SLC36	dist=1379;dis	.	.	A	G	+		0.33333333	4,0,2,0	chr5,150651	Cytosol	A > G
chr5:150651426:A:G:intergenic:GI	chr5	150651426	150651426	A	G	intergenic	GM2A,SLC36	dist=1425;dis	.	.	A	G	+		0.33333333	6,0,3,0	chr5,150651	Cytosol	A > G
chr5:150651434:A:G:intergenic:GI	chr5	150651434	150651434	A	G	intergenic	GM2A,SLC36	dist=1433;dis	.	.	A	G	+		0.41666667	7,0,5,0	chr5,150651	Cytosol	A > G
chr5:150651435:A:G:intergenic:GI	chr5	150651435	150651435	A	G	intergenic	GM2A,SLC36	dist=1434;dis	.	.	A	G	+		0.41666667	7,0,5,0	chr5,150651	Cytosol	A > G
chr5:150651450:A:G:intergenic:GI	chr5	150651450	150651450	A	G	intergenic	GM2A,SLC36	dist=1449;dis	.	.	A	G	+		0.54545455	5,0,6,0	chr5,150651	Cytosol	A > G
chr5:150651458:A:G:intergenic:GI	chr5	150651458	150651458	A	G	intergenic	GM2A,SLC36	dist=1457;dis	.	.	A	G	+		0.18181818	9,0,2,0	chr5,150651	Cytosol	A > G
chr5:150651475:A:G:intergenic:GI	chr5	150651475	150651475	A	G	intergenic	GM2A,SLC36	dist=1474;dis	.	.	A	G	+		0.2	8,0,2,0	chr5,150651	Cytosol	A > G
chr5:150651490:A:G:intergenic:GI	chr5	150651490	150651490	A	G	intergenic	GM2A,SLC36	dist=1489;dis	.	.	A	G	+		0.25	6,0,2,0	chr5,150651	Cytosol	A > G
chr5:150651494:A:G:intergenic:GI	chr5	150651494	150651494	A	G	intergenic	GM2A,SLC36	dist=1493;dis	.	.	A	G	+		0.375	5,0,3,0	chr5,150651	Cytosol	A > G
chr5:150651516:A:G:intergenic:GI	chr5	150651516	150651516	A	G	intergenic	GM2A,SLC36	dist=1515;dis	.	.	A	G	+		0.21428571	11,0,3,0	chr5,150651	Cytosol	A > G
chr5:150651517:A:G:intergenic:GI	chr5	150651517	150651517	A	G	intergenic	GM2A,SLC36	dist=1516;dis	.	.	A	G	+		0.69230769	4,0,9,0	chr5,150651	Cytosol	A > G
chr5:150651522:A:G:intergenic:GI	chr5	150651522	150651522	A	G	intergenic	GM2A,SLC36	dist=1521;dis	.	.	A	G	+		0.25	9,0,3,0	chr5,150651	Cytosol	A > G
chr5:150651526:A:G:intergenic:GI	chr5	150651526	150651526	A	G	intergenic	GM2A,SLC36	dist=1525;dis	.	.	A	G	+		0.75	3,0,9,0	chr5,150651	Cytosol	A > G
chr5:150651549:A:G:intergenic:GI	chr5	150651549	150651549	A	G	intergenic	GM2A,SLC36	dist=1548;dis	.	.	A	G	+		0.1875	13,0,3,0	chr5,150651	Cytosol	A > G
chr5:150651557:A:G:intergenic:GI	chr5	150651557	150651557	A	G	intergenic	GM2A,SLC36	dist=1556;dis	.	.	A	G	+		0.1875	13,0,3,0	chr5,150651	Cytosol	A > G
chr5:156904950:T:C:UTR3:ADAM1	chr5	156904950	156904950	T	C	UTR3	ADAM19	ENST000003	.	.	A	G	-	-	0.35294118	11,0,6,0	chr5,156904	Cytosol	A > G
chr5:156905396:T:C:UTR3:ADAM1	chr5	156905396	156905396	T	C	UTR3	ADAM19	ENST000003	.	.	A	G	-	-	0.25	9,0,3,0	chr5,156905	Cytosol	A > G

chr5:156905560:T:C:UTR3:ADAM1	chr5	156905560	156905560	T	C	UTR3	ADAM19	ENST000003	.	.	A	G	-	-	0.35714286	9,0,5,0	chr5,156905	Cytosol	A > G
chr5:157185192:A:G:UTR3:LSM11	chr5	157185192	157185192	A	G	UTR3	LSM11	ENST000002	.	.	A	G	+	+	0.1875	13,0,3,0	chr5,157185	Cytosol	A > G
chr5:157185216:A:G:UTR3:LSM11	chr5	157185216	157185216	A	G	UTR3	LSM11	ENST000002	.	.	A	G	+	+	0.6	4,0,6,0	chr5,157185	Cytosol	A > G
chr5:157185297:A:G:UTR3:LSM11	chr5	157185297	157185297	A	G	UTR3	LSM11	ENST000002	.	.	A	G	+	+	0.55555556	4,0,5,0	chr5,157185	Cytosol	A > G
chr5:157185319:A:G:UTR3:LSM11	chr5	157185319	157185319	A	G	UTR3	LSM11	ENST000002	.	.	A	G	+	+	0.8	1,0,4,0	chr5,157185	Cytosol	A > G
chr5:159377515:T:C:intronic:ADRA	chr5	159377515	159377515	T	C	intronic	ADRA1B	.	.	.	A	G	-	+-	1	0,0,6,0	chr5,159377	Cytosol	A > G
chr5:170836397:A:G:intronic:NPM	chr5	170836397	170836397	A	G	intronic	NPM1	.	.	.	A	G	+	+	0.5	3,0,3,0	chr5,170836	Cytosol	A > G
chr5:170836400:A:G:intronic:NPM	chr5	170836400	170836400	A	G	intronic	NPM1	.	.	.	A	G	+	+	0.5	3,0,3,0	chr5,170836	Cytosol	A > G
chr5:170836462:A:G:intronic:NPM	chr5	170836462	170836462	A	G	intronic	NPM1	.	.	.	A	G	+	+	0.4	3,0,2,0	chr5,170836	Cytosol	A > G
chr5:172374378:A:G:intronic:ERGI	chr5	172374378	172374378	A	G	intronic	ERGIC1	.	.	.	A	G	+	+	0.2	8,0,2,0	chr5,172374	Cytosol	A > G
chr5:172374401:A:G:intronic:ERGI	chr5	172374401	172374401	A	G	intronic	ERGIC1	.	.	.	A	G	+	+	0.16666667	10,0,2,0	chr5,172374	Cytosol	A > G
chr5:174955089:A:G:UTR3:SFXN1	chr5	174955089	174955089	A	G	UTR3	SFXN1	ENST000003	.	.	A	G	+	+	0.3015873	88,0,38,0	chr5,174955	Cytosol	A > G
chr5:174955100:A:G:UTR3:SFXN1	chr5	174955100	174955100	A	G	UTR3	SFXN1	ENST000003	.	.	A	G	+	+	0.07843137	141,0,12,0	chr5,174955	Cytosol	A > G
chr5:174955186:A:G:UTR3:SFXN1	chr5	174955186	174955186	A	G	UTR3	SFXN1	ENST000003	.	.	A	G	+	+	0.03571429	243,0,9,0	chr5,174955	Cytosol	A > G
chr5:174955189:A:G:UTR3:SFXN1	chr5	174955189	174955189	A	G	UTR3	SFXN1	ENST000003	.	.	A	G	+	+	0.05	209,0,11,0	chr5,174955	Cytosol	A > G
chr5:174956085:A:G:UTR3:SFXN1	chr5	174956085	174956085	A	G	UTR3	SFXN1	ENST000003	.	.	A	G	+	+	0.21052632	15,0,4,0	chr5,174956	Cytosol	A > G
chr5:174956178:A:G:UTR3:SFXN1	chr5	174956178	174956178	A	G	UTR3	SFXN1	ENST000003	.	.	A	G	+	+	0.19402985	54,0,13,0	chr5,174956	Cytosol	A > G
chr5:174956181:A:G:UTR3:SFXN1	chr5	174956181	174956181	A	G	UTR3	SFXN1	ENST000003	.	.	A	G	+	+	0.11267606	63,0,8,0	chr5,174956	Cytosol	A > G
chr5:174956182:A:G:UTR3:SFXN1	chr5	174956182	174956182	A	G	UTR3	SFXN1	ENST000003	.	.	A	G	+	+	0.09859155	64,0,7,0	chr5,174956	Cytosol	A > G
chr5:174956187:A:G:UTR3:SFXN1	chr5	174956187	174956187	A	G	UTR3	SFXN1	ENST000003	.	.	A	G	+	+	0.14814815	69,0,12,0	chr5,174956	Cytosol	A > G
chr5:174956218:A:G:UTR3:SFXN1	chr5	174956218	174956218	A	G	UTR3	SFXN1	ENST000003	.	.	A	G	+	+	0.20560748	85,0,22,0	chr5,174956	Cytosol	A > G
chr5:174956220:A:G:UTR3:SFXN1	chr5	174956220	174956220	A	G	UTR3	SFXN1	ENST000003	.	.	A	G	+	+	0.15966387	100,0,19,0	chr5,174956	Cytosol	A > G
chr5:174956221:A:G:UTR3:SFXN1	chr5	174956221	174956221	A	G	UTR3	SFXN1	ENST000003	.	.	A	G	+	+	0.14166667	103,0,17,0	chr5,174956	Cytosol	A > G
chr5:174956230:A:G:UTR3:SFXN1	chr5	174956230	174956230	A	G	UTR3	SFXN1	ENST000003	.	.	A	G	+	+	0.08064516	114,0,10,0	chr5,174956	Cytosol	A > G
chr5:174956261:A:G:UTR3:SFXN1	chr5	174956261	174956261	A	G	UTR3	SFXN1	ENST000003	.	.	A	G	+	+	0.06504065	115,0,8,0	chr5,174956	Cytosol	A > G
chr5:174956286:A:G:UTR3:SFXN1	chr5	174956286	174956286	A	G	UTR3	SFXN1	ENST000003	.	.	A	G	+	+	0.09708738	93,0,10,0	chr5,174956	Cytosol	A > G
chr5:174956290:A:G:UTR3:SFXN1	chr5	174956290	174956290	A	G	UTR3	SFXN1	ENST000003	.	.	A	G	+	+	0.075	111,0,9,0	chr5,174956	Cytosol	A > G
chr5:174956331:A:G:UTR3:SFXN1	chr5	174956331	174956331	A	G	UTR3	SFXN1	ENST000003	.	.	A	G	+	+	0.17365269	138,0,29,0	chr5,174956	Cytosol	A > G
chr5:174956332:A:G:UTR3:SFXN1	chr5	174956332	174956332	A	G	UTR3	SFXN1	ENST000003	.	.	A	G	+	+	0.08938547	163,0,16,0	chr5,174956	Cytosol	A > G
chr5:175735557:G:A:intronic:SIMC	chr5	175735557	175735557	G	A	intronic	SIMC1	.	.	.	G	A	+	+	1	7,0,0,0	chr5,175735	Cytosol	G > A
chr5:175803109:A:G:UTR3:ARL10	chr5	175803109	175803109	A	G	UTR3	ARL10	ENST000003	.	.	A	G	+	+	0.15	17,0,3,0	chr5,175803	Cytosol	A > G
chr5:175803179:A:G:UTR3:ARL10	chr5	175803179	175803179	A	G	UTR3	ARL10	ENST000003	.	.	A	G	+	+	0.0952381	19,0,2,0	chr5,175803	Cytosol	A > G
chr5:175803181:A:G:UTR3:ARL10	chr5	175803181	175803181	A	G	UTR3	ARL10	ENST000003	.	.	A	G	+	+	0.68181818	7,0,15,0	chr5,175803	Cytosol	A > G
chr5:175803234:A:G:UTR3:ARL10	chr5	175803234	175803234	A	G	UTR3	ARL10	ENST000003	.	.	A	G	+	+	0.08571429	32,0,3,0	chr5,175803	Cytosol	A > G
chr5:175803284:A:G:UTR3:ARL10	chr5	175803284	175803284	A	G	UTR3	ARL10	ENST000003	.	.	A	G	+	+	0.26666667	11,0,4,0	chr5,175803	Cytosol	A > G
chr5:175803285:A:G:UTR3:ARL10	chr5	175803285	175803285	A	G	UTR3	ARL10	ENST000003	.	.	A	G	+	+	0.11764706	15,0,2,0	chr5,175803	Cytosol	A > G
chr5:175804797:A:G:UTR3:ARL10	chr5	175804797	175804797	A	G	UTR3	ARL10	ENST000003	.	.	A	G	+	+	0.08	23,0,2,0	chr5,175804	Cytosol	A > G
chr5:175806241:A:G:UTR3:ARL10	chr5	175806241	175806241	A	G	UTR3	ARL10	ENST000003	.	.	A	G	+	+	0.85714286	1,0,6,0	chr5,175806	Cytosol	A > G
chr5:175806251:A:G:UTR3:ARL10	chr5	175806251	175806251	A	G	UTR3	ARL10	ENST000003	.	.	A	G	+	+	0.25	6,0,2,0	chr5,175806	Cytosol	A > G
chr5:175806265:A:G:UTR3:ARL10	chr5	175806265	175806265	A	G	UTR3	ARL10	ENST000003	.	.	A	G	+	+	0.57142857	3,0,4,0	chr5,175806	Cytosol	A > G
chr5:175806301:A:G:UTR3:ARL10	chr5	175806301	175806301	A	G	UTR3	ARL10	ENST000003	.	.	A	G	+	+	0.75	2,0,6,0	chr5,175806	Cytosol	A > G
chr5:175806311:A:G:UTR3:ARL10	chr5	175806311	175806311	A	G	UTR3	ARL10	ENST000003	.	.	A	G	+	+	0.33333333	4,0,2,0	chr5,175806	Cytosol	A > G
chr5:175806354:A:G:UTR3:ARL10	chr5	175806354	175806354	A	G	UTR3	ARL10	ENST000003	.	.	A	G	+	+	0.33333333	4,0,2,0	chr5,175806	Cytosol	A > G
chr5:175807971:A:G:UTR3:ARL10	chr5	175807971	175807971	A	G	UTR3	ARL10	ENST000003	.	.	A	G	+	+	0.28571429	5,0,2,0	chr5,175807	Cytosol	A > G
chr5:175808051:A:G:UTR3:ARL10	chr5	175808051	175808051	A	G	UTR3	ARL10	ENST000003	.	.	A	G	+	+	0.375	5,0,3,0	chr5,175808	Cytosol	A > G
chr5:176472404:A:G:intronic:ZNF3	chr5	176472404	176472404	A	G	intronic	ZNF346	.	.	.	A	G	+	+	0.625	3,0,5,0	chr5,176472	Cytosol	A > G
chr5:176472430:A:G:intronic:ZNF3	chr5	176472430	176472430	A	G	intronic	ZNF346	.	.	.	A	G	+	+	0.28571429	5,0,2,0	chr5,176472	Cytosol	A > G
chr5:176472467:A:G:intronic:ZNF3	chr5	176472467	176472467	A	G	intronic	ZNF346	.	.	.	A	G	+	+	0.2	8,0,2,0	chr5,176472	Cytosol	A > G
chr5:176472473:A:G:intronic:ZNF3	chr5	176472473	176472473	A	G	intronic	ZNF346	.	.	.	A	G	+	+	0.42857143	4,0,3,0	chr5,176472	Cytosol	A > G
chr5:176472495:A:G:intronic:ZNF3	chr5	176472495	176472495	A	G	intronic	ZNF346	.	.	.	A	G	+	+	0.71428571	2,0,5,0	chr5,176472	Cytosol	A > G
chr5:176472564:A:G:intronic:ZNF3	chr5	176472564	176472564	A	G	intronic	ZNF346	.	.	.	A	G	+	+	0.8	1,0,4,0	chr5,176472	Cytosol	A > G
chr5:176473223:A:G:intronic:ZNF3	chr5	176473223	176473223	A	G	intronic	ZNF346	.	.	.	A	G	+	+	0.41666667	7,0,5,0	chr5,176473	Cytosol	A > G
chr5:176496267:A:G:intronic:ZNF3	chr5	176496267	176496267	A	G	intronic	ZNF346	.	.	.	A	G	+	+	0.8	1,0,4,0	chr5,176496	Cytosol	A > G
chr5:176496563:A:G:intronic:ZNF3	chr5	176496563	176496563	A	G	intronic	ZNF346	.	.	.	A	G	+	+	0.75	2,0,6,0	chr5,176496	Cytosol	A > G
chr5:176499589:A:G:intronic:ZNF3	chr5	176499589	176499589	A	G	intronic	ZNF346	.	.	.	A	G	+	+	0.36363636	7,0,4,0	chr5,176499	Cytosol	A > G
chr5:176500336:A:G:intronic:ZNF3	chr5	176500336	176500336	A	G	intronic	ZNF346	.	.	.	A	G	+	+	0.66666667	2,0,4,0	chr5,176500	Cytosol	A > G
chr5:179270548:A:G:intronic:C5or	chr5	179270548	179270548	A	G	intronic	C5orf45	.	.	.	A	G	+	-	0.42857143	4,0,3,0	chr5,179270	Cytosol	A > G
chr6:3020753:A:G:ncRNA_exonic:I	chr6	3020753	3020753	A	G	ncRNA_exon	RP1-90J20.1	.	.	.	A	G	+	+-	0.64285714	5,0,9,0	chr6,302075	Cytosol	A > G
chr6:3021285:A:G:ncRNA_exonic:I	chr6	3021285	3021285	A	G	ncRNA_exon	RP1-90J20.1	.	.	.	A	G	+	+-	0.33333333	4,0,2,0	chr6,302128	Cytosol	A > G
chr6:3021318:A:G:ncRNA_exonic:I	chr6	3021318	3021318	A	G	ncRNA_exon	RP1-90J20.1	.	.	.	A	G	+	+-	0.66666667	2,0,4,0	chr6,302131	Cytosol	A > G
chr6:3021322:A:G:ncRNA_exonic:I	chr6	3021322	3021322	A	G	ncRNA_exon	RP1-90J20.1	.	.	.	A	G	+	+-	0.33333333	4,0,2,0	chr6,302132	Cytosol	A > G
chr6:3021325:A:G:ncRNA_exonic:I	chr6	3021325	3021325	A	G	ncRNA_exon	RP1-90J20.1	.	.	.	A	G	+	+-	0.83333333	1,0,5,0	chr6,302132	Cytosol	A > G
chr6:3021331:A:G:ncRNA_exonic:I	chr6	3021331	3021331	A	G	ncRNA_exon	RP1-90J20.1	.	.	.	A	G	+	+-	0.66666667	2,0,4,0	chr6,302133	Cytosol	A > G
chr6:3021393:A:G:ncRNA_exonic:I	chr6	3021393	3021393	A	G	ncRNA_exon	RP1-90J20.1	.	.	.	A	G	+	+-	0.5	3,0,3,0	chr6,302139	Cytosol	A > G

chr6:3021403:A:G:ncRNA_exonic:	chr6	3021403	3021403	A	G	ncRNA_exon	RP1-90J20.1	.	.	.	A	G	+	+-	0.4	3,0,2,0	chr6,302140	Cytosol	A > G
chr6:3022197:A:G:ncRNA_exonic:	chr6	3022197	3022197	A	G	ncRNA_exon	RP1-90J20.1	.	.	.	A	G	+	+-	0.33333333	4,0,2,0	chr6,302219	Cytosol	A > G
chr6:5047075:A:G:ncRNA_intronic	chr6	5047075	5047075	A	G	ncRNA_intro	RP11-428J1.	.	.	.	A	G	+	+-	0.5	3,0,3,0	chr6,504707	Cytosol	A > G
chr6:5047308:A:G:ncRNA_intronic	chr6	5047308	5047308	A	G	ncRNA_intro	RP11-428J1.	.	.	.	A	G	+	+-	0.66666667	2,0,4,0	chr6,504730	Cytosol	A > G
chr6:7339520:C:T:intronic:CAGE1	chr6	7339520	7339520	C	T	intronic	CAGE1	.	.	.	G	A	-	-	1	7,0,0,0	chr6,733951	Cytosol	G > A
chr6:7339534:C:T:intronic:CAGE1	chr6	7339534	7339534	C	T	intronic	CAGE1	.	.	.	G	A	-	-	1	38,0,0,0	chr6,733953	Cytosol	G > A
chr6:8013282:T:C:intronic:BLOC1S	chr6	8013282	8013282	T	C	intronic	BLOC1S5-TX	.	.	.	A	G	-	-	0.33333333	6,0,3,0	chr6,801328	Cytosol	A > G
chr6:13788200:T:C:UTR3:MCUR1	chr6	13788200	13788200	T	C	UTR3	MCUR1	ENST000003	.	.	A	G	-	-	0.13793103	25,0,4,0	chr6,137881	Cytosol	A > G
chr6:13788212:T:C:UTR3:MCUR1	chr6	13788212	13788212	T	C	UTR3	MCUR1	ENST000003	.	.	A	G	-	-	0.04347826	44,0,2,0	chr6,137882	Cytosol	A > G
chr6:13788220:T:C:UTR3:MCUR1	chr6	13788220	13788220	T	C	UTR3	MCUR1	ENST000003	.	.	A	G	-	-	0.09433962	48,0,5,0	chr6,137882	Cytosol	A > G
chr6:13788222:T:C:UTR3:MCUR1	chr6	13788222	13788222	T	C	UTR3	MCUR1	ENST000003	.	.	A	G	-	-	0.0754717	49,0,4,0	chr6,137882	Cytosol	A > G
chr6:13788253:T:C:UTR3:MCUR1	chr6	13788253	13788253	T	C	UTR3	MCUR1	ENST000003	.	.	A	G	-	-	0.24444444	34,0,11,0	chr6,137882	Cytosol	A > G
chr6:13788288:T:C:UTR3:MCUR1	chr6	13788288	13788288	T	C	UTR3	MCUR1	ENST000003	.	.	A	G	-	-	0.11111111	40,0,5,0	chr6,137882	Cytosol	A > G
chr6:13788292:T:C:UTR3:MCUR1	chr6	13788292	13788292	T	C	UTR3	MCUR1	ENST000003	.	.	A	G	-	-	0.14893617	40,0,7,0	chr6,137882	Cytosol	A > G
chr6:13788293:T:C:UTR3:MCUR1	chr6	13788293	13788293	T	C	UTR3	MCUR1	ENST000003	.	.	A	G	-	-	0.08333333	44,0,4,0	chr6,137882	Cytosol	A > G
chr6:13788294:T:C:UTR3:MCUR1	chr6	13788294	13788294	T	C	UTR3	MCUR1	ENST000003	.	.	A	G	-	-	0.1627907	36,0,7,0	chr6,137882	Cytosol	A > G
chr6:13788297:T:C:UTR3:MCUR1	chr6	13788297	13788297	T	C	UTR3	MCUR1	ENST000003	.	.	A	G	-	-	0.16981132	44,0,9,0	chr6,137882	Cytosol	A > G
chr6:13788342:T:C:UTR3:MCUR1	chr6	13788342	13788342	T	C	UTR3	MCUR1	ENST000003	.	.	A	G	-	-	0.09589041	66,0,7,0	chr6,137883	Cytosol	A > G
chr6:13788361:T:C:UTR3:MCUR1	chr6	13788361	13788361	T	C	UTR3	MCUR1	ENST000003	.	.	A	G	-	-	0.10526316	51,0,6,0	chr6,137883	Cytosol	A > G
chr6:13788387:T:C:UTR3:MCUR1	chr6	13788387	13788387	T	C	UTR3	MCUR1	ENST000003	.	.	A	G	-	-	0.16666667	35,0,7,0	chr6,137883	Cytosol	A > G
chr6:13789376:T:C:UTR3:MCUR1	chr6	13789376	13789376	T	C	UTR3	MCUR1	ENST000003	.	.	A	G	-	-	0.07692308	60,0,5,0	chr6,137893	Cytosol	A > G
chr6:13789387:T:C:UTR3:MCUR1	chr6	13789387	13789387	T	C	UTR3	MCUR1	ENST000003	.	.	A	G	-	-	0.35185185	35,0,19,0	chr6,137893	Cytosol	A > G
chr6:13789390:T:C:UTR3:MCUR1	chr6	13789390	13789390	T	C	UTR3	MCUR1	ENST000003	.	.	A	G	-	-	0.04444444	43,0,2,0	chr6,137893	Cytosol	A > G
chr6:13789450:T:C:UTR3:MCUR1	chr6	13789450	13789450	T	C	UTR3	MCUR1	ENST000003	.	.	A	G	-	-	0.63636364	8,0,14,0	chr6,137894	Cytosol	A > G
chr6:13789550:T:C:UTR3:MCUR1	chr6	13789550	13789550	T	C	UTR3	MCUR1	ENST000003	.	.	A	G	-	-	0.63157895	7,0,12,0	chr6,137895	Cytosol	A > G
chr6:13789551:T:C:UTR3:MCUR1	chr6	13789551	13789551	T	C	UTR3	MCUR1	ENST000003	.	.	A	G	-	-	0.38095238	13,0,8,0	chr6,137895	Cytosol	A > G
chr6:13789586:T:C:UTR3:MCUR1	chr6	13789586	13789586	T	C	UTR3	MCUR1	ENST000003	.	.	A	G	-	-	0.29411765	12,0,5,0	chr6,137895	Cytosol	A > G
chr6:18129043:T:C:UTR3:TPMT	chr6	18129043	18129043	T	C	UTR3	TPMT	ENST000003	.	.	A	G	-	-	0.61538462	5,0,8,0	chr6,181290	Cytosol	A > G
chr6:18129048:T:C:UTR3:TPMT	chr6	18129048	18129048	T	C	UTR3	TPMT	ENST000003	.	.	A	G	-	-	0.21428571	11,0,3,0	chr6,181290	Cytosol	A > G
chr6:18129088:T:C:UTR3:TPMT	chr6	18129088	18129088	T	C	UTR3	TPMT	ENST000003	.	.	A	G	-	-	0.25	6,0,2,0	chr6,181290	Cytosol	A > G
chr6:18129115:T:C:UTR3:TPMT	chr6	18129115	18129115	T	C	UTR3	TPMT	ENST000003	.	.	A	G	-	-	0.30769231	9,0,4,0	chr6,181291	Cytosol	A > G
chr6:18129123:T:C:UTR3:TPMT	chr6	18129123	18129123	T	C	UTR3	TPMT	ENST000003	.	.	A	G	-	-	0.11764706	15,0,2,0	chr6,181291	Cytosol	A > G
chr6:18129126:T:C:UTR3:TPMT	chr6	18129126	18129126	T	C	UTR3	TPMT	ENST000003	.	.	A	G	-	-	0.125	14,0,2,0	chr6,181291	Cytosol	A > G
chr6:18129132:T:C:UTR3:TPMT	chr6	18129132	18129132	T	C	UTR3	TPMT	ENST000003	.	.	A	G	-	-	0.26666667	11,0,4,0	chr6,181291	Cytosol	A > G
chr6:18129214:T:C:UTR3:TPMT	chr6	18129214	18129214	T	C	UTR3	TPMT	ENST000003	.	.	A	G	-	-	0.03896104	74,0,3,0	chr6,181292	Cytosol	A > G
chr6:18130367:T:C:UTR3:TPMT	chr6	18130367	18130367	T	C	UTR3	TPMT	ENST000003	.	.	A	G	-	-	0.01587302	124,0,2,0	chr6,181303	Cytosol	A > G
chr6:18130394:T:C:UTR3:TPMT	chr6	18130394	18130394	T	C	UTR3	TPMT	ENST000003	.	.	A	G	-	-	0.02325581	126,0,3,0	chr6,181303	Cytosol	A > G
chr6:18130404:T:C:UTR3:TPMT	chr6	18130404	18130404	T	C	UTR3	TPMT	ENST000003	.	.	A	G	-	-	0.01398601	141,0,2,0	chr6,181304	Cytosol	A > G
chr6:18130477:T:C:UTR3:TPMT	chr6	18130477	18130477	T	C	UTR3	TPMT	ENST000003	.	.	A	G	-	-	0.02366864	165,0,4,0	chr6,181304	Cytosol	A > G
chr6:20552508:A:G:intronic:CDKAI	chr6	20552508	20552508	A	G	intronic	CDKAL1	.	.	.	A	G	+	+	0.4	3,0,2,0	chr6,205525	Cytosol	A > G
chr6:24703244:A:G:UTR3:ACOT13	chr6	24703244	24703244	A	G	UTR3	ACOT13	ENST000005	.	.	A	G	+	+	0.17647059	14,0,3,0	chr6,247032	Cytosol	A > G
chr6:24710690:T:C:intronic:C6orf6	chr6	24710690	24710690	T	C	intronic	C6orf62	.	.	.	A	G	-	-	0.03703704	52,0,2,0	chr6,247106	Cytosol	A > G
chr6:26095454:A:G:UTR3:HFE	chr6	26095454	26095454	A	G	UTR3	HFE	ENST000003	.	.	A	G	+	+	0.375	10,0,6,0	chr6,260954	Cytosol	A > G
chr6:26095462:A:G:UTR3:HFE	chr6	26095462	26095462	A	G	UTR3	HFE	ENST000003	.	.	A	G	+	+	0.76923077	3,0,10,0	chr6,260954	Cytosol	A > G
chr6:26095463:A:G:UTR3:HFE	chr6	26095463	26095463	A	G	UTR3	HFE	ENST000003	.	.	A	G	+	+	0.53846154	6,0,7,0	chr6,260954	Cytosol	A > G
chr6:26095484:A:G:UTR3:HFE	chr6	26095484	26095484	A	G	UTR3	HFE	ENST000003	.	.	A	G	+	+	0.33333333	6,0,3,0	chr6,260954	Cytosol	A > G
chr6:26095486:A:G:UTR3:HFE	chr6	26095486	26095486	A	G	UTR3	HFE	ENST000003	.	.	A	G	+	+	0.22222222	7,0,2,0	chr6,260954	Cytosol	A > G
chr6:26095517:A:G:UTR3:HFE	chr6	26095517	26095517	A	G	UTR3	HFE	ENST000003	.	.	A	G	+	+	0.5	3,0,3,0	chr6,260955	Cytosol	A > G
chr6:26095566:A:G:UTR3:HFE	chr6	26095566	26095566	A	G	UTR3	HFE	ENST000003	.	.	A	G	+	+	0.25	6,0,2,0	chr6,260955	Cytosol	A > G
chr6:26095596:A:G:UTR3:HFE	chr6	26095596	26095596	A	G	UTR3	HFE	ENST000003	.	.	A	G	+	+	0.9	2,0,18,0	chr6,260955	Cytosol	A > G
chr6:26095602:A:G:UTR3:HFE	chr6	26095602	26095602	A	G	UTR3	HFE	ENST000003	.	.	A	G	+	+	0.13043478	20,0,3,0	chr6,260956	Cytosol	A > G
chr6:26095634:A:G:UTR3:HFE	chr6	26095634	26095634	A	G	UTR3	HFE	ENST000003	.	.	A	G	+	+	0.0952381	19,0,2,0	chr6,260956	Cytosol	A > G
chr6:26095637:A:G:UTR3:HFE	chr6	26095637	26095637	A	G	UTR3	HFE	ENST000003	.	.	A	G	+	+	0.2173913	18,0,5,0	chr6,260956	Cytosol	A > G
chr6:26095642:A:G:UTR3:HFE	chr6	26095642	26095642	A	G	UTR3	HFE	ENST000003	.	.	A	G	+	+	0.69230769	8,0,18,0	chr6,260956	Cytosol	A > G
chr6:26095645:A:G:UTR3:HFE	chr6	26095645	26095645	A	G	UTR3	HFE	ENST000003	.	.	A	G	+	+	0.23076923	20,0,6,0	chr6,260956	Cytosol	A > G
chr6:26095692:A:G:UTR3:HFE	chr6	26095692	26095692	A	G	UTR3	HFE	ENST000003	.	.	A	G	+	+	0.17647059	14,0,3,0	chr6,260956	Cytosol	A > G
chr6:26096462:A:G:UTR3:HFE	chr6	26096462	26096462	A	G	UTR3	HFE	ENST000003	.	.	A	G	+	+	0.89361702	5,0,42,0	chr6,260964	Cytosol	A > G
chr6:26096636:A:G:UTR3:HFE	chr6	26096636	26096636	A	G	UTR3	HFE	ENST000003	.	.	A	G	+	+	0.61538462	5,0,8,0	chr6,260966	Cytosol	A > G
chr6:26127959:A:G:intronic:HIST1	chr6	26127959	26127959	A	G	intronic	HIST1H2AC	.	.	.	A	G	+	+	0.05882353	48,0,3,0	chr6,261279	Cytosol	A > G
chr6:28199988:A:G:intronic:ZSCAN	chr6	28199988	28199988	A	G	intronic	ZSCAN9	.	.	.	A	G	+	+	0.8	1,0,4,0	chr6,281999	Cytosol	A > G
chr6:30229374:T:C:ncRNA_exonic:	chr6	30229374	30229374	T	C	ncRNA_exon	HLA-L	.	.	.	T	C	+	-+	0.8	0,4,0,1	chr6,302293	Cytosol	T > C
chr6:36458320:G:A:UTR3:KCTD20	chr6	36458320	36458320	G	A	UTR3	KCTD20	ENST000003	.	.	G	A	+	+	0.02857143	3,0,102,0	chr6,364583	Cytosol	G > A
chr6:36509825:T:C:intronic:STK38	chr6	36509825	36509825	T	C	intronic	STK38	.	.	.	A	G	-	-	0.22222222	7,0,2,0	chr6,365098	Cytosol	A > G
chr6:36511550:T:C:intronic:STK38	chr6	36511550	36511550	T	C	intronic	STK38	.	.	.	A	G	-	-	0.8	1,0,4,0	chr6,365115	Cytosol	A > G

chr6:36511557:T:C:intronic:STK38	chr6	36511557	36511557	T	C	intronic	STK38	.	.	.	A	G	-	-	1	0,0,6,0	chr6,365115	Cytosol	A > G
chr6:36512563:T:C:intronic:STK38	chr6	36512563	36512563	T	C	intronic	STK38	.	.	.	A	G	-	-	0.25	6,0,2,0	chr6,365125	Cytosol	A > G
chr6:36512567:T:C:intronic:STK38	chr6	36512567	36512567	T	C	intronic	STK38	.	.	.	A	G	-	-	0.28571429	5,0,2,0	chr6,365125	Cytosol	A > G
chr6:42045545:A:G:UTR3:TAF8	chr6	42045545	42045545	A	G	UTR3	TAF8	ENST000004	.	.	A	G	+	+	0.15384615	11,0,2,0	chr6,420455	Cytosol	A > G
chr6:42045563:A:G:UTR3:TAF8	chr6	42045563	42045563	A	G	UTR3	TAF8	ENST000004	.	.	A	G	+	+	0.125	14,0,2,0	chr6,420455	Cytosol	A > G
chr6:42045595:A:G:UTR3:TAF8	chr6	42045595	42045595	A	G	UTR3	TAF8	ENST000004	.	.	A	G	+	+	0.1	18,0,2,0	chr6,420455	Cytosol	A > G
chr6:42045677:A:G:UTR3:TAF8	chr6	42045677	42045677	A	G	UTR3	TAF8	ENST000004	.	.	A	G	+	+	0.13333333	13,0,2,0	chr6,420456	Cytosol	A > G
chr6:42045694:A:G:UTR3:TAF8	chr6	42045694	42045694	A	G	UTR3	TAF8	ENST000004	.	.	A	G	+	+	0.28571429	5,0,2,0	chr6,420456	Cytosol	A > G
chr6:42045888:A:G:UTR3:TAF8	chr6	42045888	42045888	A	G	UTR3	TAF8	ENST000004	.	.	A	G	+	+	0.09090909	20,0,2,0	chr6,420458	Cytosol	A > G
chr6:42045890:A:G:UTR3:TAF8	chr6	42045890	42045890	A	G	UTR3	TAF8	ENST000004	.	.	A	G	+	+	0.1	18,0,2,0	chr6,420458	Cytosol	A > G
chr6:42046582:A:G:UTR3:TAF8	chr6	42046582	42046582	A	G	UTR3	TAF8	ENST000004	.	.	A	G	+	+	0.16666667	15,0,3,0	chr6,420465	Cytosol	A > G
chr6:42046678:A:G:UTR3:TAF8	chr6	42046678	42046678	A	G	UTR3	TAF8	ENST000003	.	.	A	G	+	+	0.57142857	3,0,4,0	chr6,420466	Cytosol	A > G
chr6:42047354:A:G:UTR3:TAF8	chr6	42047354	42047354	A	G	UTR3	TAF8	ENST000003	.	.	A	G	+	+	0.33333333	24,0,12,0	chr6,420473	Cytosol	A > G
chr6:42047362:A:G:UTR3:TAF8	chr6	42047362	42047362	A	G	UTR3	TAF8	ENST000003	.	.	A	G	+	+	0.06896552	27,0,2,0	chr6,420473	Cytosol	A > G
chr6:42047375:A:G:UTR3:TAF8	chr6	42047375	42047375	A	G	UTR3	TAF8	ENST000003	.	.	A	G	+	+	0.33333333	20,0,10,0	chr6,420473	Cytosol	A > G
chr6:42047387:A:G:UTR3:TAF8	chr6	42047387	42047387	A	G	UTR3	TAF8	ENST000003	.	.	A	G	+	+	0.05882353	32,0,2,0	chr6,420473	Cytosol	A > G
chr6:42047426:A:G:UTR3:TAF8	chr6	42047426	42047426	A	G	UTR3	TAF8	ENST000003	.	.	A	G	+	+	0.39130435	14,0,9,0	chr6,420474	Cytosol	A > G
chr6:42047527:A:G:UTR3:TAF8	chr6	42047527	42047527	A	G	UTR3	TAF8	ENST000003	.	.	A	G	+	+	0.08695652	21,0,2,0	chr6,420475	Cytosol	A > G
chr6:42047552:A:G:UTR3:TAF8	chr6	42047552	42047552	A	G	UTR3	TAF8	ENST000003	.	.	A	G	+	+	0.33333333	8,0,4,0	chr6,420475	Cytosol	A > G
chr6:42047553:A:G:UTR3:TAF8	chr6	42047553	42047553	A	G	UTR3	TAF8	ENST000003	.	.	A	G	+	+	0.2	12,0,3,0	chr6,420475	Cytosol	A > G
chr6:42047554:A:G:UTR3:TAF8	chr6	42047554	42047554	A	G	UTR3	TAF8	ENST000003	.	.	A	G	+	+	0.21428571	11,0,3,0	chr6,420475	Cytosol	A > G
chr6:42048167:A:G:UTR3:TAF8	chr6	42048167	42048167	A	G	UTR3	TAF8	ENST000003	.	.	A	G	+	+	0.3125	11,0,5,0	chr6,420481	Cytosol	A > G
chr6:42048276:A:G:UTR3:TAF8	chr6	42048276	42048276	A	G	UTR3	TAF8	ENST000003	.	.	A	G	+	+	0.29166667	17,0,7,0	chr6,420482	Cytosol	A > G
chr6:42172866:T:C:intergenic:GUC	chr6	42172866	42172866	T	C	intergenic	GUCA1B,MR	dist=10212;d	.	.	A	G	-		0.44444444	5,0,4,0	chr6,421728	Cytosol	A > G
chr6:42175186:T:C:UTR3:MRPS10	chr6	42175186	42175186	T	C	UTR3	MRPS10	ENST000000	.	.	A	G	-	-	0.03968254	121,0,5,0	chr6,421751	Cytosol	A > G
chr6:42854531:A:G:UTR3:RPL7L1	chr6	42854531	42854531	A	G	UTR3	RPL7L1	ENST000004	.	.	A	G	+	+	0.05	38,0,2,0	chr6,428545	Cytosol	A > G
chr6:42854580:A:G:UTR3:RPL7L1	chr6	42854580	42854580	A	G	UTR3	RPL7L1	ENST000004	.	.	A	G	+	+	0.02631579	74,0,2,0	chr6,428545	Cytosol	A > G
chr6:42854633:A:G:UTR3:RPL7L1	chr6	42854633	42854633	A	G	UTR3	RPL7L1	ENST000004	.	.	A	G	+	+	0.53846154	6,0,7,0	chr6,428546	Cytosol	A > G
chr6:42854664:A:G:UTR3:RPL7L1	chr6	42854664	42854664	A	G	UTR3	RPL7L1	ENST000004	.	.	A	G	+	+	0.13924051	68,0,11,0	chr6,428546	Cytosol	A > G
chr6:42854681:A:G:UTR3:RPL7L1	chr6	42854681	42854681	A	G	UTR3	RPL7L1	ENST000004	.	.	A	G	+	+	0.03684211	183,0,7,0	chr6,428546	Cytosol	A > G
chr6:42855101:A:G:UTR3:RPL7L1	chr6	42855101	42855101	A	G	UTR3	RPL7L1	ENST000004	.	.	A	G	+	+	0.01025641	193,0,2,0	chr6,428551	Cytosol	A > G
chr6:42855106:A:G:UTR3:RPL7L1	chr6	42855106	42855106	A	G	UTR3	RPL7L1	ENST000004	.	.	A	G	+	+	0.02955665	197,0,6,0	chr6,428551	Cytosol	A > G
chr6:42855256:A:G:UTR3:RPL7L1	chr6	42855256	42855256	A	G	UTR3	RPL7L1	ENST000004	.	.	A	G	+	+	0.09359606	184,0,19,0	chr6,428552	Cytosol	A > G
chr6:42855948:A:G:UTR3:RPL7L1	chr6	42855948	42855948	A	G	UTR3	RPL7L1	ENST000004	.	.	A	G	+	+	0.03703704	104,0,4,0	chr6,428559	Cytosol	A > G
chr6:42855954:A:G:UTR3:RPL7L1	chr6	42855954	42855954	A	G	UTR3	RPL7L1	ENST000004	.	.	A	G	+	+	0.16564417	136,0,27,0	chr6,428559	Cytosol	A > G
chr6:42855962:A:G:UTR3:RPL7L1	chr6	42855962	42855962	A	G	UTR3	RPL7L1	ENST000004	.	.	A	G	+	+	0.47926267	113,0,104,0	chr6,428559	Cytosol	A > G
chr6:42855967:A:G:UTR3:RPL7L1	chr6	42855967	42855967	A	G	UTR3	RPL7L1	ENST000004	.	.	A	G	+	+	0.05668016	233,0,14,0	chr6,428559	Cytosol	A > G
chr6:42855972:A:G:UTR3:RPL7L1	chr6	42855972	42855972	A	G	UTR3	RPL7L1	ENST000004	.	.	A	G	+	+	0.1969697	212,0,52,0	chr6,428559	Cytosol	A > G
chr6:42855979:A:G:UTR3:RPL7L1	chr6	42855979	42855979	A	G	UTR3	RPL7L1	ENST000004	.	.	A	G	+	+	0.10738255	266,0,32,0	chr6,428559	Cytosol	A > G
chr6:42856007:A:G:UTR3:RPL7L1	chr6	42856007	42856007	A	G	UTR3	RPL7L1	ENST000004	.	.	A	G	+	+	0.03370787	344,0,12,0	chr6,428560	Cytosol	A > G
chr6:42856019:A:G:UTR3:RPL7L1	chr6	42856019	42856019	A	G	UTR3	RPL7L1	ENST000004	.	.	A	G	+	+	0.01002506	395,0,4,0	chr6,428560	Cytosol	A > G
chr6:42856029:A:G:UTR3:RPL7L1	chr6	42856029	42856029	A	G	UTR3	RPL7L1	ENST000004	.	.	A	G	+	+	0.63685637	134,0,235,0	chr6,428560	Cytosol	A > G
chr6:42856039:A:G:UTR3:RPL7L1	chr6	42856039	42856039	A	G	UTR3	RPL7L1	ENST000004	.	.	A	G	+	+	0.11733333	331,0,44,0	chr6,428560	Cytosol	A > G
chr6:42856042:A:G:UTR3:RPL7L1	chr6	42856042	42856042	A	G	UTR3	RPL7L1	ENST000004	.	.	A	G	+	+	0.03409091	339,0,12,1	chr6,428560	Cytosol	A > G
chr6:42856045:A:G:UTR3:RPL7L1	chr6	42856045	42856045	A	G	UTR3	RPL7L1	ENST000004	.	.	A	G	+	+	0.04310345	333,0,15,0	chr6,428560	Cytosol	A > G
chr6:42856047:A:G:UTR3:RPL7L1	chr6	42856047	42856047	A	G	UTR3	RPL7L1	ENST000004	.	.	A	G	+	+	0.17595308	281,0,60,0	chr6,428560	Cytosol	A > G
chr6:42856051:A:G:UTR3:RPL7L1	chr6	42856051	42856051	A	G	UTR3	RPL7L1	ENST000004	.	.	A	G	+	+	0.56574924	142,0,185,0	chr6,428560	Cytosol	A > G
chr6:42856055:A:G:UTR3:RPL7L1	chr6	42856055	42856055	A	G	UTR3	RPL7L1	ENST000004	.	.	A	G	+	+	0.1829653	259,0,58,0	chr6,428560	Cytosol	A > G
chr6:42856056:A:G:UTR3:RPL7L1	chr6	42856056	42856056	A	G	UTR3	RPL7L1	ENST000004	.	.	A	G	+	+	0.06542056	299,0,21,1	chr6,428560	Cytosol	A > G
chr6:42856073:A:G:UTR3:RPL7L1	chr6	42856073	42856073	A	G	UTR3	RPL7L1	ENST000004	.	.	A	G	+	+	0.01639344	300,0,5,0	chr6,428560	Cytosol	A > G
chr6:42856093:A:G:UTR3:RPL7L1	chr6	42856093	42856093	A	G	UTR3	RPL7L1	ENST000004	.	.	A	G	+	+	0.02932551	331,0,10,0	chr6,428560	Cytosol	A > G
chr6:42856100:A:G:UTR3:RPL7L1	chr6	42856100	42856100	A	G	UTR3	RPL7L1	ENST000004	.	.	A	G	+	+	0.40655738	181,0,124,0	chr6,428560	Cytosol	A > G
chr6:42856105:A:G:UTR3:RPL7L1	chr6	42856105	42856105	A	G	UTR3	RPL7L1	ENST000004	.	.	A	G	+	+	0.20677966	234,0,61,0	chr6,428561	Cytosol	A > G
chr6:42856110:A:G:UTR3:RPL7L1	chr6	42856110	42856110	A	G	UTR3	RPL7L1	ENST000004	.	.	A	G	+	+	0.94197952	17,0,276,0	chr6,428561	Cytosol	A > G
chr6:42856116:A:G:UTR3:RPL7L1	chr6	42856116	42856116	A	G	UTR3	RPL7L1	ENST000004	.	.	A	G	+	+	0.72053872	83,0,214,0	chr6,428561	Cytosol	A > G
chr6:42856118:A:G:UTR3:RPL7L1	chr6	42856118	42856118	A	G	UTR3	RPL7L1	ENST000004	.	.	A	G	+	+	0.17793594	231,0,50,0	chr6,428561	Cytosol	A > G
chr6:42856131:A:G:UTR3:RPL7L1	chr6	42856131	42856131	A	G	UTR3	RPL7L1	ENST000004	.	.	A	G	+	+	0.01875	314,0,6,0	chr6,428561	Cytosol	A > G
chr6:42856139:A:G:UTR3:RPL7L1	chr6	42856139	42856139	A	G	UTR3	RPL7L1	ENST000004	.	.	A	G	+	+	0.01685393	350,0,6,0	chr6,428561	Cytosol	A > G
chr6:42856156:A:G:UTR3:RPL7L1	chr6	42856156	42856156	A	G	UTR3	RPL7L1	ENST000004	.	.	A	G	+	+	0.06470588	318,0,22,0	chr6,428561	Cytosol	A > G
chr6:42856157:A:G:UTR3:RPL7L1	chr6	42856157	42856157	A	G	UTR3	RPL7L1	ENST000004	.	.	A	G	+	+	0.02941176	330,0,10,0	chr6,428561	Cytosol	A > G
chr6:42856158:A:G:UTR3:RPL7L1	chr6	42856158	42856158	A	G	UTR3	RPL7L1	ENST000004	.	.	A	G	+	+	0.06461538	304,0,21,0	chr6,428561	Cytosol	A > G
chr6:42856170:A:G:UTR3:RPL7L1	chr6	42856170	42856170	A	G	UTR3	RPL7L1	ENST000004	.	.	A	G	+	+	0.05	323,0,17,0	chr6,428561	Cytosol	A > G
chr6:42856172:A:G:UTR3:RPL7L1	chr6	42856172	42856172	A	G	UTR3	RPL7L1	ENST000004	.	.	A	G	+	+	0.02967359	327,0,10,0	chr6,428561	Cytosol	A > G
chr6:42856176:A:G:UTR3:RPL7L1	chr6	42856176	42856176	A	G	UTR3	RPL7L1	ENST000004	.	.	A	G	+	+	0.24046921	259,0,82,0	chr6,428561	Cytosol	A > G

chr6:42856905:A:G:UTR3:RPL7L1	chr6	42856905	42856905	A	G	UTR3	RPL7L1	ENST000004	.	.	A	G	+	+	0.06417112	350,0,24,0	chr6,428569	Cytosol	A > G
chr6:42856917:A:G:UTR3:RPL7L1	chr6	42856917	42856917	A	G	UTR3	RPL7L1	ENST000004	.	.	A	G	+	+	0.63300493	148,0,257,1	chr6,428569	Cytosol	A > G
chr6:42856923:A:G:UTR3:RPL7L1	chr6	42856923	42856923	A	G	UTR3	RPL7L1	ENST000004	.	.	A	G	+	+	0.02068966	426,0,9,0	chr6,428569	Cytosol	A > G
chr6:42856937:A:G:UTR3:RPL7L1	chr6	42856937	42856937	A	G	UTR3	RPL7L1	ENST000004	.	.	A	G	+	+	0.0828877	343,0,31,0	chr6,428569	Cytosol	A > G
chr6:42856954:A:G:UTR3:RPL7L1	chr6	42856954	42856954	A	G	UTR3	RPL7L1	ENST000004	.	.	A	G	+	+	0.07228916	231,0,18,0	chr6,428569	Cytosol	A > G
chr6:42856959:A:G:UTR3:RPL7L1	chr6	42856959	42856959	A	G	UTR3	RPL7L1	ENST000004	.	.	A	G	+	+	0.02755906	247,0,7,0	chr6,428569	Cytosol	A > G
chr6:42856964:A:G:UTR3:RPL7L1	chr6	42856964	42856964	A	G	UTR3	RPL7L1	ENST000004	.	.	A	G	+	+	0.03589744	188,0,7,0	chr6,428569	Cytosol	A > G
chr6:42856969:A:G:UTR3:RPL7L1	chr6	42856969	42856969	A	G	UTR3	RPL7L1	ENST000004	.	.	A	G	+	+	0.07731959	179,0,15,0	chr6,428569	Cytosol	A > G
chr6:42856978:A:G:UTR3:RPL7L1	chr6	42856978	42856978	A	G	UTR3	RPL7L1	ENST000004	.	.	A	G	+	+	0.07555556	208,0,17,0	chr6,428569	Cytosol	A > G
chr6:42856983:A:G:UTR3:RPL7L1	chr6	42856983	42856983	A	G	UTR3	RPL7L1	ENST000004	.	.	A	G	+	+	0.56916996	109,0,144,0	chr6,428569	Cytosol	A > G
chr6:42856989:A:G:UTR3:RPL7L1	chr6	42856989	42856989	A	G	UTR3	RPL7L1	ENST000004	.	.	A	G	+	+	0.0155642	253,0,4,0	chr6,428569	Cytosol	A > G
chr6:42856999:A:G:UTR3:RPL7L1	chr6	42856999	42856999	A	G	UTR3	RPL7L1	ENST000004	.	.	A	G	+	+	0.01086957	273,0,3,0	chr6,428569	Cytosol	A > G
chr6:42857014:A:G:UTR3:RPL7L1	chr6	42857014	42857014	A	G	UTR3	RPL7L1	ENST000004	.	.	A	G	+	+	0.01107011	268,0,3,0	chr6,428570	Cytosol	A > G
chr6:42857021:A:G:UTR3:RPL7L1	chr6	42857021	42857021	A	G	UTR3	RPL7L1	ENST000004	.	.	A	G	+	+	0.072	232,0,18,0	chr6,428570	Cytosol	A > G
chr6:42857023:A:G:UTR3:RPL7L1	chr6	42857023	42857023	A	G	UTR3	RPL7L1	ENST000004	.	.	A	G	+	+	0.16602317	216,0,43,0	chr6,428570	Cytosol	A > G
chr6:42857026:A:G:UTR3:RPL7L1	chr6	42857026	42857026	A	G	UTR3	RPL7L1	ENST000004	.	.	A	G	+	+	0.04150943	253,1,11,0	chr6,428570	Cytosol	A > G
chr6:42857028:A:G:UTR3:RPL7L1	chr6	42857028	42857028	A	G	UTR3	RPL7L1	ENST000004	.	.	A	G	+	+	0.07169811	246,0,19,0	chr6,428570	Cytosol	A > G
chr6:42857031:A:G:UTR3:RPL7L1	chr6	42857031	42857031	A	G	UTR3	RPL7L1	ENST000004	.	.	A	G	+	+	0.49275362	140,0,136,0	chr6,428570	Cytosol	A > G
chr6:42857037:A:G:UTR3:RPL7L1	chr6	42857037	42857037	A	G	UTR3	RPL7L1	ENST000004	.	.	A	G	+	+	0.02341137	292,0,7,0	chr6,428570	Cytosol	A > G
chr6:42857057:A:G:UTR3:RPL7L1	chr6	42857057	42857057	A	G	UTR3	RPL7L1	ENST000004	.	.	A	G	+	+	0.1042654	189,0,22,0	chr6,428570	Cytosol	A > G
chr6:42857058:A:G:UTR3:RPL7L1	chr6	42857058	42857058	A	G	UTR3	RPL7L1	ENST000004	.	.	A	G	+	+	0.04347826	198,0,9,0	chr6,428570	Cytosol	A > G
chr6:42857067:A:G:UTR3:RPL7L1	chr6	42857067	42857067	A	G	UTR3	RPL7L1	ENST000004	.	.	A	G	+	+	0.05113636	167,0,9,0	chr6,428570	Cytosol	A > G
chr6:42857136:A:G:UTR3:RPL7L1	chr6	42857136	42857136	A	G	UTR3	RPL7L1	ENST000004	.	.	A	G	+	+	0.07361963	302,0,24,0	chr6,428571	Cytosol	A > G
chr6:42857179:C:A:UTR3:RPL7L1	chr6	42857179	42857179	C	A	UTR3	RPL7L1	ENST000004	.	.	C	A	+	+	0.0104712	2,189,0,0	chr6,428571	Cytosol	C > A
chr6:42997384:A:G:intronic:RRP3	chr6	42997384	42997384	A	G	intronic	RRP36	.	.	.	A	G	+	+	0.08641975	74,0,7,0	chr6,429973	Cytosol	A > G
chr6:42997387:A:G:intronic:RRP3	chr6	42997387	42997387	A	G	intronic	RRP36	.	.	.	A	G	+	+	0.28205128	56,0,22,0	chr6,429973	Cytosol	A > G
chr6:42997403:A:G:intronic:RRP3	chr6	42997403	42997403	A	G	intronic	RRP36	.	.	.	A	G	+	+	0.1125	71,0,9,0	chr6,429974	Cytosol	A > G
chr6:42997464:A:G:intronic:RRP3	chr6	42997464	42997464	A	G	intronic	RRP36	.	.	.	A	G	+	+	0.77966102	26,0,92,0	chr6,429974	Cytosol	A > G
chr6:42997493:A:G:intronic:RRP3	chr6	42997493	42997493	A	G	intronic	RRP36	.	.	.	A	G	+	+	0.58823529	63,0,90,0	chr6,429974	Cytosol	A > G
chr6:42997538:A:G:intronic:RRP3	chr6	42997538	42997538	A	G	intronic	RRP36	.	.	.	A	G	+	+	0.49166667	61,0,59,0	chr6,429975	Cytosol	A > G
chr6:42997541:A:G:intronic:RRP3	chr6	42997541	42997541	A	G	intronic	RRP36	.	.	.	A	G	+	+	0.48	52,0,48,0	chr6,429975	Cytosol	A > G
chr6:42997547:A:G:intronic:RRP3	chr6	42997547	42997547	A	G	intronic	RRP36	.	.	.	A	G	+	+	0.79824561	23,0,91,0	chr6,429975	Cytosol	A > G
chr6:42997578:A:G:intronic:RRP3	chr6	42997578	42997578	A	G	intronic	RRP36	.	.	.	A	G	+	+	0.89230769	14,0,116,0	chr6,429975	Cytosol	A > G
chr6:42997593:A:G:intronic:RRP3	chr6	42997593	42997593	A	G	intronic	RRP36	.	.	.	A	G	+	+	0.44230769	58,0,46,0	chr6,429975	Cytosol	A > G
chr6:43000069:A:G:intronic:RRP3	chr6	43000069	43000069	A	G	intronic	RRP36	.	.	.	A	G	+	+	0.57894737	8,0,11,0	chr6,430000	Cytosol	A > G
chr6:43000214:A:G:intronic:RRP3	chr6	43000214	43000214	A	G	intronic	RRP36	.	.	.	A	G	+	+	0.07894737	35,0,3,0	chr6,430002	Cytosol	A > G
chr6:43489657:T:C:intronic:POLR1	chr6	43489657	43489657	T	C	intronic	POLR1C	.	.	.	A	G	-	+	0.2	8,0,2,0	chr6,434896	Cytosol	A > G
chr6:43504773:A:G:intronic:XPO5	chr6	43504773	43504773	A	G	intronic	XPO5	.	.	.	A	G	+	-	0.4	3,0,2,0	chr6,435047	Cytosol	A > G
chr6:43555511:A:G:intronic:POLH	chr6	43555511	43555511	A	G	intronic	POLH	.	.	.	A	G	+	+	0.8	1,0,4,0	chr6,435555	Cytosol	A > G
chr6:43555720:A:G:intronic:POLH	chr6	43555720	43555720	A	G	intronic	POLH	.	.	.	A	G	+	+	0.25	6,0,2,0	chr6,435557	Cytosol	A > G
chr6:43556314:A:G:ncRNA_exonic	chr6	43556314	43556314	A	G	ncRNA_exon	RP3-337H4.8	.	.	.	A	G	+	+-	0.375	5,0,3,0	chr6,435563	Cytosol	A > G
chr6:43556373:A:G:ncRNA_exonic	chr6	43556373	43556373	A	G	ncRNA_exon	RP3-337H4.8	.	.	.	A	G	+	+-	0.6	4,0,6,0	chr6,435563	Cytosol	A > G
chr6:43556473:A:G:ncRNA_exonic	chr6	43556473	43556473	A	G	ncRNA_exon	RP3-337H4.8	.	.	.	A	G	+	+-	0.28571429	5,0,2,0	chr6,435564	Cytosol	A > G
chr6:43557976:A:G:ncRNA_intron	chr6	43557976	43557976	A	G	ncRNA_intro	RP3-337H4.8	.	.	.	A	G	+	+-	0.54545455	5,0,6,0	chr6,435579	Cytosol	A > G
chr6:43559108:A:G:intronic:POLH	chr6	43559108	43559108	A	G	intronic	POLH	.	.	.	A	G	+	+	0.15384615	11,0,2,0	chr6,435591	Cytosol	A > G
chr6:43559129:A:G:intronic:POLH	chr6	43559129	43559129	A	G	intronic	POLH	.	.	.	A	G	+	+	0.14285714	12,0,2,0	chr6,435591	Cytosol	A > G
chr6:43559143:A:G:intronic:POLH	chr6	43559143	43559143	A	G	intronic	POLH	.	.	.	A	G	+	+	0.28571429	10,0,4,0	chr6,435591	Cytosol	A > G
chr6:43559156:A:G:intronic:POLH	chr6	43559156	43559156	A	G	intronic	POLH	.	.	.	A	G	+	+	0.27272727	8,0,3,0	chr6,435591	Cytosol	A > G
chr6:43559175:A:G:intronic:POLH	chr6	43559175	43559175	A	G	intronic	POLH	.	.	.	A	G	+	+	0.625	3,0,5,0	chr6,435591	Cytosol	A > G
chr6:43559186:A:G:intronic:POLH	chr6	43559186	43559186	A	G	intronic	POLH	.	.	.	A	G	+	+	0.5	4,0,4,0	chr6,435591	Cytosol	A > G
chr6:43559221:A:G:intronic:POLH	chr6	43559221	43559221	A	G	intronic	POLH	.	.	.	A	G	+	+	0.3	7,0,3,0	chr6,435592	Cytosol	A > G
chr6:43559239:A:G:intronic:POLH	chr6	43559239	43559239	A	G	intronic	POLH	.	.	.	A	G	+	+	0.375	5,0,3,0	chr6,435592	Cytosol	A > G
chr6:43582750:A:G:UTR3:POLH	chr6	43582750	43582750	A	G	UTR3	POLH	ENST000003	.	.	A	G	+	+-	0.0952381	19,0,2,0	chr6,435827	Cytosol	A > G
chr6:43582795:A:G:UTR3:POLH	chr6	43582795	43582795	A	G	UTR3	POLH	ENST000003	.	.	A	G	+	+-	0.3	7,0,3,0	chr6,435827	Cytosol	A > G
chr6:43582841:A:G:UTR3:POLH	chr6	43582841	43582841	A	G	UTR3	POLH	ENST000003	.	.	A	G	+	+-	0.0952381	19,0,2,0	chr6,435828	Cytosol	A > G
chr6:43582895:A:G:UTR3:POLH	chr6	43582895	43582895	A	G	UTR3	POLH	ENST000003	.	.	A	G	+	+-	0.17142857	29,0,6,0	chr6,435828	Cytosol	A > G
chr6:43582956:A:G:UTR3:POLH	chr6	43582956	43582956	A	G	UTR3	POLH	ENST000003	.	.	A	G	+	+-	0.125	21,0,3,0	chr6,435829	Cytosol	A > G
chr6:43583038:A:G:UTR3:POLH	chr6	43583038	43583038	A	G	UTR3	POLH	ENST000003	.	.	A	G	+	+-	0.18518519	22,0,5,0	chr6,435830	Cytosol	A > G
chr6:43583067:A:G:UTR3:POLH	chr6	43583067	43583067	A	G	UTR3	POLH	ENST000003	.	.	A	G	+	+-	0.125	14,0,2,0	chr6,435830	Cytosol	A > G
chr6:43583171:A:G:UTR3:POLH	chr6	43583171	43583171	A	G	UTR3	POLH	ENST000003	.	.	A	G	+	+-	0.17391304	19,0,4,0	chr6,435831	Cytosol	A > G
chr6:43583172:A:G:UTR3:POLH	chr6	43583172	43583172	A	G	UTR3	POLH	ENST000003	.	.	A	G	+	+-	0.59090909	9,0,13,0	chr6,435831	Cytosol	A > G
chr6:43583185:A:G:UTR3:POLH	chr6	43583185	43583185	A	G	UTR3	POLH	ENST000003	.	.	A	G	+	+-	0.29411765	12,0,5,0	chr6,435831	Cytosol	A > G
chr6:43583193:A:G:UTR3:POLH	chr6	43583193	43583193	A	G	UTR3	POLH	ENST000003	.	.	A	G	+	+-	0.47058824	9,0,8,0	chr6,435831	Cytosol	A > G
chr6:43583207:A:G:UTR3:POLH	chr6	43583207	43583207	A	G	UTR3	POLH	ENST000003	.	.	A	G	+	+-	0.15384615	11,0,2,0	chr6,435832	Cytosol	A > G

chr6:43583224:A:G:UTR3:POLH	chr6	43583224	43583224	A	G	UTR3	POLH	ENST000003	.	.	A	G	+	+-	0.3125	11,0,5,0	chr6,435832	Cytosol	A > G
chr6:43583233:A:G:UTR3:POLH	chr6	43583233	43583233	A	G	UTR3	POLH	ENST000003	.	.	A	G	+	+-	0.33333333	12,0,6,0	chr6,435832	Cytosol	A > G
chr6:43583458:A:G:intronic:GTPBI	chr6	43583458	43583458	A	G	intronic	GTPBP2,POLI	.	.	.	A	G	+	+-	0.875	1,0,7,0	chr6,435834	Cytosol	A > G
chr6:43583479:A:G:intronic:GTPBI	chr6	43583479	43583479	A	G	intronic	GTPBP2,POLI	.	.	.	A	G	+	+-	0.85714286	1,0,6,0	chr6,435834	Cytosol	A > G
chr6:43583644:A:G:intronic:GTPBI	chr6	43583644	43583644	A	G	intronic	GTPBP2,POLI	.	.	.	A	G	+	+-	0.58333333	5,0,7,0	chr6,435836	Cytosol	A > G
chr6:43583683:A:G:intronic:GTPBI	chr6	43583683	43583683	A	G	intronic	GTPBP2,POLI	.	.	.	A	G	+	+-	0.75	3,0,9,0	chr6,435836	Cytosol	A > G
chr6:43583896:A:G:intronic:GTPBI	chr6	43583896	43583896	A	G	intronic	GTPBP2,POLI	.	.	.	A	G	+	+-	0.4	3,0,2,0	chr6,435838	Cytosol	A > G
chr6:43584076:A:G:intronic:GTPBI	chr6	43584076	43584076	A	G	intronic	GTPBP2,POLI	.	.	.	A	G	+	+-	0.09375	29,0,3,0	chr6,435840	Cytosol	A > G
chr6:43584132:A:G:intronic:GTPBI	chr6	43584132	43584132	A	G	intronic	GTPBP2,POLI	.	.	.	A	G	+	+-	0.08571429	32,0,3,0	chr6,435841	Cytosol	A > G
chr6:43584179:A:G:intronic:GTPBI	chr6	43584179	43584179	A	G	intronic	GTPBP2,POLI	.	.	.	A	G	+	+-	0.2	20,0,5,0	chr6,435841	Cytosol	A > G
chr6:43584206:A:G:intronic:GTPBI	chr6	43584206	43584206	A	G	intronic	GTPBP2,POLI	.	.	.	A	G	+	+-	0.13793103	25,0,4,0	chr6,435842	Cytosol	A > G
chr6:43584247:A:G:intronic:GTPBI	chr6	43584247	43584247	A	G	intronic	GTPBP2,POLI	.	.	.	A	G	+	+-	0.05714286	33,0,2,0	chr6,435842	Cytosol	A > G
chr6:43584267:A:G:intronic:GTPBI	chr6	43584267	43584267	A	G	intronic	GTPBP2,POLI	.	.	.	A	G	+	+-	0.24242424	25,0,8,0	chr6,435842	Cytosol	A > G
chr6:43584281:A:G:intronic:GTPBI	chr6	43584281	43584281	A	G	intronic	GTPBP2,POLI	.	.	.	A	G	+	+-	0.09756098	37,0,4,0	chr6,435842	Cytosol	A > G
chr6:43584338:C:A:intronic:GTPBI	chr6	43584338	43584338	C	A	intronic	GTPBP2,POLI	.	.	.	C	A	+	+-	0.15909091	7,37,0,0	chr6,435843	Cytosol	C > A
chr6:43584694:A:G:intronic:GTPBI	chr6	43584694	43584694	A	G	intronic	GTPBP2,POLI	.	.	.	A	G	+	+-	0.06666667	28,0,2,0	chr6,435846	Cytosol	A > G
chr6:43584698:A:G:intronic:GTPBI	chr6	43584698	43584698	A	G	intronic	GTPBP2,POLI	.	.	.	A	G	+	+-	0.09677419	28,0,3,0	chr6,435846	Cytosol	A > G
chr6:43584713:A:G:intronic:GTPBI	chr6	43584713	43584713	A	G	intronic	GTPBP2,POLI	.	.	.	A	G	+	+-	0.08695652	21,0,2,0	chr6,435847	Cytosol	A > G
chr6:43584725:A:G:intronic:GTPBI	chr6	43584725	43584725	A	G	intronic	GTPBP2,POLI	.	.	.	A	G	+	+-	0.0952381	19,0,2,0	chr6,435847	Cytosol	A > G
chr6:43584726:A:G:intronic:GTPBI	chr6	43584726	43584726	A	G	intronic	GTPBP2,POLI	.	.	.	A	G	+	+-	0.13636364	19,0,3,0	chr6,435847	Cytosol	A > G
chr6:43584743:A:G:intronic:GTPBI	chr6	43584743	43584743	A	G	intronic	GTPBP2,POLI	.	.	.	A	G	+	+-	0.11764706	15,0,2,0	chr6,435847	Cytosol	A > G
chr6:43584765:A:G:intronic:GTPBI	chr6	43584765	43584765	A	G	intronic	GTPBP2,POLI	.	.	.	A	G	+	+-	0.33333333	6,0,3,0	chr6,435847	Cytosol	A > G
chr6:43584767:A:G:intronic:GTPBI	chr6	43584767	43584767	A	G	intronic	GTPBP2,POLI	.	.	.	A	G	+	+-	0.22222222	7,0,2,0	chr6,435847	Cytosol	A > G
chr6:43584842:A:G:intronic:GTPBI	chr6	43584842	43584842	A	G	intronic	GTPBP2,POLI	.	.	.	A	G	+	+-	0.28571429	5,0,2,0	chr6,435848	Cytosol	A > G
chr6:43584848:A:G:intronic:GTPBI	chr6	43584848	43584848	A	G	intronic	GTPBP2,POLI	.	.	.	A	G	+	+-	0.28571429	5,0,2,0	chr6,435848	Cytosol	A > G
chr6:43584859:A:G:intronic:GTPBI	chr6	43584859	43584859	A	G	intronic	GTPBP2,POLI	.	.	.	A	G	+	+-	0.66666667	3,0,6,0	chr6,435848	Cytosol	A > G
chr6:43585130:A:G:intronic:GTPBI	chr6	43585130	43585130	A	G	intronic	GTPBP2,POLI	.	.	.	A	G	+	+-	0.2	16,0,4,0	chr6,435851	Cytosol	A > G
chr6:43585217:A:G:intronic:GTPBI	chr6	43585217	43585217	A	G	intronic	GTPBP2,POLI	.	.	.	A	G	+	+-	0.17647059	14,0,3,0	chr6,435852	Cytosol	A > G
chr6:43585537:A:G:intronic:GTPBI	chr6	43585537	43585537	A	G	intronic	GTPBP2,POLI	.	.	.	A	G	+	+-	0.5	4,0,4,0	chr6,435855	Cytosol	A > G
chr6:43585643:A:G:intronic:GTPBI	chr6	43585643	43585643	A	G	intronic	GTPBP2,POLI	.	.	.	A	G	+	+-	0.16666667	15,0,3,0	chr6,435856	Cytosol	A > G
chr6:43585957:A:G:intronic:GTPBI	chr6	43585957	43585957	A	G	intronic	GTPBP2,POLI	.	.	.	A	G	+	+-	0.52631579	9,0,10,0	chr6,435859	Cytosol	A > G
chr6:43585992:A:G:intronic:GTPBI	chr6	43585992	43585992	A	G	intronic	GTPBP2,POLI	.	.	.	A	G	+	+-	0.33333333	8,0,4,0	chr6,435859	Cytosol	A > G
chr6:43585993:A:G:intronic:GTPBI	chr6	43585993	43585993	A	G	intronic	GTPBP2,POLI	.	.	.	A	G	+	+-	0.30769231	9,0,4,0	chr6,435859	Cytosol	A > G
chr6:43585997:A:G:intronic:GTPBI	chr6	43585997	43585997	A	G	intronic	GTPBP2,POLI	.	.	.	A	G	+	+-	0.46153846	7,0,6,0	chr6,435859	Cytosol	A > G
chr6:43586022:A:G:intronic:GTPBI	chr6	43586022	43586022	A	G	intronic	GTPBP2,POLI	.	.	.	A	G	+	+-	0.66666667	3,0,6,0	chr6,435860	Cytosol	A > G
chr6:43586027:A:G:intronic:GTPBI	chr6	43586027	43586027	A	G	intronic	GTPBP2,POLI	.	.	.	A	G	+	+-	0.3	7,0,3,0	chr6,435860	Cytosol	A > G
chr6:43586073:A:G:intronic:GTPBI	chr6	43586073	43586073	A	G	intronic	GTPBP2,POLI	.	.	.	A	G	+	+-	0.27272727	8,0,3,0	chr6,435860	Cytosol	A > G
chr6:43586076:A:G:intronic:GTPBI	chr6	43586076	43586076	A	G	intronic	GTPBP2,POLI	.	.	.	A	G	+	+-	0.90909091	1,0,10,0	chr6,435860	Cytosol	A > G
chr6:43586082:A:G:intronic:GTPBI	chr6	43586082	43586082	A	G	intronic	GTPBP2,POLI	.	.	.	A	G	+	+-	0.76923077	3,0,10,0	chr6,435860	Cytosol	A > G
chr6:43586149:A:G:intronic:GTPBI	chr6	43586149	43586149	A	G	intronic	GTPBP2,POLI	.	.	.	A	G	+	+-	0.11111111	16,0,2,0	chr6,435861	Cytosol	A > G
chr6:43586150:A:G:intronic:GTPBI	chr6	43586150	43586150	A	G	intronic	GTPBP2,POLI	.	.	.	A	G	+	+-	0.35294118	11,0,6,0	chr6,435861	Cytosol	A > G
chr6:43586151:A:G:intronic:GTPBI	chr6	43586151	43586151	A	G	intronic	GTPBP2,POLI	.	.	.	A	G	+	+-	0.64285714	5,0,9,0	chr6,435861	Cytosol	A > G
chr6:43586419:A:G:intronic:GTPBI	chr6	43586419	43586419	A	G	intronic	GTPBP2,POLI	.	.	.	A	G	+	+-	0.3125	11,0,5,0	chr6,435864	Cytosol	A > G
chr6:43586424:A:G:intronic:GTPBI	chr6	43586424	43586424	A	G	intronic	GTPBP2,POLI	.	.	.	A	G	+	+-	0.15384615	11,0,2,0	chr6,435864	Cytosol	A > G
chr6:43586429:A:G:intronic:GTPBI	chr6	43586429	43586429	A	G	intronic	GTPBP2,POLI	.	.	.	A	G	+	+-	0.18181818	9,0,2,0	chr6,435864	Cytosol	A > G
chr6:43586447:A:G:intronic:GTPBI	chr6	43586447	43586447	A	G	intronic	GTPBP2,POLI	.	.	.	A	G	+	+-	0.27272727	8,0,3,0	chr6,435864	Cytosol	A > G
chr6:43586448:A:G:intronic:GTPBI	chr6	43586448	43586448	A	G	intronic	GTPBP2,POLI	.	.	.	A	G	+	+-	0.27272727	8,0,3,0	chr6,435864	Cytosol	A > G
chr6:43586468:A:G:intronic:GTPBI	chr6	43586468	43586468	A	G	intronic	GTPBP2,POLI	.	.	.	A	G	+	+-	0.55555556	4,0,5,0	chr6,435864	Cytosol	A > G
chr6:43586497:A:G:intronic:GTPBI	chr6	43586497	43586497	A	G	intronic	GTPBP2,POLI	.	.	.	A	G	+	+-	0.28571429	5,0,2,0	chr6,435864	Cytosol	A > G
chr6:43586542:A:G:intronic:GTPBI	chr6	43586542	43586542	A	G	intronic	GTPBP2,POLI	.	.	.	A	G	+	+-	0.33333333	4,0,2,0	chr6,435865	Cytosol	A > G
chr6:43586548:A:G:intronic:GTPBI	chr6	43586548	43586548	A	G	intronic	GTPBP2,POLI	.	.	.	A	G	+	+-	0.375	5,0,3,0	chr6,435865	Cytosol	A > G
chr6:43586603:A:G:UTR3:POLH	chr6	43586603	43586603	A	G	UTR3	POLH	ENST000005	.	.	A	G	+	+-	0.4	3,0,2,0	chr6,435866	Cytosol	A > G
chr6:43586865:A:G:intronic:GTPBI	chr6	43586865	43586865	A	G	intronic	GTPBP2	.	.	.	A	G	+	+-	0.42857143	4,0,3,0	chr6,435868	Cytosol	A > G
chr6:43586914:A:G:intronic:GTPBI	chr6	43586914	43586914	A	G	intronic	GTPBP2	.	.	.	A	G	+	+-	0.25	6,0,2,0	chr6,435869	Cytosol	A > G
chr6:43586935:A:G:intronic:GTPBI	chr6	43586935	43586935	A	G	intronic	GTPBP2	.	.	.	A	G	+	+-	0.42857143	4,0,3,0	chr6,435869	Cytosol	A > G
chr6:43586994:A:G:intronic:GTPBI	chr6	43586994	43586994	A	G	intronic	GTPBP2	.	.	.	A	G	+	+-	0.42857143	4,0,3,0	chr6,435869	Cytosol	A > G
chr6:43587046:A:G:intronic:GTPBI	chr6	43587046	43587046	A	G	intronic	GTPBP2	.	.	.	A	G	+	+-	0.8	1,0,4,0	chr6,435870	Cytosol	A > G
chr6:44417136:A:G:UTR3:CDC5L	chr6	44417136	44417136	A	G	UTR3	CDC5L	ENST000003	.	.	A	G	+	+	0.55555556	8,0,10,0	chr6,444171	Cytosol	A > G
chr6:56921480:A:G:intergenic:KIA	chr6	56921480	56921480	A	G	intergenic	KIAA1586,ZN	dist=1457;dis	.	.	A	G	+		0.5	3,0,3,0	chr6,569214	Cytosol	A > G
chr6:56921507:A:G:intergenic:KIA	chr6	56921507	56921507	A	G	intergenic	KIAA1586,ZN	dist=1484;dis	.	.	A	G	+		0.3	7,0,3,0	chr6,569215	Cytosol	A > G
chr6:56921772:A:G:intergenic:KIA	chr6	56921772	56921772	A	G	intergenic	KIAA1586,ZN	dist=1749;dis	.	.	A	G	+		0.4	3,0,2,0	chr6,569217	Cytosol	A > G
chr6:56921927:A:G:intergenic:KIA	chr6	56921927	56921927	A	G	intergenic	KIAA1586,ZN	dist=1904;dis	.	.	A	G	+		0.33333333	4,0,2,0	chr6,569219	Cytosol	A > G
chr6:56922301:A:G:intergenic:KIA	chr6	56922301	56922301	A	G	intergenic	KIAA1586,ZN	dist=2278;dis	.	.	A	G	+		0.6	2,0,3,0	chr6,569223	Cytosol	A > G
chr6:56922718:A:G:intergenic:KIA	chr6	56922718	56922718	A	G	intergenic	KIAA1586,ZN	dist=2695;dis	.	.	A	G	+		0.53333333	7,0,8,0	chr6,569227	Cytosol	A > G

chr6:56922719:A:G:intergenic:KIA	chr6	56922719	56922719	A	G	intergenic	KIAA1586,ZN	dist=2696;dis	.	.	A	G	+		0.61111111	7,0,11,0	chr6,569227	Cytosol	A > G
chr6:56922722:A:G:intergenic:KIA	chr6	56922722	56922722	A	G	intergenic	KIAA1586,ZN	dist=2699;dis	.	.	A	G	+		0.5	7,0,7,0	chr6,569227	Cytosol	A > G
chr6:58275162:G:T:ncRNA_exonic	chr6	58275162	58275162	G	T	ncRNA_exon	LINC00680	.	.	.	C	A	-	-	0.5	14,14,0,0	chr6,582751	Cytosol	C > A
chr6:58275346:T:C:ncRNA_exonic:	chr6	58275346	58275346	T	C	ncRNA_exon	LINC00680	.	.	.	A	G	-	-	0.16666667	15,0,3,0	chr6,582753	Cytosol	A > G
chr6:71298639:A:G:UTR3:C6orf57	chr6	71298639	71298639	A	G	UTR3	C6orf57	ENST000003	.	.	A	G	+	+	0.97826087	1,0,45,0	chr6,712986	Cytosol	A > G
chr6:71298642:A:G:UTR3:C6orf57	chr6	71298642	71298642	A	G	UTR3	C6orf57	ENST000003	.	.	A	G	+	+	0.72093023	12,0,31,0	chr6,712986	Cytosol	A > G
chr6:71298643:A:G:UTR3:C6orf57	chr6	71298643	71298643	A	G	UTR3	C6orf57	ENST000003	.	.	A	G	+	+	0.59090909	18,0,26,0	chr6,712986	Cytosol	A > G
chr6:71298644:A:G:UTR3:C6orf57	chr6	71298644	71298644	A	G	UTR3	C6orf57	ENST000003	.	.	A	G	+	+	0.21428571	33,0,9,0	chr6,712986	Cytosol	A > G
chr6:71298688:A:G:UTR3:C6orf57	chr6	71298688	71298688	A	G	UTR3	C6orf57	ENST000003	.	.	A	G	+	+	0.08888889	41,0,4,0	chr6,712986	Cytosol	A > G
chr6:71298689:A:G:UTR3:C6orf57	chr6	71298689	71298689	A	G	UTR3	C6orf57	ENST000003	.	.	A	G	+	+	0.4893617	24,0,23,0	chr6,712986	Cytosol	A > G
chr6:71298692:A:G:UTR3:C6orf57	chr6	71298692	71298692	A	G	UTR3	C6orf57	ENST000003	.	.	A	G	+	+	0.11627907	38,0,5,0	chr6,712986	Cytosol	A > G
chr6:71298722:A:G:UTR3:C6orf57	chr6	71298722	71298722	A	G	UTR3	C6orf57	ENST000003	.	.	A	G	+	+	0.65116279	15,0,28,0	chr6,712987	Cytosol	A > G
chr6:71298753:A:G:UTR3:C6orf57	chr6	71298753	71298753	A	G	UTR3	C6orf57	ENST000003	.	.	A	G	+	+	0.42424242	19,0,14,0	chr6,712987	Cytosol	A > G
chr6:71298891:A:G:UTR3:C6orf57	chr6	71298891	71298891	A	G	UTR3	C6orf57	ENST000003	.	.	A	G	+	+	0.14925373	57,0,10,0	chr6,712988	Cytosol	A > G
chr6:71298923:A:G:UTR3:C6orf57	chr6	71298923	71298923	A	G	UTR3	C6orf57	ENST000003	.	.	A	G	+	+	0.09230769	59,0,6,0	chr6,712989	Cytosol	A > G
chr6:71298929:A:G:UTR3:C6orf57	chr6	71298929	71298929	A	G	UTR3	C6orf57	ENST000003	.	.	A	G	+	+	0.68181818	21,0,45,0	chr6,712989	Cytosol	A > G
chr6:71298968:A:G:UTR3:C6orf57	chr6	71298968	71298968	A	G	UTR3	C6orf57	ENST000003	.	.	A	G	+	+	0.8	8,0,32,0	chr6,712989	Cytosol	A > G
chr6:71298969:A:G:UTR3:C6orf57	chr6	71298969	71298969	A	G	UTR3	C6orf57	ENST000003	.	.	A	G	+	+	0.70731707	12,0,29,0	chr6,712989	Cytosol	A > G
chr6:71298973:A:G:UTR3:C6orf57	chr6	71298973	71298973	A	G	UTR3	C6orf57	ENST000003	.	.	A	G	+	+	0.3255814	29,0,14,0	chr6,712989	Cytosol	A > G
chr6:71298986:A:G:UTR3:C6orf57	chr6	71298986	71298986	A	G	UTR3	C6orf57	ENST000003	.	.	A	G	+	+	0.41666667	21,0,15,0	chr6,712989	Cytosol	A > G
chr6:71299008:A:G:UTR3:C6orf57	chr6	71299008	71299008	A	G	UTR3	C6orf57	ENST000003	.	.	A	G	+	+	0.13793103	25,0,4,0	chr6,712990	Cytosol	A > G
chr6:71299052:A:G:UTR3:C6orf57	chr6	71299052	71299052	A	G	UTR3	C6orf57	ENST000003	.	.	A	G	+	+	0.36	16,0,9,0	chr6,712990	Cytosol	A > G
chr6:71299053:A:G:UTR3:C6orf57	chr6	71299053	71299053	A	G	UTR3	C6orf57	ENST000003	.	.	A	G	+	+	0.24	19,0,6,0	chr6,712990	Cytosol	A > G
chr6:71299054:A:G:UTR3:C6orf57	chr6	71299054	71299054	A	G	UTR3	C6orf57	ENST000003	.	.	A	G	+	+	0.36	16,0,9,0	chr6,712990	Cytosol	A > G
chr6:71299059:A:G:UTR3:C6orf57	chr6	71299059	71299059	A	G	UTR3	C6orf57	ENST000003	.	.	A	G	+	+	0.53571429	13,0,15,0	chr6,712990	Cytosol	A > G
chr6:71299074:A:G:UTR3:C6orf57	chr6	71299074	71299074	A	G	UTR3	C6orf57	ENST000003	.	.	A	G	+	+	0.46875	17,0,15,0	chr6,712990	Cytosol	A > G
chr6:74133914:T:C:UTR3:MB21D1	chr6	74133914	74133914	T	C	UTR3	MB21D1	ENST000003	.	.	A	G	-	-	0.09756098	37,0,4,0	chr6,741339	Cytosol	A > G
chr6:74134075:T:C:UTR3:MB21D1	chr6	74134075	74134075	T	C	UTR3	MB21D1	ENST000003	.	.	A	G	-	-	0.12	22,0,3,0	chr6,741340	Cytosol	A > G
chr6:74134669:T:C:UTR3:MB21D1	chr6	74134669	74134669	T	C	UTR3	MB21D1	ENST000003	.	.	A	G	-	-	0.13636364	19,0,3,0	chr6,741346	Cytosol	A > G
chr6:74134673:T:C:UTR3:MB21D1	chr6	74134673	74134673	T	C	UTR3	MB21D1	ENST000003	.	.	A	G	-	-	0.12	22,0,3,0	chr6,741346	Cytosol	A > G
chr6:74134674:T:C:UTR3:MB21D1	chr6	74134674	74134674	T	C	UTR3	MB21D1	ENST000003	.	.	A	G	-	-	0.08333333	22,0,2,0	chr6,741346	Cytosol	A > G
chr6:74134741:T:C:UTR3:MB21D1	chr6	74134741	74134741	T	C	UTR3	MB21D1	ENST000003	.	.	A	G	-	-	0.15384615	11,0,2,0	chr6,741347	Cytosol	A > G
chr6:74134754:T:C:UTR3:MB21D1	chr6	74134754	74134754	T	C	UTR3	MB21D1	ENST000003	.	.	A	G	-	-	0.17391304	19,0,4,0	chr6,741347	Cytosol	A > G
chr6:74134755:T:C:UTR3:MB21D1	chr6	74134755	74134755	T	C	UTR3	MB21D1	ENST000003	.	.	A	G	-	-	0.16666667	20,0,4,0	chr6,741347	Cytosol	A > G
chr6:74134756:T:C:UTR3:MB21D1	chr6	74134756	74134756	T	C	UTR3	MB21D1	ENST000003	.	.	A	G	-	-	0.5	11,0,11,0	chr6,741347	Cytosol	A > G
chr6:74134759:T:C:UTR3:MB21D1	chr6	74134759	74134759	T	C	UTR3	MB21D1	ENST000003	.	.	A	G	-	-	0.5	12,0,12,0	chr6,741347	Cytosol	A > G
chr6:74134781:T:C:UTR3:MB21D1	chr6	74134781	74134781	T	C	UTR3	MB21D1	ENST000003	.	.	A	G	-	-	0.08	23,0,2,0	chr6,741347	Cytosol	A > G
chr6:74134869:T:C:UTR3:MB21D1	chr6	74134869	74134869	T	C	UTR3	MB21D1	ENST000003	.	.	A	G	-	-	0.07462687	62,0,5,0	chr6,741348	Cytosol	A > G
chr6:74303531:T:C:UTR3:SLC17A5	chr6	74303531	74303531	T	C	UTR3	SLC17A5	ENST000003	.	.	A	G	-	-	0.05952381	79,0,5,0	chr6,743035	Cytosol	A > G
chr6:74303543:T:C:UTR3:SLC17A5	chr6	74303543	74303543	T	C	UTR3	SLC17A5	ENST000003	.	.	A	G	-	-	0.1744186	71,0,15,0	chr6,743035	Cytosol	A > G
chr6:74303544:T:C:UTR3:SLC17A5	chr6	74303544	74303544	T	C	UTR3	SLC17A5	ENST000003	.	.	A	G	-	-	0.03529412	82,0,3,0	chr6,743035	Cytosol	A > G
chr6:74304013:T:C:UTR3:SLC17A5	chr6	74304013	74304013	T	C	UTR3	SLC17A5	ENST000003	.	.	A	G	-	-	0.41935484	36,0,26,0	chr6,743040	Cytosol	A > G
chr6:74304014:T:C:UTR3:SLC17A5	chr6	74304014	74304014	T	C	UTR3	SLC17A5	ENST000003	.	.	A	G	-	-	0.14516129	53,0,9,0	chr6,743040	Cytosol	A > G
chr6:88223773:T:C:downstream:R	chr6	88223773	88223773	T	C	downstream	RARS2	.	.	.	A	G	-	-	0.19354839	25,0,6,0	chr6,882237	Cytosol	A > G
chr6:89861578:A:G:intronic:PM20	chr6	89861578	89861578	A	G	intronic	PM20D2	.	.	.	A	G	+	+	0.28571429	5,0,2,0	chr6,898615	Cytosol	A > G
chr6:90344222:T:C:UTR3:LYRM2	chr6	90344222	90344222	T	C	UTR3	LYRM2	ENST000005	.	.	A	G	-	-	0.39759036	49,0,33,1	chr6,903442	Cytosol	A > G
chr6:90344571:T:C:UTR3:LYRM2	chr6	90344571	90344571	T	C	UTR3	LYRM2	ENST000005	.	.	A	G	-	-	0.04347826	88,0,4,0	chr6,903445	Cytosol	A > G
chr6:90344605:T:C:UTR3:LYRM2	chr6	90344605	90344605	T	C	UTR3	LYRM2	ENST000005	.	.	A	G	-	-	0.22340426	73,0,21,0	chr6,903446	Cytosol	A > G
chr6:90344706:T:C:UTR3:LYRM2	chr6	90344706	90344706	T	C	UTR3	LYRM2	ENST000005	.	.	A	G	-	-	0.10204082	44,0,5,0	chr6,903447	Cytosol	A > G
chr6:99321481:T:C:UTR3:FBXL4	chr6	99321481	99321481	T	C	UTR3	FBXL4	ENST000003	.	.	A	G	-	-	0.21428571	11,0,3,0	chr6,993214	Cytosol	A > G
chr6:99321573:T:C:UTR3:FBXL4	chr6	99321573	99321573	T	C	UTR3	FBXL4	ENST000003	.	.	A	G	-	-	0.11538462	23,0,3,0	chr6,993215	Cytosol	A > G
chr6:99321586:T:C:UTR3:FBXL4	chr6	99321586	99321586	T	C	UTR3	FBXL4	ENST000003	.	.	A	G	-	-	0.32258065	21,0,10,0	chr6,993215	Cytosol	A > G
chr6:99981299:A:G:intergenic:TST	chr6	99981299	99981299	A	G	intergenic	TSTD3,CCNC	dist=1505;dis	.	.	A	G	+	+	0.66666667	2,0,4,0	chr6,999812	Cytosol	A > G
chr6:99983089:A:G:intergenic:TST	chr6	99983089	99983089	A	G	intergenic	TSTD3,CCNC	dist=3295;dis	.	.	A	G	+	+	0.18181818	9,0,2,0	chr6,999830	Cytosol	A > G
chr6:99983155:A:G:intergenic:TST	chr6	99983155	99983155	A	G	intergenic	TSTD3,CCNC	dist=3361;dis	.	.	A	G	+	+	0.27272727	8,0,3,0	chr6,999831	Cytosol	A > G
chr6:99983167:A:G:intergenic:TST	chr6	99983167	99983167	A	G	intergenic	TSTD3,CCNC	dist=3373;dis	.	.	A	G	+	+	0.41666667	7,0,5,0	chr6,999831	Cytosol	A > G
chr6:99983187:A:G:intergenic:TST	chr6	99983187	99983187	A	G	intergenic	TSTD3,CCNC	dist=3393;dis	.	.	A	G	+	+	0.22222222	7,0,2,0	chr6,999831	Cytosol	A > G
chr6:99983227:A:G:intergenic:TST	chr6	99983227	99983227	A	G	intergenic	TSTD3,CCNC	dist=3433;dis	.	.	A	G	+	+	0.15384615	11,0,2,0	chr6,999832	Cytosol	A > G
chr6:99983247:A:G:intergenic:TST	chr6	99983247	99983247	A	G	intergenic	TSTD3,CCNC	dist=3453;dis	.	.	A	G	+	+	0.23076923	10,0,3,0	chr6,999832	Cytosol	A > G
chr6:99983252:A:G:intergenic:TST	chr6	99983252	99983252	A	G	intergenic	TSTD3,CCNC	dist=3458;dis	.	.	A	G	+	+	0.36363636	7,0,4,0	chr6,999832	Cytosol	A > G
chr6:99983266:A:G:intergenic:TST	chr6	99983266	99983266	A	G	intergenic	TSTD3,CCNC	dist=3472;dis	.	.	A	G	+	+	0.18181818	9,0,2,0	chr6,999832	Cytosol	A > G
chr6:107113917:A:G:UTR3:QRSL1	chr6	107113917	107113917	A	G	UTR3	QRSL1	ENST000003	.	.	A	G	+	+	0.02597403	75,0,2,0	chr6,107113	Cytosol	A > G
chr6:107113927:A:G:UTR3:QRSL1	chr6	107113927	107113927	A	G	UTR3	QRSL1	ENST000003	.	.	A	G	+	+	0.07042254	66,0,5,0	chr6,107113	Cytosol	A > G
chr6:107115931:A:G:UTR3:QRSL1	chr6	107115931	107115931	A	G	UTR3	QRSL1	ENST000003	.	.	A	G	+	+	0.46666667	8,0,7,0	chr6,107115	Cytosol	A > G

chr6:107115943:A:G:UTR3:QRSL1	chr6	107115943	107115943	A	G	UTR3	QRSL1	ENST000003	.	.	A	G	+	+	0.2	12,0,3,0	chr6,107115	Cytosol	A > G
chr6:107361834:A:G:intronic:C6or	chr6	107361834	107361834	A	G	intronic	C6orf203	.	.	.	A	G	+	+	0.0625	30,0,2,0	chr6,107361	Cytosol	A > G
chr6:107361926:A:G:intronic:C6or	chr6	107361926	107361926	A	G	intronic	C6orf203	.	.	.	A	G	+	+	0.11111111	16,0,2,0	chr6,107361	Cytosol	A > G
chr6:107509500:T:C:intronic:PDSS	chr6	107509500	107509500	T	C	intronic	PDSS2	.	.	.	A	G	-	-	0.4	3,0,2,0	chr6,107509	Cytosol	A > G
chr6:108363371:T:C:UTR3:OSTM1	chr6	108363371	108363371	T	C	UTR3	OSTM1	ENST000001	.	.	A	G	-	-	0.06666667	28,0,2,0	chr6,108363	Cytosol	A > G
chr6:108363376:T:C:UTR3:OSTM1	chr6	108363376	108363376	T	C	UTR3	OSTM1	ENST000001	.	.	A	G	-	-	0.13793103	25,0,4,0	chr6,108363	Cytosol	A > G
chr6:108363453:T:C:UTR3:OSTM1	chr6	108363453	108363453	T	C	UTR3	OSTM1	ENST000001	.	.	A	G	-	-	0.83333333	1,0,5,0	chr6,108363	Cytosol	A > G
chr6:108363758:T:C:UTR3:OSTM1	chr6	108363758	108363758	T	C	UTR3	OSTM1	ENST000001	.	.	A	G	-	-	0.10714286	25,0,3,0	chr6,108363	Cytosol	A > G
chr6:108363889:T:C:UTR3:OSTM1	chr6	108363889	108363889	T	C	UTR3	OSTM1	ENST000001	.	.	A	G	-	-	0.18181818	27,0,6,0	chr6,108363	Cytosol	A > G
chr6:108363895:T:C:UTR3:OSTM1	chr6	108363895	108363895	T	C	UTR3	OSTM1	ENST000001	.	.	A	G	-	-	0.79310345	6,0,23,0	chr6,108363	Cytosol	A > G
chr6:108363897:T:C:UTR3:OSTM1	chr6	108363897	108363897	T	C	UTR3	OSTM1	ENST000001	.	.	A	G	-	-	0.07407407	25,0,2,0	chr6,108363	Cytosol	A > G
chr6:109784286:T:C:UTR3:ZBTB24	chr6	109784286	109784286	T	C	UTR3	ZBTB24	ENST000002	.	.	A	G	-	-	0.29411765	12,0,5,0	chr6,109784	Cytosol	A > G
chr6:109784327:T:C:UTR3:ZBTB24	chr6	109784327	109784327	T	C	UTR3	ZBTB24	ENST000002	.	.	A	G	-	-	0.30769231	9,0,4,0	chr6,109784	Cytosol	A > G
chr6:109784460:T:C:UTR3:ZBTB24	chr6	109784460	109784460	T	C	UTR3	ZBTB24	ENST000002	.	.	A	G	-	-	0.09090909	20,0,2,0	chr6,109784	Cytosol	A > G
chr6:109785887:T:C:UTR3:ZBTB24	chr6	109785887	109785887	T	C	UTR3	ZBTB24	ENST000002	.	.	A	G	-	-	0.05405405	35,0,2,0	chr6,109785	Cytosol	A > G
chr6:109785909:T:C:UTR3:ZBTB24	chr6	109785909	109785909	T	C	UTR3	ZBTB24	ENST000002	.	.	A	G	-	-	0.11764706	30,0,4,0	chr6,109785	Cytosol	A > G
chr6:109785952:T:C:UTR3:ZBTB24	chr6	109785952	109785952	T	C	UTR3	ZBTB24	ENST000002	.	.	A	G	-	-	0.08823529	31,0,3,0	chr6,109785	Cytosol	A > G
chr6:111218772:A:G:intergenic:AI	chr6	111218772	111218772	A	G	intergenic	AMD1,GTF3C	dist=1856;dis	.	.	A	G	+		0.77777778	2,0,7,0	chr6,111218	Cytosol	A > G
chr6:111218773:A:G:intergenic:AI	chr6	111218773	111218773	A	G	intergenic	AMD1,GTF3C	dist=1857;dis	.	.	A	G	+		0.4	6,0,4,0	chr6,111218	Cytosol	A > G
chr6:111218776:A:G:intergenic:AI	chr6	111218776	111218776	A	G	intergenic	AMD1,GTF3C	dist=1860;dis	.	.	A	G	+		0.18181818	9,0,2,0	chr6,111218	Cytosol	A > G
chr6:111218828:A:G:intergenic:AI	chr6	111218828	111218828	A	G	intergenic	AMD1,GTF3C	dist=1912;dis	.	.	A	G	+		0.71428571	2,0,5,0	chr6,111218	Cytosol	A > G
chr6:111218832:A:G:intergenic:AI	chr6	111218832	111218832	A	G	intergenic	AMD1,GTF3C	dist=1916;dis	.	.	A	G	+		0.83333333	1,0,5,0	chr6,111218	Cytosol	A > G
chr6:111218833:A:G:intergenic:AI	chr6	111218833	111218833	A	G	intergenic	AMD1,GTF3C	dist=1917;dis	.	.	A	G	+		0.71428571	2,0,5,0	chr6,111218	Cytosol	A > G
chr6:111218857:A:G:intergenic:AI	chr6	111218857	111218857	A	G	intergenic	AMD1,GTF3C	dist=1941;dis	.	.	A	G	+		0.16666667	10,0,2,0	chr6,111218	Cytosol	A > G
chr6:111218862:A:G:intergenic:AI	chr6	111218862	111218862	A	G	intergenic	AMD1,GTF3C	dist=1946;dis	.	.	A	G	+		0.35714286	9,0,5,0	chr6,111218	Cytosol	A > G
chr6:111291548:A:G:upstream:AL	chr6	111291548	111291548	A	G	upstream	AL357515.1	.	.	.	A	G	+		0.28571429	5,0,2,0	chr6,111291	Cytosol	A > G
chr6:111588770:A:G:UTR3:KIAA19	chr6	111588770	111588770	A	G	UTR3	KIAA1919	ENST000003	.	.	A	G	+	+	0.25	9,0,3,0	chr6,111588	Cytosol	A > G
chr6:111594960:A:G:intergenic:KI	chr6	111594960	111594960	A	G	intergenic	KIAA1919,RP	dist=2590;dis	.	.	A	G	+		0.25	6,0,2,0	chr6,111594	Cytosol	A > G
chr6:136660352:T:C:intergenic:RP	chr6	136660352	136660352	T	C	intergenic	RP3-406A7.7	dist=3127;dis	.	.	A	G	-		0.83333333	1,0,5,0	chr6,136660	Cytosol	A > G
chr6:136660353:T:C:intergenic:RP	chr6	136660353	136660353	T	C	intergenic	RP3-406A7.7	dist=3128;dis	.	.	A	G	-		0.66666667	2,0,4,0	chr6,136660	Cytosol	A > G
chr6:136660411:T:C:intergenic:RP	chr6	136660411	136660411	T	C	intergenic	RP3-406A7.7	dist=3186;dis	.	.	A	G	-		0.4	3,0,2,0	chr6,136660	Cytosol	A > G
chr6:136660447:T:C:intergenic:RP	chr6	136660447	136660447	T	C	intergenic	RP3-406A7.7	dist=3222;dis	.	.	A	G	-		0.33333333	4,0,2,0	chr6,136660	Cytosol	A > G
chr6:136660456:T:C:intergenic:RP	chr6	136660456	136660456	T	C	intergenic	RP3-406A7.7	dist=3231;dis	.	.	A	G	-		0.83333333	1,0,5,0	chr6,136660	Cytosol	A > G
chr6:139113616:A:G:intronic:CCD	chr6	139113616	139113616	A	G	intronic	CCDC28A	.	.	.	A	G	+	+	0.36363636	7,0,4,0	chr6,139113	Cytosol	A > G
chr6:149912224:A:G:UTR3:GINM1	chr6	149912224	149912224	A	G	UTR3	GINM1	ENST000003	.	.	A	G	+	+	0.04237288	113,0,5,0	chr6,149912	Cytosol	A > G
chr6:149912239:A:G:UTR3:GINM1	chr6	149912239	149912239	A	G	UTR3	GINM1	ENST000003	.	.	A	G	+	+	0.04424779	108,0,5,0	chr6,149912	Cytosol	A > G
chr6:149912278:A:G:UTR3:GINM1	chr6	149912278	149912278	A	G	UTR3	GINM1	ENST000003	.	.	A	G	+	+	0.08641975	74,0,7,0	chr6,149912	Cytosol	A > G
chr6:149912281:A:G:UTR3:GINM1	chr6	149912281	149912281	A	G	UTR3	GINM1	ENST000003	.	.	A	G	+	+	0.07042254	66,0,5,0	chr6,149912	Cytosol	A > G
chr6:149912307:A:G:UTR3:GINM1	chr6	149912307	149912307	A	G	UTR3	GINM1	ENST000003	.	.	A	G	+	+	0.11688312	68,0,9,0	chr6,149912	Cytosol	A > G
chr6:149912377:A:G:UTR3:GINM1	chr6	149912377	149912377	A	G	UTR3	GINM1	ENST000003	.	.	A	G	+	+	0.04109589	70,0,3,0	chr6,149912	Cytosol	A > G
chr6:149912378:A:G:UTR3:GINM1	chr6	149912378	149912378	A	G	UTR3	GINM1	ENST000003	.	.	A	G	+	+	0.05194805	73,0,4,0	chr6,149912	Cytosol	A > G
chr6:150045747:T:C:UTR3:NUP43	chr6	150045747	150045747	T	C	UTR3	NUP43	ENST000003	.	.	A	G	-	-	0.11538462	92,0,12,0	chr6,150045	Cytosol	A > G
chr6:150045748:T:C:UTR3:NUP43	chr6	150045748	150045748	T	C	UTR3	NUP43	ENST000003	.	.	A	G	-	-	0.32727273	74,0,36,0	chr6,150045	Cytosol	A > G
chr6:150045749:T:C:UTR3:NUP43	chr6	150045749	150045749	T	C	UTR3	NUP43	ENST000003	.	.	A	G	-	-	0.25	75,0,25,0	chr6,150045	Cytosol	A > G
chr6:150045750:T:C:UTR3:NUP43	chr6	150045750	150045750	T	C	UTR3	NUP43	ENST000003	.	.	A	G	-	-	0.22522523	86,0,25,0	chr6,150045	Cytosol	A > G
chr6:150045755:T:C:UTR3:NUP43	chr6	150045755	150045755	T	C	UTR3	NUP43	ENST000003	.	.	A	G	-	-	0.67961165	33,0,70,0	chr6,150045	Cytosol	A > G
chr6:150045756:T:C:UTR3:NUP43	chr6	150045756	150045756	T	C	UTR3	NUP43	ENST000003	.	.	A	G	-	-	0.46	54,0,46,0	chr6,150045	Cytosol	A > G
chr6:150045788:T:C:UTR3:NUP43	chr6	150045788	150045788	T	C	UTR3	NUP43	ENST000003	.	.	A	G	-	-	0.7826087	15,0,54,0	chr6,150045	Cytosol	A > G
chr6:150045796:T:C:UTR3:NUP43	chr6	150045796	150045796	T	C	UTR3	NUP43	ENST000003	.	.	A	G	-	-	0.1	54,0,6,0	chr6,150045	Cytosol	A > G
chr6:150045816:T:C:UTR3:NUP43	chr6	150045816	150045816	T	C	UTR3	NUP43	ENST000003	.	.	A	G	-	-	0.31481481	37,0,17,0	chr6,150045	Cytosol	A > G
chr6:150045817:T:C:UTR3:NUP43	chr6	150045817	150045817	T	C	UTR3	NUP43	ENST000003	.	.	A	G	-	-	0.26923077	38,0,14,0	chr6,150045	Cytosol	A > G
chr6:150045818:T:C:UTR3:NUP43	chr6	150045818	150045818	T	C	UTR3	NUP43	ENST000003	.	.	A	G	-	-	0.68421053	18,0,39,0	chr6,150045	Cytosol	A > G
chr6:150045825:T:C:UTR3:NUP43	chr6	150045825	150045825	T	C	UTR3	NUP43	ENST000003	.	.	A	G	-	-	0.12903226	54,0,8,0	chr6,150045	Cytosol	A > G
chr6:150045835:T:C:UTR3:NUP43	chr6	150045835	150045835	T	C	UTR3	NUP43	ENST000003	.	.	A	G	-	-	0.03030303	64,0,2,0	chr6,150045	Cytosol	A > G
chr6:150045841:T:C:UTR3:NUP43	chr6	150045841	150045841	T	C	UTR3	NUP43	ENST000003	.	.	A	G	-	-	0.07692308	60,0,5,0	chr6,150045	Cytosol	A > G
chr6:150045848:T:C:UTR3:NUP43	chr6	150045848	150045848	T	C	UTR3	NUP43	ENST000003	.	.	A	G	-	-	0.62318841	26,0,43,0	chr6,150045	Cytosol	A > G
chr6:150045852:T:C:UTR3:NUP43	chr6	150045852	150045852	T	C	UTR3	NUP43	ENST000003	.	.	A	G	-	-	0.11666667	53,0,7,0	chr6,150045	Cytosol	A > G
chr6:150045857:T:C:UTR3:NUP43	chr6	150045857	150045857	T	C	UTR3	NUP43	ENST000003	.	.	A	G	-	-	0.47540984	32,0,29,0	chr6,150045	Cytosol	A > G
chr6:150045858:T:C:UTR3:NUP43	chr6	150045858	150045858	T	C	UTR3	NUP43	ENST000003	.	.	A	G	-	-	0.59016393	25,0,36,0	chr6,150045	Cytosol	A > G
chr6:150045860:T:C:UTR3:NUP43	chr6	150045860	150045860	T	C	UTR3	NUP43	ENST000003	.	.	A	G	-	-	0.76666667	14,0,46,0	chr6,150045	Cytosol	A > G
chr6:150045868:T:C:UTR3:NUP43	chr6	150045868	150045868	T	C	UTR3	NUP43	ENST000003	.	.	A	G	-	-	0.24489796	37,0,12,0	chr6,150045	Cytosol	A > G
chr6:150045883:T:C:UTR3:NUP43	chr6	150045883	150045883	T	C	UTR3	NUP43	ENST000003	.	.	A	G	-	-	0.27272727	48,0,18,0	chr6,150045	Cytosol	A > G
chr6:150045884:T:C:UTR3:NUP43	chr6	150045884	150045884	T	C	UTR3	NUP43	ENST000003	.	.	A	G	-	-	0.32394366	48,0,23,0	chr6,150045	Cytosol	A > G
chr6:150045900:T:C:UTR3:NUP43	chr6	150045900	150045900	T	C	UTR3	NUP43	ENST000003	.	.	A	G	-	-	0.03448276	56,0,2,0	chr6,150045	Cytosol	A > G

chr6:150045907:T:C:UTR3:NUP43	chr6	150045907	150045907	T	C	UTR3	NUP43	ENST000003	.	.	A	G	-	-	0.08695652	63,0,6,0	chr6,150045	Cytosol	A > G
chr6:150045918:T:C:UTR3:NUP43	chr6	150045918	150045918	T	C	UTR3	NUP43	ENST000003	.	.	A	G	-	-	0.5443038	36,0,43,0	chr6,150045	Cytosol	A > G
chr6:150045919:T:C:UTR3:NUP43	chr6	150045919	150045919	T	C	UTR3	NUP43	ENST000003	.	.	A	G	-	-	0.3258427	60,0,29,0	chr6,150045	Cytosol	A > G
chr6:150045920:T:C:UTR3:NUP43	chr6	150045920	150045920	T	C	UTR3	NUP43	ENST000003	.	.	A	G	-	-	0.28089888	64,0,25,0	chr6,150045	Cytosol	A > G
chr6:150045926:T:C:UTR3:NUP43	chr6	150045926	150045926	T	C	UTR3	NUP43	ENST000003	.	.	A	G	-	-	0.04395604	87,0,4,0	chr6,150045	Cytosol	A > G
chr6:150045928:T:C:UTR3:NUP43	chr6	150045928	150045928	T	C	UTR3	NUP43	ENST000003	.	.	A	G	-	-	0.65979381	32,1,64,0	chr6,150045	Cytosol	A > G
chr6:150045929:T:C:UTR3:NUP43	chr6	150045929	150045929	T	C	UTR3	NUP43	ENST000003	.	.	A	G	-	-	0.27368421	69,0,26,0	chr6,150045	Cytosol	A > G
chr6:150045951:T:C:UTR3:NUP43	chr6	150045951	150045951	T	C	UTR3	NUP43	ENST000003	.	.	A	G	-	-	0.27848101	57,0,22,0	chr6,150045	Cytosol	A > G
chr6:150045952:T:C:UTR3:NUP43	chr6	150045952	150045952	T	C	UTR3	NUP43	ENST000003	.	.	A	G	-	-	0.0375	77,0,3,0	chr6,150045	Cytosol	A > G
chr6:150045957:T:C:UTR3:NUP43	chr6	150045957	150045957	T	C	UTR3	NUP43	ENST000003	.	.	A	G	-	-	0.10843373	74,0,9,0	chr6,150045	Cytosol	A > G
chr6:150045989:T:C:UTR3:NUP43	chr6	150045989	150045989	T	C	UTR3	NUP43	ENST000003	.	.	A	G	-	-	0.12658228	69,0,10,0	chr6,150045	Cytosol	A > G
chr6:150045997:T:C:UTR3:NUP43	chr6	150045997	150045997	T	C	UTR3	NUP43	ENST000003	.	.	A	G	-	-	0.07692308	84,0,7,0	chr6,150045	Cytosol	A > G
chr6:150045998:T:C:UTR3:NUP43	chr6	150045998	150045998	T	C	UTR3	NUP43	ENST000003	.	.	A	G	-	-	0.57894737	40,0,55,0	chr6,150045	Cytosol	A > G
chr6:150046006:T:C:UTR3:NUP43	chr6	150046006	150046006	T	C	UTR3	NUP43	ENST000003	.	.	A	G	-	-	0.1097561	73,0,9,0	chr6,150046	Cytosol	A > G
chr6:150046020:T:C:UTR3:NUP43	chr6	150046020	150046020	T	C	UTR3	NUP43	ENST000003	.	.	A	G	-	-	0.04950495	96,0,5,0	chr6,150046	Cytosol	A > G
chr6:150046340:T:C:UTR3:NUP43	chr6	150046340	150046340	T	C	UTR3	NUP43	ENST000003	.	.	A	G	-	-	0.10989011	81,0,10,0	chr6,150046	Cytosol	A > G
chr6:150046349:T:C:UTR3:NUP43	chr6	150046349	150046349	T	C	UTR3	NUP43	ENST000003	.	.	A	G	-	-	0.54054054	34,0,40,0	chr6,150046	Cytosol	A > G
chr6:150046415:T:C:UTR3:NUP43	chr6	150046415	150046415	T	C	UTR3	NUP43	ENST000003	.	.	A	G	-	-	0.16176471	57,0,11,0	chr6,150046	Cytosol	A > G
chr6:150046432:T:C:UTR3:NUP43	chr6	150046432	150046432	T	C	UTR3	NUP43	ENST000003	.	.	A	G	-	-	0.4375	54,0,42,0	chr6,150046	Cytosol	A > G
chr6:150046435:T:C:UTR3:NUP43	chr6	150046435	150046435	T	C	UTR3	NUP43	ENST000003	.	.	A	G	-	-	0.0173913	113,0,2,0	chr6,150046	Cytosol	A > G
chr6:150046471:T:C:UTR3:NUP43	chr6	150046471	150046471	T	C	UTR3	NUP43	ENST000003	.	.	A	G	-	-	0.02272727	129,0,3,0	chr6,150046	Cytosol	A > G
chr6:150046484:T:C:UTR3:NUP43	chr6	150046484	150046484	T	C	UTR3	NUP43	ENST000003	.	.	A	G	-	-	0.14728682	109,0,19,1	chr6,150046	Cytosol	A > G
chr6:150046485:T:C:UTR3:NUP43	chr6	150046485	150046485	T	C	UTR3	NUP43	ENST000003	.	.	A	G	-	-	0.14179104	115,0,19,0	chr6,150046	Cytosol	A > G
chr6:150046486:T:C:UTR3:NUP43	chr6	150046486	150046486	T	C	UTR3	NUP43	ENST000003	.	.	A	G	-	-	0.03355705	144,0,5,0	chr6,150046	Cytosol	A > G
chr6:150046495:T:C:UTR3:NUP43	chr6	150046495	150046495	T	C	UTR3	NUP43	ENST000003	.	.	A	G	-	-	0.5326087	43,0,49,0	chr6,150046	Cytosol	A > G
chr6:150046545:T:C:UTR3:NUP43	chr6	150046545	150046545	T	C	UTR3	NUP43	ENST000003	.	.	A	G	-	-	0.75238095	26,0,79,0	chr6,150046	Cytosol	A > G
chr6:150046559:T:C:UTR3:NUP43	chr6	150046559	150046559	T	C	UTR3	NUP43	ENST000003	.	.	A	G	-	-	0.95714286	6,0,134,0	chr6,150046	Cytosol	A > G
chr6:150046562:T:C:UTR3:NUP43	chr6	150046562	150046562	T	C	UTR3	NUP43	ENST000003	.	.	A	G	-	-	0.48175182	71,0,66,0	chr6,150046	Cytosol	A > G
chr6:150046581:T:C:UTR3:NUP43	chr6	150046581	150046581	T	C	UTR3	NUP43	ENST000003	.	.	A	G	-	-	0.09565217	104,0,11,0	chr6,150046	Cytosol	A > G
chr6:150046601:T:C:UTR3:NUP43	chr6	150046601	150046601	T	C	UTR3	NUP43	ENST000003	.	.	A	G	-	-	0.50515464	48,0,49,0	chr6,150046	Cytosol	A > G
chr6:150046603:T:C:UTR3:NUP43	chr6	150046603	150046603	T	C	UTR3	NUP43	ENST000003	.	.	A	G	-	-	0.67961165	32,1,70,0	chr6,150046	Cytosol	A > G
chr6:150046613:T:C:UTR3:NUP43	chr6	150046613	150046613	T	C	UTR3	NUP43	ENST000003	.	.	A	G	-	-	0.52083333	46,0,50,0	chr6,150046	Cytosol	A > G
chr6:150046638:T:C:UTR3:NUP43	chr6	150046638	150046638	T	C	UTR3	NUP43	ENST000003	.	.	A	G	-	-	0.55	36,0,44,0	chr6,150046	Cytosol	A > G
chr6:150046639:T:C:UTR3:NUP43	chr6	150046639	150046639	T	C	UTR3	NUP43	ENST000003	.	.	A	G	-	-	0.20987654	64,0,17,0	chr6,150046	Cytosol	A > G
chr6:150046642:T:C:UTR3:NUP43	chr6	150046642	150046642	T	C	UTR3	NUP43	ENST000003	.	.	A	G	-	-	0.5125	39,0,41,0	chr6,150046	Cytosol	A > G
chr6:150046643:T:C:UTR3:NUP43	chr6	150046643	150046643	T	C	UTR3	NUP43	ENST000003	.	.	A	G	-	-	0.53846154	36,0,42,0	chr6,150046	Cytosol	A > G
chr6:150046649:T:C:UTR3:NUP43	chr6	150046649	150046649	T	C	UTR3	NUP43	ENST000003	.	.	A	G	-	-	0.04545455	105,0,5,0	chr6,150046	Cytosol	A > G
chr6:150046673:T:C:UTR3:NUP43	chr6	150046673	150046673	T	C	UTR3	NUP43	ENST000003	.	.	A	G	-	-	0.71774194	35,0,89,0	chr6,150046	Cytosol	A > G
chr6:150046678:T:C:UTR3:NUP43	chr6	150046678	150046678	T	C	UTR3	NUP43	ENST000003	.	.	A	G	-	-	0.15447154	104,0,19,0	chr6,150046	Cytosol	A > G
chr6:150046681:T:C:UTR3:NUP43	chr6	150046681	150046681	T	C	UTR3	NUP43	ENST000003	.	.	A	G	-	-	0.01652893	119,0,2,0	chr6,150046	Cytosol	A > G
chr6:150046686:T:C:UTR3:NUP43	chr6	150046686	150046686	T	C	UTR3	NUP43	ENST000003	.	.	A	G	-	-	0.54700855	53,0,64,0	chr6,150046	Cytosol	A > G
chr6:150046687:T:C:UTR3:NUP43	chr6	150046687	150046687	T	C	UTR3	NUP43	ENST000003	.	.	A	G	-	-	0.07619048	97,0,8,0	chr6,150046	Cytosol	A > G
chr6:150046736:T:C:UTR3:NUP43	chr6	150046736	150046736	T	C	UTR3	NUP43	ENST000003	.	.	A	G	-	-	0.89655172	3,0,26,0	chr6,150046	Cytosol	A > G
chr6:150046741:T:C:UTR3:NUP43	chr6	150046741	150046741	T	C	UTR3	NUP43	ENST000003	.	.	A	G	-	-	0.15	17,0,3,0	chr6,150046	Cytosol	A > G
chr6:150047287:T:C:UTR3:NUP43	chr6	150047287	150047287	T	C	UTR3	NUP43	ENST000003	.	.	A	G	-	-	0.02564103	76,0,2,0	chr6,150047	Cytosol	A > G
chr6:150047362:T:C:UTR3:NUP43	chr6	150047362	150047362	T	C	UTR3	NUP43	ENST000003	.	.	A	G	-	-	0.33333333	16,0,8,0	chr6,150047	Cytosol	A > G
chr6:150047370:T:C:UTR3:NUP43	chr6	150047370	150047370	T	C	UTR3	NUP43	ENST000003	.	.	A	G	-	-	0.18518519	22,0,5,0	chr6,150047	Cytosol	A > G
chr6:150047376:T:C:UTR3:NUP43	chr6	150047376	150047376	T	C	UTR3	NUP43	ENST000003	.	.	A	G	-	-	0.43333333	17,0,13,0	chr6,150047	Cytosol	A > G
chr6:150047379:T:C:UTR3:NUP43	chr6	150047379	150047379	T	C	UTR3	NUP43	ENST000003	.	.	A	G	-	-	0.12195122	36,0,5,0	chr6,150047	Cytosol	A > G
chr6:150047418:T:C:UTR3:NUP43	chr6	150047418	150047418	T	C	UTR3	NUP43	ENST000003	.	.	A	G	-	-	0.11320755	47,0,6,0	chr6,150047	Cytosol	A > G
chr6:150047420:T:C:UTR3:NUP43	chr6	150047420	150047420	T	C	UTR3	NUP43	ENST000003	.	.	A	G	-	-	0.05555556	51,0,3,0	chr6,150047	Cytosol	A > G
chr6:150047430:T:C:UTR3:NUP43	chr6	150047430	150047430	T	C	UTR3	NUP43	ENST000003	.	.	A	G	-	-	0.05660377	50,0,3,0	chr6,150047	Cytosol	A > G
chr6:150047455:T:C:UTR3:NUP43	chr6	150047455	150047455	T	C	UTR3	NUP43	ENST000003	.	.	A	G	-	-	0.03296703	88,0,3,0	chr6,150047	Cytosol	A > G
chr6:150047459:T:C:UTR3:NUP43	chr6	150047459	150047459	T	C	UTR3	NUP43	ENST000003	.	.	A	G	-	-	0.03333333	87,0,3,0	chr6,150047	Cytosol	A > G
chr6:150047460:T:C:UTR3:NUP43	chr6	150047460	150047460	T	C	UTR3	NUP43	ENST000003	.	.	A	G	-	-	0.06666667	84,0,6,0	chr6,150047	Cytosol	A > G
chr6:150047466:T:C:UTR3:NUP43	chr6	150047466	150047466	T	C	UTR3	NUP43	ENST000003	.	.	A	G	-	-	0.12195122	72,0,10,0	chr6,150047	Cytosol	A > G
chr6:150047495:T:C:UTR3:NUP43	chr6	150047495	150047495	T	C	UTR3	NUP43	ENST000003	.	.	A	G	-	-	0.19298246	46,0,11,0	chr6,150047	Cytosol	A > G
chr6:150142163:T:C:intronic:LRP1	chr6	150142163	150142163	T	C	intronic	LRP11	.	.	.	A	G	-	-	0.33333333	4,0,2,0	chr6,150142	Cytosol	A > G
chr6:150142338:T:C:intronic:LRP1	chr6	150142338	150142338	T	C	intronic	LRP11	.	.	.	A	G	-	-	1	0,0,11,0	chr6,150142	Cytosol	A > G
chr6:150143227:T:C:intronic:LRP1	chr6	150143227	150143227	T	C	intronic	LRP11	.	.	.	A	G	-	-	0.30769231	9,0,4,0	chr6,150143	Cytosol	A > G
chr6:150143311:T:C:intronic:LRP1	chr6	150143311	150143311	T	C	intronic	LRP11	.	.	.	A	G	-	-	0.28571429	5,0,2,0	chr6,150143	Cytosol	A > G
chr6:150143364:T:C:intronic:LRP1	chr6	150143364	150143364	T	C	intronic	LRP11	.	.	.	A	G	-	-	0.78571429	3,0,11,0	chr6,150143	Cytosol	A > G
chr6:150143381:T:C:intronic:LRP1	chr6	150143381	150143381	T	C	intronic	LRP11	.	.	.	A	G	-	-	0.76470588	4,0,13,0	chr6,150143	Cytosol	A > G
chr6:150143462:T:C:intronic:LRP1	chr6	150143462	150143462	T	C	intronic	LRP11	.	.	.	A	G	-	-	0.71428571	2,0,5,0	chr6,150143	Cytosol	A > G

chr6:150143470:T:C:intronic:LRP1	chr6	150143470	150143470	T	C	intronic	LRP11	.	.	.	A	G	-	-	0.875	1,0,7,0	chr6,150143	Cytosol	A > G
chr6:150143474:T:C:intronic:LRP1	chr6	150143474	150143474	T	C	intronic	LRP11	.	.	.	A	G	-	-	0.27272727	8,0,3,0	chr6,150143	Cytosol	A > G
chr6:150143568:T:C:intronic:LRP1	chr6	150143568	150143568	T	C	intronic	LRP11	.	.	.	A	G	-	-	0.2173913	18,0,5,0	chr6,150143	Cytosol	A > G
chr6:150143624:T:C:intronic:LRP1	chr6	150143624	150143624	T	C	intronic	LRP11	.	.	.	A	G	-	-	0.45	11,0,9,0	chr6,150143	Cytosol	A > G
chr6:150143637:T:C:intronic:LRP1	chr6	150143637	150143637	T	C	intronic	LRP11	.	.	.	A	G	-	-	0.4	3,0,2,0	chr6,150143	Cytosol	A > G
chr6:150146894:T:C:intronic:LRP1	chr6	150146894	150146894	T	C	intronic	LRP11	.	.	.	A	G	-	-	0.45454545	6,0,5,0	chr6,150146	Cytosol	A > G
chr6:150146949:T:C:intronic:LRP1	chr6	150146949	150146949	T	C	intronic	LRP11	.	.	.	A	G	-	-	0.75	2,0,6,0	chr6,150146	Cytosol	A > G
chr6:150146953:T:C:intronic:LRP1	chr6	150146953	150146953	T	C	intronic	LRP11	.	.	.	A	G	-	-	0.33333333	6,0,3,0	chr6,150146	Cytosol	A > G
chr6:150146962:T:C:intronic:LRP1	chr6	150146962	150146962	T	C	intronic	LRP11	.	.	.	A	G	-	-	0.22222222	7,0,2,0	chr6,150146	Cytosol	A > G
chr6:150146967:T:C:intronic:LRP1	chr6	150146967	150146967	T	C	intronic	LRP11	.	.	.	A	G	-	-	0.22222222	7,0,2,0	chr6,150146	Cytosol	A > G
chr6:150147035:T:C:intronic:LRP1	chr6	150147035	150147035	T	C	intronic	LRP11	.	.	.	A	G	-	-	0.33333333	4,0,2,0	chr6,150147	Cytosol	A > G
chr6:160100856:T:C:UTR3:SOD2	chr6	160100856	160100856	T	C	UTR3	SOD2	ENST000005	.	.	A	G	-	-	0.04411765	130,0,6,0	chr6,160100	Cytosol	A > G
chr6:160100882:T:C:UTR3:SOD2	chr6	160100882	160100882	T	C	UTR3	SOD2	ENST000005	.	.	A	G	-	-	0.23846154	99,0,31,0	chr6,160100	Cytosol	A > G
chr6:160100910:T:C:UTR3:SOD2	chr6	160100910	160100910	T	C	UTR3	SOD2	ENST000005	.	.	A	G	-	-	0.05454545	208,0,12,0	chr6,160100	Cytosol	A > G
chr7:1205200:A:G:downstream:A(	chr7	1205200	1205200	A	G	downstream	AC091729.9	.	.	.	A	G	+	+	0.29411765	12,0,5,0	chr7,120519	Cytosol	A > G
chr7:2487169:A:G:ncRNA_exonic:	chr7	2487169	2487169	A	G	ncRNA_exon	AC004840.9	.	.	.	A	G	+	+	0.66666667	2,0,4,0	chr7,248716	Cytosol	A > G
chr7:2487201:A:G:ncRNA_exonic:	chr7	2487201	2487201	A	G	ncRNA_exon	AC004840.9	.	.	.	A	G	+	+	0.25	6,0,2,0	chr7,248720	Cytosol	A > G
chr7:2487247:A:G:ncRNA_exonic:	chr7	2487247	2487247	A	G	ncRNA_exon	AC004840.9	.	.	.	A	G	+	+	0.27272727	8,0,3,0	chr7,248724	Cytosol	A > G
chr7:2762074:T:C:intergenic:AMZ1	chr7	2762074	2762074	T	C	intergenic	AMZ1,GNA12	dist=5911;dis	.	.	A	G	-	+	0.25	6,0,2,0	chr7,276207	Cytosol	A > G
chr7:2762075:T:C:intergenic:AMZ1	chr7	2762075	2762075	T	C	intergenic	AMZ1,GNA12	dist=5912;dis	.	.	A	G	-	+	0.28571429	5,0,2,0	chr7,276207	Cytosol	A > G
chr7:2767280:T:C:downstream:GN	chr7	2767280	2767280	T	C	downstream	GNA12	.	.	.	A	G	-	+	0.4	3,0,2,0	chr7,276727	Cytosol	A > G
chr7:2767597:T:C:downstream:GN	chr7	2767597	2767597	T	C	downstream	GNA12	.	.	.	A	G	-	+	0.6	2,0,3,0	chr7,276759	Cytosol	A > G
chr7:4809189:A:G:UTR3:FOXK1	chr7	4809189	4809189	A	G	UTR3	FOXK1	ENST000003	.	.	A	G	+	+	0.17857143	23,0,5,0	chr7,480918	Cytosol	A > G
chr7:4809210:A:G:UTR3:FOXK1	chr7	4809210	4809210	A	G	UTR3	FOXK1	ENST000003	.	.	A	G	+	+	0.125	28,0,4,0	chr7,480920	Cytosol	A > G
chr7:4809324:A:G:UTR3:FOXK1	chr7	4809324	4809324	A	G	UTR3	FOXK1	ENST000003	.	.	A	G	+	+	0.25581395	32,0,11,0	chr7,480932	Cytosol	A > G
chr7:4809334:A:G:UTR3:FOXK1	chr7	4809334	4809334	A	G	UTR3	FOXK1	ENST000003	.	.	A	G	+	+	0.07272727	51,0,4,0	chr7,480933	Cytosol	A > G
chr7:4809911:A:G:UTR3:FOXK1	chr7	4809911	4809911	A	G	UTR3	FOXK1	ENST000003	.	.	A	G	+	+	0.13888889	31,0,5,0	chr7,480991	Cytosol	A > G
chr7:4810015:A:G:UTR3:FOXK1	chr7	4810015	4810015	A	G	UTR3	FOXK1	ENST000003	.	.	A	G	+	+	0.33333333	6,0,3,0	chr7,481001	Cytosol	A > G
chr7:4810019:A:G:UTR3:FOXK1	chr7	4810019	4810019	A	G	UTR3	FOXK1	ENST000003	.	.	A	G	+	+	0.8	1,0,4,0	chr7,481001	Cytosol	A > G
chr7:4810042:A:G:UTR3:FOXK1	chr7	4810042	4810042	A	G	UTR3	FOXK1	ENST000003	.	.	A	G	+	+	0.22222222	7,0,2,0	chr7,481004	Cytosol	A > G
chr7:4810144:A:G:UTR3:FOXK1	chr7	4810144	4810144	A	G	UTR3	FOXK1	ENST000003	.	.	A	G	+	+	0.18181818	9,0,2,0	chr7,481014	Cytosol	A > G
chr7:5516630:T:C:ncRNA_exonic:A	chr7	5516630	5516630	T	C	ncRNA_exon	AC092171.4	.	.	.	A	G	-	-+	0.54716981	24,0,29,0	chr7,551662	Cytosol	A > G
chr7:5517083:T:C:ncRNA_intronic:	chr7	5517083	5517083	T	C	ncRNA_intro	AC092171.4	.	.	.	A	G	-	-+	0.26153846	96,0,34,0	chr7,551708	Cytosol	A > G
chr7:5888084:A:G:ncRNA_intronic	chr7	5888084	5888084	A	G	ncRNA_intro	ZNF815P	.	.	.	A	G	+	+	0.23076923	10,0,3,0	chr7,588808	Cytosol	A > G
chr7:5888102:A:G:ncRNA_intronic	chr7	5888102	5888102	A	G	ncRNA_intro	ZNF815P	.	.	.	A	G	+	+	0.7	3,0,7,0	chr7,588810	Cytosol	A > G
chr7:5888119:A:G:ncRNA_intronic	chr7	5888119	5888119	A	G	ncRNA_intro	ZNF815P	.	.	.	A	G	+	+	0.16666667	10,0,2,0	chr7,588811	Cytosol	A > G
chr7:5888169:A:G:ncRNA_intronic	chr7	5888169	5888169	A	G	ncRNA_intro	ZNF815P	.	.	.	A	G	+	+	0.92857143	1,0,13,0	chr7,588816	Cytosol	A > G
chr7:6468256:T:C:intronic:DAGLB	chr7	6468256	6468256	T	C	intronic	DAGLB	.	.	.	A	G	-	-	0.33333333	8,0,4,0	chr7,646825	Cytosol	A > G
chr7:6515026:T:C:intronic:DAGLB,	chr7	6515026	6515026	T	C	intronic	DAGLB,KDELI	.	.	.	A	G	-	-	0.27272727	16,0,6,0	chr7,651502	Cytosol	A > G
chr7:6623622:A:G:ncRNA_intronic	chr7	6623622	6623622	A	G	ncRNA_intro	AC079742.4	.	.	.	A	G	+	+-	0.27272727	8,0,3,0	chr7,662362	Cytosol	A > G
chr7:17384334:A:G:UTR3:AHR	chr7	17384334	17384334	A	G	UTR3	AHR	ENST000002	.	.	A	G	+	+	0.1025641	35,0,4,0	chr7,173843	Cytosol	A > G
chr7:17384353:A:G:UTR3:AHR	chr7	17384353	17384353	A	G	UTR3	AHR	ENST000002	.	.	A	G	+	+	0.35	13,0,7,0	chr7,173843	Cytosol	A > G
chr7:17384354:A:G:UTR3:AHR	chr7	17384354	17384354	A	G	UTR3	AHR	ENST000002	.	.	A	G	+	+	0.95652174	1,0,22,0	chr7,173843	Cytosol	A > G
chr7:17384358:A:G:UTR3:AHR	chr7	17384358	17384358	A	G	UTR3	AHR	ENST000002	.	.	A	G	+	+	0.30434783	16,0,7,0	chr7,173843	Cytosol	A > G
chr7:17384371:A:G:UTR3:AHR	chr7	17384371	17384371	A	G	UTR3	AHR	ENST000002	.	.	A	G	+	+	0.16666667	15,0,3,0	chr7,173843	Cytosol	A > G
chr7:17384393:A:G:UTR3:AHR	chr7	17384393	17384393	A	G	UTR3	AHR	ENST000002	.	.	A	G	+	+	0.25581395	32,0,11,0	chr7,173843	Cytosol	A > G
chr7:17384395:A:G:UTR3:AHR	chr7	17384395	17384395	A	G	UTR3	AHR	ENST000002	.	.	A	G	+	+	0.25581395	32,0,11,0	chr7,173843	Cytosol	A > G
chr7:17384428:A:G:UTR3:AHR	chr7	17384428	17384428	A	G	UTR3	AHR	ENST000002	.	.	A	G	+	+	0.06451613	58,0,4,0	chr7,173844	Cytosol	A > G
chr7:17384434:A:G:UTR3:AHR	chr7	17384434	17384434	A	G	UTR3	AHR	ENST000002	.	.	A	G	+	+	0.19642857	45,0,11,0	chr7,173844	Cytosol	A > G
chr7:17384437:A:G:UTR3:AHR	chr7	17384437	17384437	A	G	UTR3	AHR	ENST000002	.	.	A	G	+	+	0.69230769	16,0,36,0	chr7,173844	Cytosol	A > G
chr7:17384440:A:G:UTR3:AHR	chr7	17384440	17384440	A	G	UTR3	AHR	ENST000002	.	.	A	G	+	+	0.74418605	11,0,32,0	chr7,173844	Cytosol	A > G
chr7:17384441:A:G:UTR3:AHR	chr7	17384441	17384441	A	G	UTR3	AHR	ENST000002	.	.	A	G	+	+	0.07317073	38,0,3,0	chr7,173844	Cytosol	A > G
chr7:17384514:A:G:UTR3:AHR	chr7	17384514	17384514	A	G	UTR3	AHR	ENST000002	.	.	A	G	+	+	0.11111111	24,0,3,0	chr7,173845	Cytosol	A > G
chr7:17384515:A:G:UTR3:AHR	chr7	17384515	17384515	A	G	UTR3	AHR	ENST000002	.	.	A	G	+	+	0.11111111	24,0,3,0	chr7,173845	Cytosol	A > G
chr7:17384527:A:G:UTR3:AHR	chr7	17384527	17384527	A	G	UTR3	AHR	ENST000002	.	.	A	G	+	+	0.22222222	42,0,12,0	chr7,173845	Cytosol	A > G
chr7:17384686:A:G:UTR3:AHR	chr7	17384686	17384686	A	G	UTR3	AHR	ENST000002	.	.	A	G	+	+	0.10526316	17,0,2,0	chr7,173846	Cytosol	A > G
chr7:17384730:A:G:UTR3:AHR	chr7	17384730	17384730	A	G	UTR3	AHR	ENST000002	.	.	A	G	+	+	0.58333333	10,0,14,0	chr7,173847	Cytosol	A > G
chr7:17384763:A:G:UTR3:AHR	chr7	17384763	17384763	A	G	UTR3	AHR	ENST000002	.	.	A	G	+	+	0.19047619	17,0,4,0	chr7,173847	Cytosol	A > G
chr7:17384774:A:G:UTR3:AHR	chr7	17384774	17384774	A	G	UTR3	AHR	ENST000002	.	.	A	G	+	+	0.5	11,0,11,0	chr7,173847	Cytosol	A > G
chr7:17384778:A:G:UTR3:AHR	chr7	17384778	17384778	A	G	UTR3	AHR	ENST000002	.	.	A	G	+	+	0.42105263	11,0,8,0	chr7,173847	Cytosol	A > G
chr7:17384779:A:G:UTR3:AHR	chr7	17384779	17384779	A	G	UTR3	AHR	ENST000002	.	.	A	G	+	+	0.44444444	10,0,8,0	chr7,173847	Cytosol	A > G
chr7:17384781:A:G:UTR3:AHR	chr7	17384781	17384781	A	G	UTR3	AHR	ENST000002	.	.	A	G	+	+	0.16666667	15,0,3,0	chr7,173847	Cytosol	A > G
chr7:17384783:A:G:UTR3:AHR	chr7	17384783	17384783	A	G	UTR3	AHR	ENST000002	.	.	A	G	+	+	0.41176471	10,0,7,0	chr7,173847	Cytosol	A > G
chr7:17384792:A:G:UTR3:AHR	chr7	17384792	17384792	A	G	UTR3	AHR	ENST000002	.	.	A	G	+	+	0.25	15,0,5,0	chr7,173847	Cytosol	A > G

chr7:17384811:A:G:UTR3:AHR	chr7	17384811	17384811	A	G	UTR3	AHR	ENST000002	.	.	A	G	+	+	0.18181818	18,0,4,0	chr7,173848	Cytosol	A > G
chr7:17384818:A:G:UTR3:AHR	chr7	17384818	17384818	A	G	UTR3	AHR	ENST000002	.	.	A	G	+	+	0.74193548	8,0,23,0	chr7,173848	Cytosol	A > G
chr7:17384828:A:G:UTR3:AHR	chr7	17384828	17384828	A	G	UTR3	AHR	ENST000002	.	.	A	G	+	+	0.21621622	29,0,8,0	chr7,173848	Cytosol	A > G
chr7:17384832:A:G:UTR3:AHR	chr7	17384832	17384832	A	G	UTR3	AHR	ENST000002	.	.	A	G	+	+	0.64864865	13,0,24,0	chr7,173848	Cytosol	A > G
chr7:17384845:A:G:UTR3:AHR	chr7	17384845	17384845	A	G	UTR3	AHR	ENST000002	.	.	A	G	+	+	0.11111111	32,0,4,0	chr7,173848	Cytosol	A > G
chr7:17384880:A:G:UTR3:AHR	chr7	17384880	17384880	A	G	UTR3	AHR	ENST000002	.	.	A	G	+	+	0.19047619	34,0,8,0	chr7,173848	Cytosol	A > G
chr7:17384882:A:G:UTR3:AHR	chr7	17384882	17384882	A	G	UTR3	AHR	ENST000002	.	.	A	G	+	+	0.17073171	34,0,7,0	chr7,173848	Cytosol	A > G
chr7:17384887:A:G:UTR3:AHR	chr7	17384887	17384887	A	G	UTR3	AHR	ENST000002	.	.	A	G	+	+	0.46341463	22,0,19,0	chr7,173848	Cytosol	A > G
chr7:23227425:A:G:intronic:AC005	chr7	23227425	23227425	A	G	intronic	AC005082.1,	.	.	.	A	G	+	+	0.33333333	4,0,2,0	chr7,232274	Cytosol	A > G
chr7:23227439:A:G:intronic:AC005	chr7	23227439	23227439	A	G	intronic	AC005082.1,	.	.	.	A	G	+	+	0.25	6,0,2,0	chr7,232274	Cytosol	A > G
chr7:23227477:A:G:intronic:AC005	chr7	23227477	23227477	A	G	intronic	AC005082.1,	.	.	.	A	G	+	+	0.4	6,0,4,0	chr7,232274	Cytosol	A > G
chr7:23229270:A:G:intronic:AC005	chr7	23229270	23229270	A	G	intronic	AC005082.1,	.	.	.	A	G	+	+	0.4	6,0,4,0	chr7,232292	Cytosol	A > G
chr7:25159181:T:C:downstream:C	chr7	25159181	25159181	T	C	downstream	CYCS	.	.	.	A	G	-	-	0.5	5,0,5,0	chr7,251591	Cytosol	A > G
chr7:25159781:T:C:UTR3:CYCS	chr7	25159781	25159781	T	C	UTR3	CYCS	ENST000003	.	.	A	G	-	-	0.35714286	9,0,5,0	chr7,251597	Cytosol	A > G
chr7:25159807:T:C:UTR3:CYCS	chr7	25159807	25159807	T	C	UTR3	CYCS	ENST000003	.	.	A	G	-	-	0.83333333	2,0,10,0	chr7,251598	Cytosol	A > G
chr7:25159810:T:C:UTR3:CYCS	chr7	25159810	25159810	T	C	UTR3	CYCS	ENST000003	.	.	A	G	-	-	0.41666667	7,0,5,0	chr7,251598	Cytosol	A > G
chr7:25159842:T:C:UTR3:CYCS	chr7	25159842	25159842	T	C	UTR3	CYCS	ENST000003	.	.	A	G	-	-	0.46153846	7,0,6,0	chr7,251598	Cytosol	A > G
chr7:25159843:T:C:UTR3:CYCS	chr7	25159843	25159843	T	C	UTR3	CYCS	ENST000003	.	.	A	G	-	-	0.28571429	10,0,4,0	chr7,251598	Cytosol	A > G
chr7:25159872:T:C:UTR3:CYCS	chr7	25159872	25159872	T	C	UTR3	CYCS	ENST000003	.	.	A	G	-	-	0.30769231	9,0,4,0	chr7,251598	Cytosol	A > G
chr7:25159873:T:C:UTR3:CYCS	chr7	25159873	25159873	T	C	UTR3	CYCS	ENST000003	.	.	A	G	-	-	0.14285714	12,0,2,0	chr7,251598	Cytosol	A > G
chr7:25159908:T:C:UTR3:CYCS	chr7	25159908	25159908	T	C	UTR3	CYCS	ENST000003	.	.	A	G	-	-	0.35	13,0,7,0	chr7,251599	Cytosol	A > G
chr7:25159932:T:C:UTR3:CYCS	chr7	25159932	25159932	T	C	UTR3	CYCS	ENST000003	.	.	A	G	-	-	0.6875	5,0,11,0	chr7,251599	Cytosol	A > G
chr7:25159947:T:C:UTR3:CYCS	chr7	25159947	25159947	T	C	UTR3	CYCS	ENST000003	.	.	A	G	-	-	0.81818182	2,0,9,0	chr7,251599	Cytosol	A > G
chr7:25159956:T:C:UTR3:CYCS	chr7	25159956	25159956	T	C	UTR3	CYCS	ENST000003	.	.	A	G	-	-	0.25	9,0,3,0	chr7,251599	Cytosol	A > G
chr7:25159977:T:C:UTR3:CYCS	chr7	25159977	25159977	T	C	UTR3	CYCS	ENST000003	.	.	A	G	-	-	0.77777778	2,0,7,0	chr7,251599	Cytosol	A > G
chr7:25159978:T:C:UTR3:CYCS	chr7	25159978	25159978	T	C	UTR3	CYCS	ENST000003	.	.	A	G	-	-	0.6	4,0,6,0	chr7,251599	Cytosol	A > G
chr7:25159986:T:C:UTR3:CYCS	chr7	25159986	25159986	T	C	UTR3	CYCS	ENST000003	.	.	A	G	-	-	0.18181818	9,0,2,0	chr7,251599	Cytosol	A > G
chr7:25160487:T:C:UTR3:CYCS	chr7	25160487	25160487	T	C	UTR3	CYCS	ENST000003	.	.	A	G	-	-	0.44444444	10,0,8,0	chr7,251604	Cytosol	A > G
chr7:25160499:T:C:UTR3:CYCS	chr7	25160499	25160499	T	C	UTR3	CYCS	ENST000003	.	.	A	G	-	-	0.25	12,0,4,0	chr7,251604	Cytosol	A > G
chr7:25160500:T:C:UTR3:CYCS	chr7	25160500	25160500	T	C	UTR3	CYCS	ENST000003	.	.	A	G	-	-	0.25	12,0,4,0	chr7,251604	Cytosol	A > G
chr7:25160518:T:C:UTR3:CYCS	chr7	25160518	25160518	T	C	UTR3	CYCS	ENST000003	.	.	A	G	-	-	0.8	3,0,12,0	chr7,251605	Cytosol	A > G
chr7:25160527:T:C:UTR3:CYCS	chr7	25160527	25160527	T	C	UTR3	CYCS	ENST000003	.	.	A	G	-	-	0.5	6,0,6,0	chr7,251605	Cytosol	A > G
chr7:25160581:T:C:UTR3:CYCS	chr7	25160581	25160581	T	C	UTR3	CYCS	ENST000003	.	.	A	G	-	-	0.22222222	14,0,4,0	chr7,251605	Cytosol	A > G
chr7:25160621:T:C:UTR3:CYCS	chr7	25160621	25160621	T	C	UTR3	CYCS	ENST000003	.	.	A	G	-	-	0.10526316	17,0,2,0	chr7,251606	Cytosol	A > G
chr7:25160631:T:C:UTR3:CYCS	chr7	25160631	25160631	T	C	UTR3	CYCS	ENST000003	.	.	A	G	-	-	0.16666667	15,0,3,0	chr7,251606	Cytosol	A > G
chr7:25160660:T:C:UTR3:CYCS	chr7	25160660	25160660	T	C	UTR3	CYCS	ENST000003	.	.	A	G	-	-	0.57142857	6,0,8,0	chr7,251606	Cytosol	A > G
chr7:25160686:T:C:UTR3:CYCS	chr7	25160686	25160686	T	C	UTR3	CYCS	ENST000003	.	.	A	G	-	-	0.22222222	7,0,2,0	chr7,251606	Cytosol	A > G
chr7:25160735:T:C:UTR3:CYCS	chr7	25160735	25160735	T	C	UTR3	CYCS	ENST000003	.	.	A	G	-	-	0.5	7,0,7,0	chr7,251607	Cytosol	A > G
chr7:25160907:T:C:UTR3:CYCS	chr7	25160907	25160907	T	C	UTR3	CYCS	ENST000003	.	.	A	G	-	-	0.31034483	20,0,9,0	chr7,251609	Cytosol	A > G
chr7:25160913:T:C:UTR3:CYCS	chr7	25160913	25160913	T	C	UTR3	CYCS	ENST000003	.	.	A	G	-	-	0.4	15,0,10,0	chr7,251609	Cytosol	A > G
chr7:25161040:T:C:UTR3:CYCS	chr7	25161040	25161040	T	C	UTR3	CYCS	ENST000003	.	.	A	G	-	-	0.38461538	8,0,5,0	chr7,251610	Cytosol	A > G
chr7:25161291:T:C:UTR3:CYCS	chr7	25161291	25161291	T	C	UTR3	CYCS	ENST000003	.	.	A	G	-	-	0.21052632	15,0,4,0	chr7,251612	Cytosol	A > G
chr7:25161424:T:C:UTR3:CYCS	chr7	25161424	25161424	T	C	UTR3	CYCS	ENST000003	.	.	A	G	-	-	0.47619048	11,0,10,0	chr7,251614	Cytosol	A > G
chr7:26708301:C:T:UTR3:SKAP2	chr7	26708301	26708301	C	T	UTR3	SKAP2	ENST000003	.	.	G	A	-	-	0.07407407	8,0,100,0	chr7,267083	Cytosol	G > A
chr7:32602270:A:G:intronic:AVL9	chr7	32602270	32602270	A	G	intronic	AVL9	.	.	.	A	G	+	+	0.6	2,0,3,0	chr7,326022	Cytosol	A > G
chr7:32953633:T:C:intronic:AVL9	chr7	32953633	32953633	T	C	intronic	AVL9	.	.	.	A	G	-	+	0.54545455	5,0,6,0	chr7,329536	Cytosol	A > G
chr7:38763066:T:C:UTR3:VPS41	chr7	38763066	38763066	T	C	UTR3	VPS41	ENST000003	.	.	A	G	-	-	0.46808511	25,0,22,0	chr7,387630	Cytosol	A > G
chr7:38763068:T:C:UTR3:VPS41	chr7	38763068	38763068	T	C	UTR3	VPS41	ENST000003	.	.	A	G	-	-	0.44680851	26,0,21,0	chr7,387630	Cytosol	A > G
chr7:38763074:T:C:UTR3:VPS41	chr7	38763074	38763074	T	C	UTR3	VPS41	ENST000003	.	.	A	G	-	-	0.96	2,0,48,0	chr7,387630	Cytosol	A > G
chr7:38763131:T:C:UTR3:VPS41	chr7	38763131	38763131	T	C	UTR3	VPS41	ENST000003	.	.	A	G	-	-	0.05263158	36,0,2,0	chr7,387631	Cytosol	A > G
chr7:38763141:T:C:UTR3:VPS41	chr7	38763141	38763141	T	C	UTR3	VPS41	ENST000003	.	.	A	G	-	-	0.06976744	40,0,3,0	chr7,387631	Cytosol	A > G
chr7:38763151:T:C:UTR3:VPS41	chr7	38763151	38763151	T	C	UTR3	VPS41	ENST000003	.	.	A	G	-	-	0.76315789	9,0,29,0	chr7,387631	Cytosol	A > G
chr7:38763200:T:C:UTR3:VPS41	chr7	38763200	38763200	T	C	UTR3	VPS41	ENST000003	.	.	A	G	-	-	0.26666667	33,0,12,0	chr7,387631	Cytosol	A > G
chr7:38763202:T:C:UTR3:VPS41	chr7	38763202	38763202	T	C	UTR3	VPS41	ENST000003	.	.	A	G	-	-	0.68888889	14,0,31,0	chr7,387632	Cytosol	A > G
chr7:38763212:T:C:UTR3:VPS41	chr7	38763212	38763212	T	C	UTR3	VPS41	ENST000003	.	.	A	G	-	-	0.61363636	17,0,27,0	chr7,387632	Cytosol	A > G
chr7:38763241:T:C:UTR3:VPS41	chr7	38763241	38763241	T	C	UTR3	VPS41	ENST000003	.	.	A	G	-	-	0.26666667	22,0,8,0	chr7,387632	Cytosol	A > G
chr7:38763248:T:C:UTR3:VPS41	chr7	38763248	38763248	T	C	UTR3	VPS41	ENST000003	.	.	A	G	-	-	0.07407407	25,0,2,0	chr7,387632	Cytosol	A > G
chr7:38764259:T:C:UTR3:VPS41	chr7	38764259	38764259	T	C	UTR3	VPS41	ENST000003	.	.	A	G	-	-	0.66666667	5,0,10,0	chr7,387642	Cytosol	A > G
chr7:38764286:T:C:UTR3:VPS41	chr7	38764286	38764286	T	C	UTR3	VPS41	ENST000003	.	.	A	G	-	-	0.22727273	17,0,5,0	chr7,387642	Cytosol	A > G
chr7:38764318:T:C:UTR3:VPS41	chr7	38764318	38764318	T	C	UTR3	VPS41	ENST000003	.	.	A	G	-	-	0.26923077	19,0,7,0	chr7,387643	Cytosol	A > G
chr7:38764322:T:C:UTR3:VPS41	chr7	38764322	38764322	T	C	UTR3	VPS41	ENST000003	.	.	A	G	-	-	0.37931034	18,0,11,0	chr7,387643	Cytosol	A > G
chr7:38764331:T:C:UTR3:VPS41	chr7	38764331	38764331	T	C	UTR3	VPS41	ENST000003	.	.	A	G	-	-	0.07142857	26,0,2,0	chr7,387643	Cytosol	A > G
chr7:38764342:T:C:UTR3:VPS41	chr7	38764342	38764342	T	C	UTR3	VPS41	ENST000003	.	.	A	G	-	-	0.44444444	15,0,12,0	chr7,387643	Cytosol	A > G
chr7:38764358:T:C:UTR3:VPS41	chr7	38764358	38764358	T	C	UTR3	VPS41	ENST000003	.	.	A	G	-	-	0.45454545	12,0,10,0	chr7,387643	Cytosol	A > G

chr7:38764393:T:C:UTR3:VPS41	chr7	38764393	38764393	T	C	UTR3	VPS41	ENST000003	.	.	A	G	-	-	0.10344828	26,0,3,0	chr7,387643	Cytosol	A > G
chr7:38764395:T:C:UTR3:VPS41	chr7	38764395	38764395	T	C	UTR3	VPS41	ENST000003	.	.	A	G	-	-	0.19354839	24,0,6,1	chr7,387643	Cytosol	A > G
chr7:38764459:T:C:UTR3:VPS41	chr7	38764459	38764459	T	C	UTR3	VPS41	ENST000003	.	.	A	G	-	-	0.17647059	28,0,6,0	chr7,387644	Cytosol	A > G
chr7:39828194:G:A:ncRNA_exonic	chr7	39828194	39828194	G	A	ncRNA_exon	LINC00265	.	.	.	G	A	+	+	0.6	3,0,2,0	chr7,398281	Cytosol	G > A
chr7:39828960:C:A:ncRNA_exonic	chr7	39828960	39828960	C	A	ncRNA_exon	LINC00265	.	.	.	C	A	+	+	0.22222222	2,7,0,0	chr7,398289	Cytosol	C > A
chr7:43656262:T:C:intronic:COA1,	chr7	43656262	43656262	T	C	intronic	COA1,STK17	.	.	.	A	G	-	+-	0.8	1,0,4,0	chr7,436562	Cytosol	A > G
chr7:44024305:T:C:ncRNA_introni	chr7	44024305	44024305	T	C	ncRNA_intro	POLR2J4,RP5	.	.	.	A	G	-	-	0.71428571	2,0,5,0	chr7,440243	Cytosol	A > G
chr7:44053660:T:C:ncRNA_exonic	chr7	44053660	44053660	T	C	ncRNA_exon	POLR2J4	.	.	.	A	G	-	-	0.125	14,0,2,0	chr7,440536	Cytosol	A > G
chr7:44614322:T:C:UTR5:DDX56	chr7	44614322	44614322	T	C	UTR5	DDX56	ENST000004	.	.	A	G	-	-	0.33333333	6,0,3,0	chr7,446143	Cytosol	A > G
chr7:44616535:T:C:downstream:T	chr7	44616535	44616535	T	C	downstream	TMED4	.	.	.	A	G	-		0.4	3,0,2,0	chr7,446165	Cytosol	A > G
chr7:44616605:T:C:downstream:T	chr7	44616605	44616605	T	C	downstream	TMED4	.	.	.	A	G	-		0.5	4,0,4,0	chr7,446166	Cytosol	A > G
chr7:44616657:T:C:downstream:T	chr7	44616657	44616657	T	C	downstream	TMED4	.	.	.	A	G	-		0.5	3,0,3,0	chr7,446166	Cytosol	A > G
chr7:44616658:T:C:downstream:T	chr7	44616658	44616658	T	C	downstream	TMED4	.	.	.	A	G	-		0.33333333	4,0,2,0	chr7,446166	Cytosol	A > G
chr7:44616883:T:C:downstream:T	chr7	44616883	44616883	T	C	downstream	TMED4	.	.	.	A	G	-		0.28571429	5,0,2,0	chr7,446168	Cytosol	A > G
chr7:44618317:T:C:UTR3:TMED4	chr7	44618317	44618317	T	C	UTR3	TMED4	ENST000004	.	.	A	G	-	-	0.14545455	47,0,8,0	chr7,446183	Cytosol	A > G
chr7:44618343:T:C:UTR3:TMED4	chr7	44618343	44618343	T	C	UTR3	TMED4	ENST000004	.	.	A	G	-	-	0.20253165	63,0,16,0	chr7,446183	Cytosol	A > G
chr7:44618427:T:C:UTR3:TMED4	chr7	44618427	44618427	T	C	UTR3	TMED4	ENST000004	.	.	A	G	-	-	0.0480226	336,0,17,1	chr7,446184	Cytosol	A > G
chr7:44619821:T:C:UTR3:TMED4	chr7	44619821	44619821	T	C	UTR3	TMED4	ENST000004	.	.	A	G	-	-	0.07368421	88,0,7,0	chr7,446198	Cytosol	A > G
chr7:44619833:T:C:UTR3:TMED4	chr7	44619833	44619833	T	C	UTR3	TMED4	ENST000004	.	.	A	G	-	-	0.05319149	89,0,5,0	chr7,446198	Cytosol	A > G
chr7:44619843:T:C:UTR3:TMED4	chr7	44619843	44619843	T	C	UTR3	TMED4	ENST000004	.	.	A	G	-	-	0.03191489	91,0,3,0	chr7,446198	Cytosol	A > G
chr7:44619897:T:C:UTR3:TMED4	chr7	44619897	44619897	T	C	UTR3	TMED4	ENST000004	.	.	A	G	-	-	0.04166667	46,0,2,0	chr7,446198	Cytosol	A > G
chr7:44620001:T:C:UTR3:TMED4	chr7	44620001	44620001	T	C	UTR3	TMED4	ENST000004	.	.	A	G	-	-	0.11111111	32,0,4,0	chr7,446200	Cytosol	A > G
chr7:44620289:T:C:UTR3:TMED4	chr7	44620289	44620289	T	C	UTR3	TMED4	ENST000004	.	.	A	G	-	-	0.12244898	43,0,6,0	chr7,446202	Cytosol	A > G
chr7:44620302:T:C:UTR3:TMED4	chr7	44620302	44620302	T	C	UTR3	TMED4	ENST000004	.	.	A	G	-	-	0.06818182	41,0,3,0	chr7,446203	Cytosol	A > G
chr7:44620311:T:C:UTR3:TMED4	chr7	44620311	44620311	T	C	UTR3	TMED4	ENST000004	.	.	A	G	-	-	0.19047619	34,0,8,0	chr7,446203	Cytosol	A > G
chr7:44620424:T:C:UTR3:TMED4	chr7	44620424	44620424	T	C	UTR3	TMED4	ENST000004	.	.	A	G	-	-	0.20588235	27,0,7,0	chr7,446204	Cytosol	A > G
chr7:44620470:T:C:UTR3:TMED4	chr7	44620470	44620470	T	C	UTR3	TMED4	ENST000004	.	.	A	G	-	-	0.19354839	50,0,12,0	chr7,446204	Cytosol	A > G
chr7:44620500:T:C:UTR3:TMED4	chr7	44620500	44620500	T	C	UTR3	TMED4	ENST000004	.	.	A	G	-	-	0.05194805	73,0,4,0	chr7,446204	Cytosol	A > G
chr7:44620508:T:C:UTR3:TMED4	chr7	44620508	44620508	T	C	UTR3	TMED4	ENST000004	.	.	A	G	-	-	0.11956522	81,0,11,0	chr7,446205	Cytosol	A > G
chr7:44620546:T:C:UTR3:TMED4	chr7	44620546	44620546	T	C	UTR3	TMED4	ENST000004	.	.	A	G	-	-	0.04	96,0,4,0	chr7,446205	Cytosol	A > G
chr7:44665511:A:G:intronic:OGDH	chr7	44665511	44665511	A	G	intronic	OGDH	.	.	.	A	G	+	+	0.5	3,0,3,0	chr7,446655	Cytosol	A > G
chr7:44841489:A:G:UTR3:PPIA	chr7	44841489	44841489	A	G	UTR3	PPIA	ENST000004	.	.	A	G	+	+	0.82461538	57,0,268,0	chr7,448414	Cytosol	A > G
chr7:44841519:A:G:UTR3:PPIA	chr7	44841519	44841519	A	G	UTR3	PPIA	ENST000004	.	.	A	G	+	+	0.20812183	312,0,82,0	chr7,448415	Cytosol	A > G
chr7:44841589:A:G:UTR3:PPIA	chr7	44841589	44841589	A	G	UTR3	PPIA	ENST000004	.	.	A	G	+	+	0.05846154	306,0,19,0	chr7,448415	Cytosol	A > G
chr7:44841928:A:G:UTR3:PPIA	chr7	44841928	44841928	A	G	UTR3	PPIA	ENST000004	.	.	A	G	+	+	0.34666667	147,0,78,0	chr7,448419	Cytosol	A > G
chr7:44841931:A:G:UTR3:PPIA	chr7	44841931	44841931	A	G	UTR3	PPIA	ENST000004	.	.	A	G	+	+	0.05084746	224,0,12,0	chr7,448419	Cytosol	A > G
chr7:44841933:A:G:UTR3:PPIA	chr7	44841933	44841933	A	G	UTR3	PPIA	ENST000004	.	.	A	G	+	+	0.05555556	221,0,13,0	chr7,448419	Cytosol	A > G
chr7:44842022:A:G:UTR3:PPIA	chr7	44842022	44842022	A	G	UTR3	PPIA	ENST000004	.	.	A	G	+	+	0.01790281	384,0,7,0	chr7,448420	Cytosol	A > G
chr7:44842099:A:G:UTR3:PPIA	chr7	44842099	44842099	A	G	UTR3	PPIA	ENST000004	.	.	A	G	+	+	0.13670886	341,0,54,0	chr7,448420	Cytosol	A > G
chr7:44870379:T:C:intronic:H2AFV	chr7	44870379	44870379	T	C	intronic	H2AFV	.	.	.	A	G	-	-	0.11320755	47,0,6,0	chr7,448703	Cytosol	A > G
chr7:44870498:T:C:intronic:H2AFV	chr7	44870498	44870498	T	C	intronic	H2AFV	.	.	.	A	G	-	-	0.02083333	141,0,3,0	chr7,448704	Cytosol	A > G
chr7:44870547:T:C:intronic:H2AFV	chr7	44870547	44870547	T	C	intronic	H2AFV	.	.	.	A	G	-	-	0.07476636	99,0,8,0	chr7,448705	Cytosol	A > G
chr7:44872282:T:C:intronic:H2AFV	chr7	44872282	44872282	T	C	intronic	H2AFV	.	.	.	A	G	-	-	0.01229508	241,0,3,0	chr7,448722	Cytosol	A > G
chr7:44872283:T:C:intronic:H2AFV	chr7	44872283	44872283	T	C	intronic	H2AFV	.	.	.	A	G	-	-	0.03585657	242,0,9,0	chr7,448722	Cytosol	A > G
chr7:44872285:T:C:intronic:H2AFV	chr7	44872285	44872285	T	C	intronic	H2AFV	.	.	.	A	G	-	-	0.05555556	238,0,14,0	chr7,448722	Cytosol	A > G
chr7:44872291:T:C:intronic:H2AFV	chr7	44872291	44872291	T	C	intronic	H2AFV	.	.	.	A	G	-	-	0.03543307	245,0,9,0	chr7,448722	Cytosol	A > G
chr7:44872300:T:C:intronic:H2AFV	chr7	44872300	44872300	T	C	intronic	H2AFV	.	.	.	A	G	-	-	0.46202532	85,0,73,0	chr7,448722	Cytosol	A > G
chr7:44872303:T:C:intronic:H2AFV	chr7	44872303	44872303	T	C	intronic	H2AFV	.	.	.	A	G	-	-	0.34545455	108,0,57,0	chr7,448723	Cytosol	A > G
chr7:44872304:T:C:intronic:H2AFV	chr7	44872304	44872304	T	C	intronic	H2AFV	.	.	.	A	G	-	-	0.08	161,0,14,0	chr7,448723	Cytosol	A > G
chr7:44872409:T:C:intronic:H2AFV	chr7	44872409	44872409	T	C	intronic	H2AFV	.	.	.	A	G	-	-	0.02941176	132,0,4,0	chr7,448724	Cytosol	A > G
chr7:44872419:T:C:intronic:H2AFV	chr7	44872419	44872419	T	C	intronic	H2AFV	.	.	.	A	G	-	-	0.72727273	45,0,120,0	chr7,448724	Cytosol	A > G
chr7:44872421:T:C:intronic:H2AFV	chr7	44872421	44872421	T	C	intronic	H2AFV	.	.	.	A	G	-	-	0.26797386	112,0,41,0	chr7,448724	Cytosol	A > G
chr7:44872425:T:C:intronic:H2AFV	chr7	44872425	44872425	T	C	intronic	H2AFV	.	.	.	A	G	-	-	0.1	135,0,15,0	chr7,448724	Cytosol	A > G
chr7:44872427:T:C:intronic:H2AFV	chr7	44872427	44872427	T	C	intronic	H2AFV	.	.	.	A	G	-	-	0.68125	51,0,109,0	chr7,448724	Cytosol	A > G
chr7:44872437:T:C:intronic:H2AFV	chr7	44872437	44872437	T	C	intronic	H2AFV	.	.	.	A	G	-	-	0.28921569	145,0,59,0	chr7,448724	Cytosol	A > G
chr7:44872466:T:C:intronic:H2AFV	chr7	44872466	44872466	T	C	intronic	H2AFV	.	.	.	A	G	-	-	0.22222222	196,0,56,0	chr7,448724	Cytosol	A > G
chr7:44872473:T:C:intronic:H2AFV	chr7	44872473	44872473	T	C	intronic	H2AFV	.	.	.	A	G	-	-	0.5308642	114,0,129,0	chr7,448724	Cytosol	A > G
chr7:44872480:T:C:intronic:H2AFV	chr7	44872480	44872480	T	C	intronic	H2AFV	.	.	.	A	G	-	-	0.21235521	204,0,55,0	chr7,448724	Cytosol	A > G
chr7:44872487:T:C:intronic:H2AFV	chr7	44872487	44872487	T	C	intronic	H2AFV	.	.	.	A	G	-	-	0.12109375	225,0,31,0	chr7,448724	Cytosol	A > G
chr7:44872492:T:C:intronic:H2AFV	chr7	44872492	44872492	T	C	intronic	H2AFV	.	.	.	A	G	-	-	0.05855856	209,0,13,0	chr7,448724	Cytosol	A > G
chr7:44872502:T:C:intronic:H2AFV	chr7	44872502	44872502	T	C	intronic	H2AFV	.	.	.	A	G	-	-	0.05479452	207,0,12,0	chr7,448725	Cytosol	A > G
chr7:44872505:T:C:intronic:H2AFV	chr7	44872505	44872505	T	C	intronic	H2AFV	.	.	.	A	G	-	-	0.35483871	140,0,77,0	chr7,448725	Cytosol	A > G
chr7:44872511:T:C:intronic:H2AFV	chr7	44872511	44872511	T	C	intronic	H2AFV	.	.	.	A	G	-	-	0.12785388	191,0,28,0	chr7,448725	Cytosol	A > G
chr7:44872523:T:C:intronic:H2AFV	chr7	44872523	44872523	T	C	intronic	H2AFV	.	.	.	A	G	-	-	0.01204819	164,0,2,0	chr7,448725	Cytosol	A > G

chr7:44872689:T:C:intronic:H2AFV	chr7	44872689	44872689	T	C	intronic	H2AFV	.	.	.	A	G	-	-	0.10714286	225,0,27,0	chr7,448726	Cytosol	A > G
chr7:44872701:T:C:intronic:H2AFV	chr7	44872701	44872701	T	C	intronic	H2AFV	.	.	.	A	G	-	-	0.13157895	132,0,20,0	chr7,448727	Cytosol	A > G
chr7:44872714:T:C:intronic:H2AFV	chr7	44872714	44872714	T	C	intronic	H2AFV	.	.	.	A	G	-	-	0.20261438	122,0,31,0	chr7,448727	Cytosol	A > G
chr7:44872732:T:C:intronic:H2AFV	chr7	44872732	44872732	T	C	intronic	H2AFV	.	.	.	A	G	-	-	0.23756906	138,0,43,0	chr7,448727	Cytosol	A > G
chr7:44872739:T:C:intronic:H2AFV	chr7	44872739	44872739	T	C	intronic	H2AFV	.	.	.	A	G	-	-	0.08762887	177,0,17,0	chr7,448727	Cytosol	A > G
chr7:44872827:T:C:intronic:H2AFV	chr7	44872827	44872827	T	C	intronic	H2AFV	.	.	.	A	G	-	-	0.0877193	312,0,30,0	chr7,448728	Cytosol	A > G
chr7:44872862:T:C:intronic:H2AFV	chr7	44872862	44872862	T	C	intronic	H2AFV	.	.	.	A	G	-	-	0.10877193	254,0,31,0	chr7,448728	Cytosol	A > G
chr7:44872863:T:C:intronic:H2AFV	chr7	44872863	44872863	T	C	intronic	H2AFV	.	.	.	A	G	-	-	0.02787456	279,0,8,0	chr7,448728	Cytosol	A > G
chr7:44872899:T:C:intronic:H2AFV	chr7	44872899	44872899	T	C	intronic	H2AFV	.	.	.	A	G	-	-	0.31313131	136,0,62,0	chr7,448728	Cytosol	A > G
chr7:44872904:T:C:intronic:H2AFV	chr7	44872904	44872904	T	C	intronic	H2AFV	.	.	.	A	G	-	-	0.20879121	144,0,38,0	chr7,448729	Cytosol	A > G
chr7:44872908:T:C:intronic:H2AFV	chr7	44872908	44872908	T	C	intronic	H2AFV	.	.	.	A	G	-	-	0.02030457	193,0,4,0	chr7,448729	Cytosol	A > G
chr7:44872928:T:C:intronic:H2AFV	chr7	44872928	44872928	T	C	intronic	H2AFV	.	.	.	A	G	-	-	0.1192053	133,0,18,0	chr7,448729	Cytosol	A > G
chr7:44872929:T:C:intronic:H2AFV	chr7	44872929	44872929	T	C	intronic	H2AFV	.	.	.	A	G	-	-	0.20606061	131,0,34,0	chr7,448729	Cytosol	A > G
chr7:44872970:T:C:intronic:H2AFV	chr7	44872970	44872970	T	C	intronic	H2AFV	.	.	.	A	G	-	-	0.90839695	12,0,119,0	chr7,448729	Cytosol	A > G
chr7:44872996:T:C:intronic:H2AFV	chr7	44872996	44872996	T	C	intronic	H2AFV	.	.	.	A	G	-	-	0.85333333	11,0,64,0	chr7,448729	Cytosol	A > G
chr7:44873000:T:C:intronic:H2AFV	chr7	44873000	44873000	T	C	intronic	H2AFV	.	.	.	A	G	-	-	0.16666667	55,0,11,0	chr7,448729	Cytosol	A > G
chr7:44873001:T:C:intronic:H2AFV	chr7	44873001	44873001	T	C	intronic	H2AFV	.	.	.	A	G	-	-	0.28813559	42,0,17,0	chr7,448730	Cytosol	A > G
chr7:44873003:T:C:intronic:H2AFV	chr7	44873003	44873003	T	C	intronic	H2AFV	.	.	.	A	G	-	-	0.31578947	39,0,18,0	chr7,448730	Cytosol	A > G
chr7:44873052:T:C:intronic:H2AFV	chr7	44873052	44873052	T	C	intronic	H2AFV	.	.	.	A	G	-	-	0.25	60,0,20,0	chr7,448730	Cytosol	A > G
chr7:44873059:T:C:intronic:H2AFV	chr7	44873059	44873059	T	C	intronic	H2AFV	.	.	.	A	G	-	-	0.34285714	69,0,36,0	chr7,448730	Cytosol	A > G
chr7:44873098:T:C:intronic:H2AFV	chr7	44873098	44873098	T	C	intronic	H2AFV	.	.	.	A	G	-	-	0.15976331	142,0,27,0	chr7,448730	Cytosol	A > G
chr7:44873106:T:C:intronic:H2AFV	chr7	44873106	44873106	T	C	intronic	H2AFV	.	.	.	A	G	-	-	0.75806452	45,0,141,0	chr7,448731	Cytosol	A > G
chr7:44873177:T:C:intronic:H2AFV	chr7	44873177	44873177	T	C	intronic	H2AFV	.	.	.	A	G	-	-	0.22222222	126,0,36,0	chr7,448731	Cytosol	A > G
chr7:44873216:T:C:intronic:H2AFV	chr7	44873216	44873216	T	C	intronic	H2AFV	.	.	.	A	G	-	-	0.02054795	143,0,3,0	chr7,448732	Cytosol	A > G
chr7:44873222:T:C:intronic:H2AFV	chr7	44873222	44873222	T	C	intronic	H2AFV	.	.	.	A	G	-	-	0.0505618	169,0,9,0	chr7,448732	Cytosol	A > G
chr7:44917236:T:C:UTR3:PURB	chr7	44917236	44917236	T	C	UTR3	PURB	ENST000003	.	.	A	G	-	-	0.10526316	17,0,2,0	chr7,449172	Cytosol	A > G
chr7:55521986:T:C:intergenic:LAN	chr7	55521986	55521986	T	C	intergenic	LANCL2,VOP	dist=20551;d	.	.	A	G	-	-	0.76923077	3,0,10,0	chr7,555219	Cytosol	A > G
chr7:55522162:T:C:intergenic:LAN	chr7	55522162	55522162	T	C	intergenic	LANCL2,VOP	dist=20727;d	.	.	A	G	-	-	0.55555556	4,0,5,0	chr7,555221	Cytosol	A > G
chr7:55522508:T:C:intergenic:LAN	chr7	55522508	55522508	T	C	intergenic	LANCL2,VOP	dist=21073;d	.	.	A	G	-	-	0.33333333	6,0,3,0	chr7,555225	Cytosol	A > G
chr7:55522511:T:C:intergenic:LAN	chr7	55522511	55522511	T	C	intergenic	LANCL2,VOP	dist=21076;d	.	.	A	G	-	-	0.33333333	6,0,3,0	chr7,555225	Cytosol	A > G
chr7:55522532:T:C:intergenic:LAN	chr7	55522532	55522532	T	C	intergenic	LANCL2,VOP	dist=21097;d	.	.	A	G	-	-	0.41666667	7,0,5,0	chr7,555225	Cytosol	A > G
chr7:55522540:T:C:intergenic:LAN	chr7	55522540	55522540	T	C	intergenic	LANCL2,VOP	dist=21105;d	.	.	A	G	-	-	0.3	7,0,3,0	chr7,555225	Cytosol	A > G
chr7:55522666:T:C:intergenic:LAN	chr7	55522666	55522666	T	C	intergenic	LANCL2,VOP	dist=21231;d	.	.	A	G	-	-	0.28571429	10,0,4,0	chr7,555226	Cytosol	A > G
chr7:55522719:T:C:intergenic:LAN	chr7	55522719	55522719	T	C	intergenic	LANCL2,VOP	dist=21284;d	.	.	A	G	-	-	0.44444444	5,0,4,0	chr7,555227	Cytosol	A > G
chr7:55522742:T:C:intergenic:LAN	chr7	55522742	55522742	T	C	intergenic	LANCL2,VOP	dist=21307;d	.	.	A	G	-	-	0.5	4,0,4,0	chr7,555227	Cytosol	A > G
chr7:55522972:T:C:intergenic:LAN	chr7	55522972	55522972	T	C	intergenic	LANCL2,VOP	dist=21537;d	.	.	A	G	-	-	0.76923077	3,0,10,0	chr7,555229	Cytosol	A > G
chr7:55523108:T:C:intergenic:LAN	chr7	55523108	55523108	T	C	intergenic	LANCL2,VOP	dist=21673;d	.	.	A	G	-	-	0.66666667	2,0,4,0	chr7,555231	Cytosol	A > G
chr7:55523237:T:C:intergenic:LAN	chr7	55523237	55523237	T	C	intergenic	LANCL2,VOP	dist=21802;d	.	.	A	G	-	-	0.6	4,0,6,0	chr7,555232	Cytosol	A > G
chr7:55523276:T:C:intergenic:LAN	chr7	55523276	55523276	T	C	intergenic	LANCL2,VOP	dist=21841;d	.	.	A	G	-	-	0.54545455	5,0,6,0	chr7,555232	Cytosol	A > G
chr7:55523334:T:C:intergenic:LAN	chr7	55523334	55523334	T	C	intergenic	LANCL2,VOP	dist=21899;d	.	.	A	G	-	-	0.66666667	2,0,4,0	chr7,555233	Cytosol	A > G
chr7:55523661:T:C:intergenic:LAN	chr7	55523661	55523661	T	C	intergenic	LANCL2,VOP	dist=22226;d	.	.	A	G	-	-	0.375	10,0,6,0	chr7,555236	Cytosol	A > G
chr7:55525040:T:C:intergenic:LAN	chr7	55525040	55525040	T	C	intergenic	LANCL2,VOP	dist=23605;d	.	.	A	G	-	-	0.41666667	7,0,5,0	chr7,555250	Cytosol	A > G
chr7:55525241:T:C:intergenic:LAN	chr7	55525241	55525241	T	C	intergenic	LANCL2,VOP	dist=23806;d	.	.	A	G	-	-	0.35714286	9,0,5,0	chr7,555252	Cytosol	A > G
chr7:55525394:T:C:intergenic:LAN	chr7	55525394	55525394	T	C	intergenic	LANCL2,VOP	dist=23959;d	.	.	A	G	-	-	0.83333333	1,0,5,0	chr7,555253	Cytosol	A > G
chr7:55525401:T:C:intergenic:LAN	chr7	55525401	55525401	T	C	intergenic	LANCL2,VOP	dist=23966;d	.	.	A	G	-	-	0.55555556	4,0,5,0	chr7,555254	Cytosol	A > G
chr7:55525474:T:C:intergenic:LAN	chr7	55525474	55525474	T	C	intergenic	LANCL2,VOP	dist=24039;d	.	.	A	G	-	-	0.8	1,0,4,0	chr7,555254	Cytosol	A > G
chr7:55525480:T:C:intergenic:LAN	chr7	55525480	55525480	T	C	intergenic	LANCL2,VOP	dist=24045;d	.	.	A	G	-	-	0.83333333	1,0,5,0	chr7,555254	Cytosol	A > G
chr7:55525535:T:C:intergenic:LAN	chr7	55525535	55525535	T	C	intergenic	LANCL2,VOP	dist=24100;d	.	.	A	G	-	-	0.57142857	3,0,4,0	chr7,555255	Cytosol	A > G
chr7:55525536:T:C:intergenic:LAN	chr7	55525536	55525536	T	C	intergenic	LANCL2,VOP	dist=24101;d	.	.	A	G	-	-	0.5	3,0,3,0	chr7,555255	Cytosol	A > G
chr7:55525574:T:C:intergenic:LAN	chr7	55525574	55525574	T	C	intergenic	LANCL2,VOP	dist=24139;d	.	.	A	G	-	-	0.57142857	3,0,4,0	chr7,555255	Cytosol	A > G
chr7:55525633:T:C:intergenic:LAN	chr7	55525633	55525633	T	C	intergenic	LANCL2,VOP	dist=24198;d	.	.	A	G	-	-	0.33333333	6,0,3,0	chr7,555256	Cytosol	A > G
chr7:55525637:T:C:intergenic:LAN	chr7	55525637	55525637	T	C	intergenic	LANCL2,VOP	dist=24202;d	.	.	A	G	-	-	0.33333333	6,0,3,0	chr7,555256	Cytosol	A > G
chr7:55525638:T:C:intergenic:LAN	chr7	55525638	55525638	T	C	intergenic	LANCL2,VOP	dist=24203;d	.	.	A	G	-	-	0.5	5,0,5,0	chr7,555256	Cytosol	A > G
chr7:55525732:T:C:intergenic:LAN	chr7	55525732	55525732	T	C	intergenic	LANCL2,VOP	dist=24297;d	.	.	A	G	-	-	0.23076923	10,0,3,0	chr7,555257	Cytosol	A > G
chr7:55525773:T:C:intergenic:LAN	chr7	55525773	55525773	T	C	intergenic	LANCL2,VOP	dist=24338;d	.	.	A	G	-	-	0.38461538	8,0,5,0	chr7,555257	Cytosol	A > G
chr7:55525814:T:C:intergenic:LAN	chr7	55525814	55525814	T	C	intergenic	LANCL2,VOP	dist=24379;d	.	.	A	G	-	-	0.69230769	4,0,9,0	chr7,555258	Cytosol	A > G
chr7:55525817:T:C:intergenic:LAN	chr7	55525817	55525817	T	C	intergenic	LANCL2,VOP	dist=24382;d	.	.	A	G	-	-	0.35714286	9,0,5,0	chr7,555258	Cytosol	A > G
chr7:55525861:T:C:intergenic:LAN	chr7	55525861	55525861	T	C	intergenic	LANCL2,VOP	dist=24426;d	.	.	A	G	-	-	0.55555556	4,0,5,0	chr7,555258	Cytosol	A > G
chr7:55525996:T:C:intergenic:LAN	chr7	55525996	55525996	T	C	intergenic	LANCL2,VOP	dist=24561;d	.	.	A	G	-	-	0.35714286	9,0,5,0	chr7,555259	Cytosol	A > G
chr7:55526083:T:C:intergenic:LAN	chr7	55526083	55526083	T	C	intergenic	LANCL2,VOP	dist=24648;d	.	.	A	G	-	-	0.30769231	9,0,4,0	chr7,555260	Cytosol	A > G
chr7:55526128:T:C:intergenic:LAN	chr7	55526128	55526128	T	C	intergenic	LANCL2,VOP	dist=24693;d	.	.	A	G	-	-	0.3	7,0,3,0	chr7,555261	Cytosol	A > G
chr7:55526180:T:C:intergenic:LAN	chr7	55526180	55526180	T	C	intergenic	LANCL2,VOP	dist=24745;d	.	.	A	G	-	-	0.66666667	3,0,6,0	chr7,555261	Cytosol	A > G
chr7:55526305:T:C:intergenic:LAN	chr7	55526305	55526305	T	C	intergenic	LANCL2,VOP	dist=24870;d	.	.	A	G	-	-	0.7	3,0,7,0	chr7,555263	Cytosol	A > G
chr7:55526716:T:C:intergenic:LAN	chr7	55526716	55526716	T	C	intergenic	LANCL2,VOP	dist=25281;d	.	.	A	G	-	-	0.3	7,0,3,0	chr7,555267	Cytosol	A > G

chr7:55526758:T:C:intergenic:LAN	chr7	55526758	55526758	T	C	intergenic	LANCL2,VOP	dist=25323;d	.	.	A	G	-	-	0.57142857	3,0,4,0	chr7,555267	Cytosol	A > G
chr7:55526941:T:C:intergenic:LAN	chr7	55526941	55526941	T	C	intergenic	LANCL2,VOP	dist=25506;d	.	.	A	G	-	-	0.21428571	11,0,3,0	chr7,555269	Cytosol	A > G
chr7:55526968:T:C:intergenic:LAN	chr7	55526968	55526968	T	C	intergenic	LANCL2,VOP	dist=25533;d	.	.	A	G	-	-	0.4375	9,0,7,0	chr7,555269	Cytosol	A > G
chr7:56009028:A:G:UTR3:ZNF713	chr7	56009028	56009028	A	G	UTR3	ZNF713	ENST000004	.	.	A	G	+	+	0.83333333	1,0,5,0	chr7,560090	Cytosol	A > G
chr7:56023946:A:G:UTR3:MRPS17	chr7	56023946	56023946	A	G	UTR3	MRPS17	ENST000002	.	.	A	G	+	+	0.04545455	63,0,3,0	chr7,560239	Cytosol	A > G
chr7:56024026:A:G:UTR3:MRPS17	chr7	56024026	56024026	A	G	UTR3	MRPS17	ENST000002	.	.	A	G	+	+	0.02803738	104,0,3,0	chr7,560240	Cytosol	A > G
chr7:56078288:T:C:downstream:P	chr7	56078288	56078288	T	C	downstream	PSPH	.	.	.	A	G	-		0.27272727	8,0,3,0	chr7,560782	Cytosol	A > G
chr7:56078298:T:C:downstream:P	chr7	56078298	56078298	T	C	downstream	PSPH	.	.	.	A	G	-		1	0,0,14,0	chr7,560782	Cytosol	A > G
chr7:56078304:T:C:downstream:P	chr7	56078304	56078304	T	C	downstream	PSPH	.	.	.	A	G	-		0.27777778	13,0,5,0	chr7,560783	Cytosol	A > G
chr7:56078324:T:C:downstream:P	chr7	56078324	56078324	T	C	downstream	PSPH	.	.	.	A	G	-		0.32692308	35,0,17,0	chr7,560783	Cytosol	A > G
chr7:56078331:T:C:downstream:P	chr7	56078331	56078331	T	C	downstream	PSPH	.	.	.	A	G	-		0.05084746	56,0,3,0	chr7,560783	Cytosol	A > G
chr7:56078332:T:C:downstream:P	chr7	56078332	56078332	T	C	downstream	PSPH	.	.	.	A	G	-		0.29508197	43,0,18,0	chr7,560783	Cytosol	A > G
chr7:56078337:T:C:downstream:P	chr7	56078337	56078337	T	C	downstream	PSPH	.	.	.	A	G	-		0.17741935	51,0,11,0	chr7,560783	Cytosol	A > G
chr7:56078338:T:C:downstream:P	chr7	56078338	56078338	T	C	downstream	PSPH	.	.	.	A	G	-		0.08064516	57,0,5,0	chr7,560783	Cytosol	A > G
chr7:56078342:T:C:downstream:P	chr7	56078342	56078342	T	C	downstream	PSPH	.	.	.	A	G	-		0.49230769	33,0,32,0	chr7,560783	Cytosol	A > G
chr7:56078359:T:C:downstream:P	chr7	56078359	56078359	T	C	downstream	PSPH	.	.	.	A	G	-		0.0625	75,0,5,0	chr7,560783	Cytosol	A > G
chr7:56078361:T:C:downstream:P	chr7	56078361	56078361	T	C	downstream	PSPH	.	.	.	A	G	-		0.05617978	84,0,5,0	chr7,560783	Cytosol	A > G
chr7:56078433:T:C:downstream:P	chr7	56078433	56078433	T	C	downstream	PSPH	.	.	.	A	G	-		0.03571429	81,0,3,0	chr7,560784	Cytosol	A > G
chr7:56078441:T:C:downstream:P	chr7	56078441	56078441	T	C	downstream	PSPH	.	.	.	A	G	-		0.06849315	68,0,5,0	chr7,560784	Cytosol	A > G
chr7:56079058:T:C:UTR3:PSPH	chr7	56079058	56079058	T	C	UTR3	PSPH	ENST000002	.	.	A	G	-	-	0.04026846	143,0,6,0	chr7,560790	Cytosol	A > G
chr7:56079074:T:C:UTR3:PSPH	chr7	56079074	56079074	T	C	UTR3	PSPH	ENST000002	.	.	A	G	-	-	0.15714286	118,0,22,0	chr7,560790	Cytosol	A > G
chr7:56079213:T:C:UTR3:PSPH	chr7	56079213	56079213	T	C	UTR3	PSPH	ENST000002	.	.	A	G	-	-	0.23188406	53,0,16,0	chr7,560792	Cytosol	A > G
chr7:56079216:T:C:UTR3:PSPH	chr7	56079216	56079216	T	C	UTR3	PSPH	ENST000002	.	.	A	G	-	-	0.27536232	50,0,19,0	chr7,560792	Cytosol	A > G
chr7:56108948:T:C:intronic:PSPH	chr7	56108948	56108948	T	C	intronic	PSPH	.	.	.	A	G	-	-	1	0,0,5,0	chr7,561089	Cytosol	A > G
chr7:64259674:A:G:intronic:ZNF13	chr7	64259674	64259674	A	G	intronic	ZNF138	.	.	.	A	G	+	+	0.22222222	7,0,2,0	chr7,642596	Cytosol	A > G
chr7:64259684:A:G:intronic:ZNF13	chr7	64259684	64259684	A	G	intronic	ZNF138	.	.	.	A	G	+	+	0.28571429	5,0,2,0	chr7,642596	Cytosol	A > G
chr7:65205946:T:C:intergenic:INTS	chr7	65205946	65205946	T	C	intergenic	INTS4L2,CCT	dist=25310;d	.	.	A	G	-	-	0.09090909	20,0,2,0	chr7,652059	Cytosol	A > G
chr7:65205990:T:C:intergenic:INTS	chr7	65205990	65205990	T	C	intergenic	INTS4L2,CCT	dist=25354;d	.	.	A	G	-	-	0.07692308	24,0,2,0	chr7,652059	Cytosol	A > G
chr7:65205993:T:C:intergenic:INTS	chr7	65205993	65205993	T	C	intergenic	INTS4L2,CCT	dist=25357;d	.	.	A	G	-	-	0.16	21,0,4,0	chr7,652059	Cytosol	A > G
chr7:65205996:T:C:intergenic:INTS	chr7	65205996	65205996	T	C	intergenic	INTS4L2,CCT	dist=25360;d	.	.	A	G	-	-	0.07692308	24,0,2,0	chr7,652059	Cytosol	A > G
chr7:65422286:A:G:UTR3:VKORC1	chr7	65422286	65422286	A	G	UTR3	VKORC1L1	ENST000003	.	.	A	G	+	+	0.01626016	121,0,2,0	chr7,654222	Cytosol	A > G
chr7:65618205:A:G:ncRNA_introni	chr7	65618205	65618205	A	G	ncRNA_intro	RP5-1132H1	.	.	.	A	G	+	-+	0.06060606	93,0,6,0	chr7,656182	Cytosol	A > G
chr7:65618210:A:G:ncRNA_introni	chr7	65618210	65618210	A	G	ncRNA_intro	RP5-1132H1	.	.	.	A	G	+	-+	0.03333333	87,0,3,0	chr7,656182	Cytosol	A > G
chr7:65618239:A:G:ncRNA_introni	chr7	65618239	65618239	A	G	ncRNA_intro	RP5-1132H1	.	.	.	A	G	+	-+	0.04	120,0,5,0	chr7,656182	Cytosol	A > G
chr7:65618246:A:G:ncRNA_introni	chr7	65618246	65618246	A	G	ncRNA_intro	RP5-1132H1	.	.	.	A	G	+	-+	0.03731343	129,0,5,0	chr7,656182	Cytosol	A > G
chr7:65618254:A:G:ncRNA_introni	chr7	65618254	65618254	A	G	ncRNA_intro	RP5-1132H1	.	.	.	A	G	+	-+	0.02654867	110,0,3,0	chr7,656182	Cytosol	A > G
chr7:65618271:A:G:ncRNA_introni	chr7	65618271	65618271	A	G	ncRNA_intro	RP5-1132H1	.	.	.	A	G	+	-+	0.03472222	139,0,5,0	chr7,656182	Cytosol	A > G
chr7:65618279:A:G:ncRNA_introni	chr7	65618279	65618279	A	G	ncRNA_intro	RP5-1132H1	.	.	.	A	G	+	-+	0.08064516	114,0,10,0	chr7,656182	Cytosol	A > G
chr7:65618280:A:G:ncRNA_introni	chr7	65618280	65618280	A	G	ncRNA_intro	RP5-1132H1	.	.	.	A	G	+	-+	0.03333333	116,0,4,0	chr7,656182	Cytosol	A > G
chr7:65618295:A:G:ncRNA_introni	chr7	65618295	65618295	A	G	ncRNA_intro	RP5-1132H1	.	.	.	A	G	+	-+	0.0776699	95,0,8,0	chr7,656182	Cytosol	A > G
chr7:65618297:A:G:ncRNA_introni	chr7	65618297	65618297	A	G	ncRNA_intro	RP5-1132H1	.	.	.	A	G	+	-+	0.05504587	103,0,6,0	chr7,656182	Cytosol	A > G
chr7:65618305:A:G:ncRNA_introni	chr7	65618305	65618305	A	G	ncRNA_intro	RP5-1132H1	.	.	.	A	G	+	-+	0.13513514	96,0,15,0	chr7,656183	Cytosol	A > G
chr7:65618306:A:G:ncRNA_introni	chr7	65618306	65618306	A	G	ncRNA_intro	RP5-1132H1	.	.	.	A	G	+	-+	0.16037736	89,0,17,0	chr7,656183	Cytosol	A > G
chr7:65618315:A:G:ncRNA_introni	chr7	65618315	65618315	A	G	ncRNA_intro	RP5-1132H1	.	.	.	A	G	+	-+	0.06741573	83,0,6,0	chr7,656183	Cytosol	A > G
chr7:65618332:A:G:ncRNA_introni	chr7	65618332	65618332	A	G	ncRNA_intro	RP5-1132H1	.	.	.	A	G	+	-+	0.15714286	59,0,11,0	chr7,656183	Cytosol	A > G
chr7:65618358:A:G:ncRNA_introni	chr7	65618358	65618358	A	G	ncRNA_intro	RP5-1132H1	.	.	.	A	G	+	-+	0.03508772	55,0,2,0	chr7,656183	Cytosol	A > G
chr7:65618786:A:G:ncRNA_introni	chr7	65618786	65618786	A	G	ncRNA_intro	RP5-1132H1	.	.	.	A	G	+	-+	0.09160305	119,0,12,0	chr7,656187	Cytosol	A > G
chr7:65618792:A:G:ncRNA_introni	chr7	65618792	65618792	A	G	ncRNA_intro	RP5-1132H1	.	.	.	A	G	+	-+	0.01574803	125,0,2,0	chr7,656187	Cytosol	A > G
chr7:65618793:A:G:ncRNA_introni	chr7	65618793	65618793	A	G	ncRNA_intro	RP5-1132H1	.	.	.	A	G	+	-+	0.015625	126,0,2,0	chr7,656187	Cytosol	A > G
chr7:65618862:A:G:ncRNA_introni	chr7	65618862	65618862	A	G	ncRNA_intro	RP5-1132H1	.	.	.	A	G	+	-+	0.0375	77,0,3,0	chr7,656188	Cytosol	A > G
chr7:65618870:A:G:ncRNA_introni	chr7	65618870	65618870	A	G	ncRNA_intro	RP5-1132H1	.	.	.	A	G	+	-+	0.14285714	60,0,10,0	chr7,656188	Cytosol	A > G
chr7:65618887:A:G:ncRNA_introni	chr7	65618887	65618887	A	G	ncRNA_intro	RP5-1132H1	.	.	.	A	G	+	-+	0.04819277	79,0,4,0	chr7,656188	Cytosol	A > G
chr7:65618901:A:G:ncRNA_introni	chr7	65618901	65618901	A	G	ncRNA_intro	RP5-1132H1	.	.	.	A	G	+	-+	0.03191489	91,0,3,0	chr7,656189	Cytosol	A > G
chr7:65618928:A:G:ncRNA_introni	chr7	65618928	65618928	A	G	ncRNA_intro	RP5-1132H1	.	.	.	A	G	+	-+	0.02830189	103,0,3,0	chr7,656189	Cytosol	A > G
chr7:65618937:A:G:ncRNA_introni	chr7	65618937	65618937	A	G	ncRNA_intro	RP5-1132H1	.	.	.	A	G	+	-+	0.02459016	119,0,3,0	chr7,656189	Cytosol	A > G
chr7:65618960:A:G:ncRNA_introni	chr7	65618960	65618960	A	G	ncRNA_intro	RP5-1132H1	.	.	.	A	G	+	-+	0.03174603	122,0,4,0	chr7,656189	Cytosol	A > G
chr7:65618980:A:G:ncRNA_introni	chr7	65618980	65618980	A	G	ncRNA_intro	RP5-1132H1	.	.	.	A	G	+	-+	0.06818182	123,0,9,0	chr7,656189	Cytosol	A > G
chr7:65619237:A:G:ncRNA_introni	chr7	65619237	65619237	A	G	ncRNA_intro	RP5-1132H1	.	.	.	A	G	+	-+	0.23809524	96,0,30,0	chr7,656192	Cytosol	A > G
chr7:65619242:A:G:ncRNA_introni	chr7	65619242	65619242	A	G	ncRNA_intro	RP5-1132H1	.	.	.	A	G	+	-+	0.13868613	118,0,19,0	chr7,656192	Cytosol	A > G
chr7:65619243:A:G:ncRNA_introni	chr7	65619243	65619243	A	G	ncRNA_intro	RP5-1132H1	.	.	.	A	G	+	-+	0.125	119,0,17,0	chr7,656192	Cytosol	A > G
chr7:65619256:A:G:ncRNA_introni	chr7	65619256	65619256	A	G	ncRNA_intro	RP5-1132H1	.	.	.	A	G	+	-+	0.04022989	167,0,7,0	chr7,656192	Cytosol	A > G
chr7:65619263:A:G:ncRNA_introni	chr7	65619263	65619263	A	G	ncRNA_intro	RP5-1132H1	.	.	.	A	G	+	-+	0.03355705	144,0,5,0	chr7,656192	Cytosol	A > G
chr7:65619292:A:G:ncRNA_introni	chr7	65619292	65619292	A	G	ncRNA_intro	RP5-1132H1	.	.	.	A	G	+	-+	0.26206897	107,0,38,0	chr7,656192	Cytosol	A > G
chr7:65619313:A:G:ncRNA_introni	chr7	65619313	65619313	A	G	ncRNA_intro	RP5-1132H1	.	.	.	A	G	+	-+	0.01538462	128,0,2,0	chr7,656193	Cytosol	A > G

chr7:65619329:A:G:ncRNA_introni	chr7	65619329	65619329	A	G	ncRNA_intro	RP5-1132H1	.	.	.	A	G	+	-+	0.04255319	90,0,4,0	chr7,656193	Cytosol	A > G
chr7:65619344:A:G:ncRNA_introni	chr7	65619344	65619344	A	G	ncRNA_intro	RP5-1132H1	.	.	.	A	G	+	-+	0.05882353	112,0,7,0	chr7,656193	Cytosol	A > G
chr7:65619347:A:G:ncRNA_introni	chr7	65619347	65619347	A	G	ncRNA_intro	RP5-1132H1	.	.	.	A	G	+	-+	0.096	113,0,12,0	chr7,656193	Cytosol	A > G
chr7:65619353:A:G:ncRNA_introni	chr7	65619353	65619353	A	G	ncRNA_intro	RP5-1132H1	.	.	.	A	G	+	-+	0.03937008	122,0,5,0	chr7,656193	Cytosol	A > G
chr7:65619366:A:G:ncRNA_introni	chr7	65619366	65619366	A	G	ncRNA_intro	RP5-1132H1	.	.	.	A	G	+	-+	0.07741935	143,0,12,0	chr7,656193	Cytosol	A > G
chr7:65620307:A:G:ncRNA_introni	chr7	65620307	65620307	A	G	ncRNA_intro	RP5-1132H1	.	.	.	A	G	+	-	0.16666667	15,0,3,0	chr7,656203	Cytosol	A > G
chr7:65620547:A:G:ncRNA_introni	chr7	65620547	65620547	A	G	ncRNA_intro	RP5-1132H1	.	.	.	A	G	+	-	0.25	6,0,2,0	chr7,656205	Cytosol	A > G
chr7:65620566:A:G:ncRNA_introni	chr7	65620566	65620566	A	G	ncRNA_intro	RP5-1132H1	.	.	.	A	G	+	-	0.53846154	6,0,7,0	chr7,656205	Cytosol	A > G
chr7:65620592:A:G:ncRNA_introni	chr7	65620592	65620592	A	G	ncRNA_intro	RP5-1132H1	.	.	.	A	G	+	-	0.11764706	15,0,2,0	chr7,656205	Cytosol	A > G
chr7:65620620:A:G:ncRNA_introni	chr7	65620620	65620620	A	G	ncRNA_intro	RP5-1132H1	.	.	.	A	G	+	-	0.2	16,0,4,0	chr7,656206	Cytosol	A > G
chr7:65620621:A:G:ncRNA_introni	chr7	65620621	65620621	A	G	ncRNA_intro	RP5-1132H1	.	.	.	A	G	+	-	0.36842105	12,0,7,0	chr7,656206	Cytosol	A > G
chr7:65620624:A:G:ncRNA_introni	chr7	65620624	65620624	A	G	ncRNA_intro	RP5-1132H1	.	.	.	A	G	+	-	0.5	9,0,9,0	chr7,656206	Cytosol	A > G
chr7:65620633:A:G:ncRNA_introni	chr7	65620633	65620633	A	G	ncRNA_intro	RP5-1132H1	.	.	.	A	G	+	-	0.6	4,0,6,0	chr7,656206	Cytosol	A > G
chr7:65620650:A:G:ncRNA_introni	chr7	65620650	65620650	A	G	ncRNA_intro	RP5-1132H1	.	.	.	A	G	+	-	0.42857143	8,0,6,0	chr7,656206	Cytosol	A > G
chr7:65620660:A:G:ncRNA_introni	chr7	65620660	65620660	A	G	ncRNA_intro	RP5-1132H1	.	.	.	A	G	+	-	0.46666667	8,0,7,0	chr7,656206	Cytosol	A > G
chr7:65620697:A:G:ncRNA_introni	chr7	65620697	65620697	A	G	ncRNA_intro	RP5-1132H1	.	.	.	A	G	+	-	0.07142857	26,0,2,0	chr7,656206	Cytosol	A > G
chr7:65620734:A:G:ncRNA_introni	chr7	65620734	65620734	A	G	ncRNA_intro	RP5-1132H1	.	.	.	A	G	+	-	0.45454545	12,0,10,0	chr7,656207	Cytosol	A > G
chr7:65620759:A:G:ncRNA_introni	chr7	65620759	65620759	A	G	ncRNA_intro	RP5-1132H1	.	.	.	A	G	+	-	0.4375	9,0,7,0	chr7,656207	Cytosol	A > G
chr7:65620762:A:G:ncRNA_introni	chr7	65620762	65620762	A	G	ncRNA_intro	RP5-1132H1	.	.	.	A	G	+	-	0.23529412	13,0,4,0	chr7,656207	Cytosol	A > G
chr7:65620776:A:G:ncRNA_introni	chr7	65620776	65620776	A	G	ncRNA_intro	RP5-1132H1	.	.	.	A	G	+	-	0.54545455	10,0,12,0	chr7,656207	Cytosol	A > G
chr7:65620798:A:G:ncRNA_introni	chr7	65620798	65620798	A	G	ncRNA_intro	RP5-1132H1	.	.	.	A	G	+	-	0.26666667	11,0,4,0	chr7,656207	Cytosol	A > G
chr7:65621604:A:G:ncRNA_introni	chr7	65621604	65621604	A	G	ncRNA_intro	RP5-1132H1	.	.	.	A	G	+	-	0.23076923	10,0,3,0	chr7,656216	Cytosol	A > G
chr7:65621988:A:G:ncRNA_introni	chr7	65621988	65621988	A	G	ncRNA_intro	RP5-1132H1	.	.	.	A	G	+	-	0.33333333	4,0,2,0	chr7,656219	Cytosol	A > G
chr7:65622039:A:G:ncRNA_introni	chr7	65622039	65622039	A	G	ncRNA_intro	RP5-1132H1	.	.	.	A	G	+	-	0.125	14,0,2,0	chr7,656220	Cytosol	A > G
chr7:65622624:A:G:ncRNA_introni	chr7	65622624	65622624	A	G	ncRNA_intro	RP5-1132H1	.	.	.	A	G	+	-	0.33333333	4,0,2,0	chr7,656226	Cytosol	A > G
chr7:65622626:A:G:ncRNA_introni	chr7	65622626	65622626	A	G	ncRNA_intro	RP5-1132H1	.	.	.	A	G	+	-	0.28571429	5,0,2,0	chr7,656226	Cytosol	A > G
chr7:65622847:A:G:ncRNA_introni	chr7	65622847	65622847	A	G	ncRNA_intro	RP5-1132H1	.	.	.	A	G	+	-	0.33333333	4,0,2,0	chr7,656228	Cytosol	A > G
chr7:65622871:A:G:ncRNA_introni	chr7	65622871	65622871	A	G	ncRNA_intro	RP5-1132H1	.	.	.	A	G	+	-	0.55555556	4,0,5,0	chr7,656228	Cytosol	A > G
chr7:65622972:A:G:ncRNA_introni	chr7	65622972	65622972	A	G	ncRNA_intro	RP5-1132H1	.	.	.	A	G	+	-	0.63636364	4,0,7,0	chr7,656229	Cytosol	A > G
chr7:65623011:A:G:ncRNA_introni	chr7	65623011	65623011	A	G	ncRNA_intro	RP5-1132H1	.	.	.	A	G	+	-	0.375	5,0,3,0	chr7,656230	Cytosol	A > G
chr7:65623014:A:G:ncRNA_introni	chr7	65623014	65623014	A	G	ncRNA_intro	RP5-1132H1	.	.	.	A	G	+	-	0.75	2,0,6,0	chr7,656230	Cytosol	A > G
chr7:65623064:A:G:ncRNA_introni	chr7	65623064	65623064	A	G	ncRNA_intro	RP5-1132H1	.	.	.	A	G	+	-	0.33333333	8,0,4,0	chr7,656230	Cytosol	A > G
chr7:65623065:A:G:ncRNA_introni	chr7	65623065	65623065	A	G	ncRNA_intro	RP5-1132H1	.	.	.	A	G	+	-	0.23076923	10,0,3,0	chr7,656230	Cytosol	A > G
chr7:65623066:A:G:ncRNA_introni	chr7	65623066	65623066	A	G	ncRNA_intro	RP5-1132H1	.	.	.	A	G	+	-	0.58333333	5,0,7,0	chr7,656230	Cytosol	A > G
chr7:65956517:T:C:ncRNA_introni	chr7	65956517	65956517	T	C	ncRNA_intro	GS1-124K5.3	.	.	.	A	G	-	-	0.4	3,0,2,0	chr7,659565	Cytosol	A > G
chr7:65956534:T:C:ncRNA_introni	chr7	65956534	65956534	T	C	ncRNA_intro	GS1-124K5.3	.	.	.	A	G	-	-	0.4	3,0,2,0	chr7,659565	Cytosol	A > G
chr7:65993627:T:C:ncRNA_exonic:	chr7	65993627	65993627	T	C	ncRNA_exon	GS1-124K5.1	.	.	.	A	G	-	+-	0.6	2,0,3,0	chr7,659936	Cytosol	A > G
chr7:66010321:T:C:intergenic:GS1	chr7	66010321	66010321	T	C	intergenic	GS1-124K5.1	dist=2785;di	.	.	A	G	-	-	0.3	7,0,3,0	chr7,660103	Cytosol	A > G
chr7:66010324:T:C:intergenic:GS1	chr7	66010324	66010324	T	C	intergenic	GS1-124K5.1	dist=2788;di	.	.	A	G	-	-	0.33333333	6,0,3,0	chr7,660103	Cytosol	A > G
chr7:66010402:T:C:intergenic:GS1	chr7	66010402	66010402	T	C	intergenic	GS1-124K5.1	dist=2866;di	.	.	A	G	-	-	0.5	3,0,3,0	chr7,660104	Cytosol	A > G
chr7:66010437:T:C:intergenic:GS1	chr7	66010437	66010437	T	C	intergenic	GS1-124K5.1	dist=2901;di	.	.	A	G	-	-	0.6	2,0,3,0	chr7,660104	Cytosol	A > G
chr7:66012533:T:C:intergenic:GS1	chr7	66012533	66012533	T	C	intergenic	GS1-124K5.1	dist=4997;di	.	.	A	G	-	-	0.28571429	5,0,2,0	chr7,660125	Cytosol	A > G
chr7:66150409:A:G:intronic:KCTD7	chr7	66150409	66150409	A	G	intronic	KCTD7,RABG	.	.	.	A	G	+	+	0.14705882	29,0,5,0	chr7,661504	Cytosol	A > G
chr7:66180770:A:G:intronic:KCTD7	chr7	66180770	66180770	A	G	intronic	KCTD7,RABG	.	.	.	A	G	+	+	0.07692308	24,0,2,0	chr7,661807	Cytosol	A > G
chr7:66180775:A:G:intronic:KCTD7	chr7	66180775	66180775	A	G	intronic	KCTD7,RABG	.	.	.	A	G	+	+	0.25	21,0,7,0	chr7,661807	Cytosol	A > G
chr7:66180818:A:G:intronic:KCTD7	chr7	66180818	66180818	A	G	intronic	KCTD7,RABG	.	.	.	A	G	+	+	0.24324324	28,0,9,0	chr7,661808	Cytosol	A > G
chr7:66180840:A:G:intronic:KCTD7	chr7	66180840	66180840	A	G	intronic	KCTD7,RABG	.	.	.	A	G	+	+	0.16	21,0,4,0	chr7,661808	Cytosol	A > G
chr7:66181359:A:G:intronic:KCTD7	chr7	66181359	66181359	A	G	intronic	KCTD7,RABG	.	.	.	A	G	+	+	0.4	3,0,2,0	chr7,661813	Cytosol	A > G
chr7:66181445:A:G:intronic:KCTD7	chr7	66181445	66181445	A	G	intronic	KCTD7,RABG	.	.	.	A	G	+	+	0.5	3,0,3,0	chr7,661814	Cytosol	A > G
chr7:66181467:A:G:intronic:KCTD7	chr7	66181467	66181467	A	G	intronic	KCTD7,RABG	.	.	.	A	G	+	+	0.4	3,0,2,0	chr7,661814	Cytosol	A > G
chr7:66181843:A:G:intronic:KCTD7	chr7	66181843	66181843	A	G	intronic	KCTD7,RABG	.	.	.	A	G	+	+	0.75	2,0,6,0	chr7,661818	Cytosol	A > G
chr7:66181866:A:G:intronic:KCTD7	chr7	66181866	66181866	A	G	intronic	KCTD7,RABG	.	.	.	A	G	+	+	0.33333333	6,0,3,0	chr7,661818	Cytosol	A > G
chr7:66205084:T:C:ncRNA_exonic:	chr7	66205084	66205084	T	C	ncRNA_exon	RP11-792A8.	.	.	.	A	G	-	-+	0.13636364	19,0,3,0	chr7,662050	Cytosol	A > G
chr7:66205087:T:C:ncRNA_exonic:	chr7	66205087	66205087	T	C	ncRNA_exon	RP11-792A8.	.	.	.	A	G	-	-+	0.17391304	19,0,4,0	chr7,662050	Cytosol	A > G
chr7:66739263:T:C:intergenic:TYW	chr7	66739263	66739263	T	C	intergenic	TYW1,PMS2P	dist=34762;d	.	.	A	G	-	-	0.25	6,0,2,0	chr7,667392	Cytosol	A > G
chr7:72876369:T:C:intronic:BAZ1B	chr7	72876369	72876369	T	C	intronic	BAZ1B	.	.	.	A	G	-	-	0.4	3,0,2,0	chr7,728763	Cytosol	A > G
chr7:73247351:T:C:downstream:C	chr7	73247351	73247351	T	C	downstream	CLDN4	.	.	.	A	G	-		1	0,0,7,0	chr7,732473	Cytosol	A > G
chr7:73247378:T:C:downstream:C	chr7	73247378	73247378	T	C	downstream	CLDN4	.	.	.	A	G	-		0.36363636	7,0,4,0	chr7,732473	Cytosol	A > G
chr7:73247387:T:C:downstream:C	chr7	73247387	73247387	T	C	downstream	CLDN4	.	.	.	A	G	-		0.5	5,0,5,0	chr7,732473	Cytosol	A > G
chr7:73247390:T:C:downstream:C	chr7	73247390	73247390	T	C	downstream	CLDN4	.	.	.	A	G	-		0.5	5,0,5,0	chr7,732473	Cytosol	A > G
chr7:73248713:T:C:downstream:W	chr7	73248713	73248713	T	C	downstream	WBSCR27	.	.	.	A	G	-		0.8	1,0,4,0	chr7,732487	Cytosol	A > G
chr7:73645042:T:C:downstream:L	chr7	73645042	73645042	T	C	downstream	LAT2,RFC2	.	.	.	A	G	-		0.7	3,0,7,0	chr7,736450	Cytosol	A > G
chr7:73645068:T:C:downstream:L	chr7	73645068	73645068	T	C	downstream	LAT2,RFC2	.	.	.	A	G	-		0.69230769	4,0,9,0	chr7,736450	Cytosol	A > G
chr7:73645069:T:C:downstream:L	chr7	73645069	73645069	T	C	downstream	LAT2,RFC2	.	.	.	A	G	-		0.64285714	5,0,9,0	chr7,736450	Cytosol	A > G

chr7:73645328:T:C:downstream:R	chr7	73645328	73645328	T	C	downstream	RFC2	.	.	.	A	G	-		1	0,0,9,0	chr7,736453	Cytosol	A > G
chr7:73645340:T:C:downstream:R	chr7	73645340	73645340	T	C	downstream	RFC2	.	.	.	A	G	-		0.375	5,0,3,0	chr7,736453	Cytosol	A > G
chr7:73646107:T:C:UTR3:RFC2	chr7	73646107	73646107	T	C	UTR3	RFC2	ENST000003	.	.	A	G	-	-	0.08205128	179,0,16,0	chr7,736461	Cytosol	A > G
chr7:73646147:T:C:UTR3:RFC2	chr7	73646147	73646147	T	C	UTR3	RFC2	ENST000003	.	.	A	G	-	-	0.18072289	136,0,30,0	chr7,736461	Cytosol	A > G
chr7:73646174:T:C:UTR3:RFC2	chr7	73646174	73646174	T	C	UTR3	RFC2	ENST000003	.	.	A	G	-	-	0.17647059	56,0,12,0	chr7,736461	Cytosol	A > G
chr7:73646213:T:C:UTR3:RFC2	chr7	73646213	73646213	T	C	UTR3	RFC2	ENST000003	.	.	A	G	-	-	0.02643172	221,0,6,0	chr7,736462	Cytosol	A > G
chr7:73646214:T:C:UTR3:RFC2	chr7	73646214	73646214	T	C	UTR3	RFC2	ENST000003	.	.	A	G	-	-	0.02880658	236,0,7,0	chr7,736462	Cytosol	A > G
chr7:73646229:T:C:UTR3:RFC2	chr7	73646229	73646229	T	C	UTR3	RFC2	ENST000003	.	.	A	G	-	-	0.05015674	303,0,16,0	chr7,736462	Cytosol	A > G
chr7:74088487:A:G:intronic:GTF2I	chr7	74088487	74088487	A	G	intronic	GTF2I	.	.	.	A	G	+	+	0.21052632	15,0,4,0	chr7,740884	Cytosol	A > G
chr7:74088563:A:G:intronic:GTF2I	chr7	74088563	74088563	A	G	intronic	GTF2I	.	.	.	A	G	+	+	0.125	21,0,3,0	chr7,740885	Cytosol	A > G
chr7:74088600:A:G:intronic:GTF2I	chr7	74088600	74088600	A	G	intronic	GTF2I	.	.	.	A	G	+	+	0.11538462	23,0,3,0	chr7,740885	Cytosol	A > G
chr7:74088642:A:G:intronic:GTF2I	chr7	74088642	74088642	A	G	intronic	GTF2I	.	.	.	A	G	+	+	0.14285714	12,0,2,0	chr7,740886	Cytosol	A > G
chr7:74294767:T:C:intergenic:GTF	chr7	74294767	74294767	T	C	intergenic	GTF2IRD2,ST	dist=26920;d	.	.	A	G	-	-	0.35714286	9,0,5,0	chr7,742947	Cytosol	A > G
chr7:74294786:T:C:intergenic:GTF	chr7	74294786	74294786	T	C	intergenic	GTF2IRD2,ST	dist=26939;d	.	.	A	G	-	-	0.11111111	16,0,2,0	chr7,742947	Cytosol	A > G
chr7:74296504:T:C:intergenic:GTF	chr7	74296504	74296504	T	C	intergenic	GTF2IRD2,ST	dist=28657;d	.	.	A	G	-	-	0.6	2,0,3,0	chr7,742965	Cytosol	A > G
chr7:74296509:T:C:intergenic:GTF	chr7	74296509	74296509	T	C	intergenic	GTF2IRD2,ST	dist=28662;d	.	.	A	G	-	-	0.4	3,0,2,0	chr7,742965	Cytosol	A > G
chr7:74296521:T:C:intergenic:GTF	chr7	74296521	74296521	T	C	intergenic	GTF2IRD2,ST	dist=28674;d	.	.	A	G	-	-	1	0,0,5,0	chr7,742965	Cytosol	A > G
chr7:74296627:T:C:intergenic:GTF	chr7	74296627	74296627	T	C	intergenic	GTF2IRD2,ST	dist=28780;d	.	.	A	G	-	-	0.71428571	2,0,5,0	chr7,742966	Cytosol	A > G
chr7:74296653:T:C:intergenic:GTF	chr7	74296653	74296653	T	C	intergenic	GTF2IRD2,ST	dist=28806;d	.	.	A	G	-	-	0.18181818	9,0,2,0	chr7,742966	Cytosol	A > G
chr7:74296674:T:C:intergenic:GTF	chr7	74296674	74296674	T	C	intergenic	GTF2IRD2,ST	dist=28827;d	.	.	A	G	-	-	0.85714286	2,0,12,0	chr7,742966	Cytosol	A > G
chr7:74296985:T:C:intergenic:GTF	chr7	74296985	74296985	T	C	intergenic	GTF2IRD2,ST	dist=29138;d	.	.	A	G	-	-	0.45454545	6,0,5,0	chr7,742969	Cytosol	A > G
chr7:74297042:T:C:intergenic:GTF	chr7	74297042	74297042	T	C	intergenic	GTF2IRD2,ST	dist=29195;d	.	.	A	G	-	-	0.28	18,0,7,0	chr7,742970	Cytosol	A > G
chr7:74297047:T:C:intergenic:GTF	chr7	74297047	74297047	T	C	intergenic	GTF2IRD2,ST	dist=29200;d	.	.	A	G	-	-	0.23809524	16,0,5,0	chr7,742970	Cytosol	A > G
chr7:74297433:T:C:intergenic:GTF	chr7	74297433	74297433	T	C	intergenic	GTF2IRD2,ST	dist=29586;d	.	.	A	G	-	-	0.33333333	4,0,2,0	chr7,742974	Cytosol	A > G
chr7:74297454:T:C:intergenic:GTF	chr7	74297454	74297454	T	C	intergenic	GTF2IRD2,ST	dist=29607;d	.	.	A	G	-	-	0.13333333	13,0,2,0	chr7,742974	Cytosol	A > G
chr7:74297481:T:C:intergenic:GTF	chr7	74297481	74297481	T	C	intergenic	GTF2IRD2,ST	dist=29634;d	.	.	A	G	-	-	0.42857143	8,0,6,0	chr7,742974	Cytosol	A > G
chr7:74297485:T:C:intergenic:GTF	chr7	74297485	74297485	T	C	intergenic	GTF2IRD2,ST	dist=29638;d	.	.	A	G	-	-	0.33333333	10,0,5,0	chr7,742974	Cytosol	A > G
chr7:74297486:T:C:intergenic:GTF	chr7	74297486	74297486	T	C	intergenic	GTF2IRD2,ST	dist=29639;d	.	.	A	G	-	-	0.42857143	8,0,6,0	chr7,742974	Cytosol	A > G
chr7:74297494:T:C:intergenic:GTF	chr7	74297494	74297494	T	C	intergenic	GTF2IRD2,ST	dist=29647;d	.	.	A	G	-	-	1	0,0,11,0	chr7,742974	Cytosol	A > G
chr7:74297651:T:C:intergenic:GTF	chr7	74297651	74297651	T	C	intergenic	GTF2IRD2,ST	dist=29804;d	.	.	A	G	-	-	0.6	2,0,3,0	chr7,742976	Cytosol	A > G
chr7:74298142:T:C:intergenic:GTF	chr7	74298142	74298142	T	C	intergenic	GTF2IRD2,ST	dist=30295;d	.	.	A	G	-	-	0.66666667	2,0,4,0	chr7,742981	Cytosol	A > G
chr7:74298156:T:C:intergenic:GTF	chr7	74298156	74298156	T	C	intergenic	GTF2IRD2,ST	dist=30309;d	.	.	A	G	-	-	0.25	6,0,2,0	chr7,742981	Cytosol	A > G
chr7:74298709:T:C:intergenic:GTF	chr7	74298709	74298709	T	C	intergenic	GTF2IRD2,ST	dist=30862;d	.	.	A	G	-	-	0.93939394	2,0,31,0	chr7,742987	Cytosol	A > G
chr7:74298717:T:C:intergenic:GTF	chr7	74298717	74298717	T	C	intergenic	GTF2IRD2,ST	dist=30870;d	.	.	A	G	-	-	0.15151515	28,0,5,0	chr7,742987	Cytosol	A > G
chr7:74298734:T:C:intergenic:GTF	chr7	74298734	74298734	T	C	intergenic	GTF2IRD2,ST	dist=30887;d	.	.	A	G	-	-	0.89795918	5,0,44,0	chr7,742987	Cytosol	A > G
chr7:74298740:T:C:intergenic:GTF	chr7	74298740	74298740	T	C	intergenic	GTF2IRD2,ST	dist=30893;d	.	.	A	G	-	-	0.16	42,0,8,0	chr7,742987	Cytosol	A > G
chr7:74298777:T:C:intergenic:GTF	chr7	74298777	74298777	T	C	intergenic	GTF2IRD2,ST	dist=30930;d	.	.	A	G	-	-	0.12195122	36,0,5,0	chr7,742987	Cytosol	A > G
chr7:74298787:T:C:intergenic:GTF	chr7	74298787	74298787	T	C	intergenic	GTF2IRD2,ST	dist=30940;d	.	.	A	G	-	-	0.05405405	35,0,2,0	chr7,742987	Cytosol	A > G
chr7:74997682:A:G:ncRNA_intron	chr7	74997682	74997682	A	G	ncRNA_intro	STAG3L1	.	.	.	A	G	+	+	1	0,0,6,0	chr7,749976	Cytosol	A > G
chr7:74997690:A:G:ncRNA_intron	chr7	74997690	74997690	A	G	ncRNA_intro	STAG3L1	.	.	.	A	G	+	+	0.5	3,0,3,0	chr7,749976	Cytosol	A > G
chr7:75512716:A:G:intronic:RHBD	chr7	75512716	75512716	A	G	intronic	RHBDD2	.	.	.	A	G	+	+	0.6	2,0,3,0	chr7,755127	Cytosol	A > G
chr7:77409278:A:G:UTR3:RSBN1L	chr7	77409278	77409278	A	G	UTR3	RSBN1L	ENST000003	.	.	A	G	+	+	0.66666667	2,0,4,0	chr7,774092	Cytosol	A > G
chr7:90015263:A:G:UTR3:GTPBP1	chr7	90015263	90015263	A	G	UTR3	GTPBP10	ENST000002	.	.	A	G	+	+	0.06	47,0,3,0	chr7,900152	Cytosol	A > G
chr7:90015267:A:G:UTR3:GTPBP1	chr7	90015267	90015267	A	G	UTR3	GTPBP10	ENST000002	.	.	A	G	+	+	0.14	43,0,7,0	chr7,900152	Cytosol	A > G
chr7:90015268:A:G:UTR3:GTPBP1	chr7	90015268	90015268	A	G	UTR3	GTPBP10	ENST000002	.	.	A	G	+	+	0.06	47,0,3,0	chr7,900152	Cytosol	A > G
chr7:90018765:A:G:UTR3:GTPBP1	chr7	90018765	90018765	A	G	UTR3	GTPBP10	ENST000002	.	.	A	G	+	+	0.16666667	10,0,2,0	chr7,900187	Cytosol	A > G
chr7:90018801:A:G:UTR3:GTPBP1	chr7	90018801	90018801	A	G	UTR3	GTPBP10	ENST000002	.	.	A	G	+	+	0.54545455	5,0,6,0	chr7,900188	Cytosol	A > G
chr7:90018804:A:G:UTR3:GTPBP1	chr7	90018804	90018804	A	G	UTR3	GTPBP10	ENST000002	.	.	A	G	+	+	0.76923077	3,0,10,0	chr7,900188	Cytosol	A > G
chr7:90018805:A:G:UTR3:GTPBP1	chr7	90018805	90018805	A	G	UTR3	GTPBP10	ENST000002	.	.	A	G	+	+	0.18181818	9,0,2,0	chr7,900188	Cytosol	A > G
chr7:90018866:A:G:UTR3:GTPBP1	chr7	90018866	90018866	A	G	UTR3	GTPBP10	ENST000002	.	.	A	G	+	+	0.71428571	4,0,10,0	chr7,900188	Cytosol	A > G
chr7:90018867:A:G:UTR3:GTPBP1	chr7	90018867	90018867	A	G	UTR3	GTPBP10	ENST000002	.	.	A	G	+	+	0.2	12,0,3,0	chr7,900188	Cytosol	A > G
chr7:90018884:A:G:UTR3:GTPBP1	chr7	90018884	90018884	A	G	UTR3	GTPBP10	ENST000002	.	.	A	G	+	+	0.76470588	4,0,13,0	chr7,900188	Cytosol	A > G
chr7:90019131:A:G:UTR3:GTPBP1	chr7	90019131	90019131	A	G	UTR3	GTPBP10	ENST000002	.	.	A	G	+	+	0.15789474	16,0,3,0	chr7,900191	Cytosol	A > G
chr7:90019235:A:G:UTR3:GTPBP1	chr7	90019235	90019235	A	G	UTR3	GTPBP10	ENST000002	.	.	A	G	+	+	0.95454545	1,0,21,0	chr7,900192	Cytosol	A > G
chr7:90019241:A:G:UTR3:GTPBP1	chr7	90019241	90019241	A	G	UTR3	GTPBP10	ENST000002	.	.	A	G	+	+	0.69565217	7,0,16,0	chr7,900192	Cytosol	A > G
chr7:90019244:A:G:UTR3:GTPBP1	chr7	90019244	90019244	A	G	UTR3	GTPBP10	ENST000002	.	.	A	G	+	+	0.57142857	9,0,12,0	chr7,900192	Cytosol	A > G
chr7:90019294:A:G:UTR3:GTPBP1	chr7	90019294	90019294	A	G	UTR3	GTPBP10	ENST000002	.	.	A	G	+	+	0.5862069	12,0,17,0	chr7,900192	Cytosol	A > G
chr7:90019367:A:G:UTR3:GTPBP1	chr7	90019367	90019367	A	G	UTR3	GTPBP10	ENST000002	.	.	A	G	+	+	0.31818182	15,0,7,0	chr7,900193	Cytosol	A > G
chr7:90019373:A:G:UTR3:GTPBP1	chr7	90019373	90019373	A	G	UTR3	GTPBP10	ENST000002	.	.	A	G	+	+	0.42857143	16,0,12,0	chr7,900193	Cytosol	A > G
chr7:91829737:T:C:ncRNA_exonic:	chr7	91829737	91829737	T	C	ncRNA_exon	AC000120.7	.	.	.	A	G	-	-	0.03389831	57,0,2,0	chr7,918297	Cytosol	A > G
chr7:91829761:T:C:ncRNA_intron	chr7	91829761	91829761	T	C	ncRNA_intro	AC000120.7	.	.	.	A	G	-	-	0.06034483	109,0,7,0	chr7,918297	Cytosol	A > G
chr7:91829766:T:C:ncRNA_intron	chr7	91829766	91829766	T	C	ncRNA_intro	AC000120.7	.	.	.	A	G	-	-	0.03007519	129,0,4,0	chr7,918297	Cytosol	A > G
chr7:91829767:T:C:ncRNA_intron	chr7	91829767	91829767	T	C	ncRNA_intro	AC000120.7	.	.	.	A	G	-	-	0.05185185	128,0,7,0	chr7,918297	Cytosol	A > G
chr7:91829808:T:C:ncRNA_intron	chr7	91829808	91829808	T	C	ncRNA_intro	AC000120.7	.	.	.	A	G	-	-	0.08609272	138,0,13,0	chr7,918298	Cytosol	A > G

chr7:91829832:T:C:ncRNA_intron	chr7	91829832	91829832	T	C	ncRNA_intro	AC000120.7	.	.	.	A	G	-	-	0.0130719	151,0,2,0	chr7,918298	Cytosol	A > G
chr7:91829836:T:C:ncRNA_intron	chr7	91829836	91829836	T	C	ncRNA_intro	AC000120.7	.	.	.	A	G	-	-	0.01129944	175,0,2,0	chr7,918298	Cytosol	A > G
chr7:91829844:T:C:ncRNA_intron	chr7	91829844	91829844	T	C	ncRNA_intro	AC000120.7	.	.	.	A	G	-	-	0.01197605	165,0,2,0	chr7,918298	Cytosol	A > G
chr7:91829885:T:C:ncRNA_intron	chr7	91829885	91829885	T	C	ncRNA_intro	AC000120.7	.	.	.	A	G	-	-	0.01342282	147,0,2,0	chr7,918298	Cytosol	A > G
chr7:91829886:T:C:ncRNA_intron	chr7	91829886	91829886	T	C	ncRNA_intro	AC000120.7	.	.	.	A	G	-	-	0.03401361	142,0,5,0	chr7,918298	Cytosol	A > G
chr7:91829897:T:C:ncRNA_intron	chr7	91829897	91829897	T	C	ncRNA_intro	AC000120.7	.	.	.	A	G	-	-	0.01470588	134,0,2,0	chr7,918298	Cytosol	A > G
chr7:92121522:A:G:intronic:PEX1	chr7	92121522	92121522	A	G	intronic	PEX1	.	.	.	A	G	+	-	0.6	2,0,3,0	chr7,921215	Cytosol	A > G
chr7:92166602:A:G:UTR3:RBM48	chr7	92166602	92166602	A	G	UTR3	RBM48	ENST000002	.	.	A	G	+	+	0.04878049	39,0,2,0	chr7,921666	Cytosol	A > G
chr7:94225952:T:C:intronic:SGCE	chr7	94225952	94225952	T	C	intronic	SGCE	.	.	.	A	G	-	-	0.5	4,0,4,0	chr7,942259	Cytosol	A > G
chr7:98975782:A:G:intronic:ARPC	chr7	98975782	98975782	A	G	intronic	ARPC1A,ARP	.	.	.	A	G	+	+	0.28571429	5,0,2,0	chr7,989757	Cytosol	A > G
chr7:98976291:A:G:intronic:ARPC	chr7	98976291	98976291	A	G	intronic	ARPC1A,ARP	.	.	.	A	G	+	+	0.4	3,0,2,0	chr7,989762	Cytosol	A > G
chr7:99079103:A:G:UTR3:ZNF789	chr7	99079103	99079103	A	G	UTR3	ZNF789	ENST000004	.	.	A	G	+	+	0.33333333	4,0,2,0	chr7,990791	Cytosol	A > G
chr7:99079142:A:G:UTR3:ZNF789	chr7	99079142	99079142	A	G	UTR3	ZNF789	ENST000004	.	.	A	G	+	+	0.15384615	11,0,2,0	chr7,990791	Cytosol	A > G
chr7:99079150:A:G:UTR3:ZNF789	chr7	99079150	99079150	A	G	UTR3	ZNF789	ENST000004	.	.	A	G	+	+	0.41666667	7,0,5,0	chr7,990791	Cytosol	A > G
chr7:99079181:A:G:UTR3:ZNF789	chr7	99079181	99079181	A	G	UTR3	ZNF789	ENST000004	.	.	A	G	+	+	0.2	8,0,2,0	chr7,990791	Cytosol	A > G
chr7:99079210:A:G:UTR3:ZNF789	chr7	99079210	99079210	A	G	UTR3	ZNF789	ENST000004	.	.	A	G	+	+	0.33333333	6,0,3,0	chr7,990792	Cytosol	A > G
chr7:99079220:A:G:UTR3:ZNF789	chr7	99079220	99079220	A	G	UTR3	ZNF789	ENST000004	.	.	A	G	+	+	0.4	6,0,4,0	chr7,990792	Cytosol	A > G
chr7:99079242:A:G:UTR3:ZNF789	chr7	99079242	99079242	A	G	UTR3	ZNF789	ENST000004	.	.	A	G	+	+	0.57142857	3,0,4,0	chr7,990792	Cytosol	A > G
chr7:99101695:A:G:upstream;dow	chr7	99101695	99101695	A	G	upstream;do	ZKSCAN5;ZN	.	.	.	A	G	+		0.8	1,0,4,0	chr7,991016	Cytosol	A > G
chr7:99144090:T:C:UTR3:FAM200	chr7	99144090	99144090	T	C	UTR3	FAM200A	ENST000004	.	.	A	G	-	-	0.08695652	42,0,4,0	chr7,991440	Cytosol	A > G
chr7:99172113:A:G:UTR3:ZNF655	chr7	99172113	99172113	A	G	UTR3	ZNF655	ENST000004	.	.	A	G	+	+	0.04477612	64,0,3,0	chr7,991721	Cytosol	A > G
chr7:99172126:A:G:UTR3:ZNF655	chr7	99172126	99172126	A	G	UTR3	ZNF655	ENST000004	.	.	A	G	+	+	0.03030303	64,0,2,0	chr7,991721	Cytosol	A > G
chr7:99172127:A:G:UTR3:ZNF655	chr7	99172127	99172127	A	G	UTR3	ZNF655	ENST000004	.	.	A	G	+	+	0.1641791	56,0,11,0	chr7,991721	Cytosol	A > G
chr7:99172900:A:G:UTR3:ZNF655	chr7	99172900	99172900	A	G	UTR3	ZNF655	ENST000004	.	.	A	G	+	+	0.86	7,0,43,0	chr7,991728	Cytosol	A > G
chr7:99649124:A:G:UTR5:ZSCAN2	chr7	99649124	99649124	A	G	UTR5	ZSCAN21	ENST000005	.	.	A	G	+	+	0.72727273	3,0,8,0	chr7,996491	Cytosol	A > G
chr7:99674450:T:C:intronic:ZNF3	chr7	99674450	99674450	T	C	intronic	ZNF3	.	.	.	A	G	-	-	0.42857143	4,0,3,0	chr7,996744	Cytosol	A > G
chr7:99712381:T:C:intronic:TAF6	chr7	99712381	99712381	T	C	intronic	TAF6	.	.	.	A	G	-	-	0.6	2,0,3,0	chr7,997123	Cytosol	A > G
chr7:99712408:T:C:intronic:TAF6	chr7	99712408	99712408	T	C	intronic	TAF6	.	.	.	A	G	-	-	0.6	2,0,3,0	chr7,997124	Cytosol	A > G
chr7:99716180:T:C:ncRNA_intron	chr7	99716180	99716180	T	C	ncRNA_intro	RP11-506M1	.	.	.	A	G	-	-	0.13333333	13,0,2,0	chr7,997161	Cytosol	A > G
chr7:99716198:T:C:ncRNA_intron	chr7	99716198	99716198	T	C	ncRNA_intro	RP11-506M1	.	.	.	A	G	-	-	0.16666667	15,0,3,0	chr7,997161	Cytosol	A > G
chr7:99716236:T:C:ncRNA_intron	chr7	99716236	99716236	T	C	ncRNA_intro	RP11-506M1	.	.	.	A	G	-	-	0.5	9,0,9,0	chr7,997162	Cytosol	A > G
chr7:99716258:T:C:ncRNA_intron	chr7	99716258	99716258	T	C	ncRNA_intro	RP11-506M1	.	.	.	A	G	-	-	0.12	22,0,3,0	chr7,997162	Cytosol	A > G
chr7:99810355:T:C:intronic:GATS,	chr7	99810355	99810355	T	C	intronic	GATS,STAG3	.	.	.	A	G	-	+-	0.36363636	7,0,4,0	chr7,998103	Cytosol	A > G
chr7:99810493:T:C:intronic:GATS,	chr7	99810493	99810493	T	C	intronic	GATS,STAG3	.	.	.	A	G	-	+-	0.2	8,0,2,0	chr7,998104	Cytosol	A > G
chr7:99916728:T:C:intronic:SPDYE	chr7	99916728	99916728	T	C	intronic	SPDYE3	.	.	.	A	G	-	+	0.08888889	41,0,4,0	chr7,999167	Cytosol	A > G
chr7:99916761:T:C:intronic:SPDYE	chr7	99916761	99916761	T	C	intronic	SPDYE3	.	.	.	A	G	-	+	0.04878049	39,0,2,0	chr7,999167	Cytosol	A > G
chr7:99916870:T:C:intronic:SPDYE	chr7	99916870	99916870	T	C	intronic	SPDYE3	.	.	.	A	G	-	+	0.09090909	30,0,3,0	chr7,999168	Cytosol	A > G
chr7:99917865:T:C:intronic:SPDYE	chr7	99917865	99917865	T	C	intronic	SPDYE3	.	.	.	A	G	-	+	0.18421053	31,0,7,0	chr7,999178	Cytosol	A > G
chr7:99966651:A:G:intronic:PILRA	chr7	99966651	99966651	A	G	intronic	PILRA	.	.	.	A	G	+	+	1	0,0,7,0	chr7,999666	Cytosol	A > G
chr7:100222432:T:C:intronic:TFR2	chr7	100222432	100222432	T	C	intronic	TFR2	.	.	.	A	G	-	-	0.4	3,0,2,0	chr7,100222	Cytosol	A > G
chr7:100452844:A:G:intronic:SLC1	chr7	100452844	100452844	A	G	intronic	SLC12A9	.	.	.	A	G	+	+	0.66666667	2,0,4,0	chr7,100452	Cytosol	A > G
chr7:100452845:A:G:intronic:SLC1	chr7	100452845	100452845	A	G	intronic	SLC12A9	.	.	.	A	G	+	+	0.6	2,0,3,0	chr7,100452	Cytosol	A > G
chr7:100454369:A:G:intronic:SLC1	chr7	100454369	100454369	A	G	intronic	SLC12A9	.	.	.	A	G	+	+	0.4	3,0,2,0	chr7,100454	Cytosol	A > G
chr7:100454381:A:G:intronic:SLC1	chr7	100454381	100454381	A	G	intronic	SLC12A9	.	.	.	A	G	+	+	0.5	3,0,3,0	chr7,100454	Cytosol	A > G
chr7:100733648:A:G:UTR3:TRIM5	chr7	100733648	100733648	A	G	UTR3	TRIM56	ENST000003	.	.	A	G	+	+	0.04347826	44,0,2,0	chr7,100733	Cytosol	A > G
chr7:100734758:A:G:intronic:TRIM	chr7	100734758	100734758	A	G	intronic	TRIM56	.	.	.	A	G	+	+	0.16326531	41,0,8,0	chr7,100734	Cytosol	A > G
chr7:100734798:A:G:intronic:TRIM	chr7	100734798	100734798	A	G	intronic	TRIM56	.	.	.	A	G	+	+	0.27118644	43,0,16,0	chr7,100734	Cytosol	A > G
chr7:100734812:A:G:intronic:TRIM	chr7	100734812	100734812	A	G	intronic	TRIM56	.	.	.	A	G	+	+	0.18644068	48,0,11,0	chr7,100734	Cytosol	A > G
chr7:100734837:A:G:intronic:TRIM	chr7	100734837	100734837	A	G	intronic	TRIM56	.	.	.	A	G	+	+	0.06451613	29,0,2,0	chr7,100734	Cytosol	A > G
chr7:100734850:A:G:intronic:TRIM	chr7	100734850	100734850	A	G	intronic	TRIM56	.	.	.	A	G	+	+	0.11111111	32,0,4,0	chr7,100734	Cytosol	A > G
chr7:100734852:A:G:intronic:TRIM	chr7	100734852	100734852	A	G	intronic	TRIM56	.	.	.	A	G	+	+	0.05882353	32,0,2,0	chr7,100734	Cytosol	A > G
chr7:100734854:A:G:intronic:TRIM	chr7	100734854	100734854	A	G	intronic	TRIM56	.	.	.	A	G	+	+	0.15789474	32,0,6,0	chr7,100734	Cytosol	A > G
chr7:100734855:A:G:intronic:TRIM	chr7	100734855	100734855	A	G	intronic	TRIM56	.	.	.	A	G	+	+	0.05263158	36,0,2,0	chr7,100734	Cytosol	A > G
chr7:100734874:A:G:intronic:TRIM	chr7	100734874	100734874	A	G	intronic	TRIM56	.	.	.	A	G	+	+	0.36111111	23,0,13,0	chr7,100734	Cytosol	A > G
chr7:100734887:A:G:intronic:TRIM	chr7	100734887	100734887	A	G	intronic	TRIM56	.	.	.	A	G	+	+	0.13953488	37,0,6,0	chr7,100734	Cytosol	A > G
chr7:100734888:A:G:intronic:TRIM	chr7	100734888	100734888	A	G	intronic	TRIM56	.	.	.	A	G	+	+	0.48888889	23,0,22,0	chr7,100734	Cytosol	A > G
chr7:100734893:A:G:intronic:TRIM	chr7	100734893	100734893	A	G	intronic	TRIM56	.	.	.	A	G	+	+	0.04255319	45,0,2,0	chr7,100734	Cytosol	A > G
chr7:100956387:T:C:downstream:	chr7	100956387	100956387	T	C	downstream	RABL5	.	.	.	A	G	-	+	0.33333333	6,0,3,0	chr7,100956	Cytosol	A > G
chr7:100956388:T:C:downstream:	chr7	100956388	100956388	T	C	downstream	RABL5	.	.	.	A	G	-	+	0.44444444	5,0,4,0	chr7,100956	Cytosol	A > G
chr7:100957389:T:C:intronic:RABL	chr7	100957389	100957389	T	C	intronic	RABL5	.	.	.	A	G	-	+-	0.07692308	36,0,3,0	chr7,100957	Cytosol	A > G
chr7:100957850:T:C:UTR3:RABL5	chr7	100957850	100957850	T	C	UTR3	RABL5	ENST000004	.	.	A	G	-	+-	0.20588235	27,0,7,0	chr7,100957	Cytosol	A > G
chr7:100957851:T:C:UTR3:RABL5	chr7	100957851	100957851	T	C	UTR3	RABL5	ENST000004	.	.	A	G	-	+-	0.09090909	30,0,3,0	chr7,100957	Cytosol	A > G
chr7:102088891:A:G:UTR3:ORAI2	chr7	102088891	102088891	A	G	UTR3	ORAI2	ENST000003	.	.	A	G	+	+	0.04347826	44,0,2,0	chr7,102088	Cytosol	A > G
chr7:102088969:A:G:UTR3:ORAI2	chr7	102088969	102088969	A	G	UTR3	ORAI2	ENST000003	.	.	A	G	+	+	0.03448276	84,0,3,0	chr7,102088	Cytosol	A > G

chr7:102090205:A:G:UTR3:ORAI2	chr7	102090205	102090205	A	G	UTR3	ORAI2	ENST000003	.	.	A	G	+	+	0.16666667	10,0,2,0	chr7,102090	Cytosol	A > G
chr7:102090307:A:G:UTR3:ORAI2	chr7	102090307	102090307	A	G	UTR3	ORAI2	ENST000003	.	.	A	G	+	+	0.09677419	28,0,3,0	chr7,102090	Cytosol	A > G
chr7:102091486:A:G:UTR3:ORAI2	chr7	102091486	102091486	A	G	UTR3	ORAI2	ENST000003	.	.	A	G	+	+	0.05555556	34,0,2,0	chr7,102091	Cytosol	A > G
chr7:102091527:A:G:UTR3:ORAI2	chr7	102091527	102091527	A	G	UTR3	ORAI2	ENST000003	.	.	A	G	+	+	0.13636364	38,0,6,0	chr7,102091	Cytosol	A > G
chr7:102097455:T:C:UTR3:ALKBH4	chr7	102097455	102097455	T	C	UTR3	ALKBH4	ENST000002	.	.	A	G	-	-	0.01492537	132,0,2,0	chr7,102097	Cytosol	A > G
chr7:102097542:T:C:UTR3:ALKBH4	chr7	102097542	102097542	T	C	UTR3	ALKBH4	ENST000002	.	.	A	G	-	-	0.01785714	110,0,2,0	chr7,102097	Cytosol	A > G
chr7:102277600:T:C:UTR3:POLR2J	chr7	102277600	102277600	T	C	UTR3	POLR2J2,UP	ENST000005	.	.	A	G	-	-	0.92307692	1,0,12,0	chr7,102277	Cytosol	A > G
chr7:102277664:T:C:UTR3:POLR2J	chr7	102277664	102277664	T	C	UTR3	POLR2J2,UP	ENST000005	.	.	A	G	-	-	0.08196721	56,0,5,0	chr7,102277	Cytosol	A > G
chr7:102277678:T:C:UTR3:POLR2J	chr7	102277678	102277678	T	C	UTR3	POLR2J2,UP	ENST000005	.	.	A	G	-	-	0.63265306	18,0,31,0	chr7,102277	Cytosol	A > G
chr7:102277714:T:C:UTR3:POLR2J	chr7	102277714	102277714	T	C	UTR3	POLR2J2,UP	ENST000005	.	.	A	G	-	-	0.17391304	19,0,4,0	chr7,102277	Cytosol	A > G
chr7:102277722:T:C:UTR3:POLR2J	chr7	102277722	102277722	T	C	UTR3	POLR2J2,UP	ENST000005	.	.	A	G	-	-	0.11111111	16,0,2,0	chr7,102277	Cytosol	A > G
chr7:104308742:C:T:intronic:LHFP	chr7	104308742	104308742	C	T	intronic	LHFPL3	.	.	.	C	T	+	+	1	0,0,0,25	chr7,104308	Cytosol	C > T
chr7:105139347:T:C:intronic:PUS7	chr7	105139347	105139347	T	C	intronic	PUS7	.	.	.	A	G	-	-	0.22222222	7,0,2,0	chr7,105139	Cytosol	A > G
chr7:105142152:T:C:intronic:PUS7	chr7	105142152	105142152	T	C	intronic	PUS7	.	.	.	A	G	-	-	0.5	6,0,6,0	chr7,105142	Cytosol	A > G
chr7:105142213:T:C:intronic:PUS7	chr7	105142213	105142213	T	C	intronic	PUS7	.	.	.	A	G	-	-	0.28571429	10,0,4,0	chr7,105142	Cytosol	A > G
chr7:105211095:A:G:intronic:EFCA	chr7	105211095	105211095	A	G	intronic	EFCAB10	.	.	.	A	G	+	-	0.23076923	10,0,3,0	chr7,105211	Cytosol	A > G
chr7:107257245:A:G:intronic:BCAI	chr7	107257245	107257245	A	G	intronic	BCAP29	.	.	.	A	G	+	+	0.55555556	4,0,5,0	chr7,107257	Cytosol	A > G
chr7:107257247:A:G:intronic:BCAI	chr7	107257247	107257247	A	G	intronic	BCAP29	.	.	.	A	G	+	+	0.3	7,0,3,0	chr7,107257	Cytosol	A > G
chr7:107257253:A:G:intronic:BCAI	chr7	107257253	107257253	A	G	intronic	BCAP29	.	.	.	A	G	+	+	0.57142857	3,0,4,0	chr7,107257	Cytosol	A > G
chr7:107257323:A:G:intronic:BCAI	chr7	107257323	107257323	A	G	intronic	BCAP29	.	.	.	A	G	+	+	0.18181818	9,0,2,0	chr7,107257	Cytosol	A > G
chr7:108203870:T:C:UTR3:THAP5	chr7	108203870	108203870	T	C	UTR3	THAP5	ENST000004	.	.	A	G	-	-	0.01449275	136,0,2,0	chr7,108203	Cytosol	A > G
chr7:111985614:A:G:ncRNA_intro	chr7	111985614	111985614	A	G	ncRNA_intro	AC004112.4	.	.	.	A	G	+	-	0.33333333	4,0,2,0	chr7,111985	Cytosol	A > G
chr7:111985696:A:G:ncRNA_intro	chr7	111985696	111985696	A	G	ncRNA_intro	AC004112.4	.	.	.	A	G	+	-	0.33333333	6,0,3,0	chr7,111985	Cytosol	A > G
chr7:127945575:T:C:UTR3:RBM28	chr7	127945575	127945575	T	C	UTR3	RBM28	ENST000002	.	.	A	G	-	-	0.23529412	26,0,8,0	chr7,127945	Cytosol	A > G
chr7:127945651:T:C:UTR3:RBM28	chr7	127945651	127945651	T	C	UTR3	RBM28	ENST000002	.	.	A	G	-	-	0.375	10,0,6,0	chr7,127945	Cytosol	A > G
chr7:127947905:T:C:UTR3:RBM28	chr7	127947905	127947905	T	C	UTR3	RBM28	ENST000002	.	.	A	G	-	-	0.1627907	36,0,7,0	chr7,127947	Cytosol	A > G
chr7:127947979:T:C:UTR3:RBM28	chr7	127947979	127947979	T	C	UTR3	RBM28	ENST000002	.	.	A	G	-	-	0.58064516	13,0,18,0	chr7,127947	Cytosol	A > G
chr7:128097710:A:G:ncRNA_intro	chr7	128097710	128097710	A	G	ncRNA_intro	RP11-155G1	.	.	.	A	G	+	+	0.01197605	165,0,2,0	chr7,128097	Cytosol	A > G
chr7:128097711:A:G:ncRNA_intro	chr7	128097711	128097711	A	G	ncRNA_intro	RP11-155G1	.	.	.	A	G	+	+	0.01234568	160,0,2,0	chr7,128097	Cytosol	A > G
chr7:128097746:A:G:ncRNA_intro	chr7	128097746	128097746	A	G	ncRNA_intro	RP11-155G1	.	.	.	A	G	+	+	0.03030303	64,0,2,0	chr7,128097	Cytosol	A > G
chr7:128097756:A:G:ncRNA_intro	chr7	128097756	128097756	A	G	ncRNA_intro	RP11-155G1	.	.	.	A	G	+	+	0.025	78,0,2,0	chr7,128097	Cytosol	A > G
chr7:128097795:A:G:ncRNA_intro	chr7	128097795	128097795	A	G	ncRNA_intro	RP11-155G1	.	.	.	A	G	+	+	0.05769231	49,0,3,0	chr7,128097	Cytosol	A > G
chr7:128097813:A:G:ncRNA_intro	chr7	128097813	128097813	A	G	ncRNA_intro	RP11-155G1	.	.	.	A	G	+	+	0.02985075	65,0,2,0	chr7,128097	Cytosol	A > G
chr7:128097815:A:G:ncRNA_intro	chr7	128097815	128097815	A	G	ncRNA_intro	RP11-155G1	.	.	.	A	G	+	+	0.08695652	63,0,6,0	chr7,128097	Cytosol	A > G
chr7:128097819:A:G:ncRNA_intro	chr7	128097819	128097819	A	G	ncRNA_intro	RP11-155G1	.	.	.	A	G	+	+	0.03278689	59,0,2,0	chr7,128097	Cytosol	A > G
chr7:128097837:A:G:ncRNA_intro	chr7	128097837	128097837	A	G	ncRNA_intro	RP11-155G1	.	.	.	A	G	+	+	0.02083333	94,0,2,0	chr7,128097	Cytosol	A > G
chr7:128097845:A:G:ncRNA_intro	chr7	128097845	128097845	A	G	ncRNA_intro	RP11-155G1	.	.	.	A	G	+	+	0.03100775	125,0,4,0	chr7,128097	Cytosol	A > G
chr7:128097846:A:G:ncRNA_intro	chr7	128097846	128097846	A	G	ncRNA_intro	RP11-155G1	.	.	.	A	G	+	+	0.1023622	114,0,13,0	chr7,128097	Cytosol	A > G
chr7:128097851:A:G:ncRNA_intro	chr7	128097851	128097851	A	G	ncRNA_intro	RP11-155G1	.	.	.	A	G	+	+	0.04081633	141,0,6,0	chr7,128097	Cytosol	A > G
chr7:128142691:A:G:exonic:METT	chr7	128142691	128142691	A	G	exonic	METTL2B	.	synonymous	METTL2B:EN	A	G	+	+	0.12068966	51,0,7,0	chr7,128142	Cytosol	A > G
chr7:128142743:A:G:exonic:METT	chr7	128142743	128142743	A	G	exonic	METTL2B	.	nonsynonym	METTL2B:EN	A	G	+	+	0.26666667	99,0,36,0	chr7,128142	Cytosol	A > G
chr7:128142777:A:G:UTR3:METTL	chr7	128142777	128142777	A	G	UTR3	METTL2B	ENST000002	.	.	A	G	+	+	0.11764706	30,0,4,0	chr7,128142	Cytosol	A > G
chr7:128142798:A:G:UTR3:METTL	chr7	128142798	128142798	A	G	UTR3	METTL2B	ENST000002	.	.	A	G	+	+	0.06382979	43,0,3,1	chr7,128142	Cytosol	A > G
chr7:128142806:A:G:UTR3:METTL	chr7	128142806	128142806	A	G	UTR3	METTL2B	ENST000002	.	.	A	G	+	+	0.06521739	43,0,3,0	chr7,128142	Cytosol	A > G
chr7:128142833:A:G:UTR3:METTL	chr7	128142833	128142833	A	G	UTR3	METTL2B	ENST000002	.	.	A	G	+	+	0.18181818	45,0,10,0	chr7,128142	Cytosol	A > G
chr7:128142841:A:G:UTR3:METTL	chr7	128142841	128142841	A	G	UTR3	METTL2B	ENST000002	.	.	A	G	+	+	0.20289855	55,0,14,0	chr7,128142	Cytosol	A > G
chr7:128142939:A:G:UTR3:METTL	chr7	128142939	128142939	A	G	UTR3	METTL2B	ENST000002	.	.	A	G	+	+	0.125	14,0,2,0	chr7,128142	Cytosol	A > G
chr7:128143068:A:G:UTR3:METTL	chr7	128143068	128143068	A	G	UTR3	METTL2B	ENST000004	.	.	A	G	+	+	0.06493506	72,0,5,0	chr7,128143	Cytosol	A > G
chr7:128143162:A:G:UTR3:METTL	chr7	128143162	128143162	A	G	UTR3	METTL2B	ENST000004	.	.	A	G	+	+	0.07526882	86,0,7,0	chr7,128143	Cytosol	A > G
chr7:128143219:A:G:UTR3:METTL	chr7	128143219	128143219	A	G	UTR3	METTL2B	ENST000004	.	.	A	G	+	+	0.02678571	109,0,3,0	chr7,128143	Cytosol	A > G
chr7:128143236:A:G:UTR3:METTL	chr7	128143236	128143236	A	G	UTR3	METTL2B	ENST000004	.	.	A	G	+	+	0.01869159	105,0,2,0	chr7,128143	Cytosol	A > G
chr7:128143512:A:G:UTR3:METTL	chr7	128143512	128143512	A	G	UTR3	METTL2B	ENST000004	.	.	A	G	+	+	0.8	2,0,8,0	chr7,128143	Cytosol	A > G
chr7:128143622:A:G:UTR3:METTL	chr7	128143622	128143622	A	G	UTR3	METTL2B	ENST000004	.	.	A	G	+	+	0.57894737	24,0,33,0	chr7,128143	Cytosol	A > G
chr7:128143797:A:G:UTR3:METTL	chr7	128143797	128143797	A	G	UTR3	METTL2B	ENST000004	.	.	A	G	+	+	0.04347826	44,0,2,0	chr7,128143	Cytosol	A > G
chr7:128143842:A:G:UTR3:METTL	chr7	128143842	128143842	A	G	UTR3	METTL2B	ENST000004	.	.	A	G	+	+	0.16666667	20,0,4,0	chr7,128143	Cytosol	A > G
chr7:128143870:A:G:UTR3:METTL	chr7	128143870	128143870	A	G	UTR3	METTL2B	ENST000004	.	.	A	G	+	+	0.55555556	4,0,5,0	chr7,128143	Cytosol	A > G
chr7:128143932:A:G:UTR3:METTL	chr7	128143932	128143932	A	G	UTR3	METTL2B	ENST000004	.	.	A	G	+	+	0.1969697	53,0,13,0	chr7,128143	Cytosol	A > G
chr7:128143937:A:G:UTR3:METTL	chr7	128143937	128143937	A	G	UTR3	METTL2B	ENST000004	.	.	A	G	+	+	0.03797468	76,0,3,0	chr7,128143	Cytosol	A > G
chr7:128143945:A:G:UTR3:METTL	chr7	128143945	128143945	A	G	UTR3	METTL2B	ENST000004	.	.	A	G	+	+	0.10344828	78,0,9,0	chr7,128143	Cytosol	A > G
chr7:128144035:A:G:UTR3:METTL	chr7	128144035	128144035	A	G	UTR3	METTL2B	ENST000004	.	.	A	G	+	+	0.26923077	19,0,7,0	chr7,128144	Cytosol	A > G
chr7:128144460:A:G:UTR3:METTL	chr7	128144460	128144460	A	G	UTR3	METTL2B	ENST000004	.	.	A	G	+	+	0.70588235	5,0,12,0	chr7,128144	Cytosol	A > G
chr7:128144463:A:G:UTR3:METTL	chr7	128144463	128144463	A	G	UTR3	METTL2B	ENST000004	.	.	A	G	+	+	0.36842105	12,0,7,0	chr7,128144	Cytosol	A > G
chr7:128144468:A:G:UTR3:METTL	chr7	128144468	128144468	A	G	UTR3	METTL2B	ENST000004	.	.	A	G	+	+	0.70588235	5,0,12,0	chr7,128144	Cytosol	A > G
chr7:128144471:A:G:UTR3:METTL	chr7	128144471	128144471	A	G	UTR3	METTL2B	ENST000004	.	.	A	G	+	+	0.47058824	9,0,8,0	chr7,128144	Cytosol	A > G

chr7:128144507:A:G:UTR3:METTL	chr7	128144507	128144507	A	G	UTR3	METTL2B	ENST000004	.	.	A	G	+	+	0.35294118	11,0,6,0	chr7,128144	Cytosol	A > G
chr7:128144532:A:G:UTR3:METTL	chr7	128144532	128144532	A	G	UTR3	METTL2B	ENST000004	.	.	A	G	+	+	0.5	9,0,9,0	chr7,128144	Cytosol	A > G
chr7:128144533:A:G:UTR3:METTL	chr7	128144533	128144533	A	G	UTR3	METTL2B	ENST000004	.	.	A	G	+	+	0.17647059	14,0,3,0	chr7,128144	Cytosol	A > G
chr7:128144536:A:G:UTR3:METTL	chr7	128144536	128144536	A	G	UTR3	METTL2B	ENST000004	.	.	A	G	+	+	0.41176471	9,0,7,1	chr7,128144	Cytosol	A > G
chr7:128144546:A:G:UTR3:METTL	chr7	128144546	128144546	A	G	UTR3	METTL2B	ENST000004	.	.	A	G	+	+	0.31578947	13,0,6,0	chr7,128144	Cytosol	A > G
chr7:128144583:A:G:UTR3:METTL	chr7	128144583	128144583	A	G	UTR3	METTL2B	ENST000004	.	.	A	G	+	+	0.13333333	13,0,2,0	chr7,128144	Cytosol	A > G
chr7:128144589:A:G:UTR3:METTL	chr7	128144589	128144589	A	G	UTR3	METTL2B	ENST000004	.	.	A	G	+	+	0.46666667	8,0,7,0	chr7,128144	Cytosol	A > G
chr7:128144768:A:G:UTR3:METTL	chr7	128144768	128144768	A	G	UTR3	METTL2B	ENST000004	.	.	A	G	+	+	0.40909091	13,0,9,0	chr7,128144	Cytosol	A > G
chr7:128144769:A:G:UTR3:METTL	chr7	128144769	128144769	A	G	UTR3	METTL2B	ENST000004	.	.	A	G	+	+	0.2173913	18,0,5,0	chr7,128144	Cytosol	A > G
chr7:128144814:A:G:UTR3:METTL	chr7	128144814	128144814	A	G	UTR3	METTL2B	ENST000004	.	.	A	G	+	+	0.35714286	9,0,5,0	chr7,128144	Cytosol	A > G
chr7:128144879:A:G:UTR3:METTL	chr7	128144879	128144879	A	G	UTR3	METTL2B	ENST000004	.	.	A	G	+	+	0.05882353	32,0,2,0	chr7,128144	Cytosol	A > G
chr7:128144891:A:G:UTR3:METTL	chr7	128144891	128144891	A	G	UTR3	METTL2B	ENST000004	.	.	A	G	+	+	0.09375	29,0,3,0	chr7,128144	Cytosol	A > G
chr7:128144924:A:G:UTR3:METTL	chr7	128144924	128144924	A	G	UTR3	METTL2B	ENST000004	.	.	A	G	+	+	0.46666667	8,0,7,0	chr7,128144	Cytosol	A > G
chr7:128144976:A:G:UTR3:METTL	chr7	128144976	128144976	A	G	UTR3	METTL2B	ENST000004	.	.	A	G	+	+	0.3	13,0,6,1	chr7,128144	Cytosol	A > G
chr7:128144977:A:G:UTR3:METTL	chr7	128144977	128144977	A	G	UTR3	METTL2B	ENST000004	.	.	A	G	+	+	0.42105263	11,0,8,0	chr7,128144	Cytosol	A > G
chr7:128145008:A:G:UTR3:METTL	chr7	128145008	128145008	A	G	UTR3	METTL2B	ENST000004	.	.	A	G	+	+	0.42857143	4,0,3,0	chr7,128145	Cytosol	A > G
chr7:128145030:A:G:UTR3:METTL	chr7	128145030	128145030	A	G	UTR3	METTL2B	ENST000004	.	.	A	G	+	+	0.75	2,0,6,0	chr7,128145	Cytosol	A > G
chr7:128145031:A:G:UTR3:METTL	chr7	128145031	128145031	A	G	UTR3	METTL2B	ENST000004	.	.	A	G	+	+	0.25	6,0,2,0	chr7,128145	Cytosol	A > G
chr7:128145040:A:G:UTR3:METTL	chr7	128145040	128145040	A	G	UTR3	METTL2B	ENST000004	.	.	A	G	+	+	0.5	4,0,4,0	chr7,128145	Cytosol	A > G
chr7:128145047:A:G:UTR3:METTL	chr7	128145047	128145047	A	G	UTR3	METTL2B	ENST000004	.	.	A	G	+	+	0.22222222	7,0,2,0	chr7,128145	Cytosol	A > G
chr7:128145116:A:G:UTR3:METTL	chr7	128145116	128145116	A	G	UTR3	METTL2B	ENST000004	.	.	A	G	+	+	0.20689655	23,0,6,0	chr7,128145	Cytosol	A > G
chr7:128145201:A:G:UTR3:METTL	chr7	128145201	128145201	A	G	UTR3	METTL2B	ENST000004	.	.	A	G	+	+	0.19512195	33,0,8,0	chr7,128145	Cytosol	A > G
chr7:128145202:A:G:UTR3:METTL	chr7	128145202	128145202	A	G	UTR3	METTL2B	ENST000004	.	.	A	G	+	+	0.20930233	34,0,9,0	chr7,128145	Cytosol	A > G
chr7:128145963:A:G:UTR3:METTL	chr7	128145963	128145963	A	G	UTR3	METTL2B	ENST000004	.	.	A	G	+	+	0.125	14,0,2,0	chr7,128145	Cytosol	A > G
chr7:128145983:A:G:UTR3:METTL	chr7	128145983	128145983	A	G	UTR3	METTL2B	ENST000004	.	.	A	G	+	+	0.47058824	9,0,8,0	chr7,128145	Cytosol	A > G
chr7:128146009:A:G:UTR3:METTL	chr7	128146009	128146009	A	G	UTR3	METTL2B	ENST000004	.	.	A	G	+	+	0.4	3,0,2,0	chr7,128146	Cytosol	A > G
chr7:128146010:A:G:UTR3:METTL	chr7	128146010	128146010	A	G	UTR3	METTL2B	ENST000004	.	.	A	G	+	+	0.4	3,0,2,0	chr7,128146	Cytosol	A > G
chr7:128146023:A:G:UTR3:METTL	chr7	128146023	128146023	A	G	UTR3	METTL2B	ENST000004	.	.	A	G	+	+	0.8	1,0,4,0	chr7,128146	Cytosol	A > G
chr7:128295743:A:G:intergenic:RP	chr7	128295743	128295743	A	G	intergenic	RP11-274B2	dist=26231;d	.	.	A	G	+	+	0.4	3,0,2,0	chr7,128295	Cytosol	A > G
chr7:128301218:A:G:intergenic:RP	chr7	128301218	128301218	A	G	intergenic	RP11-274B2	dist=31706;d	.	.	A	G	+		0.13333333	13,0,2,0	chr7,128301	Cytosol	A > G
chr7:128301224:A:G:intergenic:RP	chr7	128301224	128301224	A	G	intergenic	RP11-274B2	dist=31712;d	.	.	A	G	+		0.125	14,0,2,0	chr7,128301	Cytosol	A > G
chr7:128301232:A:G:intergenic:RP	chr7	128301232	128301232	A	G	intergenic	RP11-274B2	dist=31720;d	.	.	A	G	+		0.14285714	12,0,2,0	chr7,128301	Cytosol	A > G
chr7:128301263:A:G:intergenic:RP	chr7	128301263	128301263	A	G	intergenic	RP11-274B2	dist=31751;d	.	.	A	G	+		0.25	6,0,2,0	chr7,128301	Cytosol	A > G
chr7:128301302:A:G:intergenic:RP	chr7	128301302	128301302	A	G	intergenic	RP11-274B2	dist=31790;d	.	.	A	G	+		0.14285714	12,0,2,0	chr7,128301	Cytosol	A > G
chr7:128301346:A:G:intergenic:RP	chr7	128301346	128301346	A	G	intergenic	RP11-274B2	dist=31834;d	.	.	A	G	+		0.21428571	11,0,3,0	chr7,128301	Cytosol	A > G
chr7:128301425:A:G:intergenic:RP	chr7	128301425	128301425	A	G	intergenic	RP11-274B2	dist=31913;d	.	.	A	G	+		0.23809524	16,0,5,0	chr7,128301	Cytosol	A > G
chr7:129127962:A:G:UTR3:STRIP2	chr7	129127962	129127962	A	G	UTR3	STRIP2	ENST000002	.	.	A	G	+	+	0.18367347	40,0,9,0	chr7,129127	Cytosol	A > G
chr7:129677828:T:C:intronic:ZC3H	chr7	129677828	129677828	T	C	intronic	ZC3HC1	.	.	.	A	G	-	-	0.6	2,0,3,0	chr7,129677	Cytosol	A > G
chr7:129677831:T:C:intronic:ZC3H	chr7	129677831	129677831	T	C	intronic	ZC3HC1	.	.	.	A	G	-	-	0.4	3,0,2,0	chr7,129677	Cytosol	A > G
chr7:129677868:T:C:intronic:ZC3H	chr7	129677868	129677868	T	C	intronic	ZC3HC1	.	.	.	A	G	-	-	0.33333333	4,0,2,0	chr7,129677	Cytosol	A > G
chr7:129838335:T:C:ncRNA_intror	chr7	129838335	129838335	T	C	ncRNA_intro	RP11-775D2	.	.	.	A	G	-	-+	0.71428571	2,0,5,0	chr7,129838	Cytosol	A > G
chr7:129838339:T:C:ncRNA_intror	chr7	129838339	129838339	T	C	ncRNA_intro	RP11-775D2	.	.	.	A	G	-	-+	0.875	1,0,7,0	chr7,129838	Cytosol	A > G
chr7:130629624:T:C:ncRNA_exoni	chr7	130629624	130629624	T	C	ncRNA_exon	MKLN1-AS1	.	.	.	A	G	-	-	0.8	2,0,8,0	chr7,130629	Cytosol	A > G
chr7:130629626:T:C:ncRNA_exoni	chr7	130629626	130629626	T	C	ncRNA_exon	MKLN1-AS1	.	.	.	A	G	-	-	0.25	9,0,3,0	chr7,130629	Cytosol	A > G
chr7:130629992:T:C:ncRNA_exoni	chr7	130629992	130629992	T	C	ncRNA_exon	MKLN1-AS1	.	.	.	A	G	-	-	0.3125	11,0,5,0	chr7,130629	Cytosol	A > G
chr7:130630237:T:C:ncRNA_exoni	chr7	130630237	130630237	T	C	ncRNA_exon	MKLN1-AS1	.	.	.	A	G	-	-	0.64285714	5,0,9,0	chr7,130630	Cytosol	A > G
chr7:139103718:A:G:intronic:C7or	chr7	139103718	139103718	A	G	intronic	C7orf55-LUC	.	.	.	A	G	+	+-	0.02531646	77,0,2,0	chr7,139103	Cytosol	A > G
chr7:139103805:A:G:intronic:C7or	chr7	139103805	139103805	A	G	intronic	C7orf55-LUC	.	.	.	A	G	+	+-	0.025	78,0,2,0	chr7,139103	Cytosol	A > G
chr7:139103813:A:G:intronic:C7or	chr7	139103813	139103813	A	G	intronic	C7orf55-LUC	.	.	.	A	G	+	+-	0.04545455	84,0,4,0	chr7,139103	Cytosol	A > G
chr7:141324100:A:G:intronic:AGK	chr7	141324100	141324100	A	G	intronic	AGK	.	.	.	A	G	+	+	0.66666667	3,0,6,0	chr7,141324	Cytosol	A > G
chr7:152340865:T:C:intergenic:SN	chr7	152340865	152340865	T	C	intergenic	SNORA26,XR	dist=34346;d	.	.	A	G	-		0.33333333	4,0,2,0	chr7,152340	Cytosol	A > G
chr7:152342445:T:C:intergenic:SN	chr7	152342445	152342445	T	C	intergenic	SNORA26,XR	dist=35926;d	.	.	A	G	-	-	0.2	36,0,9,0	chr7,152342	Cytosol	A > G
chr7:152342903:T:C:downstream:	chr7	152342903	152342903	T	C	downstream	XRCC2	.	.	.	A	G	-	-	0.45679012	44,0,37,0	chr7,152342	Cytosol	A > G
chr7:152342940:T:C:downstream:	chr7	152342940	152342940	T	C	downstream	XRCC2	.	.	.	A	G	-	-	0.05660377	50,0,3,0	chr7,152342	Cytosol	A > G
chr7:152342949:T:C:downstream:	chr7	152342949	152342949	T	C	downstream	XRCC2	.	.	.	A	G	-	-	0.05357143	53,0,3,0	chr7,152342	Cytosol	A > G
chr7:152342992:T:C:downstream:	chr7	152342992	152342992	T	C	downstream	XRCC2	.	.	.	A	G	-	-	0.28888889	32,0,13,0	chr7,152342	Cytosol	A > G
chr7:152342999:T:C:downstream:	chr7	152342999	152342999	T	C	downstream	XRCC2	.	.	.	A	G	-	-	0.9375	3,0,45,0	chr7,152342	Cytosol	A > G
chr7:152343658:T:C:UTR3:XRCC2	chr7	152343658	152343658	T	C	UTR3	XRCC2	ENST000003	.	.	A	G	-	-	0.36842105	12,0,7,0	chr7,152343	Cytosol	A > G
chr7:152343686:T:C:UTR3:XRCC2	chr7	152343686	152343686	T	C	UTR3	XRCC2	ENST000003	.	.	A	G	-	-	0.09090909	20,0,2,0	chr7,152343	Cytosol	A > G
chr7:152343700:T:C:UTR3:XRCC2	chr7	152343700	152343700	T	C	UTR3	XRCC2	ENST000003	.	.	A	G	-	-	0.19230769	21,0,5,0	chr7,152343	Cytosol	A > G
chr7:152343781:T:C:UTR3:XRCC2	chr7	152343781	152343781	T	C	UTR3	XRCC2	ENST000003	.	.	A	G	-	-	0.07894737	35,0,3,0	chr7,152343	Cytosol	A > G
chr7:152343829:T:C:UTR3:XRCC2	chr7	152343829	152343829	T	C	UTR3	XRCC2	ENST000003	.	.	A	G	-	-	0.27083333	35,0,13,0	chr7,152343	Cytosol	A > G
chr7:152345248:T:C:UTR3:XRCC2	chr7	152345248	152345248	T	C	UTR3	XRCC2	ENST000003	.	.	A	G	-	-	0.05555556	51,0,3,0	chr7,152345	Cytosol	A > G
chr7:152345309:T:C:UTR3:XRCC2	chr7	152345309	152345309	T	C	UTR3	XRCC2	ENST000003	.	.	A	G	-	-	0.27777778	39,0,15,0	chr7,152345	Cytosol	A > G

chr7:152345392:T:C:UTR3:XRCC2	chr7	152345392	152345392	T	C	UTR3	XRCC2	ENST000003	.	.	A	G	-	-	0.14035088	49,0,8,0	chr7,152345	Cytosol	A > G
chr7:156762727:A:G:UTR3:NOM1	chr7	156762727	156762727	A	G	UTR3	NOM1	ENST000002	.	.	A	G	+	+	0.06122449	46,0,3,0	chr7,156762	Cytosol	A > G
chr7:156762748:A:G:UTR3:NOM1	chr7	156762748	156762748	A	G	UTR3	NOM1	ENST000002	.	.	A	G	+	+	0.34782609	30,0,16,0	chr7,156762	Cytosol	A > G
chr7:156762809:A:G:UTR3:NOM1	chr7	156762809	156762809	A	G	UTR3	NOM1	ENST000002	.	.	A	G	+	+	0.08823529	62,0,6,0	chr7,156762	Cytosol	A > G
chr7:156762816:A:G:UTR3:NOM1	chr7	156762816	156762816	A	G	UTR3	NOM1	ENST000002	.	.	A	G	+	+	0.05405405	70,0,4,0	chr7,156762	Cytosol	A > G
chr7:156762821:A:G:UTR3:NOM1	chr7	156762821	156762821	A	G	UTR3	NOM1	ENST000002	.	.	A	G	+	+	0.05714286	66,0,4,0	chr7,156762	Cytosol	A > G
chr7:156762822:A:G:UTR3:NOM1	chr7	156762822	156762822	A	G	UTR3	NOM1	ENST000002	.	.	A	G	+	+	0.02739726	71,0,2,0	chr7,156762	Cytosol	A > G
chr7:156762823:A:G:UTR3:NOM1	chr7	156762823	156762823	A	G	UTR3	NOM1	ENST000002	.	.	A	G	+	+	0.30555556	50,0,22,0	chr7,156762	Cytosol	A > G
chr7:156762827:A:G:UTR3:NOM1	chr7	156762827	156762827	A	G	UTR3	NOM1	ENST000002	.	.	A	G	+	+	0.35897436	50,0,28,0	chr7,156762	Cytosol	A > G
chr7:156762831:A:G:UTR3:NOM1	chr7	156762831	156762831	A	G	UTR3	NOM1	ENST000002	.	.	A	G	+	+	0.06578947	71,0,5,0	chr7,156762	Cytosol	A > G
chr7:156762883:A:G:UTR3:NOM1	chr7	156762883	156762883	A	G	UTR3	NOM1	ENST000002	.	.	A	G	+	+	0.30769231	36,0,16,0	chr7,156762	Cytosol	A > G
chr7:156762886:A:G:UTR3:NOM1	chr7	156762886	156762886	A	G	UTR3	NOM1	ENST000002	.	.	A	G	+	+	0.04081633	47,0,2,0	chr7,156762	Cytosol	A > G
chr7:156762906:A:G:UTR3:NOM1	chr7	156762906	156762906	A	G	UTR3	NOM1	ENST000002	.	.	A	G	+	+	0.54054054	17,0,20,0	chr7,156762	Cytosol	A > G
chr7:156762924:A:G:UTR3:NOM1	chr7	156762924	156762924	A	G	UTR3	NOM1	ENST000002	.	.	A	G	+	+	0.05128205	37,0,2,0	chr7,156762	Cytosol	A > G
chr7:156763172:A:G:UTR3:NOM1	chr7	156763172	156763172	A	G	UTR3	NOM1	ENST000002	.	.	A	G	+	+	0.26666667	22,0,8,0	chr7,156763	Cytosol	A > G
chr7:156763183:A:G:UTR3:NOM1	chr7	156763183	156763183	A	G	UTR3	NOM1	ENST000002	.	.	A	G	+	+	0.36	16,0,9,0	chr7,156763	Cytosol	A > G
chr7:156763203:A:G:UTR3:NOM1	chr7	156763203	156763203	A	G	UTR3	NOM1	ENST000002	.	.	A	G	+	+	0.52173913	11,0,12,0	chr7,156763	Cytosol	A > G
chr7:156763265:A:G:UTR3:NOM1	chr7	156763265	156763265	A	G	UTR3	NOM1	ENST000002	.	.	A	G	+	+	0.17647059	14,0,3,0	chr7,156763	Cytosol	A > G
chr7:156763266:A:G:UTR3:NOM1	chr7	156763266	156763266	A	G	UTR3	NOM1	ENST000002	.	.	A	G	+	+	0.15	17,0,3,0	chr7,156763	Cytosol	A > G
chr7:156763332:A:G:UTR3:NOM1	chr7	156763332	156763332	A	G	UTR3	NOM1	ENST000002	.	.	A	G	+	+	0.5	12,0,12,0	chr7,156763	Cytosol	A > G
chr7:156763359:A:G:UTR3:NOM1	chr7	156763359	156763359	A	G	UTR3	NOM1	ENST000002	.	.	A	G	+	+	0.2	16,0,4,0	chr7,156763	Cytosol	A > G
chr7:156763360:A:G:UTR3:NOM1	chr7	156763360	156763360	A	G	UTR3	NOM1	ENST000002	.	.	A	G	+	+	0.35	13,0,7,0	chr7,156763	Cytosol	A > G
chr7:156764180:A:G:UTR3:NOM1	chr7	156764180	156764180	A	G	UTR3	NOM1	ENST000002	.	.	A	G	+	+	0.18072289	68,0,15,0	chr7,156764	Cytosol	A > G
chr7:156764267:A:G:UTR3:NOM1	chr7	156764267	156764267	A	G	UTR3	NOM1	ENST000002	.	.	A	G	+	+	0.03603604	107,0,4,0	chr7,156764	Cytosol	A > G
chr7:156764305:A:G:UTR3:NOM1	chr7	156764305	156764305	A	G	UTR3	NOM1	ENST000002	.	.	A	G	+	+	0.02097902	140,0,3,0	chr7,156764	Cytosol	A > G
chr7:156765465:A:G:UTR3:NOM1	chr7	156765465	156765465	A	G	UTR3	NOM1	ENST000002	.	.	A	G	+	+	0.2	20,0,5,0	chr7,156765	Cytosol	A > G
chr7:157025139:A:G:intronic:UBE	chr7	157025139	157025139	A	G	intronic	UBE3C	.	.	.	A	G	+	+	0.16666667	10,0,2,0	chr7,157025	Cytosol	A > G
chr7:157025146:A:G:intronic:UBE	chr7	157025146	157025146	A	G	intronic	UBE3C	.	.	.	A	G	+	+	0.25	9,0,3,0	chr7,157025	Cytosol	A > G
chr7:157025224:A:G:intronic:UBE	chr7	157025224	157025224	A	G	intronic	UBE3C	.	.	.	A	G	+	+	0.25	9,0,3,0	chr7,157025	Cytosol	A > G
chr7:158421265:T:C:intergenic:MI	chr7	158421265	158421265	T	C	intergenic	MIR5707,NC	dist=36877;d	.	.	A	G	-		0.71428571	2,0,5,0	chr7,158421	Cytosol	A > G
chr7:158423875:T:C:downstream:	chr7	158423875	158423875	T	C	downstream	NCAPG2	.	.	.	A	G	-	-	0.2	8,0,2,0	chr7,158423	Cytosol	A > G
chr7:158423882:T:C:downstream:	chr7	158423882	158423882	T	C	downstream	NCAPG2	.	.	.	A	G	-	-	0.69230769	4,0,9,0	chr7,158423	Cytosol	A > G
chr7:158423887:T:C:downstream:	chr7	158423887	158423887	T	C	downstream	NCAPG2	.	.	.	A	G	-	-	0.36363636	7,0,4,0	chr7,158423	Cytosol	A > G
chr7:158423901:T:C:downstream:	chr7	158423901	158423901	T	C	downstream	NCAPG2	.	.	.	A	G	-	-	0.27272727	8,0,3,0	chr7,158423	Cytosol	A > G
chr7:158423967:T:C:downstream:	chr7	158423967	158423967	T	C	downstream	NCAPG2	.	.	.	A	G	-	-	0.5	6,0,6,0	chr7,158423	Cytosol	A > G
chr7:158530839:T:C:intronic:ESYT	chr7	158530839	158530839	T	C	intronic	ESYT2	.	.	.	A	G	-	-	0.375	5,0,3,0	chr7,158530	Cytosol	A > G
chr7:158530842:T:C:intronic:ESYT	chr7	158530842	158530842	T	C	intronic	ESYT2	.	.	.	A	G	-	-	0.5	4,0,4,0	chr7,158530	Cytosol	A > G
chr7:158530855:T:C:intronic:ESYT	chr7	158530855	158530855	T	C	intronic	ESYT2	.	.	.	A	G	-	-	0.25	6,0,2,0	chr7,158530	Cytosol	A > G
chr7:158530891:T:C:intronic:ESYT	chr7	158530891	158530891	T	C	intronic	ESYT2	.	.	.	A	G	-	-	1	0,0,6,0	chr7,158530	Cytosol	A > G
chr7:158530894:T:C:intronic:ESYT	chr7	158530894	158530894	T	C	intronic	ESYT2	.	.	.	A	G	-	-	0.5	3,0,3,0	chr7,158530	Cytosol	A > G
chr7:158530917:T:C:intronic:ESYT	chr7	158530917	158530917	T	C	intronic	ESYT2	.	.	.	A	G	-	-	0.57142857	3,0,4,0	chr7,158530	Cytosol	A > G
chr7:158530940:T:C:intronic:ESYT	chr7	158530940	158530940	T	C	intronic	ESYT2	.	.	.	A	G	-	-	0.22222222	7,0,2,0	chr7,158530	Cytosol	A > G
chr7:158530956:T:C:intronic:ESYT	chr7	158530956	158530956	T	C	intronic	ESYT2	.	.	.	A	G	-	-	0.375	5,0,3,0	chr7,158530	Cytosol	A > G
chr7:158530957:T:C:intronic:ESYT	chr7	158530957	158530957	T	C	intronic	ESYT2	.	.	.	A	G	-	-	0.25	6,0,2,0	chr7,158530	Cytosol	A > G
chr7:158531337:T:C:intronic:ESYT	chr7	158531337	158531337	T	C	intronic	ESYT2	.	.	.	A	G	-	-	0.83333333	2,0,10,0	chr7,158531	Cytosol	A > G
chr7:158531338:T:C:intronic:ESYT	chr7	158531338	158531338	T	C	intronic	ESYT2	.	.	.	A	G	-	-	0.76923077	3,0,10,0	chr7,158531	Cytosol	A > G
chr8:1729259:A:G:UTR3:CLN8	chr8	1729259	1729259	A	G	UTR3	CLN8	ENST000003	.	.	A	G	+	+	0.2	12,0,3,0	chr8,172925	Cytosol	A > G
chr8:1730330:A:G:UTR3:CLN8	chr8	1730330	1730330	A	G	UTR3	CLN8	ENST000003	.	.	A	G	+	+	0.28571429	5,0,2,0	chr8,173032	Cytosol	A > G
chr8:1730558:A:G:UTR3:CLN8	chr8	1730558	1730558	A	G	UTR3	CLN8	ENST000003	.	.	A	G	+	+	0.125	14,0,2,0	chr8,173055	Cytosol	A > G
chr8:6501593:A:G:ncRNA_intronic	chr8	6501593	6501593	A	G	ncRNA_intro	CTD-2541M1	.	.	.	A	G	+	-	0.0952381	19,0,2,0	chr8,650159	Cytosol	A > G
chr8:6501621:A:G:ncRNA_intronic	chr8	6501621	6501621	A	G	ncRNA_intro	CTD-2541M1	.	.	.	A	G	+	-	0.25	9,0,3,0	chr8,650162	Cytosol	A > G
chr8:6501624:A:G:ncRNA_intronic	chr8	6501624	6501624	A	G	ncRNA_intro	CTD-2541M1	.	.	.	A	G	+	-	0.71428571	4,0,10,0	chr8,650162	Cytosol	A > G
chr8:6501692:A:G:ncRNA_intronic	chr8	6501692	6501692	A	G	ncRNA_intro	CTD-2541M1	.	.	.	A	G	+	-	0.58333333	5,0,7,0	chr8,650169	Cytosol	A > G
chr8:6501697:A:G:ncRNA_intronic	chr8	6501697	6501697	A	G	ncRNA_intro	CTD-2541M1	.	.	.	A	G	+	-	0.63636364	4,0,7,0	chr8,650169	Cytosol	A > G
chr8:11700213:T:C:UTR3:CTSB	chr8	11700213	11700213	T	C	UTR3	CTSB	ENST000004	.	.	A	G	-	-	0.22222222	28,0,8,0	chr8,117002	Cytosol	A > G
chr8:11700218:T:C:UTR3:CTSB	chr8	11700218	11700218	T	C	UTR3	CTSB	ENST000004	.	.	A	G	-	-	0.09677419	28,0,3,0	chr8,117002	Cytosol	A > G
chr8:11700231:T:C:UTR3:CTSB	chr8	11700231	11700231	T	C	UTR3	CTSB	ENST000004	.	.	A	G	-	-	0.41666667	13,0,10,1	chr8,117002	Cytosol	A > G
chr8:11700243:T:C:UTR3:CTSB	chr8	11700243	11700243	T	C	UTR3	CTSB	ENST000004	.	.	A	G	-	-	0.10344828	25,0,3,1	chr8,117002	Cytosol	A > G
chr8:11700251:T:C:UTR3:CTSB	chr8	11700251	11700251	T	C	UTR3	CTSB	ENST000004	.	.	A	G	-	-	0.19354839	25,0,6,0	chr8,117002	Cytosol	A > G
chr8:11700261:T:C:UTR3:CTSB	chr8	11700261	11700261	T	C	UTR3	CTSB	ENST000004	.	.	A	G	-	-	0.17647059	28,0,6,0	chr8,117002	Cytosol	A > G
chr8:11700284:T:C:UTR3:CTSB	chr8	11700284	11700284	T	C	UTR3	CTSB	ENST000004	.	.	A	G	-	-	0.12820513	34,0,5,0	chr8,117002	Cytosol	A > G
chr8:11700305:T:C:UTR3:CTSB	chr8	11700305	11700305	T	C	UTR3	CTSB	ENST000004	.	.	A	G	-	-	0.15909091	37,0,7,0	chr8,117003	Cytosol	A > G
chr8:11700321:T:C:UTR3:CTSB	chr8	11700321	11700321	T	C	UTR3	CTSB	ENST000004	.	.	A	G	-	-	0.04	48,0,2,0	chr8,117003	Cytosol	A > G
chr8:11700322:T:C:UTR3:CTSB	chr8	11700322	11700322	T	C	UTR3	CTSB	ENST000004	.	.	A	G	-	-	0.06382979	44,0,3,0	chr8,117003	Cytosol	A > G

chr8:11700327:T:C:UTR3:CTSB	chr8	11700327	11700327	T	C	UTR3	CTSB	ENST000004	.	.	A	G	-	-	0.04255319	45,0,2,0	chr8,117003	Cytosol	A > G
chr8:11700363:T:C:UTR3:CTSB	chr8	11700363	11700363	T	C	UTR3	CTSB	ENST000004	.	.	A	G	-	-	0.2	36,0,9,0	chr8,117003	Cytosol	A > G
chr8:11700373:T:C:UTR3:CTSB	chr8	11700373	11700373	T	C	UTR3	CTSB	ENST000004	.	.	A	G	-	-	0.1	36,0,4,0	chr8,117003	Cytosol	A > G
chr8:11700388:T:C:UTR3:CTSB	chr8	11700388	11700388	T	C	UTR3	CTSB	ENST000004	.	.	A	G	-	-	0.0625	30,0,2,0	chr8,117003	Cytosol	A > G
chr8:11700396:T:C:UTR3:CTSB	chr8	11700396	11700396	T	C	UTR3	CTSB	ENST000004	.	.	A	G	-	-	0.16	21,0,4,0	chr8,117003	Cytosol	A > G
chr8:11700418:T:C:UTR3:CTSB	chr8	11700418	11700418	T	C	UTR3	CTSB	ENST000004	.	.	A	G	-	-	0.22727273	17,0,5,0	chr8,117004	Cytosol	A > G
chr8:11700526:T:C:UTR3:CTSB	chr8	11700526	11700526	T	C	UTR3	CTSB	ENST000004	.	.	A	G	-	-	0.16129032	26,0,5,0	chr8,117005	Cytosol	A > G
chr8:11700555:T:C:UTR3:CTSB	chr8	11700555	11700555	T	C	UTR3	CTSB	ENST000004	.	.	A	G	-	-	0.1372549	44,0,7,0	chr8,117005	Cytosol	A > G
chr8:11700563:T:C:UTR3:CTSB	chr8	11700563	11700563	T	C	UTR3	CTSB	ENST000004	.	.	A	G	-	-	0.04444444	43,0,2,0	chr8,117005	Cytosol	A > G
chr8:11700569:T:C:UTR3:CTSB	chr8	11700569	11700569	T	C	UTR3	CTSB	ENST000004	.	.	A	G	-	-	0.06122449	46,0,3,0	chr8,117005	Cytosol	A > G
chr8:11700573:T:C:UTR3:CTSB	chr8	11700573	11700573	T	C	UTR3	CTSB	ENST000004	.	.	A	G	-	-	0.0754717	49,0,4,0	chr8,117005	Cytosol	A > G
chr8:11700586:T:C:UTR3:CTSB	chr8	11700586	11700586	T	C	UTR3	CTSB	ENST000004	.	.	A	G	-	-	0.28301887	38,0,15,0	chr8,117005	Cytosol	A > G
chr8:11700590:T:C:UTR3:CTSB	chr8	11700590	11700590	T	C	UTR3	CTSB	ENST000004	.	.	A	G	-	-	0.05660377	50,0,3,0	chr8,117005	Cytosol	A > G
chr8:11700596:T:C:UTR3:CTSB	chr8	11700596	11700596	T	C	UTR3	CTSB	ENST000004	.	.	A	G	-	-	0.21153846	41,0,11,0	chr8,117005	Cytosol	A > G
chr8:11700622:T:C:UTR3:CTSB	chr8	11700622	11700622	T	C	UTR3	CTSB	ENST000004	.	.	A	G	-	-	0.09090909	20,0,2,0	chr8,117006	Cytosol	A > G
chr8:11700631:T:C:UTR3:CTSB	chr8	11700631	11700631	T	C	UTR3	CTSB	ENST000004	.	.	A	G	-	-	0.07692308	24,0,2,0	chr8,117006	Cytosol	A > G
chr8:11700651:T:C:UTR3:CTSB	chr8	11700651	11700651	T	C	UTR3	CTSB	ENST000004	.	.	A	G	-	-	0.05	38,0,2,0	chr8,117006	Cytosol	A > G
chr8:11700652:T:C:UTR3:CTSB	chr8	11700652	11700652	T	C	UTR3	CTSB	ENST000004	.	.	A	G	-	-	0.04347826	44,0,2,0	chr8,117006	Cytosol	A > G
chr8:11700676:T:C:UTR3:CTSB	chr8	11700676	11700676	T	C	UTR3	CTSB	ENST000004	.	.	A	G	-	-	0.64814815	19,0,35,0	chr8,117006	Cytosol	A > G
chr8:11700677:T:C:UTR3:CTSB	chr8	11700677	11700677	T	C	UTR3	CTSB	ENST000004	.	.	A	G	-	-	0.22641509	41,0,12,0	chr8,117006	Cytosol	A > G
chr8:11700685:T:C:UTR3:CTSB	chr8	11700685	11700685	T	C	UTR3	CTSB	ENST000004	.	.	A	G	-	-	0.62264151	20,0,33,0	chr8,117006	Cytosol	A > G
chr8:11700707:T:C:UTR3:CTSB	chr8	11700707	11700707	T	C	UTR3	CTSB	ENST000004	.	.	A	G	-	-	0.08163265	45,0,4,0	chr8,117007	Cytosol	A > G
chr8:11700729:T:C:UTR3:CTSB	chr8	11700729	11700729	T	C	UTR3	CTSB	ENST000004	.	.	A	G	-	-	0.32258065	21,0,10,0	chr8,117007	Cytosol	A > G
chr8:11700737:T:C:UTR3:CTSB	chr8	11700737	11700737	T	C	UTR3	CTSB	ENST000004	.	.	A	G	-	-	0.5625	14,0,18,0	chr8,117007	Cytosol	A > G
chr8:11701283:T:C:UTR3:CTSB	chr8	11701283	11701283	T	C	UTR3	CTSB	ENST000004	.	.	A	G	-	-	0.33333333	18,0,9,0	chr8,117012	Cytosol	A > G
chr8:11701296:T:C:UTR3:CTSB	chr8	11701296	11701296	T	C	UTR3	CTSB	ENST000004	.	.	A	G	-	-	0.32	17,0,8,0	chr8,117012	Cytosol	A > G
chr8:11701305:T:C:UTR3:CTSB	chr8	11701305	11701305	T	C	UTR3	CTSB	ENST000004	.	.	A	G	-	-	0.5	9,0,9,0	chr8,117013	Cytosol	A > G
chr8:11701323:T:C:UTR3:CTSB	chr8	11701323	11701323	T	C	UTR3	CTSB	ENST000004	.	.	A	G	-	-	0.53333333	7,0,8,0	chr8,117013	Cytosol	A > G
chr8:11701343:T:C:UTR3:CTSB	chr8	11701343	11701343	T	C	UTR3	CTSB	ENST000004	.	.	A	G	-	-	0.55555556	8,0,10,0	chr8,117013	Cytosol	A > G
chr8:11701357:T:C:UTR3:CTSB	chr8	11701357	11701357	T	C	UTR3	CTSB	ENST000004	.	.	A	G	-	-	0.08	23,0,2,0	chr8,117013	Cytosol	A > G
chr8:11701392:T:C:UTR3:CTSB	chr8	11701392	11701392	T	C	UTR3	CTSB	ENST000004	.	.	A	G	-	-	0.05405405	35,0,2,0	chr8,117013	Cytosol	A > G
chr8:11701429:T:C:UTR3:CTSB	chr8	11701429	11701429	T	C	UTR3	CTSB	ENST000004	.	.	A	G	-	-	0.23943662	54,0,17,0	chr8,117014	Cytosol	A > G
chr8:11701458:T:C:UTR3:CTSB	chr8	11701458	11701458	T	C	UTR3	CTSB	ENST000004	.	.	A	G	-	-	0.3372093	57,0,29,0	chr8,117014	Cytosol	A > G
chr8:11701479:T:C:UTR3:CTSB	chr8	11701479	11701479	T	C	UTR3	CTSB	ENST000004	.	.	A	G	-	-	0.08235294	78,0,7,0	chr8,117014	Cytosol	A > G
chr8:11701485:T:C:UTR3:CTSB	chr8	11701485	11701485	T	C	UTR3	CTSB	ENST000004	.	.	A	G	-	-	0.06976744	80,0,6,0	chr8,117014	Cytosol	A > G
chr8:11701490:T:C:UTR3:CTSB	chr8	11701490	11701490	T	C	UTR3	CTSB	ENST000004	.	.	A	G	-	-	0.51190476	41,0,43,0	chr8,117014	Cytosol	A > G
chr8:11701504:T:C:UTR3:CTSB	chr8	11701504	11701504	T	C	UTR3	CTSB	ENST000004	.	.	A	G	-	-	0.1038961	69,0,8,0	chr8,117015	Cytosol	A > G
chr8:11701509:T:C:UTR3:CTSB	chr8	11701509	11701509	T	C	UTR3	CTSB	ENST000004	.	.	A	G	-	-	0.12658228	69,0,10,0	chr8,117015	Cytosol	A > G
chr8:12880322:A:G:UTR3:KIAA145	chr8	12880322	12880322	A	G	UTR3	KIAA1456	ENST000005	.	.	A	G	+	+	0.15789474	16,0,3,0	chr8,128803	Cytosol	A > G
chr8:12881751:A:G:UTR3:KIAA145	chr8	12881751	12881751	A	G	UTR3	KIAA1456	ENST000005	.	.	A	G	+	+	0.3	7,0,3,0	chr8,128817	Cytosol	A > G
chr8:12881759:A:G:UTR3:KIAA145	chr8	12881759	12881759	A	G	UTR3	KIAA1456	ENST000005	.	.	A	G	+	+	0.71428571	2,0,5,0	chr8,128817	Cytosol	A > G
chr8:22111289:A:G:UTR3:POLR3D	chr8	22111289	22111289	A	G	UTR3	POLR3D	ENST000003	.	.	A	G	+	+	0.05454545	52,0,3,0	chr8,221112	Cytosol	A > G
chr8:22111354:A:G:UTR3:POLR3D	chr8	22111354	22111354	A	G	UTR3	POLR3D	ENST000003	.	.	A	G	+	+	0.05555556	34,0,2,0	chr8,221113	Cytosol	A > G
chr8:22111369:A:G:UTR3:POLR3D	chr8	22111369	22111369	A	G	UTR3	POLR3D	ENST000003	.	.	A	G	+	+	0.11428571	31,0,4,0	chr8,221113	Cytosol	A > G
chr8:22111410:A:G:UTR3:POLR3D	chr8	22111410	22111410	A	G	UTR3	POLR3D	ENST000003	.	.	A	G	+	+	0.13793103	25,0,4,0	chr8,221114	Cytosol	A > G
chr8:22113955:A:G:ncRNA_introni	chr8	22113955	22113955	A	G	ncRNA_intro	CTD-2530N2	.	.	.	A	G	+	-	0.25	6,0,2,0	chr8,221139	Cytosol	A > G
chr8:22113969:A:G:ncRNA_introni	chr8	22113969	22113969	A	G	ncRNA_intro	CTD-2530N2	.	.	.	A	G	+	-	0.22222222	7,0,2,0	chr8,221139	Cytosol	A > G
chr8:22113997:A:G:ncRNA_introni	chr8	22113997	22113997	A	G	ncRNA_intro	CTD-2530N2	.	.	.	A	G	+	-	0.375	5,0,3,0	chr8,221139	Cytosol	A > G
chr8:22114838:A:G:ncRNA_introni	chr8	22114838	22114838	A	G	ncRNA_intro	CTD-2530N2	.	.	.	A	G	+	-	0.42857143	4,0,3,0	chr8,221148	Cytosol	A > G
chr8:23048940:T:C:UTR3:TNFRSF1	chr8	23048940	23048940	T	C	UTR3	TNFRSF10A	ENST000002	.	.	A	G	-	-	0.025	78,0,2,0	chr8,230489	Cytosol	A > G
chr8:23149780:A:G:intronic:R3HC	chr8	23149780	23149780	A	G	intronic	R3HCC1	.	.	.	A	G	+	+	0.33333333	4,0,2,0	chr8,231497	Cytosol	A > G
chr8:27621940:T:C:UTR3:CCDC25	chr8	27621940	27621940	T	C	UTR3	CCDC25	ENST000005	.	.	A	G	-	-	0.6	2,0,3,0	chr8,276219	Cytosol	A > G
chr8:30044855:A:G:intergenic:DC	chr8	30044855	30044855	A	G	intergenic	DCTN6,RP11	dist=3699;dis	.	.	A	G	+	-	0.8	1,0,4,0	chr8,300448	Cytosol	A > G
chr8:30044856:A:G:intergenic:DC	chr8	30044856	30044856	A	G	intergenic	DCTN6,RP11	dist=3700;dis	.	.	A	G	+	-	0.8	1,0,4,0	chr8,300448	Cytosol	A > G
chr8:30535906:T:C:UTR3:GSR	chr8	30535906	30535906	T	C	UTR3	GSR	ENST000002	.	.	A	G	-	-	0.36956522	58,0,34,0	chr8,305359	Cytosol	A > G
chr8:30535907:T:C:UTR3:GSR	chr8	30535907	30535907	T	C	UTR3	GSR	ENST000002	.	.	A	G	-	-	0.17142857	87,0,18,0	chr8,305359	Cytosol	A > G
chr8:30535953:T:C:UTR3:GSR	chr8	30535953	30535953	T	C	UTR3	GSR	ENST000002	.	.	A	G	-	-	0.08759124	250,0,24,0	chr8,305359	Cytosol	A > G
chr8:30535975:T:C:UTR3:GSR	chr8	30535975	30535975	T	C	UTR3	GSR	ENST000002	.	.	A	G	-	-	0.0529595	304,0,17,0	chr8,305359	Cytosol	A > G
chr8:30535980:T:C:UTR3:GSR	chr8	30535980	30535980	T	C	UTR3	GSR	ENST000002	.	.	A	G	-	-	0.52014652	131,0,142,0	chr8,305359	Cytosol	A > G
chr8:30536015:T:C:UTR3:GSR	chr8	30536015	30536015	T	C	UTR3	GSR	ENST000002	.	.	A	G	-	-	0.20727273	218,0,57,0	chr8,305360	Cytosol	A > G
chr8:30536016:T:C:UTR3:GSR	chr8	30536016	30536016	T	C	UTR3	GSR	ENST000002	.	.	A	G	-	-	0.3024911	196,0,85,0	chr8,305360	Cytosol	A > G
chr8:30536125:T:C:UTR3:GSR	chr8	30536125	30536125	T	C	UTR3	GSR	ENST000002	.	.	A	G	-	-	0.03931204	391,0,16,0	chr8,305361	Cytosol	A > G
chr8:30536143:T:C:UTR3:GSR	chr8	30536143	30536143	T	C	UTR3	GSR	ENST000002	.	.	A	G	-	-	0.01322751	373,0,5,0	chr8,305361	Cytosol	A > G
chr8:30536146:T:C:UTR3:GSR	chr8	30536146	30536146	T	C	UTR3	GSR	ENST000002	.	.	A	G	-	-	0.03133159	371,0,12,0	chr8,305361	Cytosol	A > G

chr8:30536151:T:C:UTR3:GSR	chr8	30536151	30536151	T	C	UTR3	GSR	ENST000002	.	.	A	G	-	-	0.26273458	275,0,98,0	chr8,305361	Cytosol	A > G
chr8:30536152:T:C:UTR3:GSR	chr8	30536152	30536152	T	C	UTR3	GSR	ENST000002	.	.	A	G	-	-	0.0952381	342,0,36,0	chr8,305361	Cytosol	A > G
chr8:30536410:T:C:UTR3:GSR	chr8	30536410	30536410	T	C	UTR3	GSR	ENST000002	.	.	A	G	-	-	0.06407323	409,0,28,0	chr8,305364	Cytosol	A > G
chr8:30536544:T:C:UTR3:GSR	chr8	30536544	30536544	T	C	UTR3	GSR	ENST000002	.	.	A	G	-	-	0.18518519	176,0,40,0	chr8,305365	Cytosol	A > G
chr8:30536554:T:C:UTR3:GSR	chr8	30536554	30536554	T	C	UTR3	GSR	ENST000002	.	.	A	G	-	-	0.10245902	219,0,25,0	chr8,305365	Cytosol	A > G
chr8:30536566:T:C:UTR3:GSR	chr8	30536566	30536566	T	C	UTR3	GSR	ENST000002	.	.	A	G	-	-	0.04511278	254,0,12,0	chr8,305365	Cytosol	A > G
chr8:30536583:T:C:UTR3:GSR	chr8	30536583	30536583	T	C	UTR3	GSR	ENST000002	.	.	A	G	-	-	0.21428571	198,0,54,0	chr8,305365	Cytosol	A > G
chr8:30536584:T:C:UTR3:GSR	chr8	30536584	30536584	T	C	UTR3	GSR	ENST000002	.	.	A	G	-	-	0.0311284	249,0,8,0	chr8,305365	Cytosol	A > G
chr8:30536603:T:C:UTR3:GSR	chr8	30536603	30536603	T	C	UTR3	GSR	ENST000002	.	.	A	G	-	-	0.51598174	106,0,113,0	chr8,305366	Cytosol	A > G
chr8:30536606:T:C:UTR3:GSR	chr8	30536606	30536606	T	C	UTR3	GSR	ENST000002	.	.	A	G	-	-	0.20197044	162,0,41,0	chr8,305366	Cytosol	A > G
chr8:30536666:C:A:UTR3:GSR	chr8	30536666	30536666	C	A	UTR3	GSR	ENST000002	.	.	G	T	-	-	0.04938272	0,0,77,4	chr8,305366	Cytosol	G > T
chr8:38104805:A:G:UTR5:DDHD2	chr8	38104805	38104805	A	G	UTR5	DDHD2	ENST000005	.	.	A	G	+	+	0.85714286	1,0,6,0	chr8,381048	Cytosol	A > G
chr8:38104814:A:G:UTR5:DDHD2	chr8	38104814	38104814	A	G	UTR5	DDHD2	ENST000005	.	.	A	G	+	+	0.28571429	5,0,2,0	chr8,381048	Cytosol	A > G
chr8:38111978:A:G:intronic:DDHD	chr8	38111978	38111978	A	G	intronic	DDHD2	.	.	.	A	G	+	+	0.4	3,0,2,0	chr8,381119	Cytosol	A > G
chr8:38111998:A:G:intronic:DDHD	chr8	38111998	38111998	A	G	intronic	DDHD2	.	.	.	A	G	+	+	0.5	3,0,3,0	chr8,381119	Cytosol	A > G
chr8:38121763:T:C:UTR3:PPAPDC1	chr8	38121763	38121763	T	C	UTR3	PPAPDC1B	ENST000005	.	.	A	G	-	+-	0.05882353	48,0,3,0	chr8,381217	Cytosol	A > G
chr8:38828238:A:G:ncRNA_introni	chr8	38828238	38828238	A	G	ncRNA_intro	CTD-2544N1	.	.	.	A	G	+	+-	0.15189873	66,1,12,0	chr8,388282	Cytosol	A > G
chr8:38828267:A:G:ncRNA_introni	chr8	38828267	38828267	A	G	ncRNA_intro	CTD-2544N1	.	.	.	A	G	+	+-	0.23287671	56,0,17,0	chr8,388282	Cytosol	A > G
chr8:38829211:A:G:ncRNA_introni	chr8	38829211	38829211	A	G	ncRNA_intro	CTD-2544N1	.	.	.	A	G	+	-+	0.05084746	112,0,6,0	chr8,388292	Cytosol	A > G
chr8:38829220:A:G:ncRNA_introni	chr8	38829220	38829220	A	G	ncRNA_intro	CTD-2544N1	.	.	.	A	G	+	-+	0.0530303	125,0,7,0	chr8,388292	Cytosol	A > G
chr8:38829345:A:G:ncRNA_introni	chr8	38829345	38829345	A	G	ncRNA_intro	CTD-2544N1	.	.	.	A	G	+	-+	0.26153846	48,0,17,0	chr8,388293	Cytosol	A > G
chr8:38829416:A:G:ncRNA_introni	chr8	38829416	38829416	A	G	ncRNA_intro	CTD-2544N1	.	.	.	A	G	+	-+	0.1563981	178,0,33,0	chr8,388294	Cytosol	A > G
chr8:38829754:A:G:ncRNA_introni	chr8	38829754	38829754	A	G	ncRNA_intro	CTD-2544N1	.	.	.	A	G	+	-+	0.14018692	184,0,30,0	chr8,388297	Cytosol	A > G
chr8:38829775:A:G:ncRNA_introni	chr8	38829775	38829775	A	G	ncRNA_intro	CTD-2544N1	.	.	.	A	G	+	-+	0.08035714	103,0,9,0	chr8,388297	Cytosol	A > G
chr8:38829839:A:G:ncRNA_introni	chr8	38829839	38829839	A	G	ncRNA_intro	CTD-2544N1	.	.	.	A	G	+	-+	0.45714286	114,0,96,0	chr8,388298	Cytosol	A > G
chr8:38829847:A:G:ncRNA_introni	chr8	38829847	38829847	A	G	ncRNA_intro	CTD-2544N1	.	.	.	A	G	+	-+	0.10606061	177,0,21,0	chr8,388298	Cytosol	A > G
chr8:38829965:A:G:ncRNA_exonic	chr8	38829965	38829965	A	G	ncRNA_exon	CTD-2544N1	.	.	.	A	G	+	-+	0.04761905	160,0,8,0	chr8,388299	Cytosol	A > G
chr8:38829966:A:G:ncRNA_exonic	chr8	38829966	38829966	A	G	ncRNA_exon	CTD-2544N1	.	.	.	A	G	+	-+	0.18604651	140,0,32,0	chr8,388299	Cytosol	A > G
chr8:38830480:A:G:ncRNA_exonic	chr8	38830480	38830480	A	G	ncRNA_exon	CTD-2544N1	.	.	.	A	G	+	+-	0.01694915	174,0,3,0	chr8,388304	Cytosol	A > G
chr8:38830496:A:G:ncRNA_exonic	chr8	38830496	38830496	A	G	ncRNA_exon	CTD-2544N1	.	.	.	A	G	+	+-	0.02758621	141,0,4,0	chr8,388304	Cytosol	A > G
chr8:38830531:A:G:UTR3:PLEKHA	chr8	38830531	38830531	A	G	UTR3	PLEKHA2	ENST000004	.	.	A	G	+	+	0.07264957	217,0,17,0	chr8,388305	Cytosol	A > G
chr8:38830536:A:G:UTR3:PLEKHA	chr8	38830536	38830536	A	G	UTR3	PLEKHA2	ENST000004	.	.	A	G	+	+	0.12692308	227,0,33,0	chr8,388305	Cytosol	A > G
chr8:38830556:A:G:UTR3:PLEKHA	chr8	38830556	38830556	A	G	UTR3	PLEKHA2	ENST000004	.	.	A	G	+	+	0.03963415	315,0,13,0	chr8,388305	Cytosol	A > G
chr8:38830557:A:G:UTR3:PLEKHA	chr8	38830557	38830557	A	G	UTR3	PLEKHA2	ENST000004	.	.	A	G	+	+	0.10946746	301,0,37,0	chr8,388305	Cytosol	A > G
chr8:38830585:A:G:UTR3:PLEKHA	chr8	38830585	38830585	A	G	UTR3	PLEKHA2	ENST000004	.	.	A	G	+	+	0.0292887	232,0,7,0	chr8,388305	Cytosol	A > G
chr8:38830586:A:G:UTR3:PLEKHA	chr8	38830586	38830586	A	G	UTR3	PLEKHA2	ENST000004	.	.	A	G	+	+	0.10869565	205,0,25,0	chr8,388305	Cytosol	A > G
chr8:38830589:A:G:UTR3:PLEKHA	chr8	38830589	38830589	A	G	UTR3	PLEKHA2	ENST000004	.	.	A	G	+	+	0.01904762	206,0,4,0	chr8,388305	Cytosol	A > G
chr8:41399640:A:G:ncRNA_introni	chr8	41399640	41399640	A	G	ncRNA_intro	RP11-360L9.	.	.	.	A	G	+	+-	0.58064516	26,0,36,0	chr8,413996	Cytosol	A > G
chr8:41399648:A:G:ncRNA_introni	chr8	41399648	41399648	A	G	ncRNA_intro	RP11-360L9.	.	.	.	A	G	+	+-	0.53246753	36,0,41,0	chr8,413996	Cytosol	A > G
chr8:41399653:A:G:ncRNA_introni	chr8	41399653	41399653	A	G	ncRNA_intro	RP11-360L9.	.	.	.	A	G	+	+-	0.38461538	48,0,30,0	chr8,413996	Cytosol	A > G
chr8:41399686:A:G:ncRNA_introni	chr8	41399686	41399686	A	G	ncRNA_intro	RP11-360L9.	.	.	.	A	G	+	+-	0.02272727	86,0,2,0	chr8,413996	Cytosol	A > G
chr8:41399700:A:G:ncRNA_introni	chr8	41399700	41399700	A	G	ncRNA_intro	RP11-360L9.	.	.	.	A	G	+	+-	0.03846154	75,0,3,0	chr8,413996	Cytosol	A > G
chr8:41399706:A:G:ncRNA_introni	chr8	41399706	41399706	A	G	ncRNA_intro	RP11-360L9.	.	.	.	A	G	+	+-	0.06756757	69,0,5,0	chr8,413997	Cytosol	A > G
chr8:41399713:A:G:ncRNA_introni	chr8	41399713	41399713	A	G	ncRNA_intro	RP11-360L9.	.	.	.	A	G	+	+-	0.1641791	56,0,11,0	chr8,413997	Cytosol	A > G
chr8:41399714:A:G:ncRNA_introni	chr8	41399714	41399714	A	G	ncRNA_intro	RP11-360L9.	.	.	.	A	G	+	+-	0.26153846	48,0,17,0	chr8,413997	Cytosol	A > G
chr8:41399722:A:G:ncRNA_introni	chr8	41399722	41399722	A	G	ncRNA_intro	RP11-360L9.	.	.	.	A	G	+	+-	0.32692308	35,0,17,0	chr8,413997	Cytosol	A > G
chr8:41399723:A:G:ncRNA_introni	chr8	41399723	41399723	A	G	ncRNA_intro	RP11-360L9.	.	.	.	A	G	+	+-	0.36956522	29,0,17,0	chr8,413997	Cytosol	A > G
chr8:41399724:A:G:ncRNA_introni	chr8	41399724	41399724	A	G	ncRNA_intro	RP11-360L9.	.	.	.	A	G	+	+-	0.35897436	25,0,14,0	chr8,413997	Cytosol	A > G
chr8:41399738:A:G:ncRNA_introni	chr8	41399738	41399738	A	G	ncRNA_intro	RP11-360L9.	.	.	.	A	G	+	+-	0.12820513	34,0,5,0	chr8,413997	Cytosol	A > G
chr8:41399739:A:G:ncRNA_introni	chr8	41399739	41399739	A	G	ncRNA_intro	RP11-360L9.	.	.	.	A	G	+	+-	0.15	34,0,6,0	chr8,413997	Cytosol	A > G
chr8:41399746:A:G:ncRNA_introni	chr8	41399746	41399746	A	G	ncRNA_intro	RP11-360L9.	.	.	.	A	G	+	+-	0.14285714	30,0,5,0	chr8,413997	Cytosol	A > G
chr8:41399755:A:G:ncRNA_introni	chr8	41399755	41399755	A	G	ncRNA_intro	RP11-360L9.	.	.	.	A	G	+	+-	0.85714286	4,0,24,0	chr8,413997	Cytosol	A > G
chr8:41399783:A:G:ncRNA_introni	chr8	41399783	41399783	A	G	ncRNA_intro	RP11-360L9.	.	.	.	A	G	+	+-	0.16666667	25,0,5,0	chr8,413997	Cytosol	A > G
chr8:41399788:A:G:ncRNA_introni	chr8	41399788	41399788	A	G	ncRNA_intro	RP11-360L9.	.	.	.	A	G	+	+-	0.06060606	31,0,2,0	chr8,413997	Cytosol	A > G
chr8:41399792:A:G:ncRNA_introni	chr8	41399792	41399792	A	G	ncRNA_intro	RP11-360L9.	.	.	.	A	G	+	+-	0.34375	21,0,11,0	chr8,413997	Cytosol	A > G
chr8:41399796:A:G:ncRNA_introni	chr8	41399796	41399796	A	G	ncRNA_intro	RP11-360L9.	.	.	.	A	G	+	+-	0.64705882	12,0,22,0	chr8,413997	Cytosol	A > G
chr8:41399823:A:G:ncRNA_introni	chr8	41399823	41399823	A	G	ncRNA_intro	RP11-360L9.	.	.	.	A	G	+	+-	0.46575342	39,0,34,0	chr8,413998	Cytosol	A > G
chr8:41399824:A:G:ncRNA_introni	chr8	41399824	41399824	A	G	ncRNA_intro	RP11-360L9.	.	.	.	A	G	+	+-	0.16883117	64,0,13,0	chr8,413998	Cytosol	A > G
chr8:41399831:A:G:ncRNA_introni	chr8	41399831	41399831	A	G	ncRNA_intro	RP11-360L9.	.	.	.	A	G	+	+-	0.04878049	78,0,4,0	chr8,413998	Cytosol	A > G
chr8:41399951:A:G:ncRNA_introni	chr8	41399951	41399951	A	G	ncRNA_intro	RP11-360L9.	.	.	.	A	G	+	+-	0.0972973	167,0,18,0	chr8,413999	Cytosol	A > G
chr8:41400010:A:G:ncRNA_introni	chr8	41400010	41400010	A	G	ncRNA_intro	RP11-360L9.	.	.	.	A	G	+	+-	0.06060606	155,0,10,0	chr8,414000	Cytosol	A > G
chr8:41400013:A:G:ncRNA_introni	chr8	41400013	41400013	A	G	ncRNA_intro	RP11-360L9.	.	.	.	A	G	+	+-	0.0931677	146,0,15,0	chr8,414000	Cytosol	A > G
chr8:41400014:A:G:ncRNA_introni	chr8	41400014	41400014	A	G	ncRNA_intro	RP11-360L9.	.	.	.	A	G	+	+-	0.09815951	147,0,16,0	chr8,414000	Cytosol	A > G
chr8:41400024:A:G:ncRNA_introni	chr8	41400024	41400024	A	G	ncRNA_intro	RP11-360L9.	.	.	.	A	G	+	+-	0.13836478	137,0,22,0	chr8,414000	Cytosol	A > G

chr8:41400026:A:G:ncRNA_introni	chr8	41400026	41400026	A	G	ncRNA_intro	RP11-360L9.	.	.	.	A	G	+	+-	0.10059172	152,0,17,0	chr8,414000	Cytosol	A > G
chr8:41400029:A:G:ncRNA_introni	chr8	41400029	41400029	A	G	ncRNA_intro	RP11-360L9.	.	.	.	A	G	+	+-	0.57317073	70,0,94,0	chr8,414000	Cytosol	A > G
chr8:41400033:A:G:ncRNA_introni	chr8	41400033	41400033	A	G	ncRNA_intro	RP11-360L9.	.	.	.	A	G	+	+-	0.15540541	125,0,23,0	chr8,414000	Cytosol	A > G
chr8:41400059:A:G:ncRNA_introni	chr8	41400059	41400059	A	G	ncRNA_intro	RP11-360L9.	.	.	.	A	G	+	+-	0.51048951	70,0,73,0	chr8,414000	Cytosol	A > G
chr8:41400086:A:G:ncRNA_introni	chr8	41400086	41400086	A	G	ncRNA_intro	RP11-360L9.	.	.	.	A	G	+	+-	0.07272727	102,0,8,0	chr8,414000	Cytosol	A > G
chr8:41400087:A:G:ncRNA_introni	chr8	41400087	41400087	A	G	ncRNA_intro	RP11-360L9.	.	.	.	A	G	+	+-	0.24528302	80,0,26,0	chr8,414000	Cytosol	A > G
chr8:41401290:A:G:ncRNA_introni	chr8	41401290	41401290	A	G	ncRNA_intro	RP11-360L9.	.	.	.	A	G	+	+-	0.11009174	97,0,12,0	chr8,414012	Cytosol	A > G
chr8:41401304:A:G:ncRNA_introni	chr8	41401304	41401304	A	G	ncRNA_intro	RP11-360L9.	.	.	.	A	G	+	+-	0.28448276	83,0,33,0	chr8,414013	Cytosol	A > G
chr8:41401324:A:G:ncRNA_introni	chr8	41401324	41401324	A	G	ncRNA_intro	RP11-360L9.	.	.	.	A	G	+	+-	0.2	84,0,21,0	chr8,414013	Cytosol	A > G
chr8:41401325:A:G:ncRNA_introni	chr8	41401325	41401325	A	G	ncRNA_intro	RP11-360L9.	.	.	.	A	G	+	+-	0.48623853	56,0,53,0	chr8,414013	Cytosol	A > G
chr8:41401351:A:G:ncRNA_introni	chr8	41401351	41401351	A	G	ncRNA_intro	RP11-360L9.	.	.	.	A	G	+	+-	0.58252427	43,0,60,0	chr8,414013	Cytosol	A > G
chr8:41401354:A:G:ncRNA_introni	chr8	41401354	41401354	A	G	ncRNA_intro	RP11-360L9.	.	.	.	A	G	+	+-	0.9375	6,0,90,0	chr8,414013	Cytosol	A > G
chr8:41401382:A:G:ncRNA_introni	chr8	41401382	41401382	A	G	ncRNA_intro	RP11-360L9.	.	.	.	A	G	+	+-	0.265625	47,0,17,0	chr8,414013	Cytosol	A > G
chr8:41401386:A:G:ncRNA_introni	chr8	41401386	41401386	A	G	ncRNA_intro	RP11-360L9.	.	.	.	A	G	+	+-	0.22033898	46,0,13,0	chr8,414013	Cytosol	A > G
chr8:41401390:A:G:ncRNA_introni	chr8	41401390	41401390	A	G	ncRNA_intro	RP11-360L9.	.	.	.	A	G	+	+-	0.3442623	40,0,21,0	chr8,414013	Cytosol	A > G
chr8:41401391:A:G:ncRNA_introni	chr8	41401391	41401391	A	G	ncRNA_intro	RP11-360L9.	.	.	.	A	G	+	+-	0.625	24,0,40,0	chr8,414013	Cytosol	A > G
chr8:41401400:A:G:ncRNA_introni	chr8	41401400	41401400	A	G	ncRNA_intro	RP11-360L9.	.	.	.	A	G	+	+-	0.19354839	49,0,12,1	chr8,414013	Cytosol	A > G
chr8:41401406:A:G:ncRNA_introni	chr8	41401406	41401406	A	G	ncRNA_intro	RP11-360L9.	.	.	.	A	G	+	+-	0.32352941	46,0,22,0	chr8,414014	Cytosol	A > G
chr8:41401409:A:G:ncRNA_introni	chr8	41401409	41401409	A	G	ncRNA_intro	RP11-360L9.	.	.	.	A	G	+	+-	0.3	42,0,18,0	chr8,414014	Cytosol	A > G
chr8:41401426:A:G:ncRNA_introni	chr8	41401426	41401426	A	G	ncRNA_intro	RP11-360L9.	.	.	.	A	G	+	+-	0.23287671	56,0,17,0	chr8,414014	Cytosol	A > G
chr8:41401436:A:G:ncRNA_introni	chr8	41401436	41401436	A	G	ncRNA_intro	RP11-360L9.	.	.	.	A	G	+	+-	0.36507937	40,0,23,0	chr8,414014	Cytosol	A > G
chr8:41401465:A:G:ncRNA_introni	chr8	41401465	41401465	A	G	ncRNA_intro	RP11-360L9.	.	.	.	A	G	+	+-	0.125	49,0,7,0	chr8,414014	Cytosol	A > G
chr8:41401469:A:G:ncRNA_introni	chr8	41401469	41401469	A	G	ncRNA_intro	RP11-360L9.	.	.	.	A	G	+	+-	0.63793103	21,0,37,0	chr8,414014	Cytosol	A > G
chr8:41401473:A:G:ncRNA_splicin	chr8	41401473	41401473	A	G	ncRNA_splici	RP11-360L9.	ENST000005	.	.	A	G	+	+-	0.25581395	32,0,11,0	chr8,414014	Cytosol	A > G
chr8:41401487:A:G:ncRNA_exonic	chr8	41401487	41401487	A	G	ncRNA_exon	RP11-360L9.	.	.	.	A	G	+	+-	0.11904762	37,0,5,0	chr8,414014	Cytosol	A > G
chr8:41401488:A:G:ncRNA_exonic	chr8	41401488	41401488	A	G	ncRNA_exon	RP11-360L9.	.	.	.	A	G	+	+-	0.66666667	10,0,20,0	chr8,414014	Cytosol	A > G
chr8:41401497:A:G:ncRNA_exonic	chr8	41401497	41401497	A	G	ncRNA_exon	RP11-360L9.	.	.	.	A	G	+	+-	0.15	17,0,3,0	chr8,414014	Cytosol	A > G
chr8:41403073:A:G:ncRNA_introni	chr8	41403073	41403073	A	G	ncRNA_intro	RP11-360L9.	.	.	.	A	G	+	-	0.8	1,0,4,0	chr8,414030	Cytosol	A > G
chr8:42386059:T:C:intronic:SLC20	chr8	42386059	42386059	T	C	intronic	SLC20A2	.	.	.	A	G	-	-	0.38461538	8,0,5,0	chr8,423860	Cytosol	A > G
chr8:42387034:T:C:intronic:SLC20	chr8	42387034	42387034	T	C	intronic	SLC20A2	.	.	.	A	G	-	-	0.44444444	5,0,4,0	chr8,423870	Cytosol	A > G
chr8:42387058:T:C:intronic:SLC20	chr8	42387058	42387058	T	C	intronic	SLC20A2	.	.	.	A	G	-	-	0.33333333	8,0,4,0	chr8,423870	Cytosol	A > G
chr8:42387100:T:C:intronic:SLC20	chr8	42387100	42387100	T	C	intronic	SLC20A2	.	.	.	A	G	-	-	0.6	2,0,3,0	chr8,423870	Cytosol	A > G
chr8:42387243:T:C:intronic:SLC20	chr8	42387243	42387243	T	C	intronic	SLC20A2	.	.	.	A	G	-	-	0.23529412	13,0,4,0	chr8,423872	Cytosol	A > G
chr8:42387261:T:C:intronic:SLC20	chr8	42387261	42387261	T	C	intronic	SLC20A2	.	.	.	A	G	-	-	0.17647059	14,0,3,0	chr8,423872	Cytosol	A > G
chr8:42387265:T:C:intronic:SLC20	chr8	42387265	42387265	T	C	intronic	SLC20A2	.	.	.	A	G	-	-	0.125	14,0,2,0	chr8,423872	Cytosol	A > G
chr8:42388502:T:C:intronic:SLC20	chr8	42388502	42388502	T	C	intronic	SLC20A2	.	.	.	A	G	-	-	0.28571429	5,0,2,0	chr8,423885	Cytosol	A > G
chr8:42388593:T:C:intronic:SLC20	chr8	42388593	42388593	T	C	intronic	SLC20A2	.	.	.	A	G	-	-	1	0,0,13,0	chr8,423885	Cytosol	A > G
chr8:42388610:T:C:intronic:SLC20	chr8	42388610	42388610	T	C	intronic	SLC20A2	.	.	.	A	G	-	-	0.38461538	8,0,5,0	chr8,423886	Cytosol	A > G
chr8:42388615:T:C:intronic:SLC20	chr8	42388615	42388615	T	C	intronic	SLC20A2	.	.	.	A	G	-	-	0.33333333	8,0,4,0	chr8,423886	Cytosol	A > G
chr8:42388631:T:C:intronic:SLC20	chr8	42388631	42388631	T	C	intronic	SLC20A2	.	.	.	A	G	-	-	0.13333333	13,0,2,0	chr8,423886	Cytosol	A > G
chr8:42388637:T:C:intronic:SLC20	chr8	42388637	42388637	T	C	intronic	SLC20A2	.	.	.	A	G	-	-	0.13333333	13,0,2,0	chr8,423886	Cytosol	A > G
chr8:42388673:T:C:intronic:SLC20	chr8	42388673	42388673	T	C	intronic	SLC20A2	.	.	.	A	G	-	-	0.90909091	1,0,10,0	chr8,423886	Cytosol	A > G
chr8:42388676:T:C:intronic:SLC20	chr8	42388676	42388676	T	C	intronic	SLC20A2	.	.	.	A	G	-	-	0.9	1,0,9,0	chr8,423886	Cytosol	A > G
chr8:42388927:T:C:intronic:SLC20	chr8	42388927	42388927	T	C	intronic	SLC20A2	.	.	.	A	G	-	-	1	0,0,7,0	chr8,423889	Cytosol	A > G
chr8:42388939:T:C:intronic:SLC20	chr8	42388939	42388939	T	C	intronic	SLC20A2	.	.	.	A	G	-	-	0.57142857	3,0,4,0	chr8,423889	Cytosol	A > G
chr8:42388940:T:C:intronic:SLC20	chr8	42388940	42388940	T	C	intronic	SLC20A2	.	.	.	A	G	-	-	0.75	2,0,6,0	chr8,423889	Cytosol	A > G
chr8:42388966:T:C:intronic:SLC20	chr8	42388966	42388966	T	C	intronic	SLC20A2	.	.	.	A	G	-	-	0.83333333	1,0,5,0	chr8,423889	Cytosol	A > G
chr8:42388969:T:C:intronic:SLC20	chr8	42388969	42388969	T	C	intronic	SLC20A2	.	.	.	A	G	-	-	0.42857143	4,0,3,0	chr8,423889	Cytosol	A > G
chr8:42388970:T:C:intronic:SLC20	chr8	42388970	42388970	T	C	intronic	SLC20A2	.	.	.	A	G	-	-	0.57142857	3,0,4,0	chr8,423889	Cytosol	A > G
chr8:42388975:T:C:intronic:SLC20	chr8	42388975	42388975	T	C	intronic	SLC20A2	.	.	.	A	G	-	-	0.28571429	5,0,2,0	chr8,423889	Cytosol	A > G
chr8:42388984:T:C:intronic:SLC20	chr8	42388984	42388984	T	C	intronic	SLC20A2	.	.	.	A	G	-	-	0.28571429	5,0,2,0	chr8,423889	Cytosol	A > G
chr8:42389215:T:C:intronic:SLC20	chr8	42389215	42389215	T	C	intronic	SLC20A2	.	.	.	A	G	-	-	0.16666667	10,0,2,0	chr8,423892	Cytosol	A > G
chr8:42389248:T:C:intronic:SLC20	chr8	42389248	42389248	T	C	intronic	SLC20A2	.	.	.	A	G	-	-	0.16666667	10,0,2,0	chr8,423892	Cytosol	A > G
chr8:42389275:T:C:intronic:SLC20	chr8	42389275	42389275	T	C	intronic	SLC20A2	.	.	.	A	G	-	-	0.69230769	4,0,9,0	chr8,423892	Cytosol	A > G
chr8:42389294:T:C:intronic:SLC20	chr8	42389294	42389294	T	C	intronic	SLC20A2	.	.	.	A	G	-	-	0.91666667	1,0,11,0	chr8,423892	Cytosol	A > G
chr8:42389297:T:C:intronic:SLC20	chr8	42389297	42389297	T	C	intronic	SLC20A2	.	.	.	A	G	-	-	0.23076923	10,0,3,0	chr8,423892	Cytosol	A > G
chr8:42389336:T:C:intronic:SLC20	chr8	42389336	42389336	T	C	intronic	SLC20A2	.	.	.	A	G	-	-	0.46666667	8,0,7,0	chr8,423893	Cytosol	A > G
chr8:42389339:T:C:intronic:SLC20	chr8	42389339	42389339	T	C	intronic	SLC20A2	.	.	.	A	G	-	-	0.71428571	4,0,10,0	chr8,423893	Cytosol	A > G
chr8:42389342:T:C:intronic:SLC20	chr8	42389342	42389342	T	C	intronic	SLC20A2	.	.	.	A	G	-	-	0.14285714	12,0,2,0	chr8,423893	Cytosol	A > G
chr8:42389346:T:C:intronic:SLC20	chr8	42389346	42389346	T	C	intronic	SLC20A2	.	.	.	A	G	-	-	0.75	3,0,9,0	chr8,423893	Cytosol	A > G
chr8:42389359:T:C:intronic:SLC20	chr8	42389359	42389359	T	C	intronic	SLC20A2	.	.	.	A	G	-	-	0.17647059	14,0,3,0	chr8,423893	Cytosol	A > G
chr8:42389365:T:C:intronic:SLC20	chr8	42389365	42389365	T	C	intronic	SLC20A2	.	.	.	A	G	-	-	0.29411765	12,0,5,0	chr8,423893	Cytosol	A > G
chr8:42875593:A:G:UTR3:HOOK3	chr8	42875593	42875593	A	G	UTR3	HOOK3	ENST000003	.	.	A	G	+	+	0.25	6,0,2,0	chr8,428755	Cytosol	A > G
chr8:42875602:A:G:UTR3:HOOK3	chr8	42875602	42875602	A	G	UTR3	HOOK3	ENST000003	.	.	A	G	+	+	0.57142857	3,0,4,0	chr8,428756	Cytosol	A > G

chr8:42875696:A:G:UTR3:HOOK3	chr8	42875696	42875696	A	G	UTR3	HOOK3	ENST000003	.	.	A	G	+	+	0.57142857	3,0,4,0	chr8,428756	Cytosol	A > G
chr8:42875710:A:G:UTR3:HOOK3	chr8	42875710	42875710	A	G	UTR3	HOOK3	ENST000003	.	.	A	G	+	+	0.6	4,0,6,0	chr8,428757	Cytosol	A > G
chr8:42875736:A:G:UTR3:HOOK3	chr8	42875736	42875736	A	G	UTR3	HOOK3	ENST000003	.	.	A	G	+	+	0.38461538	8,0,5,0	chr8,428757	Cytosol	A > G
chr8:42875737:A:G:UTR3:HOOK3	chr8	42875737	42875737	A	G	UTR3	HOOK3	ENST000003	.	.	A	G	+	+	0.66666667	4,0,8,0	chr8,428757	Cytosol	A > G
chr8:42875746:A:G:UTR3:HOOK3	chr8	42875746	42875746	A	G	UTR3	HOOK3	ENST000003	.	.	A	G	+	+	0.15384615	11,0,2,0	chr8,428757	Cytosol	A > G
chr8:42875782:A:G:UTR3:HOOK3	chr8	42875782	42875782	A	G	UTR3	HOOK3	ENST000003	.	.	A	G	+	+	0.1875	13,0,3,0	chr8,428757	Cytosol	A > G
chr8:42875789:A:G:UTR3:HOOK3	chr8	42875789	42875789	A	G	UTR3	HOOK3	ENST000003	.	.	A	G	+	+	0.13333333	13,0,2,0	chr8,428757	Cytosol	A > G
chr8:42875814:A:G:UTR3:HOOK3	chr8	42875814	42875814	A	G	UTR3	HOOK3	ENST000003	.	.	A	G	+	+	0.41666667	7,0,5,0	chr8,428758	Cytosol	A > G
chr8:42875980:A:G:UTR3:HOOK3	chr8	42875980	42875980	A	G	UTR3	HOOK3	ENST000003	.	.	A	G	+	+	0.25	15,0,5,0	chr8,428759	Cytosol	A > G
chr8:42875986:A:G:UTR3:HOOK3	chr8	42875986	42875986	A	G	UTR3	HOOK3	ENST000003	.	.	A	G	+	+	0.15	17,0,3,0	chr8,428759	Cytosol	A > G
chr8:42875988:A:G:UTR3:HOOK3	chr8	42875988	42875988	A	G	UTR3	HOOK3	ENST000003	.	.	A	G	+	+	0.14285714	18,0,3,0	chr8,428759	Cytosol	A > G
chr8:42875989:A:G:UTR3:HOOK3	chr8	42875989	42875989	A	G	UTR3	HOOK3	ENST000003	.	.	A	G	+	+	0.33333333	14,0,7,0	chr8,428759	Cytosol	A > G
chr8:42876015:A:G:UTR3:HOOK3	chr8	42876015	42876015	A	G	UTR3	HOOK3	ENST000003	.	.	A	G	+	+	0.10526316	17,0,2,0	chr8,428760	Cytosol	A > G
chr8:42876048:A:G:UTR3:HOOK3	chr8	42876048	42876048	A	G	UTR3	HOOK3	ENST000003	.	.	A	G	+	+	0.17391304	19,0,4,0	chr8,428760	Cytosol	A > G
chr8:42876058:A:G:UTR3:HOOK3	chr8	42876058	42876058	A	G	UTR3	HOOK3	ENST000003	.	.	A	G	+	+	0.33333333	18,0,9,0	chr8,428760	Cytosol	A > G
chr8:42876068:A:G:UTR3:HOOK3	chr8	42876068	42876068	A	G	UTR3	HOOK3	ENST000003	.	.	A	G	+	+	0.2173913	18,0,5,0	chr8,428760	Cytosol	A > G
chr8:42876098:A:G:UTR3:HOOK3	chr8	42876098	42876098	A	G	UTR3	HOOK3	ENST000003	.	.	A	G	+	+	0.33333333	6,0,3,0	chr8,428760	Cytosol	A > G
chr8:42876102:A:G:UTR3:HOOK3	chr8	42876102	42876102	A	G	UTR3	HOOK3	ENST000003	.	.	A	G	+	+	0.28571429	5,0,2,0	chr8,428761	Cytosol	A > G
chr8:42876124:A:G:UTR3:HOOK3	chr8	42876124	42876124	A	G	UTR3	HOOK3	ENST000003	.	.	A	G	+	+	0.2	8,0,2,0	chr8,428761	Cytosol	A > G
chr8:42876177:A:G:UTR3:HOOK3	chr8	42876177	42876177	A	G	UTR3	HOOK3	ENST000003	.	.	A	G	+	+	0.5	3,0,3,0	chr8,428761	Cytosol	A > G
chr8:42876497:A:G:UTR3:HOOK3	chr8	42876497	42876497	A	G	UTR3	HOOK3	ENST000003	.	.	A	G	+	+	0.09090909	20,0,2,0	chr8,428764	Cytosol	A > G
chr8:42876521:A:G:UTR3:HOOK3	chr8	42876521	42876521	A	G	UTR3	HOOK3	ENST000003	.	.	A	G	+	+	0.13333333	13,0,2,0	chr8,428765	Cytosol	A > G
chr8:42876526:A:G:UTR3:HOOK3	chr8	42876526	42876526	A	G	UTR3	HOOK3	ENST000003	.	.	A	G	+	+	0.15789474	16,0,3,0	chr8,428765	Cytosol	A > G
chr8:42876546:A:G:UTR3:HOOK3	chr8	42876546	42876546	A	G	UTR3	HOOK3	ENST000003	.	.	A	G	+	+	0.25	9,0,3,0	chr8,428765	Cytosol	A > G
chr8:42876547:A:G:UTR3:HOOK3	chr8	42876547	42876547	A	G	UTR3	HOOK3	ENST000003	.	.	A	G	+	+	0.21428571	11,0,3,0	chr8,428765	Cytosol	A > G
chr8:42876551:A:G:UTR3:HOOK3	chr8	42876551	42876551	A	G	UTR3	HOOK3	ENST000003	.	.	A	G	+	+	0.23076923	10,0,3,0	chr8,428765	Cytosol	A > G
chr8:42876586:A:G:UTR3:HOOK3	chr8	42876586	42876586	A	G	UTR3	HOOK3	ENST000003	.	.	A	G	+	+	0.5	7,0,7,0	chr8,428765	Cytosol	A > G
chr8:42876621:A:G:UTR3:HOOK3	chr8	42876621	42876621	A	G	UTR3	HOOK3	ENST000003	.	.	A	G	+	+	0.30769231	9,0,4,0	chr8,428766	Cytosol	A > G
chr8:42876626:A:G:UTR3:HOOK3	chr8	42876626	42876626	A	G	UTR3	HOOK3	ENST000003	.	.	A	G	+	+	0.41666667	7,0,5,0	chr8,428766	Cytosol	A > G
chr8:42876718:A:G:UTR3:HOOK3	chr8	42876718	42876718	A	G	UTR3	HOOK3	ENST000003	.	.	A	G	+	+	0.36363636	7,0,4,0	chr8,428767	Cytosol	A > G
chr8:42878657:A:G:UTR3:HOOK3	chr8	42878657	42878657	A	G	UTR3	HOOK3	ENST000003	.	.	A	G	+	+	0.1	18,0,2,0	chr8,428786	Cytosol	A > G
chr8:42878722:A:G:UTR3:HOOK3	chr8	42878722	42878722	A	G	UTR3	HOOK3	ENST000003	.	.	A	G	+	+	0.28571429	5,0,2,0	chr8,428787	Cytosol	A > G
chr8:42878725:A:G:UTR3:HOOK3	chr8	42878725	42878725	A	G	UTR3	HOOK3	ENST000003	.	.	A	G	+	+	0.66666667	2,0,4,0	chr8,428787	Cytosol	A > G
chr8:42878729:A:G:UTR3:HOOK3	chr8	42878729	42878729	A	G	UTR3	HOOK3	ENST000003	.	.	A	G	+	+	0.6	2,0,3,0	chr8,428787	Cytosol	A > G
chr8:42878826:A:G:UTR3:HOOK3	chr8	42878826	42878826	A	G	UTR3	HOOK3	ENST000003	.	.	A	G	+	+	0.28571429	10,0,4,0	chr8,428788	Cytosol	A > G
chr8:42878838:A:G:UTR3:HOOK3	chr8	42878838	42878838	A	G	UTR3	HOOK3	ENST000003	.	.	A	G	+	+	0.27272727	8,0,3,0	chr8,428788	Cytosol	A > G
chr8:42878851:A:G:UTR3:HOOK3	chr8	42878851	42878851	A	G	UTR3	HOOK3	ENST000003	.	.	A	G	+	+	0.46153846	7,0,6,0	chr8,428788	Cytosol	A > G
chr8:42878878:A:G:UTR3:HOOK3	chr8	42878878	42878878	A	G	UTR3	HOOK3	ENST000003	.	.	A	G	+	+	0.36363636	7,0,4,0	chr8,428788	Cytosol	A > G
chr8:42882066:A:G:UTR3:HOOK3	chr8	42882066	42882066	A	G	UTR3	HOOK3	ENST000003	.	.	A	G	+	+	0.30769231	9,0,4,0	chr8,428820	Cytosol	A > G
chr8:42882152:A:G:UTR3:HOOK3	chr8	42882152	42882152	A	G	UTR3	HOOK3	ENST000003	.	.	A	G	+	+	0.35	13,0,7,0	chr8,428821	Cytosol	A > G
chr8:42883363:A:G:UTR3:HOOK3	chr8	42883363	42883363	A	G	UTR3	HOOK3	ENST000003	.	.	A	G	+	+	0.14285714	12,0,2,0	chr8,428833	Cytosol	A > G
chr8:42883416:A:G:UTR3:HOOK3	chr8	42883416	42883416	A	G	UTR3	HOOK3	ENST000003	.	.	A	G	+	+	0.33333333	10,0,5,0	chr8,428834	Cytosol	A > G
chr8:42883417:A:G:UTR3:HOOK3	chr8	42883417	42883417	A	G	UTR3	HOOK3	ENST000003	.	.	A	G	+	+	0.2	12,0,3,0	chr8,428834	Cytosol	A > G
chr8:42883422:A:G:UTR3:HOOK3	chr8	42883422	42883422	A	G	UTR3	HOOK3	ENST000003	.	.	A	G	+	+	0.14285714	12,0,2,0	chr8,428834	Cytosol	A > G
chr8:42884354:A:G:UTR3:HOOK3	chr8	42884354	42884354	A	G	UTR3	HOOK3	ENST000003	.	.	A	G	+	+	0.17241379	24,0,5,0	chr8,428843	Cytosol	A > G
chr8:42884367:A:G:UTR3:HOOK3	chr8	42884367	42884367	A	G	UTR3	HOOK3	ENST000003	.	.	A	G	+	+	0.05882353	32,0,2,0	chr8,428843	Cytosol	A > G
chr8:42884393:A:G:UTR3:HOOK3	chr8	42884393	42884393	A	G	UTR3	HOOK3	ENST000003	.	.	A	G	+	+	0.1	27,0,3,0	chr8,428843	Cytosol	A > G
chr8:42884422:A:G:UTR3:HOOK3	chr8	42884422	42884422	A	G	UTR3	HOOK3	ENST000003	.	.	A	G	+	+	0.13157895	33,0,5,0	chr8,428844	Cytosol	A > G
chr8:42884423:A:G:UTR3:HOOK3	chr8	42884423	42884423	A	G	UTR3	HOOK3	ENST000003	.	.	A	G	+	+	0.11111111	32,0,4,0	chr8,428844	Cytosol	A > G
chr8:42884473:A:G:UTR3:HOOK3	chr8	42884473	42884473	A	G	UTR3	HOOK3	ENST000003	.	.	A	G	+	+	0.08	23,0,2,0	chr8,428844	Cytosol	A > G
chr8:42884565:A:G:UTR3:HOOK3	chr8	42884565	42884565	A	G	UTR3	HOOK3	ENST000003	.	.	A	G	+	+	0.10344828	26,0,3,0	chr8,428845	Cytosol	A > G
chr8:48889633:A:G:UTR3:MCM4	chr8	48889633	48889633	A	G	UTR3	MCM4	ENST000005	.	.	A	G	+	+	0.13414634	355,0,55,0	chr8,488896	Cytosol	A > G
chr8:48889659:A:G:UTR3:MCM4	chr8	48889659	48889659	A	G	UTR3	MCM4	ENST000005	.	.	A	G	+	+	0.04356061	505,0,23,0	chr8,488896	Cytosol	A > G
chr8:48889672:A:G:UTR3:MCM4	chr8	48889672	48889672	A	G	UTR3	MCM4	ENST000005	.	.	A	G	+	+	0.03416149	622,0,22,0	chr8,488896	Cytosol	A > G
chr8:48890109:A:G:UTR3:MCM4	chr8	48890109	48890109	A	G	UTR3	MCM4	ENST000005	.	.	A	G	+	+	0.30973451	546,0,245,0	chr8,488901	Cytosol	A > G
chr8:48890114:A:G:UTR3:MCM4	chr8	48890114	48890114	A	G	UTR3	MCM4	ENST000005	.	.	A	G	+	+	0.03305785	702,0,24,0	chr8,488901	Cytosol	A > G
chr8:48890247:A:G:UTR3:MCM4	chr8	48890247	48890247	A	G	UTR3	MCM4	ENST000005	.	.	A	G	+	+	0.08333333	561,0,51,0	chr8,488902	Cytosol	A > G
chr8:48890288:A:G:UTR3:MCM4	chr8	48890288	48890288	A	G	UTR3	MCM4	ENST000005	.	.	A	G	+	+	0.03605016	615,0,23,0	chr8,488902	Cytosol	A > G
chr8:56737575:A:G:UTR3:TGS1	chr8	56737575	56737575	A	G	UTR3	TGS1	ENST000002	.	.	A	G	+	+	0.06493506	72,0,5,0	chr8,567375	Cytosol	A > G
chr8:56737580:A:G:UTR3:TGS1	chr8	56737580	56737580	A	G	UTR3	TGS1	ENST000002	.	.	A	G	+	+	0.03076923	63,0,2,0	chr8,567375	Cytosol	A > G
chr8:56980055:T:C:UTR3:RPS20	chr8	56980055	56980055	T	C	UTR3	RPS20	ENST000005	.	.	A	G	-	-	0.11764706	15,0,2,0	chr8,569800	Cytosol	A > G
chr8:56980900:T:C:UTR3:RPS20	chr8	56980900	56980900	T	C	UTR3	RPS20	ENST000005	.	.	A	G	-	-	0.46153846	7,0,6,0	chr8,569808	Cytosol	A > G
chr8:56980985:T:C:UTR3:RPS20	chr8	56980985	56980985	T	C	UTR3	RPS20	ENST000005	.	.	A	G	-	-	0.61538462	5,0,8,0	chr8,569809	Cytosol	A > G
chr8:56981830:T:C:intronic:RPS20	chr8	56981830	56981830	T	C	intronic	RPS20	.	.	.	A	G	-	-	0.14285714	12,0,2,0	chr8,569818	Cytosol	A > G

chr8:56981943:T:C:intronic:RPS20	chr8	56981943	56981943	T	C	intronic	RPS20	.	.	.	A	G	-	-	0.22222222	14,0,4,0	chr8,569819	Cytosol	A > G
chr8:56981972:T:C:intronic:RPS20	chr8	56981972	56981972	T	C	intronic	RPS20	.	.	.	A	G	-	-	0.23076923	10,0,3,0	chr8,569819	Cytosol	A > G
chr8:56984986:T:C:intronic:RPS20	chr8	56984986	56984986	T	C	intronic	RPS20	.	.	.	A	G	-	-	0.26666667	11,0,4,0	chr8,569849	Cytosol	A > G
chr8:82610475:T:C:intergenic:SLC1	chr8	82610475	82610475	T	C	intergenic	SLC10A5,ZFA	dist=2066;dis	.	.	A	G	-		0.4	3,0,2,0	chr8,826104	Cytosol	A > G
chr8:95804469:A:G:ncRNA_exonic	chr8	95804469	95804469	A	G	ncRNA_exon	KB-1608C10.	.	.	.	A	G	+	+-	0.28571429	10,0,4,0	chr8,958044	Cytosol	A > G
chr8:95804605:A:G:ncRNA_introni	chr8	95804605	95804605	A	G	ncRNA_intro	KB-1608C10.	.	.	.	A	G	+	+-	0.88235294	2,0,15,0	chr8,958046	Cytosol	A > G
chr8:95804659:A:G:ncRNA_introni	chr8	95804659	95804659	A	G	ncRNA_intro	KB-1608C10.	.	.	.	A	G	+	+-	0.26315789	14,0,5,0	chr8,958046	Cytosol	A > G
chr8:95804662:A:G:ncRNA_introni	chr8	95804662	95804662	A	G	ncRNA_intro	KB-1608C10.	.	.	.	A	G	+	+-	0.375	10,0,6,0	chr8,958046	Cytosol	A > G
chr8:95804676:A:G:ncRNA_introni	chr8	95804676	95804676	A	G	ncRNA_intro	KB-1608C10.	.	.	.	A	G	+	+-	0.5625	7,0,9,0	chr8,958046	Cytosol	A > G
chr8:95804719:A:G:ncRNA_introni	chr8	95804719	95804719	A	G	ncRNA_intro	KB-1608C10.	.	.	.	A	G	+	+-	0.22222222	7,0,2,0	chr8,958047	Cytosol	A > G
chr8:95804750:A:G:ncRNA_introni	chr8	95804750	95804750	A	G	ncRNA_intro	KB-1608C10.	.	.	.	A	G	+	+-	1	0,0,8,0	chr8,958047	Cytosol	A > G
chr8:95804829:A:G:ncRNA_introni	chr8	95804829	95804829	A	G	ncRNA_intro	KB-1608C10.	.	.	.	A	G	+	+-	1	0,0,8,0	chr8,958048	Cytosol	A > G
chr8:95804830:A:G:ncRNA_introni	chr8	95804830	95804830	A	G	ncRNA_intro	KB-1608C10.	.	.	.	A	G	+	+-	0.85714286	1,0,6,0	chr8,958048	Cytosol	A > G
chr8:95804860:A:G:ncRNA_introni	chr8	95804860	95804860	A	G	ncRNA_intro	KB-1608C10.	.	.	.	A	G	+	+-	0.15789474	16,0,3,0	chr8,958048	Cytosol	A > G
chr8:95804907:A:G:ncRNA_introni	chr8	95804907	95804907	A	G	ncRNA_intro	KB-1608C10.	.	.	.	A	G	+	+-	0.375	10,0,6,0	chr8,958049	Cytosol	A > G
chr8:95804927:A:G:ncRNA_introni	chr8	95804927	95804927	A	G	ncRNA_intro	KB-1608C10.	.	.	.	A	G	+	+-	0.5	9,0,9,0	chr8,958049	Cytosol	A > G
chr8:95804937:A:G:ncRNA_introni	chr8	95804937	95804937	A	G	ncRNA_intro	KB-1608C10.	.	.	.	A	G	+	+-	0.72222222	5,0,13,0	chr8,958049	Cytosol	A > G
chr8:95804963:A:G:ncRNA_exonic	chr8	95804963	95804963	A	G	ncRNA_exon	KB-1608C10.	.	.	.	A	G	+	+-	0.6875	5,0,11,0	chr8,958049	Cytosol	A > G
chr8:95804969:A:G:ncRNA_exonic	chr8	95804969	95804969	A	G	ncRNA_exon	KB-1608C10.	.	.	.	A	G	+	+-	0.6	6,0,9,0	chr8,958049	Cytosol	A > G
chr8:95804973:A:G:ncRNA_exonic	chr8	95804973	95804973	A	G	ncRNA_exon	KB-1608C10.	.	.	.	A	G	+	+-	0.30769231	9,0,4,0	chr8,958049	Cytosol	A > G
chr8:95805038:A:G:ncRNA_exonic	chr8	95805038	95805038	A	G	ncRNA_exon	KB-1608C10.	.	.	.	A	G	+	+-	0.61904762	8,0,13,0	chr8,958050	Cytosol	A > G
chr8:95805070:A:G:ncRNA_exonic	chr8	95805070	95805070	A	G	ncRNA_exon	KB-1608C10.	.	.	.	A	G	+	+-	0.57142857	6,0,8,0	chr8,958050	Cytosol	A > G
chr8:95805071:A:G:ncRNA_exonic	chr8	95805071	95805071	A	G	ncRNA_exon	KB-1608C10.	.	.	.	A	G	+	+-	0.21052632	15,0,4,0	chr8,958050	Cytosol	A > G
chr8:96257845:T:C:ncRNA_introni	chr8	96257845	96257845	T	C	ncRNA_intro	KB-1047C11.	.	.	.	A	G	-	+-	0.42857143	4,0,3,0	chr8,962578	Cytosol	A > G
chr8:96257855:T:C:ncRNA_introni	chr8	96257855	96257855	T	C	ncRNA_intro	KB-1047C11.	.	.	.	A	G	-	+-	0.85714286	1,0,6,0	chr8,962578	Cytosol	A > G
chr8:96257867:T:C:ncRNA_introni	chr8	96257867	96257867	T	C	ncRNA_intro	KB-1047C11.	.	.	.	A	G	-	+-	1	0,0,6,0	chr8,962578	Cytosol	A > G
chr8:96257923:T:C:ncRNA_introni	chr8	96257923	96257923	T	C	ncRNA_intro	KB-1047C11.	.	.	.	A	G	-	+-	0.5	3,0,3,0	chr8,962579	Cytosol	A > G
chr8:96258302:T:C:ncRNA_introni	chr8	96258302	96258302	T	C	ncRNA_intro	KB-1047C11.	.	.	.	A	G	-	+-	0.28571429	5,0,2,0	chr8,962583	Cytosol	A > G
chr8:96258354:T:C:ncRNA_introni	chr8	96258354	96258354	T	C	ncRNA_intro	KB-1047C11.	.	.	.	A	G	-	+-	0.66666667	2,0,4,0	chr8,962583	Cytosol	A > G
chr8:96258355:T:C:ncRNA_introni	chr8	96258355	96258355	T	C	ncRNA_intro	KB-1047C11.	.	.	.	A	G	-	+-	1	0,0,5,0	chr8,962583	Cytosol	A > G
chr8:96258381:T:C:ncRNA_introni	chr8	96258381	96258381	T	C	ncRNA_intro	KB-1047C11.	.	.	.	A	G	-	+-	1	0,0,5,0	chr8,962583	Cytosol	A > G
chr8:96258396:T:C:ncRNA_introni	chr8	96258396	96258396	T	C	ncRNA_intro	KB-1047C11.	.	.	.	A	G	-	+-	0.6	2,0,3,0	chr8,962583	Cytosol	A > G
chr8:96258399:T:C:ncRNA_introni	chr8	96258399	96258399	T	C	ncRNA_intro	KB-1047C11.	.	.	.	A	G	-	+-	0.4	3,0,2,0	chr8,962583	Cytosol	A > G
chr8:96258435:T:C:ncRNA_introni	chr8	96258435	96258435	T	C	ncRNA_intro	KB-1047C11.	.	.	.	A	G	-	+-	0.27272727	8,0,3,0	chr8,962584	Cytosol	A > G
chr8:96258473:T:C:ncRNA_introni	chr8	96258473	96258473	T	C	ncRNA_intro	KB-1047C11.	.	.	.	A	G	-	+-	0.7	3,0,7,0	chr8,962584	Cytosol	A > G
chr8:98739048:A:G:UTR3:MTDH	chr8	98739048	98739048	A	G	UTR3	MTDH	ENST000003	.	.	A	G	+	+	0.03571429	54,0,2,0	chr8,987390	Cytosol	A > G
chr8:98739208:A:G:UTR3:MTDH	chr8	98739208	98739208	A	G	UTR3	MTDH	ENST000003	.	.	A	G	+	+	0.02564103	76,0,2,0	chr8,987392	Cytosol	A > G
chr8:98739594:A:G:UTR3:MTDH	chr8	98739594	98739594	A	G	UTR3	MTDH	ENST000003	.	.	A	G	+	+	0.09459459	67,0,7,0	chr8,987395	Cytosol	A > G
chr8:98740894:A:G:UTR3:MTDH	chr8	98740894	98740894	A	G	UTR3	MTDH	ENST000003	.	.	A	G	+	+	0.03030303	64,0,2,0	chr8,987408	Cytosol	A > G
chr8:98740895:A:G:UTR3:MTDH	chr8	98740895	98740895	A	G	UTR3	MTDH	ENST000003	.	.	A	G	+	+	0.08955224	61,0,6,0	chr8,987408	Cytosol	A > G
chr8:98740900:A:G:UTR3:MTDH	chr8	98740900	98740900	A	G	UTR3	MTDH	ENST000003	.	.	A	G	+	+	0.10169492	53,0,6,0	chr8,987408	Cytosol	A > G
chr8:98740935:A:G:UTR3:MTDH	chr8	98740935	98740935	A	G	UTR3	MTDH	ENST000003	.	.	A	G	+	+	0.05084746	56,0,3,0	chr8,987409	Cytosol	A > G
chr8:98740940:A:G:UTR3:MTDH	chr8	98740940	98740940	A	G	UTR3	MTDH	ENST000003	.	.	A	G	+	+	0.03448276	56,0,2,0	chr8,987409	Cytosol	A > G
chr8:98741163:A:G:downstream:M	chr8	98741163	98741163	A	G	downstream	MTDH	.	.	.	A	G	+	+	0.52631579	9,0,10,0	chr8,987411	Cytosol	A > G
chr8:98741259:A:G:downstream:M	chr8	98741259	98741259	A	G	downstream	MTDH	.	.	.	A	G	+	+	0.12048193	73,0,10,0	chr8,987412	Cytosol	A > G
chr8:98741287:A:G:downstream:M	chr8	98741287	98741287	A	G	downstream	MTDH	.	.	.	A	G	+	+	0.05813953	80,0,5,1	chr8,987412	Cytosol	A > G
chr8:98741314:A:G:downstream:M	chr8	98741314	98741314	A	G	downstream	MTDH	.	.	.	A	G	+	+	0.25925926	20,0,7,0	chr8,987413	Cytosol	A > G
chr8:98741344:A:G:downstream:M	chr8	98741344	98741344	A	G	downstream	MTDH	.	.	.	A	G	+	+	0.19047619	17,0,4,0	chr8,987413	Cytosol	A > G
chr8:98741390:A:G:downstream:M	chr8	98741390	98741390	A	G	downstream	MTDH	.	.	.	A	G	+	+	0.16216216	62,0,12,0	chr8,987413	Cytosol	A > G
chr8:98741416:A:G:downstream:M	chr8	98741416	98741416	A	G	downstream	MTDH	.	.	.	A	G	+	+	0.07894737	70,0,6,0	chr8,987414	Cytosol	A > G
chr8:98741417:A:G:downstream:M	chr8	98741417	98741417	A	G	downstream	MTDH	.	.	.	A	G	+	+	0.27272727	56,0,21,0	chr8,987414	Cytosol	A > G
chr8:98741429:A:G:downstream:M	chr8	98741429	98741429	A	G	downstream	MTDH	.	.	.	A	G	+	+	0.05333333	71,0,4,0	chr8,987414	Cytosol	A > G
chr8:98741914:A:G:downstream:M	chr8	98741914	98741914	A	G	downstream	MTDH	.	.	.	A	G	+	+	0.05494505	86,0,5,0	chr8,987419	Cytosol	A > G
chr8:98741955:A:G:downstream:M	chr8	98741955	98741955	A	G	downstream	MTDH	.	.	.	A	G	+	+	0.04301075	89,0,4,0	chr8,987419	Cytosol	A > G
chr8:98741972:A:G:downstream:M	chr8	98741972	98741972	A	G	downstream	MTDH	.	.	.	A	G	+	+	0.1971831	57,0,14,0	chr8,987419	Cytosol	A > G
chr8:98741993:A:G:downstream:M	chr8	98741993	98741993	A	G	downstream	MTDH	.	.	.	A	G	+	+	0.06060606	62,0,4,0	chr8,987419	Cytosol	A > G
chr8:99055437:T:C:ncRNA_exonic:	chr8	99055437	99055437	T	C	ncRNA_exon	KB-1208A12.	.	.	.	A	G	-	+-	0.55555556	4,0,5,0	chr8,990554	Cytosol	A > G
chr8:99055492:T:C:ncRNA_exonic:	chr8	99055492	99055492	T	C	ncRNA_exon	KB-1208A12.	.	.	.	A	G	-	+-	0.22222222	7,0,2,0	chr8,990554	Cytosol	A > G
chr8:99055493:T:C:ncRNA_exonic:	chr8	99055493	99055493	T	C	ncRNA_exon	KB-1208A12.	.	.	.	A	G	-	+-	0.42857143	4,0,3,0	chr8,990554	Cytosol	A > G
chr8:99055911:T:C:ncRNA_introni	chr8	99055911	99055911	T	C	ncRNA_intro	KB-1208A12.	.	.	.	A	G	-	+-	0.28571429	10,0,4,0	chr8,990559	Cytosol	A > G
chr8:99055914:T:C:ncRNA_introni	chr8	99055914	99055914	T	C	ncRNA_intro	KB-1208A12.	.	.	.	A	G	-	+-	0.52380952	10,0,11,0	chr8,990559	Cytosol	A > G
chr8:99056286:T:C:ncRNA_exonic:	chr8	99056286	99056286	T	C	ncRNA_exon	KB-1208A12.	.	.	.	A	G	-	+-	0.28571429	5,0,2,0	chr8,990562	Cytosol	A > G
chr8:99056338:T:C:ncRNA_exonic:	chr8	99056338	99056338	T	C	ncRNA_exon	KB-1208A12.	.	.	.	A	G	-	+-	0.3125	11,0,5,0	chr8,990563	Cytosol	A > G
chr8:103256346:A:G:ncRNA_exoni	chr8	103256346	103256346	A	G	ncRNA_exon	KB-431C1.4	.	.	.	A	G	+	+	0.1875	13,0,3,0	chr8,103256	Cytosol	A > G

chr8:104411926:T:C:UTR3:SLC25A	chr8	104411926	104411926	T	C	UTR3	SLC25A32	ENST000002	.	.	A	G	-	-	0.04864865	176,0,9,0	chr8,104411	Cytosol	A > G
chr8:104411941:T:C:UTR3:SLC25A	chr8	104411941	104411941	T	C	UTR3	SLC25A32	ENST000002	.	.	A	G	-	-	0.04278075	179,0,8,0	chr8,104411	Cytosol	A > G
chr8:104412011:T:C:UTR3:SLC25A	chr8	104412011	104412011	T	C	UTR3	SLC25A32	ENST000002	.	.	A	G	-	-	0.03012048	161,0,5,0	chr8,104412	Cytosol	A > G
chr8:128170700:T:C:intergenic:PC	chr8	128170700	128170700	T	C	intergenic	PCAT2,RP11-	dist=76234;d	.	.	A	G	-		0.8	1,0,4,0	chr8,128170	Cytosol	A > G
chr8:141526475:A:G:UTR3:CHRAC	chr8	141526475	141526475	A	G	UTR3	CHRAC1	ENST000002	.	.	A	G	+	+	0.01025641	193,0,2,0	chr8,141526	Cytosol	A > G
chr8:143741910:T:C:ncRNA_exoni	chr8	143741910	143741910	T	C	ncRNA_exon	JRK	.	.	.	A	G	-	-	0.21052632	15,0,4,0	chr8,143741	Cytosol	A > G
chr8:143741972:T:C:ncRNA_exoni	chr8	143741972	143741972	T	C	ncRNA_exon	JRK	.	.	.	A	G	-	-	0.125	14,0,2,0	chr8,143741	Cytosol	A > G
chr8:144672885:T:C:UTR5:EEF1D	chr8	144672885	144672885	T	C	UTR5	EEF1D	ENST000004	.	.	A	G	-	-	0.71428571	4,0,10,0	chr8,144672	Cytosol	A > G
chr8:144737578:A:G:intergenic:ZN	chr8	144737578	144737578	A	G	intergenic	ZNF623,ZNF7	dist=1678;dis	.	.	A	G	+		0.33333333	18,0,9,0	chr8,144737	Cytosol	A > G
chr8:144737595:A:G:intergenic:ZN	chr8	144737595	144737595	A	G	intergenic	ZNF623,ZNF7	dist=1695;dis	.	.	A	G	+		0.16129032	26,0,5,0	chr8,144737	Cytosol	A > G
chr8:144737716:A:G:intergenic:ZN	chr8	144737716	144737716	A	G	intergenic	ZNF623,ZNF7	dist=1816;dis	.	.	A	G	+		0.4	3,0,2,0	chr8,144737	Cytosol	A > G
chr8:144738033:A:G:intergenic:ZN	chr8	144738033	144738033	A	G	intergenic	ZNF623,ZNF7	dist=2133;dis	.	.	A	G	+		0.57142857	3,0,4,0	chr8,144738	Cytosol	A > G
chr8:144738136:A:G:intergenic:ZN	chr8	144738136	144738136	A	G	intergenic	ZNF623,ZNF7	dist=2236;dis	.	.	A	G	+		0.07692308	24,0,2,0	chr8,144738	Cytosol	A > G
chr8:145157092:T:C:intronic:SHAR	chr8	145157092	145157092	T	C	intronic	SHARPIN	.	.	.	A	G	-	-	0.83333333	1,0,5,0	chr8,145157	Cytosol	A > G
chr8:146034692:T:C:UTR3:ZNF517	chr8	146034692	146034692	T	C	UTR3	ZNF517	ENST000005	.	.	A	G	-	+	0.25	6,0,2,0	chr8,146034	Cytosol	A > G
chr8:146199743:T:C:ncRNA_exoni	chr8	146199743	146199743	T	C	ncRNA_exon	ZNF252P	.	.	.	A	G	-	-	0.66666667	9,0,18,0	chr8,146199	Cytosol	A > G
chr8:146200271:T:C:ncRNA_exoni	chr8	146200271	146200271	T	C	ncRNA_exon	ZNF252P	.	.	.	A	G	-	-	0.15789474	16,0,3,0	chr8,146200	Cytosol	A > G
chr8:146200315:T:C:ncRNA_exoni	chr8	146200315	146200315	T	C	ncRNA_exon	ZNF252P	.	.	.	A	G	-	-	0.24	19,0,6,0	chr8,146200	Cytosol	A > G
chr8:146200325:T:C:ncRNA_exoni	chr8	146200325	146200325	T	C	ncRNA_exon	ZNF252P	.	.	.	A	G	-	-	0.20689655	23,0,6,0	chr8,146200	Cytosol	A > G
chr9:2760408:T:C:intronic:KIAA00	chr9	2760408	2760408	T	C	intronic	KIAA0020	.	.	.	A	G	-	-	0.71428571	2,0,5,0	chr9,276040	Cytosol	A > G
chr9:4710319:T:C:downstream:AK	chr9	4710319	4710319	T	C	downstream	AK3	.	.	.	A	G	-	-	0.73684211	5,0,14,0	chr9,471031	Cytosol	A > G
chr9:4710815:T:C:downstream:AK	chr9	4710815	4710815	T	C	downstream	AK3	.	.	.	A	G	-	-	0.87179487	5,0,34,0	chr9,471081	Cytosol	A > G
chr9:4710877:T:C:downstream:AK	chr9	4710877	4710877	T	C	downstream	AK3	.	.	.	A	G	-	-	1	0,0,19,0	chr9,471087	Cytosol	A > G
chr9:4729148:T:C:intronic:AK3	chr9	4729148	4729148	T	C	intronic	AK3	.	.	.	A	G	-	-	0.5	3,0,3,0	chr9,472914	Cytosol	A > G
chr9:4885692:A:G:intergenic:RCL1	chr9	4885692	4885692	A	G	intergenic	RCL1,AL1581	dist=24628;d	.	.	A	G	+	+	0.83333333	1,0,5,0	chr9,488569	Cytosol	A > G
chr9:15463323:A:G:UTR3:SNAPC3	chr9	15463323	15463323	A	G	UTR3	SNAPC3	ENST000003	.	.	A	G	+	+	0.22222222	7,0,2,0	chr9,154633	Cytosol	A > G
chr9:15466143:A:G:intronic:PSIP1	chr9	15466143	15466143	A	G	intronic	PSIP1	.	.	.	A	G	+	-	0.28571429	5,0,2,0	chr9,154661	Cytosol	A > G
chr9:35087761:T:C:downstream:P	chr9	35087761	35087761	T	C	downstream	PIGO	.	.	.	A	G	-		0.6	2,0,3,0	chr9,350877	Cytosol	A > G
chr9:36215077:T:C:UTR3:GNE	chr9	36215077	36215077	T	C	UTR3	GNE	ENST000003	.	.	A	G	-	+-	0.28	18,0,7,0	chr9,362150	Cytosol	A > G
chr9:36215117:T:C:UTR3:GNE	chr9	36215117	36215117	T	C	UTR3	GNE	ENST000003	.	.	A	G	-	+-	0.42857143	8,0,6,0	chr9,362151	Cytosol	A > G
chr9:36215154:T:C:UTR3:GNE	chr9	36215154	36215154	T	C	UTR3	GNE	ENST000003	.	.	A	G	-	+-	0.15384615	11,0,2,0	chr9,362151	Cytosol	A > G
chr9:36215201:T:C:UTR3:GNE	chr9	36215201	36215201	T	C	UTR3	GNE	ENST000003	.	.	A	G	-	+-	0.40909091	13,0,9,0	chr9,362152	Cytosol	A > G
chr9:36215211:T:C:UTR3:GNE	chr9	36215211	36215211	T	C	UTR3	GNE	ENST000003	.	.	A	G	-	+-	0.61904762	8,0,13,0	chr9,362152	Cytosol	A > G
chr9:36215240:T:C:UTR3:GNE	chr9	36215240	36215240	T	C	UTR3	GNE	ENST000003	.	.	A	G	-	+-	0.71428571	2,0,5,0	chr9,362152	Cytosol	A > G
chr9:36215241:T:C:UTR3:GNE	chr9	36215241	36215241	T	C	UTR3	GNE	ENST000003	.	.	A	G	-	+-	0.875	1,0,7,0	chr9,362152	Cytosol	A > G
chr9:36215246:T:C:UTR3:GNE	chr9	36215246	36215246	T	C	UTR3	GNE	ENST000003	.	.	A	G	-	+-	0.6	4,0,6,0	chr9,362152	Cytosol	A > G
chr9:36215298:T:C:UTR3:GNE	chr9	36215298	36215298	T	C	UTR3	GNE	ENST000003	.	.	A	G	-	+-	0.62962963	30,0,51,0	chr9,362152	Cytosol	A > G
chr9:36216780:T:C:UTR3:GNE	chr9	36216780	36216780	T	C	UTR3	GNE	ENST000003	.	.	A	G	-	+-	0.24358974	59,0,19,0	chr9,362167	Cytosol	A > G
chr9:36216797:T:C:UTR3:GNE	chr9	36216797	36216797	T	C	UTR3	GNE	ENST000003	.	.	A	G	-	+-	0.10144928	62,0,7,0	chr9,362167	Cytosol	A > G
chr9:36216898:T:C:UTR3:GNE	chr9	36216898	36216898	T	C	UTR3	GNE	ENST000003	.	.	A	G	-	+-	0.35042735	76,0,41,0	chr9,362168	Cytosol	A > G
chr9:36678473:A:G:downstream:N	chr9	36678473	36678473	A	G	downstream	MELK	.	.	.	A	G	+		0.83333333	1,0,5,0	chr9,366784	Cytosol	A > G
chr9:37161016:A:G:intronic:ZCCH	chr9	37161016	37161016	A	G	intronic	ZCCHC7	.	.	.	A	G	+	+	1	0,0,6,0	chr9,371610	Cytosol	A > G
chr9:37432552:A:G:intronic:GRHP	chr9	37432552	37432552	A	G	intronic	GRHPR	.	.	.	A	G	+	+	0.4	3,0,2,0	chr9,374325	Cytosol	A > G
chr9:37432619:A:G:intronic:GRHP	chr9	37432619	37432619	A	G	intronic	GRHPR	.	.	.	A	G	+	+	0.30769231	9,0,4,0	chr9,374326	Cytosol	A > G
chr9:37503313:A:G:UTR3:POLR1E	chr9	37503313	37503313	A	G	UTR3	POLR1E	ENST000003	.	.	A	G	+	+	0.08648649	169,0,16,0	chr9,375033	Cytosol	A > G
chr9:37503346:A:G:UTR3:POLR1E	chr9	37503346	37503346	A	G	UTR3	POLR1E	ENST000003	.	.	A	G	+	+	0.10552764	178,0,21,0	chr9,375033	Cytosol	A > G
chr9:37503350:A:G:UTR3:POLR1E	chr9	37503350	37503350	A	G	UTR3	POLR1E	ENST000003	.	.	A	G	+	+	0.01648352	179,0,3,0	chr9,375033	Cytosol	A > G
chr9:37503367:A:G:UTR3:POLR1E	chr9	37503367	37503367	A	G	UTR3	POLR1E	ENST000003	.	.	A	G	+	+	0.04102564	187,0,8,0	chr9,375033	Cytosol	A > G
chr9:37503392:A:G:UTR3:POLR1E	chr9	37503392	37503392	A	G	UTR3	POLR1E	ENST000003	.	.	A	G	+	+	0.07207207	206,0,16,0	chr9,375033	Cytosol	A > G
chr9:37503454:A:G:UTR3:POLR1E	chr9	37503454	37503454	A	G	UTR3	POLR1E	ENST000003	.	.	A	G	+	+	0.07949791	220,0,19,0	chr9,375034	Cytosol	A > G
chr9:37503455:A:G:UTR3:POLR1E	chr9	37503455	37503455	A	G	UTR3	POLR1E	ENST000003	.	.	A	G	+	+	0.08050847	217,0,19,0	chr9,375034	Cytosol	A > G
chr9:37503464:A:G:UTR3:POLR1E	chr9	37503464	37503464	A	G	UTR3	POLR1E	ENST000003	.	.	A	G	+	+	0.0990566	191,0,21,0	chr9,375034	Cytosol	A > G
chr9:37503490:A:G:UTR3:POLR1E	chr9	37503490	37503490	A	G	UTR3	POLR1E	ENST000003	.	.	A	G	+	+	0.08333333	187,0,17,0	chr9,375034	Cytosol	A > G
chr9:37503538:A:G:UTR3:POLR1E	chr9	37503538	37503538	A	G	UTR3	POLR1E	ENST000003	.	.	A	G	+	+	0.06481481	202,0,14,0	chr9,375035	Cytosol	A > G
chr9:37504414:A:G:downstream:P	chr9	37504414	37504414	A	G	downstream	POLR1E	.	.	.	A	G	+		0.11111111	16,0,2,0	chr9,375044	Cytosol	A > G
chr9:37504429:A:G:downstream:P	chr9	37504429	37504429	A	G	downstream	POLR1E	.	.	.	A	G	+		0.25	18,0,6,0	chr9,375044	Cytosol	A > G
chr9:37504499:A:G:downstream:P	chr9	37504499	37504499	A	G	downstream	POLR1E	.	.	.	A	G	+		0.15	17,0,3,0	chr9,375044	Cytosol	A > G
chr9:37504535:A:G:downstream:P	chr9	37504535	37504535	A	G	downstream	POLR1E	.	.	.	A	G	+		0.18181818	9,0,2,0	chr9,375045	Cytosol	A > G
chr9:37505573:A:G:intergenic:POL	chr9	37505573	37505573	A	G	intergenic	POLR1E,RP1	dist=1879;dis	.	.	A	G	+		0.42857143	8,0,6,0	chr9,375055	Cytosol	A > G
chr9:37505692:A:G:intergenic:POL	chr9	37505692	37505692	A	G	intergenic	POLR1E,RP1	dist=1998;dis	.	.	A	G	+	+	0.10204082	44,0,5,0	chr9,375056	Cytosol	A > G
chr9:37505697:A:G:intergenic:POL	chr9	37505697	37505697	A	G	intergenic	POLR1E,RP1	dist=2003;dis	.	.	A	G	+	+	0.06	47,0,3,0	chr9,375056	Cytosol	A > G
chr9:37505720:A:G:intergenic:POL	chr9	37505720	37505720	A	G	intergenic	POLR1E,RP1	dist=2026;dis	.	.	A	G	+	+	0.15217391	39,0,7,0	chr9,375057	Cytosol	A > G
chr9:37510062:A:G:ncRNA_introni	chr9	37510062	37510062	A	G	ncRNA_intro	RP11-613M1	.	.	.	A	G	+	+	0.13793103	50,0,8,0	chr9,375100	Cytosol	A > G
chr9:37510102:A:G:ncRNA_introni	chr9	37510102	37510102	A	G	ncRNA_intro	RP11-613M1	.	.	.	A	G	+	+	0.1875	52,0,12,0	chr9,375101	Cytosol	A > G

chr9:37510105:A:G:ncRNA_introni	chr9	37510105	37510105	A	G	ncRNA_intro	RP11-613M1	.	.	.	A	G	+	+	0.45454545	30,0,25,0	chr9,375101(	Cytosol	A > G
chr9:37510114:A:G:ncRNA_introni	chr9	37510114	37510114	A	G	ncRNA_intro	RP11-613M1	.	.	.	A	G	+	+	0.05769231	49,0,3,0	chr9,375101	Cytosol	A > G
chr9:37510143:A:G:ncRNA_introni	chr9	37510143	37510143	A	G	ncRNA_intro	RP11-613M1	.	.	.	A	G	+	+	0.33333333	18,0,9,0	chr9,375101	Cytosol	A > G
chr9:37510179:A:G:ncRNA_introni	chr9	37510179	37510179	A	G	ncRNA_intro	RP11-613M1	.	.	.	A	G	+	+	0.39130435	14,0,9,0	chr9,375101	Cytosol	A > G
chr9:37510184:A:G:ncRNA_introni	chr9	37510184	37510184	A	G	ncRNA_intro	RP11-613M1	.	.	.	A	G	+	+	0.12	22,0,3,0	chr9,375101	Cytosol	A > G
chr9:37510185:A:G:ncRNA_introni	chr9	37510185	37510185	A	G	ncRNA_intro	RP11-613M1	.	.	.	A	G	+	+	0.26086957	17,0,6,0	chr9,375101	Cytosol	A > G
chr9:37510188:A:G:ncRNA_introni	chr9	37510188	37510188	A	G	ncRNA_intro	RP11-613M1	.	.	.	A	G	+	+	0.52380952	10,0,11,0	chr9,375101	Cytosol	A > G
chr9:37510192:A:G:ncRNA_introni	chr9	37510192	37510192	A	G	ncRNA_intro	RP11-613M1	.	.	.	A	G	+	+	0.45	11,0,9,0	chr9,375101	Cytosol	A > G
chr9:37510214:A:G:ncRNA_introni	chr9	37510214	37510214	A	G	ncRNA_intro	RP11-613M1	.	.	.	A	G	+	+	0.15789474	16,0,3,0	chr9,375102	Cytosol	A > G
chr9:37510274:A:G:ncRNA_exonic	chr9	37510274	37510274	A	G	ncRNA_exon	RP11-613M1	.	.	.	A	G	+	+	0.08	23,0,2,0	chr9,375102	Cytosol	A > G
chr9:37510289:A:G:ncRNA_exonic	chr9	37510289	37510289	A	G	ncRNA_exon	RP11-613M1	.	.	.	A	G	+	+	0.15	17,0,3,0	chr9,375102	Cytosol	A > G
chr9:37510295:A:G:ncRNA_exonic	chr9	37510295	37510295	A	G	ncRNA_exon	RP11-613M1	.	.	.	A	G	+	+	0.16	21,0,4,0	chr9,375102	Cytosol	A > G
chr9:37510297:A:G:downstream:F	chr9	37510297	37510297	A	G	downstream	FBXO10,RP1	.	.	.	A	G	+	+	0.57142857	12,0,16,0	chr9,375102	Cytosol	A > G
chr9:37775602:T:C:intronic:EXOSC	chr9	37775602	37775602	T	C	intronic	EXOSC3,RP1	.	.	.	A	G	-	+-	0.5	3,0,3,0	chr9,377756	Cytosol	A > G
chr9:37775695:T:C:intronic:EXOSC	chr9	37775695	37775695	T	C	intronic	EXOSC3,RP1	.	.	.	A	G	-	+-	0.66666667	2,0,4,0	chr9,377756	Cytosol	A > G
chr9:37775733:T:C:intronic:EXOSC	chr9	37775733	37775733	T	C	intronic	EXOSC3,RP1	.	.	.	A	G	-	+-	0.6	2,0,3,0	chr9,377757	Cytosol	A > G
chr9:37885587:T:C:intronic:RP11-(	chr9	37885587	37885587	T	C	intronic	RP11-613M1	.	.	.	A	G	-	-	0.09090909	20,0,2,0	chr9,378855	Cytosol	A > G
chr9:80886883:A:G:UTR3:CEP78	chr9	80886883	80886883	A	G	UTR3	CEP78	ENST000004	.	.	A	G	+	+	0.84615385	2,0,11,0	chr9,808868	Cytosol	A > G
chr9:80886886:A:G:UTR3:CEP78	chr9	80886886	80886886	A	G	UTR3	CEP78	ENST000004	.	.	A	G	+	+	0.58333333	5,0,7,0	chr9,808868	Cytosol	A > G
chr9:80887177:A:G:UTR3:CEP78	chr9	80887177	80887177	A	G	UTR3	CEP78	ENST000004	.	.	A	G	+	+	0.3	7,0,3,0	chr9,808871	Cytosol	A > G
chr9:80887203:A:G:UTR3:CEP78	chr9	80887203	80887203	A	G	UTR3	CEP78	ENST000004	.	.	A	G	+	+	0.125	14,0,2,0	chr9,808872	Cytosol	A > G
chr9:80887216:A:G:UTR3:CEP78	chr9	80887216	80887216	A	G	UTR3	CEP78	ENST000004	.	.	A	G	+	+	0.16666667	10,0,2,0	chr9,808872	Cytosol	A > G
chr9:80888543:A:G:UTR3:CEP78	chr9	80888543	80888543	A	G	UTR3	CEP78	ENST000004	.	.	A	G	+	+	0.75	2,0,6,0	chr9,808885	Cytosol	A > G
chr9:80888620:A:G:UTR3:CEP78	chr9	80888620	80888620	A	G	UTR3	CEP78	ENST000004	.	.	A	G	+	+	0.52941176	8,0,9,0	chr9,808886	Cytosol	A > G
chr9:80888645:A:G:UTR3:CEP78	chr9	80888645	80888645	A	G	UTR3	CEP78	ENST000004	.	.	A	G	+	+	0.55555556	8,0,10,0	chr9,808886	Cytosol	A > G
chr9:80890742:A:G:UTR3:CEP78	chr9	80890742	80890742	A	G	UTR3	CEP78	ENST000004	.	.	A	G	+	+	0.09090909	20,0,2,0	chr9,808907	Cytosol	A > G
chr9:80890752:A:G:UTR3:CEP78	chr9	80890752	80890752	A	G	UTR3	CEP78	ENST000004	.	.	A	G	+	+	0.08695652	21,0,2,0	chr9,808907	Cytosol	A > G
chr9:80890756:A:G:UTR3:CEP78	chr9	80890756	80890756	A	G	UTR3	CEP78	ENST000004	.	.	A	G	+	+	0.20833333	19,0,5,0	chr9,808907	Cytosol	A > G
chr9:94054689:T:C:intronic:AUH	chr9	94054689	94054689	T	C	intronic	AUH	.	.	.	A	G	-	-	0.6	2,0,3,0	chr9,940546	Cytosol	A > G
chr9:94054698:T:C:intronic:AUH	chr9	94054698	94054698	T	C	intronic	AUH	.	.	.	A	G	-	-	0.4	3,0,2,0	chr9,940546	Cytosol	A > G
chr9:94054699:T:C:intronic:AUH	chr9	94054699	94054699	T	C	intronic	AUH	.	.	.	A	G	-	-	0.4	3,0,2,0	chr9,940546	Cytosol	A > G
chr9:94056597:T:C:intronic:AUH	chr9	94056597	94056597	T	C	intronic	AUH	.	.	.	A	G	-	-	0.28571429	5,0,2,0	chr9,940565	Cytosol	A > G
chr9:94841443:T:C:UTR3:SPTLC1	chr9	94841443	94841443	T	C	UTR3	SPTLC1	ENST000003	.	.	A	G	-	-	1	0,0,7,0	chr9,948414	Cytosol	A > G
chr9:96210723:T:C:UTR3:FAM120	chr9	96210723	96210723	T	C	UTR3	FAM120AOS	ENST000004	.	.	A	G	-	-	0.06521739	43,0,3,0	chr9,962107	Cytosol	A > G
chr9:98575711:T:C:ncRNA_exonic:	chr9	98575711	98575711	T	C	ncRNA_exon	LINC00476	.	.	.	A	G	-	-	0.4	3,0,2,0	chr9,985757	Cytosol	A > G
chr9:98778072:A:G:intergenic:ERC	chr9	98778072	98778072	A	G	intergenic	ERCC6L2,LIN	dist=1230;di	.	.	A	G	+	+	0.28571429	5,0,2,0	chr9,987780	Cytosol	A > G
chr9:103152766:A:G:intergenic:TE	chr9	103152766	103152766	A	G	intergenic	TEX10,AL353	dist=37545;d	.	.	A	G	+	+	0.4	3,0,2,0	chr9,103152	Cytosol	A > G
chr9:111781206:G:T:UTR3:TMEM2	chr9	111781206	111781206	G	T	UTR3	TMEM245	ENST000003	.	.	C	A	-	-	0.02173913	2,89,0,1	chr9,111781	Cytosol	C > A
chr9:114352776:T:C:intronic:PTGR	chr9	114352776	114352776	T	C	intronic	PTGR1	.	.	.	A	G	-	-	0.16666667	10,0,2,0	chr9,114352	Cytosol	A > G
chr9:115236049:A:G:intronic:C9or	chr9	115236049	115236049	A	G	intronic	C9orf147	.	.	.	A	G	+	-	0.85714286	2,0,12,0	chr9,115236	Cytosol	A > G
chr9:115237496:A:G:intronic:C9or	chr9	115237496	115237496	A	G	intronic	C9orf147	.	.	.	A	G	+	-	0.52941176	8,0,9,0	chr9,115237	Cytosol	A > G
chr9:115237497:A:G:intronic:C9or	chr9	115237497	115237497	A	G	intronic	C9orf147	.	.	.	A	G	+	-	0.875	2,0,14,0	chr9,115237	Cytosol	A > G
chr9:115237504:A:G:intronic:C9or	chr9	115237504	115237504	A	G	intronic	C9orf147	.	.	.	A	G	+	-	0.76470588	4,0,13,0	chr9,115237	Cytosol	A > G
chr9:115237516:A:G:intronic:C9or	chr9	115237516	115237516	A	G	intronic	C9orf147	.	.	.	A	G	+	-	0.625	6,0,10,0	chr9,115237	Cytosol	A > G
chr9:115446633:T:C:UTR3:INIP	chr9	115446633	115446633	T	C	UTR3	INIP	ENST000003	.	.	A	G	-	-	0.325	27,0,13,0	chr9,115446	Cytosol	A > G
chr9:115446721:T:C:UTR3:INIP	chr9	115446721	115446721	T	C	UTR3	INIP	ENST000003	.	.	A	G	-	-	0.56756757	16,0,21,0	chr9,115446	Cytosol	A > G
chr9:115446725:T:C:UTR3:INIP	chr9	115446725	115446725	T	C	UTR3	INIP	ENST000003	.	.	A	G	-	-	0.17073171	34,0,7,0	chr9,115446	Cytosol	A > G
chr9:115446750:T:C:UTR3:INIP	chr9	115446750	115446750	T	C	UTR3	INIP	ENST000003	.	.	A	G	-	-	0.09230769	59,0,6,0	chr9,115446	Cytosol	A > G
chr9:116054346:A:G:UTR3:PRPF4	chr9	116054346	116054346	A	G	UTR3	PRPF4	ENST000003	.	.	A	G	+	+	0.07291667	178,0,14,0	chr9,116054	Cytosol	A > G
chr9:116054413:A:G:UTR3:PRPF4	chr9	116054413	116054413	A	G	UTR3	PRPF4	ENST000003	.	.	A	G	+	+	0.01869159	210,0,4,0	chr9,116054	Cytosol	A > G
chr9:116054435:A:G:UTR3:PRPF4	chr9	116054435	116054435	A	G	UTR3	PRPF4	ENST000003	.	.	A	G	+	+	0.02777778	210,0,6,0	chr9,116054	Cytosol	A > G
chr9:116078139:T:C:UTR3:WDR31	chr9	116078139	116078139	T	C	UTR3	WDR31	ENST000003	.	.	A	G	-	-	0.33333333	4,0,2,0	chr9,116078	Cytosol	A > G
chr9:125579454:T:C:intronic:PDCL	chr9	125579454	125579454	T	C	intronic	PDCL	.	.	.	A	G	-	-	0.42857143	4,0,3,0	chr9,125579	Cytosol	A > G
chr9:127983230:A:G:UTR3:RABEPI	chr9	127983230	127983230	A	G	UTR3	RABEPK	ENST000003	.	.	A	G	+	+	0.8	1,0,4,0	chr9,127983	Cytosol	A > G
chr9:127984327:A:G:UTR3:RABEPI	chr9	127984327	127984327	A	G	UTR3	RABEPK	ENST000003	.	.	A	G	+	+	0.25	6,0,2,0	chr9,127984	Cytosol	A > G
chr9:130553170:A:G:downstream:	chr9	130553170	130553170	A	G	downstream	CDK9	.	.	.	A	G	+		0.17647059	14,0,3,0	chr9,130553	Cytosol	A > G
chr9:130553175:A:G:downstream:	chr9	130553175	130553175	A	G	downstream	CDK9	.	.	.	A	G	+		0.1875	13,0,3,0	chr9,130553	Cytosol	A > G
chr9:130553279:A:G:downstream:	chr9	130553279	130553279	A	G	downstream	CDK9	.	.	.	A	G	+		0.20689655	23,0,6,0	chr9,130553	Cytosol	A > G
chr9:130553653:A:G:downstream:	chr9	130553653	130553653	A	G	downstream	CDK9	.	.	.	A	G	+		0.15789474	16,0,3,0	chr9,130553	Cytosol	A > G
chr9:130557459:A:G:intergenic:CD	chr9	130557459	130557459	A	G	intergenic	CDK9,FPGS	dist=4393;di	.	.	A	G	+	+	0.16666667	10,0,2,0	chr9,130557	Cytosol	A > G
chr9:130557465:A:G:intergenic:CD	chr9	130557465	130557465	A	G	intergenic	CDK9,FPGS	dist=4399;di	.	.	A	G	+	+	0.15384615	11,0,2,0	chr9,130557	Cytosol	A > G
chr9:130557476:A:G:intergenic:CD	chr9	130557476	130557476	A	G	intergenic	CDK9,FPGS	dist=4410;di	.	.	A	G	+	+	0.16666667	10,0,2,0	chr9,130557	Cytosol	A > G
chr9:130557501:A:G:intergenic:CD	chr9	130557501	130557501	A	G	intergenic	CDK9,FPGS	dist=4435;di	.	.	A	G	+	+	0.25	6,0,2,0	chr9,130557	Cytosol	A > G
chr9:130669228:T:C:downstream:	chr9	130669228	130669228	T	C	downstream	ST6GALNAC4	.	.	.	A	G	-		0.4	3,0,2,0	chr9,130669	Cytosol	A > G

chr9:130671546:T:C:intronic:ST6G	chr9	130671546	130671546	T	C	intronic	ST6GALNAC4	.	.	.	A	G	-	-	0.14285714	12,0,2,0	chr9,130671	Cytosol	A > G
chr9:131067681:T:C:ncRNA_exoni	chr9	131067681	131067681	T	C	ncRNA_exon	RP11-339B21	.	.	.	A	G	-	-	0.05555556	34,0,2,0	chr9,131067	Cytosol	A > G
chr9:131067719:T:C:ncRNA_exoni	chr9	131067719	131067719	T	C	ncRNA_exon	RP11-339B21	.	.	.	A	G	-	-	0.17857143	23,0,5,0	chr9,131067	Cytosol	A > G
chr9:131067721:T:C:ncRNA_exoni	chr9	131067721	131067721	T	C	ncRNA_exon	RP11-339B21	.	.	.	A	G	-	-	0.24	19,0,6,0	chr9,131067	Cytosol	A > G
chr9:131067751:T:C:ncRNA_exoni	chr9	131067751	131067751	T	C	ncRNA_exon	RP11-339B21	.	.	.	A	G	-	-	0.75	5,0,15,0	chr9,131067	Cytosol	A > G
chr9:131067923:T:C:upstream;do	chr9	131067923	131067923	T	C	upstream;do	RP11-339B21	.	.	.	A	G	-		0.13333333	13,0,2,0	chr9,131067	Cytosol	A > G
chr9:131067940:T:C:upstream;do	chr9	131067940	131067940	T	C	upstream;do	RP11-339B21	.	.	.	A	G	-		0.07407407	25,0,2,0	chr9,131067	Cytosol	A > G
chr9:131067949:T:C:upstream;do	chr9	131067949	131067949	T	C	upstream;do	RP11-339B21	.	.	.	A	G	-		0.14814815	23,0,4,0	chr9,131067	Cytosol	A > G
chr9:131068011:T:C:upstream;do	chr9	131068011	131068011	T	C	upstream;do	RP11-339B21	.	.	.	A	G	-		0.24	19,0,6,0	chr9,131068	Cytosol	A > G
chr9:131068061:T:C:upstream;do	chr9	131068061	131068061	T	C	upstream;do	RP11-339B21	.	.	.	A	G	-		0.38888889	22,0,14,0	chr9,131068	Cytosol	A > G
chr9:131068857:T:C:upstream:RP1	chr9	131068857	131068857	T	C	upstream	RP11-339B21	.	.	.	A	G	-		0.70967742	9,0,22,0	chr9,131068	Cytosol	A > G
chr9:131068896:T:C:upstream:RP1	chr9	131068896	131068896	T	C	upstream	RP11-339B21	.	.	.	A	G	-		0.2	20,0,5,0	chr9,131068	Cytosol	A > G
chr9:131068897:T:C:upstream:RP1	chr9	131068897	131068897	T	C	upstream	RP11-339B21	.	.	.	A	G	-		0.22727273	17,0,5,0	chr9,131068	Cytosol	A > G
chr9:131068920:T:C:upstream:RP1	chr9	131068920	131068920	T	C	upstream	RP11-339B21	.	.	.	A	G	-		0.11111111	16,0,2,0	chr9,131068	Cytosol	A > G
chr9:131068921:T:C:upstream:RP1	chr9	131068921	131068921	T	C	upstream	RP11-339B21	.	.	.	A	G	-		0.11111111	16,0,2,0	chr9,131068	Cytosol	A > G
chr9:131068936:T:C:upstream:RP1	chr9	131068936	131068936	T	C	upstream	RP11-339B21	.	.	.	A	G	-		0.5	8,0,8,0	chr9,131068	Cytosol	A > G
chr9:131069050:T:C:upstream:RP1	chr9	131069050	131069050	T	C	upstream	RP11-339B21	.	.	.	A	G	-		0.65217391	8,0,15,0	chr9,131069	Cytosol	A > G
chr9:131069123:T:C:upstream:RP1	chr9	131069123	131069123	T	C	upstream	RP11-339B21	.	.	.	A	G	-		0.69047619	13,0,29,0	chr9,131069	Cytosol	A > G
chr9:131069436:T:C:upstream:RP1	chr9	131069436	131069436	T	C	upstream	RP11-339B21	.	.	.	A	G	-		0.5	13,0,13,0	chr9,131069	Cytosol	A > G
chr9:131070231:T:C:intergenic:RP	chr9	131070231	131070231	T	C	intergenic	RP11-339B21	dist=1602;dis	.	.	A	G	-		0.18518519	22,0,5,0	chr9,131070	Cytosol	A > G
chr9:131070337:T:C:intergenic:RP	chr9	131070337	131070337	T	C	intergenic	RP11-339B21	dist=1708;dis	.	.	A	G	-		0.1	18,0,2,0	chr9,131070	Cytosol	A > G
chr9:131070343:T:C:intergenic:RP	chr9	131070343	131070343	T	C	intergenic	RP11-339B21	dist=1714;dis	.	.	A	G	-		0.75	5,0,15,0	chr9,131070	Cytosol	A > G
chr9:131070352:T:C:intergenic:RP	chr9	131070352	131070352	T	C	intergenic	RP11-339B21	dist=1723;dis	.	.	A	G	-		0.23076923	20,0,6,0	chr9,131070	Cytosol	A > G
chr9:131070433:T:C:downstream:	chr9	131070433	131070433	T	C	downstream	TRUB2	.	.	.	A	G	-		0.43333333	17,0,13,0	chr9,131070	Cytosol	A > G
chr9:131070457:T:C:downstream:	chr9	131070457	131070457	T	C	downstream	TRUB2	.	.	.	A	G	-		0.15625	27,0,5,0	chr9,131070	Cytosol	A > G
chr9:131070487:T:C:downstream:	chr9	131070487	131070487	T	C	downstream	TRUB2	.	.	.	A	G	-		0.37037037	17,0,10,0	chr9,131070	Cytosol	A > G
chr9:131070554:T:C:downstream:	chr9	131070554	131070554	T	C	downstream	TRUB2	.	.	.	A	G	-		0.11111111	16,0,2,0	chr9,131070	Cytosol	A > G
chr9:131070556:T:C:downstream:	chr9	131070556	131070556	T	C	downstream	TRUB2	.	.	.	A	G	-		0.68421053	6,0,13,0	chr9,131070	Cytosol	A > G
chr9:131070565:T:C:downstream:	chr9	131070565	131070565	T	C	downstream	TRUB2	.	.	.	A	G	-		0.6	8,0,12,0	chr9,131070	Cytosol	A > G
chr9:131070568:T:C:downstream:	chr9	131070568	131070568	T	C	downstream	TRUB2	.	.	.	A	G	-		0.66666667	6,0,12,0	chr9,131070	Cytosol	A > G
chr9:131070570:T:C:downstream:	chr9	131070570	131070570	T	C	downstream	TRUB2	.	.	.	A	G	-		0.29411765	12,0,5,0	chr9,131070	Cytosol	A > G
chr9:131070608:T:C:downstream:	chr9	131070608	131070608	T	C	downstream	TRUB2	.	.	.	A	G	-		0.42105263	11,0,8,0	chr9,131070	Cytosol	A > G
chr9:131070623:T:C:downstream:	chr9	131070623	131070623	T	C	downstream	TRUB2	.	.	.	A	G	-		0.69230769	4,0,9,0	chr9,131070	Cytosol	A > G
chr9:131070645:T:C:downstream:	chr9	131070645	131070645	T	C	downstream	TRUB2	.	.	.	A	G	-		0.45	11,0,9,0	chr9,131070	Cytosol	A > G
chr9:131070648:T:C:downstream:	chr9	131070648	131070648	T	C	downstream	TRUB2	.	.	.	A	G	-		0.57692308	11,0,15,0	chr9,131070	Cytosol	A > G
chr9:131070669:T:C:downstream:	chr9	131070669	131070669	T	C	downstream	TRUB2	.	.	.	A	G	-		0.4	15,0,10,0	chr9,131070	Cytosol	A > G
chr9:131070737:T:C:downstream:	chr9	131070737	131070737	T	C	downstream	TRUB2	.	.	.	A	G	-		0.3	14,0,6,0	chr9,131070	Cytosol	A > G
chr9:131071533:T:C:UTR3:TRUB2	chr9	131071533	131071533	T	C	UTR3	TRUB2	ENST000003	.	.	A	G	-	-	0.12254902	179,0,25,0	chr9,131071	Cytosol	A > G
chr9:131071538:T:C:UTR3:TRUB2	chr9	131071538	131071538	T	C	UTR3	TRUB2	ENST000003	.	.	A	G	-	-	0.01129944	175,0,2,0	chr9,131071	Cytosol	A > G
chr9:131071578:T:C:UTR3:TRUB2	chr9	131071578	131071578	T	C	UTR3	TRUB2	ENST000003	.	.	A	G	-	-	0.01136364	174,0,2,0	chr9,131071	Cytosol	A > G
chr9:131071579:T:C:UTR3:TRUB2	chr9	131071579	131071579	T	C	UTR3	TRUB2	ENST000003	.	.	A	G	-	-	0.01117318	177,0,2,0	chr9,131071	Cytosol	A > G
chr9:131093909:A:G:intronic:COQ	chr9	131093909	131093909	A	G	intronic	COQ4	.	.	.	A	G	+	+	0.57142857	3,0,4,0	chr9,131093	Cytosol	A > G
chr9:131093963:A:G:intronic:COQ	chr9	131093963	131093963	A	G	intronic	COQ4	.	.	.	A	G	+	+	0.2	8,0,2,0	chr9,131093	Cytosol	A > G
chr9:131093965:A:G:intronic:COQ	chr9	131093965	131093965	A	G	intronic	COQ4	.	.	.	A	G	+	+	0.66666667	3,0,6,0	chr9,131093	Cytosol	A > G
chr9:131093969:A:G:intronic:COQ	chr9	131093969	131093969	A	G	intronic	COQ4	.	.	.	A	G	+	+	0.4	6,0,4,0	chr9,131093	Cytosol	A > G
chr9:131279291:A:G:intronic:GLE1	chr9	131279291	131279291	A	G	intronic	GLE1	.	.	.	A	G	+	+	0.33333333	4,0,2,0	chr9,131279	Cytosol	A > G
chr9:131279501:A:G:intronic:GLE1	chr9	131279501	131279501	A	G	intronic	GLE1	.	.	.	A	G	+	+	1	0,0,5,0	chr9,131279	Cytosol	A > G
chr9:132266421:A:G:ncRNA_exoni	chr9	132266421	132266421	A	G	ncRNA_exon	LINC00963	.	.	.	A	G	+	+	0.17647059	28,0,6,0	chr9,132266	Cytosol	A > G
chr9:132266582:A:G:ncRNA_exoni	chr9	132266582	132266582	A	G	ncRNA_exon	LINC00963	.	.	.	A	G	+	+	0.66666667	2,0,4,0	chr9,132266	Cytosol	A > G
chr9:132266583:A:G:ncRNA_exoni	chr9	132266583	132266583	A	G	ncRNA_exon	LINC00963	.	.	.	A	G	+	+	0.71428571	2,0,5,0	chr9,132266	Cytosol	A > G
chr9:132266655:A:G:ncRNA_exoni	chr9	132266655	132266655	A	G	ncRNA_exon	LINC00963	.	.	.	A	G	+	+	0.07142857	26,0,2,0	chr9,132266	Cytosol	A > G
chr9:132266666:A:G:ncRNA_exoni	chr9	132266666	132266666	A	G	ncRNA_exon	LINC00963	.	.	.	A	G	+	+	0.23076923	30,0,9,0	chr9,132266	Cytosol	A > G
chr9:132268957:A:G:ncRNA_exoni	chr9	132268957	132268957	A	G	ncRNA_exon	LINC00963	.	.	.	A	G	+	+	0.4	9,0,6,0	chr9,132268	Cytosol	A > G
chr9:132268982:A:G:ncRNA_exoni	chr9	132268982	132268982	A	G	ncRNA_exon	LINC00963	.	.	.	A	G	+	+	0.92307692	1,0,12,0	chr9,132268	Cytosol	A > G
chr9:132268997:A:G:ncRNA_exoni	chr9	132268997	132268997	A	G	ncRNA_exon	LINC00963	.	.	.	A	G	+	+	0.57142857	6,0,8,0	chr9,132268	Cytosol	A > G
chr9:132269037:A:G:ncRNA_exoni	chr9	132269037	132269037	A	G	ncRNA_exon	LINC00963	.	.	.	A	G	+	+	0.125	28,0,4,0	chr9,132269	Cytosol	A > G
chr9:132572026:A:G:UTR3:TOR1B	chr9	132572026	132572026	A	G	UTR3	TOR1B	ENST000002	.	.	A	G	+	+	0.02985075	65,0,2,0	chr9,132572	Cytosol	A > G
chr9:133575535:A:G:intronic:EXOS	chr9	133575535	133575535	A	G	intronic	EXOSC2	.	.	.	A	G	+	+	0.8	1,0,4,0	chr9,133575	Cytosol	A > G
chr9:133579674:A:G:UTR3:EXOSC	chr9	133579674	133579674	A	G	UTR3	EXOSC2	ENST000003	.	.	A	G	+	+	0.0952381	114,0,12,0	chr9,133579	Cytosol	A > G
chr9:133579734:A:G:UTR3:EXOSC	chr9	133579734	133579734	A	G	UTR3	EXOSC2	ENST000003	.	.	A	G	+	+	0.13414634	71,0,11,0	chr9,133579	Cytosol	A > G
chr9:133579756:A:G:UTR3:EXOSC	chr9	133579756	133579756	A	G	UTR3	EXOSC2	ENST000003	.	.	A	G	+	+	0.05714286	33,0,2,0	chr9,133579	Cytosol	A > G
chr9:133579775:A:G:UTR3:EXOSC	chr9	133579775	133579775	A	G	UTR3	EXOSC2	ENST000003	.	.	A	G	+	+	0.10526316	34,0,4,0	chr9,133579	Cytosol	A > G
chr9:133579797:A:G:UTR3:EXOSC	chr9	133579797	133579797	A	G	UTR3	EXOSC2	ENST000003	.	.	A	G	+	+	0.19791667	77,0,19,0	chr9,133579	Cytosol	A > G
chr9:133579833:A:G:UTR3:EXOSC	chr9	133579833	133579833	A	G	UTR3	EXOSC2	ENST000003	.	.	A	G	+	+	0.1958042	115,0,28,0	chr9,133579	Cytosol	A > G

chr9:133579839:A:G:UTR3:EXOSC	chr9	133579839	133579839	A	G	UTR3	EXOSC2	ENST000003	.	.	A	G	+	+	0.05	171,0,9,0	chr9,133579	Cytosol	A > G
chr9:133580397:A:G:downstream:	chr9	133580397	133580397	A	G	downstream	EXOSC2	.	.	.	A	G	+	+	0.19402985	54,0,13,0	chr9,133580	Cytosol	A > G
chr9:133580406:A:G:downstream:	chr9	133580406	133580406	A	G	downstream	EXOSC2	.	.	.	A	G	+	+	0.07352941	63,0,5,0	chr9,133580	Cytosol	A > G
chr9:133580425:A:G:downstream:	chr9	133580425	133580425	A	G	downstream	EXOSC2	.	.	.	A	G	+	+	0.03448276	56,0,2,0	chr9,133580	Cytosol	A > G
chr9:133580451:A:G:downstream:	chr9	133580451	133580451	A	G	downstream	EXOSC2	.	.	.	A	G	+	+	0.44067797	33,0,26,0	chr9,133580	Cytosol	A > G
chr9:133580454:A:G:downstream:	chr9	133580454	133580454	A	G	downstream	EXOSC2	.	.	.	A	G	+		0.38888889	33,0,21,0	chr9,133580	Cytosol	A > G
chr9:133580460:A:G:downstream:	chr9	133580460	133580460	A	G	downstream	EXOSC2	.	.	.	A	G	+		0.4	33,0,22,0	chr9,133580	Cytosol	A > G
chr9:133580498:A:G:downstream:	chr9	133580498	133580498	A	G	downstream	EXOSC2	.	.	.	A	G	+		0.07462687	62,0,5,0	chr9,133580	Cytosol	A > G
chr9:133580502:A:G:downstream:	chr9	133580502	133580502	A	G	downstream	EXOSC2	.	.	.	A	G	+		0.58730159	26,0,37,0	chr9,133580	Cytosol	A > G
chr9:133581468:A:G:intergenic:EX	chr9	133581468	133581468	A	G	intergenic	EXOSC2,ABL1	dist=1220;dis	.	.	A	G	+		0.41176471	20,0,14,0	chr9,133581	Cytosol	A > G
chr9:133582127:A:G:intergenic:EX	chr9	133582127	133582127	A	G	intergenic	EXOSC2,ABL1	dist=1879;dis	.	.	A	G	+		0.10714286	25,0,3,0	chr9,133582	Cytosol	A > G
chr9:139695975:T:C:ncRNA_intror	chr9	139695975	139695975	T	C	ncRNA_intro	RP11-216L13	.	.	.	A	G	-	+	0.85714286	1,0,6,0	chr9,139695	Cytosol	A > G
chr9:139696036:T:C:ncRNA_intror	chr9	139696036	139696036	T	C	ncRNA_intro	RP11-216L13	.	.	.	A	G	-	+	0.25	6,0,2,0	chr9,139696	Cytosol	A > G
chr9:139696076:T:C:ncRNA_intror	chr9	139696076	139696076	T	C	ncRNA_intro	RP11-216L13	.	.	.	A	G	-	+	0.72727273	3,0,8,0	chr9,139696	Cytosol	A > G
chr9:139696377:T:C:ncRNA_intror	chr9	139696377	139696377	T	C	ncRNA_intro	RP11-216L13	.	.	.	A	G	-	+	0.58333333	5,0,7,0	chr9,139696	Cytosol	A > G
chr9:139696378:T:C:ncRNA_intror	chr9	139696378	139696378	T	C	ncRNA_intro	RP11-216L13	.	.	.	A	G	-	+	0.75	3,0,9,0	chr9,139696	Cytosol	A > G
chr9:139696391:T:C:ncRNA_intror	chr9	139696391	139696391	T	C	ncRNA_intro	RP11-216L13	.	.	.	A	G	-	+	0.88888889	1,0,8,0	chr9,139696	Cytosol	A > G
chr9:139696442:T:C:ncRNA_intror	chr9	139696442	139696442	T	C	ncRNA_intro	RP11-216L13	.	.	.	A	G	-	+	0.84615385	2,0,11,0	chr9,139696	Cytosol	A > G
chr9:139696513:T:C:ncRNA_intror	chr9	139696513	139696513	T	C	ncRNA_intro	RP11-216L13	.	.	.	A	G	-	+	0.36363636	14,0,8,0	chr9,139696	Cytosol	A > G
chrX:2824239:T:C:UTR3:ARSD	chrX	2824239	2824239	T	C	UTR3	ARSD	ENST000003	.	.	A	G	-	-	0.25	6,0,2,0	chrX,282423	Cytosol	A > G
chrX:2824246:T:C:UTR3:ARSD	chrX	2824246	2824246	T	C	UTR3	ARSD	ENST000003	.	.	A	G	-	-	0.33333333	6,0,3,0	chrX,282424	Cytosol	A > G
chrX:2824254:T:C:UTR3:ARSD	chrX	2824254	2824254	T	C	UTR3	ARSD	ENST000003	.	.	A	G	-	-	0.2	8,0,2,0	chrX,282425	Cytosol	A > G
chrX:2824311:T:C:UTR3:ARSD	chrX	2824311	2824311	T	C	UTR3	ARSD	ENST000003	.	.	A	G	-	-	0.15384615	11,0,2,0	chrX,282431	Cytosol	A > G
chrX:2824317:T:C:UTR3:ARSD	chrX	2824317	2824317	T	C	UTR3	ARSD	ENST000003	.	.	A	G	-	-	0.75	3,0,9,0	chrX,282431	Cytosol	A > G
chrX:2824343:T:C:UTR3:ARSD	chrX	2824343	2824343	T	C	UTR3	ARSD	ENST000003	.	.	A	G	-	-	0.375	5,0,3,0	chrX,282434	Cytosol	A > G
chrX:2824377:T:C:UTR3:ARSD	chrX	2824377	2824377	T	C	UTR3	ARSD	ENST000003	.	.	A	G	-	-	0.16666667	10,0,2,0	chrX,282437	Cytosol	A > G
chrX:2824385:T:C:UTR3:ARSD	chrX	2824385	2824385	T	C	UTR3	ARSD	ENST000003	.	.	A	G	-	-	0.5	6,0,6,0	chrX,282438	Cytosol	A > G
chrX:2824433:T:C:UTR3:ARSD	chrX	2824433	2824433	T	C	UTR3	ARSD	ENST000003	.	.	A	G	-	-	1	0,0,7,0	chrX,282443	Cytosol	A > G
chrX:2824434:T:C:UTR3:ARSD	chrX	2824434	2824434	T	C	UTR3	ARSD	ENST000003	.	.	A	G	-	-	1	0,0,8,0	chrX,282443	Cytosol	A > G
chrX:2824916:T:C:UTR3:ARSD	chrX	2824916	2824916	T	C	UTR3	ARSD	ENST000003	.	.	A	G	-	-	0.85714286	1,0,6,0	chrX,282491	Cytosol	A > G
chrX:2824979:T:C:UTR3:ARSD	chrX	2824979	2824979	T	C	UTR3	ARSD	ENST000003	.	.	A	G	-	-	0.22222222	7,0,2,0	chrX,282497	Cytosol	A > G
chrX:2824985:T:C:UTR3:ARSD	chrX	2824985	2824985	T	C	UTR3	ARSD	ENST000003	.	.	A	G	-	-	1	0,0,8,0	chrX,282498	Cytosol	A > G
chrX:2824988:T:C:UTR3:ARSD	chrX	2824988	2824988	T	C	UTR3	ARSD	ENST000003	.	.	A	G	-	-	0.90909091	1,0,10,0	chrX,282498	Cytosol	A > G
chrX:2825075:T:C:UTR3:ARSD	chrX	2825075	2825075	T	C	UTR3	ARSD	ENST000003	.	.	A	G	-	-	0.69230769	4,0,9,0	chrX,282507	Cytosol	A > G
chrX:3527770:T:C:intronic:PRKX	chrX	3527770	3527770	T	C	intronic	PRKX	.	.	.	A	G	-	-	0.4	3,0,2,0	chrX,352776	Cytosol	A > G
chrX:3528517:T:C:intronic:PRKX	chrX	3528517	3528517	T	C	intronic	PRKX	.	.	.	A	G	-	-	0.08823529	31,0,3,0	chrX,352851	Cytosol	A > G
chrX:3744704:T:C:intronic:RP11-7	chrX	3744704	3744704	T	C	intronic	RP11-706O1	.	.	.	A	G	-	-	0.11111111	16,0,2,0	chrX,374470	Cytosol	A > G
chrX:3744720:T:C:intronic:RP11-7	chrX	3744720	3744720	T	C	intronic	RP11-706O1	.	.	.	A	G	-	-	0.46666667	8,0,7,0	chrX,374471	Cytosol	A > G
chrX:15801739:A:G:UTR3:CA5B	chrX	15801739	15801739	A	G	UTR3	CA5B	ENST000003	.	.	A	G	+	+	0.33333333	4,0,2,0	chrX,158017	Cytosol	A > G
chrX:15801750:A:G:UTR3:CA5B	chrX	15801750	15801750	A	G	UTR3	CA5B	ENST000003	.	.	A	G	+	+	0.75	2,0,6,0	chrX,158017	Cytosol	A > G
chrX:15801759:A:G:UTR3:CA5B	chrX	15801759	15801759	A	G	UTR3	CA5B	ENST000003	.	.	A	G	+	+	0.625	3,0,5,0	chrX,158017	Cytosol	A > G
chrX:16779094:A:G:UTR3:SYAP1	chrX	16779094	16779094	A	G	UTR3	SYAP1	ENST000003	.	.	A	G	+	+	0.01777778	221,0,4,0	chrX,167790	Cytosol	A > G
chrX:16779266:A:G:UTR3:SYAP1	chrX	16779266	16779266	A	G	UTR3	SYAP1	ENST000003	.	.	A	G	+	+	0.04950495	96,0,5,0	chrX,167792	Cytosol	A > G
chrX:16779284:A:G:UTR3:SYAP1	chrX	16779284	16779284	A	G	UTR3	SYAP1	ENST000003	.	.	A	G	+	+	0.02941176	99,0,3,0	chrX,167792	Cytosol	A > G
chrX:16781149:A:G:UTR3:SYAP1	chrX	16781149	16781149	A	G	UTR3	SYAP1	ENST000003	.	.	A	G	+	+	0.77777778	6,0,21,0	chrX,167811	Cytosol	A > G
chrX:16781155:A:G:UTR3:SYAP1	chrX	16781155	16781155	A	G	UTR3	SYAP1	ENST000003	.	.	A	G	+	+	0.37037037	17,0,10,0	chrX,167811	Cytosol	A > G
chrX:16781156:A:G:UTR3:SYAP1	chrX	16781156	16781156	A	G	UTR3	SYAP1	ENST000003	.	.	A	G	+	+	0.23076923	20,0,6,0	chrX,167811	Cytosol	A > G
chrX:16781201:A:G:UTR3:SYAP1	chrX	16781201	16781201	A	G	UTR3	SYAP1	ENST000003	.	.	A	G	+	+	0.42307692	15,0,11,0	chrX,167812	Cytosol	A > G
chrX:16781275:A:G:UTR3:SYAP1	chrX	16781275	16781275	A	G	UTR3	SYAP1	ENST000003	.	.	A	G	+	+	0.84482759	9,0,49,0	chrX,167812	Cytosol	A > G
chrX:16781311:A:G:UTR3:SYAP1	chrX	16781311	16781311	A	G	UTR3	SYAP1	ENST000003	.	.	A	G	+	+	0.2826087	33,0,13,0	chrX,167813	Cytosol	A > G
chrX:16781315:A:G:UTR3:SYAP1	chrX	16781315	16781315	A	G	UTR3	SYAP1	ENST000003	.	.	A	G	+	+	0.19047619	34,0,8,0	chrX,167813	Cytosol	A > G
chrX:16781358:A:G:UTR3:SYAP1	chrX	16781358	16781358	A	G	UTR3	SYAP1	ENST000003	.	.	A	G	+	+	0.05084746	56,0,3,0	chrX,167813	Cytosol	A > G
chrX:16781364:A:G:UTR3:SYAP1	chrX	16781364	16781364	A	G	UTR3	SYAP1	ENST000003	.	.	A	G	+	+	0.05172414	55,0,3,0	chrX,167813	Cytosol	A > G
chrX:16781502:A:G:UTR3:SYAP1	chrX	16781502	16781502	A	G	UTR3	SYAP1	ENST000003	.	.	A	G	+	+	0.30952381	29,0,13,0	chrX,167815	Cytosol	A > G
chrX:16781576:A:G:UTR3:SYAP1	chrX	16781576	16781576	A	G	UTR3	SYAP1	ENST000003	.	.	A	G	+	+	0.3	21,0,9,0	chrX,167815	Cytosol	A > G
chrX:16781615:A:G:UTR3:SYAP1	chrX	16781615	16781615	A	G	UTR3	SYAP1	ENST000003	.	.	A	G	+	+	0.32	17,0,8,0	chrX,167816	Cytosol	A > G
chrX:16781658:A:G:UTR3:SYAP1	chrX	16781658	16781658	A	G	UTR3	SYAP1	ENST000003	.	.	A	G	+	+	0.07692308	36,0,3,0	chrX,167816	Cytosol	A > G
chrX:16781687:A:G:UTR3:SYAP1	chrX	16781687	16781687	A	G	UTR3	SYAP1	ENST000003	.	.	A	G	+	+	0.4516129	17,0,14,0	chrX,167816	Cytosol	A > G
chrX:16781698:A:G:UTR3:SYAP1	chrX	16781698	16781698	A	G	UTR3	SYAP1	ENST000003	.	.	A	G	+	+	0.30555556	23,0,11,2	chrX,167816	Cytosol	A > G
chrX:16781739:A:G:UTR3:SYAP1	chrX	16781739	16781739	A	G	UTR3	SYAP1	ENST000003	.	.	A	G	+	+	0.19512195	33,0,8,0	chrX,167817	Cytosol	A > G
chrX:16781740:A:G:UTR3:SYAP1	chrX	16781740	16781740	A	G	UTR3	SYAP1	ENST000003	.	.	A	G	+	+	0.05555556	34,0,2,0	chrX,167817	Cytosol	A > G
chrX:16781752:A:G:UTR3:SYAP1	chrX	16781752	16781752	A	G	UTR3	SYAP1	ENST000003	.	.	A	G	+	+	0.13333333	39,0,6,0	chrX,167817	Cytosol	A > G
chrX:16781758:A:G:UTR3:SYAP1	chrX	16781758	16781758	A	G	UTR3	SYAP1	ENST000003	.	.	A	G	+	+	0.28888889	32,0,13,0	chrX,167817	Cytosol	A > G
chrX:16782048:A:G:UTR3:SYAP1	chrX	16782048	16782048	A	G	UTR3	SYAP1	ENST000003	.	.	A	G	+	+	0.5	4,0,4,0	chrX,167820	Cytosol	A > G

chrX:16782053:A:G:UTR3:SYAP1	chrX	16782053	16782053	A	G	UTR3	SYAP1	ENST000003	.	.	A	G	+	+	0.53333333	7,0,8,0	chrX,167820	Cytosol	A > G
chrX:16782164:A:G:UTR3:SYAP1	chrX	16782164	16782164	A	G	UTR3	SYAP1	ENST000003	.	.	A	G	+	+	0.25	21,0,7,0	chrX,167821	Cytosol	A > G
chrX:16782172:A:G:UTR3:SYAP1	chrX	16782172	16782172	A	G	UTR3	SYAP1	ENST000003	.	.	A	G	+	+	0.51612903	15,0,16,0	chrX,167821	Cytosol	A > G
chrX:16782259:A:G:UTR3:SYAP1	chrX	16782259	16782259	A	G	UTR3	SYAP1	ENST000003	.	.	A	G	+	+	0.42857143	8,0,6,0	chrX,167822	Cytosol	A > G
chrX:16782272:A:G:UTR3:SYAP1	chrX	16782272	16782272	A	G	UTR3	SYAP1	ENST000003	.	.	A	G	+	+	0.20833333	19,0,5,0	chrX,167822	Cytosol	A > G
chrX:16782866:A:G:UTR3:SYAP1	chrX	16782866	16782866	A	G	UTR3	SYAP1	ENST000003	.	.	A	G	+	+	0.32	17,0,8,0	chrX,167828	Cytosol	A > G
chrX:16782887:A:G:UTR3:SYAP1	chrX	16782887	16782887	A	G	UTR3	SYAP1	ENST000003	.	.	A	G	+	+	0.25	27,0,9,0	chrX,167828	Cytosol	A > G
chrX:16782957:A:G:UTR3:SYAP1	chrX	16782957	16782957	A	G	UTR3	SYAP1	ENST000003	.	.	A	G	+	+	0.14814815	23,0,4,0	chrX,167829	Cytosol	A > G
chrX:16783019:A:G:UTR3:SYAP1	chrX	16783019	16783019	A	G	UTR3	SYAP1	ENST000003	.	.	A	G	+	+	0.06666667	42,0,3,0	chrX,167830	Cytosol	A > G
chrX:23719788:T:C:downstream:A	chrX	23719788	23719788	T	C	downstream	ACOT9	.	.	.	A	G	-		0.51612903	15,0,16,0	chrX,237197	Cytosol	A > G
chrX:23719873:T:C:downstream:A	chrX	23719873	23719873	T	C	downstream	ACOT9	.	.	.	A	G	-		0.28571429	10,0,4,0	chrX,237198	Cytosol	A > G
chrX:23720654:T:C:UTR3:ACOT9	chrX	23720654	23720654	T	C	UTR3	ACOT9	ENST000003	.	.	A	G	-	-	0.23809524	16,0,5,0	chrX,237206	Cytosol	A > G
chrX:23721532:T:C:UTR3:ACOT9	chrX	23721532	23721532	T	C	UTR3	ACOT9	ENST000003	.	.	A	G	-	-	0.4	6,0,4,0	chrX,237215	Cytosol	A > G
chrX:23721536:T:C:UTR3:ACOT9	chrX	23721536	23721536	T	C	UTR3	ACOT9	ENST000003	.	.	A	G	-	-	0.30769231	9,0,4,0	chrX,237215	Cytosol	A > G
chrX:23721572:T:C:UTR3:ACOT9	chrX	23721572	23721572	T	C	UTR3	ACOT9	ENST000003	.	.	A	G	-	-	0.21428571	11,0,3,0	chrX,237215	Cytosol	A > G
chrX:23721616:T:C:UTR3:ACOT9	chrX	23721616	23721616	T	C	UTR3	ACOT9	ENST000003	.	.	A	G	-	-	0.14285714	18,0,3,0	chrX,237216	Cytosol	A > G
chrX:24093954:A:G:intronic:EIF2S	chrX	24093954	24093954	A	G	intronic	EIF2S3	.	.	.	A	G	+	+	0.18181818	9,0,2,0	chrX,240939	Cytosol	A > G
chrX:24094073:A:G:intronic:EIF2S	chrX	24094073	24094073	A	G	intronic	EIF2S3	.	.	.	A	G	+	+	0.1875	13,0,3,0	chrX,240940	Cytosol	A > G
chrX:24094467:A:G:intronic:EIF2S	chrX	24094467	24094467	A	G	intronic	EIF2S3	.	.	.	A	G	+	+	0.23529412	13,0,4,0	chrX,240944	Cytosol	A > G
chrX:24095285:A:G:UTR3:EIF2S3	chrX	24095285	24095285	A	G	UTR3	EIF2S3	ENST000002	.	.	A	G	+	+	0.0347541	1472,0,53,0	chrX,240952	Cytosol	A > G
chrX:24095286:A:G:UTR3:EIF2S3	chrX	24095286	24095286	A	G	UTR3	EIF2S3	ENST000002	.	.	A	G	+	+	0.0509165	1398,0,75,0	chrX,240952	Cytosol	A > G
chrX:24095320:A:G:UTR3:EIF2S3	chrX	24095320	24095320	A	G	UTR3	EIF2S3	ENST000002	.	.	A	G	+	+	0.02042901	959,0,20,0	chrX,240953	Cytosol	A > G
chrX:24095325:A:G:UTR3:EIF2S3	chrX	24095325	24095325	A	G	UTR3	EIF2S3	ENST000002	.	.	A	G	+	+	0.04545455	986,0,47,1	chrX,240953	Cytosol	A > G
chrX:24095383:A:G:UTR3:EIF2S3	chrX	24095383	24095383	A	G	UTR3	EIF2S3	ENST000002	.	.	A	G	+	+	0.02296296	1319,0,31,0	chrX,240953	Cytosol	A > G
chrX:30854777:T:C:UTR3:TAB3	chrX	30854777	30854777	T	C	UTR3	TAB3	ENST000004	.	.	A	G	-	-	0.55555556	4,0,5,0	chrX,308547	Cytosol	A > G
chrX:30854836:T:C:UTR3:TAB3	chrX	30854836	30854836	T	C	UTR3	TAB3	ENST000004	.	.	A	G	-	-	0.6	2,0,3,0	chrX,308548	Cytosol	A > G
chrX:30854878:T:C:UTR3:TAB3	chrX	30854878	30854878	T	C	UTR3	TAB3	ENST000004	.	.	A	G	-	-	0.28571429	5,0,2,0	chrX,308548	Cytosol	A > G
chrX:46333208:A:G:UTR3:KRBOX4	chrX	46333208	46333208	A	G	UTR3	KRBOX4	ENST000003	.	.	A	G	+	+	0.05714286	66,0,4,0	chrX,463332	Cytosol	A > G
chrX:46333219:A:G:UTR3:KRBOX4	chrX	46333219	46333219	A	G	UTR3	KRBOX4	ENST000003	.	.	A	G	+	+	0.02631579	74,0,2,0	chrX,463332	Cytosol	A > G
chrX:46358822:T:C:UTR3:ZNF674	chrX	46358822	46358822	T	C	UTR3	ZNF674	ENST000005	.	.	A	G	-	-	0.4	3,0,2,0	chrX,463588	Cytosol	A > G
chrX:46407512:A:G:ncRNA_exonic	chrX	46407512	46407512	A	G	ncRNA_exon	ZNF674-AS1	.	.	.	A	G	+	+	0.05405405	35,0,2,0	chrX,464075	Cytosol	A > G
chrX:46463205:T:C:intergenic:CHS	chrX	46463205	46463205	T	C	intergenic	CHST7,SLC9A	dist=5362;di	.	.	A	G	-	-	0.38888889	11,0,7,0	chrX,464632	Cytosol	A > G
chrX:46463953:T:C:downstream:S	chrX	46463953	46463953	T	C	downstream	SLC9A7	.	.	.	A	G	-	-	0.89473684	2,0,17,0	chrX,464639	Cytosol	A > G
chrX:46464006:T:C:downstream:S	chrX	46464006	46464006	T	C	downstream	SLC9A7	.	.	.	A	G	-	-	0.33333333	18,0,9,0	chrX,464640	Cytosol	A > G
chrX:46464041:T:C:downstream:S	chrX	46464041	46464041	T	C	downstream	SLC9A7	.	.	.	A	G	-	-	0.38709677	19,0,12,0	chrX,464640	Cytosol	A > G
chrX:46464049:T:C:downstream:S	chrX	46464049	46464049	T	C	downstream	SLC9A7	.	.	.	A	G	-	-	0.2	23,0,6,1	chrX,464640	Cytosol	A > G
chrX:46464504:T:C:downstream:S	chrX	46464504	46464504	T	C	downstream	SLC9A7	.	.	.	A	G	-	-	0.22222222	7,0,2,0	chrX,464645	Cytosol	A > G
chrX:46465334:T:C:UTR3:SLC9A7	chrX	46465334	46465334	T	C	UTR3	SLC9A7	ENST000003	.	.	A	G	-	-	0.16666667	20,0,4,0	chrX,464653	Cytosol	A > G
chrX:46465423:T:C:UTR3:SLC9A7	chrX	46465423	46465423	T	C	UTR3	SLC9A7	ENST000003	.	.	A	G	-	-	0.13333333	13,0,2,0	chrX,464654	Cytosol	A > G
chrX:47515642:T:C:intronic:UXT	chrX	47515642	47515642	T	C	intronic	UXT	.	.	.	A	G	-	-	0.16666667	10,0,2,0	chrX,475156	Cytosol	A > G
chrX:47515668:T:C:intronic:UXT	chrX	47515668	47515668	T	C	intronic	UXT	.	.	.	A	G	-	-	0.38461538	8,0,5,0	chrX,475156	Cytosol	A > G
chrX:48341920:A:G:intronic:FTSJ1	chrX	48341920	48341920	A	G	intronic	FTSJ1	.	.	.	A	G	+	+	0.75	2,0,6,0	chrX,483419	Cytosol	A > G
chrX:48341927:A:G:intronic:FTSJ1	chrX	48341927	48341927	A	G	intronic	FTSJ1	.	.	.	A	G	+	+	0.28571429	5,0,2,0	chrX,483419	Cytosol	A > G
chrX:48342940:A:G:intronic:FTSJ1	chrX	48342940	48342940	A	G	intronic	FTSJ1	.	.	.	A	G	+	+	0.9	1,0,9,0	chrX,483429	Cytosol	A > G
chrX:48342958:A:G:intronic:FTSJ1	chrX	48342958	48342958	A	G	intronic	FTSJ1	.	.	.	A	G	+	+	0.61538462	5,0,8,0	chrX,483429	Cytosol	A > G
chrX:48342972:A:G:intronic:FTSJ1	chrX	48342972	48342972	A	G	intronic	FTSJ1	.	.	.	A	G	+	+	0.2	8,0,2,0	chrX,483429	Cytosol	A > G
chrX:48436322:A:G:UTR3:RBM3	chrX	48436322	48436322	A	G	UTR3	RBM3	ENST000003	.	.	A	G	+	+	0.01755669	1343,0,24,0	chrX,484363	Cytosol	A > G
chrX:48436348:A:G:UTR3:RBM3	chrX	48436348	48436348	A	G	UTR3	RBM3	ENST000003	.	.	A	G	+	+	0.01957295	1101,1,22,0	chrX,484363	Cytosol	A > G
chrX:48436762:A:G:UTR3:RBM3	chrX	48436762	48436762	A	G	UTR3	RBM3	ENST000003	.	.	A	G	+	+	0.6875	5,0,11,0	chrX,484367	Cytosol	A > G
chrX:48436805:A:G:UTR3:RBM3	chrX	48436805	48436805	A	G	UTR3	RBM3	ENST000003	.	.	A	G	+	+	0.70588235	5,0,12,0	chrX,484368	Cytosol	A > G
chrX:48436808:A:G:UTR3:RBM3	chrX	48436808	48436808	A	G	UTR3	RBM3	ENST000003	.	.	A	G	+	+	0.69230769	4,0,9,0	chrX,484368	Cytosol	A > G
chrX:48436809:A:G:UTR3:RBM3	chrX	48436809	48436809	A	G	UTR3	RBM3	ENST000003	.	.	A	G	+	+	0.28571429	10,0,4,0	chrX,484368	Cytosol	A > G
chrX:48436810:A:G:UTR3:RBM3	chrX	48436810	48436810	A	G	UTR3	RBM3	ENST000003	.	.	A	G	+	+	0.66666667	5,0,10,0	chrX,484368	Cytosol	A > G
chrX:48436814:A:G:UTR3:RBM3	chrX	48436814	48436814	A	G	UTR3	RBM3	ENST000003	.	.	A	G	+	+	0.30769231	9,0,4,0	chrX,484368	Cytosol	A > G
chrX:48436896:A:G:UTR3:RBM3	chrX	48436896	48436896	A	G	UTR3	RBM3	ENST000003	.	.	A	G	+	+	0.88888889	1,0,8,0	chrX,484368	Cytosol	A > G
chrX:48436936:A:G:UTR3:RBM3	chrX	48436936	48436936	A	G	UTR3	RBM3	ENST000003	.	.	A	G	+	+	0.25	6,0,2,0	chrX,484369	Cytosol	A > G
chrX:48438849:A:G:ncRNA_intron	chrX	48438849	48438849	A	G	ncRNA_intro	RP11-1148L	.	.	.	A	G	+	+-	0.76470588	4,0,13,0	chrX,484388	Cytosol	A > G
chrX:48438859:A:G:ncRNA_intron	chrX	48438859	48438859	A	G	ncRNA_intro	RP11-1148L	.	.	.	A	G	+	+-	0.5	6,0,6,0	chrX,484388	Cytosol	A > G
chrX:48438885:A:G:ncRNA_intron	chrX	48438885	48438885	A	G	ncRNA_intro	RP11-1148L	.	.	.	A	G	+	+-	0.625	3,0,5,0	chrX,484388	Cytosol	A > G
chrX:48438914:A:G:ncRNA_intron	chrX	48438914	48438914	A	G	ncRNA_intro	RP11-1148L	.	.	.	A	G	+	+-	0.42857143	4,0,3,0	chrX,484389	Cytosol	A > G
chrX:48438924:A:G:ncRNA_intron	chrX	48438924	48438924	A	G	ncRNA_intro	RP11-1148L	.	.	.	A	G	+	+-	1	0,0,7,0	chrX,484389	Cytosol	A > G
chrX:48438962:A:G:ncRNA_intron	chrX	48438962	48438962	A	G	ncRNA_intro	RP11-1148L	.	.	.	A	G	+	+-	0.16666667	20,0,4,0	chrX,484389	Cytosol	A > G
chrX:48438971:A:G:ncRNA_intron	chrX	48438971	48438971	A	G	ncRNA_intro	RP11-1148L	.	.	.	A	G	+	+-	0.08	23,0,2,0	chrX,484389	Cytosol	A > G
chrX:48438979:A:G:ncRNA_intron	chrX	48438979	48438979	A	G	ncRNA_intro	RP11-1148L	.	.	.	A	G	+	+-	0.46428571	15,0,13,0	chrX,484389	Cytosol	A > G

chrX:48439017:A:G:ncRNA_introni	chrX	48439017	48439017	A	G	ncRNA_intro	RP11-1148L6	.	.	.	A	G	+	+-	0.10869565	41,0,5,0	chrX,484390	Cytosol	A > G
chrX:48439056:A:G:ncRNA_introni	chrX	48439056	48439056	A	G	ncRNA_intro	RP11-1148L6	.	.	.	A	G	+	+-	0.04081633	47,0,2,0	chrX,484390	Cytosol	A > G
chrX:48893874:T:C:intronic:TFE3	chrX	48893874	48893874	T	C	intronic	TFE3	.	.	.	A	G	-	-	0.28571429	5,0,2,0	chrX,488938	Cytosol	A > G
chrX:48927158:A:G:UTR3:CCDC12	chrX	48927158	48927158	A	G	UTR3	CCDC120	ENST000003	.	.	A	G	+	+	0.06779661	55,0,4,0	chrX,489271	Cytosol	A > G
chrX:48969680:T:C:downstream:G	chrX	48969680	48969680	T	C	downstream	GPKOW	.	.	.	A	G	-	-	0.30769231	9,0,4,0	chrX,489696	Cytosol	A > G
chrX:54587196:A:G:UTR3:GNL3L	chrX	54587196	54587196	A	G	UTR3	GNL3L	ENST000003	.	.	A	G	+	+	0.63043478	16,0,29,1	chrX,545871	Cytosol	A > G
chrX:54587219:A:G:UTR3:GNL3L	chrX	54587219	54587219	A	G	UTR3	GNL3L	ENST000003	.	.	A	G	+	+	0.75	7,0,21,0	chrX,545872	Cytosol	A > G
chrX:54587224:A:G:UTR3:GNL3L	chrX	54587224	54587224	A	G	UTR3	GNL3L	ENST000003	.	.	A	G	+	+	0.12	22,0,3,0	chrX,545872	Cytosol	A > G
chrX:54587249:A:G:UTR3:GNL3L	chrX	54587249	54587249	A	G	UTR3	GNL3L	ENST000003	.	.	A	G	+	+	0.70833333	7,0,17,0	chrX,545872	Cytosol	A > G
chrX:54587264:A:G:UTR3:GNL3L	chrX	54587264	54587264	A	G	UTR3	GNL3L	ENST000003	.	.	A	G	+	+	0.9	3,0,27,0	chrX,545872	Cytosol	A > G
chrX:54587297:A:G:UTR3:GNL3L	chrX	54587297	54587297	A	G	UTR3	GNL3L	ENST000003	.	.	A	G	+	+	0.27027027	27,0,10,0	chrX,545872	Cytosol	A > G
chrX:54587298:A:G:UTR3:GNL3L	chrX	54587298	54587298	A	G	UTR3	GNL3L	ENST000003	.	.	A	G	+	+	0.44117647	19,0,15,0	chrX,545872	Cytosol	A > G
chrX:54587312:A:G:UTR3:GNL3L	chrX	54587312	54587312	A	G	UTR3	GNL3L	ENST000003	.	.	A	G	+	+	0.05714286	33,0,2,0	chrX,545873	Cytosol	A > G
chrX:54587340:A:G:UTR3:GNL3L	chrX	54587340	54587340	A	G	UTR3	GNL3L	ENST000003	.	.	A	G	+	+	0.17142857	29,0,6,0	chrX,545873	Cytosol	A > G
chrX:54587346:A:G:UTR3:GNL3L	chrX	54587346	54587346	A	G	UTR3	GNL3L	ENST000003	.	.	A	G	+	+	0.05714286	33,0,2,0	chrX,545873	Cytosol	A > G
chrX:54587350:A:G:UTR3:GNL3L	chrX	54587350	54587350	A	G	UTR3	GNL3L	ENST000003	.	.	A	G	+	+	0.56756757	16,0,21,0	chrX,545873	Cytosol	A > G
chrX:54587352:A:G:UTR3:GNL3L	chrX	54587352	54587352	A	G	UTR3	GNL3L	ENST000003	.	.	A	G	+	+	0.44444444	20,0,16,0	chrX,545873	Cytosol	A > G
chrX:54587353:A:G:UTR3:GNL3L	chrX	54587353	54587353	A	G	UTR3	GNL3L	ENST000003	.	.	A	G	+	+	0.60526316	15,0,23,0	chrX,545873	Cytosol	A > G
chrX:54587362:A:G:UTR3:GNL3L	chrX	54587362	54587362	A	G	UTR3	GNL3L	ENST000003	.	.	A	G	+	+	0.56756757	16,0,21,0	chrX,545873	Cytosol	A > G
chrX:54587425:A:G:UTR3:GNL3L	chrX	54587425	54587425	A	G	UTR3	GNL3L	ENST000003	.	.	A	G	+	+	0.56451613	27,0,35,0	chrX,545874	Cytosol	A > G
chrX:54587426:A:G:UTR3:GNL3L	chrX	54587426	54587426	A	G	UTR3	GNL3L	ENST000003	.	.	A	G	+	+	0.33898305	39,0,20,0	chrX,545874	Cytosol	A > G
chrX:54587437:A:G:UTR3:GNL3L	chrX	54587437	54587437	A	G	UTR3	GNL3L	ENST000003	.	.	A	G	+	+	0.25373134	50,0,17,0	chrX,545874	Cytosol	A > G
chrX:54587462:A:G:UTR3:GNL3L	chrX	54587462	54587462	A	G	UTR3	GNL3L	ENST000003	.	.	A	G	+	+	0.02739726	71,0,2,0	chrX,545874	Cytosol	A > G
chrX:54588535:A:G:intergenic:GNI	chrX	54588535	54588535	A	G	intergenic	GNL3L,ITIH6	dist=1031;dis	.	.	A	G	+	+	0.26923077	38,0,14,0	chrX,545885	Cytosol	A > G
chrX:54588543:A:G:intergenic:GNI	chrX	54588543	54588543	A	G	intergenic	GNL3L,ITIH6	dist=1039;dis	.	.	A	G	+	+	0.2	32,0,8,0	chrX,545885	Cytosol	A > G
chrX:54588553:A:G:intergenic:GNI	chrX	54588553	54588553	A	G	intergenic	GNL3L,ITIH6	dist=1049;dis	.	.	A	G	+	+	0.23529412	26,0,8,0	chrX,545885	Cytosol	A > G
chrX:54588578:A:G:intergenic:GNI	chrX	54588578	54588578	A	G	intergenic	GNL3L,ITIH6	dist=1074;dis	.	.	A	G	+	+	0.1627907	36,0,7,0	chrX,545885	Cytosol	A > G
chrX:54588579:A:G:intergenic:GNI	chrX	54588579	54588579	A	G	intergenic	GNL3L,ITIH6	dist=1075;dis	.	.	A	G	+	+	0.65	14,0,26,0	chrX,545885	Cytosol	A > G
chrX:54588605:A:G:intergenic:GNI	chrX	54588605	54588605	A	G	intergenic	GNL3L,ITIH6	dist=1101;dis	.	.	A	G	+	+	0.25925926	20,0,7,0	chrX,545886	Cytosol	A > G
chrX:54588608:A:G:intergenic:GNI	chrX	54588608	54588608	A	G	intergenic	GNL3L,ITIH6	dist=1104;dis	.	.	A	G	+	+	0.23076923	20,0,6,0	chrX,545886	Cytosol	A > G
chrX:54588618:A:G:intergenic:GNI	chrX	54588618	54588618	A	G	intergenic	GNL3L,ITIH6	dist=1114;dis	.	.	A	G	+	+	0.05263158	36,0,2,0	chrX,545886	Cytosol	A > G
chrX:54588644:A:G:intergenic:GNI	chrX	54588644	54588644	A	G	intergenic	GNL3L,ITIH6	dist=1140;dis	.	.	A	G	+	+	0.12820513	34,0,5,0	chrX,545886	Cytosol	A > G
chrX:54588653:A:G:intergenic:GNI	chrX	54588653	54588653	A	G	intergenic	GNL3L,ITIH6	dist=1149;dis	.	.	A	G	+	+	0.07272727	51,0,4,0	chrX,545886	Cytosol	A > G
chrX:54588659:A:G:intergenic:GNI	chrX	54588659	54588659	A	G	intergenic	GNL3L,ITIH6	dist=1155;dis	.	.	A	G	+	+	0.37704918	38,0,23,0	chrX,545886	Cytosol	A > G
chrX:54588662:A:G:intergenic:GNI	chrX	54588662	54588662	A	G	intergenic	GNL3L,ITIH6	dist=1158;dis	.	.	A	G	+	+	0.21311475	48,0,13,0	chrX,545886	Cytosol	A > G
chrX:54588679:A:G:intergenic:GNI	chrX	54588679	54588679	A	G	intergenic	GNL3L,ITIH6	dist=1175;dis	.	.	A	G	+	+	0.06593407	85,0,6,0	chrX,545886	Cytosol	A > G
chrX:54588685:A:G:intergenic:GNI	chrX	54588685	54588685	A	G	intergenic	GNL3L,ITIH6	dist=1181;dis	.	.	A	G	+	+	0.27272727	56,0,21,0	chrX,545886	Cytosol	A > G
chrX:54588688:A:G:intergenic:GNI	chrX	54588688	54588688	A	G	intergenic	GNL3L,ITIH6	dist=1184;dis	.	.	A	G	+	+	0.15384615	66,0,12,0	chrX,545886	Cytosol	A > G
chrX:54588701:A:G:intergenic:GNI	chrX	54588701	54588701	A	G	intergenic	GNL3L,ITIH6	dist=1197;dis	.	.	A	G	+	+	0.11538462	69,0,9,0	chrX,545887	Cytosol	A > G
chrX:54588713:A:G:intergenic:GNI	chrX	54588713	54588713	A	G	intergenic	GNL3L,ITIH6	dist=1209;dis	.	.	A	G	+	+	0.13580247	70,0,11,0	chrX,545887	Cytosol	A > G
chrX:54588714:A:G:intergenic:GNI	chrX	54588714	54588714	A	G	intergenic	GNL3L,ITIH6	dist=1210;dis	.	.	A	G	+	+	0.75342466	18,0,55,0	chrX,545887	Cytosol	A > G
chrX:54588718:A:G:intergenic:GNI	chrX	54588718	54588718	A	G	intergenic	GNL3L,ITIH6	dist=1214;dis	.	.	A	G	+	+	0.13559322	51,0,8,0	chrX,545887	Cytosol	A > G
chrX:54588722:A:G:intergenic:GNI	chrX	54588722	54588722	A	G	intergenic	GNL3L,ITIH6	dist=1218;dis	.	.	A	G	+	+	0.65079365	22,0,41,0	chrX,545887	Cytosol	A > G
chrX:54588739:A:G:intergenic:GNI	chrX	54588739	54588739	A	G	intergenic	GNL3L,ITIH6	dist=1235;dis	.	.	A	G	+	+	0.04878049	39,0,2,0	chrX,545887	Cytosol	A > G
chrX:54588740:A:G:intergenic:GNI	chrX	54588740	54588740	A	G	intergenic	GNL3L,ITIH6	dist=1236;dis	.	.	A	G	+	+	0.28947368	27,0,11,0	chrX,545887	Cytosol	A > G
chrX:54588741:A:G:intergenic:GNI	chrX	54588741	54588741	A	G	intergenic	GNL3L,ITIH6	dist=1237;dis	.	.	A	G	+	+	0.54545455	15,0,18,0	chrX,545887	Cytosol	A > G
chrX:54588762:A:G:intergenic:GNI	chrX	54588762	54588762	A	G	intergenic	GNL3L,ITIH6	dist=1258;dis	.	.	A	G	+	+	0.05952381	79,0,5,0	chrX,545887	Cytosol	A > G
chrX:54589084:A:G:intergenic:GNI	chrX	54589084	54589084	A	G	intergenic	GNL3L,ITIH6	dist=1580;dis	.	.	A	G	+	+	0.13103448	126,0,19,0	chrX,545890	Cytosol	A > G
chrX:54589087:A:G:intergenic:GNI	chrX	54589087	54589087	A	G	intergenic	GNL3L,ITIH6	dist=1583;dis	.	.	A	G	+	+	0.01948052	151,0,3,0	chrX,545890	Cytosol	A > G
chrX:54589099:A:G:intergenic:GNI	chrX	54589099	54589099	A	G	intergenic	GNL3L,ITIH6	dist=1595;dis	.	.	A	G	+	+	0.07194245	129,0,10,0	chrX,545890	Cytosol	A > G
chrX:54589105:A:G:intergenic:GNI	chrX	54589105	54589105	A	G	intergenic	GNL3L,ITIH6	dist=1601;dis	.	.	A	G	+	+	0.04794521	139,0,7,0	chrX,545891	Cytosol	A > G
chrX:54589106:A:G:intergenic:GNI	chrX	54589106	54589106	A	G	intergenic	GNL3L,ITIH6	dist=1602;dis	.	.	A	G	+	+	0.37226277	86,0,51,0	chrX,545891	Cytosol	A > G
chrX:54589119:A:G:intergenic:GNI	chrX	54589119	54589119	A	G	intergenic	GNL3L,ITIH6	dist=1615;dis	.	.	A	G	+	+	0.21428571	88,0,24,0	chrX,545891	Cytosol	A > G
chrX:54589141:A:G:intergenic:GNI	chrX	54589141	54589141	A	G	intergenic	GNL3L,ITIH6	dist=1637;dis	.	.	A	G	+	+	0.4025974	46,0,31,0	chrX,545891	Cytosol	A > G
chrX:54589151:A:G:intergenic:GNI	chrX	54589151	54589151	A	G	intergenic	GNL3L,ITIH6	dist=1647;dis	.	.	A	G	+	+	0.02439024	80,0,2,0	chrX,545891	Cytosol	A > G
chrX:54589160:A:G:intergenic:GNI	chrX	54589160	54589160	A	G	intergenic	GNL3L,ITIH6	dist=1656;dis	.	.	A	G	+	+	0.05555556	68,0,4,0	chrX,545891	Cytosol	A > G
chrX:54589171:A:G:intergenic:GNI	chrX	54589171	54589171	A	G	intergenic	GNL3L,ITIH6	dist=1667;dis	.	.	A	G	+	+	0.10126582	71,0,8,0	chrX,545891	Cytosol	A > G
chrX:54589181:A:G:intergenic:GNI	chrX	54589181	54589181	A	G	intergenic	GNL3L,ITIH6	dist=1677;dis	.	.	A	G	+	+	0.14772727	75,0,13,0	chrX,545891	Cytosol	A > G
chrX:54589183:A:G:intergenic:GNI	chrX	54589183	54589183	A	G	intergenic	GNL3L,ITIH6	dist=1679;dis	.	.	A	G	+	+	0.45652174	50,0,42,0	chrX,545891	Cytosol	A > G
chrX:54589233:A:G:intergenic:GNI	chrX	54589233	54589233	A	G	intergenic	GNL3L,ITIH6	dist=1729;dis	.	.	A	G	+	+	0.02840909	171,0,5,0	chrX,545892	Cytosol	A > G
chrX:54589236:A:G:intergenic:GNI	chrX	54589236	54589236	A	G	intergenic	GNL3L,ITIH6	dist=1732;dis	.	.	A	G	+	+	0.01666667	177,0,3,0	chrX,545892	Cytosol	A > G
chrX:54589948:A:G:intergenic:GNI	chrX	54589948	54589948	A	G	intergenic	GNL3L,ITIH6	dist=2444;dis	.	.	A	G	+	+	0.24277457	131,0,42,0	chrX,545899	Cytosol	A > G
chrX:54589955:A:G:intergenic:GNI	chrX	54589955	54589955	A	G	intergenic	GNL3L,ITIH6	dist=2451;dis	.	.	A	G	+	+	0.13609467	146,0,23,0	chrX,545899	Cytosol	A > G
chrX:54589961:A:G:intergenic:GNI	chrX	54589961	54589961	A	G	intergenic	GNL3L,ITIH6	dist=2457;dis	.	.	A	G	+	+	0.29090909	117,0,48,0	chrX,545899	Cytosol	A > G

chrX:54589962:A:G:intergenic:GNI	chrX	54589962	54589962	A	G	intergenic	GNL3L,ITIH6	dist=2458;dis	.	.	A	G	+	+	0.6626506	56,0,110,0	chrX,545899	Cytosol	A > G
chrX:54589988:A:G:intergenic:GNI	chrX	54589988	54589988	A	G	intergenic	GNL3L,ITIH6	dist=2484;dis	.	.	A	G	+	+	0.33557047	99,0,50,0	chrX,545899	Cytosol	A > G
chrX:54590002:A:G:intergenic:GNI	chrX	54590002	54590002	A	G	intergenic	GNL3L,ITIH6	dist=2498;dis	.	.	A	G	+	+	0.05223881	127,0,7,0	chrX,545900	Cytosol	A > G
chrX:54590007:A:G:intergenic:GNI	chrX	54590007	54590007	A	G	intergenic	GNL3L,ITIH6	dist=2503;dis	.	.	A	G	+	+	0.13907285	130,0,21,0	chrX,545900	Cytosol	A > G
chrX:54590015:A:G:intergenic:GNI	chrX	54590015	54590015	A	G	intergenic	GNL3L,ITIH6	dist=2511;dis	.	.	A	G	+	+	0.17197452	130,0,27,0	chrX,545900	Cytosol	A > G
chrX:54590026:A:G:intergenic:GNI	chrX	54590026	54590026	A	G	intergenic	GNL3L,ITIH6	dist=2522;dis	.	.	A	G	+	+	0.14375	137,0,23,0	chrX,545900	Cytosol	A > G
chrX:54590033:A:G:intergenic:GNI	chrX	54590033	54590033	A	G	intergenic	GNL3L,ITIH6	dist=2529;dis	.	.	A	G	+	+	0.29054054	105,0,43,0	chrX,545900	Cytosol	A > G
chrX:54590046:A:G:intergenic:GNI	chrX	54590046	54590046	A	G	intergenic	GNL3L,ITIH6	dist=2542;dis	.	.	A	G	+	+	0.0703125	119,0,9,0	chrX,545900	Cytosol	A > G
chrX:54590052:A:G:intergenic:GNI	chrX	54590052	54590052	A	G	intergenic	GNL3L,ITIH6	dist=2548;dis	.	.	A	G	+	+	0.1637931	97,0,19,0	chrX,545900	Cytosol	A > G
chrX:54590055:A:G:intergenic:GNI	chrX	54590055	54590055	A	G	intergenic	GNL3L,ITIH6	dist=2551;dis	.	.	A	G	+	+	0.1826087	94,0,21,0	chrX,545900	Cytosol	A > G
chrX:54590056:A:G:intergenic:GNI	chrX	54590056	54590056	A	G	intergenic	GNL3L,ITIH6	dist=2552;dis	.	.	A	G	+	+	0.12820513	102,0,15,0	chrX,545900	Cytosol	A > G
chrX:54590058:A:G:intergenic:GNI	chrX	54590058	54590058	A	G	intergenic	GNL3L,ITIH6	dist=2554;dis	.	.	A	G	+	+	0.02702703	108,0,3,0	chrX,545900	Cytosol	A > G
chrX:54590063:A:G:intergenic:GNI	chrX	54590063	54590063	A	G	intergenic	GNL3L,ITIH6	dist=2559;dis	.	.	A	G	+	+	0.30973451	78,0,35,0	chrX,545900	Cytosol	A > G
chrX:54590066:A:G:intergenic:GNI	chrX	54590066	54590066	A	G	intergenic	GNL3L,ITIH6	dist=2562;dis	.	.	A	G	+	+	0.38938053	69,0,44,0	chrX,545900	Cytosol	A > G
chrX:54590067:A:G:intergenic:GNI	chrX	54590067	54590067	A	G	intergenic	GNL3L,ITIH6	dist=2563;dis	.	.	A	G	+	+	0.25892857	83,0,29,0	chrX,545900	Cytosol	A > G
chrX:54590080:A:G:intergenic:GNI	chrX	54590080	54590080	A	G	intergenic	GNL3L,ITIH6	dist=2576;dis	.	.	A	G	+	+	0.35294118	55,0,30,0	chrX,545900	Cytosol	A > G
chrX:54590098:A:G:intergenic:GNI	chrX	54590098	54590098	A	G	intergenic	GNL3L,ITIH6	dist=2594;dis	.	.	A	G	+	+	0.05454545	104,0,6,0	chrX,545900	Cytosol	A > G
chrX:54590108:A:G:intergenic:GNI	chrX	54590108	54590108	A	G	intergenic	GNL3L,ITIH6	dist=2604;dis	.	.	A	G	+	+	0.16393443	102,0,20,0	chrX,545901	Cytosol	A > G
chrX:54590125:A:G:intergenic:GNI	chrX	54590125	54590125	A	G	intergenic	GNL3L,ITIH6	dist=2621;dis	.	.	A	G	+	+	0.41860465	50,0,36,0	chrX,545901	Cytosol	A > G
chrX:54590144:A:G:intergenic:GNI	chrX	54590144	54590144	A	G	intergenic	GNL3L,ITIH6	dist=2640;dis	.	.	A	G	+	+	0.01298701	152,0,2,0	chrX,545901	Cytosol	A > G
chrX:54590222:G:A:intergenic:GNI	chrX	54590222	54590222	G	A	intergenic	GNL3L,ITIH6	dist=2718;dis	.	.	G	A	+	+	0.01863354	3,0,158,0	chrX,545902	Cytosol	G > A
chrX:54590921:A:G:intergenic:GNI	chrX	54590921	54590921	A	G	intergenic	GNL3L,ITIH6	dist=3417;dis	.	.	A	G	+	+	0.16666667	70,0,14,0	chrX,545909	Cytosol	A > G
chrX:54590933:A:G:intergenic:GNI	chrX	54590933	54590933	A	G	intergenic	GNL3L,ITIH6	dist=3429;dis	.	.	A	G	+	+	0.27380952	61,0,23,0	chrX,545909	Cytosol	A > G
chrX:54590940:A:G:intergenic:GNI	chrX	54590940	54590940	A	G	intergenic	GNL3L,ITIH6	dist=3436;dis	.	.	A	G	+	+	0.31081081	51,0,23,0	chrX,545909	Cytosol	A > G
chrX:54590941:A:G:intergenic:GNI	chrX	54590941	54590941	A	G	intergenic	GNL3L,ITIH6	dist=3437;dis	.	.	A	G	+	+	0.5890411	30,0,43,0	chrX,545909	Cytosol	A > G
chrX:54591006:A:G:intergenic:GNI	chrX	54591006	54591006	A	G	intergenic	GNL3L,ITIH6	dist=3502;dis	.	.	A	G	+	+	0.18421053	31,0,7,0	chrX,545910	Cytosol	A > G
chrX:54591021:A:G:intergenic:GNI	chrX	54591021	54591021	A	G	intergenic	GNL3L,ITIH6	dist=3517;dis	.	.	A	G	+	+	0.43636364	31,0,24,0	chrX,545910	Cytosol	A > G
chrX:54591038:A:G:intergenic:GNI	chrX	54591038	54591038	A	G	intergenic	GNL3L,ITIH6	dist=3534;dis	.	.	A	G	+	+	0.26	37,0,13,0	chrX,545910	Cytosol	A > G
chrX:54591050:A:G:intergenic:GNI	chrX	54591050	54591050	A	G	intergenic	GNL3L,ITIH6	dist=3546;dis	.	.	A	G	+	+	0.17021277	39,0,8,0	chrX,545910	Cytosol	A > G
chrX:54591063:A:G:intergenic:GNI	chrX	54591063	54591063	A	G	intergenic	GNL3L,ITIH6	dist=3559;dis	.	.	A	G	+	+	0.04166667	46,0,2,0	chrX,545910	Cytosol	A > G
chrX:54591091:A:G:intergenic:GNI	chrX	54591091	54591091	A	G	intergenic	GNL3L,ITIH6	dist=3587;dis	.	.	A	G	+	+	0.81395349	8,0,35,0	chrX,545910	Cytosol	A > G
chrX:54591198:A:G:intergenic:GNI	chrX	54591198	54591198	A	G	intergenic	GNL3L,ITIH6	dist=3694;dis	.	.	A	G	+	+	0.11111111	32,0,4,0	chrX,545911	Cytosol	A > G
chrX:54591241:A:G:intergenic:GNI	chrX	54591241	54591241	A	G	intergenic	GNL3L,ITIH6	dist=3737;dis	.	.	A	G	+	+	0.05263158	90,0,5,0	chrX,545912	Cytosol	A > G
chrX:54591247:A:G:intergenic:GNI	chrX	54591247	54591247	A	G	intergenic	GNL3L,ITIH6	dist=3743;dis	.	.	A	G	+	+	0.08641975	74,0,7,0	chrX,545912	Cytosol	A > G
chrX:54591253:A:G:intergenic:GNI	chrX	54591253	54591253	A	G	intergenic	GNL3L,ITIH6	dist=3749;dis	.	.	A	G	+	+	0.25	63,0,21,0	chrX,545912	Cytosol	A > G
chrX:54591266:A:G:intergenic:GNI	chrX	54591266	54591266	A	G	intergenic	GNL3L,ITIH6	dist=3762;dis	.	.	A	G	+	+	0.23076923	60,0,18,0	chrX,545912	Cytosol	A > G
chrX:54591267:A:G:intergenic:GNI	chrX	54591267	54591267	A	G	intergenic	GNL3L,ITIH6	dist=3763;dis	.	.	A	G	+	+	0.44736842	42,0,34,0	chrX,545912	Cytosol	A > G
chrX:54591271:A:G:intergenic:GNI	chrX	54591271	54591271	A	G	intergenic	GNL3L,ITIH6	dist=3767;dis	.	.	A	G	+	+	0.12162162	65,0,9,0	chrX,545912	Cytosol	A > G
chrX:54591296:A:G:intergenic:GNI	chrX	54591296	54591296	A	G	intergenic	GNL3L,ITIH6	dist=3792;dis	.	.	A	G	+	+	0.2173913	54,0,15,0	chrX,545912	Cytosol	A > G
chrX:54591306:A:G:intergenic:GNI	chrX	54591306	54591306	A	G	intergenic	GNL3L,ITIH6	dist=3802;dis	.	.	A	G	+	+	0.14285714	78,0,13,0	chrX,545913	Cytosol	A > G
chrX:54591308:A:G:intergenic:GNI	chrX	54591308	54591308	A	G	intergenic	GNL3L,ITIH6	dist=3804;dis	.	.	A	G	+	+	0.03225806	89,0,3,1	chrX,545913	Cytosol	A > G
chrX:54591347:A:G:intergenic:GNI	chrX	54591347	54591347	A	G	intergenic	GNL3L,ITIH6	dist=3843;dis	.	.	A	G	+	+	0.34259259	71,0,37,0	chrX,545913	Cytosol	A > G
chrX:54591370:A:G:intergenic:GNI	chrX	54591370	54591370	A	G	intergenic	GNL3L,ITIH6	dist=3866;dis	.	.	A	G	+	+	0.0875	73,0,7,0	chrX,545913	Cytosol	A > G
chrX:54591377:A:G:intergenic:GNI	chrX	54591377	54591377	A	G	intergenic	GNL3L,ITIH6	dist=3873;dis	.	.	A	G	+	+	0.13333333	65,0,10,0	chrX,545913	Cytosol	A > G
chrX:54591402:A:G:intergenic:GNI	chrX	54591402	54591402	A	G	intergenic	GNL3L,ITIH6	dist=3898;dis	.	.	A	G	+	+	0.08510638	86,0,8,0	chrX,545914	Cytosol	A > G
chrX:54591403:A:G:intergenic:GNI	chrX	54591403	54591403	A	G	intergenic	GNL3L,ITIH6	dist=3899;dis	.	.	A	G	+	+	0.13636364	76,0,12,0	chrX,545914	Cytosol	A > G
chrX:54591404:A:G:intergenic:GNI	chrX	54591404	54591404	A	G	intergenic	GNL3L,ITIH6	dist=3900;dis	.	.	A	G	+	+	0.35294118	55,0,30,0	chrX,545914	Cytosol	A > G
chrX:54591405:A:G:intergenic:GNI	chrX	54591405	54591405	A	G	intergenic	GNL3L,ITIH6	dist=3901;dis	.	.	A	G	+	+	0.26829268	60,0,22,0	chrX,545914	Cytosol	A > G
chrX:54591411:A:G:intergenic:GNI	chrX	54591411	54591411	A	G	intergenic	GNL3L,ITIH6	dist=3907;dis	.	.	A	G	+	+	0.32857143	47,0,23,0	chrX,545914	Cytosol	A > G
chrX:54591415:A:G:intergenic:GNI	chrX	54591415	54591415	A	G	intergenic	GNL3L,ITIH6	dist=3911;dis	.	.	A	G	+	+	0.26470588	50,0,18,0	chrX,545914	Cytosol	A > G
chrX:54591430:A:G:intergenic:GNI	chrX	54591430	54591430	A	G	intergenic	GNL3L,ITIH6	dist=3926;dis	.	.	A	G	+	+	0.02941176	66,0,2,0	chrX,545914	Cytosol	A > G
chrX:54591442:A:G:intergenic:GNI	chrX	54591442	54591442	A	G	intergenic	GNL3L,ITIH6	dist=3938;dis	.	.	A	G	+	+	0.02298851	85,0,2,0	chrX,545914	Cytosol	A > G
chrX:54591448:A:G:intergenic:GNI	chrX	54591448	54591448	A	G	intergenic	GNL3L,ITIH6	dist=3944;dis	.	.	A	G	+	+	0.04494382	85,0,4,0	chrX,545914	Cytosol	A > G
chrX:54592003:A:G:intergenic:GNI	chrX	54592003	54592003	A	G	intergenic	GNL3L,ITIH6	dist=4499;dis	.	.	A	G	+	+	0.21428571	11,0,3,0	chrX,545920	Cytosol	A > G
chrX:54592006:A:G:intergenic:GNI	chrX	54592006	54592006	A	G	intergenic	GNL3L,ITIH6	dist=4502;dis	.	.	A	G	+	+	0.26666667	11,0,4,0	chrX,545920	Cytosol	A > G
chrX:54592036:A:G:intergenic:GNI	chrX	54592036	54592036	A	G	intergenic	GNL3L,ITIH6	dist=4532;dis	.	.	A	G	+	+	0.13333333	13,0,2,0	chrX,545920	Cytosol	A > G
chrX:54593276:A:G:intergenic:GNI	chrX	54593276	54593276	A	G	intergenic	GNL3L,ITIH6	dist=5772;dis	.	.	A	G	+	+	0.13043478	20,0,3,0	chrX,545932	Cytosol	A > G
chrX:54593319:A:G:intergenic:GNI	chrX	54593319	54593319	A	G	intergenic	GNL3L,ITIH6	dist=5815;dis	.	.	A	G	+	+	0.38095238	13,0,8,0	chrX,545933	Cytosol	A > G
chrX:70275158:T:C:intergenic:RNU	chrX	70275158	70275158	T	C	intergenic	RNU1-56P,SN	dist=34870;d	.	.	A	G	-	-	0.10344828	26,0,3,0	chrX,702751	Cytosol	A > G
chrX:70275165:T:C:intergenic:RNU	chrX	70275165	70275165	T	C	intergenic	RNU1-56P,SN	dist=34877;d	.	.	A	G	-	-	0.38709677	19,0,12,0	chrX,702751	Cytosol	A > G
chrX:70275210:T:C:intergenic:RNU	chrX	70275210	70275210	T	C	intergenic	RNU1-56P,SN	dist=34922;d	.	.	A	G	-	-	0.12121212	29,0,4,0	chrX,702752	Cytosol	A > G
chrX:70276129:T:C:intergenic:RNU	chrX	70276129	70276129	T	C	intergenic	RNU1-56P,SN	dist=35841;d	.	.	A	G	-	-	0.30769231	27,0,12,0	chrX,702761	Cytosol	A > G
chrX:70276146:T:C:intergenic:RNU	chrX	70276146	70276146	T	C	intergenic	RNU1-56P,SN	dist=35858;d	.	.	A	G	-	-	0.2972973	26,0,11,0	chrX,702761	Cytosol	A > G
chrX:70276153:T:C:intergenic:RNU	chrX	70276153	70276153	T	C	intergenic	RNU1-56P,SN	dist=35865;d	.	.	A	G	-	-	0.3030303	23,0,10,0	chrX,702761	Cytosol	A > G

chrX:70277639:T:C:intergenic:RNU	chrX	70277639	70277639	T	C	intergenic	RNU1-56P,SN	dist=37351;d	.	.	A	G	-	-	0.48214286	29,0,27,0	chrX,702776	Cytosol	A > G
chrX:70278356:T:C:downstream:S	chrX	70278356	70278356	T	C	downstream	SNX12	.	.	.	A	G	-	-	0.15	17,0,3,0	chrX,702783	Cytosol	A > G
chrX:70278357:T:C:downstream:S	chrX	70278357	70278357	T	C	downstream	SNX12	.	.	.	A	G	-	-	0.0952381	19,0,2,0	chrX,702783	Cytosol	A > G
chrX:73166845:A:G:ncRNA_exonic	chrX	73166845	73166845	A	G	ncRNA_exon	JPX	.	.	.	A	G	+	+	0.14049587	104,0,17,0	chrX,731668	Cytosol	A > G
chrX:73166889:A:G:ncRNA_exonic	chrX	73166889	73166889	A	G	ncRNA_exon	JPX	.	.	.	A	G	+	+	0.07692308	36,0,3,0	chrX,731668	Cytosol	A > G
chrX:73166893:A:G:ncRNA_exonic	chrX	73166893	73166893	A	G	ncRNA_exon	JPX	.	.	.	A	G	+	+	0.05128205	37,0,2,0	chrX,731668	Cytosol	A > G
chrX:73166906:A:G:ncRNA_exonic	chrX	73166906	73166906	A	G	ncRNA_exon	JPX	.	.	.	A	G	+	+	0.17073171	34,0,7,0	chrX,731669	Cytosol	A > G
chrX:73166962:A:G:ncRNA_exonic	chrX	73166962	73166962	A	G	ncRNA_exon	JPX	.	.	.	A	G	+	+	0.05	57,0,3,0	chrX,731669	Cytosol	A > G
chrX:73166972:A:G:ncRNA_exonic	chrX	73166972	73166972	A	G	ncRNA_exon	JPX	.	.	.	A	G	+	+	0.0483871	59,0,3,0	chrX,731669	Cytosol	A > G
chrX:73167063:A:G:ncRNA_exonic	chrX	73167063	73167063	A	G	ncRNA_exon	JPX	.	.	.	A	G	+	+	0.08196721	56,0,5,0	chrX,731670	Cytosol	A > G
chrX:73218844:A:G:ncRNA_exonic	chrX	73218844	73218844	A	G	ncRNA_exon	JPX	.	.	.	A	G	+	+-	0.11764706	30,0,4,0	chrX,732188	Cytosol	A > G
chrX:73218886:A:G:ncRNA_exonic	chrX	73218886	73218886	A	G	ncRNA_exon	JPX	.	.	.	A	G	+	+-	0.08571429	32,0,3,0	chrX,732188	Cytosol	A > G
chrX:73219017:A:G:ncRNA_exonic	chrX	73219017	73219017	A	G	ncRNA_exon	JPX	.	.	.	A	G	+	+-	0.1025641	35,0,4,0	chrX,732190	Cytosol	A > G
chrX:73228796:A:G:ncRNA_introni	chrX	73228796	73228796	A	G	ncRNA_intro	FTX,JPX	.	.	.	A	G	+	+-	0.33333333	6,0,3,0	chrX,732287	Cytosol	A > G
chrX:73229968:A:G:ncRNA_introni	chrX	73229968	73229968	A	G	ncRNA_intro	FTX,JPX,RP13	.	.	.	A	G	+	+-	0.6	2,0,3,0	chrX,732299	Cytosol	A > G
chrX:73230354:A:G:ncRNA_introni	chrX	73230354	73230354	A	G	ncRNA_intro	FTX,JPX,RP13	.	.	.	A	G	+	+-	0.55555556	4,0,5,0	chrX,732303	Cytosol	A > G
chrX:73230436:A:G:ncRNA_introni	chrX	73230436	73230436	A	G	ncRNA_intro	FTX,JPX,RP13	.	.	.	A	G	+	+-	0.33333333	6,0,3,0	chrX,732304	Cytosol	A > G
chrX:73416963:T:C:ncRNA_introni	chrX	73416963	73416963	T	C	ncRNA_intro	FTX	.	.	.	A	G	-	-	0.35294118	11,0,6,0	chrX,734169	Cytosol	A > G
chrX:73416986:T:C:ncRNA_introni	chrX	73416986	73416986	T	C	ncRNA_intro	FTX	.	.	.	A	G	-	-	0.5	9,0,9,0	chrX,734169	Cytosol	A > G
chrX:73417037:T:C:ncRNA_introni	chrX	73417037	73417037	T	C	ncRNA_intro	FTX	.	.	.	A	G	-	-	0.17647059	14,0,3,0	chrX,734170	Cytosol	A > G
chrX:73417059:T:C:ncRNA_introni	chrX	73417059	73417059	T	C	ncRNA_intro	FTX	.	.	.	A	G	-	-	0.5	7,0,7,0	chrX,734170	Cytosol	A > G
chrX:73417103:T:C:ncRNA_introni	chrX	73417103	73417103	T	C	ncRNA_intro	FTX	.	.	.	A	G	-	-	0.26666667	11,0,4,0	chrX,734171	Cytosol	A > G
chrX:73417105:T:C:ncRNA_introni	chrX	73417105	73417105	T	C	ncRNA_intro	FTX	.	.	.	A	G	-	-	0.26666667	11,0,4,0	chrX,734171	Cytosol	A > G
chrX:73417147:T:C:ncRNA_introni	chrX	73417147	73417147	T	C	ncRNA_intro	FTX	.	.	.	A	G	-	-	0.13043478	20,0,3,0	chrX,734171	Cytosol	A > G
chrX:73419961:T:C:ncRNA_introni	chrX	73419961	73419961	T	C	ncRNA_intro	FTX	.	.	.	A	G	-	-	0.5	3,0,3,0	chrX,734199	Cytosol	A > G
chrX:73421672:T:C:ncRNA_exonic:	chrX	73421672	73421672	T	C	ncRNA_exon	RP3-368A4.5	.	.	.	A	G	-	-	0.41666667	7,0,5,0	chrX,734216	Cytosol	A > G
chrX:73434194:T:C:ncRNA_introni	chrX	73434194	73434194	T	C	ncRNA_intro	FTX,RP3-368	.	.	.	A	G	-	-	0.4	3,0,2,0	chrX,734341	Cytosol	A > G
chrX:73434235:T:C:ncRNA_introni	chrX	73434235	73434235	T	C	ncRNA_intro	FTX,RP3-368	.	.	.	A	G	-	-	0.33333333	4,0,2,0	chrX,734342	Cytosol	A > G
chrX:77082975:T:C:UTR3:MAGT1	chrX	77082975	77082975	T	C	UTR3	MAGT1	ENST000003	.	.	A	G	-	-	0.03211009	211,0,7,0	chrX,770829	Cytosol	A > G
chrX:77083006:T:C:UTR3:MAGT1	chrX	77083006	77083006	T	C	UTR3	MAGT1	ENST000003	.	.	A	G	-	-	0.11983471	213,0,29,0	chrX,770830	Cytosol	A > G
chrX:77083007:T:C:UTR3:MAGT1	chrX	77083007	77083007	T	C	UTR3	MAGT1	ENST000003	.	.	A	G	-	-	0.23430962	183,0,56,0	chrX,770830	Cytosol	A > G
chrX:77083015:T:C:UTR3:MAGT1	chrX	77083015	77083015	T	C	UTR3	MAGT1	ENST000003	.	.	A	G	-	-	0.03875969	248,0,10,0	chrX,770830	Cytosol	A > G
chrX:77083041:T:C:UTR3:MAGT1	chrX	77083041	77083041	T	C	UTR3	MAGT1	ENST000003	.	.	A	G	-	-	0.18699187	200,0,46,0	chrX,770830	Cytosol	A > G
chrX:77083054:T:C:UTR3:MAGT1	chrX	77083054	77083054	T	C	UTR3	MAGT1	ENST000003	.	.	A	G	-	-	0.02690583	217,0,6,0	chrX,770830	Cytosol	A > G
chrX:77083063:T:C:UTR3:MAGT1	chrX	77083063	77083063	T	C	UTR3	MAGT1	ENST000003	.	.	A	G	-	-	0.04587156	208,0,10,0	chrX,770830	Cytosol	A > G
chrX:77083067:T:C:UTR3:MAGT1	chrX	77083067	77083067	T	C	UTR3	MAGT1	ENST000003	.	.	A	G	-	-	0.0199005	197,0,4,0	chrX,770830	Cytosol	A > G
chrX:77083076:T:C:UTR3:MAGT1	chrX	77083076	77083076	T	C	UTR3	MAGT1	ENST000003	.	.	A	G	-	-	0.02702703	180,0,5,0	chrX,770830	Cytosol	A > G
chrX:77083107:T:C:UTR3:MAGT1	chrX	77083107	77083107	T	C	UTR3	MAGT1	ENST000003	.	.	A	G	-	-	0.01052632	188,0,2,0	chrX,770831	Cytosol	A > G
chrX:77083109:T:C:UTR3:MAGT1	chrX	77083109	77083109	T	C	UTR3	MAGT1	ENST000003	.	.	A	G	-	-	0.01036269	191,0,2,0	chrX,770831	Cytosol	A > G
chrX:77083116:T:C:UTR3:MAGT1	chrX	77083116	77083116	T	C	UTR3	MAGT1	ENST000003	.	.	A	G	-	-	0.03626943	186,0,7,0	chrX,770831	Cytosol	A > G
chrX:77083117:T:C:UTR3:MAGT1	chrX	77083117	77083117	T	C	UTR3	MAGT1	ENST000003	.	.	A	G	-	-	0.0255102	191,0,5,0	chrX,770831	Cytosol	A > G
chrX:77083118:T:C:UTR3:MAGT1	chrX	77083118	77083118	T	C	UTR3	MAGT1	ENST000003	.	.	A	G	-	-	0.12698413	165,0,24,0	chrX,770831	Cytosol	A > G
chrX:77083121:T:C:UTR3:MAGT1	chrX	77083121	77083121	T	C	UTR3	MAGT1	ENST000003	.	.	A	G	-	-	0.11	178,0,22,0	chrX,770831	Cytosol	A > G
chrX:77083132:T:C:UTR3:MAGT1	chrX	77083132	77083132	T	C	UTR3	MAGT1	ENST000003	.	.	A	G	-	-	0.10659898	176,0,21,0	chrX,770831	Cytosol	A > G
chrX:77083179:T:C:UTR3:MAGT1	chrX	77083179	77083179	T	C	UTR3	MAGT1	ENST000003	.	.	A	G	-	-	0.10222222	202,0,23,0	chrX,770831	Cytosol	A > G
chrX:77083183:T:C:UTR3:MAGT1	chrX	77083183	77083183	T	C	UTR3	MAGT1	ENST000003	.	.	A	G	-	-	0.03286385	206,0,7,0	chrX,770831	Cytosol	A > G
chrX:77083187:T:C:UTR3:MAGT1	chrX	77083187	77083187	T	C	UTR3	MAGT1	ENST000003	.	.	A	G	-	-	0.02197802	267,0,6,0	chrX,770831	Cytosol	A > G
chrX:77083209:T:C:UTR3:MAGT1	chrX	77083209	77083209	T	C	UTR3	MAGT1	ENST000003	.	.	A	G	-	-	0.03780069	280,0,11,0	chrX,770832	Cytosol	A > G
chrX:77083517:T:C:UTR3:MAGT1	chrX	77083517	77083517	T	C	UTR3	MAGT1	ENST000003	.	.	A	G	-	-	0.01298701	228,0,3,0	chrX,770835	Cytosol	A > G
chrX:77083538:T:C:UTR3:MAGT1	chrX	77083538	77083538	T	C	UTR3	MAGT1	ENST000003	.	.	A	G	-	-	0.02892562	235,0,7,0	chrX,770835	Cytosol	A > G
chrX:77083548:T:C:UTR3:MAGT1	chrX	77083548	77083548	T	C	UTR3	MAGT1	ENST000003	.	.	A	G	-	-	0.02955665	197,0,6,0	chrX,770835	Cytosol	A > G
chrX:77083575:T:C:UTR3:MAGT1	chrX	77083575	77083575	T	C	UTR3	MAGT1	ENST000003	.	.	A	G	-	-	0.06896552	324,0,24,0	chrX,770835	Cytosol	A > G
chrX:77083592:T:C:UTR3:MAGT1	chrX	77083592	77083592	T	C	UTR3	MAGT1	ENST000003	.	.	A	G	-	-	0.01075269	276,0,3,0	chrX,770835	Cytosol	A > G
chrX:77083614:T:C:UTR3:MAGT1	chrX	77083614	77083614	T	C	UTR3	MAGT1	ENST000003	.	.	A	G	-	-	0.02684564	290,0,8,0	chrX,770836	Cytosol	A > G
chrX:77083615:T:C:UTR3:MAGT1	chrX	77083615	77083615	T	C	UTR3	MAGT1	ENST000003	.	.	A	G	-	-	0.03870968	298,0,12,0	chrX,770836	Cytosol	A > G
chrX:77083617:T:C:UTR3:MAGT1	chrX	77083617	77083617	T	C	UTR3	MAGT1	ENST000003	.	.	A	G	-	-	0.23232323	227,1,69,0	chrX,770836	Cytosol	A > G
chrX:77083641:T:C:UTR3:MAGT1	chrX	77083641	77083641	T	C	UTR3	MAGT1	ENST000003	.	.	A	G	-	-	0.075	148,0,12,0	chrX,770836	Cytosol	A > G
chrX:77083644:T:C:UTR3:MAGT1	chrX	77083644	77083644	T	C	UTR3	MAGT1	ENST000003	.	.	A	G	-	-	0.03448276	168,0,6,0	chrX,770836	Cytosol	A > G
chrX:77083645:T:C:UTR3:MAGT1	chrX	77083645	77083645	T	C	UTR3	MAGT1	ENST000003	.	.	A	G	-	-	0.15934066	153,0,29,0	chrX,770836	Cytosol	A > G
chrX:77083653:T:C:UTR3:MAGT1	chrX	77083653	77083653	T	C	UTR3	MAGT1	ENST000003	.	.	A	G	-	-	0.21195652	145,0,39,0	chrX,770836	Cytosol	A > G
chrX:77083654:T:C:UTR3:MAGT1	chrX	77083654	77083654	T	C	UTR3	MAGT1	ENST000003	.	.	A	G	-	-	0.10194175	185,0,21,0	chrX,770836	Cytosol	A > G
chrX:77083670:T:C:UTR3:MAGT1	chrX	77083670	77083670	T	C	UTR3	MAGT1	ENST000003	.	.	A	G	-	-	0.17687075	121,0,26,0	chrX,770836	Cytosol	A > G
chrX:77083673:T:C:UTR3:MAGT1	chrX	77083673	77083673	T	C	UTR3	MAGT1	ENST000003	.	.	A	G	-	-	0.31958763	132,0,62,0	chrX,770836	Cytosol	A > G
chrX:77083697:T:C:UTR3:MAGT1	chrX	77083697	77083697	T	C	UTR3	MAGT1	ENST000003	.	.	A	G	-	-	0.01276596	232,0,3,0	chrX,770836	Cytosol	A > G

chrX:77083723:T:C:UTR3:MAGT1	chrX	77083723	77083723	T	C	UTR3	MAGT1	ENST000003	.	.	A	G	-	-	0.16877637	197,0,40,0	chrX,770837	Cytosol	A > G
chrX:77083828:T:C:UTR3:MAGT1	chrX	77083828	77083828	T	C	UTR3	MAGT1	ENST000003	.	.	A	G	-	-	0.34210526	175,0,91,0	chrX,770838	Cytosol	A > G
chrX:77083845:T:C:UTR3:MAGT1	chrX	77083845	77083845	T	C	UTR3	MAGT1	ENST000003	.	.	A	G	-	-	0.0155642	253,0,4,0	chrX,770838	Cytosol	A > G
chrX:77083846:T:C:UTR3:MAGT1	chrX	77083846	77083846	T	C	UTR3	MAGT1	ENST000003	.	.	A	G	-	-	0.0112782	263,0,3,0	chrX,770838	Cytosol	A > G
chrX:77083891:T:C:UTR3:MAGT1	chrX	77083891	77083891	T	C	UTR3	MAGT1	ENST000003	.	.	A	G	-	-	0.42236025	93,0,68,0	chrX,770838	Cytosol	A > G
chrX:77083897:T:C:UTR3:MAGT1	chrX	77083897	77083897	T	C	UTR3	MAGT1	ENST000003	.	.	A	G	-	-	0.109375	171,0,21,0	chrX,770838	Cytosol	A > G
chrX:77083900:T:C:UTR3:MAGT1	chrX	77083900	77083900	T	C	UTR3	MAGT1	ENST000003	.	.	A	G	-	-	0.11059908	193,0,24,0	chrX,770838	Cytosol	A > G
chrX:77083914:T:C:UTR3:MAGT1	chrX	77083914	77083914	T	C	UTR3	MAGT1	ENST000003	.	.	A	G	-	-	0.28384279	164,0,65,0	chrX,770839	Cytosol	A > G
chrX:77083915:T:C:UTR3:MAGT1	chrX	77083915	77083915	T	C	UTR3	MAGT1	ENST000003	.	.	A	G	-	-	0.51293103	113,0,119,0	chrX,770839	Cytosol	A > G
chrX:77083939:T:C:UTR3:MAGT1	chrX	77083939	77083939	T	C	UTR3	MAGT1	ENST000003	.	.	A	G	-	-	0.03003003	323,0,10,0	chrX,770839	Cytosol	A > G
chrX:77083955:T:C:UTR3:MAGT1	chrX	77083955	77083955	T	C	UTR3	MAGT1	ENST000003	.	.	A	G	-	-	0.02153846	318,0,7,0	chrX,770839	Cytosol	A > G
chrX:77083964:T:C:UTR3:MAGT1	chrX	77083964	77083964	T	C	UTR3	MAGT1	ENST000003	.	.	A	G	-	-	0.04123711	279,0,12,0	chrX,770839	Cytosol	A > G
chrX:77083981:T:C:UTR3:MAGT1	chrX	77083981	77083981	T	C	UTR3	MAGT1	ENST000003	.	.	A	G	-	-	0.08823529	310,0,30,0	chrX,770839	Cytosol	A > G
chrX:77083982:T:C:UTR3:MAGT1	chrX	77083982	77083982	T	C	UTR3	MAGT1	ENST000003	.	.	A	G	-	-	0.07458564	335,0,27,0	chrX,770839	Cytosol	A > G
chrX:77084001:T:C:UTR3:MAGT1	chrX	77084001	77084001	T	C	UTR3	MAGT1	ENST000003	.	.	A	G	-	-	0.13313609	293,0,45,0	chrX,770840	Cytosol	A > G
chrX:77084060:T:C:UTR3:MAGT1	chrX	77084060	77084060	T	C	UTR3	MAGT1	ENST000003	.	.	A	G	-	-	0.03188406	334,0,11,0	chrX,770840	Cytosol	A > G
chrX:77157468:A:G:intronic:COX7I	chrX	77157468	77157468	A	G	intronic	COX7B	.	.	.	A	G	+	+	0.40909091	13,0,9,0	chrX,771574	Cytosol	A > G
chrX:77387591:A:G:intronic:TAF9E	chrX	77387591	77387591	A	G	intronic	TAF9B	.	.	.	A	G	+	-	0.1875	13,0,3,0	chrX,773875	Cytosol	A > G
chrX:77387674:A:G:intronic:TAF9E	chrX	77387674	77387674	A	G	intronic	TAF9B	.	.	.	A	G	+	-	0.16666667	10,0,2,0	chrX,773876	Cytosol	A > G
chrX:84345835:A:G:intergenic:APC	chrX	84345835	84345835	A	G	intergenic	APOOL,SATL	dist=2766;dis	.	.	A	G	+	+	0.42372881	34,0,25,0	chrX,843458	Cytosol	A > G
chrX:84345851:A:G:intergenic:APC	chrX	84345851	84345851	A	G	intergenic	APOOL,SATL	dist=2782;dis	.	.	A	G	+	+	0.37974684	49,0,30,0	chrX,843458	Cytosol	A > G
chrX:84345871:A:G:intergenic:APC	chrX	84345871	84345871	A	G	intergenic	APOOL,SATL	dist=2802;dis	.	.	A	G	+	+	0.10958904	65,0,8,0	chrX,843458	Cytosol	A > G
chrX:84345873:A:G:intergenic:APC	chrX	84345873	84345873	A	G	intergenic	APOOL,SATL	dist=2804;dis	.	.	A	G	+	+	0.41666667	42,0,30,0	chrX,843458	Cytosol	A > G
chrX:84345885:A:G:intergenic:APC	chrX	84345885	84345885	A	G	intergenic	APOOL,SATL	dist=2816;dis	.	.	A	G	+	+	0.45833333	39,0,33,0	chrX,843458	Cytosol	A > G
chrX:84345886:A:G:intergenic:APC	chrX	84345886	84345886	A	G	intergenic	APOOL,SATL	dist=2817;dis	.	.	A	G	+	+	0.49295775	36,0,35,0	chrX,843458	Cytosol	A > G
chrX:84345903:A:G:intergenic:APC	chrX	84345903	84345903	A	G	intergenic	APOOL,SATL	dist=2834;dis	.	.	A	G	+	+	0.12962963	47,0,7,0	chrX,843459	Cytosol	A > G
chrX:84346012:A:G:intergenic:APC	chrX	84346012	84346012	A	G	intergenic	APOOL,SATL	dist=2943;dis	.	.	A	G	+	+	0.42857143	16,0,12,0	chrX,843460	Cytosol	A > G
chrX:84346026:A:G:intergenic:APC	chrX	84346026	84346026	A	G	intergenic	APOOL,SATL	dist=2957;dis	.	.	A	G	+	+	0.65714286	12,0,23,0	chrX,843460	Cytosol	A > G
chrX:84346031:A:G:intergenic:APC	chrX	84346031	84346031	A	G	intergenic	APOOL,SATL	dist=2962;dis	.	.	A	G	+	+	0.09090909	30,0,3,0	chrX,843460	Cytosol	A > G
chrX:84346113:A:G:intergenic:APC	chrX	84346113	84346113	A	G	intergenic	APOOL,SATL	dist=3044;dis	.	.	A	G	+	+	0.05405405	35,0,2,0	chrX,843461	Cytosol	A > G
chrX:84346136:A:G:intergenic:APC	chrX	84346136	84346136	A	G	intergenic	APOOL,SATL	dist=3067;dis	.	.	A	G	+	+	0.21621622	29,0,8,0	chrX,843461	Cytosol	A > G
chrX:84346241:A:G:intergenic:APC	chrX	84346241	84346241	A	G	intergenic	APOOL,SATL	dist=3172;dis	.	.	A	G	+	+	0.3125	33,0,15,0	chrX,843462	Cytosol	A > G
chrX:84346279:A:G:intergenic:APC	chrX	84346279	84346279	A	G	intergenic	APOOL,SATL	dist=3210;dis	.	.	A	G	+	+	0.4	21,0,14,0	chrX,843462	Cytosol	A > G
chrX:84346959:A:G:downstream:S	chrX	84346959	84346959	A	G	downstream	SATL1	.	.	.	A	G	+	+	0.39726027	44,0,29,0	chrX,843469	Cytosol	A > G
chrX:84346982:A:G:downstream:S	chrX	84346982	84346982	A	G	downstream	SATL1	.	.	.	A	G	+	+	0.22222222	77,0,22,0	chrX,843469	Cytosol	A > G
chrX:84346983:A:G:downstream:S	chrX	84346983	84346983	A	G	downstream	SATL1	.	.	.	A	G	+	+	0.16326531	82,0,16,0	chrX,843469	Cytosol	A > G
chrX:100262831:T:C:intergenic:AR	chrX	100262831	100262831	T	C	intergenic	ARL13A,TRM	dist=17013;d	.	.	A	G	-		0.92857143	1,0,13,0	chrX,100262	Cytosol	A > G
chrX:100262833:T:C:intergenic:AR	chrX	100262833	100262833	T	C	intergenic	ARL13A,TRM	dist=17015;d	.	.	A	G	-		0.85714286	2,0,12,0	chrX,100262	Cytosol	A > G
chrX:100263576:T:C:downstream:	chrX	100263576	100263576	T	C	downstream	TRMT2B	.	.	.	A	G	-		1	0,0,12,0	chrX,100263	Cytosol	A > G
chrX:100264544:T:C:UTR3:TRMT2I	chrX	100264544	100264544	T	C	UTR3	TRMT2B	ENST000005	.	.	A	G	-	-	0.03448276	84,0,3,0	chrX,100264	Cytosol	A > G
chrX:100264559:T:C:UTR3:TRMT2I	chrX	100264559	100264559	T	C	UTR3	TRMT2B	ENST000005	.	.	A	G	-	-	0.140625	55,0,9,0	chrX,100264	Cytosol	A > G
chrX:100264582:T:C:UTR3:TRMT2I	chrX	100264582	100264582	T	C	UTR3	TRMT2B	ENST000005	.	.	A	G	-	-	0.30666667	52,0,23,0	chrX,100264	Cytosol	A > G
chrX:100264587:T:C:UTR3:TRMT2I	chrX	100264587	100264587	T	C	UTR3	TRMT2B	ENST000005	.	.	A	G	-	-	0.07462687	62,0,5,0	chrX,100264	Cytosol	A > G
chrX:100264596:T:C:UTR3:TRMT2I	chrX	100264596	100264596	T	C	UTR3	TRMT2B	ENST000005	.	.	A	G	-	-	0.83606557	10,0,51,0	chrX,100264	Cytosol	A > G
chrX:100264597:T:C:UTR3:TRMT2I	chrX	100264597	100264597	T	C	UTR3	TRMT2B	ENST000005	.	.	A	G	-	-	0.22807018	44,0,13,0	chrX,100264	Cytosol	A > G
chrX:100264602:T:C:UTR3:TRMT2I	chrX	100264602	100264602	T	C	UTR3	TRMT2B	ENST000005	.	.	A	G	-	-	0.5	28,0,28,0	chrX,100264	Cytosol	A > G
chrX:100264619:T:C:UTR3:TRMT2I	chrX	100264619	100264619	T	C	UTR3	TRMT2B	ENST000005	.	.	A	G	-	-	0.04166667	46,0,2,0	chrX,100264	Cytosol	A > G
chrX:100264626:T:C:UTR3:TRMT2I	chrX	100264626	100264626	T	C	UTR3	TRMT2B	ENST000005	.	.	A	G	-	-	0.56521739	20,0,26,0	chrX,100264	Cytosol	A > G
chrX:100264627:T:C:UTR3:TRMT2I	chrX	100264627	100264627	T	C	UTR3	TRMT2B	ENST000005	.	.	A	G	-	-	0.75	12,0,36,0	chrX,100264	Cytosol	A > G
chrX:100264658:T:C:UTR3:TRMT2I	chrX	100264658	100264658	T	C	UTR3	TRMT2B	ENST000005	.	.	A	G	-	-	0.07692308	72,0,6,0	chrX,100264	Cytosol	A > G
chrX:100264662:T:C:UTR3:TRMT2I	chrX	100264662	100264662	T	C	UTR3	TRMT2B	ENST000005	.	.	A	G	-	-	0.05128205	74,0,4,0	chrX,100264	Cytosol	A > G
chrX:100264667:T:C:UTR3:TRMT2I	chrX	100264667	100264667	T	C	UTR3	TRMT2B	ENST000005	.	.	A	G	-	-	0.25333333	56,0,19,0	chrX,100264	Cytosol	A > G
chrX:100264668:T:C:UTR3:TRMT2I	chrX	100264668	100264668	T	C	UTR3	TRMT2B	ENST000005	.	.	A	G	-	-	0.25333333	56,0,19,0	chrX,100264	Cytosol	A > G
chrX:100264674:T:C:UTR3:TRMT2I	chrX	100264674	100264674	T	C	UTR3	TRMT2B	ENST000005	.	.	A	G	-	-	0.14864865	63,0,11,0	chrX,100264	Cytosol	A > G
chrX:100265279:T:C:UTR3:TRMT2I	chrX	100265279	100265279	T	C	UTR3	TRMT2B	ENST000005	.	.	A	G	-	-	0.08860759	72,0,7,0	chrX,100265	Cytosol	A > G
chrX:100265285:T:C:UTR3:TRMT2I	chrX	100265285	100265285	T	C	UTR3	TRMT2B	ENST000005	.	.	A	G	-	-	0.25316456	59,0,20,0	chrX,100265	Cytosol	A > G
chrX:100265289:T:C:UTR3:TRMT2I	chrX	100265289	100265289	T	C	UTR3	TRMT2B	ENST000005	.	.	A	G	-	-	0.1686747	69,0,14,0	chrX,100265	Cytosol	A > G
chrX:100265318:T:C:UTR3:TRMT2I	chrX	100265318	100265318	T	C	UTR3	TRMT2B	ENST000005	.	.	A	G	-	-	0.25333333	56,0,19,0	chrX,100265	Cytosol	A > G
chrX:100265319:T:C:UTR3:TRMT2I	chrX	100265319	100265319	T	C	UTR3	TRMT2B	ENST000005	.	.	A	G	-	-	0.2	60,0,15,0	chrX,100265	Cytosol	A > G
chrX:100265338:T:C:UTR3:TRMT2I	chrX	100265338	100265338	T	C	UTR3	TRMT2B	ENST000005	.	.	A	G	-	-	0.15254237	50,0,9,0	chrX,100265	Cytosol	A > G
chrX:100265344:T:C:UTR3:TRMT2I	chrX	100265344	100265344	T	C	UTR3	TRMT2B	ENST000005	.	.	A	G	-	-	0.34615385	34,0,18,0	chrX,100265	Cytosol	A > G
chrX:100265349:T:C:UTR3:TRMT2I	chrX	100265349	100265349	T	C	UTR3	TRMT2B	ENST000005	.	.	A	G	-	-	0.59259259	22,0,32,0	chrX,100265	Cytosol	A > G
chrX:100265391:T:C:UTR3:TRMT2I	chrX	100265391	100265391	T	C	UTR3	TRMT2B	ENST000005	.	.	A	G	-	-	0.3015873	44,0,19,0	chrX,100265	Cytosol	A > G
chrX:100265393:T:C:UTR3:TRMT2I	chrX	100265393	100265393	T	C	UTR3	TRMT2B	ENST000005	.	.	A	G	-	-	0.81818182	12,0,54,0	chrX,100265	Cytosol	A > G

chrX:100265397:T:C:UTR3:TRMT2	chrX	100265397	100265397	T	C	UTR3	TRMT2B	ENST000005	.	.	A	G	-	-	0.19354839	50,0,12,0	chrX,100265	Cytosol	A > G
chrX:100354876:A:G:UTR5:CENPI	chrX	100354876	100354876	A	G	UTR5	CENPI	ENST000004	.	.	A	G	+	+	0.0625	45,0,3,0	chrX,100354	Cytosol	A > G
chrX:100419420:A:G:downstream:	chrX	100419420	100419420	A	G	downstream	CENPI	.	.	.	A	G	+		0.54716981	24,0,29,0	chrX,100419	Cytosol	A > G
chrX:100419421:A:G:downstream:	chrX	100419421	100419421	A	G	downstream	CENPI	.	.	.	A	G	+		0.32692308	35,0,17,0	chrX,100419	Cytosol	A > G
chrX:100419482:A:G:downstream:	chrX	100419482	100419482	A	G	downstream	CENPI	.	.	.	A	G	+		0.93333333	2,0,28,0	chrX,100419	Cytosol	A > G
chrX:100599055:T:C:intergenic:RN	chrX	100599055	100599055	T	C	intergenic	RNU6-934P,1	dist=18557;d	.	.	A	G	-		0.21428571	11,0,3,0	chrX,100599	Cytosol	A > G
chrX:100599444:T:C:intergenic:RN	chrX	100599444	100599444	T	C	intergenic	RNU6-934P,1	dist=18946;d	.	.	A	G	-		0.33333333	8,0,4,0	chrX,100599	Cytosol	A > G
chrX:100599454:T:C:intergenic:RN	chrX	100599454	100599454	T	C	intergenic	RNU6-934P,1	dist=18956;d	.	.	A	G	-		0.53846154	6,0,7,0	chrX,100599	Cytosol	A > G
chrX:100599456:T:C:intergenic:RN	chrX	100599456	100599456	T	C	intergenic	RNU6-934P,1	dist=18958;d	.	.	A	G	-		0.5	6,0,6,0	chrX,100599	Cytosol	A > G
chrX:100599515:T:C:intergenic:RN	chrX	100599515	100599515	T	C	intergenic	RNU6-934P,1	dist=19017;d	.	.	A	G	-		0.28571429	5,0,2,0	chrX,100599	Cytosol	A > G
chrX:103366707:T:C:intronic:SLC2	chrX	103366707	103366707	T	C	intronic	SLC25A53	.	.	.	A	G	-	-	0.33333333	4,0,2,0	chrX,103366	Cytosol	A > G
chrX:103366708:T:C:intronic:SLC2	chrX	103366708	103366708	T	C	intronic	SLC25A53	.	.	.	A	G	-	-	0.25	6,0,2,0	chrX,103366	Cytosol	A > G
chrX:103371102:T:C:intronic:SLC2	chrX	103371102	103371102	T	C	intronic	SLC25A53	.	.	.	A	G	-	-	0.33333333	4,0,2,0	chrX,103371	Cytosol	A > G
chrX:103371251:T:C:intronic:SLC2	chrX	103371251	103371251	T	C	intronic	SLC25A53	.	.	.	A	G	-	-	0.15384615	11,0,2,0	chrX,103371	Cytosol	A > G
chrX:118152907:A:G:downstream:	chrX	118152907	118152907	A	G	downstream	LONRF3	.	.	.	A	G	+		0.4	3,0,2,0	chrX,118152	Cytosol	A > G
chrX:118587521:A:G:UTR3:SLC25A	chrX	118587521	118587521	A	G	UTR3	SLC25A43	ENST000002	.	.	A	G	+	+	0.0443038	151,0,7,0	chrX,118587	Cytosol	A > G
chrX:118672397:T:C:UTR3:CXorf5	chrX	118672397	118672397	T	C	UTR3	CXorf56	ENST000003	.	.	A	G	-	-	0.02325581	84,0,2,0	chrX,118672	Cytosol	A > G
chrX:118672469:T:C:UTR3:CXorf5	chrX	118672469	118672469	T	C	UTR3	CXorf56	ENST000003	.	.	A	G	-	-	0.25	9,0,3,0	chrX,118672	Cytosol	A > G
chrX:118672473:T:C:UTR3:CXorf5	chrX	118672473	118672473	T	C	UTR3	CXorf56	ENST000003	.	.	A	G	-	-	0.3125	11,0,5,0	chrX,118672	Cytosol	A > G
chrX:118672514:T:C:UTR3:CXorf5	chrX	118672514	118672514	T	C	UTR3	CXorf56	ENST000003	.	.	A	G	-	-	0.16	21,0,4,0	chrX,118672	Cytosol	A > G
chrX:118672525:T:C:UTR3:CXorf5	chrX	118672525	118672525	T	C	UTR3	CXorf56	ENST000003	.	.	A	G	-	-	0.59259259	11,0,16,0	chrX,118672	Cytosol	A > G
chrX:118672535:T:C:UTR3:CXorf5	chrX	118672535	118672535	T	C	UTR3	CXorf56	ENST000003	.	.	A	G	-	-	0.73529412	9,0,25,0	chrX,118672	Cytosol	A > G
chrX:118672561:T:C:UTR3:CXorf5	chrX	118672561	118672561	T	C	UTR3	CXorf56	ENST000003	.	.	A	G	-	-	0.37037037	17,0,10,0	chrX,118672	Cytosol	A > G
chrX:118672573:T:C:UTR3:CXorf5	chrX	118672573	118672573	T	C	UTR3	CXorf56	ENST000003	.	.	A	G	-	-	0.13888889	31,0,5,0	chrX,118672	Cytosol	A > G
chrX:118672577:T:C:UTR3:CXorf5	chrX	118672577	118672577	T	C	UTR3	CXorf56	ENST000003	.	.	A	G	-	-	0.23076923	30,0,9,0	chrX,118672	Cytosol	A > G
chrX:118672578:T:C:UTR3:CXorf5	chrX	118672578	118672578	T	C	UTR3	CXorf56	ENST000003	.	.	A	G	-	-	0.18918919	29,1,7,0	chrX,118672	Cytosol	A > G
chrX:118672600:T:C:UTR3:CXorf5	chrX	118672600	118672600	T	C	UTR3	CXorf56	ENST000003	.	.	A	G	-	-	0.10344828	26,0,3,0	chrX,118672	Cytosol	A > G
chrX:118672602:T:C:UTR3:CXorf5	chrX	118672602	118672602	T	C	UTR3	CXorf56	ENST000003	.	.	A	G	-	-	0.3	21,0,9,0	chrX,118672	Cytosol	A > G
chrX:118672641:T:C:UTR3:CXorf5	chrX	118672641	118672641	T	C	UTR3	CXorf56	ENST000003	.	.	A	G	-	-	0.34782609	15,0,8,0	chrX,118672	Cytosol	A > G
chrX:118672671:T:C:UTR3:CXorf5	chrX	118672671	118672671	T	C	UTR3	CXorf56	ENST000003	.	.	A	G	-	-	0.65686275	35,0,67,0	chrX,118672	Cytosol	A > G
chrX:118672672:T:C:UTR3:CXorf5	chrX	118672672	118672672	T	C	UTR3	CXorf56	ENST000003	.	.	A	G	-	-	0.85	15,0,85,0	chrX,118672	Cytosol	A > G
chrX:118673236:T:C:UTR3:CXorf5	chrX	118673236	118673236	T	C	UTR3	CXorf56	ENST000003	.	.	A	G	-	-	0.03174603	122,0,4,0	chrX,118673	Cytosol	A > G
chrX:118673238:T:C:UTR3:CXorf5	chrX	118673238	118673238	T	C	UTR3	CXorf56	ENST000003	.	.	A	G	-	-	0.52066116	58,0,63,0	chrX,118673	Cytosol	A > G
chrX:118673250:T:C:UTR3:CXorf5	chrX	118673250	118673250	T	C	UTR3	CXorf56	ENST000003	.	.	A	G	-	-	0.48360656	63,0,59,0	chrX,118673	Cytosol	A > G
chrX:118673251:T:C:UTR3:CXorf5	chrX	118673251	118673251	T	C	UTR3	CXorf56	ENST000003	.	.	A	G	-	-	0.136	108,0,17,0	chrX,118673	Cytosol	A > G
chrX:118673269:T:C:UTR3:CXorf5	chrX	118673269	118673269	T	C	UTR3	CXorf56	ENST000003	.	.	A	G	-	-	0.30188679	37,0,16,0	chrX,118673	Cytosol	A > G
chrX:118673312:T:C:UTR3:CXorf5	chrX	118673312	118673312	T	C	UTR3	CXorf56	ENST000003	.	.	A	G	-	-	0.44117647	19,0,15,0	chrX,118673	Cytosol	A > G
chrX:118673328:T:C:UTR3:CXorf5	chrX	118673328	118673328	T	C	UTR3	CXorf56	ENST000003	.	.	A	G	-	-	0.42424242	19,0,14,0	chrX,118673	Cytosol	A > G
chrX:118673331:T:C:UTR3:CXorf5	chrX	118673331	118673331	T	C	UTR3	CXorf56	ENST000003	.	.	A	G	-	-	0.38235294	21,0,13,0	chrX,118673	Cytosol	A > G
chrX:118673359:T:C:UTR3:CXorf5	chrX	118673359	118673359	T	C	UTR3	CXorf56	ENST000003	.	.	A	G	-	-	0.13333333	26,0,4,0	chrX,118673	Cytosol	A > G
chrX:118673370:T:C:UTR3:CXorf5	chrX	118673370	118673370	T	C	UTR3	CXorf56	ENST000003	.	.	A	G	-	-	0.0625	30,0,2,0	chrX,118673	Cytosol	A > G
chrX:118673372:T:C:UTR3:CXorf5	chrX	118673372	118673372	T	C	UTR3	CXorf56	ENST000003	.	.	A	G	-	-	0.1875	26,0,6,0	chrX,118673	Cytosol	A > G
chrX:118673382:T:C:UTR3:CXorf5	chrX	118673382	118673382	T	C	UTR3	CXorf56	ENST000003	.	.	A	G	-	-	0.08333333	33,0,3,0	chrX,118673	Cytosol	A > G
chrX:118673411:T:C:UTR3:CXorf5	chrX	118673411	118673411	T	C	UTR3	CXorf56	ENST000003	.	.	A	G	-	-	0.52777778	17,0,19,0	chrX,118673	Cytosol	A > G
chrX:118673412:T:C:UTR3:CXorf5	chrX	118673412	118673412	T	C	UTR3	CXorf56	ENST000003	.	.	A	G	-	-	0.57142857	15,0,20,0	chrX,118673	Cytosol	A > G
chrX:118673431:T:C:UTR3:CXorf5	chrX	118673431	118673431	T	C	UTR3	CXorf56	ENST000003	.	.	A	G	-	-	0.32142857	19,0,9,0	chrX,118673	Cytosol	A > G
chrX:118673437:T:C:UTR3:CXorf5	chrX	118673437	118673437	T	C	UTR3	CXorf56	ENST000003	.	.	A	G	-	-	0.28	18,0,7,0	chrX,118673	Cytosol	A > G
chrX:118673487:T:C:UTR3:CXorf5	chrX	118673487	118673487	T	C	UTR3	CXorf56	ENST000003	.	.	A	G	-	-	0.69444444	11,0,25,0	chrX,118673	Cytosol	A > G
chrX:119571796:T:C:UTR3:LAMP2	chrX	119571796	119571796	T	C	UTR3	LAMP2	ENST000003	.	.	A	G	-	-	0.10810811	99,0,12,0	chrX,119571	Cytosol	A > G
chrX:119571813:T:C:UTR3:LAMP2	chrX	119571813	119571813	T	C	UTR3	LAMP2	ENST000003	.	.	A	G	-	-	0.01923077	102,0,2,0	chrX,119571	Cytosol	A > G
chrX:119571848:T:C:UTR3:LAMP2	chrX	119571848	119571848	T	C	UTR3	LAMP2	ENST000003	.	.	A	G	-	-	0.05660377	100,0,6,0	chrX,119571	Cytosol	A > G
chrX:119571849:T:C:UTR3:LAMP2	chrX	119571849	119571849	T	C	UTR3	LAMP2	ENST000003	.	.	A	G	-	-	0.05882353	96,0,6,0	chrX,119571	Cytosol	A > G
chrX:119571961:T:C:UTR3:LAMP2	chrX	119571961	119571961	T	C	UTR3	LAMP2	ENST000003	.	.	A	G	-	-	0.04545455	63,0,3,0	chrX,119571	Cytosol	A > G
chrX:123042979:A:G:UTR3:XIAP	chrX	123042979	123042979	A	G	UTR3	XIAP	ENST000003	.	.	A	G	+	+	0.5	16,0,16,0	chrX,123042	Cytosol	A > G
chrX:123043064:A:G:UTR3:XIAP	chrX	123043064	123043064	A	G	UTR3	XIAP	ENST000003	.	.	A	G	+	+	0.33333333	10,0,5,0	chrX,123043	Cytosol	A > G
chrX:123043081:A:G:UTR3:XIAP	chrX	123043081	123043081	A	G	UTR3	XIAP	ENST000003	.	.	A	G	+	+	0.21052632	15,0,4,0	chrX,123043	Cytosol	A > G
chrX:123043116:A:G:UTR3:XIAP	chrX	123043116	123043116	A	G	UTR3	XIAP	ENST000003	.	.	A	G	+	+	0.10344828	26,0,3,0	chrX,123043	Cytosol	A > G
chrX:123043143:A:G:UTR3:XIAP	chrX	123043143	123043143	A	G	UTR3	XIAP	ENST000003	.	.	A	G	+	+	0.28571429	30,0,12,0	chrX,123043	Cytosol	A > G
chrX:123043155:A:G:UTR3:XIAP	chrX	123043155	123043155	A	G	UTR3	XIAP	ENST000003	.	.	A	G	+	+	0.14285714	36,0,6,0	chrX,123043	Cytosol	A > G
chrX:123044464:A:G:UTR3:XIAP	chrX	123044464	123044464	A	G	UTR3	XIAP	ENST000003	.	.	A	G	+	+	0.08695652	42,0,4,0	chrX,123044	Cytosol	A > G
chrX:123044465:A:G:UTR3:XIAP	chrX	123044465	123044465	A	G	UTR3	XIAP	ENST000003	.	.	A	G	+	+	0.19047619	34,0,8,0	chrX,123044	Cytosol	A > G
chrX:123044532:A:G:UTR3:XIAP	chrX	123044532	123044532	A	G	UTR3	XIAP	ENST000003	.	.	A	G	+	+	0.12903226	27,0,4,0	chrX,123044	Cytosol	A > G
chrX:123044559:A:G:UTR3:XIAP	chrX	123044559	123044559	A	G	UTR3	XIAP	ENST000003	.	.	A	G	+	+	0.05405405	35,0,2,0	chrX,123044	Cytosol	A > G
chrX:123044571:A:G:UTR3:XIAP	chrX	123044571	123044571	A	G	UTR3	XIAP	ENST000003	.	.	A	G	+	+	0.12121212	29,0,4,0	chrX,123044	Cytosol	A > G

chrX:123044597:A:G:UTR3:XIAP	chrX	123044597	123044597	A	G	UTR3	XIAP	ENST000003	.	.	A	G	+	+	0.10909091	49,0,6,0	chrX,123044	Cytosol	A > G
chrX:123045381:A:G:UTR3:XIAP	chrX	123045381	123045381	A	G	UTR3	XIAP	ENST000003	.	.	A	G	+	+	0.08974359	71,0,7,0	chrX,123045	Cytosol	A > G
chrX:123045385:A:G:UTR3:XIAP	chrX	123045385	123045385	A	G	UTR3	XIAP	ENST000003	.	.	A	G	+	+	0.12857143	61,0,9,0	chrX,123045	Cytosol	A > G
chrX:123045405:A:G:UTR3:XIAP	chrX	123045405	123045405	A	G	UTR3	XIAP	ENST000003	.	.	A	G	+	+	0.13095238	73,0,11,0	chrX,123045	Cytosol	A > G
chrX:123045407:A:G:UTR3:XIAP	chrX	123045407	123045407	A	G	UTR3	XIAP	ENST000003	.	.	A	G	+	+	0.11956522	81,0,11,0	chrX,123045	Cytosol	A > G
chrX:123045443:A:G:UTR3:XIAP	chrX	123045443	123045443	A	G	UTR3	XIAP	ENST000003	.	.	A	G	+	+	0.0625	45,0,3,0	chrX,123045	Cytosol	A > G
chrX:123046290:A:G:UTR3:XIAP	chrX	123046290	123046290	A	G	UTR3	XIAP	ENST000003	.	.	A	G	+	+	0.03773585	102,0,4,0	chrX,123046	Cytosol	A > G
chrX:123046306:A:G:UTR3:XIAP	chrX	123046306	123046306	A	G	UTR3	XIAP	ENST000003	.	.	A	G	+	+	0.05714286	99,0,6,0	chrX,123046	Cytosol	A > G
chrX:123046590:A:G:UTR3:XIAP	chrX	123046590	123046590	A	G	UTR3	XIAP	ENST000003	.	.	A	G	+	+	0.16	42,0,8,0	chrX,123046	Cytosol	A > G
chrX:123046639:A:G:UTR3:XIAP	chrX	123046639	123046639	A	G	UTR3	XIAP	ENST000003	.	.	A	G	+	+	0.10465116	77,0,9,0	chrX,123046	Cytosol	A > G
chrX:123046644:A:G:UTR3:XIAP	chrX	123046644	123046644	A	G	UTR3	XIAP	ENST000003	.	.	A	G	+	+	0.05319149	89,0,5,0	chrX,123046	Cytosol	A > G
chrX:123046686:A:G:UTR3:XIAP	chrX	123046686	123046686	A	G	UTR3	XIAP	ENST000003	.	.	A	G	+	+	0.32926829	55,0,27,0	chrX,123046	Cytosol	A > G
chrX:123046695:A:G:UTR3:XIAP	chrX	123046695	123046695	A	G	UTR3	XIAP	ENST000003	.	.	A	G	+	+	0.36708861	50,0,29,0	chrX,123046	Cytosol	A > G
chrX:123046741:A:G:UTR3:XIAP	chrX	123046741	123046741	A	G	UTR3	XIAP	ENST000003	.	.	A	G	+	+	0.09677419	56,0,6,0	chrX,123046	Cytosol	A > G
chrX:123046758:A:G:UTR3:XIAP	chrX	123046758	123046758	A	G	UTR3	XIAP	ENST000003	.	.	A	G	+	+	0.27586207	63,0,24,0	chrX,123046	Cytosol	A > G
chrX:123046772:A:G:UTR3:XIAP	chrX	123046772	123046772	A	G	UTR3	XIAP	ENST000003	.	.	A	G	+	+	0.14102564	67,0,11,0	chrX,123046	Cytosol	A > G
chrX:123046865:A:G:UTR3:XIAP	chrX	123046865	123046865	A	G	UTR3	XIAP	ENST000003	.	.	A	G	+	+	0.06521739	43,0,3,0	chrX,123046	Cytosol	A > G
chrX:123046893:A:G:UTR3:XIAP	chrX	123046893	123046893	A	G	UTR3	XIAP	ENST000003	.	.	A	G	+	+	0.04347826	66,0,3,0	chrX,123046	Cytosol	A > G
chrX:123046905:A:G:UTR3:XIAP	chrX	123046905	123046905	A	G	UTR3	XIAP	ENST000003	.	.	A	G	+	+	0.12903226	54,0,8,0	chrX,123046	Cytosol	A > G
chrX:123047066:A:G:UTR3:XIAP	chrX	123047066	123047066	A	G	UTR3	XIAP	ENST000003	.	.	A	G	+	+	0.0375	77,0,3,0	chrX,123047	Cytosol	A > G
chrX:123047169:A:G:UTR3:XIAP	chrX	123047169	123047169	A	G	UTR3	XIAP	ENST000003	.	.	A	G	+	+	0.04938272	77,0,4,0	chrX,123047	Cytosol	A > G
chrX:123047199:A:G:UTR3:XIAP	chrX	123047199	123047199	A	G	UTR3	XIAP	ENST000003	.	.	A	G	+	+	0.02752294	106,0,3,0	chrX,123047	Cytosol	A > G
chrX:123047285:A:G:UTR3:XIAP	chrX	123047285	123047285	A	G	UTR3	XIAP	ENST000003	.	.	A	G	+	+	0.15384615	55,0,10,0	chrX,123047	Cytosol	A > G
chrX:133975122:A:G:intronic:FAM	chrX	133975122	133975122	A	G	intronic	FAM122C	.	.	.	A	G	+	+	0.6	2,0,3,0	chrX,133975	Cytosol	A > G
chrX:133977083:A:G:intronic:FAM	chrX	133977083	133977083	A	G	intronic	FAM122C	.	.	.	A	G	+	+	0.4	3,0,2,0	chrX,133977	Cytosol	A > G
chrX:135300016:T:C:UTR3:MAP7D	chrX	135300016	135300016	T	C	UTR3	MAP7D3	ENST000003	.	.	A	G	-	-	0.39473684	23,0,15,0	chrX,135300	Cytosol	A > G
chrX:135300092:T:C:UTR3:MAP7D	chrX	135300092	135300092	T	C	UTR3	MAP7D3	ENST000003	.	.	A	G	-	-	0.23809524	16,0,5,0	chrX,135300	Cytosol	A > G
chrX:153275682:T:C:downstream:	chrX	153275682	153275682	T	C	downstream	IRAK1	.	.	.	A	G	-		0.8	1,0,4,0	chrX,153275	Cytosol	A > G
chrX:153275686:T:C:downstream:	chrX	153275686	153275686	T	C	downstream	IRAK1	.	.	.	A	G	-		1	0,0,5,0	chrX,153275	Cytosol	A > G
chrX:153275698:T:C:downstream:	chrX	153275698	153275698	T	C	downstream	IRAK1	.	.	.	A	G	-		0.5	3,0,3,0	chrX,153275	Cytosol	A > G
chrX:153276566:T:C:exonic:IRAK1	chrX	153276566	153276566	T	C	exonic	IRAK1	.	nonsynonym	IRAK1:ENST0	A	G	-	-	0.04022989	835,0,35,0	chrX,153276	Cytosol	A > G
chrX:153276668:T:C:UTR3:IRAK1	chrX	153276668	153276668	T	C	UTR3	IRAK1	ENST000003	.	.	A	G	-	-	0.02435897	761,0,19,0	chrX,153276	Cytosol	A > G
chrX:153276687:T:C:UTR3:IRAK1	chrX	153276687	153276687	T	C	UTR3	IRAK1	ENST000003	.	.	A	G	-	-	0.0338295	714,0,25,0	chrX,153276	Cytosol	A > G
chrX:153276787:T:C:UTR3:IRAK1	chrX	153276787	153276787	T	C	UTR3	IRAK1	ENST000003	.	.	A	G	-	-	0.01245552	555,0,7,0	chrX,153276	Cytosol	A > G
chrX:153355698:T:C:intronic:MECI	chrX	153355698	153355698	T	C	intronic	MECP2	.	.	.	A	G	-	-	0.22222222	7,0,2,0	chrX,153355	Cytosol	A > G
chrX:153355766:T:C:intronic:MECI	chrX	153355766	153355766	T	C	intronic	MECP2	.	.	.	A	G	-	-	0.375	5,0,3,0	chrX,153355	Cytosol	A > G
chrX:154353124:A:G:intronic:MTC	chrX	154353124	154353124	A	G	intronic	MTCP1	.	.	.	A	G	+	-	0.57142857	6,0,8,0	chrX,154353	Cytosol	A > G
chr1:662647:T:C:intergenic:RP5-8	chr1	662647	662647	T	C	intergenic	RP5-857K21.	dist=2717;dis	.	.	A	G	-	-	0.76190476	5,0,16,0	chr1,662646,	nuRNA-WT	A > G
chr1:676236:T:C:downstream:RP1	chr1	676236	676236	T	C	downstream	RP11-206L10	.	.	.	A	G	-	-	0.125	14,0,2,0	chr1,676235,	nuRNA-WT	A > G
chr1:714881:A:G:ncRNA_intronic:	chr1	714881	714881	A	G	ncRNA_intro	RP11-206L10	.	.	.	A	G	+	+	0.28571429	5,0,2,0	chr1,714880,	nuRNA-WT	A > G
chr1:714882:A:G:ncRNA_intronic:	chr1	714882	714882	A	G	ncRNA_intro	RP11-206L10	.	.	.	A	G	+	+	0.66666667	2,0,4,0	chr1,714881,	nuRNA-WT	A > G
chr1:1028368:T:C:intronic:C1orf15	chr1	1028368	1028368	T	C	intronic	C1orf159	.	.	.	A	G	-	-	0.125	14,0,2,0	chr1,102836	nuRNA-WT	A > G
chr1:1029575:T:C:intronic:C1orf15	chr1	1029575	1029575	T	C	intronic	C1orf159	.	.	.	A	G	-	-	0.26923077	19,0,7,0	chr1,102957	nuRNA-WT	A > G
chr1:1031712:T:C:intronic:C1orf15	chr1	1031712	1031712	T	C	intronic	C1orf159	.	.	.	A	G	-	-	0.2	12,0,3,0	chr1,103171	nuRNA-WT	A > G
chr1:1032391:T:C:intronic:C1orf15	chr1	1032391	1032391	T	C	intronic	C1orf159	.	.	.	A	G	-	-	0.11111111	16,0,2,0	chr1,103239	nuRNA-WT	A > G
chr1:1032680:T:C:intronic:C1orf15	chr1	1032680	1032680	T	C	intronic	C1orf159	.	.	.	A	G	-	-	0.3	7,0,3,0	chr1,103267	nuRNA-WT	A > G
chr1:1032736:T:C:intronic:C1orf15	chr1	1032736	1032736	T	C	intronic	C1orf159	.	.	.	A	G	-	-	0.11111111	16,0,2,0	chr1,103273	nuRNA-WT	A > G
chr1:1035397:T:C:intronic:C1orf15	chr1	1035397	1035397	T	C	intronic	C1orf159	.	.	.	A	G	-	-	0.15	17,0,3,0	chr1,103539	nuRNA-WT	A > G
chr1:1035414:T:C:intronic:C1orf15	chr1	1035414	1035414	T	C	intronic	C1orf159	.	.	.	A	G	-	-	0.1875	13,0,3,0	chr1,103541	nuRNA-WT	A > G
chr1:1036697:T:C:intronic:C1orf15	chr1	1036697	1036697	T	C	intronic	C1orf159	.	.	.	A	G	-	-	0.72727273	3,0,8,0	chr1,103669	nuRNA-WT	A > G
chr1:1036700:T:C:intronic:C1orf15	chr1	1036700	1036700	T	C	intronic	C1orf159	.	.	.	A	G	-	-	0.18181818	9,0,2,0	chr1,103669	nuRNA-WT	A > G
chr1:1037255:T:C:intronic:C1orf15	chr1	1037255	1037255	T	C	intronic	C1orf159	.	.	.	A	G	-	-	0.42857143	4,0,3,0	chr1,103725	nuRNA-WT	A > G
chr1:1038441:T:C:intronic:C1orf15	chr1	1038441	1038441	T	C	intronic	C1orf159	.	.	.	A	G	-	-	0.66666667	2,0,4,0	chr1,103844	nuRNA-WT	A > G
chr1:1039203:T:C:intronic:C1orf15	chr1	1039203	1039203	T	C	intronic	C1orf159	.	.	.	A	G	-	-	0.13333333	13,0,2,0	chr1,103920	nuRNA-WT	A > G
chr1:1039216:T:C:intronic:C1orf15	chr1	1039216	1039216	T	C	intronic	C1orf159	.	.	.	A	G	-	-	0.125	14,0,2,0	chr1,103921	nuRNA-WT	A > G
chr1:1040075:T:C:intronic:C1orf15	chr1	1040075	1040075	T	C	intronic	C1orf159	.	.	.	A	G	-	-	0.5	6,0,6,0	chr1,104007	nuRNA-WT	A > G
chr1:1048186:T:C:intronic:C1orf15	chr1	1048186	1048186	T	C	intronic	C1orf159	.	.	.	A	G	-	-	0.57142857	6,0,8,0	chr1,104818	nuRNA-WT	A > G
chr1:1048331:T:C:intronic:C1orf15	chr1	1048331	1048331	T	C	intronic	C1orf159	.	.	.	A	G	-	-	0.66666667	4,0,8,0	chr1,104833	nuRNA-WT	A > G
chr1:1048365:T:C:intronic:C1orf15	chr1	1048365	1048365	T	C	intronic	C1orf159	.	.	.	A	G	-	-	0.5	3,0,3,0	chr1,104836	nuRNA-WT	A > G
chr1:1156882:T:C:intronic:SDF4	chr1	1156882	1156882	T	C	intronic	SDF4	.	.	.	A	G	-	-	0.24705882	64,0,21,0	chr1,115688	nuRNA-WT	A > G
chr1:1157460:T:C:intronic:SDF4	chr1	1157460	1157460	T	C	intronic	SDF4	.	.	.	A	G	-	-	0.17142857	58,0,12,0	chr1,115745	nuRNA-WT	A > G
chr1:1200212:T:C:splicing:UBE2J2	chr1	1200212	1200212	T	C	splicing	UBE2J2	ENST000004	.	.	A	G	-	-	0.28571429	5,0,2,0	chr1,120021	nuRNA-WT	A > G
chr1:1201179:T:C:intronic:UBE2J2	chr1	1201179	1201179	T	C	intronic	UBE2J2	.	.	.	A	G	-	-	0.4	3,0,2,0	chr1,120117	nuRNA-WT	A > G
chr1:1202107:T:C:intronic:UBE2J2	chr1	1202107	1202107	T	C	intronic	UBE2J2	.	.	.	A	G	-	-	0.33333333	4,0,2,0	chr1,120210	nuRNA-WT	A > G

chr1:1251538:T:C:intronic:CPSF3L	chr1	1251538	1251538	T	C	intronic	CPSF3L	.	.	.	A	G	-	-	0.02020202	97,0,2,0	chr1,125153	nuRNA-WT	A > G
chr1:1251540:T:C:intronic:CPSF3L	chr1	1251540	1251540	T	C	intronic	CPSF3L	.	.	.	A	G	-	-	0.03092784	94,0,3,0	chr1,125153	nuRNA-WT	A > G
chr1:1251610:T:C:intronic:CPSF3L	chr1	1251610	1251610	T	C	intronic	CPSF3L	.	.	.	A	G	-	-	0.046875	61,0,3,0	chr1,125160	nuRNA-WT	A > G
chr1:1251631:T:C:intronic:CPSF3L	chr1	1251631	1251631	T	C	intronic	CPSF3L	.	.	.	A	G	-	-	0.04705882	81,0,4,0	chr1,125163	nuRNA-WT	A > G
chr1:1251841:T:C:intronic:CPSF3L	chr1	1251841	1251841	T	C	intronic	CPSF3L	.	.	.	A	G	-	-	0.18367347	40,0,9,0	chr1,125184	nuRNA-WT	A > G
chr1:1251844:T:C:intronic:CPSF3L	chr1	1251844	1251844	T	C	intronic	CPSF3L	.	.	.	A	G	-	-	0.2	40,0,10,0	chr1,125184	nuRNA-WT	A > G
chr1:1251857:T:C:intronic:CPSF3L	chr1	1251857	1251857	T	C	intronic	CPSF3L	.	.	.	A	G	-	-	0.08196721	56,0,5,0	chr1,125185	nuRNA-WT	A > G
chr1:1251914:T:C:intronic:CPSF3L	chr1	1251914	1251914	T	C	intronic	CPSF3L	.	.	.	A	G	-	-	0.08	23,0,2,0	chr1,125191	nuRNA-WT	A > G
chr1:1252180:T:C:intronic:CPSF3L	chr1	1252180	1252180	T	C	intronic	CPSF3L	.	.	.	A	G	-	-	0.83333333	2,0,10,0	chr1,125217	nuRNA-WT	A > G
chr1:1252182:T:C:intronic:CPSF3L	chr1	1252182	1252182	T	C	intronic	CPSF3L	.	.	.	A	G	-	-	0.66666667	4,0,8,0	chr1,125218	nuRNA-WT	A > G
chr1:1252221:T:C:intronic:CPSF3L	chr1	1252221	1252221	T	C	intronic	CPSF3L	.	.	.	A	G	-	-	0.21428571	11,0,3,0	chr1,125222	nuRNA-WT	A > G
chr1:1252441:T:C:intronic:CPSF3L	chr1	1252441	1252441	T	C	intronic	CPSF3L	.	.	.	A	G	-	-	0.91891892	3,0,34,0	chr1,125244	nuRNA-WT	A > G
chr1:1252468:T:C:intronic:CPSF3L	chr1	1252468	1252468	T	C	intronic	CPSF3L	.	.	.	A	G	-	-	0.25	30,0,10,0	chr1,125246	nuRNA-WT	A > G
chr1:1252510:T:C:intronic:CPSF3L	chr1	1252510	1252510	T	C	intronic	CPSF3L	.	.	.	A	G	-	-	0.04166667	46,0,2,0	chr1,125250	nuRNA-WT	A > G
chr1:1252513:T:C:intronic:CPSF3L	chr1	1252513	1252513	T	C	intronic	CPSF3L	.	.	.	A	G	-	-	0.07692308	48,0,4,0	chr1,125251	nuRNA-WT	A > G
chr1:1252587:T:C:intronic:CPSF3L	chr1	1252587	1252587	T	C	intronic	CPSF3L	.	.	.	A	G	-	-	0.19047619	17,0,4,0	chr1,125258	nuRNA-WT	A > G
chr1:1252654:A:G:intronic:CPSF3L	chr1	1252654	1252654	A	G	intronic	CPSF3L	.	.	.	A	G	+	-	0.11764706	15,0,2,0	chr1,125265	nuRNA-WT	A > G
chr1:1252674:T:C:intronic:CPSF3L	chr1	1252674	1252674	T	C	intronic	CPSF3L	.	.	.	A	G	-	-	0.10638298	42,0,5,0	chr1,125267	nuRNA-WT	A > G
chr1:1252723:T:C:intronic:CPSF3L	chr1	1252723	1252723	T	C	intronic	CPSF3L	.	.	.	A	G	-	-	0.16923077	54,0,11,0	chr1,125272	nuRNA-WT	A > G
chr1:1252749:T:C:intronic:CPSF3L	chr1	1252749	1252749	T	C	intronic	CPSF3L	.	.	.	A	G	-	-	0.05882353	32,0,2,0	chr1,125274	nuRNA-WT	A > G
chr1:1252786:G:A:intronic;intronic	chr1	1252786	1252786	G	A	intronic;intro	CPSF3L	.	.	.	C	T	-	-	0.11428571	0,31,0,4	chr1,125278	nuRNA-WT	C > T
chr1:1252786:G:A:intronic;intronic	chr1	1252786	1252786	G	A	intronic;intro	CPSF3L	.	.	.	G	A	+	-	0.18181818	2,0,9,0	chr1,125278	nuRNA-WT	G > A
chr1:1253313:T:C:ncRNA_intronic:	chr1	1253313	1253313	T	C	ncRNA_intro	RP5-890O3.9	.	.	.	A	G	-	-	0.38235294	21,0,13,0	chr1,125331	nuRNA-WT	A > G
chr1:1253335:T:C:ncRNA_intronic:	chr1	1253335	1253335	T	C	ncRNA_intro	RP5-890O3.9	.	.	.	A	G	-	-	0.10344828	26,0,3,0	chr1,125333	nuRNA-WT	A > G
chr1:1253343:T:C:ncRNA_intronic:	chr1	1253343	1253343	T	C	ncRNA_intro	RP5-890O3.9	.	.	.	A	G	-	-	0.32	17,0,8,0	chr1,125334	nuRNA-WT	A > G
chr1:1253351:T:C:ncRNA_intronic:	chr1	1253351	1253351	T	C	ncRNA_intro	RP5-890O3.9	.	.	.	A	G	-	-	0.15	17,0,3,0	chr1,125335	nuRNA-WT	A > G
chr1:1253354:T:C:ncRNA_intronic:	chr1	1253354	1253354	T	C	ncRNA_intro	RP5-890O3.9	.	.	.	A	G	-	-	0.0952381	19,0,2,0	chr1,125335	nuRNA-WT	A > G
chr1:1253357:T:C:ncRNA_intronic:	chr1	1253357	1253357	T	C	ncRNA_intro	RP5-890O3.9	.	.	.	A	G	-	-	0.47368421	10,0,9,0	chr1,125335	nuRNA-WT	A > G
chr1:1253376:T:C:ncRNA_intronic:	chr1	1253376	1253376	T	C	ncRNA_intro	RP5-890O3.9	.	.	.	A	G	-	-	0.21052632	15,0,4,0	chr1,125337	nuRNA-WT	A > G
chr1:1253411:T:C:ncRNA_intronic:	chr1	1253411	1253411	T	C	ncRNA_intro	RP5-890O3.9	.	.	.	A	G	-	-	0.11764706	15,0,2,0	chr1,125341	nuRNA-WT	A > G
chr1:1253419:T:C:ncRNA_intronic:	chr1	1253419	1253419	T	C	ncRNA_intro	RP5-890O3.9	.	.	.	A	G	-	-	0.2	12,0,3,0	chr1,125341	nuRNA-WT	A > G
chr1:1253422:T:C:ncRNA_intronic:	chr1	1253422	1253422	T	C	ncRNA_intro	RP5-890O3.9	.	.	.	A	G	-	-	0.125	14,0,2,0	chr1,125342	nuRNA-WT	A > G
chr1:1253424:T:C:ncRNA_intronic:	chr1	1253424	1253424	T	C	ncRNA_intro	RP5-890O3.9	.	.	.	A	G	-	-	0.14285714	12,0,2,0	chr1,125342	nuRNA-WT	A > G
chr1:1253434:T:C:ncRNA_intronic:	chr1	1253434	1253434	T	C	ncRNA_intro	RP5-890O3.9	.	.	.	A	G	-	-	0.125	14,0,2,0	chr1,125343	nuRNA-WT	A > G
chr1:1253455:T:C:ncRNA_intronic:	chr1	1253455	1253455	T	C	ncRNA_intro	RP5-890O3.9	.	.	.	A	G	-	-	0.25	9,0,3,0	chr1,125345	nuRNA-WT	A > G
chr1:1253514:T:C:ncRNA_intronic:	chr1	1253514	1253514	T	C	ncRNA_intro	RP5-890O3.9	.	.	.	A	G	-	-	0.07692308	36,0,3,0	chr1,125351	nuRNA-WT	A > G
chr1:1253522:T:C:ncRNA_intronic:	chr1	1253522	1253522	T	C	ncRNA_intro	RP5-890O3.9	.	.	.	A	G	-	-	0.0952381	38,0,4,0	chr1,125352	nuRNA-WT	A > G
chr1:1253523:T:C:ncRNA_intronic:	chr1	1253523	1253523	T	C	ncRNA_intro	RP5-890O3.9	.	.	.	A	G	-	-	0.11363636	39,0,5,0	chr1,125352	nuRNA-WT	A > G
chr1:1253531:T:C:ncRNA_intronic:	chr1	1253531	1253531	T	C	ncRNA_intro	RP5-890O3.9	.	.	.	A	G	-	-	0.1	36,0,4,0	chr1,125353	nuRNA-WT	A > G
chr1:1253793:T:C:ncRNA_intronic:	chr1	1253793	1253793	T	C	ncRNA_intro	RP5-890O3.9	.	.	.	A	G	-	-	0.02803738	104,0,3,0	chr1,125379	nuRNA-WT	A > G
chr1:1253803:T:C:ncRNA_intronic:	chr1	1253803	1253803	T	C	ncRNA_intro	RP5-890O3.9	.	.	.	A	G	-	-	0.05434783	87,0,5,0	chr1,125380	nuRNA-WT	A > G
[truncated: 1,022,426 more chars]
